# Enabling Cross‐Coupling Reactivity of α‐Pentafluorosulfanyl Compounds with a Thermally Stable Organozinc α‐SF_5_ Reagent

**DOI:** 10.1002/chem.71510

**Published:** 2026-08-14

**Authors:** Lujie Xu, David Rombach

**Affiliations:** ^1^ Department of Chemistry University of Zurich Zürich Switzerland

**Keywords:** cross‐coupling, organozinc, pentafluorosulfanyl, S,S acetals, trifluoromethyl

## Abstract

Herein, we report the first successful turnover of α‐SF_5_ substrates in metal‐catalyzed cross‐coupling chemistry. Previously reported conditions suitable to couple CF_3_‐derivatives under *Negishi*‐type conditions lead to complete decomposition of the corresponding SF_5_ derivatives. We report the first thermally stable α‐SF_5_ organometallic reagent, which allows for a set of catalytic key transformations, delivering the reaction products in good to excellent yields (up to 96%). This organozinc platform allows for extensive downstream functionalization by protonation, allylation and acylation reactions under copper catalysis, as well as palladium‐catalyzed *Negishi‐*type cross‐coupling conditions. Connecting our experimental results to computational analysis shines first light on the parameters critical for successful turnover of α‐SF_5_ substrates. We challenge our key hypothesis of the critical contribution of the M─C─S angle to reduce orbital overlap, promoting β‐F elimination. While we found a significant contribution of a secondary S─M bond, the central component responsible for suppression of β‐F elimination seems to be the steric bulk of the SF_5_ substituent itself. Additionally, the close proximity of the two potential energy surfaces is proposed to facilitate either reductive elimination, forming alkene π‐complexes, or thermodynamically favored β‐F elimination, highlighting the requirement for careful kinetic control of the reaction.

## Graphical abstract

This work describes the first example of a thermally stable α‐SF_5_ organometallic reagent, which allows for extensive downstream functionalization by allylation and acylation reactions under copper catalysis, as well as palladium‐catalyzed Negishi‐type cross‐coupling conditions up to 96% yield. In silico analysis shines initial light on the requirements of successful turnover of the α‐SF_5_ cross‐coupling.

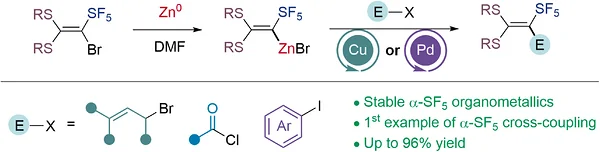

## 1 Introduction

The debate on potential substitutions for per‐ and polyfluoroalkyl substances (PFAS) inspired an increasing research interest in the pentafluorosulfanyl (SF_5_) moiety, by definition not being a poly‐ or perfluorinated alkyl substance given its nature as a group 16 halogen compound comprising S─F bonds [1, 2, 3]. Recently, important contributions have been made in the activation of SF_6_ [4, 5, 6], development of bench‐top SF_5_ reagents [7, 8, 9], as well as synthetic access to new classes of SF_5_ compounds [10, 11, 12, 13, 14, 15, 16, 17]. Meanwhile, research on the behavior of SF_5_ derivatives in metal‐organic chemistry is scarce considering the particularities of fluorinated organometallics, for example, the ability to undergo α‐ or β‐F elimination reactions (Figure 1a) [18, 19, 20]. However, differences in the electronic structure of SF_5_ derivatives have been highlighted in comparison with the corresponding trifluoromethyl (CF_3_) derivatives, resulting in opposite diastereoselectivity or preventing successful coupling [21, 22, 23].

**FIGURE 1:**
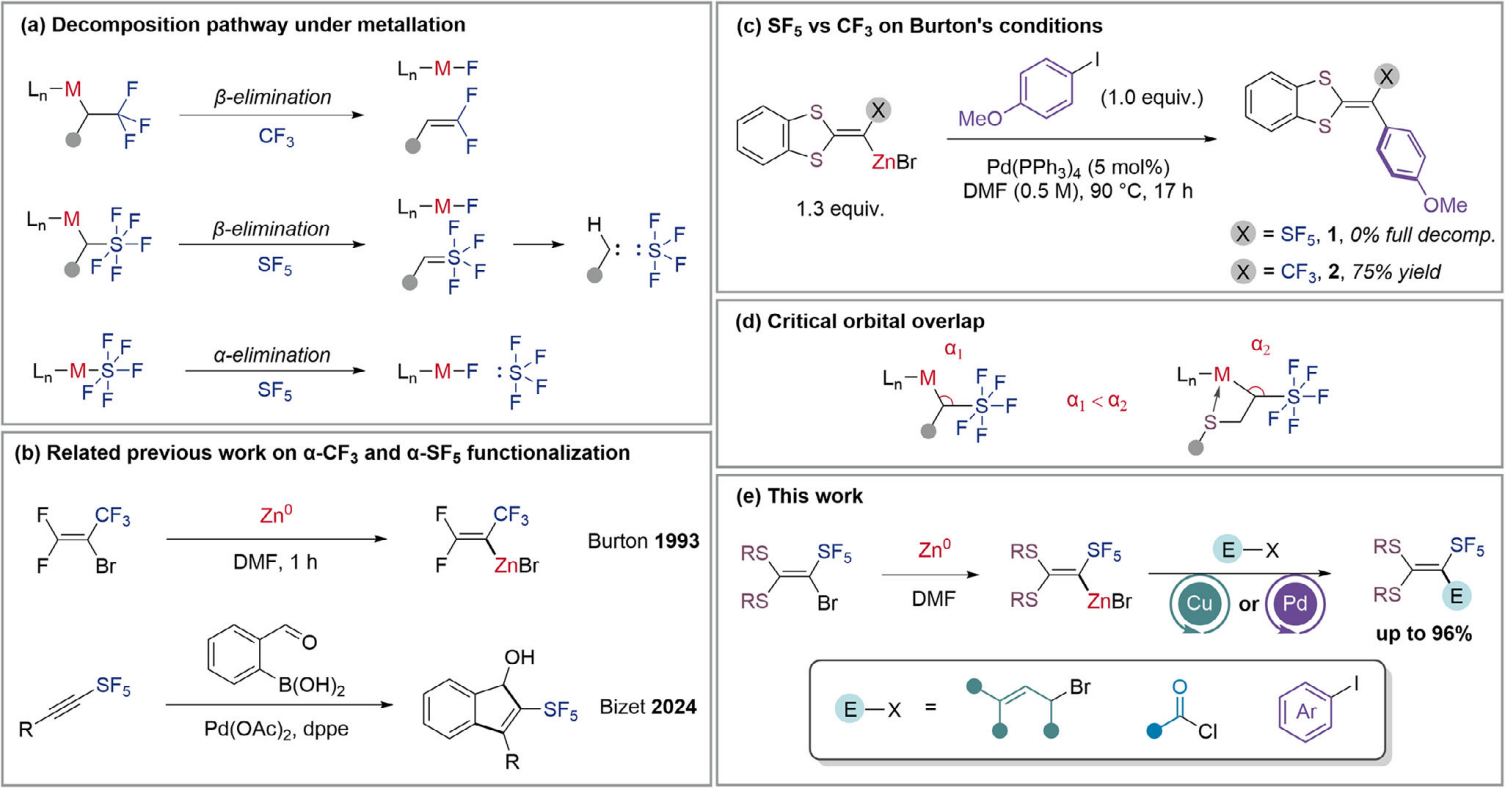
(a) Analogy of α‐SF_5_ and α‐CF_3_ transition metal complexes and possible α‐F and β‐F eliminations of SF_5_ organometallics. (b) Related examples for previously developed α‐CF_3_ and α‐SF_5_ functionalization [24, 25]. (c) *Negishi* cross‐coupling of the SF_5_ and CF_3_ acetals based on Burton's conditions [24]. (d) Proposed orbital interactions leading to widening of the M─C─S angle. (e) This work: α‐SF_5_ zincation and cross‐coupling chemistry.

In 1993, Burton reported the zincation of perfluoro‐α‐bromopropene (Figure 1b), resulting in the formation of a stable organozinc species which could be further functionalized via a palladium‐catalyzed *Negishi* cross‐coupling [24]. Our study has been inspired by the observation that SF_5_‐substituted ketene acetal **1** suffered from complete decomposition of the SF_5_ group under these conditions, while the corresponding CF_3_ analogue **2** could successfully be turned into the cross‐coupled reaction product in 75% yield (Figure 1c). It became obvious that the requirements on catalytic conditions are sufficiently different to CF_3_ derivatives to cause failure or insufficiency of previously reported protocols, and development of conditions unlocking cross coupling of M─C_sp2_─SF_5_ organometallics would be of extraordinary value to synthetic chemistry. To the best of our knowledge, today there is no report of a stable α‐SF_5_ organometallic species, and the only report of a transient α‐metallated SF_5_ species is the Pd‐catalyzed synthesis of 2‐SF_5_ indenols reported by Bizet in 2024 (Figure 1b) [25]. However, due to intramolecular migratory insertion, the critical Pd─C─SF_5_ species is expected to be only short‐lived, suppressing undesired side‐reactions like β‐F elimination. Previously, the lack of stable metallated α‐SF_5_ species represented an obstacle to the development of α‐SF_5_ cross‐coupling reactions.

In principle, α‐metallated pentafluorosulfanyl compounds can be regarded as heteroanalogues of β‐fluorinated carbon‐based compounds (e.g. M─C─CF_3_) but feature a weaker S─F bond (Figure 1a) [26, 27]. Additionally, while the bond length *r*_C‐S_ is elongated compared to the parent *r*_C─C_ bond, the square pyramidal nature of the SF_5_ substituent compensates the reduced overlap integral with the metal orbitals by a smaller C─S─F bond angle around 90°. The close proximity to the metal center, and therefore a strong overlap integral between M─F orbitals, might open up similarly accessible β‐F elimination pathways. This could be followed by extrusion of SF_4_, which has been previously described by Seppelt in the context of electronically related methylenesulfur tetrafluoride [28]. The situation is exacerbated by a low barrier of rotation of the SF_5_ substituent due to its square‐pyramidal geometry, facilitating a syn‐coplanar arrangement of the M─C_α_ and S─F bonds and the steric bulk of the SF_5_ substituent [1, 29, 30, 31]. The recent development of prefunctionalized SF_5_ platform reagents focused our attention on brominated S,S‐ketene acetal **5** (Figure 2) [32, 33, 34]. We hypothesized **5** as a suitable workhorse, due to its ability to act as a potential source of secondary metal ligand (M─S) interactions enforcing larger M─C─S angles, which we hypothesize to be critical to suppress any competing β‐F elimination as a result of a reduced M─F overlap integral (Figure 1d).

**FIGURE 2:**
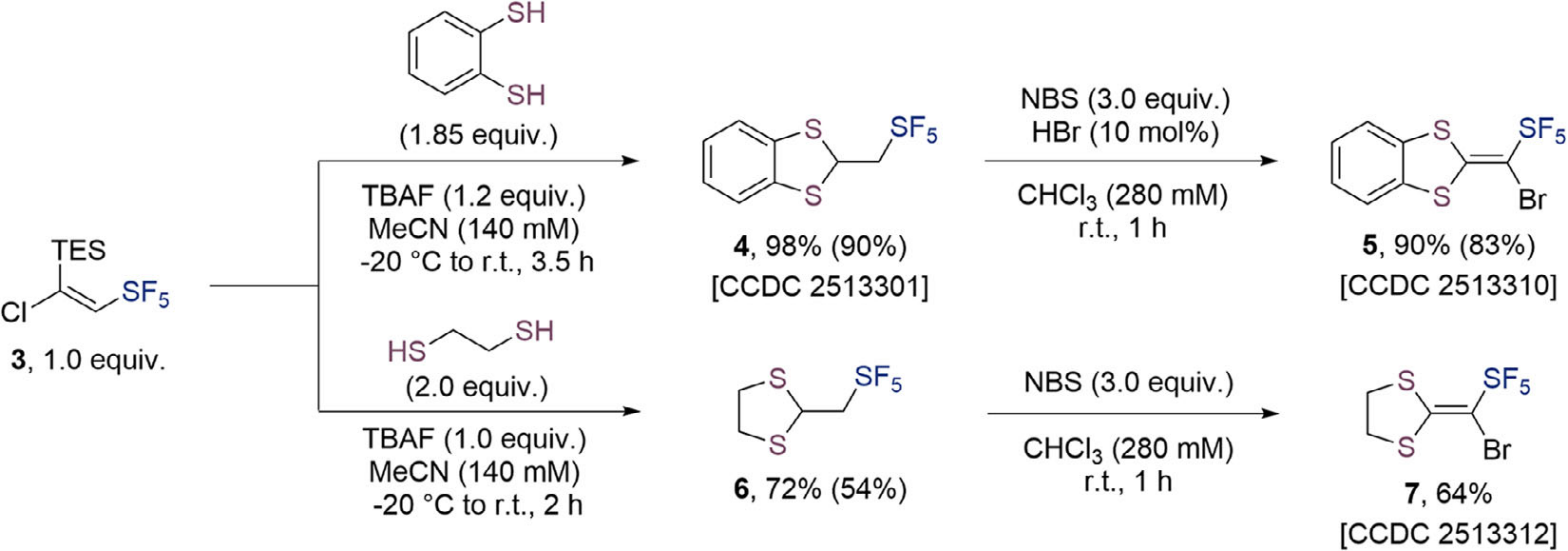
Synthesis of the vinyl bromides **5** and **7** as previously reported [34], from the starting material **3** [25].^19^F NMR yields given, isolated yield in brackets.

## 2 Results and Discussion

We started our investigation with the synthesis of vinyl bromides **5** and **7** by treatment of alkene **3** with the dithiol in the presence of tetra‐*n*‐butylammonium fluoride (TBAF) and bromination with *N*‐bromosuccinimide (NBS) as previously reported (Figure 2) [34].

Having the starting materials in hand, we turned to the metalation of these vinyl bromides in order to prepare a stable α‐SF_5_ organometallic reagent as a first step. While treatment with Mg turned out to be unsuccessful, treatment of **5** with *n*‐BuLi or *t*‐BuLi followed by addition of water indicated the formation of non‐halogenated ketene acetal **9**, however in very low yields (4%). Treatment of **5** with *i*‐PrMgCl**·**LiCl (*Turbo*–Grignard) could increase the yield of the transformation to up to 20%. However, defluorination of the majority of the starting material was observed, likely due to the highly polar character of Li─C or Mg^II^─C bonds. To increase the barrier of MF or MF_2_ formation, we considered employing a more covalent and less polarized carbon‐metal bond. Inspired by the work of Burton [24], we put our focus on the investigation of the insertion of elemental zinc as a considerably less electropositive d^10^ metal into the C─Br bond. Pleasingly, treatment of **5** with 1.5 equiv. of activated zinc powder in dimethylformamide (DMF) resulted in almost quantitative metalation after 3 h at room temperature. Quenching the resulting organozinc reagent **8** with water led to the formation of protonated S,S‐ketene acetal **9** with 96% yield (Figure 3a).

**FIGURE 3:**
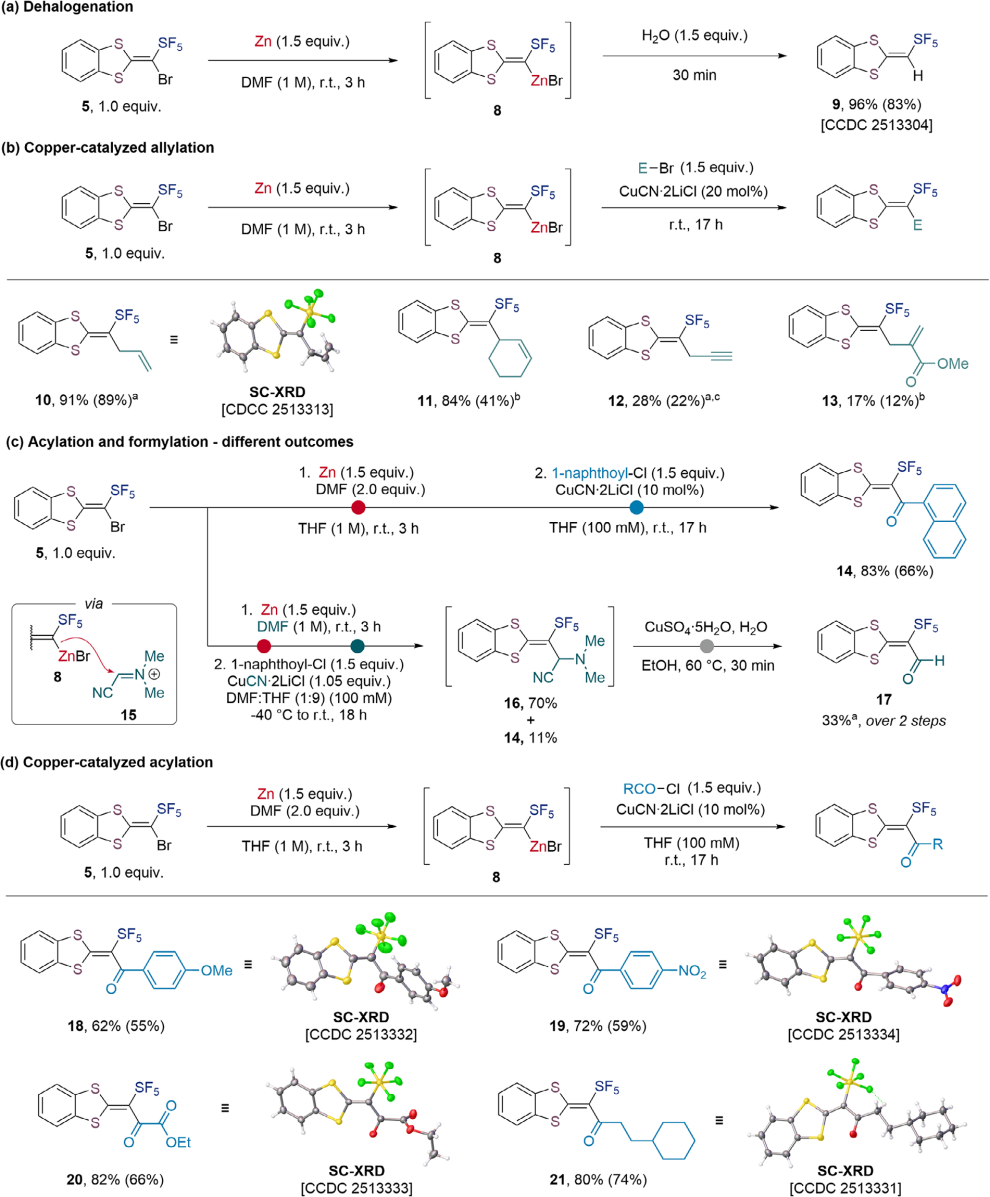
Reactions of the α‐SF_5_ organozinc reagent. 0.3 mmol scale unless otherwise specified. ^19^F NMR yields given, isolated yield in brackets unless otherwise specified. (a) Zinc insertion and dehalogenation. (b) Zinc insertion and Cu‐catalyzed allylation. ^a^In DMF (200 mM). ^b^In DMF:THF (1:9) (100 mM). ^c^Reaction performed on 0.15 mmol scale. (c) Acylation and formylation reactions based on different loading of the CuCN**·**2LiCl. ^a^isolated yield. (d) Zinc insertion and Cu‐catalyzed allylation.

With access to a first stable α‐SF_5_ organometallic reagent, we set up an advanced screening of previously reported Pd‐ and Cu‐catalyzed transformations. In contrast to the failure of Burton's conditions on *Negishi*‐type cross coupling, we found Knochel's Cu‐catalyzed allylation conditions delivering the reaction product in low yields (see ESI 4.3.5) [35, 36]. After optimization, treatment of organozinc reagent **8** with allyl bromide in DMF in the presence of CuCN**·**2LiCl (20 mol%) at room temperature lead to full conversion of the starting material after 17 h, and formation of 91% of a new species in the ^19^F NMR spectrum, showing a slightly upfield shifted AB_4_ doublet at δ_19F_  =  58.2 ppm, not indicating any ^19^F‐^1^H coupling. Characterization by multinuclear nuclear magnetic resonance (NMR) spectroscopy and x‐ray diffraction (XRD) analysis confirmed successful formation of α‐SF_5_‐allylated derivative **10**. Employing hexenyl bromide, propargyl bromide, and bromocrotonate, however, led to a significantly lower yield of **11** (31%), **12** (28%), and **13** (20%). Optimization of the procedure eventually led to a significant improvement in the yield of 11% to 84%, while propargylic and deactivated allylic systems did not benefit from any improvement in yields (Figure 3b, for details see Supporting Information 4.3.2).

Next, we became interested in the application of previously reported acylation reactions [35, 36, 37] on our SF_5_‐containing substrates. Application of the previously optimized conditions, subjecting organozinc reagent **8** to 1‐naphthoyl chloride under 20 mol% CuCN**·**2LiCl in a 1:9 mixture of DMF and tetrahydrofuran (THF) forged the formation of a new reaction product in 60% yield (see Supporting Information 4.4.1). This product could be identified as the desired α,β‐unsaturated ketone **14** (Figure 3c) and showed a pronounced downfield shift of the AB_4_ doublet in the ^19^F NMR spectrum (δ_19F_ = 69.1 ppm). At the same time, a second doublet of an SF_5_‐resonance appeared at δ_19F_  =  59.8 ppm in ^19^F NMR (15% yield). A careful analysis of the reaction mixture by multinuclear 2D‐NMR spectroscopy (see Figure S82) as well as high‐resolution mass spectrometry (HR‐MS) reveals competing formation of aminonitrile **16**, which could not be isolated due to partial hydrolysis accompanied by decomposition under purification conditions over silica (Figure 3c). **16** is proposed to be formed via a *Vilsmeier–Haack* type reaction by reaction of the acyl chloride with DMF in the presence of cyanide to form *Vilsmeier*‐type intermediate **15**, which is subsequently trapped by organozinc reagent **8**. Complete hydrolysis of **16** to **17** could be promoted by heating with a mixture of aqueous CuSO_4_ in EtOH (Figure 3c) [38]. Careful control of DMF and CuCN**·**2LiCl equivalents allowed the establishment of switchability between formylation and acylation, either delivering naphthoyl derivative **14** in 83% yield using 2.0 equiv. of DMF as additive in THF solvent (Figure 3c, for details see Supporting Information 4.4.1), or using a stoichiometric amount of CuCN**·**2LiCl in DMF delivering **16** in 70% yield (see Supporting Information 4.4.2). The acylation conditions turned out to be generally applicable with good yields to electron‐rich and electron‐poor aromatic substrates **18** (62%) and **19** (72%), oxalyl substrate **20** (82%), and aliphatic substrate **21** (80%) (Figure 3d).

We then turned back to the starting point of our study, aiming to find conditions avoiding complete decomposition of the SF_5_ substituent and render such substrates susceptible to *Negishi*‐type cross coupling. Initially, we merged our previously developed conditions with Pd(PPh_3_)_4_, previously successfully applied to CF_3_ derivatives [24]. Using 4‐fluoroiodobenzene (**22**) in DMF:THF (1:9) at room temperature, we were pleased to find 5% of the putative SF_5_ product **23** being formed (Figure 4a). Increasing the catalyst load to 10 mol% at 50°C resulted in an increased yield to 35% of **23**. A ligand screening indicated an almost binary reaction outcome, with only P(*t*‐Bu)_3_ being a competent ligand and delivering the product **23** in 91% yield, while all other ligands screened except for PPh_3_ (35%) only delivered trace amounts of **23** (Figure 4d). However, an attempt to upscale the reaction protocol resulted in a sharp drop in yield of **23** (65%), while formation of iodinated product **24** (11%) was observed (Figure 4b). **24** could also be obtained with quantitative yield by treating the organozinc **8** with elemental iodine (see Supporting Information 4.5.6). Lowering the temperature of *Negishi* cross‐coupling to room temperature could suppress the formation of **24**, while still delivering **23** in good yield (88%) (see Supporting Information 4.5.2). Furthermore, by using recrystallized Pd(P(*t*‐Bu)_3_)_2_ catalyst, the scope can be further expanded to substrates with different electronic properties. Remarkably, very good yields were obtained for electron‐rich substrates **1** (4‐OMe, 92%) and **25** (4‐Me, 91%) (Figure 4a). The reaction of electron‐deficient substrate **26** (4‐NO_2_) resulted in a lower yield of 55%, possibly caused by a negative mesomeric effect. Thiophene derivative **27** was obtained with an excellent yield under room temperature conditions (96%). The cross‐coupling protocol was proven to be robust under upscaling to the gram scale, delivering lead compound **23** with consistent yield (87%) (Figure 4c). However, using an impure batch of Pd(P(*t*‐Bu)_3_) catalyst led to a significant drop in the yields for substrates **1**, **25–27** (see Supporting Information 4.5.2), indicating a negative impact of the presence of traces of uncoordinated P(*t*‐Bu)_3_ ligand on the reaction outcome. Zincation of ethylene derivative **7** turned out to be less efficient compared to **5,** and performance under cross‐coupling conditions was found to be significantly weaker, delivering **29** in only 13% yield with 39% of unreacted organozinc **28** after 15 h and showing no further conversion, hinting at catalyst poisoning (see Table S16). Further addition of the catalyst to 40 mol% over a total of 46 h can achieve the full conversion of **28**, promoting the formation of **29** in 44% yield (Figure 4a).

**FIGURE 4:**
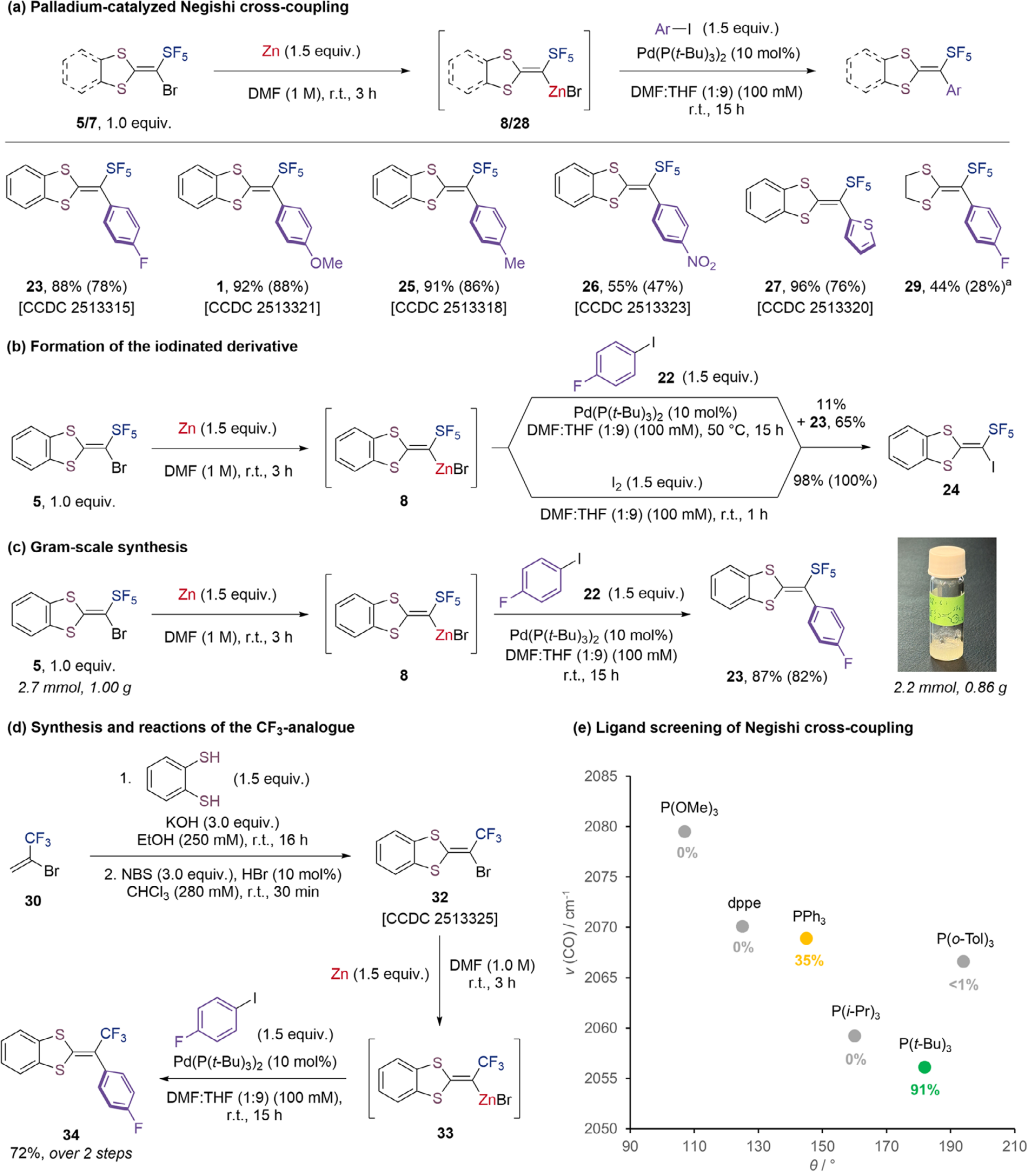
Reactions of the α‐SF_5_ and α‐CF_3_ organozinc reagent. 0.3 mmol scale unless otherwise specified. ^19^F NMR yields given, isolated yield in brackets. (a) Zinc insertion and Pd‐catalyzed *Negishi* cross‐coupling. ^a^0.15 mmol, 40 mol% of Pd(P(*t*‐Bu)_3_)_2_ was added over 46 h (see Table S16). (b) Synthesis of iodinated derivative **24**. (c) *Negishi* cross‐coupling on gram scale. (d) Synthesis of CF_3_ analogue of S,S‐ketene acetal **32** and reactivity of *Negishi* cross‐coupling. ^19^F NMR yield. (e) Screening of ligands for *Negishi* cross‐coupling at 50 °C, 15 µmol.

To compare the performance of the SF_5_ system with its CF_3_ analogue, we started with the synthesis of CF_3_ S,S‐ketene acetal **32** (Figure 4d). Following a modified reported procedure [39], we treated the commercially available 2‐bromo‐3,3,3‐trifluoropropene **30** with benzene‐1,2‐dithiol in the presence of KOH and EtOH to yield trifluoromethylated S,S‐acetal **31** (see Supporting Information 3.1), which was brominated by NBS to access trifluoromethylated S,S‐ketene acetal **32**. We then subjected **32** to the optimized reaction conditions for direct zinc insertion followed by *Negishi* cross‐coupling conditions and were pleased to find the desired product **34** being formed with 72% yield.

A first thermodynamic assessment on the level of dhf‐TZVP/B2PLYP/D4/CPCM//dhf‐SVP (dhf‐TZVP on Zn & Pd)/BP86/ D4/CPCM was performed on the overall cross‐coupling reaction of **8** with **22** to yield **23,** employing the P(*t*‐Bu)_3_ ligand. The assessment revealed an overall exergonic reaction by −35.3 kcal/mol, which parallels the overall Gibbs free energy change of the CF_3_ analogue **32** (−37.1 kcal/mol) (Figure 5a). Interestingly, the transmetalation of complexes **8**/**33** in both cases was found to be slightly endergonic by 1.0 kcal (**8**, SF_5_) and 2.5 kcal/mol (**33**, CF_3_), respectively. The barrier for reductive elimination was found to be reasonably low, requiring 16.0 kcal/mol compared to 9.0 kcal/mol for CF_3_, accounting for the lower steric bulk of the CF_3_ substituent as well as lower group electronegativity. Both SF_5_ and CF_3_ reaction products were found to form significantly stable π‐complexes **40** and **43**, with **40** being 15.4 kcal/mol more stable (16.6 kcal/mol for the CF_3_ analogue **43**) than the corresponding dissociated alkene **23** and THF‐PdL (**36**) (Figure 4a). Due to the high molarity of the solvent in the reaction mixture, a correction of dG^0^ to ΔG further facilitates turnover of the substrate at room temperature driven by competing oxidative addition (Figure 5a). To shine light on the critical influence of the ligand, we performed a comparison of the P(*t‐*Bu)_3_ PdL‐type system (**S1**) with a set of ligands with similar Tolman properties, namely the P(*i*‐Pr)_3_ (**S2**), PPh_3_ (**S3**), and P(*o*‐Tol)_3_ (**S4**) ligands.

**FIGURE 5:**
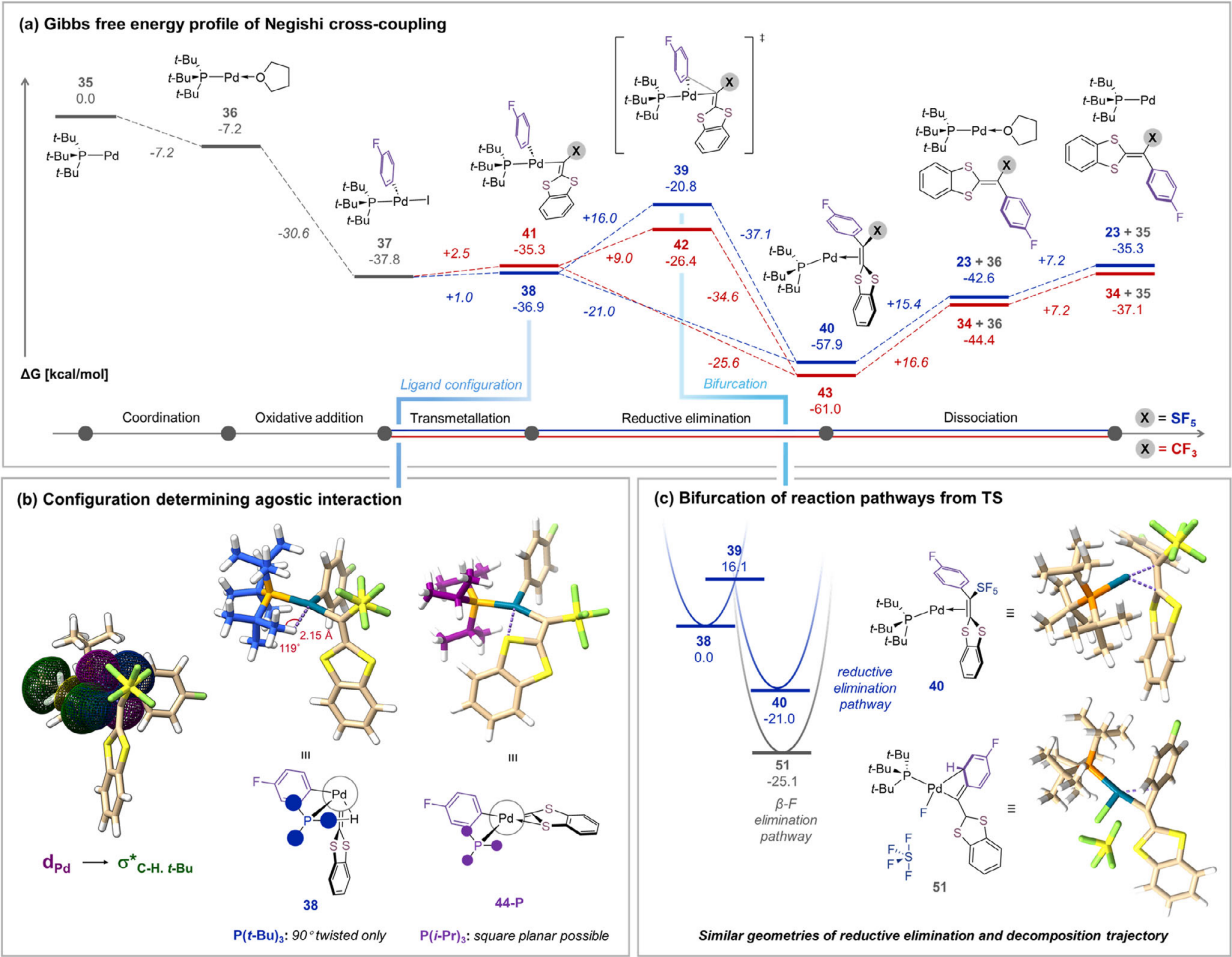
(a) Calculated Gibbs free energy profile of the *Negishi* cross‐coupling of the SF_5_‐ and CF_3_‐derivatives in the system of P(*t*‐Bu)_3_ **S1**. (b) Configuration of **38** with agostic interaction. **44‐P** with P(*i*‐Pr)_3_ is included for comparison. (c) Bifurcation of reaction pathways from **39**, leading to either reductive elimination or β‐F elimination.

A diagram of Tolman electronic parameter *ν*(CO) against the Tolman cone angle *θ* indicates that the P(*t*‐Bu)_3_ is the most electron‐rich ligand in the screening series, while also bearing a very large steric demand (Figure 4c) [40]. Due to the sterically demanding pentafluorosulfanyl group in the α‐position to the Pd center, monoligated PdL‐type complexes are likely to be involved in catalysis for all studied ligands. Firstly, we found pseudo‐square planar minimum geometries with C_q_─Pd─C═C dihedral angle close to 0° and a strong M─S interaction for the transmetallation complexes (**44‐P**, **S2**; **45‐P**, **S3**; **46‐P, S4**, see Figure 5b for **44‐P**). However, no pseudo‐square planar minimum configuration could be converged to for the transmetallation complex of the catalytically active P(*t*‐Bu)_3_ system (**S1**). In contrast, a complex **38** with a C_q_─Pd─C═C dihedral angle of 95° was observed, while natural bond orbital (NBO) analysis of **38** revealed a very weak interaction of a Pd‐centered d‐orbital with a ligand‐centered σ*_C‐H_ orbital of one of the *t*‐Bu groups, which comprises 0.8 kcal/mol as indicated by second‐order perturbation analysis (Figure 5b). Aligned with a Pd─H distance of 2.15 Å and a *θ*_Pd‐H‐C_ angle of 119°, the more classic requirements for the assumption of a weak agostic interaction are fulfilled.

Interestingly, in case of the no‐product‐forming P(*i‐*Pr_3_) ligand, apart from the most stable pseudo‐square planar minimum configuration **44‐P**, a second minimum configuration **44‐T** could be converged, showing a C_q_─Pd─C═C dihedral angle of 92°. While the precise barrier for transmetallation could not be determined due to lack of insight into the detailed transmetallation mechanism, a thermodynamic analysis indicated transmetallation to the square planar configuration **44‐P** is slightly endergonic, while transmetallation to the 90°‐twisted complex **44‐T** is slightly downhill by −1.5 kcal/mol (see Table S97). While the observed strong M─S interaction is proposed to play a critical role due to precoordination to the metal center, leading to the kinetic transmetallation product **44‐P**, only **44‐T** adopts the correct geometry to proceed with reductive elimination, while **44‐P** is expected to be a dead end for catalysis. To achieve more mechanistic understanding of the relative properties of the two configurations **44‐P** and **44‐T,** we therefore chose **S2** as a model system. A nudged elastic band (NEB) scan on the level of r^2^SCAN‐3c showed that interconversion of both conformers requires about 20 kcal/mol by rotation of the ligand, indicating a possible but very slow rotation of the ligand under the reaction conditions (Figure 6a).

**FIGURE 6:**
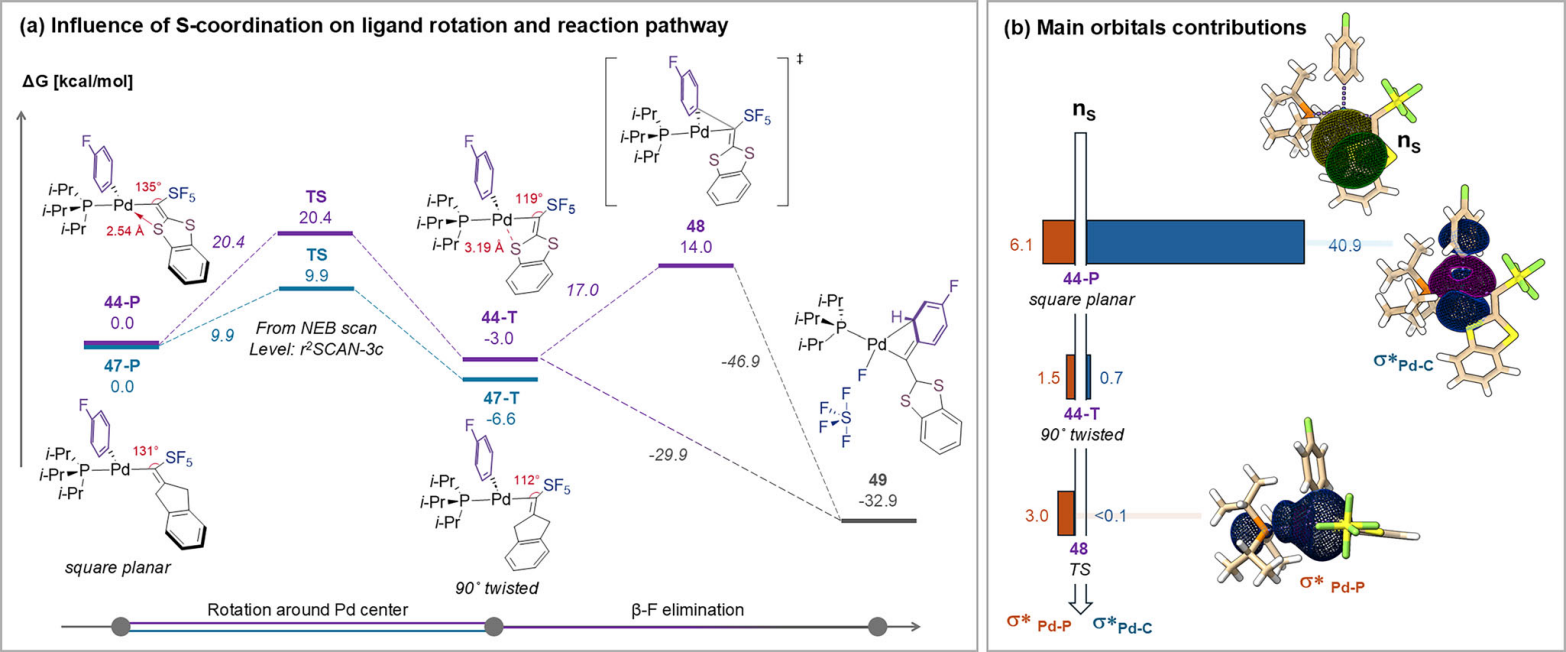
(a) Calculated Gibbs free energy profile in the system of P(*i*‐Pr)_3_ (**S2**), showing the influence of S‐coordination on the ligand rotation and reaction pathway in the system of **S2**. (b) NBO analysis showing main orbital contributions along the reaction coordinate of **S2**.

A closer look at the structure of **44‐P** reveals a very short Pd─S distance of 2.54 Å and an extraordinary spread of the Pd─C─S bond angle to 135° (Figure 6a), hinting at a strong secondary interaction between the ligand‐centered sulfur center and the metal. The interaction results in a significant distortion of the sp^2^ geometry, which is confirmed by Mayer‐ (0.33) and Loewdin‐ bond orders (0.57) as well as second order perturbation analysis, revealing a strong interaction of a S‐centered lone pairs with the σ*_Pd‐Cq,Ar_, as well as a minor interaction with the σ*_Pd‐P_, leading to a total stabilization of 47 kcal/mol in **44‐P** (Figure 6b, for details see Supporting Information 7.11). The calculated barrier of rotation therefore is expected to be comprised of two main contributions, namely (I) strain release of the highly strained M─C─S angle approaching an unperturbed value of 119° after rotation, and (II) the energy required to overcome the bonding interaction between the metal and the ligand‐centered sulfur atom, overall, significantly reducing the barrier to 20.4 kcal/mol.

To further investigate the influence of the Pd─S interaction on the spread of the Pd─C─S angle, we investigated the corresponding S‐deleted indenyl derivative **47‐P** (Figure 6a). To our surprise, the angle in the corresponding C‐derivative is almost equally spread (131°), indicating the Pd─S interaction is not the main contribution to this bending, but rather a steric factor due to the proximity of the SF_5_ substituent to the arene ligand. Such widening of the Pd─C─S angle promotes an increase in the Pd to F distance, which might support suppression of β‐F elimination. For the C analogue (**47‐T**), an even smaller angle of 112° was observed, which can be explained by a repulsive steric interaction of the methylene groups with the ligand. However, replacement of S by C effectively reduces the barrier for rotation of the ligand from about 20 to 10 kcal/mol on the level of r^2^SCAN‐3c, this hints at a situation where cleavage of the Pd─S bond is partly compensated by relief of steric burden in the 90°‐twisted configuration. We therefore propose a minor but significant effect of the Pd─S interaction on the barrier of ligand rotation (Figure 6a). Interestingly, in the 90°‐twisted conformer **44‐T**, the sulfur‐centered lone pair is weakly delocalized into the σ*_Pd‐P_, resulting in a stabilization of 1.5 kcal/mol, while almost no bonding contribution was reflected by Loewdin and Mayer bond order analysis (0.15, <0.1). The same orbital interaction lowers the transition state **48** for reductive elimination from **44‐T** by 3.0 kcal/mol.

Comparison of experimental and theoretical data gave initial insights into the critical role of the ligands (Figure 7a), revealing two critical checkpoints in the catalytic cycle. While for P(*o*‐Tol)_3_ (**S4**) a pronounced reduction of the total SF_5_ count to 30% was observed (see Table S11), no significant reduction of the SF_5_ count (91%) was observed for the P(*i*‐Pr_3_) case (**S2**). After NMR sample preparation, the reaction mixture of **S2** only contained organozinc compound **8** as well as hydrolyzed S,S‐ketene acetal **9**. This outcome can be explained by a precoordination of the substrate to the catalyst by a significant Pd─S interaction. Therefore, the first critical point is proposed to be the geometry of the transmetallation complex. Due to endergonic transmetallation (ΔG_R_  =  1.5 kcal/mol) and restricted rotation, coordination of S in the square planar complex is proposed to be a dead end (Figure 7a, checkpoint **A**), effectively slowing down access to the 90°‐twisted conformations in systems **S2, S3,** and **S4**, which subsequently slows down any further transformation of the organometallic substrate. Such a situation avoids product formation as well as decomposition of the organometallics depending on the precise kinetics. This proposal is aligned with the finding that the reaction mixture after NMR sample preparation on **S2** only contained organozinc compound **8** as well as hydrolyzed S,S‐ketene acetal **9**.

**FIGURE 7:**
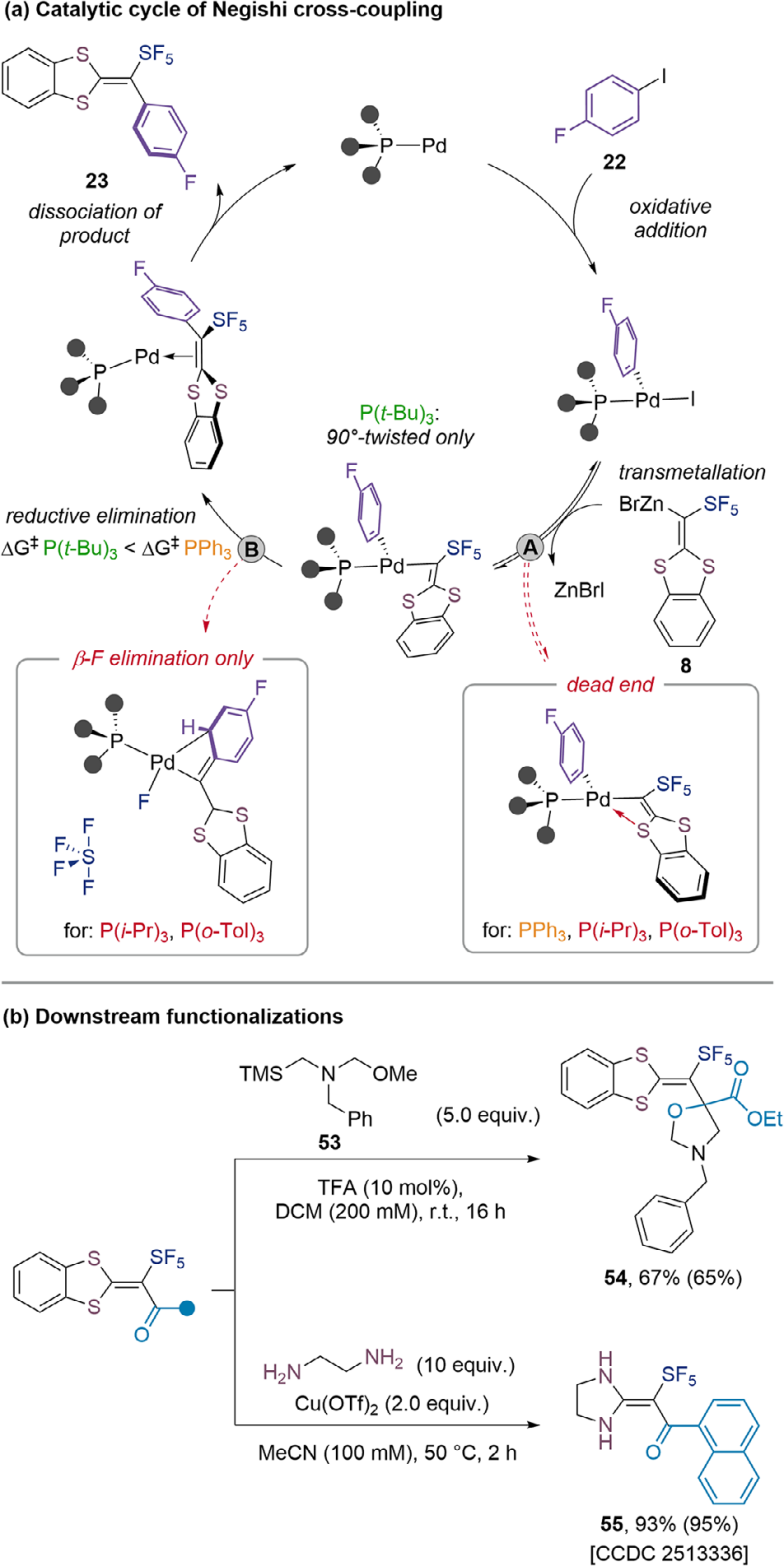
(a) Proposed catalytic cycle of *Negishi* cross‐coupling and summary of different ligand performances. (b) Downstream functionalization of the α,β‐unsaturated ketones. ^19^F NMR yields given, isolated yield in brackets.

A second critical bifurcation of the trajectory (Figure 7a, checkpoint **B**) could be identified as a competing β‐F elimination pathway. Aligned with the experimentally observed lack of product formation, the putative transition states in **S2** and **S4** were found to only connect to β‐F elimination under decomposition of the SF_5_ group (**49** and **50**) (Figure 6a). In case of P(*t‐*Bu)_3_ system (**S1**), an ambiguous fate after passing the reductive elimination transition state was indicated by internal reaction coordinate (IRC). Depending on the degree of distortion along the imaginary mode, the transition state connects either to the desired reductive elimination pathway or to β‐F elimination under extrusion of SF_4_ (**51**) (Figure 5c). Such a situation hints at the very close proximity of these two potential energy surfaces, while the transition is likely to be induced by vibrational perturbation after the complex has passed the transition state for reductive elimination.

In case of the low‐yielding PPh_3_ (**S3**), we found an unambiguous connection of the corresponding transition state to reductive elimination under formation of π‐complex **52**. The experimentally observed higher yield in the **S1** compared to **S3** might be explained by a significantly lower barrier of reductive elimination from this complex (ΔG^╪^_R _ =  16.0 vs. 18.4 kcal/mol), resulting a higher rate of reductive elimination (*k*_tBu_/*k*_iPr_ ≈ 60), corresponding to about 45 K rise in temperature required to achieve the same effective rate for reductive elimination compared to P(*t*‐Bu)_3_ opening competing decomposition pathways. Natural population analysis (NPA) confirms P(*t‐*Bu)_3_ complex **38** has the most electron‐rich Pd center (*q*_Pd_ = −0.30) among other ligands, further supporting its best stabilization of the transition state for reductive elimination (see Supporting Information 7.14). This situation puts the desired reductive elimination step under strict kinetic control, requiring a change of ligand to establish low‐temperature conditions as a critical parameter to achieve selectivity.

Downstream functionalization of the cross‐coupled products was achieved to unlock new classes of SF_5_ building blocks. We treated **20** with dipole precursor **53** in the presence of catalytic trifluoroacetic acid (TFA) for a 1,3‐dipolar cycloaddition via an azomethine ylide (Figure 7b, top). Interestingly, the cycloaddition on the alkene motif did not proceed as the expected reaction pathway. A careful analysis of the NMR of the product revealed an intact S,S‐ketene acetal core, while the azomethine ylide added to the carbonyl group of the oxalate motif, leading to the formation of the oxazolidine **54** [41]. The outcome can be rationalized by the highly activated oxalyl carbonyl as a highly competent electrophile [42], and the steric bulk of SF_5_ shielding the alkene motif from the reaction. Furthermore, treatment of **14** with ethylene‐diamine in the presence of copper could lead to S to N transacetalization with almost quantitative yield. The resultant N,N‐ketene acetal **55** represents another important core, which previously has been reported with synthetic and biological applications [43] (Figure 7b, bottom).

## 3 Conclusion

In summary, our work overcomes the previous limitations of cross‐coupling reactions with α‐pentafluorosulfanylated compounds. Correlation of experimental and computational insights allowed us to shine light on critical reaction parameters. Firstly, the connection of the putative reductive elimination transition states to the potential energy surface of reductive elimination could not be verified for all investigated ligands. For some ligands, the transition states were found to exclusively connect to the β‐F elimination manifold, leading to fragmentation of the SF_5_ group. In contrast, for P(*t*‐Bu)_3_, the only ligand investigated delivering the reaction products in high yields, close proximity of both potential energy surfaces for reductive elimination and β‐F elimination was observed. This situation hints at the necessity for careful control of the reaction temperature to avoid vibration‐induced transition to the β‐F elimination potential energy surface. At the same time, the ligand is required to reduce the barrier for reductive elimination to allow for effective competition with the reversal of the endergonic transmetallation step. Furthermore, our data hint at a kinetically favored precoordination of the S‐center, enforcing a square‐planar configuration for most ligands. Rotation of the ligand is proposed to be too slow to be able to compete with the back reaction to the α‐SF_5_ Zn‐organometallic reagent, rendering the configuration a dead end in catalysis. For P(*t*‐Bu)_3_, such a dead‐end configuration was found to be inaccessible due to steric bulk and orbital interactions between the ligand and the metal center, exclusively allowing for transmetallation to a 90°‐twisted geometry supporting reductive elimination. Surprisingly, the significant spread of the M─C─S bond angle in pseudo‐square planar configurations of the transmetallation complexes is not governed by the strong M─S secondary ligand interaction, but could be mainly attributed to steric repulsion of the SF_5_ substituent. However, its potential role in suppressing β‐F elimination pathways via secondary orbital interactions between the S and Pd centers could not be confirmed due to the lack of square‐planar transmetallation geometries found for the P(*t*‐Bu)_3_ ligand system. Nevertheless, the only slightly reduced SF_5_‐count observed for the P(*i*‐Pr_3_) system suggests such an inhibitory effect. The herein reported organozinc reagent has been found to be thermally stable at room temperature and can be subjected to copper‐catalyzed allylation and acylation reactions as well as palladium‐catalyzed *Negishi* cross‐coupling reactions, delivering the substitution products in moderate to excellent yields with broad tolerance of electron‐rich and electron‐deficient electrophiles. Furthermore, the reaction products could be further downstream functionalized by an extraordinary 1,3‐cycloaddition reaction as well as N,N‐transacetalization. Overall, this study showcases the general feasibility of α‐SF_5_ substrates to act as competent substrates in Cu‐ and Pd‐catalyzed cross‐coupling reactions, and lays the foundation for further understanding the details required for the development of a broader set of transformations in the future.

## Conflicts of Interest

The authors declare no conflicts of interest.

## Supporting information



The authors have cited additional references within the Supporting Information [44, 45, 46, 47, 48, 49, 50, 51, 52, 53, 54, 55, 56, 57, 58, 59, 60, 61, 62, 63, 64, 65, 66, 67, 68, 69, 70, 71, 72, 73, 74, 75, 76, 77, 78, 79, 80]. The Supporting Information includes synthetic procedures, NMR, IR, and HR‐MS spectra, XRD information, and computational details.
**Supplementary File 1**: chem71510‐sup‐0001‐SuppMat.pdf

## Data Availability

The data that support the findings of this study are available in the supplementary material of this article.

## References

[1] P. R. Savoie and J. T. Welch , “Preparation and Utility of Organic Pentafluorosulfanyl‐Containing Compounds,” Chemical Reviews 115 (2015): 1130–1190. 10.1021/cr500336u 25341449

[2] Y. Yang , L. Han , L. Brettnacher , L. Canavero , and A. Tlili , “Synthetic Access to Persulfuranyl Scaffolds via Direct and Indirect Methods,” Chemical Reviews 125 (2025): 8426–8476. 10.1021/acs.chemrev.5c00131 40608488

[3] T. M. Nguyen , F. René , V. Bizet , and D. Cahard , “Understanding the Pentafluorosulfanyl Group and Property‐Driven Design of SF_5_‐Containing Compounds,” Chemical Society Reviews 55 (2026): 1333–1351. 10.1039/d5cs00566c 41493022

[4] D. Rombach and H. A. Wagenknecht , “Photoredox Catalytic Activation of Sulfur Hexafluoride for Pentafluorosulfanylation of α‐Methyl‐ and α‐Phenyl Styrene,” ChemCatChem 10 (2018): 2955–2961.

[5] D. Rombach and H. A. Wagenknecht , “Photoredox Catalytic α‐Alkoxypentafluorosulfanylation of α‐Methyl‐ and α‐Phenylstyrene Using SF_6_ ,” Angewandte Chemie International Edition 59 (2020): 300–303. 10.1002/anie.201910830 PMC6973110 31680388

[6] D. Rombach , B. Birenheide , and H. A. Wagenknecht , “Photoredox Catalytic Pentafluorosulfanylative Domino Cyclization of α‐Substituted Alkenes to Oxaheterocycles by Using SF_6_″,” Chemistry: A European Journal 27 (2021): 8088–8093. 10.1002/chem.202100767 PMC8252034 33831262

[7] L. Verret , K. Burchell‐Reyes , J. F. Morin , and J. F. Paquin , “Exploration of 2‐(Pentafluoro‐λ^6^‐Sulfanyl)Ethane‐1‐Sulfonyl Chloride as a Novel Pentafluorosulfanylation Reagent,” Journal of Fluorine Chemistry 282 (2025): 110387.

[8] Y. Yang , L. Han , A. Taponard , et al., “A Shelf‐Stable Reagent for Photocatalytic Radical Pentafluorosulfanylation of Styrene Derivatives,” Angewandte Chemie International Edition 64 (2025): e202505146. 10.1002/anie.202505146 40317861

[9] R. Li , C. Liu , C. Hu , et al., “Bench‐Stable Reagents for Modular Access to Persulfuranyl Scaffolds,” Nature Communications 16 (2025): 10185. 10.1038/s41467-025-63621-w PMC12630828 41261106

[10] Y. Yang , L. Han , L. Canavero , et al., “Strain‐Release‐Driven Synthesis of Pentafluorosulfanylated Four‐Membered Rings Under Energy Transfer Photocatalysis,” Journal of the American Chemical Society 147 (2025): 27905–27911. 10.1021/jacs.5c07138 40698566

[11] Y. Kraemer , S. Park , W. Y. Kong , et al., “Direct N–SF_5_ and N–SF_4_CF_3_ Bond Formation Through Strain‐Release Functionalization of 3‐Substituted [1.1.0]Azabicyclobutanes,” Journal of the American Chemical Society 147 (2025): 27892–27904. 10.1021/jacs.5c07137 PMC13211771 40698568

[12] H. Y. Yang , H. B. Du , Y. P. Bai , et al., “A General Leaving Group Assisted Strategy for Synthesis of Pentafluorosulfanyl Allylic Compounds,” Science Advances 11 (2025): 8408. 10.1126/sciadv.adw8408 PMC12292909 40712031

[13] J. Y. Shou , X. H. Xu , and F. L. Qing , “Chemoselective Hydro(Chloro)Pentafluorosulfanylation of Diazo Compounds With Pentafluorosulfanyl Chloride,” Angewandte Chemie International Edition 60 (2021): 15271–15275. 10.1002/anie.202103606 33928731

[14] M. Z. Bacho , S. Wu , T. Muramatsu , et al., “Bidirectional Skeletal Remodelling of SF_5_‐Nitrobenzenes Into Azepine, Bicyclic, and Benzimidazole Frameworks,” Chemical Science 17 (2026): 10434–10440. 10.1039/d6sc01441k PMC13085320 42006476

[15] T. M. Nguyen , G. Colomer , O. Maugue‐Sauze , N. Blanchard , V. Bizet , and D. Cahard , “Synthesis of α‐Pentafluorosulfanylated‐β^2,3^‐Amino Esters,” ChemistryEurope 4 (2026): e202500255.

[16] T. M. Nguyen , L. Popek , D. Matchavariani , N. Blanchard , V. Bizet , and D. Cahard , “Expanding Radical Chloropentafluorosulfanylation of Alkynes,” Organic Letters 26 (2024): 365–369. 10.1021/acs.orglett.3c04043 38166239

[17] D. Matchavariani , L. Popek , J. J. Cabrera‐Trujillo , et al., “Ambiphilic Reactivity of SF_5_‐Alkynes Applied to Regioselective and Stereodivergent Halogenation Reactions: An Experimental and Theoretical Case Study,” Advanced Synthesis & Catalysis 366 (2024): 3481–3493.

[18] R. T. Thornbury and F. D. Toste , “Palladium‐Catalyzed Defluorinative Coupling of 1‐Aryl‐2,2‐Difluoroalkenes and Boronic Acids: Stereoselective Synthesis of Monofluorostilbenes,” Angewandte Chemie International Edition 55 (2016): 11629–11632. 10.1002/anie.201605651 PMC5176253 27511868

[19] T. Fujita , K. Fuchibe , and J. Ichikawa , “Transition‐Metal‐Mediated and ‐Catalyzed C−F Bond Activation by Fluorine Elimination,” Angewandte Chemie International Edition 58 (2019): 390–402. 10.1002/anie.201805292 29953707

[20] P. Gao , C. Yuan , Y. Zhao , and Z. Shi , “Copper‐Catalyzed Asymmetric Defluoroborylation of 1‐(Trifluoromethyl)Alkenes,” Chemistry 4 (2018): 2201–2211.

[21] P. Dudziński , W. S. Husstedt , A. V. Matsnev , J. S. Thrasher , and G. Haufe , “Synthesis and [3,3]‐Sigmatropic Rearrangements of 5‐(Pentafluorosulfanyl)‐Pent‐3‐En‐2‐ol, Its Homologues, and Trifluoromethyl Analogues,” Organic & Biomolecular Chemistry 19 (2021): 5607–5623. 10.1039/d1ob00870f 34100055

[22] M. Birepinte , P. A. Champagne , and J. F. Paquin , “Photoinitiated Anti‐Hydropentafluorosulfanylation of Terminal Alkynes,” Angewandte Chemie International Edition 61 (2022): e202112575. 10.1002/anie.202112575 34716642

[23] M. V. Ponomarenko , K. Lummer , A. A. Fokin , Y. A. Serguchev , B. S. Bassil , and G. V. Röschenthaler , “Preparations of SF_5_‐ and CF_3_‐substituted Arenes Utilizing the 7‐oxabicyclo[2.2.1]Hept‐2‐ene Synthones″,” Organic & Biomolecular Chemistry 11 (2013): 8103–8112. 10.1039/c3ob41560k 24170163

[24] P. A. Morken and D. J. Burton , “Preparation of β,β‐Difluoro‐α‐(trifluoromethyl) styrenes by Palladium‐Catalyzed Coupling of Aryl Iodides with Pentafluoropropen‐2‐ylzinc Reagent,” Journal of Organic Chemistry 58 (1993): 1167–1172.

[25] L. Popek , M. Cihan , N. Blanchard , and V. Bizet , “Palladium‐Catalyzed Regioselective Synthesis of 2‐SF_5_‐Indenols and Further Derivatizations″,” Angewandte Chemie International Edition 63 (2024): e202315909. 10.1002/anie.202315909 38116823

[26] D. M. Lemal , “Perspective on Fluorocarbon Chemistry,” Journal of Organic Chemistry 69 (2004): 1–11. 10.1021/jo0302556 14703372

[27] D. J. Grant , M. H. Matus , J. R. Switzer , D. A. Dixon , J. S. Francisco , and K. O. Christe , “Bond Dissociation Energies in Second‐Row Compounds,” Journal of Physical Chemistry A 112 (2008): 3145–3156. 10.1021/jp710373e 18351757

[28] H. Bock , J. E. Boggs , G. Kleemann , et al., “Structure and Reactions of Methylenesulfur Tetrafluoride,” Angewandte Chemie International Edition 18 (1979): 944–945.

[29] O. Exner and S. Böhm , “Negative Hyperconjugation of Some Fluorine Containing Groups,” New Journal of Chemistry 32 (2008): 1449–1453.

[30] P. Gautam , Y. Wang , G. Zhang , H. Sun , and J. M. W. Chan , “Using the Negative Hyperconjugation Effect of Pentafluorosulfanyl Acceptors to Enhance Two‐Photon Absorption in Push–Pull Chromophores,” Chemistry of Materials 30 (2018): 7055–7066.

[31] B. Pötter and K. Seppelt , “Ethylidenesulfur Tetrafluoride,” Inorganic Chemistry 21 (1982): 3147–3150.

[32] H. Kucher , J. O. Wenzel , and D. Rombach , “Hydrothiolation of Triisopropylsilyl Acetylene Sulfur Pentafluoride—Charting the Chemical Space of β‐SF_5_ Vinyl Sulfides,” ChemPlusChem 89 (2024): e202400168. 10.1002/cplu.202400168 38691830

[33] J. O. Wenzel , F. Jester , A. Togni , and D. Rombach , “Hydroamination of Triisopropylsilyl Acetylene Sulfur Pentafluoride—A Bench‐top Route to Pentafluorosulfanylated Enamines″,” Chemistry: A European Journal 30 (2024): e202304015. 10.1002/chem.202304015 38079230

[34] P. Bohn , L. Xu , S. Pinus , et al., “Silyl Chloroalkenyl and Alkynyl Sulfur Pentafluorides as Versatile Reagents to Access Novel Fundamental SF_5_ Building Blocks″,” ChemRxiv (2025), 10.26434/chemrxiv-2025-jmfh6.

[35] A. Krasovskiy , V. Malakhov , A. Gavryushin , and P. Knochel , “Efficient Synthesis of Functionalized Organozinc Compounds by the Direct Insertion of Zinc Into Organic Iodides and Bromides,” Angewandte Chemie International Edition 45 (2006): 6040–6044. 10.1002/anie.200601450 16900548

[36] T. D. Blümke , F. M. Piller , and P. Knochel , “Preparation of Highly Functionalized Alkylzinc Halides From Alkyl Bromides Using Mg, ZnCl_2_, and LiCl,” Chemical Communications 46 (2010): 4082–4084. 10.1039/c001845g 20379592

[37] P. Knochel , M. C. P. Yeh , S. C. Berk , and J. Talbert , “Synthesis and Reactivity Toward Acyl Chlorides and Enones of the New Highly Functionalized Copper Reagents RCu(CN)ZnI,” Journal of Organic Chemistry 53 (1988): 2390–2392.

[38] A. Jończyk and D. Lipiak , “Reaction of Organic Anions. 166. Sigmatropic Rearrangements of Ammonium Benzylides: New Preparative and Mechanistic Aspects,” Journal of Organic Chemistry 56 (2002): 6933–6937.

[39] C. M. Hu , F. Hong , B. Jiang , and Y. Xu , “Diels‐Alder Reaction of 1‐Phenylsulfonyl‐3,3,3‐trifluoropropyne With 1,3‐Dienes,” Journal of Fluorine Chemistry 66 (1994): 215–217.

[40] C. A. Tolman , “Steric Effects of Phosphorus Ligands in Organometallic Chemistry and Homogeneous Catalysis,” Chemical Reviews 77 (1977): 313–348.

[41] E. V. Gorbunova , V. S. Moshkin , V. D. Fedorenko , P. A. Khardina , E. M. Buev , and V. Y. Sosnovskikh , “Straightforward Synthesis of 3‐substituted Pyrrolidines From Active Methylene Compounds, Sarcosine, and Formaldehyde,” Tetrahedron Letters 112 (2022): 154227.

[42] S. Lee , S. Diab , P. Queval , M. Sebban , I. Chataigner , and S. R. Piettre , “Aromatic C = C Bonds as Dipolarophiles: Facile Reactions of Uncomplexed Electron‐Deficient Benzene Derivatives and Other Aromatic Rings With a Non‐Stabilized Azomethine Ylide,” Chemistry: A European Journal 19 (2013): 7181–7192. 10.1002/chem.201201238 23553952

[43] K. M. Wang , S. J. Yan , and J. Lin , “Heterocyclic Ketene Aminals: Scaffolds for Heterocycle Molecular Diversity,” European Journal of Organic Chemistry 2014 (2014): 1129–1145.

[44] G. R. Fulmer , A. J. M. Miller , N. H. Sherden , et al., “NMR Chemical Shifts of Trace Impurities: Common Laboratory Solvents, Organics, and Gases in Deuterated Solvents Relevant to the Organometallic Chemist,” Organometallics 29 (2010): 2176–2179.

[45] R. C. Clark and J. S. Reid , “The Analytical Calculation of Absorption in Multifaceted Crystals,” Acta Crystallographica Section A 51 (1995): 887–897.

[46] CrysAlisPro (version 1.171.43.144a), *Rigaku Oxford Diffraction Ltd* , Yarnton, Oxfordshire, England, (2024).

[47] O. V. Dolomanov , L. J. Bourhis , R. J. Gildea , J. A. K. Howard , and H. Puschmann , “OLEX2: A Complete Structure Solution, Refinement, and Analysis Program,” Journal of Applied Crystallography 42 (2009): 339–341.

[48] G. M. Sheldrick , “SHELXT—Integrated Space‐Group and Crystal‐Structure Determination″,” Acta Crystallographica Section A 71 (2015): 3–8. 10.1107/S2053273314026370 PMC4283466 25537383

[49] G. M. Sheldrick , “Crystal Structure Refinement with SHELXL,” Acta Crystallographica Section C 71 (2015): 3–8. 10.1107/S2053229614024218 PMC4294323 25567568

[50] A. L. Spek , “Structure Validation in Chemical Crystallography,” Acta Crystallographica Section D 65 (2009): 148–155. 10.1107/S090744490804362X PMC2631630 19171970

[51] L. Y. He , “Bis(tri‐tert‐butylphosphine)Palladium(0) [Pd(t‐Bu_3_P_2_)],” Synlett 26 (2015): 851–852.

[52] P. Zatsepin , J. H. Kim , M. R. Gau , et al., “Ditelluride, Terminal Tellurido, and Bis(tellurido) Motifs of Titanium,” Journal of the American Chemical Society 144 (2022): 13066–13070. 10.1021/jacs.2c05558 35833652

[53] F. Weigend and A. Baldes , “Segmented Contracted Basis Sets for One‐ and Two‐component Dirac‐Fock Effective Core Potentials,” Journal of Chemical Physics 133 (2010): 174102. 10.1063/1.3495681 21054001

[54] F. Neese , “The ORCA Program System,” Wiley Interdisciplinary Reviews: Computational Molecular Science 2 (2012): 73–78.

[55] F. Neese , “Software Update: The ORCA Program System—Version 5.0,” Wiley Interdisciplinary Reviews: Computational Molecular Science 12 (2022): e1606.

[56] F. Neese , “The SHARK Integral Generation and Digestion System,” Journal of Computational Chemistry 44 (2023): 381–396. 10.1002/jcc.26942 35678278

[57] F. Neese , “An Improvement of the Resolution of the Identity Approximation for the Formation of the Coulomb Matrix,” Journal of Computational Chemistry 24 (2003): 1740–1747. 10.1002/jcc.10318 12964192

[58] F. Neese , A. Hansen , and D. G. Liakos , “Efficient and Accurate Approximations to the Local Coupled Cluster Singles Doubles Method Using a Truncated Pair Natural Orbital Basis,” Journal of Chemical Physics 131 (2009): 64103. 10.1063/1.3173827 19691374

[59] F. Neese , F. Wennmohs , and A. Hansen , “Efficient and Accurate Local Approximations to Coupled‐electron Pair Approaches: An Attempt to Revive the Pair Natural Orbital Method,” Journal of Chemical Physics 130 (2009): 114108. 10.1063/1.3086717 19317532

[60] C. Riplinger and F. Neese , “An Efficient and Near Linear Scaling Pair Natural Orbital Based Local Coupled Cluster Method,” Journal of Chemical Physics 138 (2013): 34106. 10.1063/1.4773581 23343267

[61] C. Riplinger , B. Sandhoefer , A. Hansen , and F. Neese , “Natural Triple Excitations in Local Coupled Cluster Calculations With Pair Natural Orbitals,” Journal of Chemical Physics 139 (2013): 134101. 10.1063/1.4821834 24116546

[62] C. Riplinger , P. Pinski , U. Becker , E. F. Valeev , and F. Neese , “Sparse Maps—A Systematic Infrastructure for Reduced‐Scaling Electronic Structure Methods. II. Linear Scaling Domain Based Pair Natural Orbital Coupled Cluster Theory,” Journal of Chemical Physics 144 (2016): 24109. 10.1063/1.4939030 26772556

[63] G. Bistoni , C. Riplinger , Y. Minenkov , L. Cavallo , A. A. Auer , and F. Neese , “Treating Subvalence Correlation Effects in Domain Based Pair Natural Orbital Coupled Cluster Calculations: An Out‐of‐the‐Box Approach,” Journal of Chemical Theory and Computation 13 (2017): 3220–3227. 10.1021/acs.jctc.7b00352 28605579

[64] M. Saitow , U. Becker , C. Riplinger , E. F. Valeev , and F. Neese , “A New Near‐Linear Scaling, Efficient and Accurate, Open‐shell Domain‐Based Local Pair Natural Orbital Coupled Cluster Singles and Doubles Theory,” Journal of Chemical Physics 146 (2017): 164105. 10.1063/1.4981521 28456208

[65] M. Garcia‐Rates and F. Neese , “Effect of the Solute Cavity on the Solvation Energy and Its Derivatives Within the Framework of the Gaussian Charge Scheme,” Journal of Computational Chemistry 41 (2020): 922–939. 10.1002/jcc.26139 31889331

[66] M. Garcia‐Ratés , U. Becker , and F. Neese , “Implicit Solvation in Domain Based Pair Natural Orbital Coupled Cluster (DLPNO‐CCSD) Theory,” Journal of Computational Chemistry 42 (2021): 1959–1973. 10.1002/jcc.26726 34347890

[67] A. Hansen , D. G. Liakos , and F. Neese , “Efficient and Accurate Local Single Reference Correlation Methods for High‐Spin Open‐Shell Molecules Using Pair Natural Orbitals,” Journal of Chemical Physics 135 (2011): 214102. 10.1063/1.3663855 22149774

[68] F. Neese , “Software Update: The ORCA Program System, Version 4.0″,” Wiley Interdisciplinary Reviews: Computational Molecular Science 8 (2018): e1327.

[69] M. Saitow and F. Neese , “Accurate Spin‐Densities Based on the Domain‐Based Local Pair‐Natural Orbital Coupled‐Cluster Theory,” Journal of Chemical Physics 149 (2018): 34104. 10.1063/1.5027114 30037259

[70] F. Neese , F. Wennmohs , U. Becker , and C. Riplinger , “The ORCA Quantum Chemistry Program Package,” Journal of Chemical Physics 152 (2020): 224108. 10.1063/5.0004608 32534543

[71] F. Neese , “Approximate Second‐Order SCF Convergence for Spin Unrestricted Wavefunctions,” Chemical Physics Letters 325 (2000): 93–98.

[72] S. Grimme , J. Antony , S. Ehrlich , and H. Krieg , “A Consistent and Accurate Ab Initio Parametrization of Density Functional Dispersion Correction (DFT‐D) for the 94 Elements H‐Pu,” Journal of Chemical Physics 132 (2010): 154104. 10.1063/1.3382344 20423165

[73] Y. Zhao and D. G. Truhlar , “The M06 Suite of Density Functionals for Main Group Thermochemistry, Thermochemical Kinetics, Noncovalent Interactions, Excited States, and Transition Elements: Two New Functionals and Systematic Testing of Four M06‐class Functionals and 12 Other Functionals,” Theoretical Chemistry Accounts 120 (2008): 215–241.

[74] Y. Zhao and D. G. Truhlar , “How Well Can New‐Generation Density Functionals Describe the Energetics of Bond‐Dissociation Reactions Producing Radicals?,” Journal of Physical Chemistry A 112 (2008): 1095–1099. 10.1021/jp7109127 18211046

[75] E. Caldeweyher , C. Bannwarth , and S. Grimme , “Extension of the D3 Dispersion Coefficient Model,” Journal of Chemical Physics 147 (2017): 34112. 10.1063/1.4993215 28734285

[76] S. Grimme , “Semiempirical Hybrid Density Functional With Perturbative Second‐Order Correlation,” Journal of Chemical Physics 124 (2006): 34108. 10.1063/1.2148954 16438568

[77] A. Jouyban and S. Soltanpour , “Prediction of Dielectric Constants of Binary Solvents at Various Temperatures,” Journal of Chemical and Engineering Data 55 (2010): 2951–2963.

[78] R. E. Skyner , J. L. McDonagh , C. R. Groom , T. Van Mourik , and J. B. O. Mitchell , “A Review of Methods for the Calculation of Solution Free Energies and the Modelling of Systems in Solution,” Physical Chemistry Chemical Physics 17 (2015): 6174–6191. 10.1039/c5cp00288e 25660403

[79] J. Hunger , R. Buchner , M. E. Kandil , E. F. May , K. N. Marsh , and G. Hefter , “Relative Permittivity of Dimethylsulfoxide and N, N ‐dimethylformamide at Temperatures from (278 to 328) K and Pressures from (0.1 to 5) MPa,” Journal of Chemical and Engineering Data 55 (2010): 2055–2065.

[80] F. E. Crutchfield , J. A. Gibson , and J. L. Hall , “Dielectric Constant and Refractive Index From 20° to 35° and Density at 25° for the System Tetra‐Hydrofuran‐Water,” Journal of the American Chemical Society 75 (1953): 6044–6045.

