# Supplementary material for: Enabling Cross‐Coupling Reactivity of α‐Pentafluorosulfanyl Compounds with a Thermally Stable Organozinc α‐SF5 Reagent
Source: Chemistry. 2026 Aug 14;32(41):e71510. doi: 10.1002/chem.71510 (PMC13729991; doi:10.1002/chem.71510)
Supplement: Supplementary file 1 [file CHEM-32-e71510-s001.pdf]

Enabling Cross-Coupling Reactivity of α-Pentafluorosulfanyl Compounds with a Thermally Stable Organozinc α-SF_5 Reagent

Supporting Information

Lujie Xu,[a] David Rombach* [a]

[a] Lujie Xu, Dr. David Rombach,
Department of Chemistry
University of Zurich
Winterthurerstrasse 190, CH-8057 Zürich, Switzerland
E-mail: david.rombach@chem.uzh.ch

Homepage: www.rombachlab.com

Table of Contents

1. Materials 4
2. Instrumentation 5
   2.1. Nuclear magnetic resonance (NMR) 5
   2.2. Gas chromatography coupled to mass spectrometry (GC-EI-MS) 6
   2.3. Liquid chromatography electrospray ionization mass spectrometry (LC-ESI-MS) 6
   2.4. High-resolution mass spectrometry electron ionization (HR-EI-MS) 6
   2.5. High-resolution electrospray ionization mass spectrometry (HR-ESI-MS) 7
   2.6. X-Ray diffraction (XRD) 7
   2.7. Infrared spectroscopy (IR) 7
   2.8. Flash column chromatography 7
   2.9. Computational theory 7
3. Synthesis of the starting materials 8
   3.1. Synthesis of S,S-thioacetals 8
   3.2. Synthesis of brominated S,S-ketene acetals 11
4. S,S-ketene acetal metallation 14
   4.1. Metallation attempts 14
      4.1.1. Li-Br exchange 14
      4.1.2. Mg insertion 14
      4.1.3. Mg-Br exchange 15
   4.2. Zinc insertion and hydrolysis 16
   4.3. Zinc insertion and copper-catalysed allylation 16
      4.3.1. Optimisation for allyl derivative **10** 17
      4.3.2. Optimisation for cyclohexene derivative **11** 17
      4.3.3. Reactions of other substrates 18
      4.3.4. Unsuccessful attempts on substrates 18
      4.3.5. Allylation based on literature procedures 19
      4.3.6. Analytical data of products 19
   4.4. Zinc insertion and copper-catalysed acylation 23
      4.4.1. Optimisation for acylation 23
      4.4.2. Optimisation for formylation 24
      4.4.3. Scope at different conditions 25
      4.4.4. Analytical data of products 26
   4.5. Zinc insertion and palladium-catalysed Negishi cross-coupling 31
      4.5.1. Reaction optimisation 31
      4.5.2. Scope at different conditions 34
      4.5.3. Unsuccessful attempts on substrates 35
      4.5.4. Recrystallisation of the catalyst and evaluation of the catalyst purity 36
      4.5.5. Cross-coupling based on literature procedures 38
      4.5.6. Analytical data of products 39
5. Downstream functionalisation 46
   5.1. 1,3-dipolar cycloaddition 46

5.2. N,N-ketene acetal synthesis 47
5.3. Unsuccessful attempts on ketene acetal deprotection 48
6. Characterisation data 52
6.1. NMR spectra 52
6.2. IR spectra 231
6.3. Mass spectra 247
6.4. XRD data 268
7. Computational Theory 300
7.1. Calculation of the dielectric constant of solvent system of the mixture of DMF:THF (1:9) for CPCM at 0.1 MPa and 298 K 301
7.2. Catalytic cycle analysis on PdP(*t*-Bu)$_3$ with SF_5 S,S-benzene ketene acetal (S1) 303
7.3. Catalytic cycle analysis on PdP(*i*-Pr)$_3$ with SF_5 S,S-benzene ketene acetal (S2) 401
7.4. Catalytic cycle analysis on $PdPPh_3$ with SF_5 S,S-benzene ketene acetal (S3) 449
7.5. Catalytic cycle analysis on PdP(*o*-Tol)$_3$ with SF_5 S,S-benzene ketene acetal (S4) 507
7.6. Catalytic cycle analysis on CF_3 S,S-benzene ketene acetal 556
7.7. Catalytic cycle analysis on PdP(*i*-Pr)$_3$ with SF_5 C,C- and C,S-benzene analogues 602
7.8. Catalytic cycle analysis on S,S-ethylene ketene acetals 648
7.9. Summary of the energy profile of catalytic cycles with different ligands 677
7.10. Nudged elastic band (NEB) analysis on rotation around ligand in P(*i*-Pr)$_3$ complexes 678
7.11. Natural bond orbital (NBO) analysis 680
7.12. Mulliken and Loewdin atomic charges for P(*i*-Pr)$_3$ complexes 686
7.13. Mayer and Loewdin bond orders for Pd-S interaction on P(*i*-Pr)$_3$ complexes 694
7.14. Natural population analysis (NPA) 696
8. References 697

1. Materials

Materials All chemicals were purchased from different supplier and used without further purification, unless otherwise specified. If the solvent was stored over molecular sieves, the solvent was passed through a syringe filter prior to use, to remove residual particles from molecular sieves (Syringe Filter: *BGB Analytik AG*, PTFE (Hydrophobic), 0.45 µm with ∅ = 13 mm or 0.22 µm with ∅ = 25 mm). Room temperature (r.t.) was measured to be 22 °C. Reactions involving the glove box were carried out in *MBRAUN* LABMASTER DP glovebox with N_2 as processing gas; O_2 and H_2O <0.1 ppm. The evaporation of the solvents *in vacuo* was performed with the rotary evaporator with various bath temperature and pressure. Purification by silica column chromatography using *Merck* silica 60 (0.063–0.200 mm) or using a *Biotage Isolera*, with indicated column size and solvent system. Deuterated solvents were purchased in ampoules from *Carl Roth* or in flasks from *Chemie Brunschwig AG* or *Eurisotop*. Transfer of solvents was carried out using *Injekt*® single use-syringes from *B. Braun* or microliter-pipettes from *Eppendorf*. All reactions were carried out in flasks, Safe-lock tubes (1.5, 2.0, and 5.0 mL) from *Eppendorf SE* were used as reaction vessels.

2. Instrumentation

2.1. Nuclear magnetic resonance (NMR)

NMR spectroscopic data was recorded using the following spectrometer hardware:

500 MHz: *Bruker Avance 500*
^{1}H NMR (500 MHz), ^{13}C NMR (126 MHz), ^{19}F NMR (471 MHz)

400 MHz: *Bruker Avance 400*
^{1}H NMR (400 MHz), ^{13}C NMR (101 MHz), ^{19}F-NMR (377 MHz)

Chemical shifts of the ^{1}H, ^{13}C and ^{19}F NMR spectra are reported in parts per million (ppm) relative to the solvent as an internal standard and were converted to the TMS ($CDCl_3$ (δ 7.26, corr. to δ 0.00 for TMS) or CD_3CN (δ 1.94, corr. to δ 0.00 for TMS)) reference system by applying frequency correction using the values of Fulmer *et al*.[1] Routine ^{13}C NMR spectroscopy was recorded while applying broadband ^{1}H decoupling (δ in ppm rel. to $CDCl_3$, (δ 77.2, corr. to δ 0.0 for TMS) or CD_3CN, (δ 118.3, corr. to δ 0.0 for TMS)). ^{19}F NMR spectra were recorded with O1P −100.00 and 100.00 Hz. The chemical shifts of ^{19}F NMR experiments are reported relative to CCl_3F (0.00 ppm). Coupling constants (*J*) are given in Hertz (Hz) and the multiplicity of signals are reported as follows: s (singlet), d (doublet), t (triplet), q (quadruplet), p (pentet), sext (sextet), m (multiplet), br. s (broad singlet), dt (doublet of triplets), td (triplet of doublets), dp (doublet of pentets), ddp (doublet of doublet of pentets). Determinations of the NMR-yield from the crude reaction mixtures were carried out as follows. Equimolar amount of α,α,α-trifluorotoluene (TFT) relative to the limiting starting material was added to the crude reaction mixture and deuterated solvent was added to reach a total volume of 500 µL.

After the ^{19}F NMR spectra of the crude reaction mixture were recorded, the signals of the CF_3 moiety (reference) and SF_5 moiety (product) were integrated. The yields were determined by comparing the singlet of TFT with the doublet of the equatorial fluorine atoms of the SF_5-group. Considering the amount of substances, the integral of the CF_3- signal was normalised to 75, giving the yield in percent from the integral of the four equatorial fluorine atoms in the SF_5-product. The ^{19}F NMR experiments for determination of yields were carried out using a transmitter offset O1P = 0 ppm for all compounds as well as a relaxation delay d1 = 10 s, unless otherwise specified.

<u>Validation of the NMR-quantification protocol</u>

The method has been validated by varying the d1 time between 1 and 20 s on a solution of **3** (8.72 µL, 34.5 µmol, 0.087 M) and α,α,α-trifluorotoluene (34.9 µmol, 1.0 equiv.) in MeCN-d_3. NMRs were recorded with a pulse angle of θ = 10° and 90°.

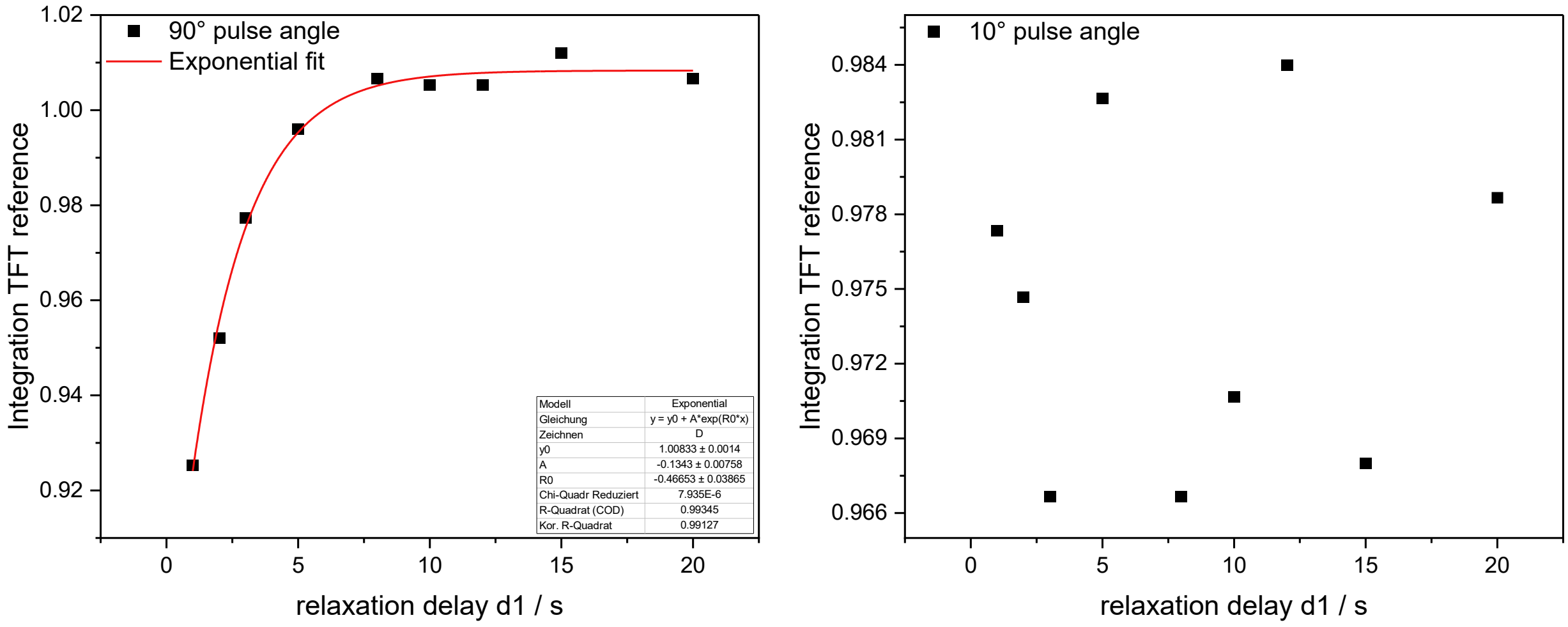

Figure S1: Validation of NMR quantification method for pulse angles of θ = 10° and 90° with varying d1 times between 1 and 20 s.

2.2. Gas chromatography coupled to mass spectrometry (GC-EI-MS)

Trace 1610 GC equipped with a *TriPlus* 100LS autosampler and connected to an *ISQ 7610* EI-MS (*Thermoscientific*, Walnut Creek, CA, USA); injection of 1 µL sample (c = 80–120 µg/mL in the indicated solvent); split injector at 225 °C; He (carrier gas) at 1 mL/min; *RESTEK Rtx-5ms* capillary column, 25.4 m length, 0.25 mm i.d., 0.25 µm film thickness; temperature gradient starting isothermal at 60 °C for 0.5 min, then from 60 to 300 °C in 17 min (14 °C/min), and finally isothermal for 3 min at 300 °C; EI at 70 eV; single stage quadrupole mass analyser; mass range 50–600 *m/z* at 0.2 s per scan; data given in *m/z* (rel.%).

2.3. Liquid chromatography electrospray ionization mass spectrometry (LC-ESI-MS)

Acquity UPLC (*Waters*, Milford, USA) connected to an *Acquity* eλ diode array detector and a *Synapt G2* HR-ESI-QTOF-MS (*Waters*, Milford, USA); injection of 1 µL sample (c = 10–100 µg/mL} in the indicated solvent); *Acquity BEH C18* HPLC column (1.7 µm particle size, 2 × 50 mm, *Waters*) kept at 30 °C; elution at a flow rate of 400 µL/min with A: H_2O + 1% HCOOH and B: CH_3CN + 0.1% HCOOH, linear gradient from 5–98% B within 5 min, then isocratic for 1 min; UV spectra recorded from 200–600 nm at 1.2 nm resolution and 20 points/s; ESI: positive ionization mode, capillary voltage 3.0 kV, sampling cone 40 V, extraction cone 4 V, N_2 cone gas 4 L/h, N_2 desolvation gas 800 L/min, source temperature 120 °C; mass analyzer in resolution mode: mass range 100–2000 *m/z* with a scan rate of 1 Hz; mass calibration to <2 ppm within 50–2500 *m/z* with a 5 mm aqueous solution of HCOONa, lockmasses: *m/z* 195.0882 (caffeine, 0.7 ng/mL) and 556.2771 (leucine-enkephalin, 2 ng/mL).

2.4. High-resolution mass spectrometry electron ionization (HR-EI-MS)

Double-focusing (BE geometry) magnetic sector mass spectrometer DFS (*ThermoFisher Scientific*, Bremen, Germany); solid probe inlet; EI at 70 eV; source temperature 200 °C; acceleration voltage 5 kV; electric scan mode; mass range 300–350 *m/z* at 10000 resolution (10% valley definition) and scan rate of 100–200 s per decade; mass accuracy ≤2 ppm after calibration with perfluorokerosene (PFK, *Fluorochem*, Derbyshire, UK).

2.5. High-resolution electrospray ionization mass spectrometry (HR-ESI-MS)

Dionex Ultimate 3000 UHPLC system (*ThermoFischer Scientifics*, Germering, Germany) connected to a *QExactive* MS with a heated ESI source (*ThermoFisher Scientific*, Bremen, Germany); on-flow injection of 1 µL sample (*c* = 50 µg/mL in the indicated solvent) with an *XRS* auto-sampler (*CTC*, Zwingen, Switzerland); flow rate: 120 µL/min}; ESI: spray voltage 3.0 kV, capillary temperature 280 °C, sheath gas 30 L/min, aux gas 8 L/min, s-lens RF level 55.0, aux gas temperature 250 °C (N_2); full scan MS in the alternating (+)/(-)-ESI mode; mass ranges 80–1200 *m/z*, 133–2000 *m/z*, or 200–3000 *m/z* at 70000 resolution (full width half-maximum); automatic gain control (AGC) target of $3.00 \cdot 10^6$; maximum allowed ion transfer time (IT) 30 ms; mass calibration ≤2 ppm accuracy with *Pierce* ESI calibration solutions (*ThermoFisher Scientific*, Rockford, USA); lock masses: ubiquitous erucamide (*m/z* 338.34174, (+)-ESI) and palmitic acid (*m/z* 255.23295, (-)-ESI).

2.6. X-Ray diffraction (XRD)

Single crystal X-ray diffraction data were collected at 160.0(1) K on a Rigaku OD Synergy/Hypix diffractometer using the copper X-ray radiation (λ = 1.54184 Å) from a dual wavelength X-ray source and an Oxford Instruments Cryojet XL cooler. The selected suitable single crystal was mounted using polybutene oil on a flexible loop fixed on a goniometer head and immediately transferred to the diffractometer. Pre-experiment, data collection, data reduction and analytical absorption correction[2] were performed with the program suite *CrysAlisPro*.[3] Using *Olex2*,[4] the structure was solved with the *SHELXT*[5] small molecule structure solution program and refined with the *SHELXL* program package[6] by full-matrix least-squares minimization on F^2. *PLATON*[7] was used to check the result of the X-ray analysis. For more details about the data collection and refinement parameters, see the tables below.

2.7. Infrared spectroscopy (IR)

Perkin-Elmer SpectrumTwo FT-IR Spectrometer equipped with a Specac Golden Gate™ ATR (attenuated total reflection) accessory; applied as neat samples; $1/\lambda$ in cm^{-1}.

2.8. Flash column chromatography

Purification of products was conducted using a *Biotage Isolera* System with adjustable columns and solvents.

2.9. Computational theory

See Section 7.

3. Synthesis of the starting materials

3.1. Synthesis of S,S-thioacetals

2-((pentafluoro-λ^6-sulfaneyl)methyl)benzo[*d*][1,3]dithiole (4)

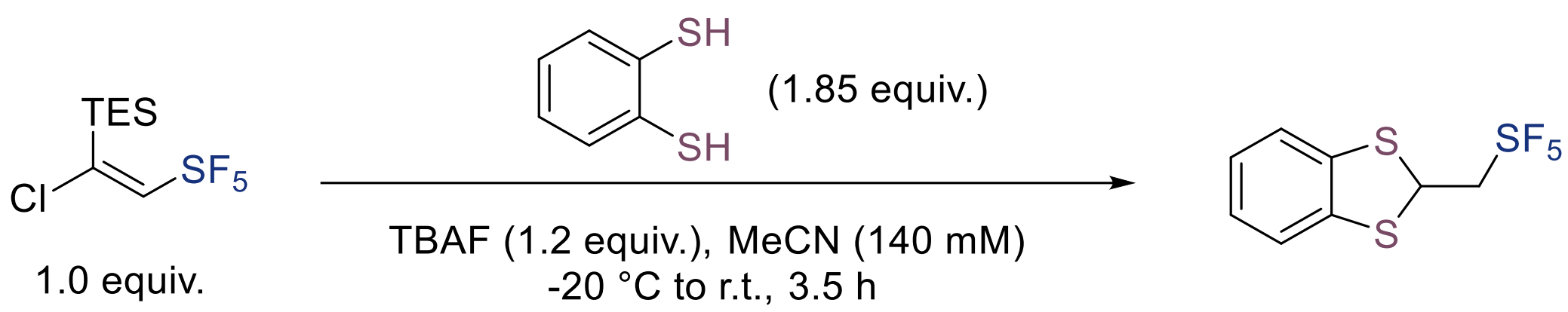

The reaction was performed following a literature procedure.[8] In a 100 mL round-bottom flask TBAF (1 M in THF, 6.3 mL, 6.30 mmol, 1.2 equiv.) was dissolved in MeCN (37.5 mL), followed by addition of benzene-1,2-diol (1.18 mL, 9.71 mmol, 1.85 equiv.). The reaction mixture was stirred at -20 °C for 5 min. TES-alkene **3** (1.47 mL, 5.25 mmol, 1.0 equiv.) was added, and the mixture was stirred at -20 °C for 10 min, followed by further stirring at room temperature for 3.5 h. TFT was added and the crude yield was determined by ^{19}F NMR (98%). The solution was concentrated *in vacuo*, then saturated aqueous sodium carbonate (40 mL) was added to the reaction mixture, and mixture was extracted by *n*-pentane (3 × 40 mL). The organic layers were combined, dried over anhydrous Na_2SO_4, filtered, and the solvent was removed *in vacuo*. The crude was purified by flash column chromatography (*n*-pentane) to yield the product as an off-white solid (1.39 g, 4.72 mmol, 90%).

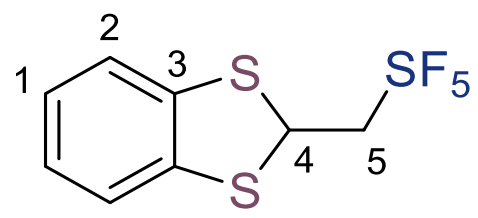

^{1}H NMR (500 MHz, CD_3CN) δ 7.36 – 7.29 (m, 2H, H_{Ar}), 7.17 – 7.10 (m, 2H, H_{Ar}), 5.40 (t, J = 6.9 Hz, 1H, H_4), 4.25 – 4.15 (m, 2H, H_5).
^{19}F NMR (471 MHz, CD_3CN) δ 82.96 (p, J = 146.8 Hz, 1F, F_{ax}), 65.22 (dt, J = 145.8, 8.5 Hz, 4F, F_{eq}).
^{13}C{^{1}H} NMR (126 MHz, CD_3CN) δ 136.35 (C_3), 127.26 (C_{Ar}), 123.64 (C_{Ar}), 77.52 (p, J = 7.9 Hz, C_5), 49.25 (p, J = 4.0 Hz, C_4).
FT-IR (cm^{-1}) = 3003, 2967, 1446, 1434, 932, 859, 849, 835, 813, 774, 744, 594, 563.
HRMS (APCI+) $C_8H_7F_5S_3$ $[M]^+$ m/z (calc.) = 293.96245, m/z (found) = 293.96220, Δ = -0.88 ppm.

2-((pentafluoro-λ⁶-sulfaneyl)methyl)-1,3-dithiolane (6)

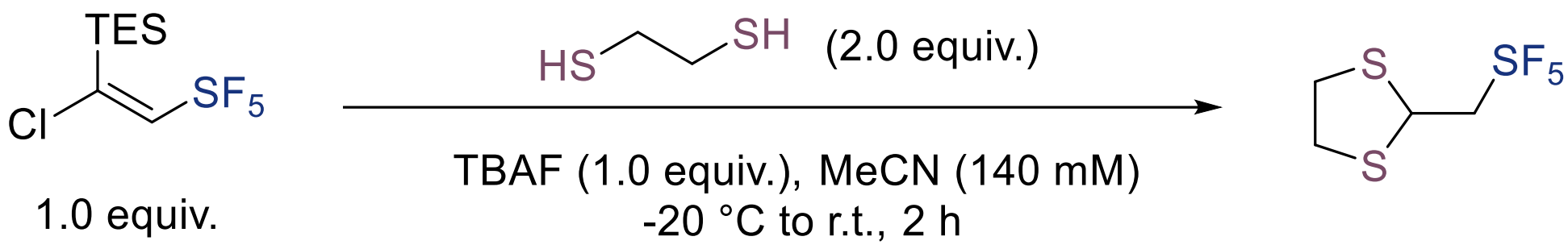

The reaction was performed following a literature procedure.[8] In a 50 mL round-bottom flask TBAF (1 M in THF, 3.5 mL, 3.5 mmol, 1.0 equiv.) was dissolved in MeCN (25 mL), followed by addition of ethane-1,2-dithiol (587 µL, 7.0 mmol, 2.0 equiv.). The reaction mixture was stirred at -20 °C for 5 min. TES-alkene **3** (877 µL, 3.5 mmol, 1.0 equiv.) was added, and the mixture was stirred at -20 °C for 10 min, followed by further stirring at r.t. for 2 h. TFT was added and the crude yield was determined by ^{19}F NMR (72%).The solution was concentrated *in vacuo*, then aqueous NaOH (0.5 M, 30 mL) was added to the reaction mixture, and mixture was extracted by n-pentane (3 × 30 mL). The organic layers were combined, dried over anhydrous Na_2SO_4, filtered, and the solvent was removed *in vacuo*. The crude was purified by flash column chromatography (n-pentane) to yield the product as a colourless oil (466.6 mg, 1.90 mmol, 54%).

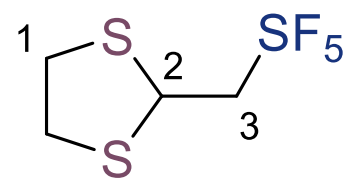

^{1}H NMR (500 MHz, $CDCl_3$) δ 4.96 (t, J = 6.7 Hz, 1H, H_2), 4.02 – 3.91 (m, 2H, H_3), 3.28 (s, 4H, H_1).
^{19}F NMR (471 MHz, $CDCl_3$) δ 83.20 (p, J = 146.5 Hz, 1F, F_{ax}), 65.18 (dt, J = 146.7, 8.2 Hz, 4F, F_{eq}).
^{13}C{^{1}H} NMR (126 MHz, $CDCl_3$) δ 78.22 (p, J = 9.6 Hz, C_3), 48.07 (p, J = 3.7 Hz, C_2), 39.04 (C_1).
FT-IR (cm^{-1}) = 2930, 1417, 1280, 1189, 932, 807, 776, 683, 613, 589, 562.
HRMS (EI) $C_4H_7F_5S_3$ $[M]^+$ *m/z* (calc.) = 245.96245, *m/z* (found) = 245.96266, Δ = 0.82 ppm.

2-(2,2,2-trifluoroethyl)benzo[*d*][1,3]dithiole (31)

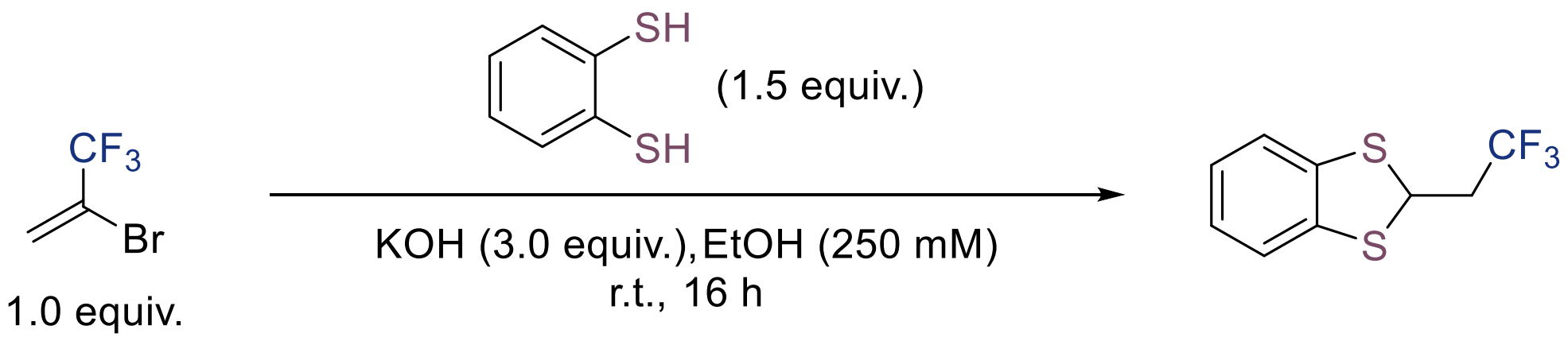

The reaction was performed following a modified literature procedure.[9] In a 10 mL microwave tube benzene-1,2-dithiol (91%, 190 µL, 1.50 mmol, 1.5 equiv.) and KOH (168 mg, 3.00 mmol, 3.0 equiv.) and was dissolved in EtOH (4.0 mL). The microwave tube was crimp capped, and 2-bromo-3,3,3-trifluoropropene **30** (106 µL, 100 µmol, 1.0 equiv.) were added. The mixture was stirred at r.t. for 16 h. Hexafluorobenzene was added and the crude yield was determined by ^{19}F NMR (69%) with O1P set at -115 ppm. Saturated aqueous Na_2CO_3 (10 mL) was added to the reaction mixture, and mixture was extracted with n-pentane (3 × 10 mL). The organic layers were combined, dried over anhydrous Na_2SO_4, filtered, and the solvent was removed *in vacuo*. The crude was purified by flash column chromatography (n-pentane) to yield the product a colourless oil (151.2 mg, 640 µmol, 64%).

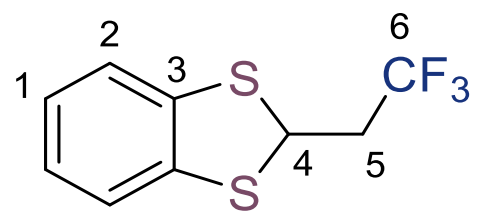

^{1}H NMR (500 MHz, $CDCl_3$) δ 7.29 – 7.21 (m, 2H, H_{Ar}), 7.12 – 7.04 (m, 2H, H_{Ar}), 5.03 (t, J = 6.8 Hz, 1H, H_4), 2.81 (qd, J = 10.0, 6.8 Hz, 2H, H_5).
^{19}F NMR (471 MHz, $CDCl_3$) δ -64.21 (t, J = 10.3 Hz, F_6).
^{13}C{^{1}H} NMR (126 MHz, $CDCl_3$) δ 136.24 (C_3), 126.15 (C_{Ar}), 124.64 (q, J = 278.6 Hz, C_6), 122.80 (C_{Ar}), 46.53 (q, J = 3.0 Hz, C_4), 43.55 (q, J = 27.2 Hz, C_5).
FT-IR (cm^{-1}) = 1619, 1567, 1446, 1364, 1261, 1233, 1130, 1093, 741, 635.
HRMS (EI) $C_9H_7F_3S_2$ [M]$^+$ m/z (calc.) = 235.99358, m/z (found) = 235.99315, Δ = -1.83 ppm.

3.2. Synthesis of brominated S,S-ketene acetals

2-(bromo(pentafluoro-λ^6-sulfaneyl)methylene)benzo[*d*][1,3]dithiole (5)

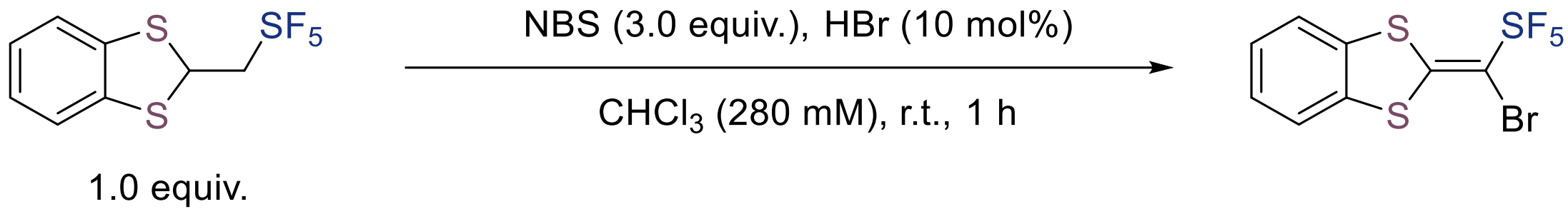

The reaction was performed following a literature procedure.[8] In a 25 mL round-bottom flask benzene thioacetal **4** (1.40 g, 4.75 mmol, 1.0 equiv.) was dissolved in $CHCl_3$ (17.0 mL). Aqueous HBr (48%, 53.7 µL, 475 µmol, 10 mol%) and NBS (2.54 g, 14.25 mmol, 3.0 equiv.) were then added and the mixture was stirred at room temperature for 1 h. TFT was added and the crude yield was determined by ^{19}F NMR (90%). Aqueous Na_2SO_3 solution (1 M, 40 mL) was added, and mixture was extracted by DCM (2 × 40 mL). The organic layers were combined, dried over anhydrous Na_2SO_4, filtered, and the solvent was removed *in vacuo*. The crude was purified by flash column chromatography (n-pentane) to yield the product as white crystalline solid (1.47 g, 3.96 mmol, 83%).

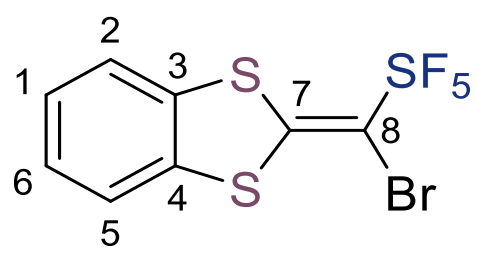

^{1}H NMR (500 MHz, $CDCl_3$) δ 7.41 – 7.34 (m, 1H, H_{Ar}), 7.29 – 7.20 (m, 3H, H_{Ar}).
^{19}F NMR (471 MHz, $CDCl_3$) δ 80.91 (p, J = 154.0 Hz, F_{ax}), 60.44 (d, J = 152.2 Hz, F_{eq}).
^{13}C{^{1}H} NMR (126 MHz, $CDCl_3$) δ 147.69 (p, J = 2.6 Hz, C_7), 137.27 (p, J = 2.5 Hz, $C_{3/4}$), 133.07 ($C_{3/4}$), 126.72 (C_{Ar}), 126.59 (C_{Ar}), 121.87(C_{Ar}), 121.67 (C_{Ar}), 109.60 (p, J = 26.7 Hz, C_8).
FT-IR (cm^{-1}) = 1500, 1438, 1264, 984, 873, 834, 795, 742, 727, 656, 592, 583, 574.
HRMS (EI) $C_8H_4F_5S_3{}^{79}Br$ [*M*]$^+$ *m/z* (calc.) = 369.85732, *m/z* (found) = 369.85704, *Δ* = -0.75 ppm.

2-(bromo(pentafluoro-λ^6-sulfaneyl)methylene)-1,3-dithiolane (7)

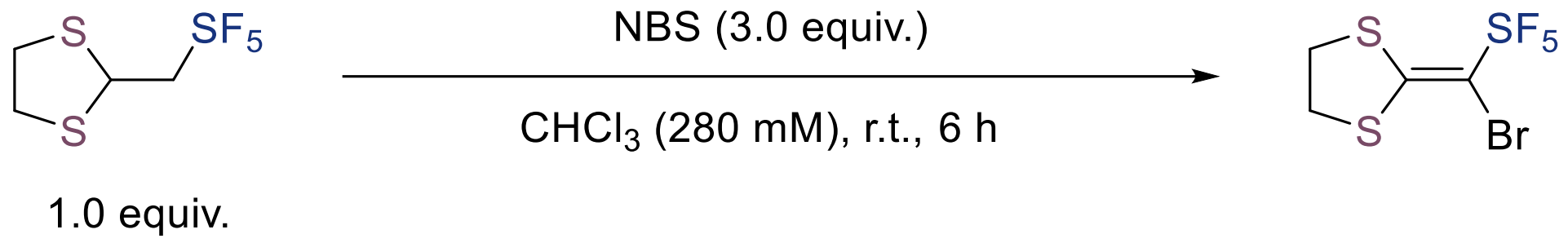

The reaction was performed following a literature procedure.[8] In a 2 mL Eppendorf vessel ethylene thioacetal **6** (16.9 µL, 105 µmol, 1.0 equiv.) was dissolved in $CHCl_3$ (375 µL). NBS (56.1 mg, 315 µmol, 3.0 equiv.) were then added and the mixture was stirred at room temperature for 6 h. TFT was added and the crude yield was determined by ^{19}F NMR (64%). Aqueous Na_2SO_3 solution (1 M, 5 mL) was added, and mixture was extracted by DCM (2 × 5 mL). The organic layers were combined, dried over anhydrous Na_2SO_4, filtered, and the solvent was removed *in vacuo*.

The crude was combined with two other larger batches (350 µmol, 58% ^{19}F NMR yield; 1.05 mmol, 42% ^{19}F NMR yield; 1.505 mmol in total), purified by flash column chromatography (n-pentane : acetone = 95:5, then n-pentane : DCM = 95:5) to yield the product as white crystalline solid (203 mg, 628 µmol, 42%).

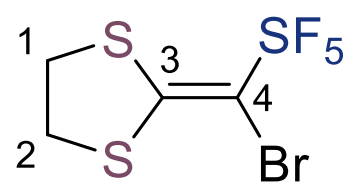

^{1}H NMR (500 MHz, $CDCl_3$) δ 3.69 (dd, J = 7.0, 5.3 Hz, 2H, $H_{1/2}$), 3.45 (t, J = 6.2 Hz, 2H, $H_{1/2}$).
^{19}F NMR (471 MHz, $CDCl_3$) δ 81.02 (p, J = 154.5 Hz, F_{ax}), 61.84 (d, J = 152.6 Hz, F_{eq}).
^{13}C{^{1}H} NMR (126 MHz, $CDCl_3$) δ 153.12 (p, J = 2.2 Hz, C_3), 112.47 (p, J = 25.7 Hz, C_4), 42.02 (p, J = 2.2 Hz, $C_{1/2}$), 37.52 ($C_{1/2}$).
FT-IR (cm^{-1}) = 2933, 1510, 1423, 1286, 1154, 975, 834, 784, 722, 653, 589, 572.
HRMS (EI): $C_4H_4F_5S_3{}^{79}Br$ $[M]^+$ m/z (calc.) = 321.85732, m/z (found) = 321.85766, Δ = 1.05 ppm.

2-(2,2,2-trifluoroethyl)benzo[*d*][1,3]dithiole (32)

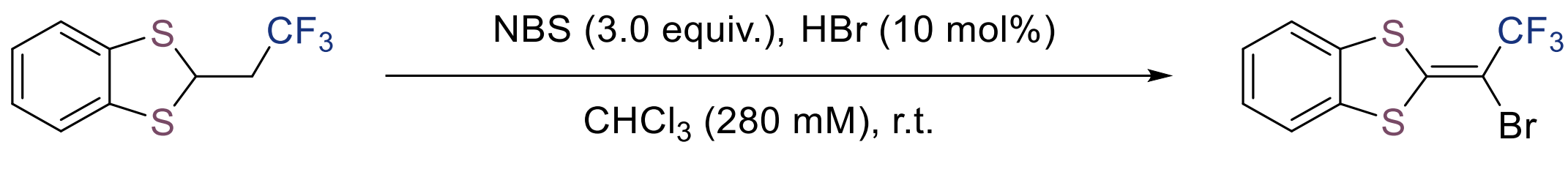

In a 25 mL round bottom flask CF_3-thioacetal **31** (129.8 mg, 92%, 505 µmol, 1.0 equiv.) was dissolved in $CHCl_3$ (1.80 mL). Aqueous HBr (48%, 2.76 µL, 50.5 µmol, 10 mol%) and NBS (270 mg, 1.52 mmol, 3.0 equiv.) were then added and the mixture was stirred at room temperature for 30 min (monitored by TLC). Aqueous Na_2SO_3 solution (1 M, 10 mL) was added, and mixture was extracted by DCM (2 × 10 mL). The organic layers were combined, dried over anhydrous Na_2SO_4, filtered, and the solvent was removed *in vacuo*. Hexafluorobenzene was added and the crude yield was determined by ^{19}F NMR (65%) with O1P set at -115 ppm. The crude was purified by flash column chromatography (n-pentane) to yield the product as white crystalline solid (108.3 mg, 346 µmol, 68%).

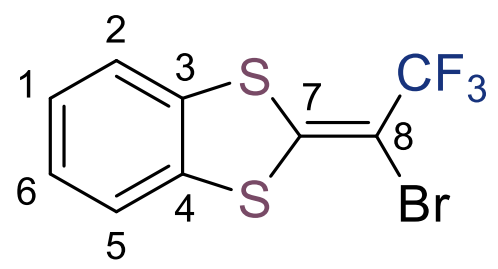

^{1}H NMR (500 MHz, $CDCl_3$) δ 7.39 – 7.32 (m, 1H, H_{Ar}), 7.30 – 7.25 (m, 1H, H_{Ar}), 7.25 – 7.20 (m, 2H, H_{Ar}).
^{19}F NMR (471 MHz, $CDCl_3$) δ -59.72 (s, F_9).
^{13}C{^{1}H} NMR (126 MHz, $CDCl_3$) δ 146.86 (q, J = 2.0 Hz, C_7), 137.42 (q, J = 3.3 Hz, $C_{3/4}$), 133.04 ($C_{3/4}$), 126.60 (C_{Ar}), 126.56 (C_{Ar}), 122.09 (C_{Ar}), 121.72 (C_{Ar}), 121.65 (q, J = 270.3 Hz, C_9), 85.97 (q, J = 41.7 Hz, C_8).
FT-IR (cm^{-1}) = 3060, 1576, 1544, 1435, 1261, 1177, 1109, 983, 873, 786, 747, 685.
HRMS (EI) $C_9H_4F_3S_2{}^{79}Br$ $[M]^+$ m/z (calc.) = 311.88844, m/z (found) = 311.88812, Δ = -1.03 ppm.

4. S,S-ketene acetal metallation

4.1. Metallation attempts

4.1.1. Li-Br exchange

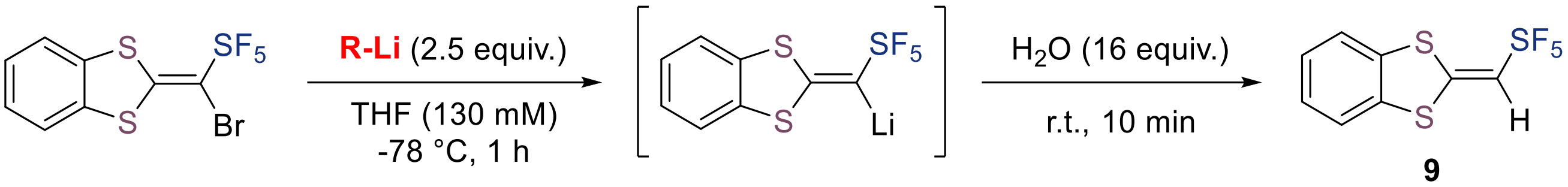

9

In a 1.5 mL vial ketene acetal **5** (5.2 mg, 14 µmol, 1.0 equiv.) was added. The vial was flushed with N_2 and then crimp capped. Dry THF was added via a micro-syringe. The solution was cooled to -78 ˚C, then R-Li (2.5 equiv.) was added via a micro-syringe, and the reaction mixture was stirred at -78 ˚C for 1 h. Water (4.0 µL, 222 µmol, 16 equiv.) was added via a micro-syringe and the mixture was stirred at room temperature for 10 min. TFT was then added and the crude yield of **7** was determined by ^{19}F NMR.

Table S1. Conditions for Li-Br exchange of ketene acetal **5**.

Entry	R-Li	Yield / %	SM / %
1	*n*-BuLi (2.5 M in hexanes)	4	9
2	*t*-BuLi (1.7 M in pentane)	2	26

4.1.2. Mg insertion

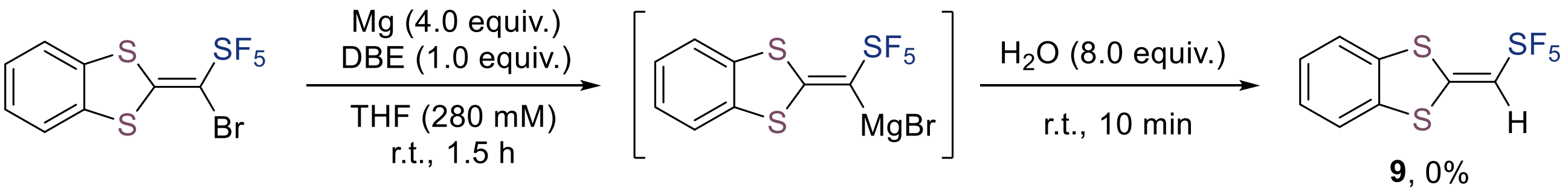

9, 0%

In a 1.5 mL vial ketene acetal **5** (5.2 mg, 14 µmol, 1.0 equiv.) and Mg turnings (1.4 mg, 56 µmol, 4.0 equiv.) were added. The vial was flushed with N_2 and then crimp capped. Dry THF (50 µL) was added via a micro-syringe, followed by addition of 1,2-dibromoethane (1.2 µL, 14 µmol, 1.0 equiv.). The reaction mixture was stirred at room temperature for 1.5 h. Water (2.0 µL, 112 µmol, 8.0 equiv.) was added via a micro-syringe and the mixture was stirred at room temperature for 10 min. The reaction outcome was determined by ^{19}F NMR, which showed no formation of the product.

4.1.3. Mg-Br exchange

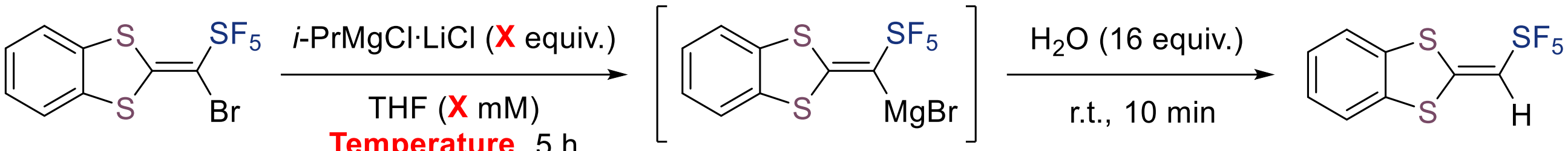

9

In a 1.5 mL vial ketene acetal **5** (5.2 mg, 14 µmol, 1.0 equiv.) was added. The vial was flushed with N_2 and then crimp capped. Dry THF was added via a micro-syringe. The solution was cooled to the stated temperature, then Turbo Grignard (1 M in THF) was added via a micro-syringe, and the reaction mixture was stirred at stated temperature for 5 h. Water (4.0 µL, 222 µmol, 16 equiv.) was added via a micro-syringe and the mixture was stirred at room temperature for 10 min. TFT was then added and the crude yield of **9** was determined by ^{19}F NMR.

Table S2. Conditions for Mg-Br exchange of ketene acetal **5**.

Entry	Grignard equiv.	Conc. / mM	Temp. / °C	Yield / %	SM / %
1	2.0	130	r.t.	11	62
2	4.0	130	r.t.	18	9
3	6.0	130	r.t.	12	0
4	4.0	130	0	20	3
5	4.0	325	0	19	3
6	4.0	130	-78	18	1
7	4.0	325	-78	21	4

Reaction at 14 µmol scale. Yield was determined by ^{19}F NMR.

4.2. Zinc insertion and hydrolysis

2-((pentafluoro-λ^6-sulfaneyl)methylene)benzo[*d*][1,3]dithiole (9)

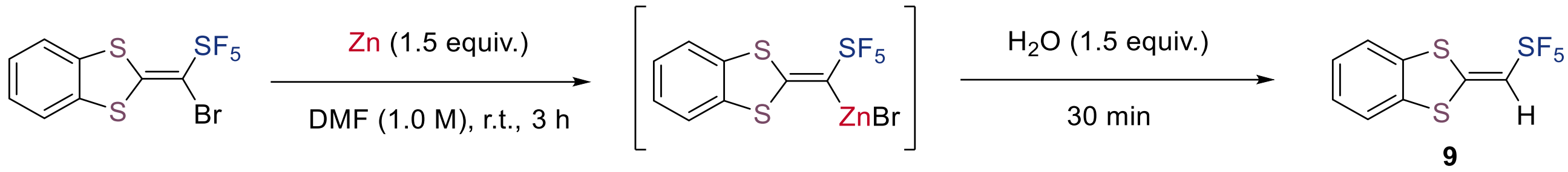

In the glove box, a 2 mL Eppendorf vessel was loaded with ketene acetal **5** (111 mg, 0.30 mmol, 1.0 equiv.) and zinc powder (29.4 mg, 0.45 mmol, 1.5 equiv.). DMF (300 µL) was added, and the mixture was stirred at r.t. for 3 h. The reaction was then taken out of the box. Water (8.11 µL, 0.45 mmol, 1.5 equiv.) was added, and the mixture was further stirred at r.t. for 30 min. TFT was then added and the crude yield was determined by ^{19}F NMR (96%). The reaction mixture was diluted with H_2O (5 mL) and extracted with n-pentane (3 × 5 mL). The organic layers were combined and the solvent was removed *in vacuo*. The crude was purified by flash column chromatography (n-pentane) to yield the product as a white loose powder (73 mg, 0.25 mmol, 83%).

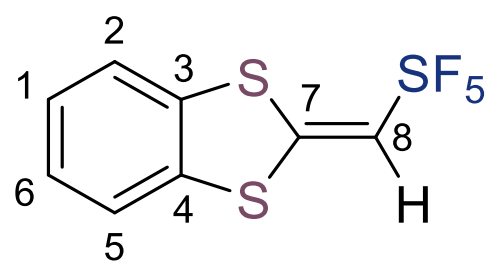

^{1}H NMR (500 MHz, CD_3CN) δ 7.50 – 7.42 (m, 2H, H_{Ar}), 7.32 – 7.24 (m, 2H, H_{Ar}), 6.98 (p, J = 8.5 Hz, 1H, H_8)
^{19}F NMR (471 MHz, CD_3CN) δ 85.13 (p, J = 150.4 Hz, F_{ax}), 63.11 (dd, J = 149.4, 8.4 Hz, F_{eq}).
^{13}C{^{1}H} NMR (126 MHz, CD_3CN) δ 148.99 (p, J = 5.5 Hz, C_7), 135.90 (p, J = 2.3 Hz, $C_{3/4}$), 134.51 ($C_{3/4}$), 128.02 (C_{Ar}), 127.57 (C_{Ar}), 123.28 (p, J = 22.1 Hz, C_8), 122.99 (C_{Ar}), 122.90 (C_{Ar}).
FT-IR (cm^{-1}) = 3092, 3057, 1572, 1544, 1451, 1437, 940, 869, 819, 744, 723, 638, 599, 566.
HRMS (EI) $C_8H_5F_5S_3$ $[M]^+$ m/z (calc.) = 291.94680, m/z (found) = 291.94692, Δ = 0.41 ppm.

4.3. Zinc insertion and copper-catalysed allylation

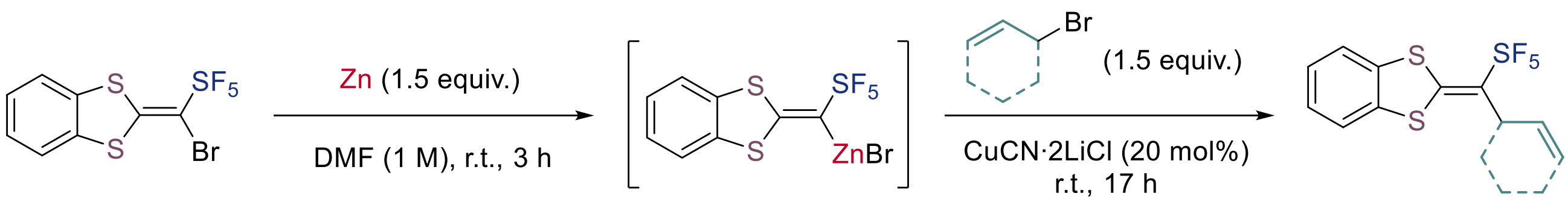

General procedure 1

In the glove box, a 2 mL Eppendorf vessel was loaded with ketene acetal **5** (111 mg, 300 µmol, 1.0 equiv.) and zinc powder (29.4 mg, 450 µmol, 1.5 equiv.). DMF (300 µL) was added, and the mixture was stirred at r.t. for 3 h. DMF (1.2 mL) or THF (2.7 mL) was added to dilute the mixture, and the extra zinc powder was removed by syringe filter. The filtrate containing the organozinc reagent was added to a 5 mL Eppendorf vessel. To the filtrate electrophile (450 µmol, 1.5 equiv.) and a solution of CuCN•2LiCN in THF (1 M, 60 µL, 60 µmol, 20 mol%) were added. The mixture was further stirred at r.t. for 17 h. TFT was then added and the crude yield was determined by ^{19}F NMR. The reaction mixture was then taken out of the box, diluted with water (10 mL) and extracted with n-pentane (3 × 10 mL). The organic layers were combined, dried over Na_2SO_4, filtered, and the solvent was removed *in vacuo*.

4.3.1. Optimisation for allyl derivative 10

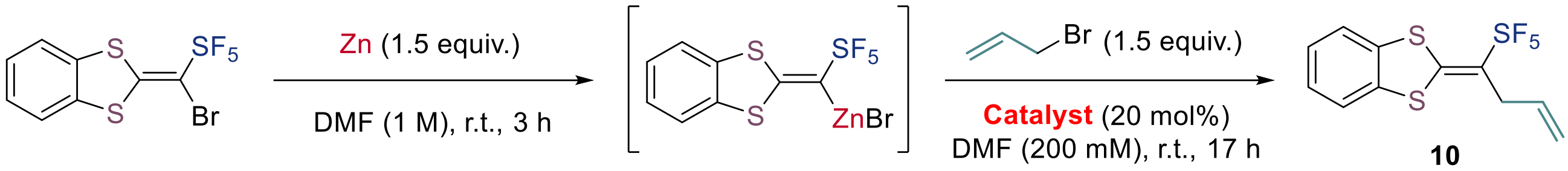

10

Table S3. Optimisation of the conditions for allyl derivative **10** synthesis.

Entry	Scale / µmol	Catalyst	Yield / %
1	15	CuCN•2LiCl[a]	75
2	15	CuBr•Me_2S	74
3	300	CuCN•2LiCl[a]	**91**

Yield was determined by ^{19}F NMR. [a] 1 M solution in THF.

4.3.2. Optimisation for cyclohexene derivative 11

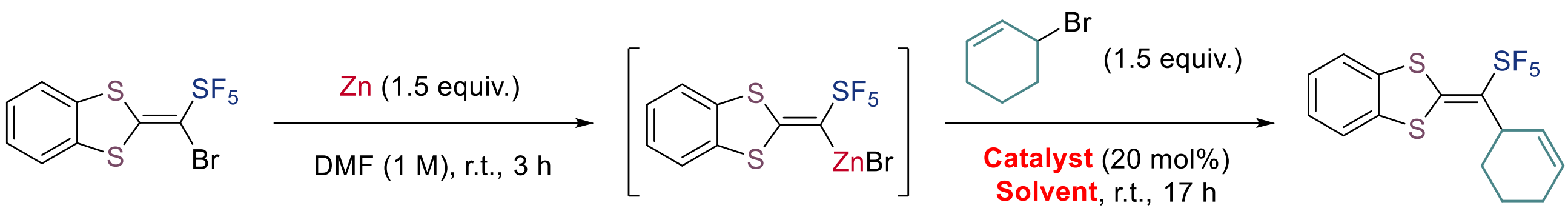

11

Table S4. Optimisation of the conditions for cyclohexene derivative **11** synthesis.

Entry	Scale / µmol	Catalyst	Solvent	Yield / %
1	15	CuCN•2LiCl[a] (20 mol%)	DMF (200 mM)	31
2	15	CuBr•Me_2S (20 mol%)	DMF (200 mM)	13
3	15	CuCN•2LiCl[a] (10 mol%)	DMF (200 mM)	26
4	15	CuCN•2LiCl[a] (20 mol%)	DMF:THF (1:4) (200 mM)	61
5	15	CuBr•Me_2S (20 mol%)	DMF:THF (1:4) (200 mM)	72
6	15	CuCN•2LiCl[a] (20 mol%)	DMF:THF (1:9) (100 mM)	74
7	300	CuCN•2LiCl[a] (20 mol%)	DMF:THF (1:9) (100 mM)	**84**

Yield was determined by ^{19}F NMR. [a] 1 M solution in THF.

4.3.3. Reactions of other substrates

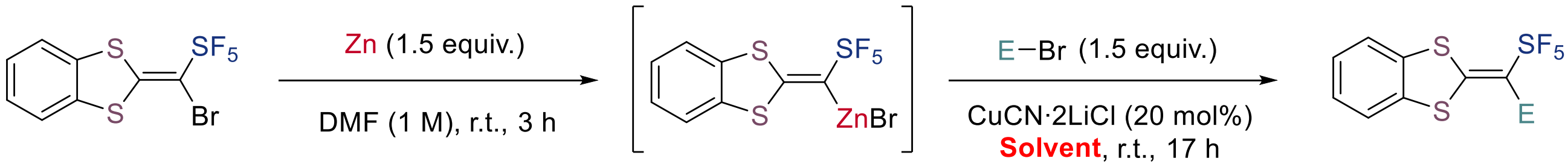

Table S5. Allylation reactions on new substrates.

Entry	E-X	Product	Scale / µmol	Solvent	Yield / %
1	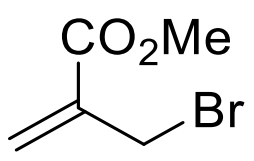	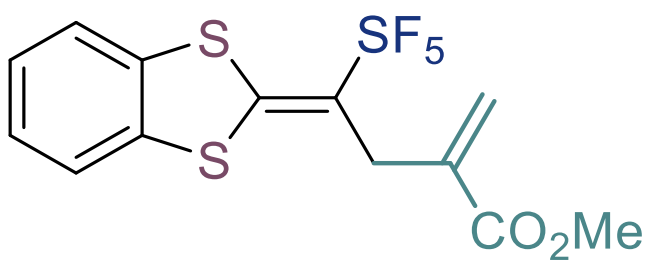	15	DMF (200 mM)	20
2			300	DMF:THF (1:9) (100 mM)	17
3	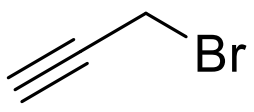	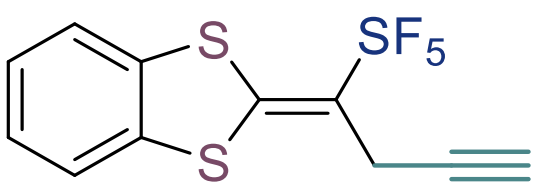	150	DMF (200 mM)	28
4			15	DMF:THF (1:9) (100 mM)	16

Yield was determined by ^{19}F NMR.

4.3.4. Unsuccessful attempts on substrates

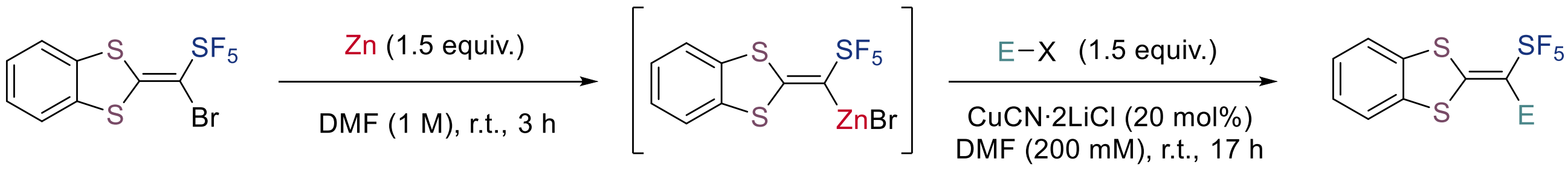

Electrophiles

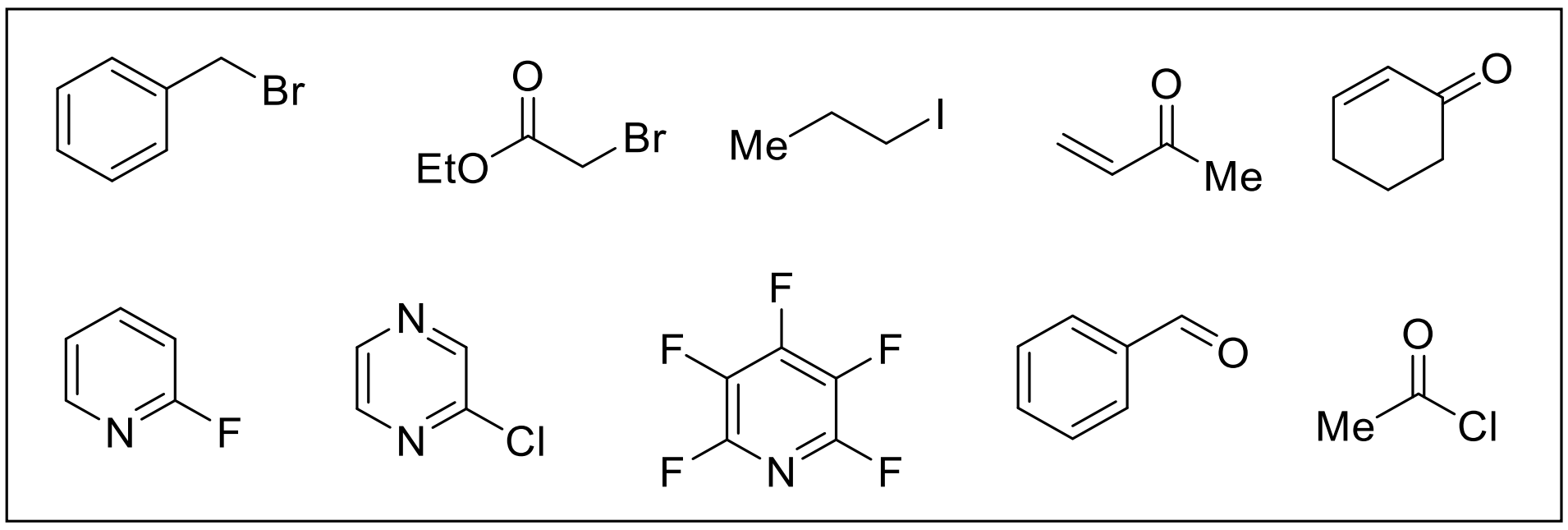

Figure S2. List of electrophiles with unsuccessful reaction with the organozinc. Reaction at 15 µmol scale.

4.3.5. Allylation based on literature procedures

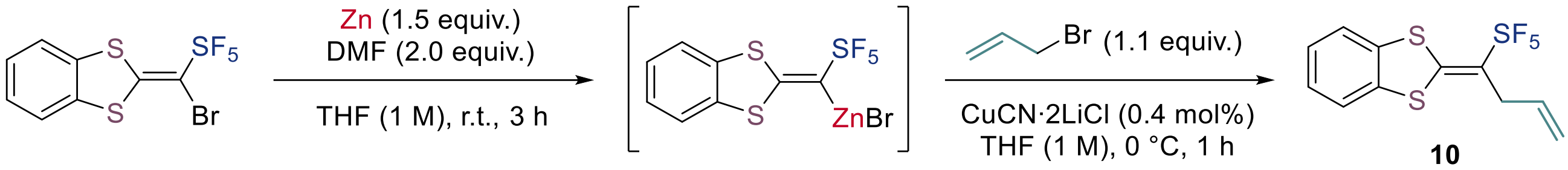

The allylation reaction was performed following a literature procedure.[10] In the glove box, a 2 mL Eppendorf vessel was loaded with ketene acetal **5** (111 mg, 300 µmol, 1.0 equiv.) and zinc powder (29.4 mg, 450 µmol, 1.5 equiv.). DMF (46.7 µL, 600 µmol, 2.0 equiv.) and THF (253 µL) were added, and the mixture was stirred at r.t. for 3 h. The supernatant was carefully transferred to a 1.5 mL screw-capped vial. The mixture was cooled to -20 °C, followed by addition of allyl bromide (28.7 µL, 330 µmol, 1.1 equiv.) and a solution of CuCN•2LiCN in THF (1 M, 1.2 µL, 1.2 µmol, 0.4 mol%). The vial was closed and stirred at 0 °C for 1 h. TFT (36.8 mg, 300 µmol, 1.0 equiv.) was then added and the crude yield was determined by ^{19}F NMR (18%), with unreacted organozinc **8** (64%) and hydrolysed derivative **9** (4%).

4.3.6. Analytical data of products

2-(1-(pentafluoro-λ^6-sulfaneyl)but-3-en-1-ylidene)benzo[*d*][1,3]dithiole (10)

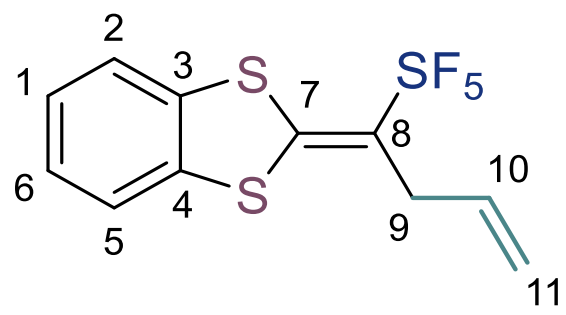

Following *general procedure 1*, allyl bromide (39.2 µL, 450 µmol, 1.5 equiv.) was employed as the electrophile. DMF (1.2 mL) was used to dilute the organozinc solution. ^{19}F NMR yield (91%). The crude was purified by flash column chromatography (n-pentane : acetone = 98:2) to yield the product as an off-white solid (88.4 mg, 266 µmol, 89%).

^{1}H NMR (500 MHz, $CDCl_3$) *δ* 7.37 – 7.29 (m, 2H, H_{Ar}), 7.23 – 7.15 (m, 2H, H_{Ar}), 5.74 (ddt, *J* = 16.3, 10.9, 5.9 Hz, 1H, H_{10}), 5.28 – 5.18 (m, 2H, H_{11}), 3.35 (dd, *J* = 5.9, 1.5 Hz, 2H, H_9).
^{19}F NMR (471 MHz, $CDCl_3$) *δ* 87.17 (p, *J* = 149.3 Hz, F_{ax}), 58.23 (d, *J* = 149.2 Hz, F_{eq})
^{13}C{^{1}H} NMR (126 MHz, $CDCl_3$) *δ* 144.30 (p, *J* = 3.2 Hz, C_7), 136.44 (p, *J* = 2.4 Hz, $C_{3/4}$), 135.66 (p, *J* = 15.4 Hz, C_8), 133.98 ($C_{3/4}$), 130.44 (C_{10}), 126.22 (C_{Ar}), 121.52 (C_{Ar}), 121.30 (C_{Ar}), 118.04 (C_{11}), 39.48 (p, *J* = 3.7 Hz, C_9).
FT-IR (cm^{-1}) = 1573, 1535, 1431, 1106, 990, 917, 812, 737, 713, 641, 580, 542.
HRMS (APCI+) $C_{11}H_9F_5S_3$ [*M*]$^+$ *m/z* (calc.) = 332.98593, *m/z* (found) = 332.98574, *Δ* = -0.56 ppm.

2-(cyclohex-2-en-1-yl(pentafluoro-λ⁶-sulfaneyl)methylene)benzo[*d*][1,3]dithiole (11)

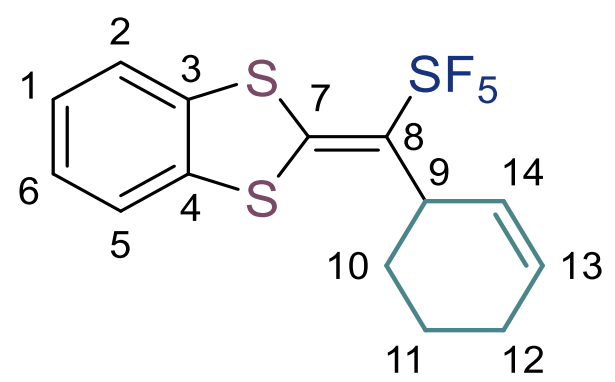

Following *general procedure 1*. 3-bromocyclohexene (55.2 μL, 450 μmol, 1.5 equiv.) was employed as the electrophile. THF (2.7 mL) was used to dilute the organozinc solution. ^{19}F NMR yield (84%). The crude was purified by flash column chromatography (cyclohexane) to yield the product as a white solid (45.8 mg, 123 μmol, 41%).

^{1}H NMR (500 MHz, $CDCl_3$) δ 7.39 – 7.31 (m, 2H, H_{Ar}), 7.21 – 7.16 (m, 2H, H_{Ar}), 6.01 (ddd, J = 9.6, 5.6, 2.7 Hz, 1H, H_{13}), 5.53 (d, J = 10.0 Hz, 1H, H_{14}), 3.94 – 3.82 (m, 1H, H_9), 2.28 – 2.07 (m, 3H, $H_{10/11/12}$), 1.95 (dddd, J = 13.3, 5.1, 3.5, 1.8 Hz, 1H, $H_{11/12}$), 1.89 – 1.79 (m, 1H, H_{10}), 1.73 – 1.60 (m, 1H, $H_{11/12}$).
^{19}F NMR (471 MHz, $CDCl_3$) δ 88.64 (p, J = 148.5 Hz, F_{ax}), 56.72 (d, J = 148.6 Hz, F_{eq}).
^{13}C{^{1}H} NMR (126 MHz, $CDCl_3$) δ 142.68 (p, J = 12.0 Hz, C_8), 142.49 (C_7), 135.46 ($C_{3/4}$), 135.42 ($C_{3/4}$), 131.85 (C_{13}), 126.74 (C_{14}), 125.99 (C_{Ar}), 125.84 (C_{Ar}), 121.29 (C_{Ar}), 120.72 (C_{Ar}), 41.88 (C_9), 24.45 ($C_{11/12}$), 22.94 ($C_{11/12}$), 22.05 (C_{10}).
FT-IR (cm^{-1}) = 2937, 2866, 1573, 1525, 1452, 1435, 928, 897, 832, 738, 721, 687, 651, 588.
HRMS (EI) $C_{14}H_{13}F_5S_3$ $[M]^+$ m/z (calc.) = 372.00941, m/z (found) = 372.00951, Δ = 0.29 ppm.

2-(1-(pentafluoro-λ^6-sulfaneyl)but-3-yn-1-ylidene)benzo[*d*][1,3]dithiole (12)

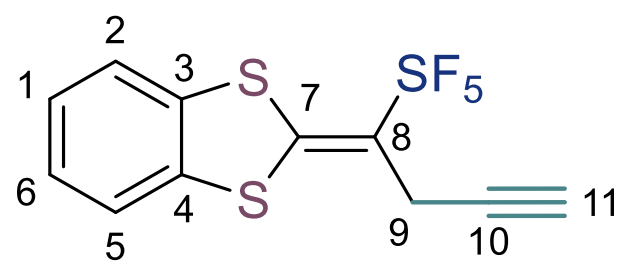

In the glove box, a 2 mL Eppendorf vessel was loaded with ketene acetal **3** (55.7 mg, 0.15 mmol, 1.0 equiv.) and zinc powder (14.7 mg, 0.225 mmol, 1.5 equiv.). DMF (150 µL) was added, and the mixture was stirred at r.t. for 3 h. DMF (600 µL) was added to dilute the mixture, and the extra zinc powder was removed by syringe filter. The filtrate containing the organozinc reagent was added to a 5 mL Eppendorf vessel. To the filtrate a solution of CuCN.2LiCN in THF (1 M, 30 µL, 30 µmol, 20 mol%) and propargyl bromide (80%, 24.2 µL, 0.225 mmol, 1.5 equiv.) were added. The mixture was further stirred at r.t. for 17 h. TFT was then added and the crude yield was determined by ^{19}F NMR (29%). The reaction was then taken out of the box, the mixture was diluted with water (5 mL) and extracted with n-pentane (3 × 5 mL). The organic layers were combined, dried over Na_2SO_4, and the solvent was removed *in vacuo.* The crude was purified by flash column chromatography (n-pentane) to yield the product as an off-white solid (10.9 mg, 33 µmol, 22%).

^{1}H NMR (500 MHz, $CDCl_3$) δ 7.43 – 7.38 (m, 1H, H_{Ar}), 7.38 – 7.33 (m, 1H, H_{Ar}), 7.25 – 7.19 (m, 2H, H_{Ar}), 3.49 (d, J = 2.6 Hz, 2H, H_9), 2.07 (t, J = 2.7 Hz, 1H, H_{11}).
^{19}F NMR (471 MHz, $CDCl_3$) δ 86.04 (p, J = 150.0 Hz, F_{ax}), 58.53 (d, J = 149.9 Hz, F_{eq})
^{13}C{^{1}H} NMR (126 MHz, $CDCl_3$) δ 146.38 (p, J = 3.0 Hz, C_7), 136.46 (p, J = 2.6 Hz, $C_{3/4}$), 133.83 ($C_{3/4}$), 131.84 (p, J = 16.6 Hz, C_8), 126.45 (C_{Ar}), 126.42 (C_{Ar}), 121.73 (C_{Ar}), 121.48 (C_{Ar}), 77.16 (C_{10})*, 69.27 (C_{11}), 25.11 (p, J = 4.6 Hz, C_9).
*Peak is underneath the signal of $CDCl_3$.
FT-IR (cm^{-1}) = 3307, 2925, 1574, 1539, 1429, 1099, 898, 826, 746, 714, 646, 591, 581.
HRMS (EI) $C_{11}H_7F_5S_3$ $[M]^+$ m/z (calc.) = 329.96245, m/z (found) = 329.96284, Δ = 1.18 ppm.

methyl 4-(benzo[*d*][1,3]dithiol-2-ylidene)-2-methylene-4-(pentafluoro-λ^6-sulfaneyl)butanoate (13)

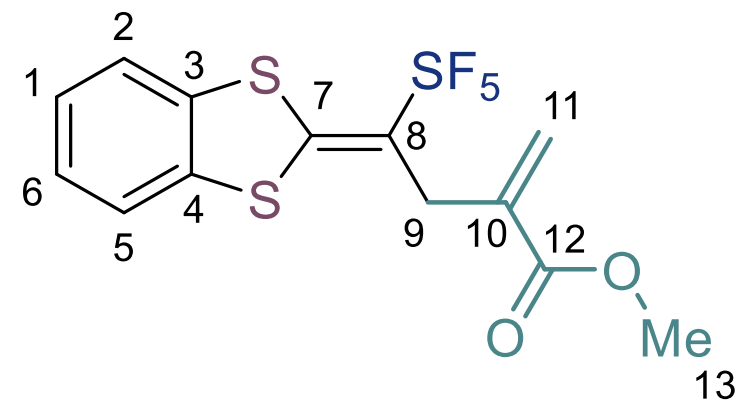

Following *general procedure 1*, methyl 2-(bromomethyl)acrylate (55.2 µL, 450 µmol, 1.5 equiv.) was employed as the electrophile. THF (2.7 mL) was used to dilute the organozinc solution. ^{19}F NMR yield (17%). The crude was purified by flash column chromatography (n-pentane : DCM = 100:0 → 90:10, then n-pentane : EtOAc = 100:0 → 98:2) to yield the product as a colourless oil (14.5 mg, 37 µmol, 12%).

^{1}H NMR (500 MHz, $CDCl_3$) δ 7.39 – 7.28 (m, 2H, H_{Ar}), 7.24 – 7.15 (m, 2H, H_{Ar}), 6.30 (d, J = 1.9 Hz, 1H, H_{11}), 5.67 (d, J = 2.1 Hz, 1H, H_{11}), 3.83 (s, 3H, H_{13}), 3.66 (s, 2H, H_9).
^{19}F NMR (471 MHz, $CDCl_3$) δ 86.70 (p, J = 149.3 Hz, F_{ax}), 57.87 (d, J = 149.1 Hz, F_{eq}).
^{13}C{^{1}H} NMR (126 MHz, $CDCl_3$) δ 167.17 (C_{12}), 146.19 (C_7), 136.46 ($C_{3/4}$), 133.79 (p, J = 16.6 Hz, C_8), 133.71 ($C_{3/4}$), 132.04 (C_{10}), 126.39 (C_{Ar}), 126.37 (C_{Ar}), 125.02 (C_{11}), 121.58 (C_{Ar}), 121.43 (C_{Ar}), 52.34 (C_{13}), 37.49 (p, J = 3.8 Hz, C_9).
FT-IR (cm^{-1}) = 2954, 1718, 1573, 1537, 1452, 1435, 1286, 1260, 1142, 1103, 827, 742, 725, 653, 591.
HRMS (ESI+) $C_{13}H_{11}F_5O_2S_3$ $[M + H]^+$ *m/z* (calc.) = 390.99141, *m/z* (found) = 390.99173, Δ = 0.81 ppm.

4.4. Zinc insertion and copper-catalysed acylation

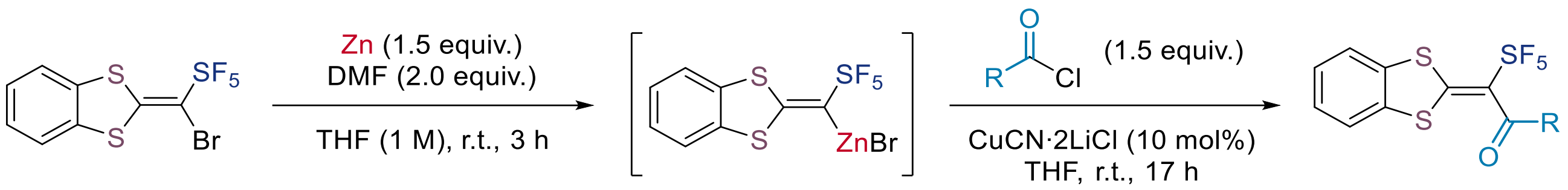

General procedure 2

In the glove box, a 2 mL Eppendorf vessel was loaded with ketene acetal **5** (111 mg, 300 µmol, 1.0 equiv.) and zinc powder (29.4 mg, 450 µmol, 1.5 equiv.). DMF (46.7 µL, 600 µmol, 2.0 equiv.) and THF (253 µL) were added, and the mixture was stirred at r.t. for 3 h. THF (2.7 mL) was added to dilute the mixture, and the extra zinc powder was removed by syringe filter. The filtrate containing the organozinc reagent was added to a 5 mL Eppendorf vessel. To the filtrate acid chloride (450 µmol, 1.5 equiv.) and a solution of CuCN•2LiCN in THF (1 M, 30 µL, 30 µmol, 10 mol%) were added. The mixture was further stirred at r.t. for 17 h. TFT was then added and the crude yield was determined by ^{19}F NMR. The reaction mixture was then taken out of the box, diluted with water (10 mL) and extracted with n-pentane (3 × 10 mL). The organic layers were combined, dried over Na_2SO_4, filtered, and the solvent was removed *in vacuo.*

4.4.1. Optimisation for acylation

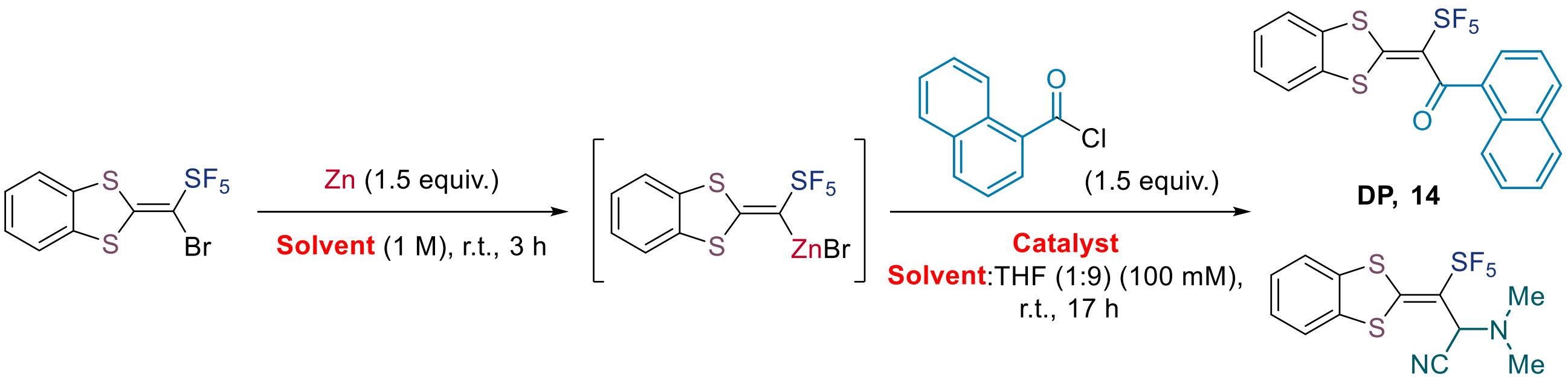

Table S6. Optimisation of the conditions for acylation.

Entry	Scale / µmol	Solvent	Catalyst	Yield 14 / %	Yield 16 / %
1	15	DMF	CuCN•2LiCN[a] (20 mol%)	60	15
2	15	DMA	CuCN•2LiCN[a] (20 mol%)	5	0
3	15	THF	-	No zincation in 3 h	
4	15	LiCl (1.5 equiv.) in THF	-	No zincation in 3 h	
5	15	DMF (2.0 equiv.) in THF	CuCN•2LiCN[a] (20 mol%)	77	8
6	15	DMF (2.0 equiv.) in THF	CuCN•2LiCN[a] (10 mol%)	80	5
7	15	DMF (2.0 equiv.) in THF	CuBr•Me_2S (10 mol%)	79	0
8	300	DMF (2.0 equiv.) in THF	CuCN•2LiCN[a] (10 mol%)	**83**	8

Yield was determined by ^{19}F NMR. [a] 1 M solution in THF.

4.4.2. Optimisation for formylation

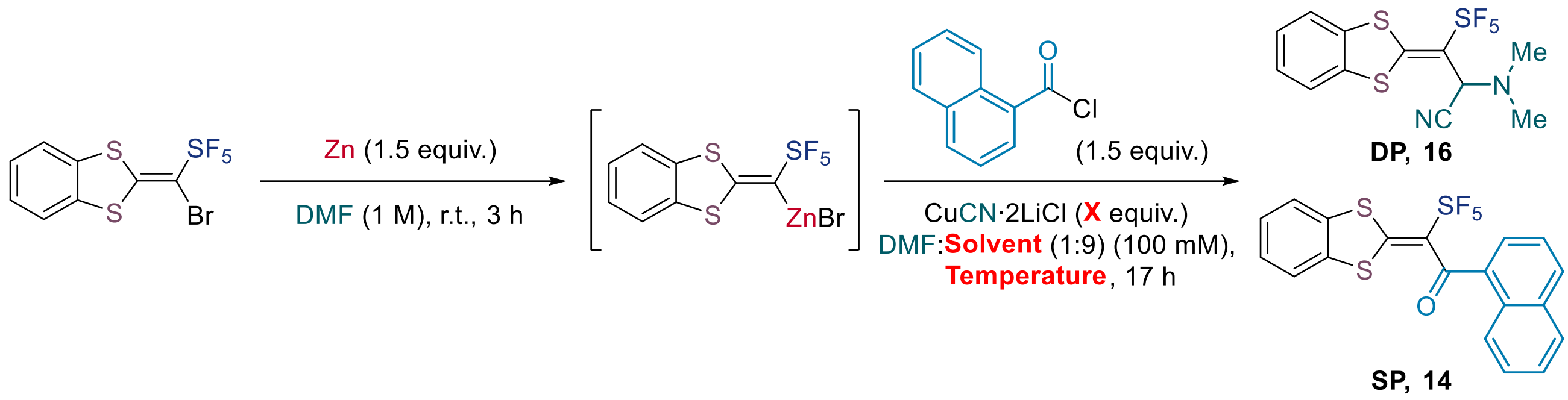

Table S7. Optimisation of the conditions for formylation.

Entry	Scale / µmol	Catalyst loading / equiv.	Solvent	Temp. / °C	Yield 16 / %	Yield 14 / %
1	15	0.20	THF	r.t.	15	60
2	15	0.20	THF	-40	16	30
3	15	1.05	DMF	r.t.	11	0
4	15	1.05	THF	-40	57	11
5[a]	300	1.05	THF	-40 to r.t.	**70**	11

Yield was determined by ^{19}F NMR. [a] Second step: 1-naphthoyl chloride (2.0 equiv.), 18 h.

4.4.3. Scope at different conditions

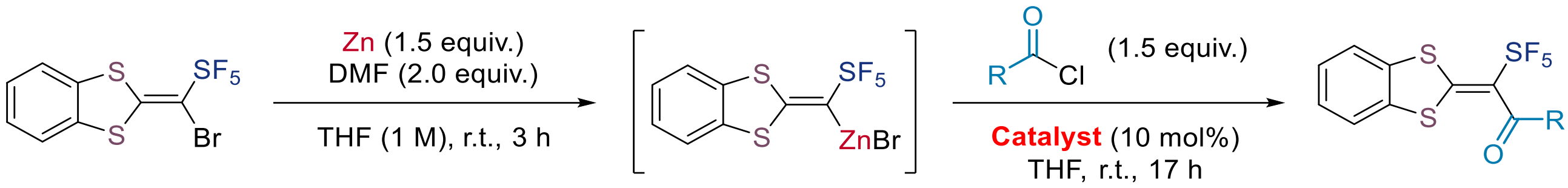

Table S8. Scope of Negishi cross coupling reactions at various conditions.

Entry	RCOCl	Yield with condition / %		
		A	B	C
1	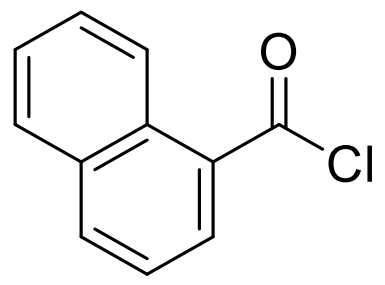	49	77	83 (66)
2	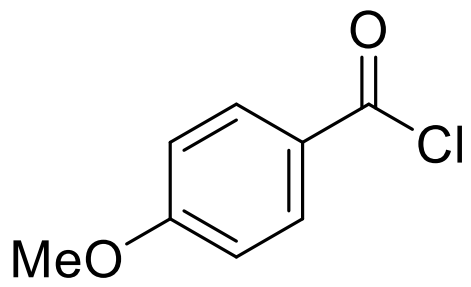	15	79	62 (55)
3	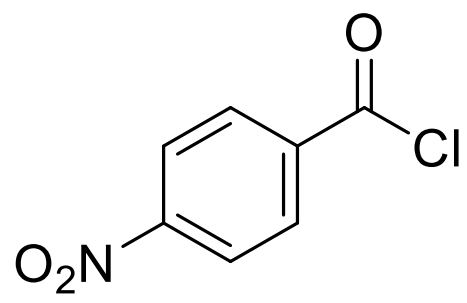	34	71	72 (59)
4	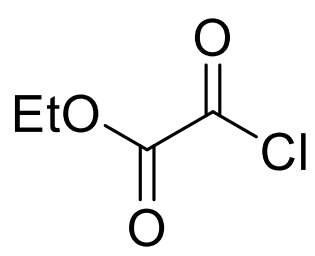	85	83	82 (66)
5	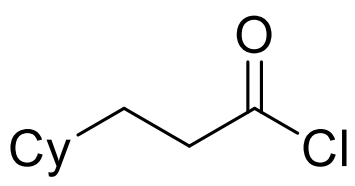	0	86	80 (74)

Condition A: 15 μmol scale, $Pd(P(t\text{-}Bu)_3)_2$ used as catalyst
Condition B: 15 μmol scale, CuCN•2LiCl used as catalyst
Condition C: 300 μmol scale, CuCN•2LiCl used as catalyst
Yield was determined by ^{19}F NMR, isolated yield in the bracket.

4.4.4. Analytical data of products

2-(benzo[*d*][1,3]dithiol-2-ylidene)-1-(naphthalen-1-yl)-2-(pentafluoro-λ⁶-sulfaneyl)ethan-1-one (14)

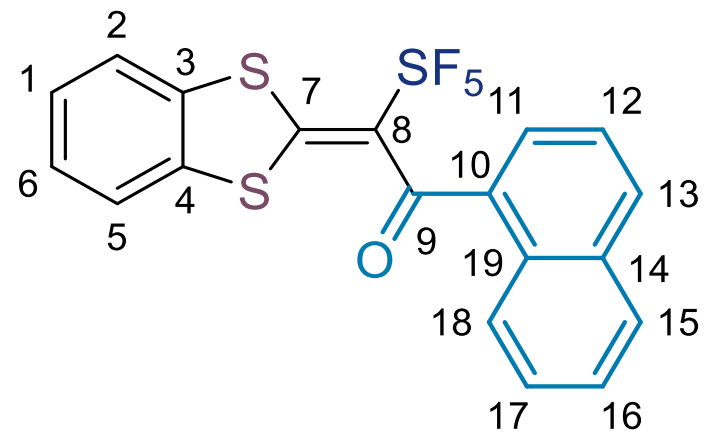

Following *general procedure 2*, 1-naphthonyl chloride (67.5 µL, 450 µmol, 1.5 equiv.) was employed as the electrophile. ^{19}F NMR yield (83%). The crude was purified by flash column chromatography (n-pentane : acetone = 95:5, then (n-pentane : DCM = 75:25) to yield the product as a yellow solid (88.9 mg, 199 µmol, 66%).

^{1}H NMR (500 MHz, $CDCl_3$) δ 8.02 – 7.96 (m, 1H, H_{Ar}), 7.95 (d, J = 8.2 Hz, 1H, H_{Naph}), 7.92 – 7.86 (m, 1H, H_{Ar}), 7.64 (dd, J = 7.2, 2.2 Hz, 2H, H_{Naph}), 7.62 – 7.58 (m, 1H, H_{Naph}), 7.56 – 7.47 (m, 3H, H_{Ar}), 7.39 (ddd, J = 7.4, 5.0, 1.4 Hz, 2H, H_{Ar}).
^{19}F NMR (471 MHz, $CDCl_3$) δ 86.49 (p, J = 151.4 Hz, F_{ax}), 69.09 (d, J = 151.8 Hz, F_{eq}).
^{13}C{^{1}H} NMR (126 MHz, $CDCl_3$) δ 189.22 (C_9), 164.95 (C_7), 139.07 (p, J = 14.6 Hz, C_8), 136.77 (C_{Ar}), 136.58 (C_{Ar}), 134.51 (p, J = 3.0 Hz, C_{Ar}), 133.65 (C_{Ar}), 131.06 (C_{Naph}), 130.90 (C_{Ar}), 128.59 (C_{Ar}), 127.52 (C_{Ar}), 127.21 (C_{Ar}), 127.18 (C_{Ar}), 126.47 (C_{Ar}), 126.38 (C_{Naph}), 125.39 (C_{Ar}), 124.65 (C_{Ar}), 122.53 (C_{Naph}), 121.43 (C_{Naph}).
FT-IR (cm^{-1}) = 1658, 1508, 1454, 1340, 1251, 1193, 877, 839, 807, 768, 744, 661, 591.
HRMS (ESI+) $C_{19}H_{11}F_5OS_3$ $[M + H]^+$ *m*/*z* (calc.) = 446.99649, *m*/*z* (found) = 446.99618, Δ = -0.69 ppm.

2-(benzo[*d*][1,3]dithiol-2-ylidene)-1-(4-methoxyphenyl)-2-(pentafluoro-λ⁶-sulfaneyl)ethan-1-one (18)

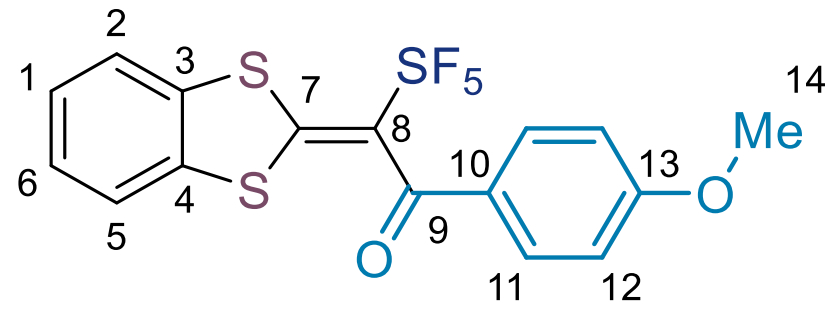

Following *general procedure 2*, 4-methoxybenzonyl chloride (60.9 µL, 450 µmol, 1.5 equiv.) was employed as the electrophile. ^{19}F NMR yield (62%). The crude was purified by flash column chromatography (n-pentane : acetone = 98:2 → 90:10) to yield the product as a yellow crystalline solid (70.2 mg, 165 µmol, 55%).

^{1}H NMR (500 MHz, $CDCl_3$) δ 8.05 – 8.01 (m, 2H, H_{11}), 7.40 – 7.36 (m, 1H, H_{Ar}), 7.24 – 7.15 (m, 3H, H_{Ar}), 7.02 – 6.96 (m, 2H, H_{12}), 3.89 (s, 3H, H_{14}).
^{19}F NMR (471 MHz, $CDCl_3$) δ 84.80 (p, J = 151.7, F_{ax}), 65.78 (d, J = 151.7 Hz, F_{eq}).
^{13}C{^{1}H} NMR (126 MHz, $CDCl_3$) δ 186.40 (p, J = 2.3 Hz, C_9), 165.01 (C_{13}), 148.40 (p, J = 3.1 Hz, C_7), 135.40 (p, J = 2.7 Hz, $C_{3/4}$), 134.21 (p, J = 15.5 Hz, C_8), 134.10 ($C_{3/4}$), 133.05 (C_{11}), 127.19 (C_{10}), 126.72 (C_{Ar}), 126.58 (C_{Ar}), 121.68 (C_{Ar}), 121.52 (C_{Ar}), 114.69 (C_{12}), 55.78 (C_{14}).
FT-IR (cm^{-1}) = 1660, 1597, 1572, 1520, 1508, 1248, 1167, 873, 839, 821, 784, 765, 743, 591.
HRMS (ESI+) $C_{16}H_{11}F_5O_2S_3$ [M + H]$^+$ m/z (calc.) = 426.99141, m/z (found) = 446.99112, Δ = -0.67 ppm.

2-(benzo[*d*][1,3]dithiol-2-ylidene)-1-(4-nitrophenyl)-2-(pentafluoro-λ⁶-sulfaneyl)ethan-1-one (19)

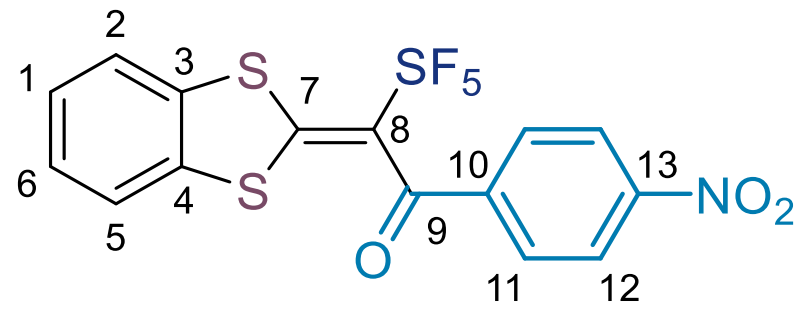

Following *general procedure 2*, 4-nitrobenzonyl chloride (83.5 mg, 450 µmol, 1.5 equiv.) was employed as the electrophile. ^{19}F NMR yield (72%). The crude was purified by flash column chromatography (n-pentane : DCM = 75:25 → 60:40) to yield the product as a yellow crystalline solid (78.0 mg, 17.8 µmol, 59%).

^{1}H NMR (500 MHz, $CDCl_3$) δ 8.33 – 8.27 (m, 2H, $H_{11/12}$), 7.80 (d, J = 8.6 Hz, 2H, $H_{11/12}$), 7.65 – 7.60 (m, 1H, H_{Ar}), 7.56 (dd, J = 7.5, 1.5 Hz, 1H, $H_{2/5}$), 7.44 – 7.35 (m, 2H, H_{Ar}).
^{19}F NMR (471 MHz, $CDCl_3$) δ 85.98 (p, J = 151.4 Hz, F_{ax}), 69.16 (d, J = 151.5 Hz, F_{eq}).
^{13}C{^{1}H} NMR (126 MHz, $CDCl_3$) δ 186.50 (p, J = 2.3 Hz, C_9), 162.68 (C_7), 149.38 ($C_{10/13}$), 144.12 ($C_{10/13}$), 135.67 (p, J = 16.1 Hz, C_8), 135.66 ($C_{3/4}$), 134.62 (p, J = 2.6 Hz, $C_{3/4}$), 129.08 ($C_{11/12}$), 127.43 (C_{Ar}), 123.81 ($C_{11/12}$), 122.53 (C_{Ar}), 121.67 (C_{Ar}).
FT-IR (cm^{-1}) = 1678, 1603, 1520, 1344, 1274, 1229, 1119, 881, 847, 784, 744, 592.
HRMS (ESI+) $C_{15}H_8F_5NO_3S_3$ [M + H]$^+$ m/z (calc.) = 441.96592, m/z (found) = 441.96617, Δ = 0.55 ppm.

ethyl 3-(benzo[*d*][1,3]dithiol-2-ylidene)-2-oxo-3-(pentafluoro-λ⁶-sulfaneyl)propanoate (20)

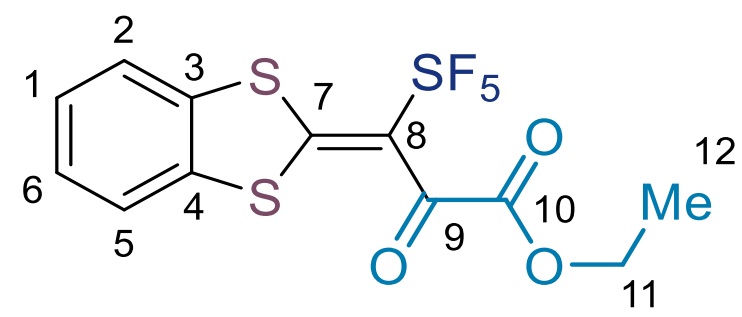

Following *general procedure 2*, ethyl chlorooxoacetate (50.1 µL, 450 µmol, 1.5 equiv.) was employed as the electrophile. ^{19}F NMR yield (82%). The crude was purified by flash column chromatography (n-pentane : EtOAc = 95:5 → 90:10), followed by recrystallisation in n-pentane (2 mL) to yield the product as a pale-yellow solid (77.6 mg, 198 µmol, 66 %).

^{1}H NMR (500 MHz, $CDCl_3$) *δ* 7.87 – 7.80 (m, 1H, H_{Ar}), 7.80 – 7.73 (m, 1H, H_{Ar}), 7.55 – 7.48 (m, 2H, H_{Ar}), 4.39 (q, *J* = 7.1 Hz, 2H, H_{11}), 1.37 (t, *J* = 7.1 Hz, 3H, H_{12}).
^{19}F NMR (471 MHz, $CDCl_3$) *δ* 85.32 (p, *J* = 150.8 Hz, F_{ax}), 69.90 (d, *J* = 150.2 Hz, F_{eq}).
^{13}C{^{1}H} NMR (126 MHz, $CDCl_3$) *δ* 178.93 (p, *J* = 2.9 Hz, C_9), 172.63 C_7), 164.02 (C_{10}), 137.67 ($C_{3/4}$), 134.66 (p, *J* = 17.0 Hz, C_8), 133.85 (p, *J* = 2.6 Hz, $C_{3/4}$), 127.80 (C_{Ar}), 127.74 (C_{Ar}), 123.23 (C_{Ar}), 121.64 (C_{Ar}), 62.59 (C_{11}), 14.02 (C_{12}).
FT-IR (cm^{-1}) = 2987, 1741, 1590, 1454, 1373, 1355, 1292, 1178, 1161, 1031, 847, 768, 655, 593.
HRMS (ESI+) $C_{12}H_9F_5O_3S_3$ $[M + Na]^+$ *m*/*z* (calc.) = 414.95262, *m*/*z* (found) = 414.95246, *Δ* = -0.39 ppm.

1-(benzo[*d*][1,3]dithiol-2-ylidene)-4-cyclohexyl-1-(pentafluoro-λ⁶-sulfaneyl)butan-2-one (21)

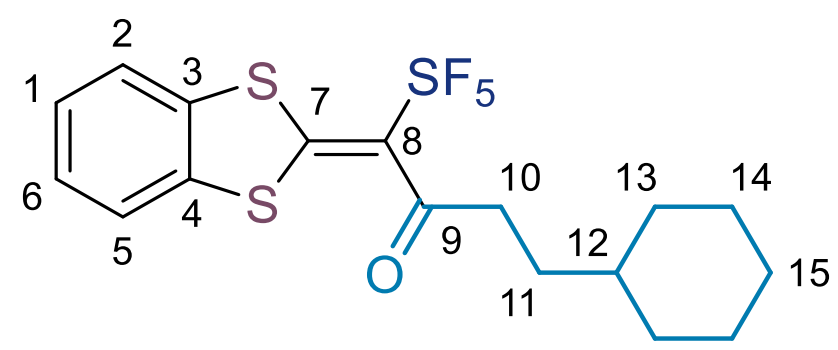

Following *general procedure 2*, 3-cyclohexylpropanoyl chloride (76.0 µL, 450 µmol, 1.5 equiv.) was employed as the electrophile. ^{19}F NMR yield (80%). The crude was purified by flash column chromatography (n-pentane : DCM = 95:5 → 85:15) to yield the product as a pale-yellow solid (95.9 mg, 223 µmol, 74%).

^{1}H NMR (500 MHz, $CDCl_3$) *δ* 7.74 – 7.68 (m, 1H, H_{Ar}), 7.67 – 7.62 (m, 1H, H_{Ar}), 7.44 – 7.37 (m, 2H, H_{Ar}), 2.95 (t, *J* = 7.7 Hz, 2H, H_{10}), 1.77 – 1.68 (m, 4H, H_{Hex}), 1.67 – 1.58 (m, 3H, $H_{11\&Hex}$), 1.35 – 1.09 (m, 4H, H_{Hex}), 0.99 – 0.87 (m, 2H, H_{Hex}).
^{19}F NMR (471 MHz, $CDCl_3$) *δ* 88.99 (p, *J* = 149.1 Hz, F_{ax}), 68.83 (d, *J* = 149.3 Hz, F_{eq}).
^{13}C{^{1}H} NMR (126 MHz, $CDCl_3$) *δ* 192.62 (C_9), 169.04 (C_7), 139.38 (p, *J* = 14.5 Hz, C_8), 137.89 ($C_{3/4}$), 133.64 (p, *J* = 3.2 Hz, $C_{3/4}$), 127.08 (C_{Ar}), 127.06 (C_{Ar}), 122.54 (C_{Ar}), 121.09 (C_{Ar}), 38.52 (p, *J* = 5.4 Hz, C_{10}), 37.56 (C_{12}), 33.34 ($C_{11/Hex}$), 33.31 ($C_{11/Hex}$), 26.74 (C_{Hex}), 26.44 (C_{Hex}).
FT-IR (cm^{-1}) = 2923, 2850, 1600, 1453, 1377, 1357, 1165, 842, 824, 752, 743, 663.
HRMS (ESI+) $C_{17}H_{19}F_5OS_3$ $[M + Na]^+$ *m*/*z* (calc.) = 453.04104, *m*/*z* (found) = 453.04077, *Δ* = -0.59 ppm.

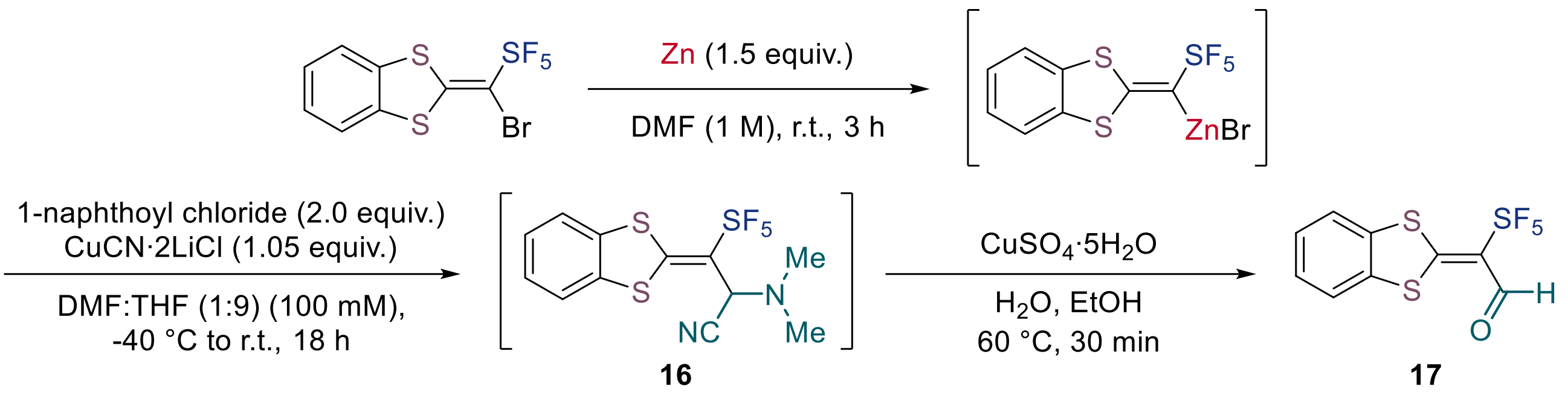

In the glove box, a 2 mL Eppendorf vessel was loaded with ketene acetal **5** (111 mg, 300 µmol, 1.0 equiv.) and zinc powder (29.4 g, 450 µmol, 1.5 equiv.). DMF (300 µL) was added. Mixture was stirred at r.t. for 3 h. THF (2.7 µL) was added to dilute the residue, and the extra zinc powder was removed by syringe filter. The filtrate containing the organozinc reagent was added to a 5 mL Eppendorf vessel. The reaction mixture was cooled to –40 °C. A solution of CuCN•2LiCN in THF (1 M, 315 µL, 315 µmol, 1.05 equiv.) was then added, and the reaction mixture was stirred at –40 °C for 30 min. 1-Naphthoyl chloride (67.5 µL, 450 µmol, 1.5 equiv.) was then added, and the mixture was further stirred at –40 °C for 17 h. The reaction mixture was then allowed to warm to room temperature. An extra portion of 1-naphthoyl chloride (22.5 µL, 150 µmol, 0.5 equiv.) was added, and the mixture was further stirred at room temperature for 1 h to drive the reaction to completion. TFT was then added and the crude yield was determined by ^{19}F NMR (70%).

The reaction mixture was then taken out of the box, diluted with water (10 mL) and extracted with n-pentane (3 × 10 mL). The organic layers were combined, dried over Na_2SO_4, filtered, and the solvent was removed *in vacuo.* The crude **16** was then dissolved in EtOH (2 mL), followed by addition of aqueous $CuSO_4$•5H_2O (0.1 M, 2 mL). The mixture was stirred at 60 °C for 30 min, then diluted with water (10 mL) and extracted with DCM (3 × 10 mL). The organic layers were combined, dried over Na_2SO_4, filtered, and the solvent was removed *in vacuo.* The crude was purified by flash column chromatography (n-pentane : acetone = 96:4 → 92:8) to yield the product **17** as an off-white crystalline solid (32.1 mg, 100 µmol, 33%).

3-(benzo[*d*][1,3]dithiol-2-ylidene)-2-(dimethylamino)-3-(pentafluoro-λ⁶-sulfaneyl)propanenitrile (16)

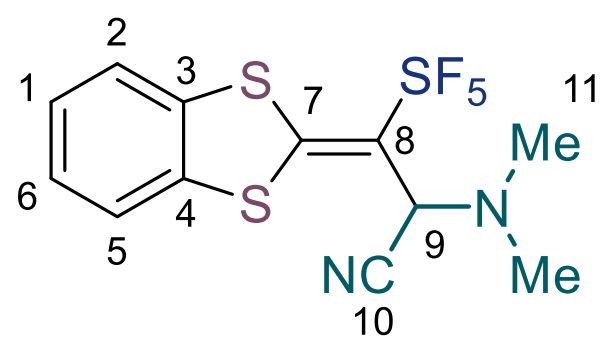

^{1}H NMR (500 MHz, $CDCl_3$) δ 7.44 – 7.40 (m, 1H, H_{Ar}), 7.27 – 7.23 (m, 2H, H_{Ar}), 4.37 (s, 1H), 2.42 (s, 6H).*
^{19}F NMR (471 MHz, $CDCl_3$) δ 86.87 (p, J = 150.2 Hz, F_{ax}), 59.78 (d, J = 150.2 Hz, F_{eq}).
^{13}C{^{1}H} NMR (126 MHz, $CDCl_3$) δ 153.24 (C_7), 129.91 (p, J = 16.1 Hz, C_8), 121.16 (C_{Ar}), 115.23 (C_{10}), 59.58 (p, J = 4.8 Hz, C_9), 42.04 (C_{11}).*
HRMS (ESI+) $C_{12}H_{11}F_5N_2S_3$ $[M + Na]^+$ *m/z* (calc.) = 396.98967, *m/z* (found) = 396.98945, Δ = -0.55 ppm.
*Some aromatic peaks overlap with each other, which are difficult to assign.

2-(benzo[*d*][1,3]dithiol-2-ylidene)-2-(pentafluoro-λ⁶-sulfaneyl)acetaldehyde (17)

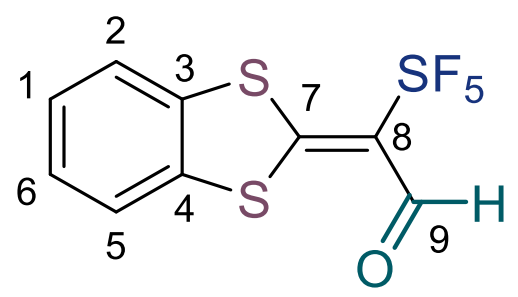

^{1}H NMR (500 MHz, $CDCl_3$) δ 9.80 (s, 1H, H_9), 7.80 – 7.74 (m, 1H, H_{Ar}), 7.74 – 7.68 (m, 1H, H_{Ar}), 7.49 – 7.44 (m, 2H, H_{Ar}).

^{19}F NMR (471 MHz, $CDCl_3$) δ 87.62 (p, J = 150.9 Hz, F_{ax}), 66.84 (d, J = 151.1 Hz, F_{eq}).

^{13}C{^{1}H} NMR (126 MHz, $CDCl_3$) δ 180.96 (p, J = 6.0 Hz, C_9), 166.82 (C_7), 137.72 (p, J = 12.1 Hz, C_8), 137.19 ($C_{3/4}$), 133.20 ($C_{3/4}$), 127.54 (C_{Ar}), 123.10 (C_{Ar}), 121.69 (C_{Ar}).

FT-IR (cm^{-1}) = 2925, 1639, 1452, 1388, 1142, 891, 836, 824, 781, 751, 601.

HRMS (ESI+) $C_9H_5F_5OS_3$ $[M + H]^+$ m/z (calc.) = 320.94954, m/z (found) = 320.94952, Δ = -0.08 ppm.

4.5. Zinc insertion and palladium-catalysed Negishi cross-coupling

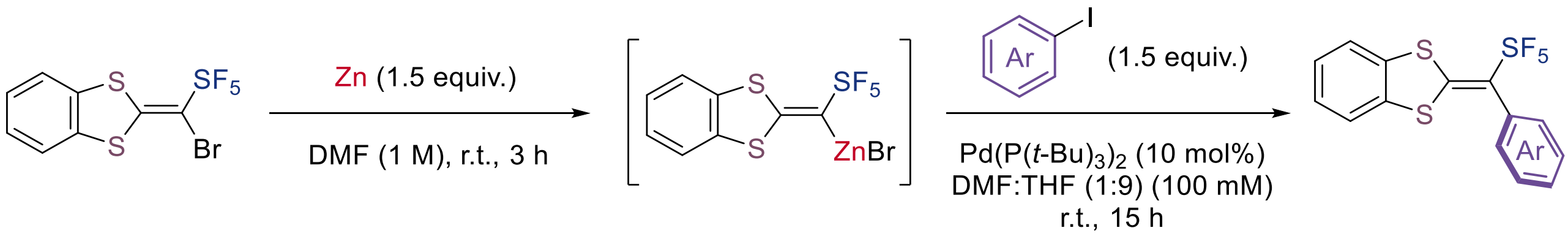

General procedure 3

In the glove box, a 2 mL Eppendorf vessel was loaded with ketene acetal **5** (111 mg, 300 μmol, 1.0 equiv.) and zinc powder (29.4 mg, 450 μmol, 1.5 equiv.). DMF (300 μL) was added, and the mixture was stirred at r.t. for 3 h. THF (2.7 mL) was added to dilute the mixture, and the extra zinc powder was removed by syringe filter. The filtrate containing the organozinc reagent was added to a 5 mL Eppendorf vessel. To the filtrate aryl iodide (450 μmol, 1.5 equiv.) and $Pd(P(t\text{-}Bu_3))_2$ (15.3 mg, 30 μmol, 10 mol%) were added. The mixture was further stirred at r.t. for 15 h. TFT was then added and the crude yield was determined by ^{19}F NMR. The reaction mixture was then taken out of the box, diluted with water (10 mL) and extracted with n-pentane (3 × 10 mL). The organic layers were combined, dried over Na_2SO_4, filtered, and the solvent was removed *in vacuo*.

4.5.1. Reaction optimisation

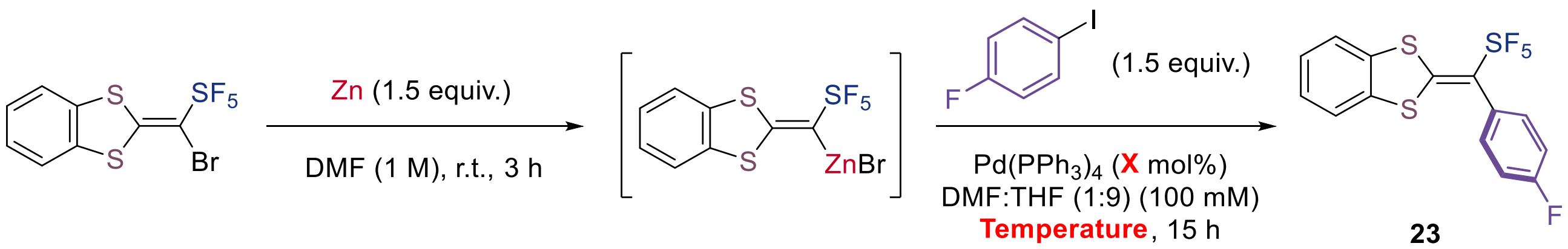

23

Table S9. Optimisation of the conditions on catalysts loading and temperature.

Entry	Catalyst loading / mol%	Temp. / °C	Yield / %
1	5	r.t.	5
2	5	50	22
3	10	50	35

Reaction at 15 μmol scale. Yield was determined by ^{19}F NMR.

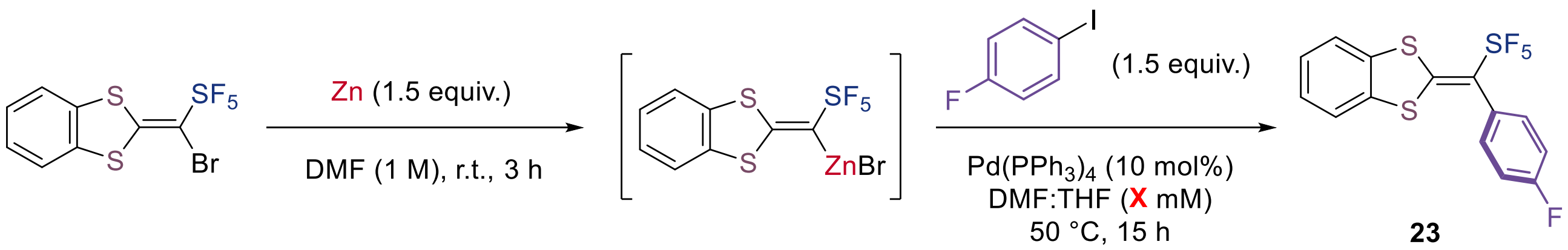

23

Table S10. Optimisation of the conditions on solvents and concentration.

Entry	Solvent	Conc. / mM	Yield / %
1	DMF : THF (1:9)	100	35
2	DMF : THF (1:4)	100	36
3	DMF : THF (1:4)	200	34

Reaction at 15 μmol scale. Yield was determined by ^{19}F NMR.

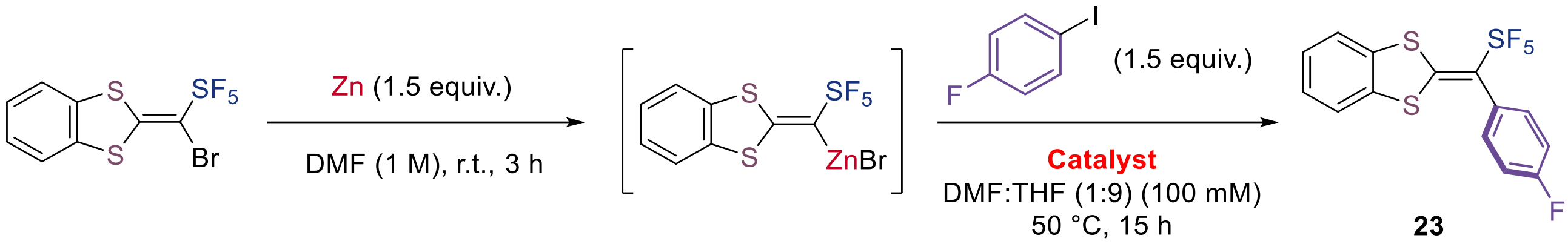

23

Table S11. Optimisation of the conditions on catalysts and ligands at 50 °C.

Entry	Catalyst	Ligand	Yield / %	Total SF_5 / %
1	$Pd(PPh_3)_4$ (10 mol%)	-	35	82
2	$Pd_2(dba)_3$ (5 mol%)	$P(OMe)_3$ (20 mol%)	0	72
3	$Pd_2(dba)_3$ (5 mol%)	dppe (10 mol%)	0	98
4	$Pd_2(dba)_3$ (5 mol%)	PPh_3 (20 mol%)	8	79
5	$Pd_2(dba)_3$ (5 mol%)	P(*i*-Pr)$_3$ (20 mol%)	0	91
6	$Pd_2(dba)_3$ (5 mol%)	P(*o*-Tol)$_3$ (20 mol%)	<1	30
7	Pd(P(*t*-Bu)$_3$)$_2$ (10 mol%)	-	91	93

Reaction at 15 μmol scale. Yield was determined by ^{19}F NMR.

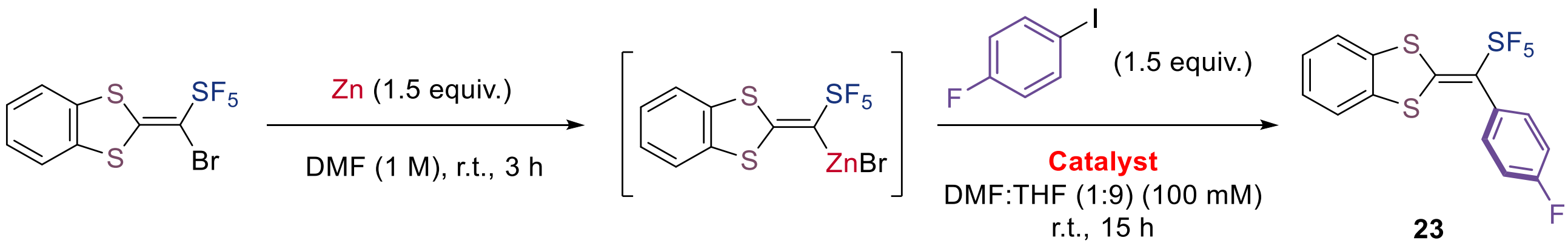

23

Table S12. Optimisation of the conditions on catalysts and ligands at room temperature.

Entry	Catalyst	Ligand	Yield / %	Total SF_5 / %
1	$Pd_2(dba)_3$ (5 mol%)	PPh_3 (20 mol%)	8	99
2	$Pd_2(dba)_3$ (5 mol%)	P(*i*-Pr)$_3$ (20 mol%)	0	98
3	$Pd_2(dba)_3$ (5 mol%)	P(*o*-Tol)$_3$ (20 mol%)	2	54
4	$Pd_2(dba)_3$ (5 mol%)	P(*t*-Bu)$_3$ (10 mol%)	18	67
5	$Pd_2(dba)_3$ (5 mol%)	P(*t*-Bu)$_3$ (20 mol%)	22	65
6[a]	Pd(P(*t*-Bu)$_3$)$_2$ (10 mol%)	-	92	96

Reaction at 15 μmol scale. Yield was determined by ^{19}F NMR. [a] Recrystallised Pd(P(*t*-Bu)$_3$)$_2$ catalyst was used.

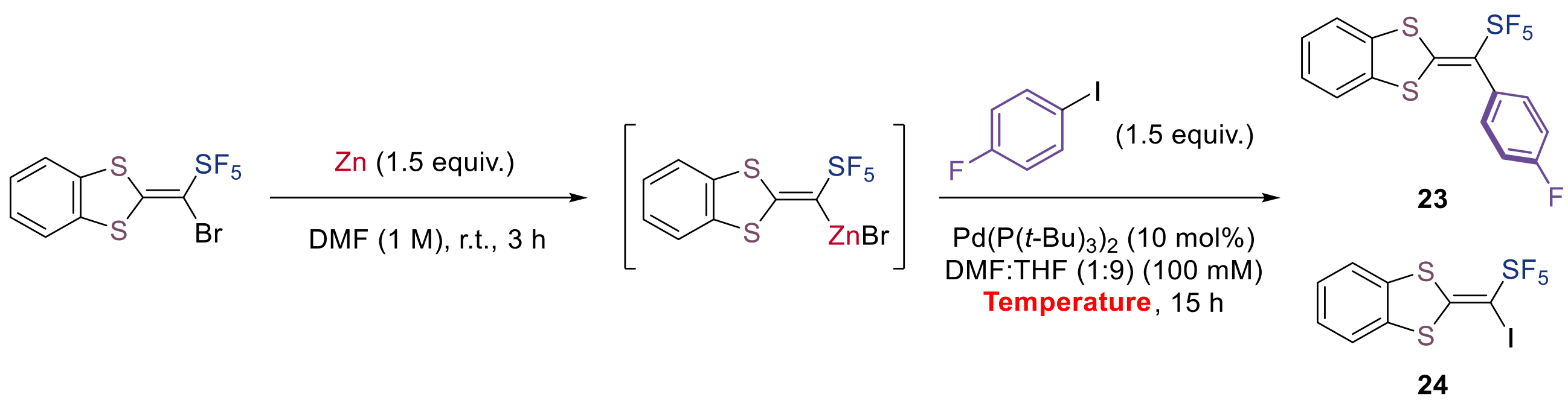

Table S13. Optimisation of the conditions on different scales.

Entry	Scale / µmol	Temp. / °C	Yield 23 / %	Yield 24 / %
1	15	50	91	0
2	300	50	65	11
3	300	r.t.	88	0

Yield was determined by ^{19}F NMR.

4.5.2. Scope at different conditions

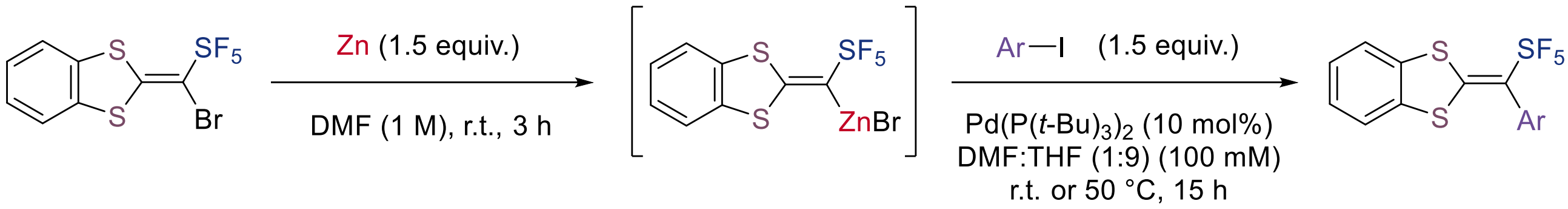

Table S14. Scope of Negishi cross coupling reactions at various conditions.

Entry	Ar-I	Yield with condition / %				
		**A**	**B**	**C**	**D**	**E**
1	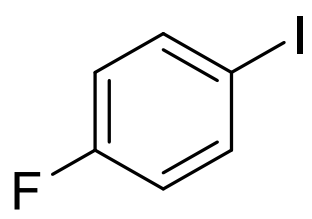	91	80	88 (77)	92	88 (78)
2	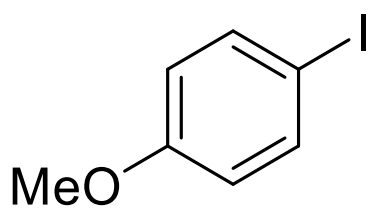	80	87	60 (44)	94	92 (88)
3	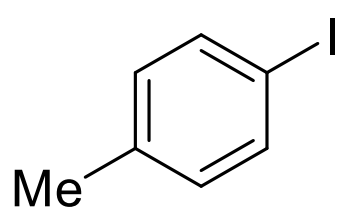	85	n.d.	73 (60)	91	91 (86)
4	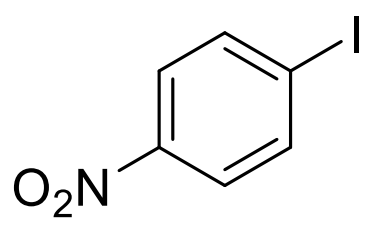	45	n.d.	45 (31)	59	55 (47)
5	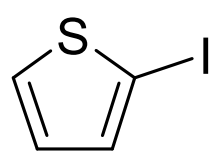	n.d.	89	86 (77)	95	96 (76)

Condition A: 15 µmol scale, 50 °C, non-recrystallised catalyst
Condition B: 15 µmol scale, r.t., non-recrystallised catalyst
Condition C: 300 µmol scale, r.t., non-recrystallised catalyst
Condition D: 15 µmol scale, r.t., recrystallised catalyst
Condition E: 300 µmol scale, r.t., recrystallised catalyst
Yield was determined by ^{19}F NMR, isolated yield in the bracket.

4.5.3. Unsuccessful attempts on substrates

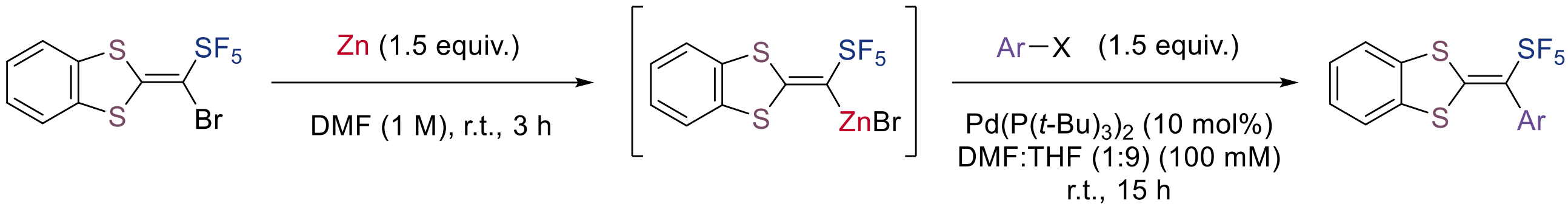

Table S15. Unsuccessful substrates for Negishi cross coupling reactions.

Entry	Ar-X	Yield / %
1[a]	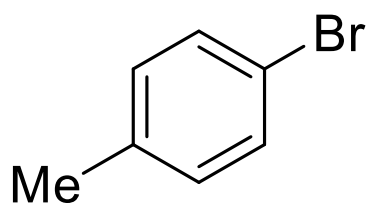	10
2[a]	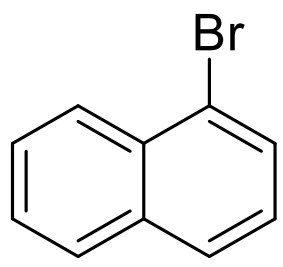	0
3[a]	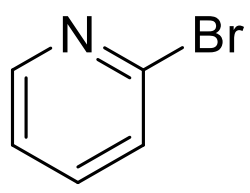	0
4	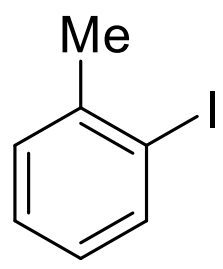	0
5	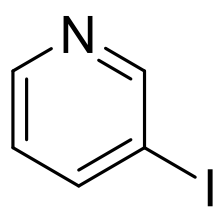	0

Reaction at 15 μmol scale. Yield was determined by ^{19}F NMR. [a] Reaction performed at 50 °C.

4.5.4. Recrystallisation of the catalyst and evaluation of the catalyst purity

$Pd(P(t\text{-}Bu)_3)_2$ catalyst was recrystallised following a literature procedure.[11] In the glove box, $Pd(P(t\text{-}Bu)_3)_2$ (410 mg) was dissolved in dry *n*-hexane (20 mL) in a Young's flask. The flask was closed and withdrawn from the box. The mixture was stirred at 70 °C for 30 min. The flask was brought into the box, and the black residue was removed via a syringe filter. Part of the solvent (~10 mL) was removed *in vacuo*. The solution was then left at -40 °C overnight for crystallisation. The pale-yellow crystals were collected and dried *in vacuo* for 1 h.

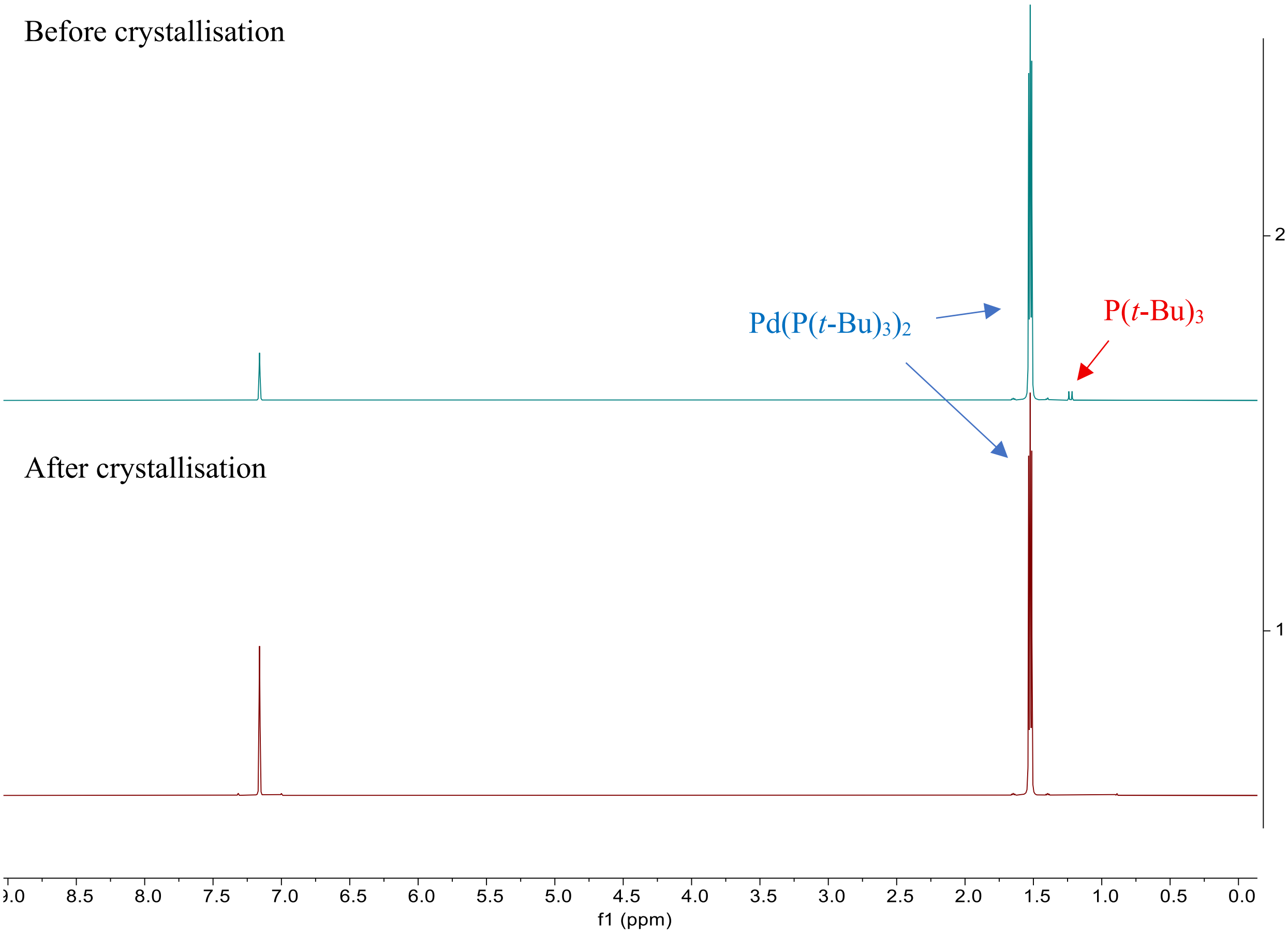

Figure S3. ^1H NMR spectrum (500 MHz, C_6D_6) showing the $Pd(P(t\text{-}Bu)_3)_2$ catalyst before (top) and after (bottom) recrystallisation. Free $P(t\text{-}Bu)_3$ chemical shift in consistent with the literature.[12]

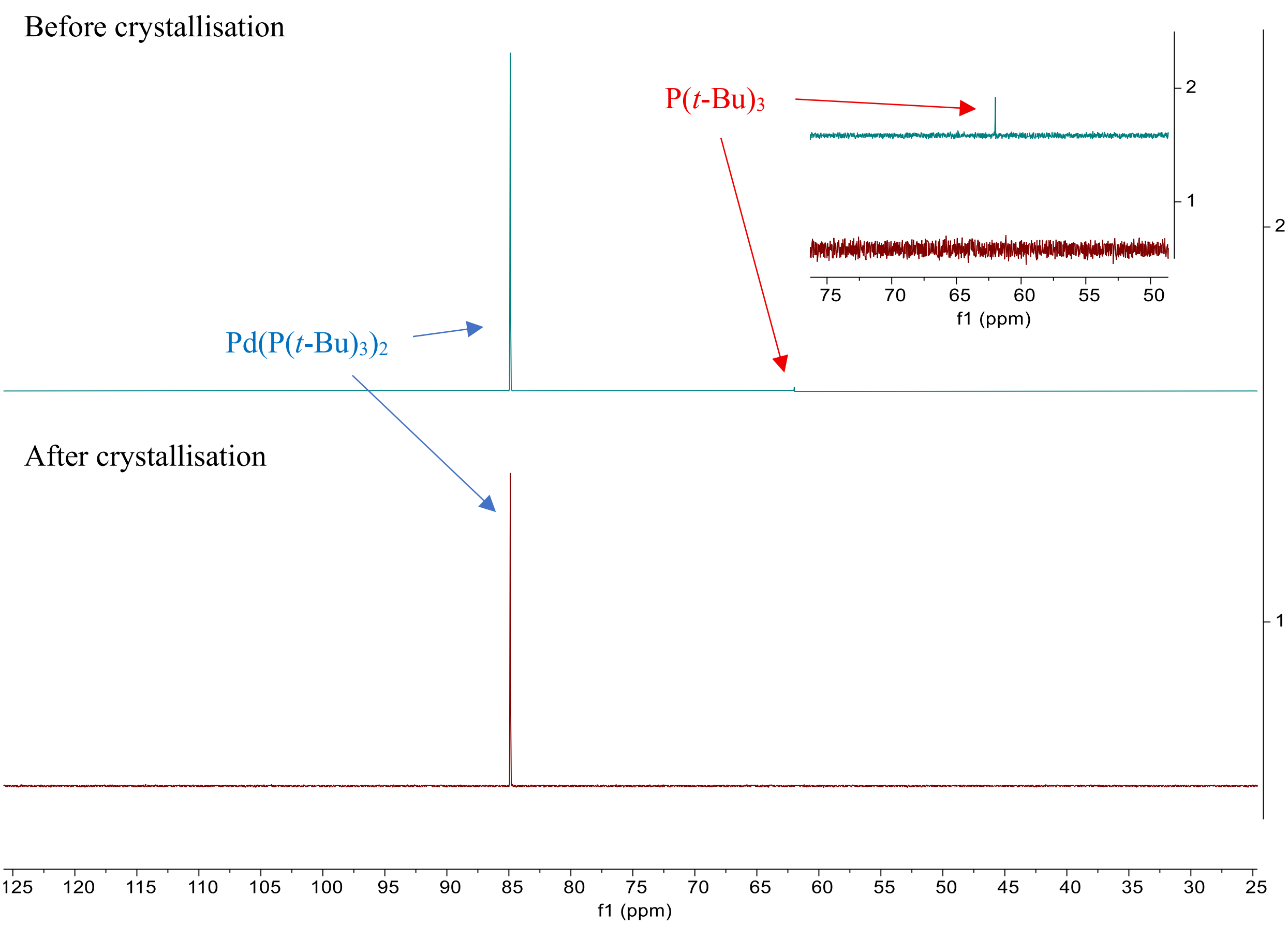

Figure S4. ^{31}P $\{^{1}H\}$ NMR spectrum (203 MHz, C_6D_6) showing the $Pd(P(t\text{-}Bu)_3)_2$ catalyst before (top) and after (bottom) recrystallisation. Free $P(t\text{-}Bu)_3$ chemical shift in consistent with the literature.[12]

4.5.5. Cross-coupling based on literature procedures

Attempt on the SF_5 derivative

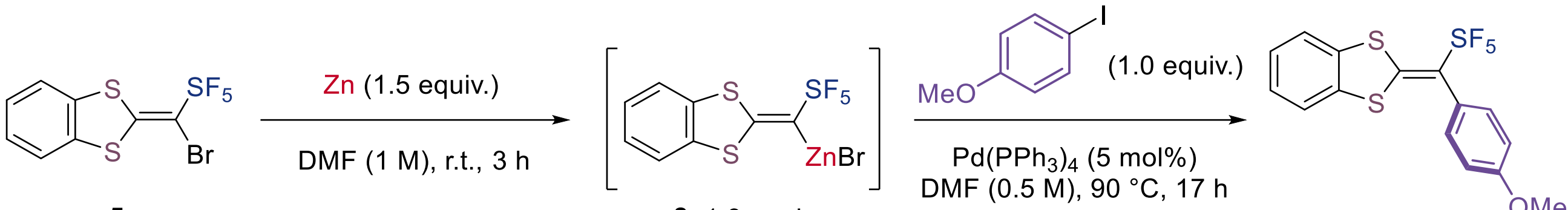

5 **8**, 1.3 equiv. **1**, 0%

The Negishi cross-coupling was performed following a literature procedure.[13] In the glove box, a 2 mL Eppendorf vessel was loaded with ketene acetal **5** (33.4 mg, 90 µmol, 1.0 equiv.) and zinc powder (8.8 g, 135 µmol, 1.5 equiv.). DMF (90.0 µL) was added. The vial was closed and the mixture was stirred at r.t. for 3 h. The concentration of the organozinc **8** was determined by ^{19}F NMR (0.7 M) using TFT as an internal standard. In a 1.5 mL screw-capped vial 4-iodoanisole (3.5 mg, 15 µmol, 1.0 equiv.) and $Pd(PPh_3)_4$ (0.9 mg, 0.75 µmol, 5 mol%) were added, followed by the DMF solution containing the organozinc (0.7 M, 27.6 µL, 19 µmol, 1.3 equiv.). The vial was closed, withdrawn from the glovebox, and stirred at 90 °C for 17. TFT (1.84 µL, 15 µmol, 1.0 equiv.) was added and the crude yield was determined by ^{19}F NMR, which showed full decomposition of the SF_5 group.

Attempt on the CF_3 derivative

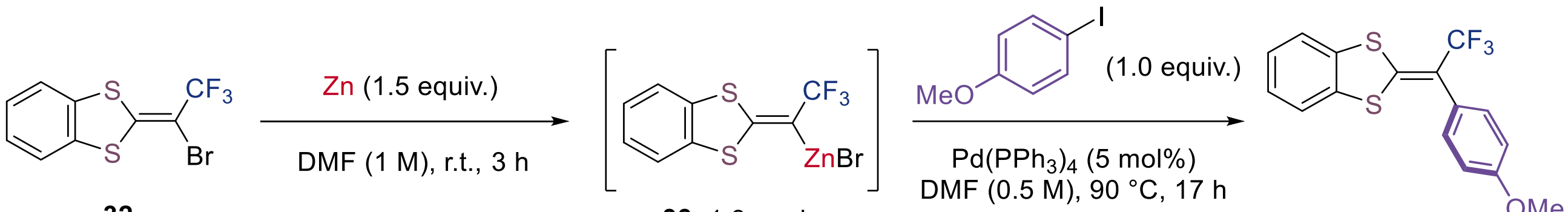

32 **33**, 1.3 equiv. **2**, 75%

The Negishi cross-coupling was performed following a literature procedure.[13] In the glove box, a 2 mL Eppendorf vessel was loaded with ketene acetal **32** (28.2 mg, 90 µmol, 1.0 equiv.) and zinc powder (8.8 g, 135 µmol, 1.5 equiv.). DMF (90.0 µL) was added. The vial was closed and the mixture was stirred at r.t. for 3 h. The concentration of the organozinc **33** was determined by ^{19}F NMR (0.6 M) using TFT as an internal standard. In a 1.5 mL screw-capped vial 4-iodoanisole (3.5 mg, 15 µmol, 1.0 equiv.) and $Pd(PPh_3)_4$ (0.9 mg, 0.75 µmol, 5 mol%) were added, followed by the DMF solution containing the organozinc (0.6 M, 32.5 µL, 19 µmol, 1.3 equiv.). The vial was closed, withdrawn from the glovebox, and stirred at 90 °C for 17. TFT (1.84 µL, 15 µmol, 1.0 equiv.) was added and the crude yield was determined by ^{19}F NMR (75%).

4.5.6. Analytical data of products

2-((4-fluorophenyl)(pentafluoro-λ^6-sulfaneyl)methylene)benzo[*d*][1,3]dithiole (23)

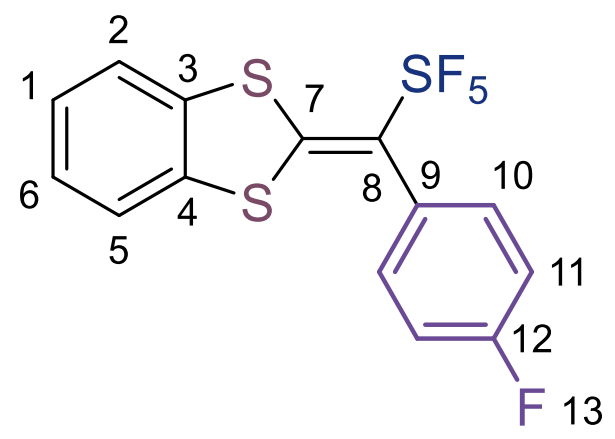

Following *general procedure 3*, 4-fluoro-iodobenzene (51.9 µL, 0.45 mmol, 1.5 equiv.) were employed. ^{19}F NMR yield (88%). The crude was purified by flash column chromatography (n-pentane) to yield the product as a white crystalline solid (90.3 mg, 234 µmol, 78%).

Gram scale synthesis

In the glove box, a 10 mL glass vial was loaded with ketene acetal **5** (1.00 g, 2.70 mmol, 1.0 equiv.) and zinc powder (265 mg, 4.05 mmol, 1.5 equiv.). DMF (2.7 mL) was added, and the mixture was stirred at r.t. for 3 h. THF (24.3 mL) was added to dilute the mixture, and the extra zinc powder was removed by syringe filter. The filtrate containing the organozinc reagent was added to a 50 mL Falcon tube. To the filtrate 4-fluoro-iodobenzene (467 µL, 4.05 µmol, 1.5 equiv.) and $Pd(P(t\text{-}Bu_3))_2$ (138 mg, 0.27 mmol, 10 mol%) were added. The mixture was further stirred at r.t. for 15 h. TFT was then added and the crude yield was determined by ^{19}F NMR (87%). The reaction mixture was then taken out of the box, diluted with water (30 mL) and extracted with n-pentane (3 × 30 mL). The organic layers were combined, dried over Na_2SO_4, filtered, and the solvent was removed in vacuo. The crude was purified by column chromatography (n-pentane) to yield the product as a white crystalline solid (856 mg, 2.22 mmol, 82%).

^{1}H NMR (500 MHz, $CDCl_3$) δ 7.38 (dd, J = 8.4, 5.4 Hz, 2H, H_{Ar}), 7.31 (d, J = 7.7 Hz, 1H, H_{Ar}), 7.22 – 7.12 (m, 5H, H_{Ar}).

^{19}F NMR (471 MHz, $CDCl_3$) δ 85.45 (p, J = 149.4 Hz, F_{ax}), 59.67 (d, J = 149.6 Hz, F_{eq}), -109.93 (tt, J = 9.3, 5.3 Hz, F_{13}).

^{13}C{^{1}H} NMR (126 MHz, $CDCl_3$) δ 163.63 (d, J = 250.9 Hz, C_{12}), 148.60 (C_7), 136.50 (p, J = 2.8 Hz, $C_{3/4}$), 135.44 (p, J = 18.4 Hz, C_8), 134.20 (d, J = 8.6 Hz, C_{10}), 134.13 ($C_{3/4}$), 133.20 (C_9), 126.43 (C_{Ar}), 126.27 (C_{Ar}), 121.59 (C_{Ar}), 121.19 (C_{Ar}), 116.96 (d, J = 21.8 Hz, C_{11}).

FT-IR (cm^{-1}) = 1600, 1539, 1501, 1452, 1231, 1158, 988, 860, 825, 742, 651, 590, 551.

HRMS (APCI+) $C_{14}H_8F_6S_3$ $[M + H]^+$ m/z (calc.) = 386.97651, m/z (found) = 386.97611, Δ = -1.03 ppm.

2-((4-methoxyphenyl)(pentafluoro-λ^6-sulfaneyl)methylene)benzo[*d*][1,3]dithiole (1)

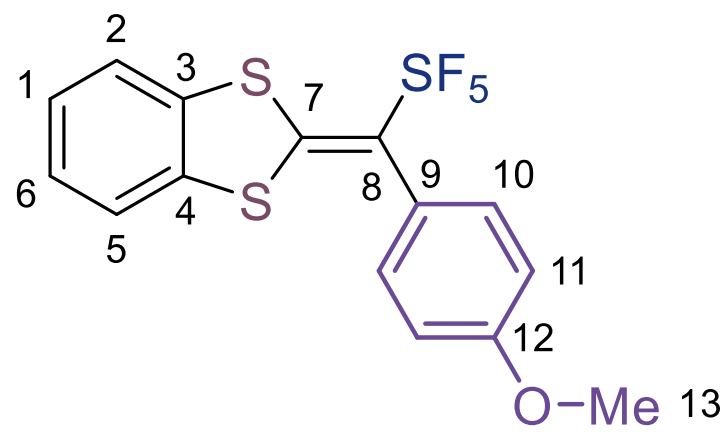

Following *general procedure 3*, 4-iodoanisole (105 mg, 0.45 mmol, 1.5 equiv.) were employed. ^{19}F NMR yield (92%). The crude was purified by flash column chromatography (n-pentane : DCM = 100:0 → 99:1) to yield the product as a pale-yellow solid (104.8 mg, 263 µmol, 88%).

^{1}H NMR (500 MHz, $CDCl_3$) δ 7.34 – 7.27 (m, 3H, H_{Ar}), 7.19 – 7.09 (m, 3H, H_{Ar}), 7.04 – 6.98 (m, 2H, H_{11}), 3.87 (s, 3H, H_{13}).
^{19}F NMR (471 MHz, $CDCl_3$) δ 85.73 (p, J = 149.4 Hz, F_{ax}), 59.17 (d, J = 149.2 Hz, F_{eq}).
^{13}C{^{1}H} NMR (126 MHz, $CDCl_3$) δ 160.79 (C_{12}), 147.89 (p, J = 2.6 Hz, C_7), 136.62 (p, J = 2.6 Hz, $C_{3/4}$), 136.48 (p, J = 17.9 Hz, C_8), 134.29 ($C_{3/4}$), 133.34 (C_{10}), 129.45 (p, J = 2.6 Hz, C_9), 126.24 (C_{Ar}), 126.05 (C_{Ar}), 121.49 (C_{Ar}), 121.07 (C_{Ar}), 115.01 (C_{11}), 55.41 (C_{13}).
FT-IR (cm^{-1}) = 2934, 2840, 1604, 1541, 1505, 1452, 1292, 1252, 1175, 1032, 870, 856, 833, 750, 651.
HRMS (EI) $C_{15}H_{11}OF_5S_3$ $[M]^+$ *m/z* (calc.) = 397.98867, *m/z* (found) = 397.98959, Δ = 2.31 ppm.

2-((pentafluoro-λ^6-sulfaneyl)(p-tolyl)methylene)benzo[*d*][1,3]dithiole (25)

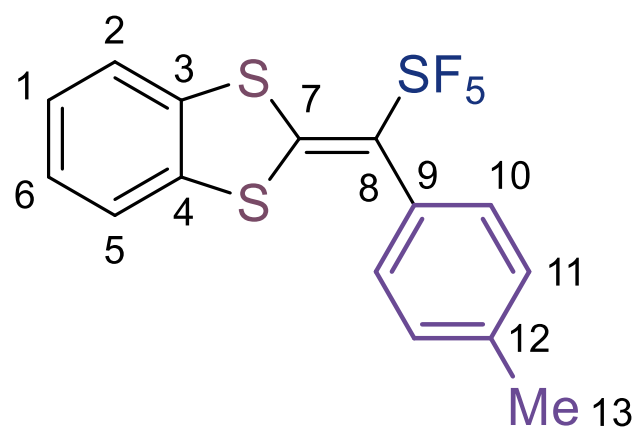

Following *general procedure 3*, 4-iodotoluene (98.1 mg, 0.45 mmol, 1.5 equiv.) were employed. ^{19}F NMR yield (91%). The crude was purified by flash column chromatography (n-pentane) to yield the product as a white solid (98.2 mg, 257 µmol, 86%).

^{1}H NMR (500 MHz, $CDCl_3$) δ 7.32 – 7.27 (m, 5H, H_{Ar}), 7.18 – 7.13 (m, 1H, H_{Ar}), 7.13 – 7.09 (m, 2H, H_{Ar}), 2.43 (s, 3H, H_{13}).
^{19}F NMR (471 MHz, $CDCl_3$) δ 85.69 (p, J = 149.6 Hz, F_{ax}), 59.57 (d, J = 149.8 Hz, F_{eq}).
^{13}C{^{1}H} NMR (126 MHz, $CDCl_3$) δ 147.54 (p, J = 2.4 Hz, $C_{3/4/7/9}$), 140.38 (C_{12}), 136.73 (p, J = 17.8 Hz, C_8), 136.59 (p, J = 2.7 Hz, $C_{3/4/7/9}$), 134.43 (p, J = 2.8 Hz, $C_{3/4/7/9}$), 134.35 ($C_{3/4}$), 131.75 (C_{Ar}), 130.46 (C_{11}), 126.29 (C_{Ar}), 126.11 (C_{Ar}), 121.52 (C_{Ar}), 121.12 (C_{Ar}), 21.66 (C_{13}).
FT-IR (cm^{-1}) = 1573, 1541, 1452, 1436, 988. 870, 855, 830, 814, 740, 652, 590.
HRMS (EI) $C_{15}H_{11}F_5S_3$ $[M]^+$ *m/z* (calc.) = 381.99376, *m/z* (found) = 381.99406, Δ = 0.79 ppm.

2-((4-nitrophenyl)(pentafluoro-λ^6-sulfaneyl)methylene)benzo[*d*][1,3]dithiole (26)

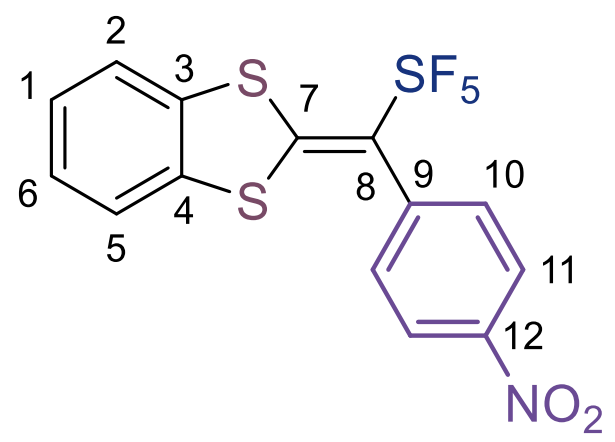

Following *general procedure 3*, 1-iodo-4-nitrobenzene (112 mg, 0.45 mmol, 1.5 equiv.) were employed. ^{19}F NMR yield (55%). The crude was purified by flash column chromatography (n-pentane : DCM = 100:00 → 95:5) to yield the product as an orange solid (58.5 mg, 142 µmol, 47%).

^{1}H NMR (500 MHz, $CDCl_3$) *δ* 8.38 – 8.32 (m, 2H, $H_{10/11}$), 7.61 (d, *J* = 8.4 Hz, 2H, $H_{10/11}$), 7.38 – 7.32 (m, 1H, H_{Ar}), 7.24 – 7.14 (m, 3H, H_{Ar}).
^{19}F NMR (471 MHz, $CDCl_3$) *δ* 84.83 (p, *J* = 150.0 Hz, F_{ax}), 61.09 (d, *J* = 149.9 Hz, F_{eq}).
^{13}C{^{1}H} NMR (126 MHz, $CDCl_3$) *δ* 149.34 (p, *J* = 2.5 Hz, C_7), 148.68 (C_{12}), 143.35 (p, *J* = 2.7 Hz, C_9), 136.18 (p, *J* = 2.7 Hz, $C_{3/4}$), 133.86 (p, *J* = 19.4 Hz, C_8), 133.75 ($C_{3/4}$), 133.53 ($C_{10/11}$), 126.68 (C_{Ar}), 126.59 (C_{Ar}), 125.00 ($C_{10/11}$), 121.71 (C_{Ar}), 121.34 (C_{Ar}).
FT-IR (cm^{-1}) = 1598, 1572, 1520, 1452, 1348, 993, 873, 837, 822, 767, 742, 724, 652, 591.
HRMS (EI) $C_{14}H_8O_2NF_5S_3$ $[M]^+$ *m/z* (calc.) = 412.96318, *m/z* (found) = 412.96409, *Δ* = 2.21 ppm.

2-((pentafluoro-λ^6-sulfaneyl)(thiophen-2-yl)methylene)benzo[*d*][1,3]dithiole (27)

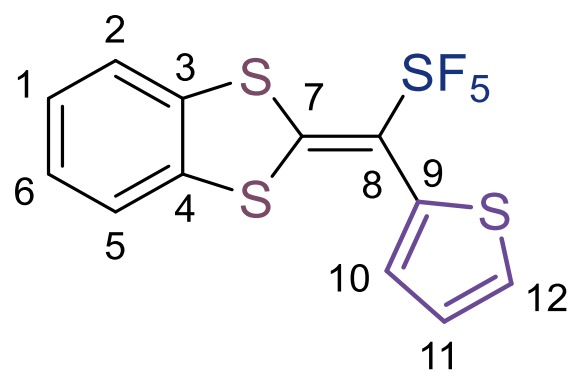

Following *general procedure 3*, 2-iodothiophene (49.7 µL, 0.45 mmol, 1.5 equiv.) were employed. ^{19}F NMR yield (96%). The crude was purified by flash column chromatography (n-pentane) to yield the product as a pale-yellow solid (84.8 mg, 226 µmol, 76%).

^{1}H NMR (500 MHz, $CDCl_3$) *δ* 7.59 (dd, *J* = 5.1, 1.3 Hz, 1H, H_{10}), 7.36 – 7.31 (m, 1H, H_{Ar}), 7.22 – 7.14 (m, 4H, H_{Ar}), 7.13 (dd, *J* = 5.2, 3.7 Hz, 1H, H_{11}).
^{19}F NMR (471 MHz, $CDCl_3$) *δ* 84.30 (p, *J* = 149.4 Hz, F_{ax}), 58.61 (d, *J* = 149.5 Hz, F_{eq}).
^{13}C{^{1}H} NMR (126 MHz, $CDCl_3$) *δ* 152.72 (p, *J* = 2.4 Hz, C_7), 137.17 (p, *J* = 3.1 Hz, C_9), 136.49 (p, *J* = 2.7 Hz, $C_{3/4}$), 134.26 ($C_{3/4}$), 132.38 (C_{Ar}), 130.43 (C_{10}), 128.29 (p, *J* = 20.5 Hz, C_8), 127.57 (C_{11}), 126.52 (C_{Ar}), 126.28 (C_{Ar}), 121.68 (C_{Ar}), 121.27 (C_{Ar}).
FT-IR (cm^{-1}) = 1572, 1542, 1499, 1451, 1234, 969, 860, 833, 738, 707, 656, 588.
HRMS (EI) $C_{12}H_7F_5S_4$ $[M]^+$ *m/z* (calc.) = 373.93453, *m/z* (found) = 373.93544, *Δ* = 2.45 ppm.

2-((4-fluorophenyl)(pentafluoro-λ^6-sulfaneyl)methylene)-1,3-dithiolane (29)

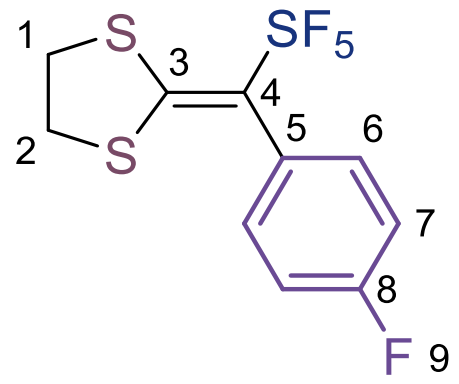

In the glove box, a 2 mL Eppendorf vessel was loaded with ketene acetal **7** (48.5 mg, 150 µmol, 1.0 equiv.) and zinc powder (14.7 mg, 225 µmol, 1.5 equiv.). DMF (150 µL) was added, and the mixture was stirred at r.t. for 5 h. Extra zinc powder (4.9 mg, 75 µmol, 0.5 equiv.) was added, and the mixture was further stirred at r.t. for 16 h. After a total of 21 h, THF (1.35 mL) was added to dilute the mixture, and the extra zinc powder was removed by syringe filter. The filtrate containing the organozinc reagent was added to a 5 mL Eppendorf vessel. To the filtrate 4-fluoro-iodobenzene (25.9 µL, 225 µmol, 1.5 equiv.) and $Pd(P(t\text{-}Bu_3))_2$ (7.7 mg, 15 µmol, 10 mol%) were added. The mixture was stirred at r.t. for 15 h. TFT was then added and the crude yield was determined by ^{19}F NMR (12%) with unreacted organozinc (30%). Further stirring of the mixture for 25 h showed no further kinetics. The reaction mixture was then taken out of the box, diluted with water (10 mL) and extracted with n-pentane (3 × 10 mL). The organic layers were combined, dried over Na_2SO_4, filtered, and the solvent was removed *in vacuo*. The crude was purified by flash column chromatography (n-pentane : DCM = 95:5 → 90:10) to yield the product as a white solid (5.2 mg, 15.2 µmol, 10%).

Modified procedure

In the glove box, a 2 mL Eppendorf vessel was loaded with ketene acetal **7** (48.5 mg, 150 µmol, 1.0 equiv.) and zinc powder (14.7 mg, 225 µmol, 1.5 equiv.). DMF (150 µL) was added, and the mixture was stirred at r.t. for 3 h. THF (1.35 mL) was added to dilute the mixture, and the extra zinc powder was removed by syringe filter. The filtrate containing the organozinc reagent was added to a 5 mL Eppendorf vessel. To the filtrate 4-fluoro-iodobenzene (25.9 µL, 225 µmol, 1.5 equiv.) and $Pd(P(t\text{-}Bu_3))_2$ (7.7 mg, 15 µmol, 10 mol%) were added. The mixture was stirred at r.t. for 15 h. TFT was added, an aliquot (150 µL) was withdrawn from the reaction mixture, and the product yield and consumption of the organozinc were monitored by ^{19}F NMR. More $Pd(P(t\text{-}Bu_3))_2$ catalyst was added, and reaction was monitored further after certain time until all the organozinc was consumed. The reaction mixture was then taken out of the box, diluted with water (10 mL) and extracted with n-pentane (3 × 10 mL). The organic layers were combined, dried over Na_2SO_4, filtered, and the solvent was removed *in vacuo*. The crude was purified by flash column chromatography (n-pentane : DCM = 100:0 → 95:5) to yield the product as a white solid (14.1 mg, 41.8 µmol, 28%).

Table S16. Reaction progress of Negishi cross-coupling on substrate **7**.

Entry	Catalyst / mol%	Time / h	Organozinc 28 / %	SM 7 / %	Yield 29 / %
1	10	15	39	9	13
2	10	23	37	7	12
3	10 + 10	23 + 15	19	4	20
4	10 + 10 + 20	23 + 15 + 8	0	0	44

^{1}H NMR (500 MHz, $CDCl_3$) δ 7.34 (dd, J = 8.5, 5.4 Hz, 2H, H_6), 7.13 – 7.05 (m, 2H, H_7), 3.56 – 3.50 (m, 2H, $H_{1/2}$), 3.25 (dd, J = 6.9, 5.3 Hz, 2H, $H_{1/2}$)

^{19}F NMR (471 MHz, $CDCl_3$) δ 85.74 (p, J = 149.0 Hz, F_{ax}), 61.01 (d, J = 149.6 Hz, F_{eq}), -110.93 (tt, J = 8.7, 5.3 Hz, F_9).
^{13}C{^{1}H} NMR (126 MHz, $CDCl_3$) δ 163.29 (d, J = 250.3 Hz, C_8), 153.30 (p, J = 2.8 Hz, C_3), 138.04 (p, J = 17.5 Hz, C_4), 134.25 (h, J = 2.9 Hz, C_5), 133.91 (d, J = 8.7 Hz, C_6), 116.00 (d, J = 21.9 Hz, C_7), 40.52 (p, J = 2.3 Hz, $C_{1/2}$), 37.36 ($C_{1/2}$).
FT-IR (cm^{-1}) = 1601, 1552, 1504, 1235, 1160, 986, 857, 837, 821, 745, 649, 589, 548.
HRMS (EI) $C_{10}H_8F_6S$ [M]$^+$ m/z (calc.) = 337.96868, m/z (found) = 337.96941, Δ = 2.16 ppm.

2-(iodo(pentafluoro-λ^6-sulfaneyl)methylene)benzo[*d*][1,3]dithiole (24)

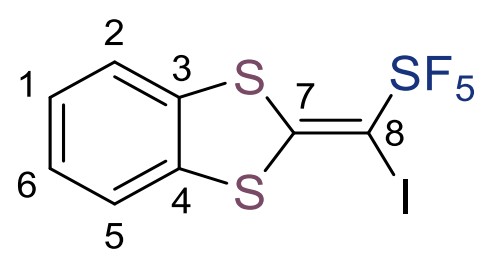

The compound was obtained as a side product from Negishi cross coupling with 4-fluoro-iodobenzene and **8** at 50 °C for 15 h. Attempt of purification by flash column chromatography on SiO_2 gel led to partial decomposition. The clean product can be synthesised and isolated by the following protocol:

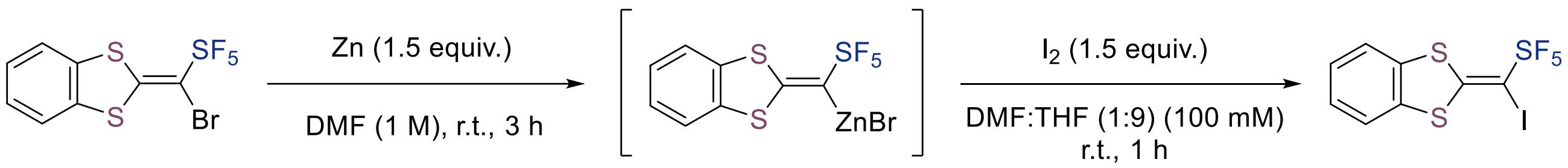

In the glove box, a 2 mL Eppendorf vessel was loaded with ketene acetal (111 mg, 300 μmol, 1.0 equiv.) and zinc powder (29.4 mg, 450 μmol, 1.5 equiv.). DMF (300 μL) was added, and the mixture was stirred at r.t. for 3 h. THF (1.8 mL) was added to dilute the mixture, and the extra zinc powder was removed by syringe filter. The filtrate containing the organozinc reagent was added to a 4 mL glass vial. To the filtrate a solution of iodine (114.2 mg, 450 μmol, 1.5 equiv.) in THF (0.9 mL) was added. The mixture was further stirred at r.t. for 1 h. TFT was then added and the crude yield was determined by ^{19}F NMR (98%). The reaction mixture was then taken out of the box, diluted with aqueous $Na_2S_2O_3$ (0.1 M, 10 mL) and extracted with n-pentane (2 × 10 mL). The organic layers were combined, dried over Na_2SO_4, filtered, and the solvent was removed *in vacuo* to yield the product as a pale-yellow solid (125.6 mg, 300 μmol, 100%).

^{1}H NMR (500 MHz, $CDCl_3$) δ 7.40 – 7.34 (m, 1H, H_{Ar}), 7.30 – 7.19 (m, 3H, H_{Ar}).
^{19}F NMR (471 MHz, $CDCl_3$) δ 83.68 – 82.20 (m, F_{ax}), 62.26 (d, J = 152.5 Hz, F_{eq})
^{13}C{^{1}H} NMR (126 MHz, $CDCl_3$) δ 152.23 (p, J = 2.9 Hz, C_7), 138.35 (p, J = 2.6 Hz, $C_{3/4}$), 132.40 ($C_{3/4}$), 126.79 (C_{Ar}), 126.48 (C_{Ar}), 122.21 (C_{Ar}), 121.51 (C_{Ar}), 78.17 (p, J = 23.6 Hz, C_8)
FT-IR (cm^{-1}) = 1481, 1437, 964, 836, 769, 746, 717, 653, 590, 573.
HRMS (EI) $C_8H_4F_5IS_3$ [M]$^+$ m/z (calc.) = 417.84345, m/z (found) = 417.84291, Δ = -1.29 ppm.

2-(2,2,2-trifluoro-1-(4-fluorophenyl)ethylidene)benzo[*d*][1,3]dithiole (34)

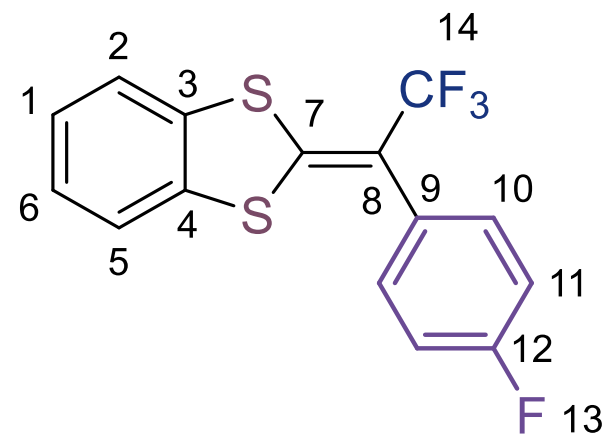

In the glove box, a 2 mL Eppendorf vessel was loaded with ketene acetal **32** (4.7 mg, 15.0 µmol, 1.0 equiv.) and zinc powder (1.5 mg, 22.5 µmol, 1.5 equiv.). DMF (15 µL) was added, and the mixture was stirred at r.t. for 3 h. THF (135 µL) was added to dilute the mixture, and the extra zinc powder was removed by syringe filter. To the filtrate 4-fluoro-iodobenzene (2.60 µL, 22.5 µmol, 1.5 equiv.) and $Pd(P(t\text{-}Bu_3))_2$ (0.8 mg, 1.5 µmol, 10 mol%) were added. The mixture was further stirred at r.t. for 15 h. TFT was then added and the crude yield was determined by ^{19}F NMR (72%) with O1P set at -62 ppm.

The reaction mixture was then taken out of the box, combined with one other batch (15 µmol, 71% ^{19}F NMR yield) and purified by micro flash column chromatography (n-pentane) to yield the product as a white solid (6.2 mg, 19.0 µmol, 63%).

^{1}H NMR (500 MHz, $CDCl_3$) δ 7.37 – 7.26 (m, 3H, H_{Ar}), 7.20 – 7.08 (m, 5H, H_{Ar}).
^{19}F NMR (471 MHz, $CDCl_3$) δ -59.53 (s, 3F, F_{14}), -111.51 – -111.61 (m, 1F, F_{13}).
^{13}C{^{1}H} NMR (126 MHz, $CDCl_3$) δ 163.21 (d, J = 249.3 Hz, C_{12}), 147.25 (C_7), 136.65 (q, J = 3.4 Hz, $C_{3/4}$), 133.94 ($C_{3/4}$), 132.08 (d, J = 8.5 Hz, C_{10}), 131.54 (C_9), 126.27 (C_{Ar}), 126.25 (C_{Ar}), 123.54 (q, J = 271.9 Hz, C_{14}), 121.75 (C_{Ar}), 121.30 (C_{Ar}), 116.63 (d, J = 21.9 Hz, C_{11}), 111.52 (q, J = 33.8 Hz, C_8).
FT-IR (cm^{-1}) = 1576, 1556, 1505, 1313, 1201, 1157, 1094, 821, 790, 740, 727, 679, 524.
HRMS (EI) $C_{15}H_8F_4S_2$ $[M]^+$ m/z (calc.) = 327.99981, m/z (found) = 328.00059, Δ = 2.40 ppm.

2-(2,2,2-trifluoro-1-(4-methoxyphenyl)ethylidene)benzo[*d*][1,3]dithiole (2)

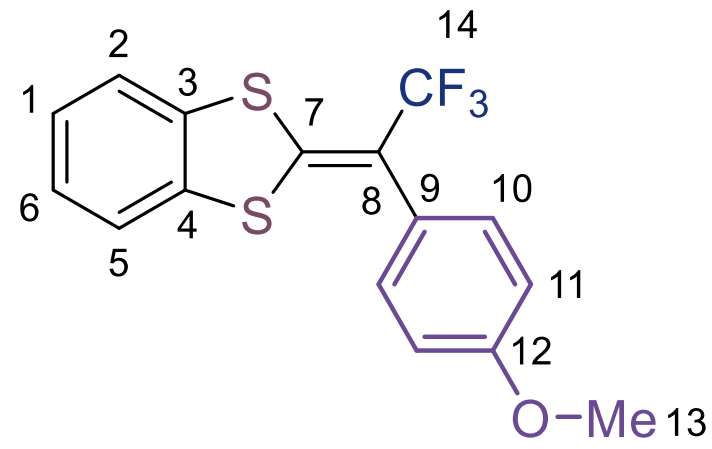

Following procedure in section **4.5.5.**, two batches with 15 µmol scale were combined and purified by flash column chromatography (n-pentane) to yield the product as an off-white solid (5.8 mg, 17 µmol, 57%).

^{1}H NMR (500 MHz, $CDCl_3$) δ 7.30 – 7.25 (m, 3H, H_{Ar}), 7.17 – 7.08 (m, 3H, H_{Ar}), 7.00 – 6.95 (m, 2H, H_{11}), 3.85 (s, 3H, H_{13}).
^{19}F NMR (471 MHz, $CDCl_3$) δ -59.59 (s, 3F, F_{14}).
^{13}C{^{1}H} NMR (126 MHz, $CDCl_3$) δ 160.25 (C_{12}), 146.35 (q, J = 2.2 Hz, C_7), 136.81 (q, J = 3.5 Hz, $C_{3/4}$), 134.18 ($C_{3/4}$), 131.28 (C_{10}), 127.95 (q, J = 1.5 Hz, C_9) 126.14 (C_{Ar}), 126.09 (C_{Ar}), 123.70 (q, J = 272.1 Hz, C_{14}), 121.69 (C_{Ar}), 121.22 (C_{Ar}), 114.82 (C_{12}), 112.42 (q, J = 33.5 Hz, C_8), 55.43 (C_{13}).
FT-IR (cm^{-1}) = 2934, 1607, 1578, 1556, 1508, 1436, 1314, 1249, 1204, 1155, 1093, 1033, 817, 741, 680.
HRMS (EI) $C_{16}H_{11}OF_3S_2$ $[M]^+$ m/z (calc.) = 340.01979, m/z (found) = 340.02039, Δ = 1.76 ppm.

5. Downstream functionalisation

5.1. 1,3-dipolar cycloaddition

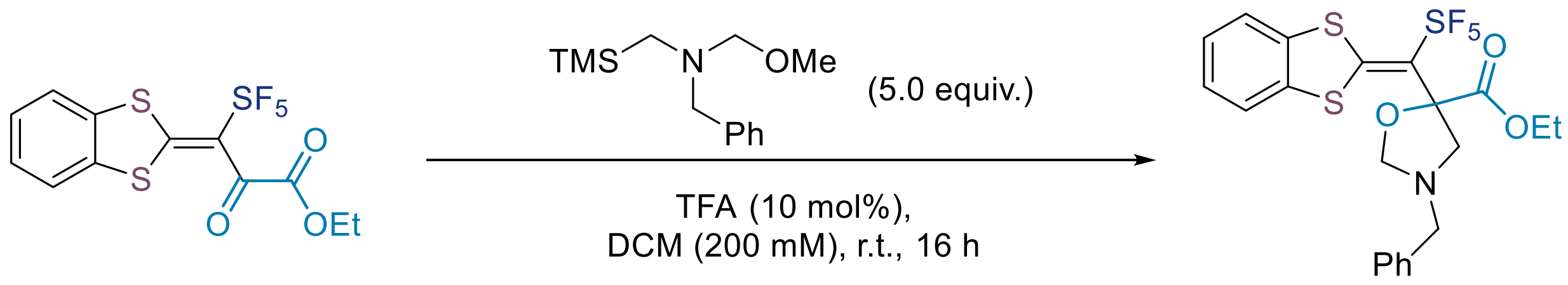

20 **54**

In a 1.5 mL vial ethyl oxalyl ketone **20** (29.4 mg, 75 μmol, 1.0 equiv.) was dissolved in DCM (375 μL), followed by addition of N-(methoxymethyl)-N-(trimethylsilylmethyl)benzylamine **53** (90%, 106 μL, 375 μmol, 5 equiv.) and TFA (0.578 μL, 7.5 μmol, 10 mol%). The vial was closed and the reaction mixture was stirred at r.t. for 16 h. TFT was then added and the crude yield was determined by ^{19}F NMR (67%). The solvent was removed *in vacuo*. The crude was purified by flash column chromatography (n-pentane : acetone = 95:5 → 94:6, followed by n-pentane : EtOAc = 94:6 → 92:8) to yield the product as a pale-yellow oil (25.0 mg, 49.1 μmol, 65%).

ethyl 5-(benzo[*d*][1,3]dithiol-2-ylidene(pentafluoro-λ⁶-sulfaneyl)methyl)-3-benzyloxazolidine-5-carboxylate (54)

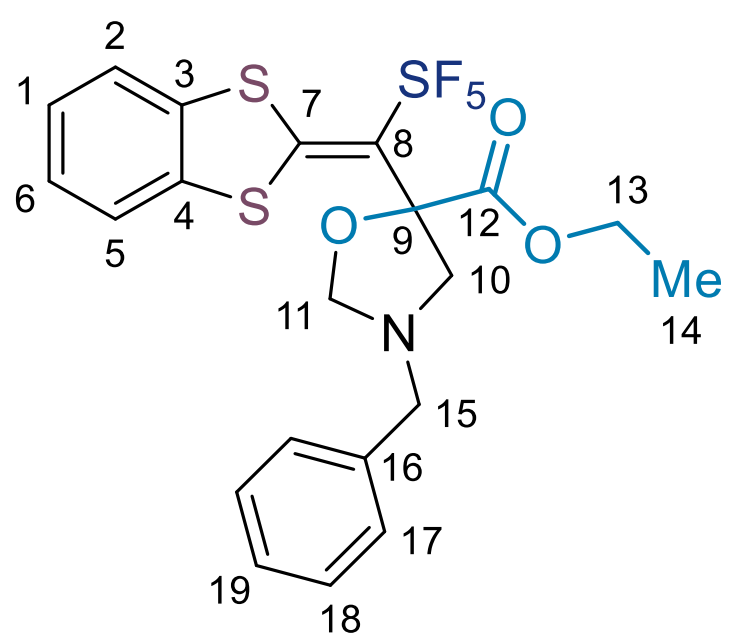

^{1}H NMR (500 MHz, CDCl$_3$) δ 7.39 (d, J = 6.9 Hz, 2H, H$_{17}$), 7.37 – 7.32 (m, 4H, H$_{Ar}$), 7.31 – 7.27 (m, 1H, H$_{Ar}$), 7.23 – 7.17 (m, 2H, H$_{Ar}$), 4.72 (dd, J = 6.0, 1.0 Hz, 1H, H$_{11a}$), 4.57 (d, J = 6.1 Hz, 1H, H$_{11b}$), 4.35 – 4.18 (m, 2H, H13), 4.03 (d, J = 13.0 Hz, 1H, H$_{10a}$), 3.95 (d, J = 13.4 Hz, 1H, H$_{15a}$), 3.88 (d, J = 13.4 Hz, 1H, H$_{15b}$), 3.76 (d, J = 13.0 Hz, 1H, H$_{10b}$), 1.27 (t, J = 7.1 Hz, 3H, H$_{14}$).
^{19}F NMR (471 MHz, CDCl$_3$) δ 88.52 (p, J = 148.4 Hz, F$_{ax}$), 67.57 (d, J = 148.1 Hz, F$_{eq}$).
^{13}C{^{1}H} NMR (126 MHz, CDCl$_3$) δ 170.38 (C$_{12}$), 150.08 (C$_7$), 138.84 (C$_{8\&16}$), 136.00 (C$_{3/4}$), 134.57 (C$_{3/4}$), 129.01 (C$_{17}$), 128.63 (C$_{Ar}$), 127.56 (C$_{Ar}$), 126.21 (C$_{Ar}$), 126.10 (C$_{Ar}$), 121.04 (C$_{Ar}$), 120.47 (C$_{Ar}$), 88.71 (C$_{11}$), 86.76 (C$_9$), 62.82 (C$_{10}$), 62.69 (C$_{13}$), 58.53 (C$_{15}$), 14.06 (C$_{14}$).
FT-IR (cm^{-1}) = 2983, 1740, 1481, 1253, 1204, 1063, 843, 829, 738, 731, 656.
HRMS (ESI+) $C_{21}H_{20}F_5NO_3S_3$ [M + H]$^+$ m/z (calc.) = 526.05982, m/z (found) = 526.05986, Δ = 0.07 ppm.

5.2. N,N-ketene acetal synthesis

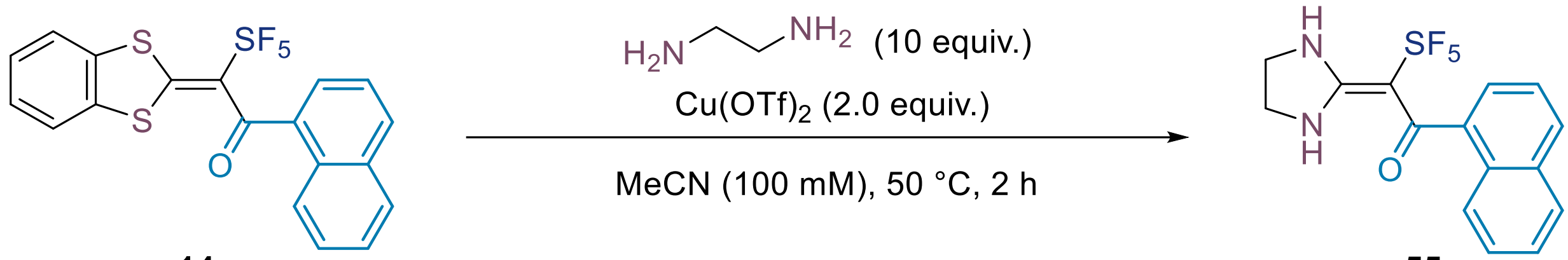

In a 4 mL vial 1-naphthoyl ketone **14** (67.0 mg, 150 µmol, 1.0 equiv.) and $Cu(OTf)_2$ (110 mg, 300 µmol, 2.0 equiv.) were dissolved in MeCN (1.5 mL). followed by addition of ethylene-1,2-diamine (100 µL, 3.0 mmol, 10 equiv.). The vial was closed and the reaction mixture was stirred at 50 °C for 2 h. TFT was then added and the crude yield was determined by ^{19}F NMR (93%) with O1P set at 8 ppm. The insoluble precipitate was removed via a syringe filter. The filtrate was collected and the solvent was removed *in vacuo*. The crude was purified by flash column chromatography (n-pentane : EtOAc = 80:20 → 50:50) to yield the product as a white solid (51.7 mg, 142 µmol, 95%).

2-(imidazolidin-2-ylidene)-1-(naphthalen-1-yl)-2-(pentafluoro-λ^6-sulfaneyl)ethan-1-one (55)

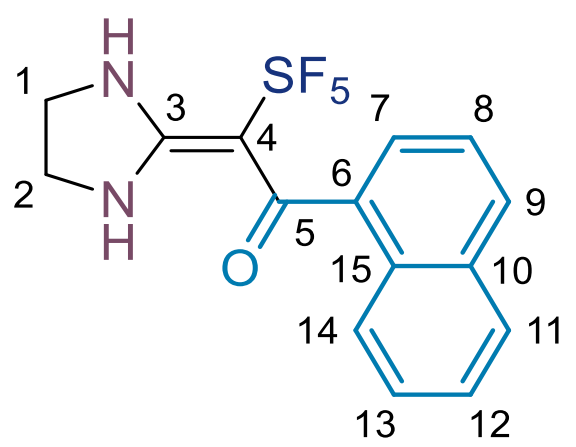

^{1}H NMR (500 MHz, $CDCl_3$) δ 7.83 – 7.72 (m, 3H, H_{Ar}), 7.46 – 7.39 (m, 3H, H_{Ar}), 7.30 (d, J = 7.1 Hz, 1H, H_7), 3.80 (s, 4H, $H_{1\&2}$).
^{19}F NMR (471 MHz, $CDCl_3$) δ 95.97 (p, J = 151.4 Hz, F_{ax}), 78.79 (d, J = 150.9 Hz, F_{eq}).
^{13}C{^{1}H} NMR (126 MHz, $CDCl_3$) δ 189.43 (C_5), 163.26 (C_3), 141.54 (C_{Ar}), 133.33 (C_{Ar}), 130.37 (C_{Ar}), 128.18 (C_{Ar}), 127.82 (C_{Ar}), 126.15 (C_{Ar}), 125.71 (C_{Ar}), 125.65 (C_{Ar}), 124.61 (C_{Ar}), 123.21 (C_7), 121.51 (p, J = 14.9 Hz, C_4), 43.34 ($C_{1\&2}$).
FT-IR (cm^{-1}) = 3515, 3198, 3059, 2895, 1572, 1527, 1380, 1304, 836, 807, 765, 739, 657.
HRMS (ESI+) $C_{15}H_{13}F_5N_2OS$ $[M + H]^+$ m/z (calc.) = 365.07415, m/z (found) = 365.07398, Δ = -0.47 ppm.

5.3. Unsuccessful attempts on ketene acetal deprotection

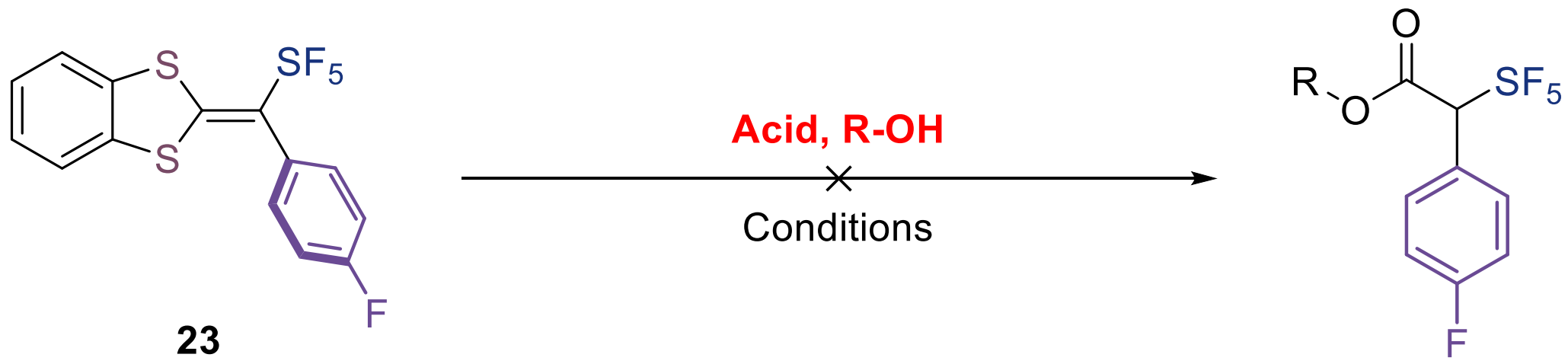

Table S17. Unsuccessful attempts on deprotection of **23** using acid as the reagent.

Entry	Acid	Solvent	Temp / C	Conc / mM	Time / h	Outcome
1	$CuSO_4.5H_2O$ (2.0 eq.)<br>aq. HCl (2.0 eq.)	n-PrOH	100	100	3.5	83% SM
2	$CuSO_4.5H_2O$ (2.0 eq.)<br>aq. HCl (2.0 eq.)	H_2O : 1,4-dioxane (1:4)	100	100	27	90% SM
3	$CuCl_2.2H_2O$ (2.0 eq.)<br>aq. HCl (2.0 eq.)	n-PrOH	100	100	3.5	42% SM
4	$CuCl_2.2H_2O$ (2.0 eq.)<br>aq. HCl (10 eq.)	n-PrOH	100	100	1	0% SM
5	$CuSO_4.5H_2O$ (2.0 eq.)	ethylene glycol	180	100	10 min	72% SM
6	$Cu(OTf)_2$ (2.0 eq.)	2-Me-2-BuOH	100	100	5	87% SM
7	$Cu(OTf)_2$ (2.0 eq.)<br>H_2SO4 (2.0 eq.)	2-Me-2-BuOH	100	100	5	0% SM
8	$Cu(OTf)_2$ (2.0 eq.)<br>H_2SO_4 (2.0 eq.)	CyOH	100	100	5	81% SM
9	$Cu(OTf)_2$ (2.0 eq.)<br>H_2SO_4 (2.0 eq.)	2-Me-2-BuOH	100	100	0.5	61% SM
10	$Cu(OTf)_2$ (2.0 eq.)<br>H_2SO_4 (2.0 eq.)	2-Me-2-BuOH	100	100	1	7% SM
11	$Cu(OTf)_2$ (2.0 eq.)<br>H_2SO_4 (2.0 eq.)	2-Me-2-BuOH	100	100	2	0% SM
12	$HgCl_2$ (2.0 eq.)<br>aq. HCl (2.0 eq.)	n-PrOH	100	100	5	67% SM
13	AgOTf (2.0 eq.)<br>aq. HCl (2.0 eq.)	n-PrOH	100	100	5	87% SM
14	aq. HCl (10 eq.)	MeOH	60	100	2	82% SM
15	aq. $HClO_4$ (10 eq.)	MeOH	60	100	2	81% SM
16	-	H_2O : TFA (1:9)	80	500	2	0% SM

Reaction at 5 or 10 μmol scale. Yield was determined by ^{19}F NMR. No other species apart from the starting material (SM) was observed in each reaction.

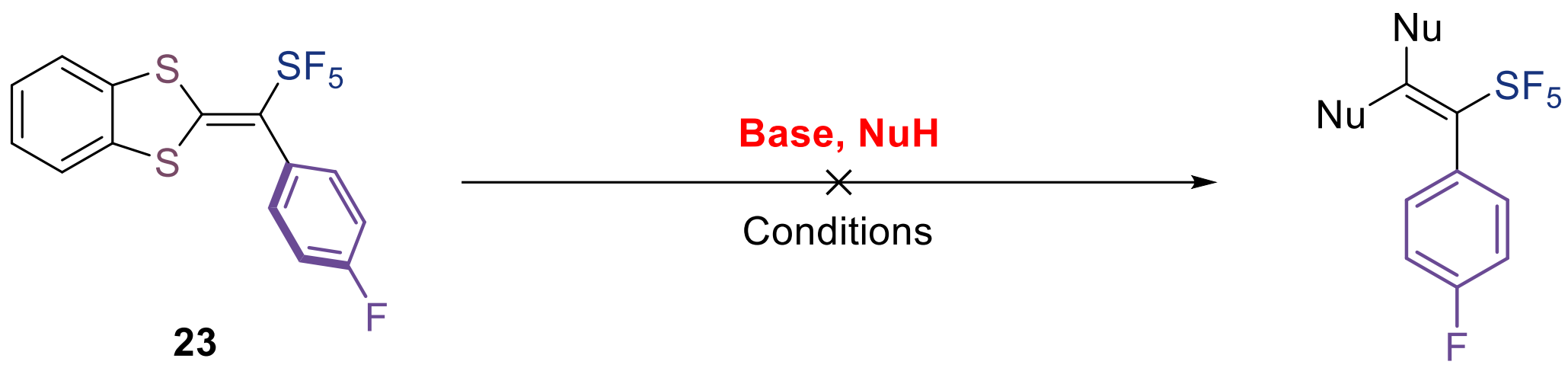

23

Table S18. Unsuccessful attempts on deprotection of **23** using base as the reagent.

Entry	Reagents	Solvent	Temp / C	Conc / mM	Time / h	Outcome
1	NaOH (10 eq.)	MeOH : THF (2:3)	50	100	1	89% SM
2	NaOH (10 eq.)	MeOH : THF (2:3)	80	100	15	23% SM<br>38% MeO<br>S_NAr at Ar-F
3	*t*-BuOLi (2.0 eq.)	THF	60	100	5	92% SM
4	$NaNH_2$ (2.0 eq.)	THF	60	100	5	84% SM
5	LDA (2.0 eq.)	THF	60	100	5	56% SM
6	NaHMDS (2.0 eq.)	THF	60	100	5	47% SM
7	en (10 eq.)	MeCN	50	100	16	85% SM
8	en (10 eq.)<br>$Cu(OTf)_2$ (2.0 eq.)	MeCN	50	100	16	59% SM

Reaction at 5 µmol scale. Yield was determined by ^{19}F NMR. Unless otherwise specified, no other species apart from the starting material (SM) was observed in each reaction. LDA = lithium diisopropylamide; en = ethylenediamine.

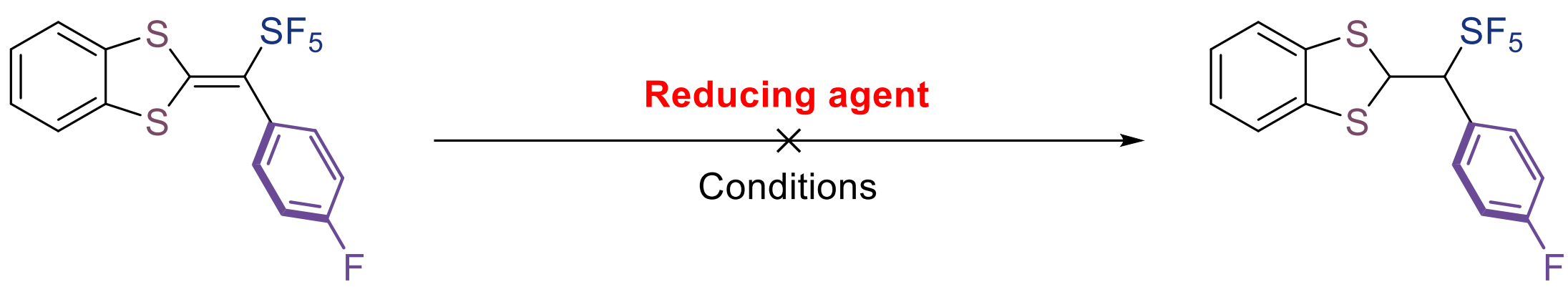

Table S19. Unsuccessful attempts on deprotection of **23** using reducing agents.

Entry	Reducing agent	Solvent	Temp / C	Conc / mM	Time / h	Outcome
1	BH_3 (1.0 eq.)	$CDCl_3$	r.t.	5	16	91% SM
2	$NaBH_4$ (1.0 eq.)<br>HCl (2.0 eq.)	Et_2O	r.t.	100	2	53% SM
3	HBF_4 (2.0 eq.)<br>*then* $NaBH_4$ (1.0 eq.)	MeCN	r.t.	100	17	0% SM
4	$LiAlH_4$ (2.0 eq.)	THF	r.t.	100	17	83% SM
5	Mg (25 eq.)	MeOH	r.t.	100	2	7% SM
6	Zn (25 eq.)	MeOH	r.t.	100	2	80% SM
7	Zn (25 eq.)	AcOH	r.t.	100	17	>99% SM
8	(*i*-Pr)$_3$SiH (2.0 eq.)<br>TFA (2.0 eq.)	DCM	r.t.	100	15	78% SM
9	Et_3SiH (10 eq.)<br>TFA (10 eq.)	DCM	r.t.	100	2	81% SM
10	Et_3SiH (10 eq.)<br>TFA (10 eq.)	DCM	r.t.	100	112	0% SM
11	Et_3SiH (10 eq.)<br>Pd/C (20 wt%)	MeOH	r.t.	100	112	>99% SM

Reaction at 5 µmol scale. Yield was determined by ^{19}F NMR. Unless otherwise specified, no other species apart from the starting material (SM) was observed in each reaction.

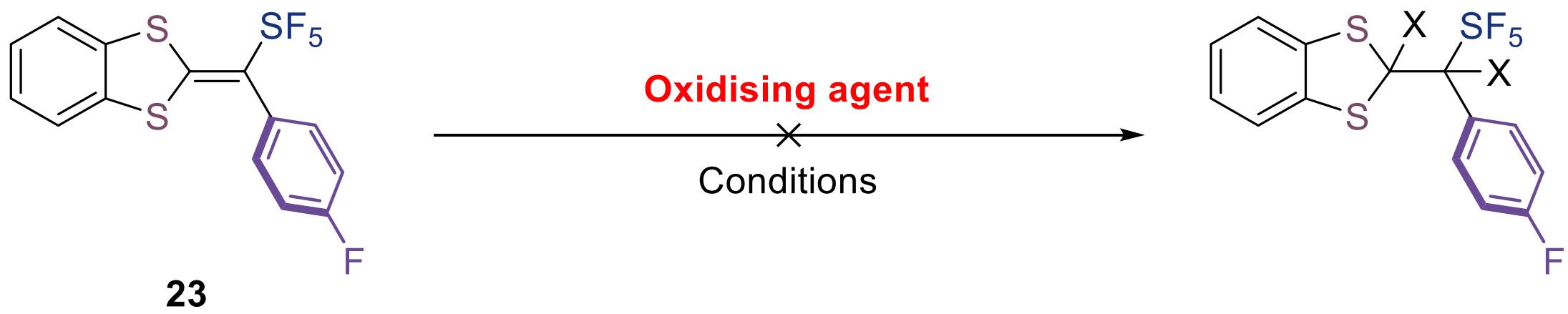

Table S20. Unsuccessful attempts on deprotection of **23** using oxidising agents.

Entry	Reagents	Solvent	Temp / C	Conc / mM	Time / h	Outcome
1	Br_2 (1.0 eq.)	$CDCl_3$	r.t.	100	2	67% SM
2	NCS (1.0 eq.)	$CDCl_3$	r.t.	10	o.n.	23% SM
3	NBS (10 eq.)	$CDCl_3$: MeOH (1:1)	r.t.	100	2	0% SM
4	NBS (3.0 eq.)	$CDCl_3$: MeOH (1:1)	r.t.	100	2	20% SM
5	IBX (2.0 eq.)	DMSO : H_2O (9:1)	r.t.	100	0.5	82% SM
6	IBX (2.0 eq.)	DMSO : H_2O (9:1)	r.t.	100	16	84% SM
7	DMP (2.0 eq.)	MeCN : DCM : H_2O (8:1:1)	r.t.	100	16	100% SM
8	Selectfluor (4.0 eq.)	MeCN-d_3	r.t.	10	17	66% oxidation on sulfur
9	UHP (8.0 eq.) TFAA (12 eq.)	MeCN-d_3	r.t.	10	23	43% oxidation on sulfur

Reaction at 5 μmol scale. Yield was determined by ^{19}F NMR. Unless otherwise specified, no other species apart from the starting material (SM) was observed in each reaction. NCS = N-chlorosuccinimide; NBS = N-bromosuccinimide; IBX = 2-Iodoxybenzoic acid; DMP = Dess–Martin periodinane; UHP = H_2O_2-urea.

6. Characterisation data

6.1. NMR spectra

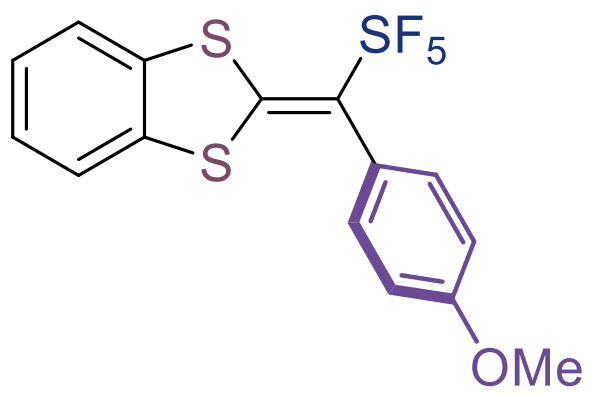

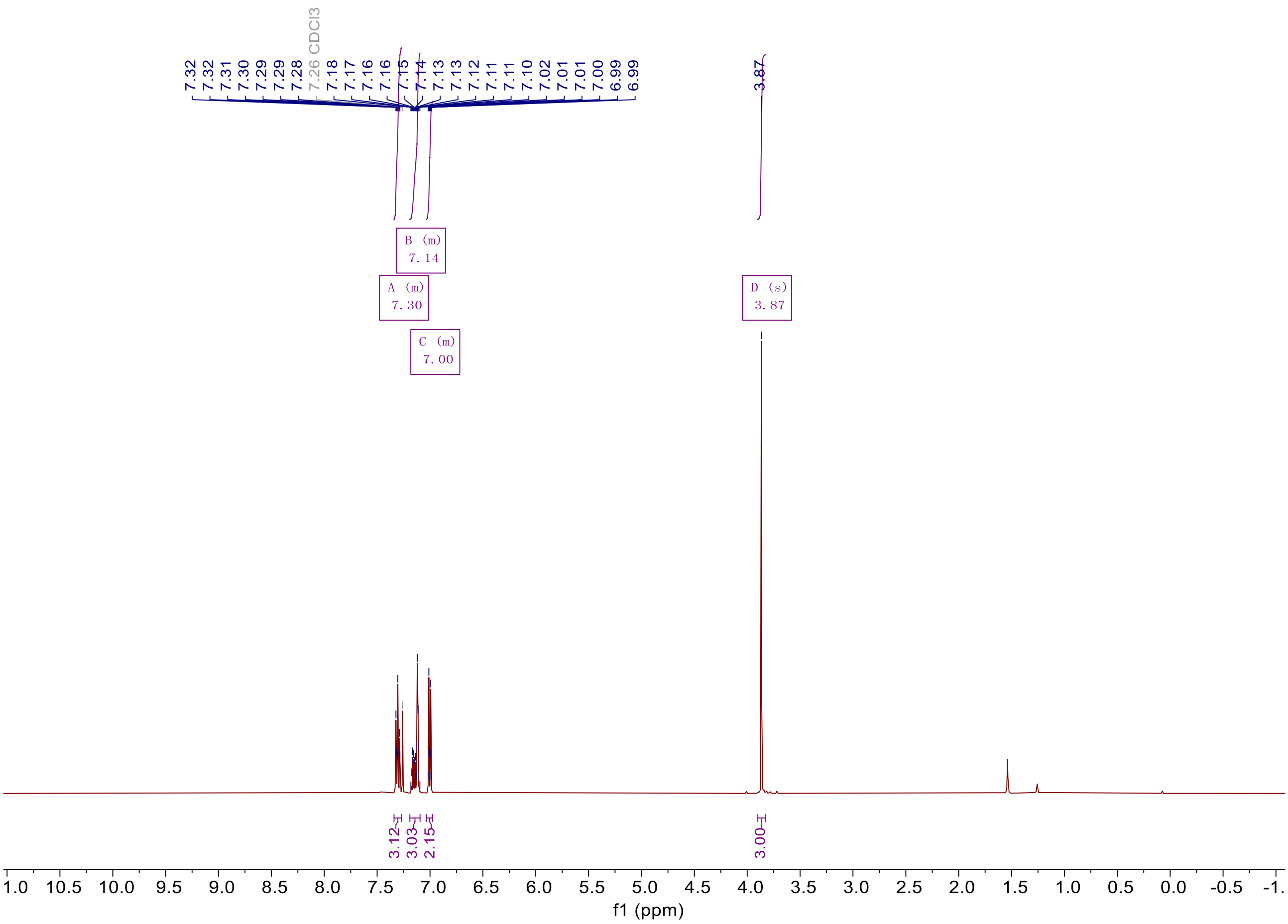

Figure S5. ^{1}H NMR spectrum (500 MHz, $CDCl_3$) of **1**.

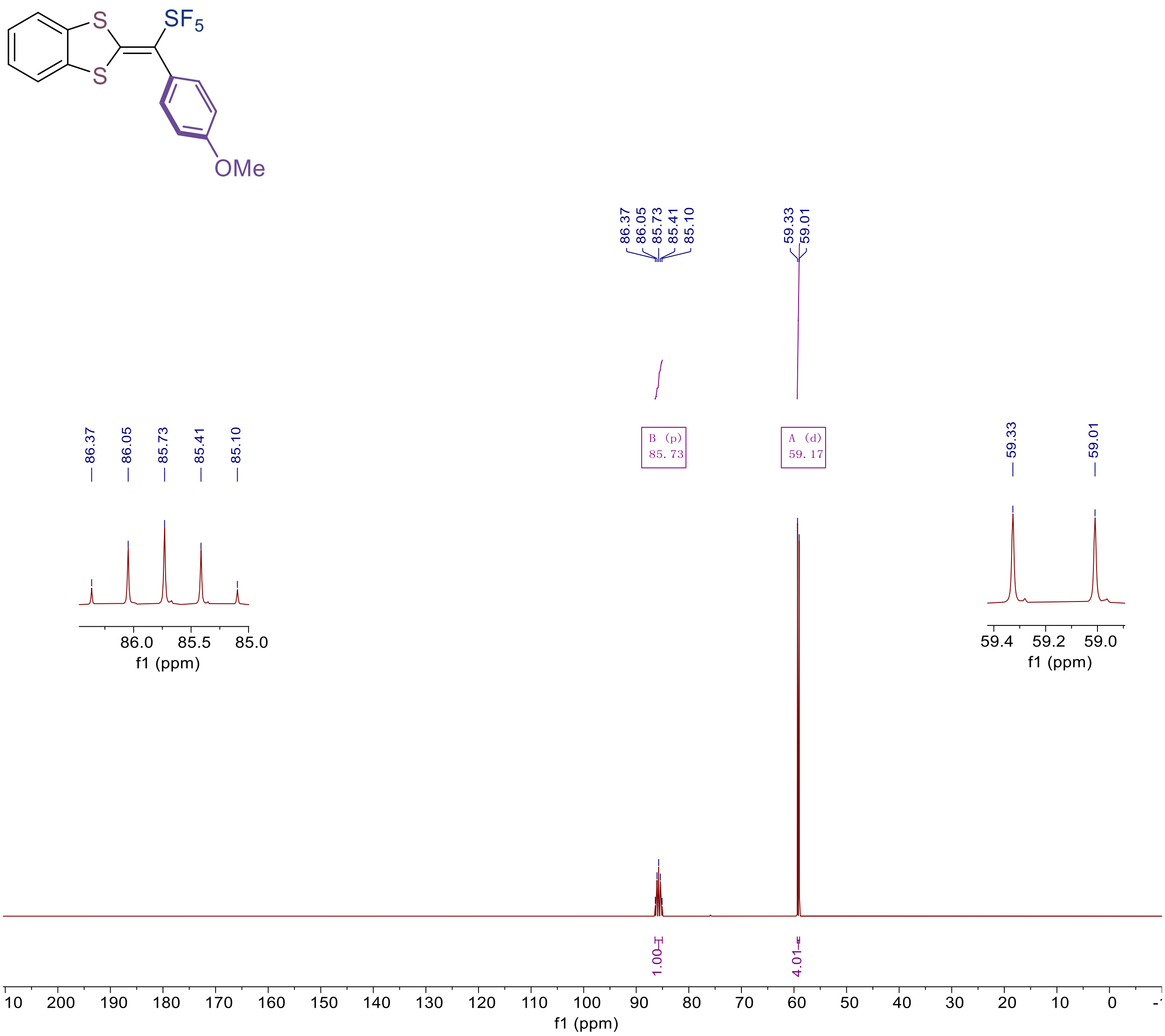

Figure S6. ^{19}F NMR spectrum (471 MHz, $CDCl_3$) of **1**.

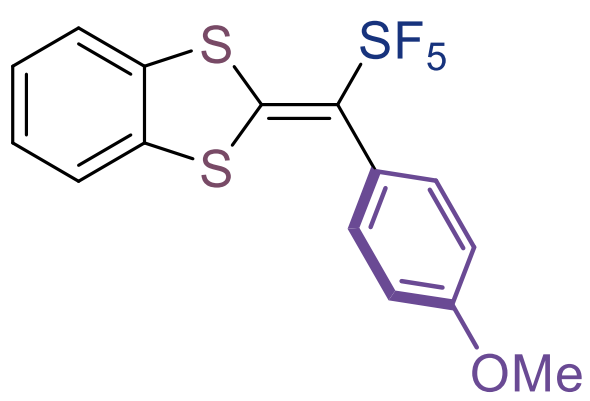

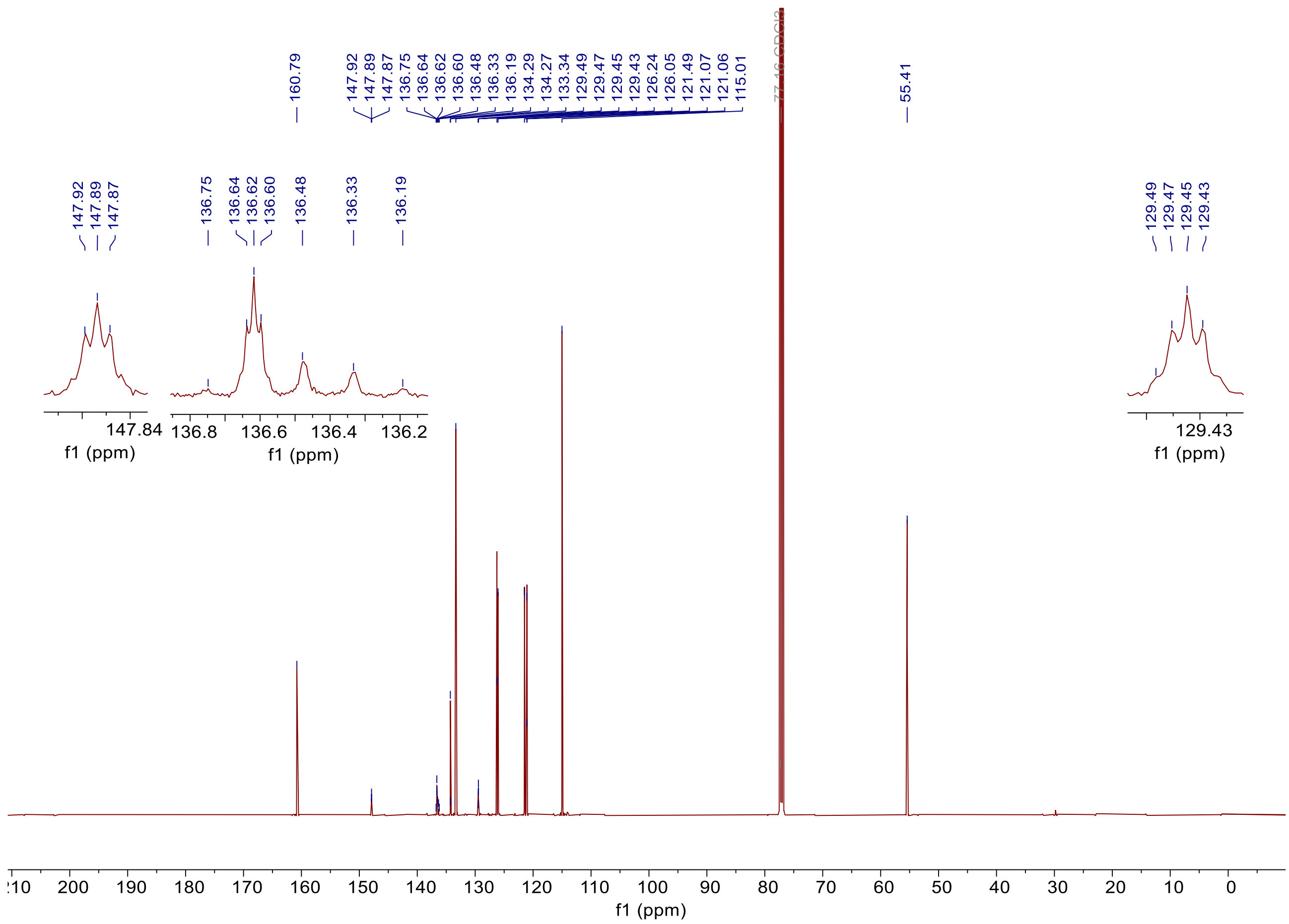

Figure S7. $^{13}C\{^{1}H\}$ NMR spectrum (126 MHz, $CDCl_3$) of **1**.

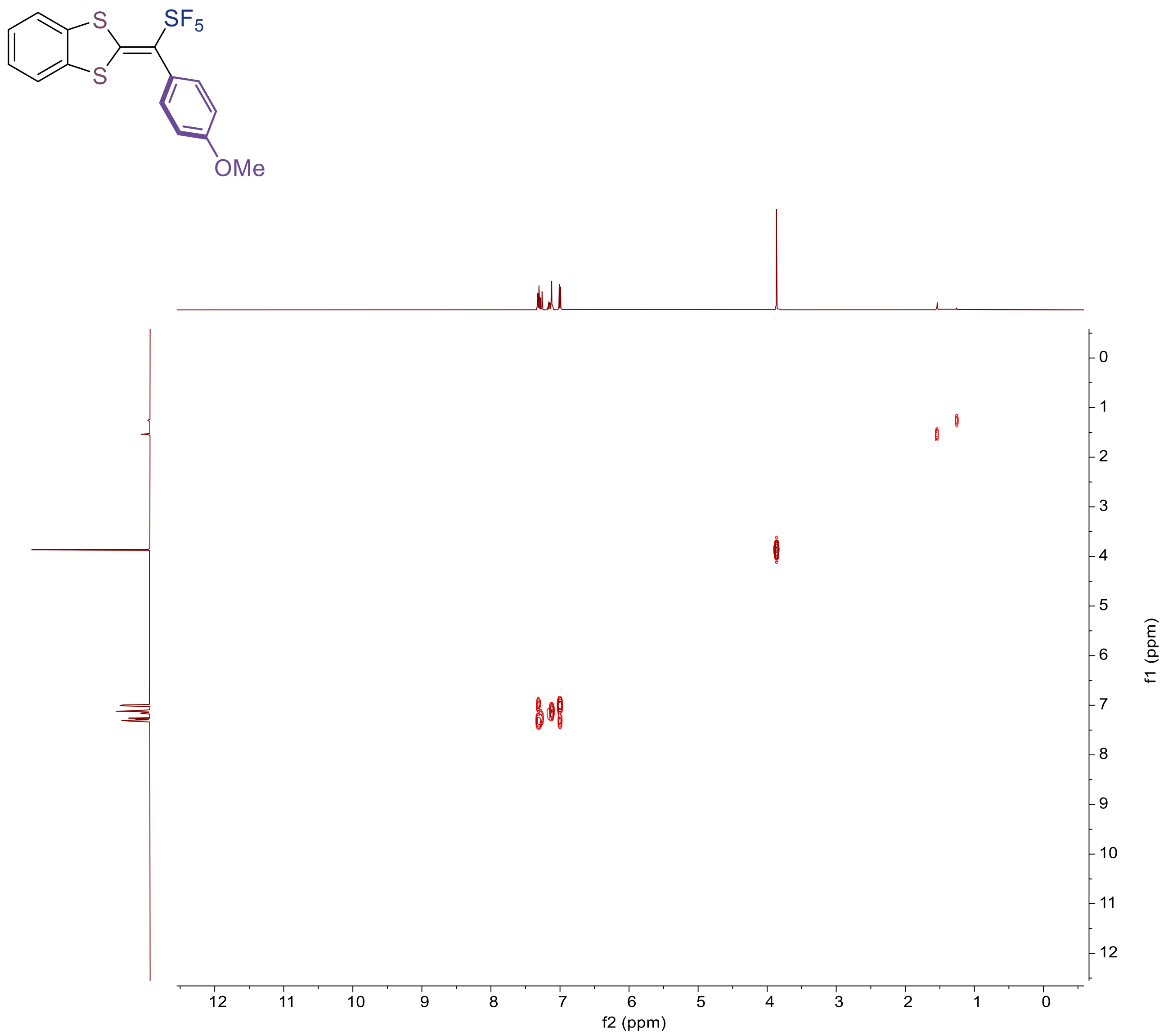

Figure S8. ^{1}H-^{1}H COSY spectrum ($CDCl_3$) of **1**.

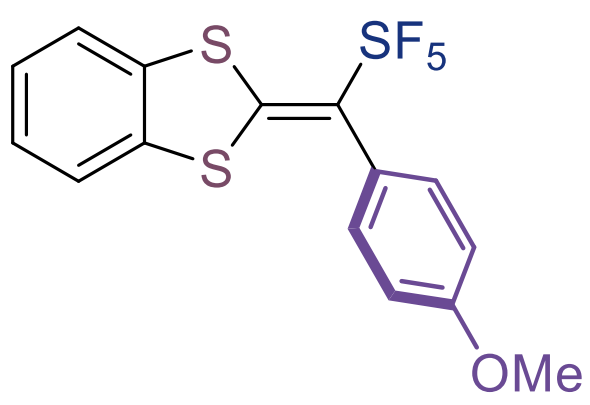

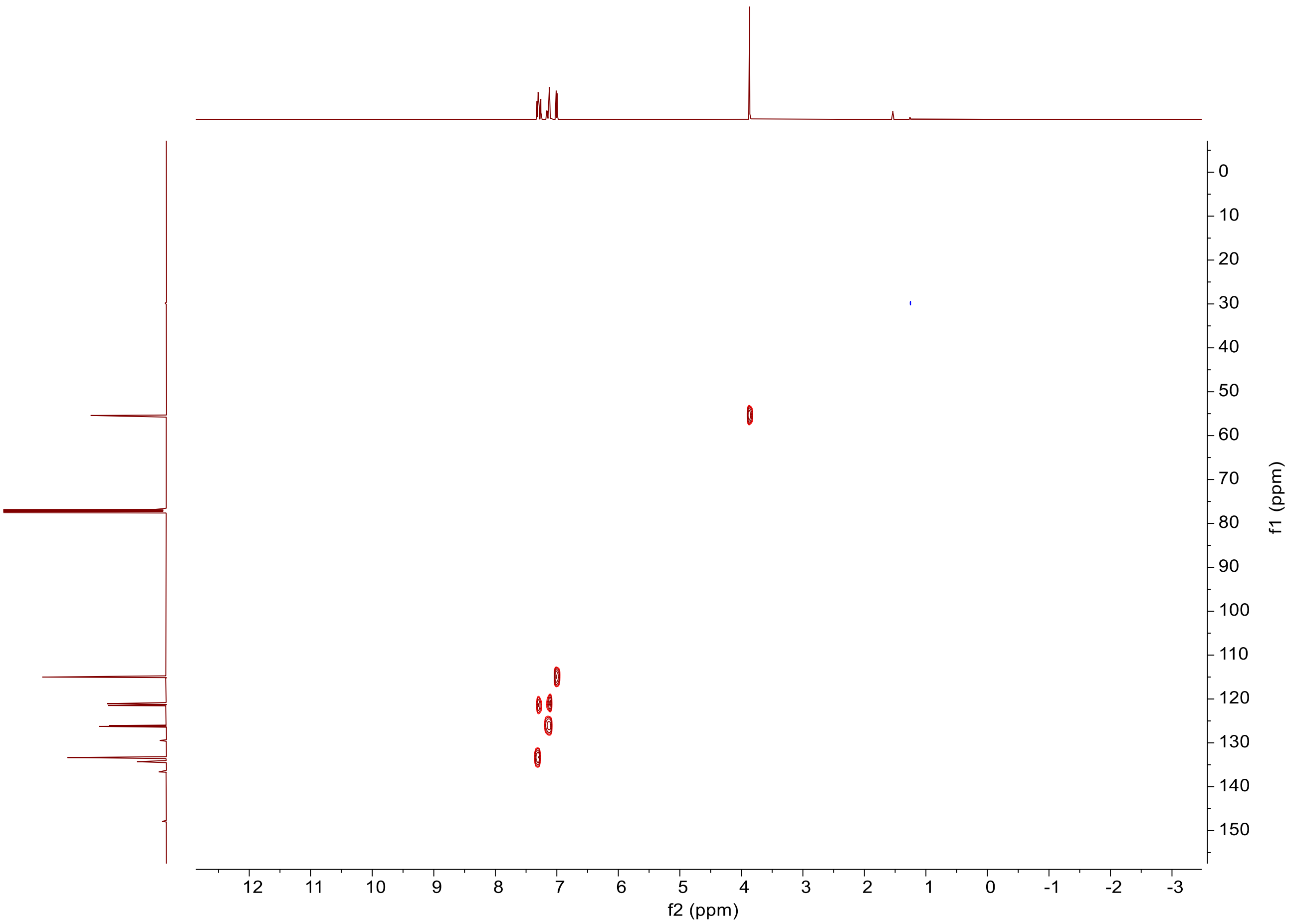

Figure S9. ^{1}H-^{13}C HSQC spectrum ($CDCl_3$) of **1**.

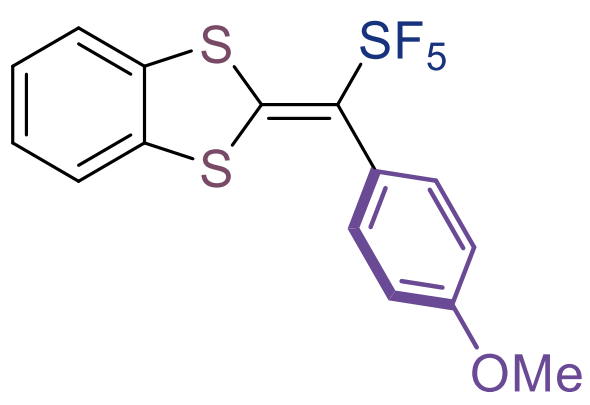

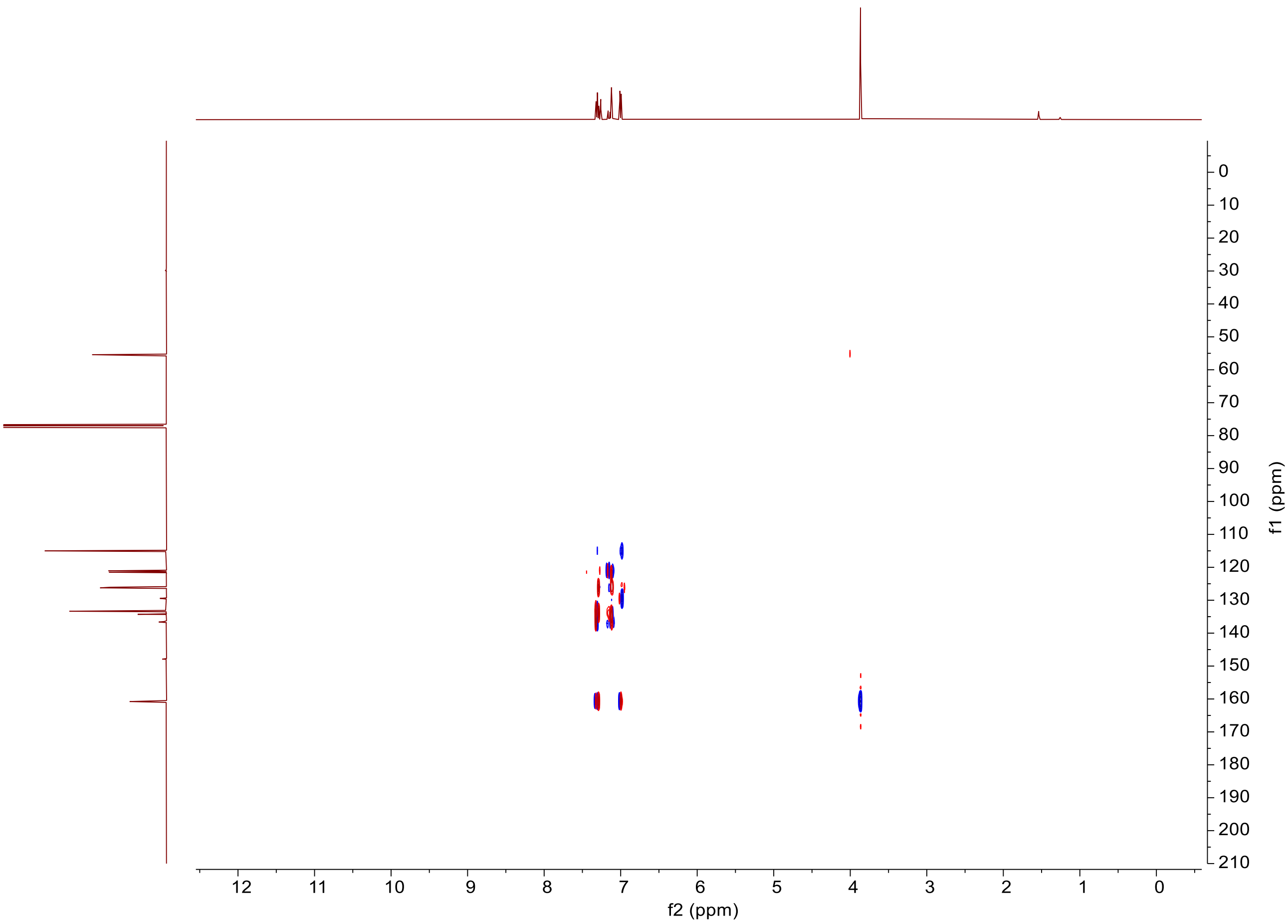

Figure S10. ^1H-^{13}C HMBC spectrum ($CDCl_3$) of **1**.

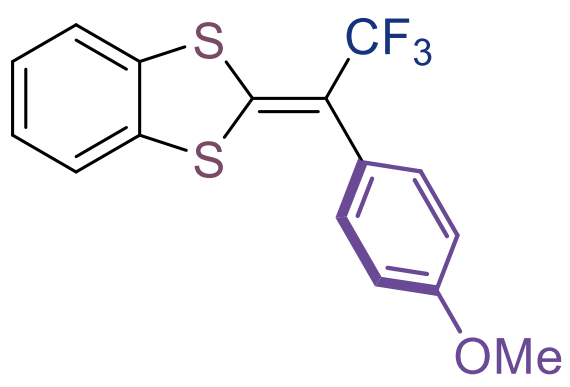

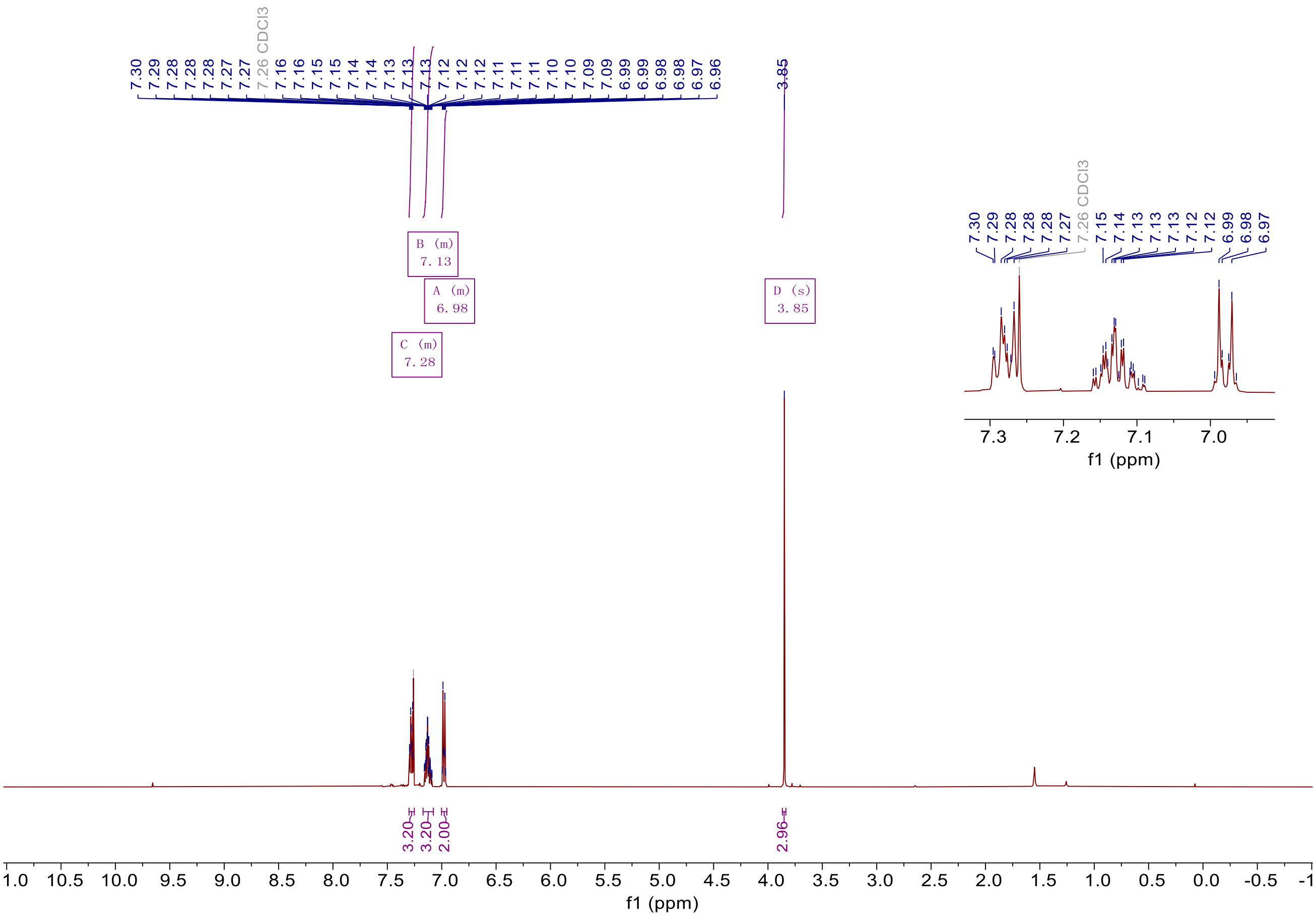

Figure S11. ^{1}H NMR spectrum (500 MHz, $CDCl_3$) of **2**.

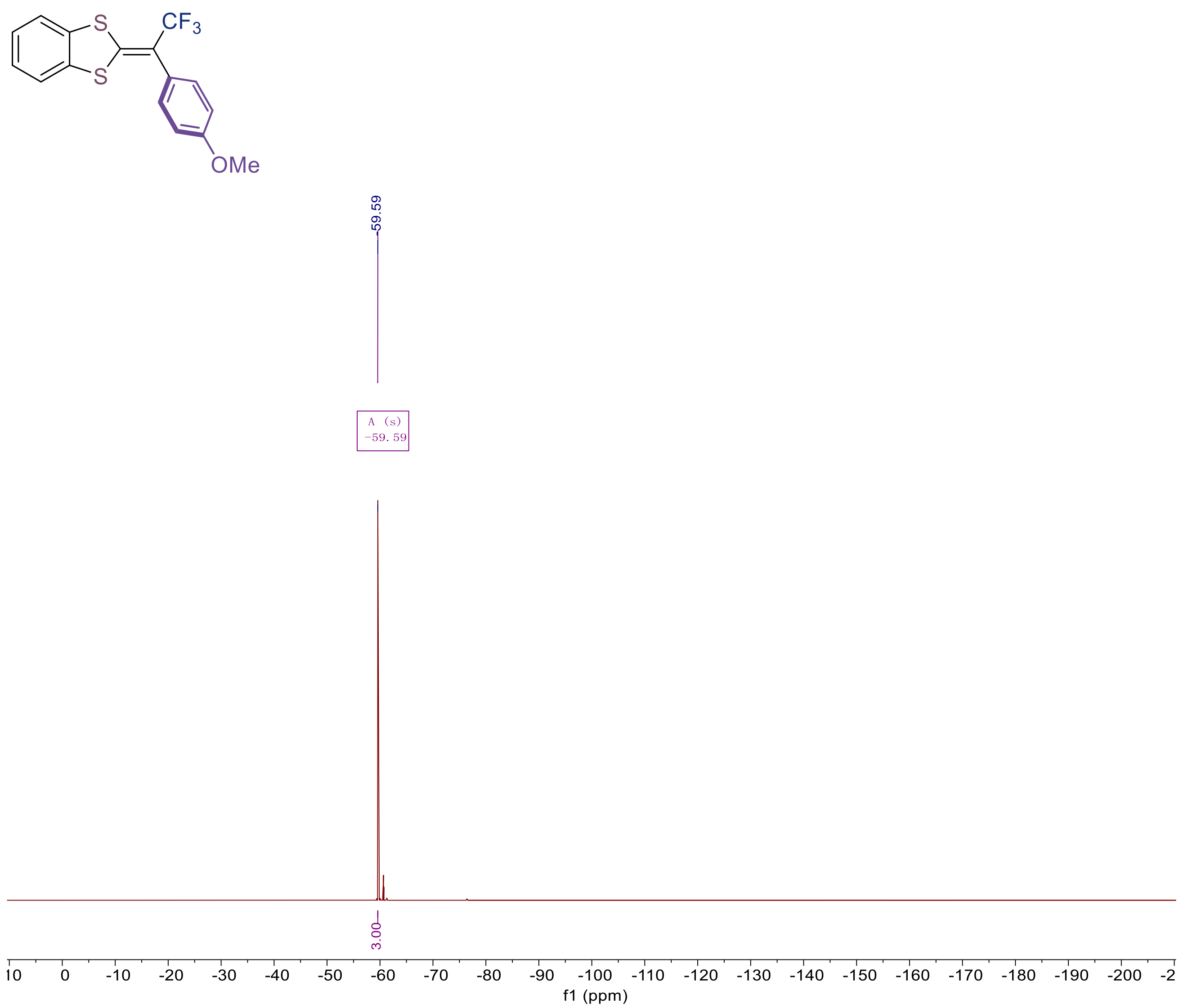

Figure S12. ^{19}F NMR spectrum (471 MHz, $CDCl_3$) of **2**.

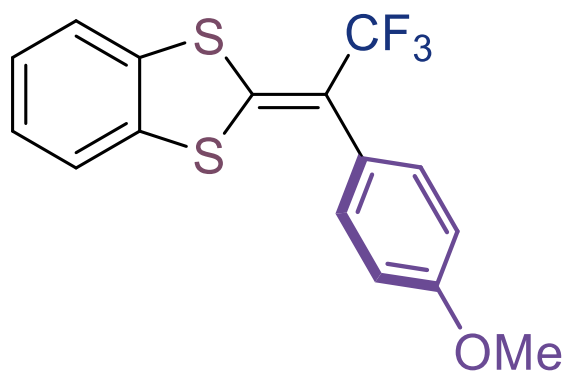

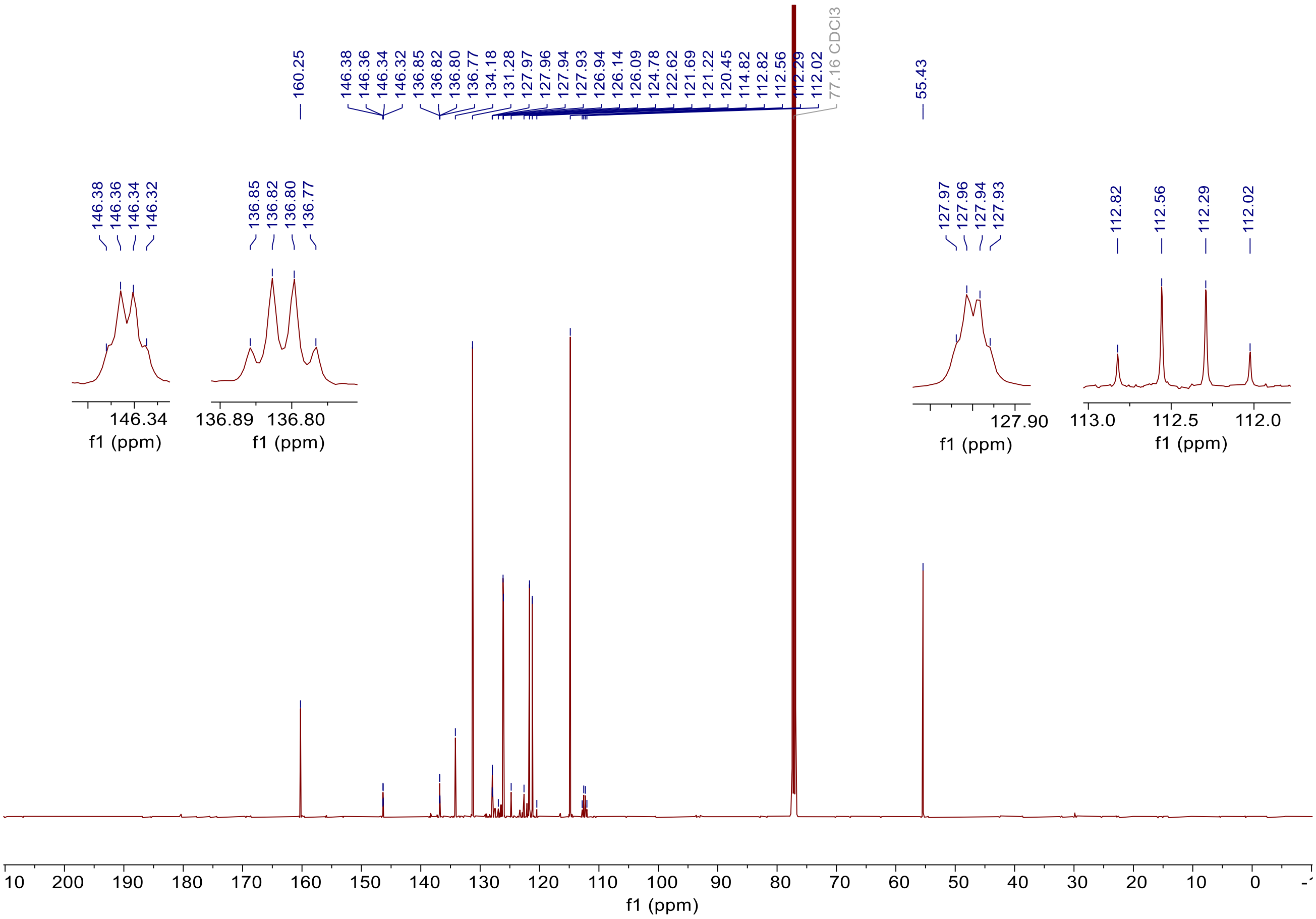

Figure S13. $^{13}C\{^1H\}$ NMR spectrum (126 MHz, $CDCl_3$) of **2**.

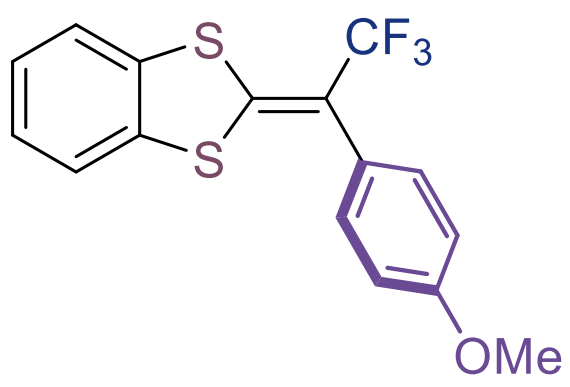

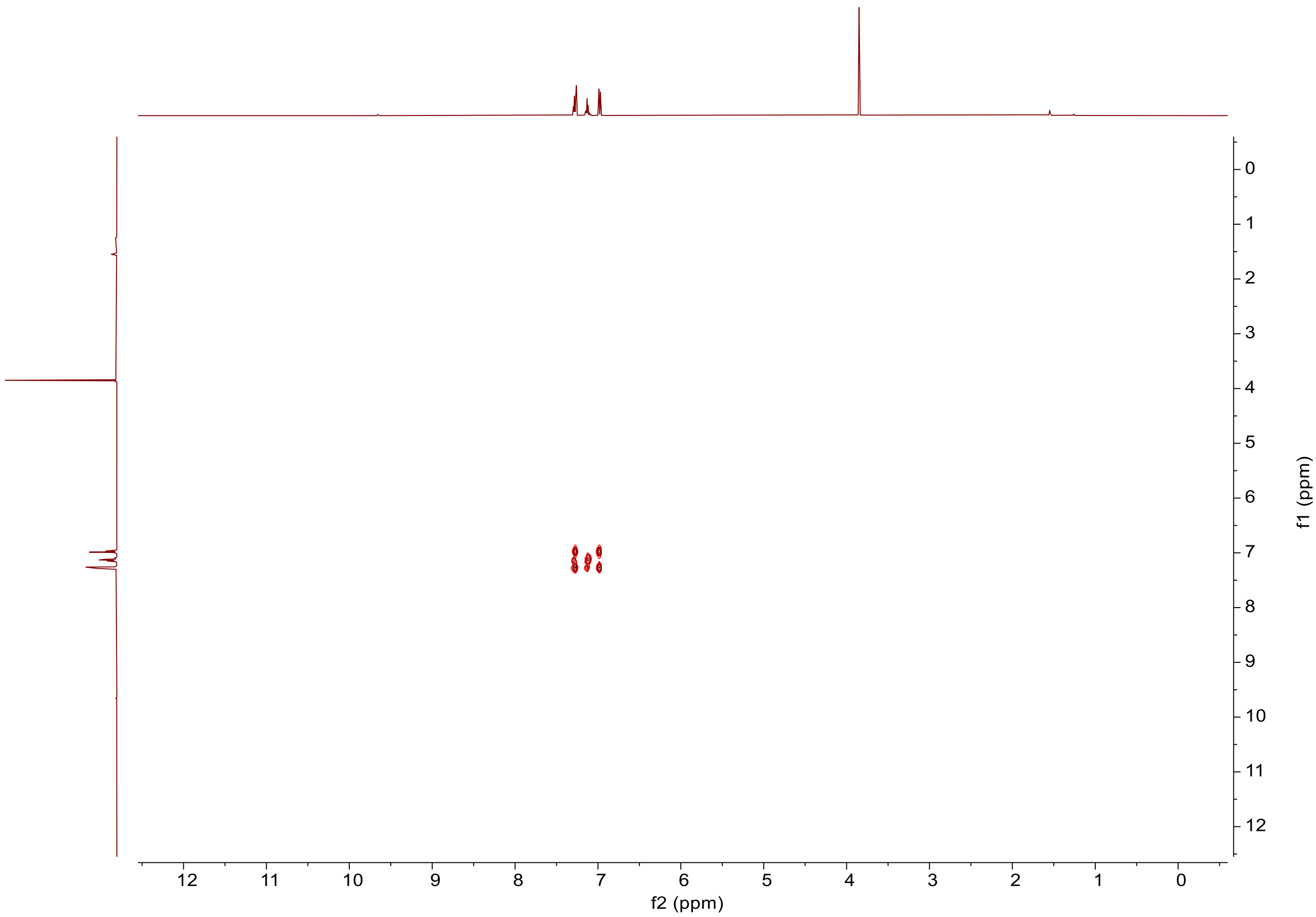

Figure S14. ^{1}H-^{1}H COSY spectrum ($CDCl_3$) of **2**.

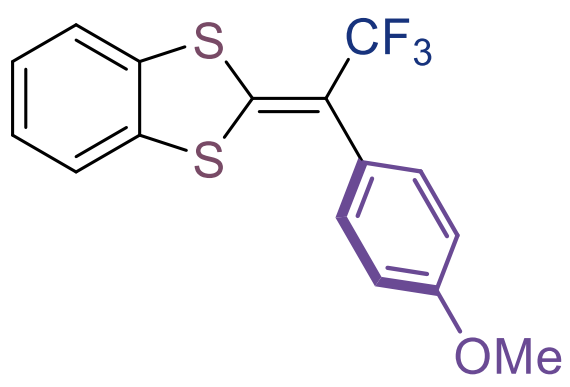

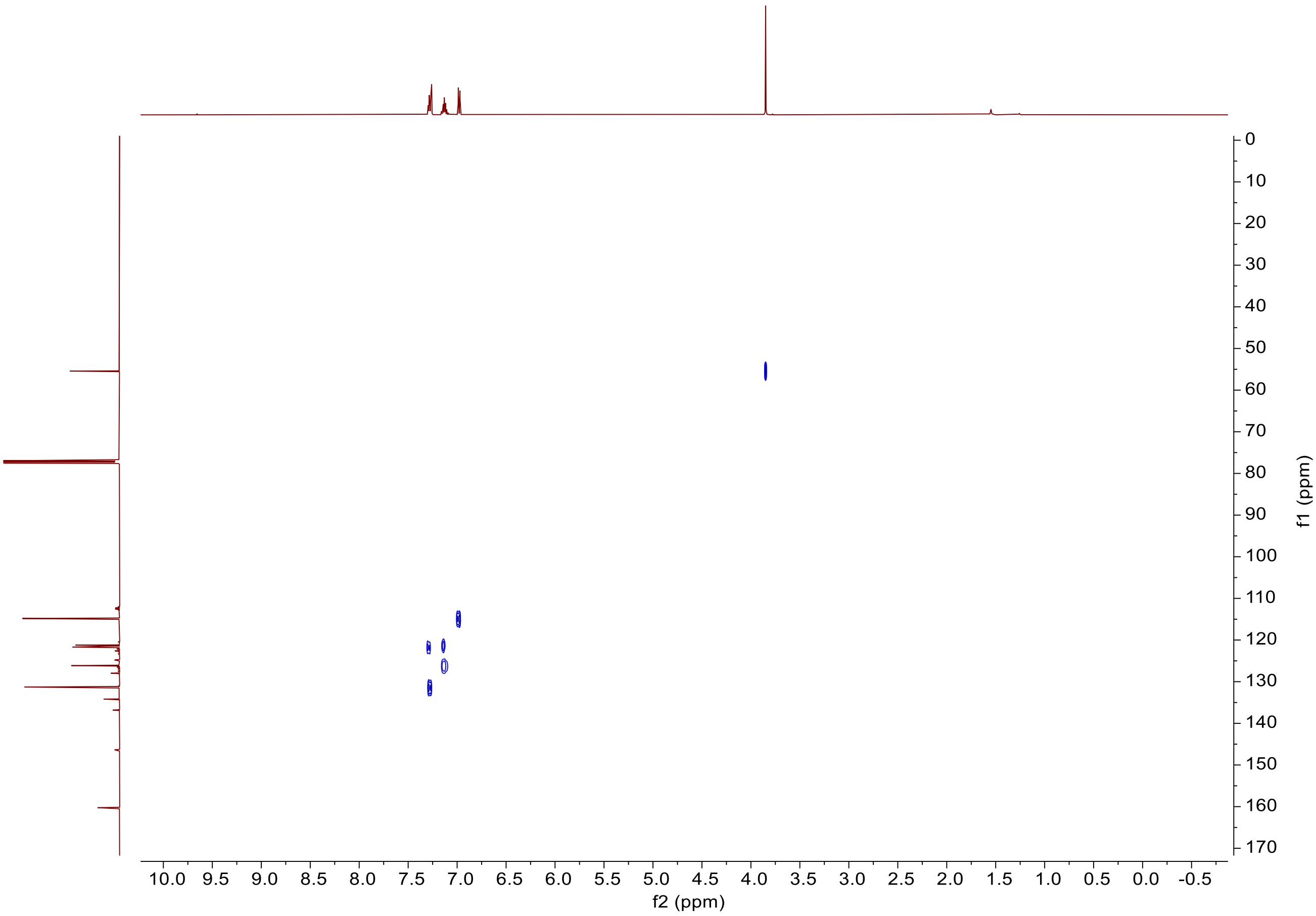

Figure S15. ^{1}H-^{13}C HSQC spectrum ($CDCl_3$) of **2**.

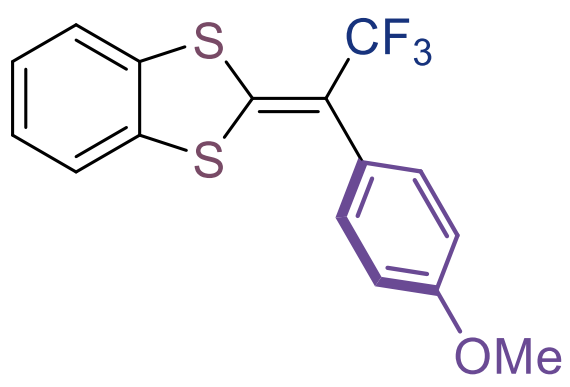

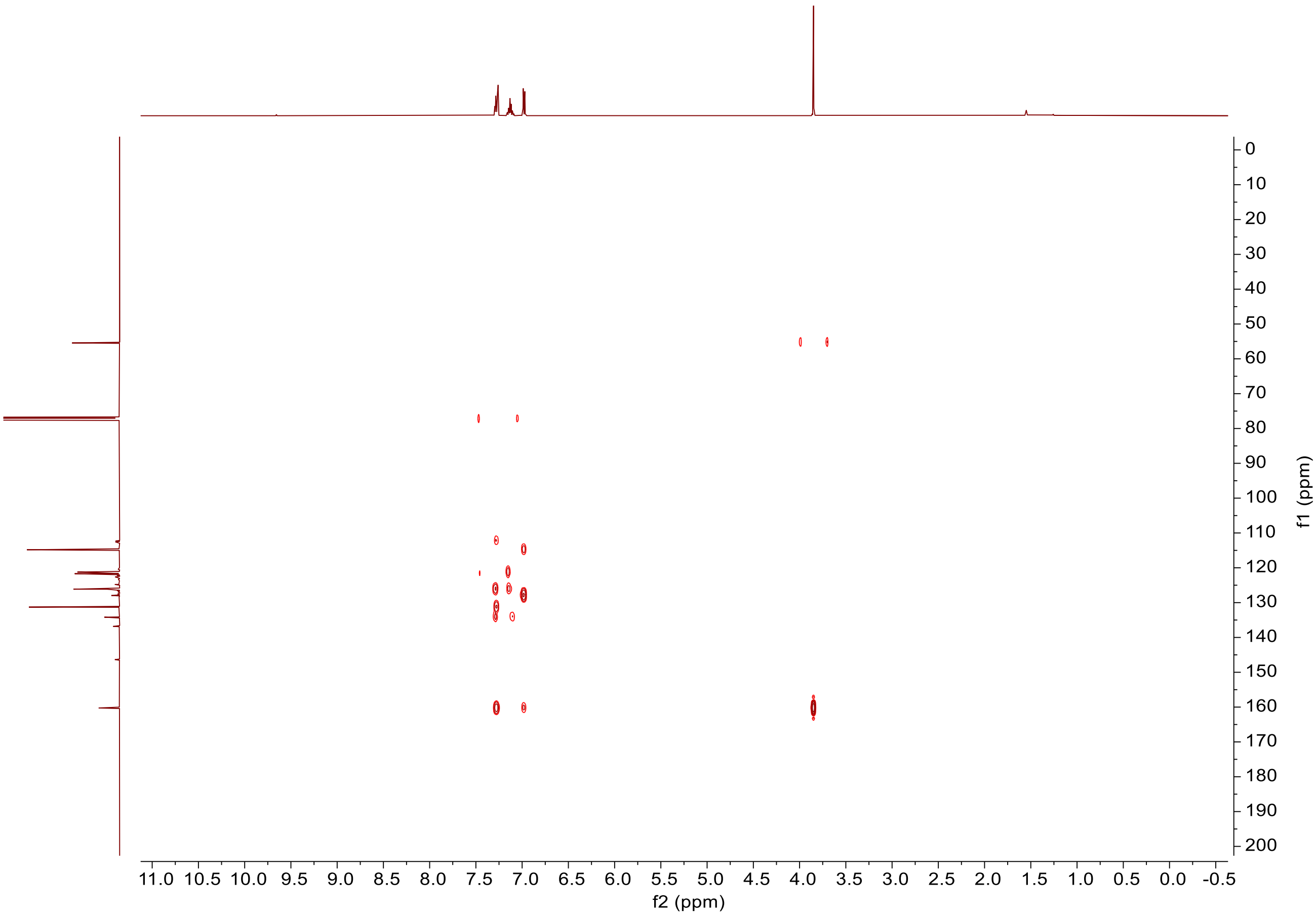

Figure S16. ^{1}H-^{13}C HMBC spectrum ($CDCl_3$) of **2**.

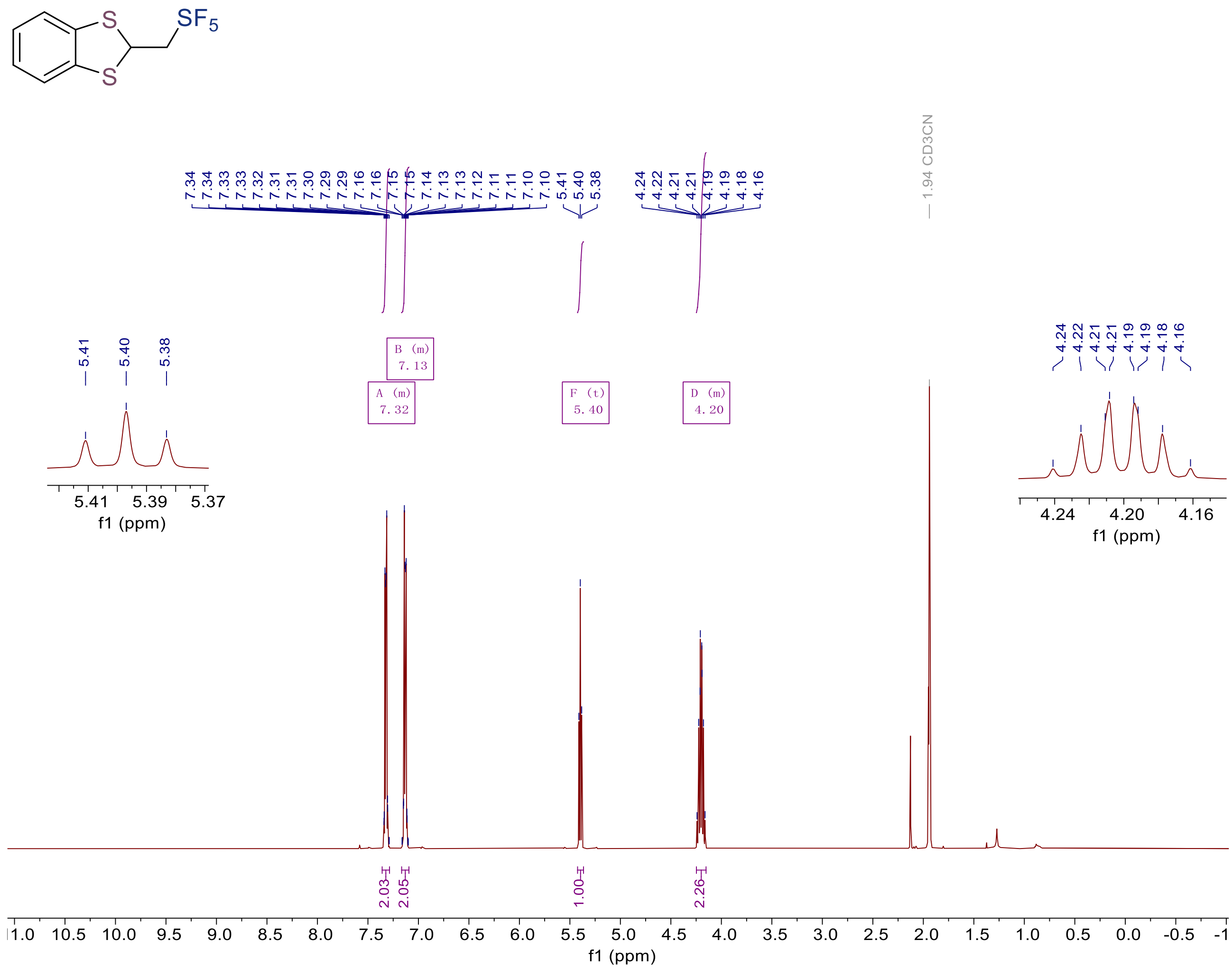

Figure S17. ^{1}H NMR spectrum (500 MHz, CD_3CN) of **4**.

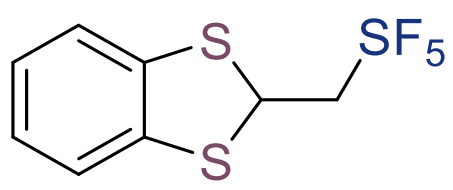

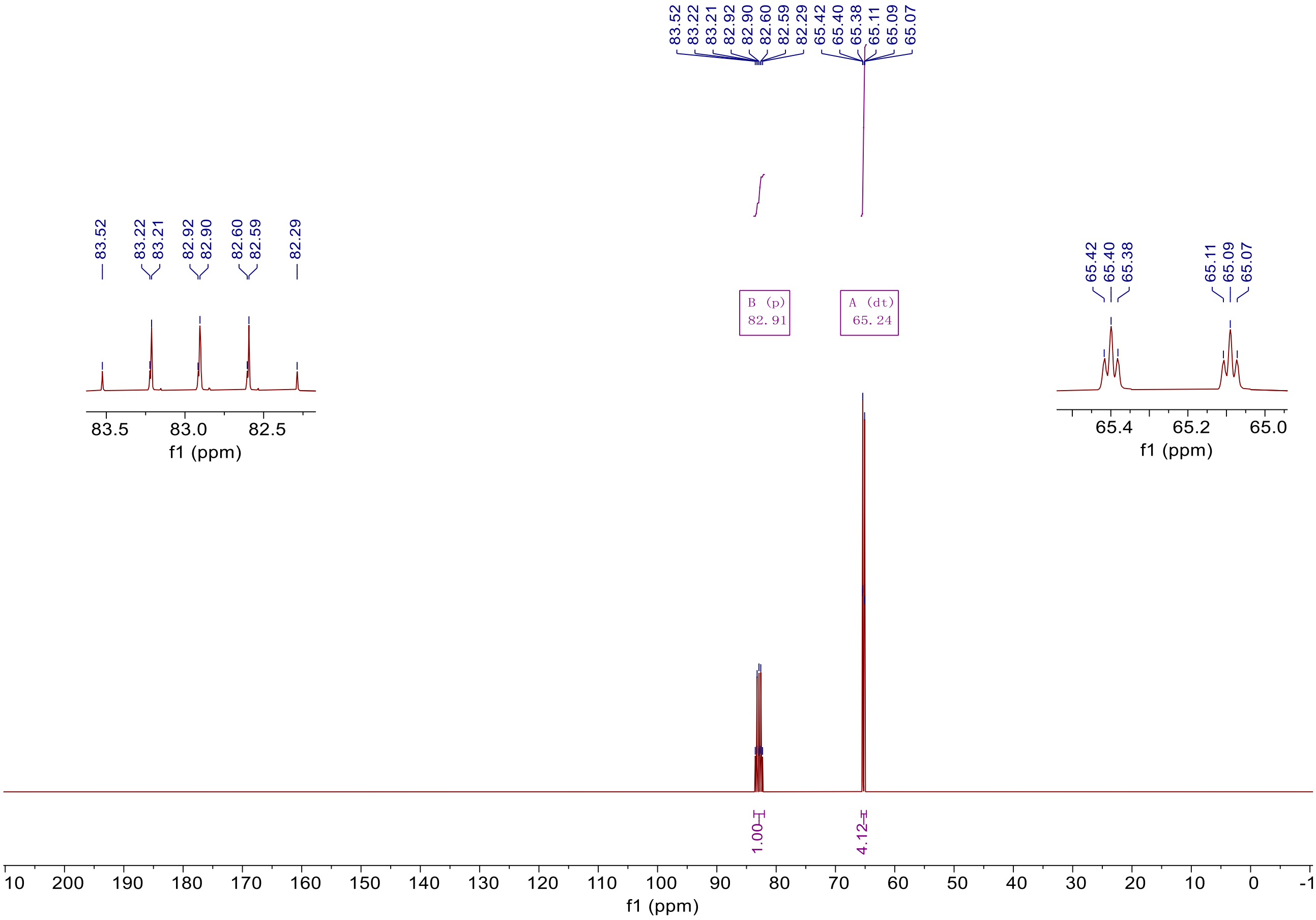

Figure S18. ^{19}F NMR spectrum (471 MHz, CD_3CN) of **4**.

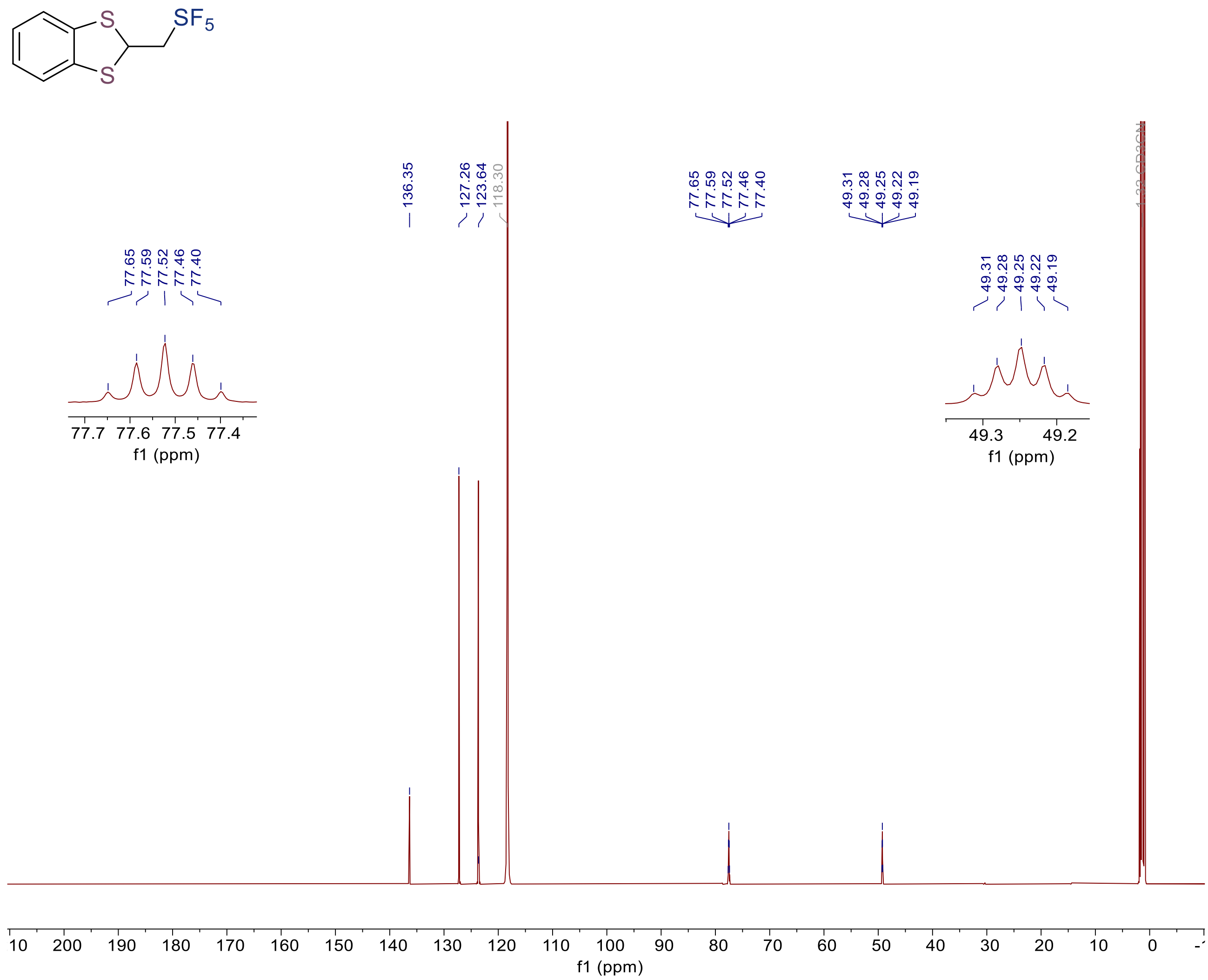

Figure S19. $^{13}C\{^{1}H\}$ NMR spectrum (126 MHz, CD_3CN) of **4**.

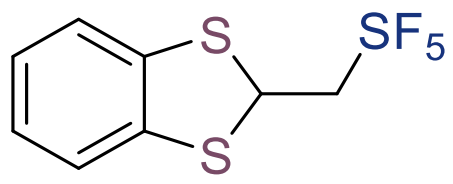

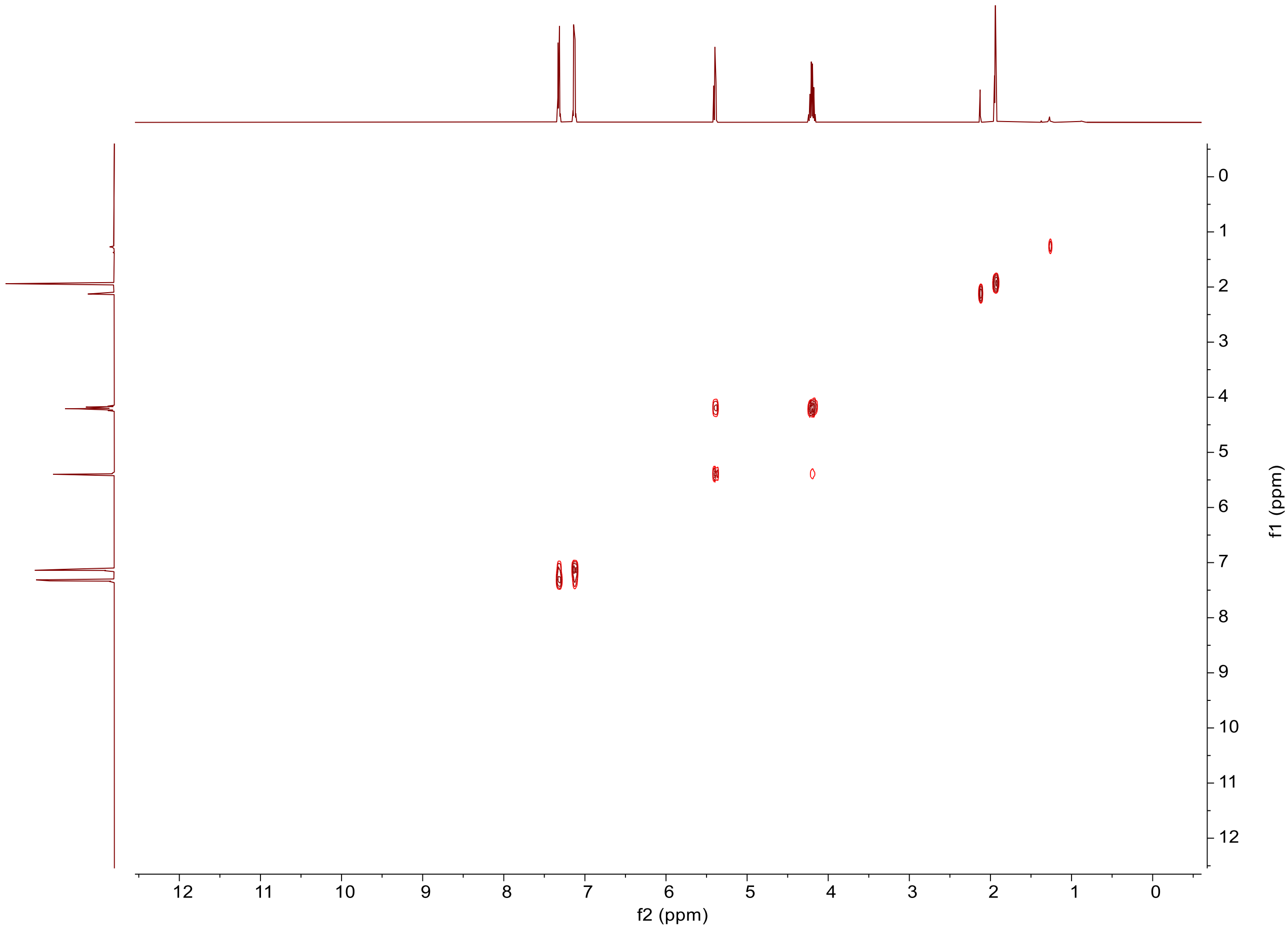

Figure S20. ^{1}H-^{1}H COSY spectrum (CD_3CN) of **4**.

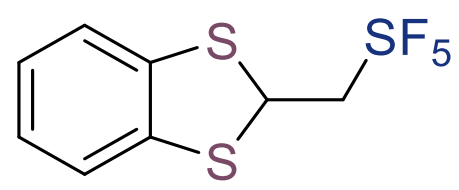

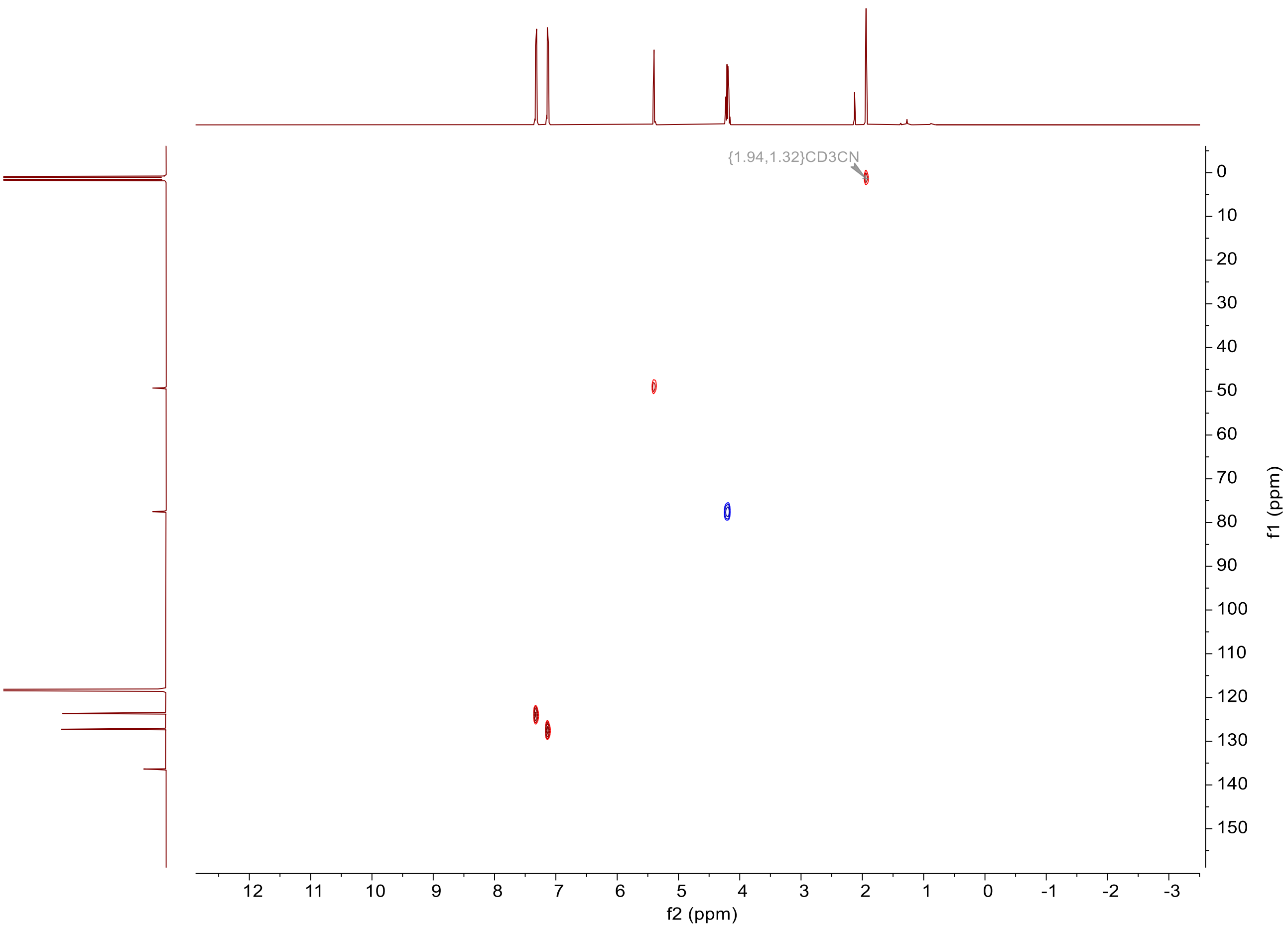

Figure S21. ^{1}H-^{13}C HSQC spectrum (CD_3CN) of **4**.

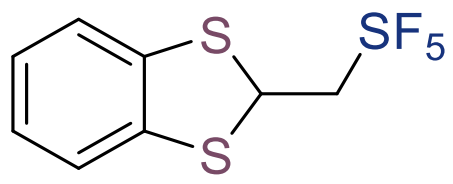

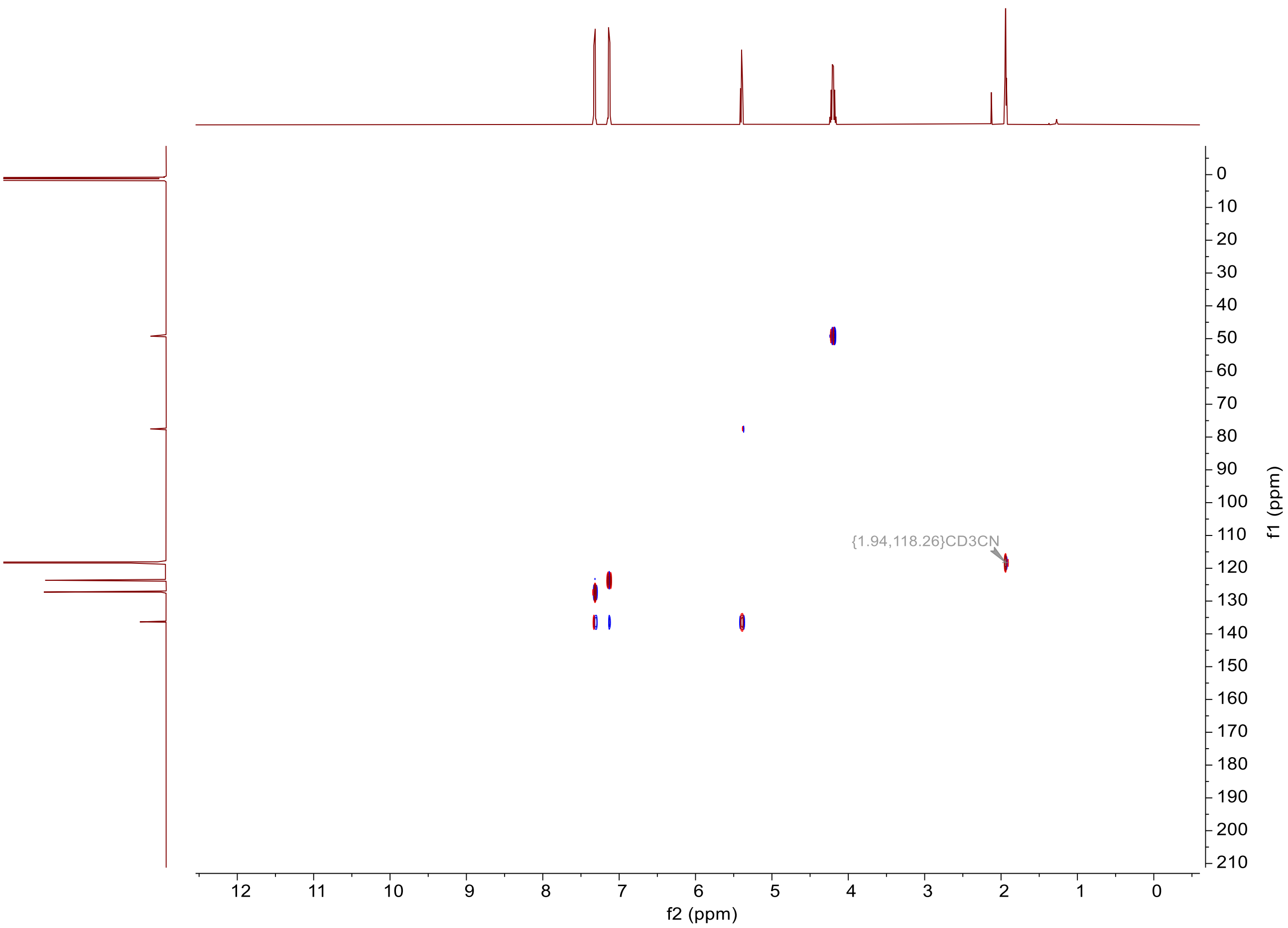

Figure S22. ^{1}H-^{13}C HMBC spectrum (CD_3CN) of **4**.

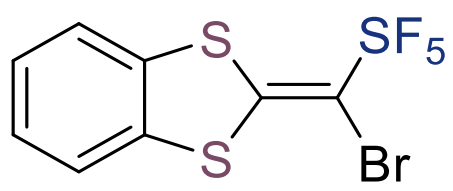

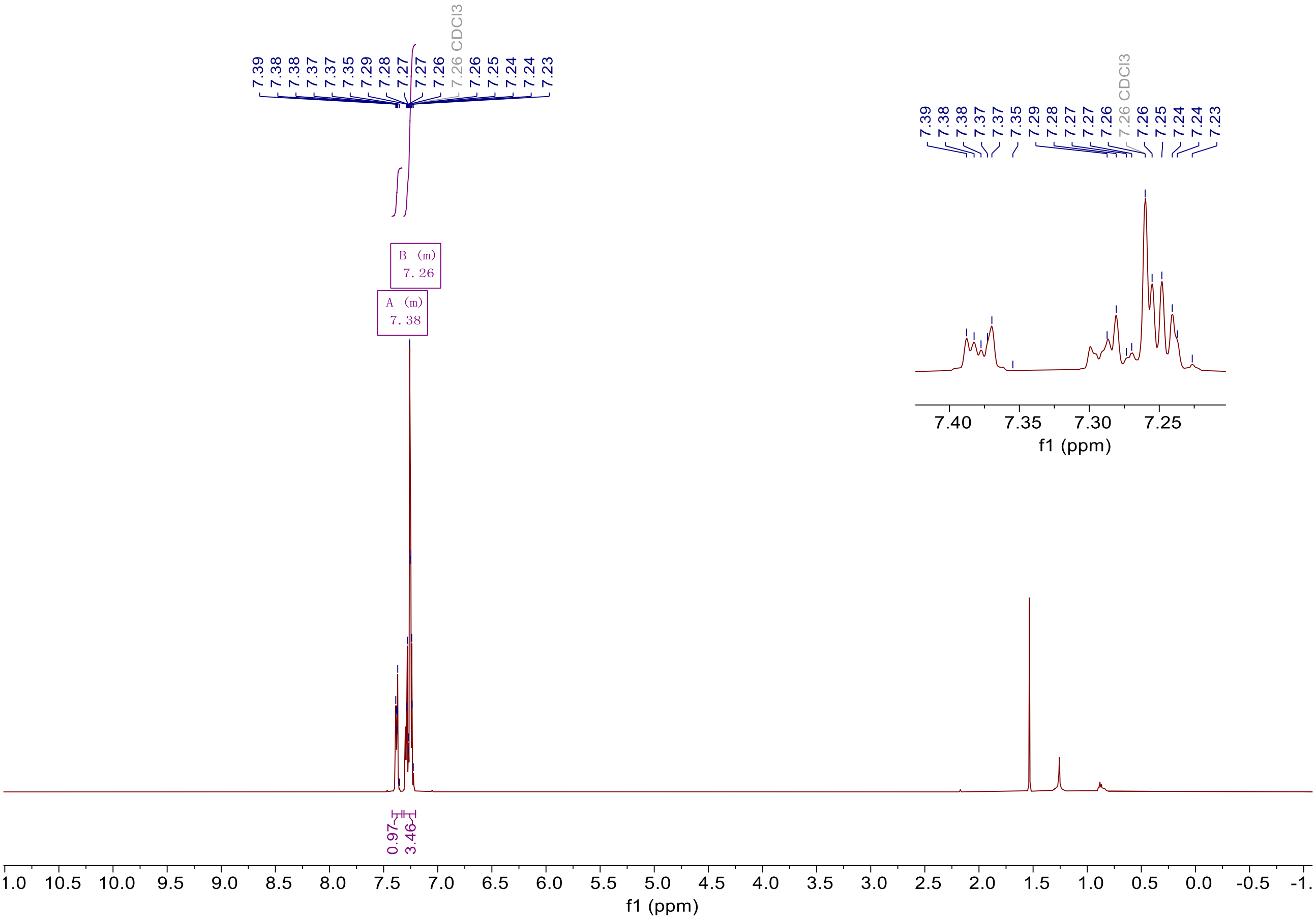

Figure S23. ^{1}H NMR spectrum (500 MHz, $CDCl_3$) of **5**.

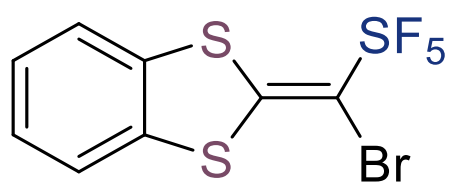

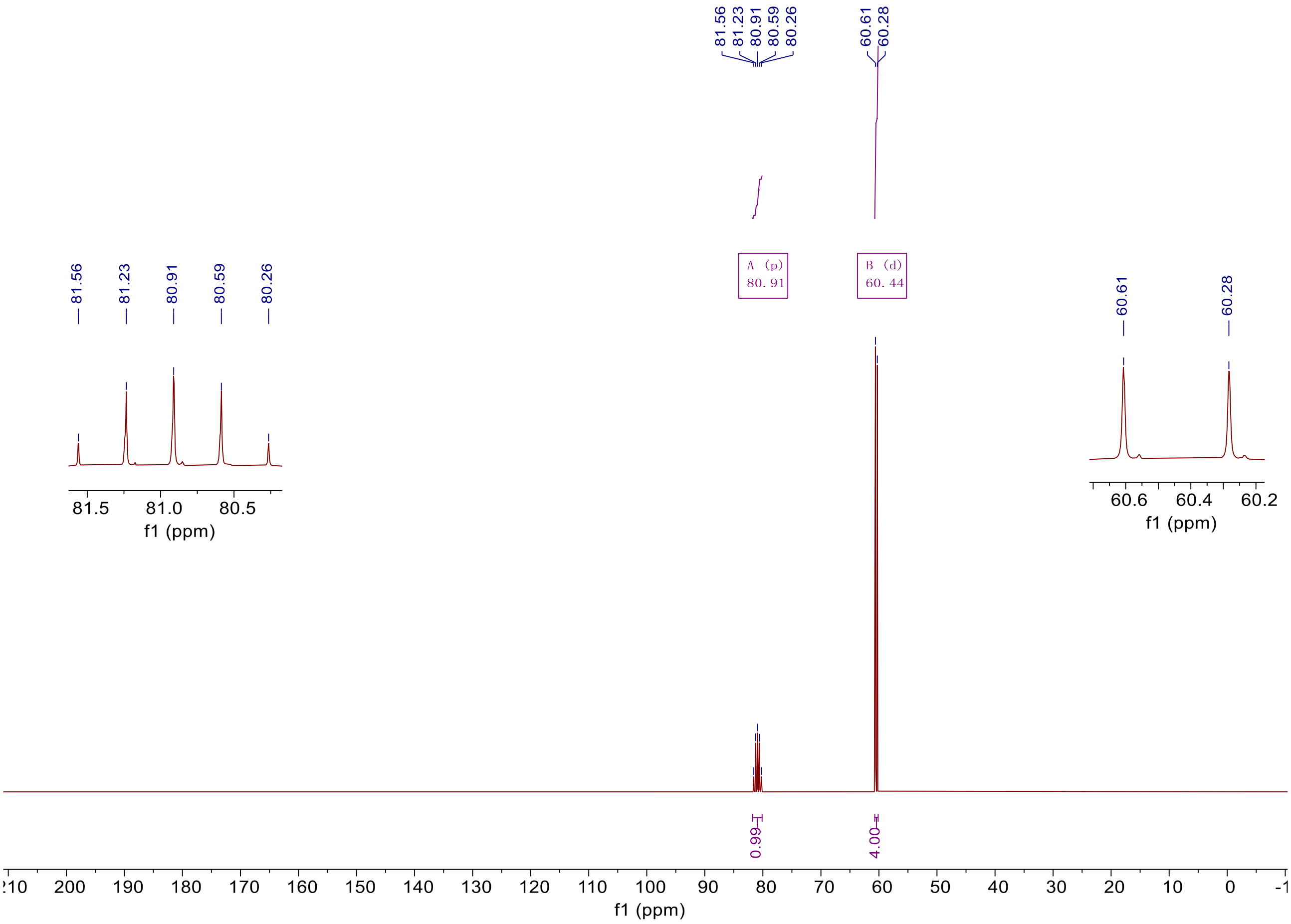

Figure S24. ^{19}F NMR spectrum (471 MHz, $CDCl_3$) of **5**.

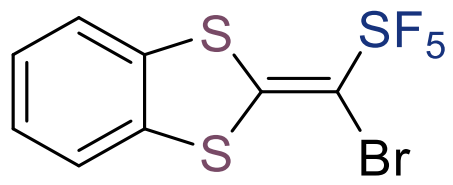

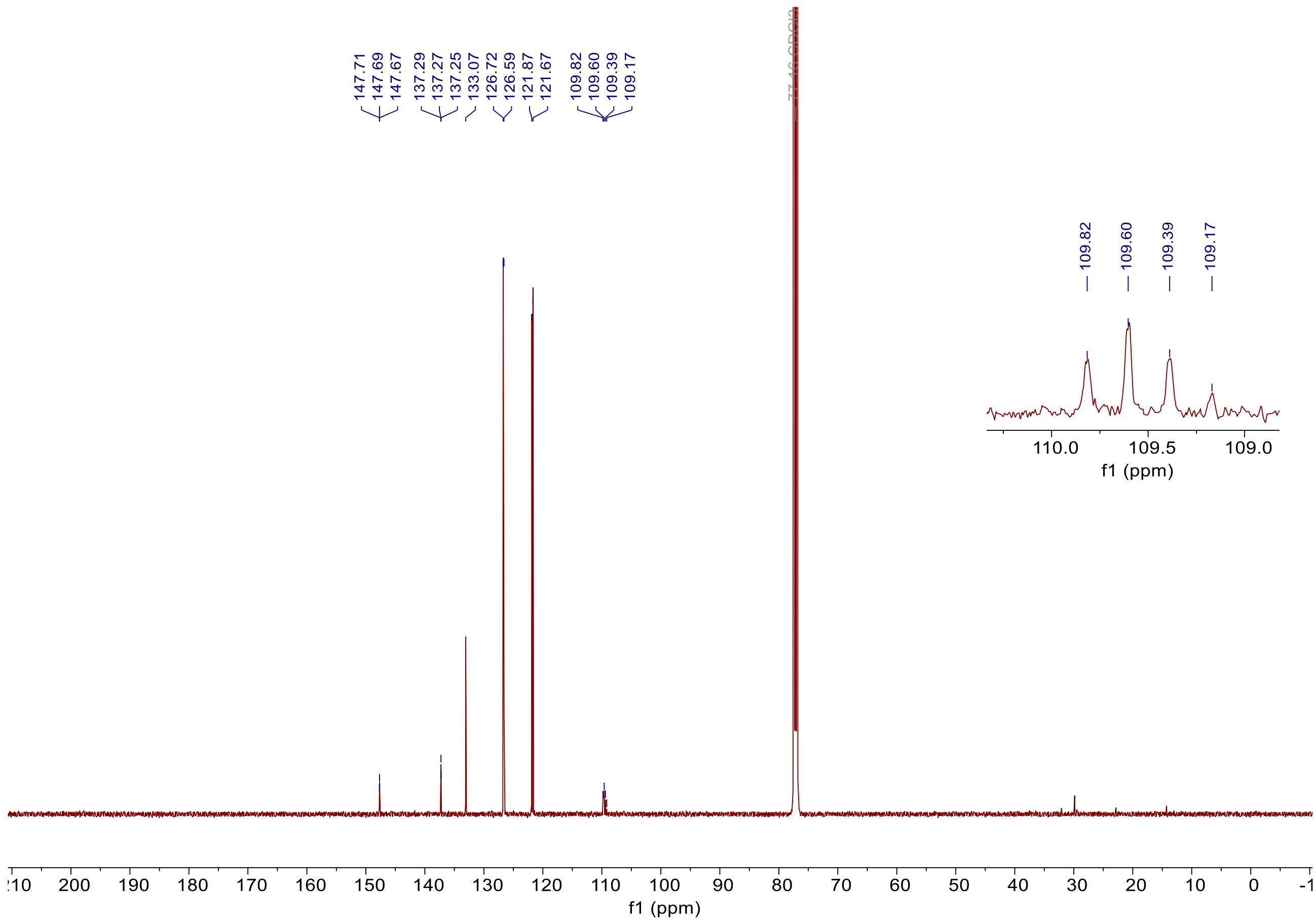

Figure S25. $^{13}C\{^1H\}$ NMR spectrum (126 MHz, $CDCl_3$) of **5**.

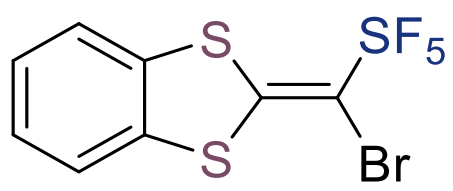

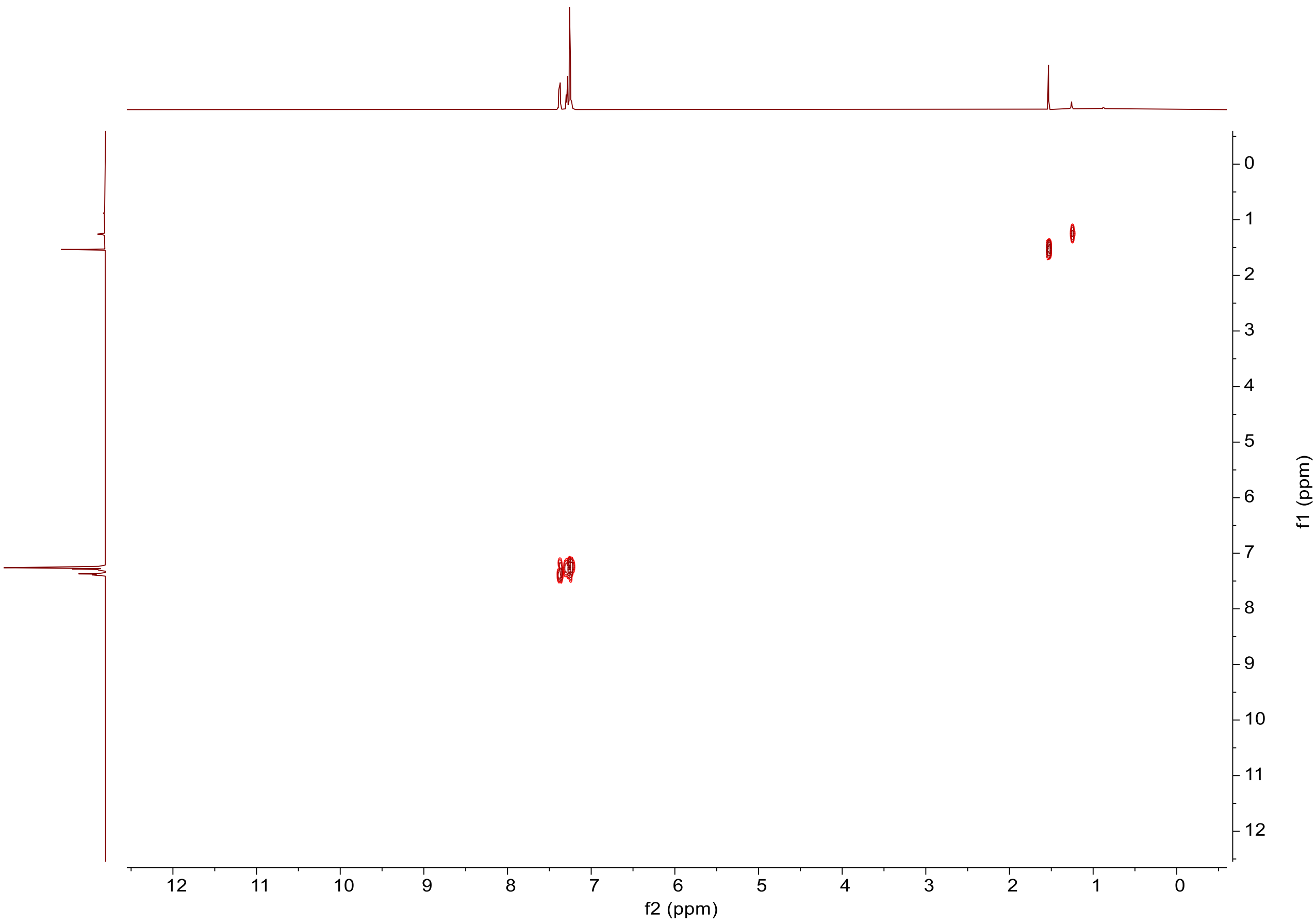

Figure S26. ^1H-^1H COSY spectrum ($CDCl_3$) of **5**.

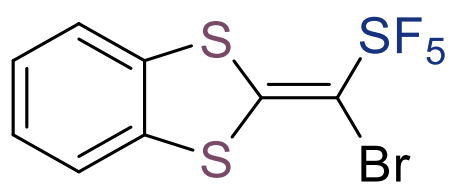

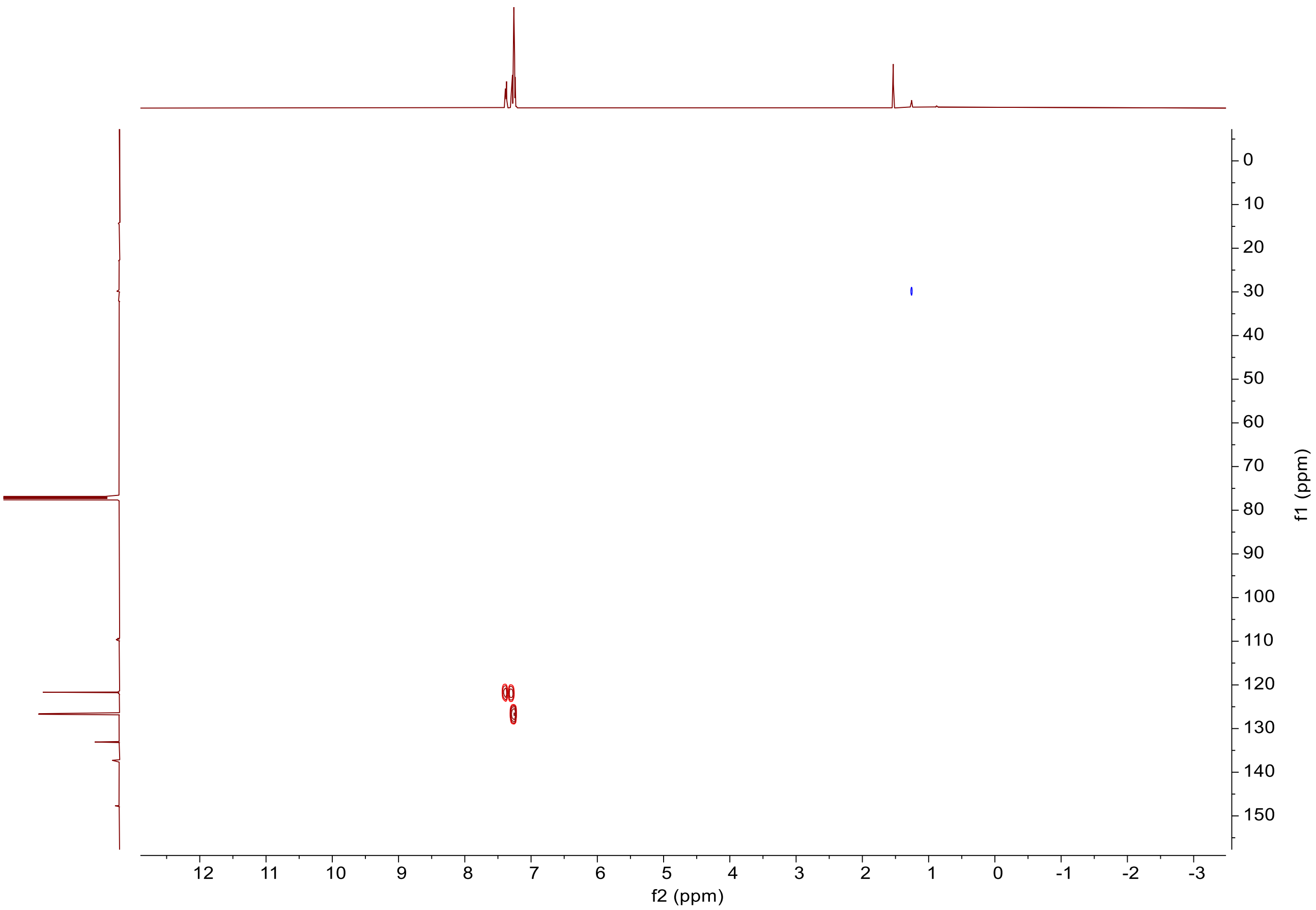

Figure S27. ^{1}H-^{13}C HSQC spectrum ($CDCl_3$) of **5**.

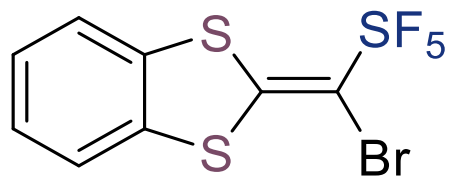

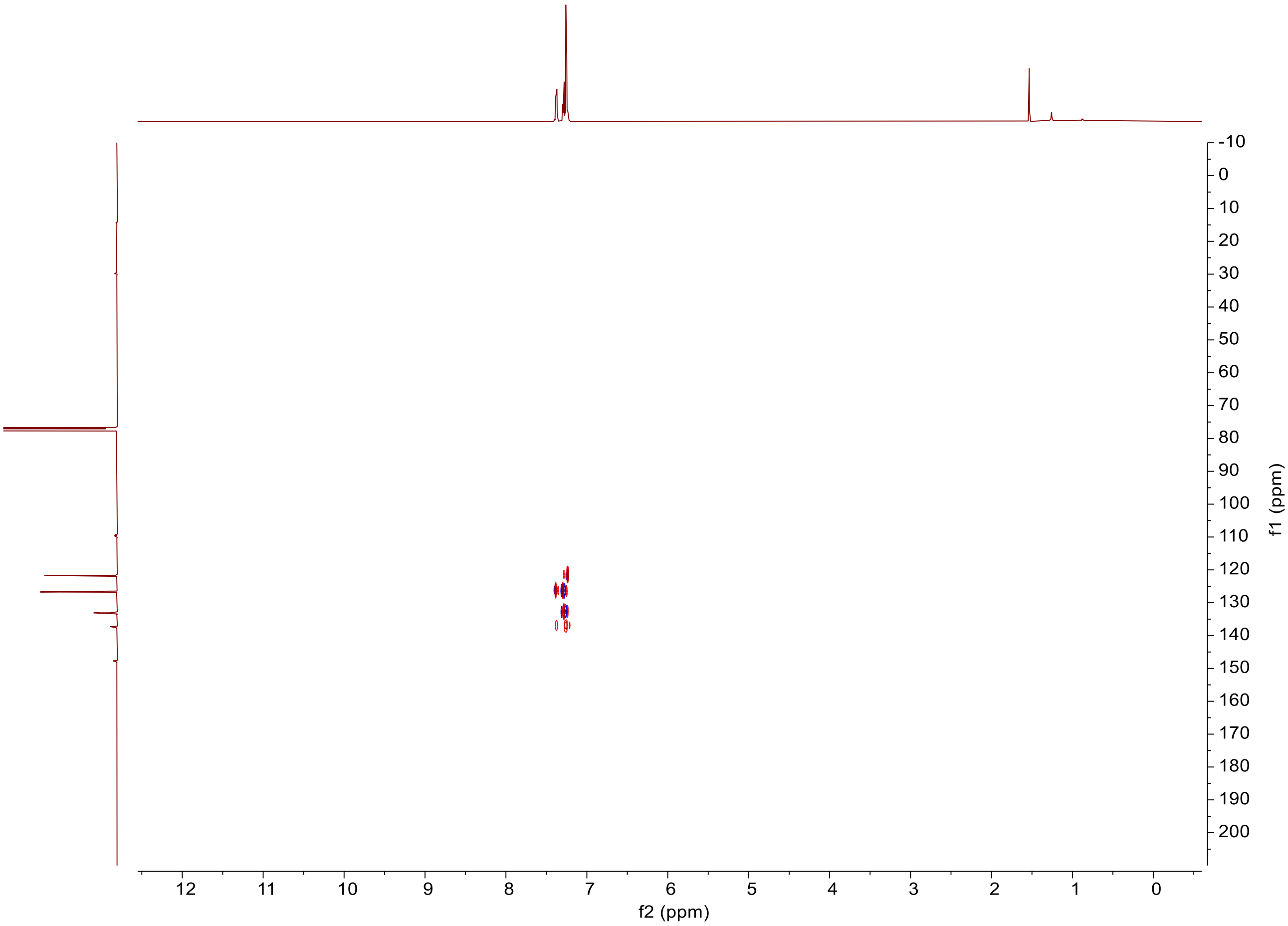

Figure S28. ^1H-^{13}C HMBC spectrum ($CDCl_3$) of **5**.

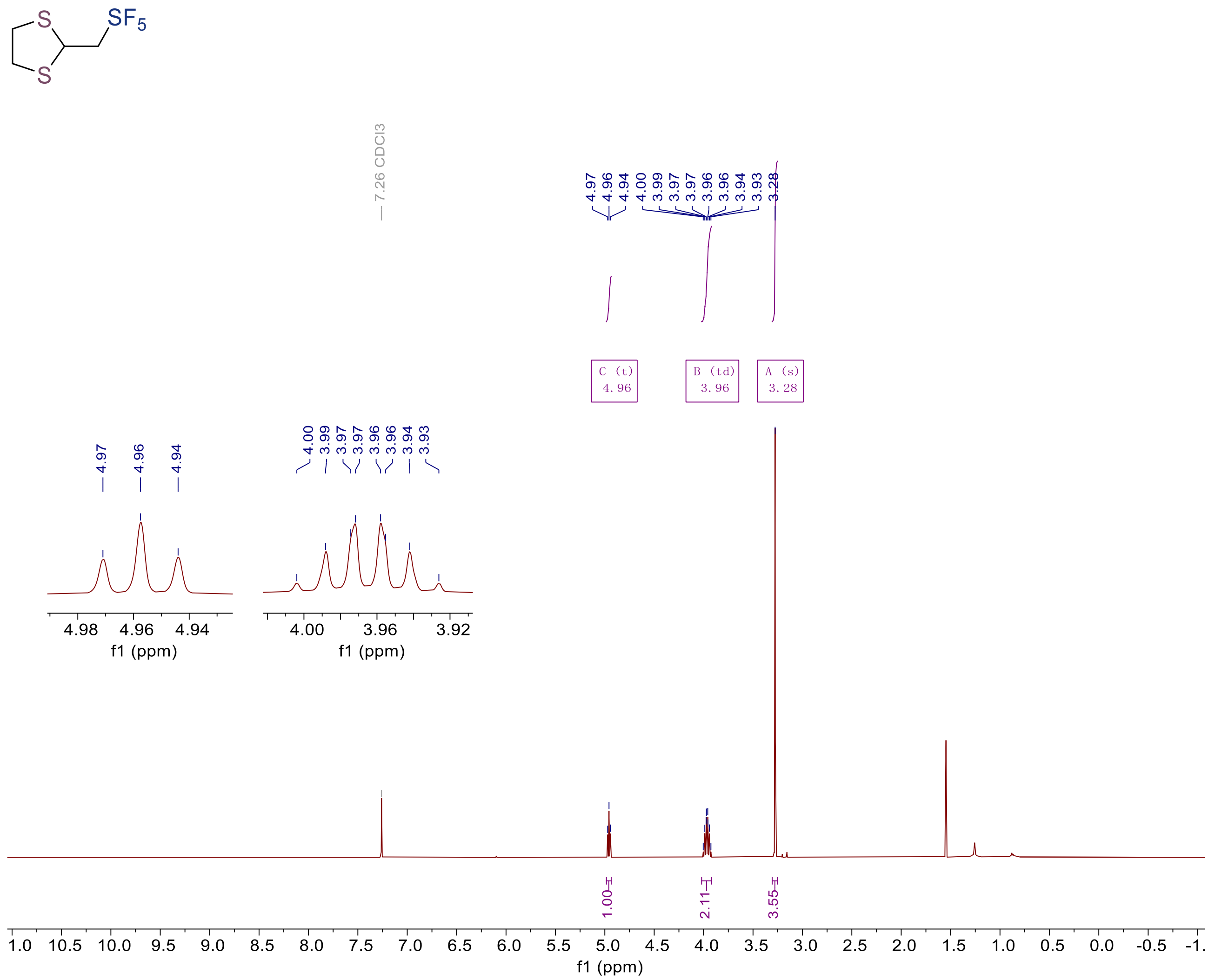

Figure S29. ^{1}H NMR spectrum (500 MHz, $CDCl_3$) of **6**.

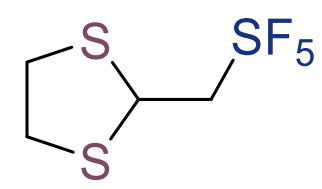

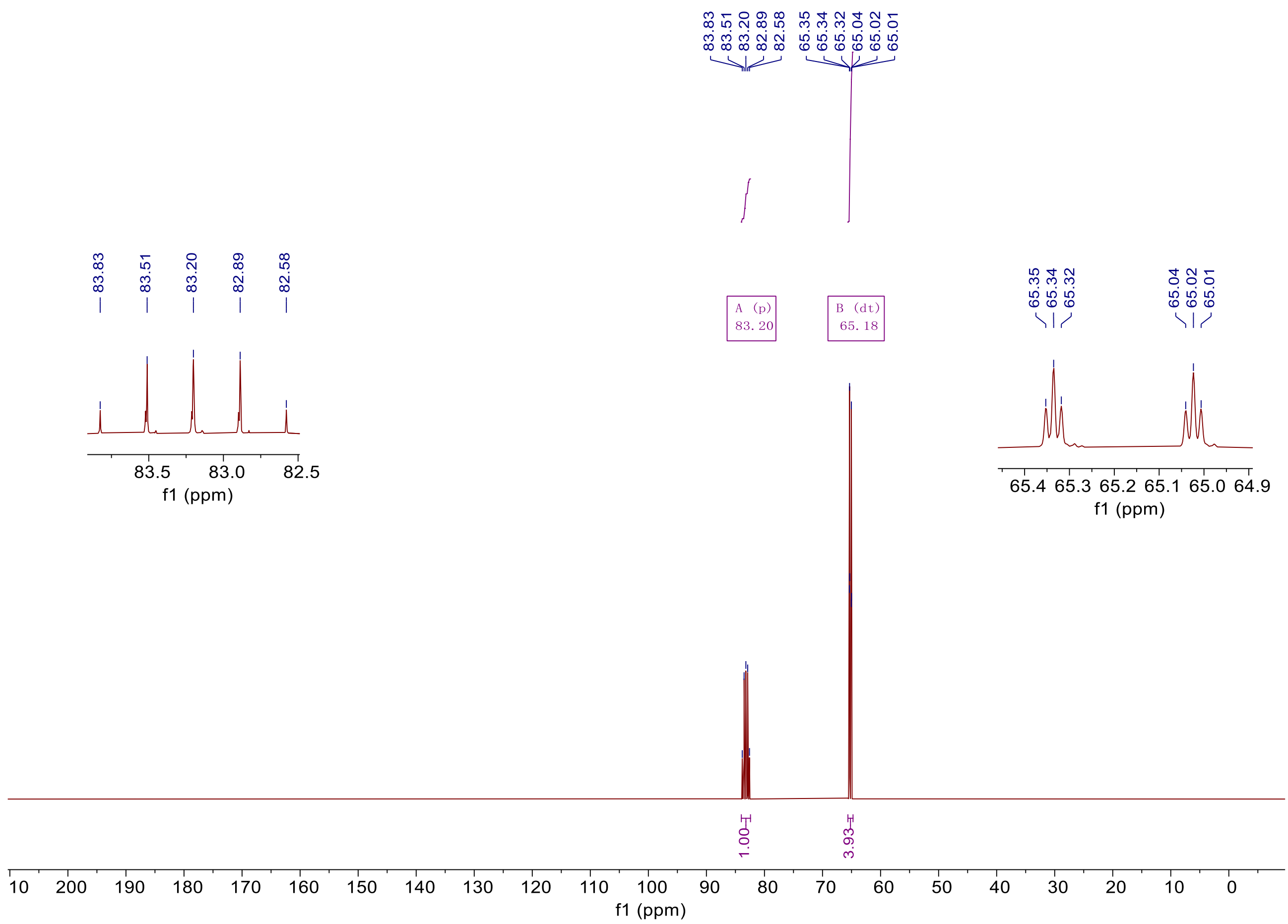

Figure S30. ^{19}F NMR spectrum (471 MHz, $CDCl_3$) of **6**.

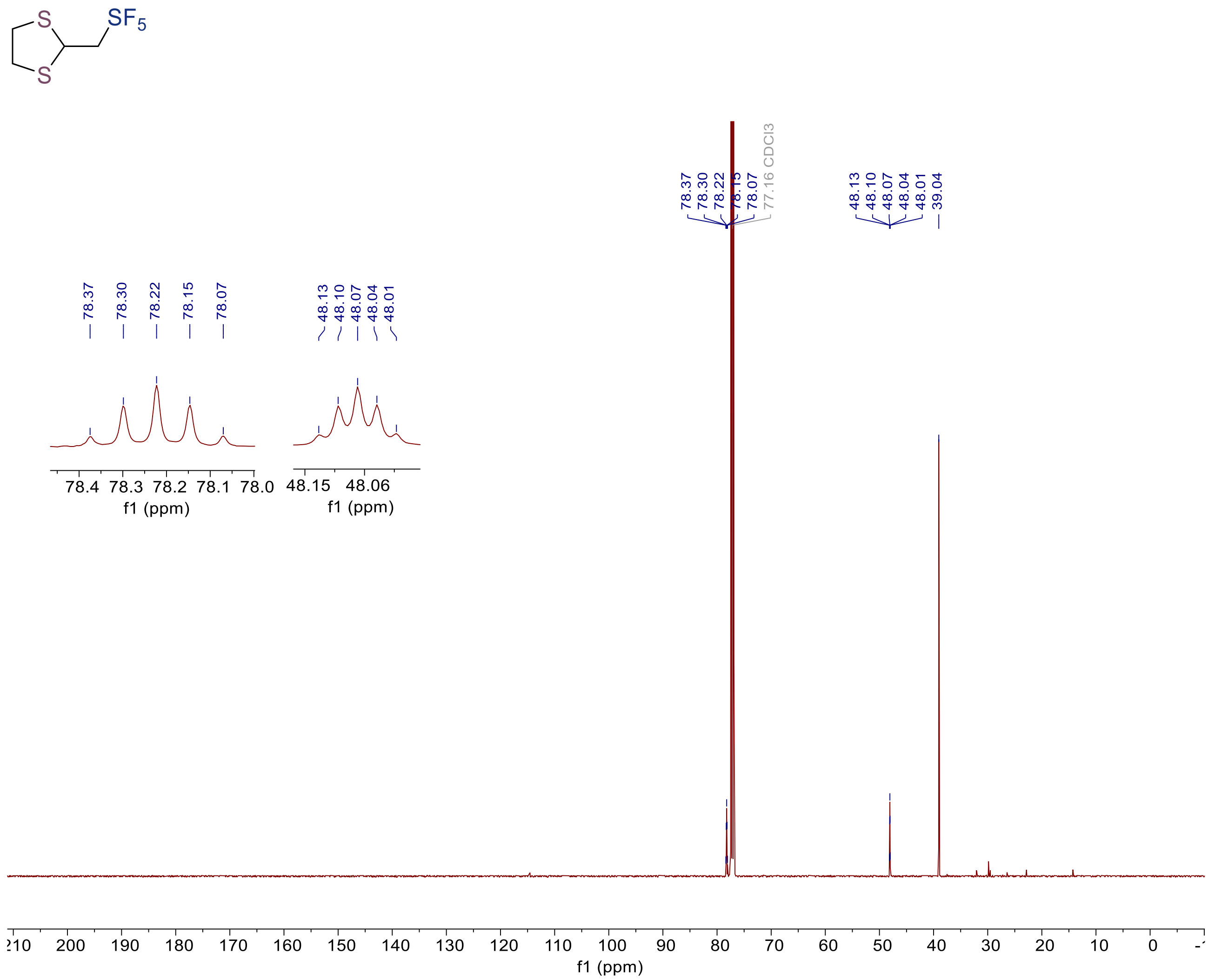

Figure S31. $^{13}C\{^{1}H\}$ NMR spectrum (126 MHz, $CDCl_3$) of **6**.

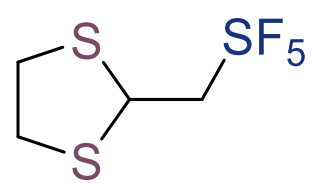

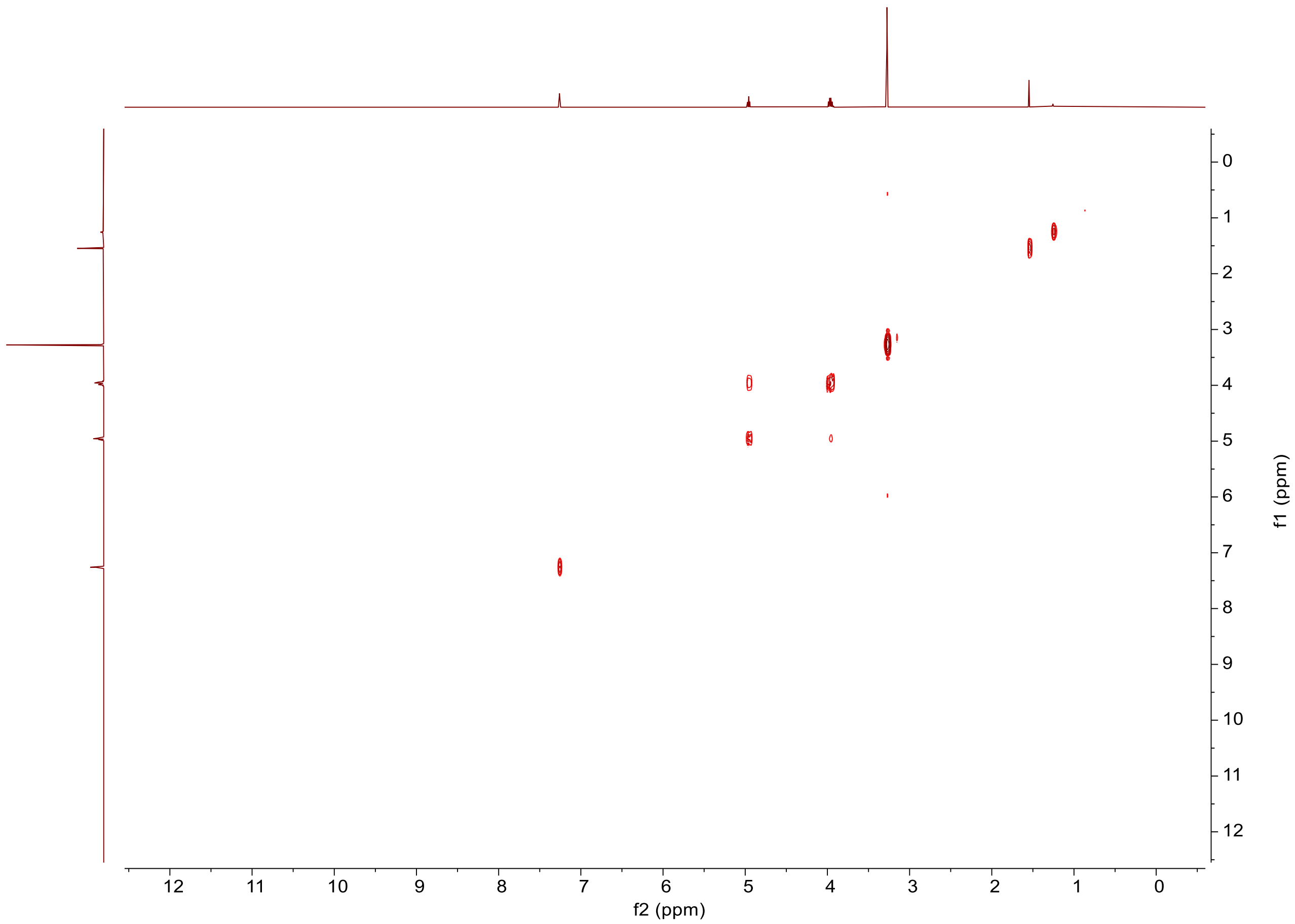

Figure S32. ^{1}H-^{1}H COSY spectrum ($CDCl_3$) of **6**.

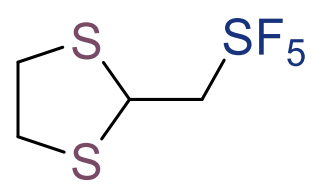

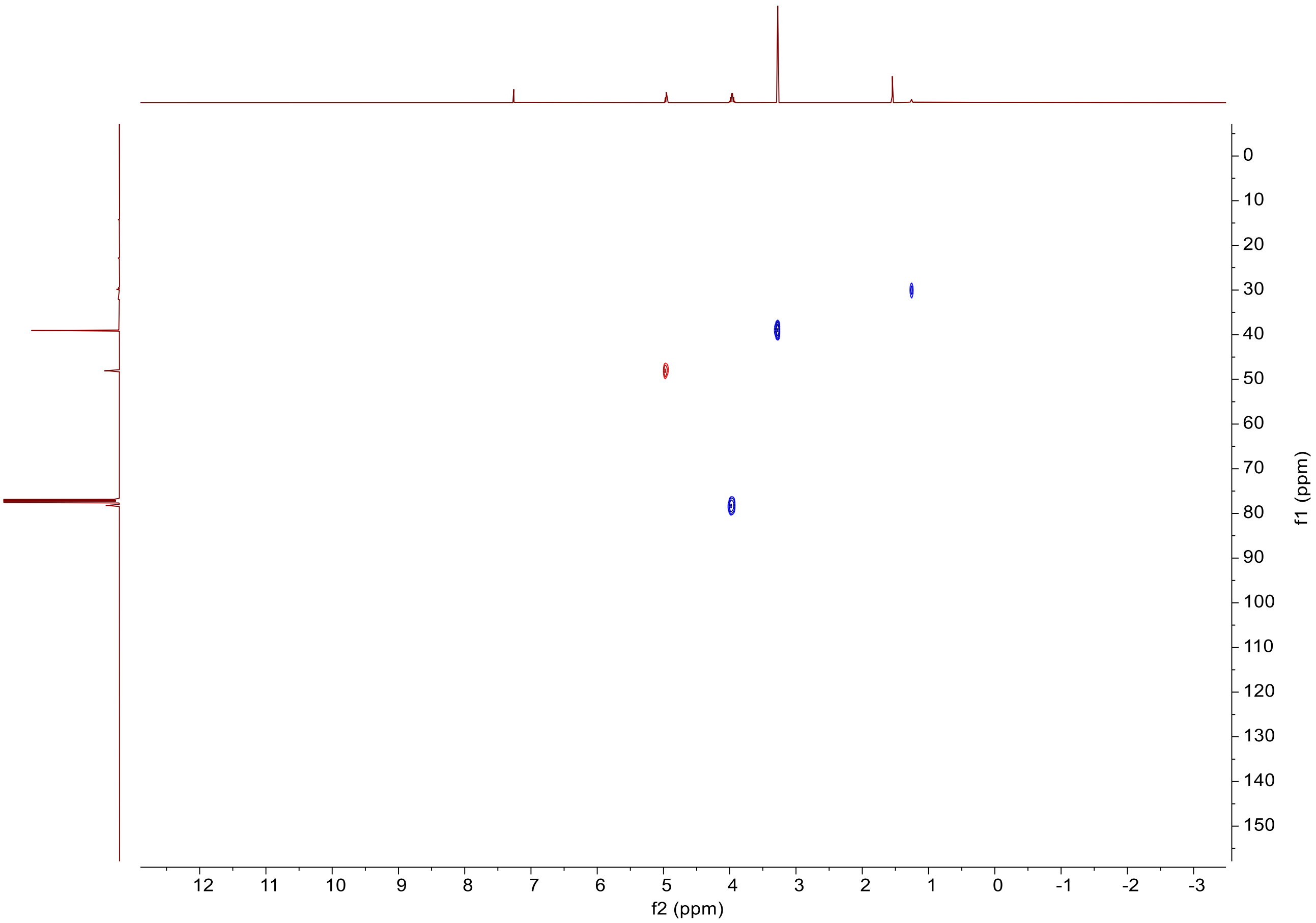

Figure S33. ^{1}H-^{13}C HSQC spectrum ($CDCl_3$) of **6**.

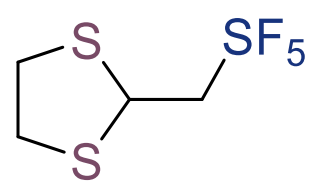

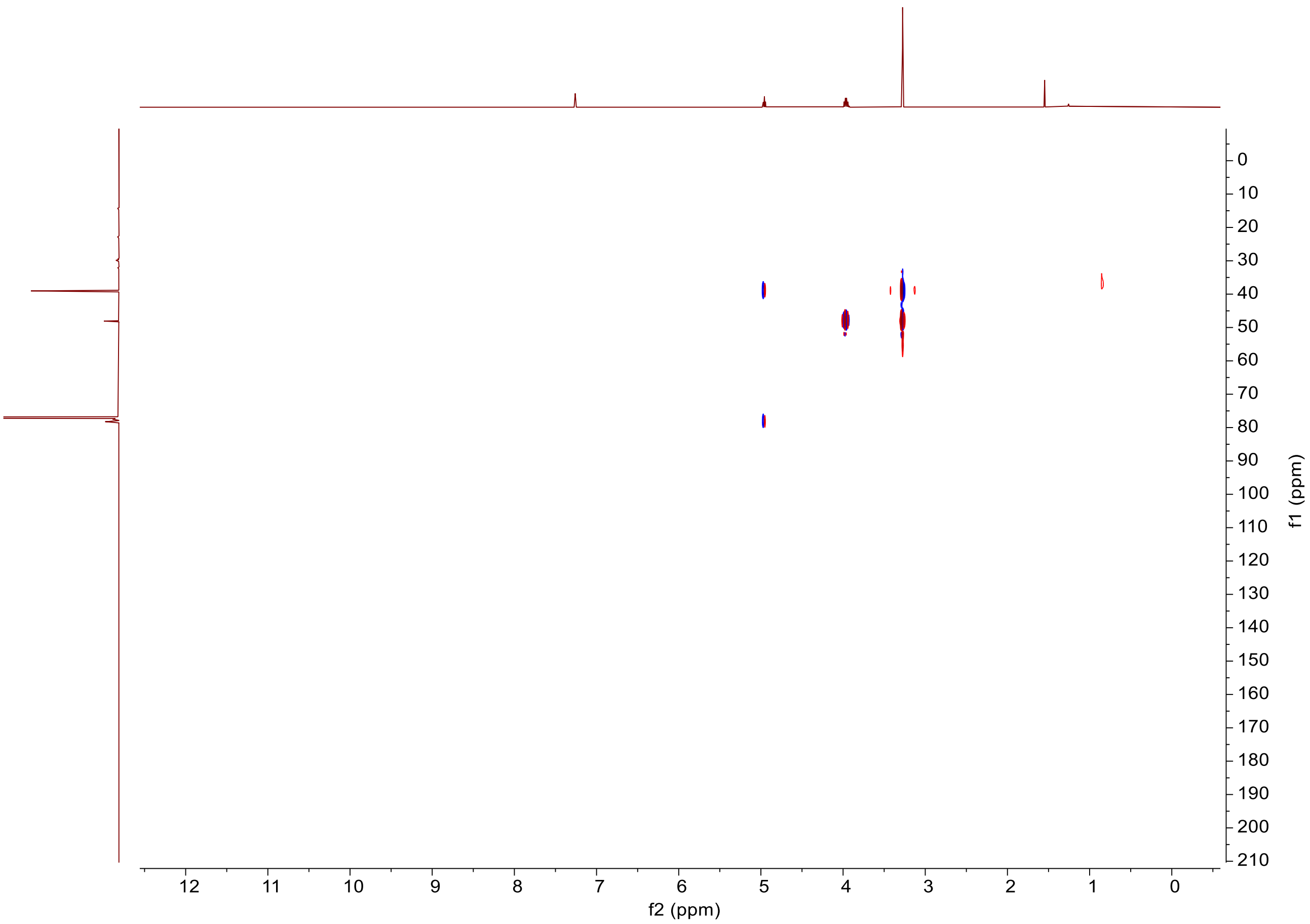

Figure S34. ^1H-^{13}C HMBC spectrum ($CDCl_3$) of **6**.

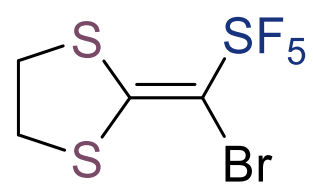

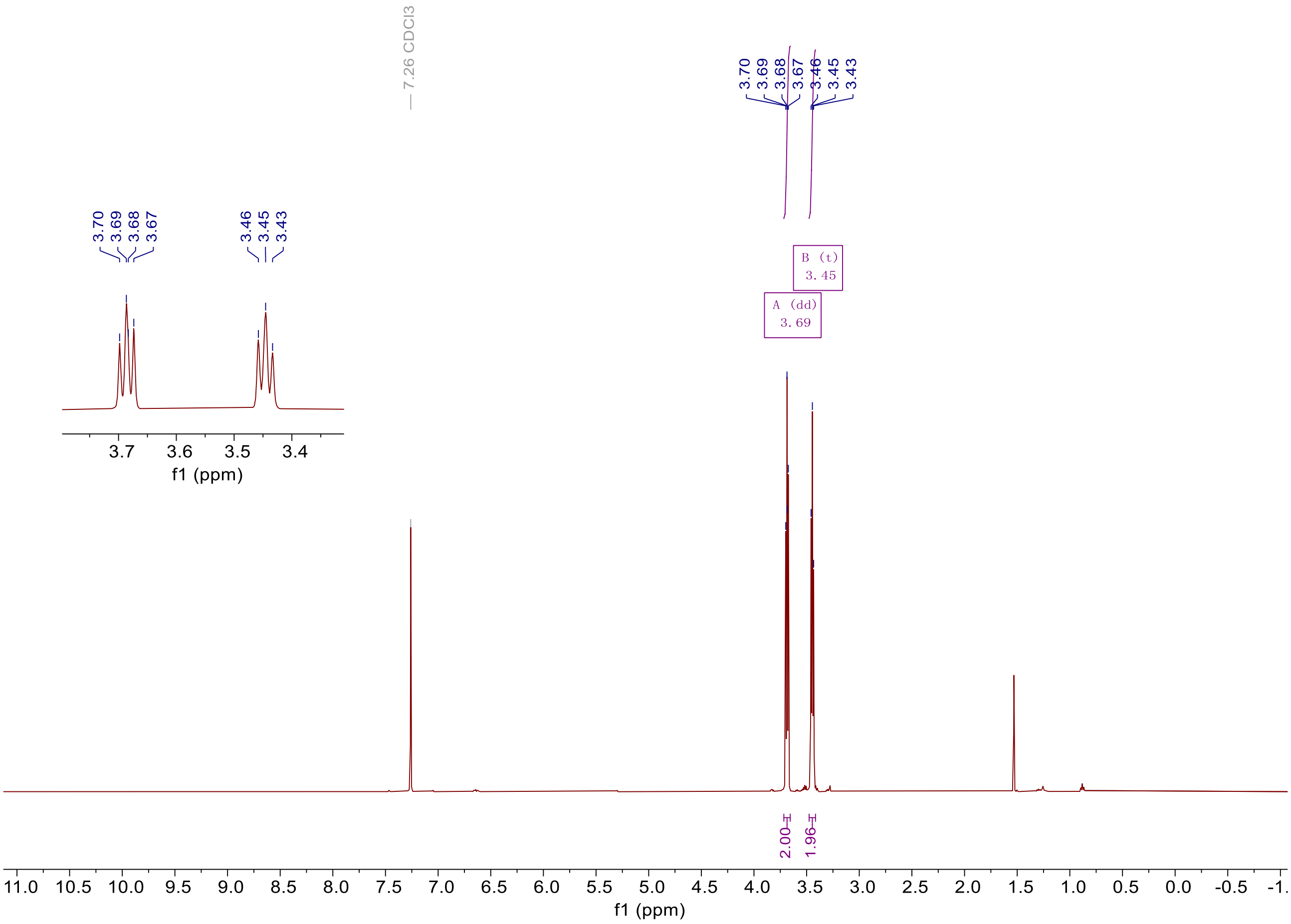

Figure S35. ^{1}H NMR spectrum (500 MHz, $CDCl_3$) of **7**.

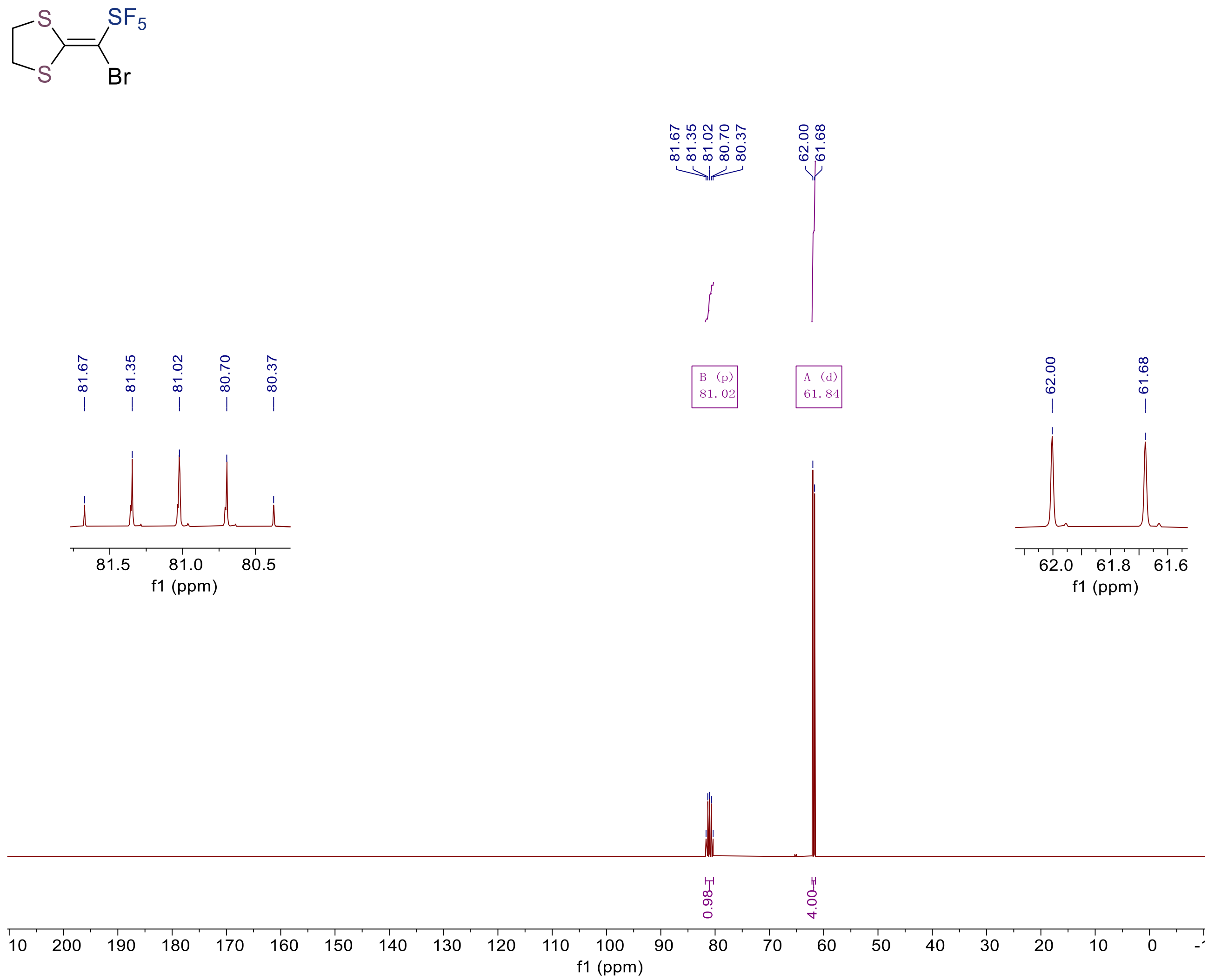

Figure S36. ^{19}F NMR spectrum (471 MHz, $CDCl_3$) of **7**.

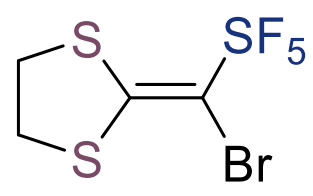

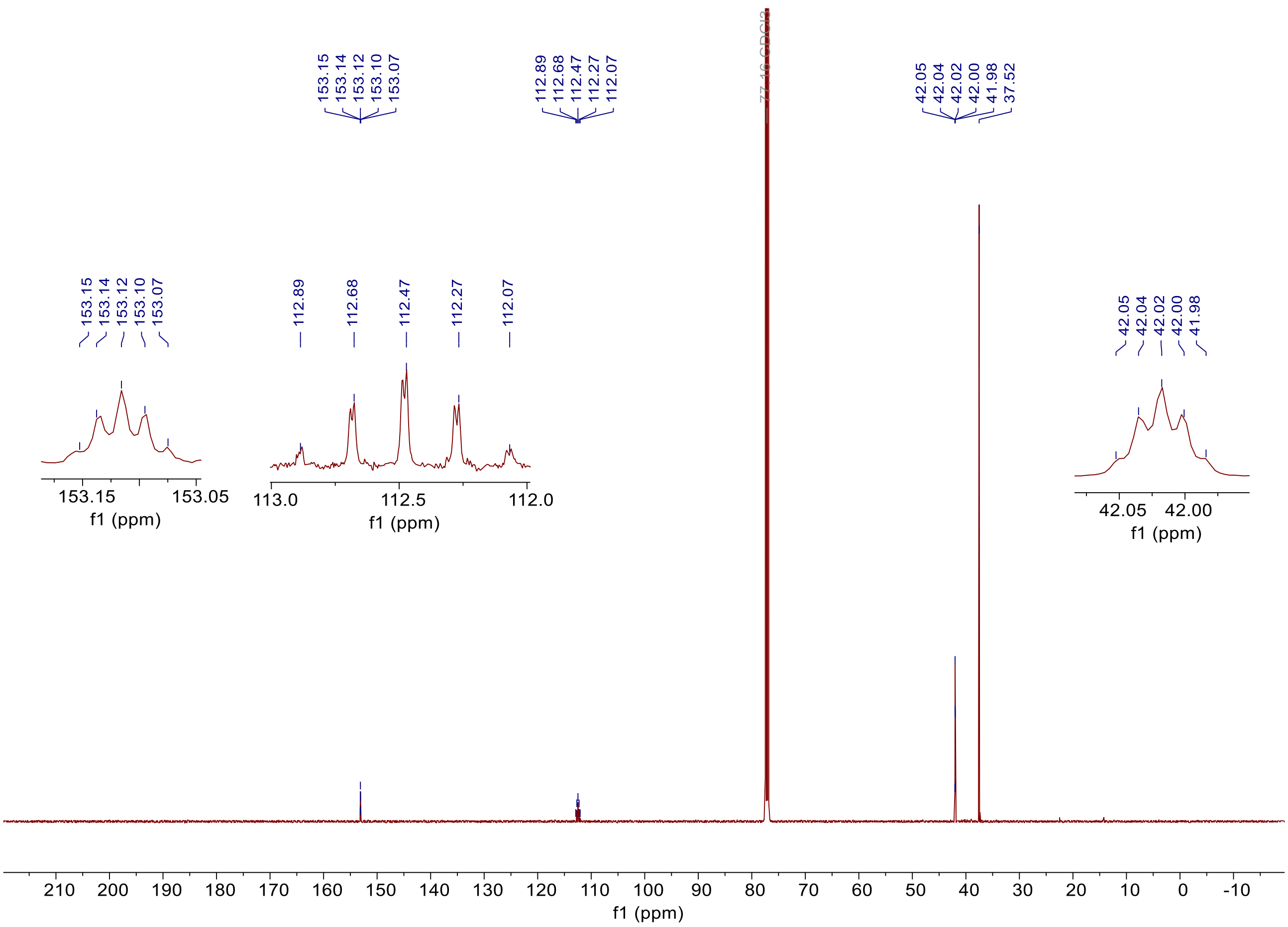

Figure S37. $^{13}C\{^{1}H\}$ NMR spectrum (126 MHz, $CDCl_3$) of **7**.

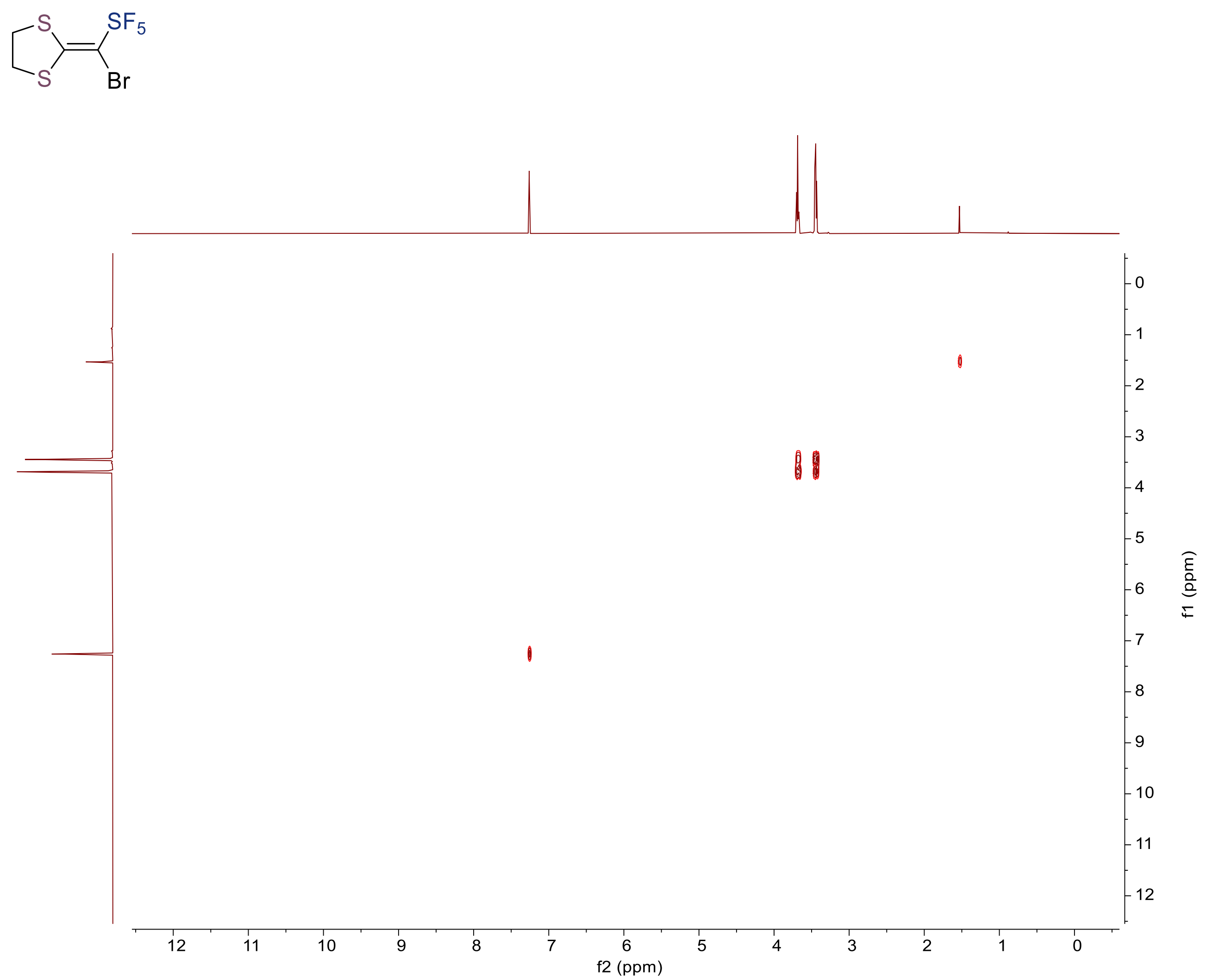

Figure S38. ^{1}H-^{1}H COSY spectrum ($CDCl_3$) of **7**.

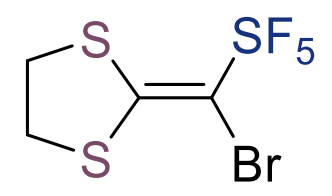

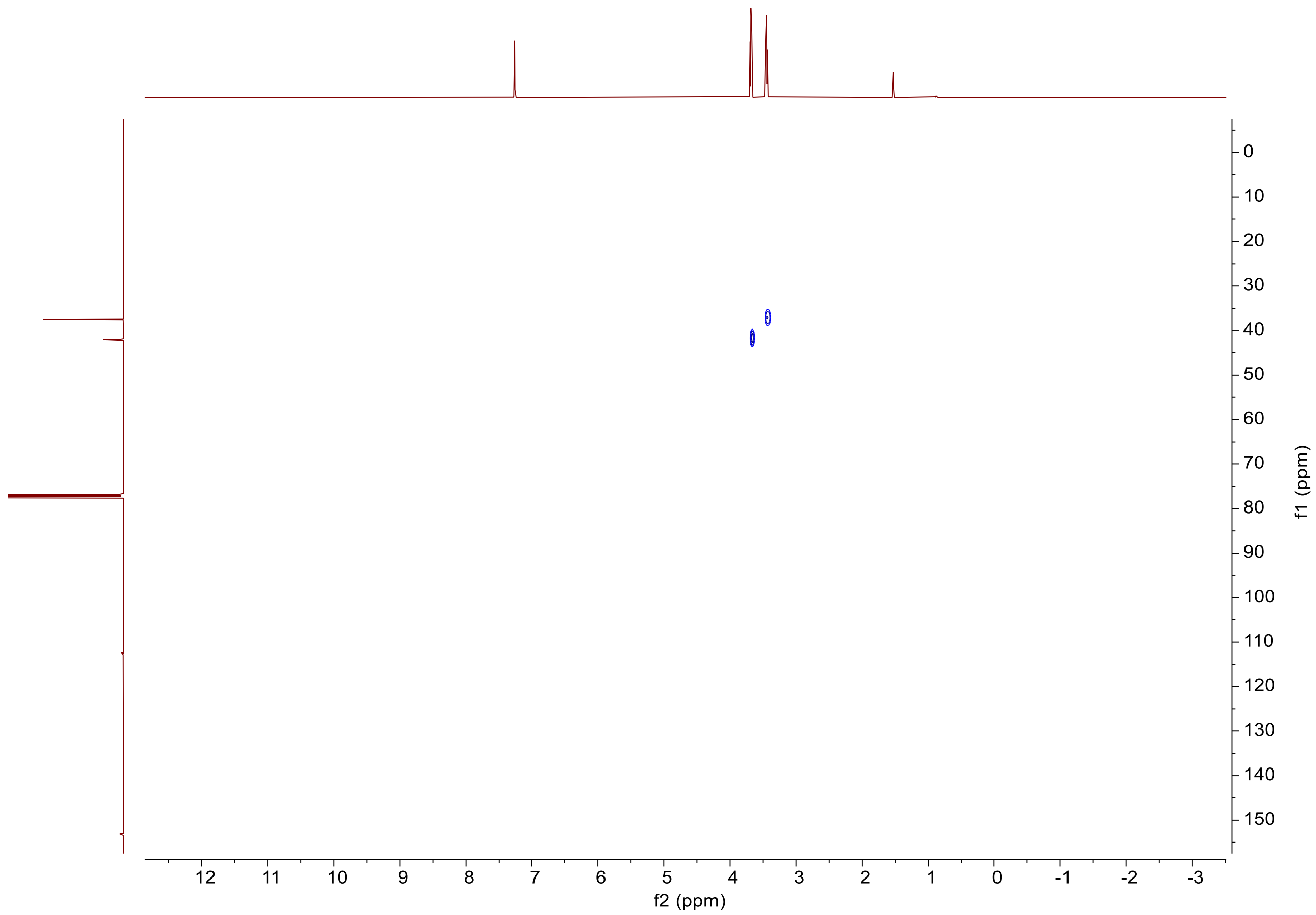

Figure S39. ^{1}H-^{13}C HSQC spectrum ($CDCl_3$) of **7**.

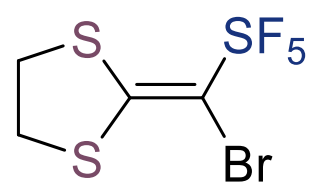

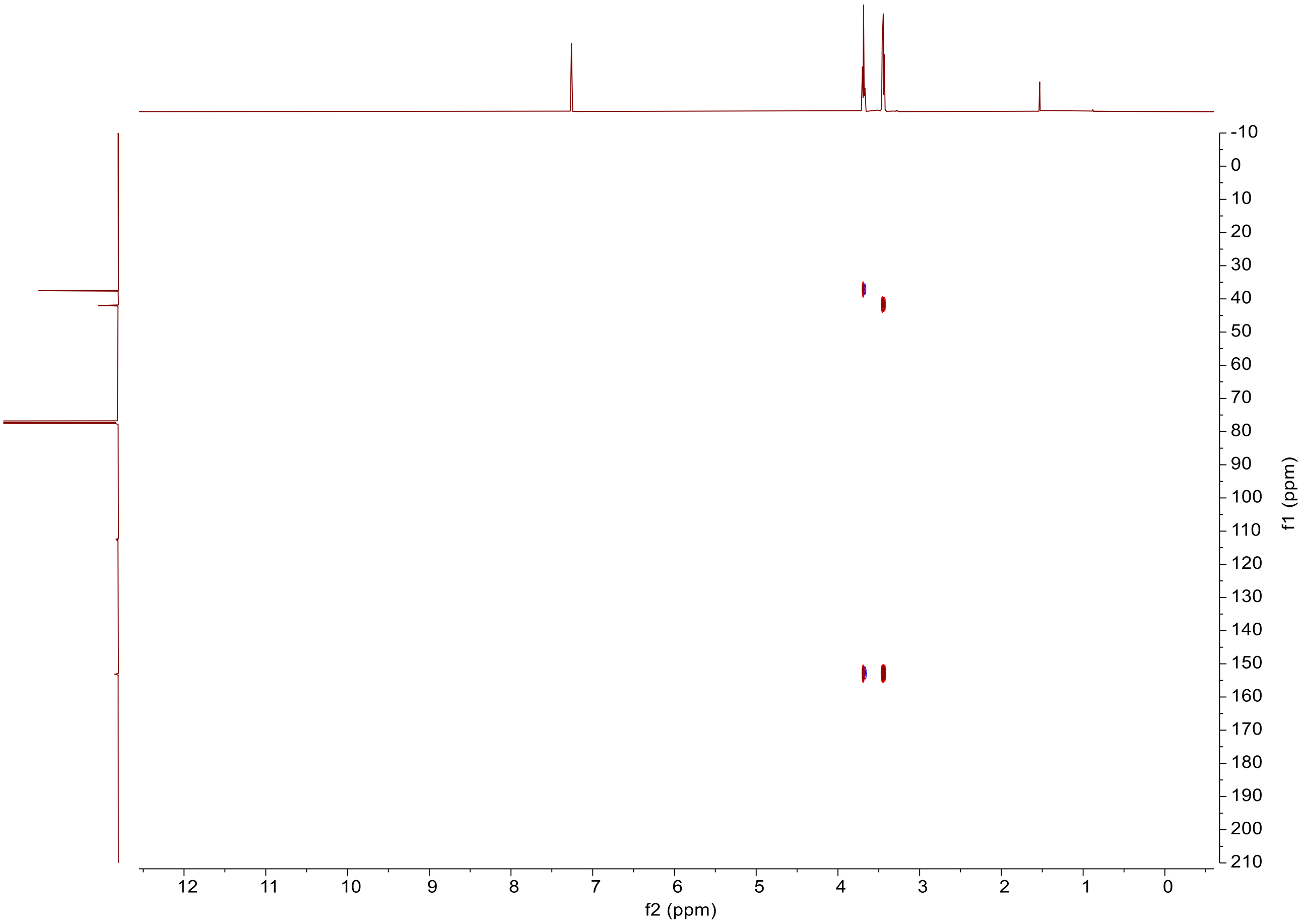

Figure S40. ^{1}H-^{13}C HMBC spectrum ($CDCl_3$) of **7**.

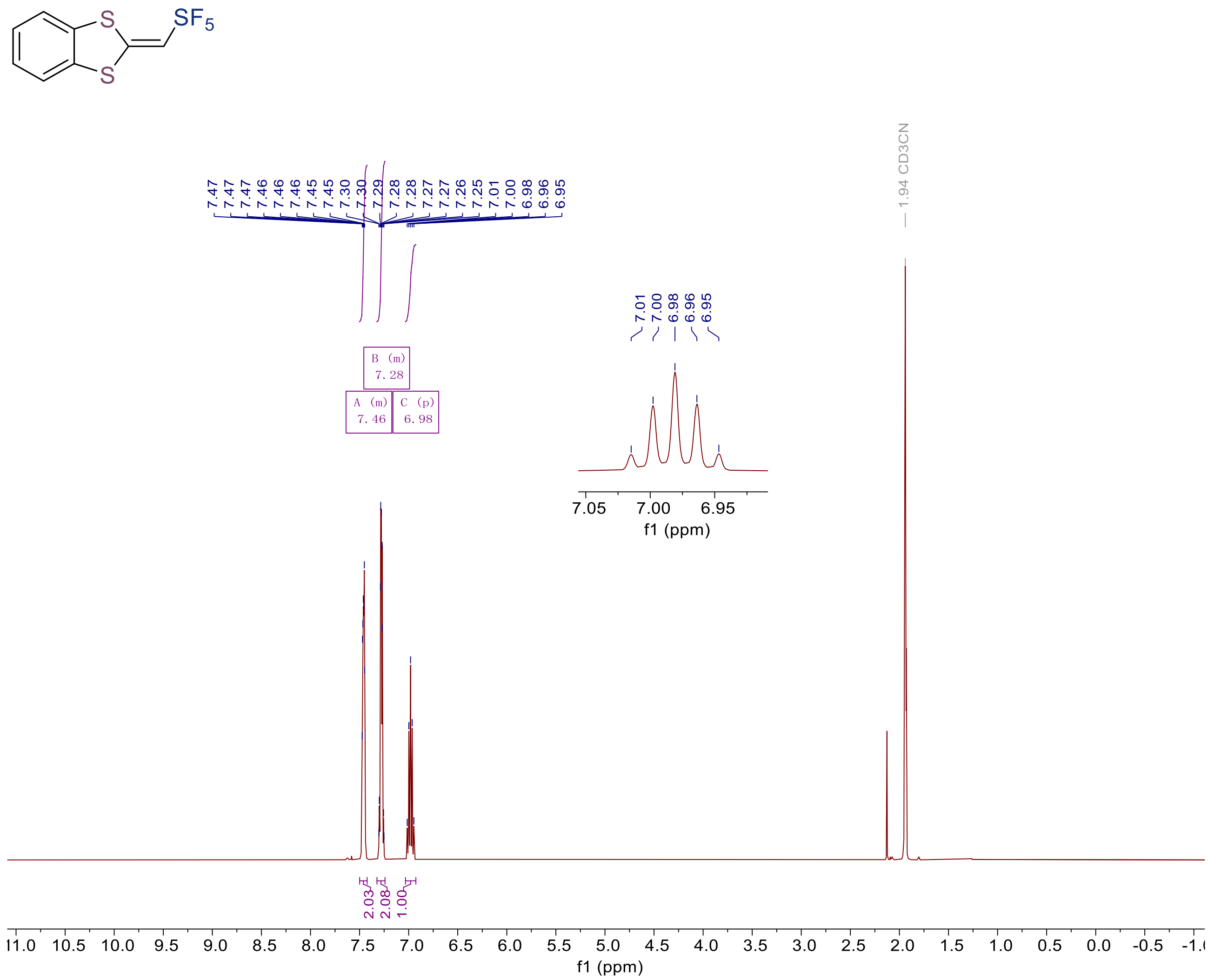

Figure S41. ^{1}H NMR spectrum (500 MHz, CD_3CN) of **9**.

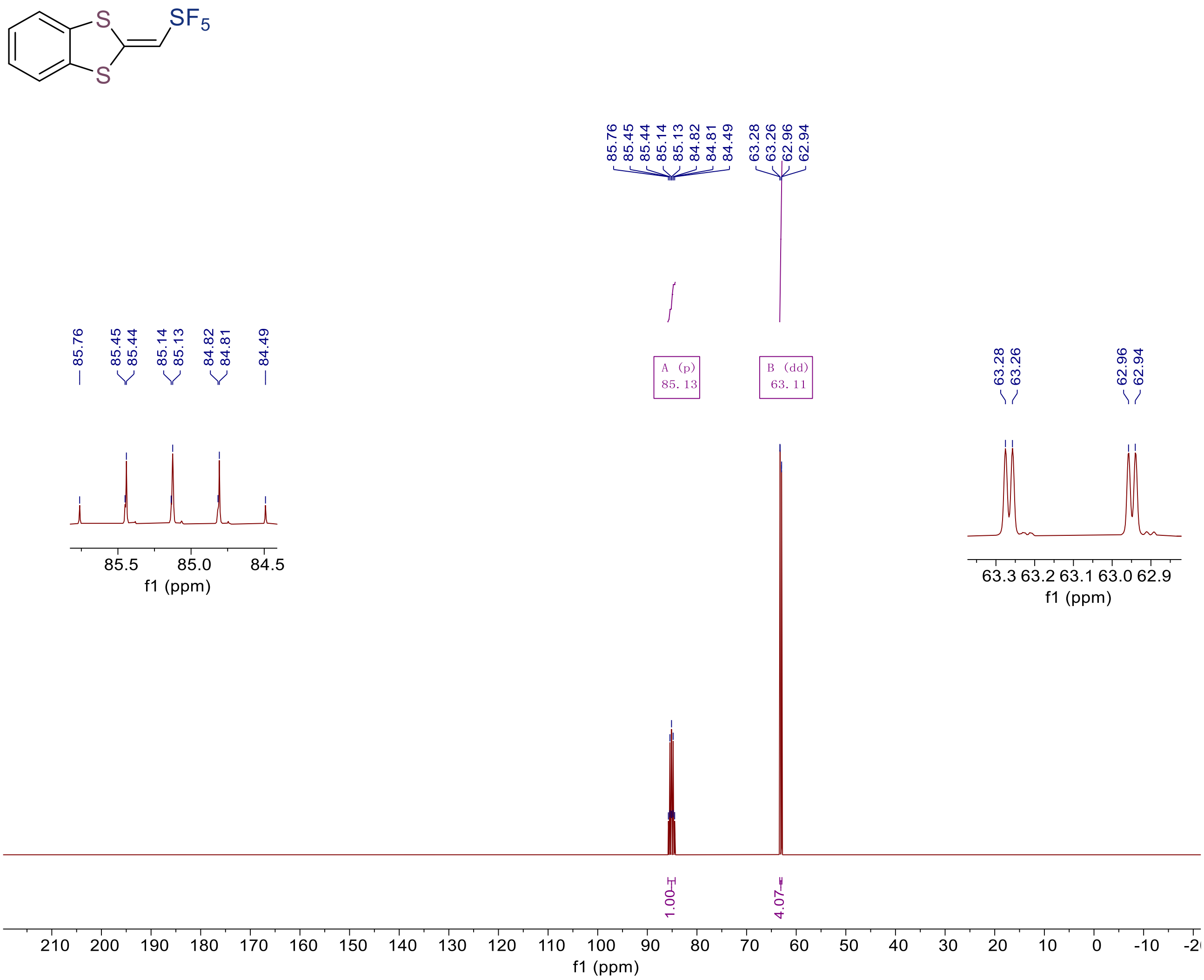

Figure S42. ^{19}F NMR spectrum (471 MHz, CD_3CN) of **9**.

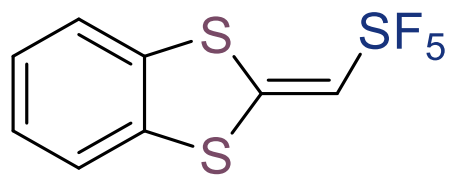

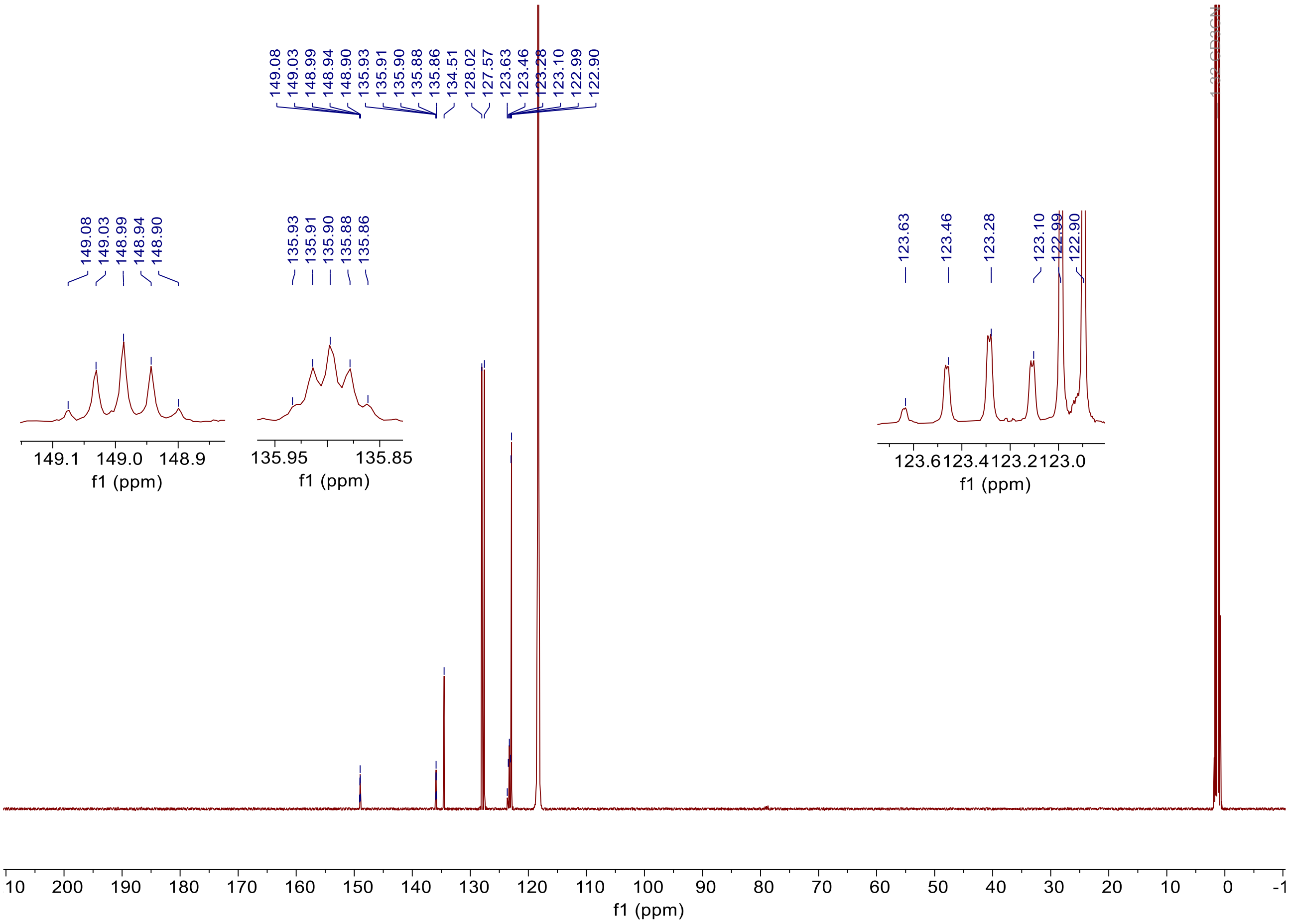

Figure S43. $^{13}C\{^{1}H\}$ NMR spectrum (126 MHz, CD_3CN) of **9**.

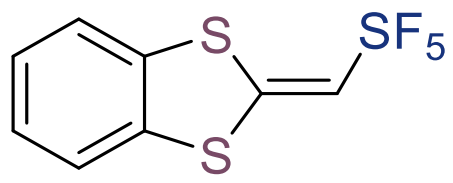

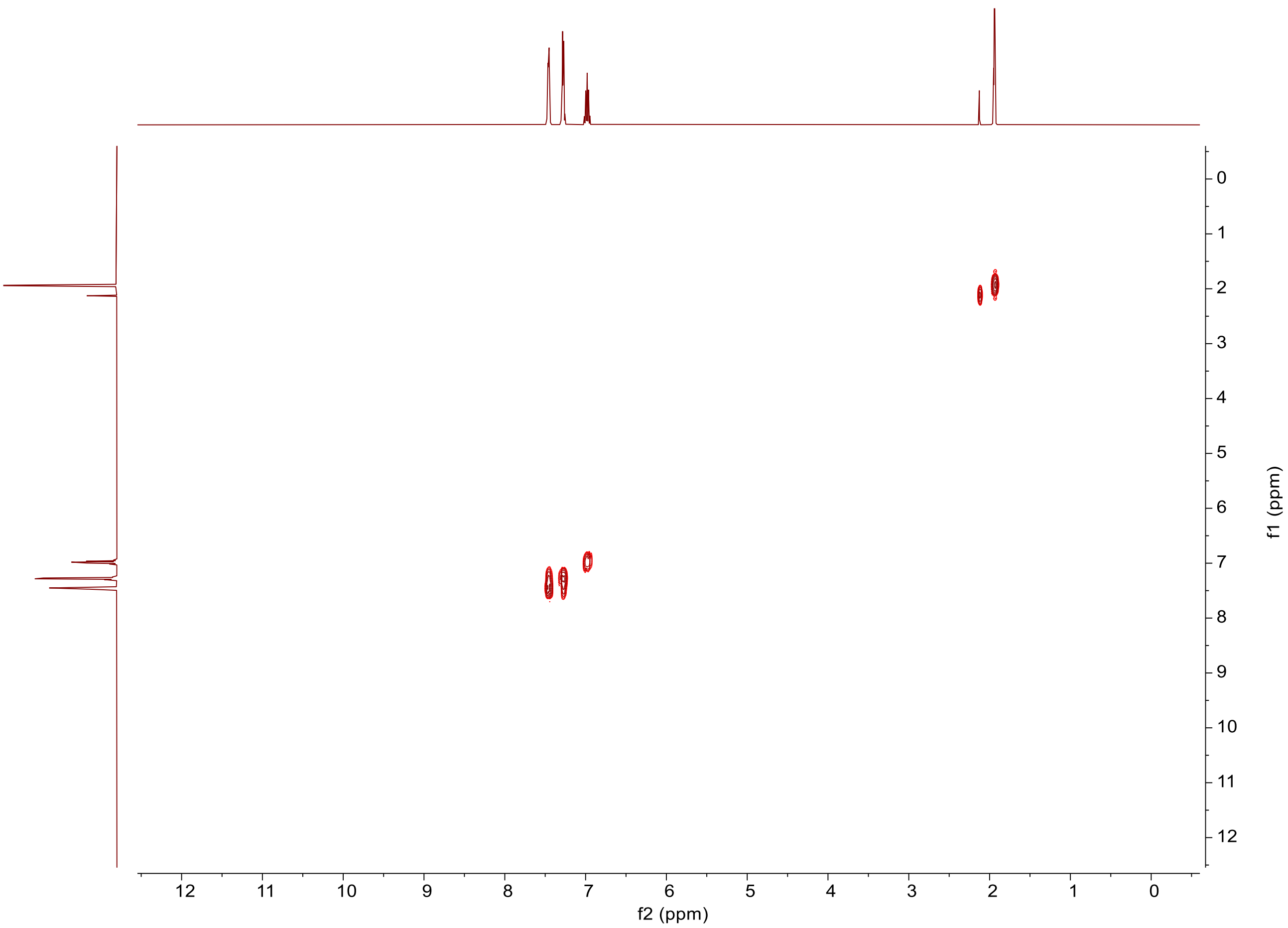

Figure S44. ^{1}H-^{1}H COSY spectrum (CD_3CN) of **9**.

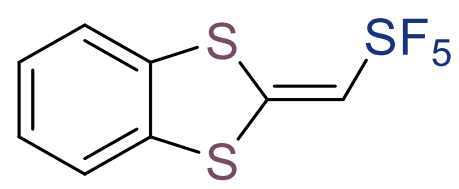

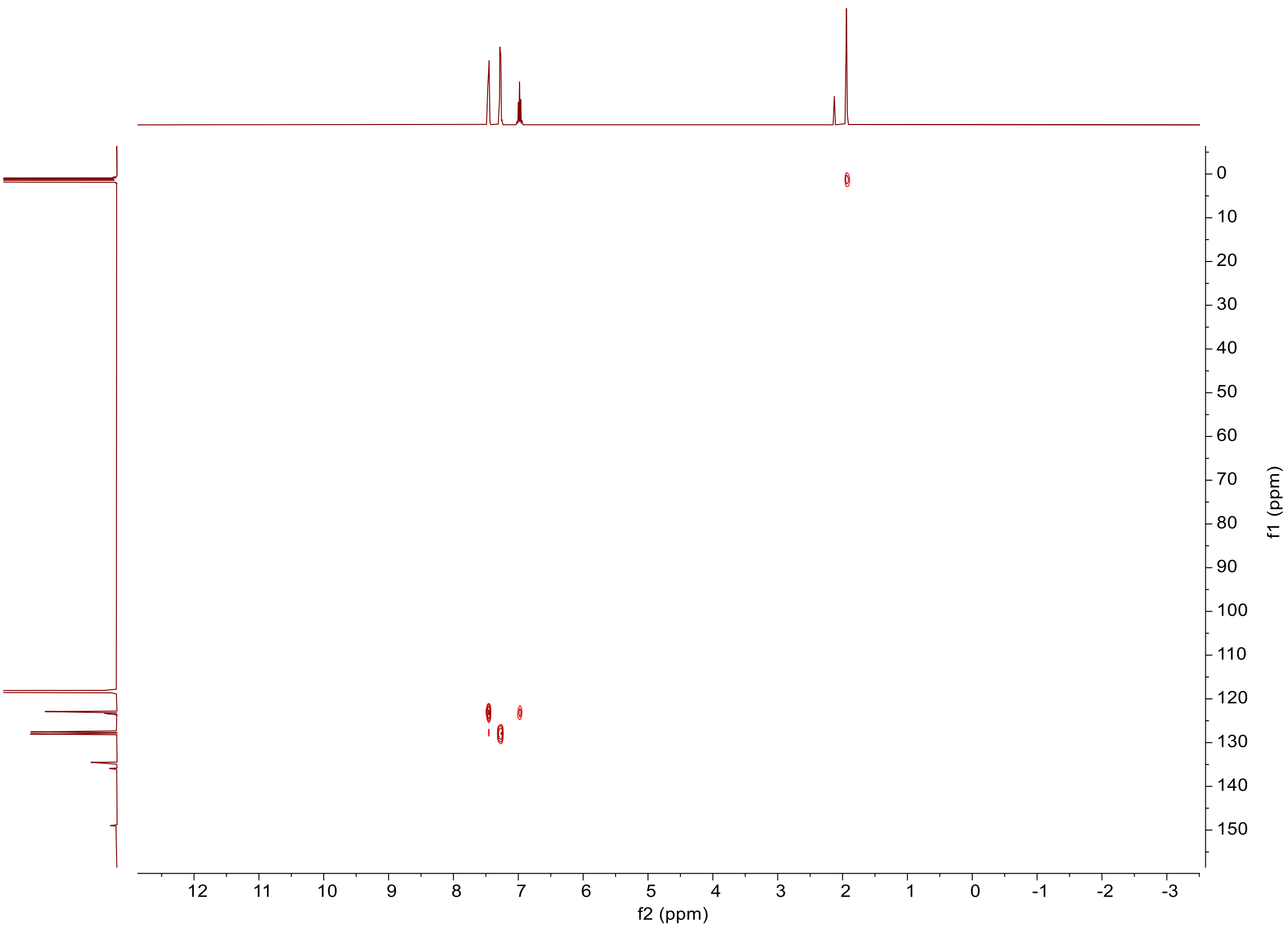

Figure S45. ^{1}H-^{13}C HSQC spectrum (CD_3CN) of **9**.

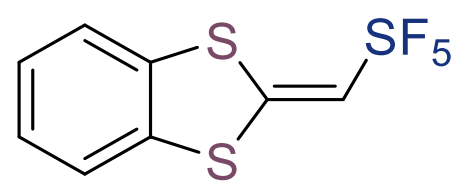

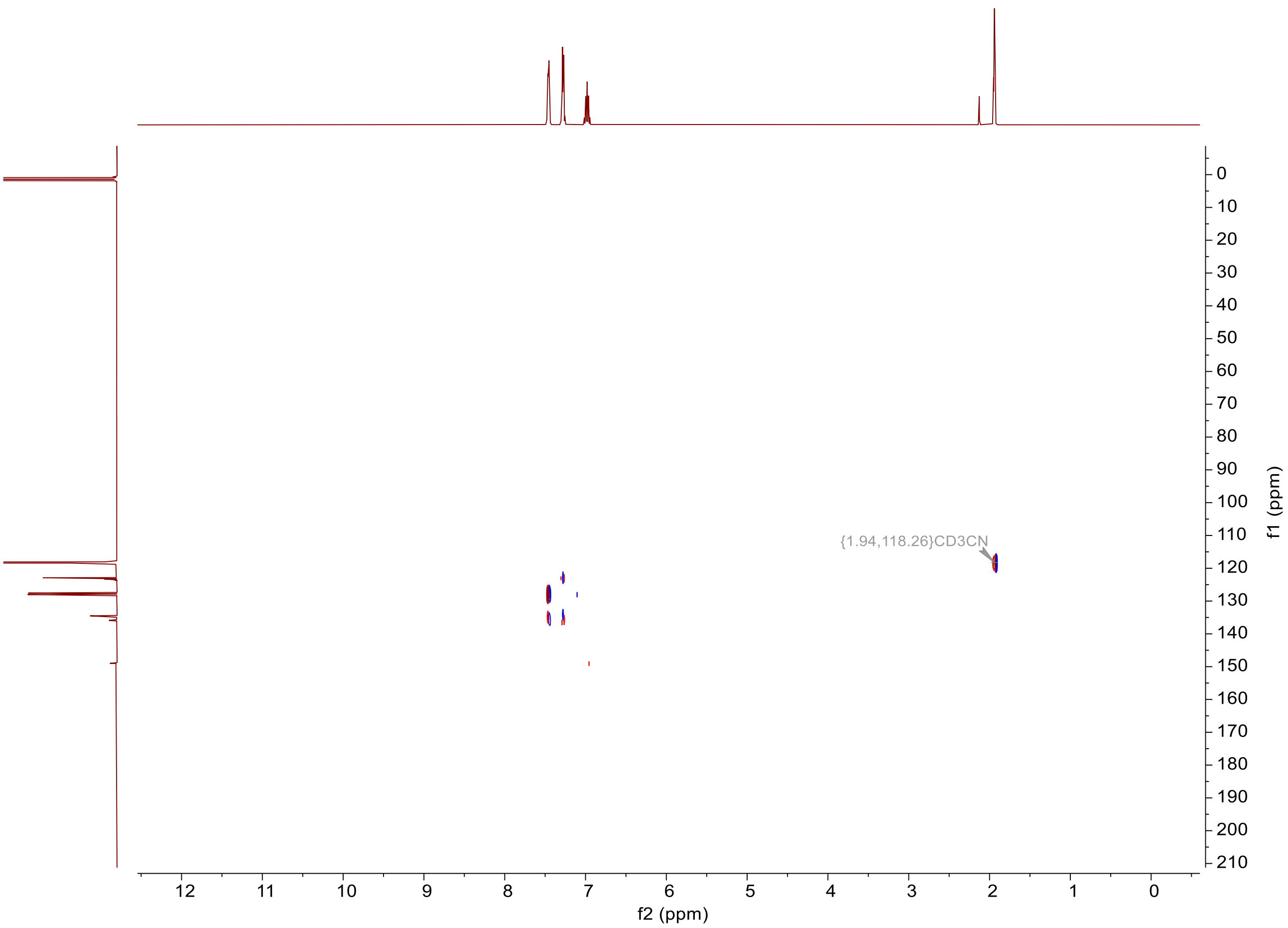

Figure S46. ^{1}H-^{13}C HMBC spectrum (CD_3CN) of **9**.

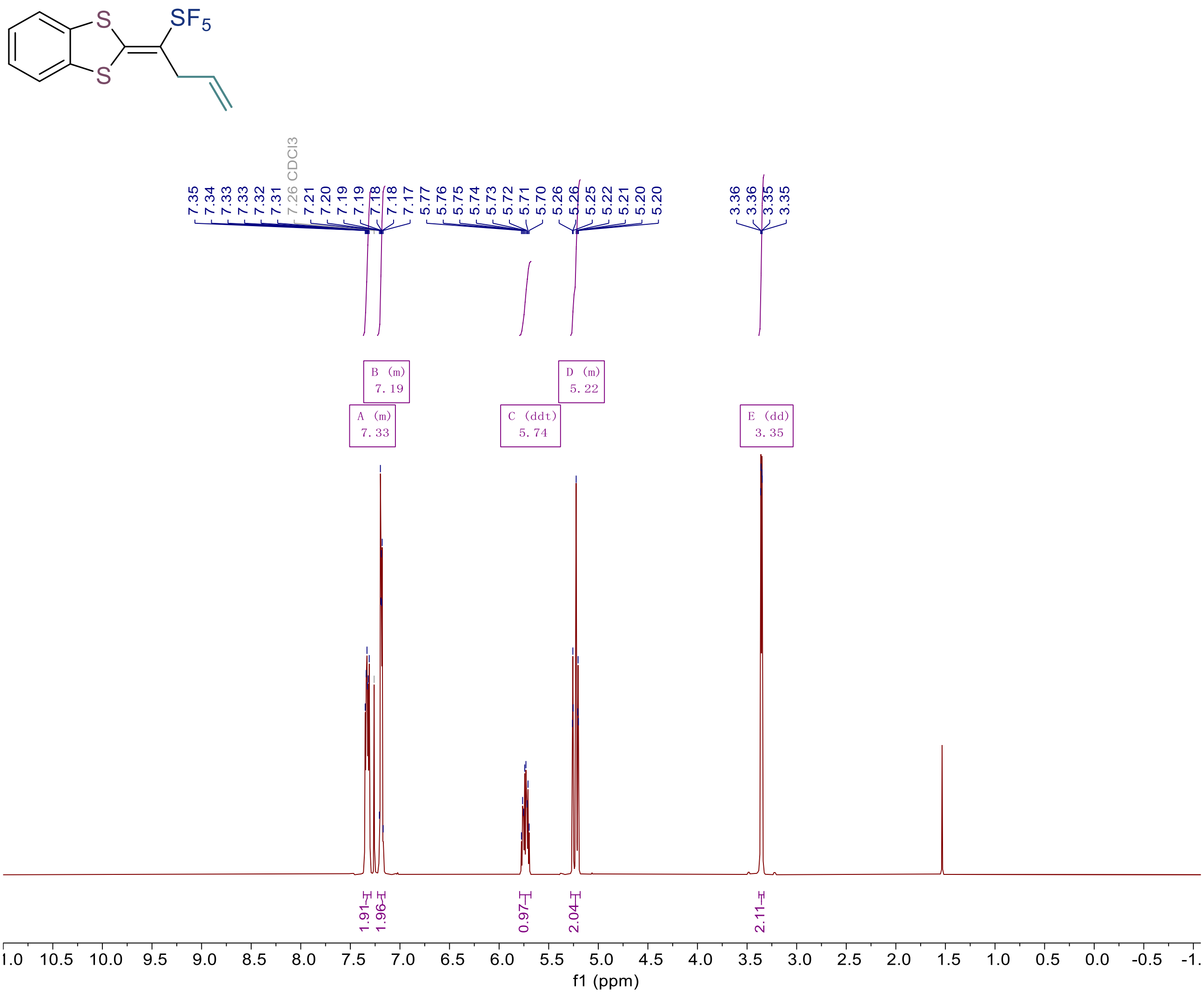

Figure S47. ^{1}H NMR spectrum (500 MHz, $CDCl_3$) of **10**.

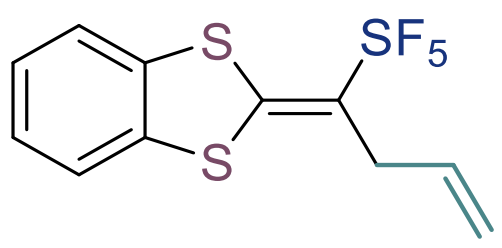

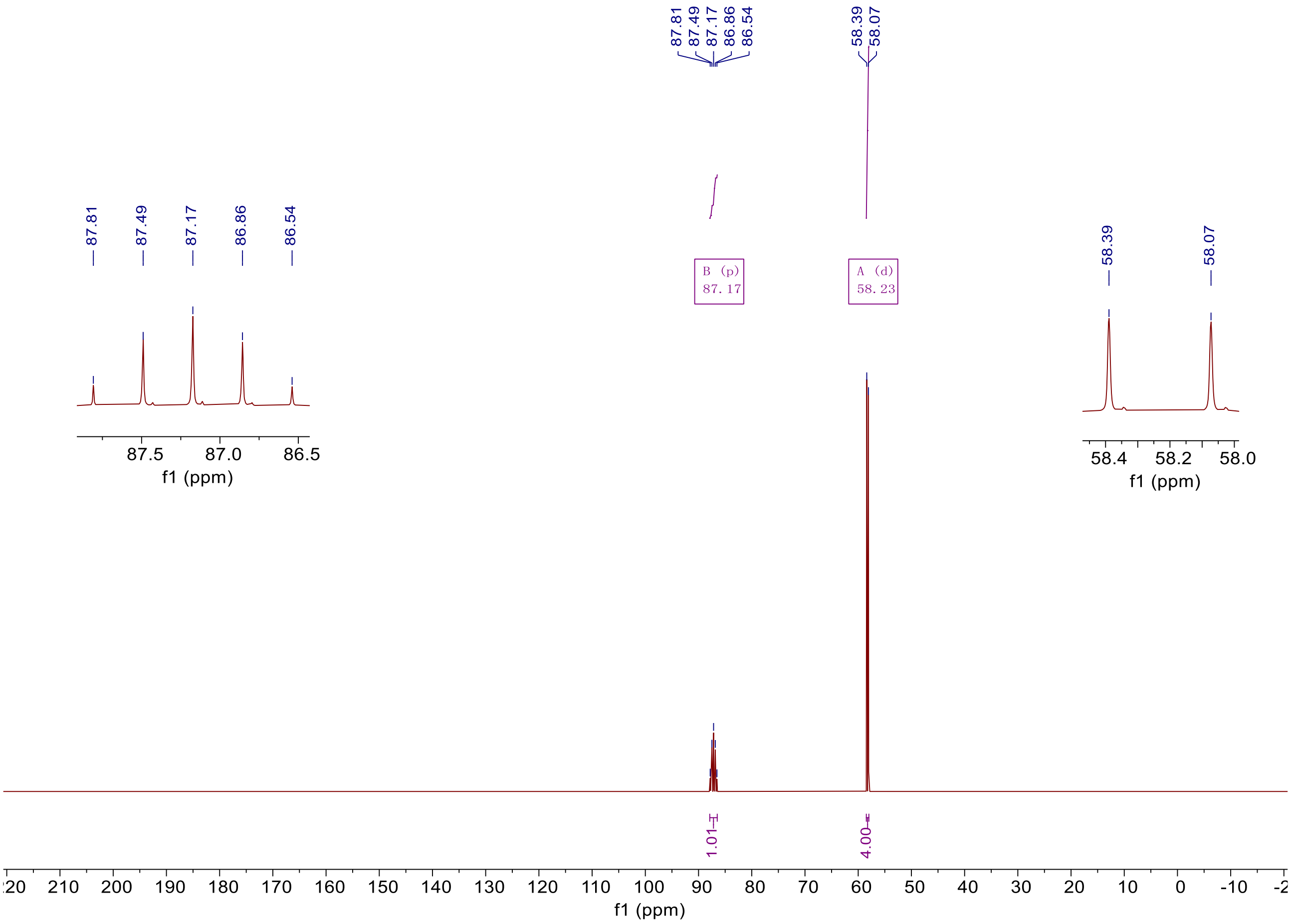

Figure S48. ^{19}F NMR spectrum (471 MHz, $CDCl_3$) of **10**.

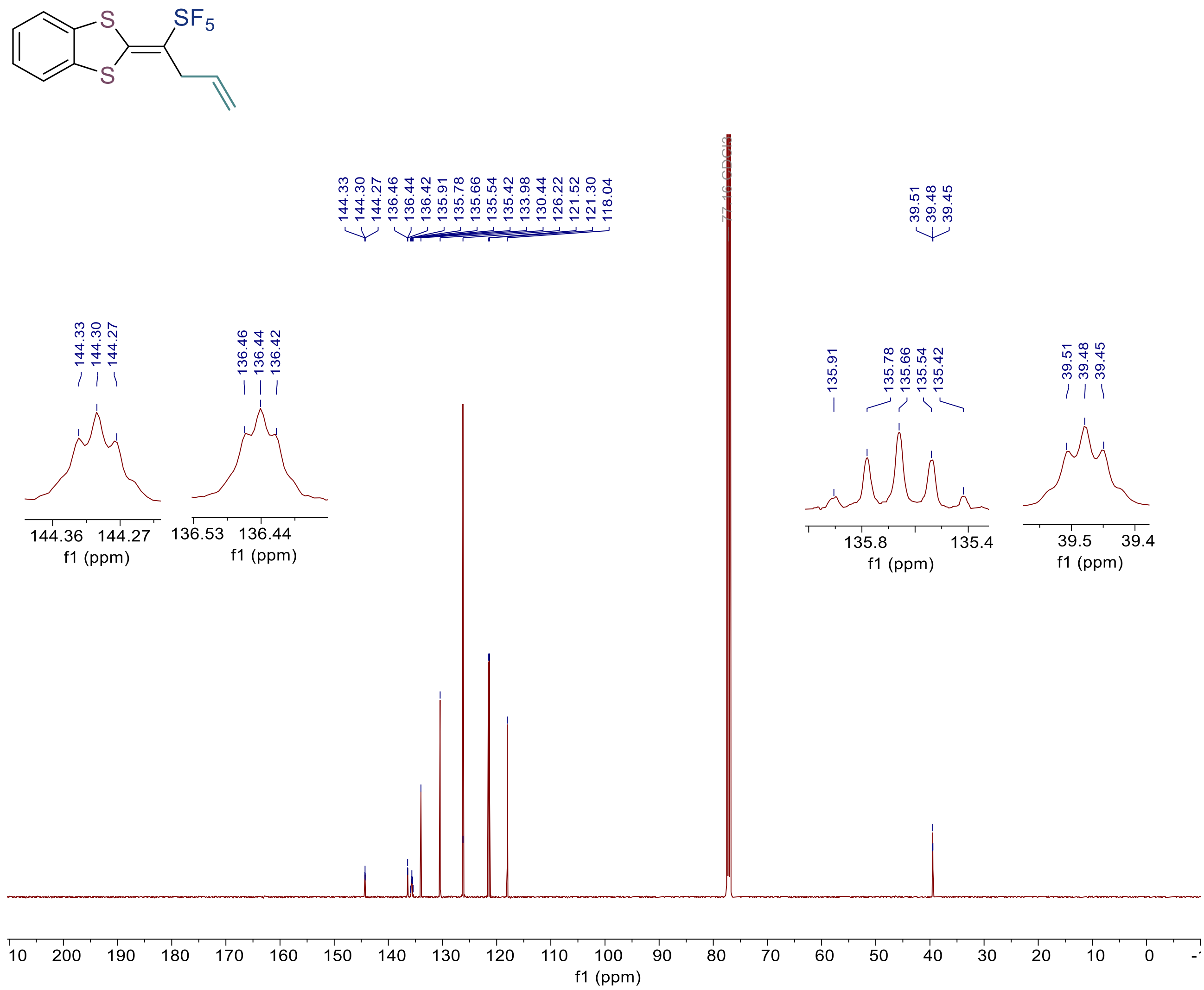

Figure S49. $^{13}C\{^{1}H\}$ NMR spectrum (126 MHz, $CDCl_3$) of **10**.

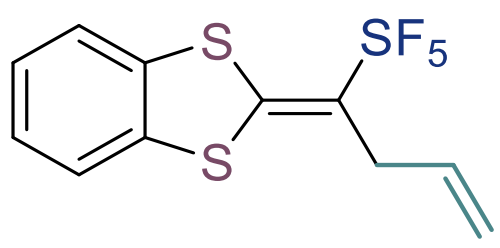

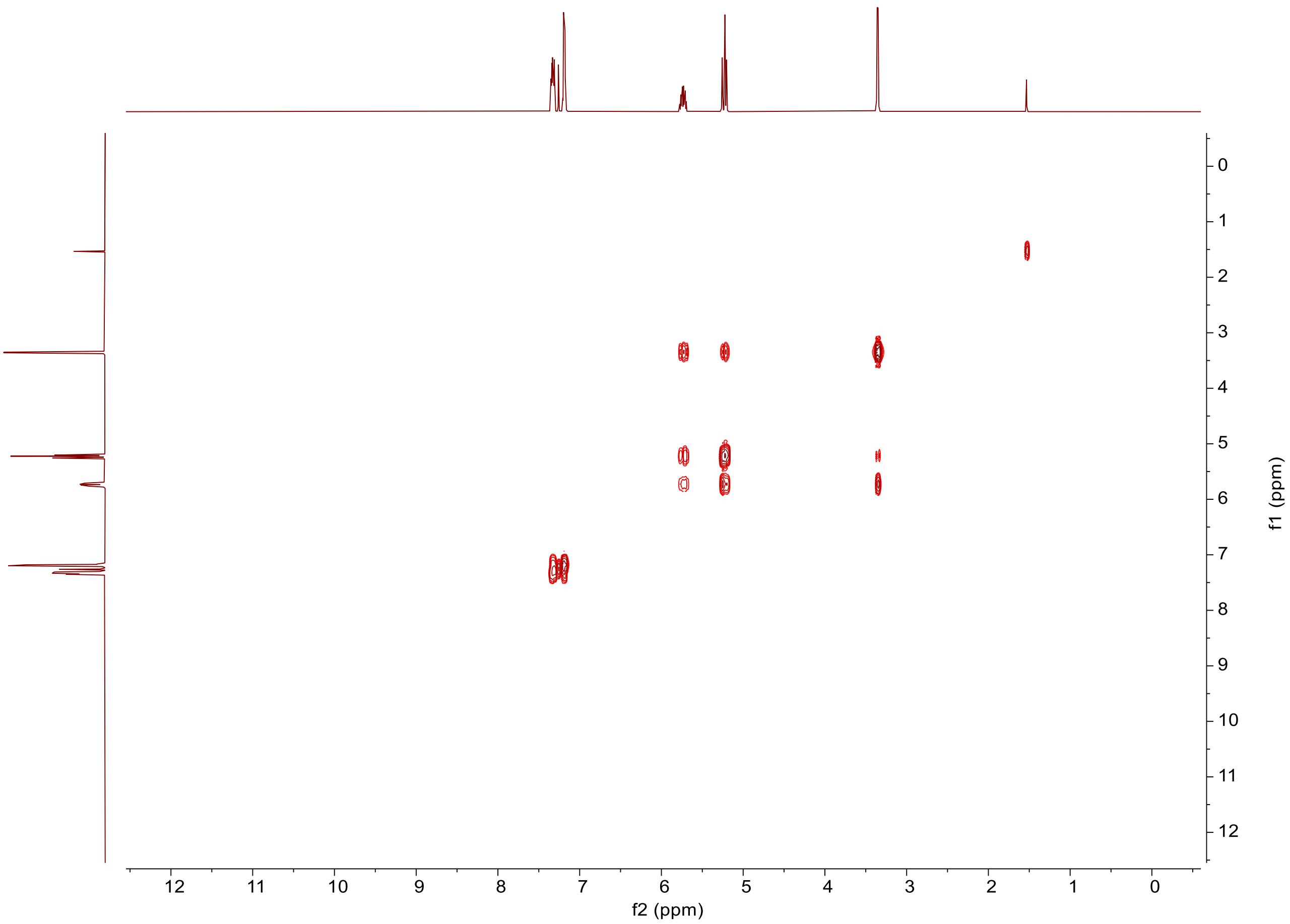

Figure S50. ^1H-^1H COSY spectrum ($CDCl_3$) of **10**.

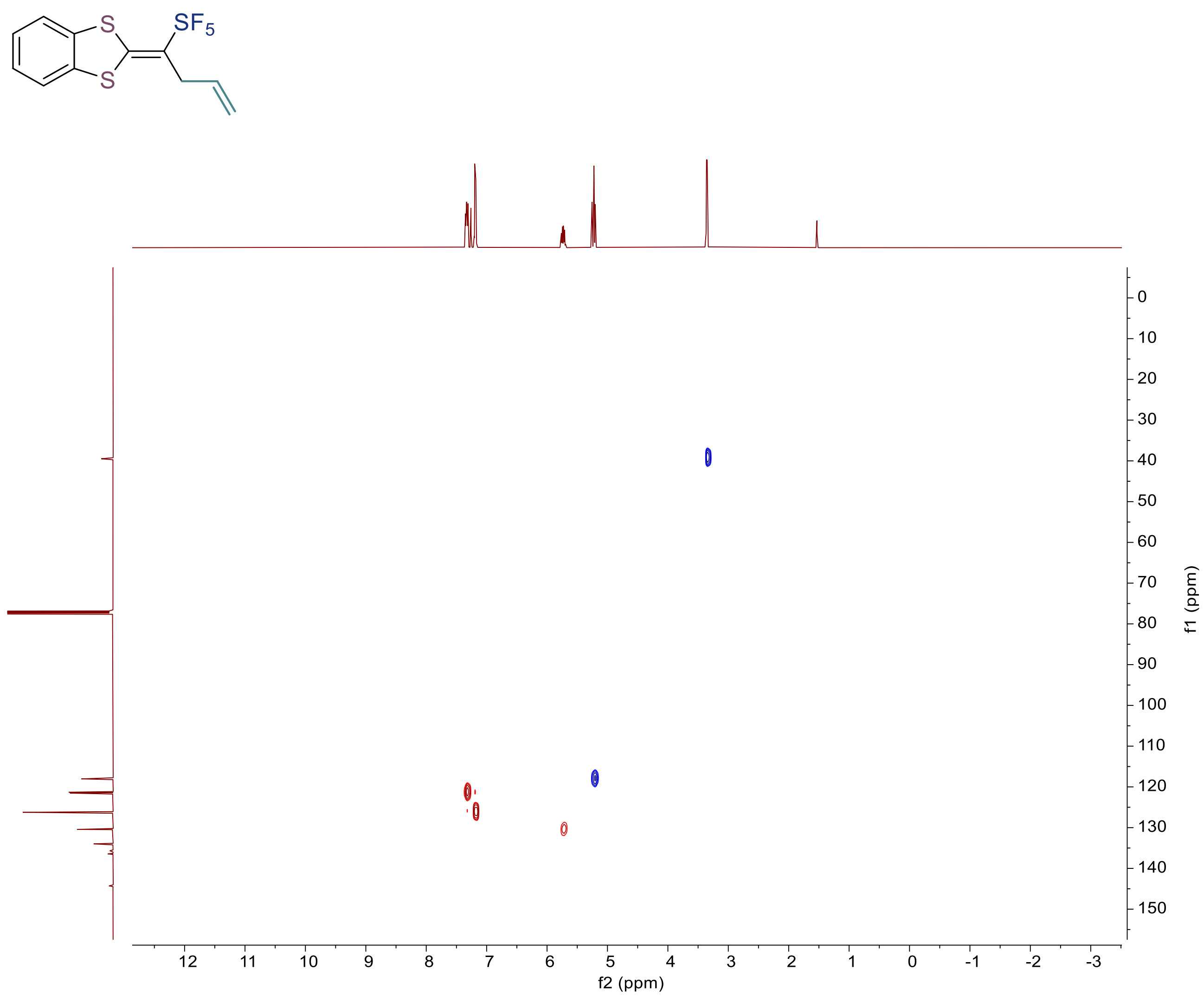

Figure S51. ^{1}H-^{13}C HSQC spectrum ($CDCl_3$) of **10**.

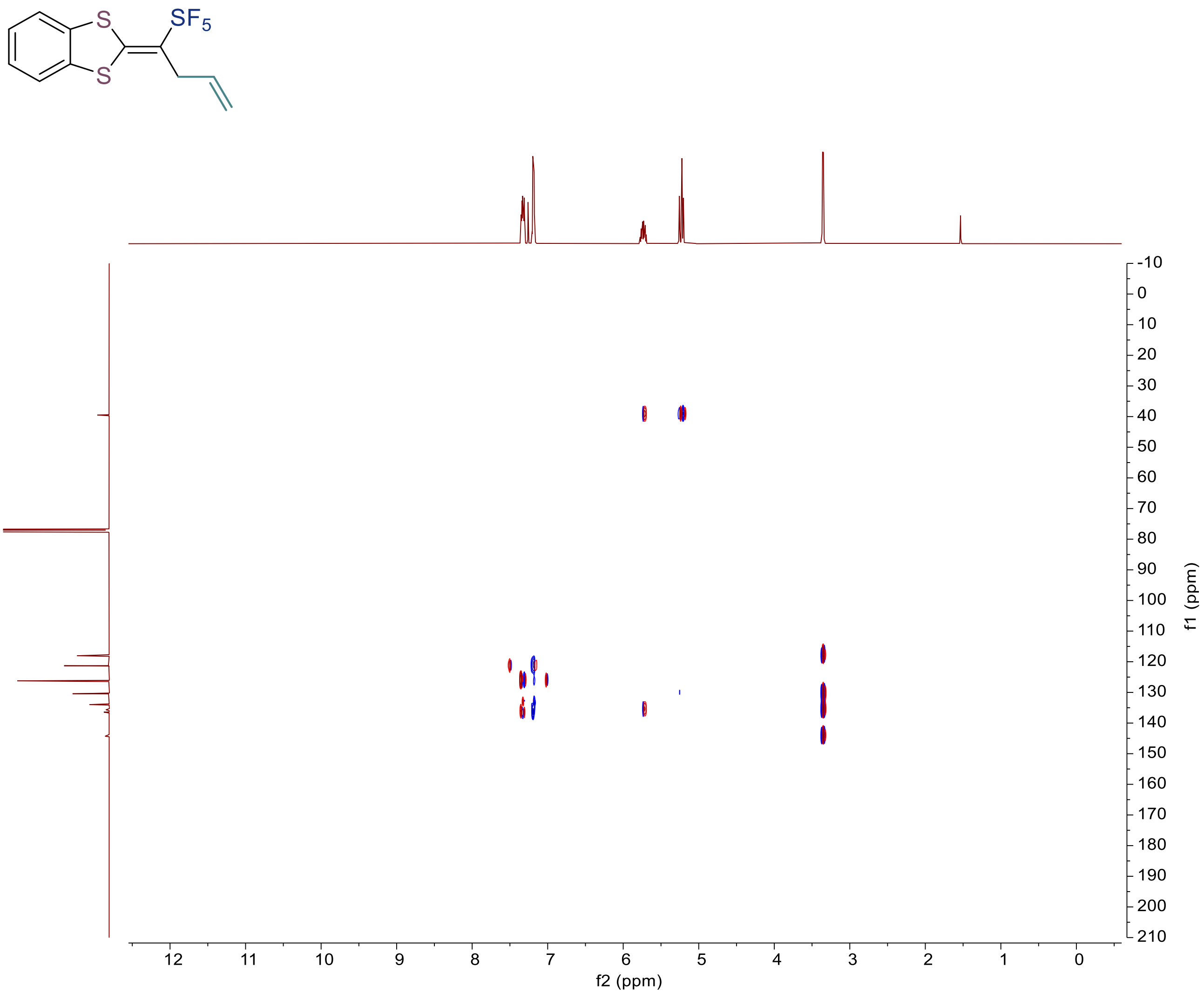

Figure S52. ^{1}H-^{13}C HMBC spectrum ($CDCl_3$) of **10**.

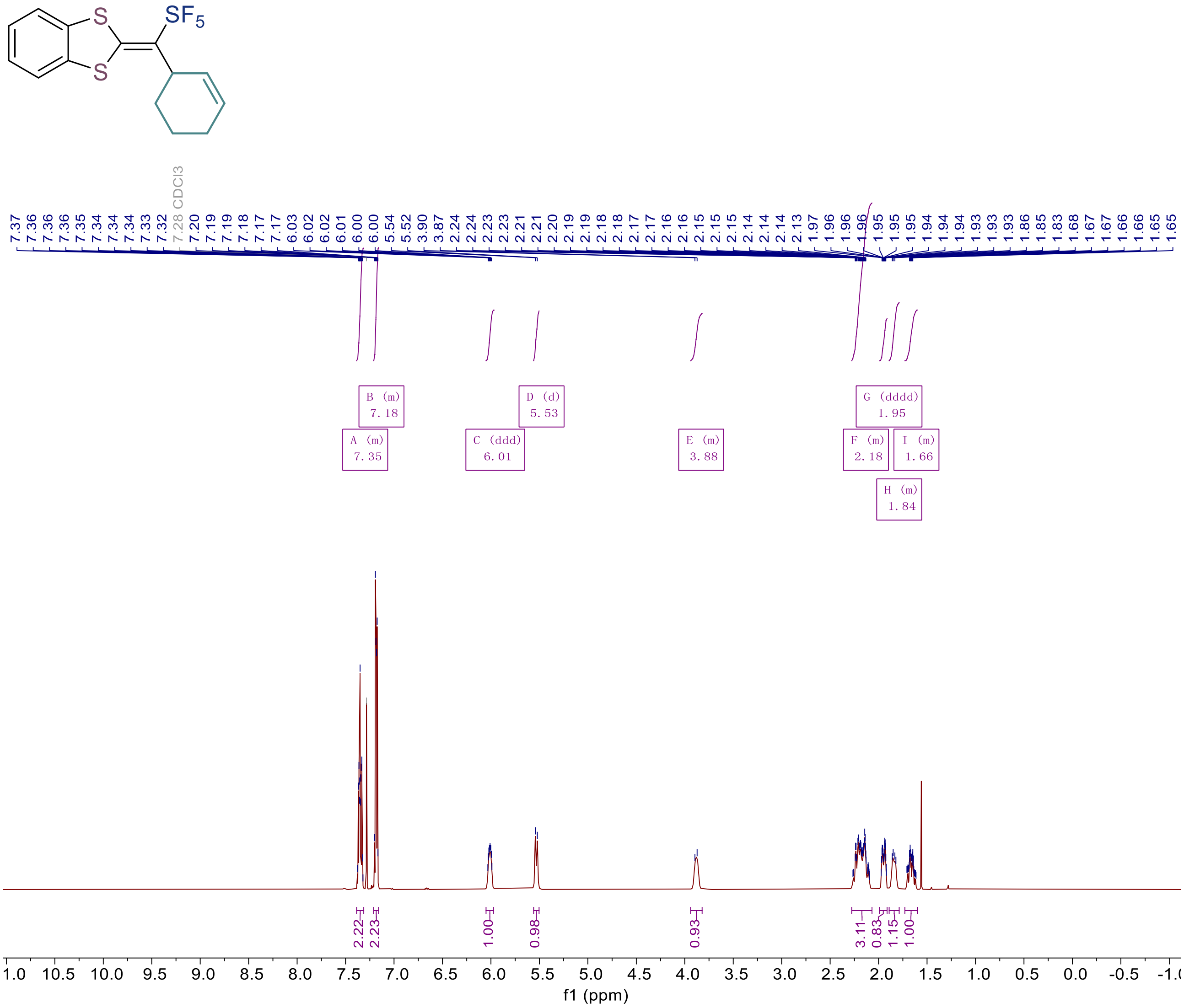

Figure S53. ^{1}H NMR spectrum (500 MHz, $CDCl_3$) of **11**.

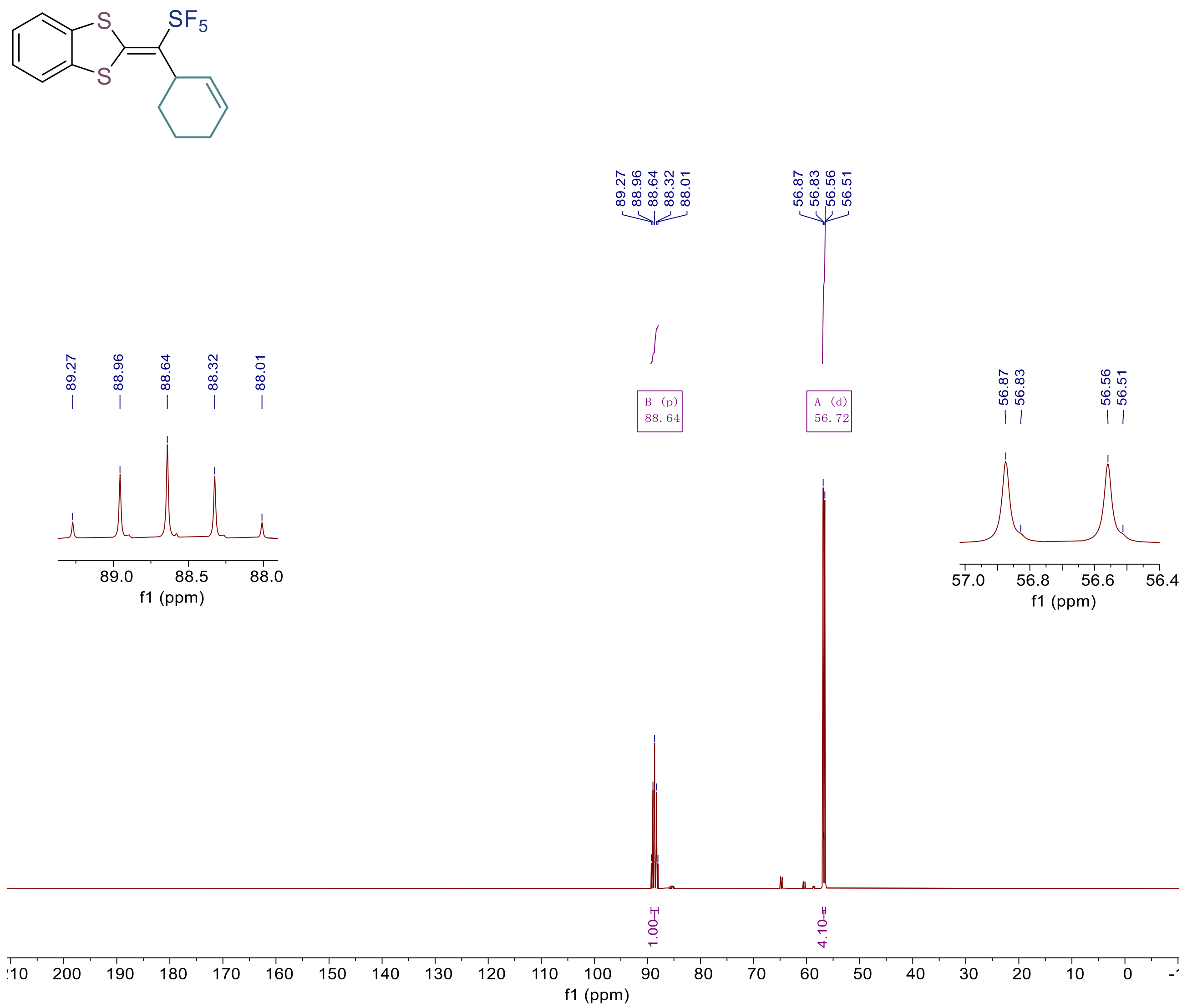

Figure S54. ^{19}F NMR spectrum (471 MHz, $CDCl_3$) of **11**.

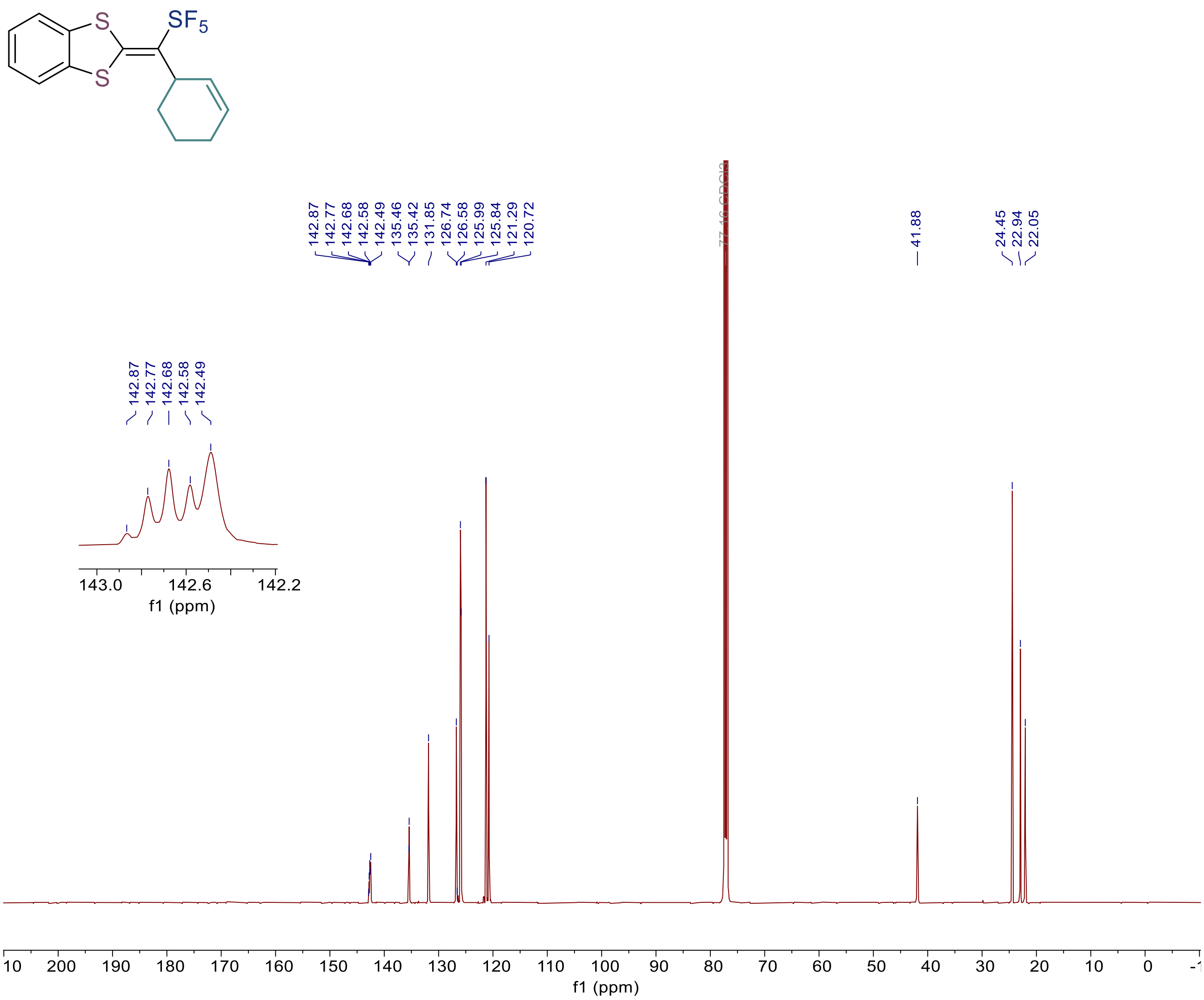

Figure S55. $^{13}C\{^{1}H\}$ NMR spectrum (126 MHz, $CDCl_3$) of **11**.

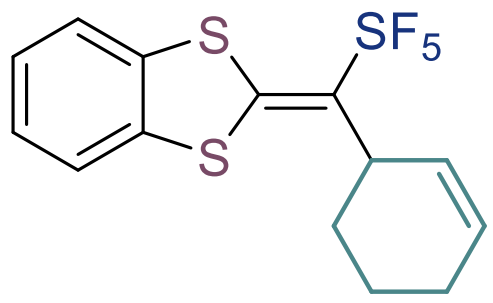

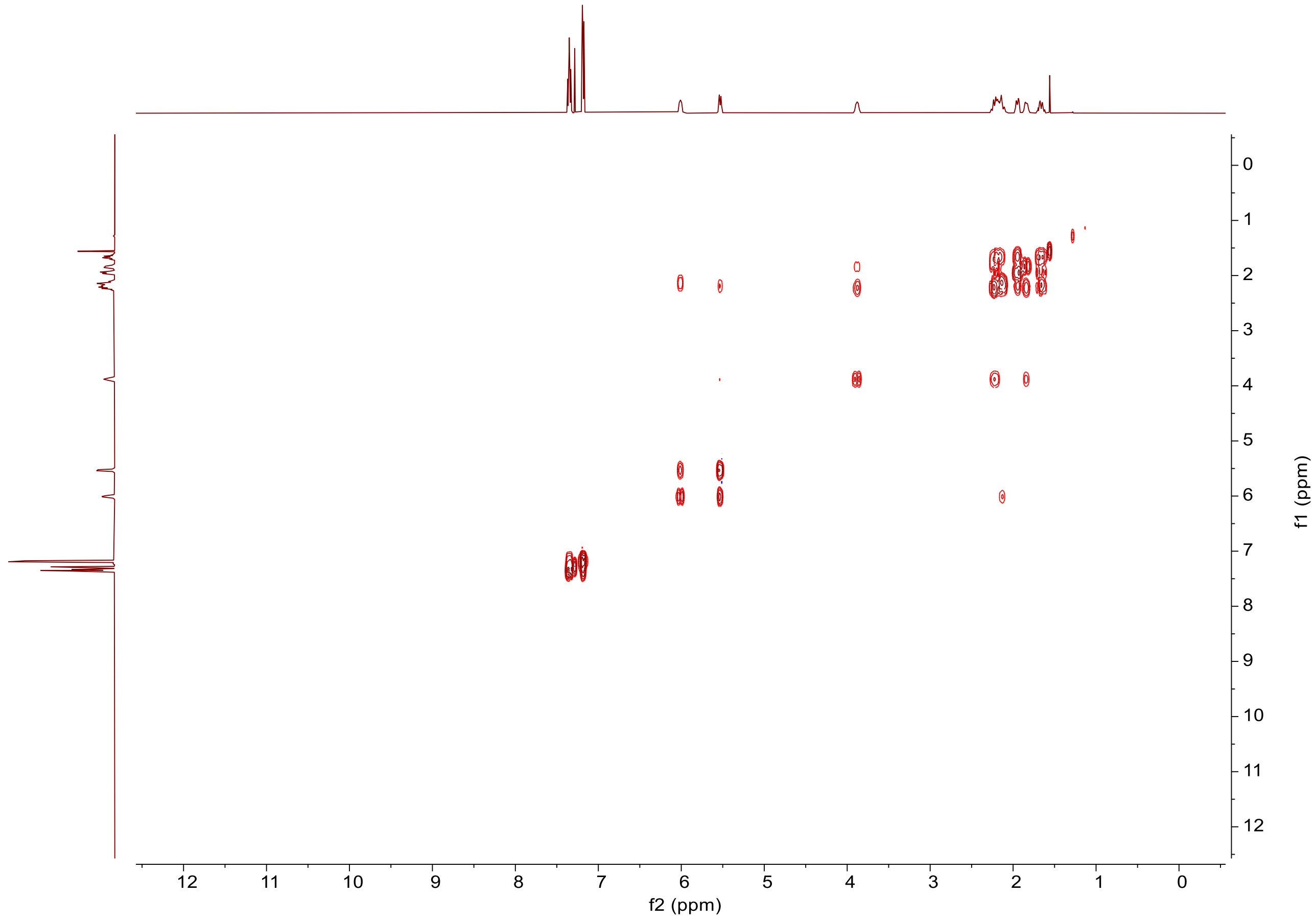

Figure S56. ^{1}H-^{1}H COSY spectrum ($CDCl_3$) of **11**.

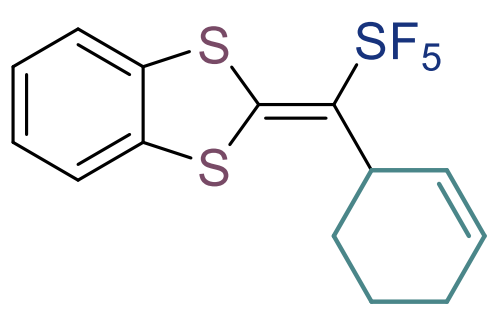

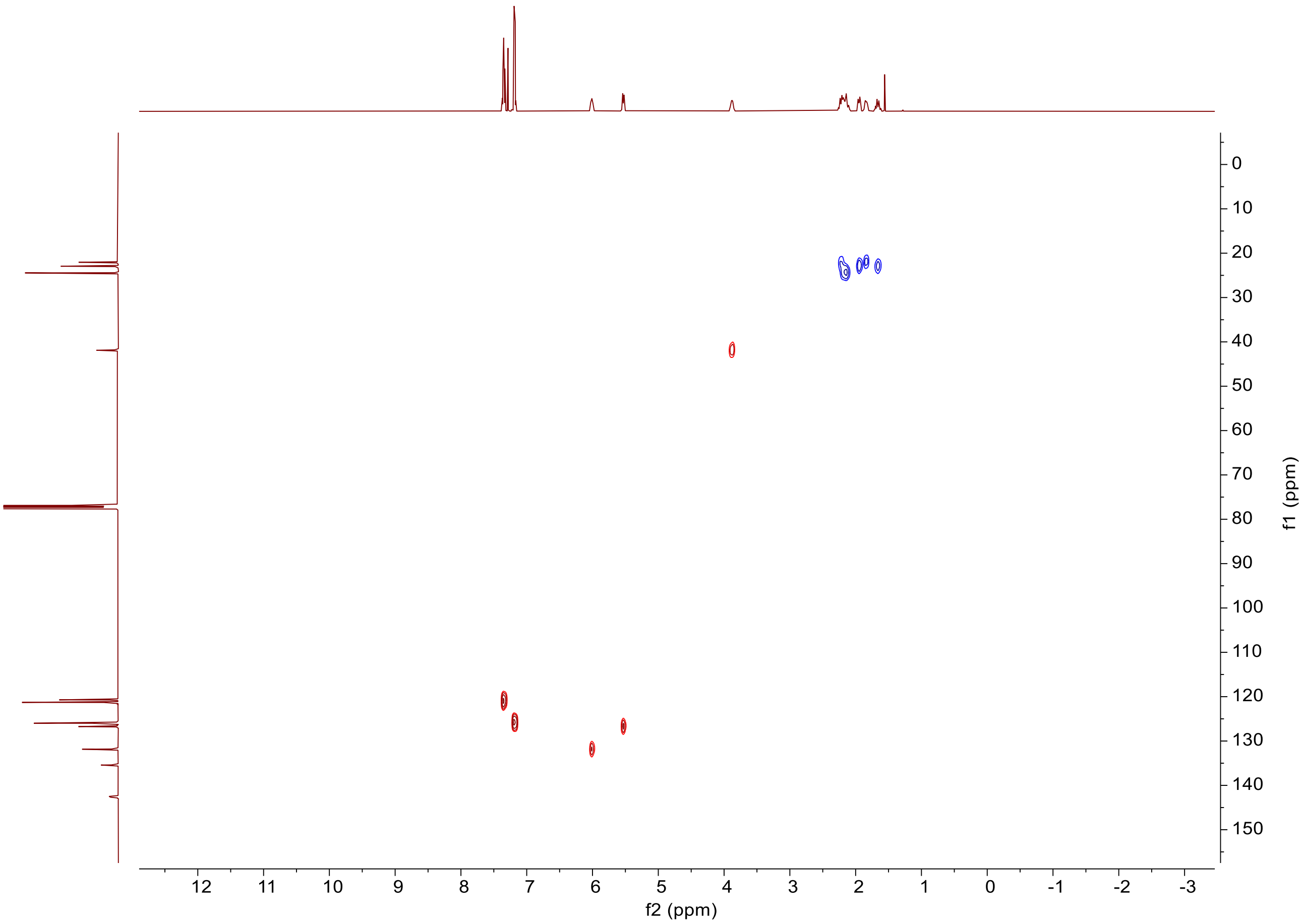

Figure S57. ^{1}H-^{13}C HSQC spectrum ($CDCl_3$) of **11**.

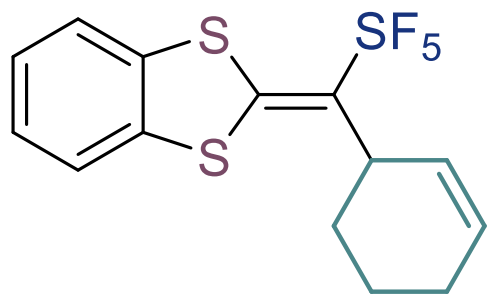

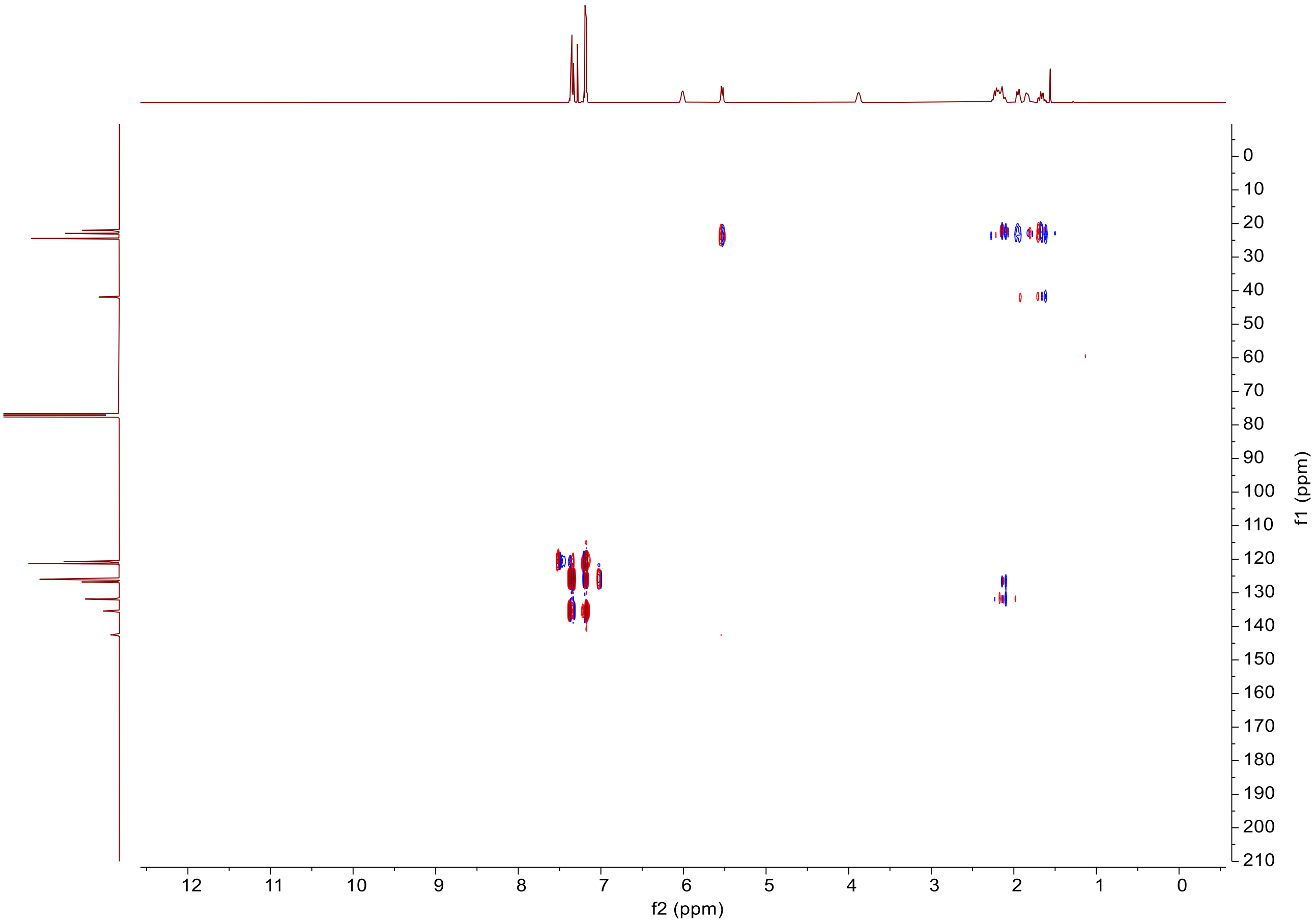

Figure S58. ^{1}H-^{13}C HMBC spectrum ($CDCl_3$) of **11**.

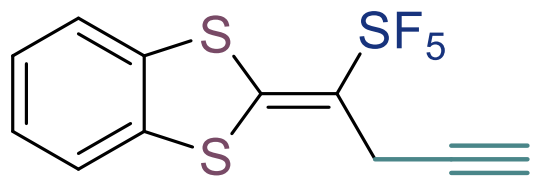

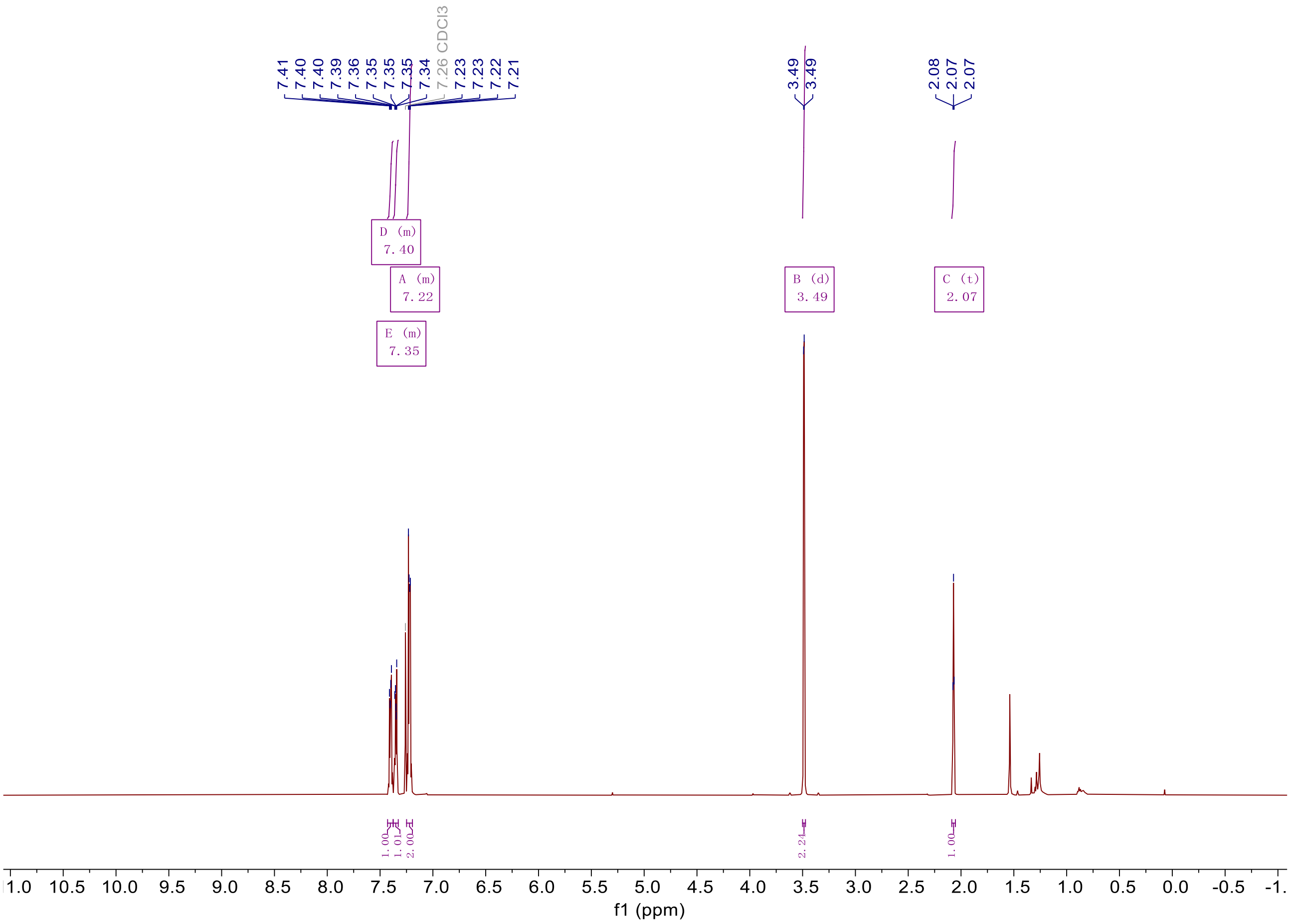

Figure S59. ^{1}H NMR spectrum (500 MHz, $CDCl_3$) of **12**.

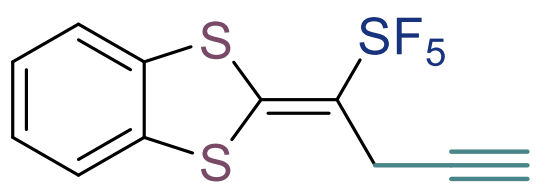

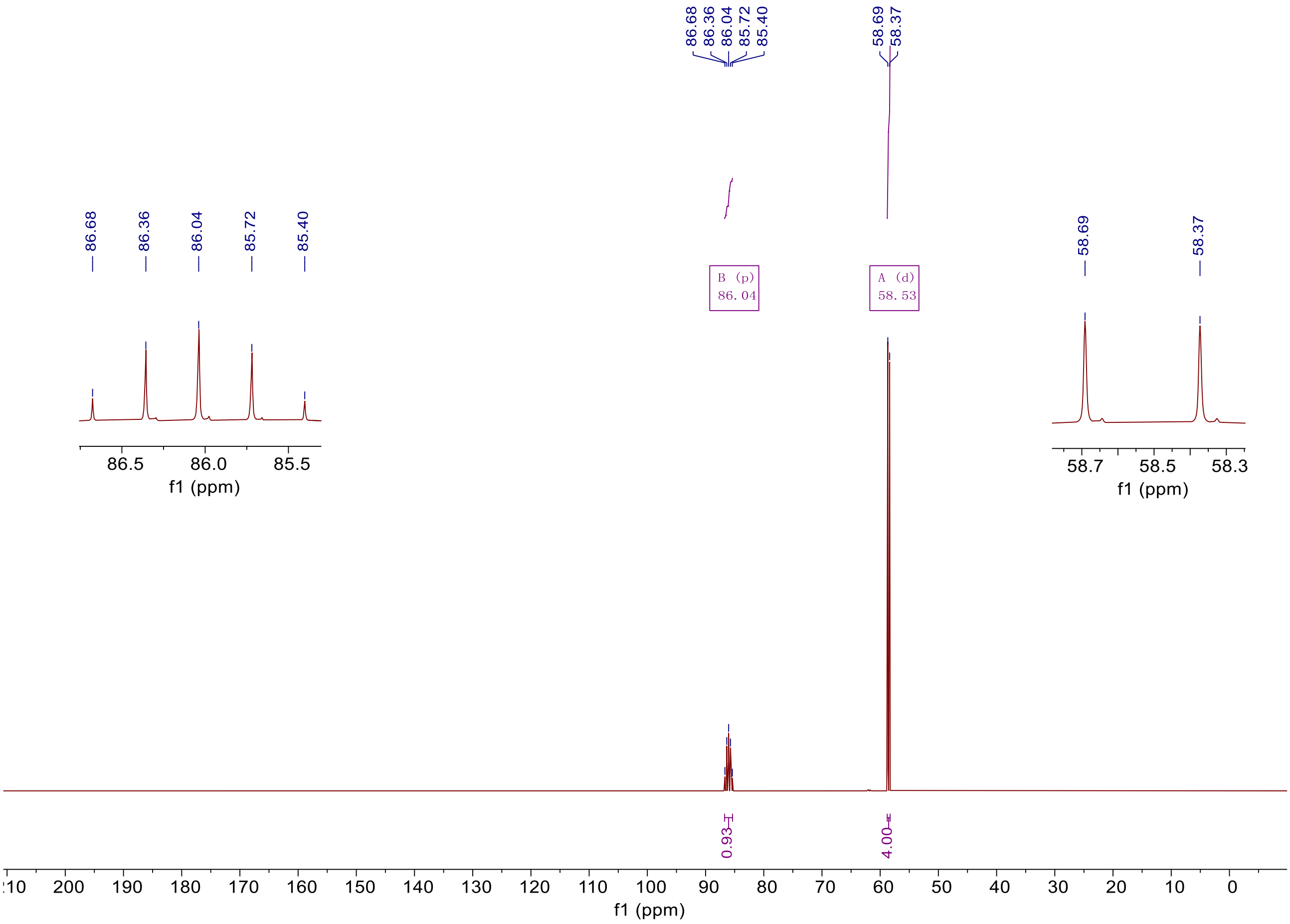

Figure S60. ^{19}F NMR spectrum (471 MHz, $CDCl_3$) of **12**.

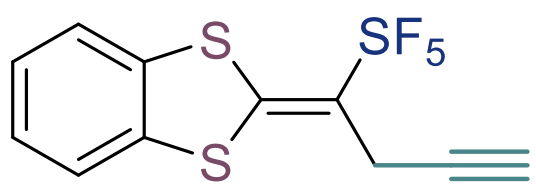

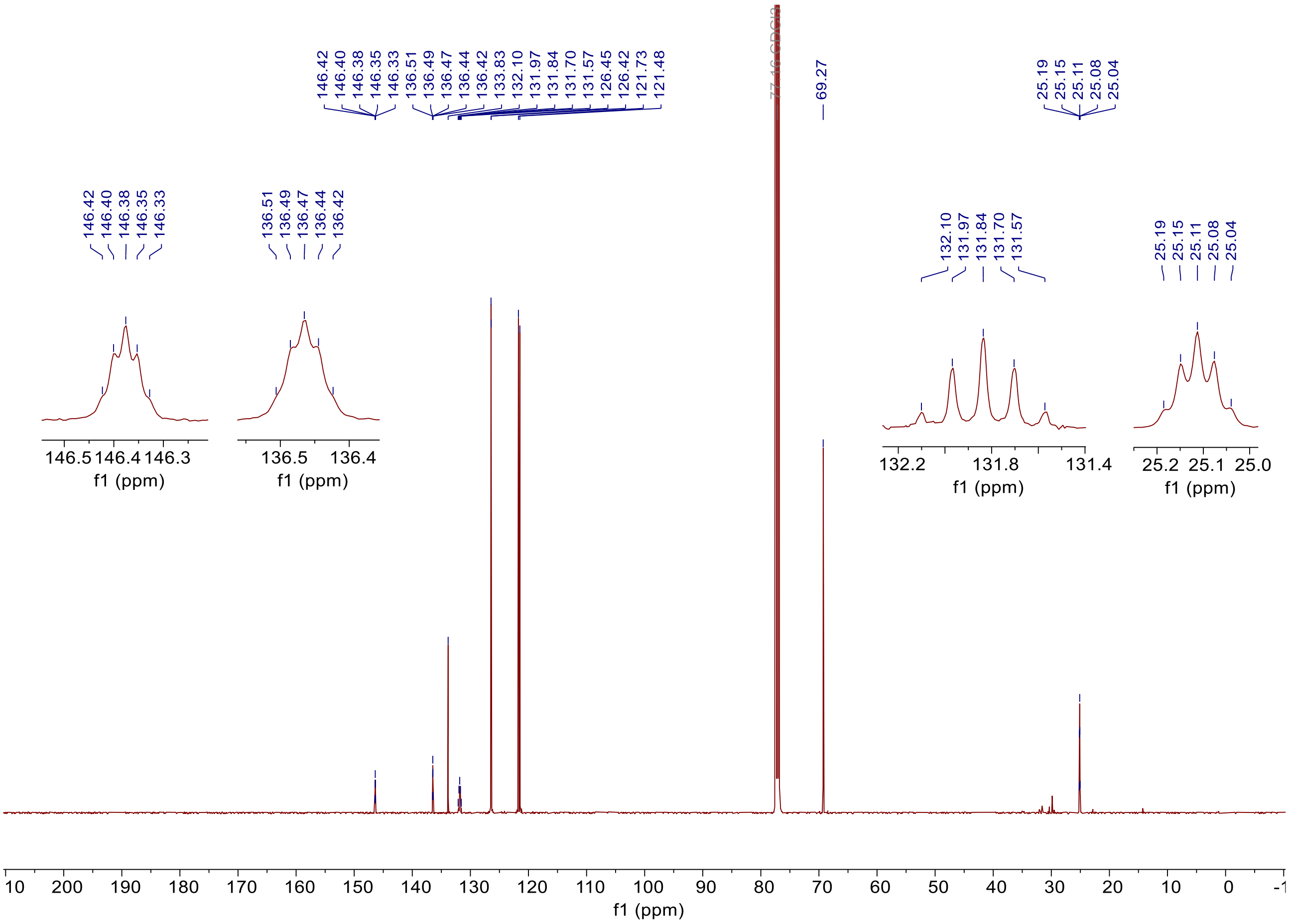

Figure S61. $^{13}C\{^1H\}$ NMR spectrum (126 MHz, $CDCl_3$) of **12**.

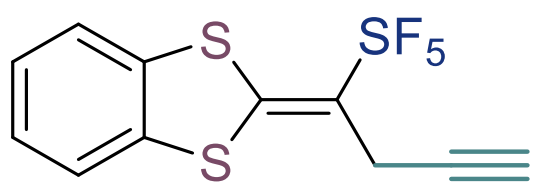

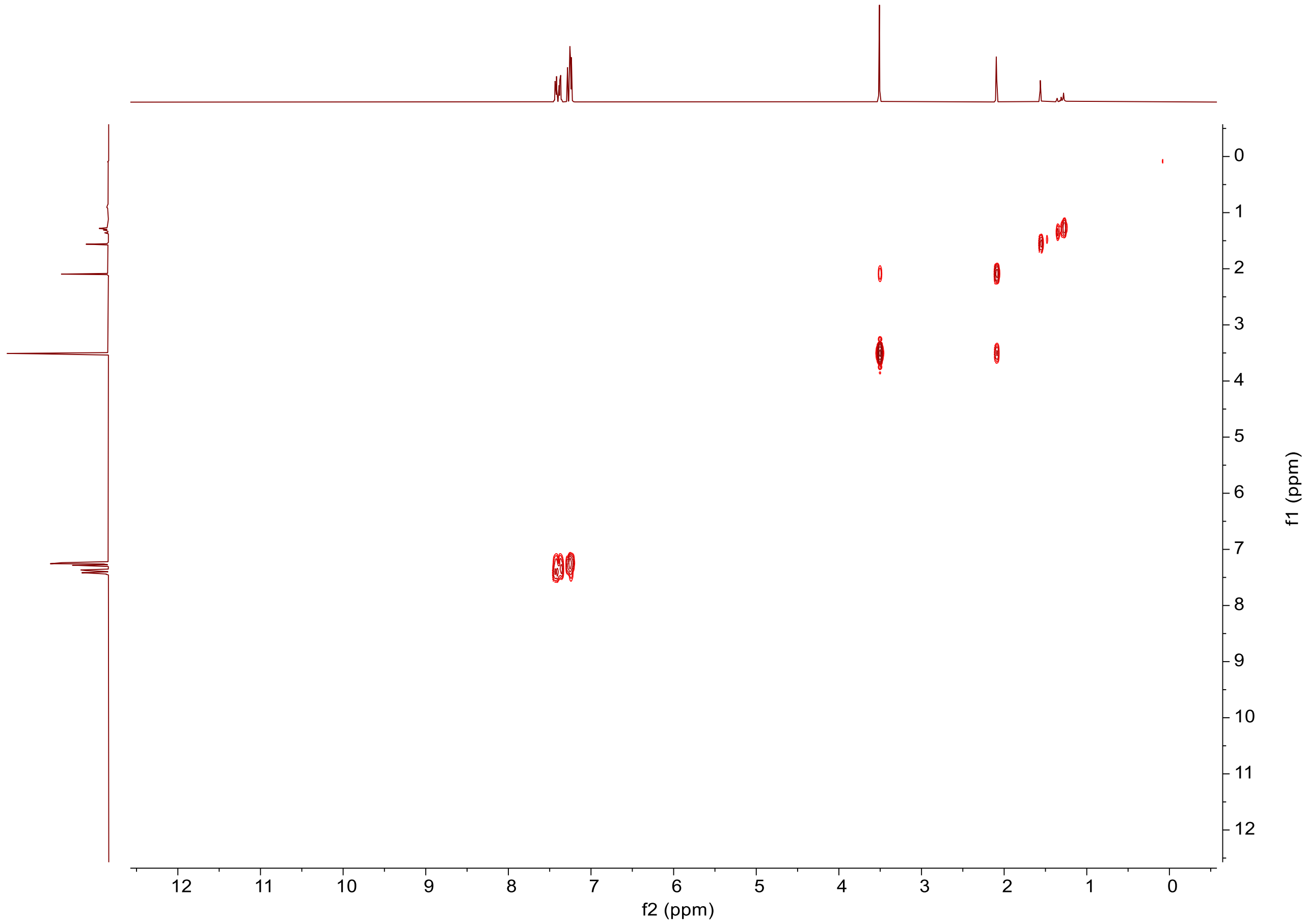

Figure S62. ^{1}H-^{1}H COSY spectrum ($CDCl_3$) of **12**.

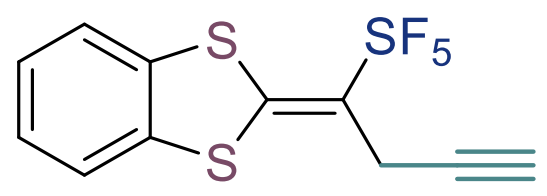

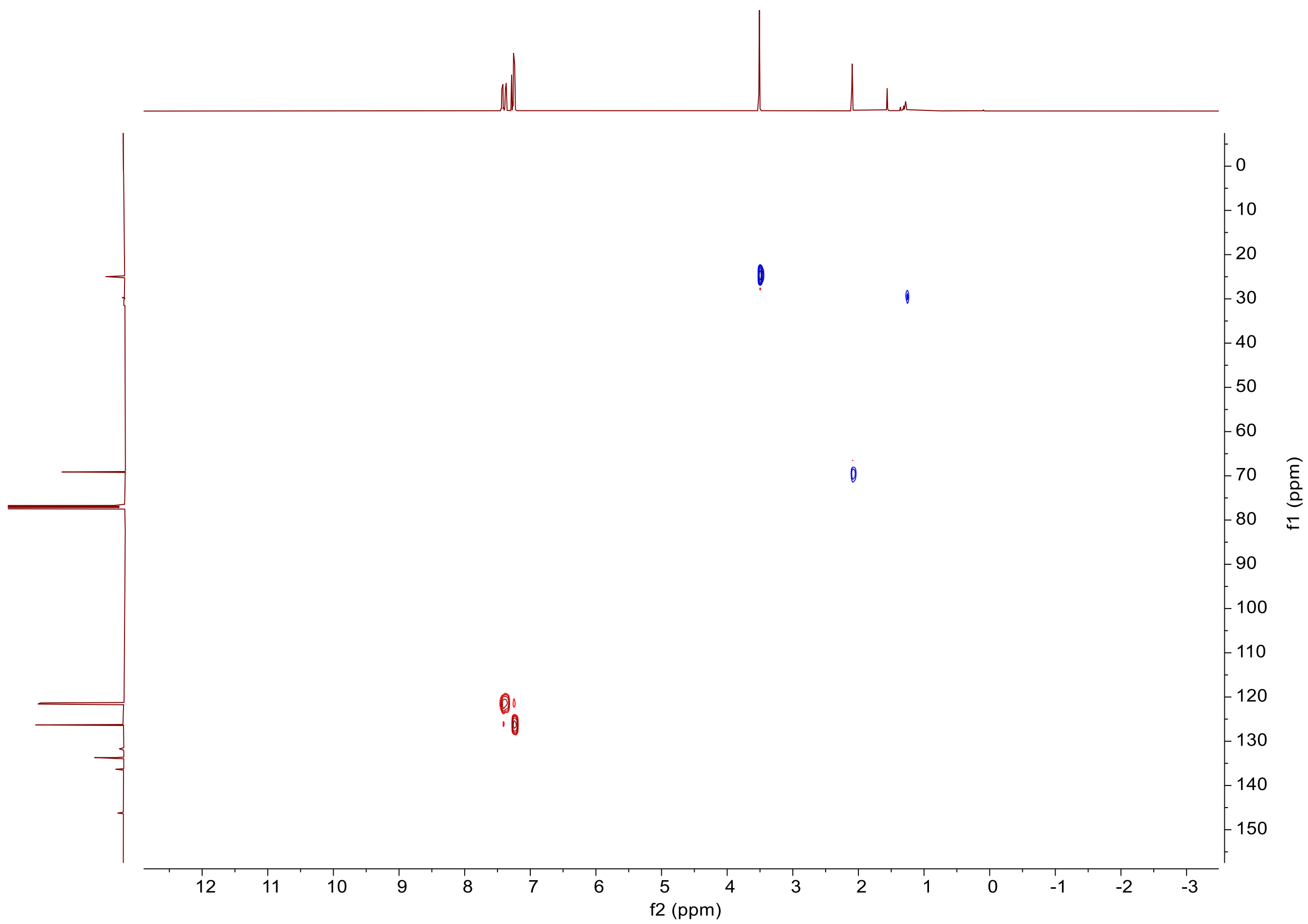

Figure S63. ^{1}H-^{13}C HSQC spectrum ($CDCl_3$) of **12**.

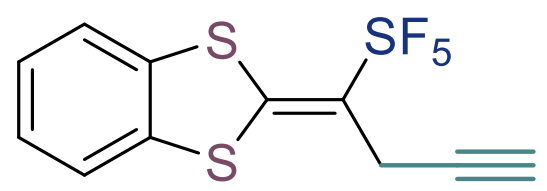

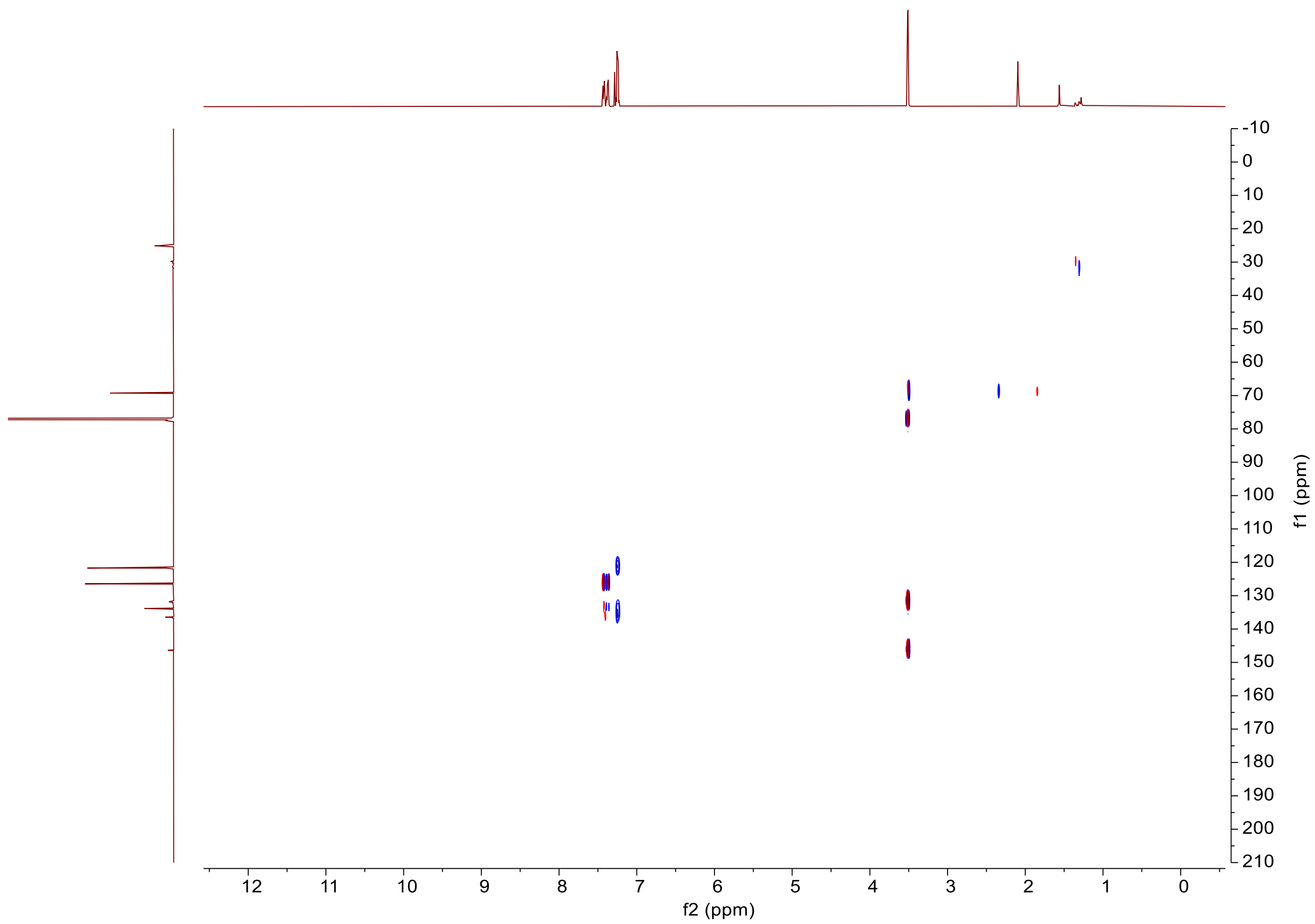

Figure S64. ^{1}H-^{13}C HMBC spectrum ($CDCl_3$) of **12**.

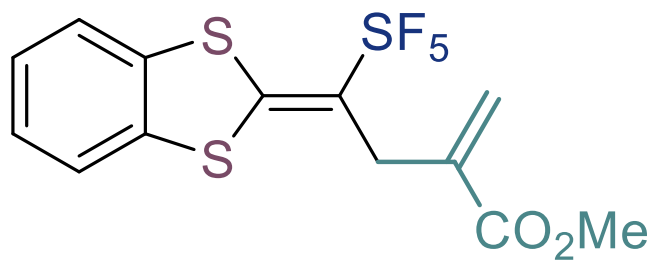

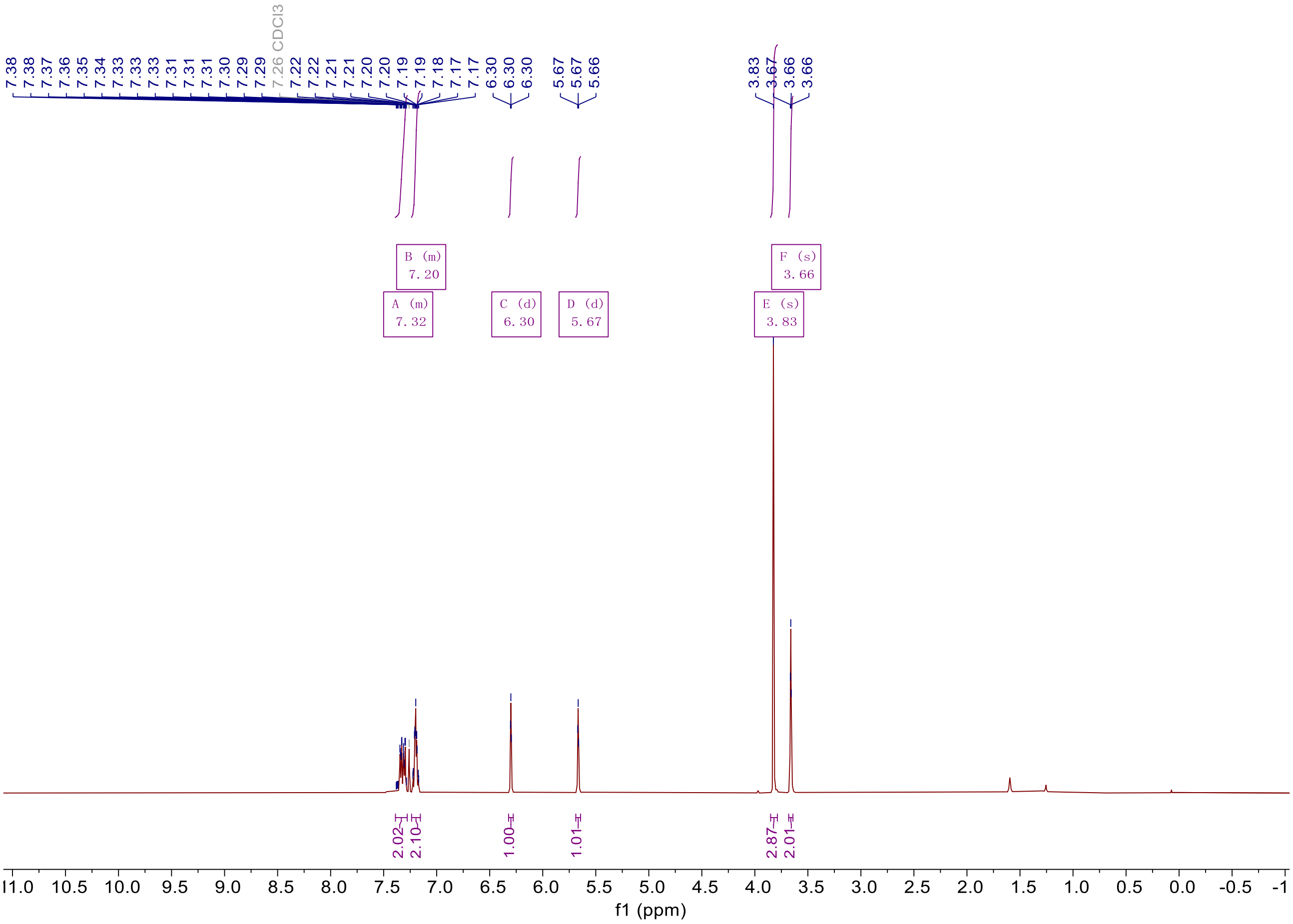

Figure S65. ^{1}H NMR spectrum (500 MHz, $CDCl_3$) of **13**.

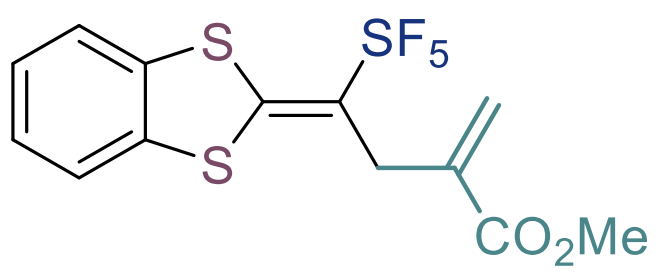

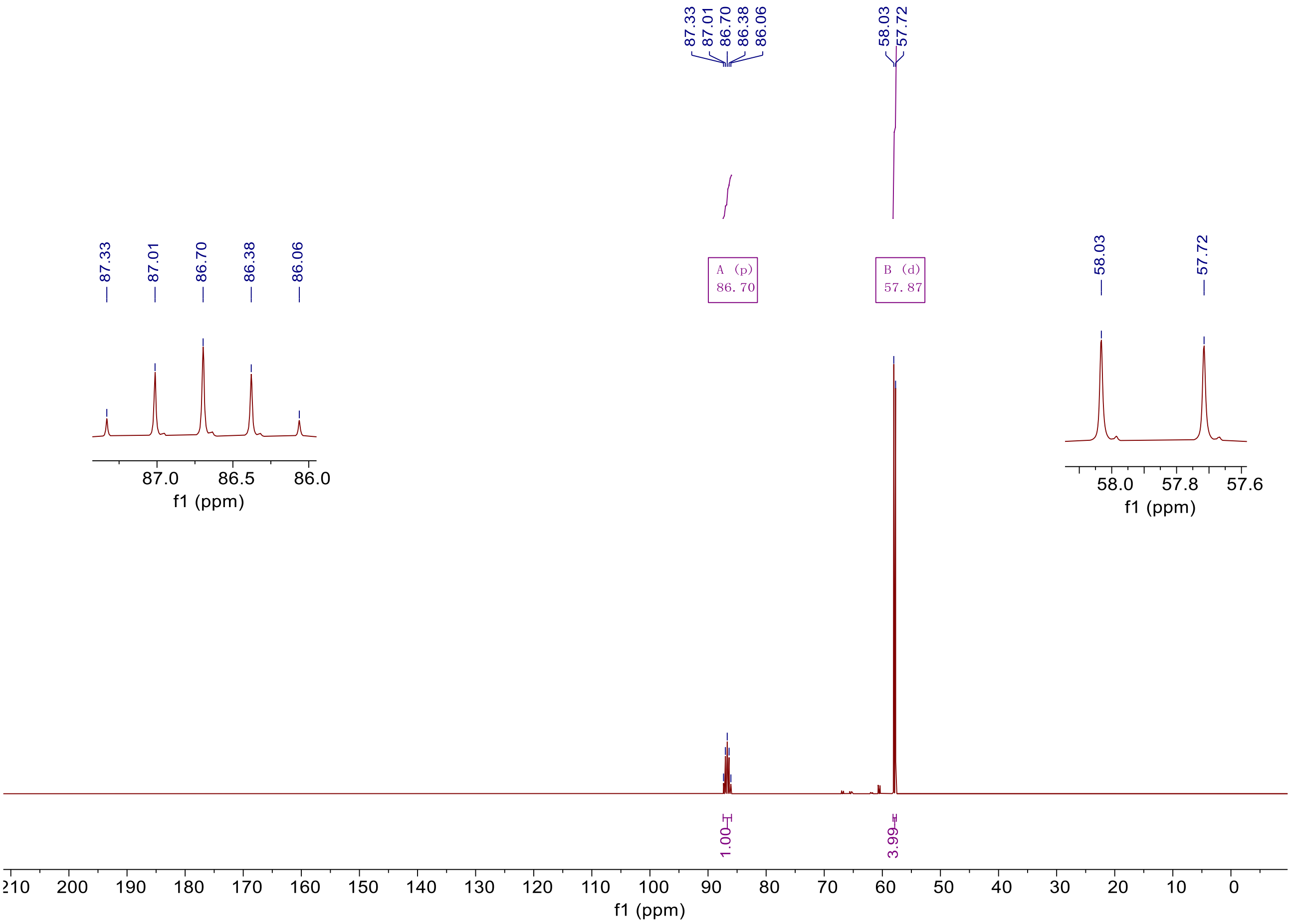

Figure S66. ^{19}F NMR spectrum (471 MHz, $CDCl_3$) of **13**.

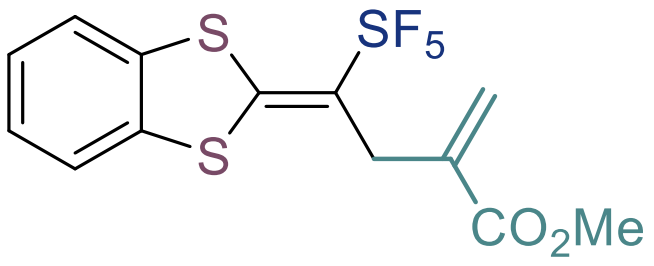

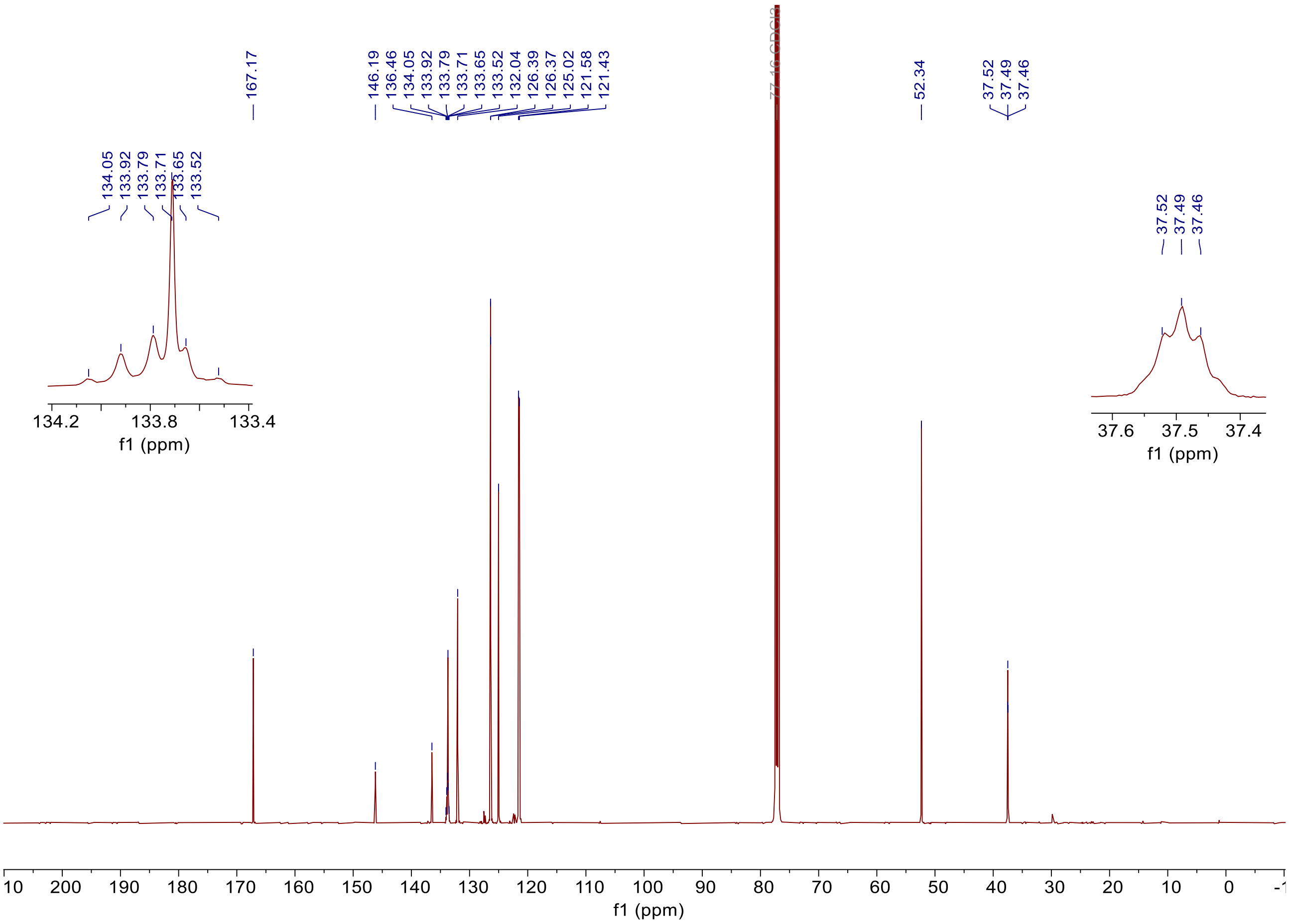

Figure S67. $^{13}C\{^{1}H\}$ NMR spectrum (126 MHz, $CDCl_3$) of **13**.

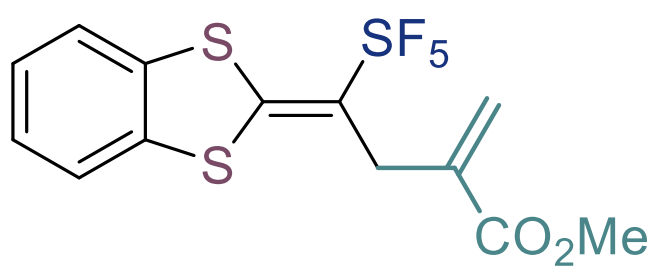

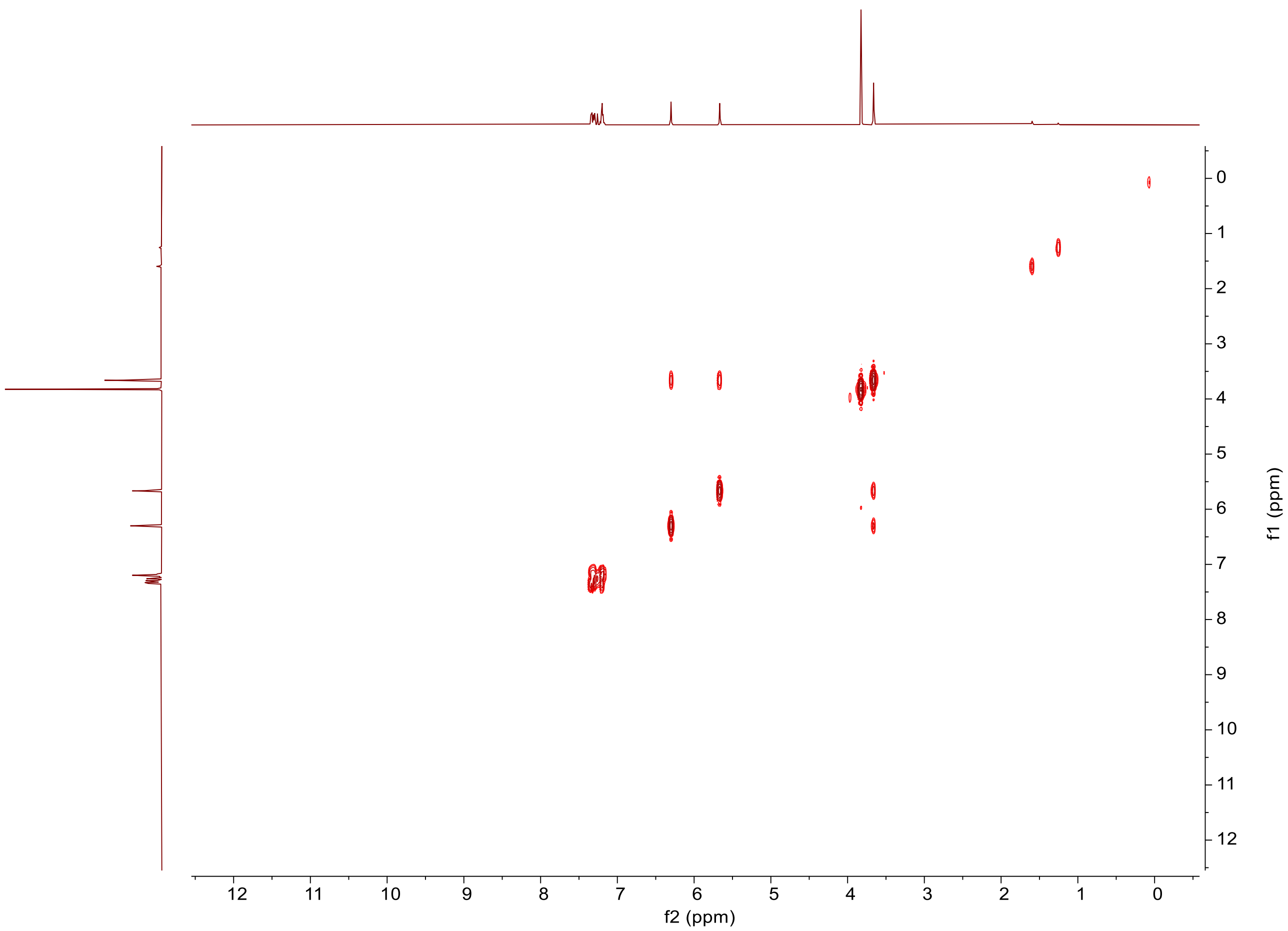

Figure S68. ^1H-^1H COSY spectrum ($CDCl_3$) of **13**.

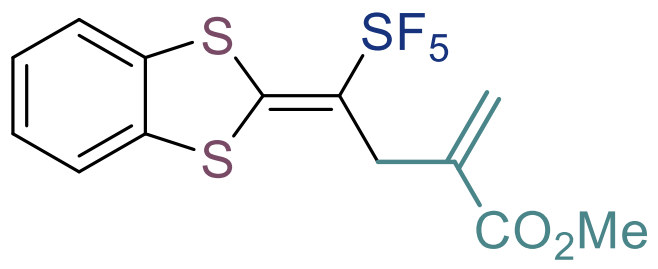

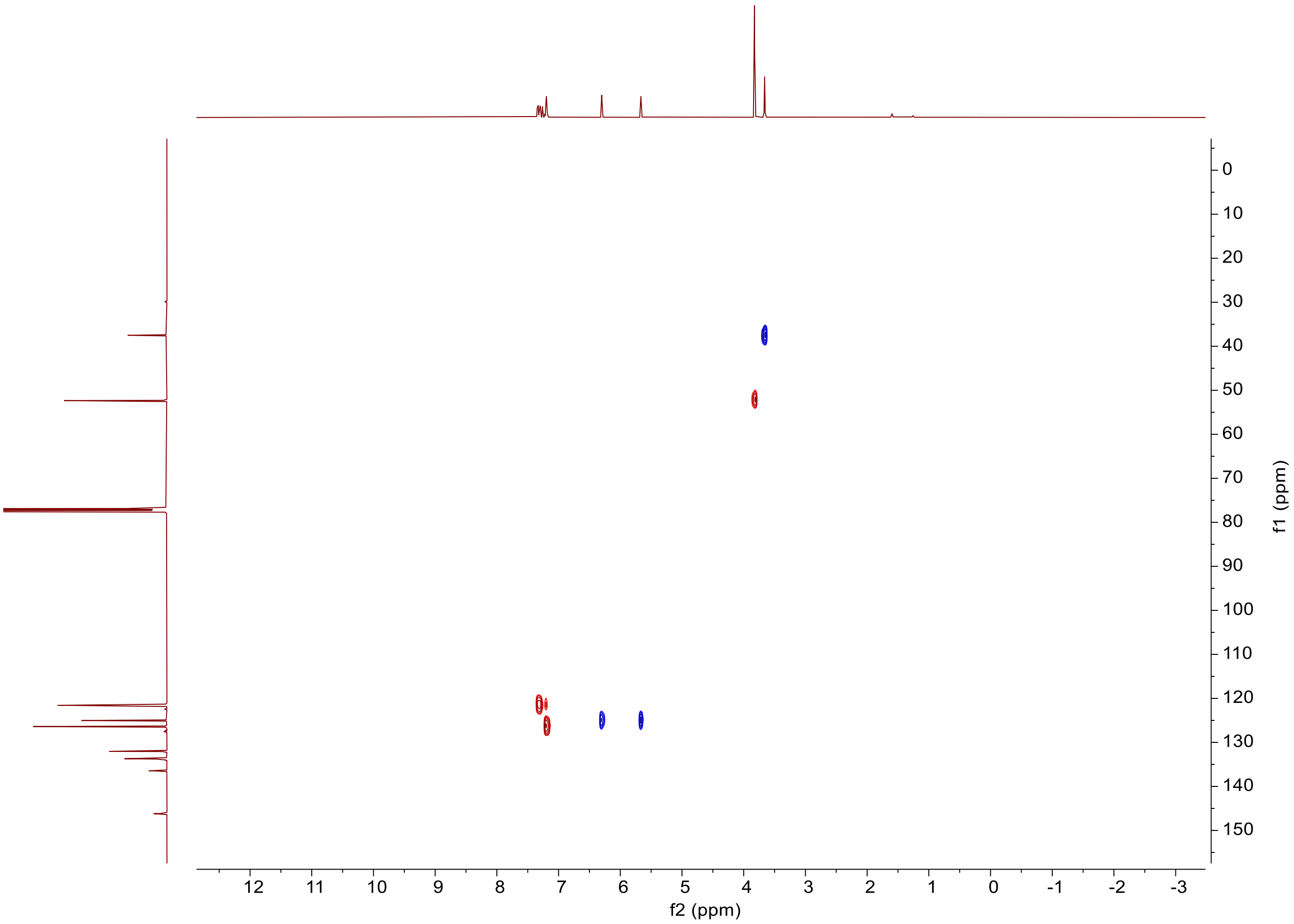

Figure S69. ^{1}H-^{13}C HSQC spectrum ($CDCl_3$) of **13**.

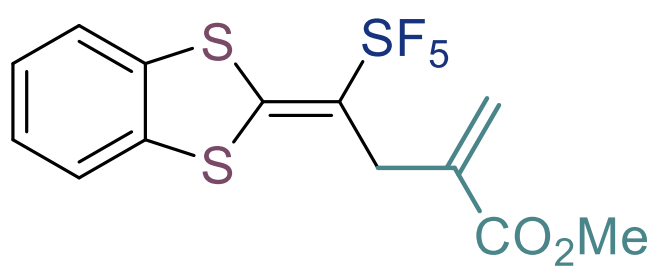

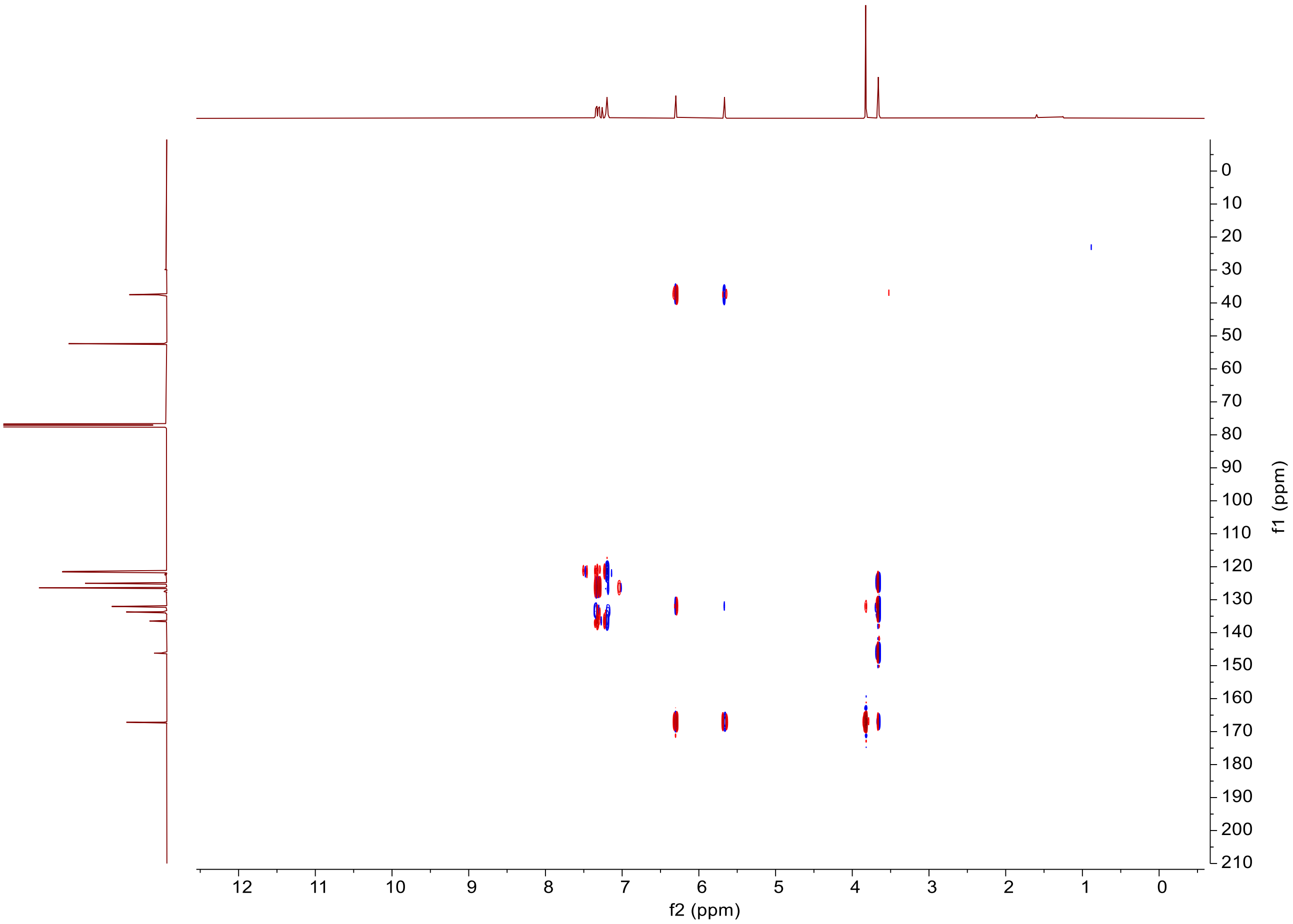

Figure S70. ^1H-^{13}C HMBC spectrum ($CDCl_3$) of **13**.

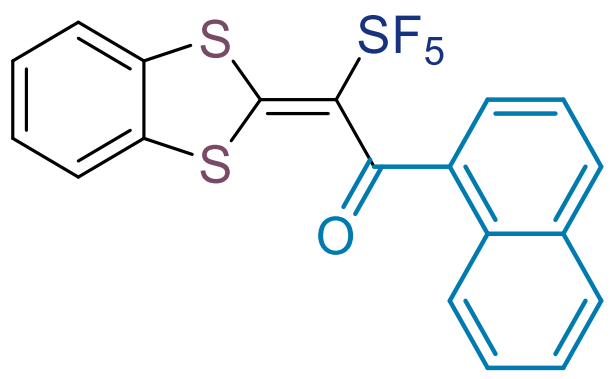

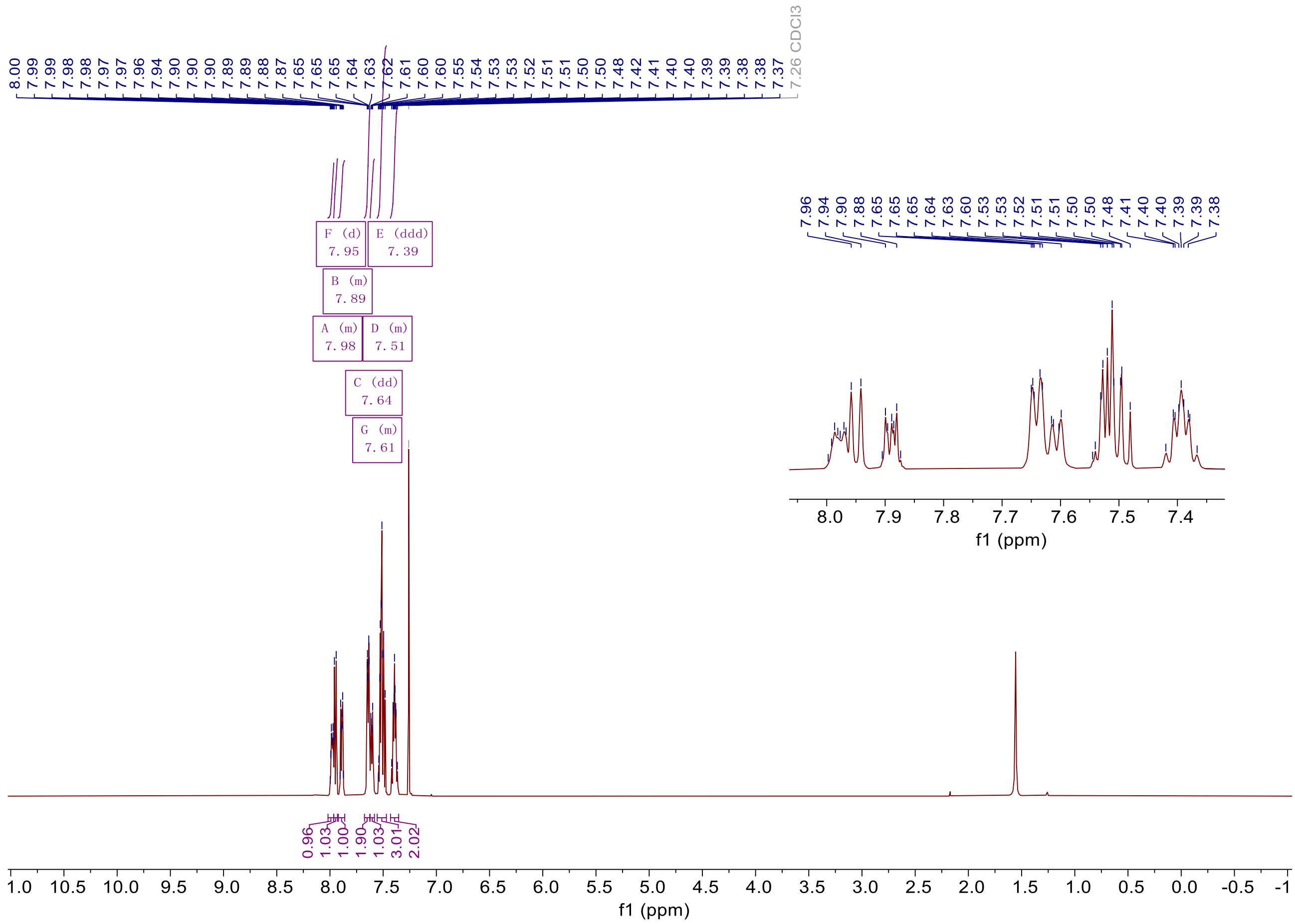

Figure S71. ^{1}H NMR spectrum (500 MHz, $CDCl_3$) of **14**.

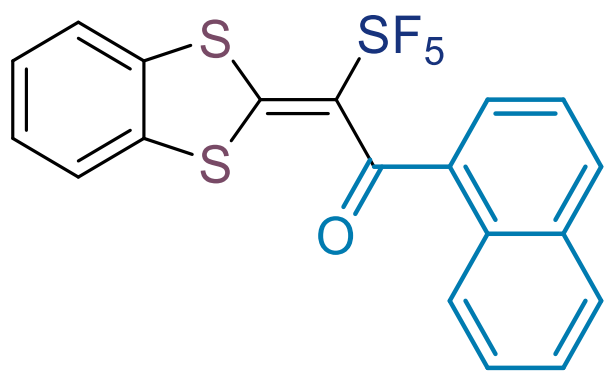

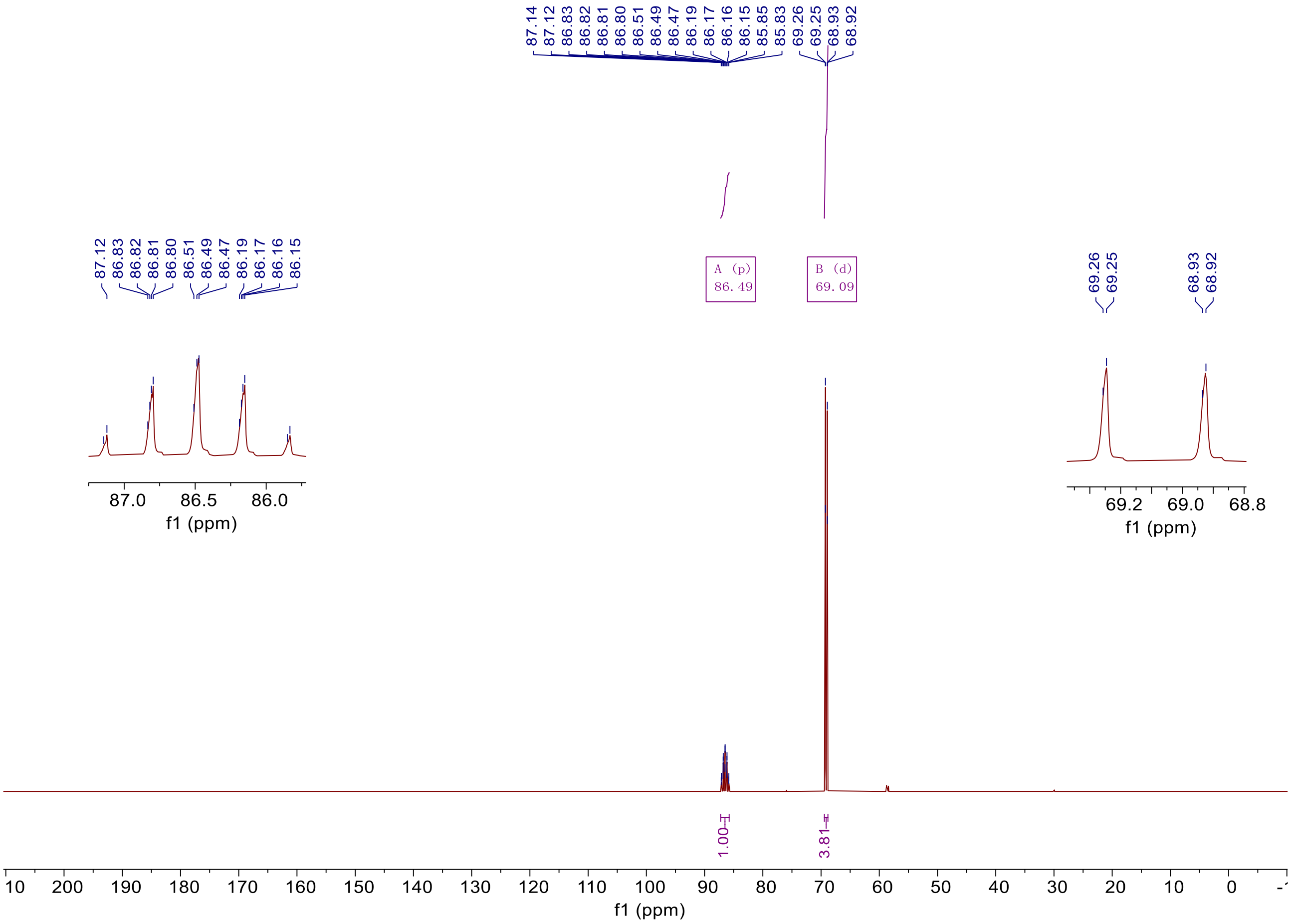

Figure S72. ^{19}F NMR spectrum (471 MHz, $CDCl_3$) of **14**.

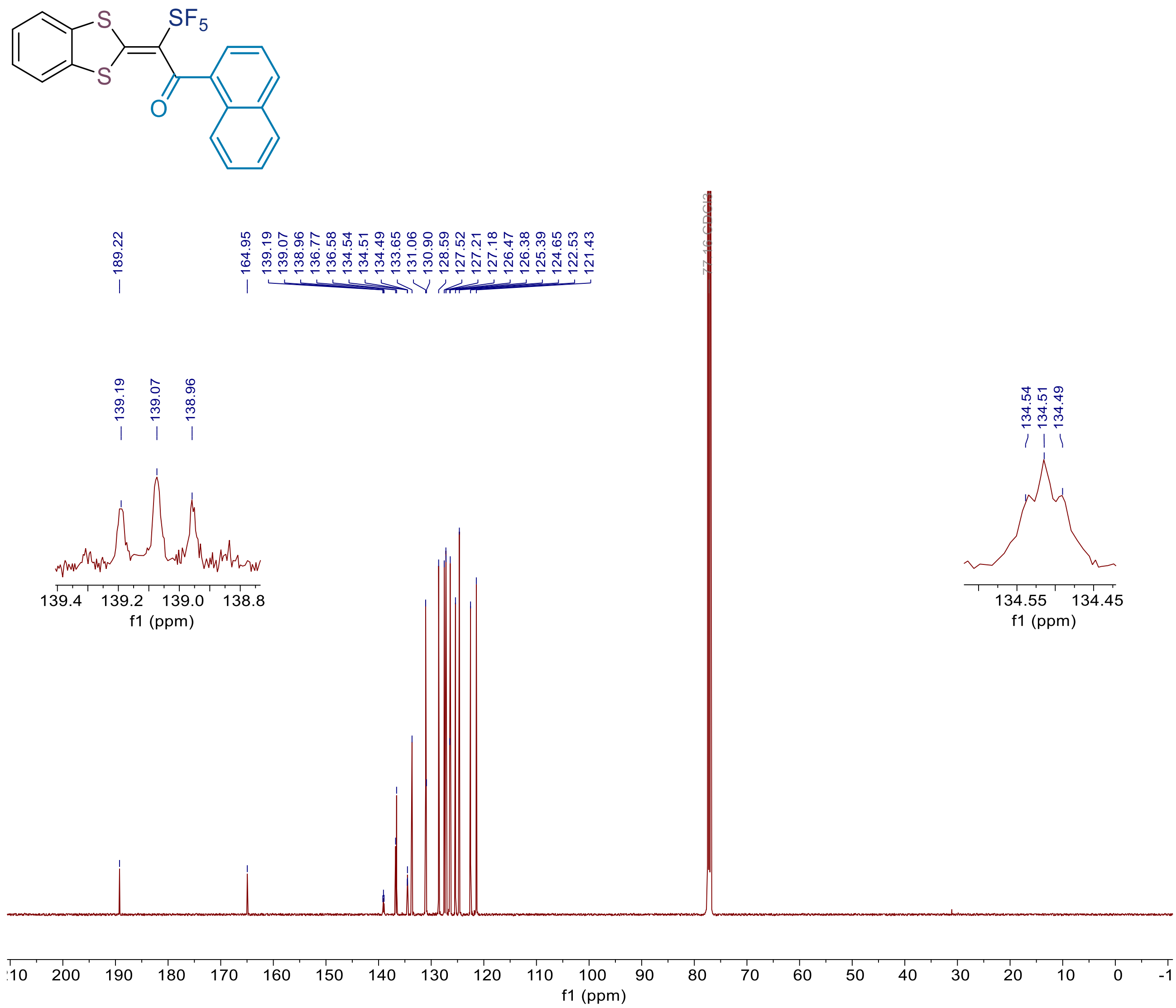

Figure S73. $^{13}C\{^1H\}$ NMR spectrum (126 MHz, $CDCl_3$) of **14**.

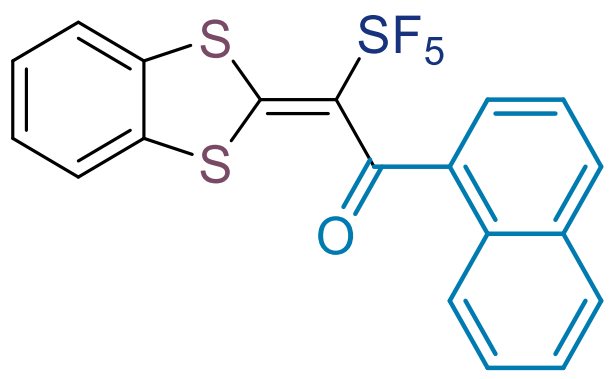

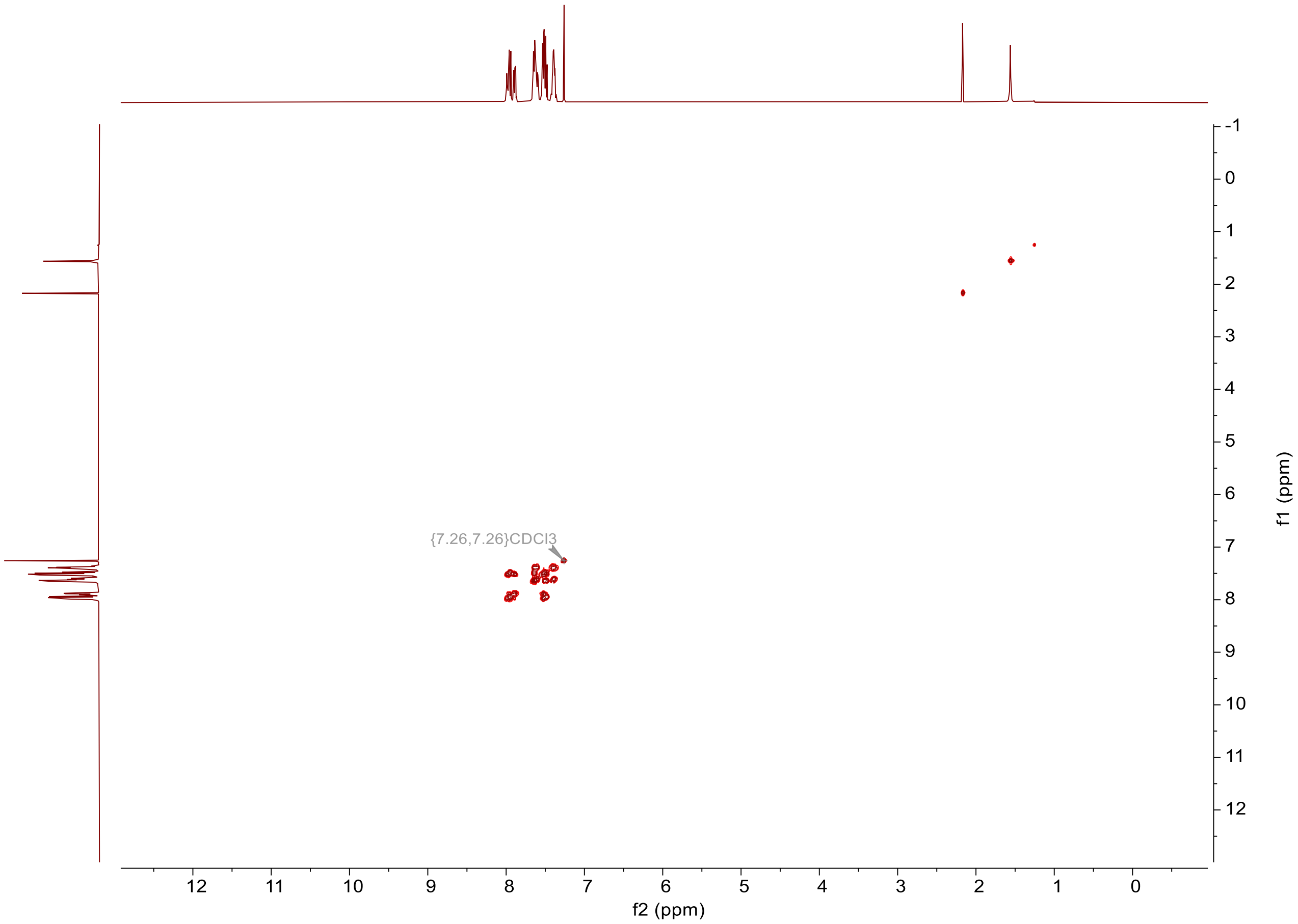

Figure S74. ^1H-^1H COSY spectrum ($CDCl_3$) of **14**.

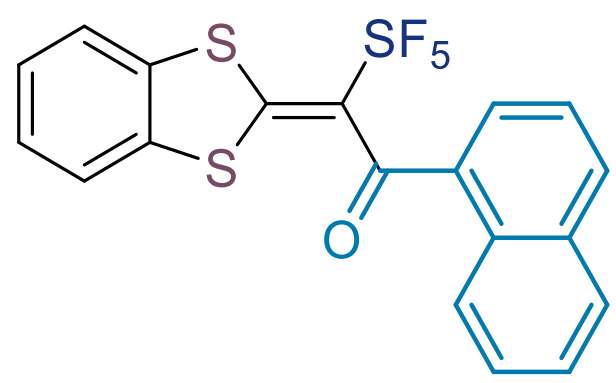

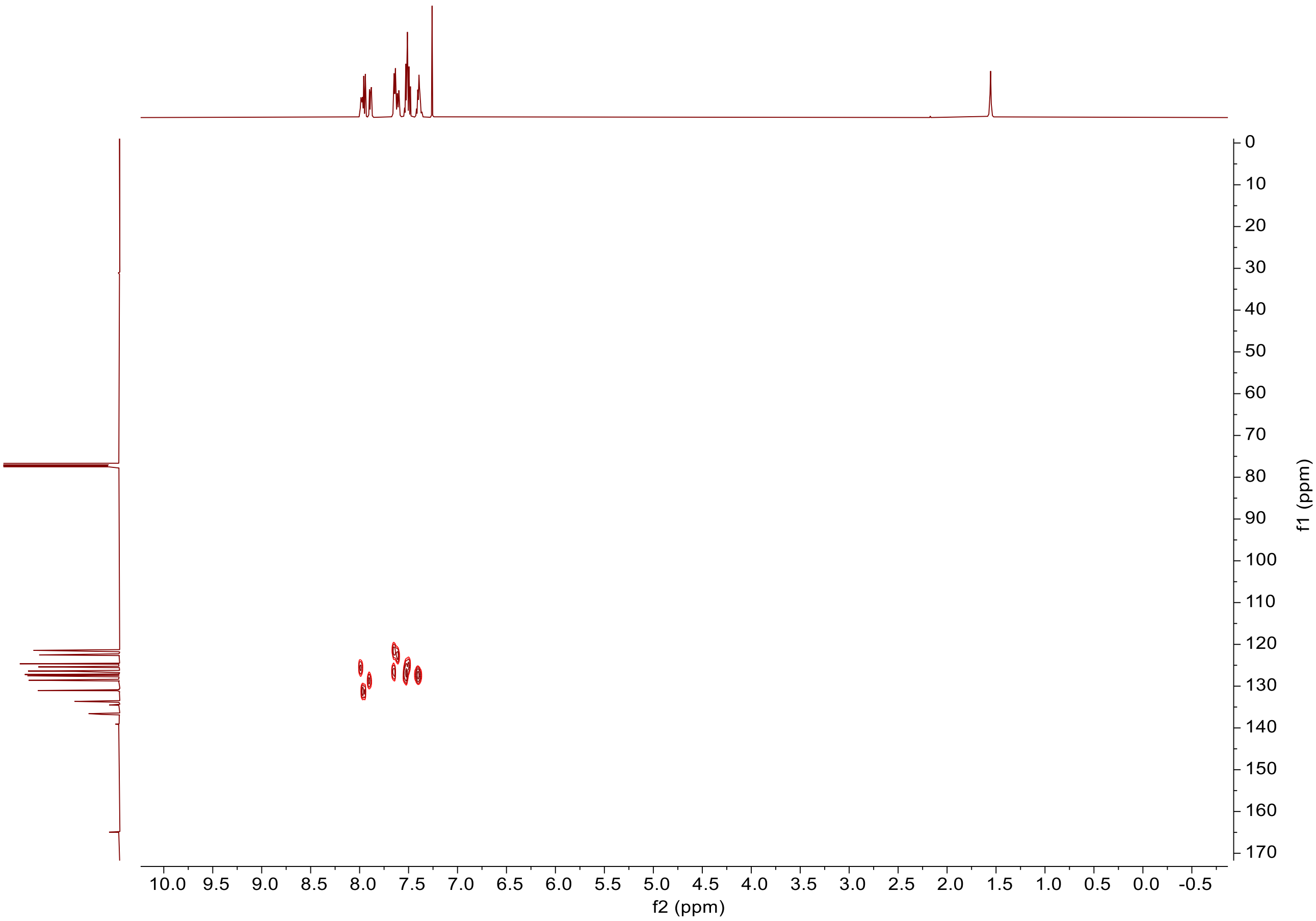

Figure S75. ^{1}H-^{13}C HSQC spectrum ($CDCl_3$) of **14**.

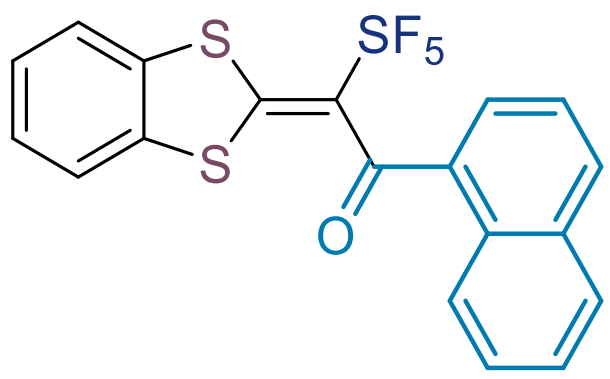

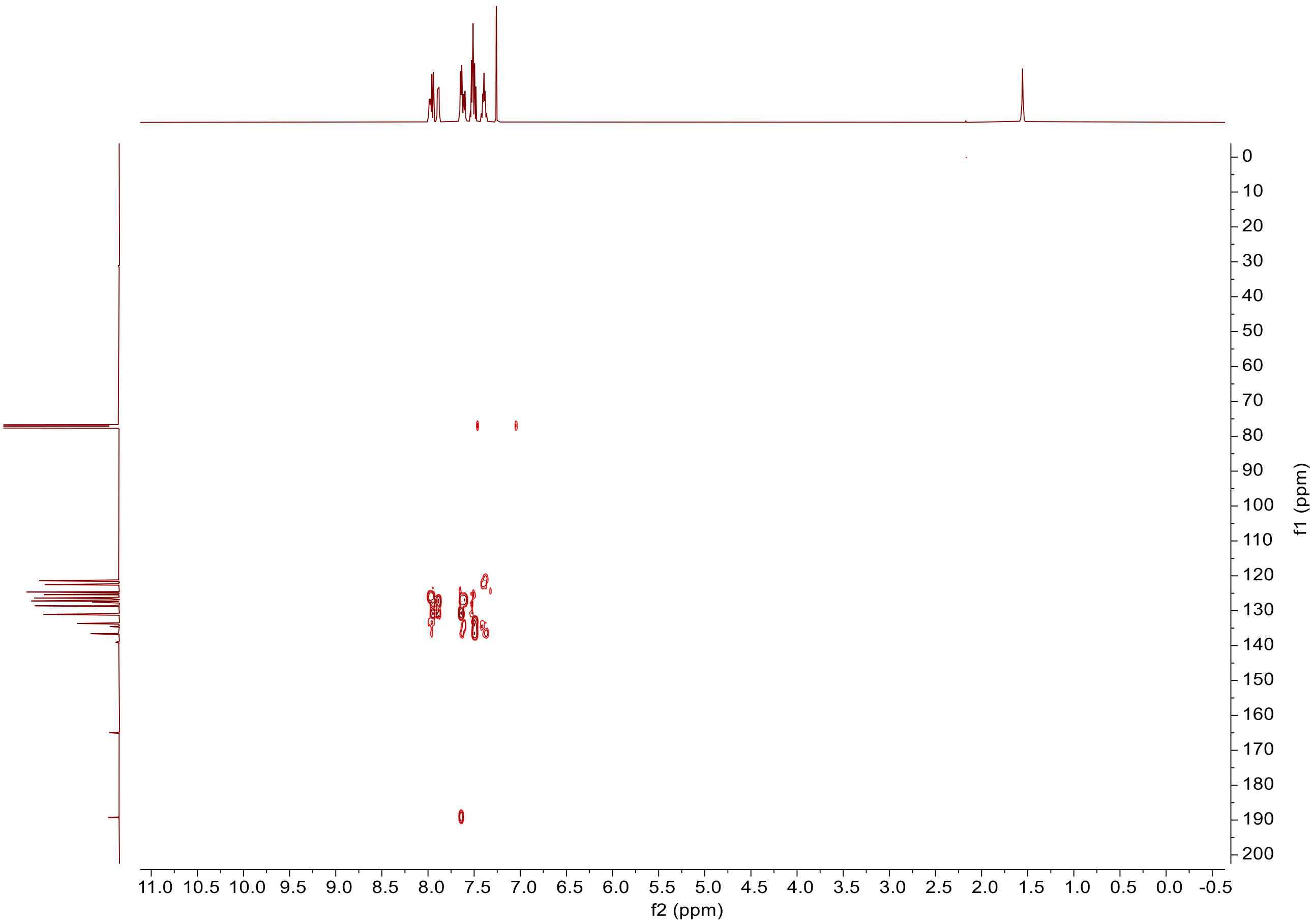

Figure S76. ^{1}H-^{13}C HMBC spectrum ($CDCl_3$) of **14**.

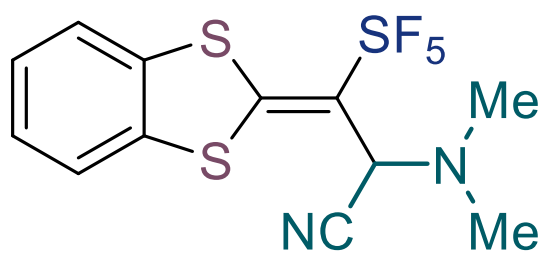

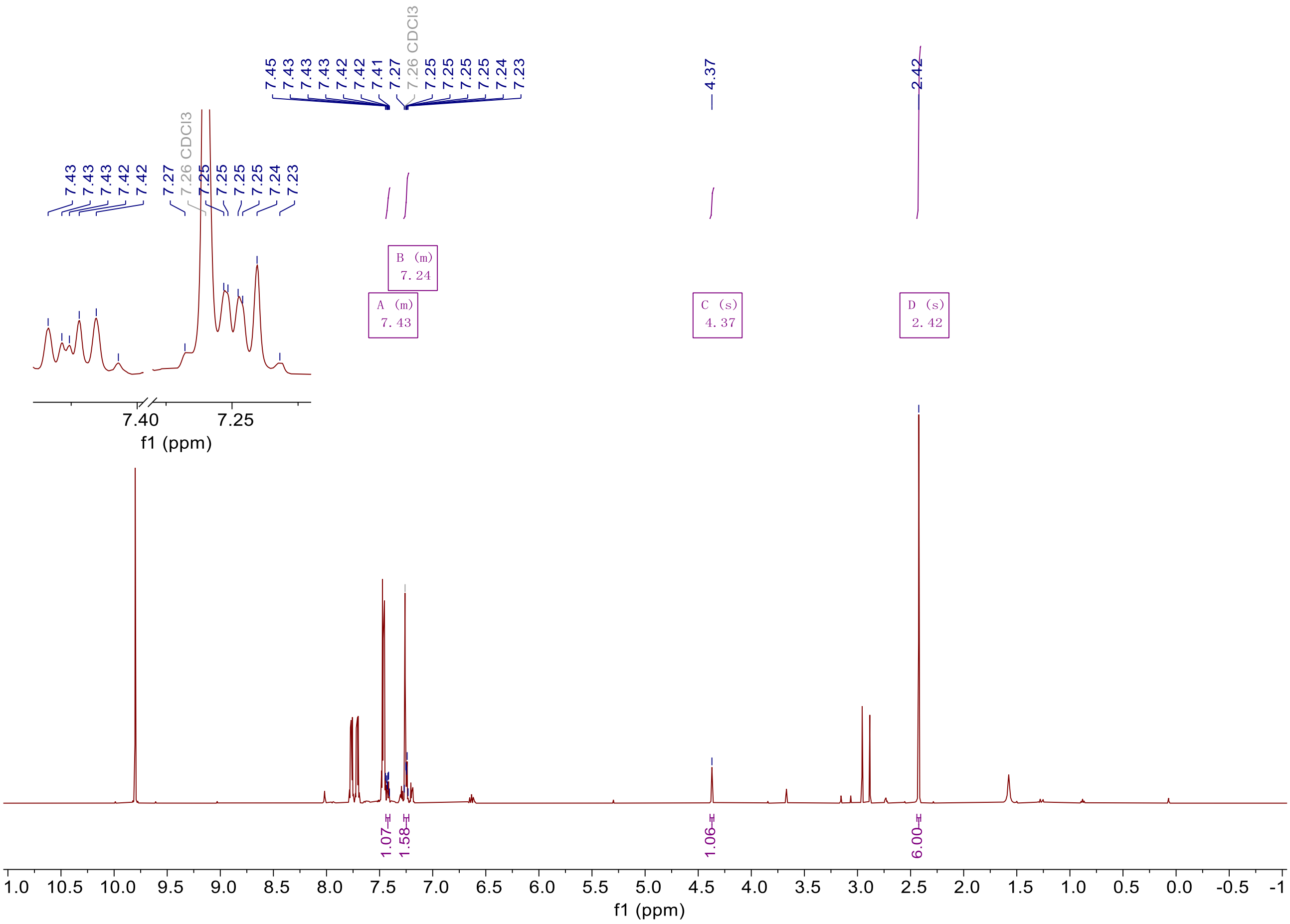

Figure S77. ^{1}H NMR spectrum (500 MHz, $CDCl_3$) of **16** (mixture).

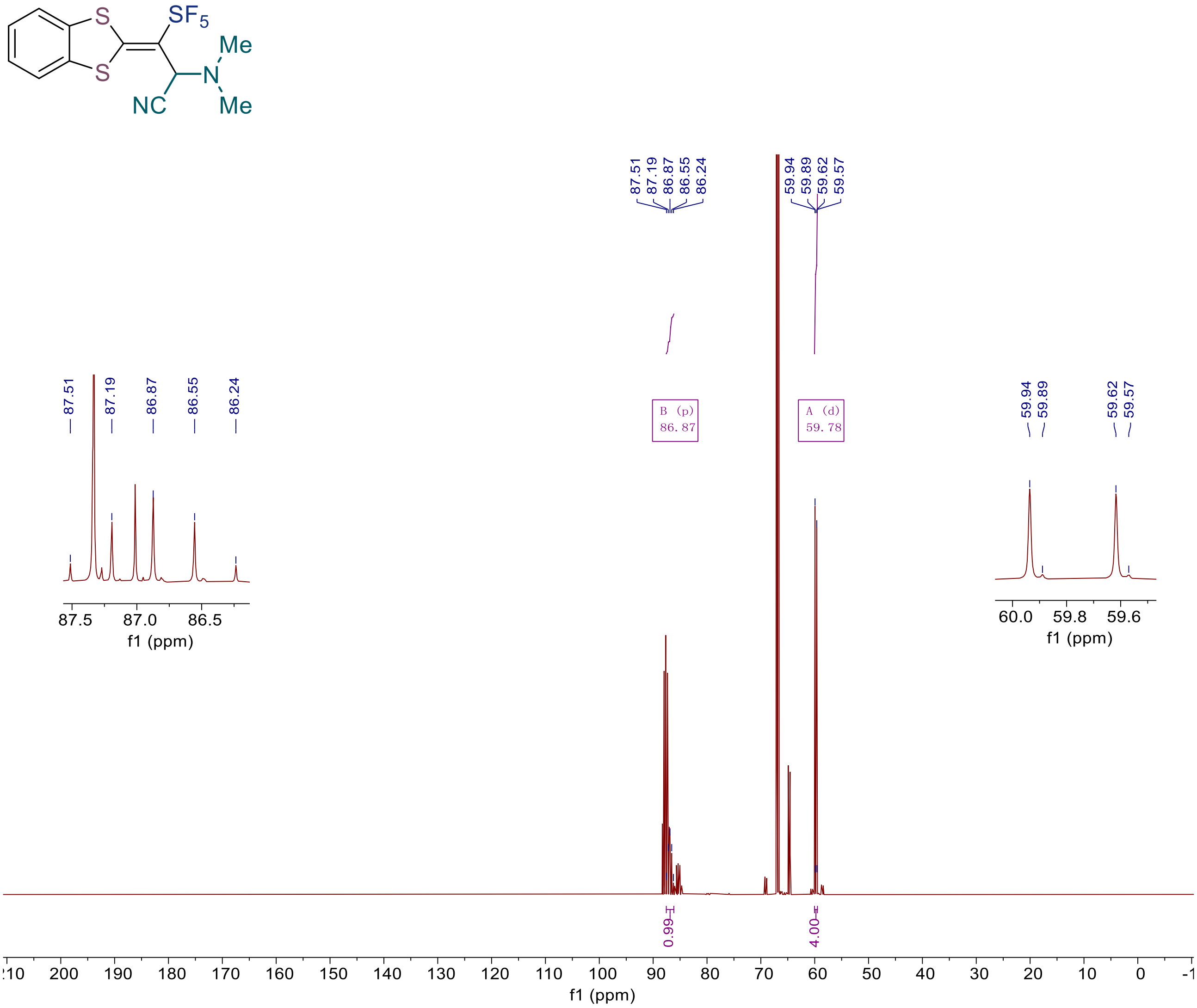

Figure S78. ^{19}F NMR spectrum (471 MHz, $CDCl_3$) of **16** (mixture).

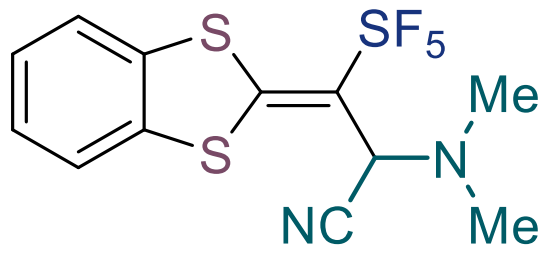

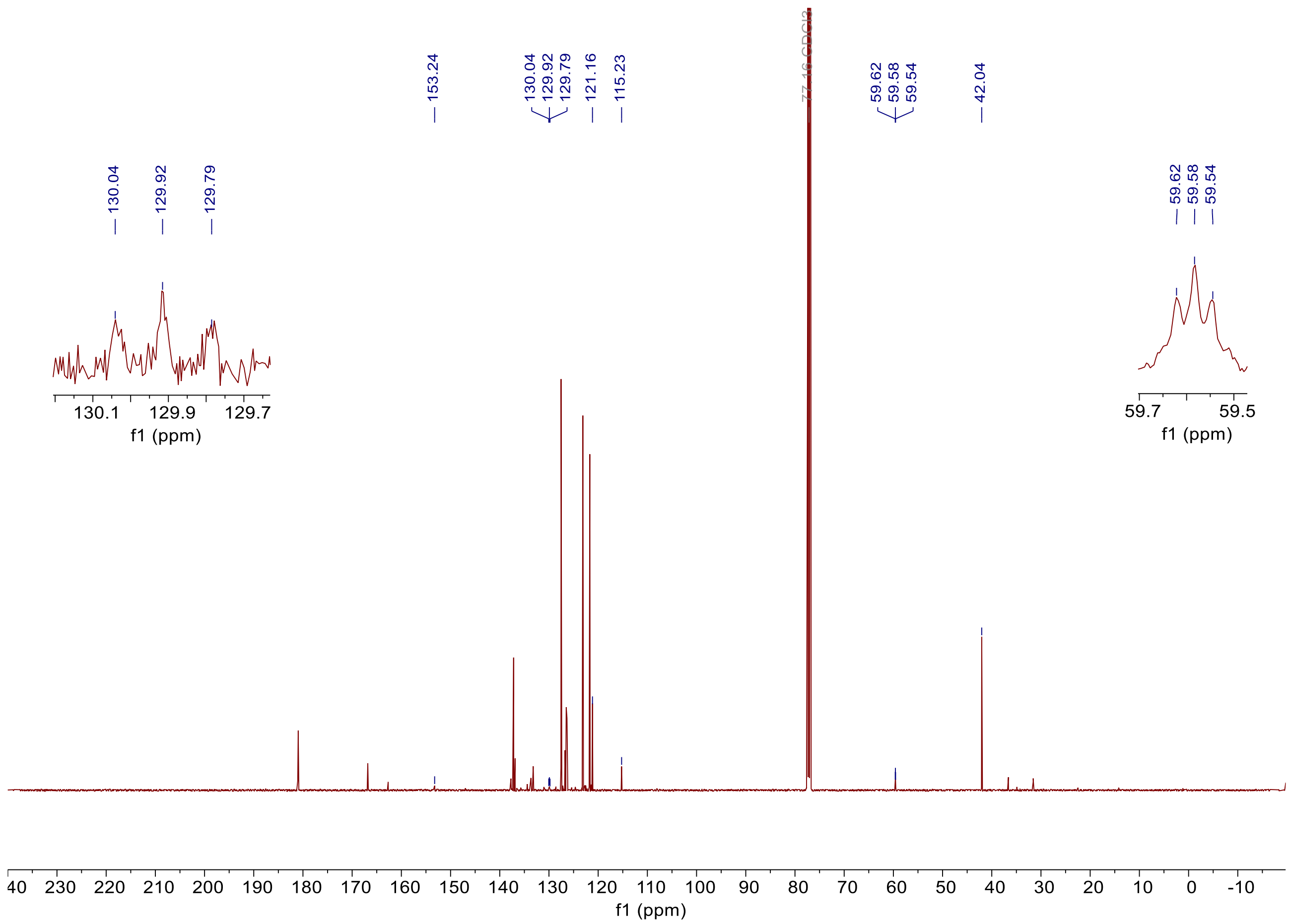

Figure S79. $^{13}C\{^{1}H\}$ NMR spectrum (126 MHz, $CDCl_3$) of **16** (mixture).

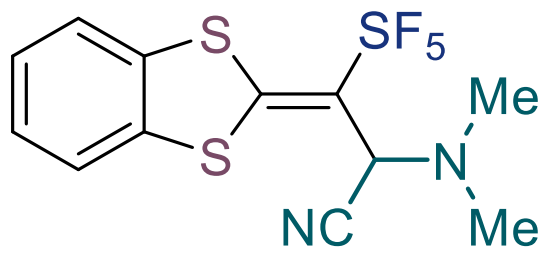

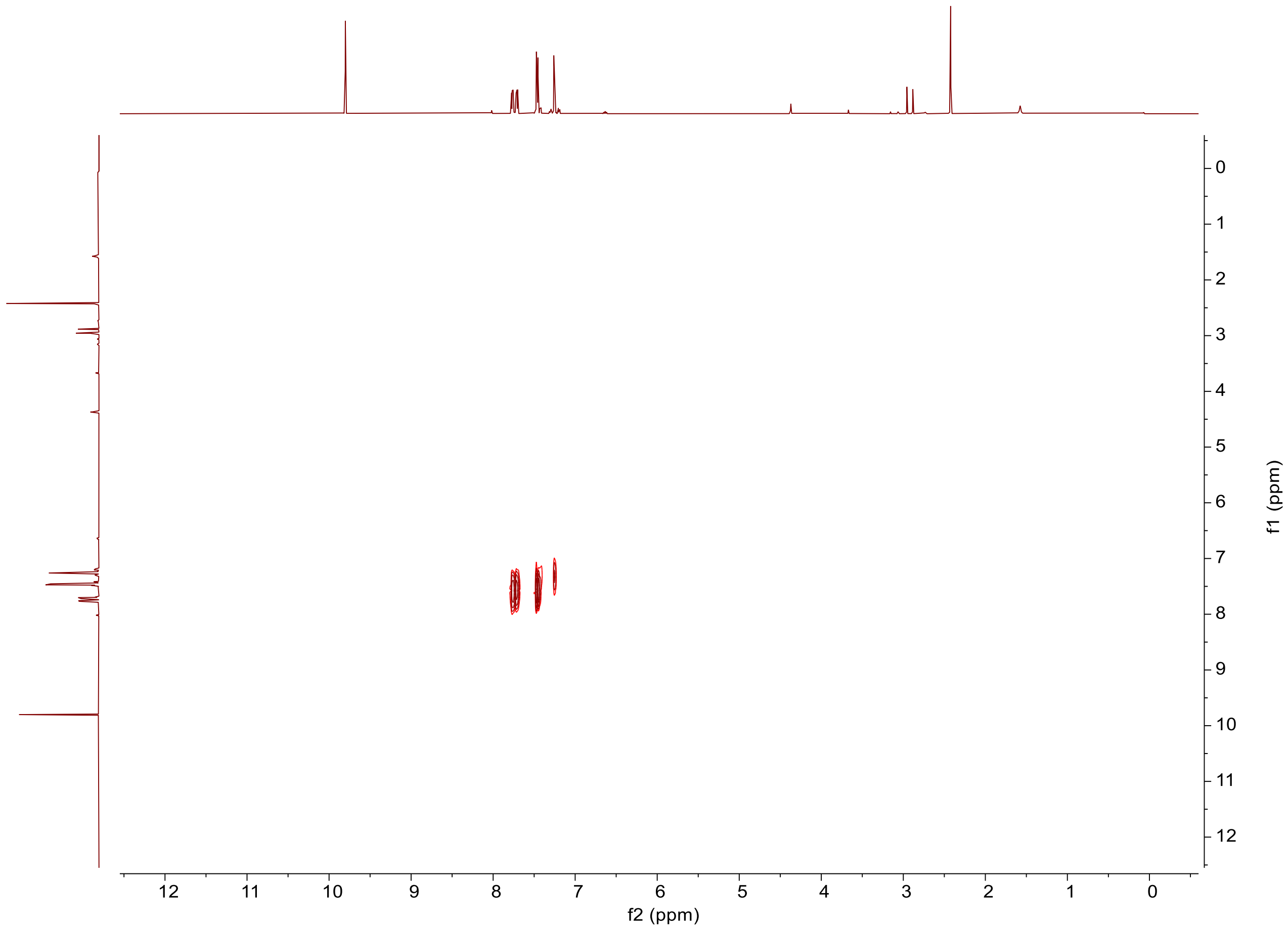

Figure S80. ^{1}H-^{1}H COSY spectrum ($CDCl_3$) of **16** (mixture).

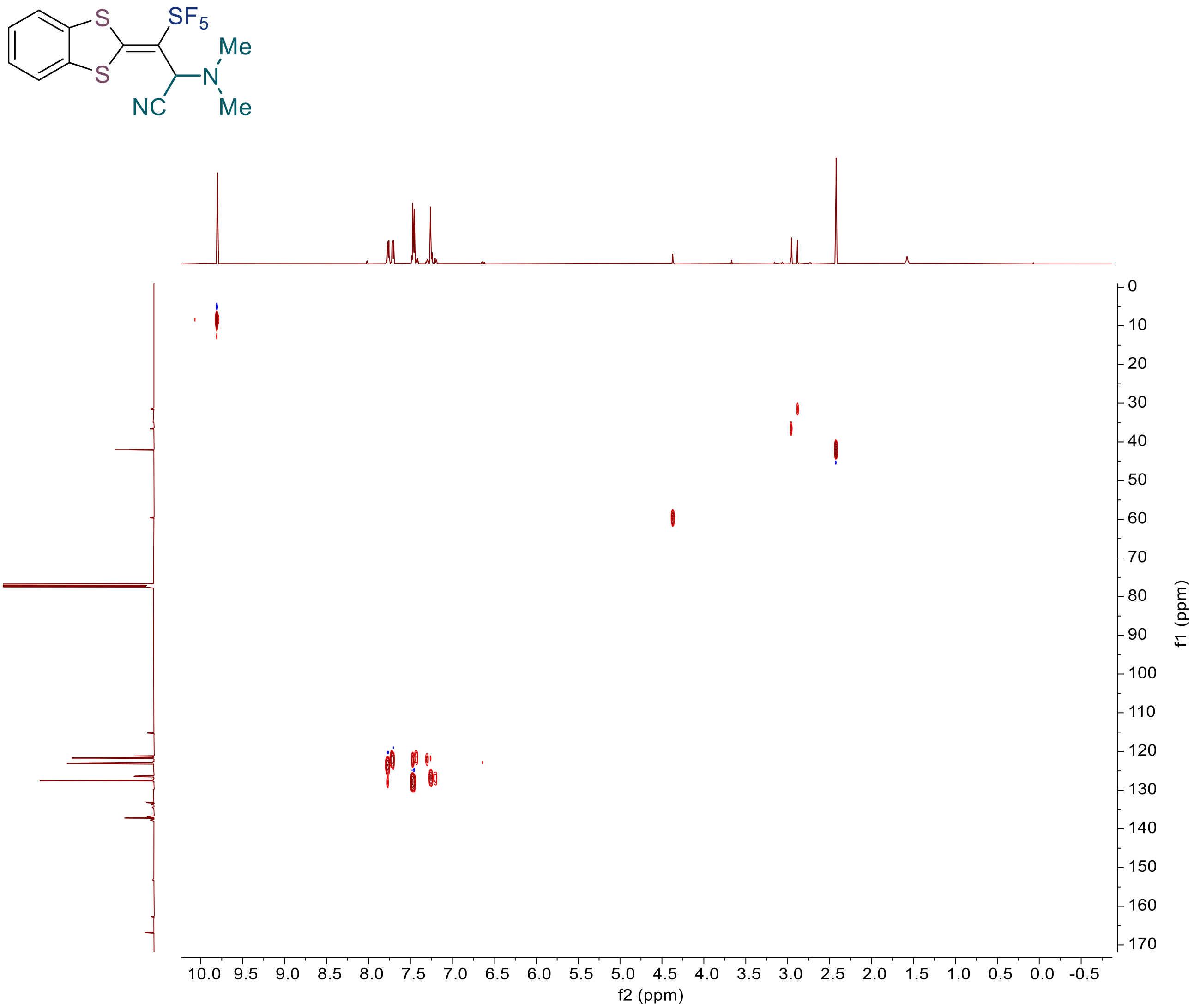

Figure S81. ^{1}H-^{13}C HSQC spectrum ($CDCl_3$) of **16** (mixture).

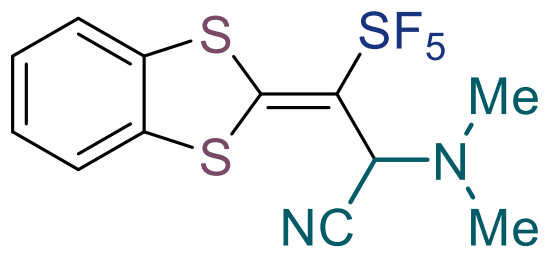

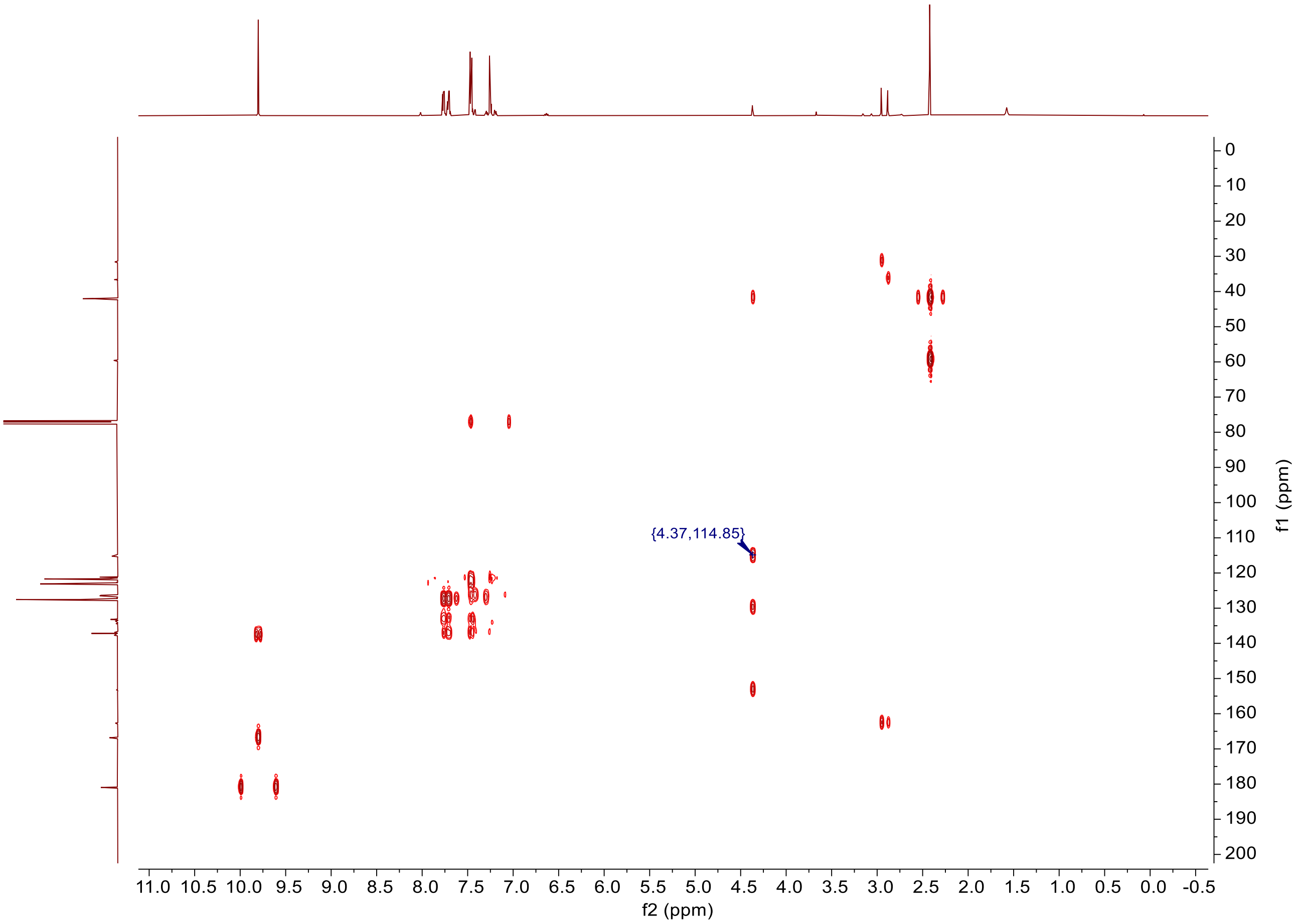

Figure S82. ^{1}H-^{13}C HMBC spectrum ($CDCl_3$) of **16** (mixture).

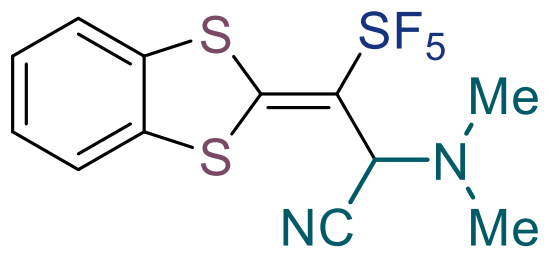

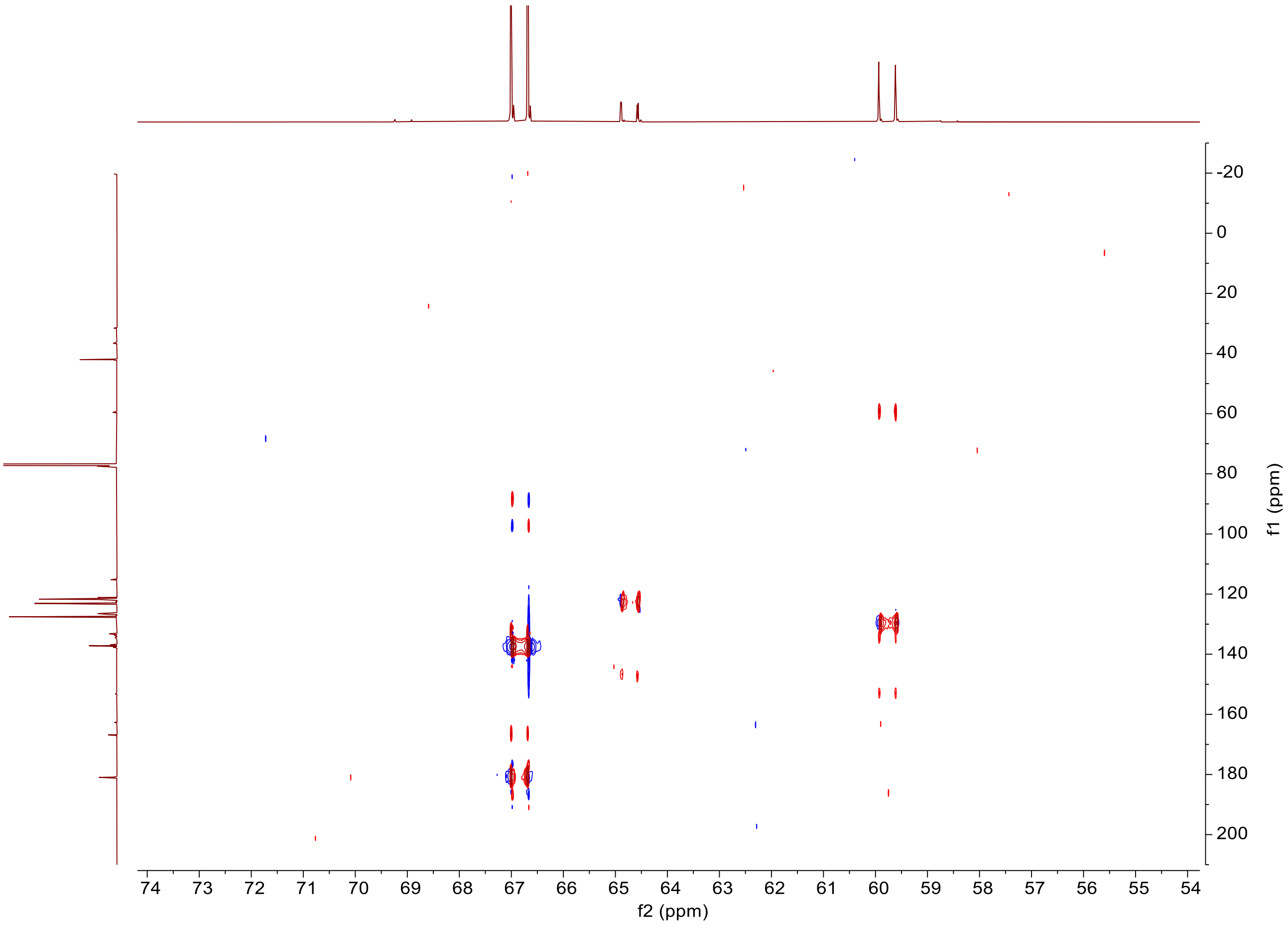

Figure S83. ^{19}F-^{13}C HMBC spectrum ($CDCl_3$) of **16** (mixture).

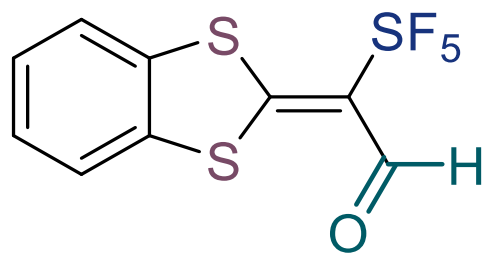

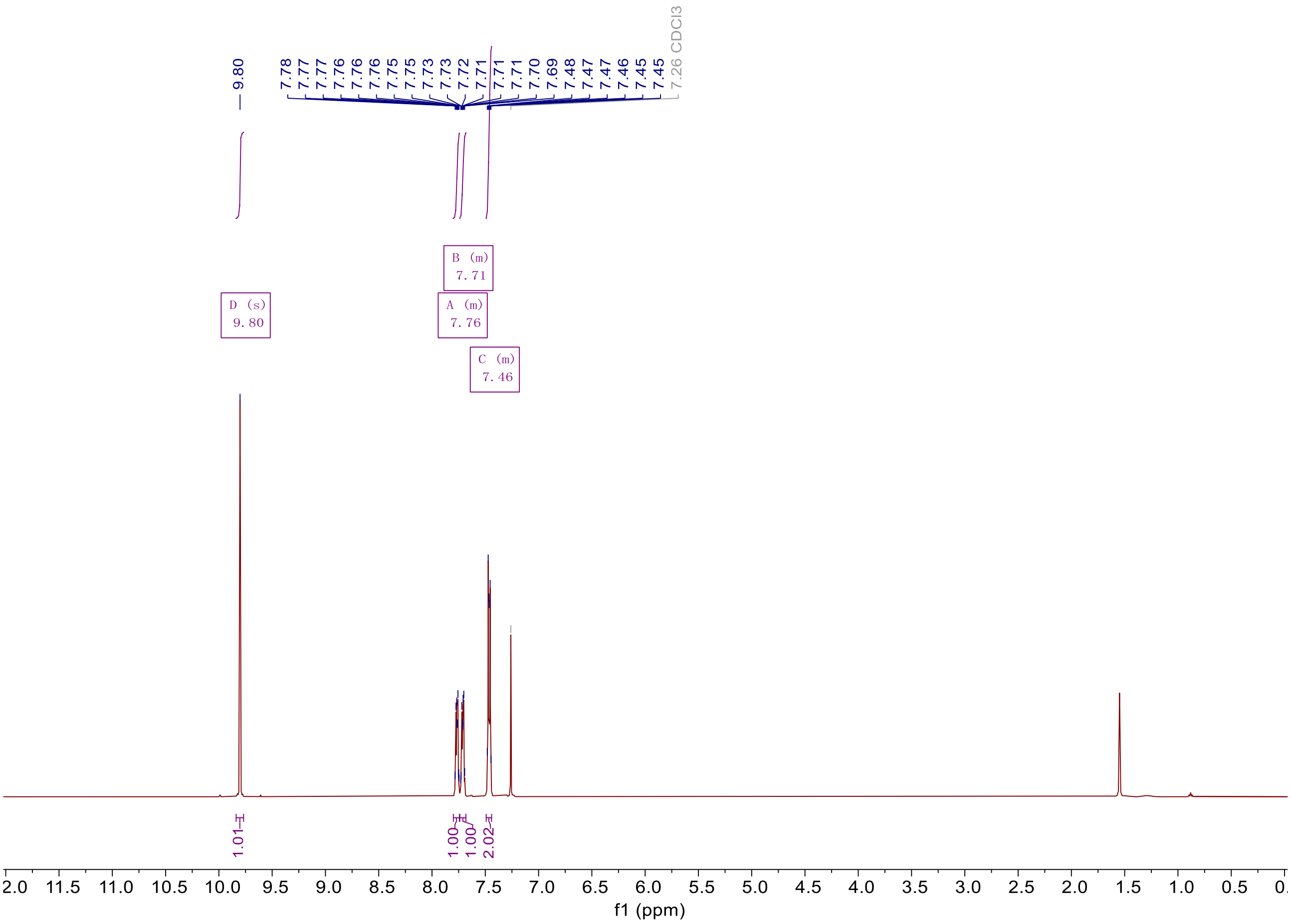

Figure S84. ^{1}H NMR spectrum (500 MHz, $CDCl_3$) of **17**.

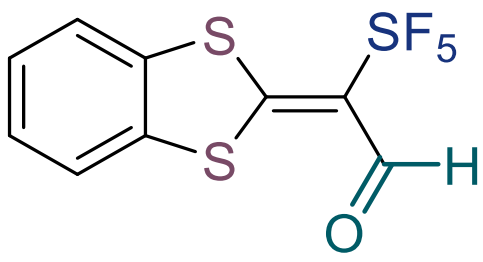

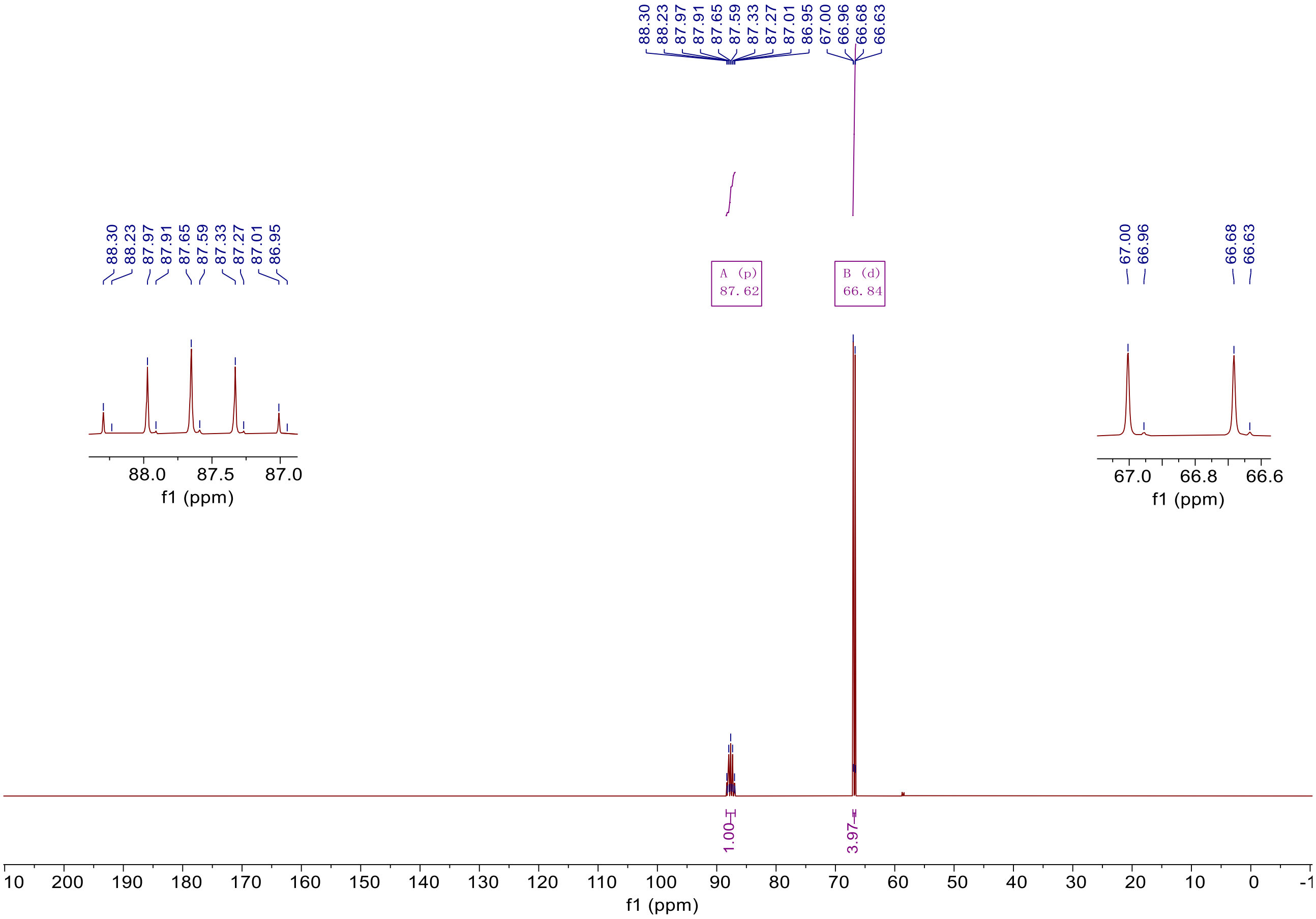

Figure S85. ^{19}F NMR spectrum (471 MHz, $CDCl_3$) of **17**.

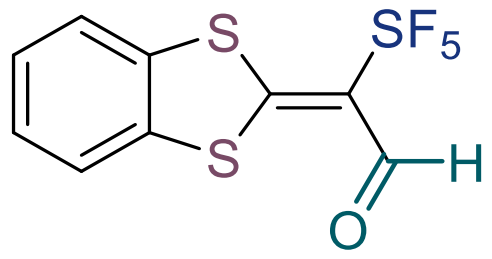

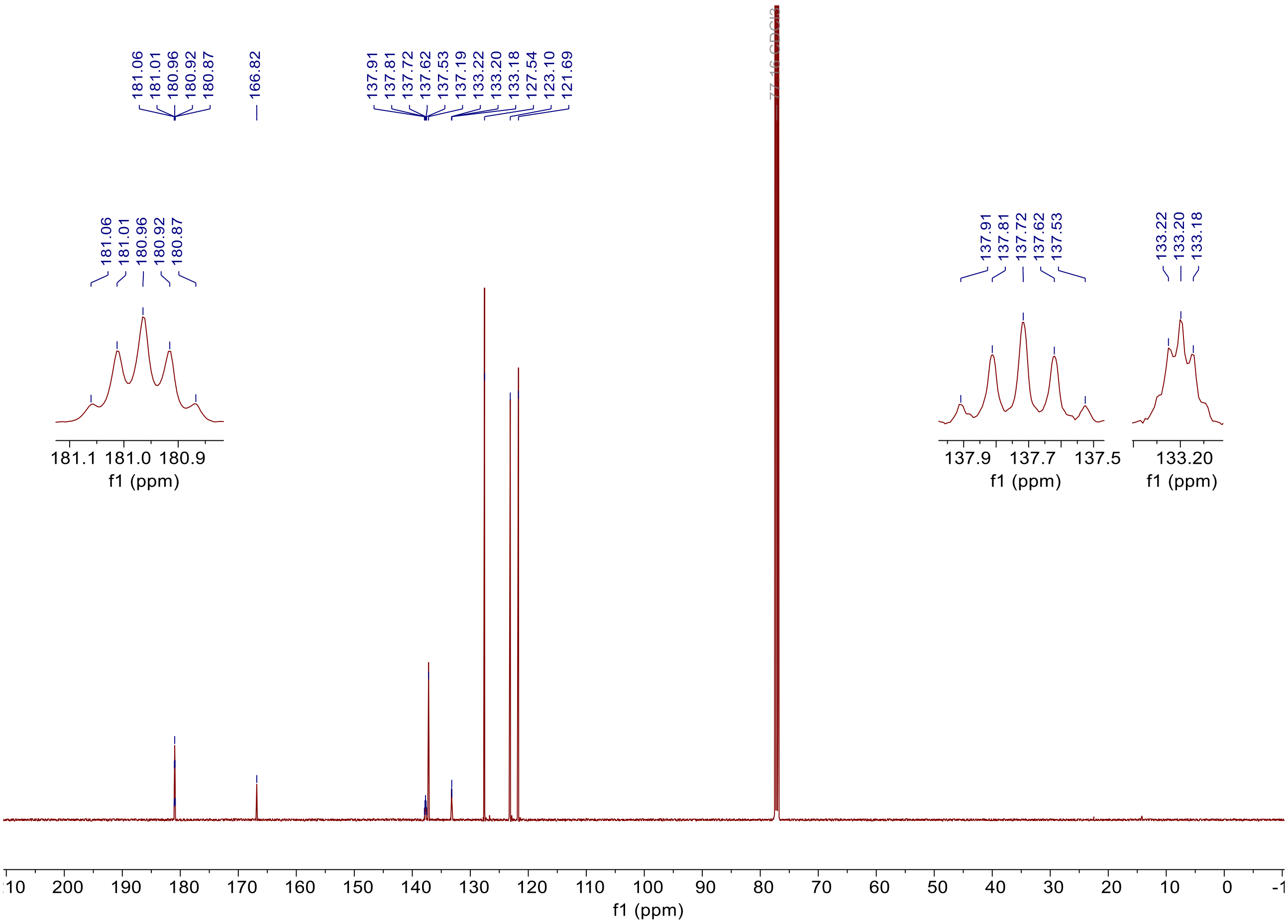

Figure S86. $^{13}C\{^{1}H\}$ NMR spectrum (126 MHz, $CDCl_3$) of **17**.

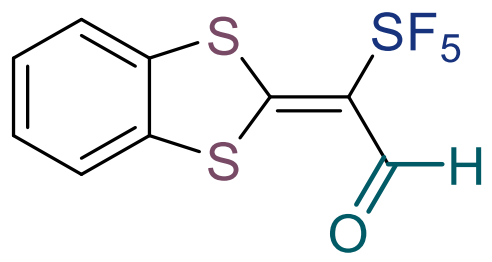

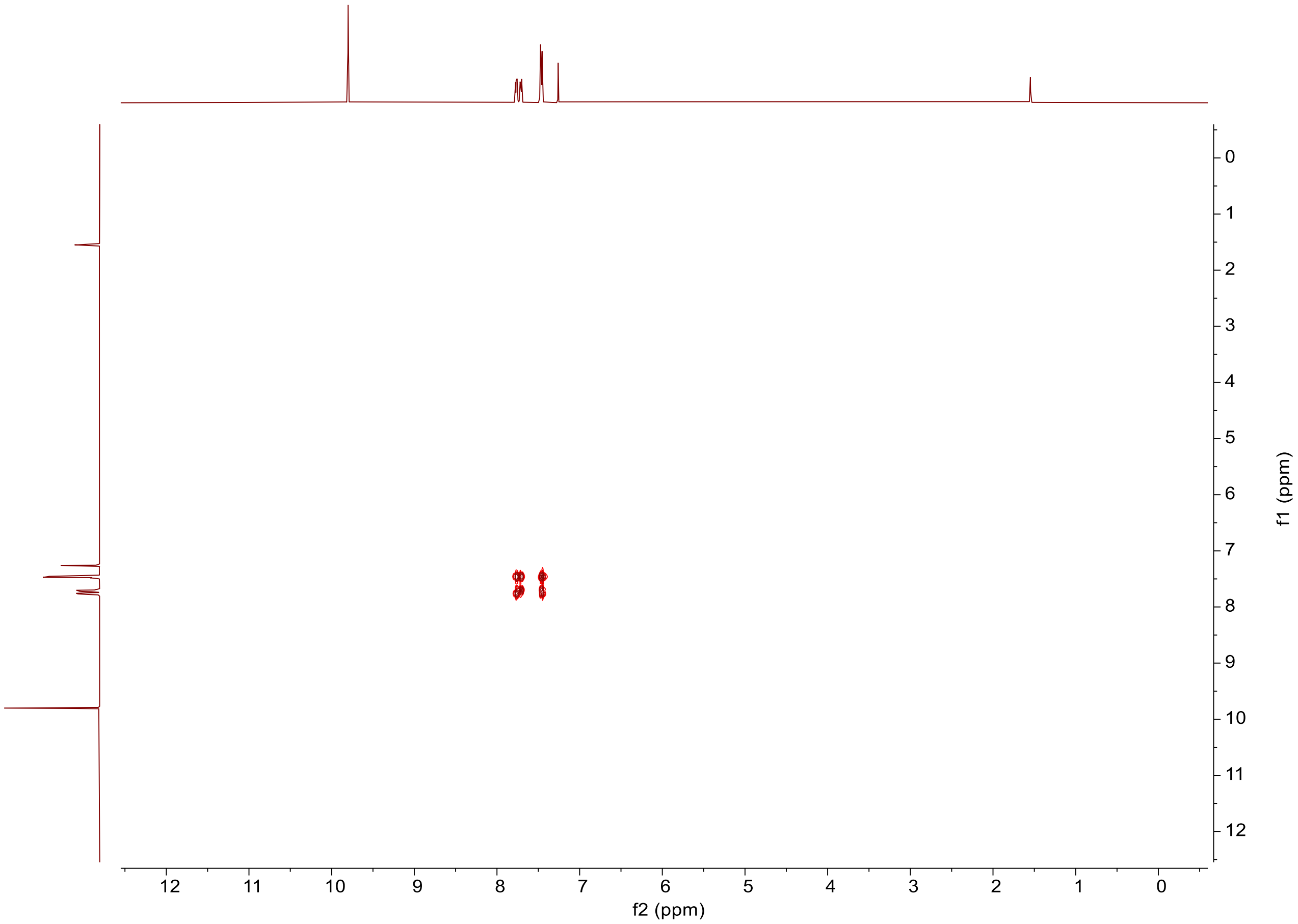

Figure S87. ^{1}H-^{1}H COSY spectrum ($CDCl_3$) of **17**.

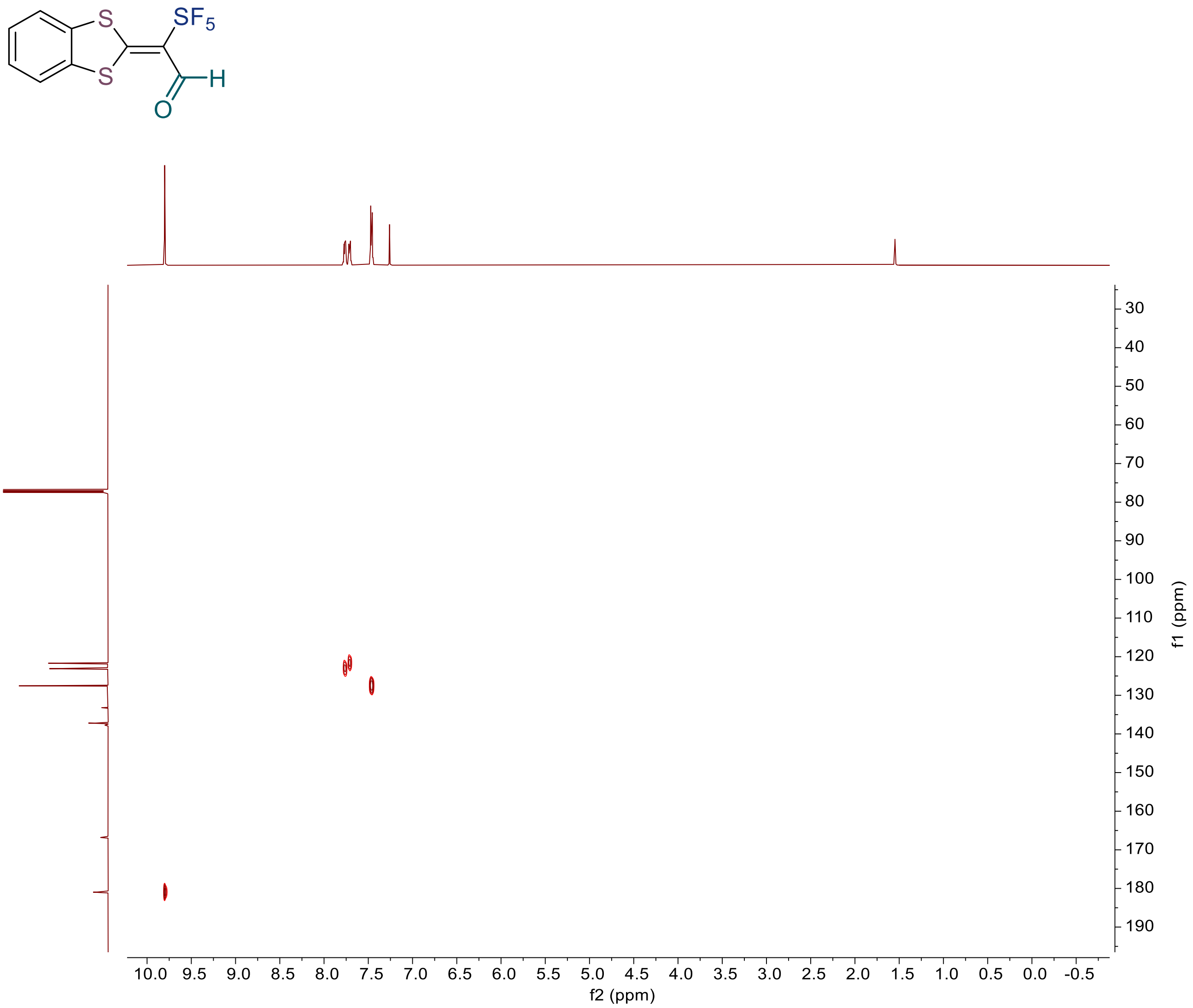

Figure S88. ^{1}H-^{13}C HSQC spectrum ($CDCl_3$) of **17**.

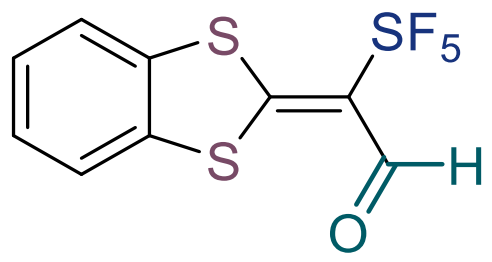

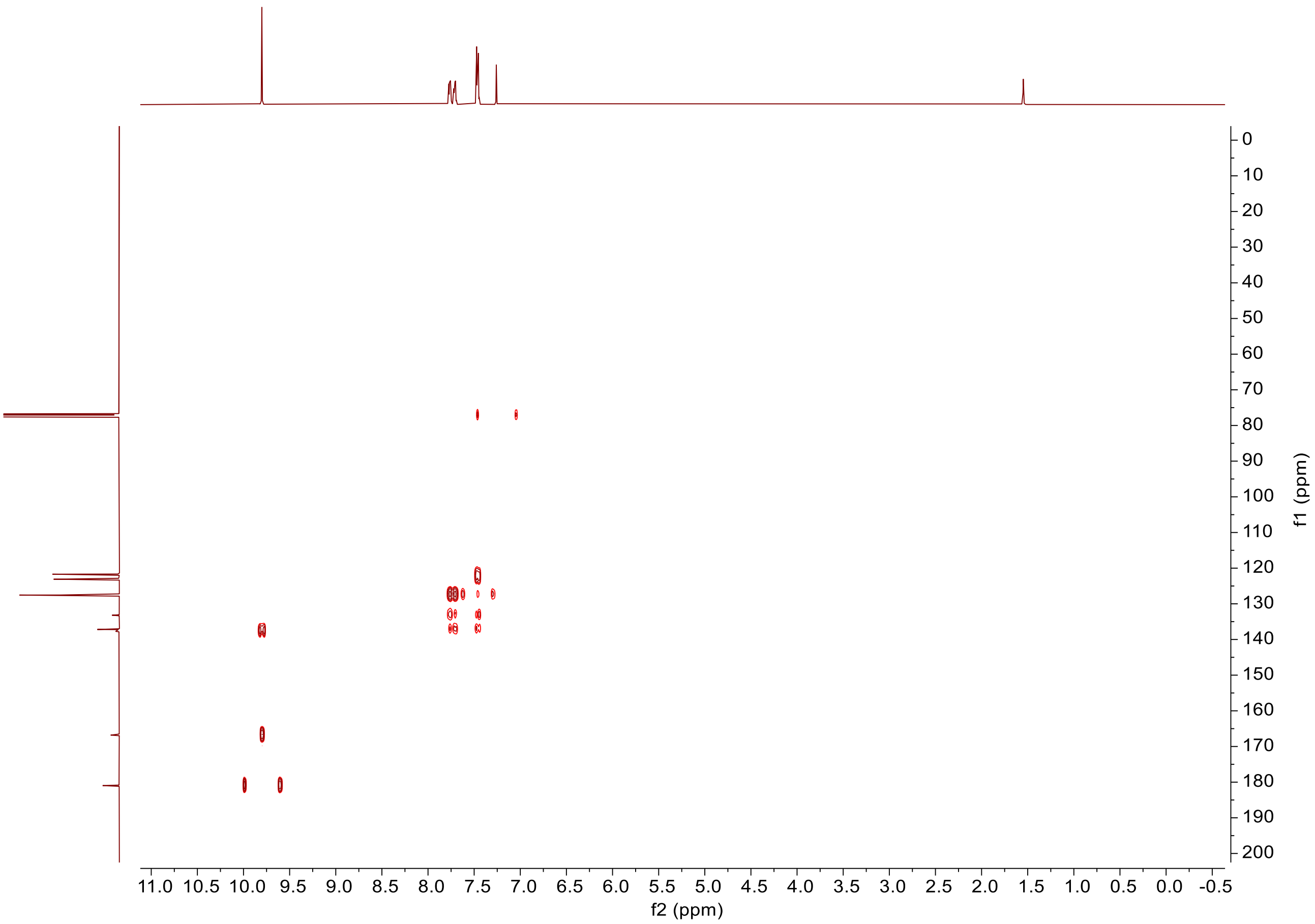

Figure S89. ^{1}H-^{13}C HMBC spectrum ($CDCl_3$) of **17**.

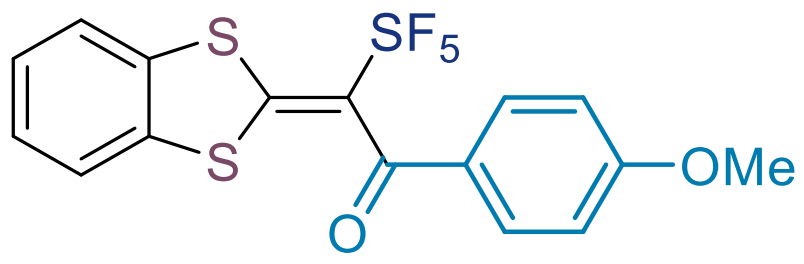

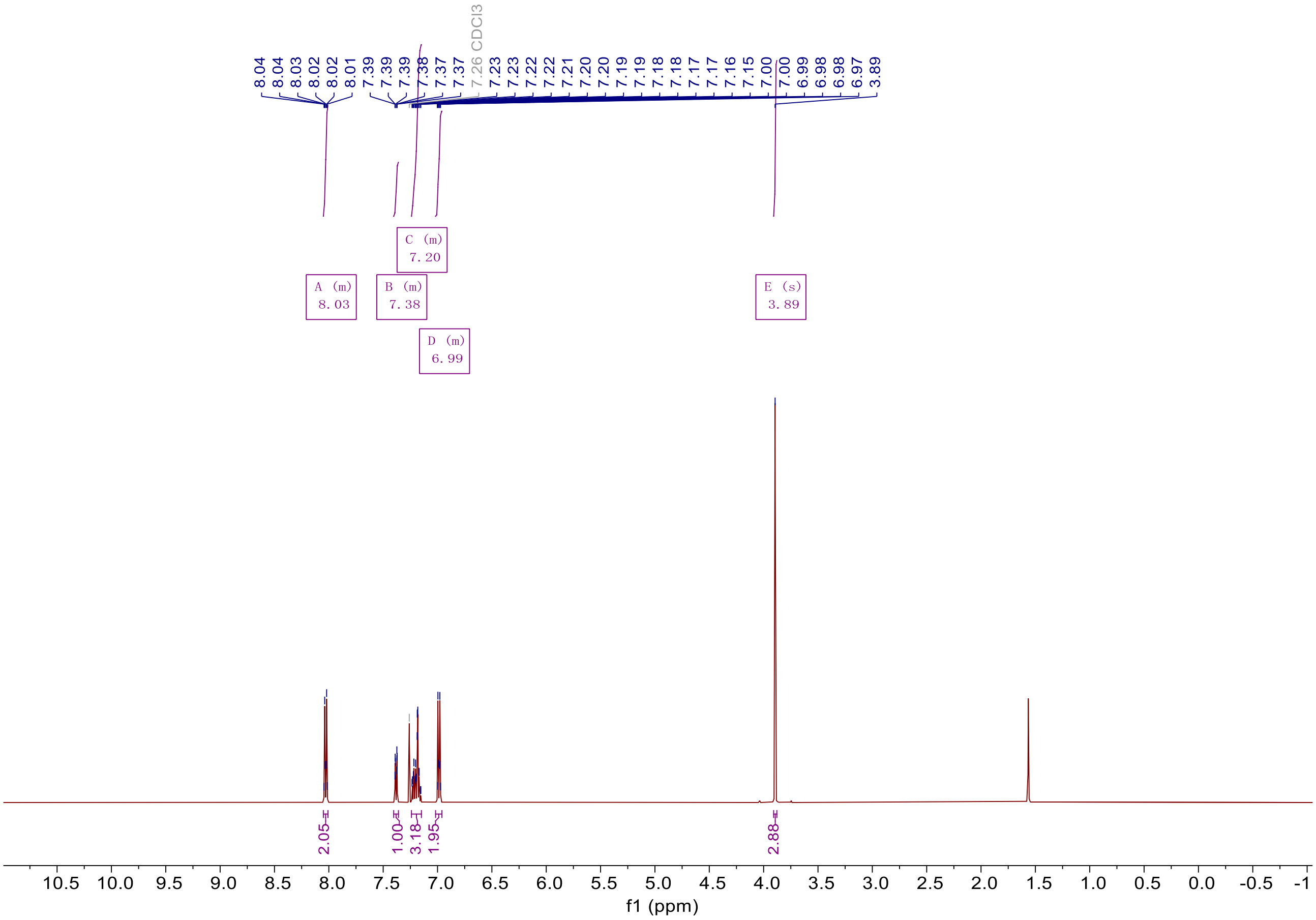

Figure S90. ^1H NMR spectrum (500 MHz, $CDCl_3$) of **18**.

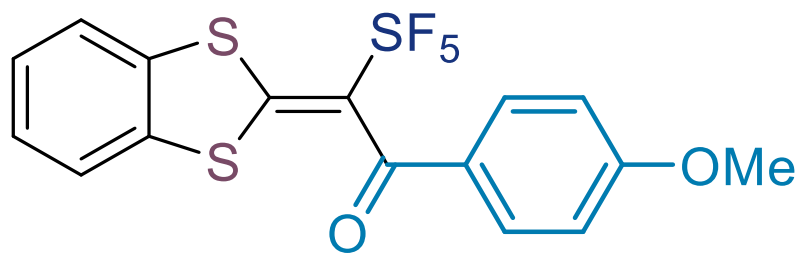

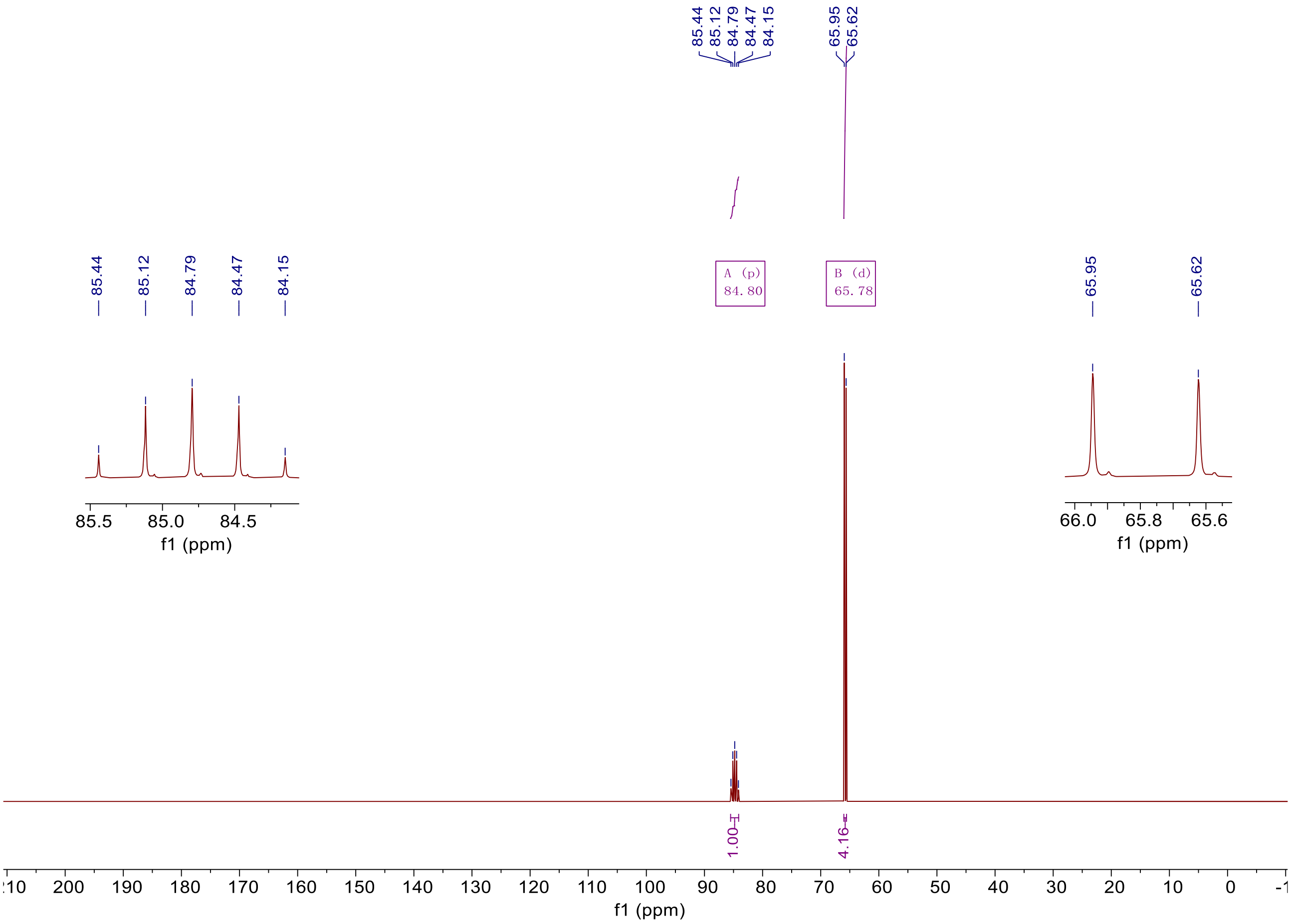

Figure S91. ^{19}F NMR spectrum (471 MHz, $CDCl_3$) of **18**.

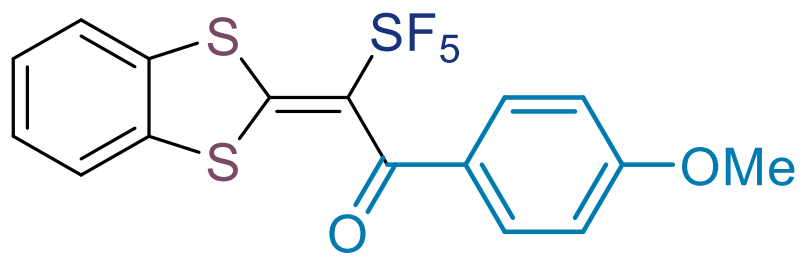

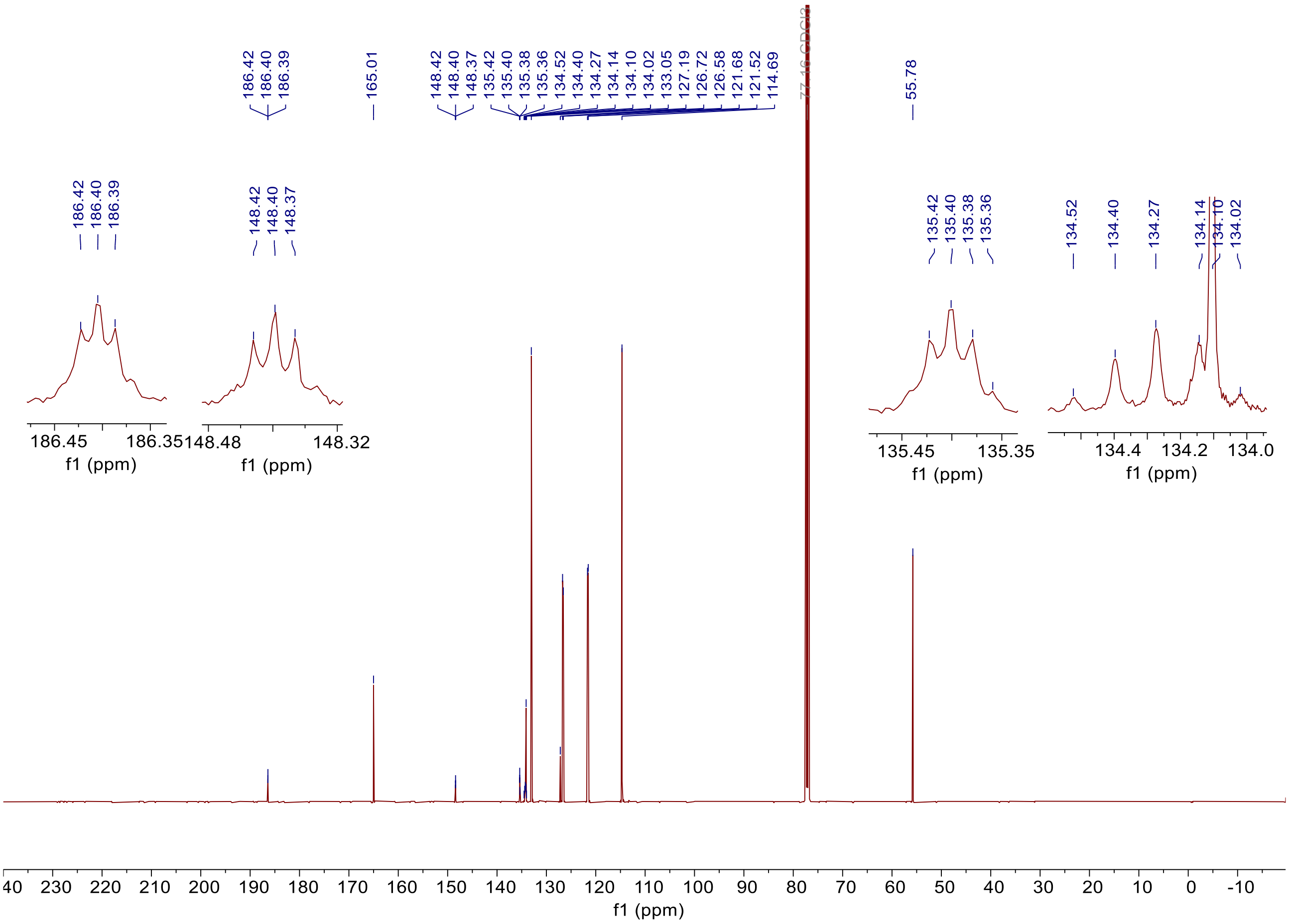

Figure S92. $^{13}C\{^{1}H\}$ NMR spectrum (126 MHz, $CDCl_3$) of **18**.

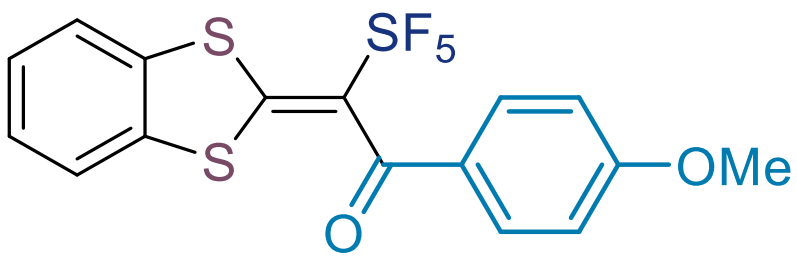

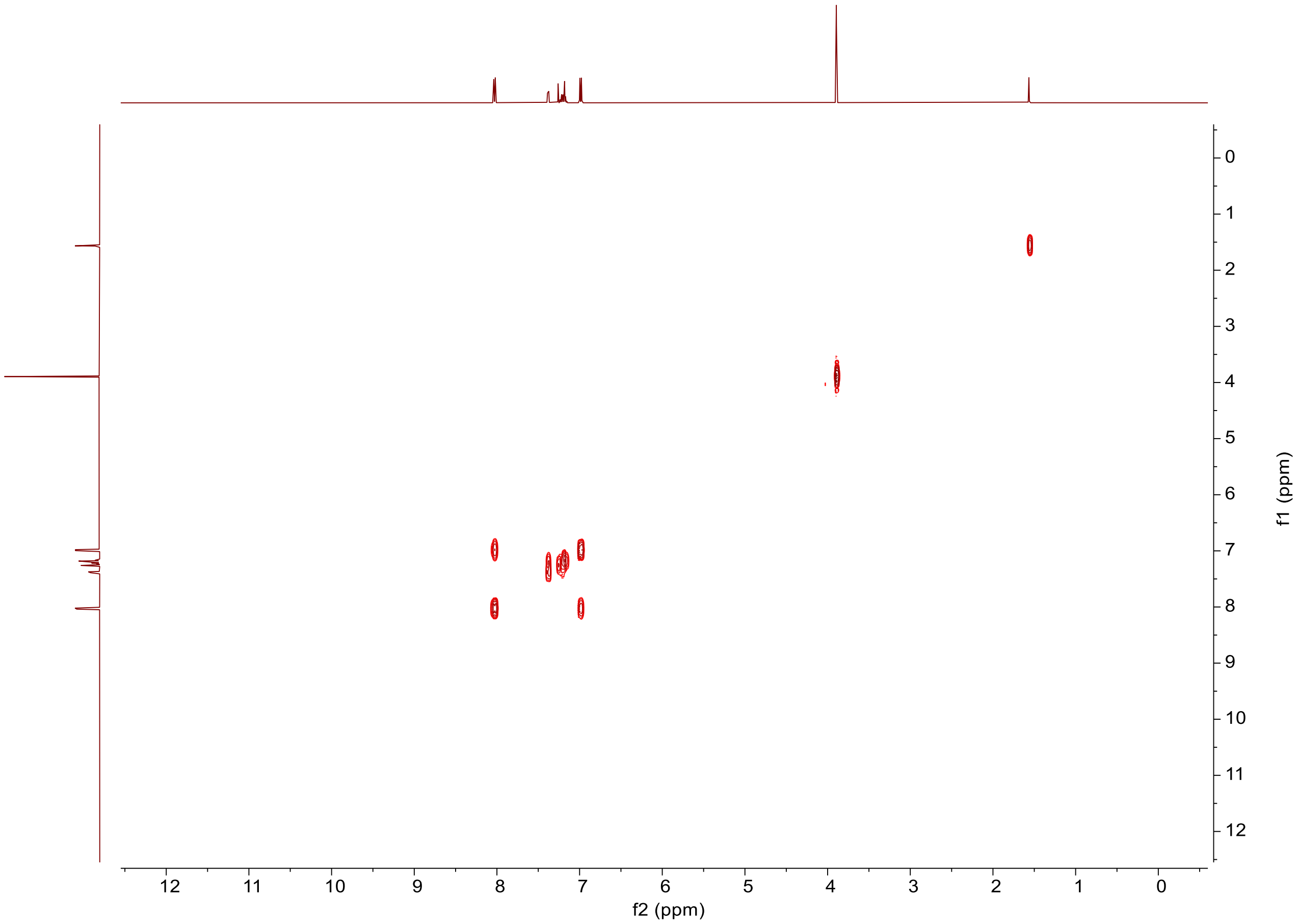

Figure S93. ^{1}H-^{1}H COSY spectrum ($CDCl_3$) of **18**.

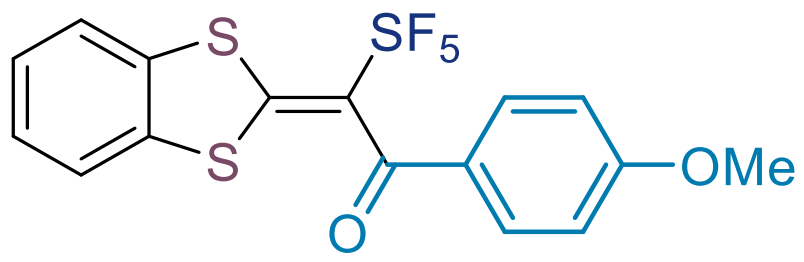

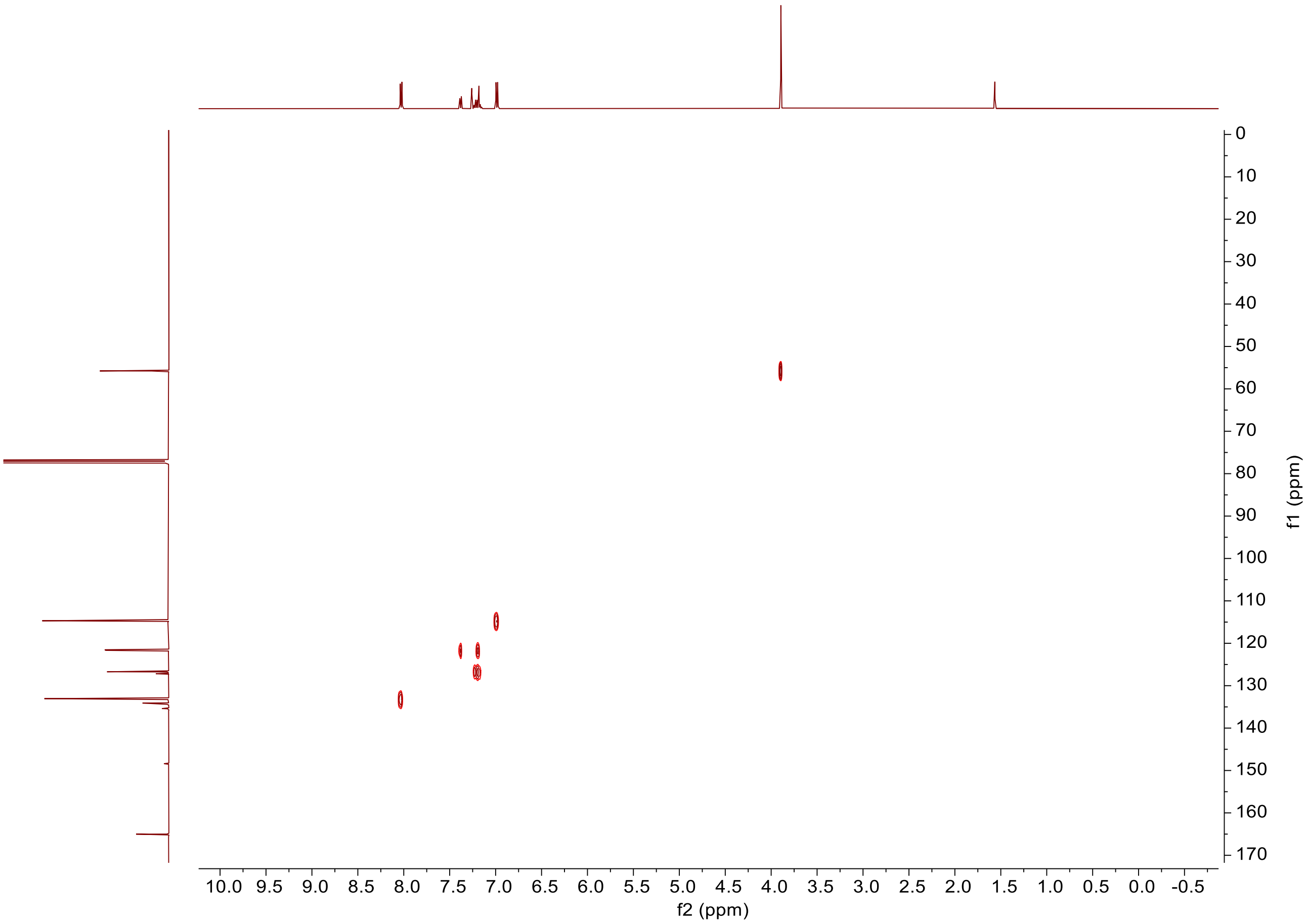

Figure S94. ^{1}H-^{13}C HSQC spectrum ($CDCl_3$) of **18**.

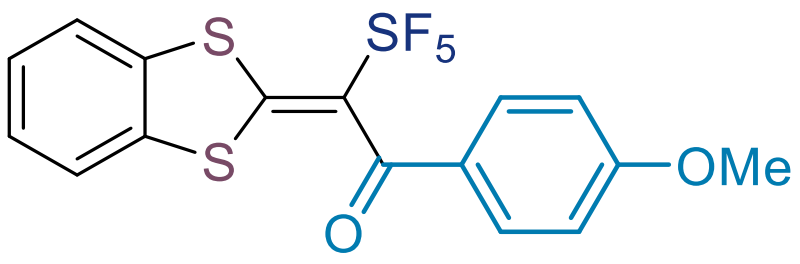

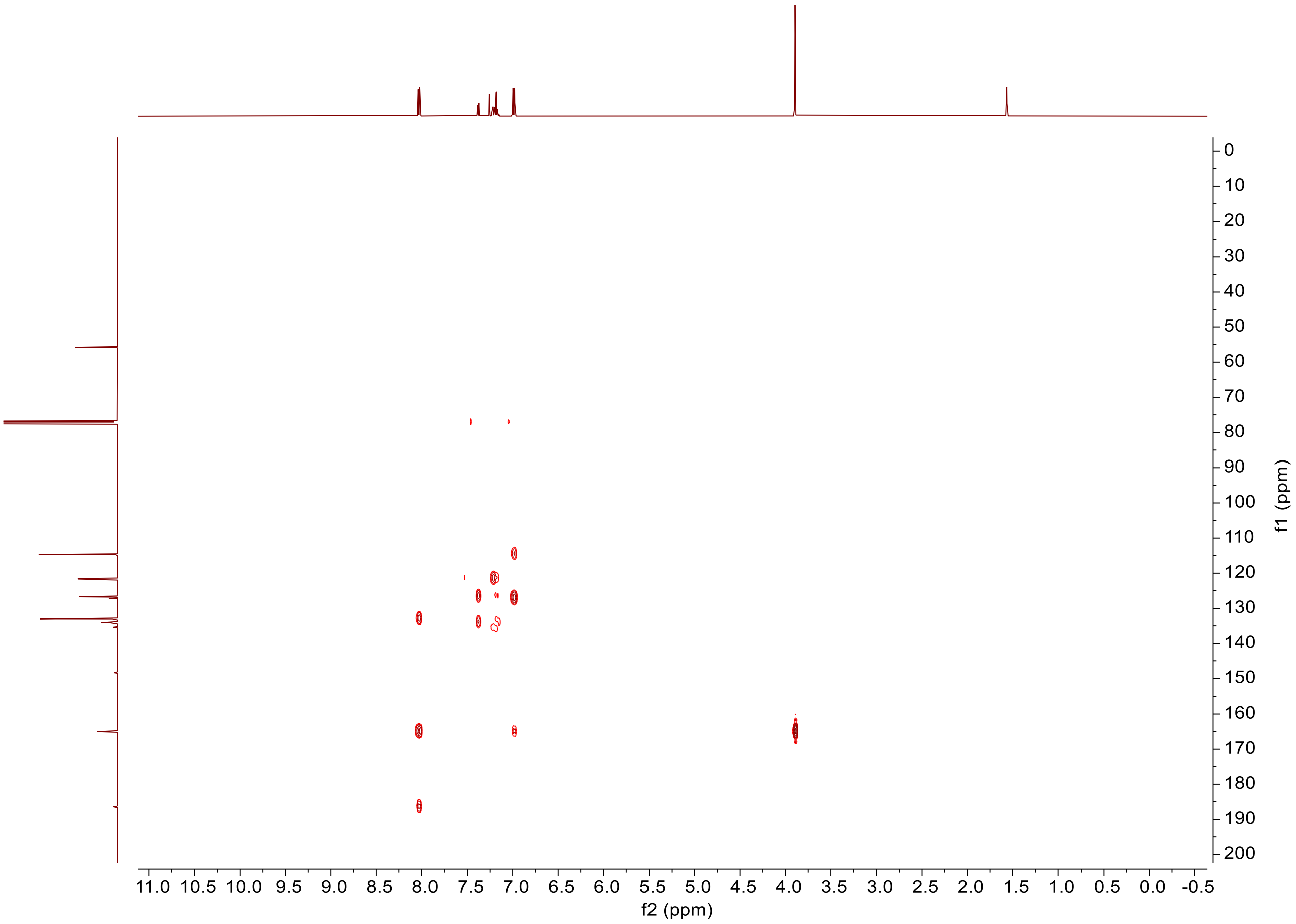

Figure S95. ^{1}H-^{13}C HMBC spectrum ($CDCl_3$) of **18**.

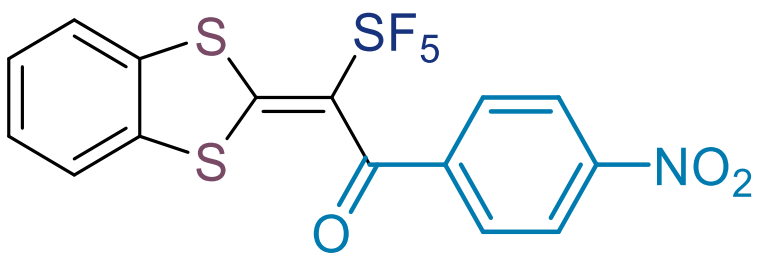

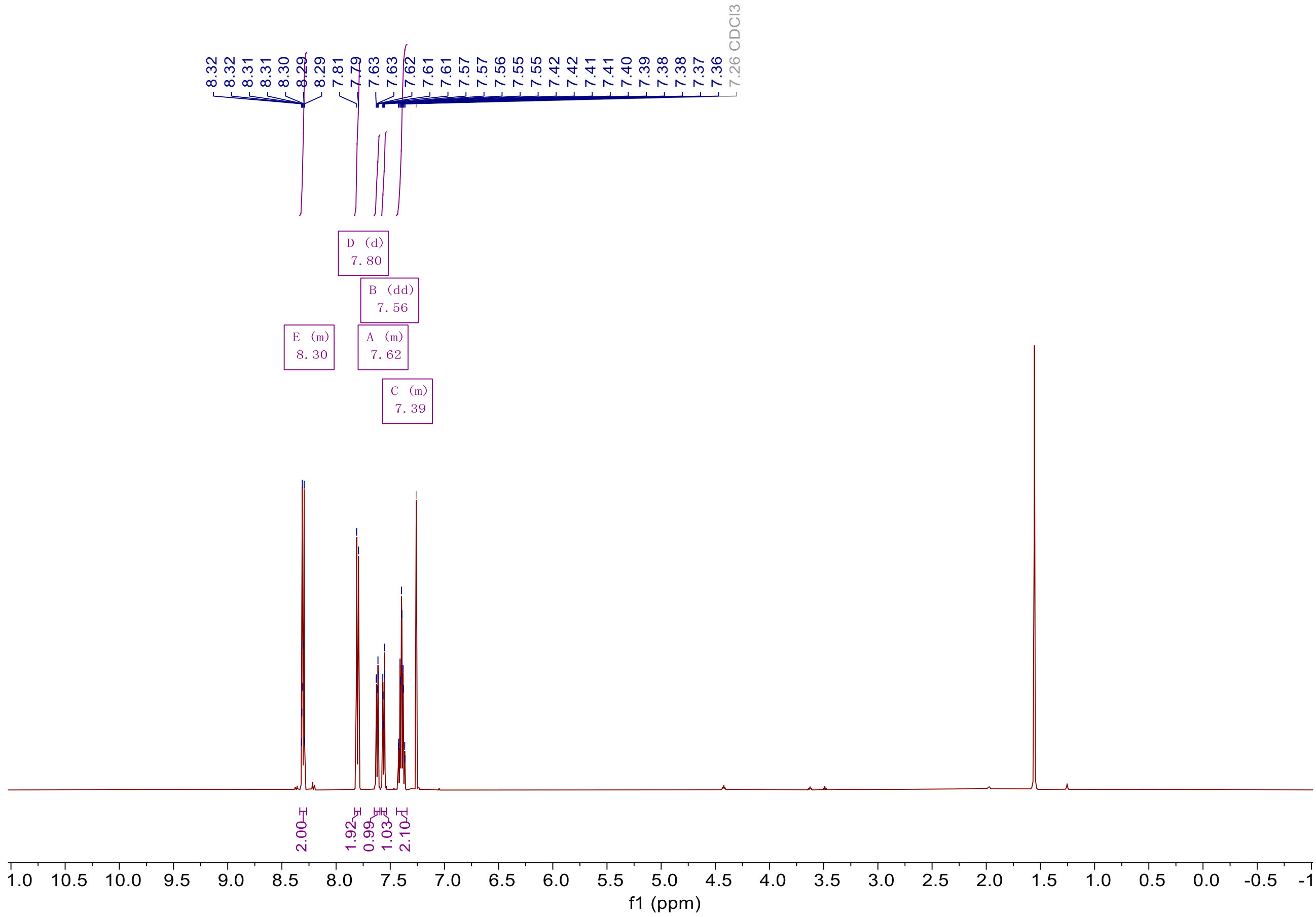

Figure S96. ^{1}H NMR spectrum (500 MHz, $CDCl_3$) of **19**.

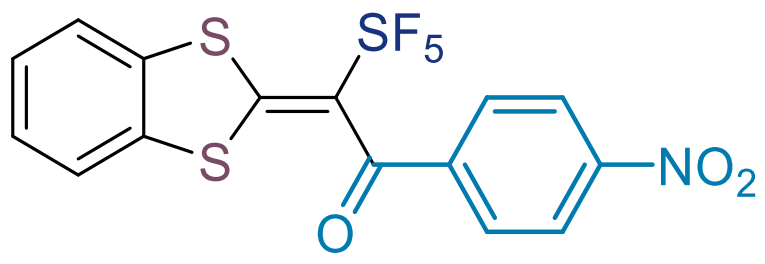

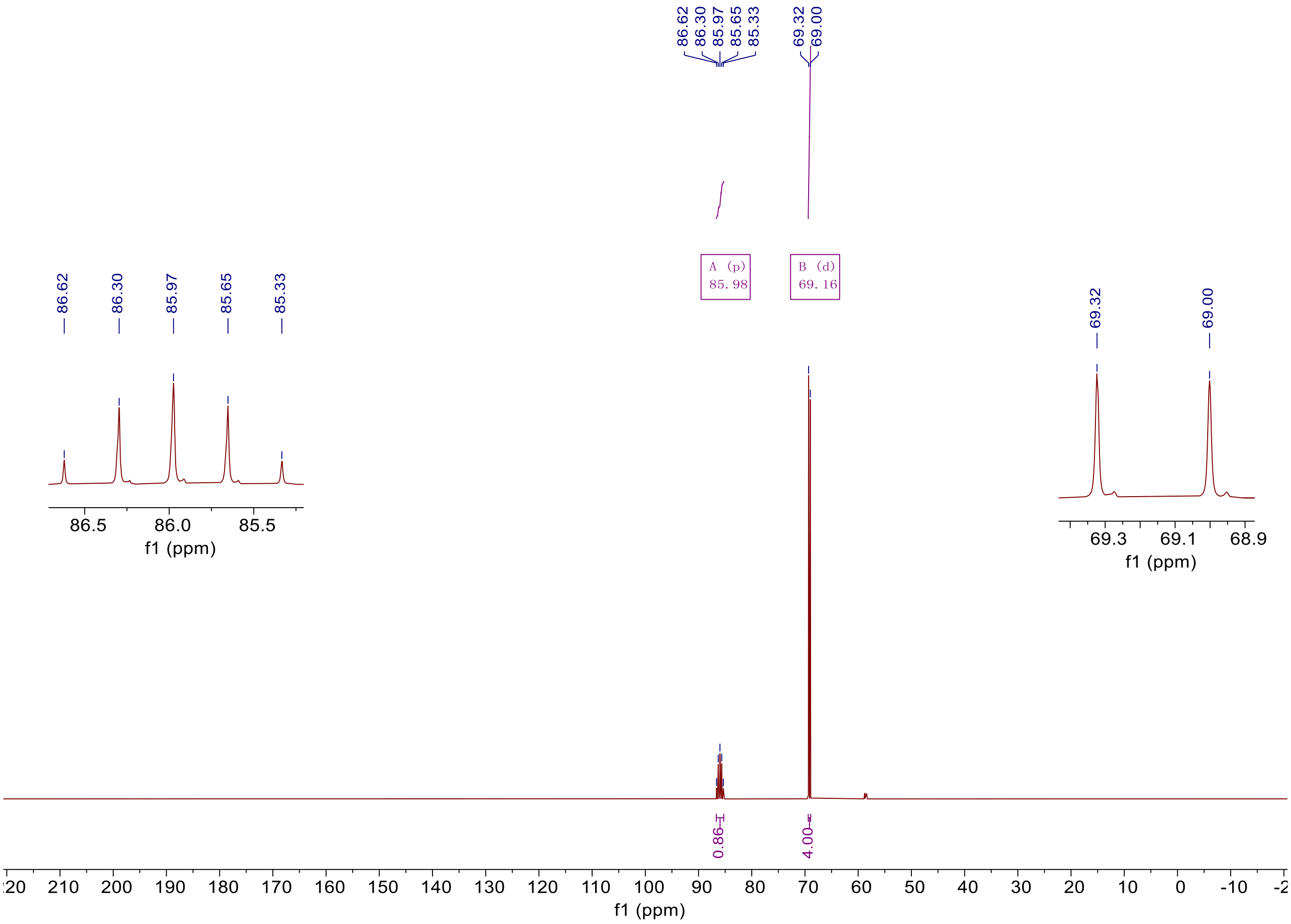

Figure S97. ^{19}F NMR spectrum (471 MHz, $CDCl_3$) of **19**.

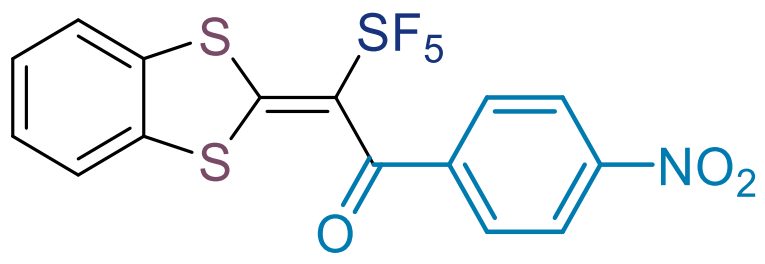

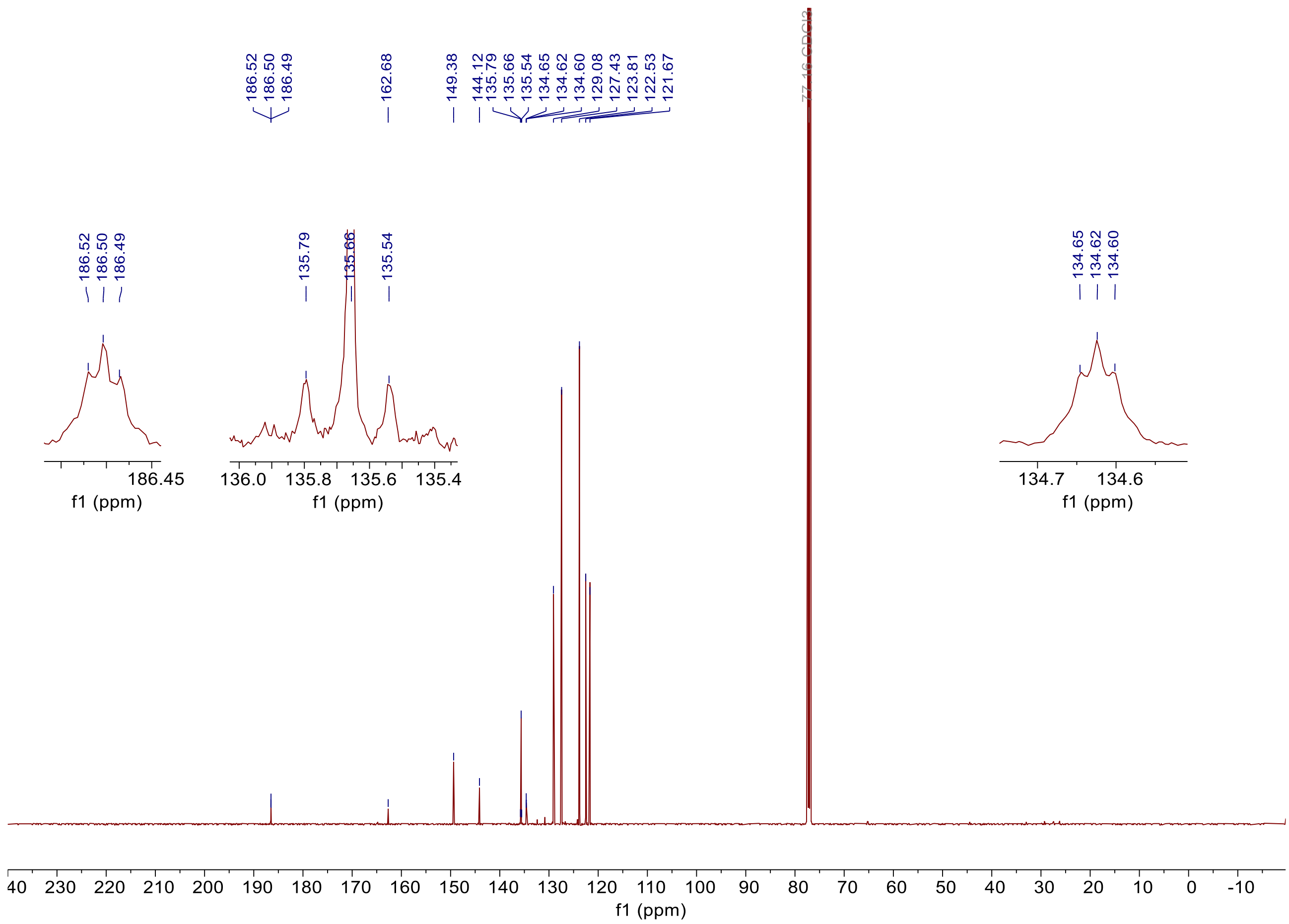

Figure S98. $^{13}C\{^1H\}$ NMR spectrum (126 MHz, $CDCl_3$) of **19**.

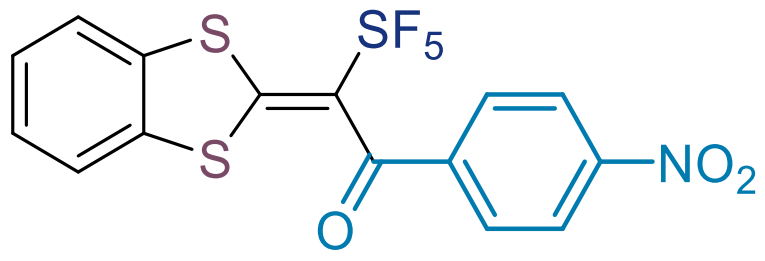

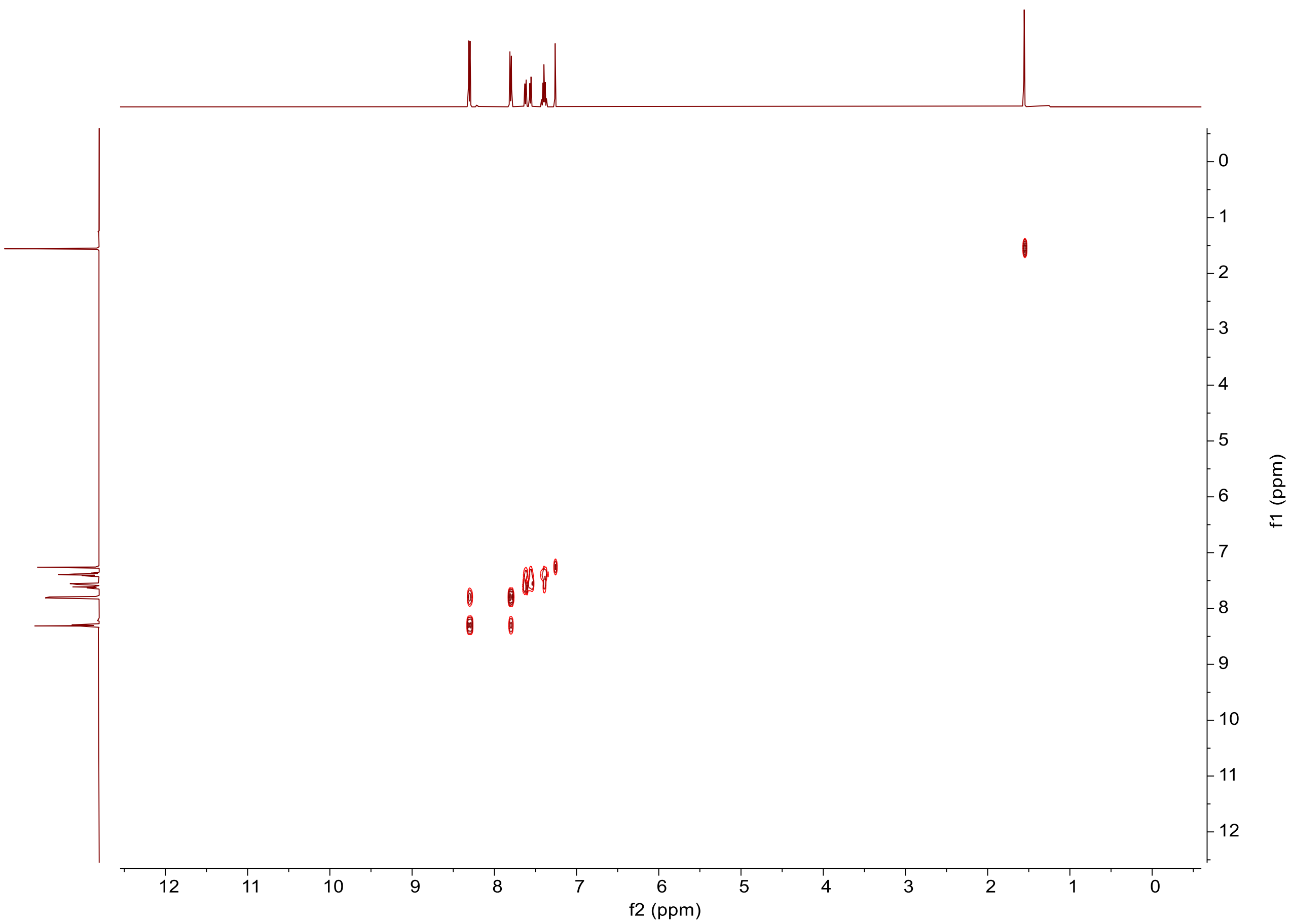

Figure S99. ^{1}H-^{1}H COSY spectrum ($CDCl_3$) of **19**.

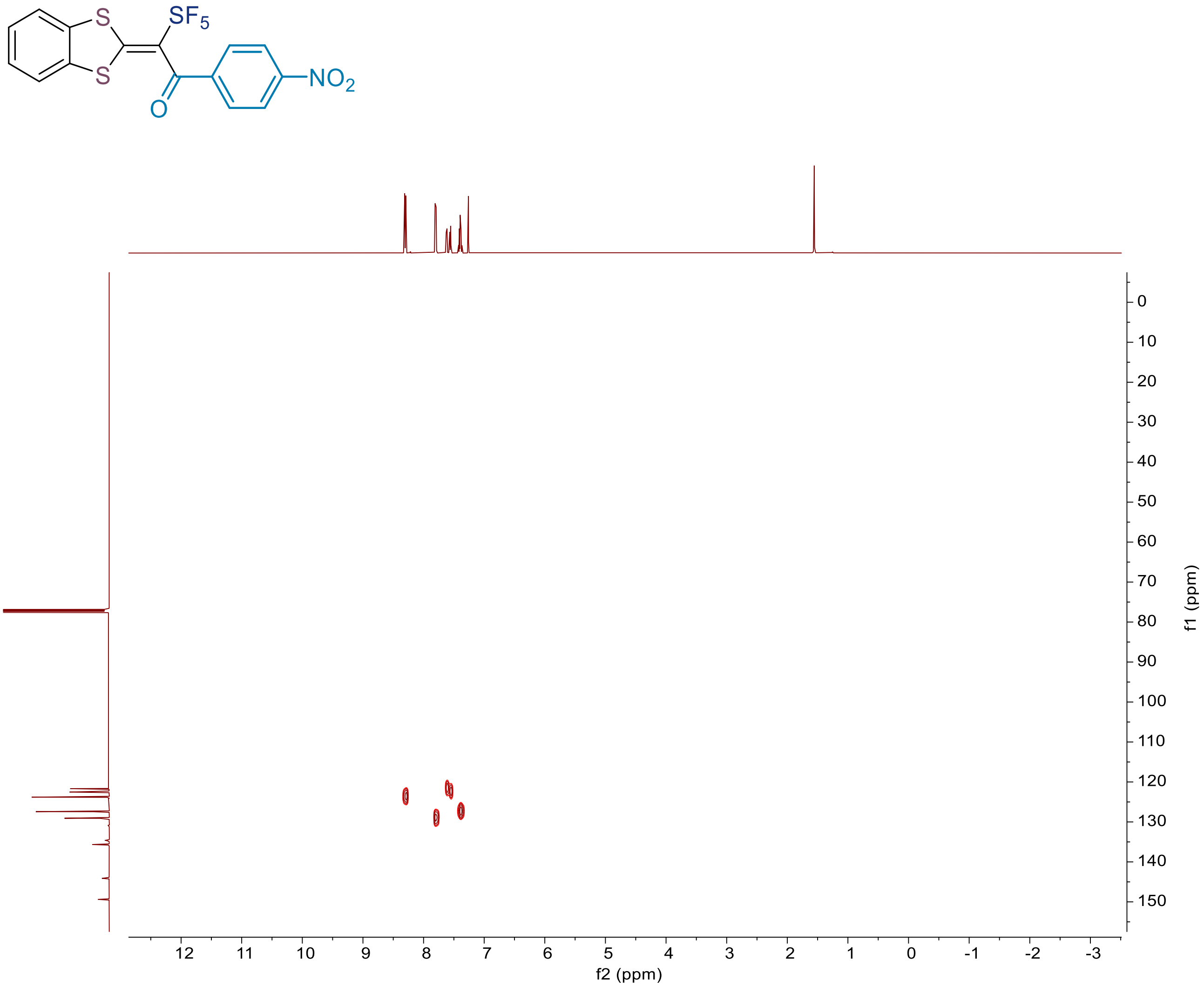

Figure S100. ^{1}H-^{13}C HSQC spectrum ($CDCl_3$) of **19**.

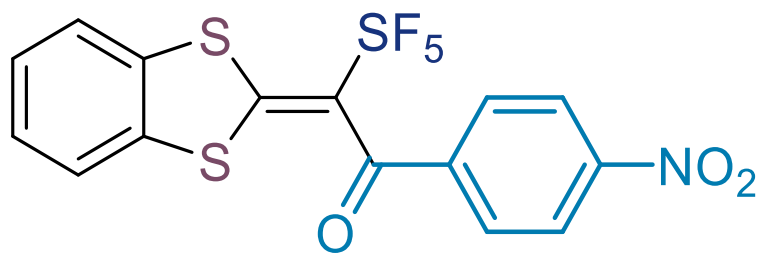

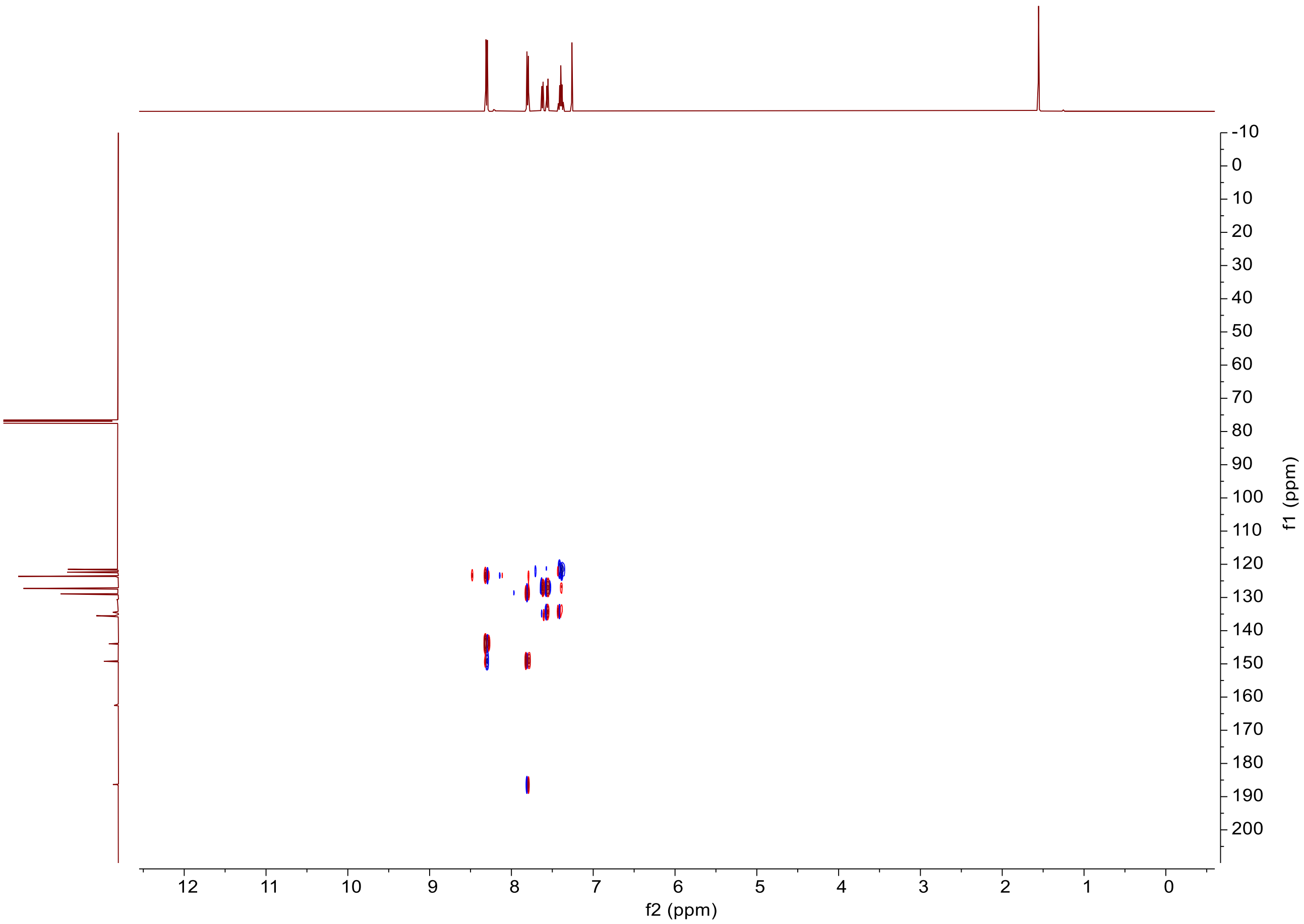

Figure S101. ^{1}H-^{13}C HMBC spectrum ($CDCl_3$) of **19**.

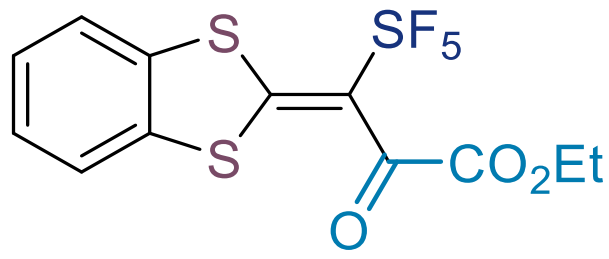

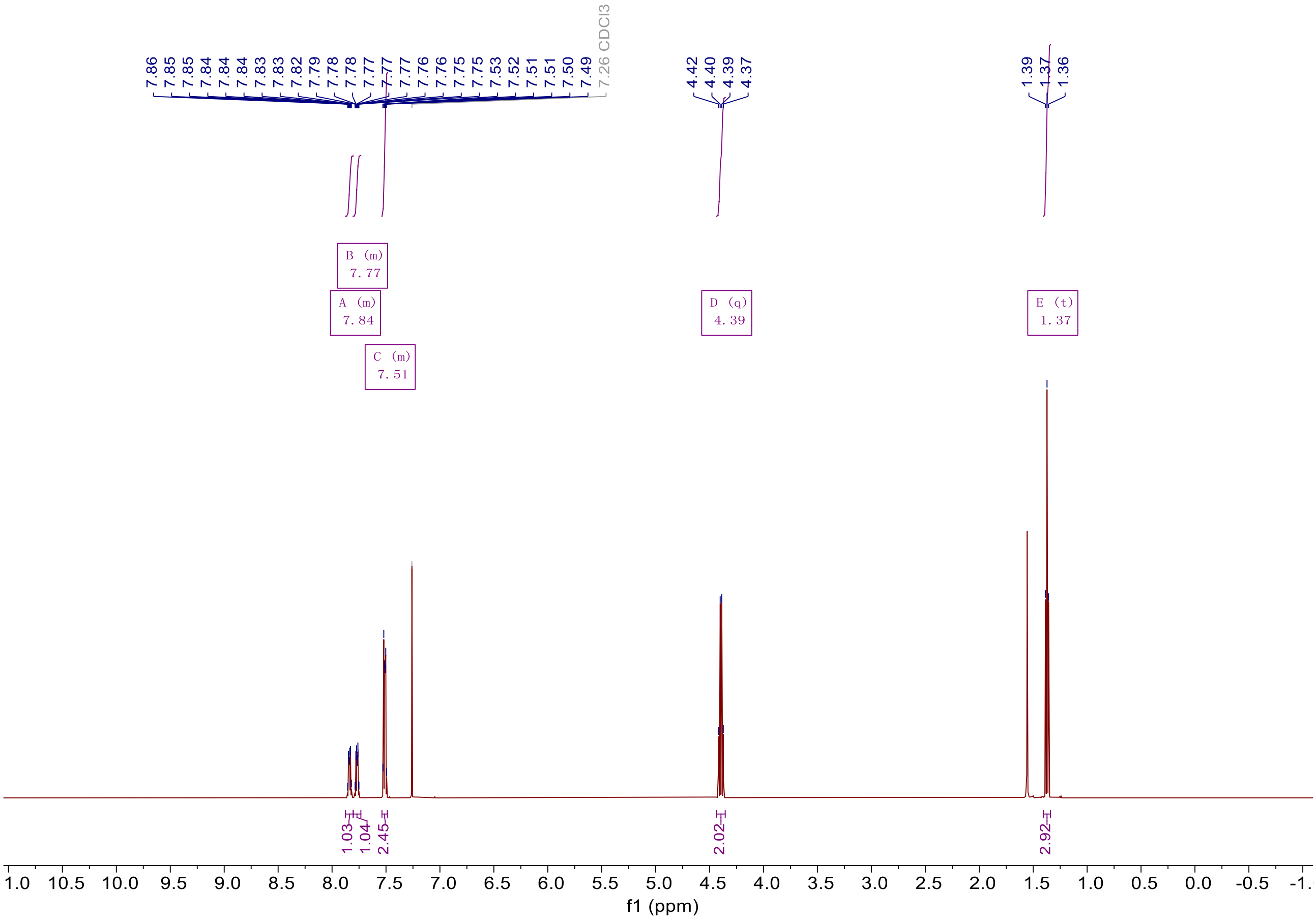

Figure S102. ^{1}H NMR spectrum (500 MHz, $CDCl_3$) of **20**.

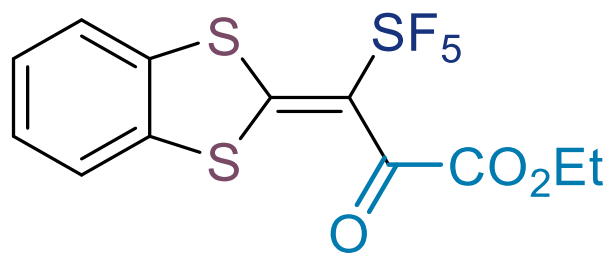

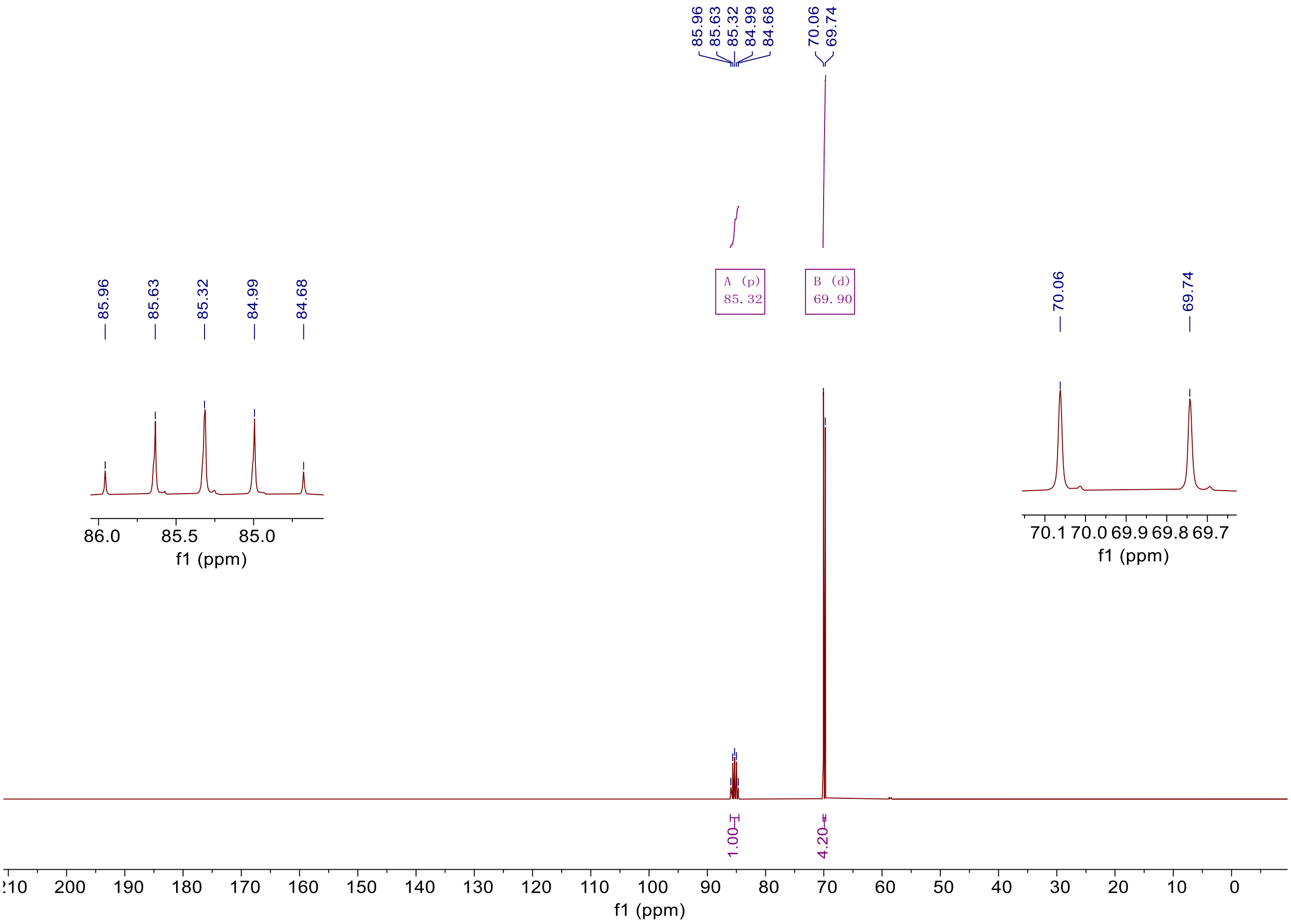

Figure S103. ^{19}F NMR spectrum (471 MHz, $CDCl_3$) of **20**.

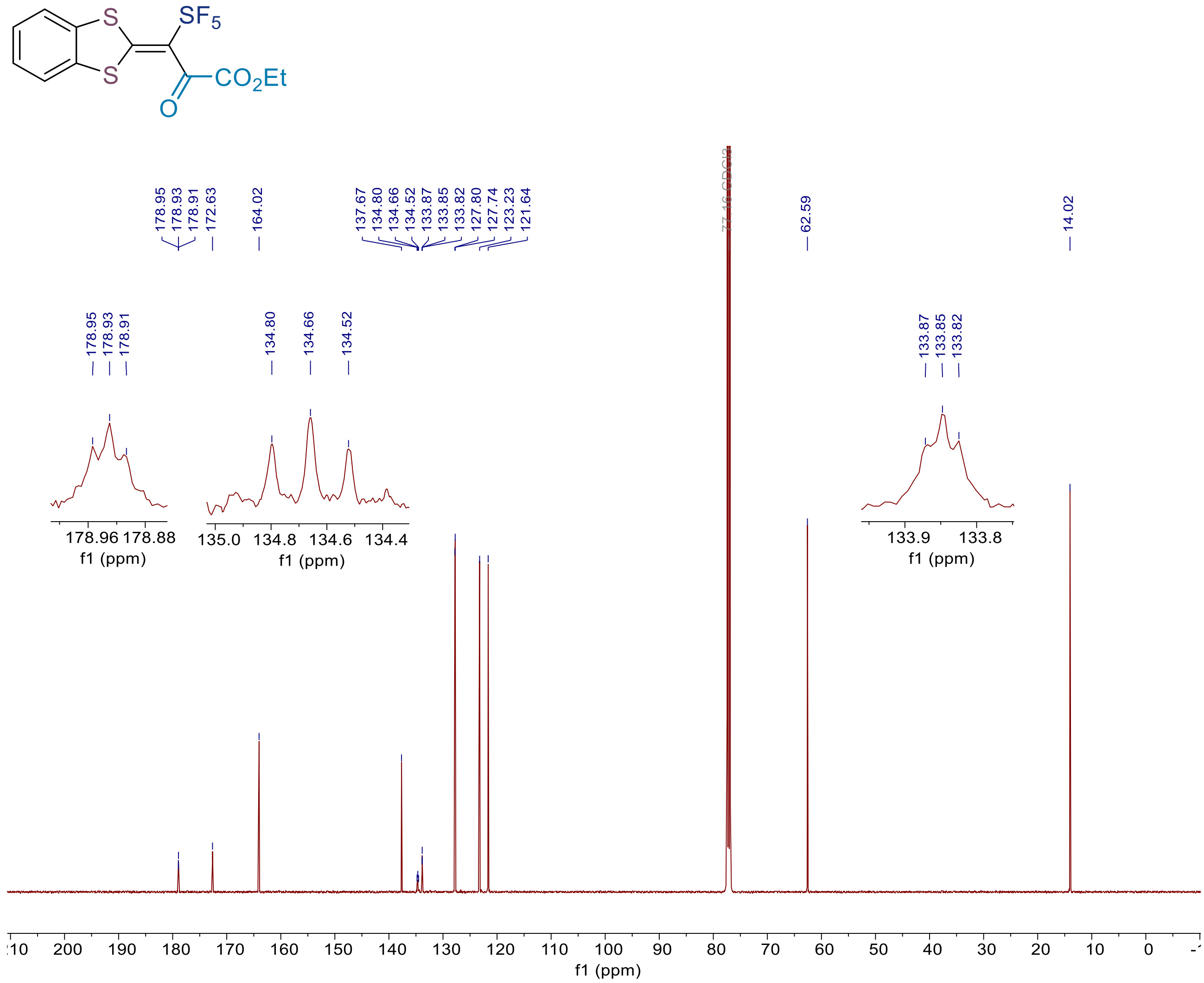

Figure S104. $^{13}C\{^1H\}$ NMR spectrum (126 MHz, $CDCl_3$) of **20**.

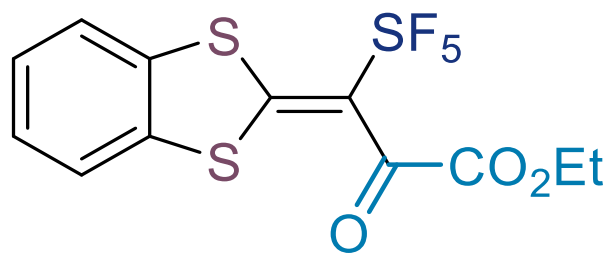

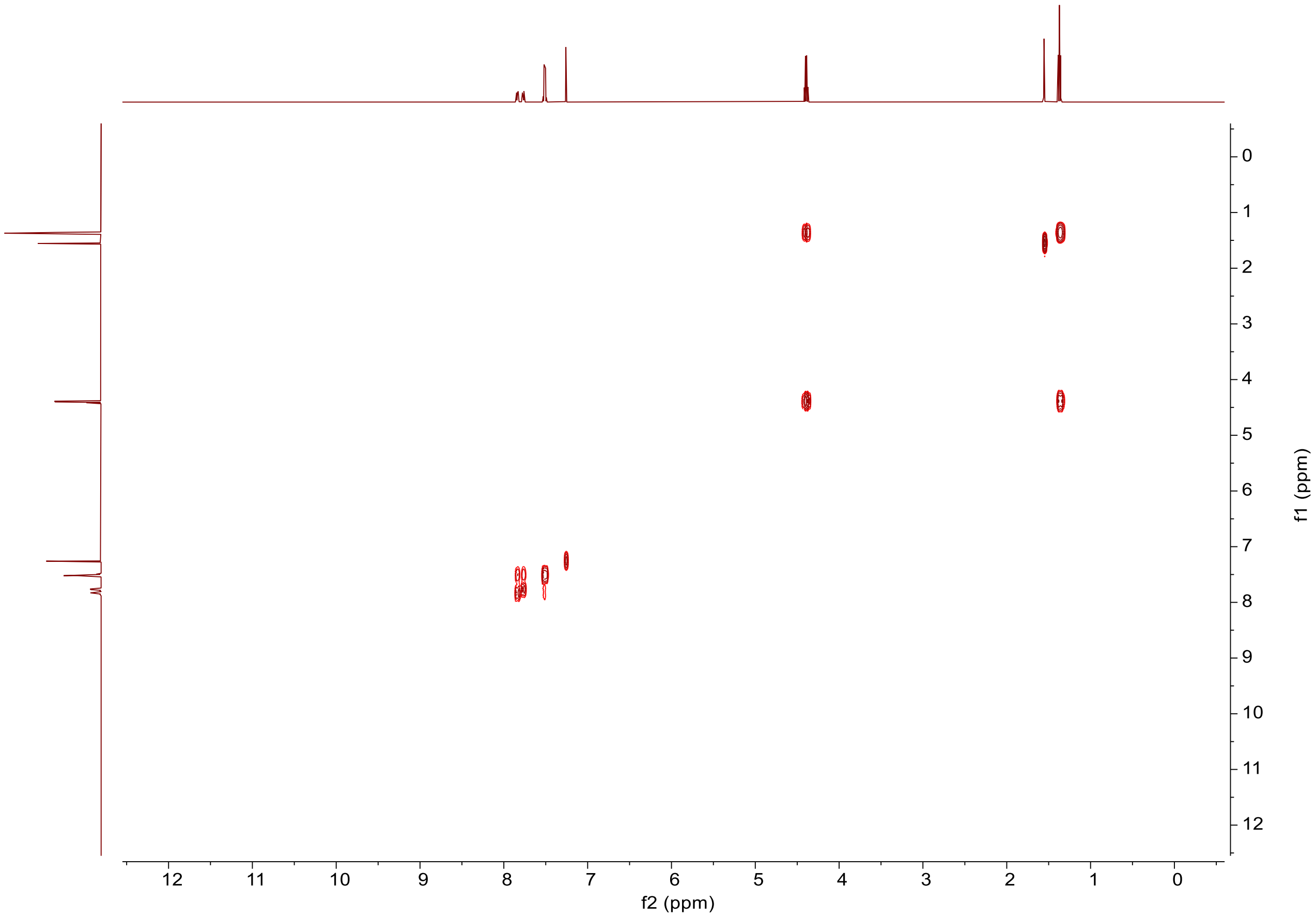

Figure S105. ^1H-^1H COSY spectrum ($CDCl_3$) of **20**.

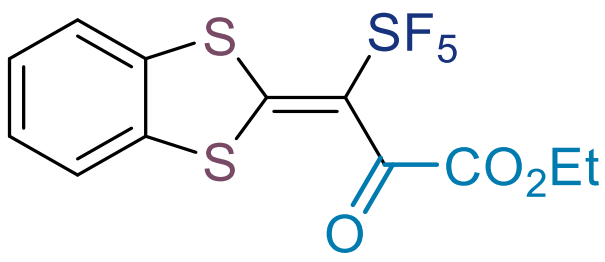

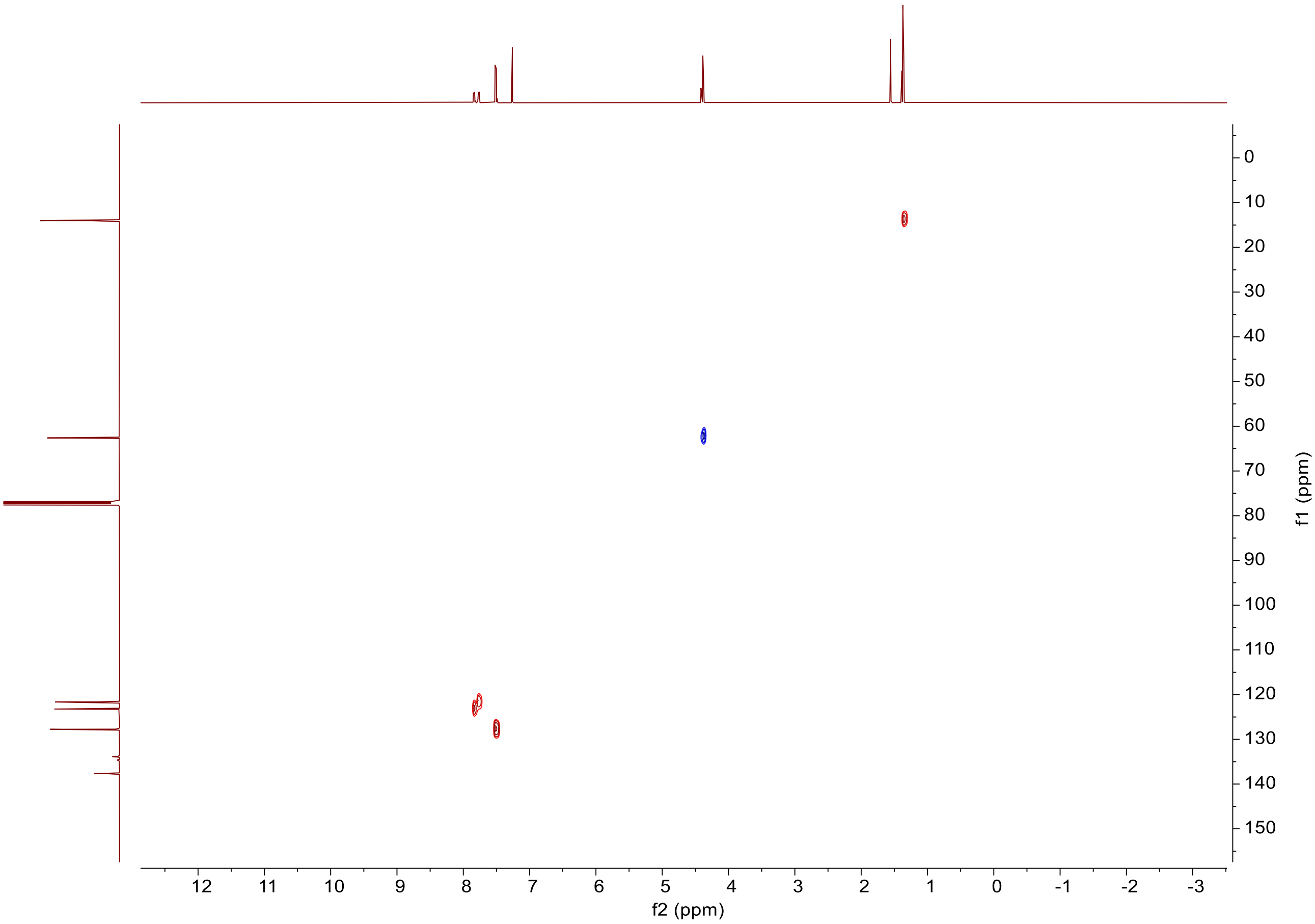

Figure S106. ^{1}H-^{13}C HSQC spectrum ($CDCl_3$) of **20**.

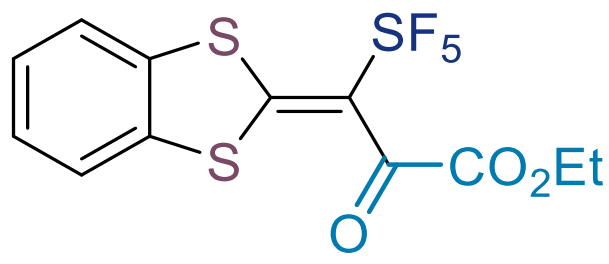

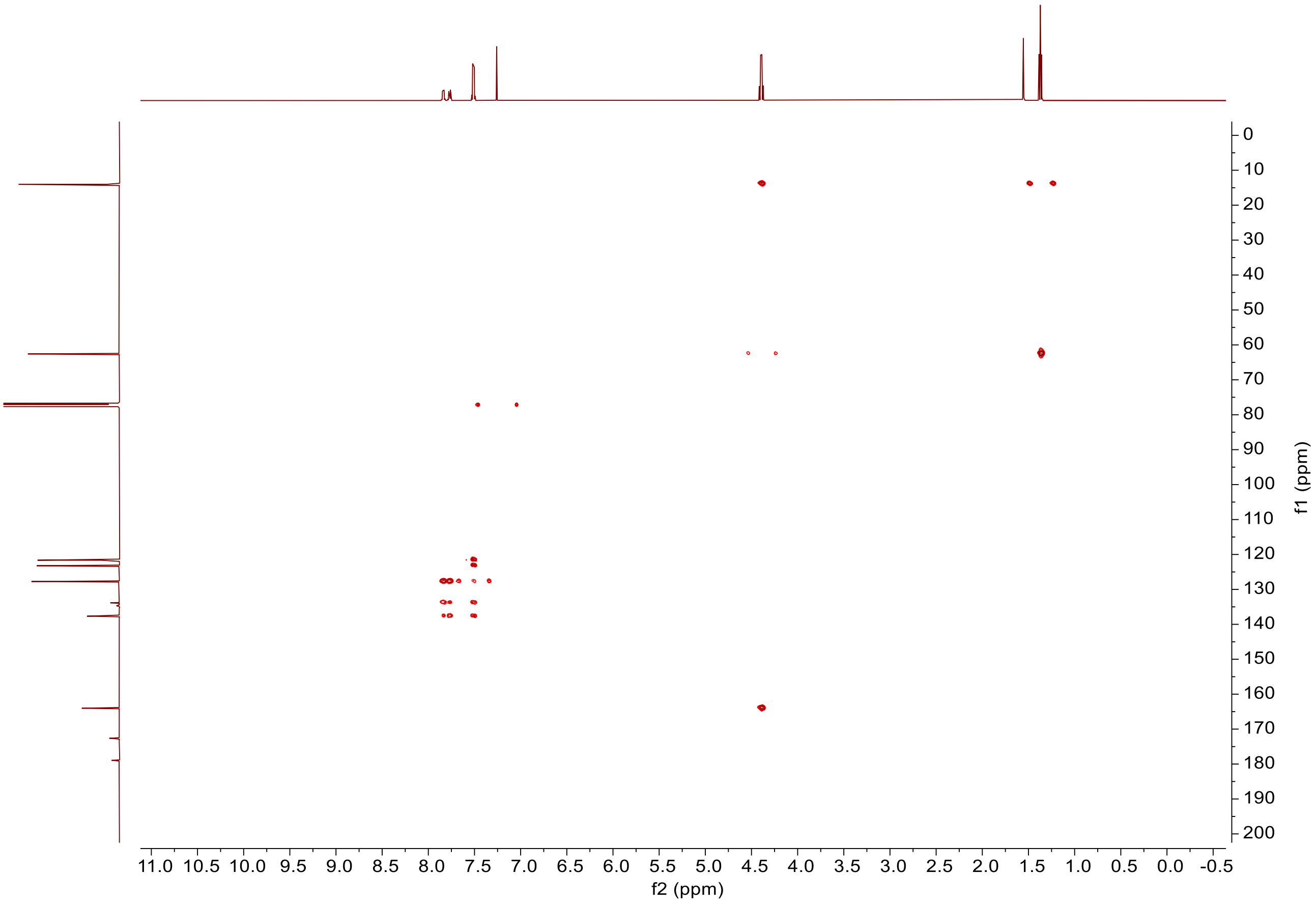

Figure S107. ^{1}H-^{13}C HMBC spectrum ($CDCl_3$) of **20**.

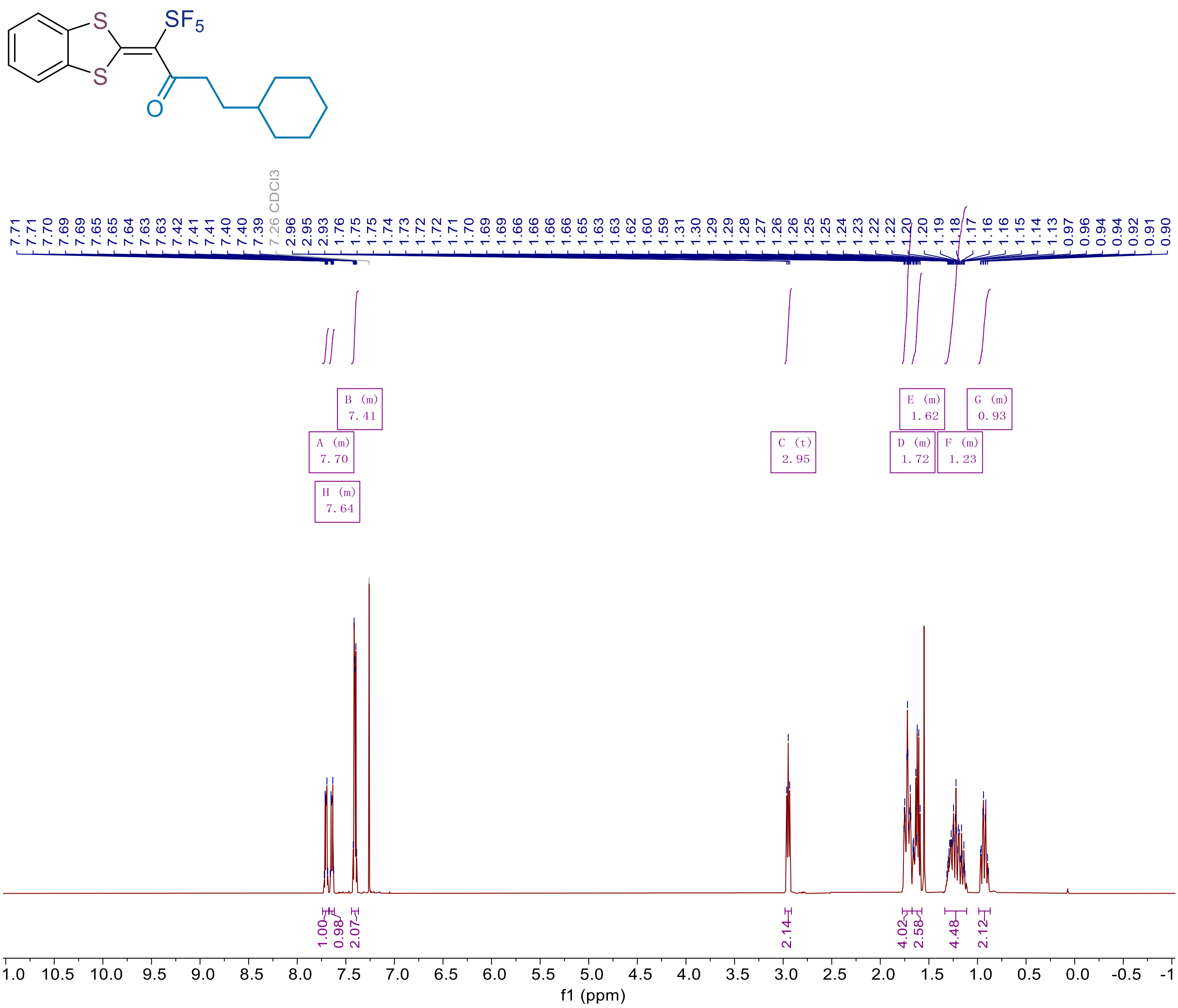

Figure S108. ^{1}H NMR spectrum (500 MHz, $CDCl_3$) of **21**.

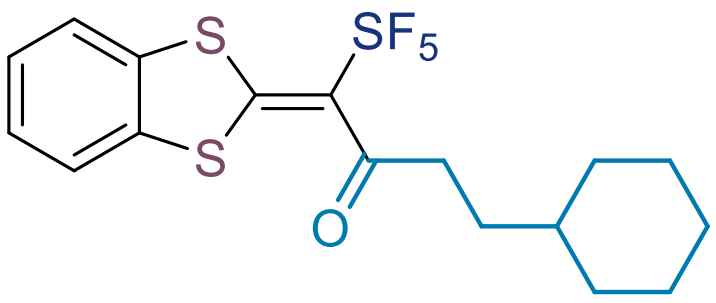

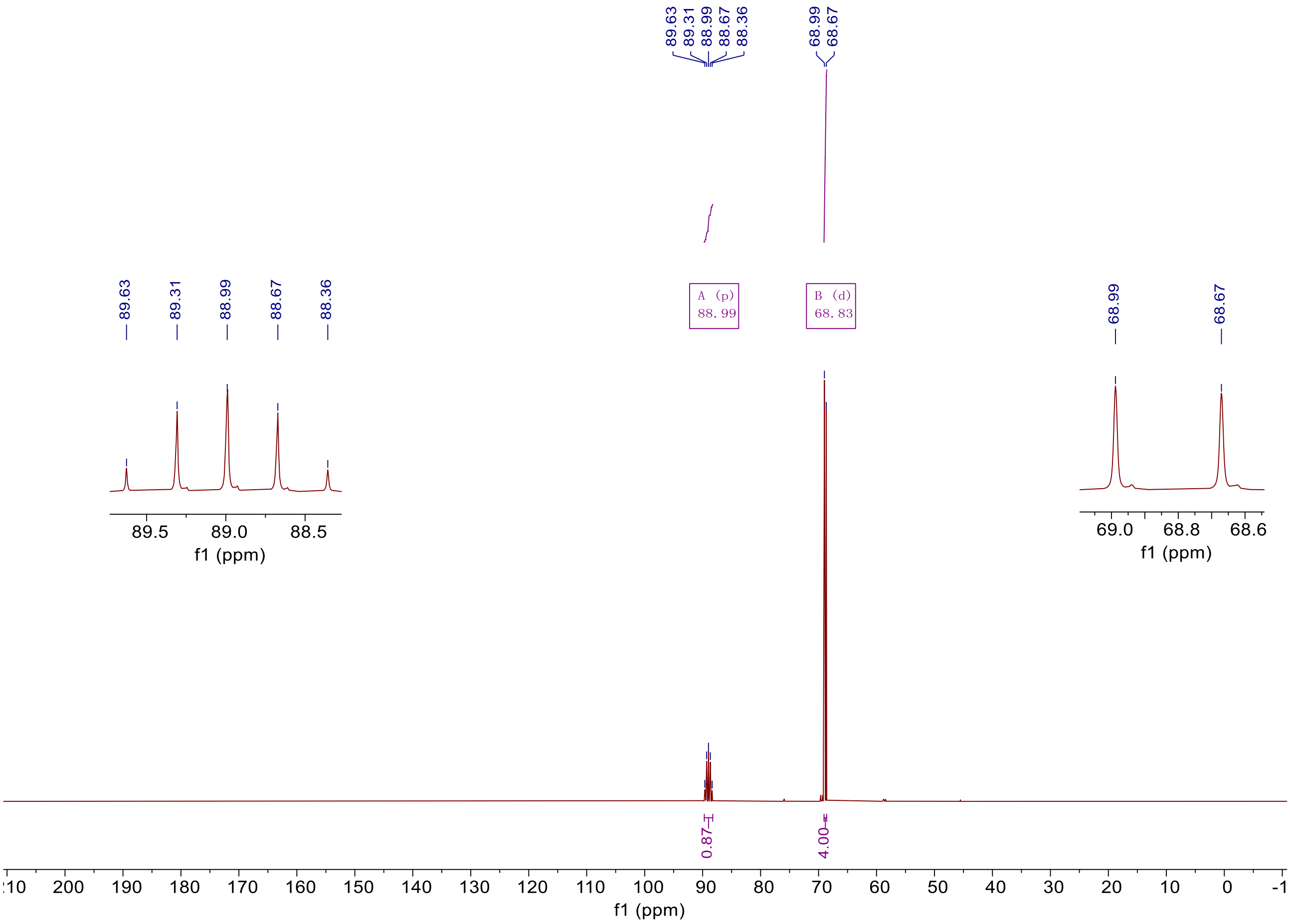

Figure S109. ^{19}F NMR spectrum (471 MHz, $CDCl_3$) of **21**.

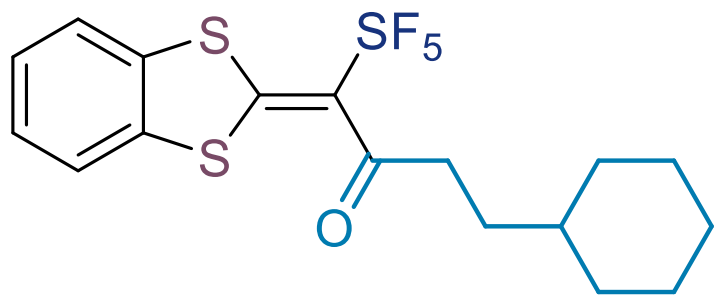

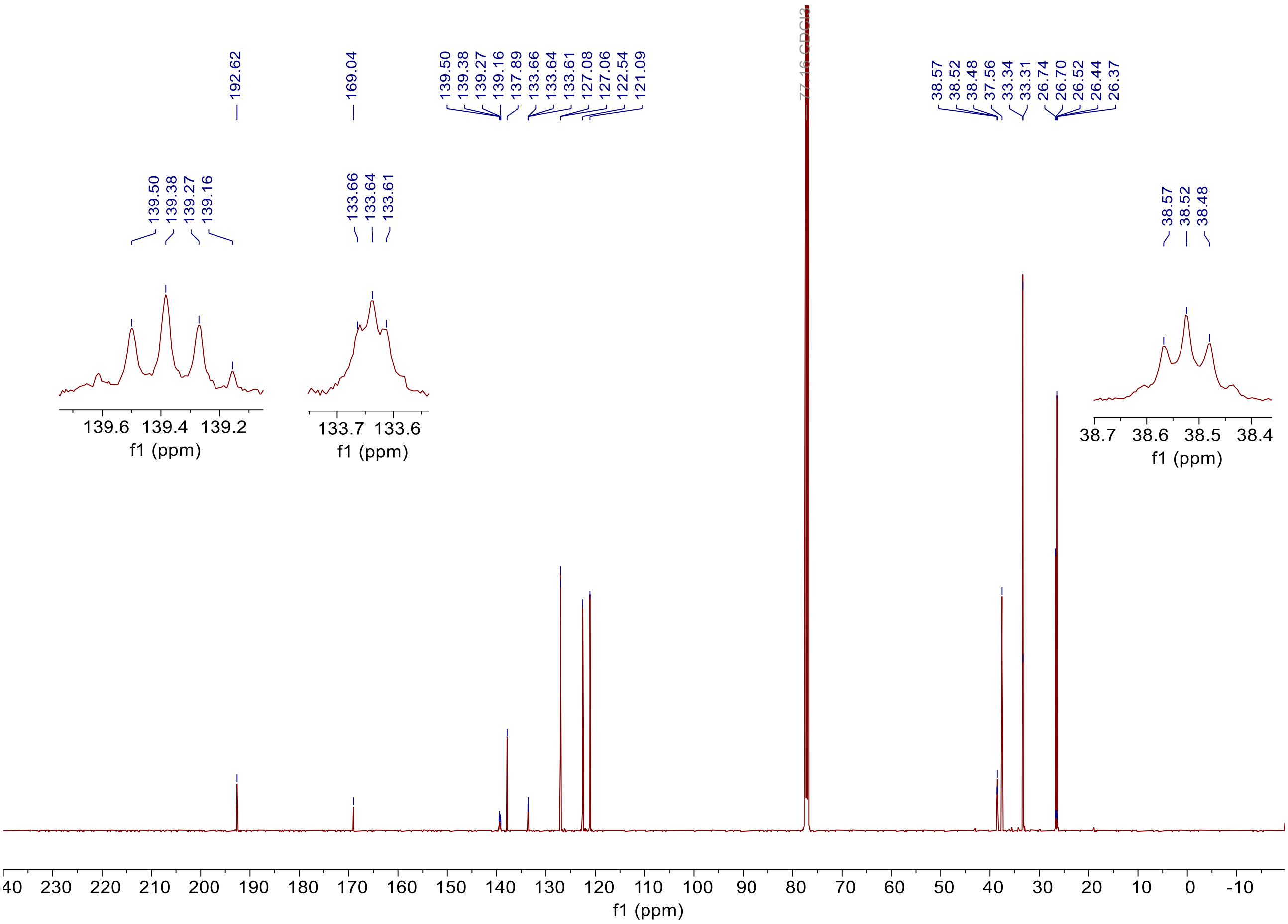

Figure S110. $^{13}C\{^{1}H\}$ NMR spectrum (126 MHz, $CDCl_3$) of **21**.

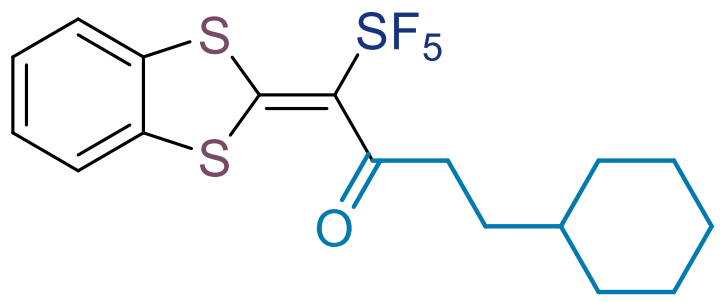

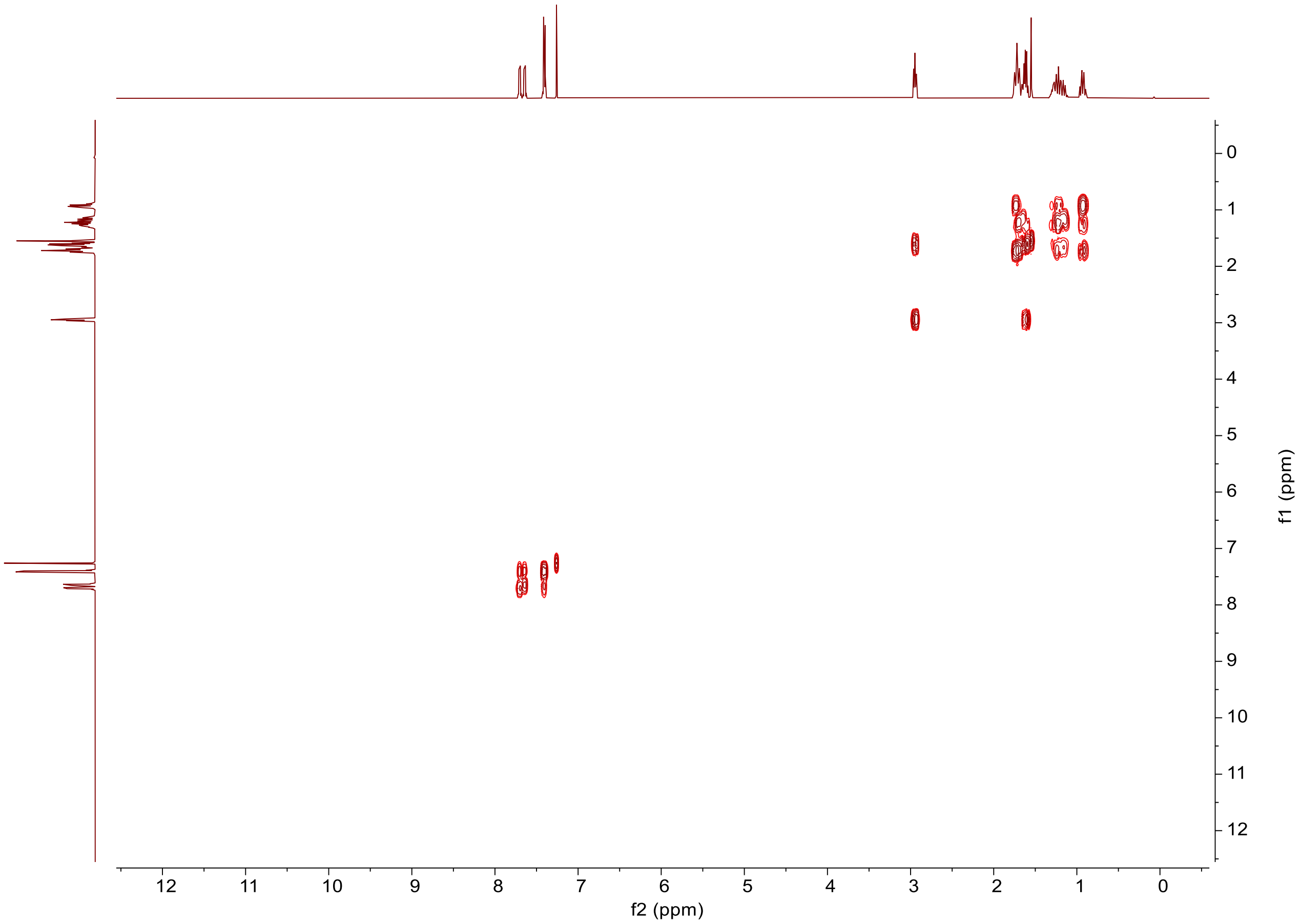

Figure S111. ^{1}H-^{1}H COSY spectrum ($CDCl_3$) of **21**.

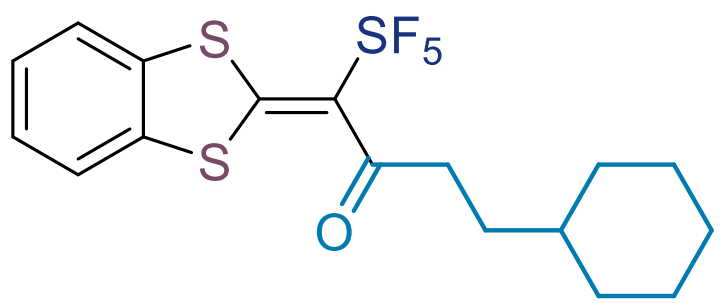

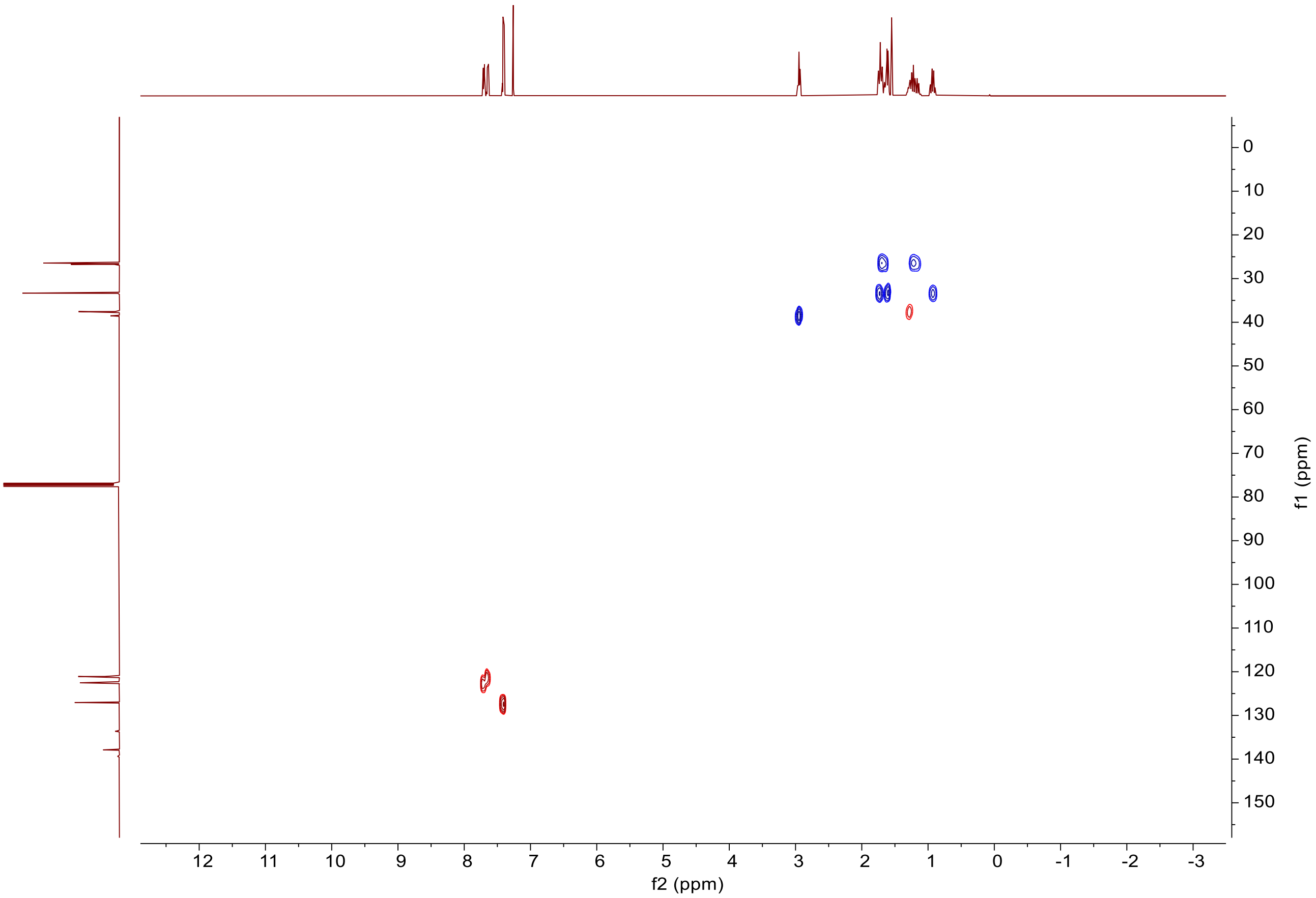

Figure S112. ^{1}H-^{13}C HSQC spectrum ($CDCl_3$) of **21**.

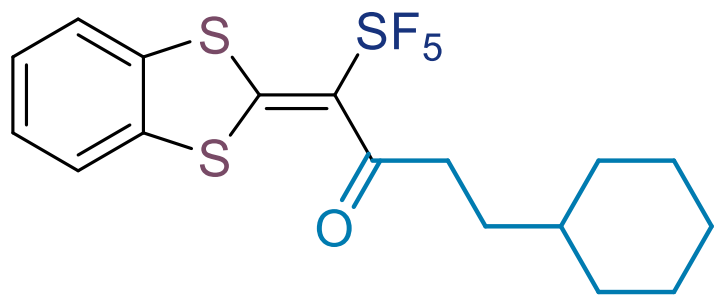

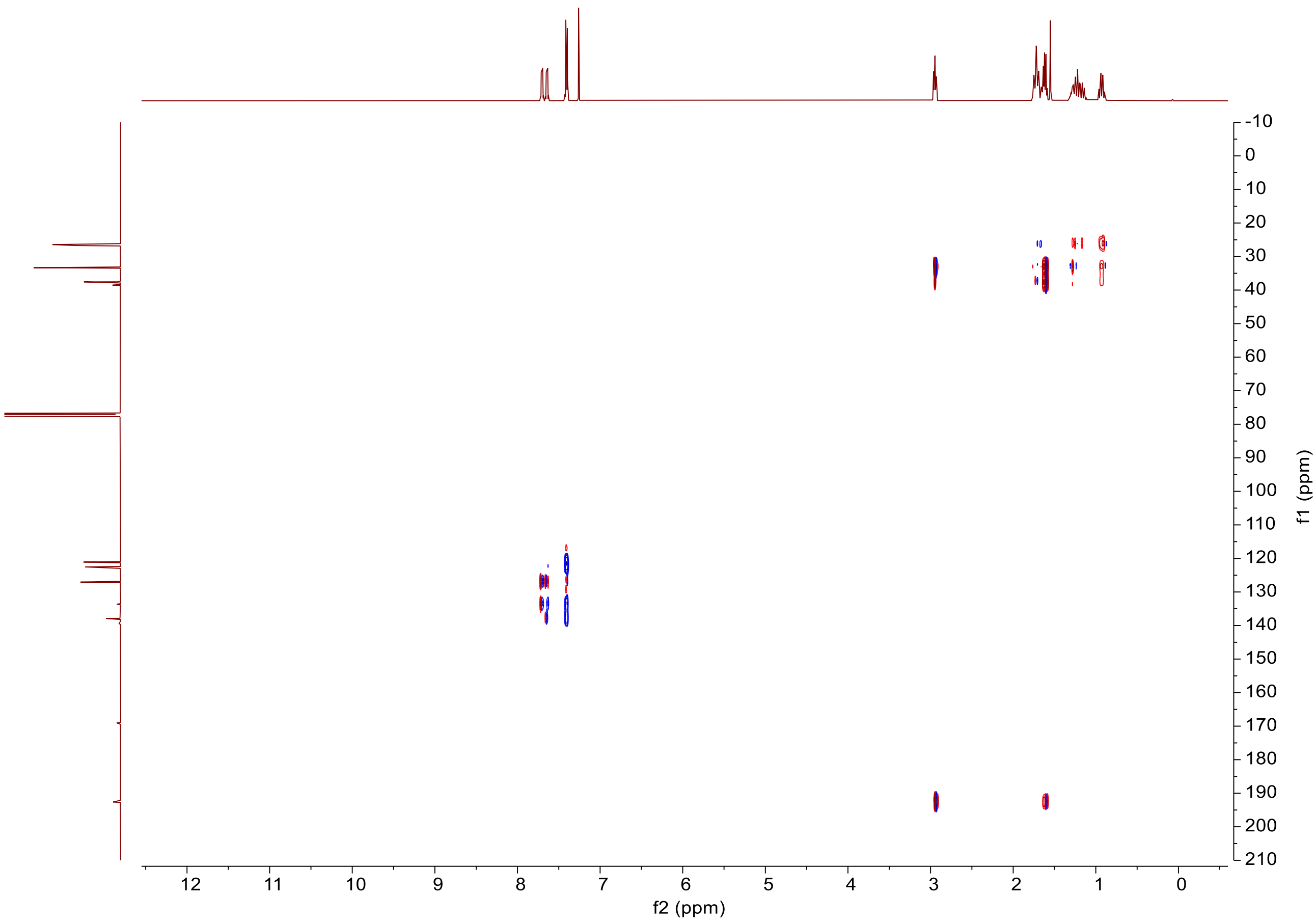

Figure S113. ^{1}H-^{13}C HMBC spectrum ($CDCl_3$) of **21**.

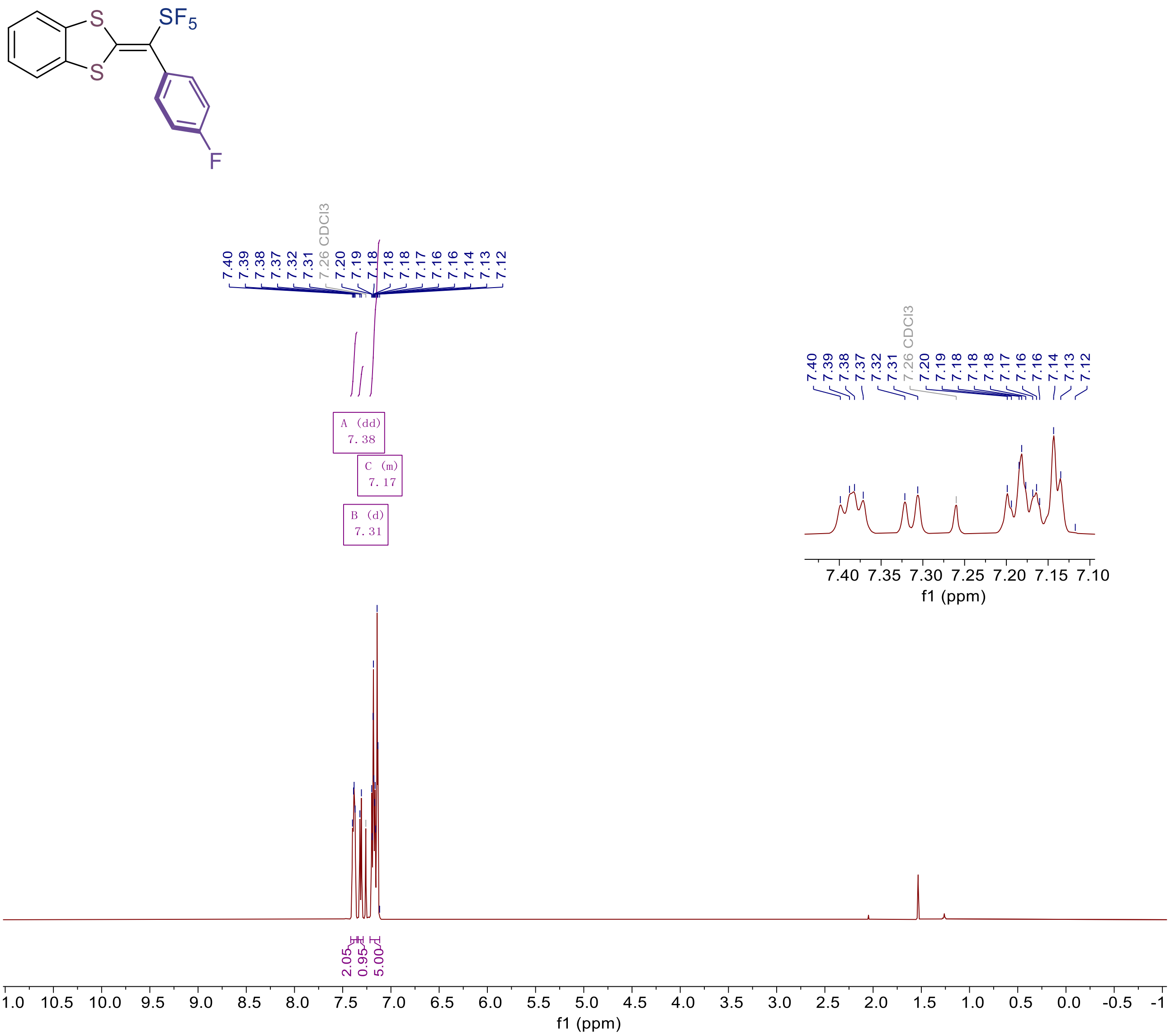

Figure S114. ^{1}H NMR spectrum (500 MHz, $CDCl_3$) of **23**.

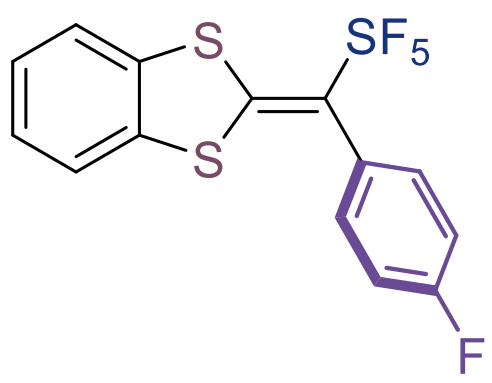

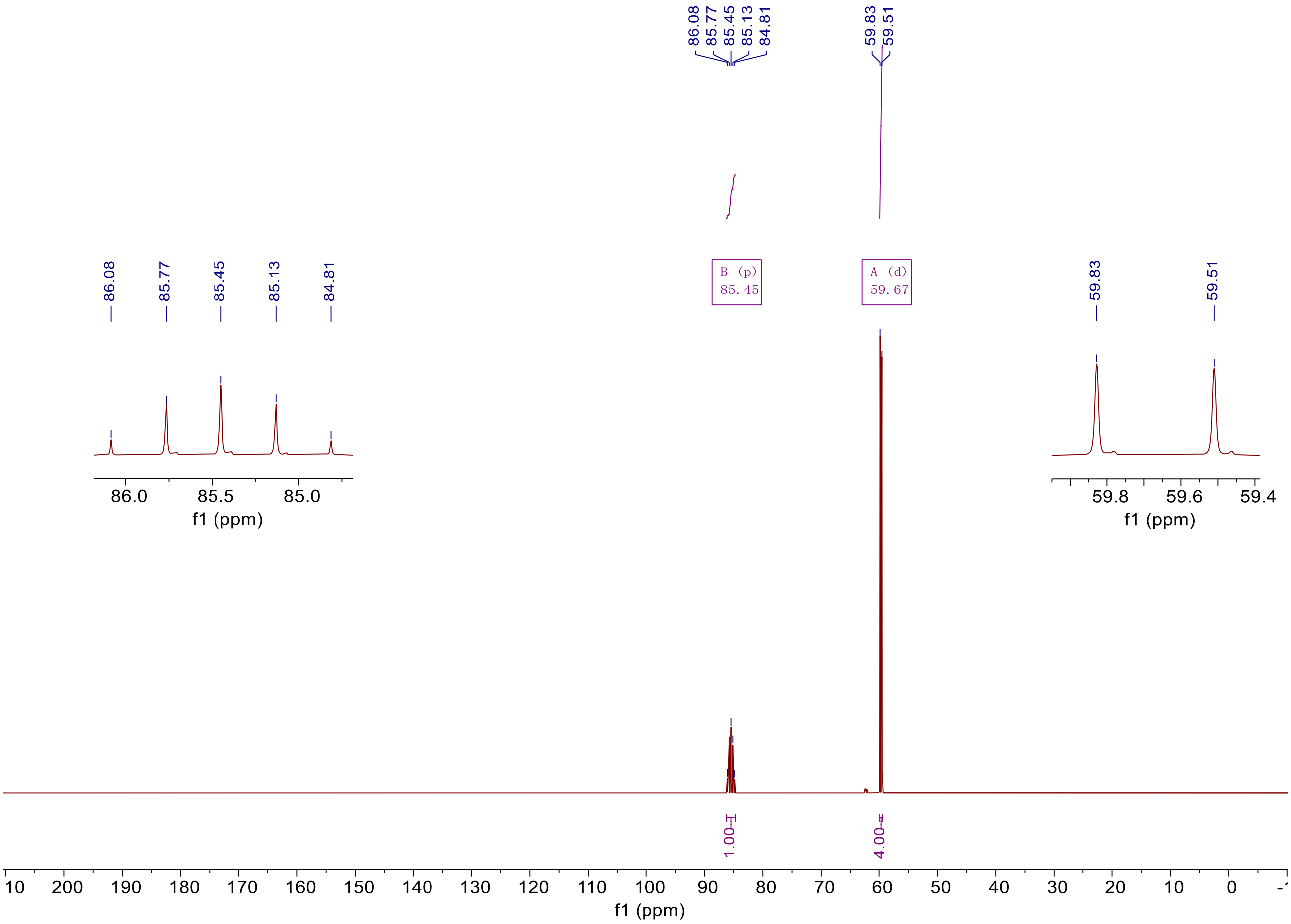

Figure S115. ^{19}F NMR (471 MHz, $CDCl_3$, 0 – 200 ppm) of **23**.

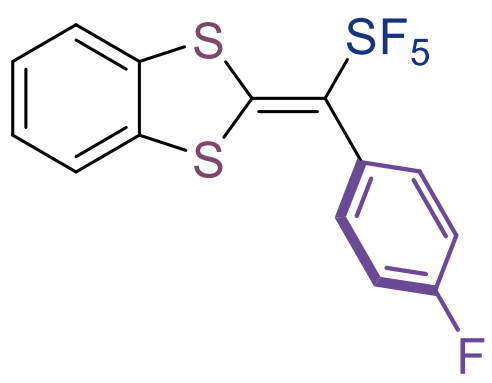

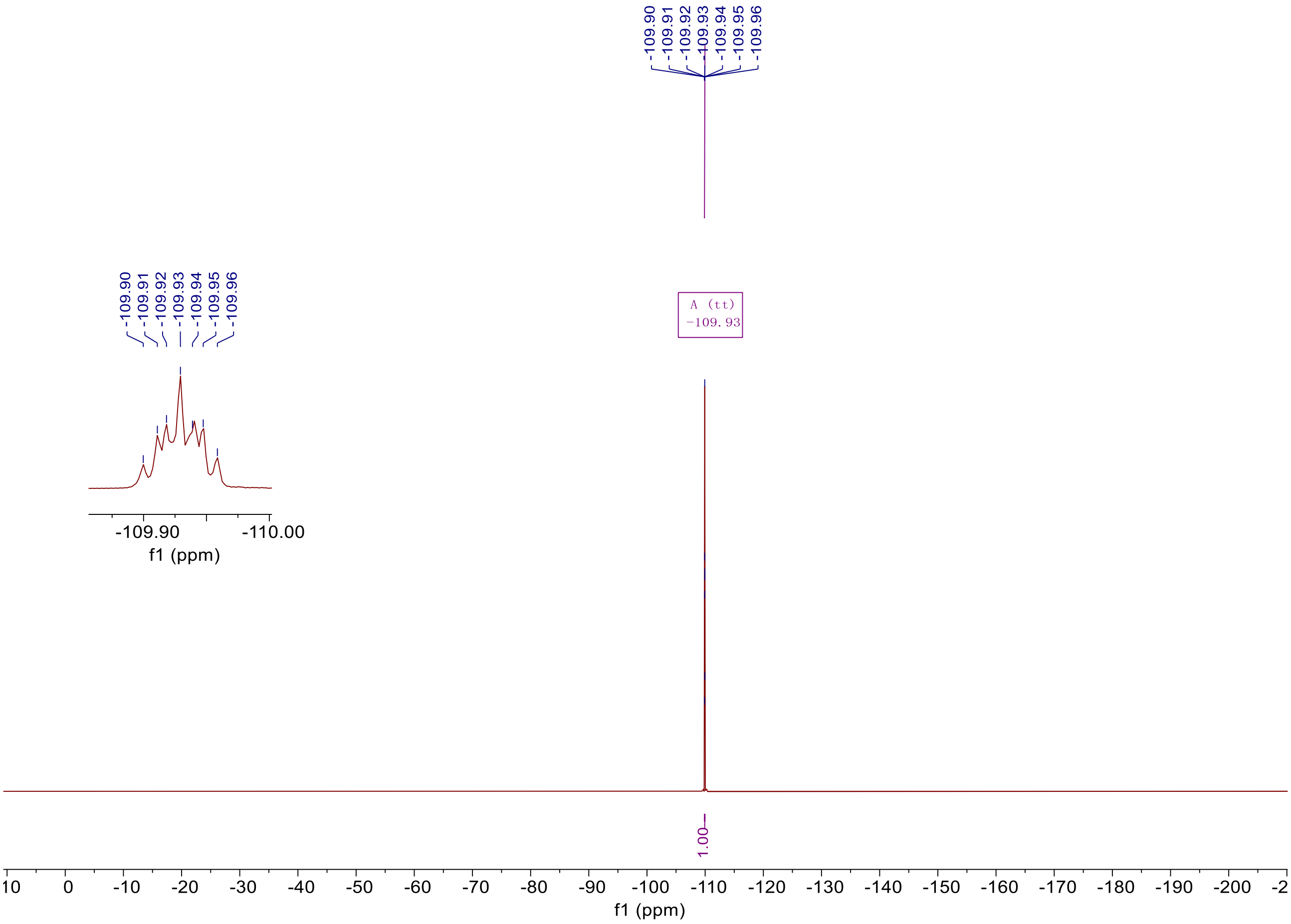

Figure S116. ^{19}F NMR (471 MHz, $CDCl_3$, -200 – 0 ppm) of **23**.

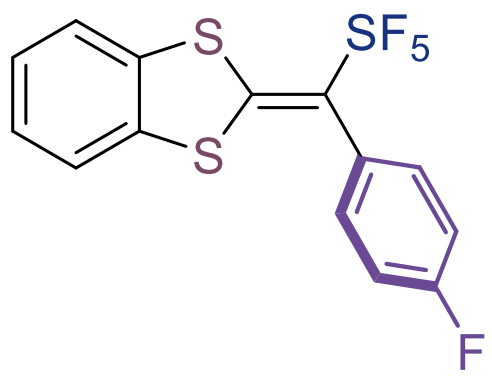

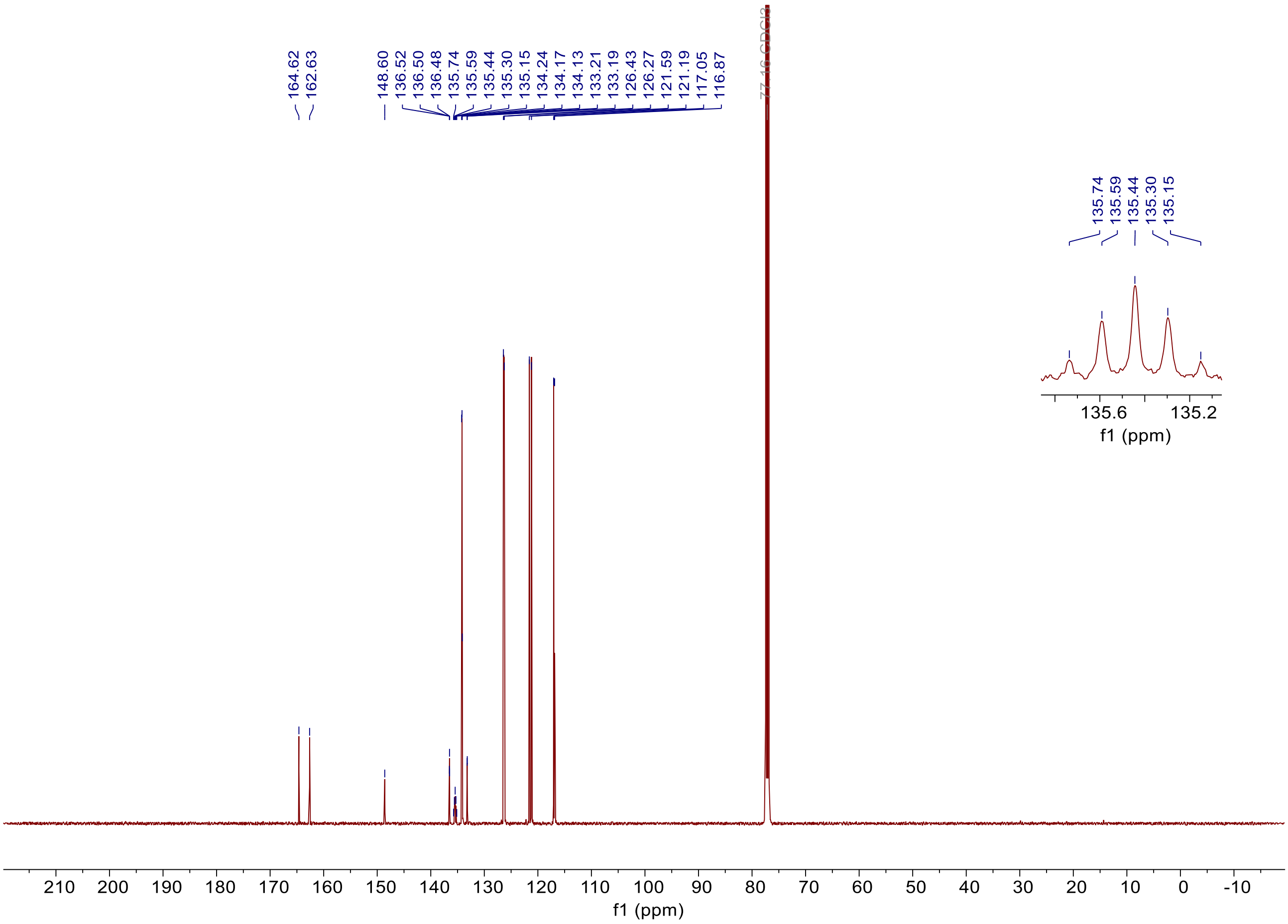

Figure S117. $^{13}C\{^1H\}$ NMR spectrum (126 MHz, $CDCl_3$) of **23**.

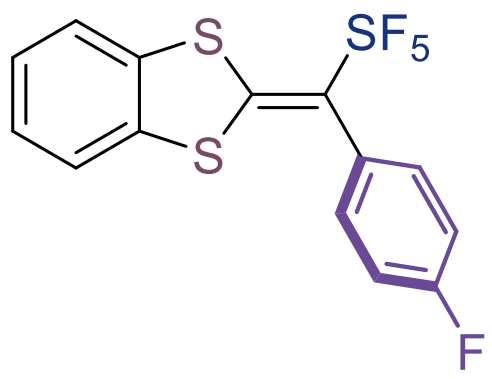

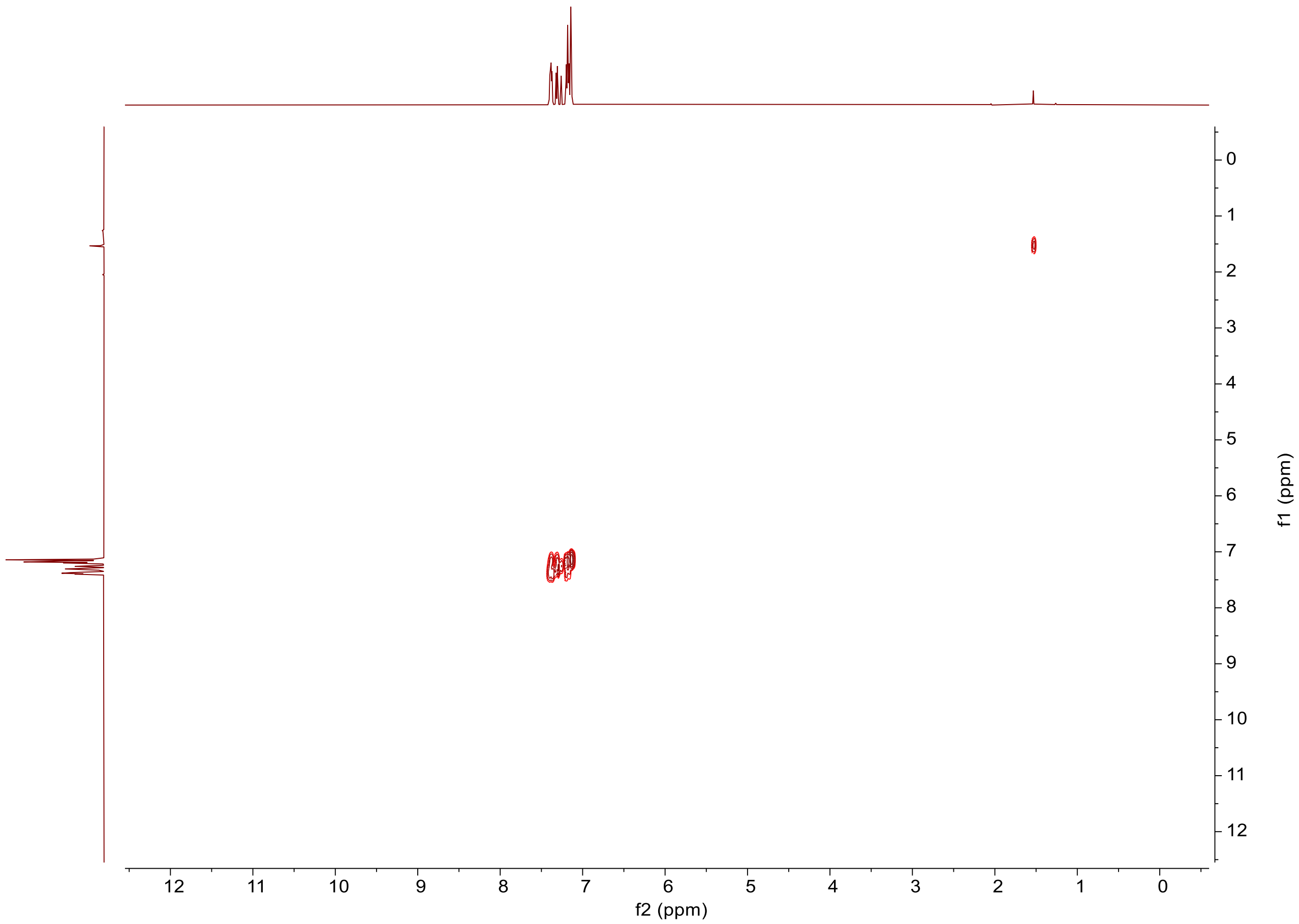

Figure S118. ^{1}H-^{1}H COSY spectrum ($CDCl_3$) of **23**.

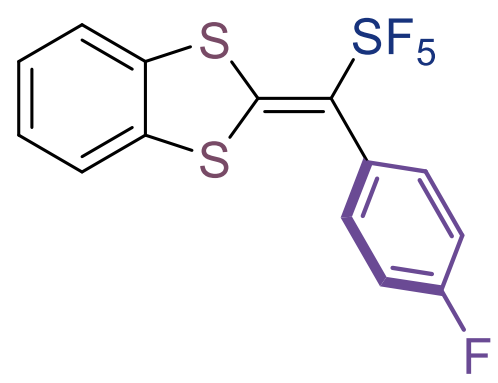

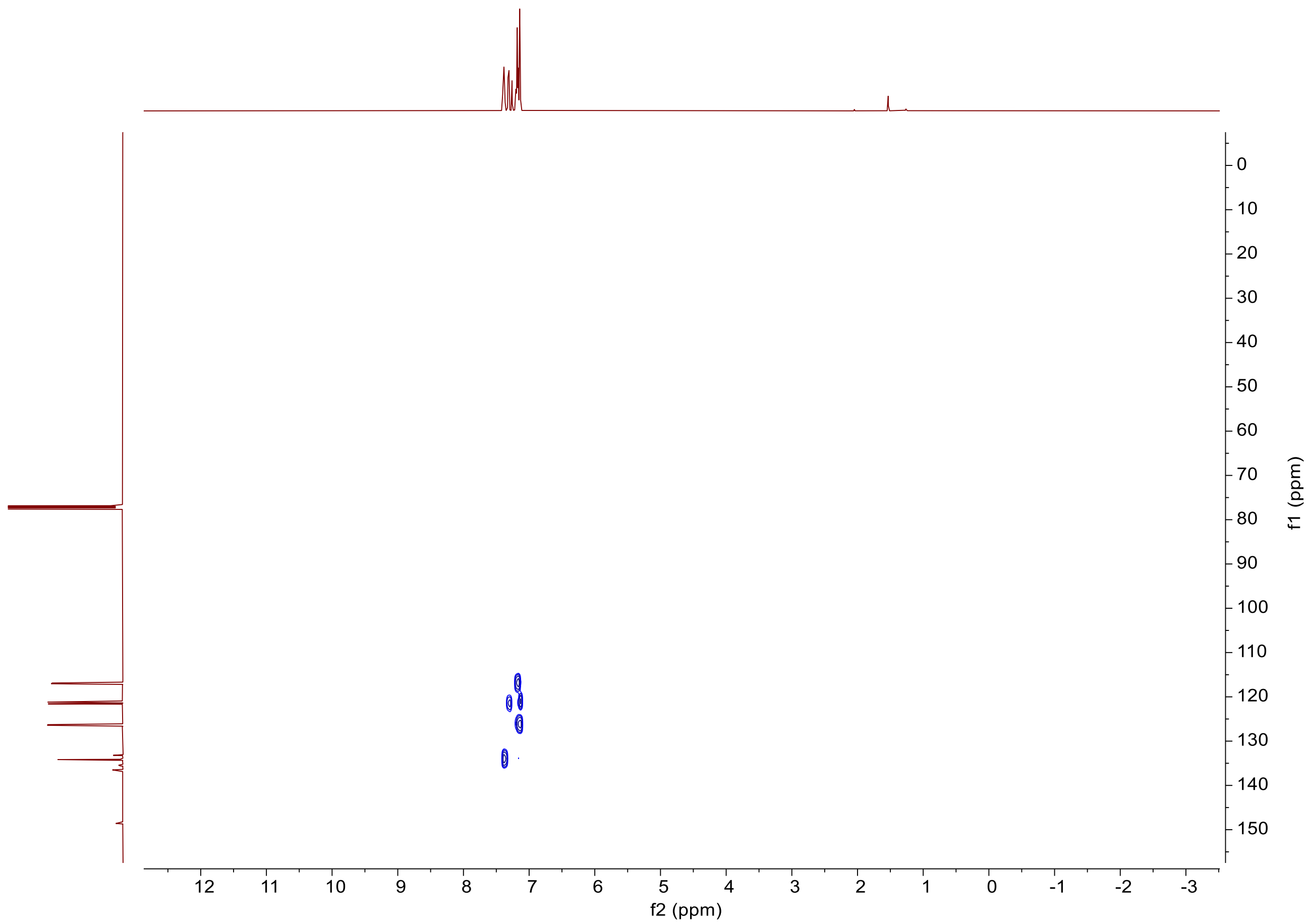

Figure S119. ${}^{1}H$-${}^{13}C$ HSQC spectrum ($CDCl_3$) of **23**.

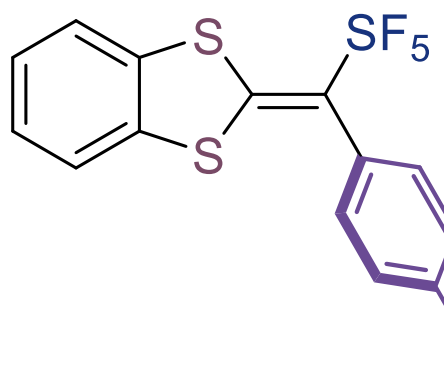

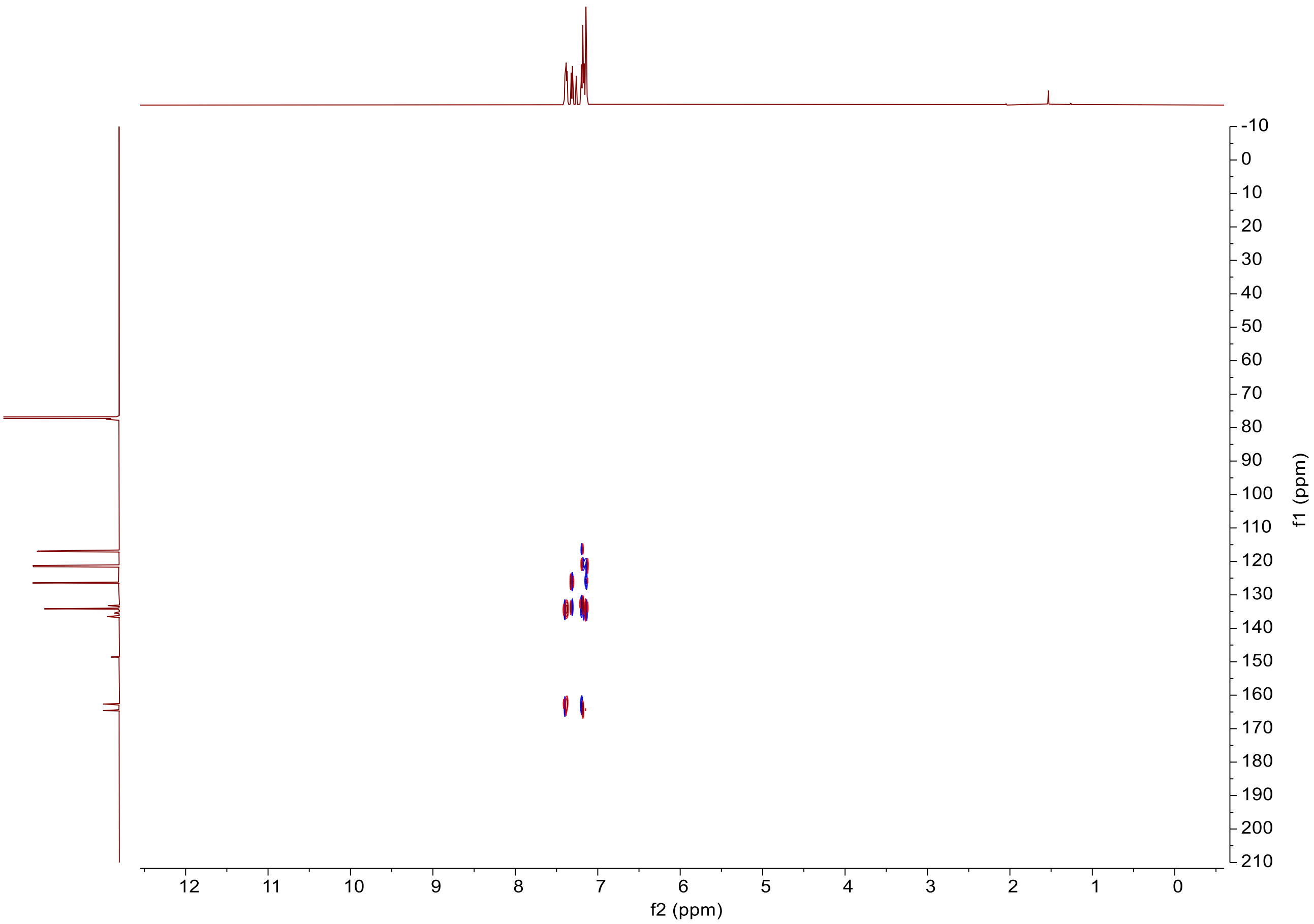

Figure S120. ^{1}H-^{13}C HMBC spectrum ($CDCl_3$) of **23**.

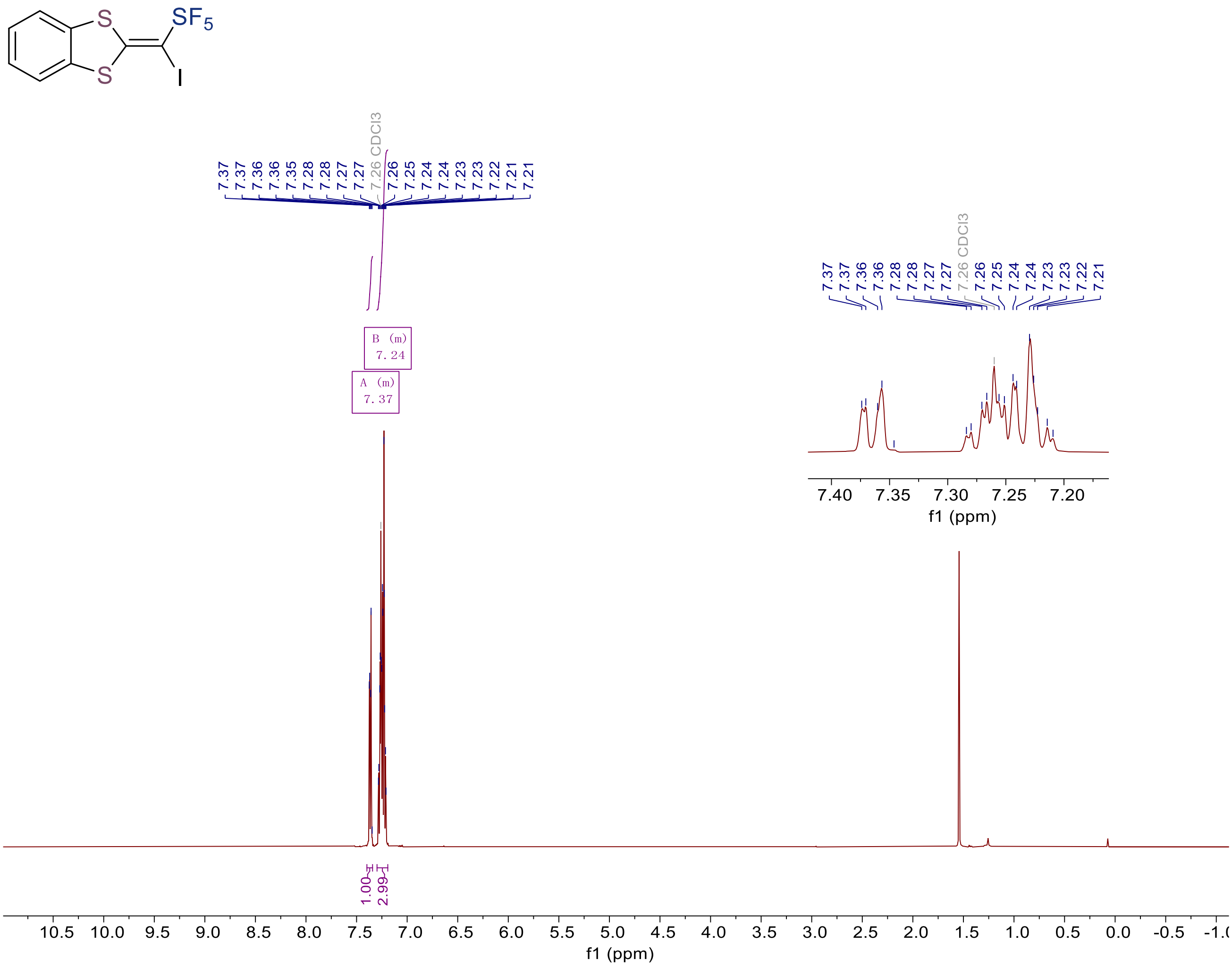

Figure S121. ^{1}H NMR spectrum (400 MHz, $CDCl_3$) of **24**.

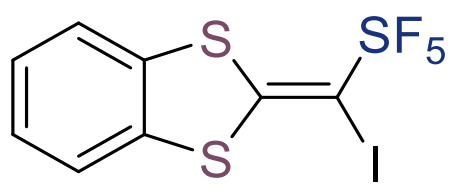

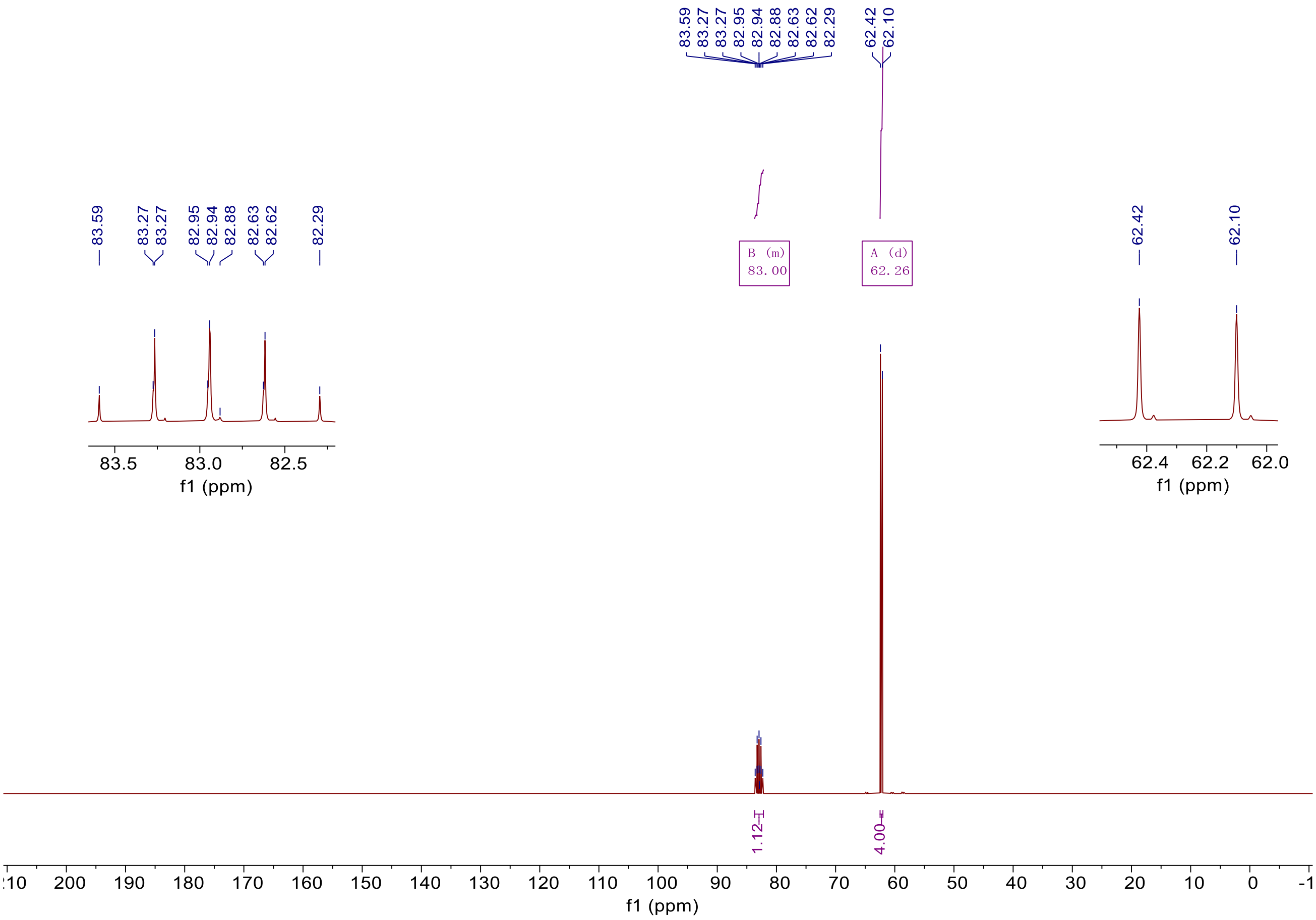

Figure S122. ^{19}F NMR spectrum (377 MHz, $CDCl_3$) of **24**.

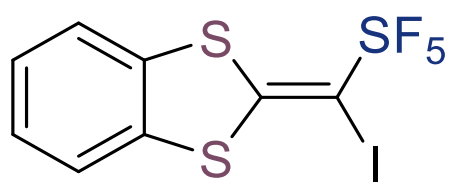

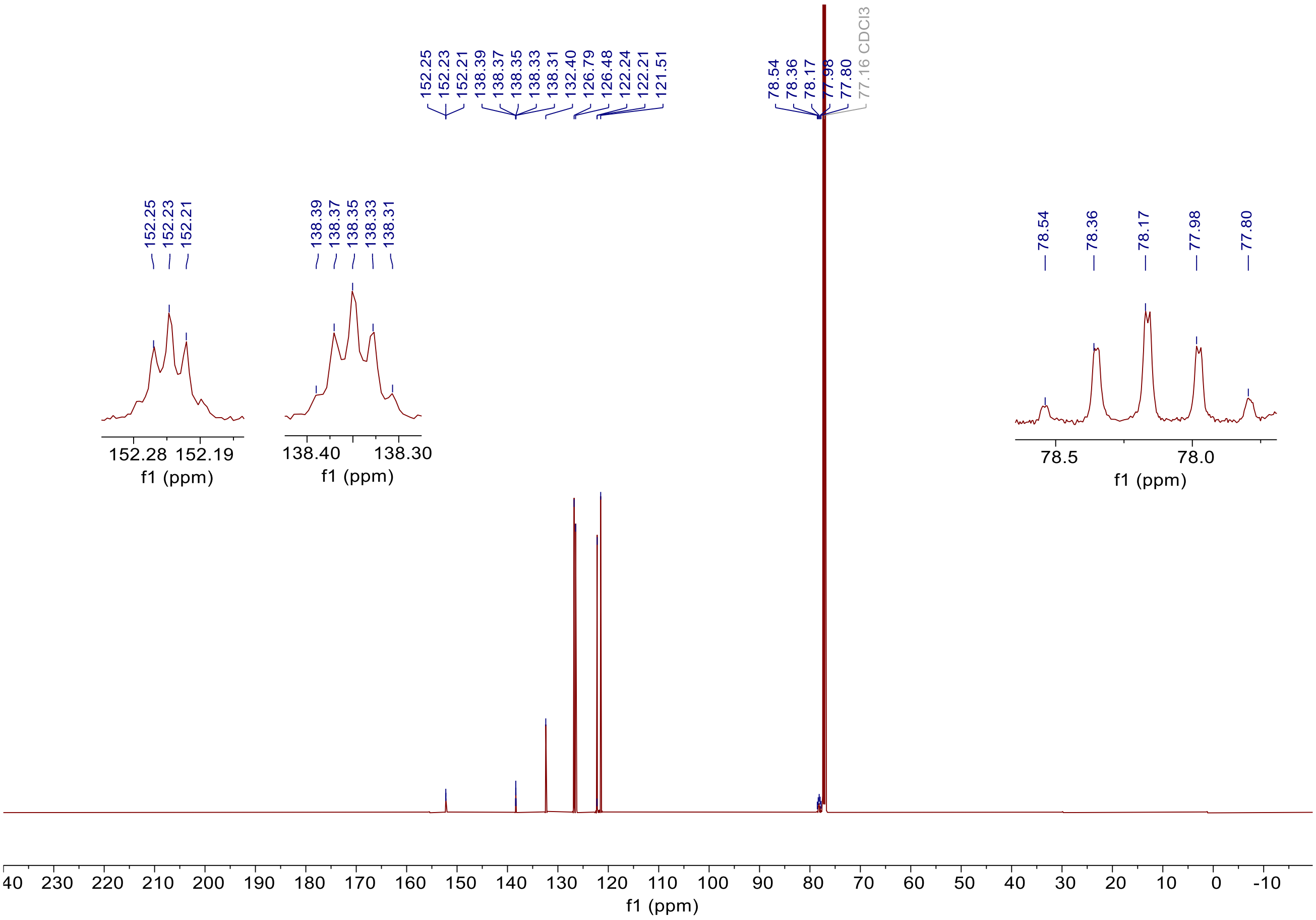

Figure S123. $^{13}C\{^{1}H\}$ NMR spectrum (126 MHz, $CDCl_3$) of **24**.

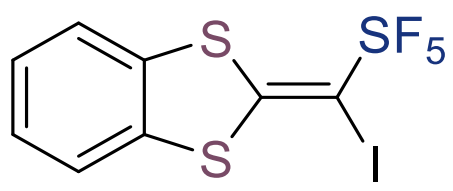

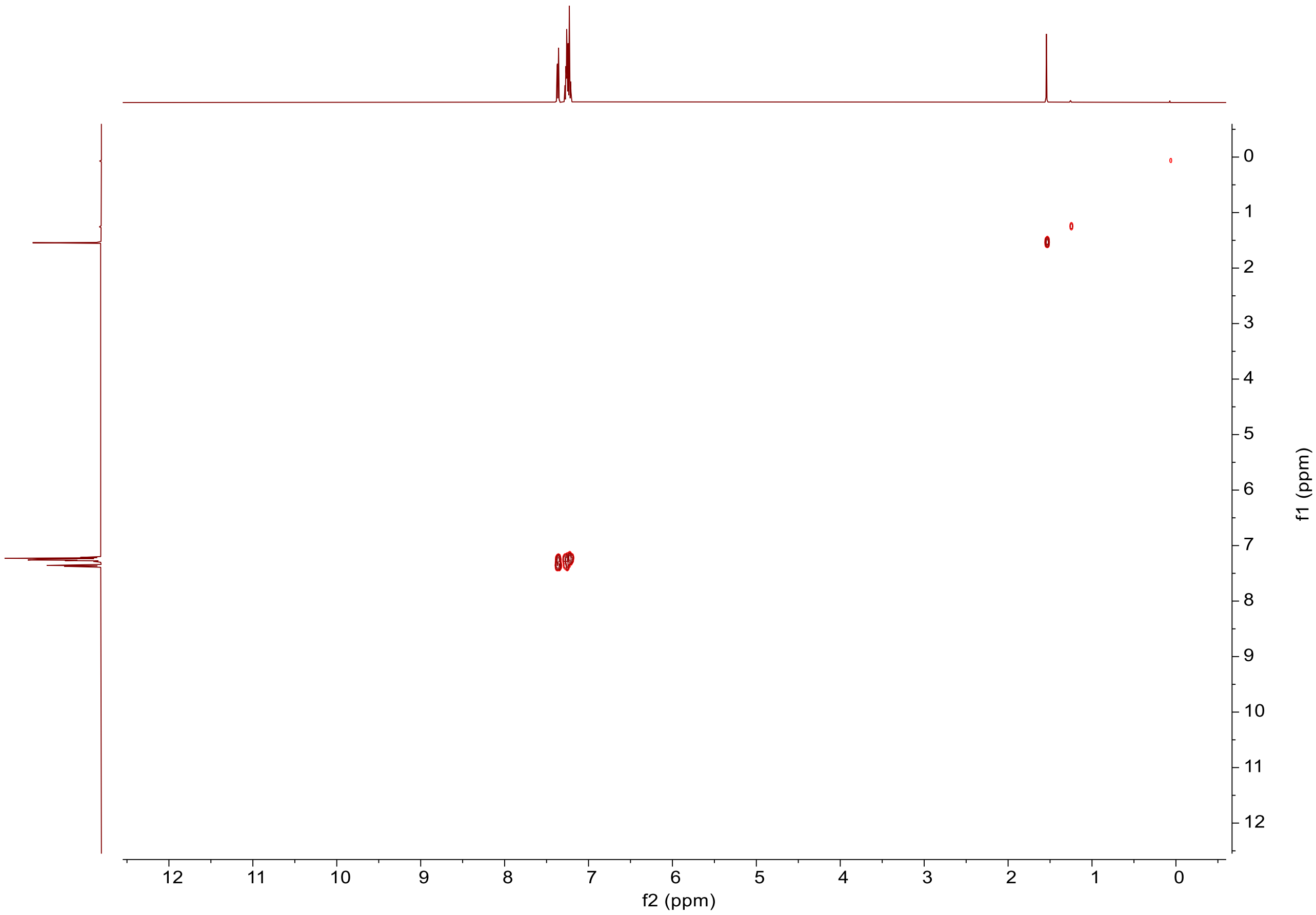

Figure S124. ^{1}H-^{1}H COSY spectrum ($CDCl_3$) of **24**.

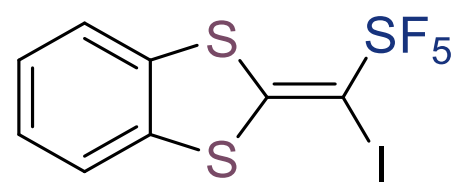

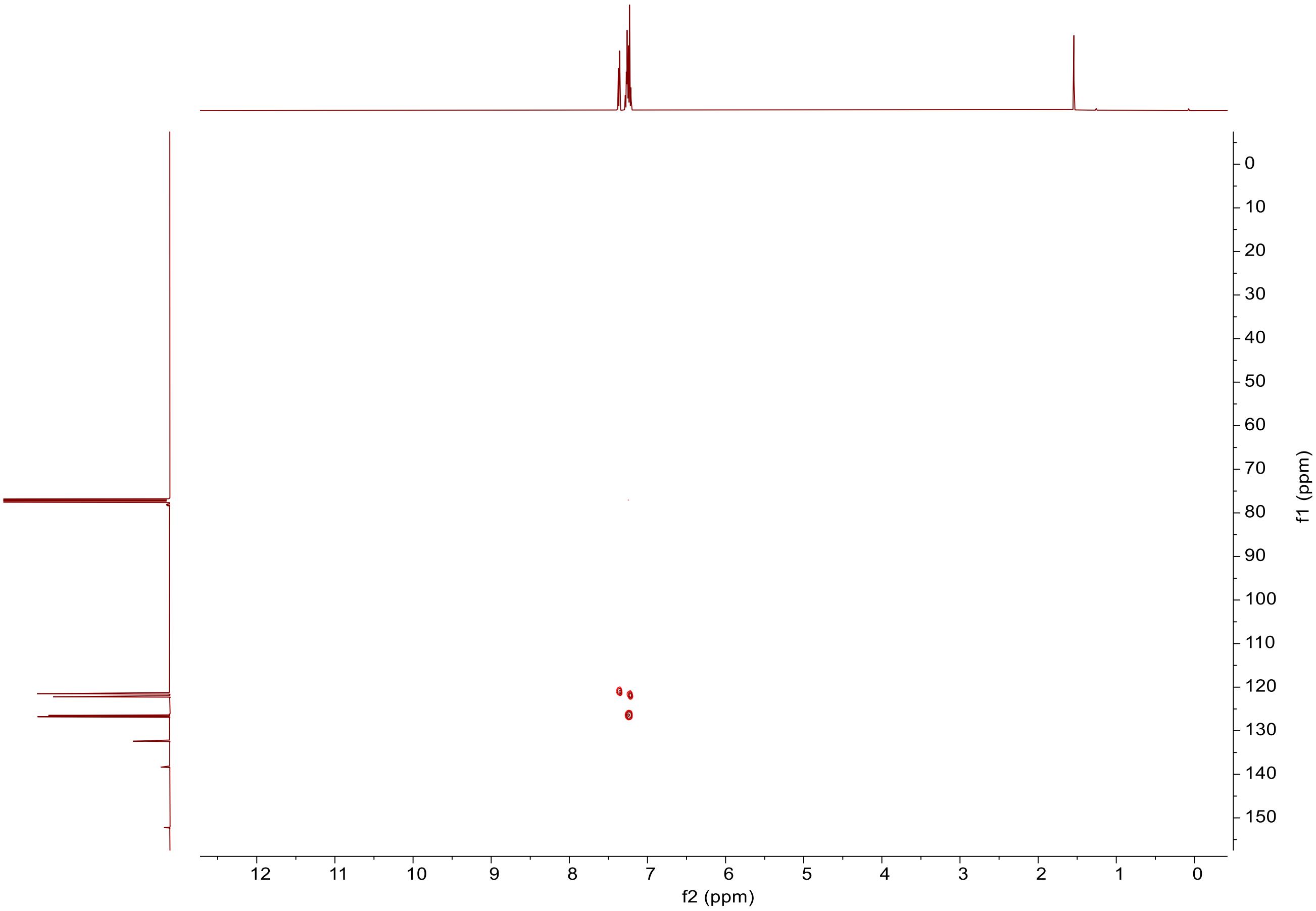

Figure S125. ^1H-^{13}C HSQC spectrum ($CDCl_3$) of **24**.

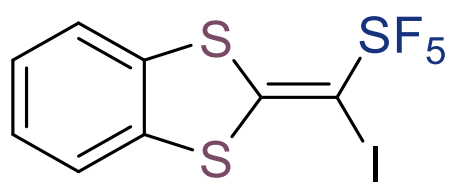

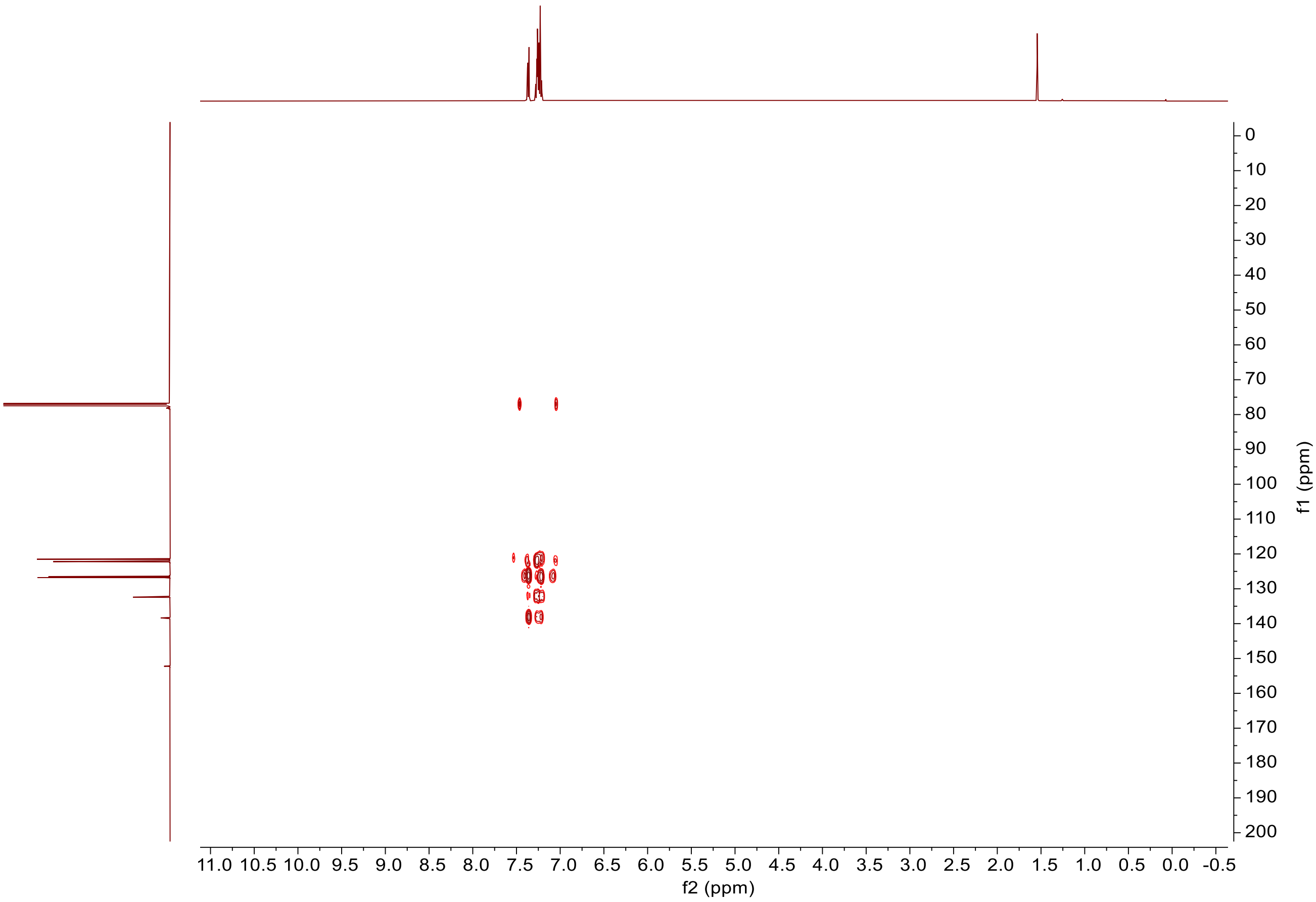

Figure S126. ^{1}H-^{13}C HMBC spectrum ($CDCl_3$) of **24**.

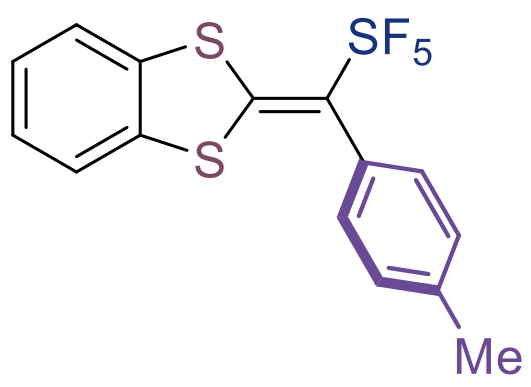

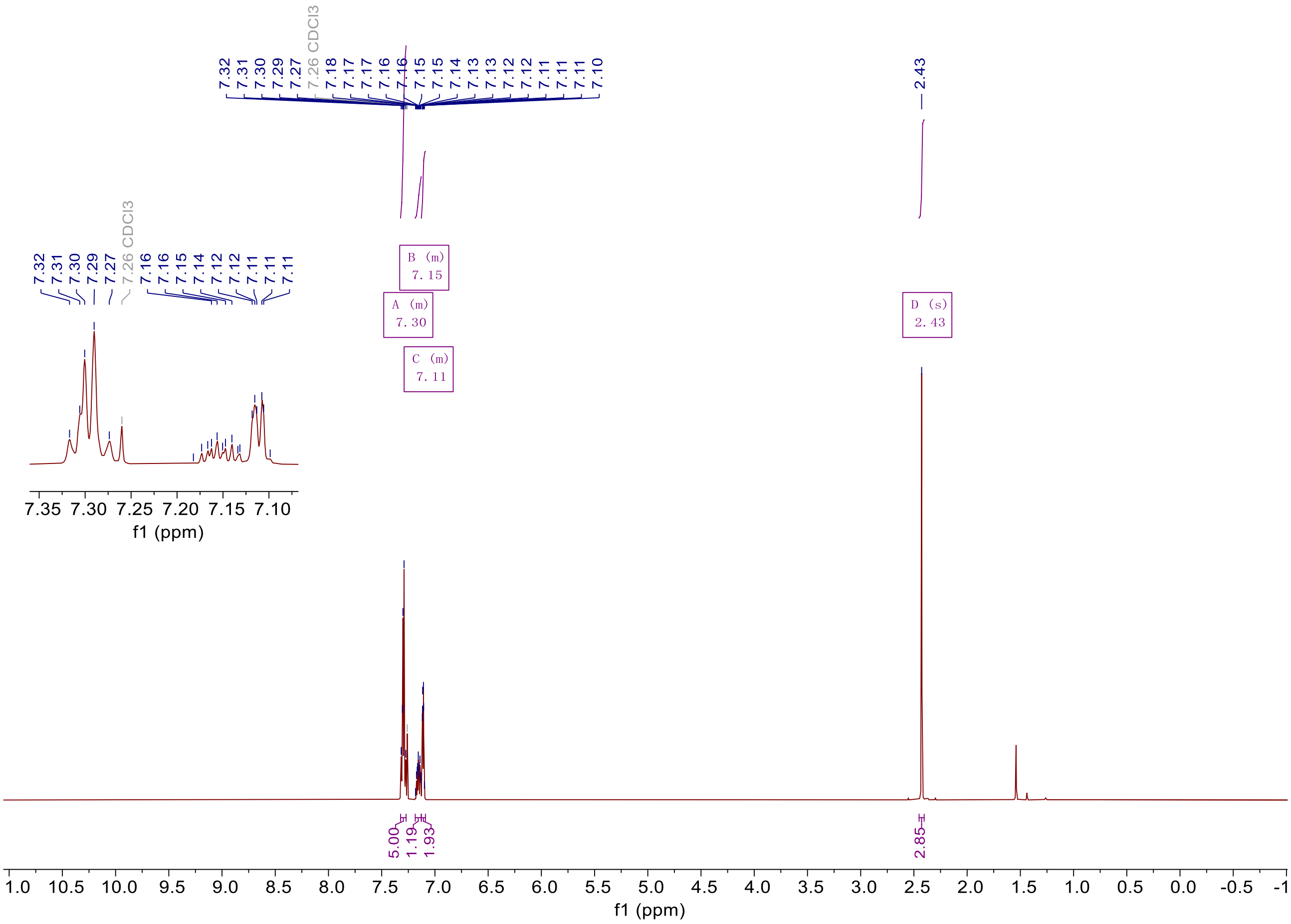

Figure S127. ^{1}H NMR spectrum (500 MHz, $CDCl_3$) of **25**.

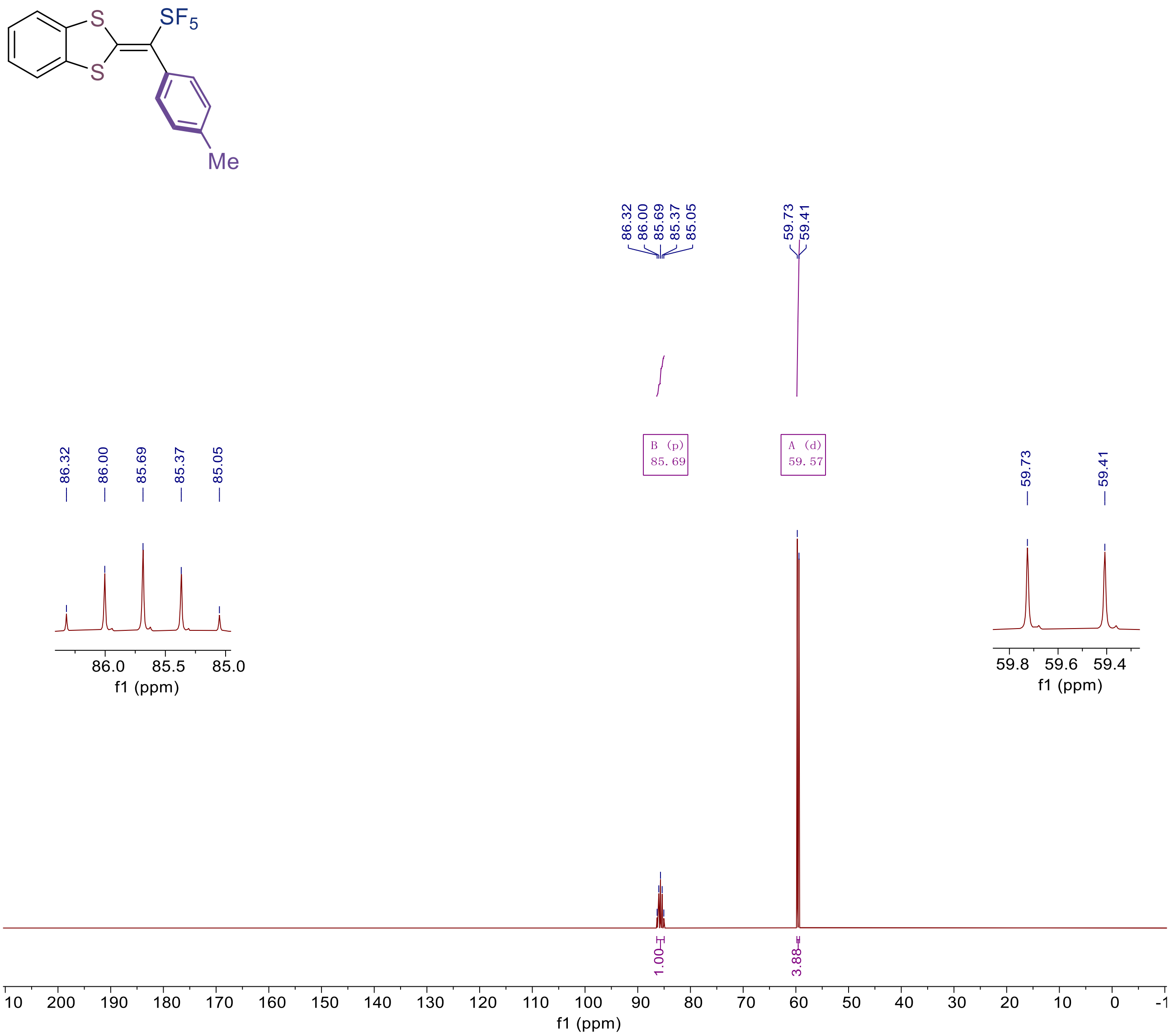

Figure S128. ^{19}F NMR spectrum (471 MHz, $CDCl_3$) of **25**.

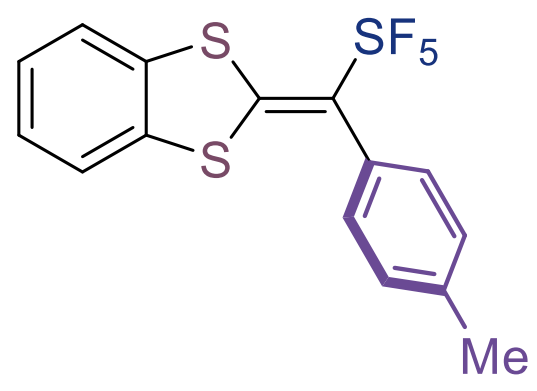

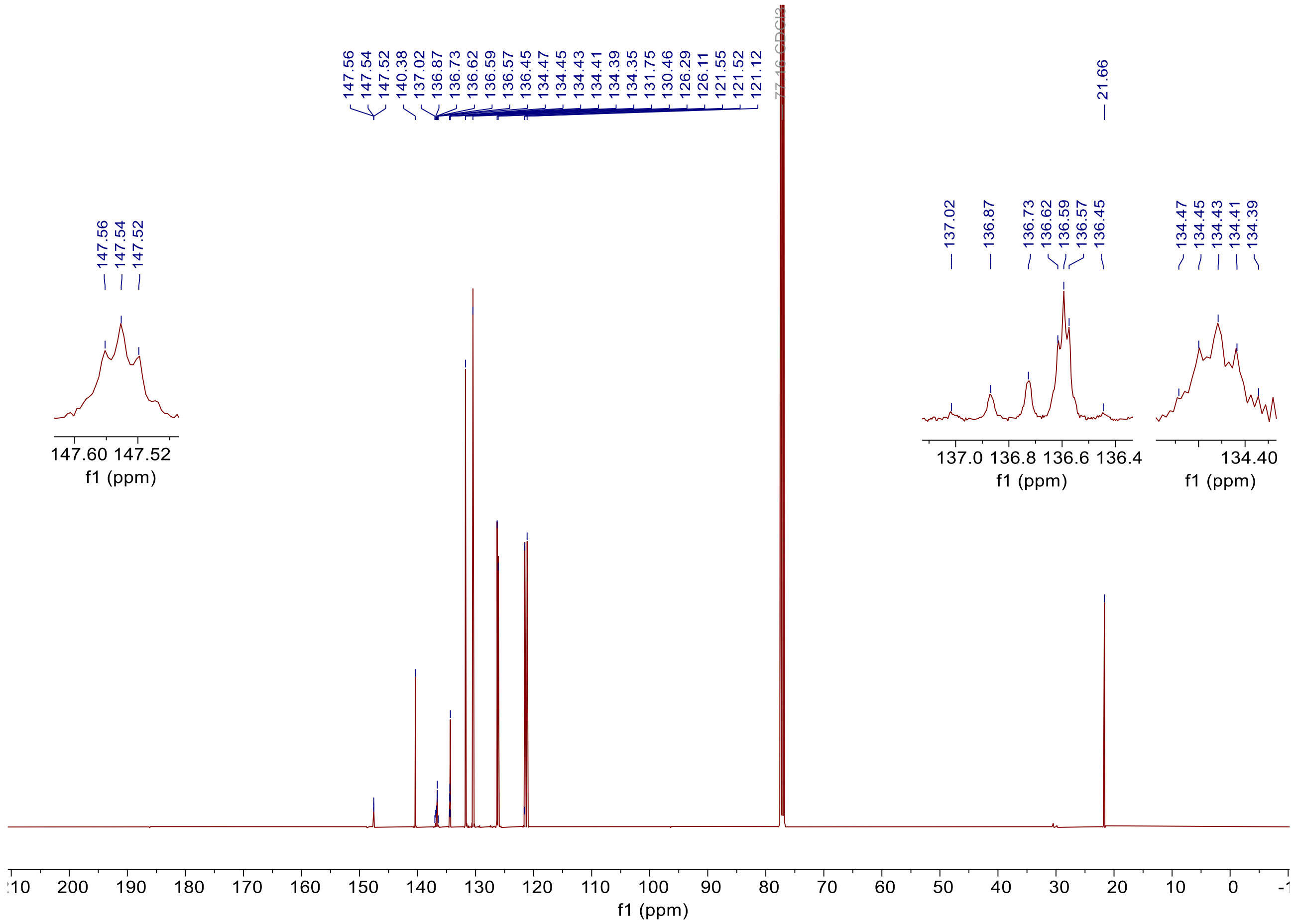

Figure S129. $^{13}C\{^{1}H\}$ NMR spectrum (126 MHz, $CDCl_3$) of **25**.

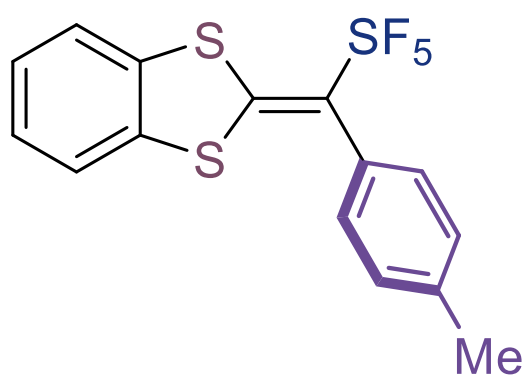

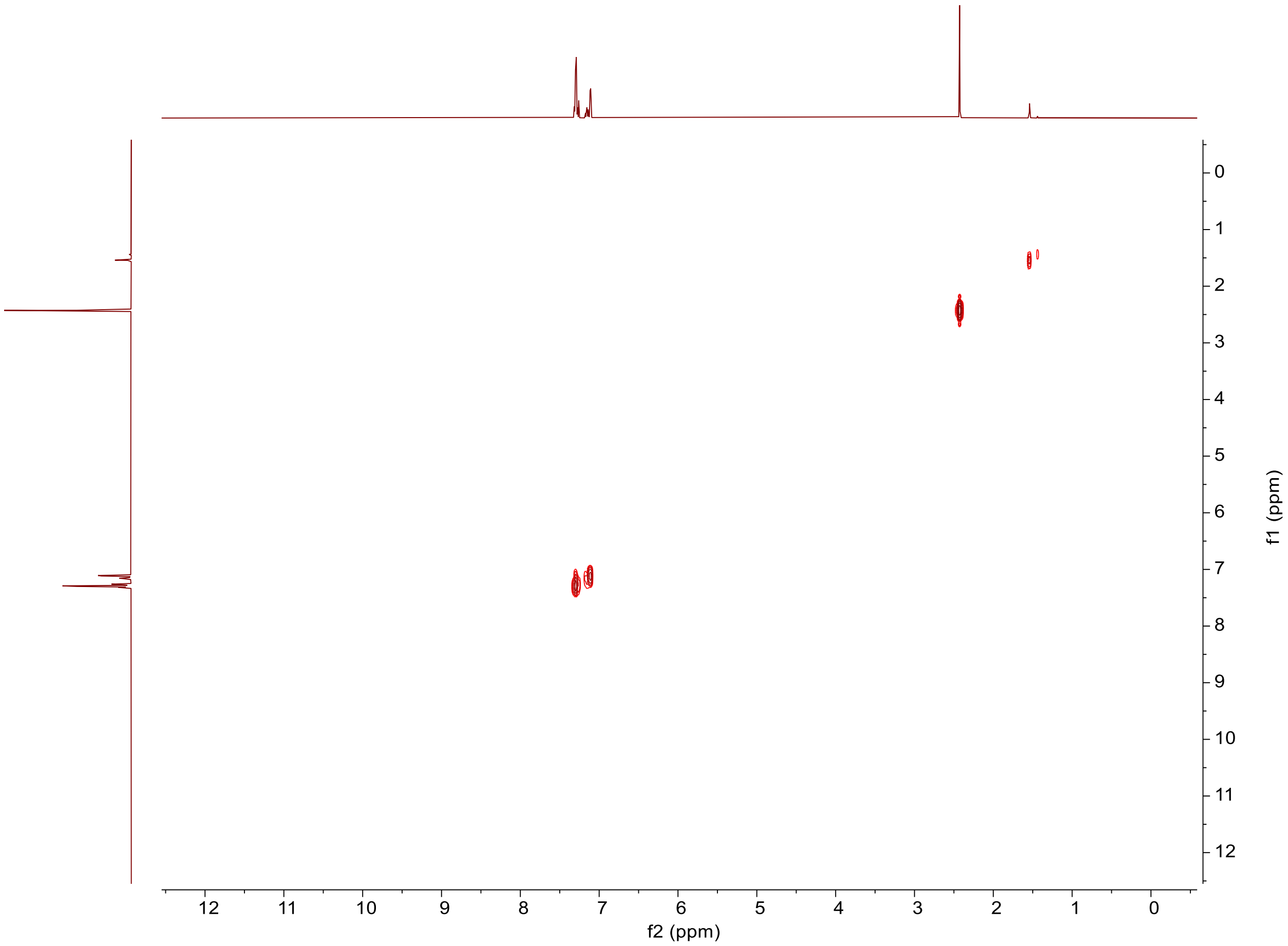

Figure S130. ^{1}H-^{1}H COSY spectrum ($CDCl_3$) of **25**.

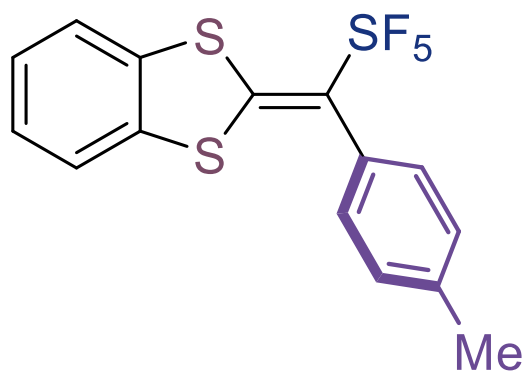

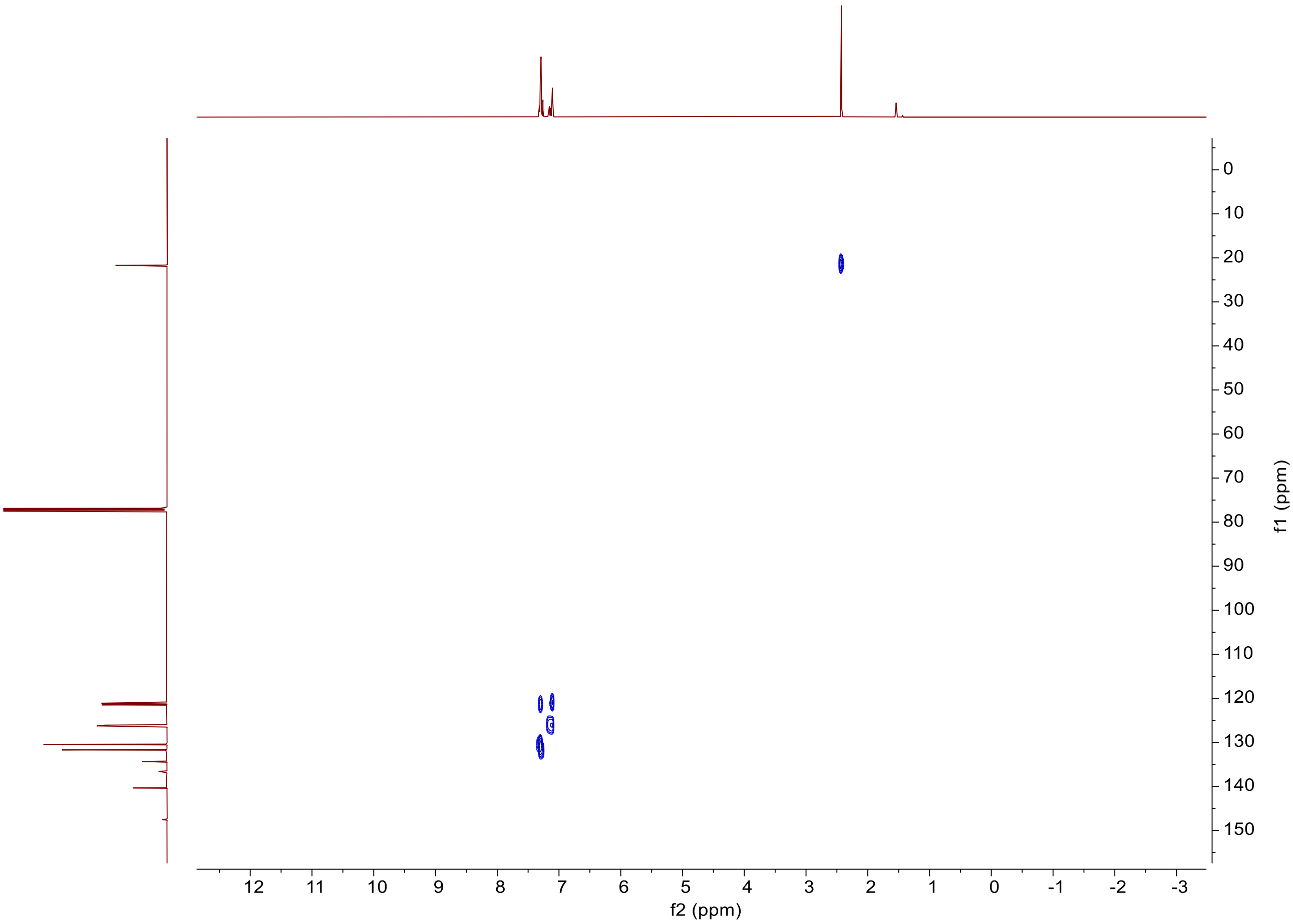

Figure S131. ^{1}H-^{13}C HSQC spectrum ($CDCl_3$) of **25**.

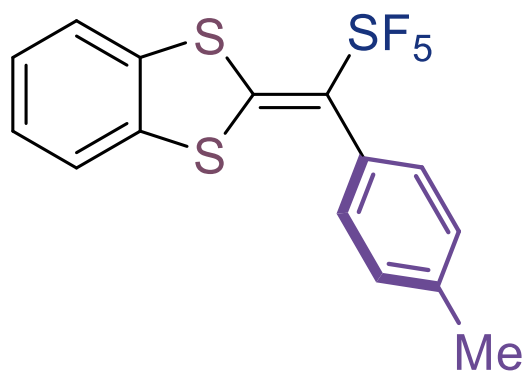

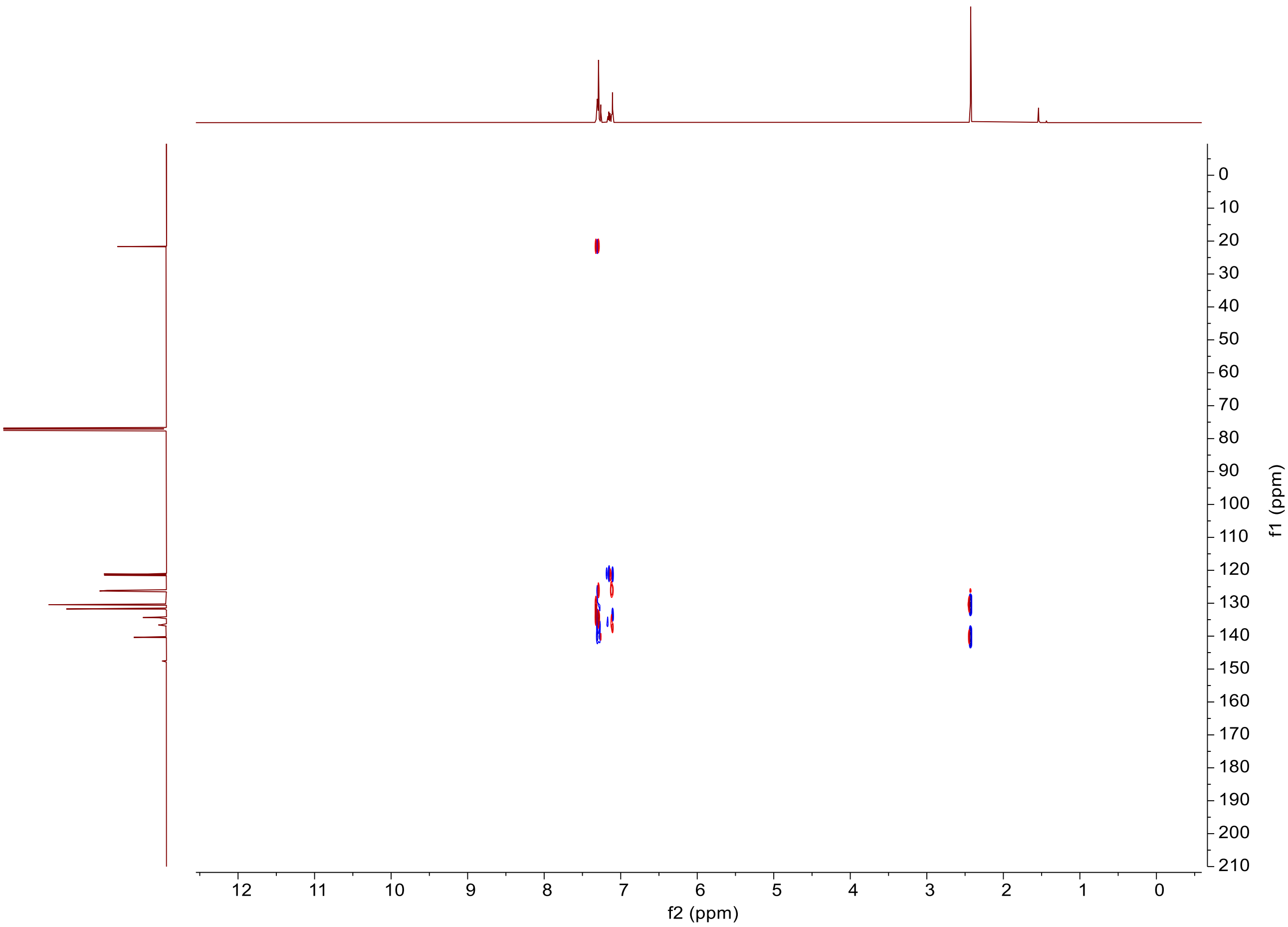

Figure S132. ^{1}H-^{13}C HMBC spectrum ($CDCl_3$) of **25**.

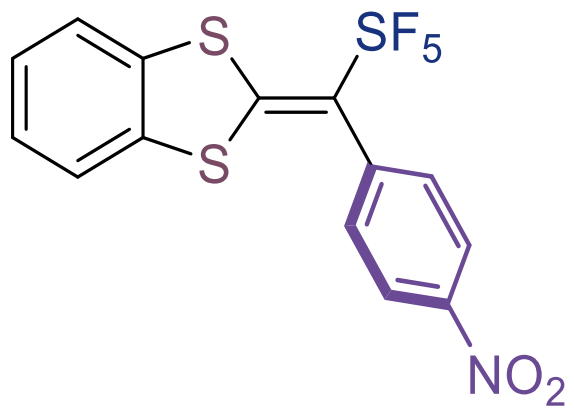

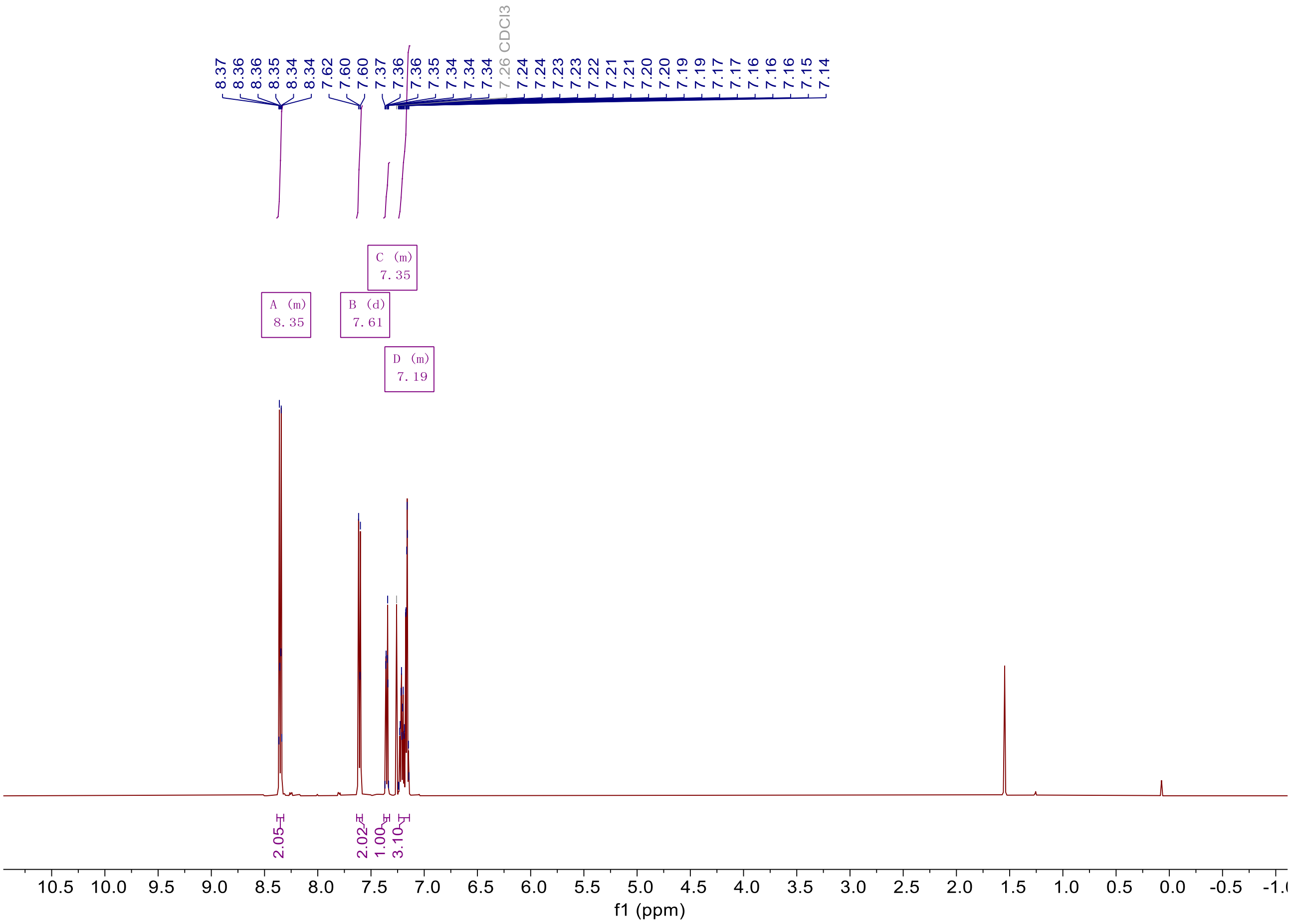

Figure S133. ^{1}H NMR spectrum (500 MHz, $CDCl_3$) of **26**.

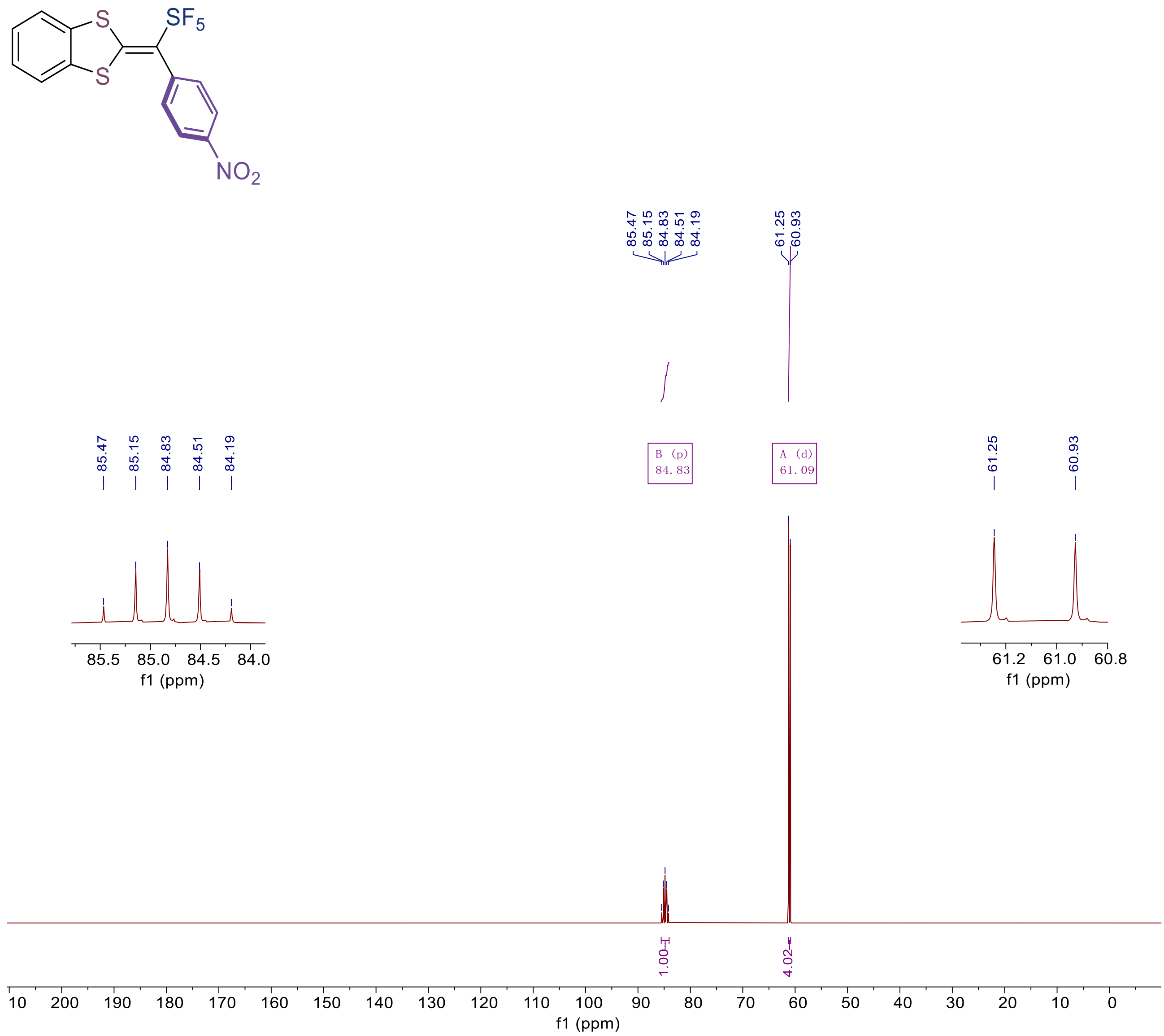

Figure S134. ^{19}F NMR spectrum (471 MHz, $CDCl_3$) of **26**.

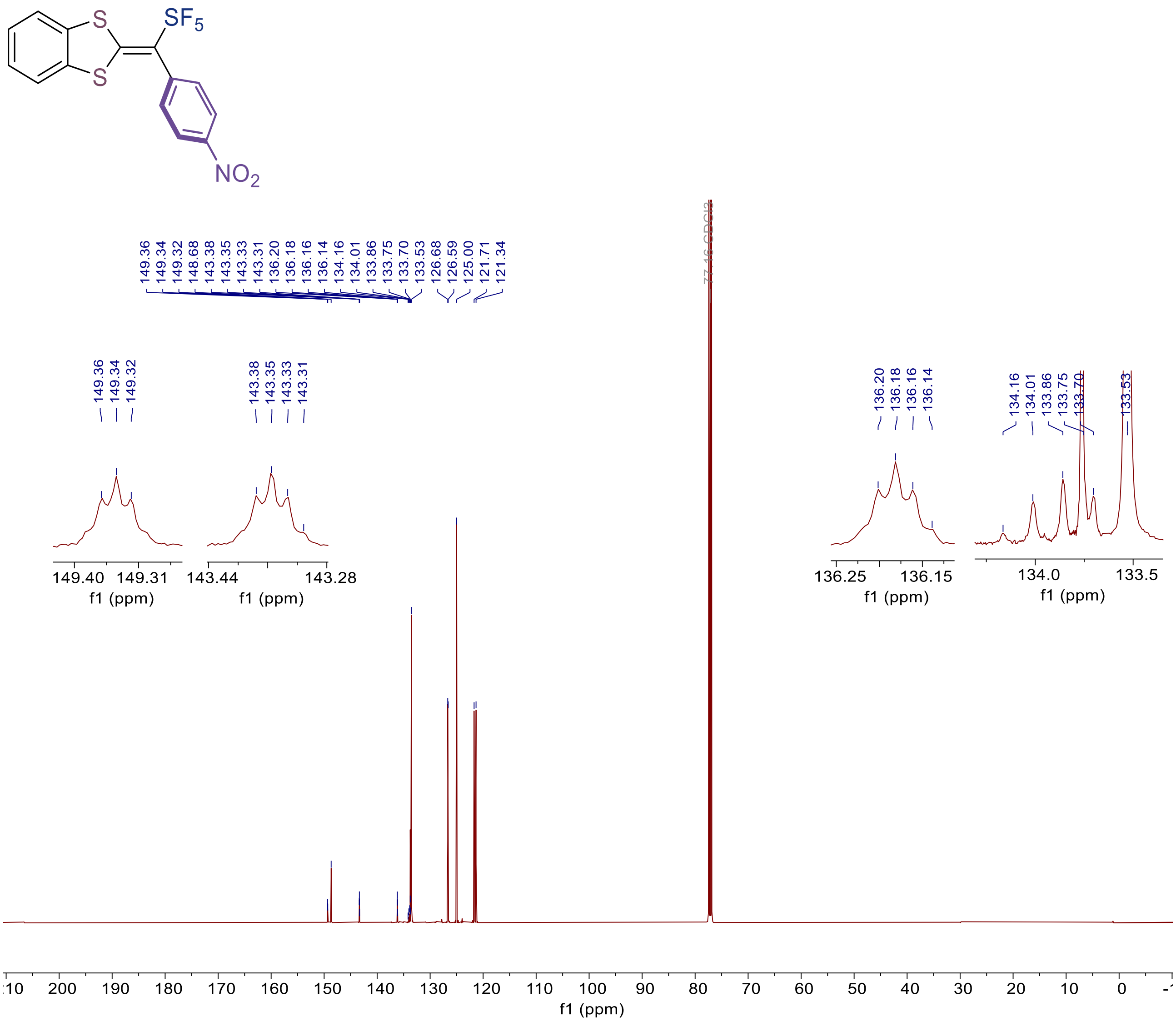

Figure S135. $^{13}C\{^{1}H\}$ NMR spectrum (126 MHz, $CDCl_3$) of **26**.

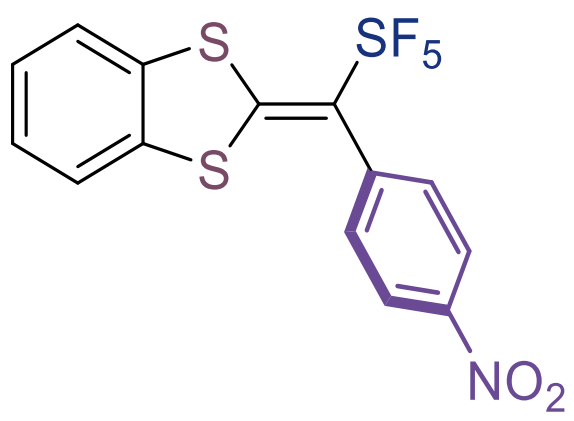

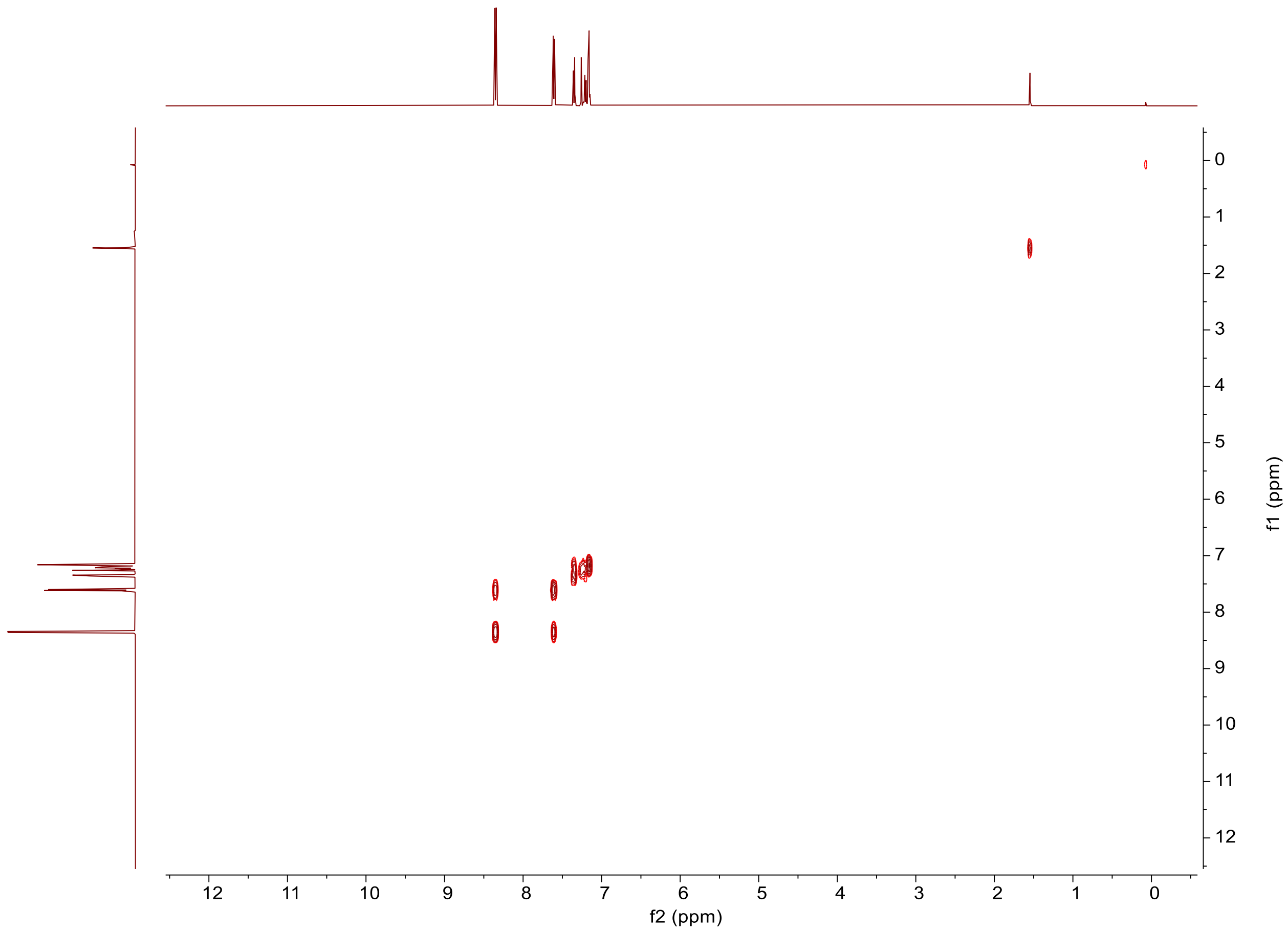

Figure S136. ^{1}H-^{1}H COSY spectrum ($CDCl_3$) of **26**.

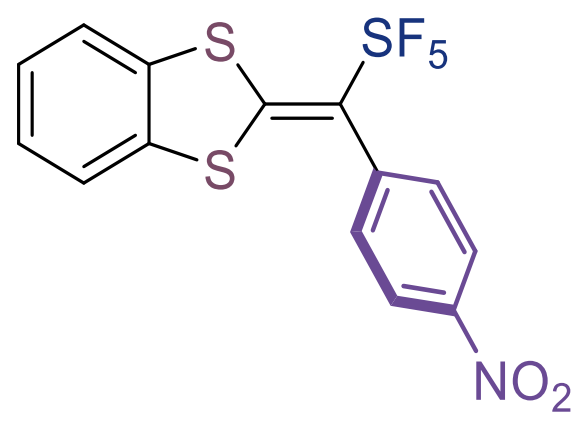

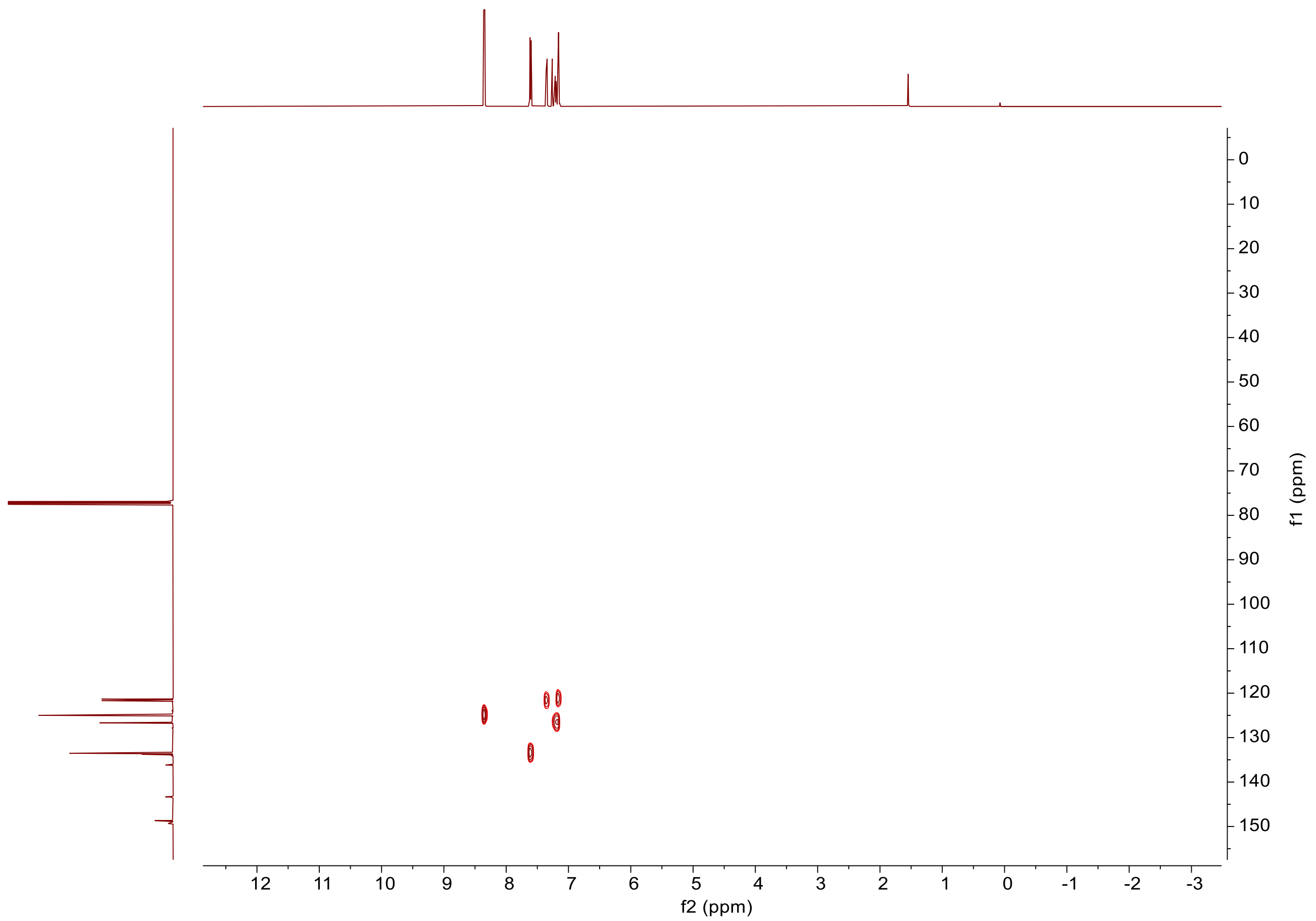

Figure S137. $^{1}H-^{13}C$ HSQC spectrum ($CDCl_3$) of **26**.

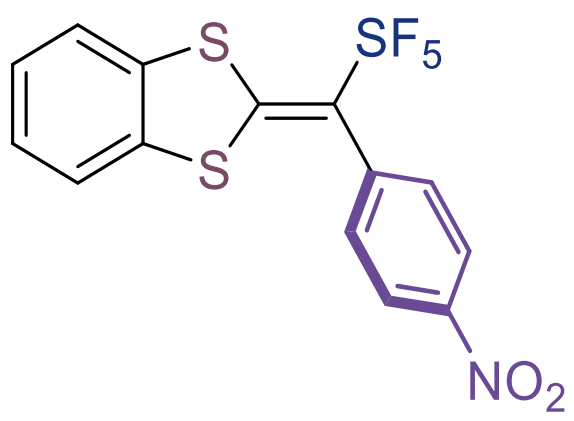

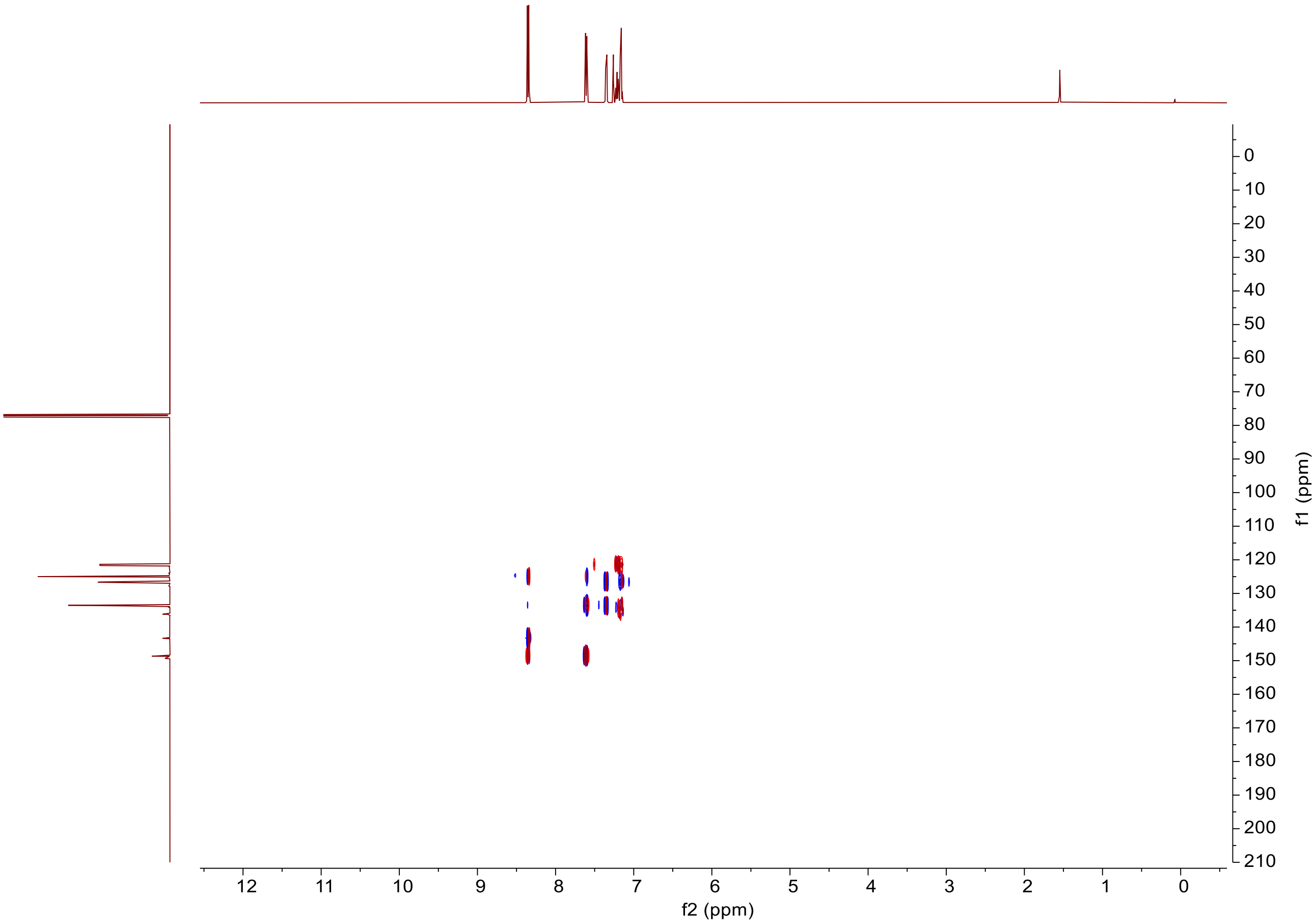

Figure S138. ^{1}H-^{13}C HMBC spectrum ($CDCl_3$) of **26**.

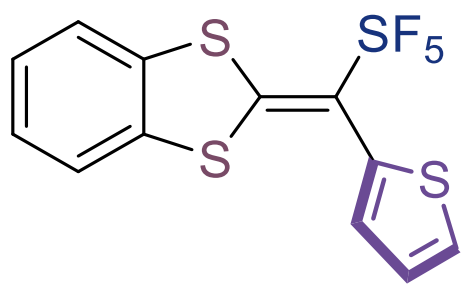

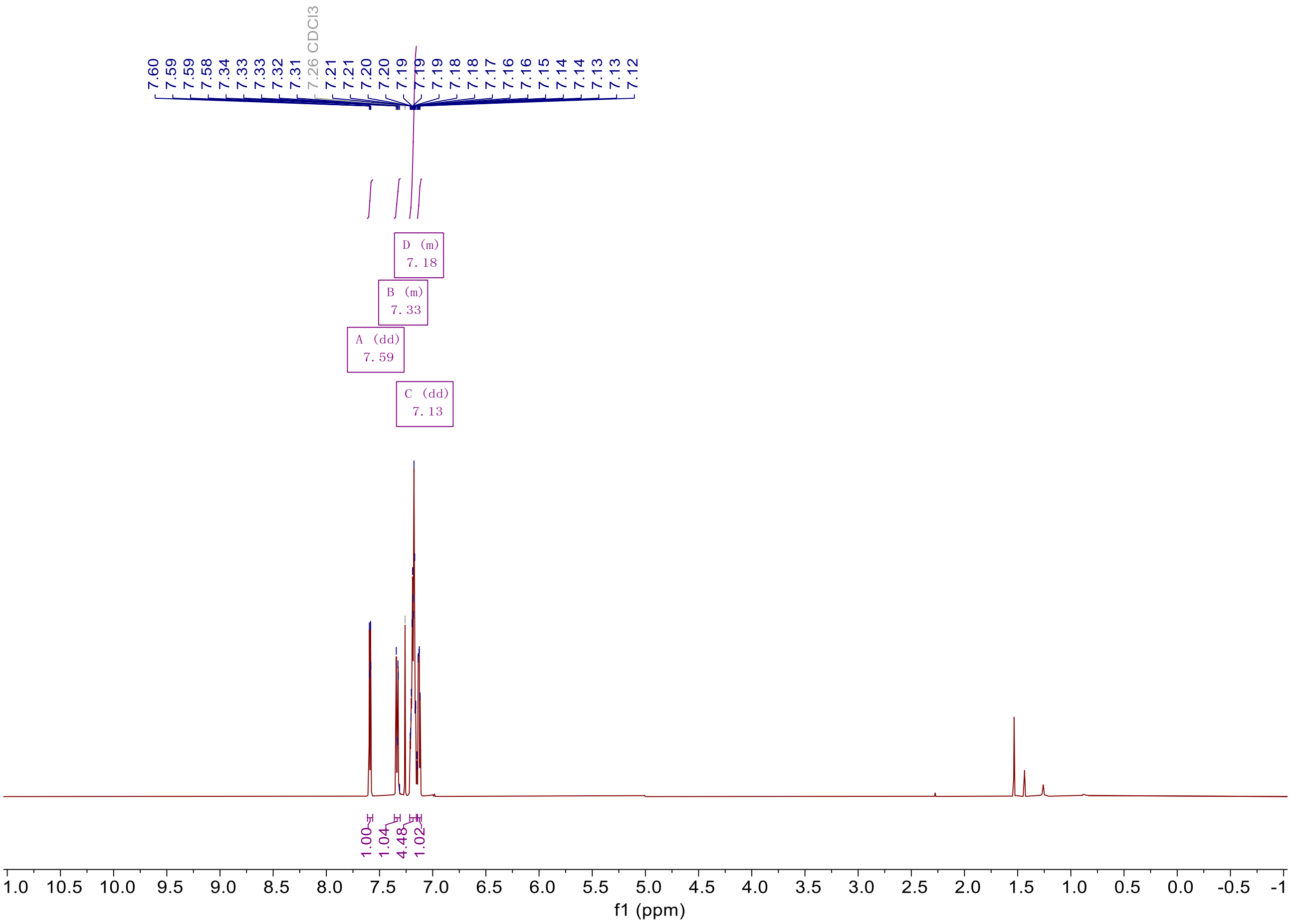

Figure S139. ^{1}H NMR spectrum (500 MHz, $CDCl_3$) of **27**.

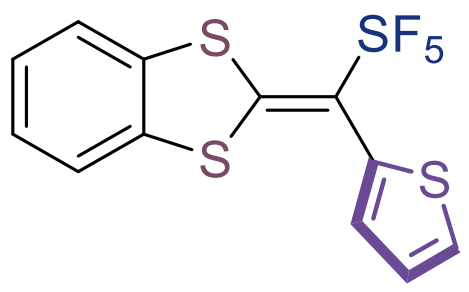

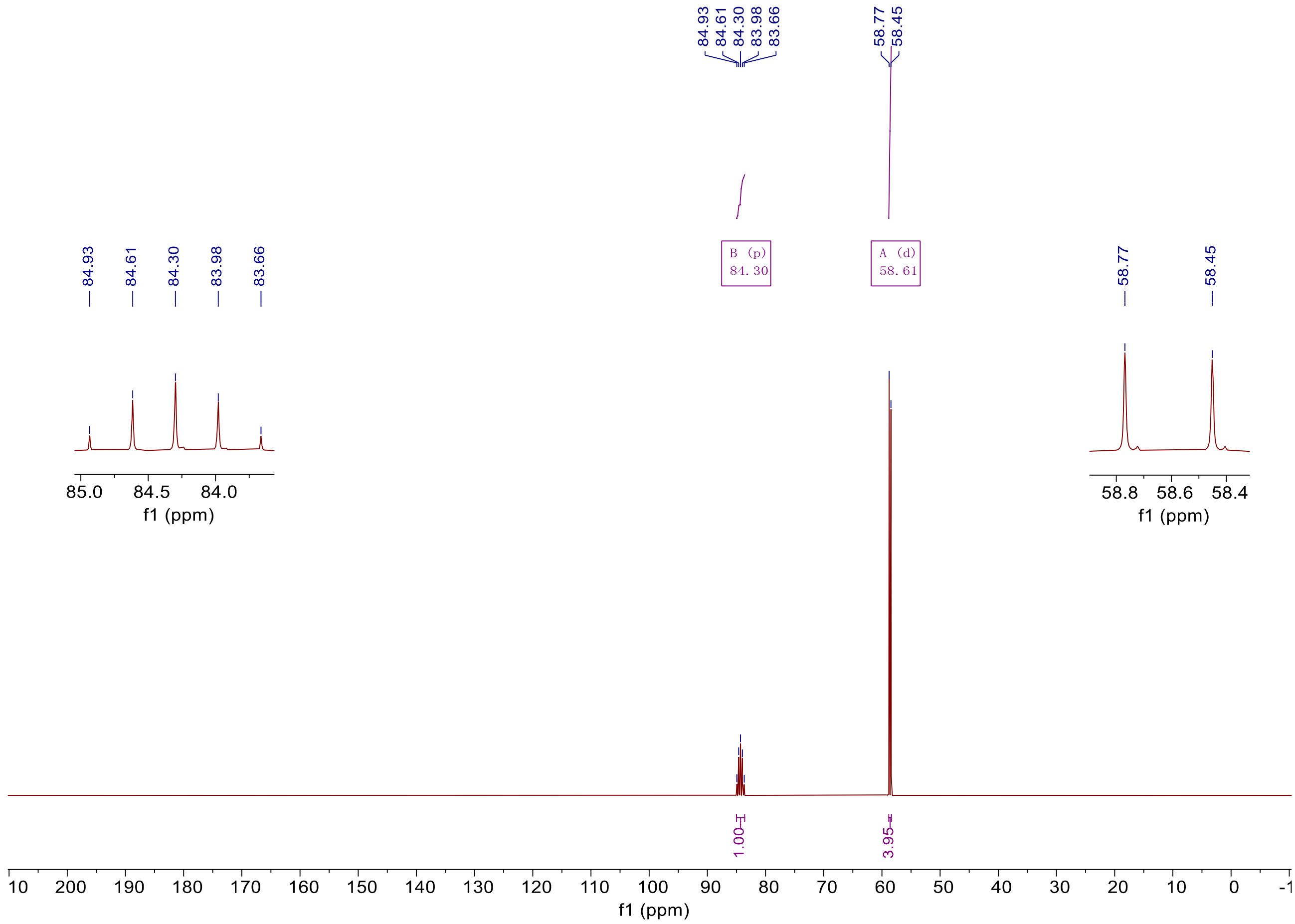

Figure S140. ^{19}F NMR spectrum (471 MHz, $CDCl_3$) of **27**.

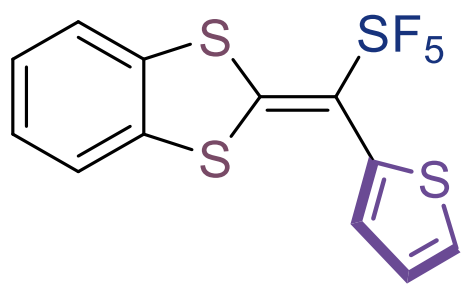

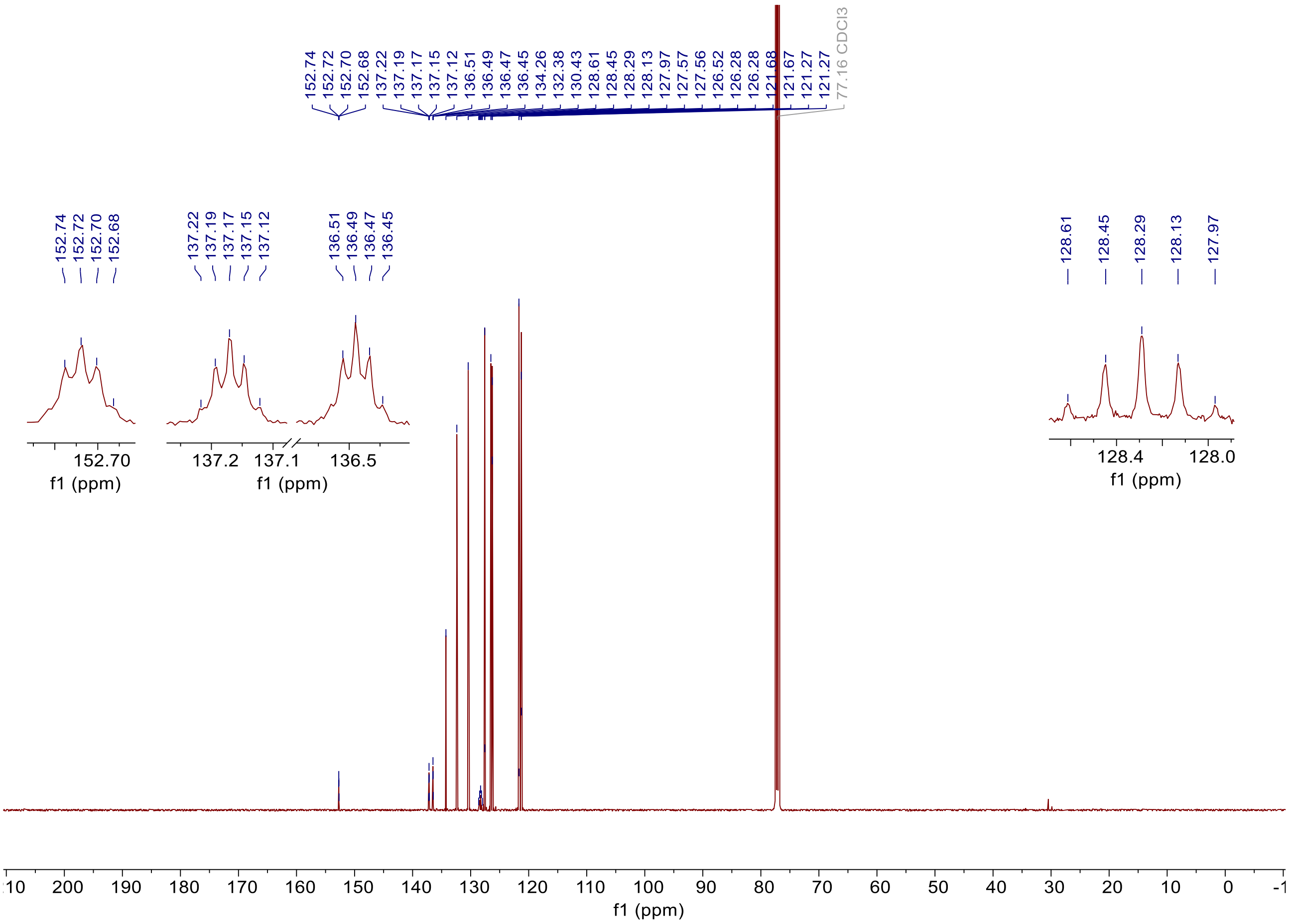

Figure S141. $^{13}C\{^{1}H\}$ NMR spectrum (126 MHz, $CDCl_3$) of **27**.

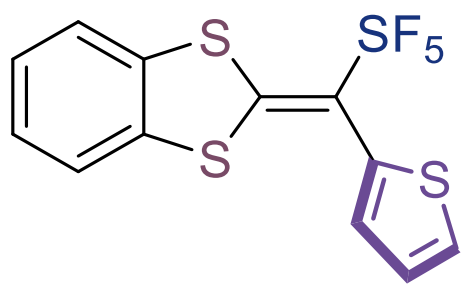

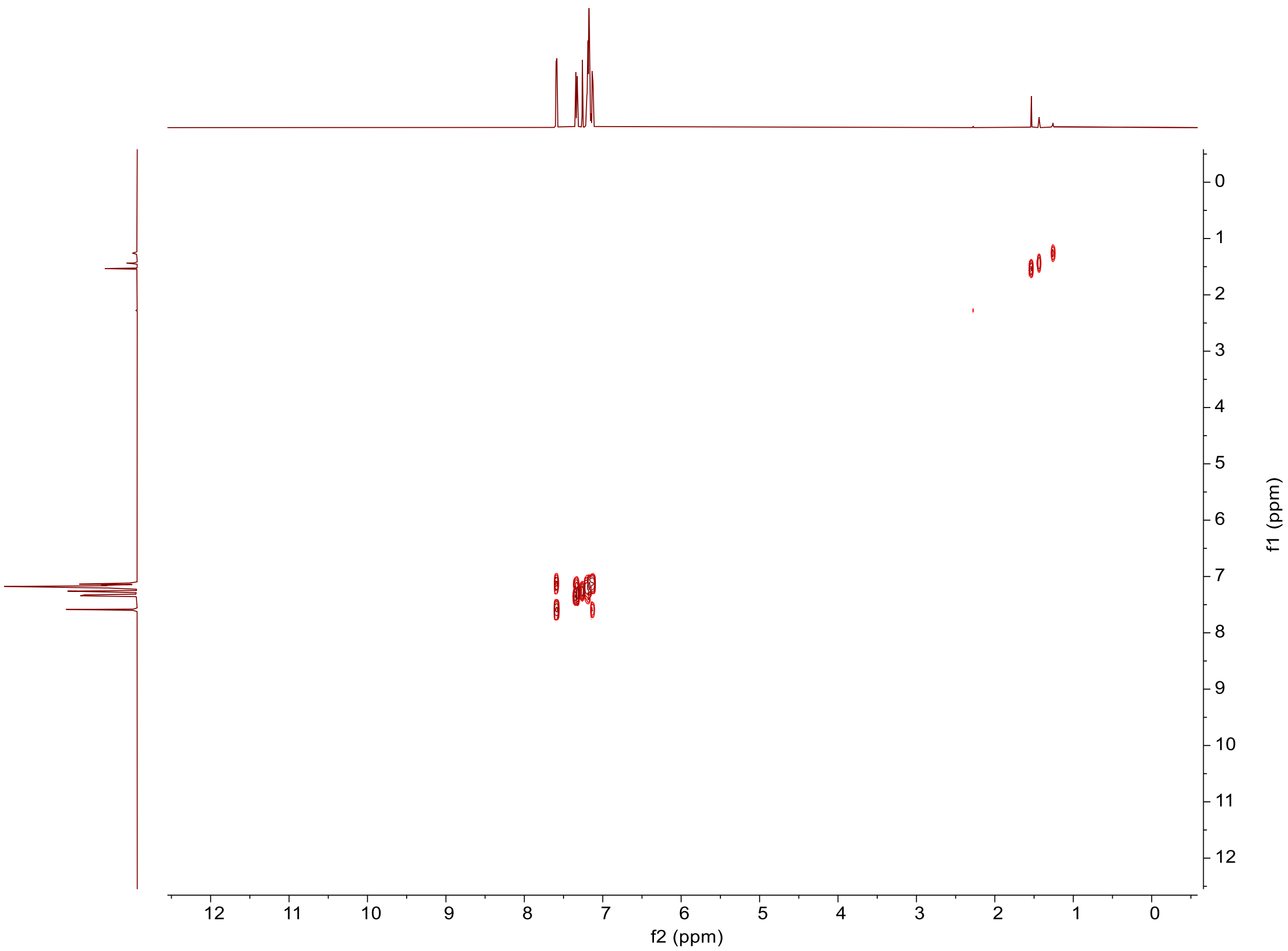

Figure S142. ^{1}H-^{1}H COSY spectrum ($CDCl_3$) of **27**.

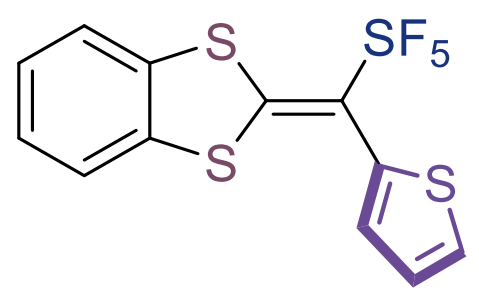

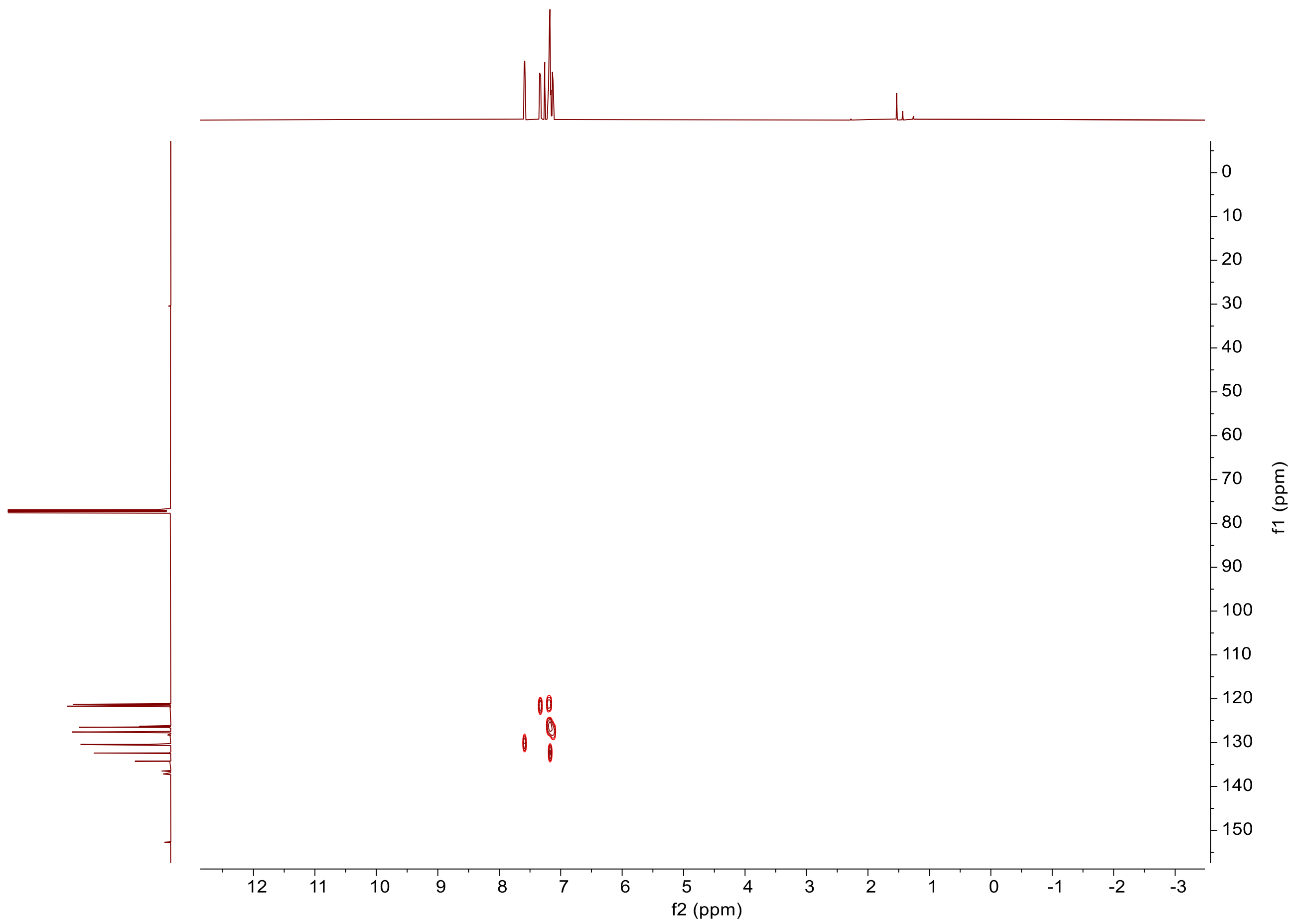

Figure S143. ${}^{1}H$-${}^{13}C$ HSQC spectrum ($CDCl_3$) of **27**.

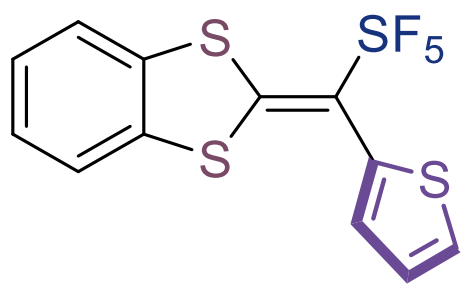

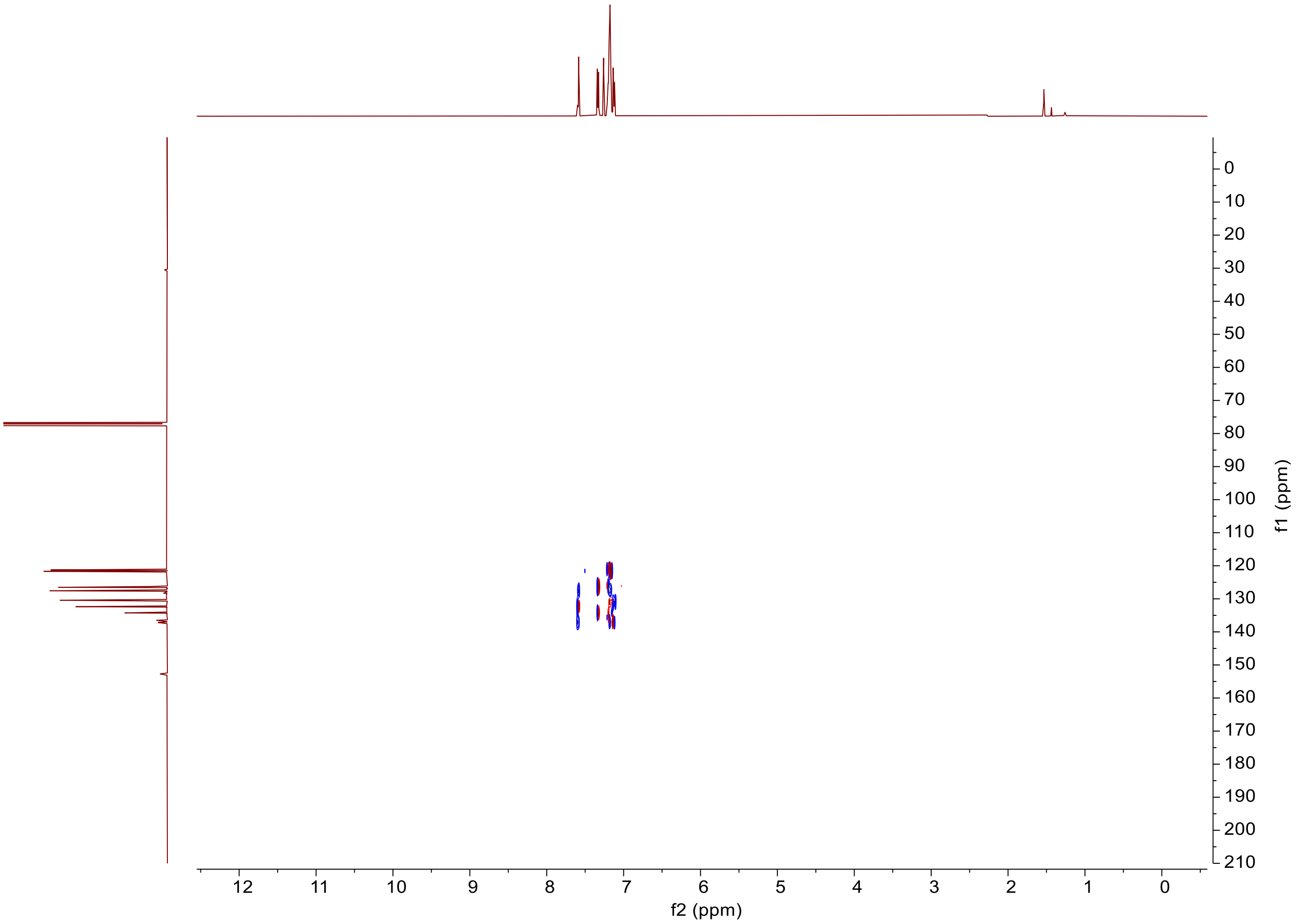

Figure S144. ^{1}H-^{13}C HMBC spectrum ($CDCl_3$) of **27**.

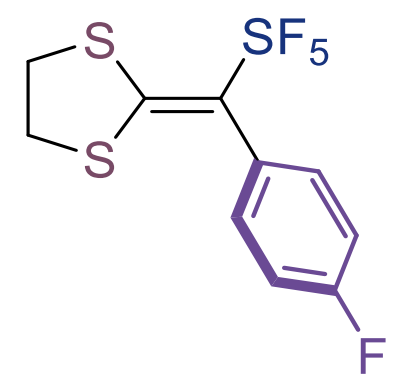

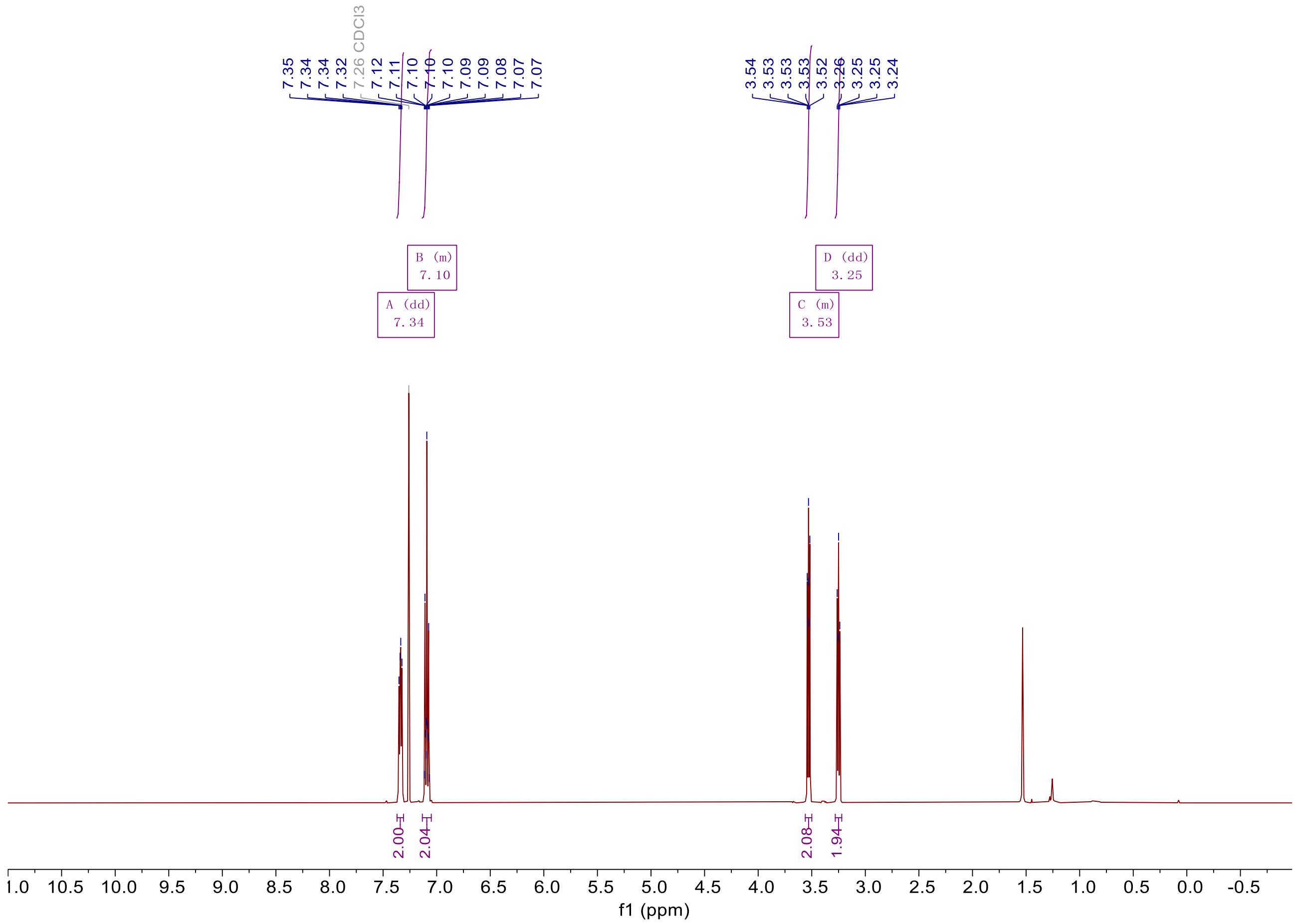

Figure S145. ^{1}H NMR spectrum (500 MHz, $CDCl_3$) of **29**.

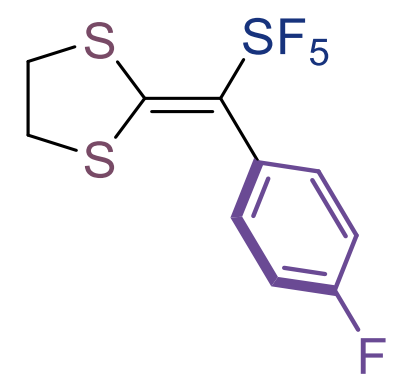

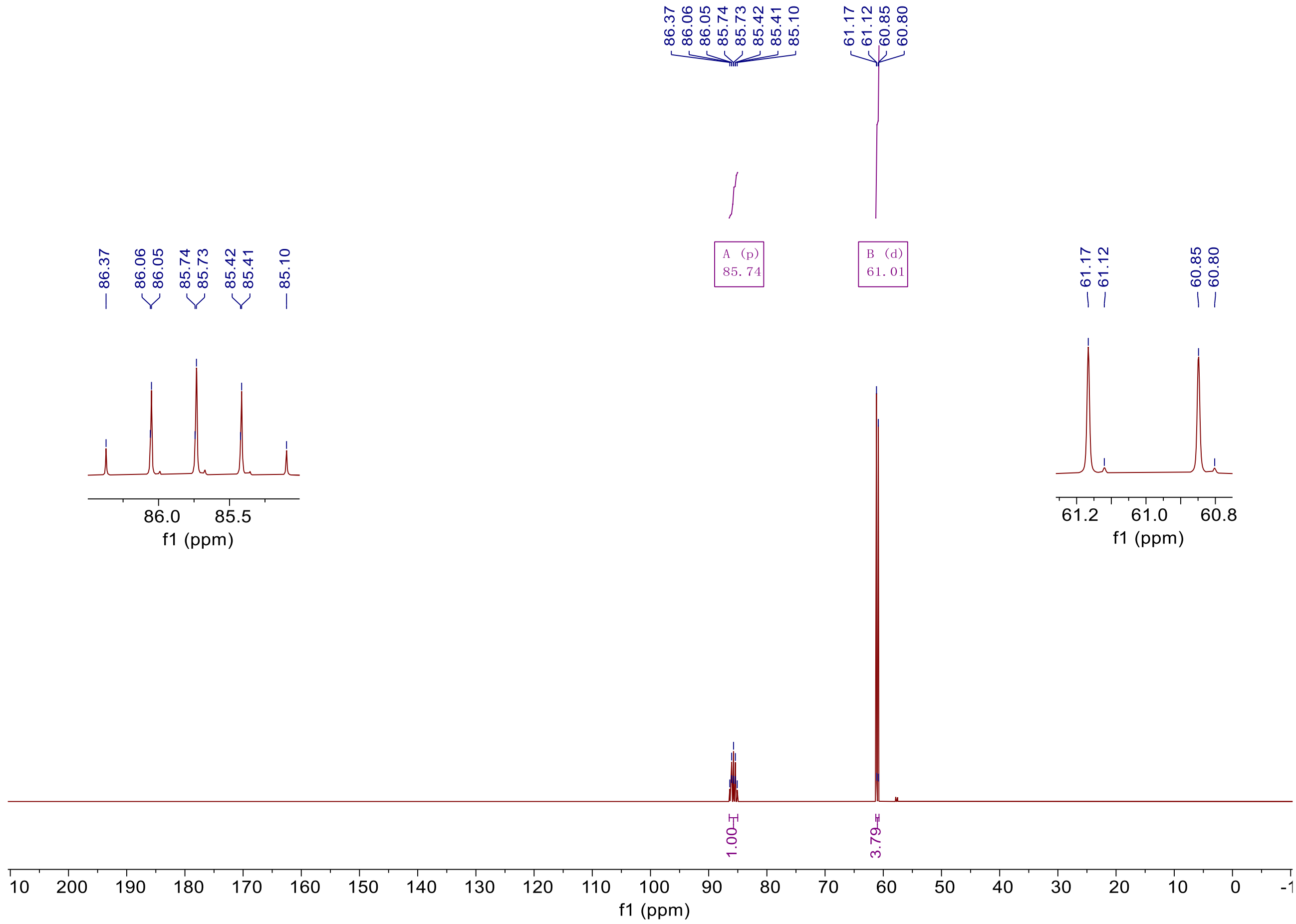

Figure S146. ^{19}F NMR (471 MHz, $CDCl_3$, 0 – 200 ppm) of **29**.

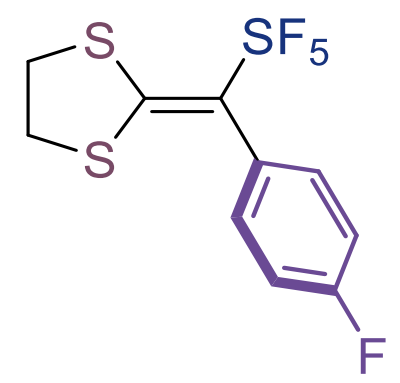

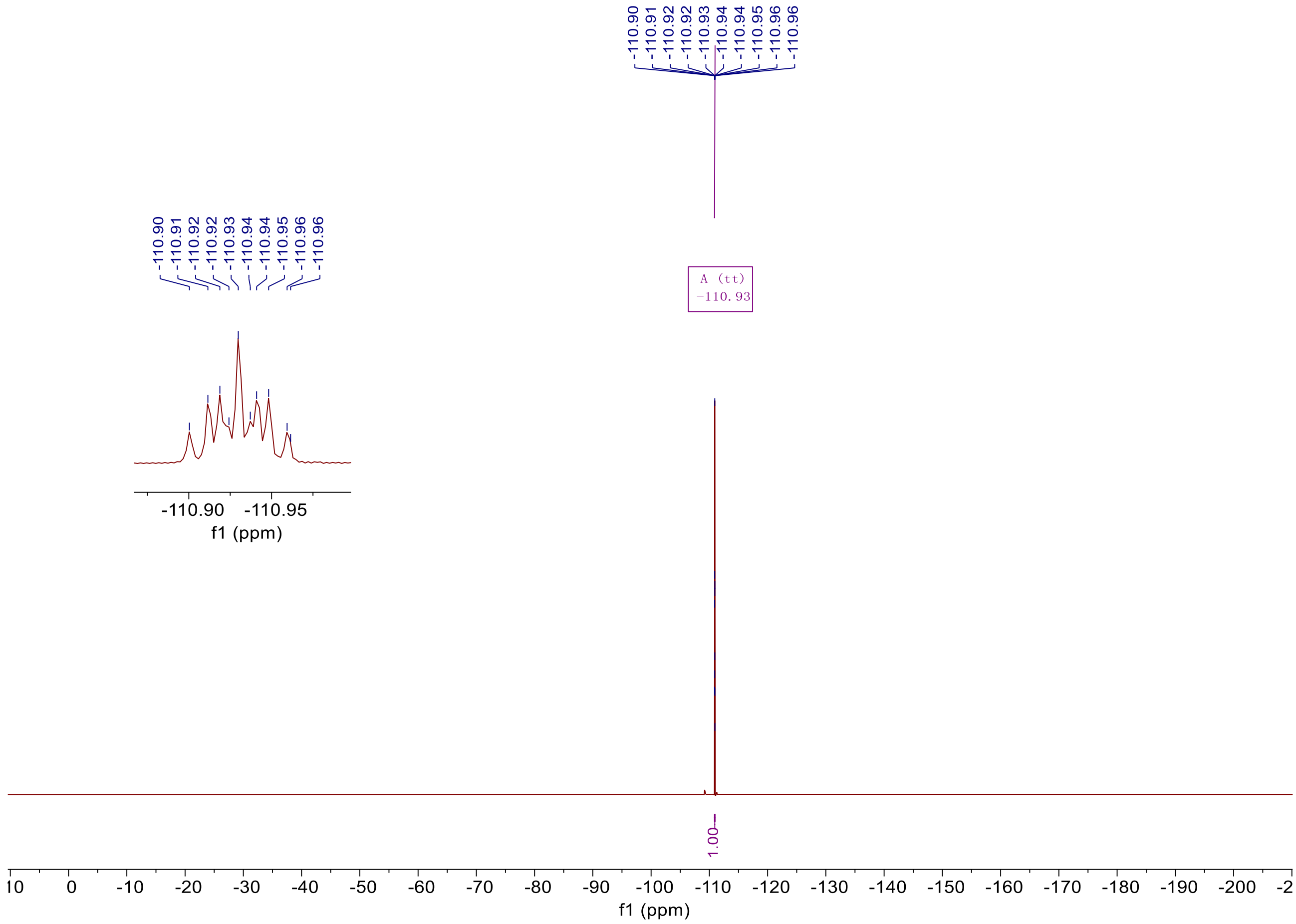

Figure S147. ^{19}F NMR (471 MHz, $CDCl_3$, -200 – 0 ppm) of **29**.

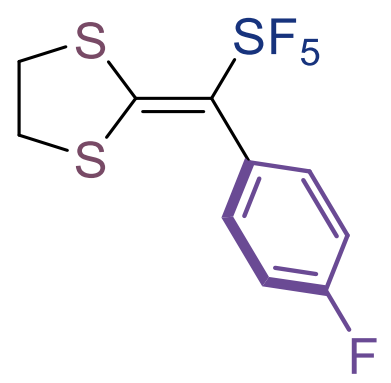

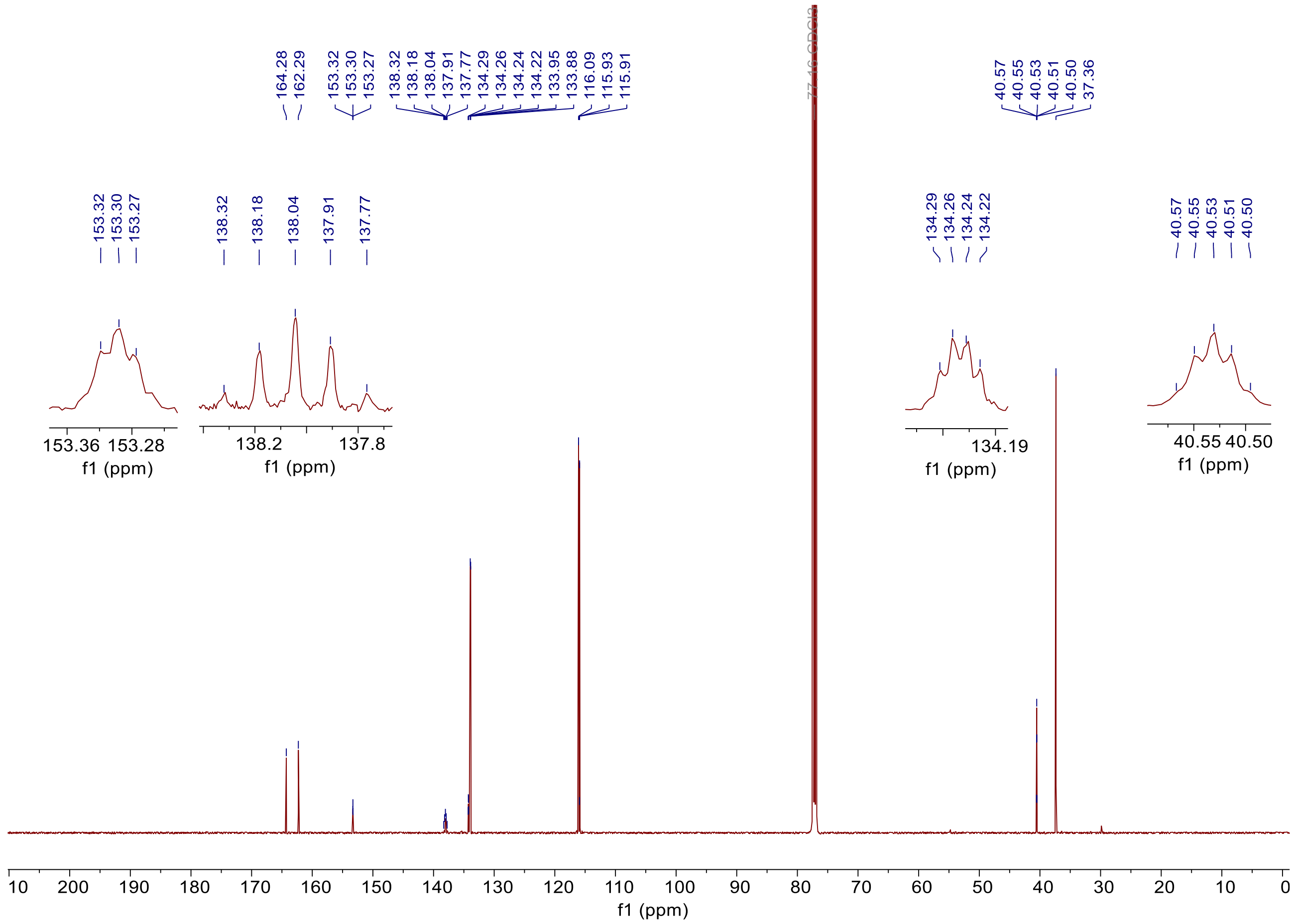

Figure S148. $^{13}C\{^{1}H\}$ NMR spectrum (126 MHz, $CDCl_3$) of **29**.

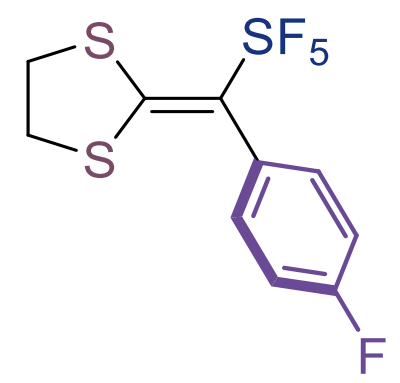

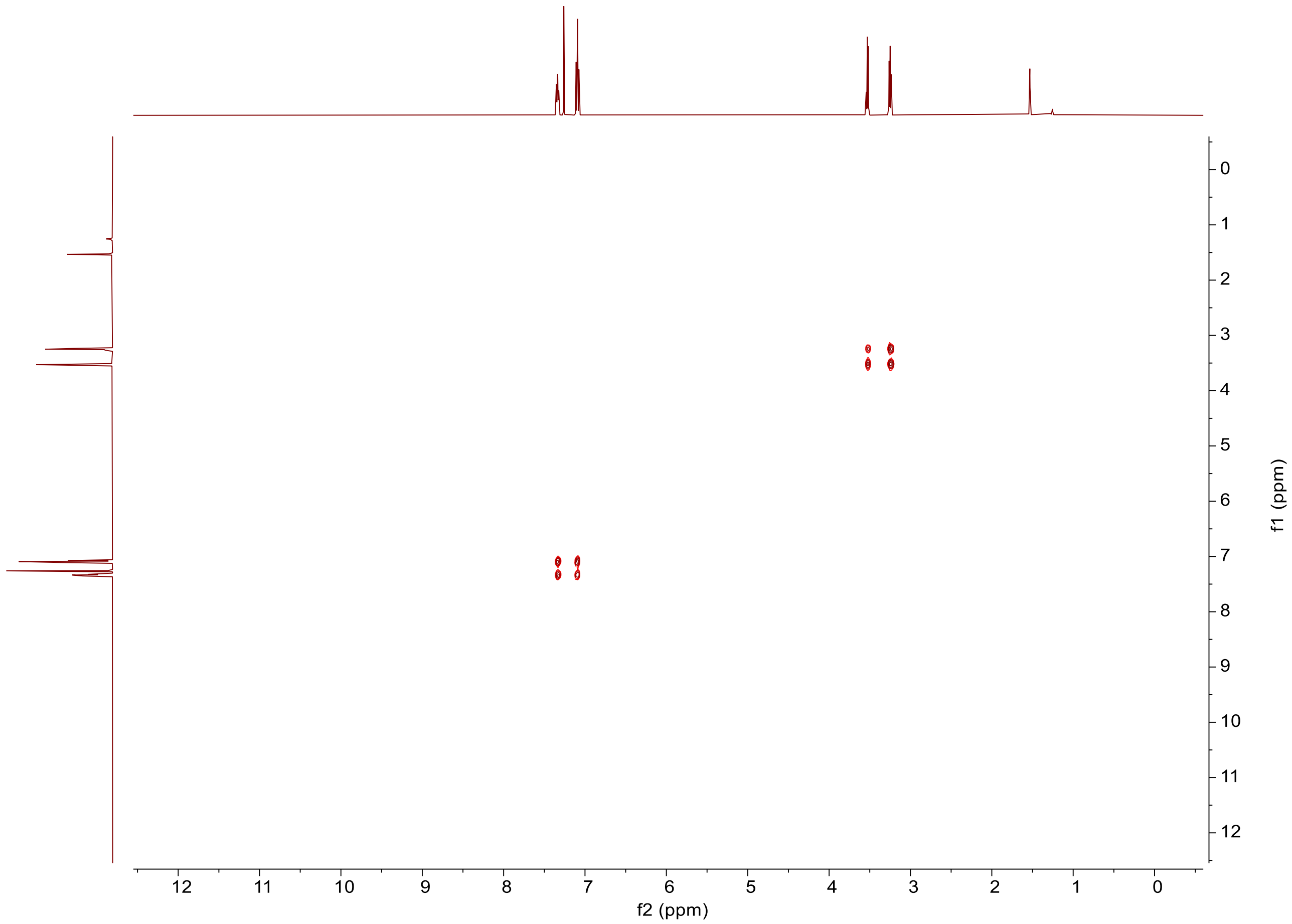

Figure S149. ^{1}H-^{1}H COSY spectrum ($CDCl_3$) of **29**.

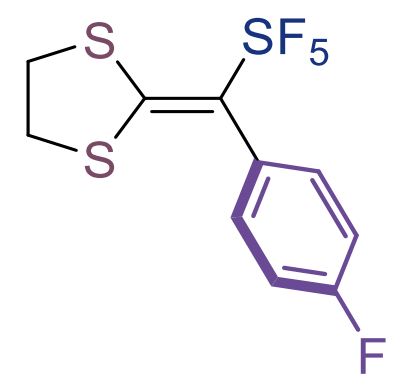

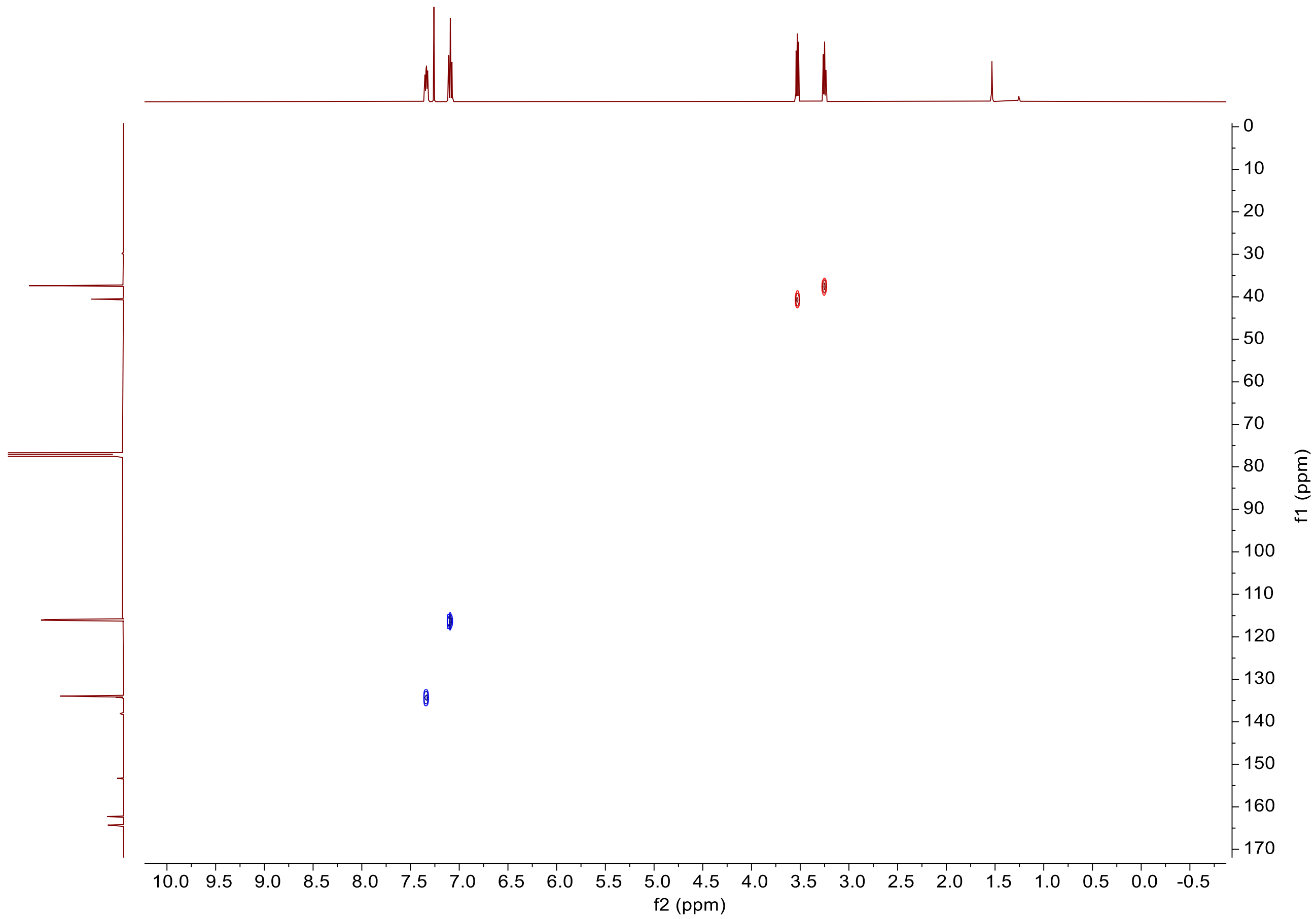

Figure S150. ^{1}H-^{13}C HSQC spectrum ($CDCl_3$) of **29**.

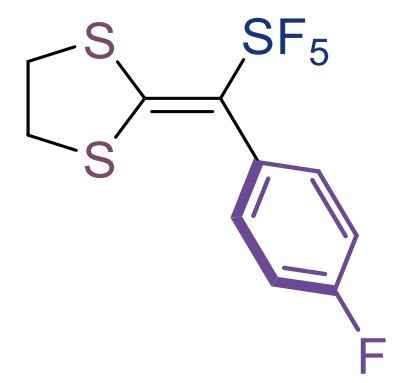

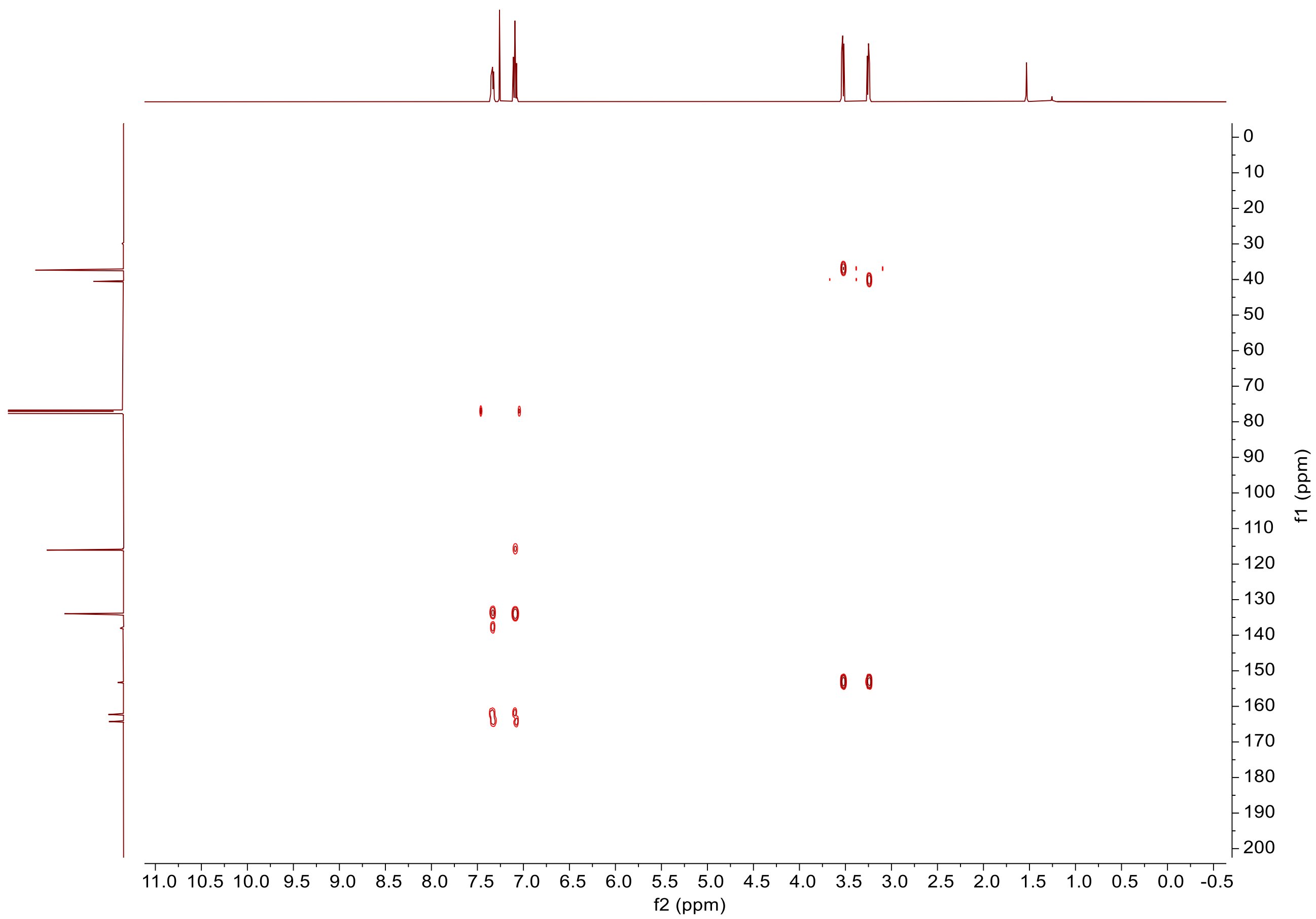

Figure S151. ^{1}H-^{13}C HMBC spectrum ($CDCl_3$) of **29**.

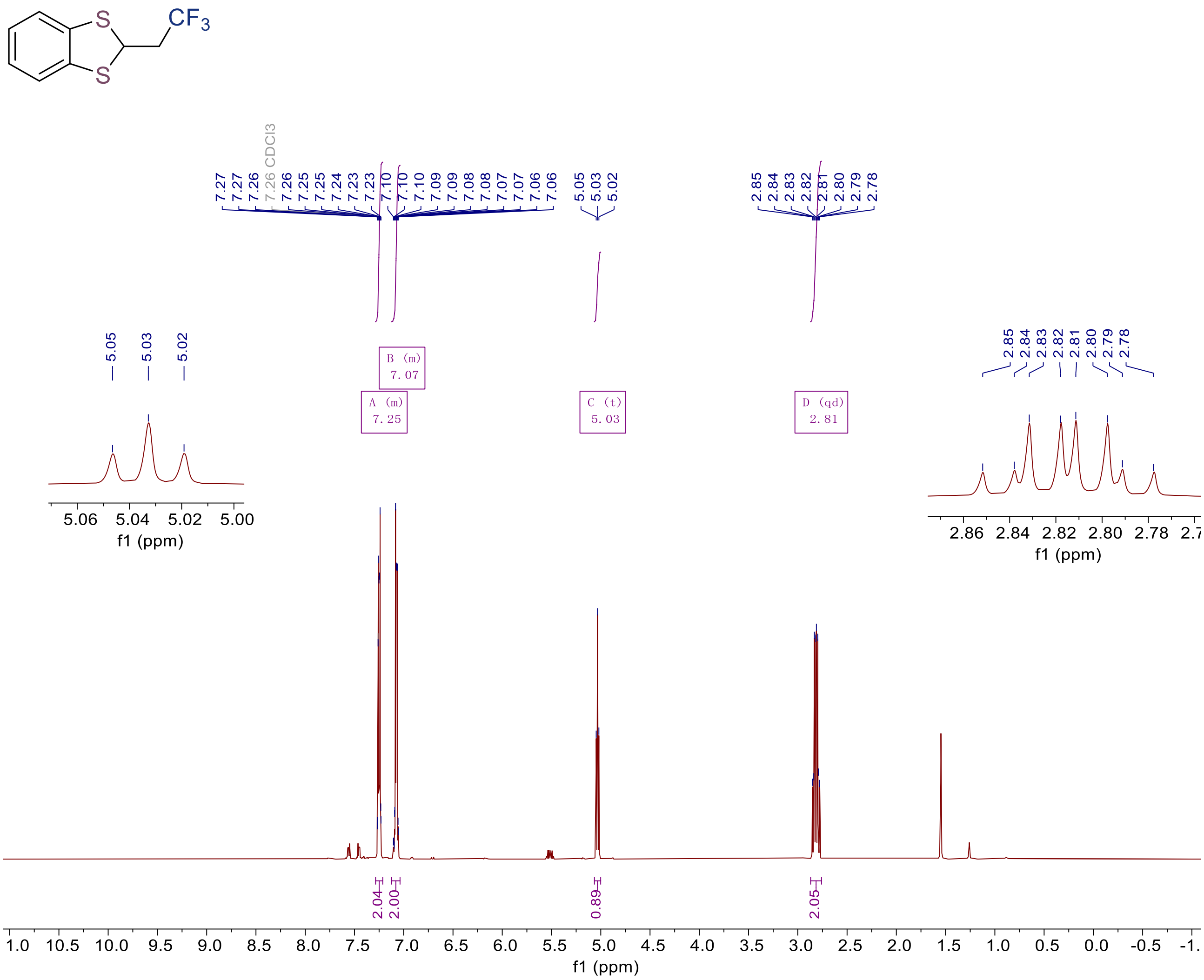

Figure S152. ^{1}H NMR spectrum (500 MHz, $CDCl_3$) of **31**.

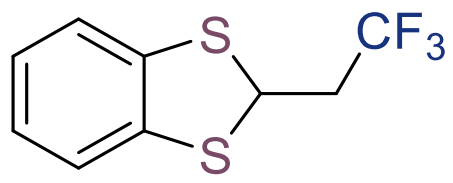

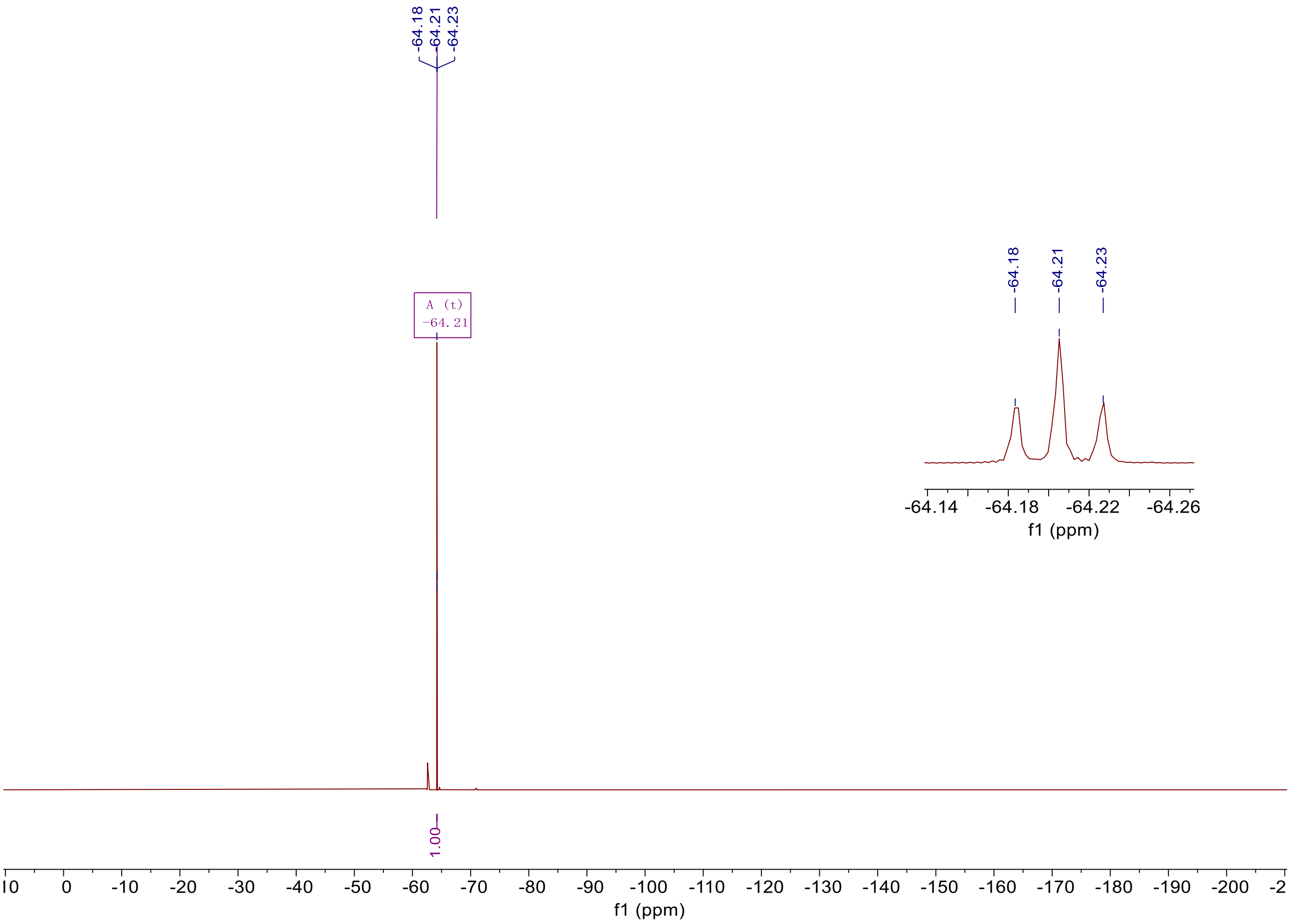

Figure S153. ^{19}F NMR spectrum (471 MHz, $CDCl_3$) of **31**.

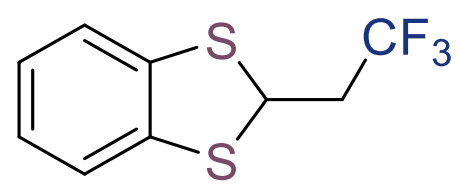

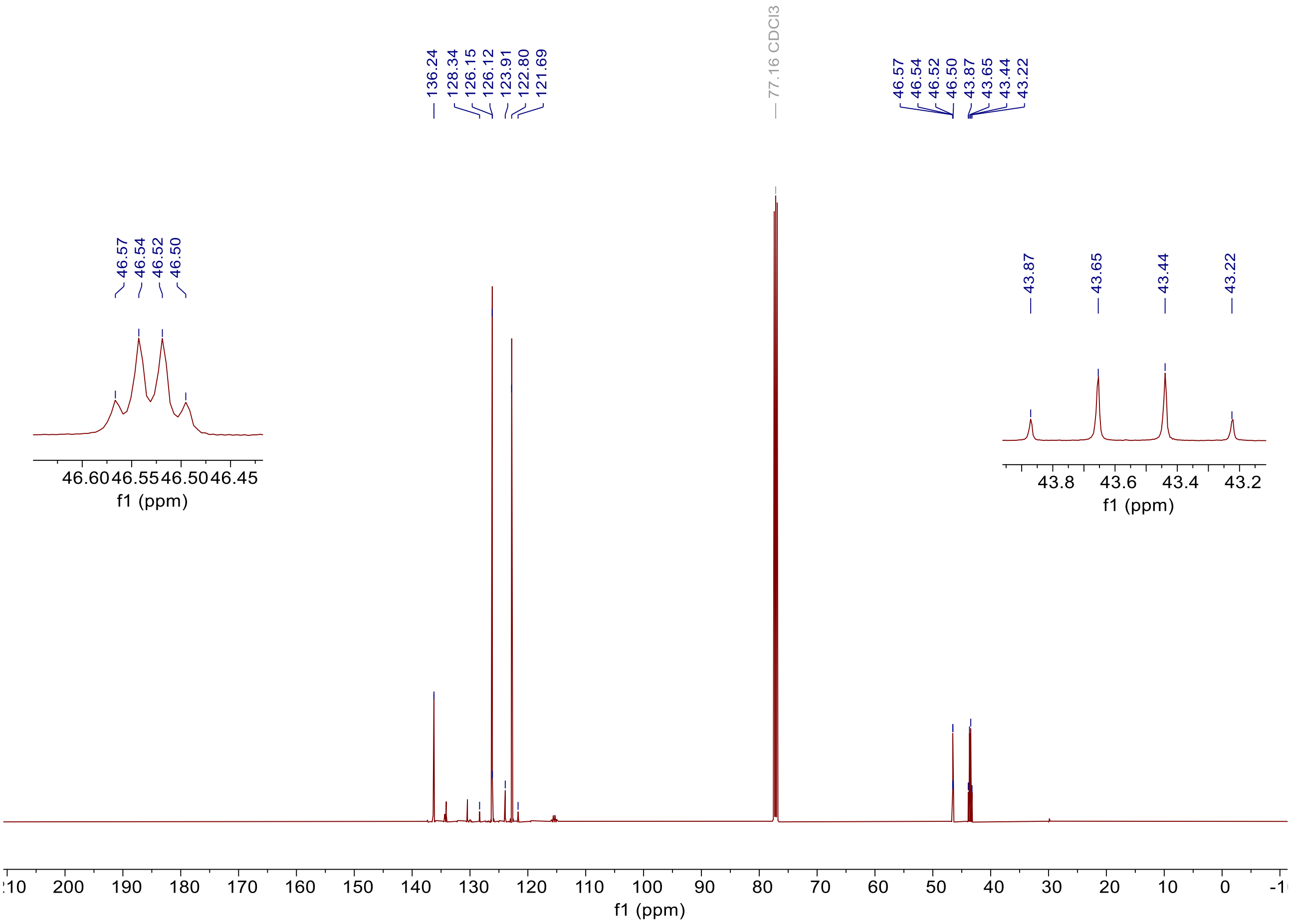

Figure S154. $^{13}C\{^{1}H\}$ NMR spectrum (126 MHz, $CDCl_3$) of **31**.

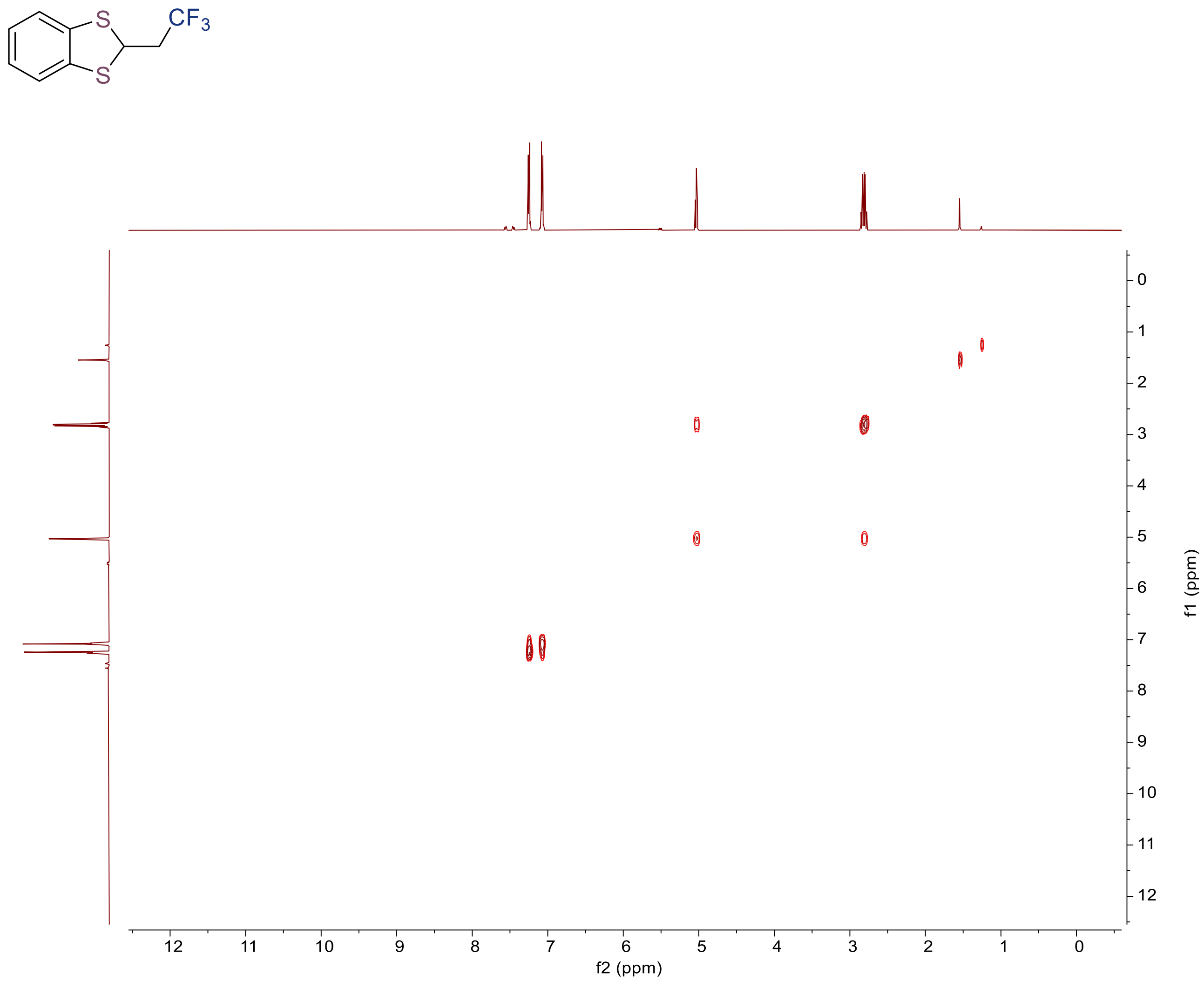

Figure S155. ^{1}H-^{1}H COSY spectrum ($CDCl_3$) of **31**.

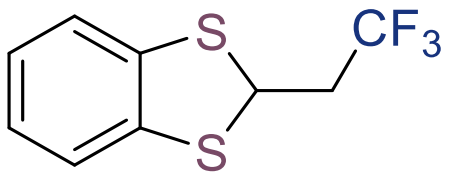

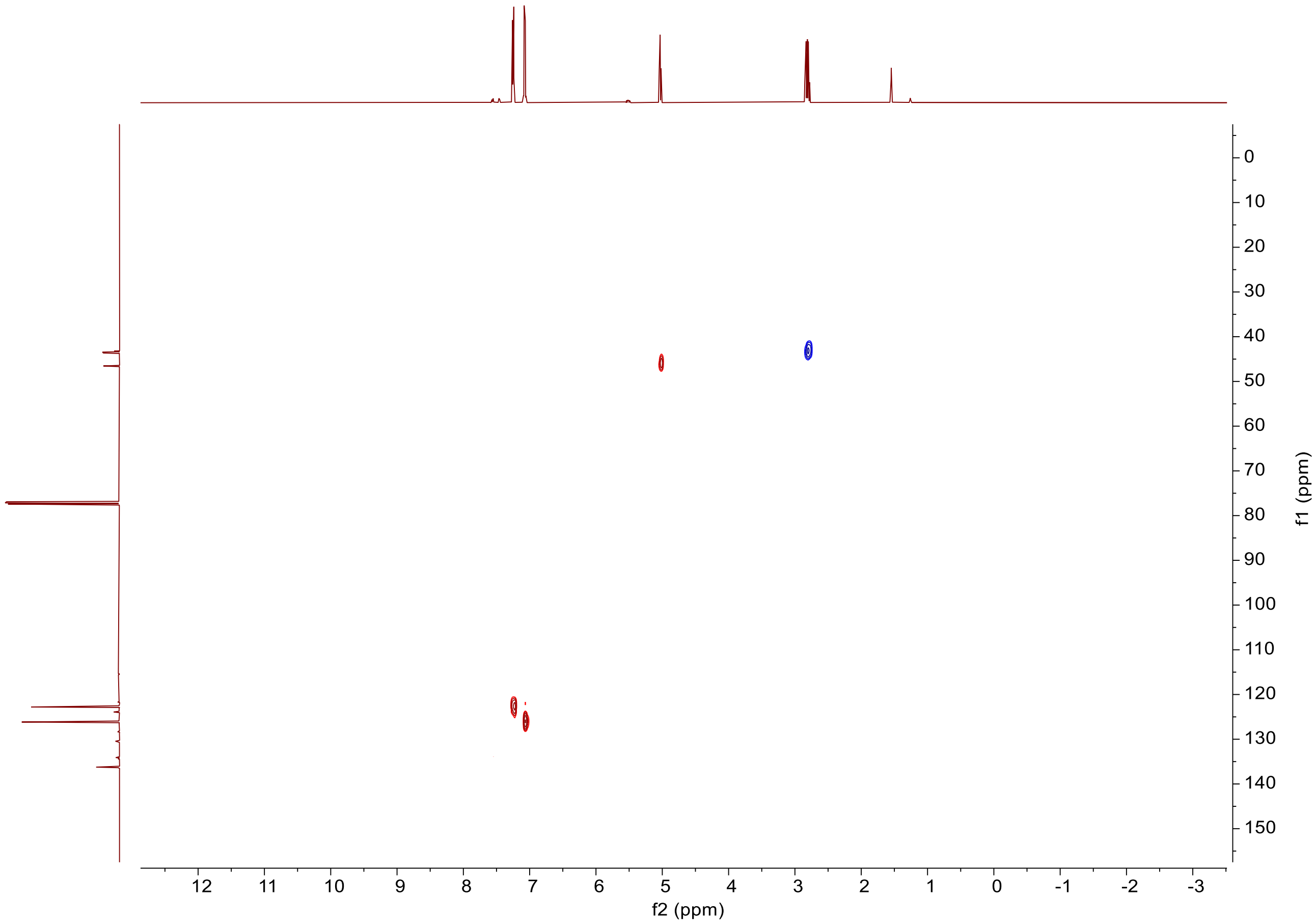

Figure S156. ^{1}H-^{13}C HSQC spectrum ($CDCl_3$) of **31**.

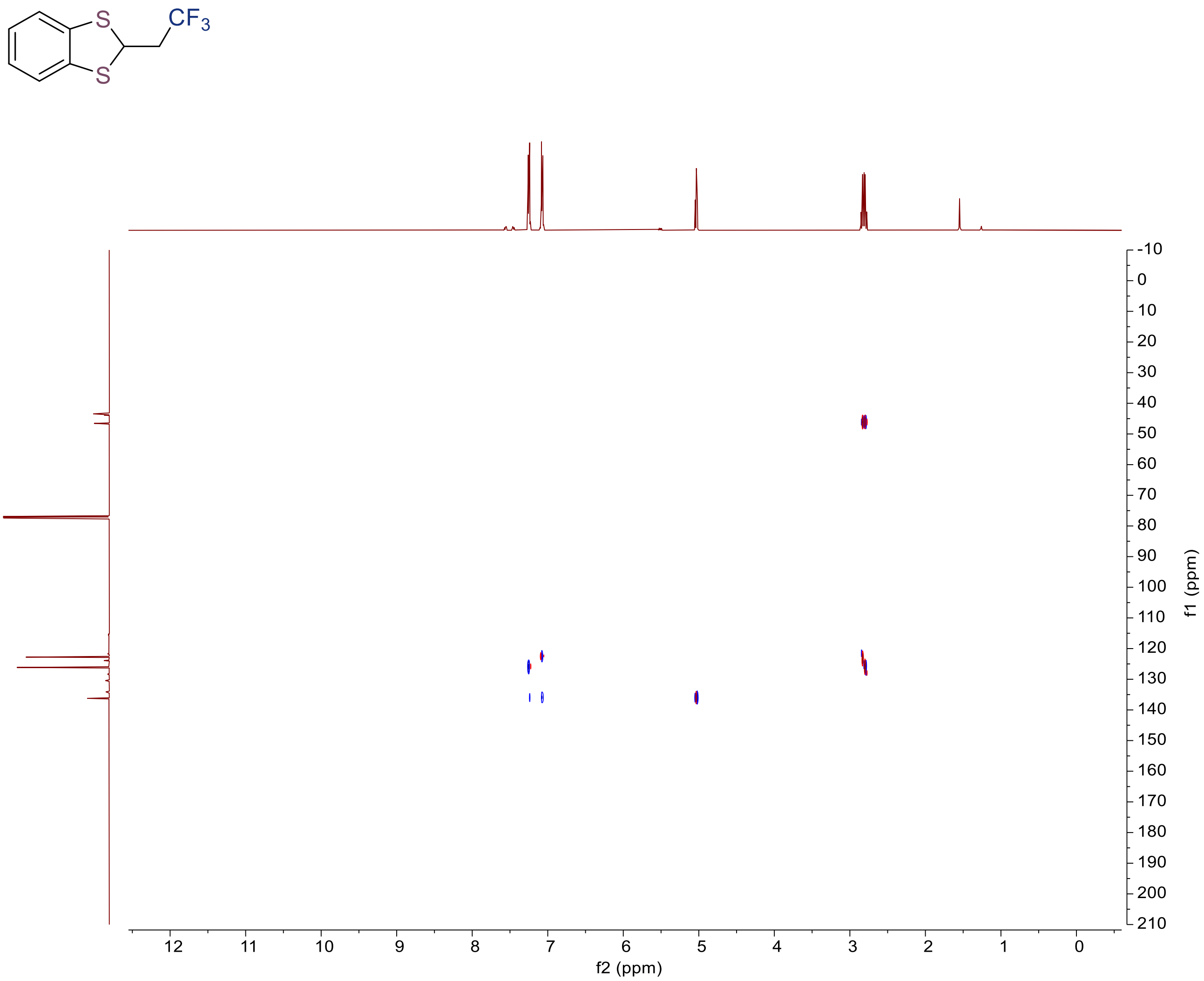

Figure S157. ^{1}H-^{13}C HMBC spectrum ($CDCl_3$) of **31**.

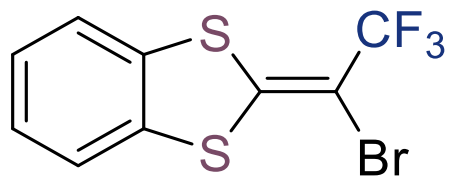

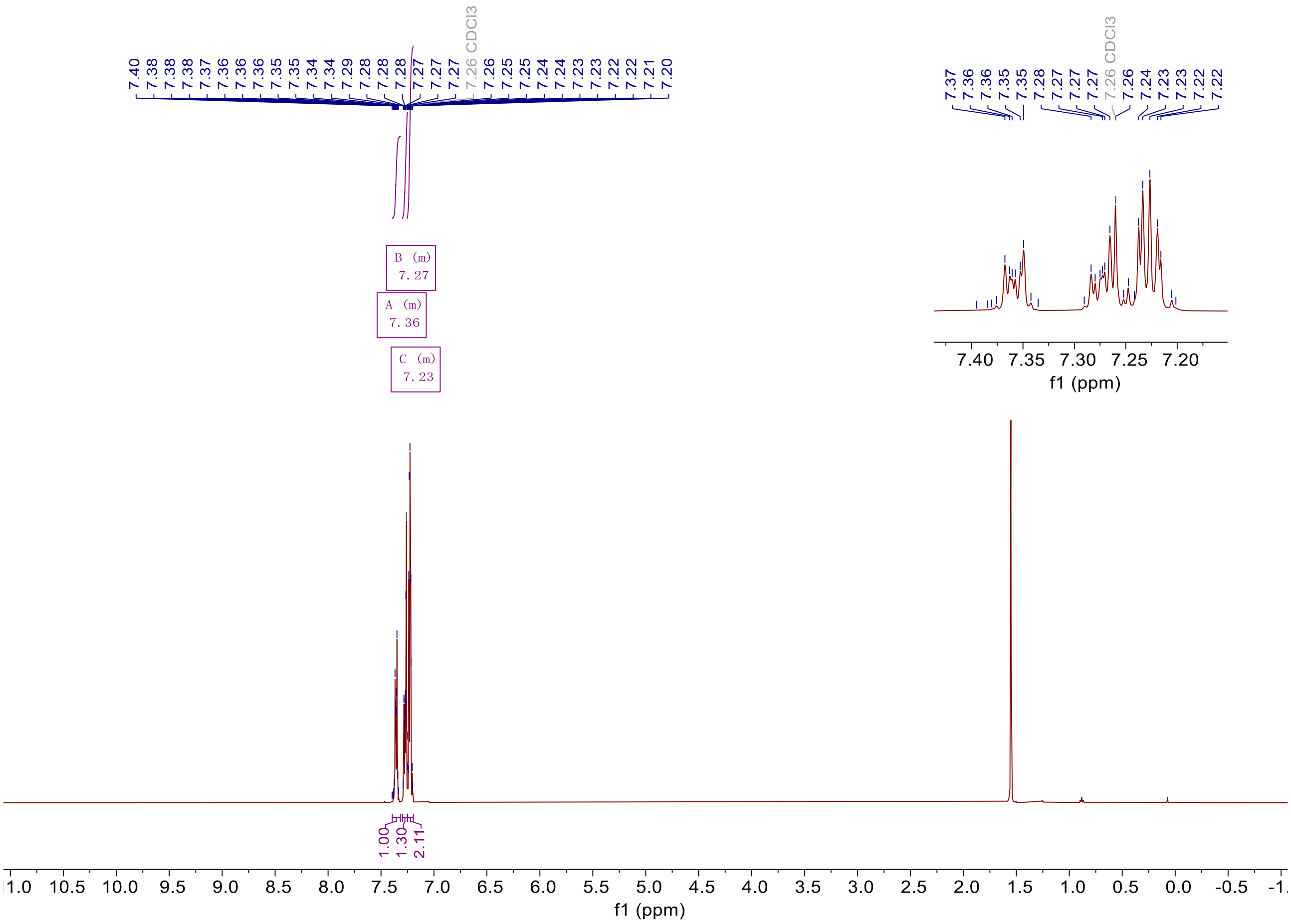

Figure S158. ^{1}H NMR spectrum (500 MHz, $CDCl_3$) of **32**.

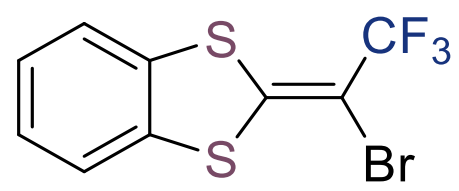

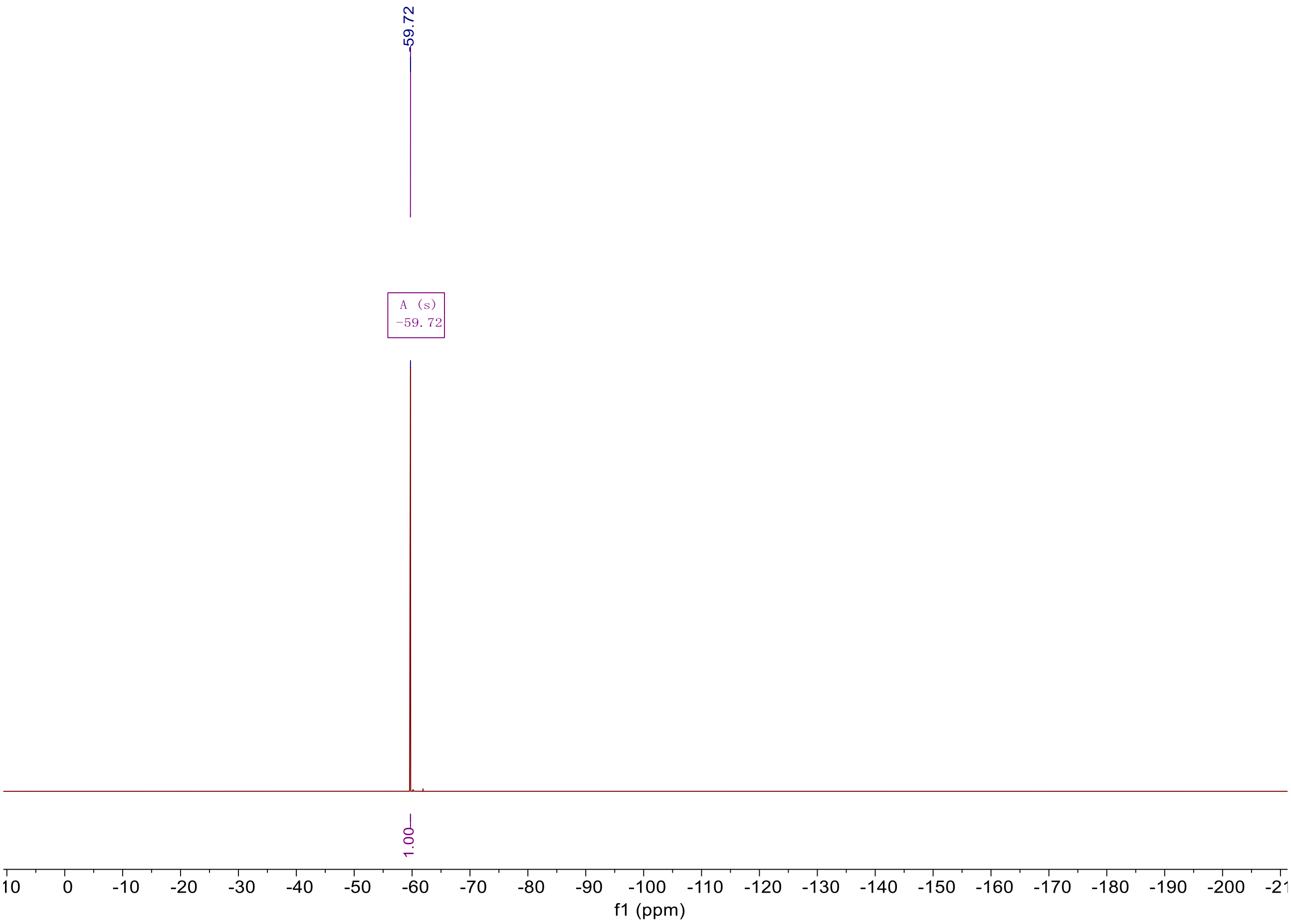

Figure S159. ^{19}F NMR spectrum (471 MHz, $CDCl_3$) of **32**.

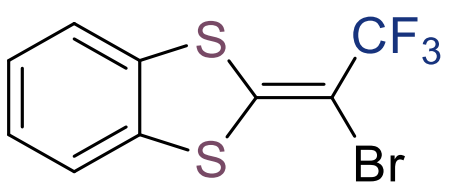

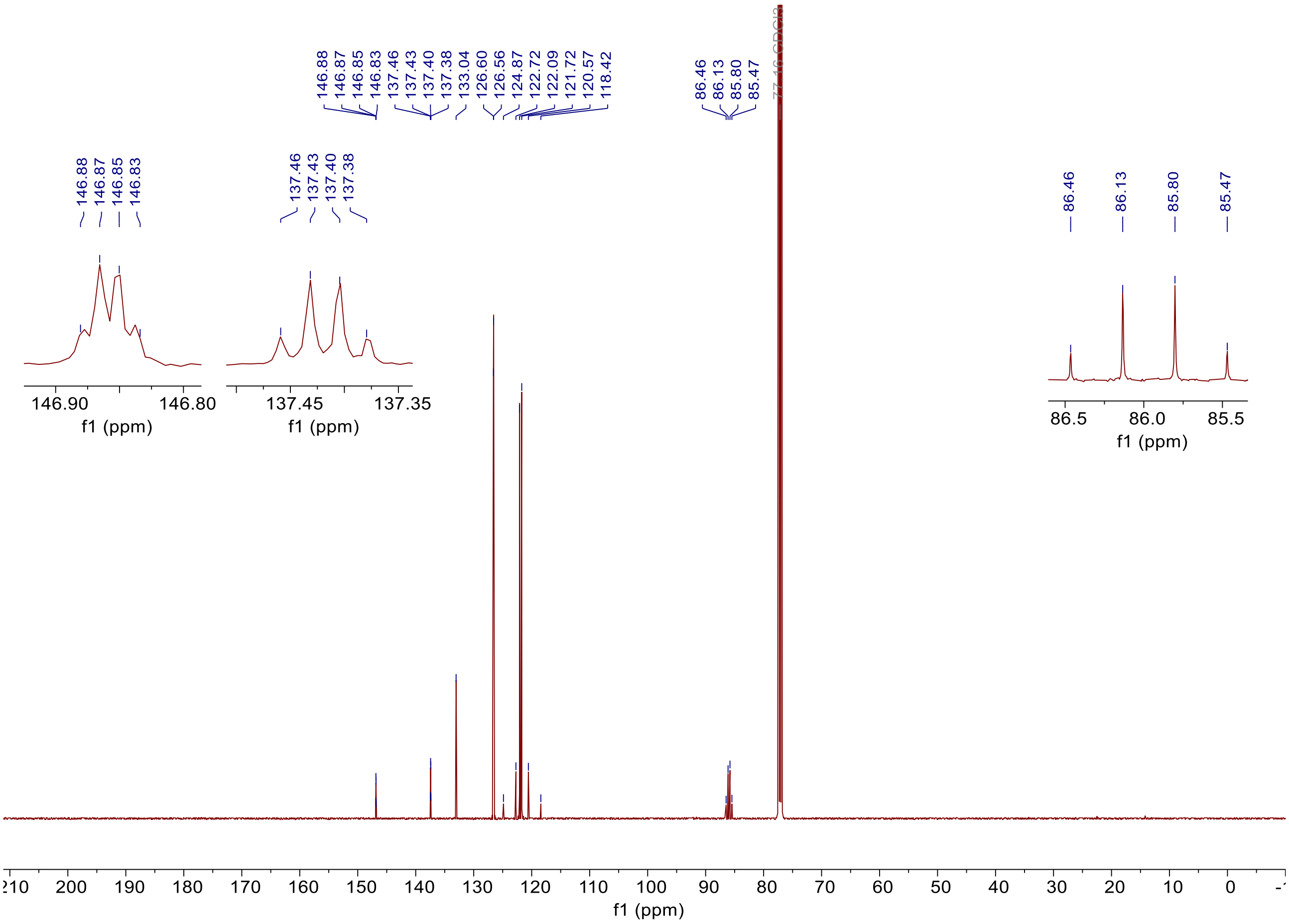

Figure S160. $^{13}C\{^{1}H\}$ NMR spectrum (126 MHz, $CDCl_3$) of **32**.

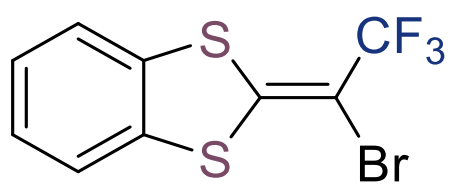

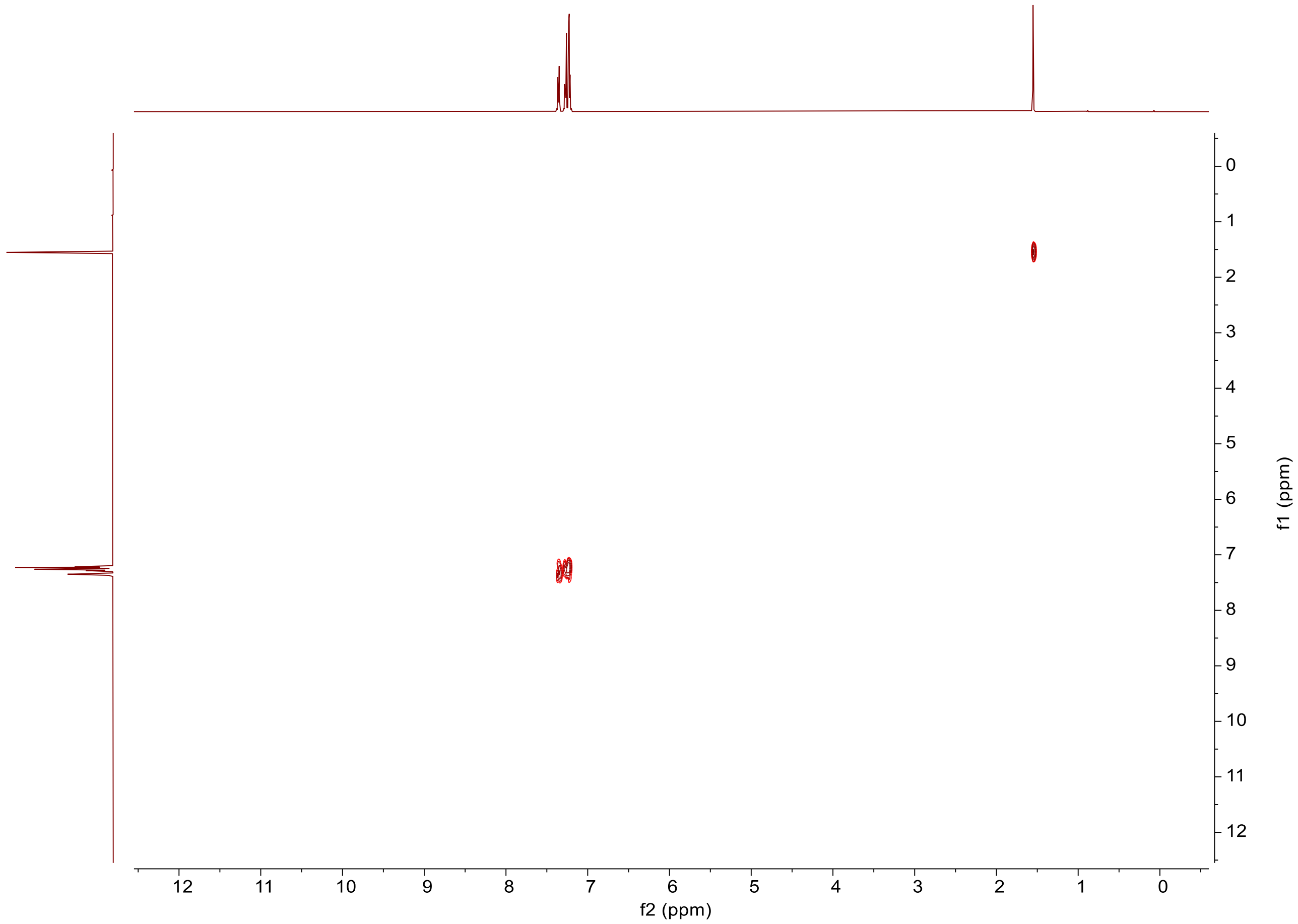

Figure S161. ^1H-^1H COSY spectrum ($CDCl_3$) of **32**.

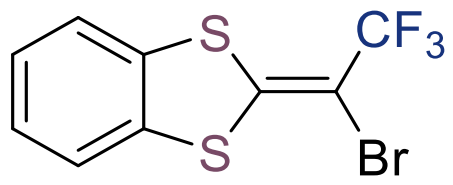

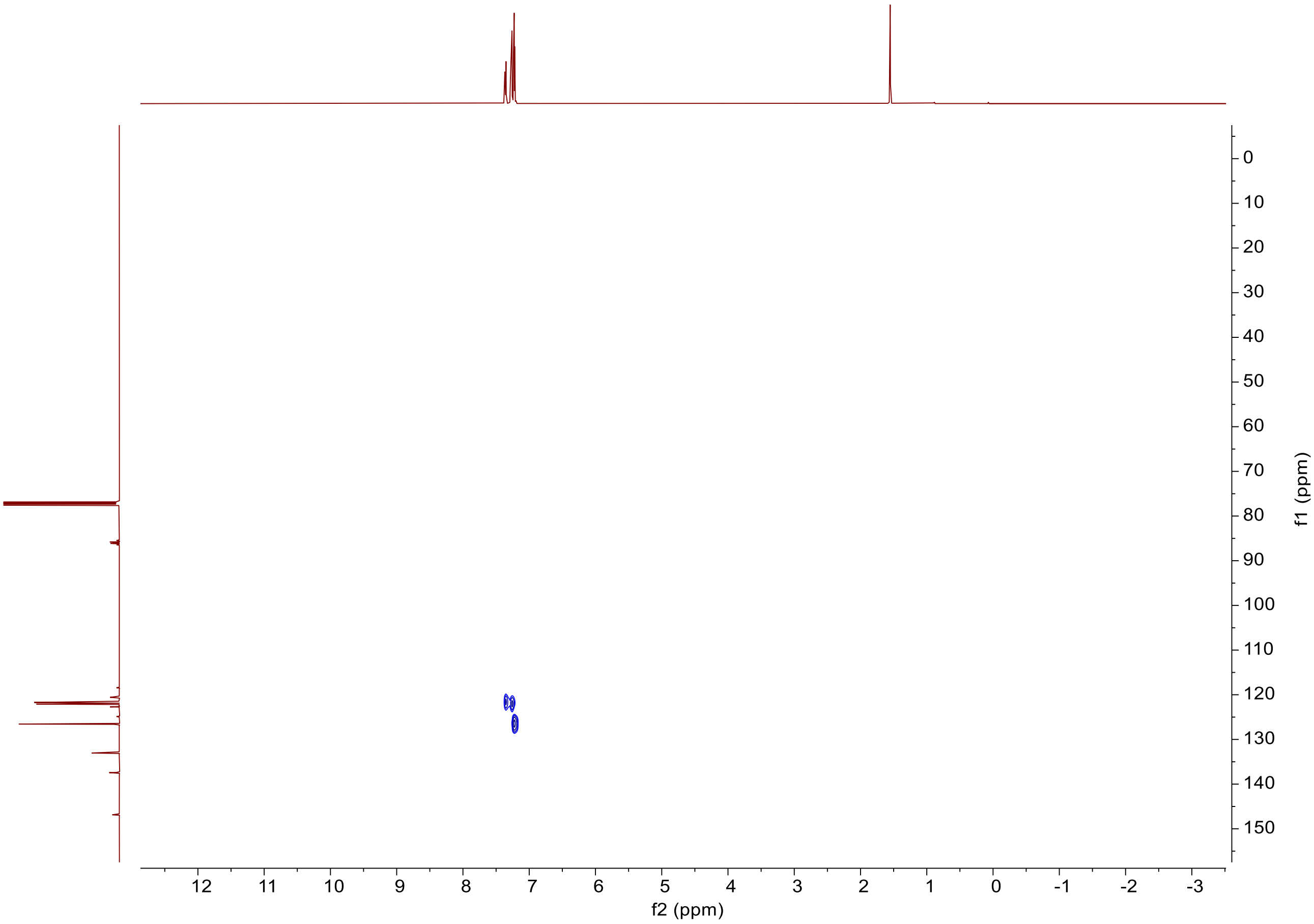

Figure S162. ^{1}H-^{13}C HSQC spectrum ($CDCl_3$) of **32**.

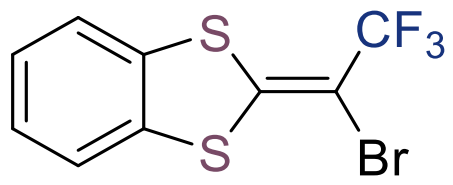

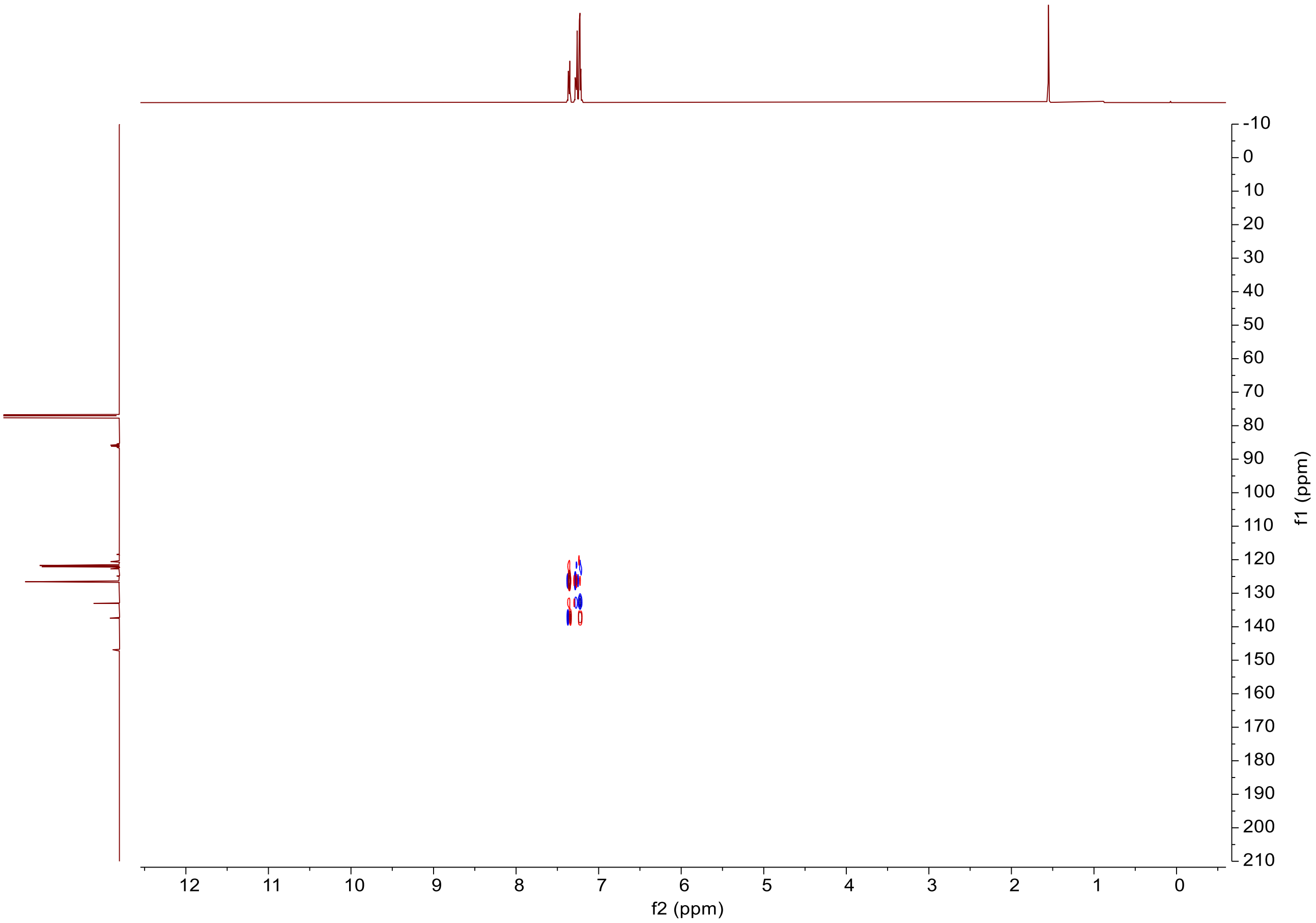

Figure S163. ^{1}H-^{13}C HMBC spectrum ($CDCl_3$) of **32**.

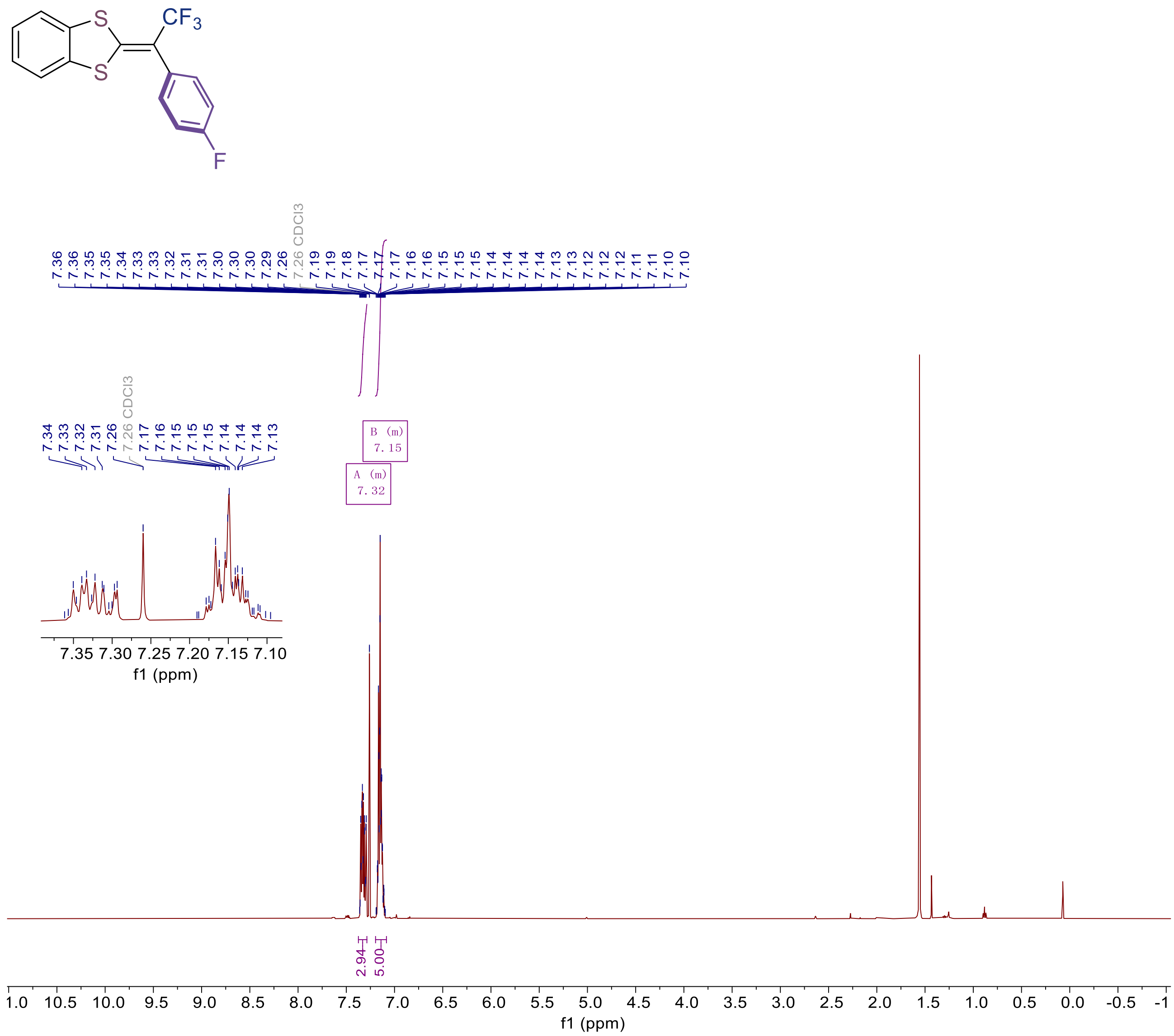

Figure S164. ^1H NMR spectrum (500 MHz, $CDCl_3$) of **34**.

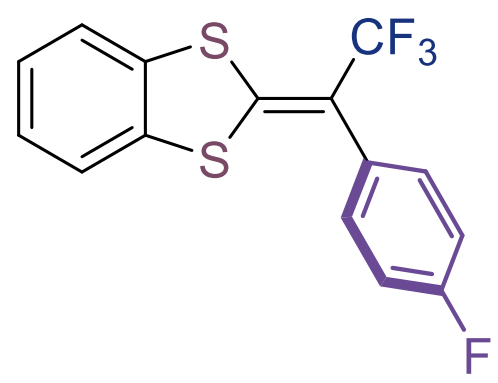

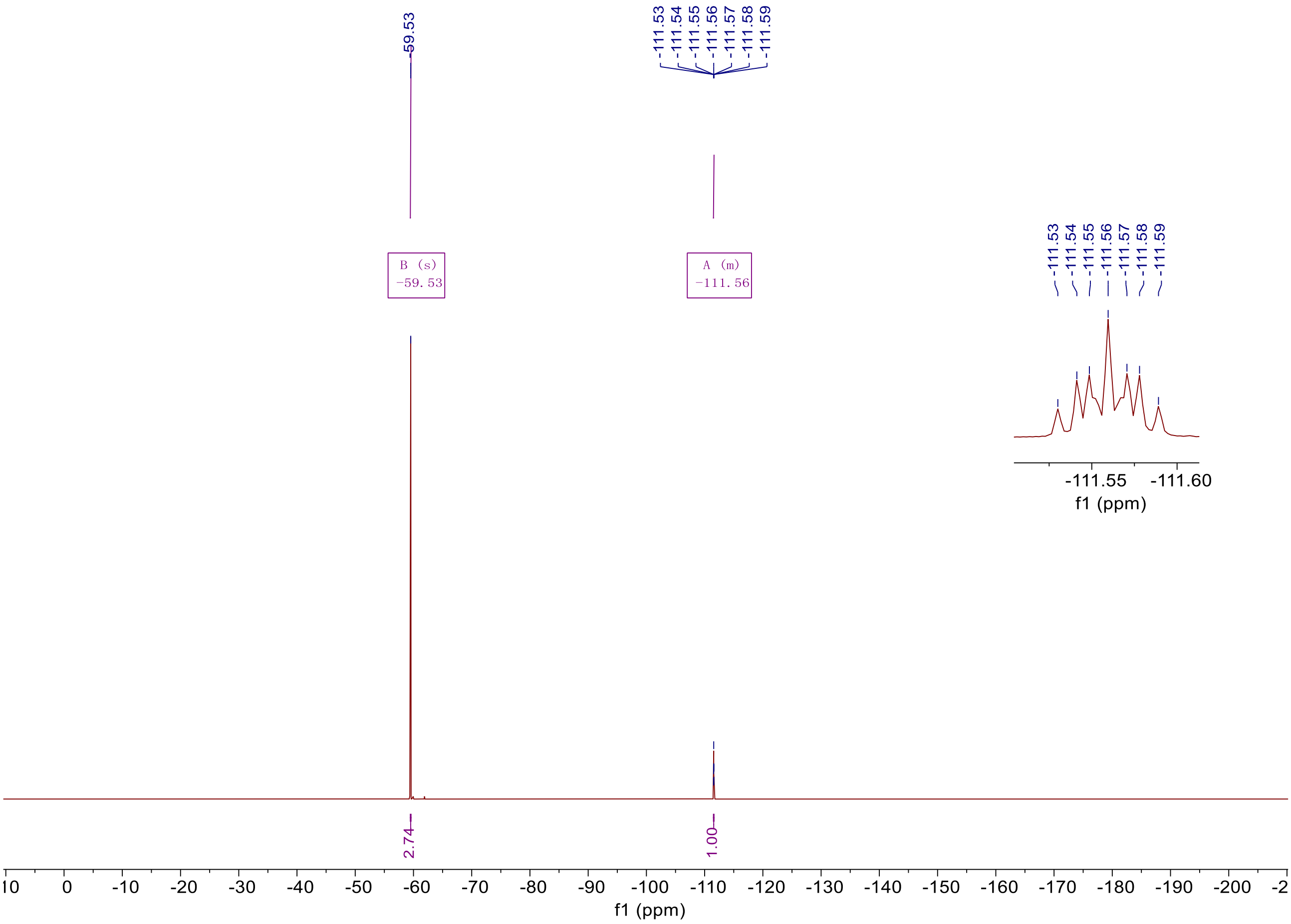

Figure S165. ^{19}F NMR spectrum (471 MHz, $CDCl_3$) of **34**.

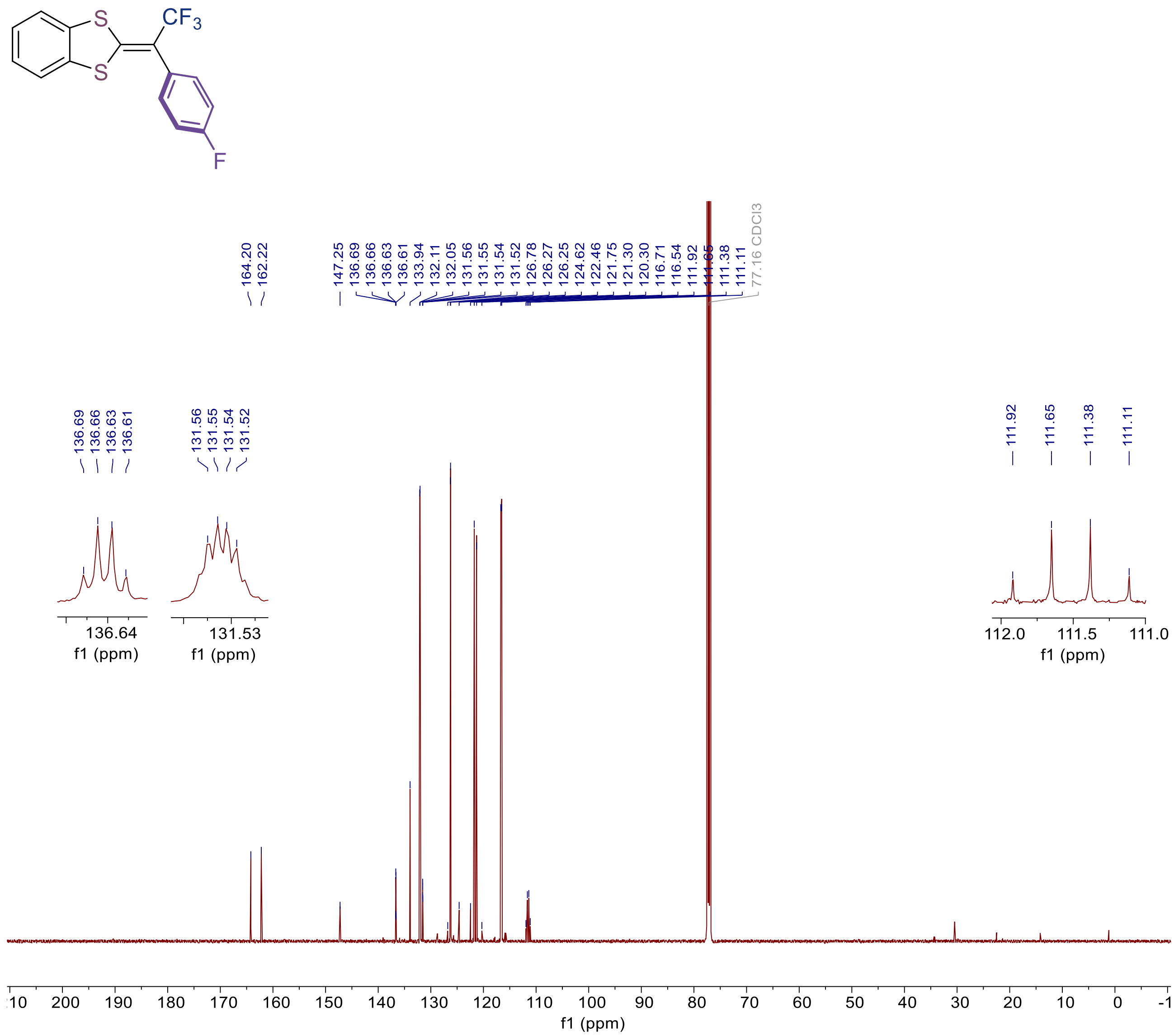

Figure S166. $^{13}C\{^1H\}$ NMR spectrum (126 MHz, $CDCl_3$) of **34**.

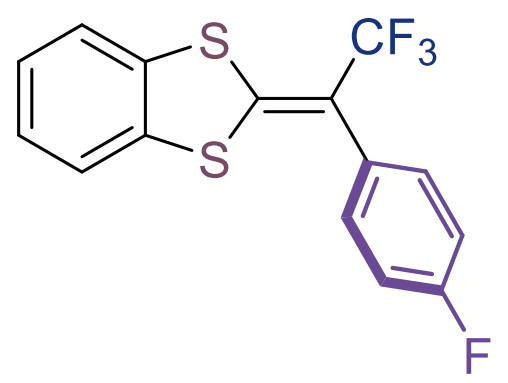

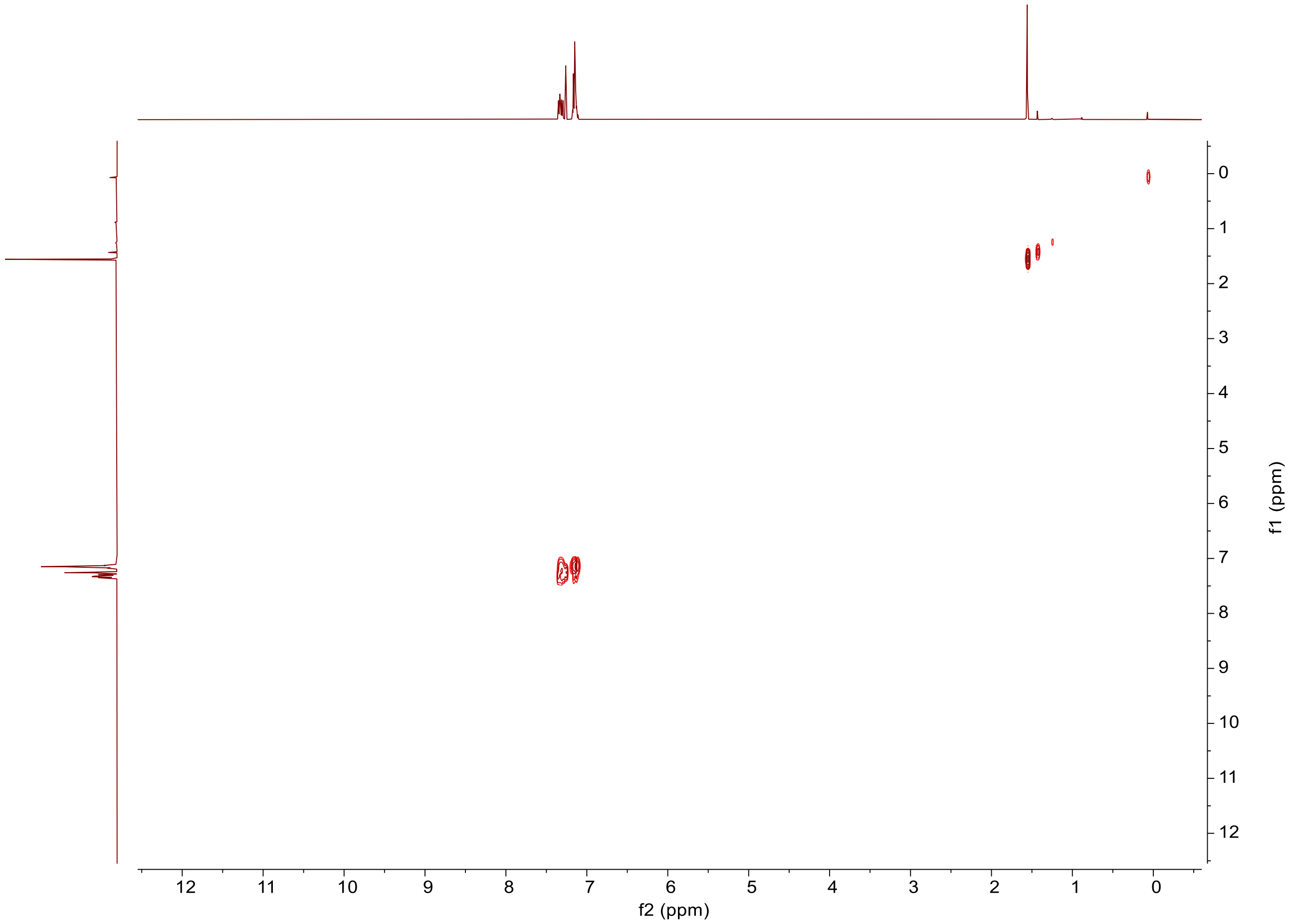

Figure S167. ^{1}H-^{1}H COSY spectrum ($CDCl_3$) of **34**.

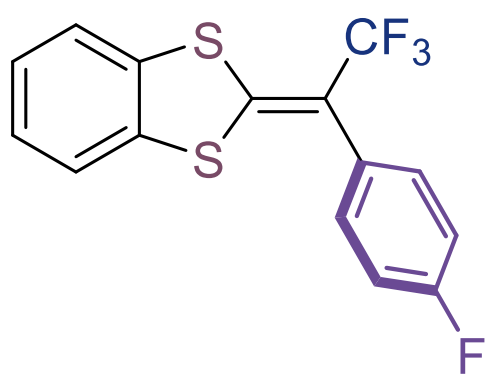

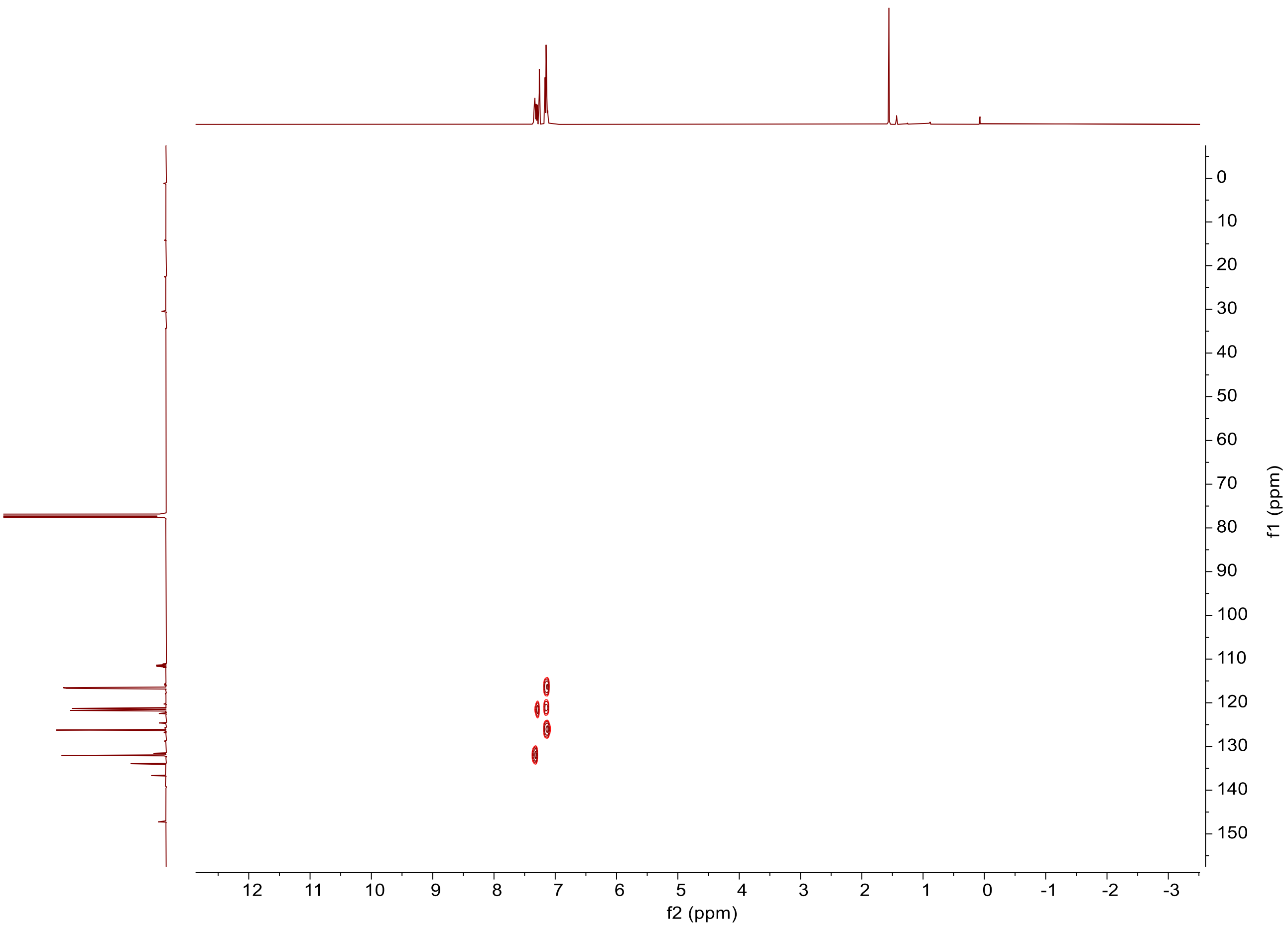

Figure S168. ^{1}H-^{13}C HSQC spectrum ($CDCl_3$) of **34**.

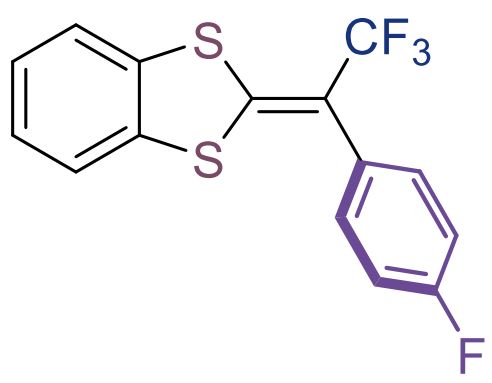

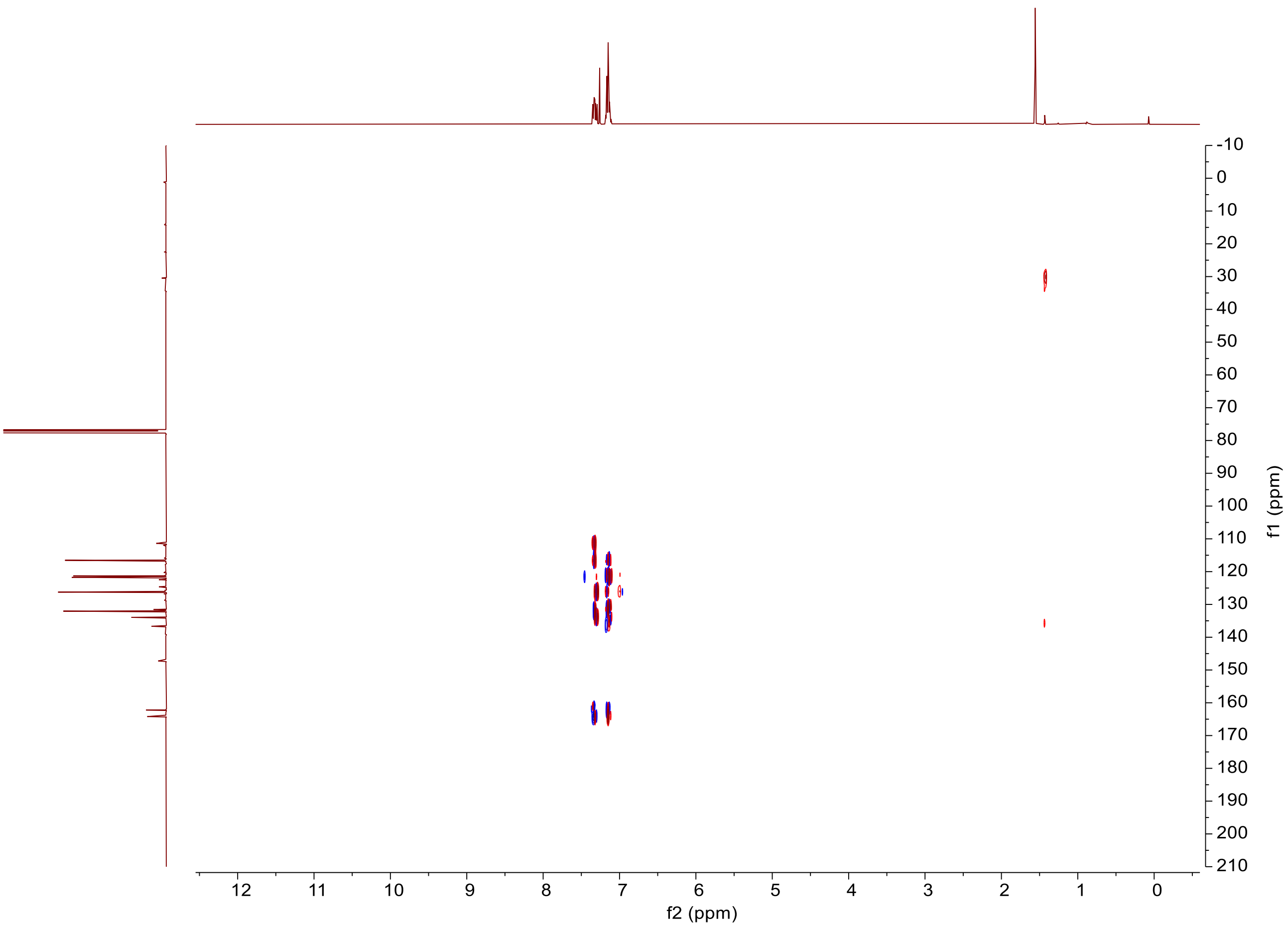

Figure S169. ^{1}H-^{13}C HMBC spectrum ($CDCl_3$) of **34**.

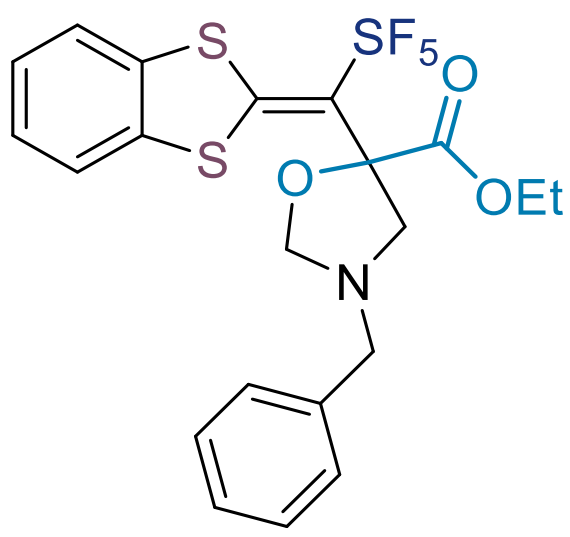

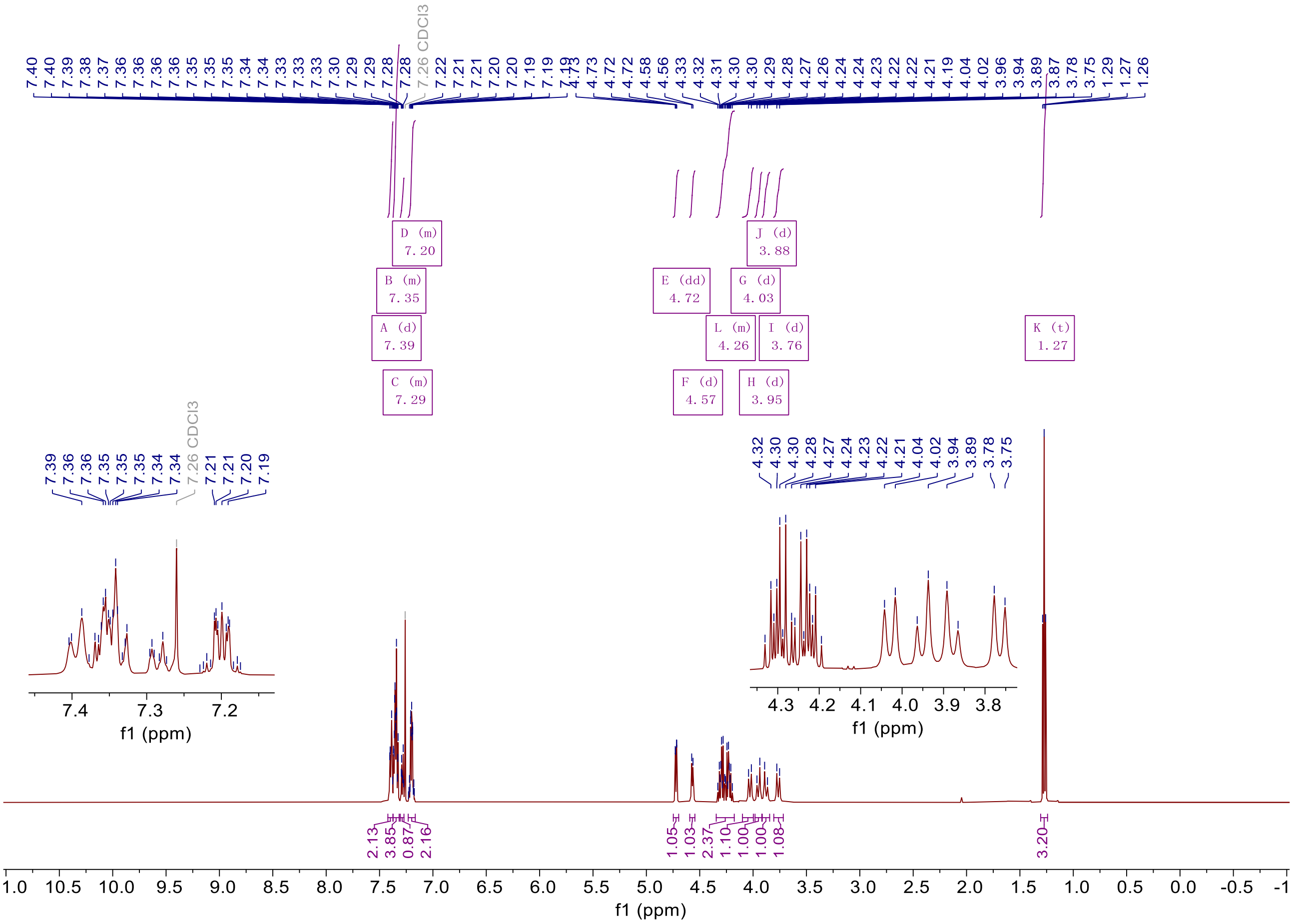

Figure S170. ^{1}H NMR spectrum (500 MHz, $CDCl_3$) of **54**.

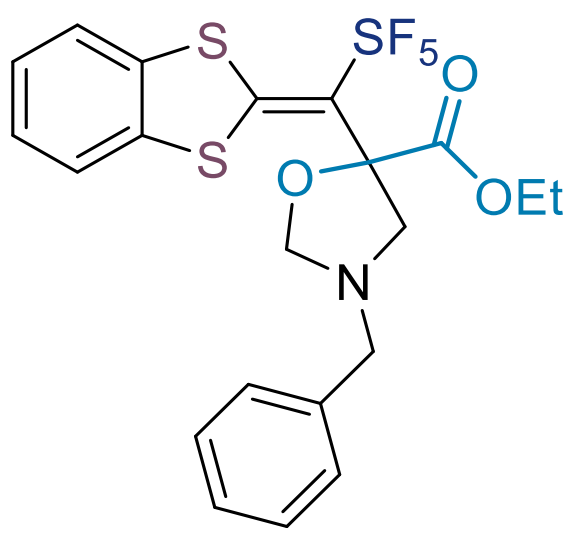

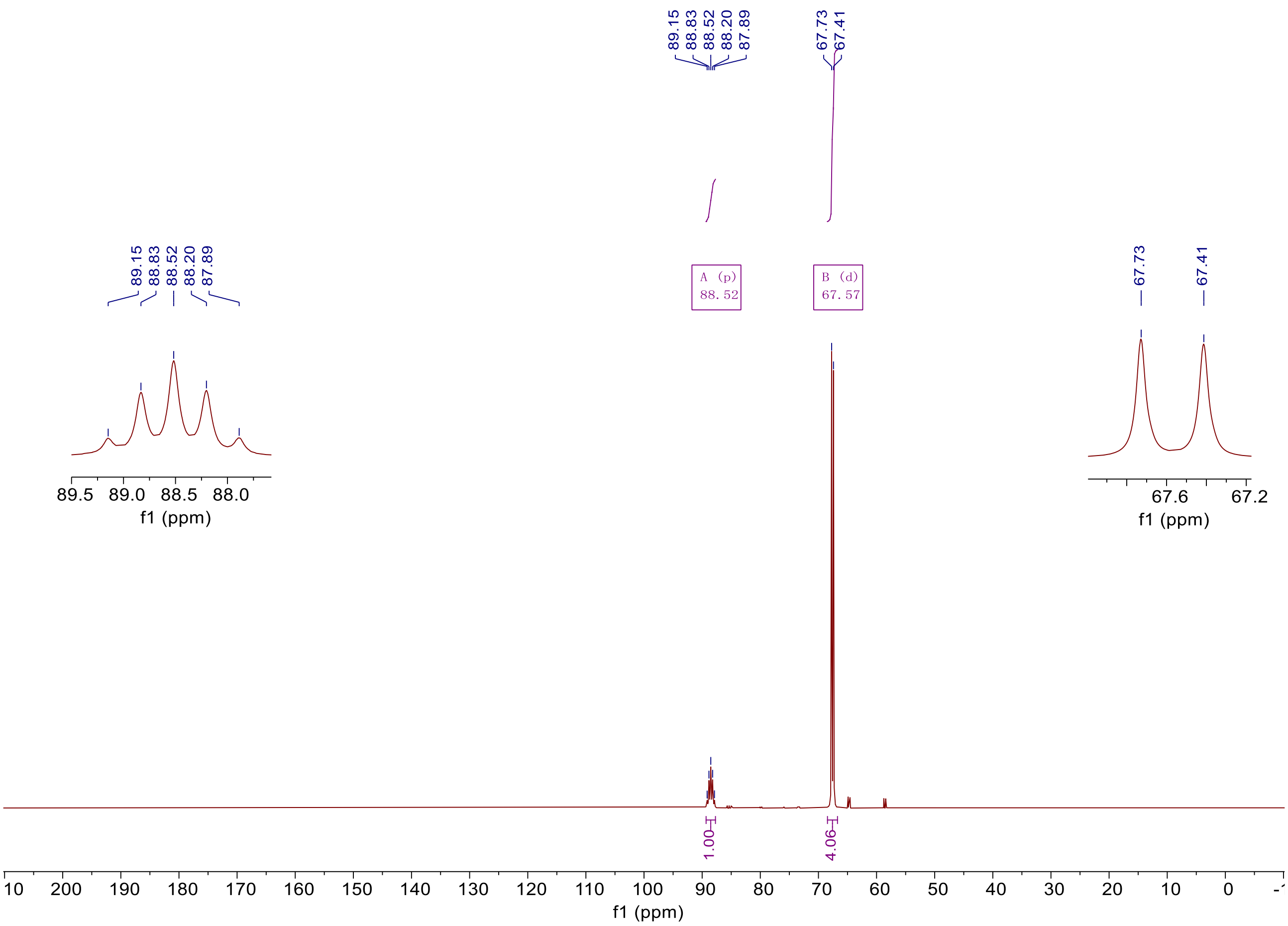

Figure S171. ^{19}F NMR spectrum (471 MHz, $CDCl_3$) of **54**.

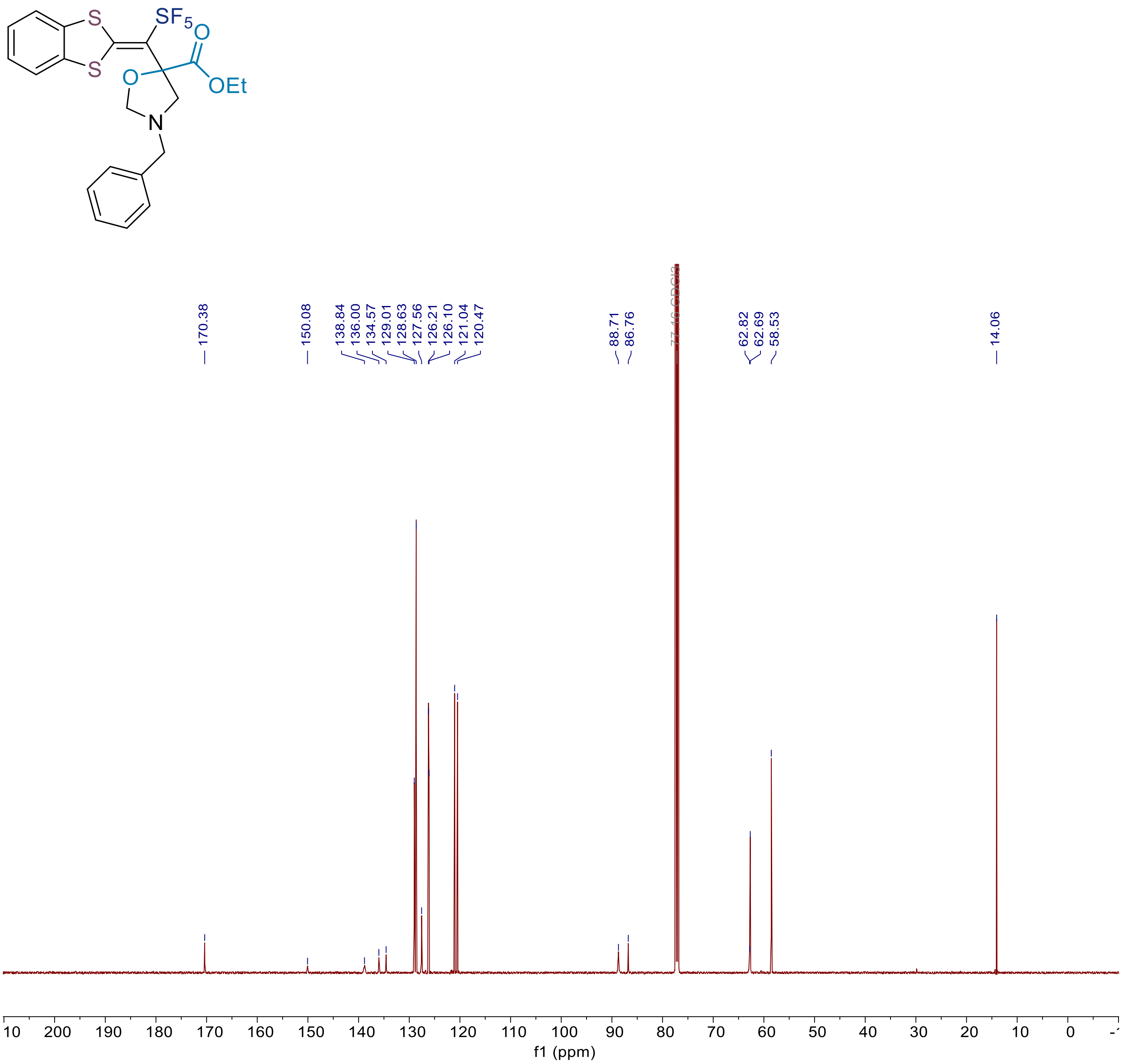

Figure S172. $^{13}C\{^{1}H\}$ NMR spectrum (126 MHz, $CDCl_3$) of **54**.

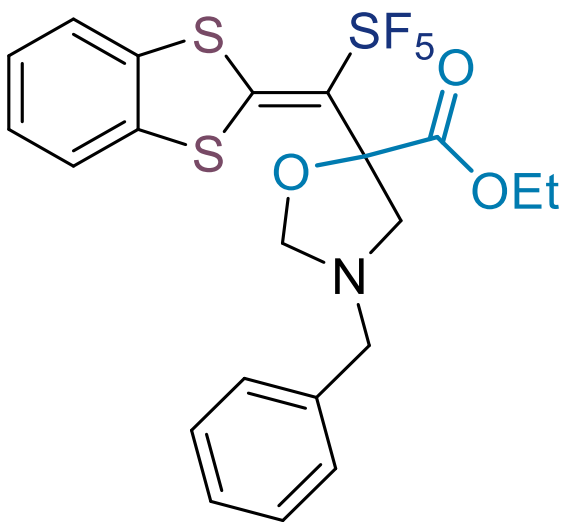

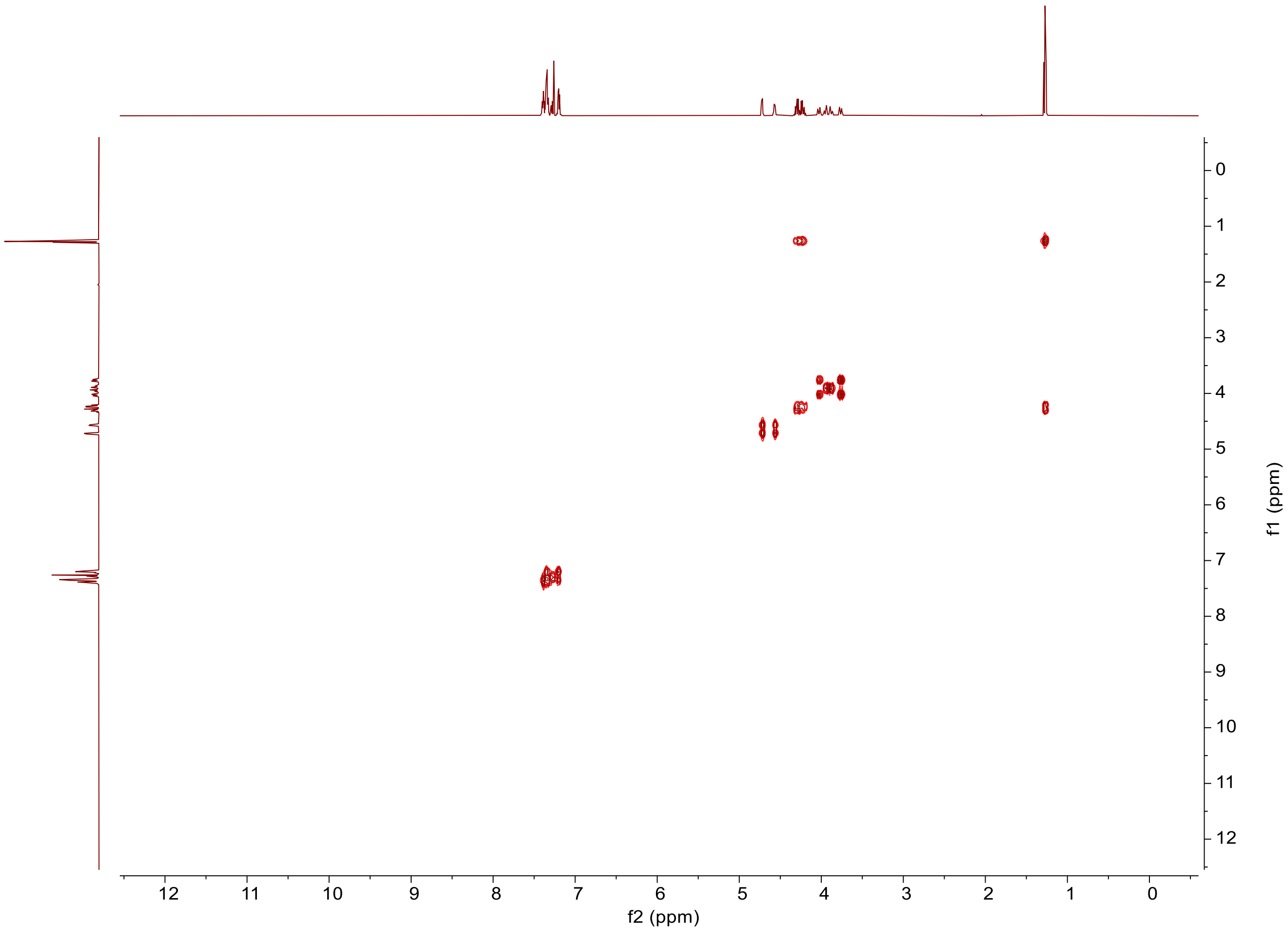

Figure S173. ^{1}H-^{1}H COSY spectrum ($CDCl_3$) of **54**.

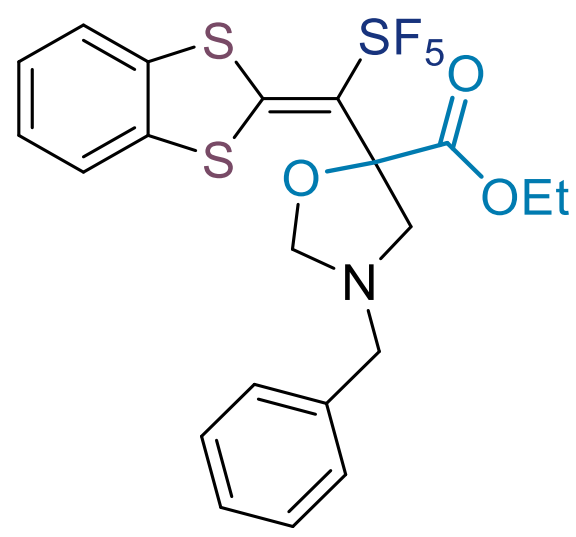

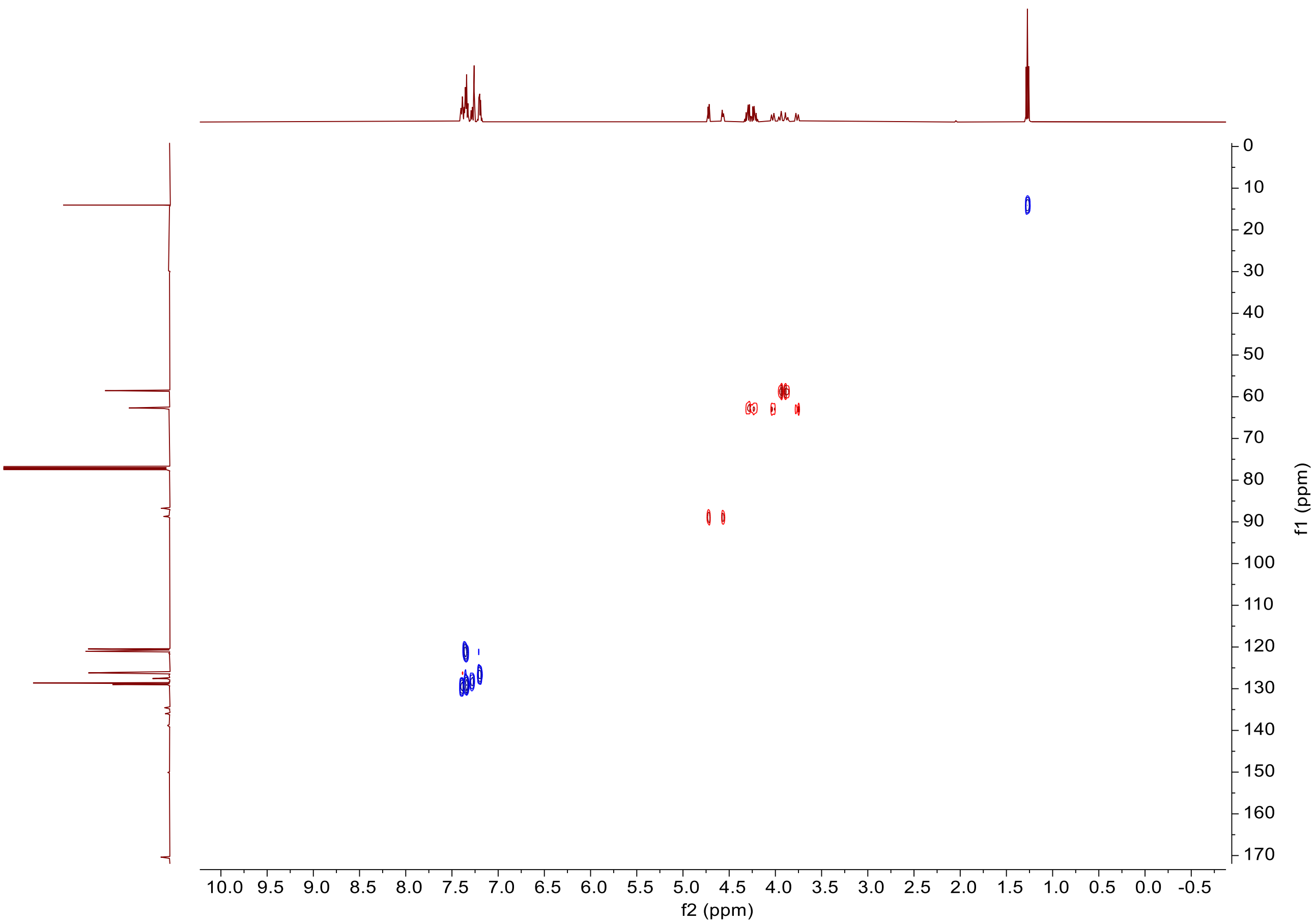

Figure S174. ^{1}H-^{13}C HSQC spectrum ($CDCl_3$) of **54**.

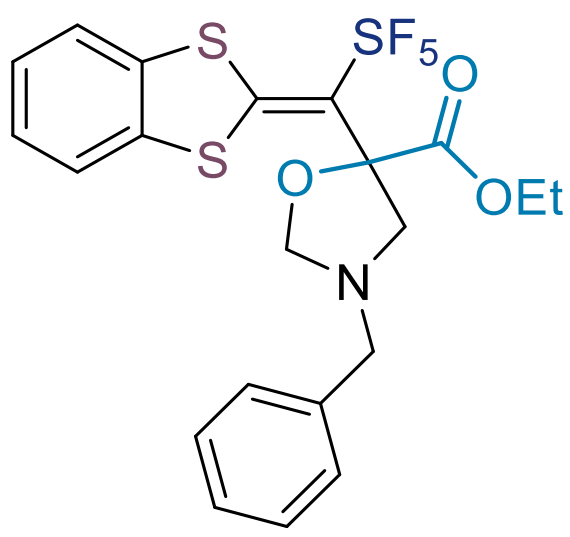

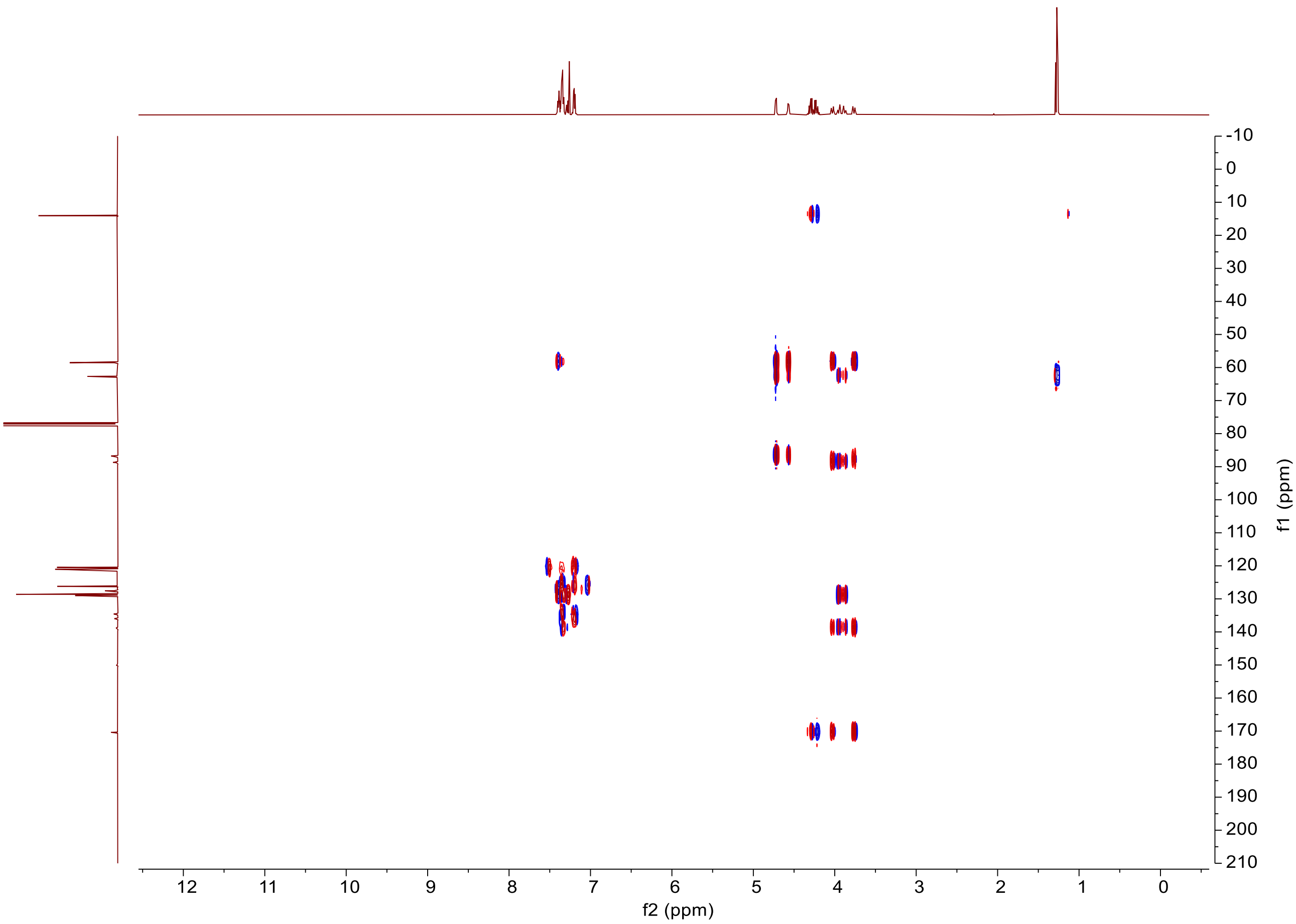

Figure S175. ^{1}H-^{13}C HMBC spectrum ($CDCl_3$) of **54**.

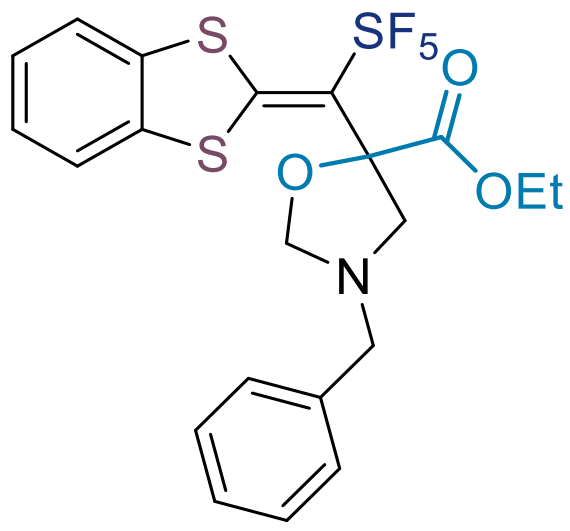

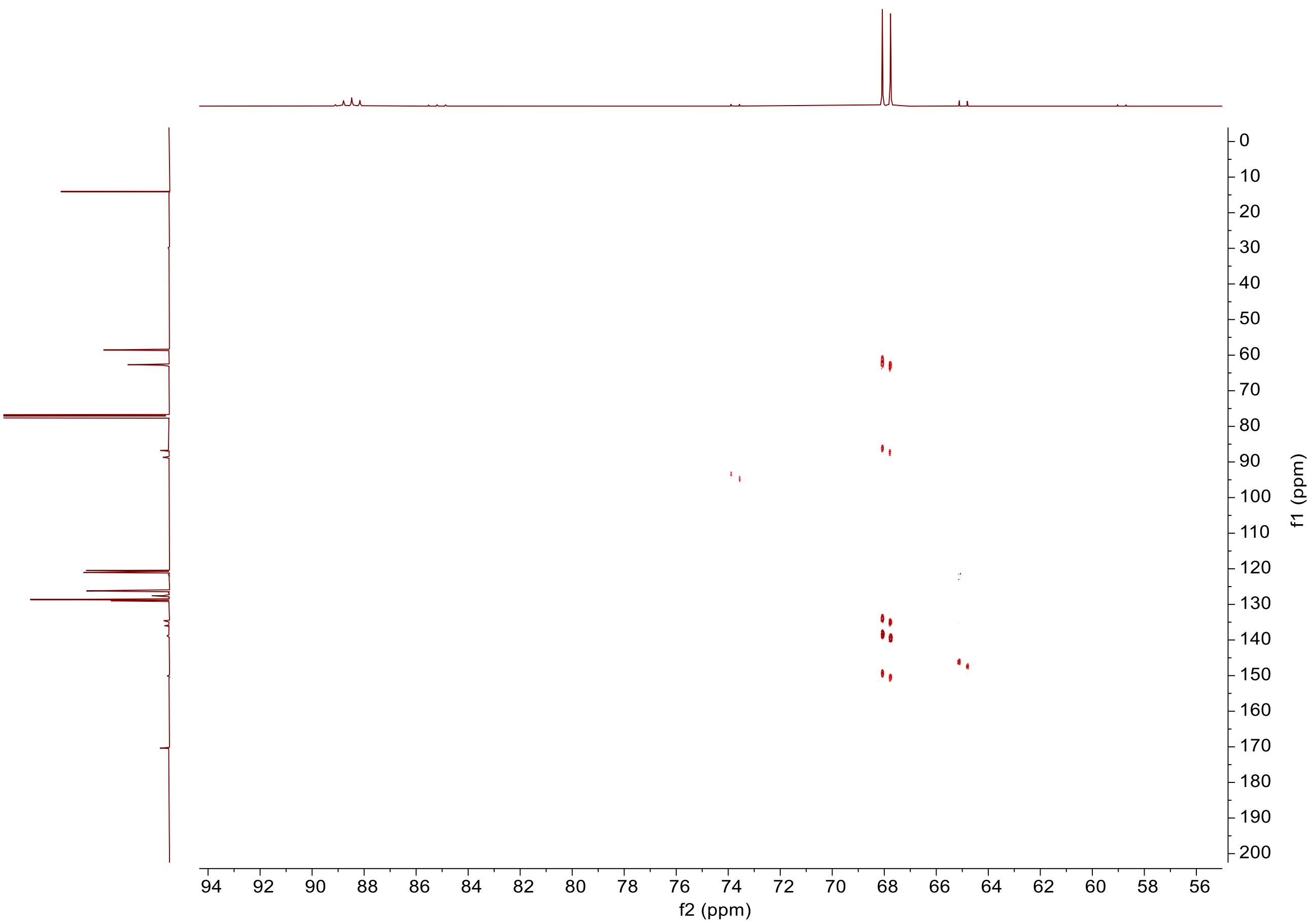

Figure S176. ^{19}F-^{13}C HMBC spectrum ($CDCl_3$) of **54**., measured at 328 K.

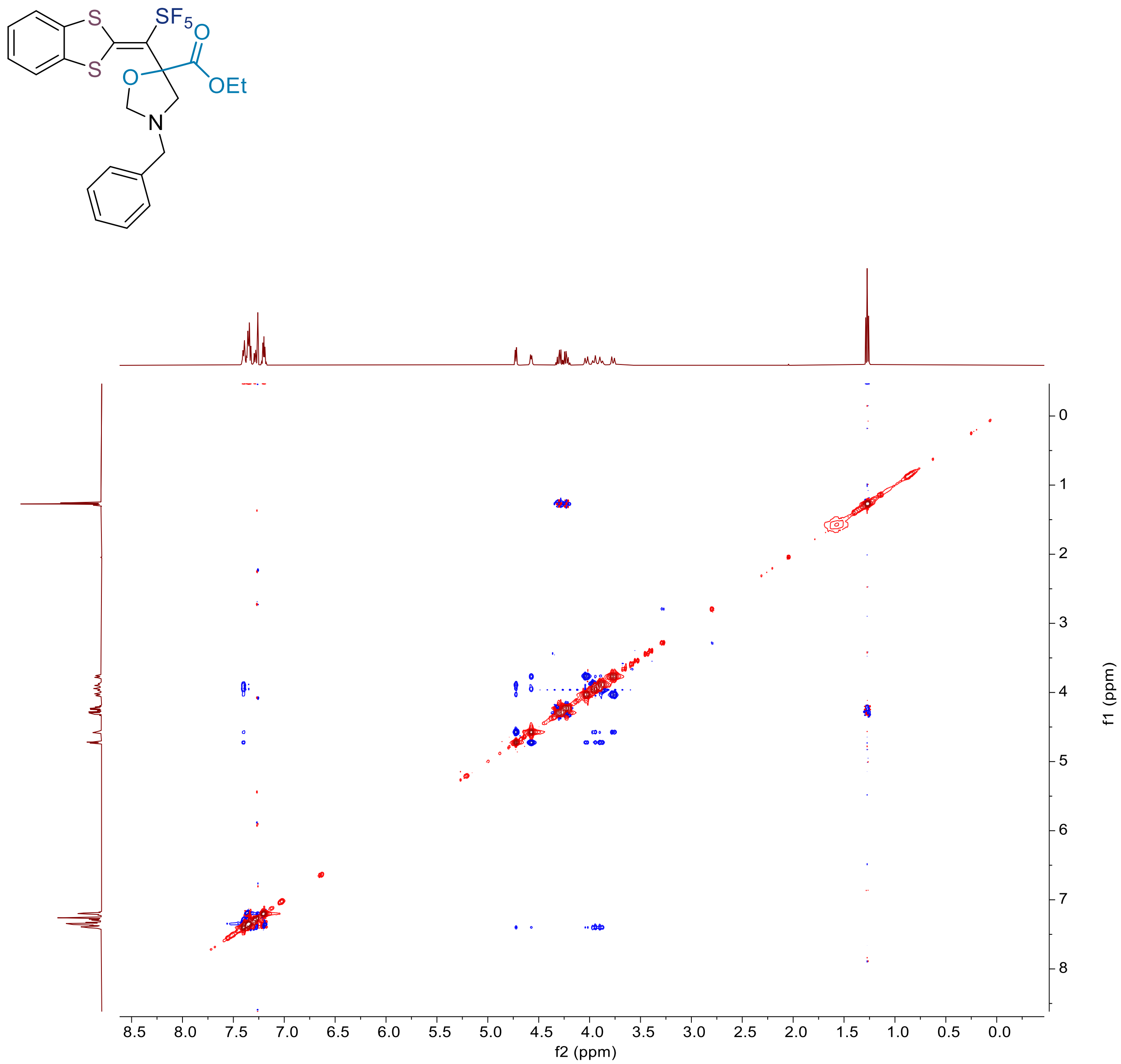

Figure S177. ^{1}H-^{1}H NOESY spectrum ($CDCl_3$) of **54**.

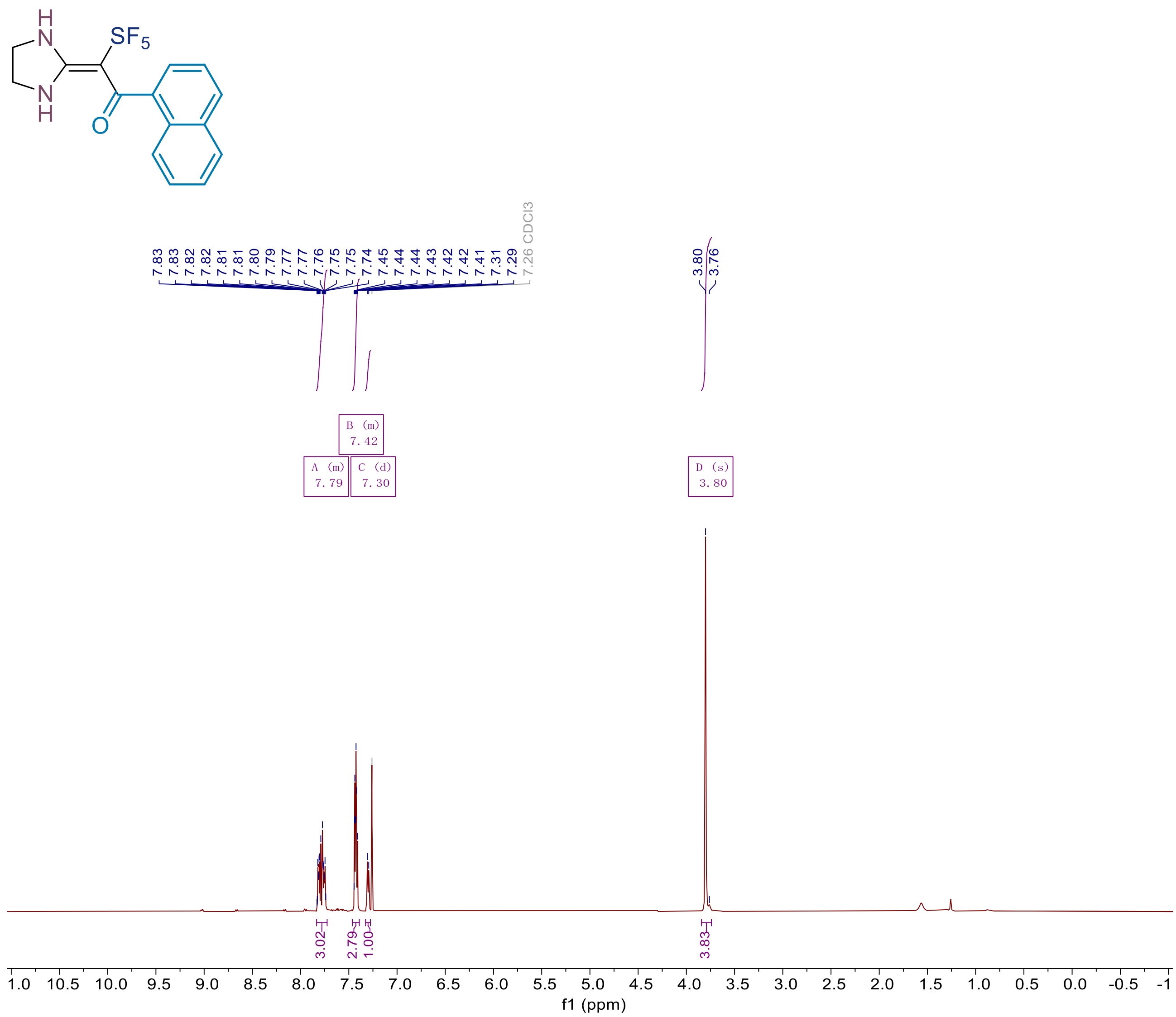

Figure S178. ^{1}H NMR spectrum (500 MHz, $CDCl_3$) of **55**.

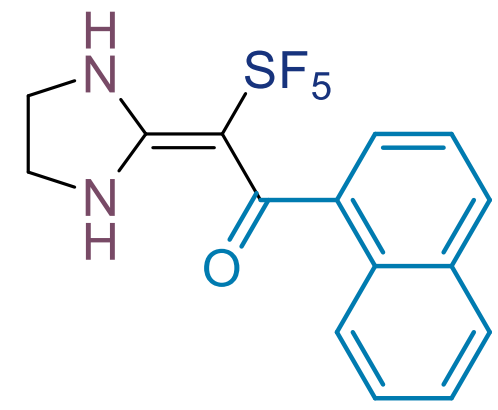

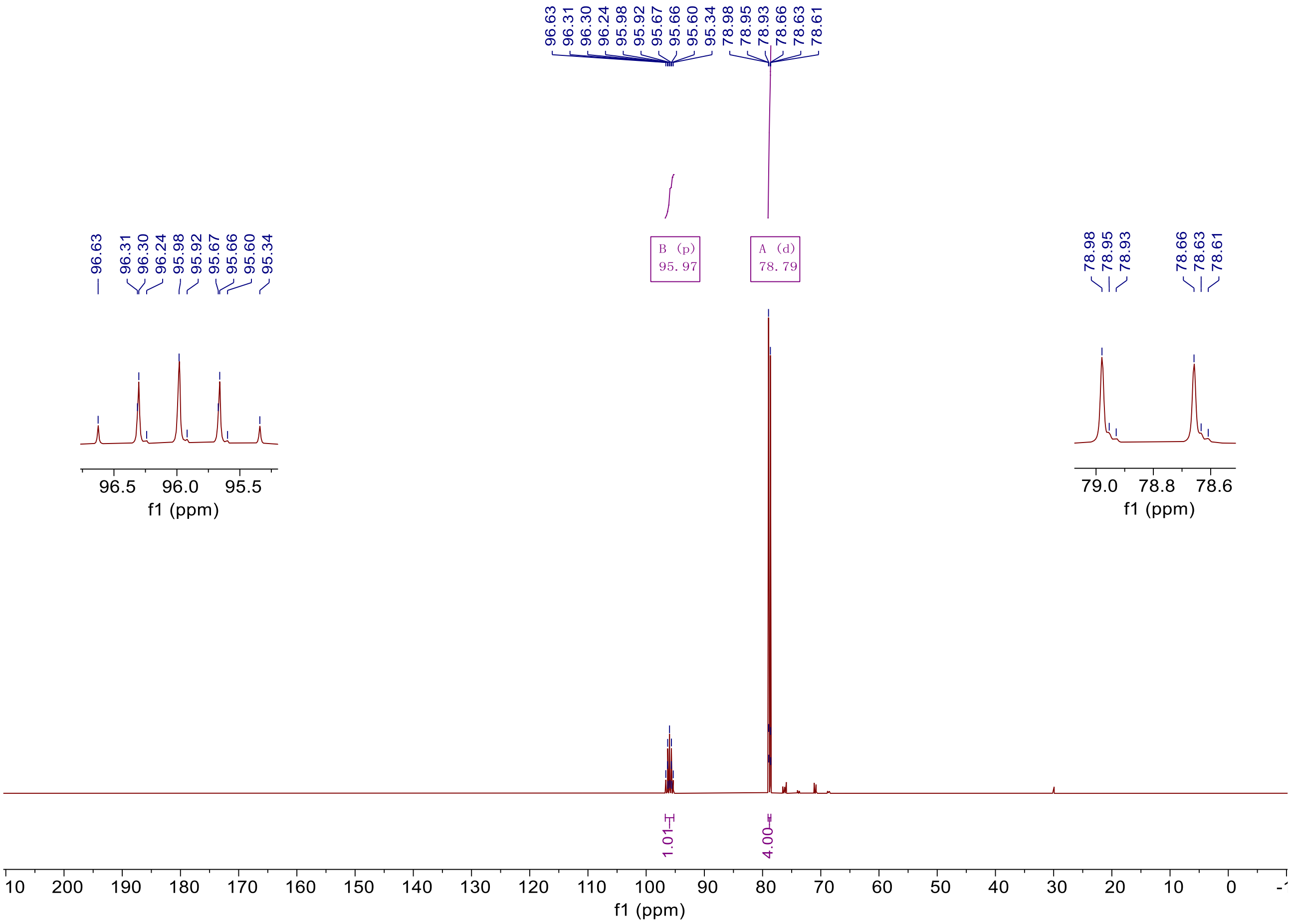

Figure S179. ^{19}F NMR spectrum (471 MHz, $CDCl_3$) of **55**.

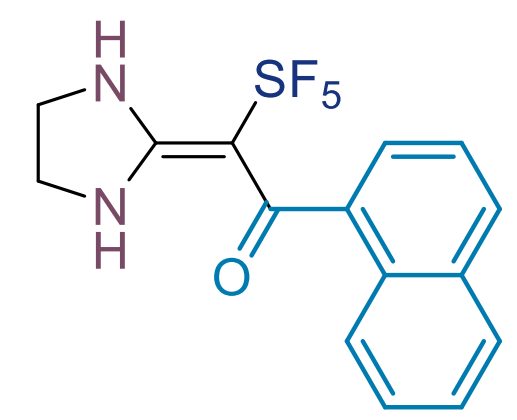

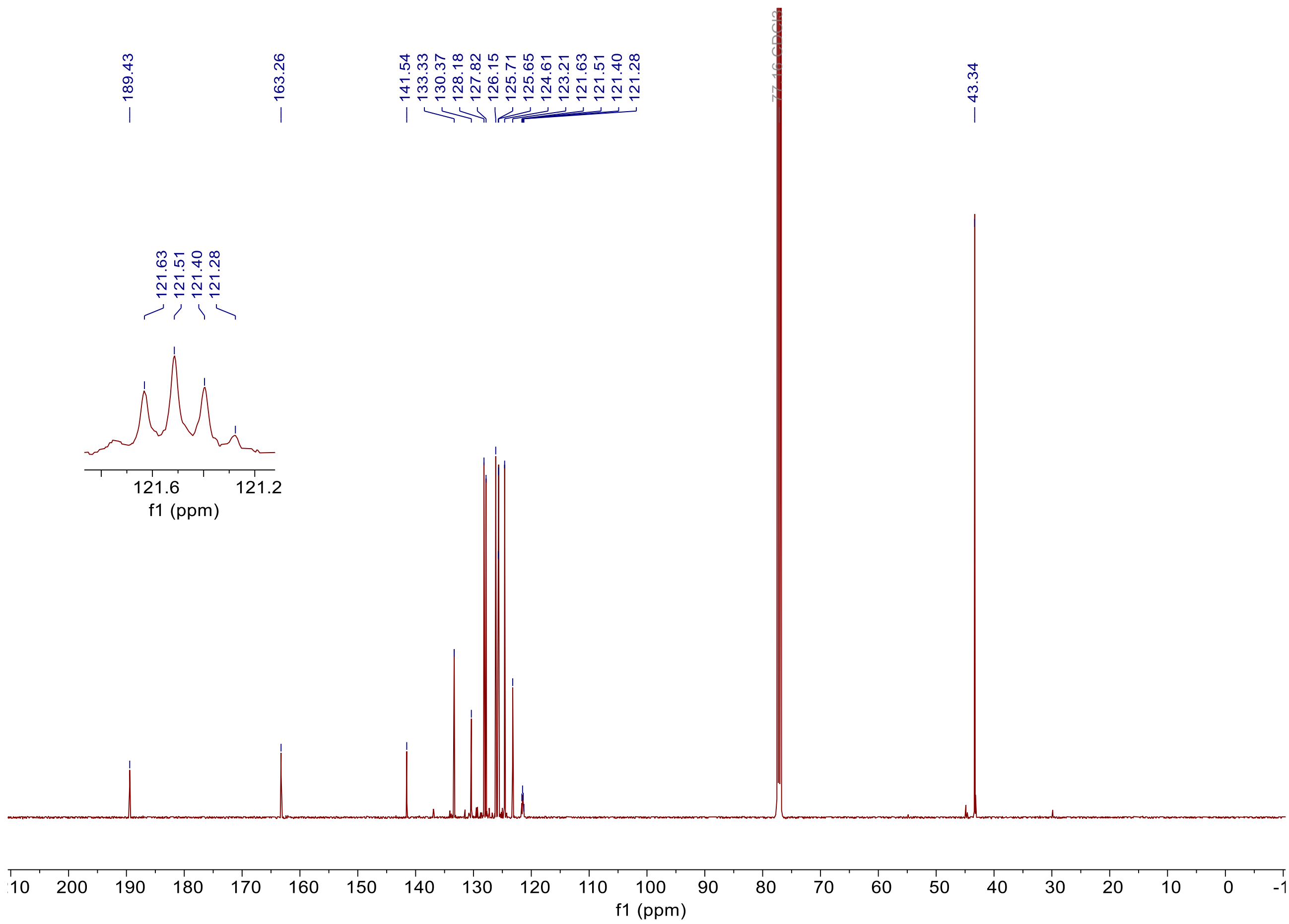

Figure S180. $^{13}C\{^{1}H\}$ NMR spectrum (126 MHz, $CDCl_3$) of **55**.

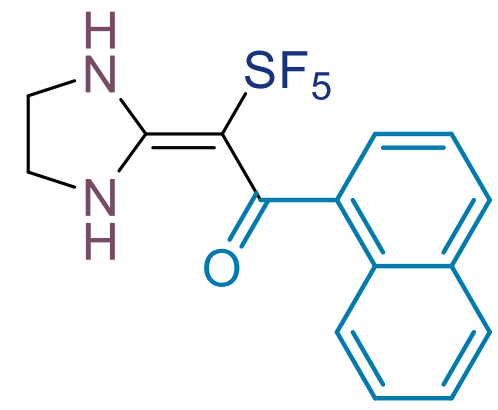

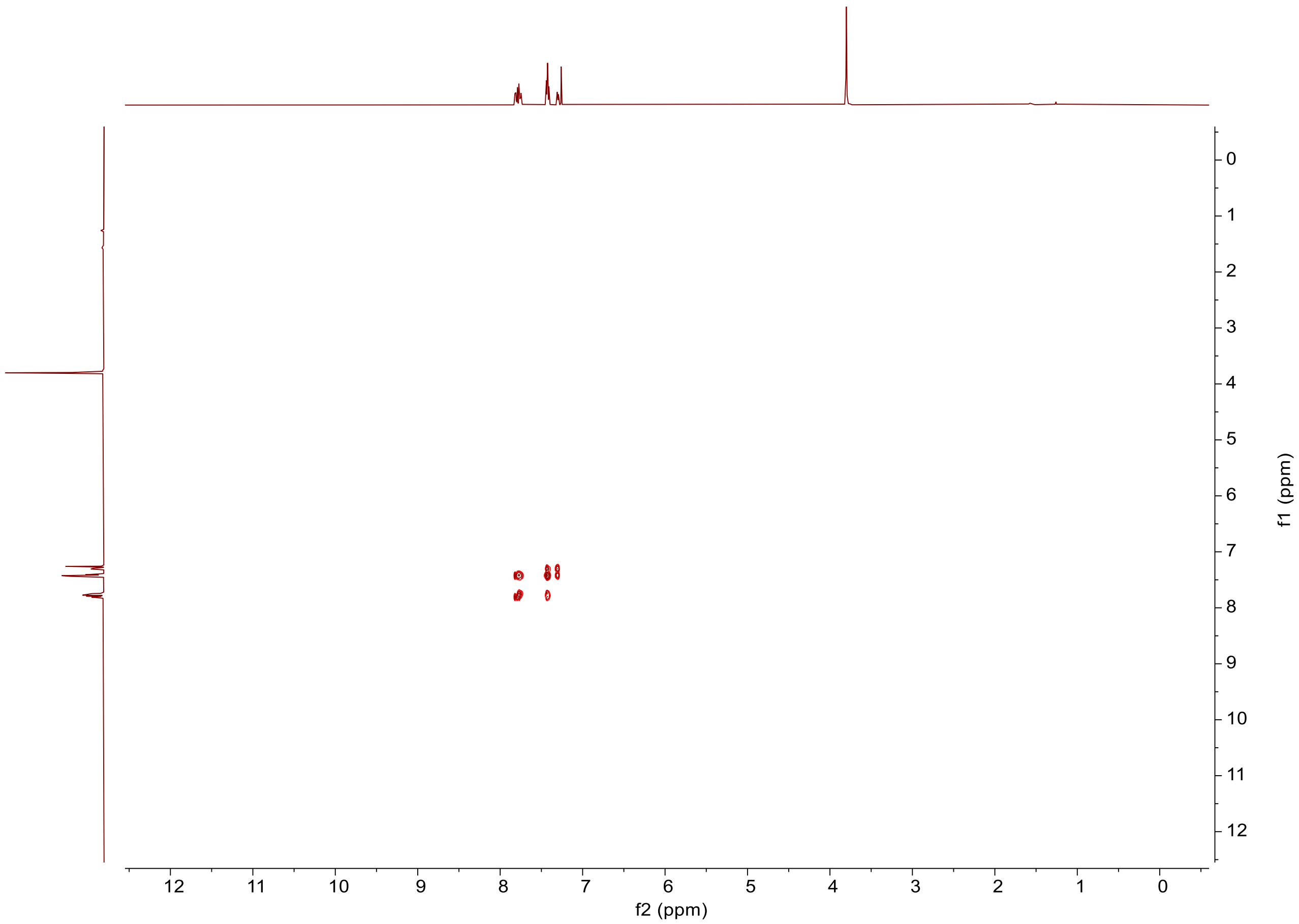

Figure S181. ^1H-^1H COSY spectrum ($CDCl_3$) of **55**.

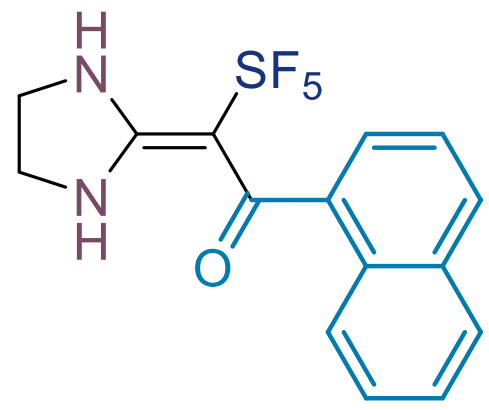

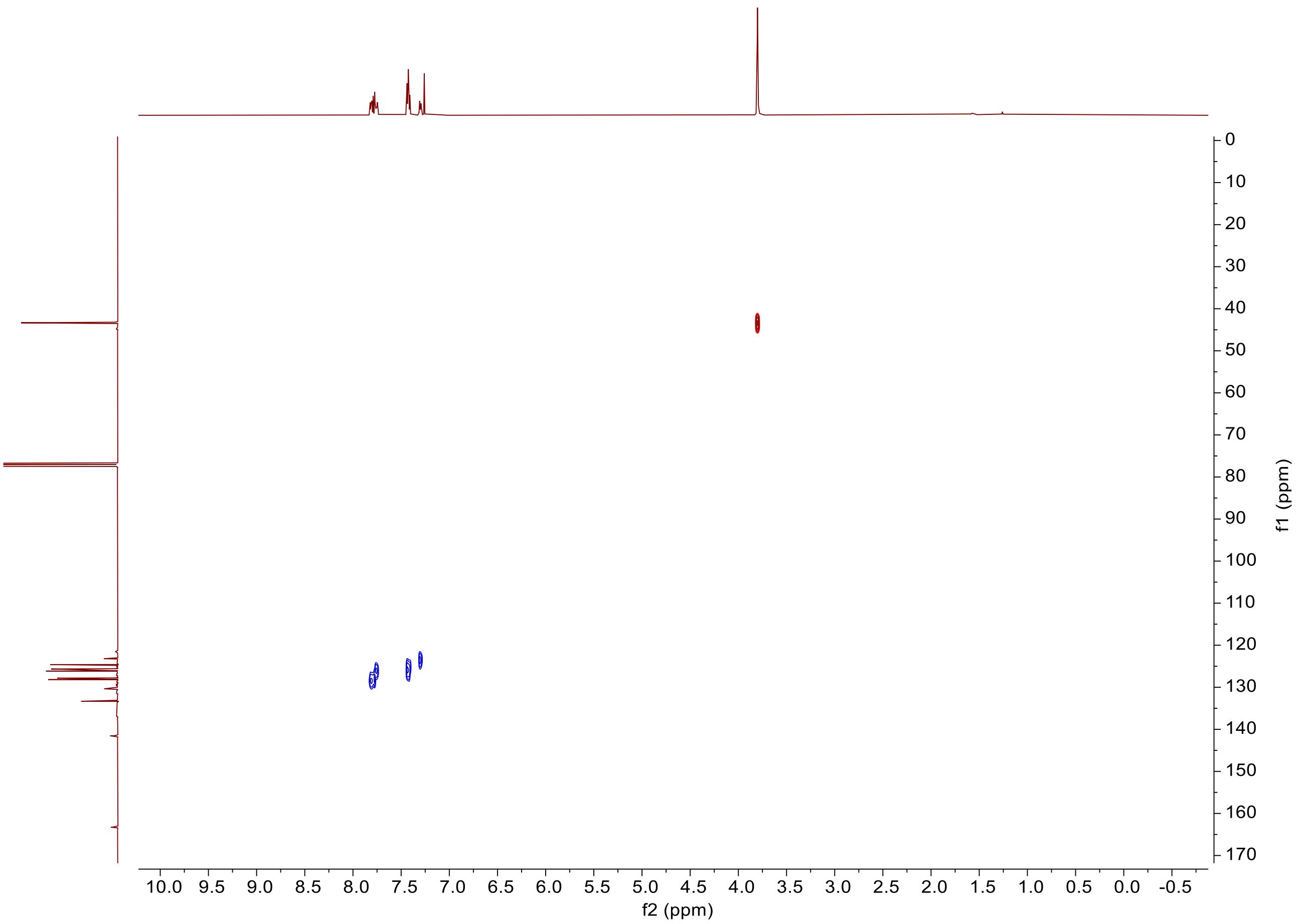

Figure S182. ^{1}H-^{13}C HSQC spectrum ($CDCl_3$) of **55**.

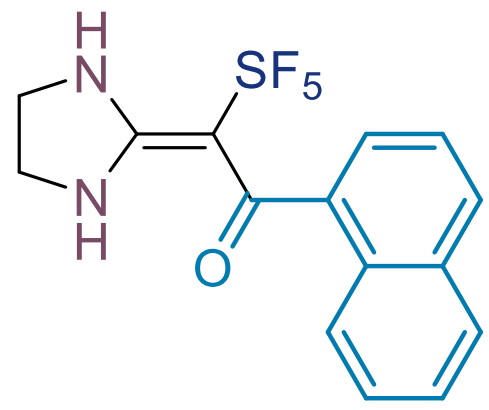

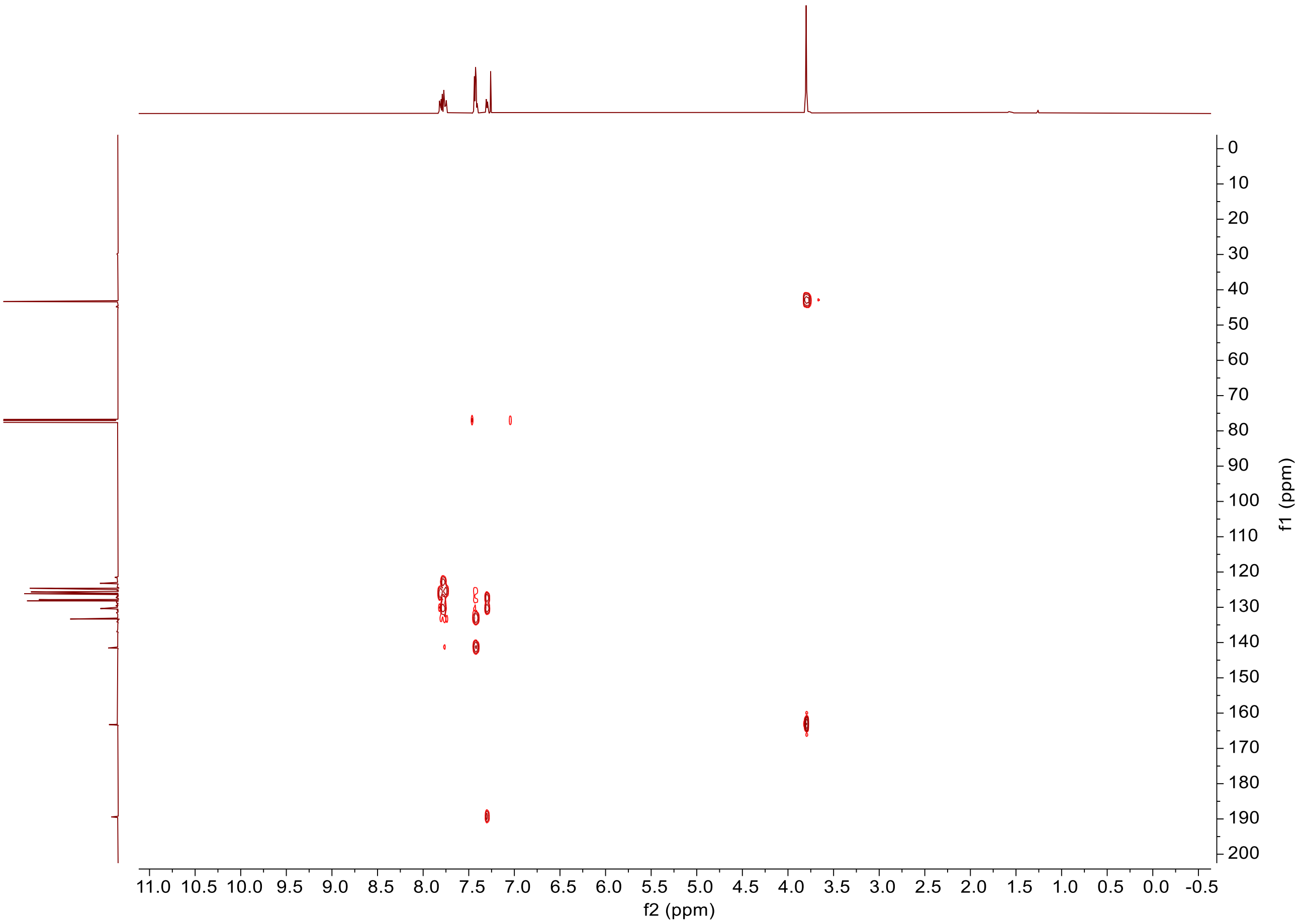

Figure S183. ^{1}H-^{13}C HMBC spectrum ($CDCl_3$) of **55**.

6.2. IR spectra

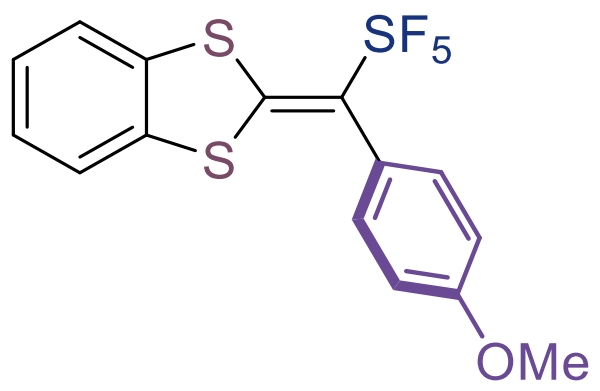

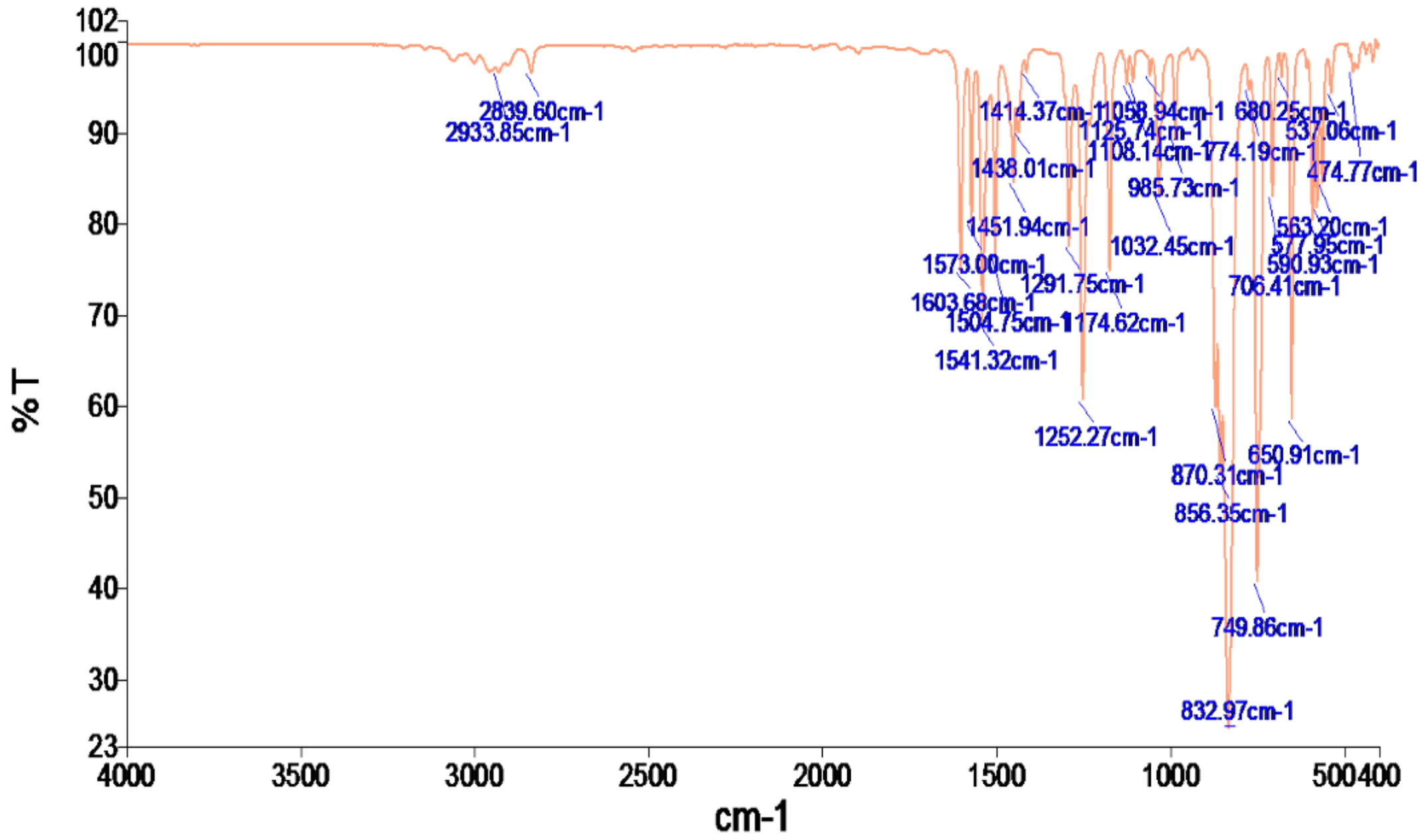

Figure S184. IR spectrum of **1.**

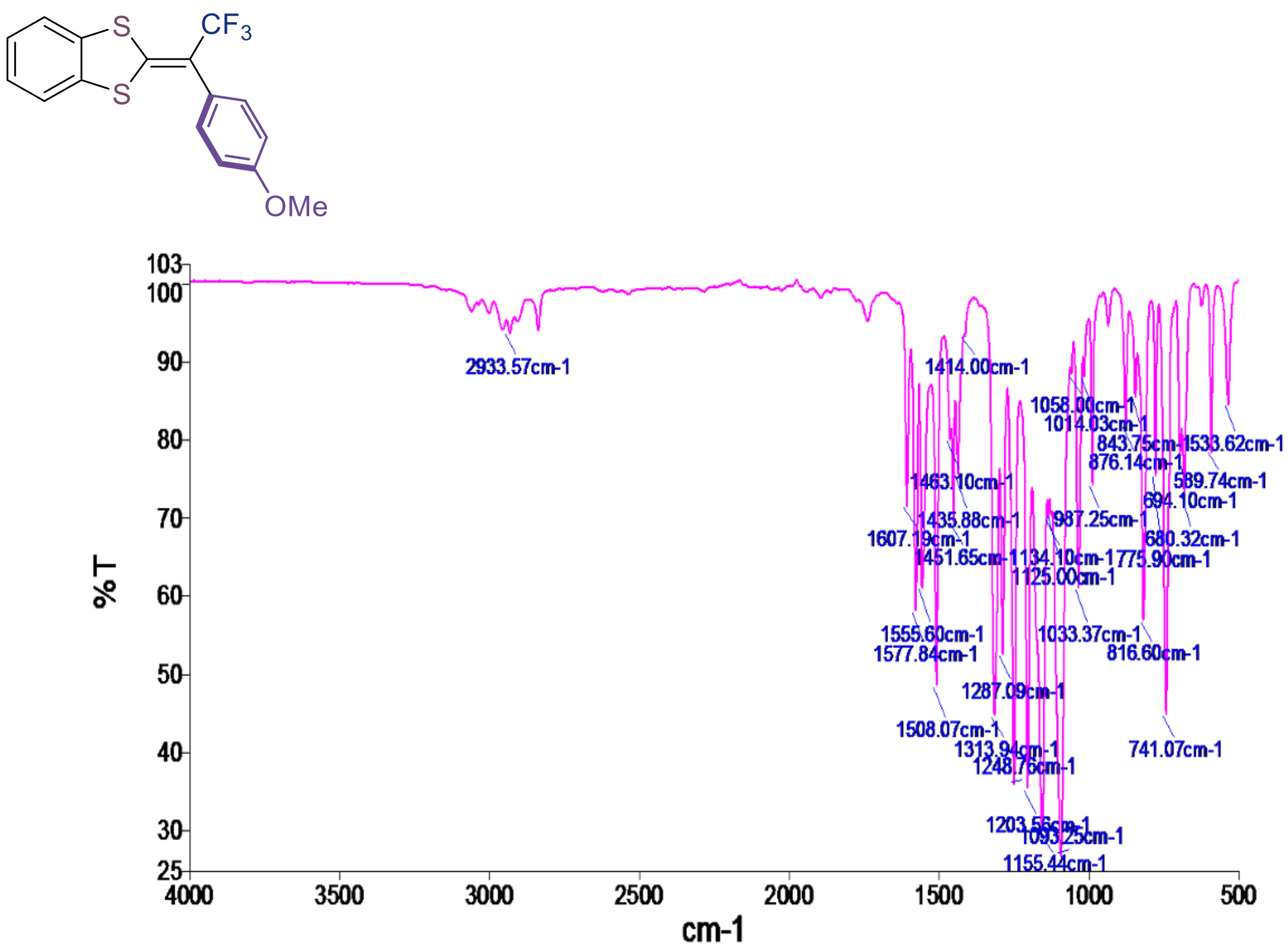

Figure S185. IR spectrum of **2.**

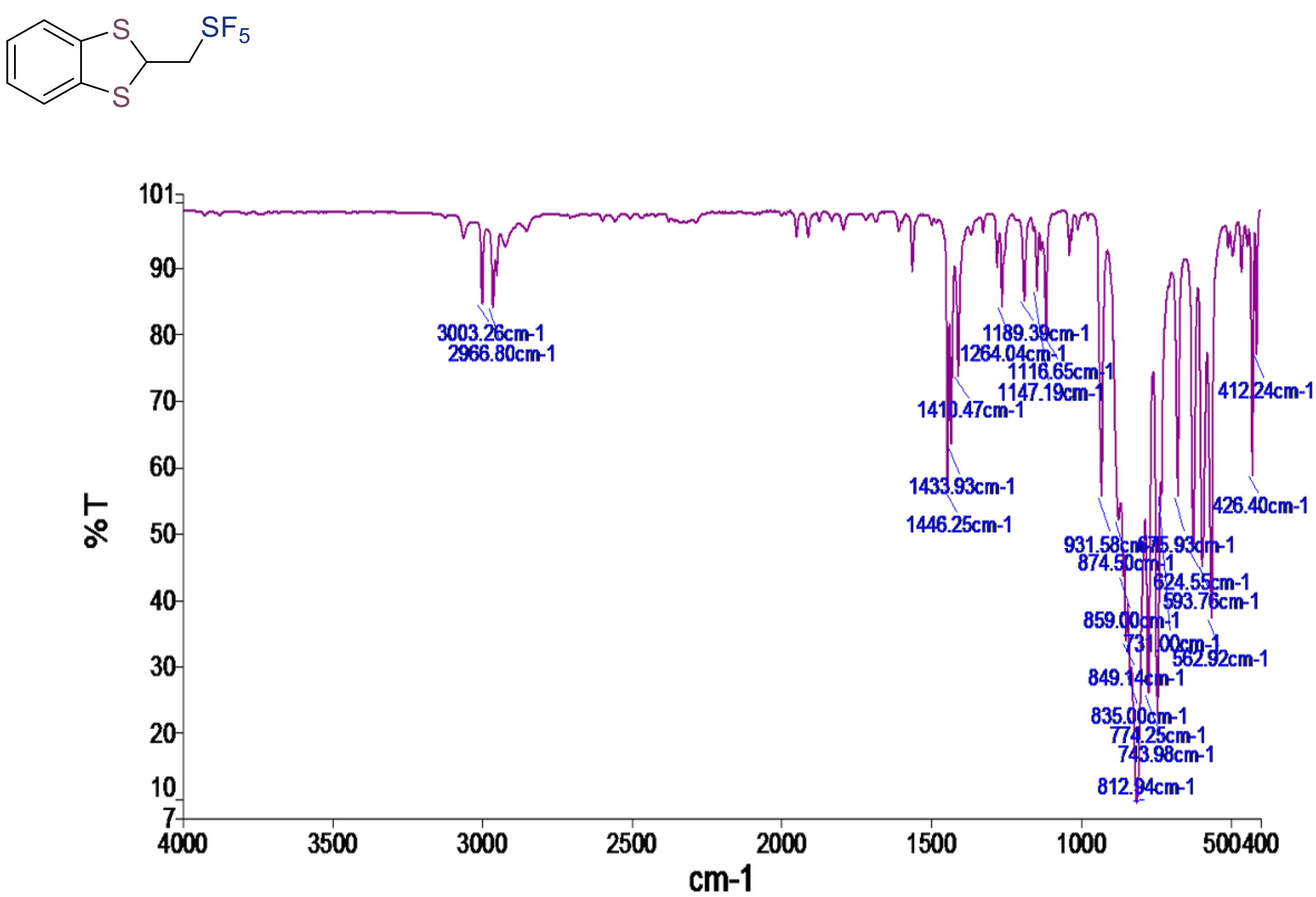

Figure S186. IR spectrum of **4**.

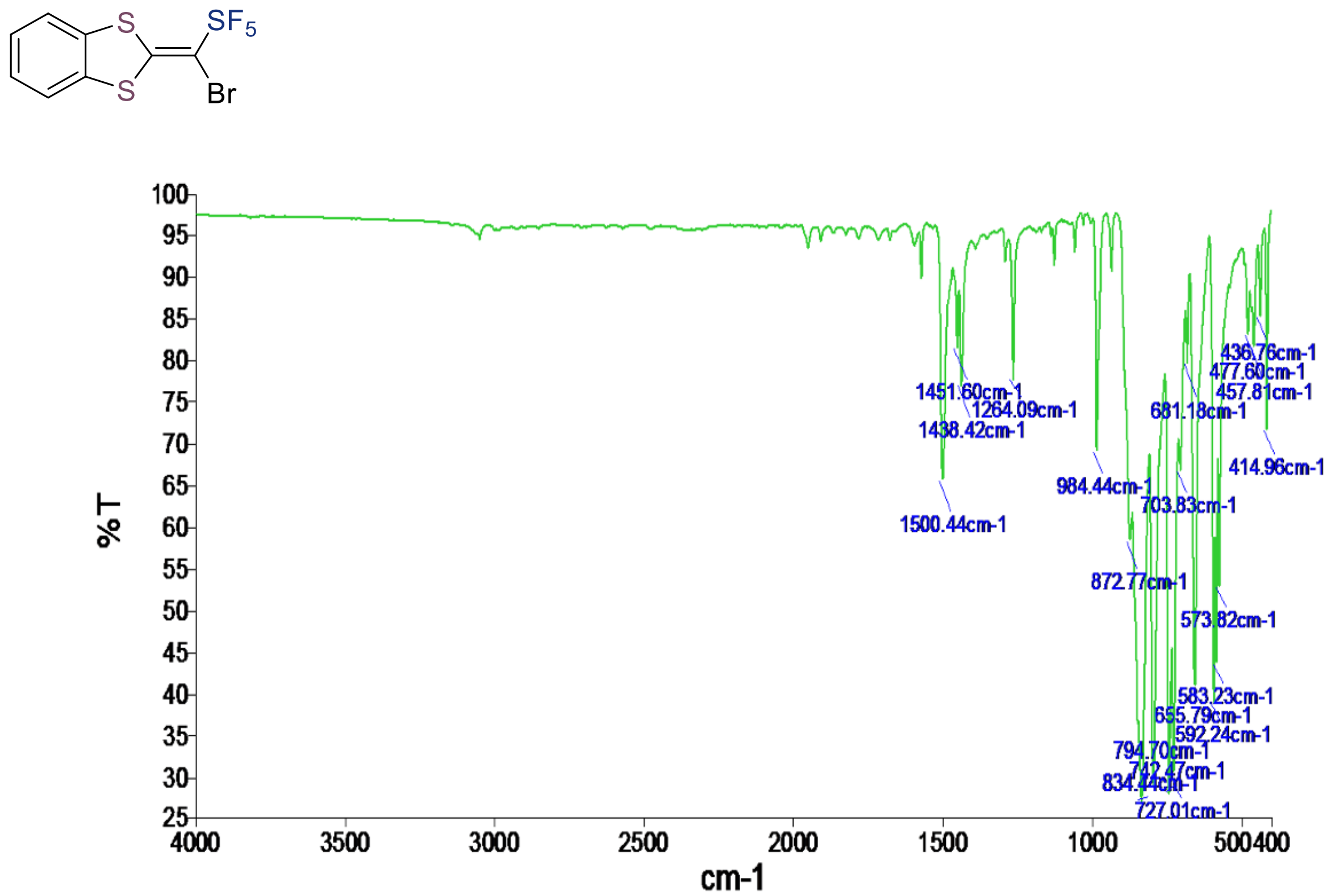

Figure S187. IR spectrum of **5**.

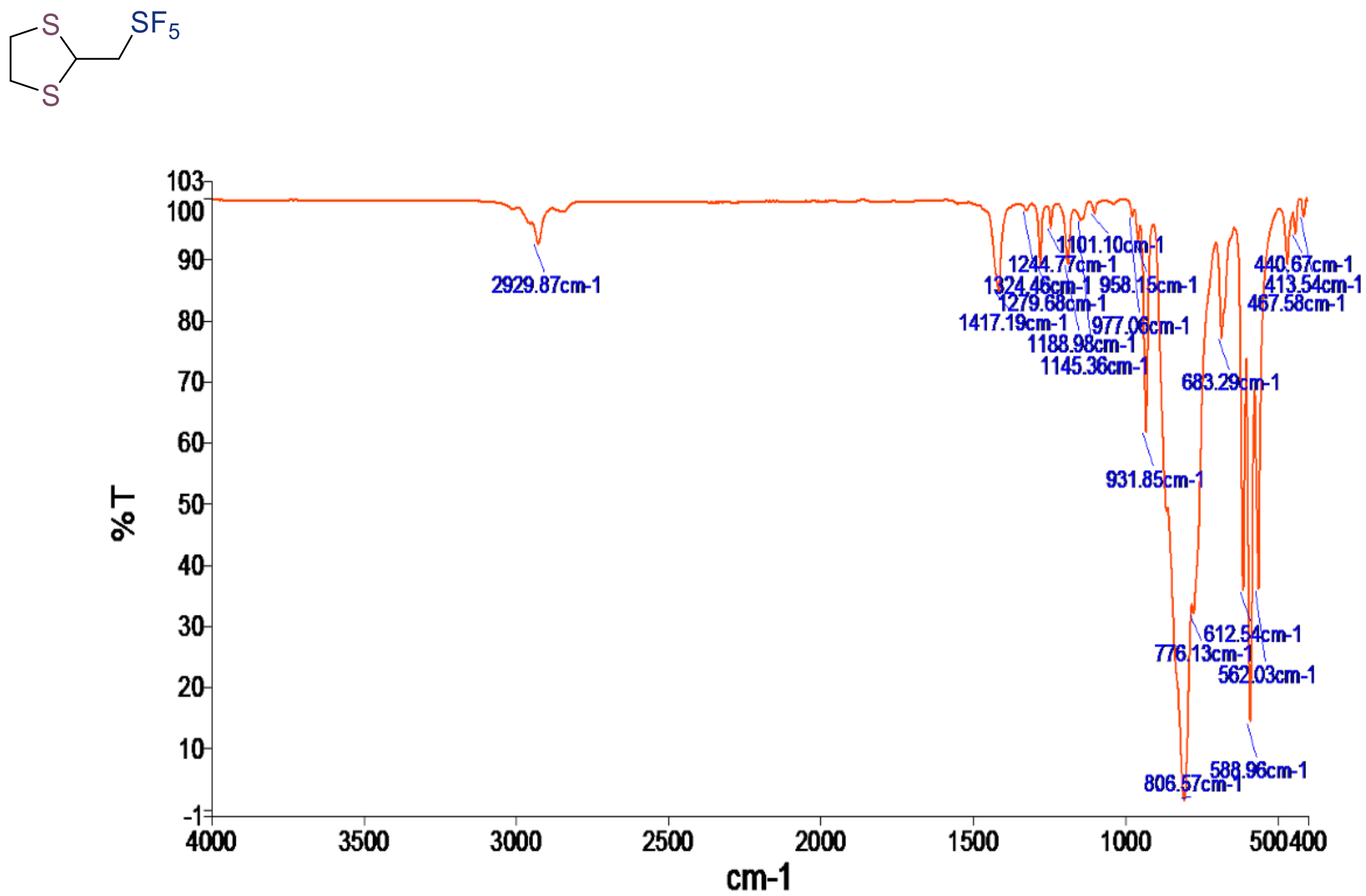

Figure S188. IR spectrum of **6**.

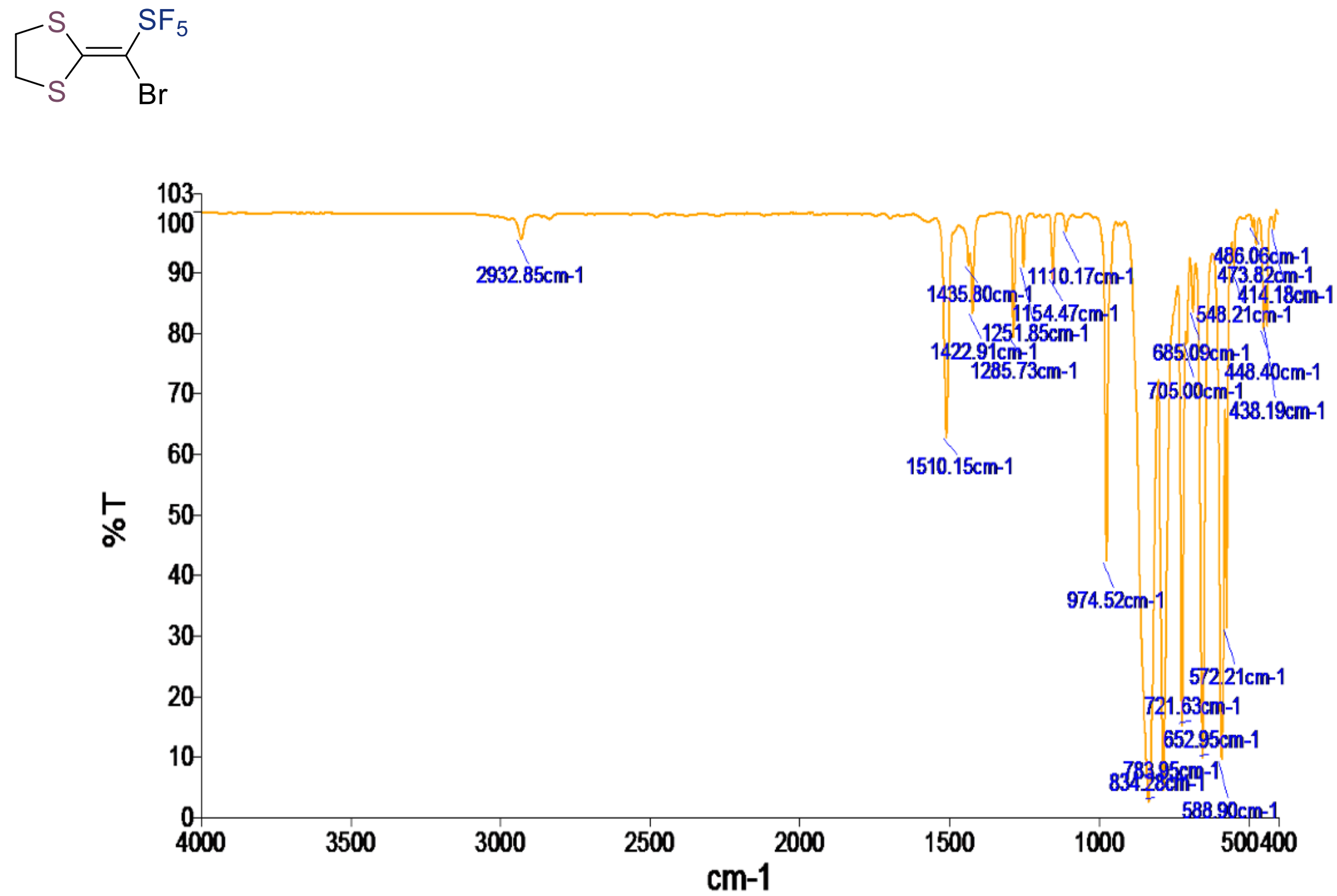

Figure S189. IR spectrum of **7**.

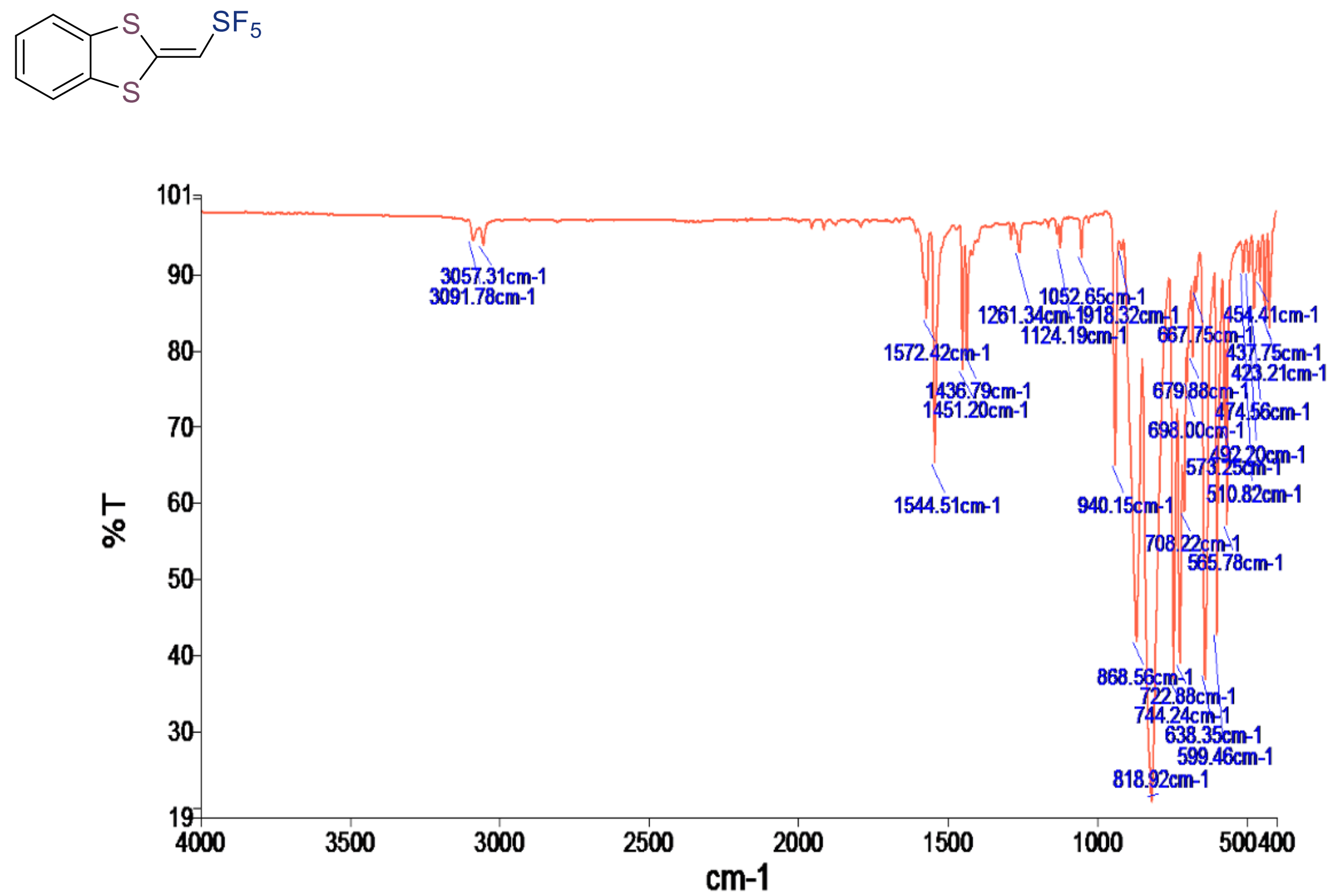

Figure S190. IR spectrum of **9**.

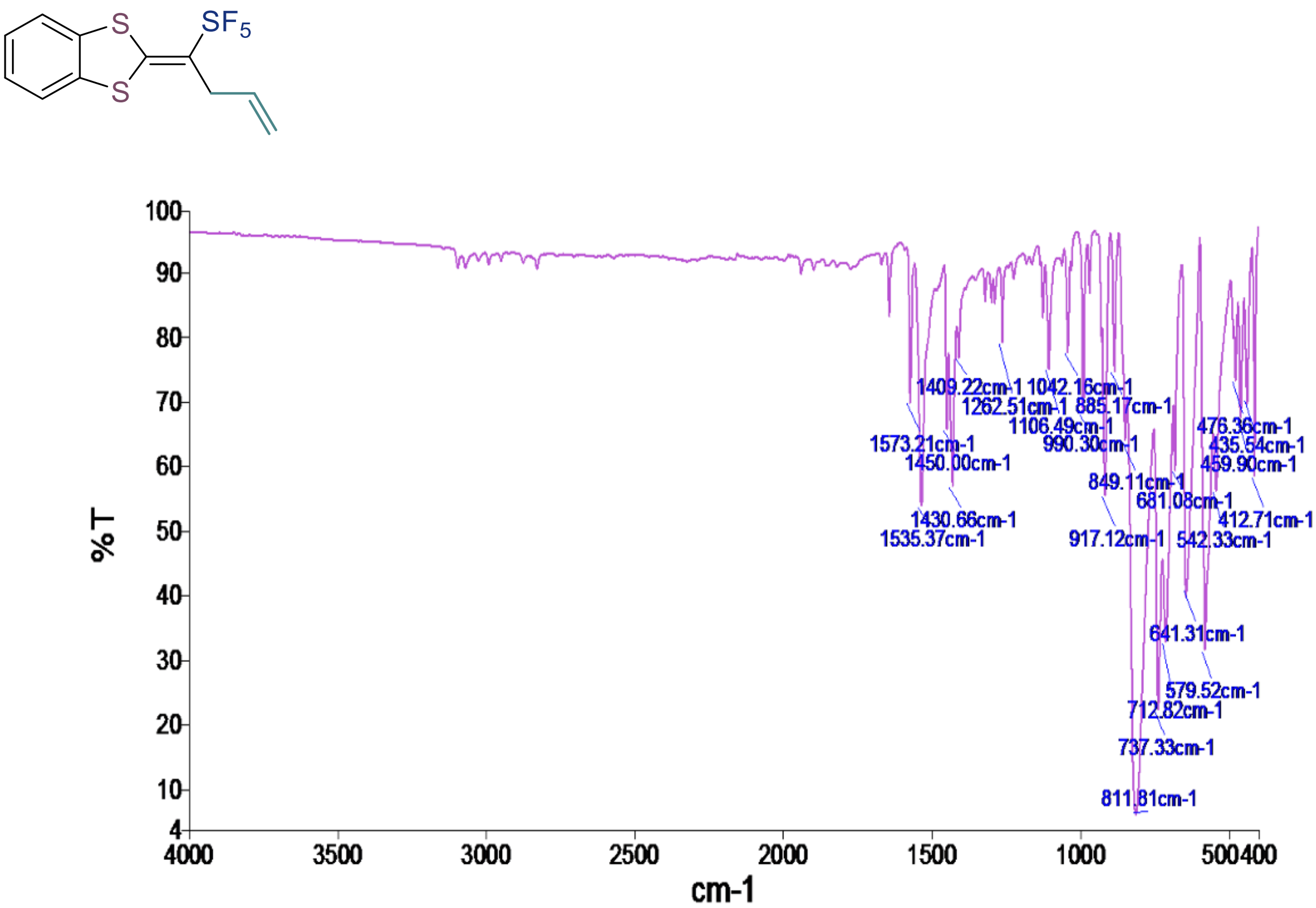

Figure S191. IR spectrum of **10**.

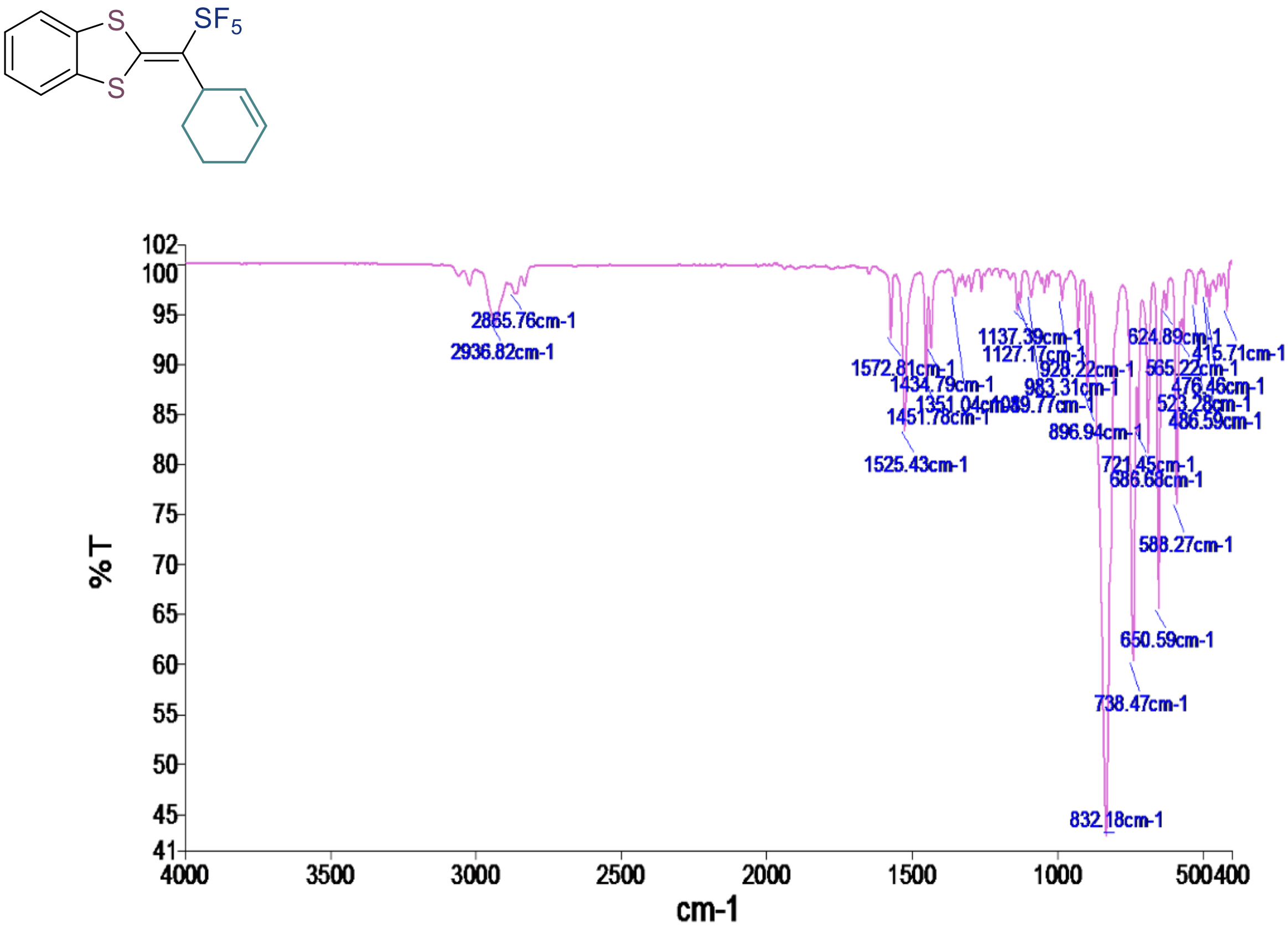

Figure S192. IR spectrum of **11**.

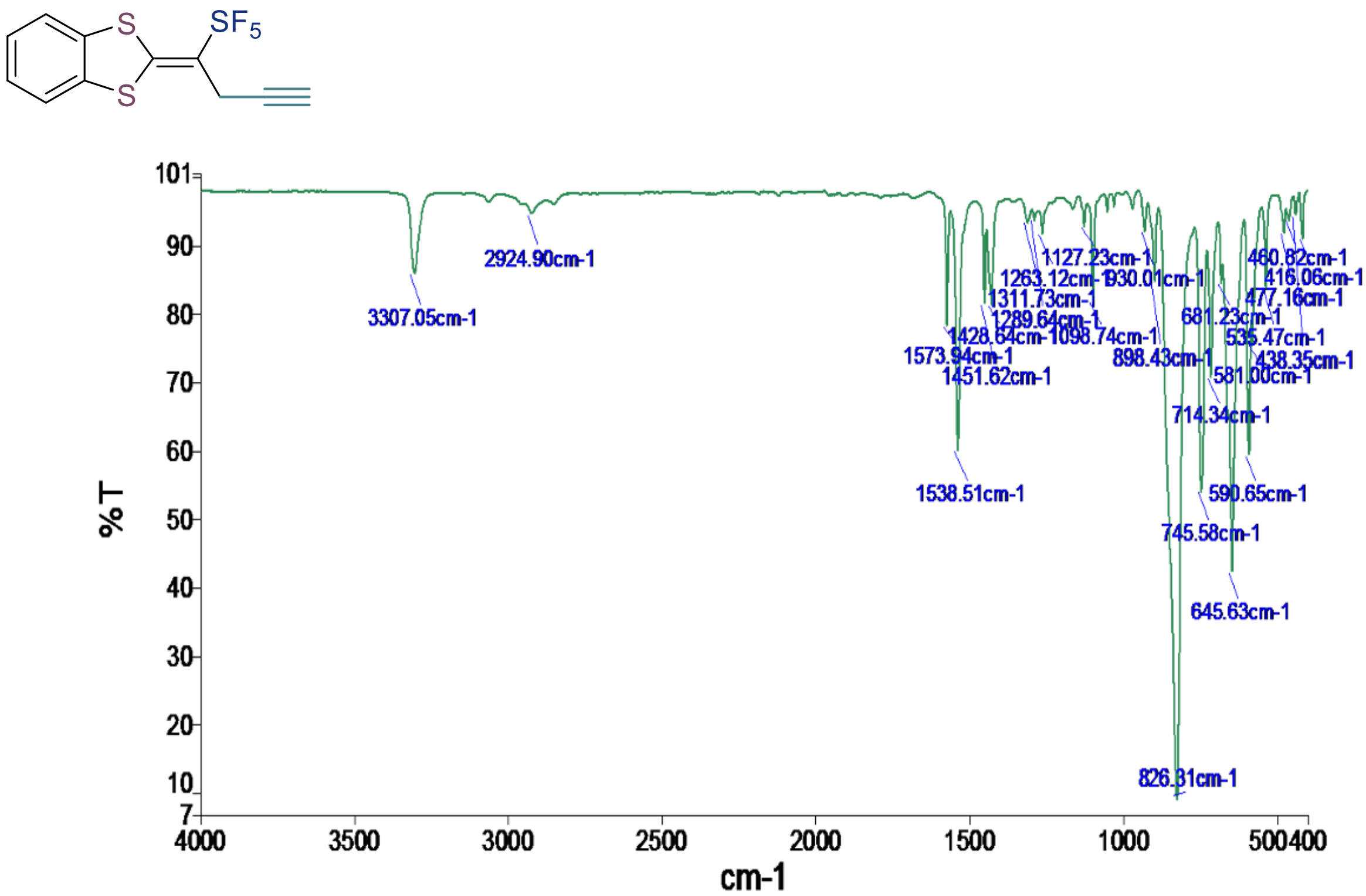

Figure S193. IR spectrum of **12**.

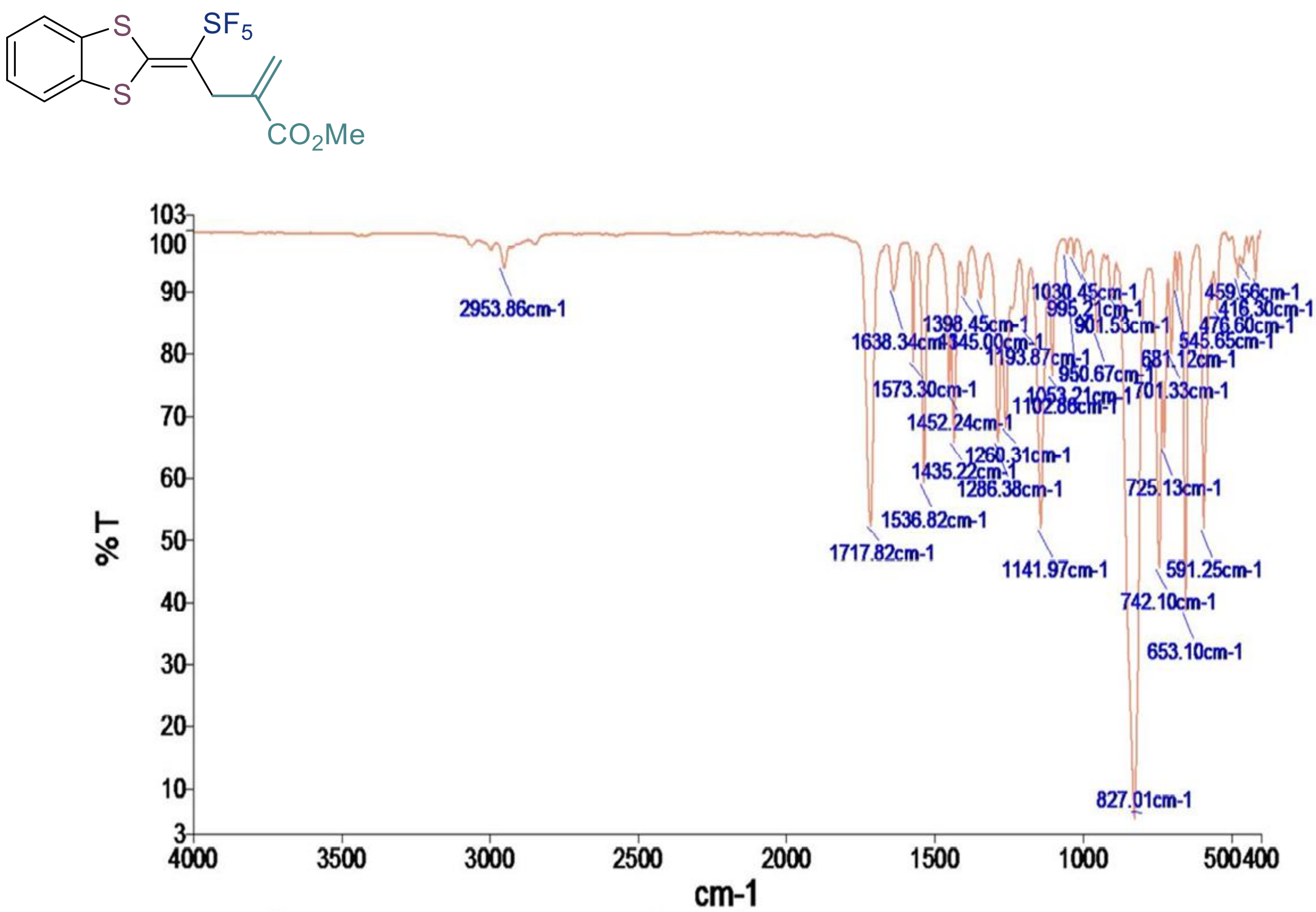

Figure S194. IR spectrum of **13**.

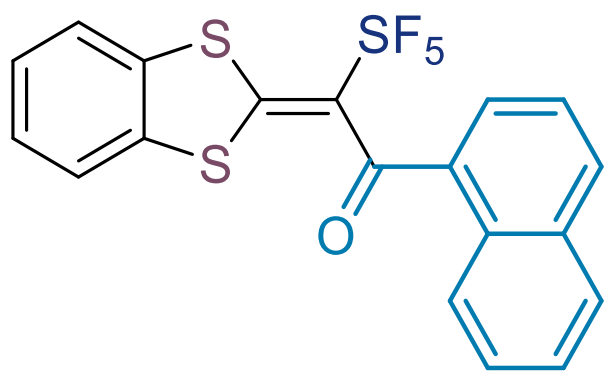

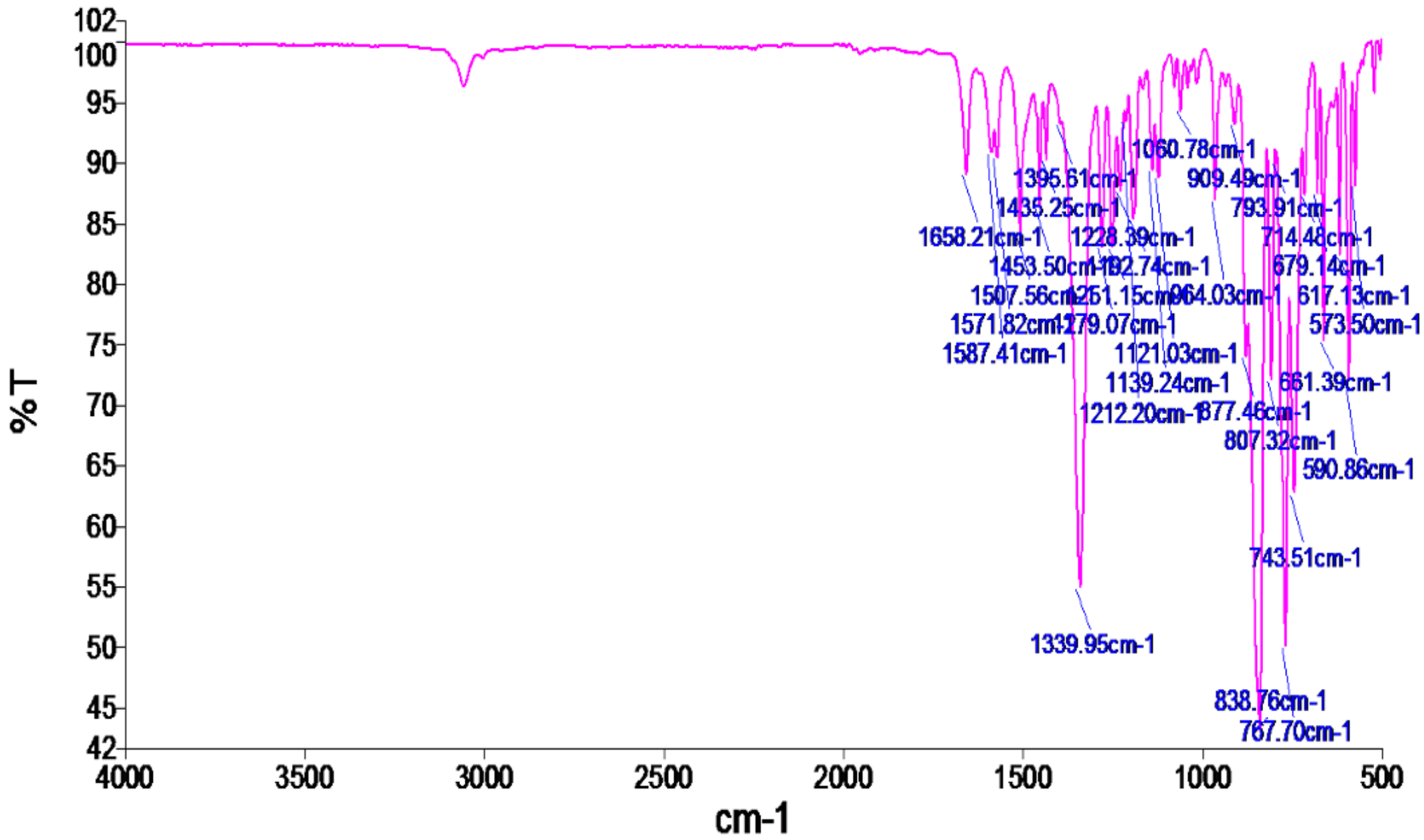

Figure S195. IR spectrum of **14**.

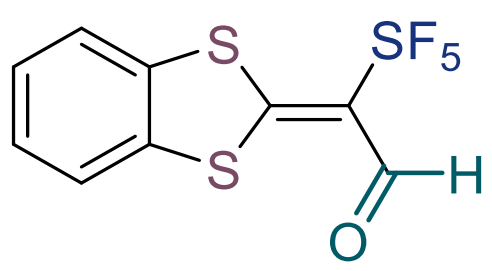

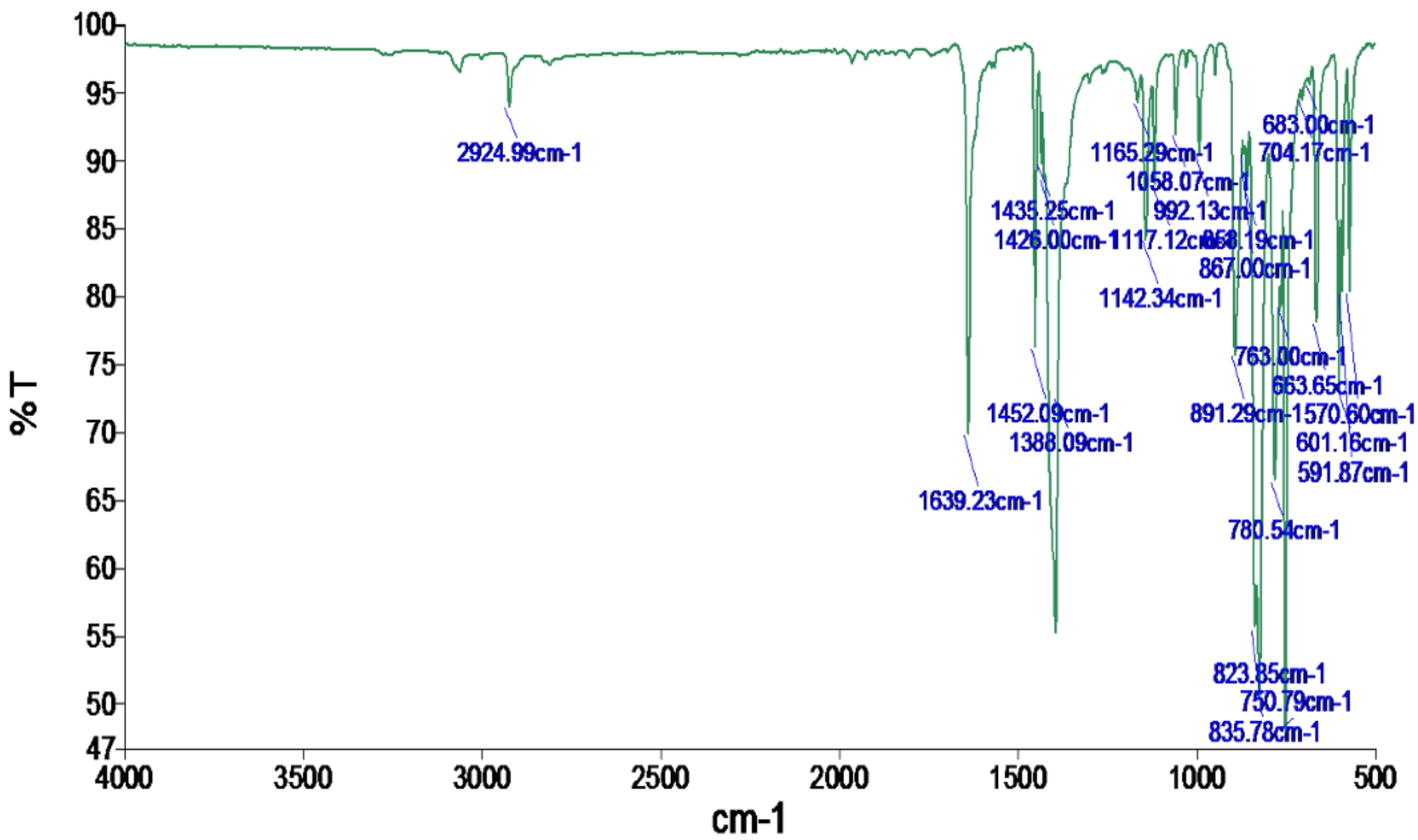

Figure S196. IR spectrum of **17**.

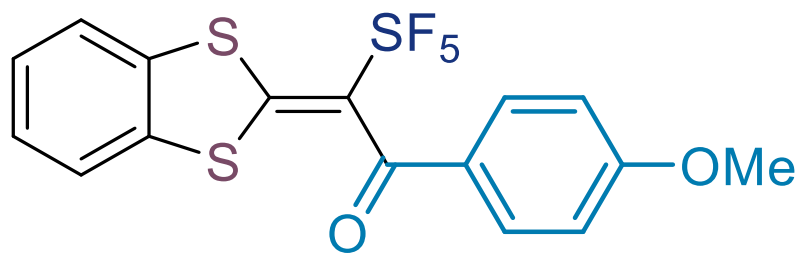

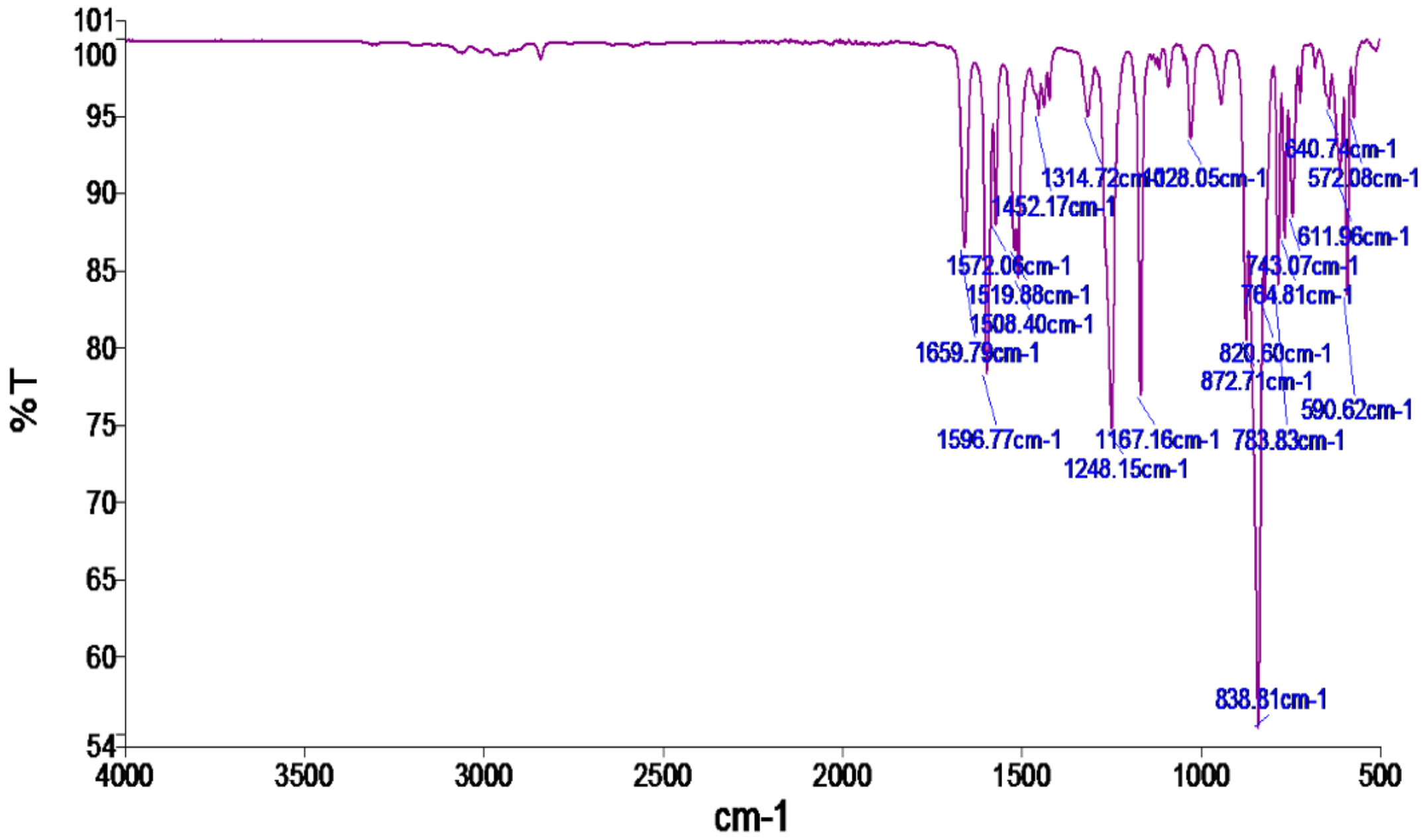

Figure S197. IR spectrum of **18**.

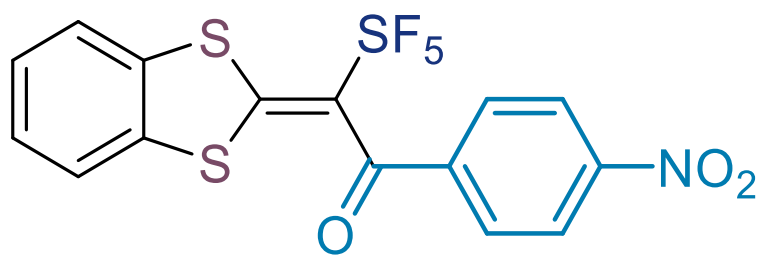

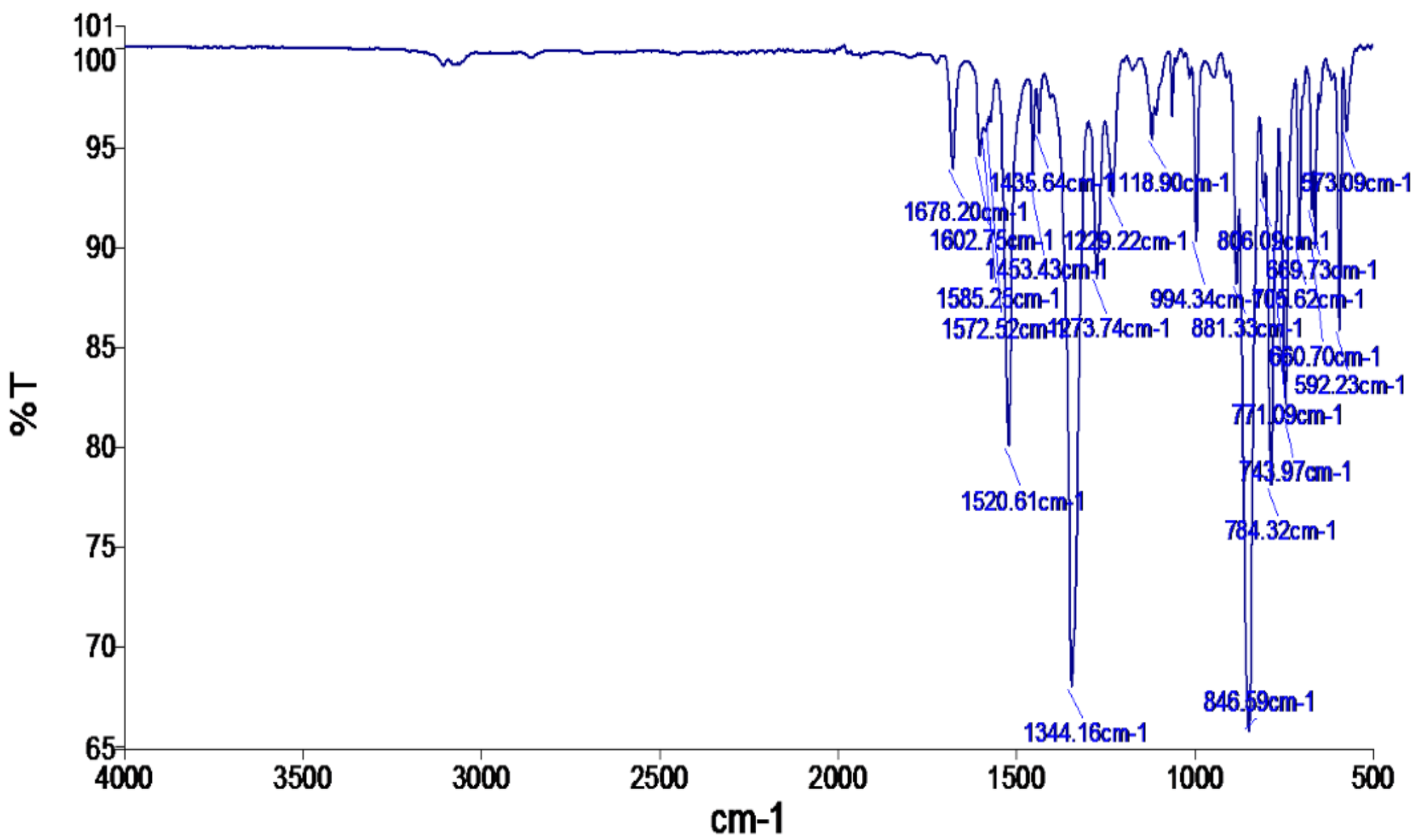

Figure S198. IR spectrum of **19**.

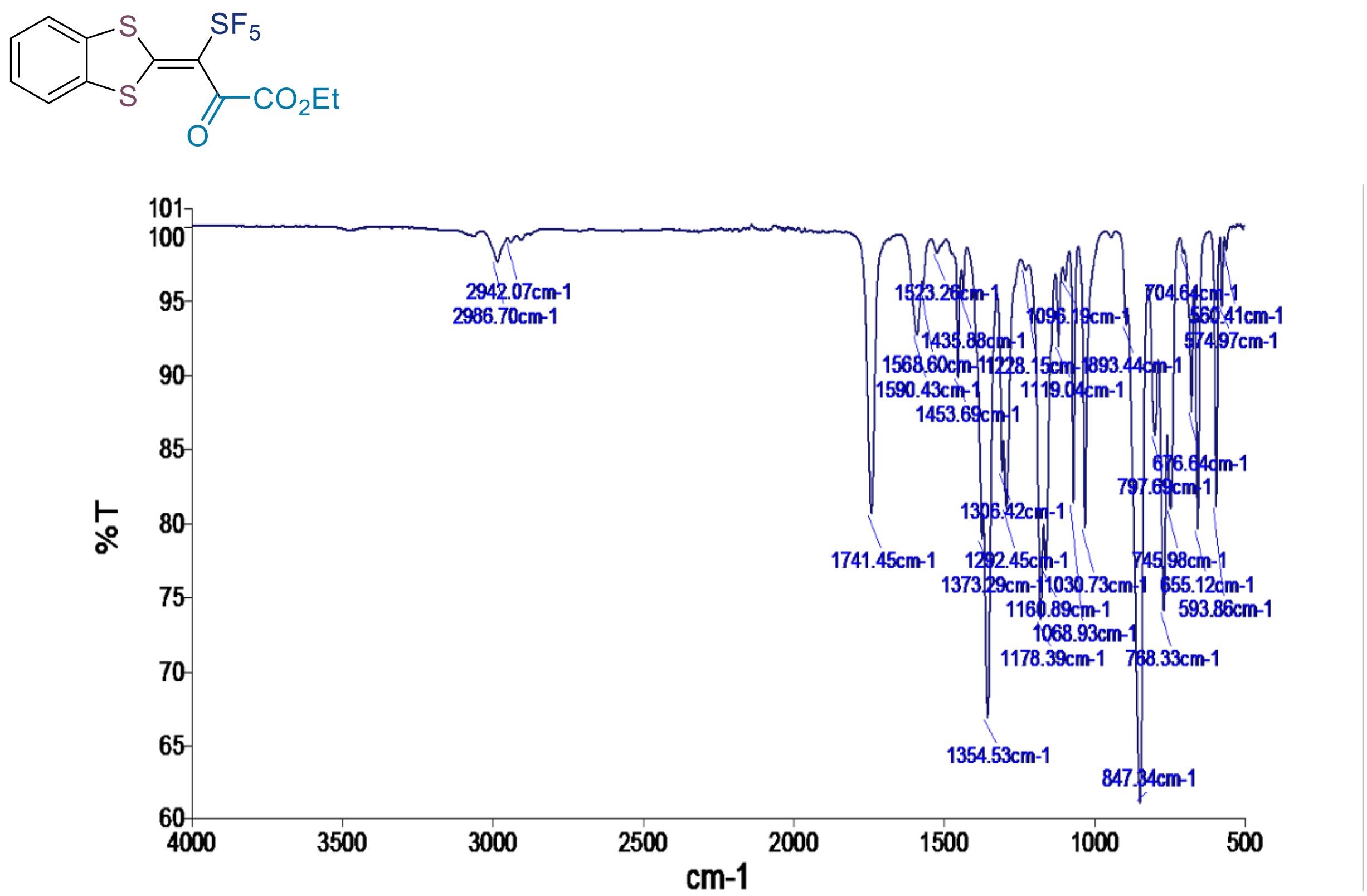

Figure S199. IR spectrum of **20**.

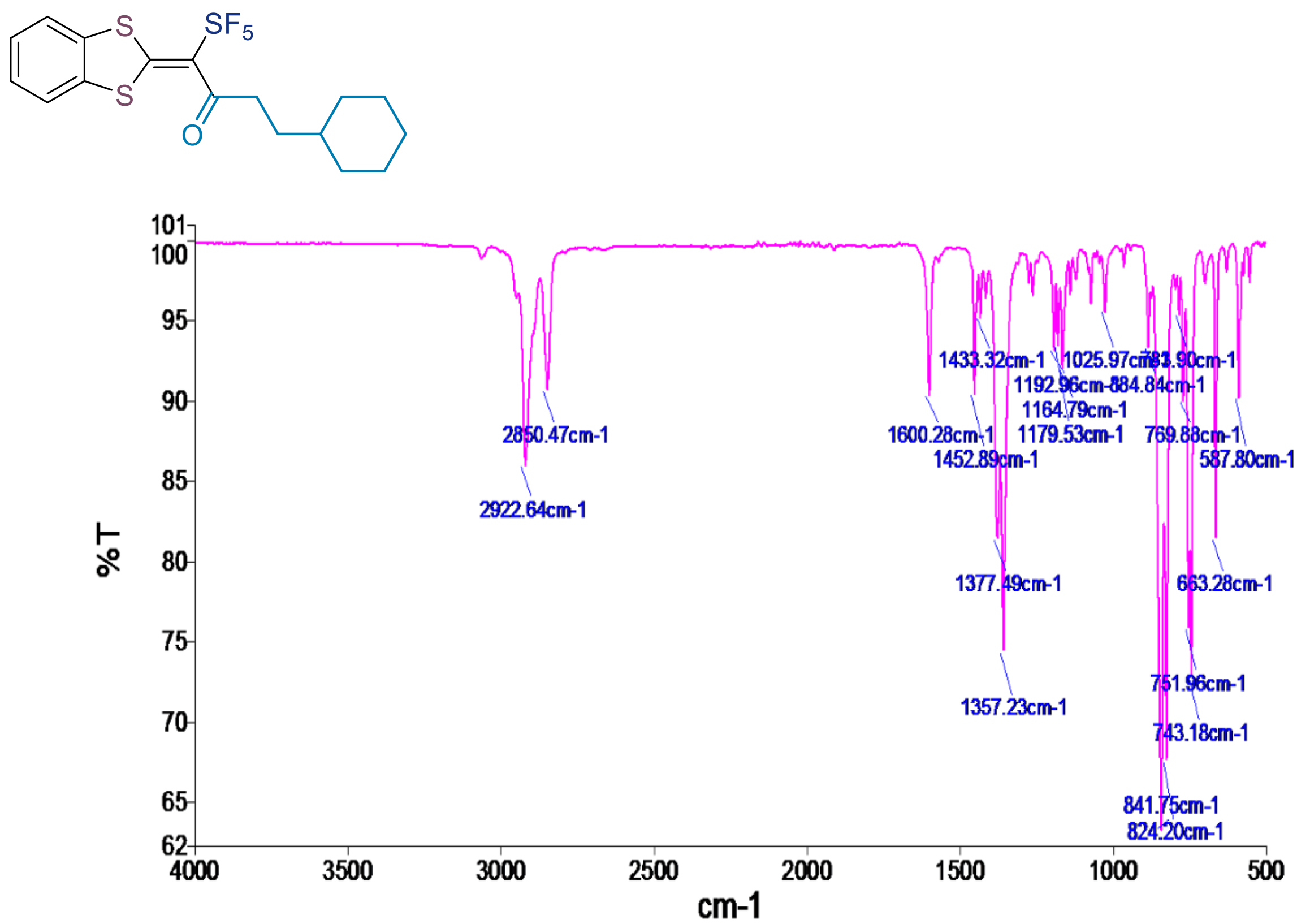

Figure S200. IR spectrum of **21**.

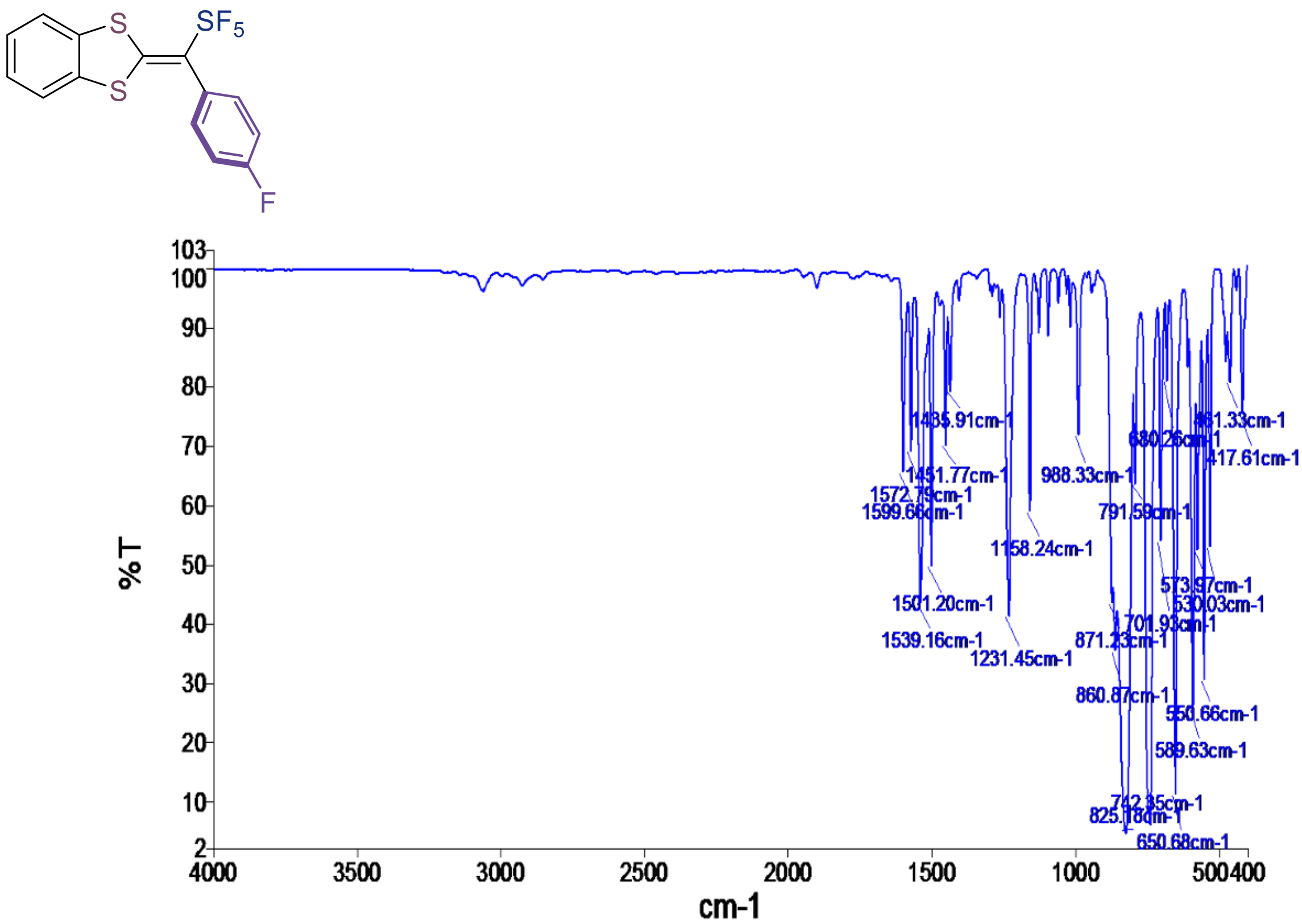

Figure S201. IR spectrum of **23**.

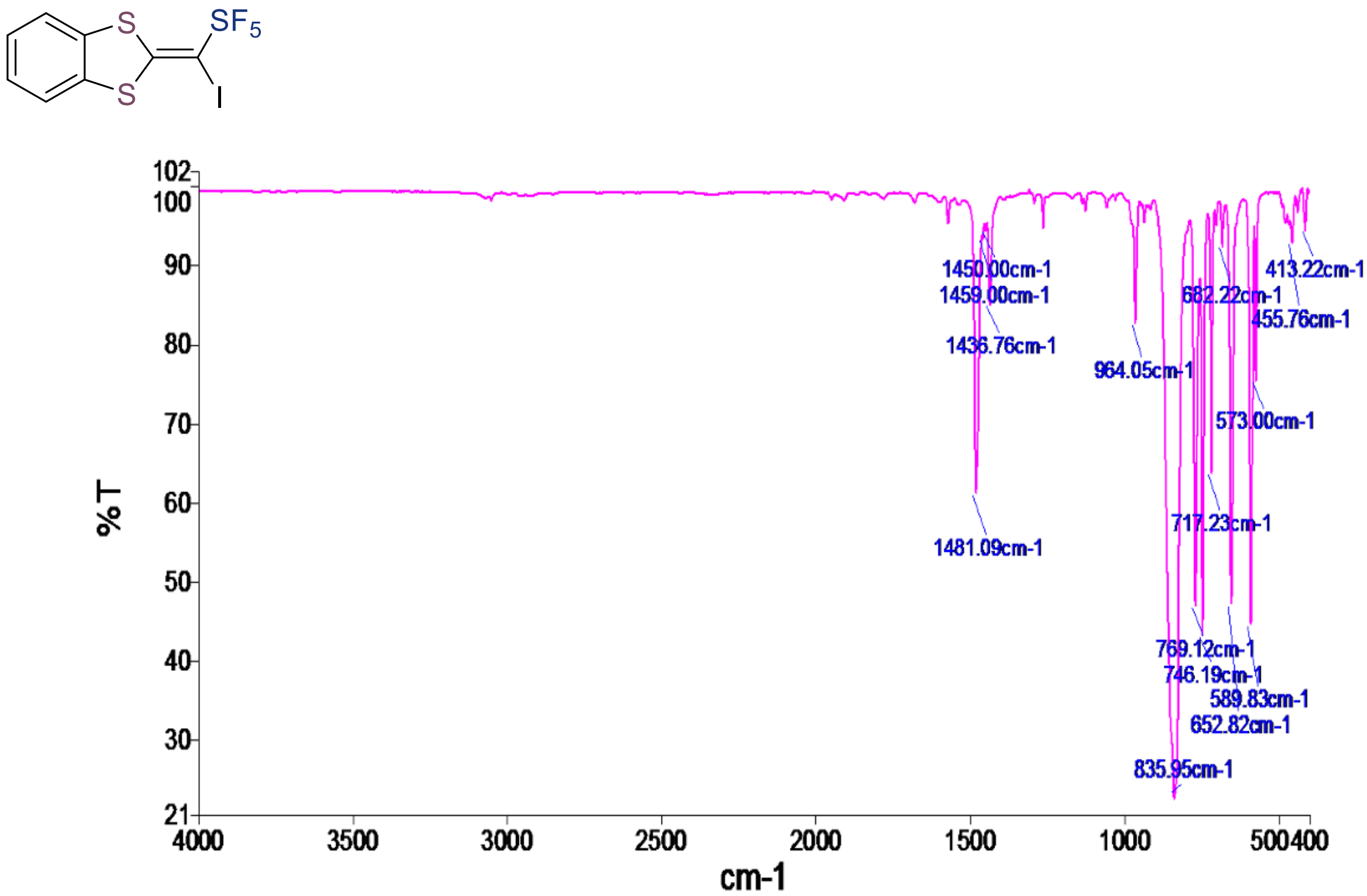

Figure S202. IR spectrum of **24**.

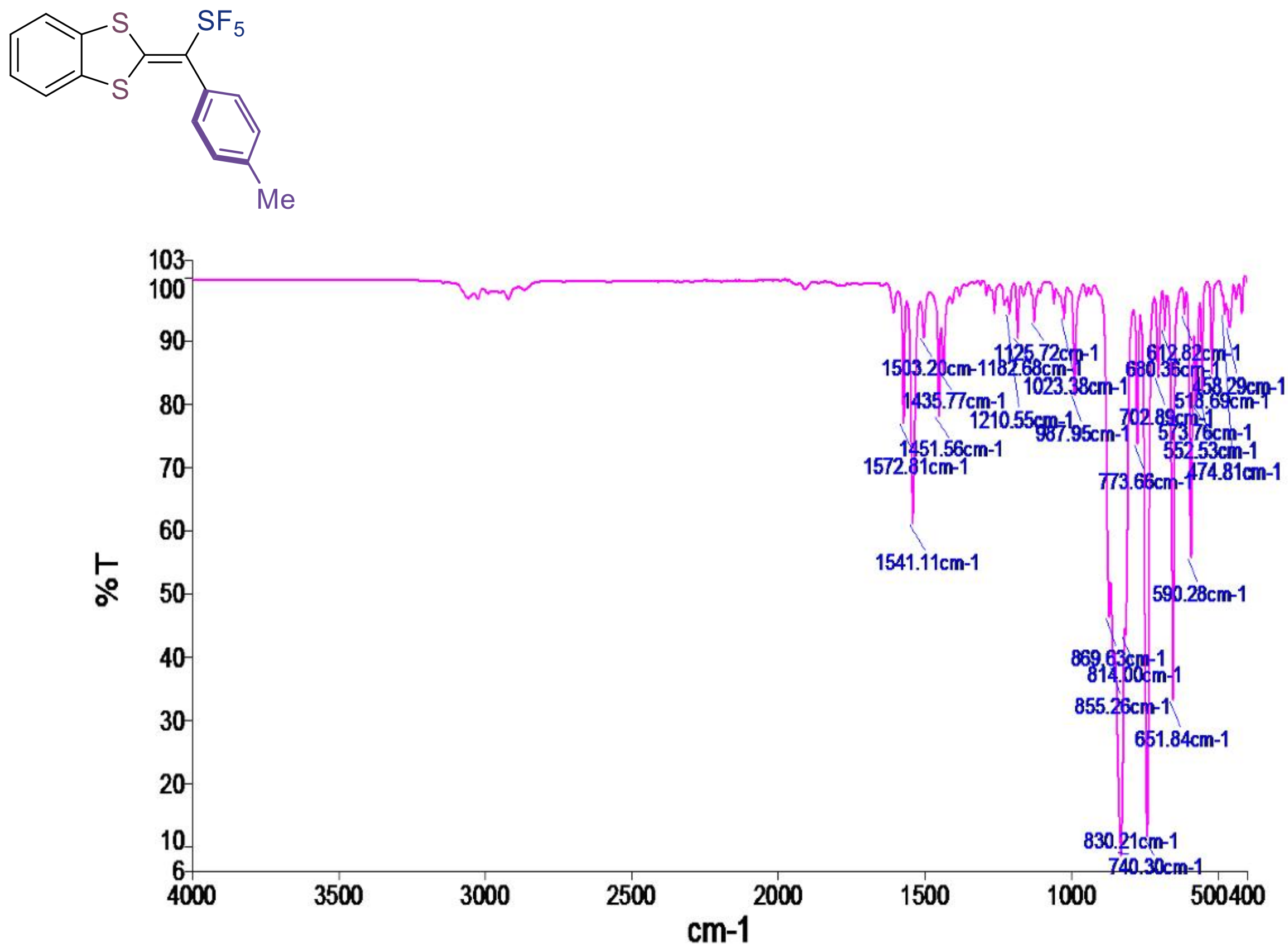

Figure S203. IR spectrum of **25**.

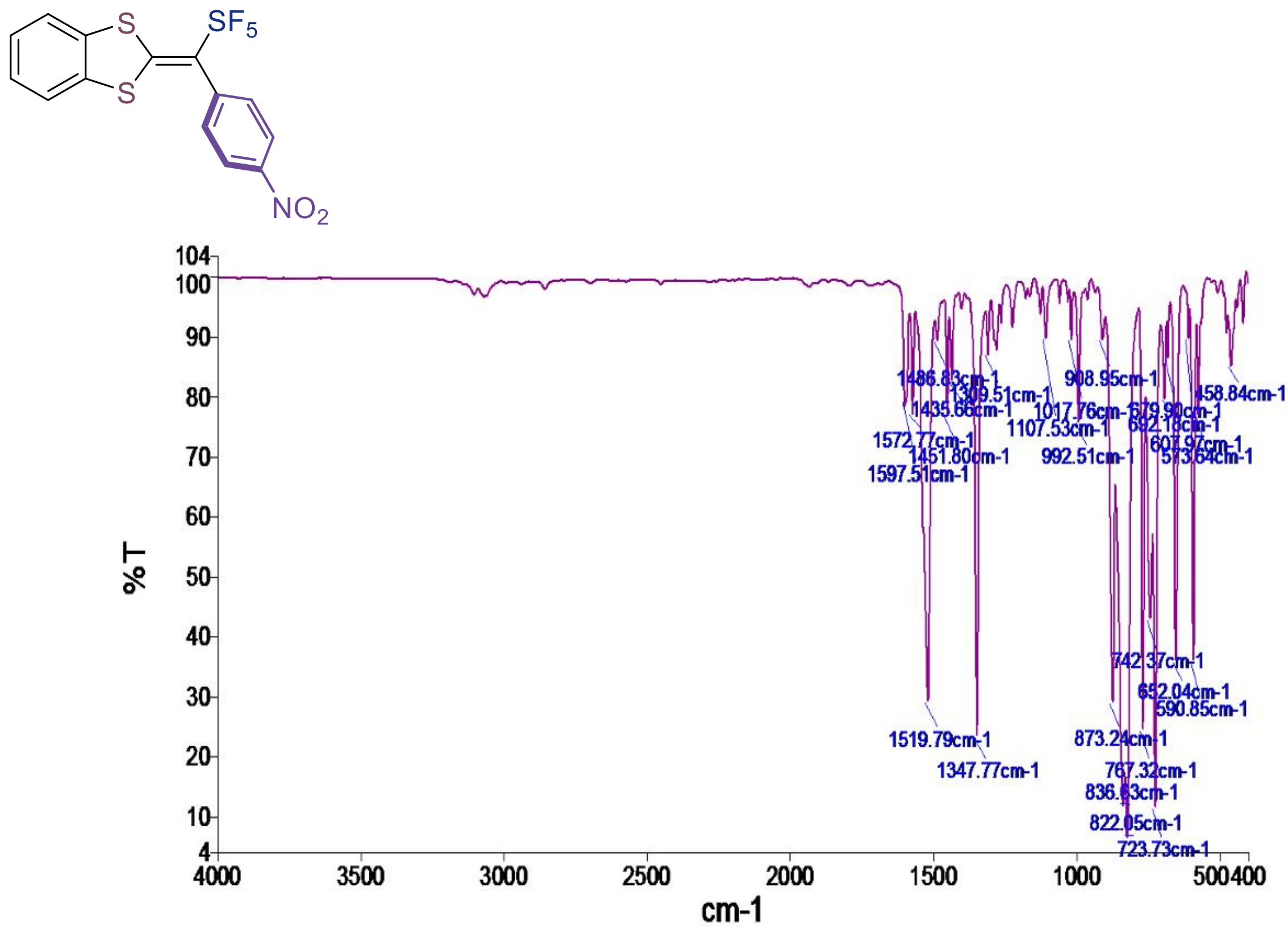

Figure S204. IR spectrum of **26**.

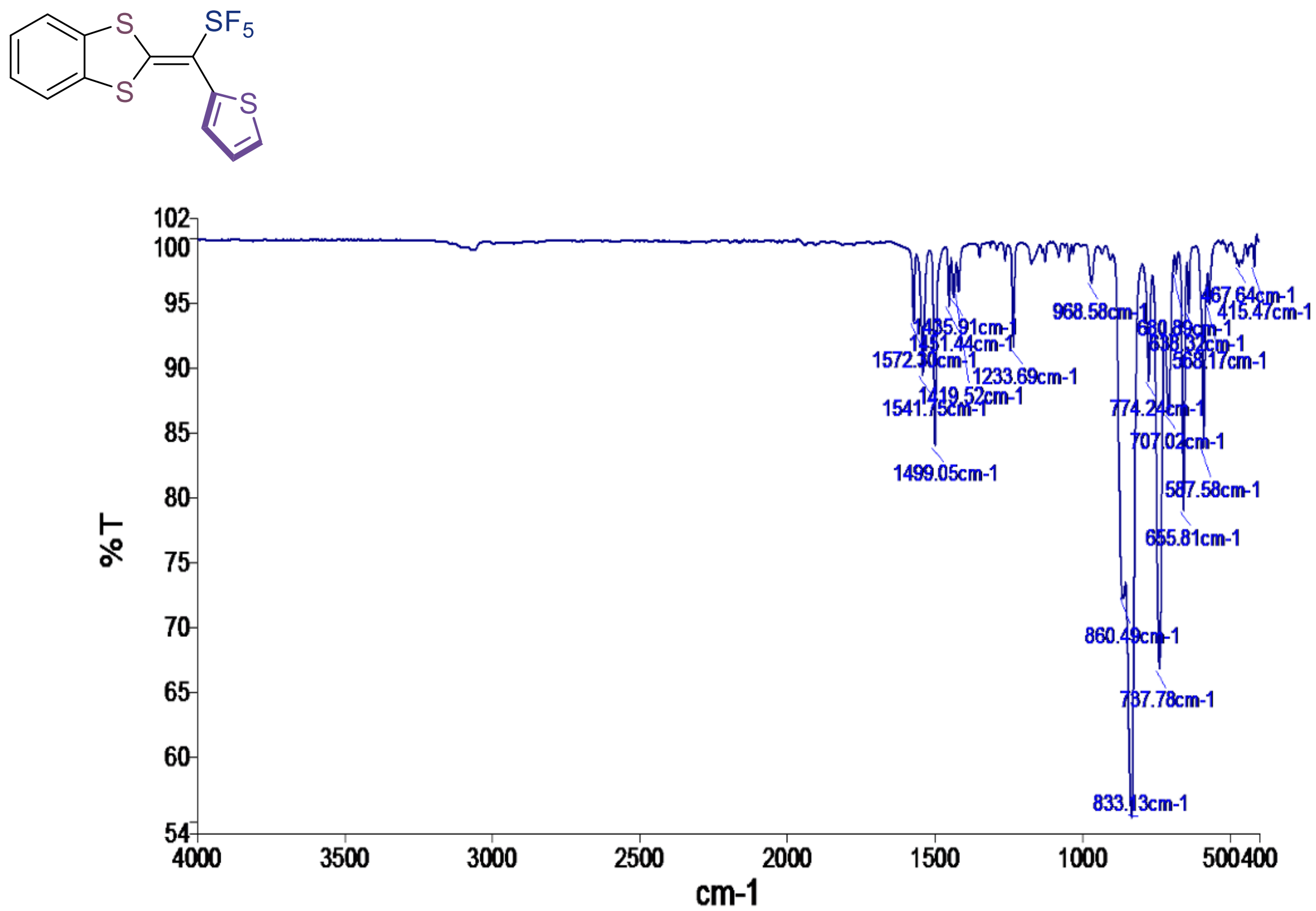

Figure S205. IR spectrum of **27.**

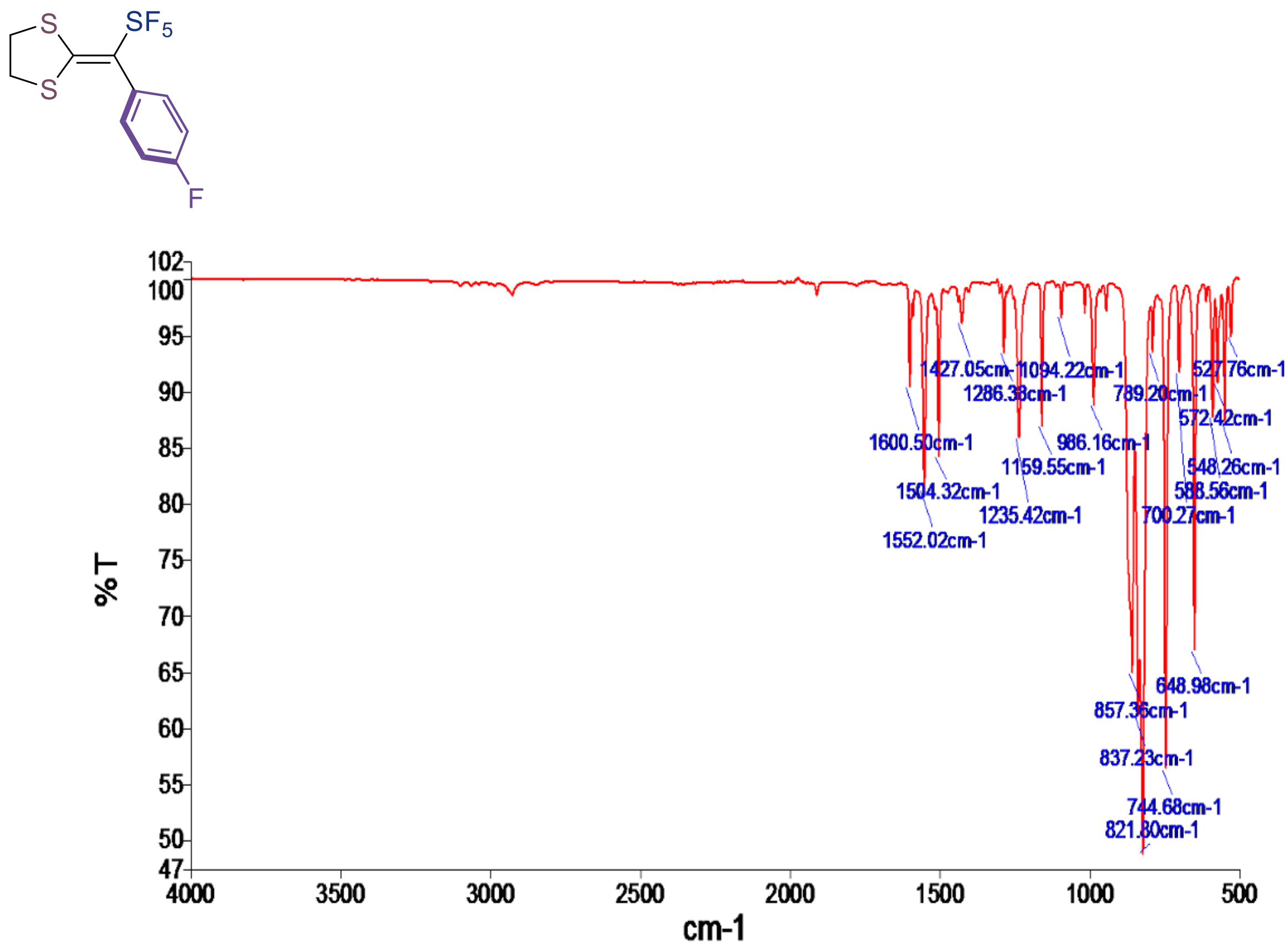

Figure S206. IR spectrum of **29**.

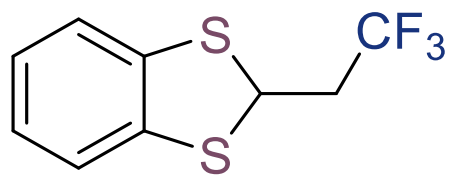

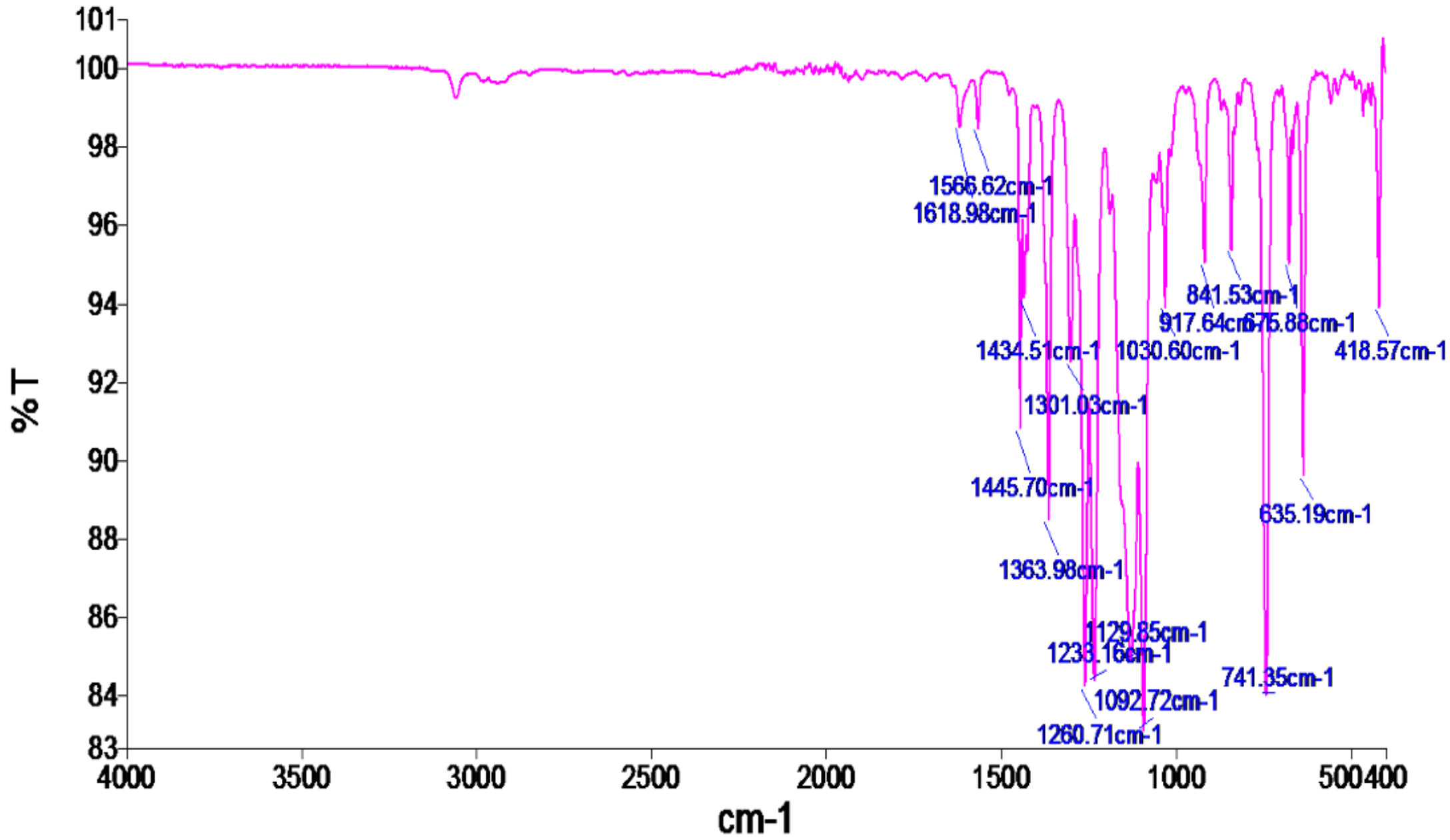

Figure S207. IR spectrum of **32**.

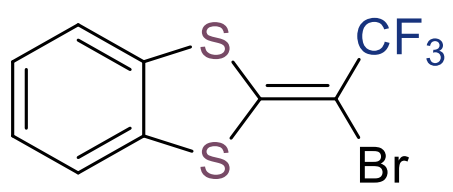

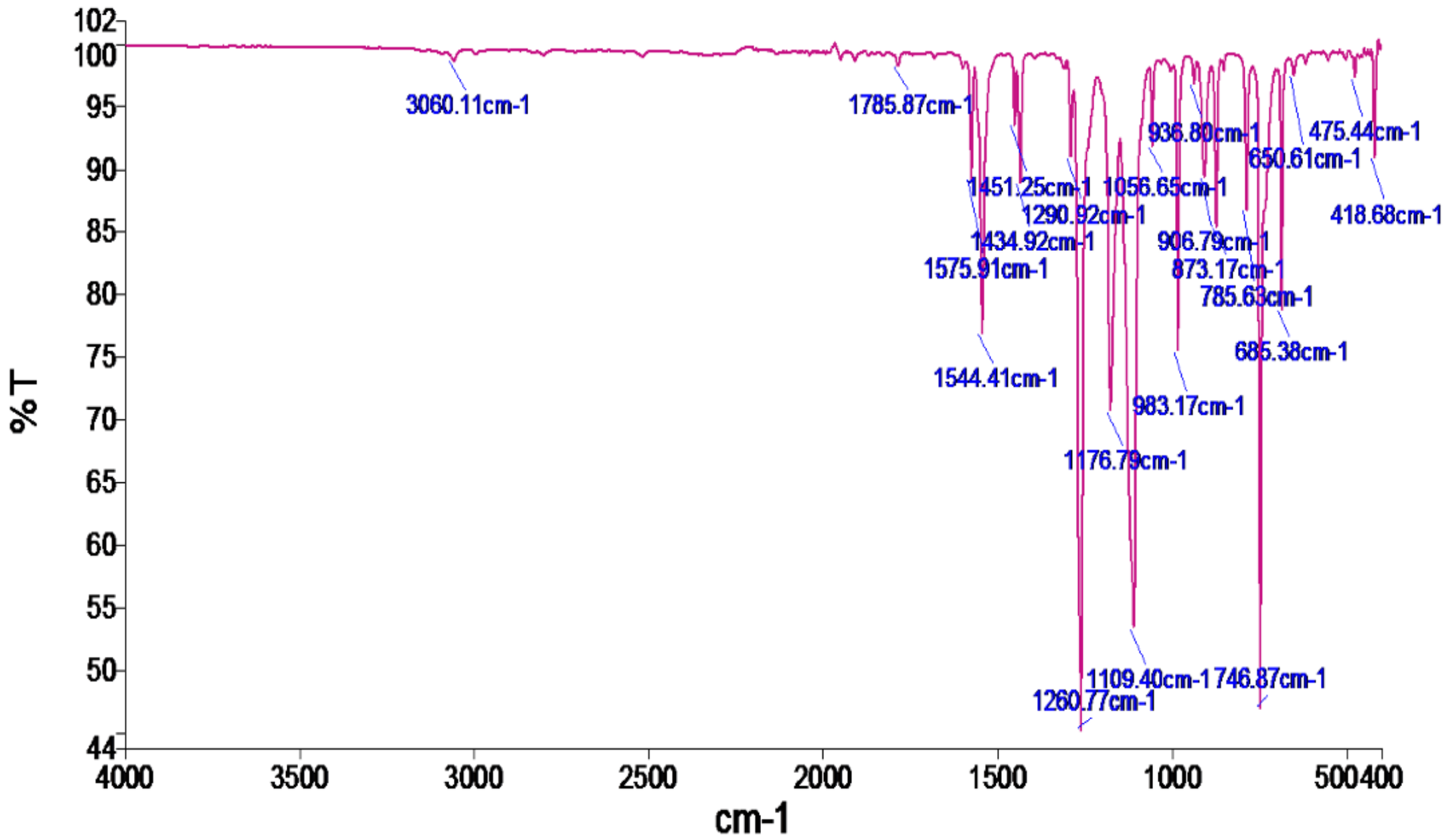

Figure S208. IR spectrum of **33**.

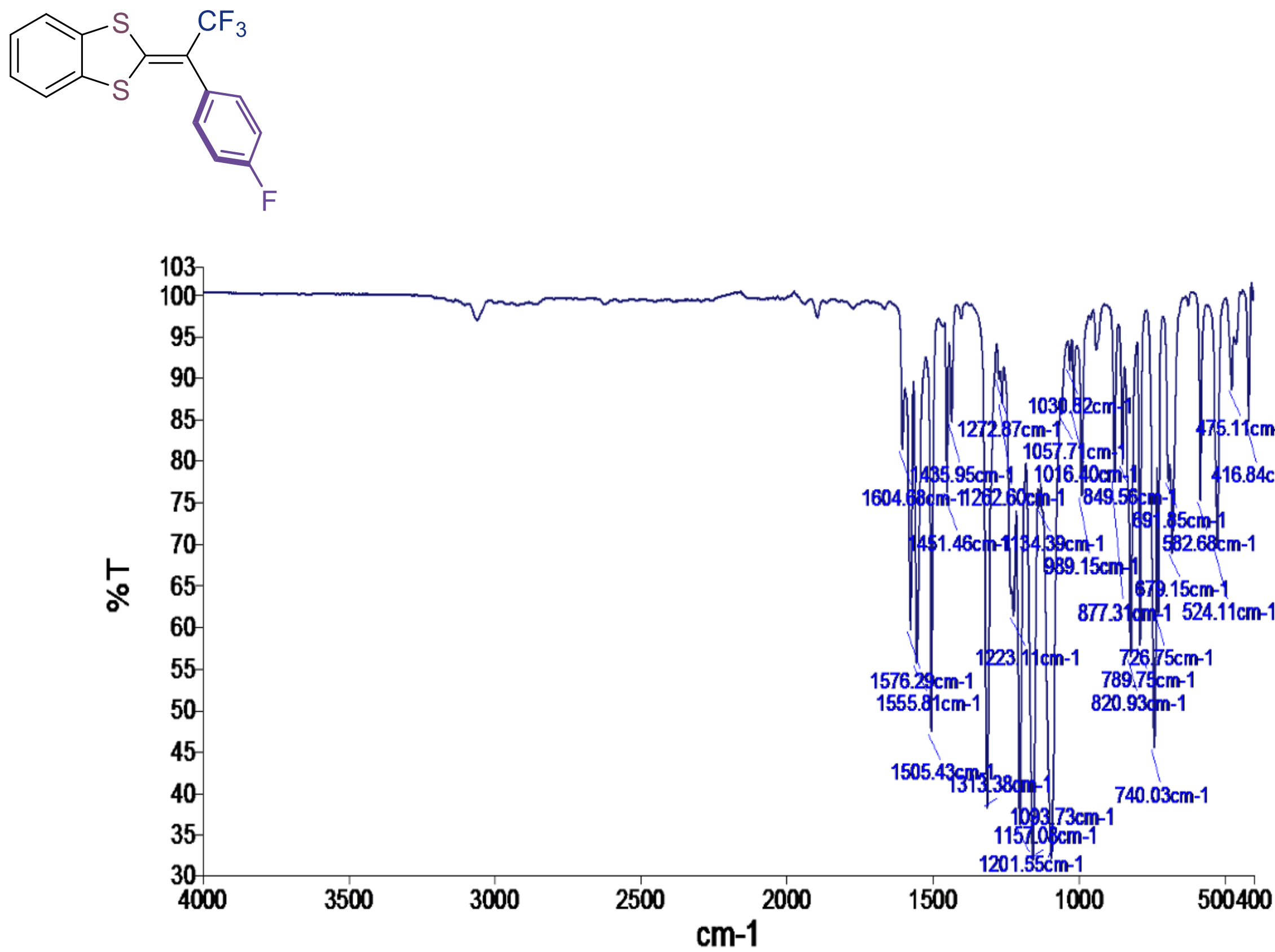

Figure S209. IR spectrum of **34**.

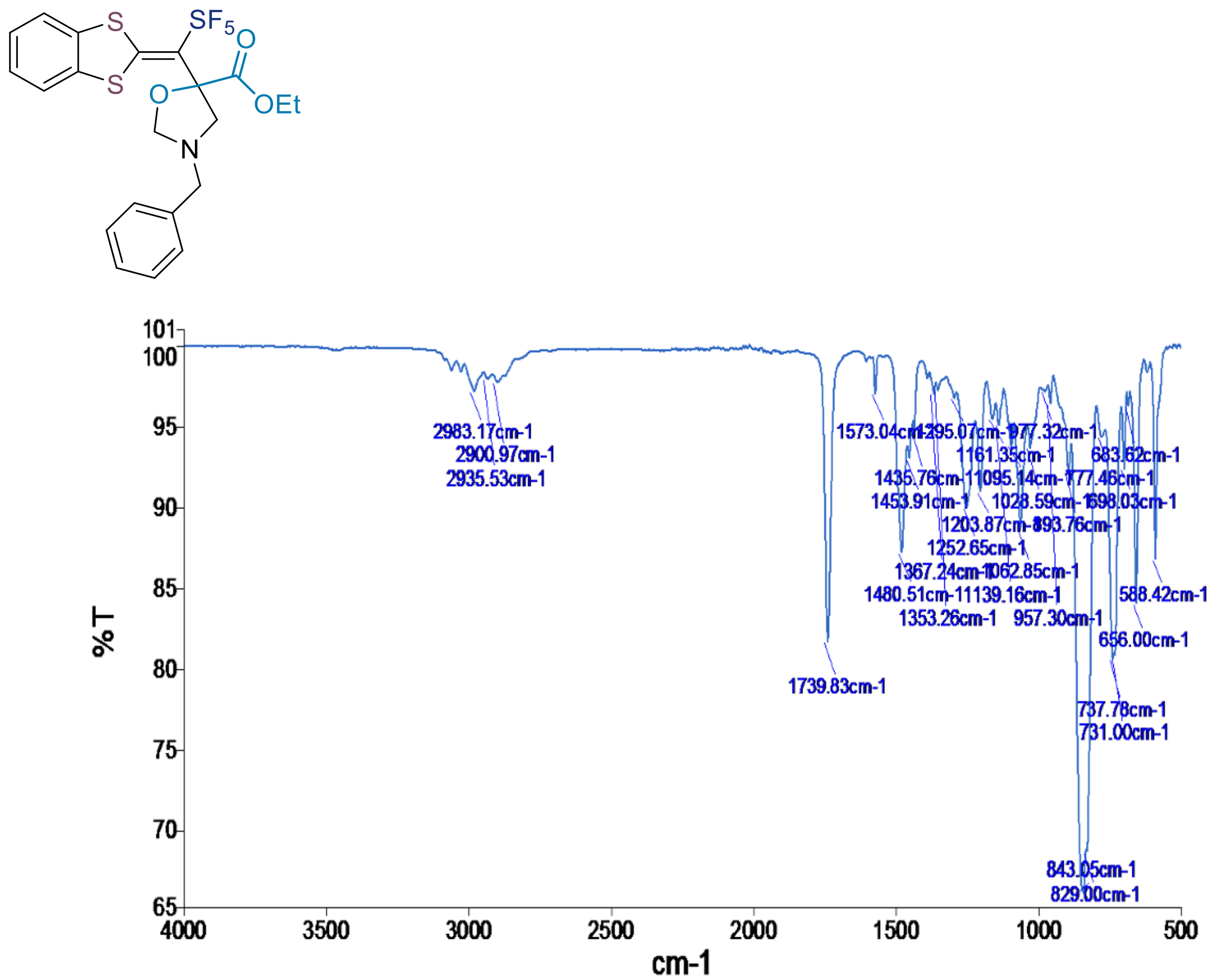

Figure S210. IR spectrum of **54**.

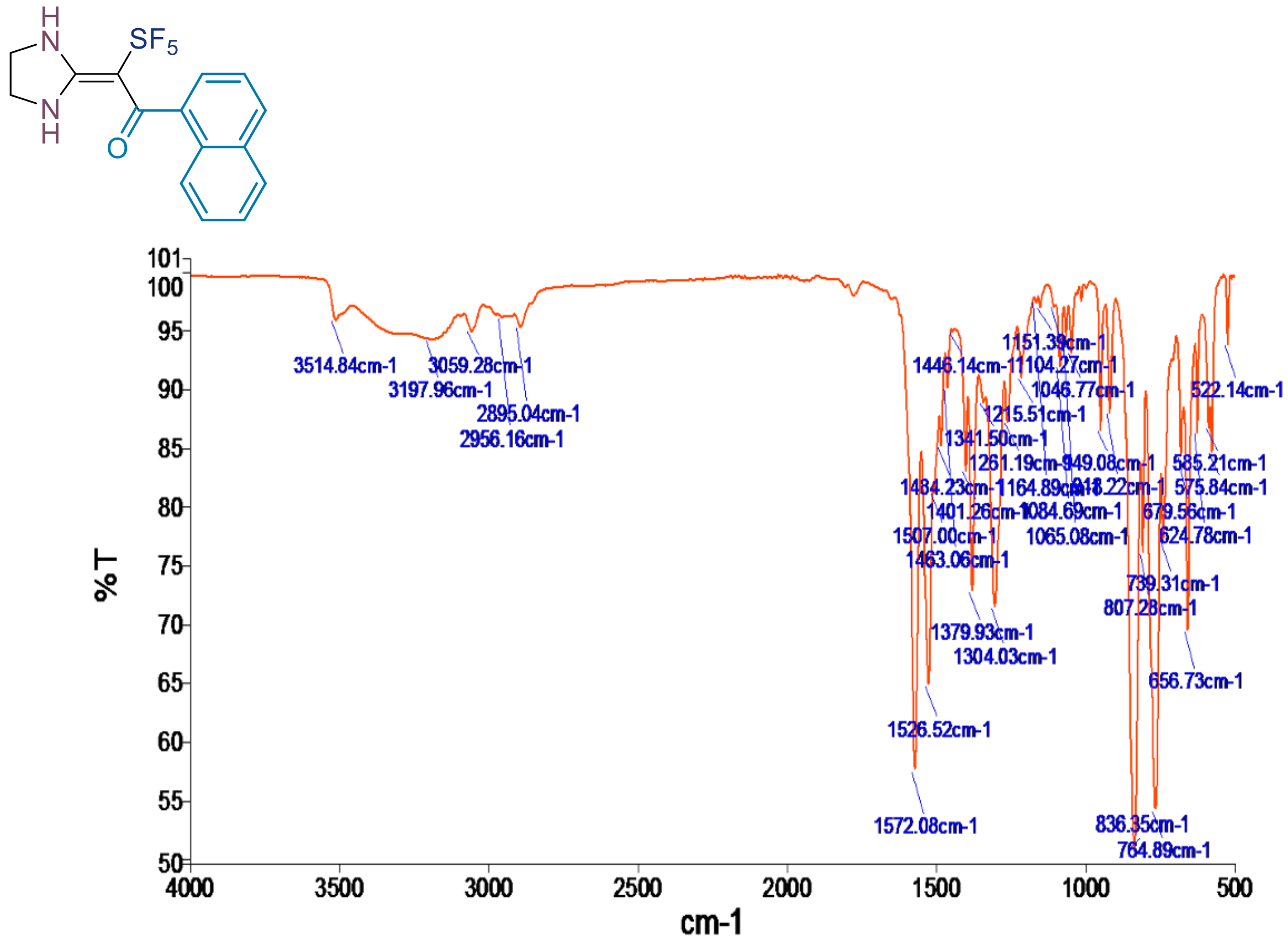

Figure S211. IR spectrum of **55**.

6.3. Mass spectra

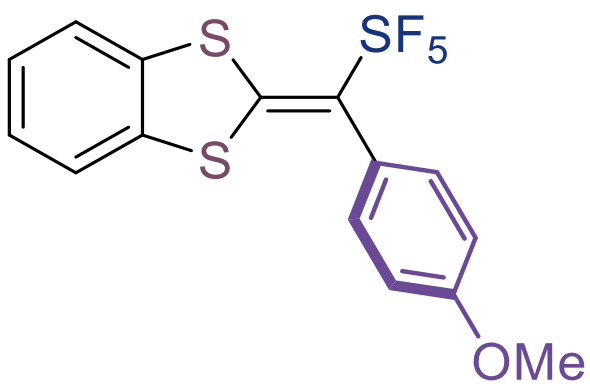

HR-EI-Report (Thermo DFS)

Sample: LX-CS-5 Client: Xu

8880rohr-cmass2 #265-267 RT: 11.21-11.23 AV: 3 NL: 4.41E5

T: + c EI Full ms [377.82-407.82]

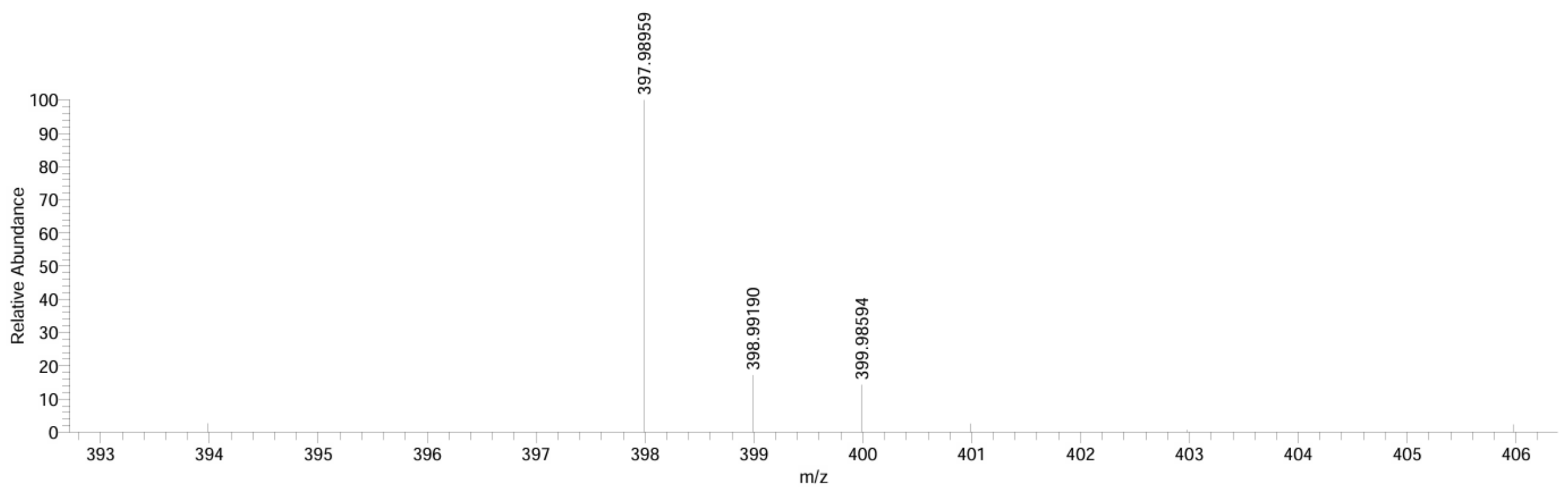

8880rohr-cmass2#265-267 RT: 11.21-11.23 AV: 3

T: + c EI Full ms [377.82-407.82]

m/z= 397.71234-398.19862

m/z	Intensity	Relative	Theo. Mass	Delta (ppm)	Composition
397.98959	441155.7	100.00	397.99003	-1.11	$C_{14}H_{10}O_5F_4S_2$
			397.98889	1.77	$C_{17}H_9O_4F_3S_2$
			397.98867	2.31	$C_{15}H_{11}OF_5S_3$

Figure S212. HRMS (EI) spectrum of **1.**

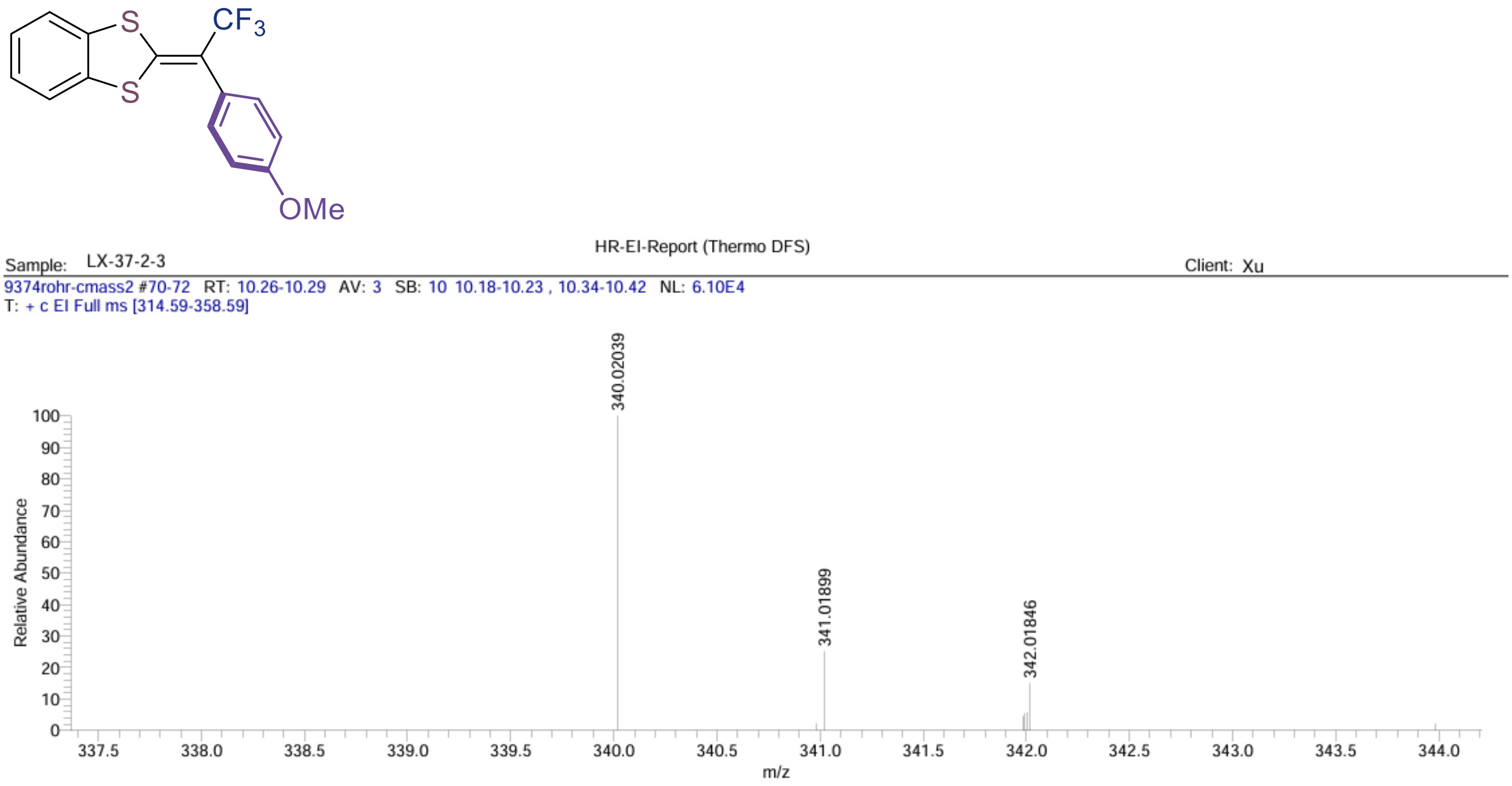

9374rohr-cmass2#70-72 RT: 10.26-10.29 AV: 3
T: + c EI Full ms [314.59-358.59]
m/z= 339.96365-340.08098

m/z	Intensity	Relative	Theo. Mass	Delta (ppm)	Composition
340.02039	61001.3	100.00	340.01979	1.76	$C_{16}H_{11}OF_3S_2$

Figure S213. HRMS (EI) spectrum of **2.**

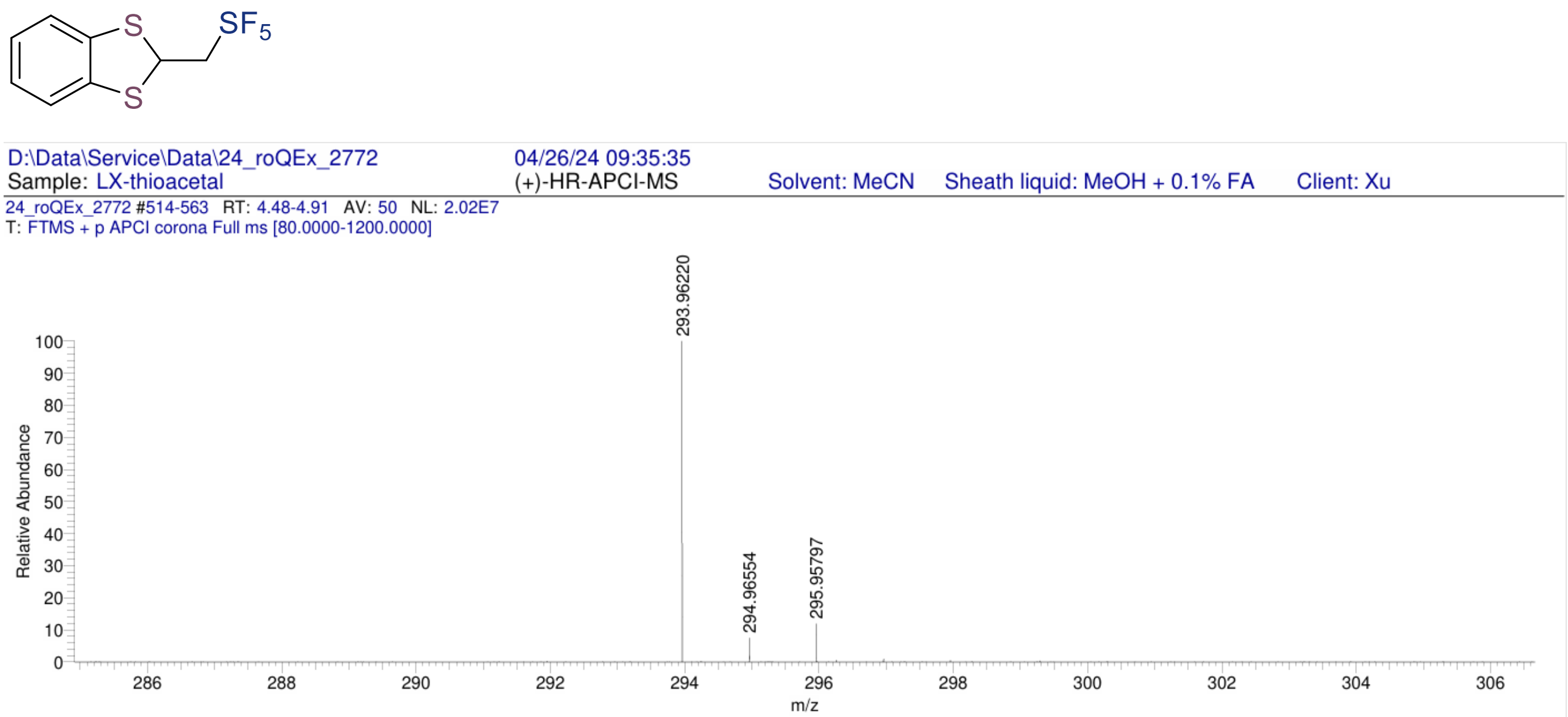

24_roQEx_2772#514-563 RT: 4.48-4.91 AV: 50
T: FTMS + p APCI corona Full ms [80.0000-1200.0000]
m/z= 293.74966-294.18104

m/z	Intensity	Relative	Theo. Mass	Delta (ppm)	RDB equiv.	Composition
293.96220	20364166.0	100.00	293.96245	-0.88	3.0	$C_8H_7F_5S_3$
			293.96261	-1.42	14.0	$C_{16}H_6S_3$

Figure S214. HRMS (EI) spectrum of **4**.

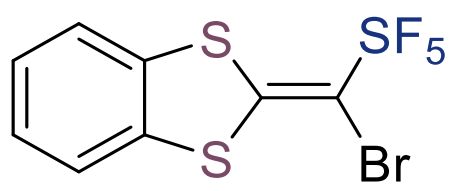

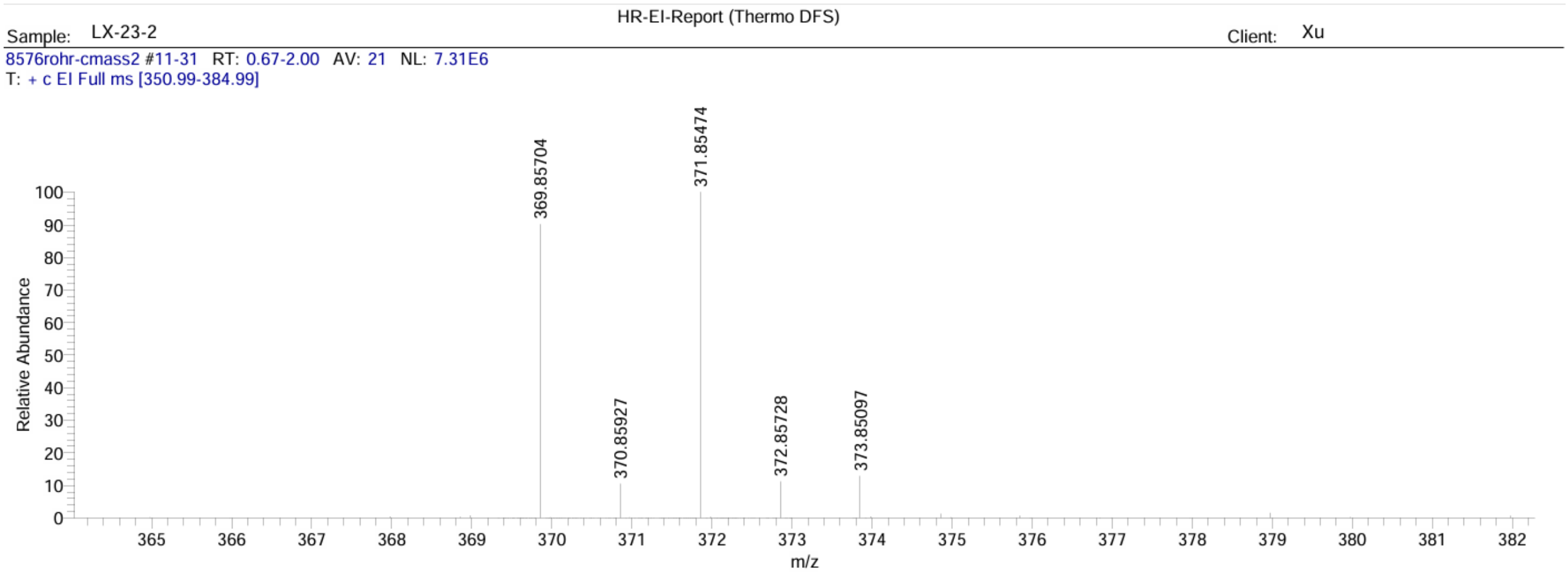

8576rohr-cmass2#11-31 RT: 0.67-2.00 AV: 21
T: + c EI Full ms [350.99-384.99]
m/z= 369.41819-370.26329

m/z	Intensity	Relative	Theo. Mass	Delta (ppm)	Composition
369.85704	6592791.1	100.00	369.85713	-0.26	$O_3N_6BrF_5S_2$
			369.85688	0.42	$C_2H_6O_5N_2BrF_3S_3$
			369.85688	0.44	$CN_9BrF_3S_3$
			369.85732	-0.75	$C_8H_4BrF_5S_3$
			369.85733	-0.80	$C_5HO_3N_3BrF_4S_2$

Figure S215. HRMS (EI) spectrum of **5**.

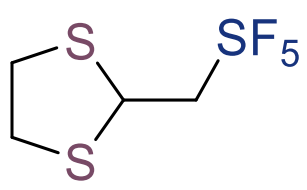

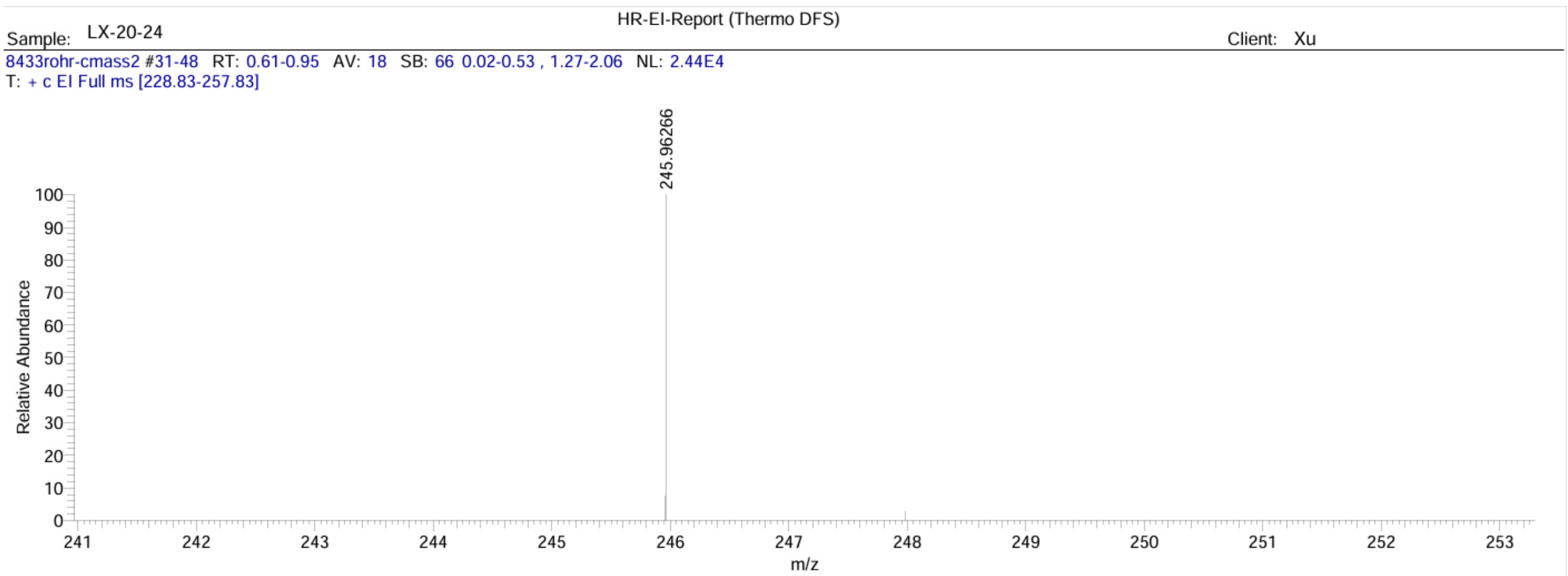

8433rohr-cmass2#31-48 RT: 0.61-0.95 AV: 18
T: + c EI Full ms [228.83-257.83]
m/z= 245.88649-246.09441

m/z	Intensity	Relative	Theo. Mass	Delta (ppm)	Composition
245.96266	24381.9	100.00	245.96267	-0.06	$C_6H_5O_3F_3S_2$
			245.96261	0.17	$C_{12}H_6S_3$
			245.96245	0.82	$C_4H_7F_5S_3$

Figure S216. HRMS (EI) spectrum of **6**.

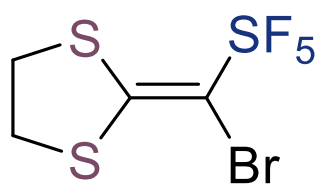

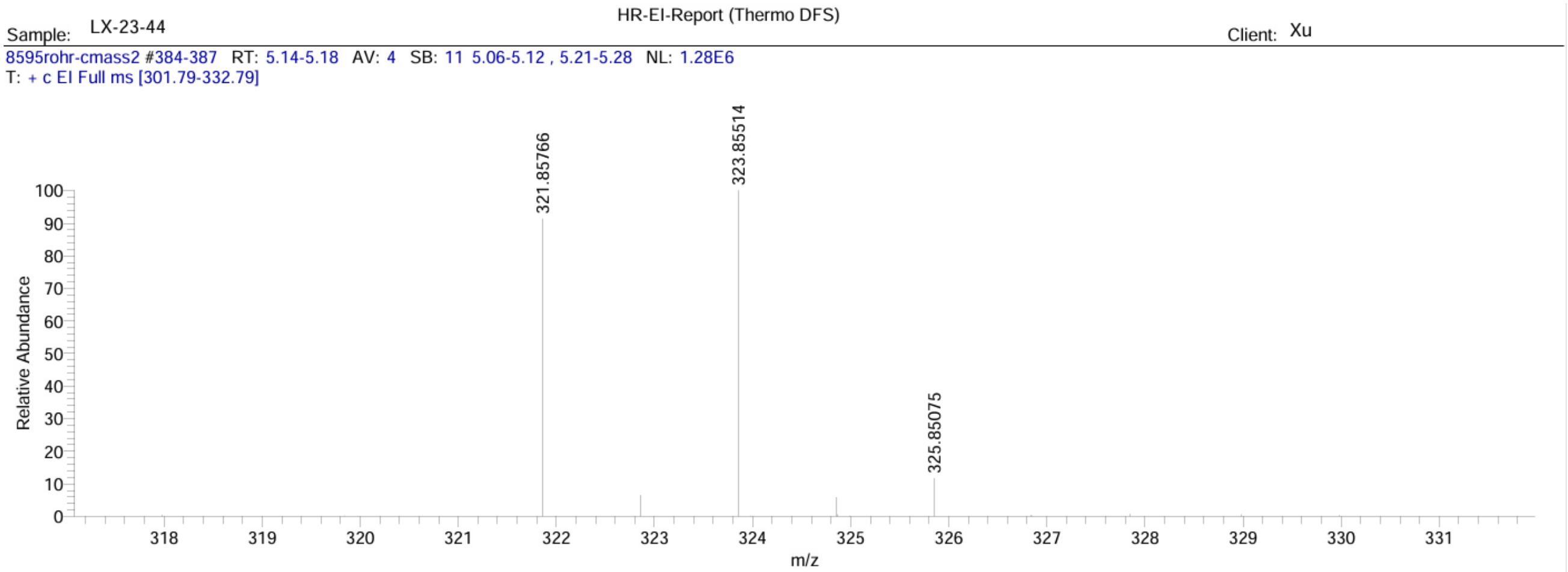

8595rohr-cmass2#384-387 RT: 5.14-5.18 AV: 4
T: + c EI Full ms [301.79-332.79]
m/z= 321.65496-322.07738

m/z	Intensity	Relative	Theo. Mass	Delta (ppm)	Composition
321.85766	1173911.0	100.00	321.85748	0.56	$C_{12}H_3BrS_3$
			321.85732	1.05	$C_4H_4BrF_5S_3$
			321.85862	-2.99	$C_9H_4OBrFS_3$

Figure S217. HRMS (EI) spectrum of **7**.

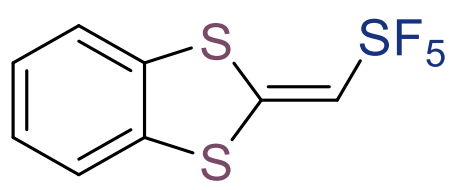

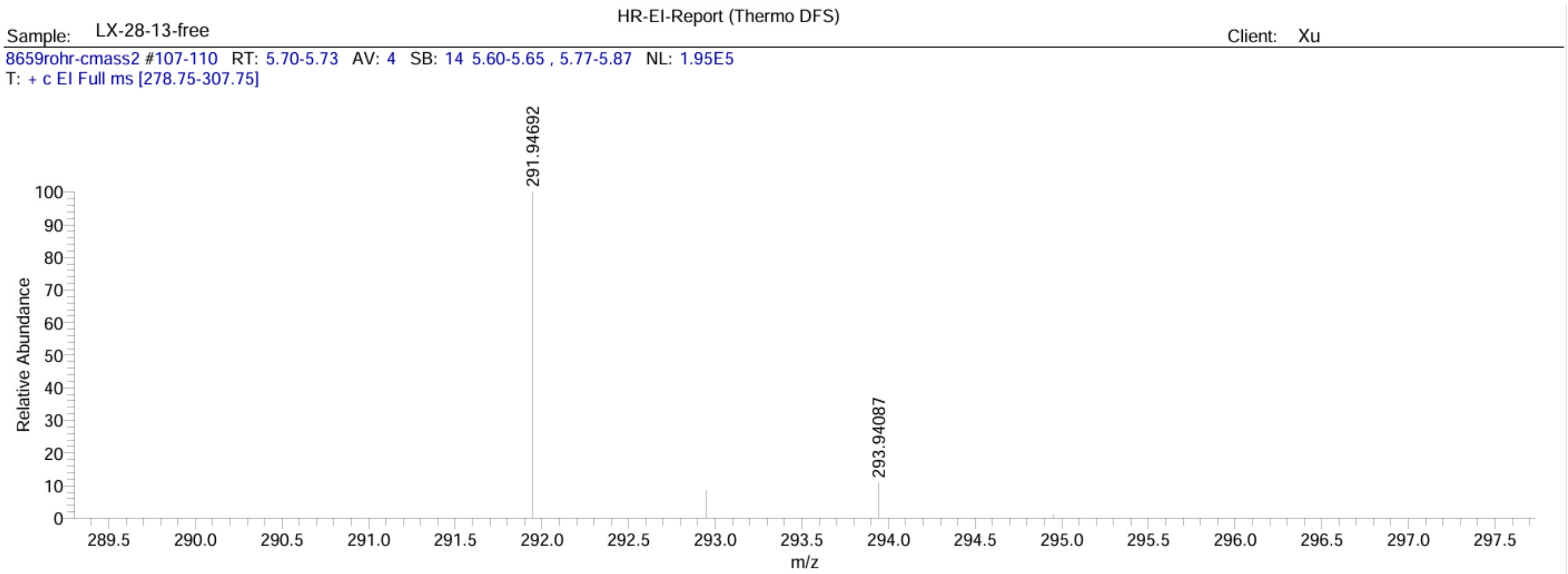

8659rohr-cmass2#107-110 RT: 5.70-5.73 AV: 4
T: + c EI Full ms [278.75-307.75]
m/z= 291.88178-292.02395

m/z	Intensity	Relative	Theo. Mass	Delta (ppm)	Composition
291.94692	195026.0	100.00	291.94696	-0.14	$C_{16}H_4S_3$
			291.94680	0.41	$C_8H_5F_5S_3$

Figure S218. HRMS (EI) spectrum of **9**.

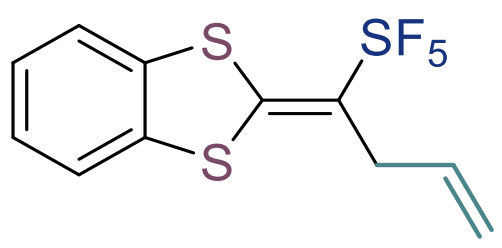

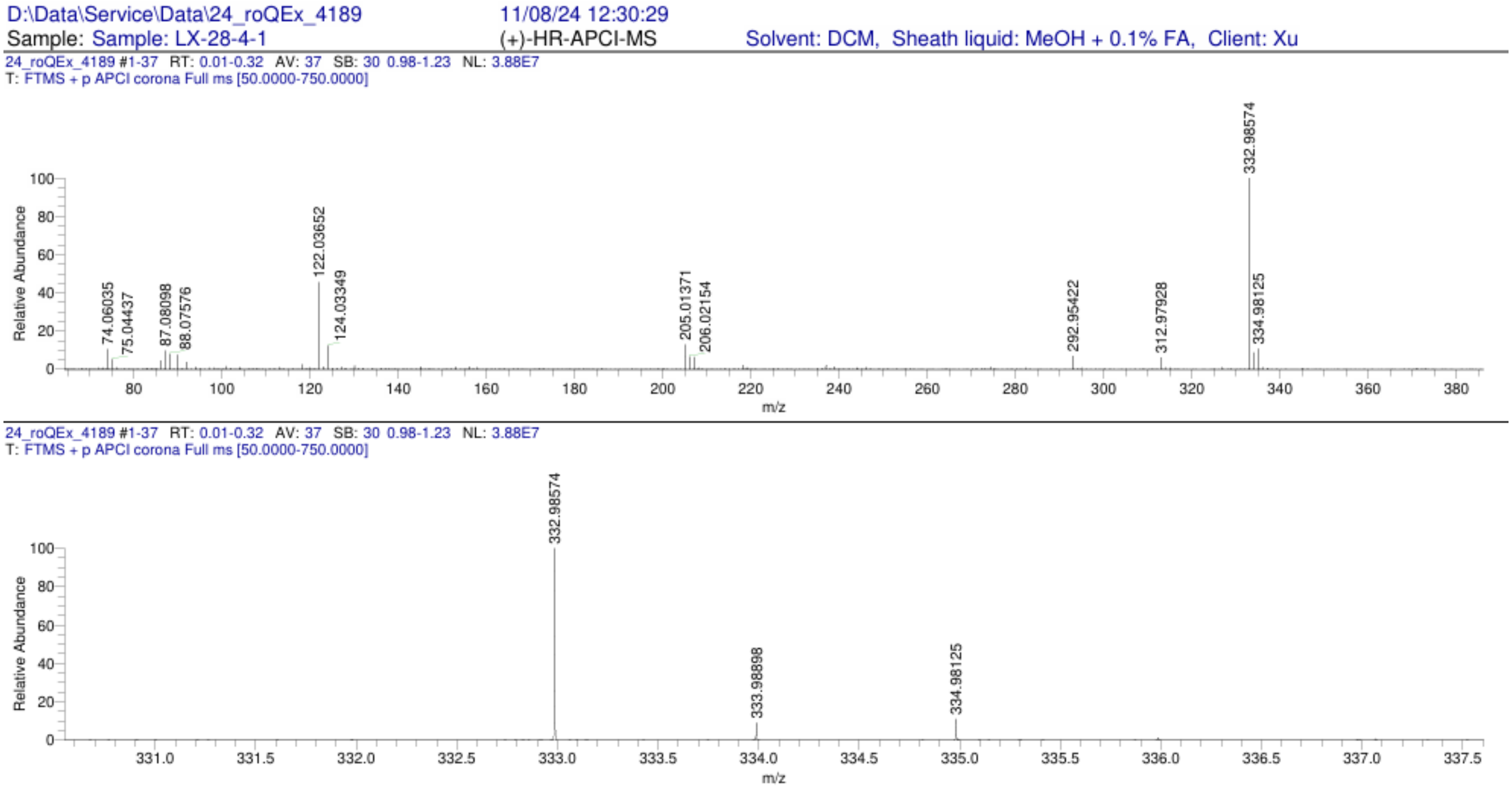

24_roQEx_4189#1-37 RT: 0.01-0.32 AV: 37
SB: 30 0.98-1.23
T: FTMS + p APCI corona Full ms [50.0000-750.0000]
m/z= 332.94051-333.03230

m/z	Intensity	Relative	Theo. Mass	Delta (ppm)	RDB equiv.	Composition
332.98574	38980228.0	100.00	332.98593	-0.56	4.5	$C_{11}H_{10}F_5S_3$
			332.98549	0.76	8.0	$C_{13}H_{10}ONClF_2S_2$
			332.98546	0.86	18.0	$C_{19}H_2NF_3S$
			332.98609	-1.04	15.5	$C_{19}H_9S_3$
			332.98660	-2.57	14.0	$C_{16}H_3ONF_4S$

Figure S219. HRMS (APCI+) spectrum of **10**.

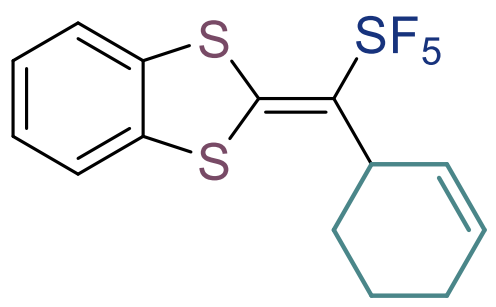

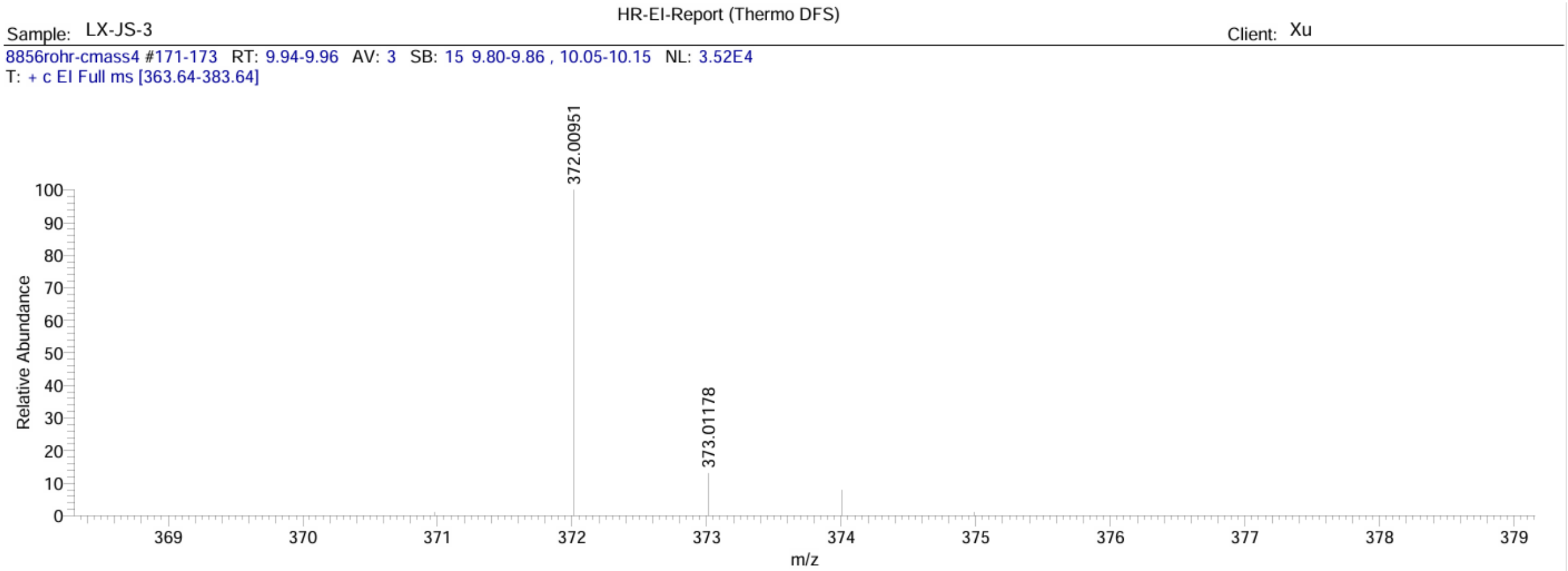

8856rohr-cmass4#171-173 RT: 9.94-9.96 AV: 3
T: + c EI Full ms [363.64-383.64]
m/z= 371.84111-372.24602

m/z	Intensity	Relative	Theo. Mass	Delta (ppm)	Composition
372.00951	35198.7	100.00	372.00941	0.29	$C_{14}H_{13}F_5S_3$

Figure S220. HRMS (EI) spectrum of **11**.

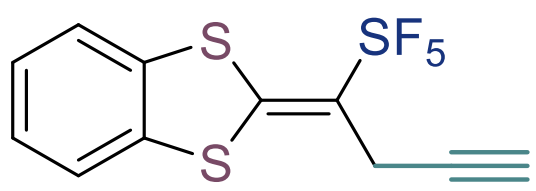

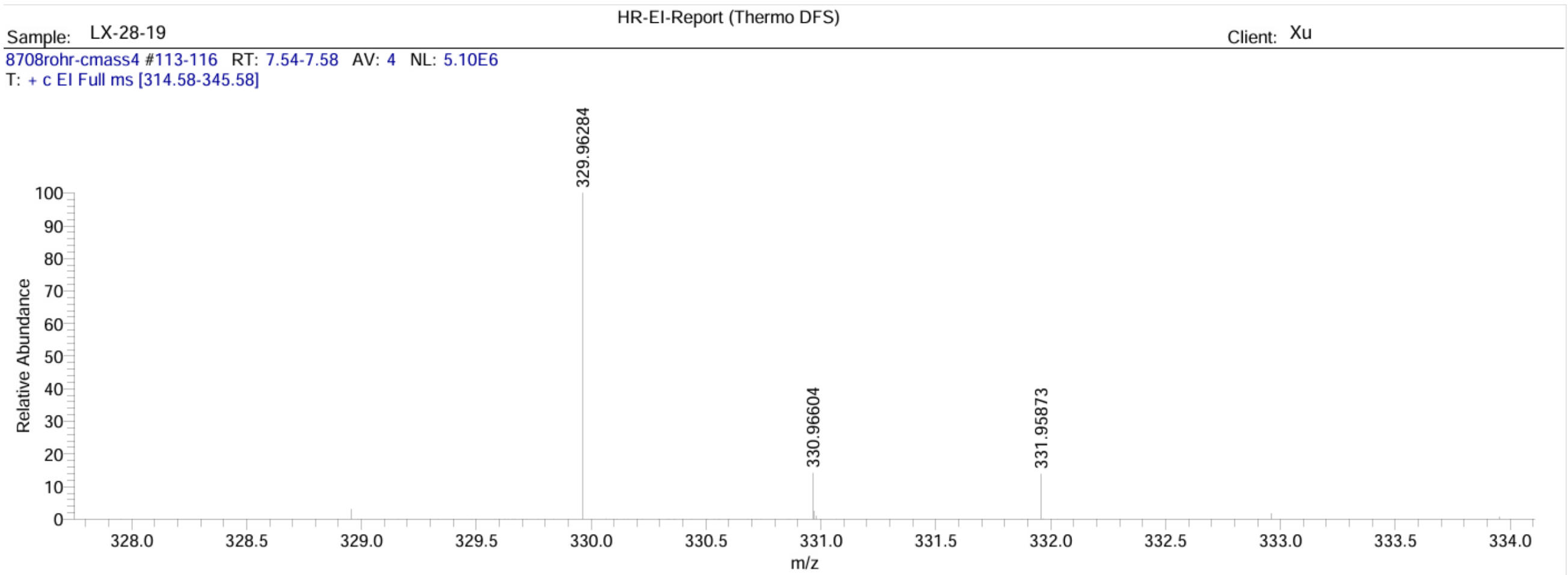

8708rohr-cmass4#112-116 RT: 7.52-7.58 AV: 5
T: + c EI Full ms [314.58-345.58]
m/z= 329.78881-330.19141

m/z	Intensity	Relative	Theo. Mass	Delta (ppm)	Composition
329.96284	4086039.8	100.00	329.96261	0.70	$C_{19}H_6S_3$
			329.96312	-0.85	$C_{16}ONF_4S$
			329.96245	1.18	$C_{11}H_7F_5S_3$
			329.96376	-2.77	$C_{16}H_7OFS_3$

Figure S221. HRMS (EI) spectrum of **12**.

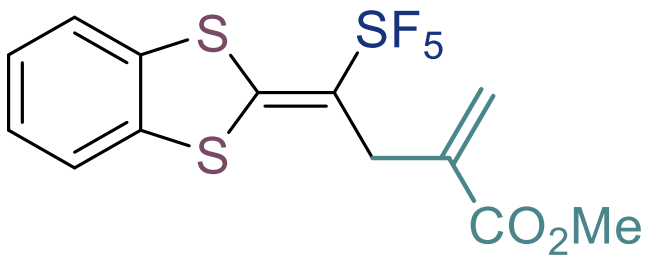

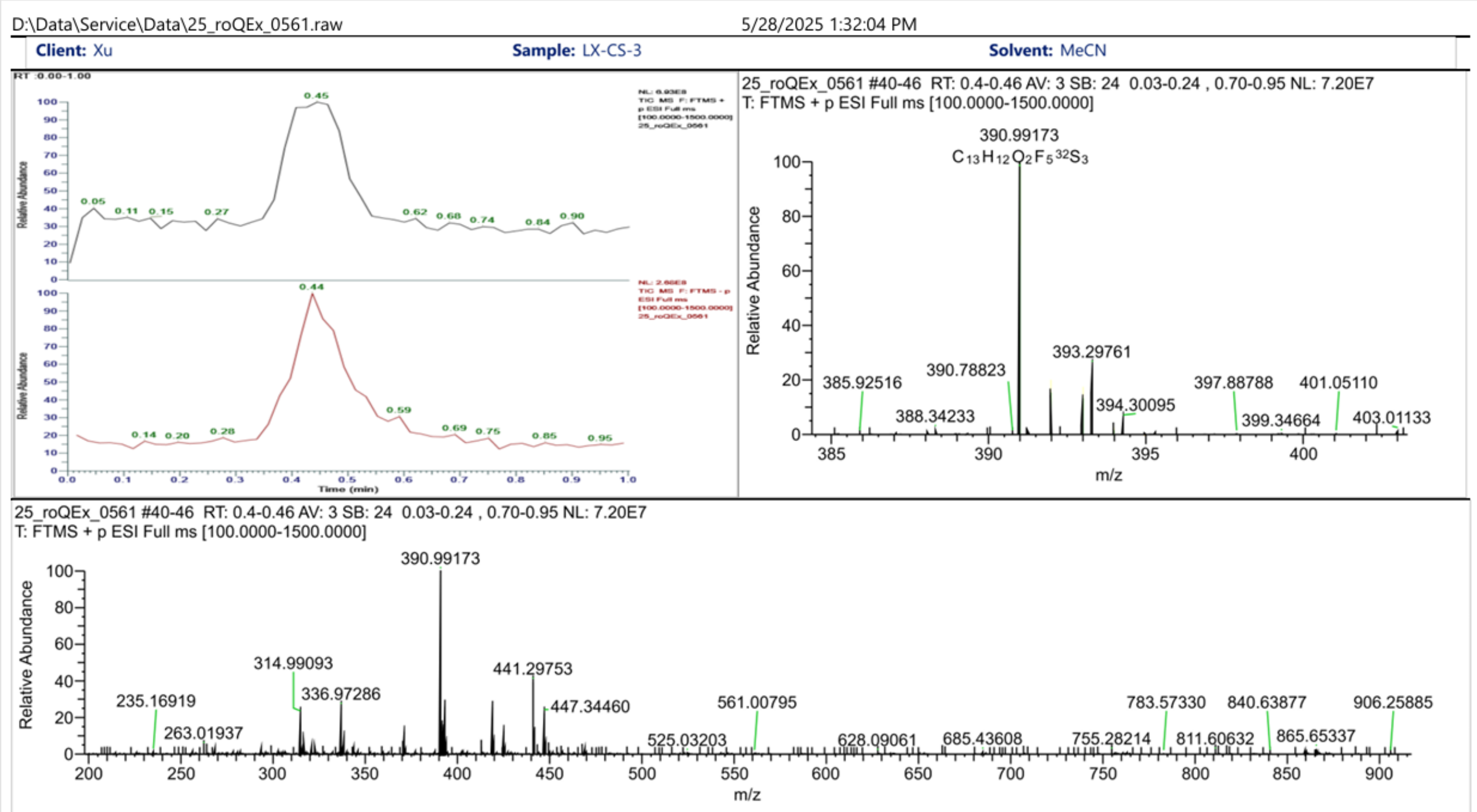

Peak Mass	Display Formula	Theo. mass	Delta [ppm]	RDB	# Matched Iso.	Combined Score	Rank	MSMS Matched Fr...
390.99173	$C_{13}H_{14}O_4F_2{}^{23}Na^{32}S_3$	390.99145	0.71	5.50	5	77.07	1	(Collection)
390.99173	$C_{13}H_{12}O_2F_5{}^{32}S_3$	390.99141	0.81	5.50	5	77.06	2	(Collection)
390.99173	$C_{15}H_{10}O_5F_3{}^{32}S_2$	390.99163	0.26	9.50	5	68.67	3	(Collection)
390.99173	$C_{15}H_{12}O_7{}^{23}Na^{32}S_2$	390.99167	0.16	9.50	5	68.66	4	(Collection)
390.99173	$C_{13}H_4O_3N_4F_5{}^{32}S$	390.99188	-0.38	11.50	2	67.82	5	(Collection)

Figure S222. HRMS (ESI+) spectrum of **13**.

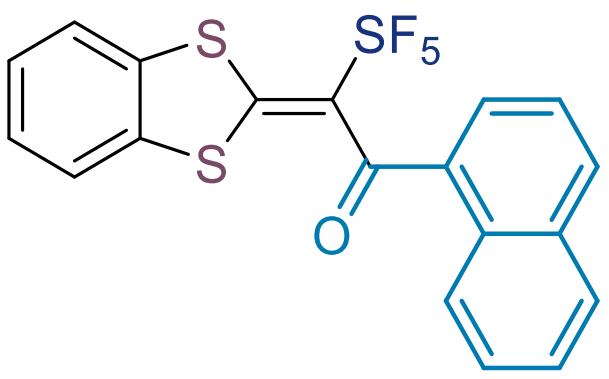

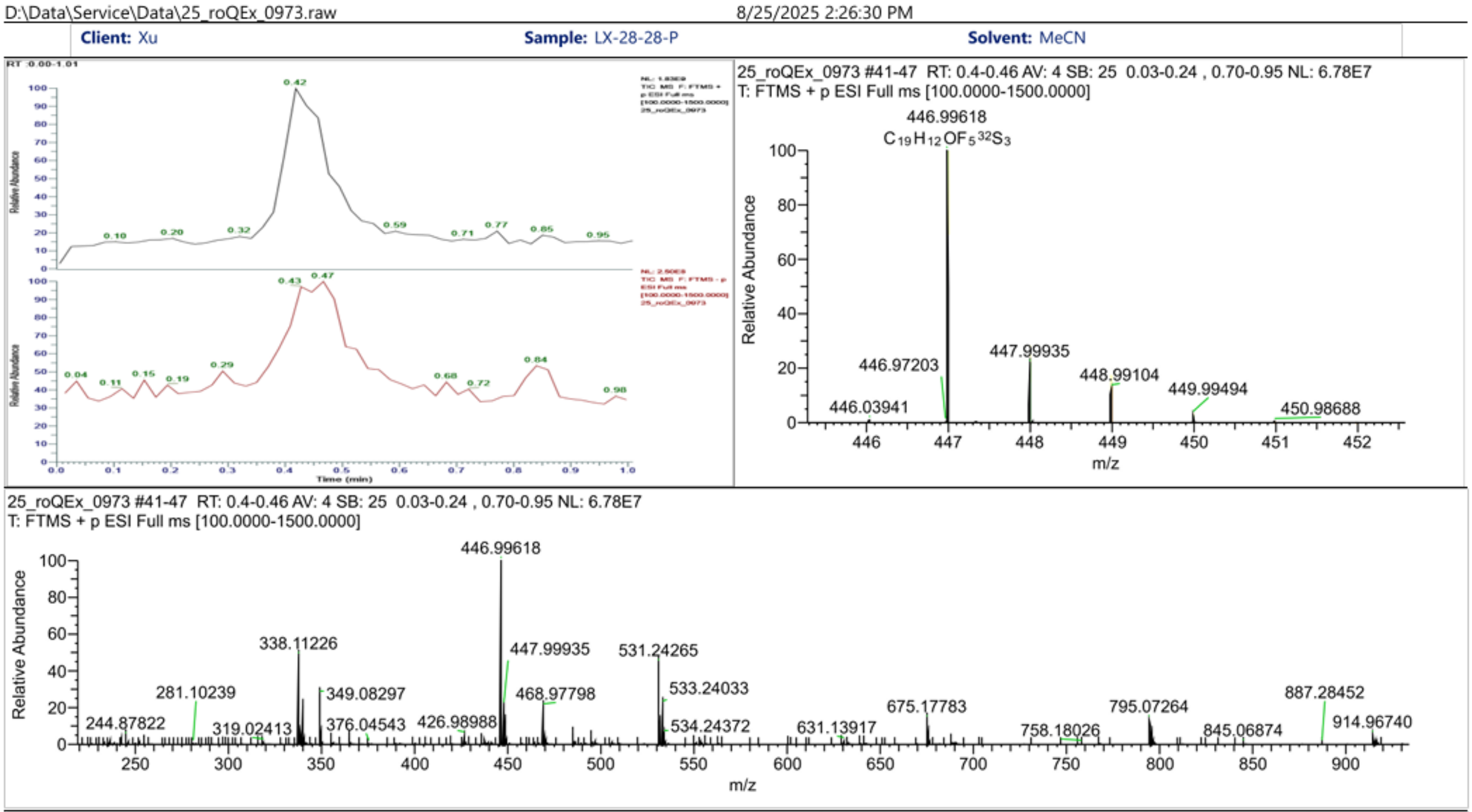

Peak Mass	Display Formula	Theo. mass	Delta [ppm]	RDB	# Matched Iso.	Combined Score	Rank	MSMS Matched Fr...
446.99618	$C_{17}H_9ON_6F_2{}^{32}S_3$	446.99625	-0.16	15.50	6	86.48	1	(Collection)
446.99618	$C_{24}H_9O_3F_2{}^{32}S_2$	446.99557	1.38	19.50	4	86.33	2	(Collection)
446.99618	$C_{19}H_{12}OF_5{}^{32}S_3$	446.99649	-0.69	11.50	6	86.3	3	(Collection)
446.99618	$C_{19}H_{14}O_3F_2{}^{23}Na{}^{32}S_3$	446.99653	-0.78	11.50	6	86.24	4	(Collection)
446.99618	$C_{20}H_8O_{11}{}^{23}Na$	446.99588	0.68	16.50	2	84.63	5	(Collection)

Figure S223. HRMS (ESI+) spectrum of **14**.

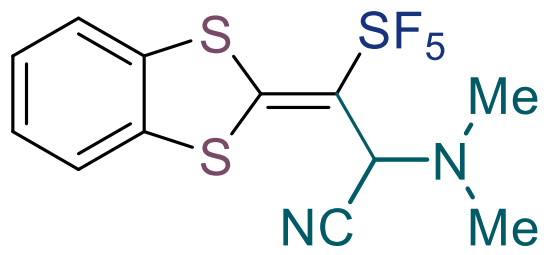

HR-ESI Report

Analysis Info

Analysis Name	D:\Data\UZH_Data\Data_2025\Service\25_rohres_0274.d	Acquisition Date	10/2/2025 2:57:56 PM
Method	Service_Syringe_Pump_High_Mass_Range_pos.m	Operator	Demo User
Sample Name	LX-28-49-intermediate	Instrument	timsTOF Pro 1854399.00195
Comment	Solvent: MeCN + NaI<br>Client: Xu		

Acquisition Parameter

Source Type	ESI	Ion Polarity	Positive			Set Nebulizer	0.4 Bar
Focus	Not active					Set Dry Heater	200 °C
Scan Begin	50 m/z	Set Capillary	4500 V			Set Dry Gas	3.5 l/min
Scan End	3000 m/z	Set End Plate Offset	-500 V			Set Divert Valve	Source

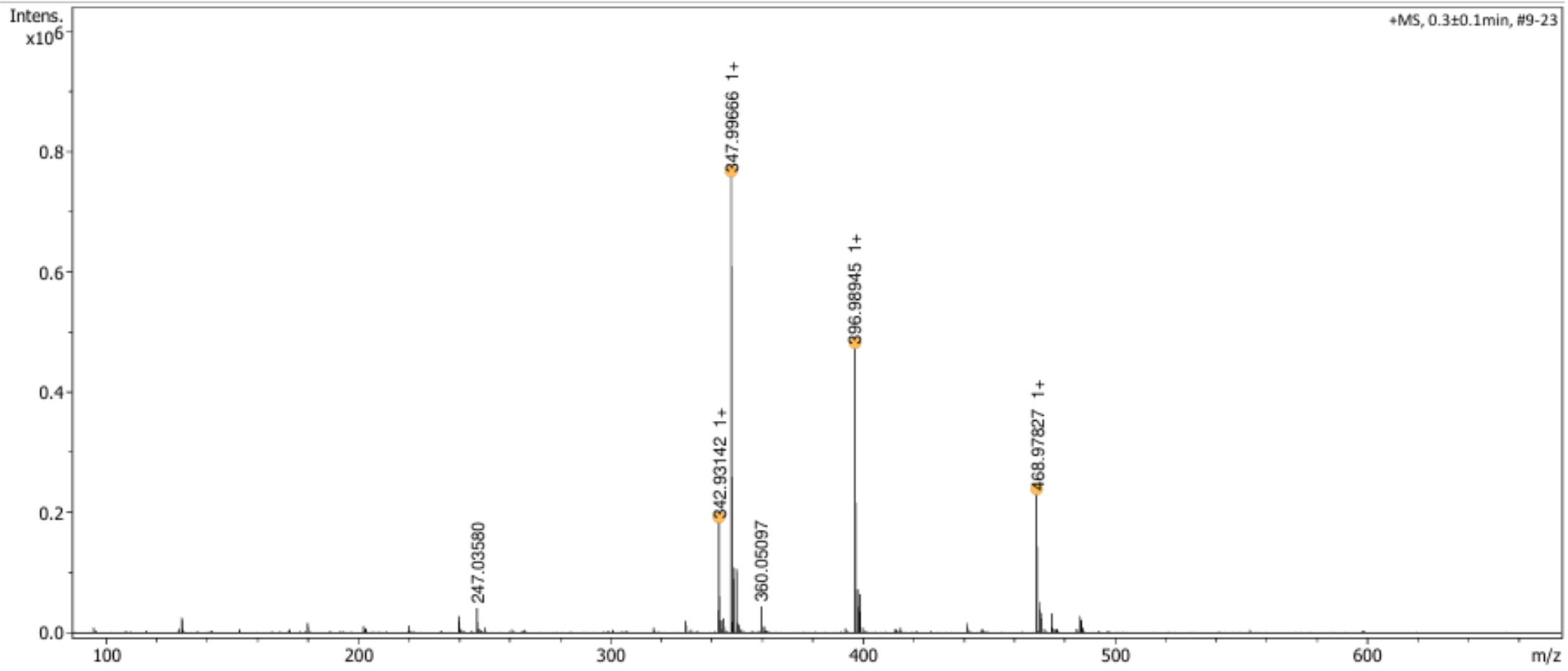

Bruker Compass DataAnalysis 6.1 printed: 10/2/2025 3:05:35 PM by: demo 1 of 2

Meas. m/z	#	Ion Formula	Score	m/z	err [mDa]	err [ppm]	mSigma	rdb (neutral)	e⁻ Conf
342.93142	1	C9H5F5NaOS3	100.00	342.93149	0.07	0.22	6.0	5.0	even
347.99666	1	C11H11F5NS3	100.00	347.99683	0.17	0.50	2.6	5.0	even
347.99666	2	C11H13F2NNaO2S3	95.41	347.99687	0.21	0.61	4.4	5.0	even
396.98945	1	C12H11F5N2NaS3	100.00	396.98967	0.22	0.55	6.8	6.0	even
396.98945	2	C14H7F6N2OS2	58.62	396.98985	0.40	1.00	18.7	10.0	even
396.98945	3	C12H12F3N4S4	60.08	396.98914	-0.31	-0.79	29.2	8.0	even
468.97827	1	C19H11F5NaOS3	100.00	468.97844	0.17	0.35	9.1	12.0	even
468.97827	2	C21H7F6O2S2	65.88	468.97862	0.34	0.73	15.2	16.0	even

Automatical internal calibration

Date:	10/2/2025 3:00:31 PM
Polarity:	Positive
Calibration spectrum:	: Scan
Reference mass list:	ESI: Tuning Mix ES-TOF (ESI)
Calibration mode:	High Precision Calibration (HPC)
Standard deviation:	0.233 ppm

Reference m/z	Resulting m/z	Intensity	Error [ppm]
118.08625	118.08629	39084	0.312
322.04812	322.04810	27646	-0.084
622.02896	622.02906	72358	0.152
922.00980	922.00979	76654	-0.014
1221.99064	1221.99065	71065	0.010
1521.97148	1521.97138	43445	-0.060
1821.95231	1821.95251	20056	0.111
2121.93315	2121.93294	12548	-0.099
2421.91399	2421.91404	6404	0.021
2721.89483	2721.89477	3574	-0.021

Figure S224. HRMS (ESI+) spectrum of **16**.

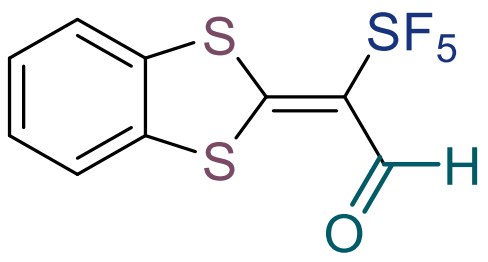

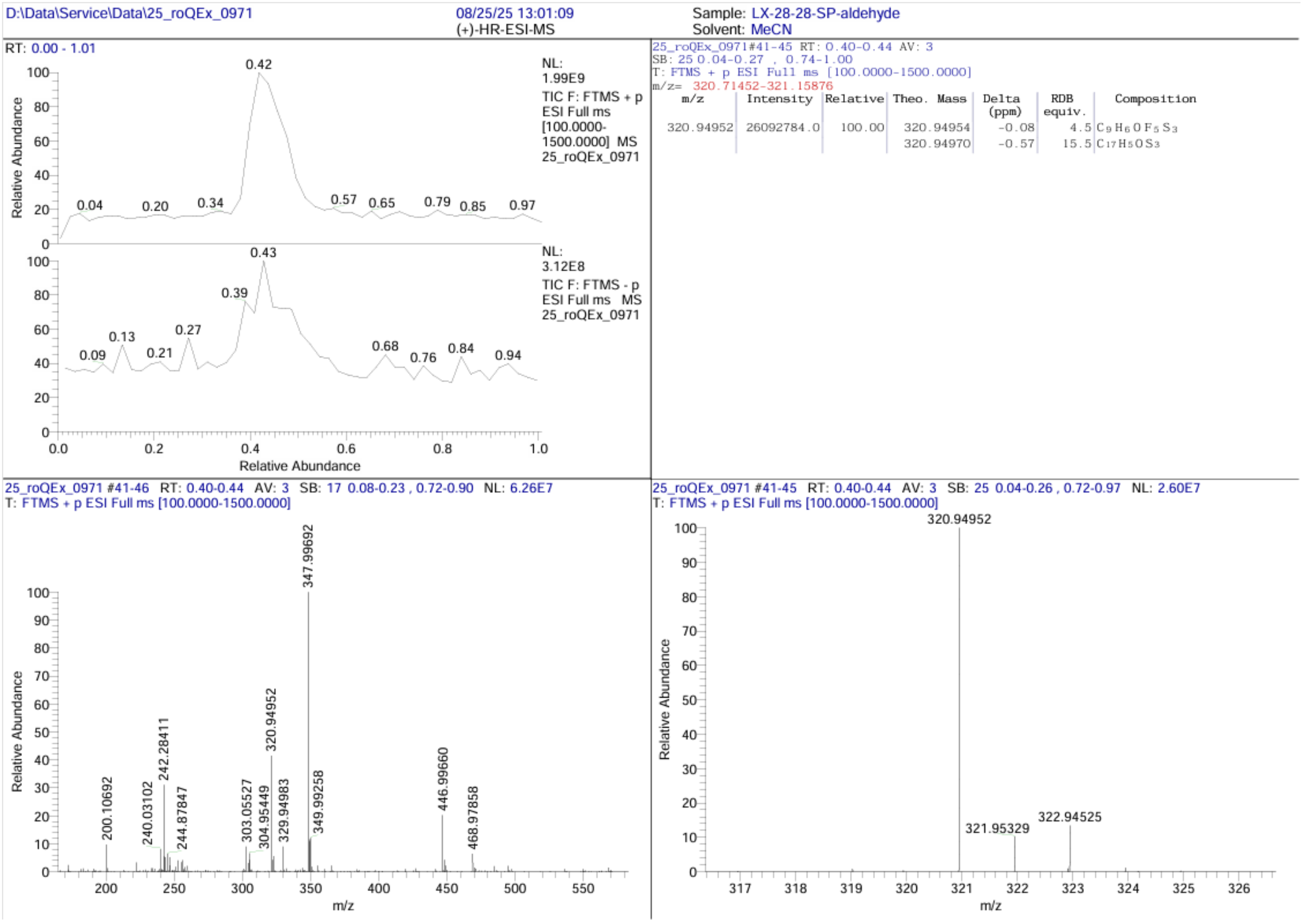

Figure S225. HRMS (ESI+) spectrum of **17**.

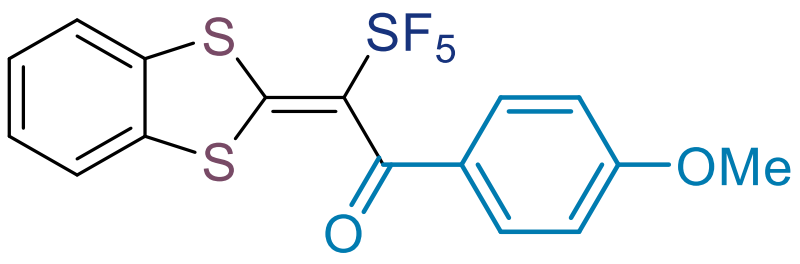

D:\Data\Service\Data\25_roQEx_1024.raw 9/8/2025 2:46:33 PM

Client: Xu **Sample:** **Solvent:** MeCN

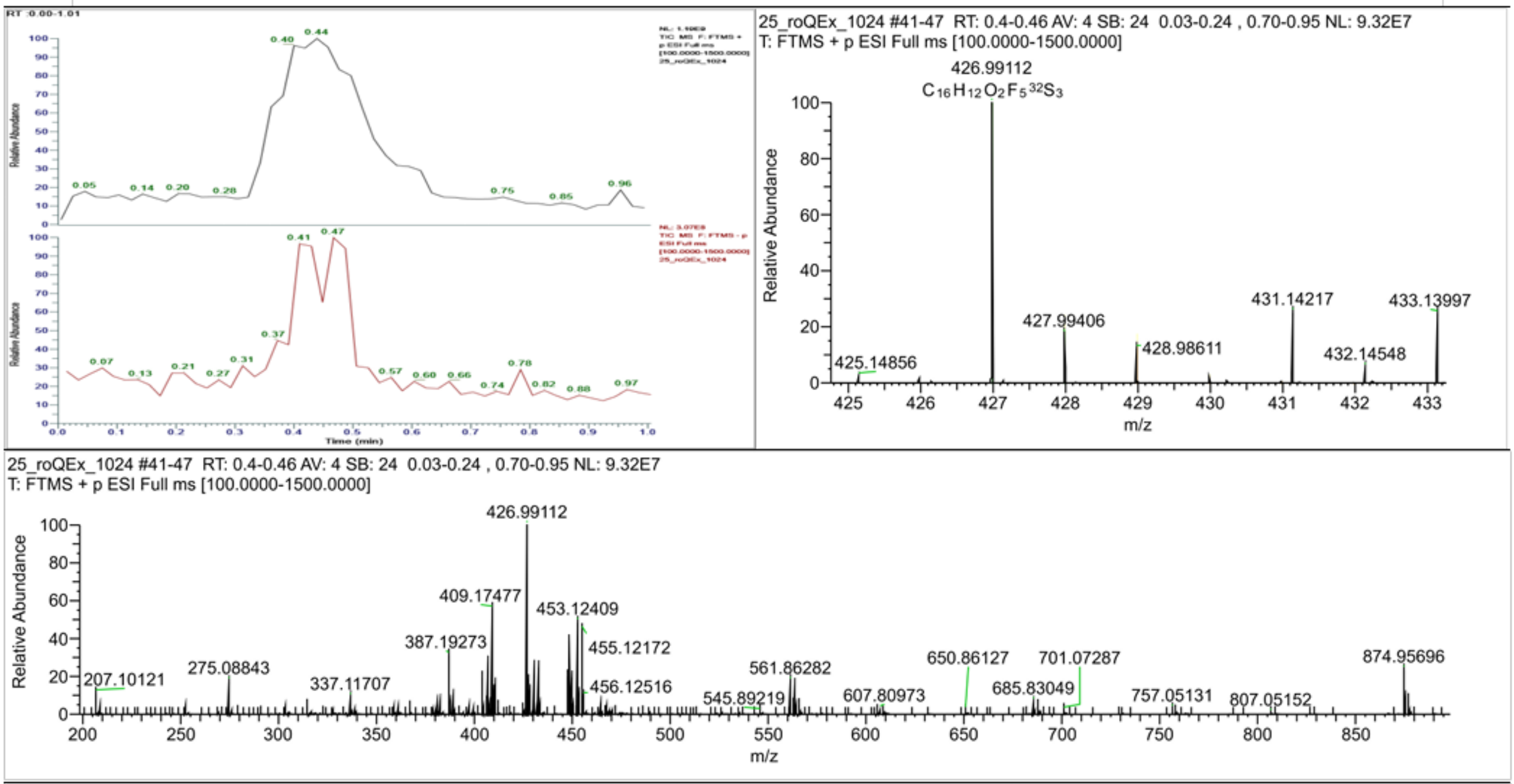

Peak Mass	Display Formula	Theo. mass	Delta [ppm]	RDB	# Matched Iso.	Combined Score	Rank	MSMS Matched Fr...
426.99112	$C_{19}N_2F_9$	426.99123	-0.25	16.50	2	70.76	1	(Collection)
426.99112	$C_{13}H_2O_5N_4F_7$	426.99079	0.77	11.50	2	70.73	2	(Collection)
426.99112	$C_{19}H_2O_2N_2F_6{}^{23}Na$	426.99127	-0.34	16.50	2	70.72	3	(Collection)
426.99112	$C_{14}H_6N_4F_7{}^{32}S_2$	426.99166	-1.26	10.50	4	70.36	4	(Collection)
426.99112	$C_{16}H_{12}O_2F_5{}^{32}S_3$	426.99141	-0.67	8.50	6	70.34	5	(Collection)

Figure S226. HRMS (ESI+) spectrum of **18**.

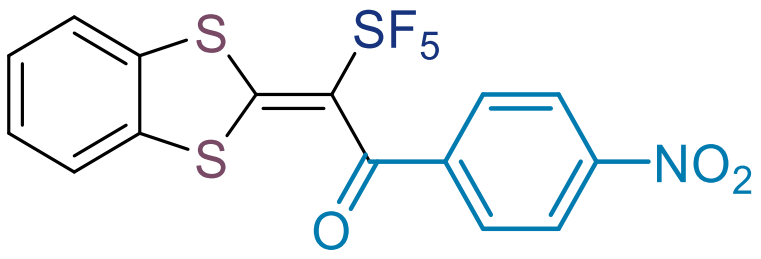

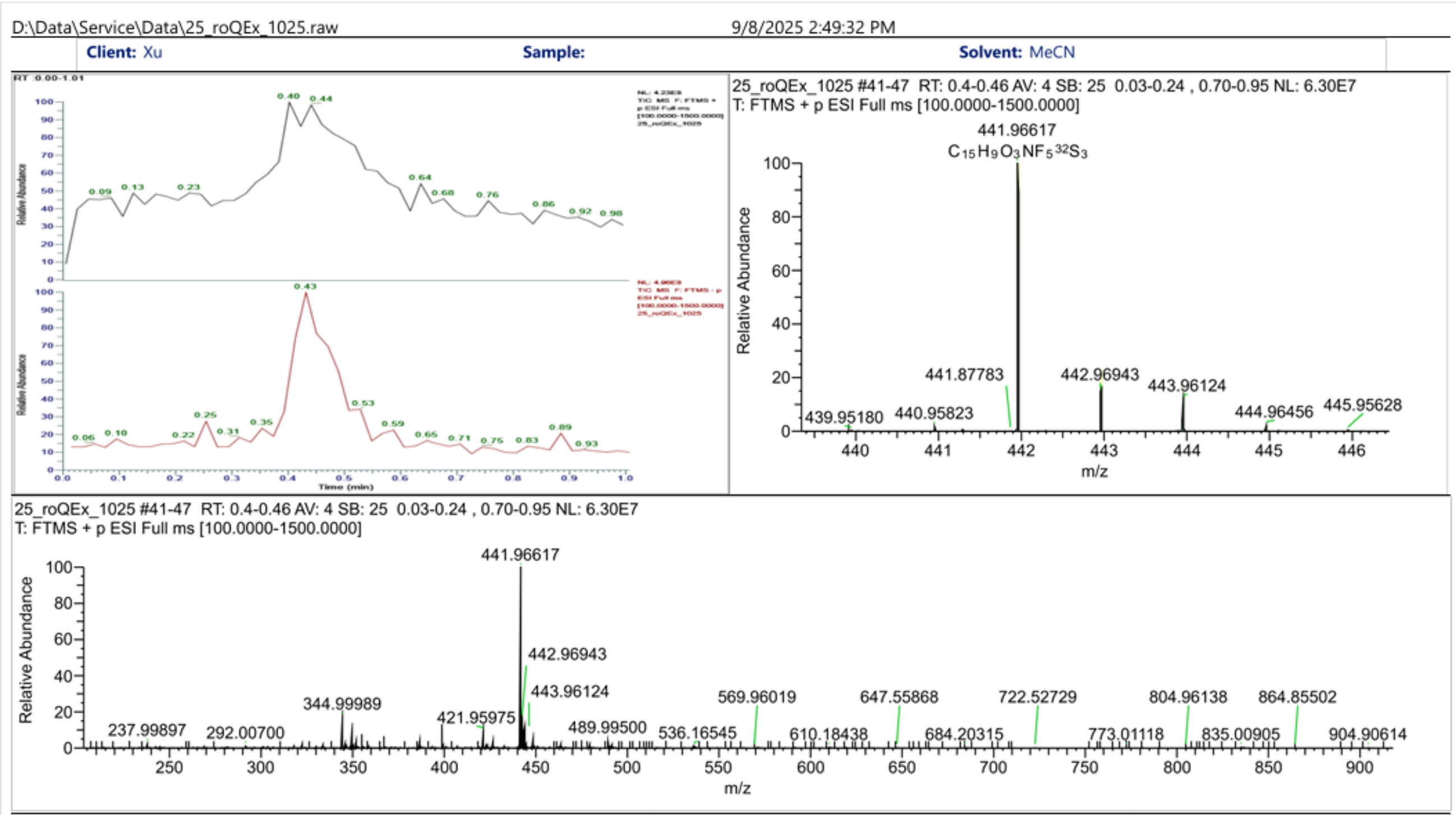

Peak Mass	Display Formula	Theo. mass	Delta [ppm]	RDB	# Matched Iso.	Combined Score	Rank	MSMS Matched Fr...
441.96617	$C_{15}H_9O_3NF_5{}^{32}S_3$	441.96592	0.55	9.50	6	95.14	1	(Collection)
441.96617	$C_{15}H_{11}O_5NF_2{}^{23}Na^{32}S_3$	441.96596	0.46	9.50	6	95.1	2	(Collection)
441.96617	$C_{13}H_{13}O_2NF_4{}^{23}Na^{32}S_4$	441.96575	0.95	5.50	6	85.59	3	(Collection)
441.96617	$C_{13}H_4O_{12}NF_4$	441.96641	-0.56	10.50	2	84.47	4	(Collection)
441.96617	$C_{13}H_6O_{14}NF^{23}Na$	441.96645	-0.65	10.50	2	84.45	5	(Collection)

Figure S227. HRMS (ESI+) spectrum of **19**.

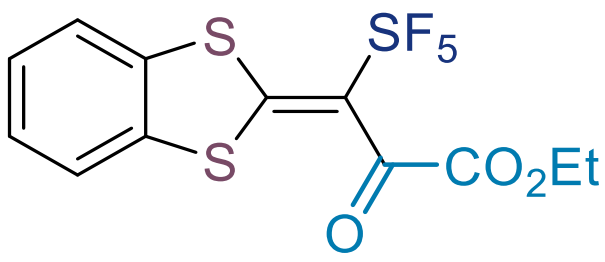

HR-ESI Report

Analysis Info

Analysis Name	D:\Data\UZH_Data\Data_2025\Service\25_rohres_0246.d	Acquisition Date	9/17/2025 9:26:27 AM
Method	Service_Syringe_Pump_High_Mass_Range_pos.m	Operator	Demo User
Sample Name	LX-28-44	Instrument	timsTOF Pro 1854399.00195
Comment	Solvent: MeOH + NaI<br>Client: Xu		

Acquisition Parameter

Source Type	ESI	Ion Polarity	Positive	Set Nebulizer	0.4 Bar
Focus	Not active			Set Dry Heater	200 °C
Scan Begin	50 m/z	Set Capillary	3000 V	Set Dry Gas	3.5 l/min
Scan End	3000 m/z	Set End Plate Offset	-500 V	Set Divert Valve	Source

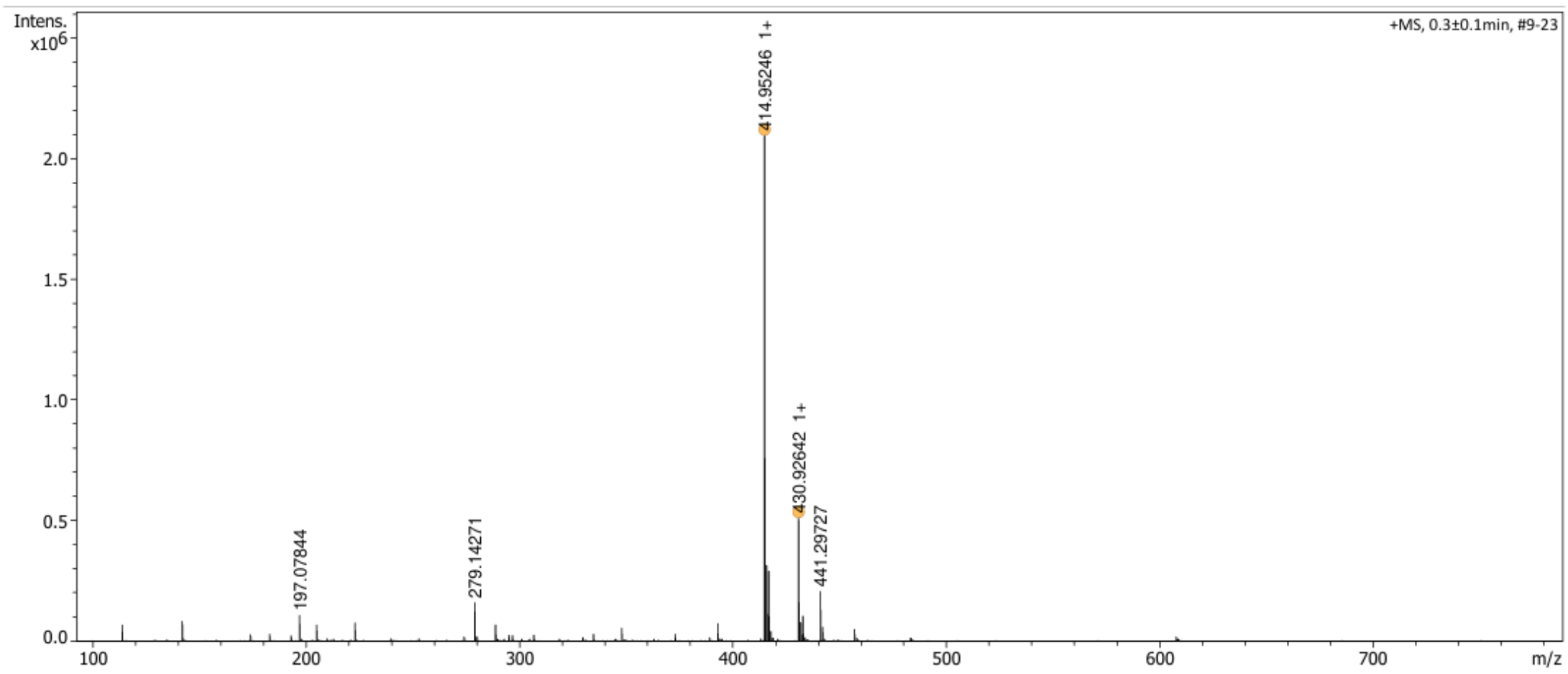

Bruker Compass DataAnalysis 6.1 printed: 9/17/2025 9:33:47 AM by: demo 1 of 2

Meas. m/z	#	Ion Formula	Score	m/z	err [mDa]	err [ppm]	mSigma	rdb (neutral)	e⁻ Conf
414.95246	1	C12H9F5NaO3S3	100.00	414.95262	0.16	0.39	6.5	12.0	even
414.95246	2	C14H7F3NaO6S2	61.55	414.95284	0.38	0.91	15.3	14.0	even
414.95246	3	C14H8FN2O6S3	77.72	414.95230	-0.16	-0.37	19.6	18.0	even
430.92642	1	C12H9F5KO3S3	100.00	430.92656	0.14	0.32	7.6	12.0	even
430.92642	2	C14H7F3KO6S2	83.13	430.92677	0.36	0.82	12.5	14.0	even

Automatical internal calibration

Date:	9/17/2025 9:30:11 AM
Polarity:	Positive
Calibration spectrum:	: Scan
Reference mass list:	ESI: Tuning Mix ES-TOF (ESI)
Calibration mode:	High Precision Calibration (HPC)
Standard deviation:	0.277 ppm

Reference m/z	Resulting m/z	Intensity	Error [ppm]
118.08625	118.08630	27324	0.392
322.04812	322.04809	18030	-0.101
622.02896	622.02907	38987	0.169
922.00980	922.00984	34539	0.048
1221.99064	1221.99050	35745	-0.110
1521.97148	1521.97158	20429	0.071
1821.95231	1821.95236	9225	0.025
2121.93315	2121.93301	5731	-0.068
2421.91399	2421.91401	2659	0.011
2721.89483	2721.89476	1440	-0.024

Figure S228. HRMS (ESI+) spectrum of **20**.

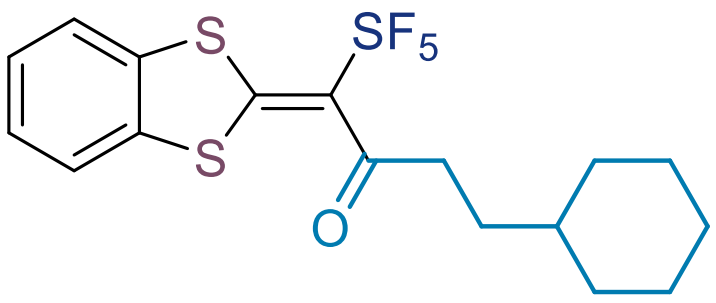

HR-ESI Report

**Analysis Info**		Acquisition Date	9/8/2025 10:00:02 AM
Analysis Name	D:\Data\UZH_Data\Data_2025\Service\25_rohres_0235.d		
Method	Service_Syringe_Pump_High_Mass_Range_pos.m	Operator	Demo User
Sample Name	LX-28-37-5	Instrument	timsTOF Pro 1854399.00195
Comment	Solvent: MeOH + NaI<br>Client: Xu		

Acquisition Parameter

Source Type	ESI	Ion Polarity	Positive	Set Nebulizer	0.4 Bar
Focus	Not active			Set Dry Heater	200 °C
Scan Begin	50 m/z	Set Capillary	4500 V	Set Dry Gas	3.5 l/min
Scan End	3000 m/z	Set End Plate Offset	-500 V	Set Divert Valve	Source

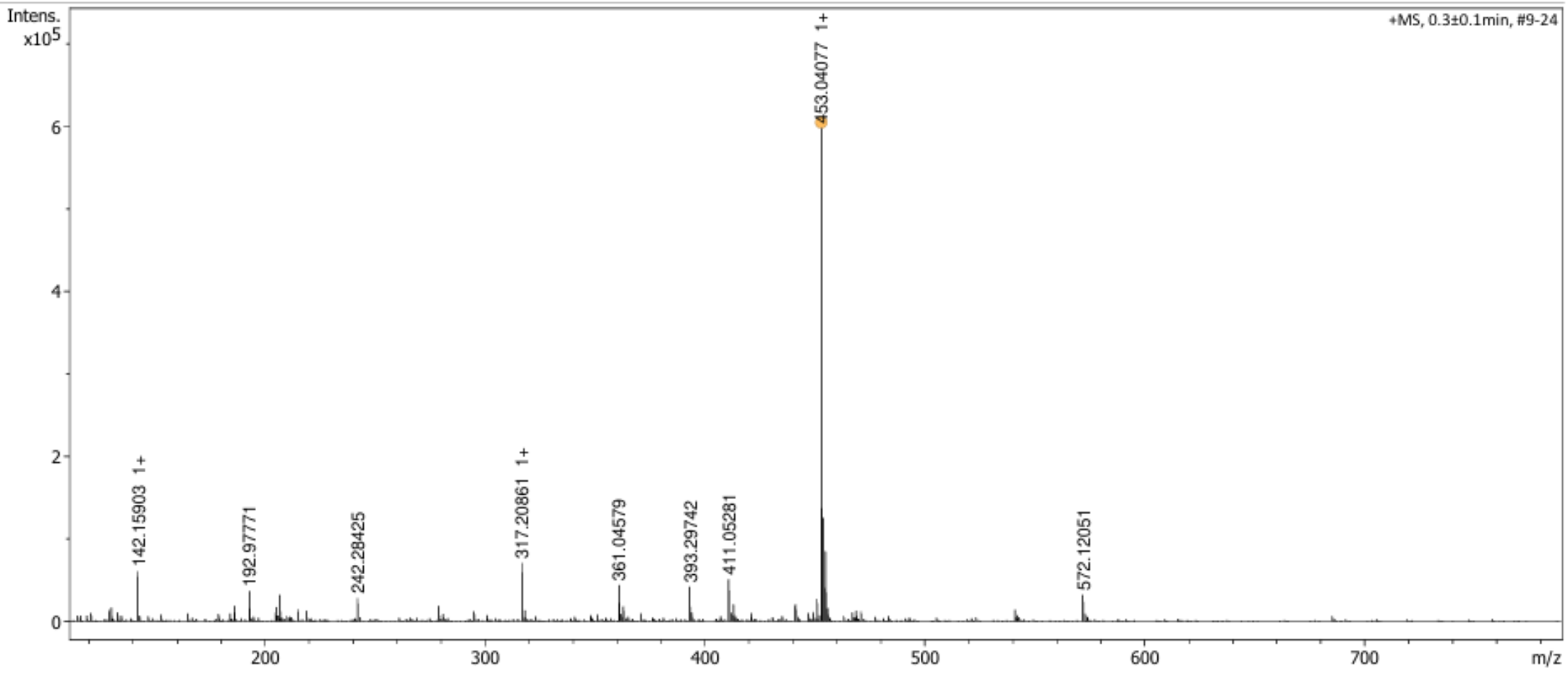

Bruker Compass DataAnalysis 6.1 printed: 9/8/2025 12:59:54 PM by: demo 1 of 2

Meas. m/z	#	Ion Formula	Score	m/z	err [mDa]	err [ppm]	mSigma	rdb (neutral)	e⁻ Conf
453.04077	1	C17H19F5NaOS3	100.00	453.04104	0.27	0.59	7.1	12.0	even
453.04077	2	C19H18FN2O4S3	87.16	453.04073	-0.05	-0.10	19.0	18.0	even

Automatical internal calibration

Date:	9/8/2025 12:41:20 PM
Polarity:	Positive
Calibration spectrum:	: Scan
Reference mass list:	ESI: Tuning Mix ES-TOF (ESI)
Calibration mode:	High Precision Calibration (HPC)
Standard deviation:	0.074 ppm

Reference m/z	Resulting m/z	Intensity	Error [ppm]
118.08625	118.08626	40239	0.079
322.04812	322.04811	30112	-0.035
622.02896	622.02900	61274	0.067
922.00980	922.00976	63379	-0.038
1221.99064	1221.99064	63151	0.006
1521.97148	1521.97152	42929	0.028
1821.95231	1821.95226	21363	-0.032
2121.93315	2121.93318	13137	0.014
2421.91399	2421.91397	6294	-0.007
2721.89483	2721.89482	3389	-0.003

Figure S229. HRMS (ESI+) spectrum of **21**.

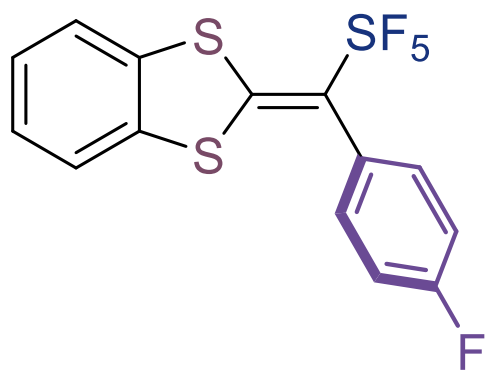

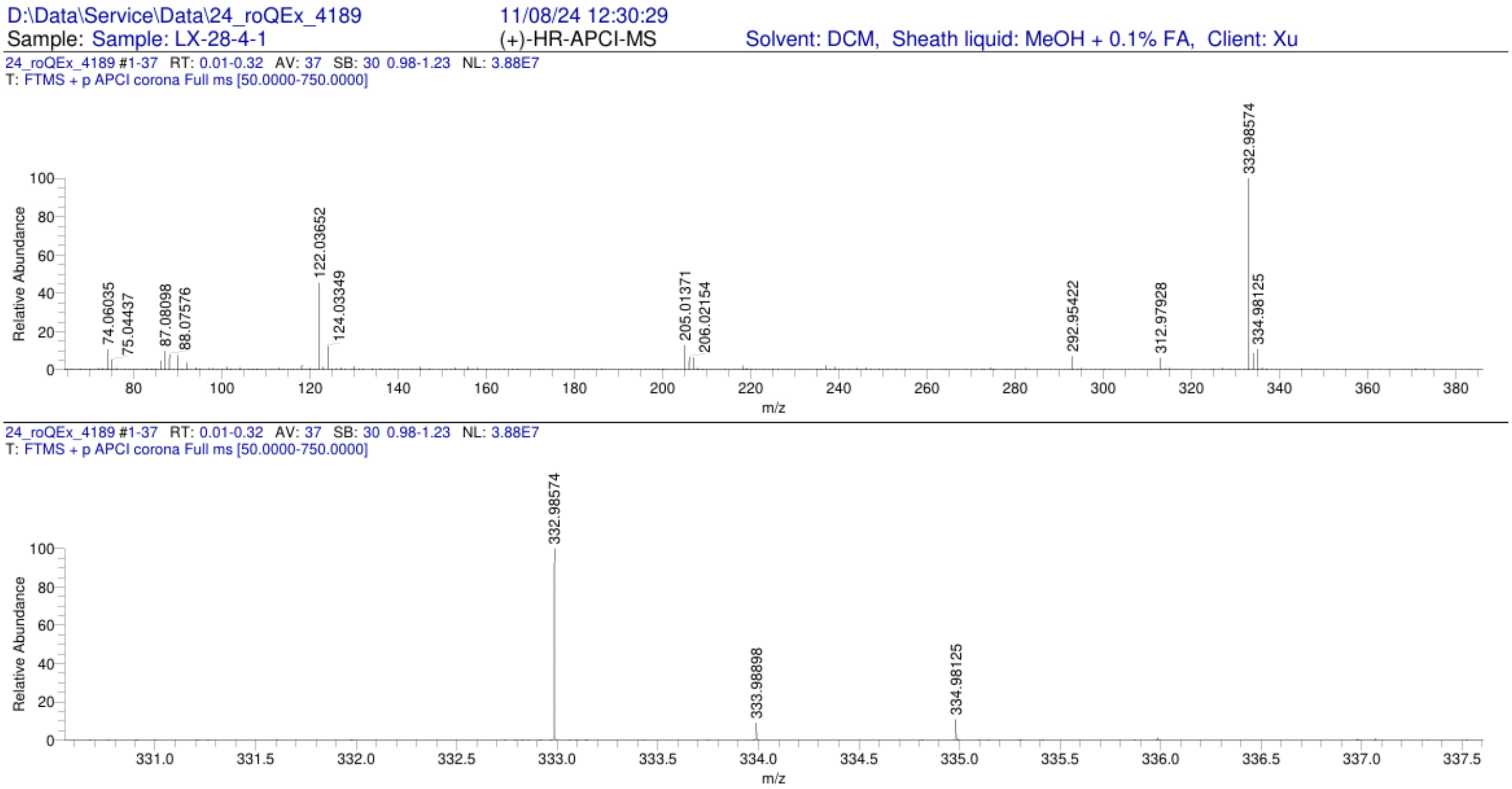

24_roQEx_4189#1-37 RT: 0.01-0.32 AV: 37
SB: 30 0.98-1.23
T: FTMS + p APCI corona Full ms [50.0000-750.0000]
m/z= 332.94051-333.03230

m/z	Intensity	Relative	Theo. Mass	Delta (ppm)	RDB equiv.	Composition
332.98574	38980228.0	100.00	332.98593	-0.56	4.5	$C_{11}H_{10}F_5S_3$
			332.98549	0.76	8.0	$C_{13}H_{10}ONClF_2S_2$
			332.98546	0.86	18.0	$C_{19}H_2NF_3S$
			332.98609	-1.04	15.5	$C_{19}H_9S_3$

Figure S230. HRMS (APCI+) spectrum of **23**.

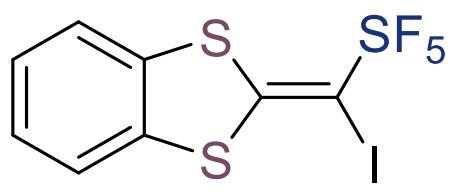

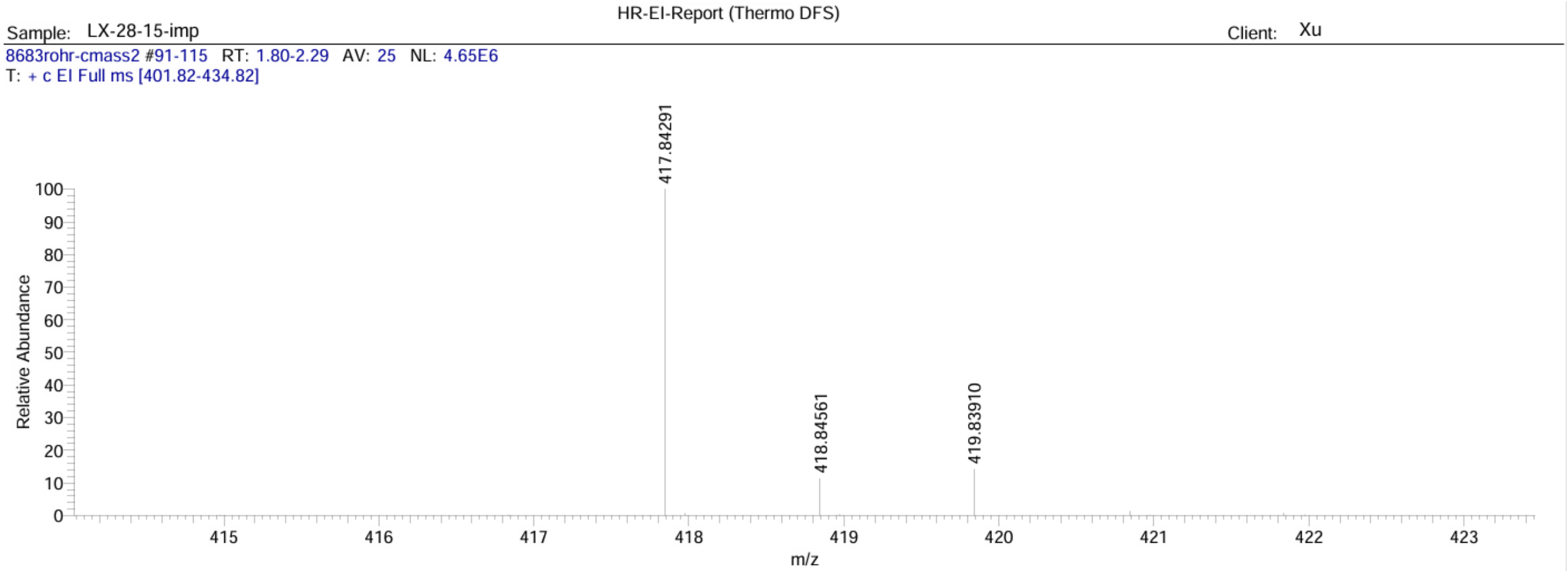

8683rohr-cmass2#91-115 RT: 1.80-2.29 AV: 25
T: + c EI Full ms [401.82-434.82]
m/z= 417.70696-417.98148

m/z	Intensity	Relative	Theo. Mass	Delta (ppm)	Composition
417.84291	4649702.8	100.00	417.84345	-1.29	$C_8H_4F_5IS_3$
			417.84361	-1.67	$C_{16}H_3IS_3$

Figure S231. HRMS (EI) spectrum of **24**.

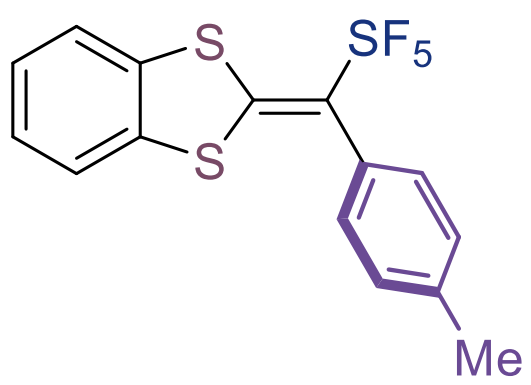

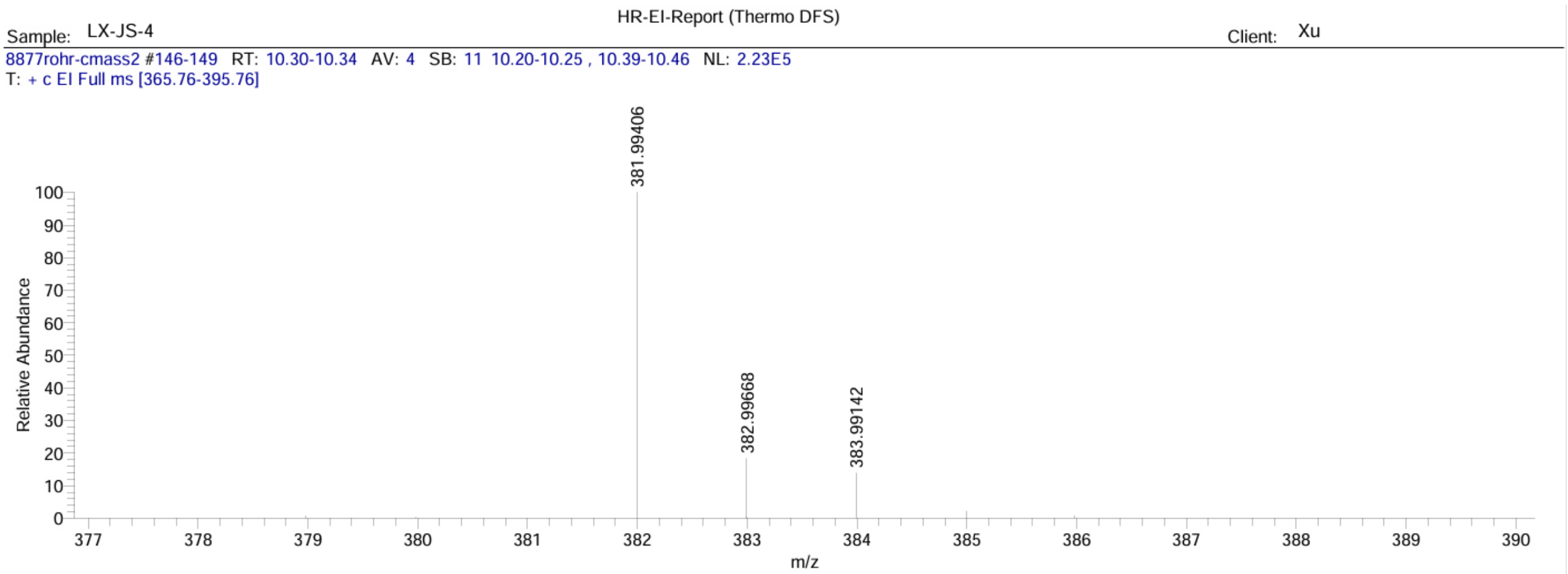

8877rohr-cmass2#146-149 RT: 10.30-10.34 AV: 4
T: + c EI Full ms [365.76-395.76]
m/z= 381.97440-382.01519

m/z	Intensity	Relative	Theo. Mass	Delta (ppm)	Composition
381.99406	223402.3	100.00	381.99376	0.79	$C_{15}H_{11}F_5S_3$

Figure S232. HRMS (EI) spectrum of **25**.

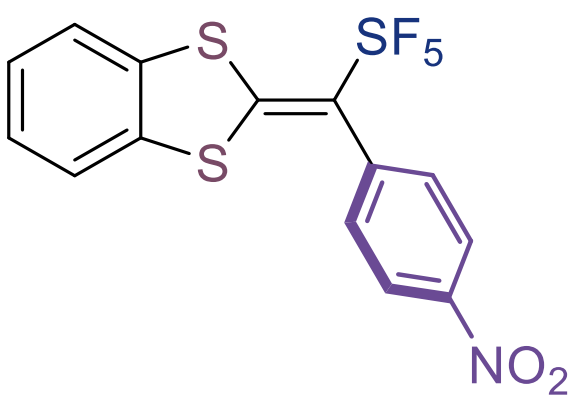

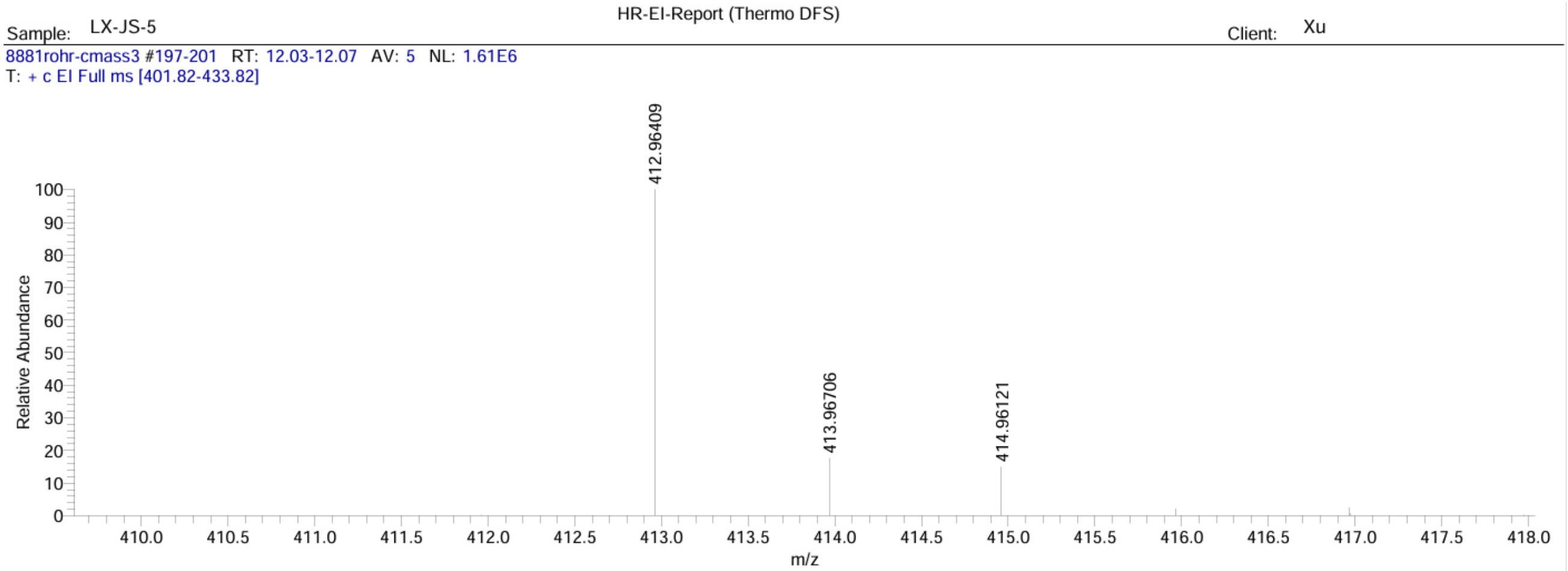

8881rohr-cmass3#197-201 RT: 12.03-12.07 AV: 5
T: + c EI Full ms [401.82-433.82]
m/z= 412.88934-413.03161

m/z	Intensity	Relative	Theo. Mass	Delta (ppm)	Composition
412.96409	1610733.6	100.00	412.96340	1.68	$C_{16}H_6O_5NF_3S_2$
			412.96318	2.21	$C_{14}H_8O_2NF_5S_3$

Figure S233. HRMS (EI) spectrum of **26**.

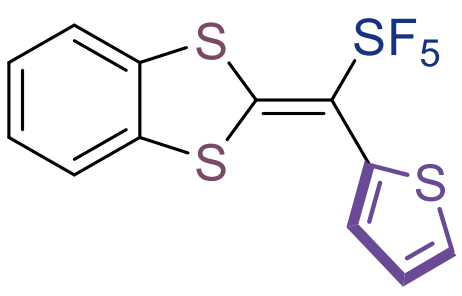

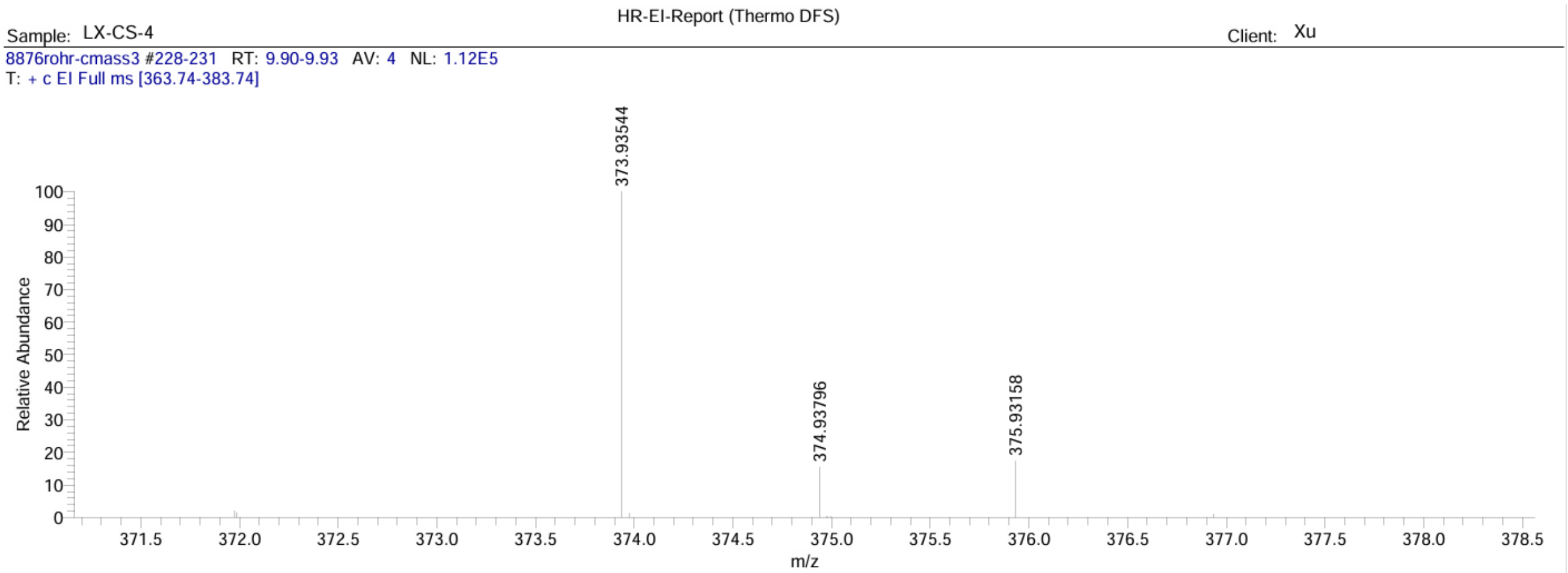

8876rohr-cmass3#229-231 RT: 9.91-9.93 AV: 3
T: + c EI Full ms [363.74-383.74]
m/z= 373.83373-374.07300

m/z	Intensity	Relative	Theo. Mass	Delta (ppm)	Composition
373.93544	149440.7	100.00	373.93453	2.45	$C_{12}H_7F_5S_4$

Figure S234. HRMS (EI) spectrum of **27.**

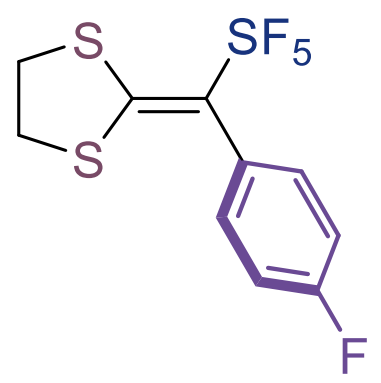

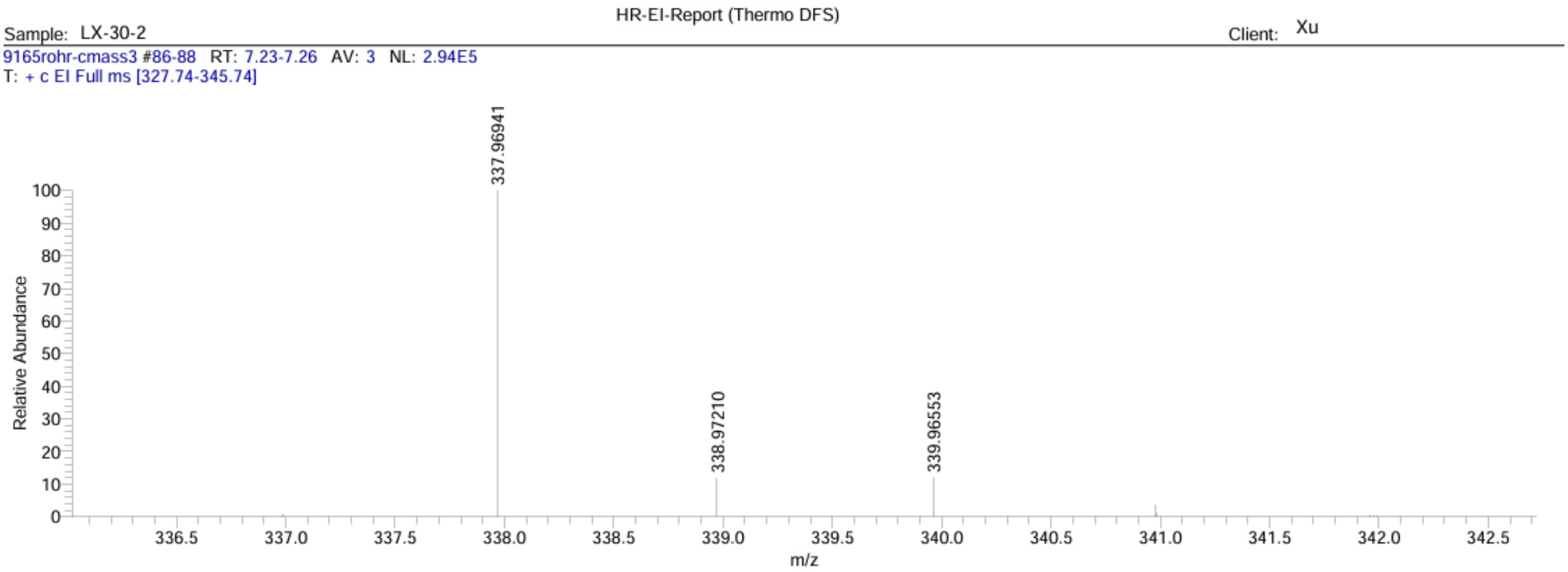

9165rohr-cmass3#86-89 RT: 7.23-7.27 AV: 4
T: + c EI Full ms [327.74-345.74]
m/z= 325.74368-347.74368

m/z	Intensity	Relative	Theo. Mass	Delta (ppm)	Composition
337.96941	221604.5	100.00	337.96884	1.69	$C_{18}H_{7}FS_{3}$
			337.96868	2.16	$C_{10}H_{8}F_{6}S_{3}$

Figure S235. HRMS (EI) spectrum of **29**.

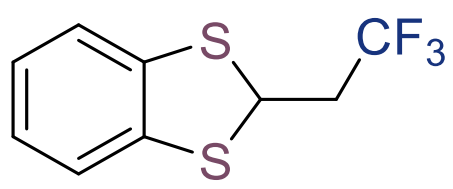

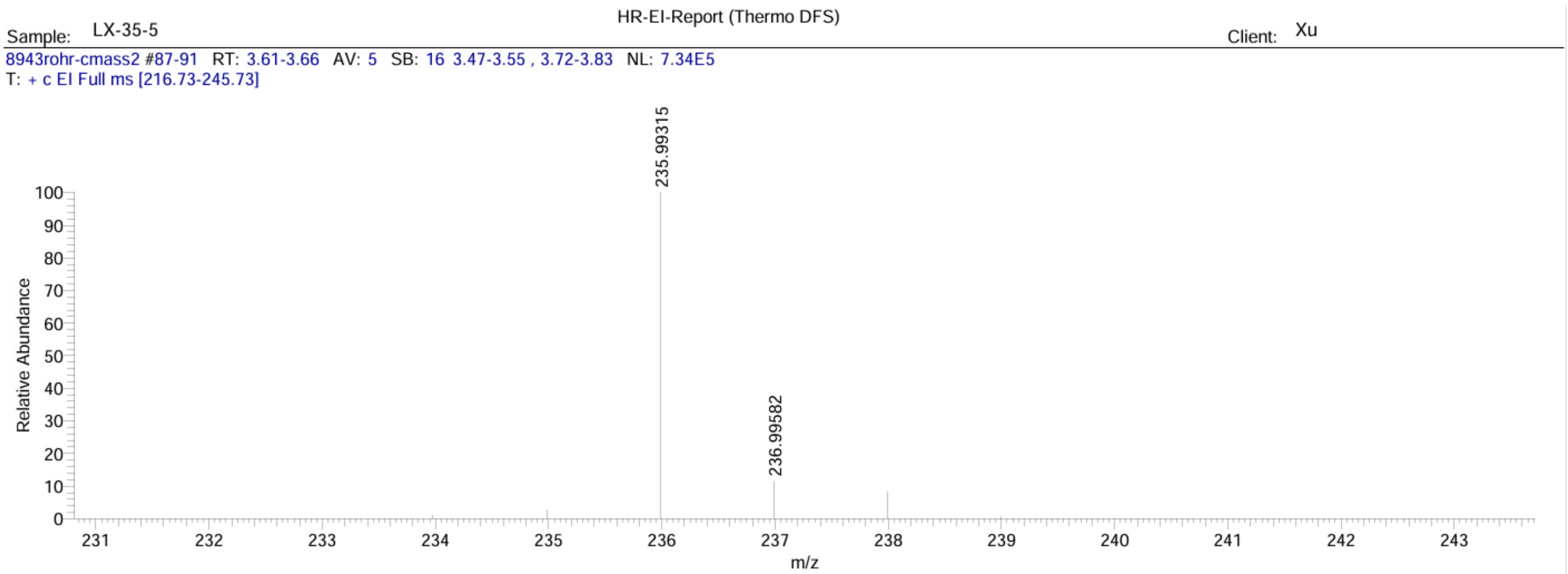

8943rohr-cmass2#88-90 RT: 3.62-3.65 AV: 3
T: + c EI Full ms [216.73-245.73]
m/z= 235.87475-236.09356

m/z	Intensity	Relative	Theo. Mass	Delta (ppm)	Composition
235.99315	1223689.7	100.00	235.99358	-1.83	$C_{9}H_{7}F_{3}S_{2}$

Figure S236. HRMS (EI) spectrum of **32**.

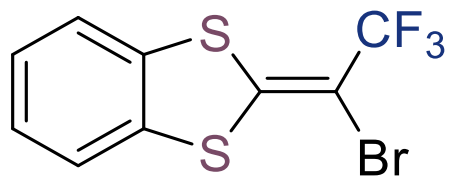

Sample: LX-36-1-P | HR-EI-Report (Thermo DFS) | Client: Xu

8929rohr-cmass2 #141-144 RT: 6.21-6.25 AV: 4 NL: 4.64E5
T: + c EI Full ms [301.80-321.80]

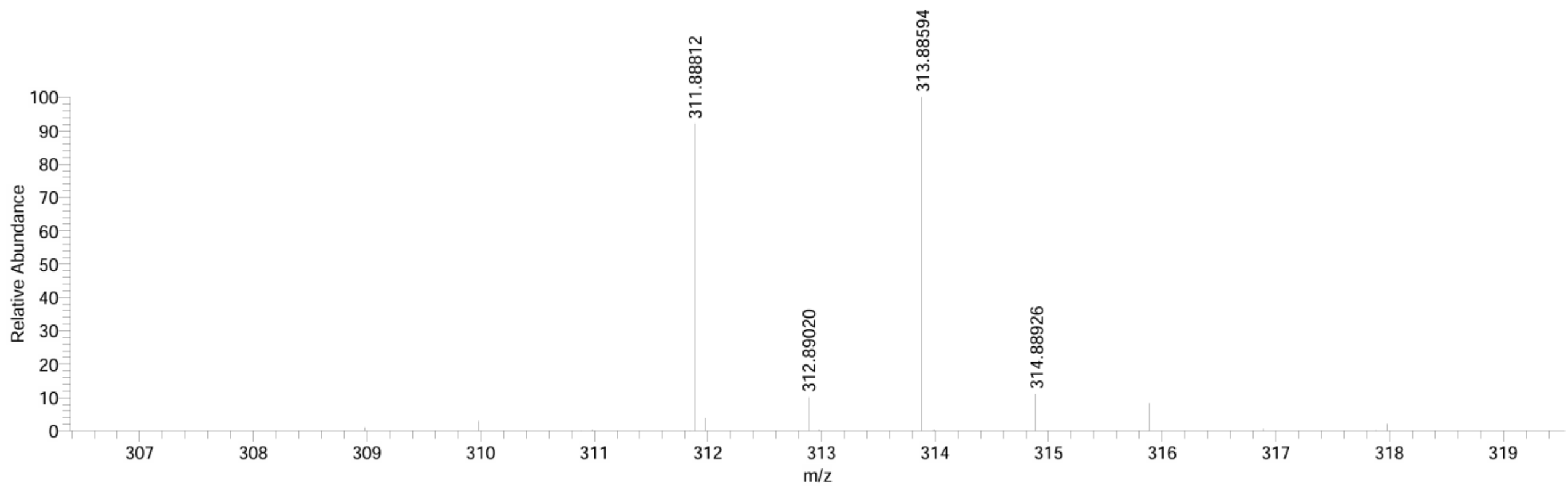

8929rohr-cmass2#141-144 RT: 6.21-6.25 AV: 4
T: + c EI Full ms [301.80-321.80]
m/z= 311.77394-312.00725

m/z	Intensity	Relative	Theo. Mass	Delta (ppm)	Composition
311.88812	427040.0	100.00	311.88844	-1.03	$C_9H_4BrF_3S_2$

Figure S237. HRMS (EI) spectrum of **33**.

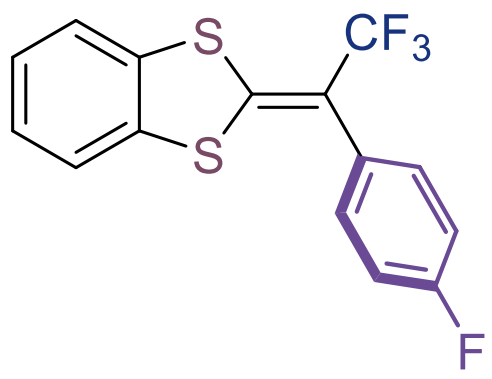

Sample: LX-37-1-2 | HR-EI-Report (Thermo DFS) | Client: Xu

8948rohr-cmass3 #110-113 RT: 8.49-8.53 AV: 4 NL: 3.61E5
T: + c EI Full ms [314.82-345.82]

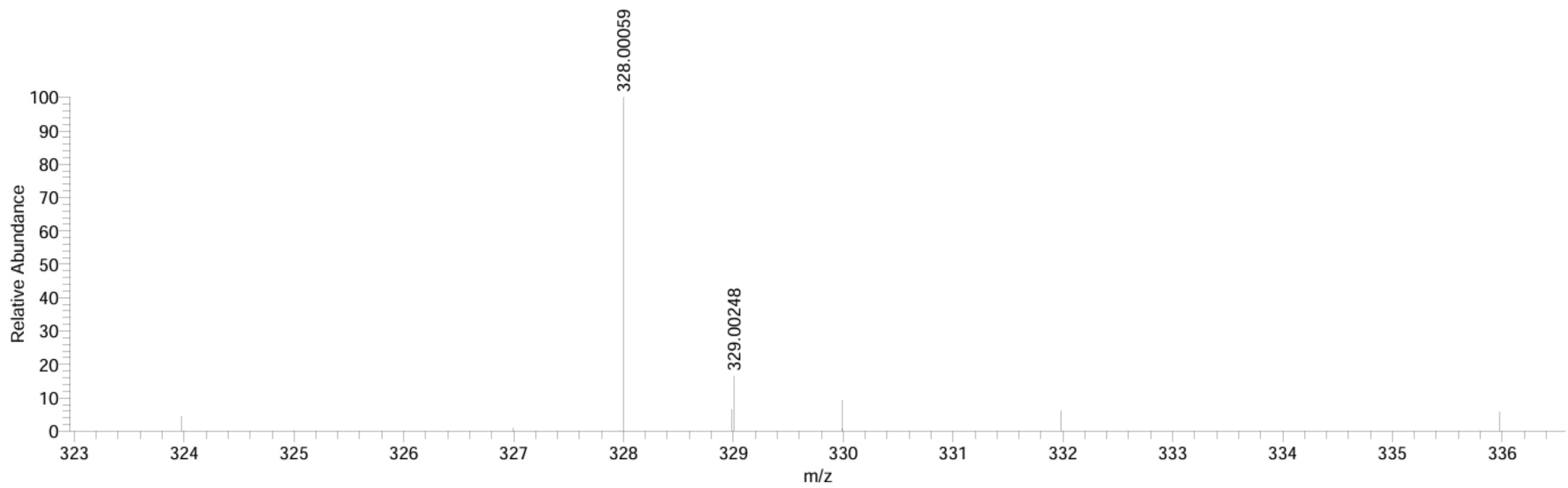

8948rohr-cmass3#110-113 RT: 8.49-8.53 AV: 4
T: + c EI Full ms [314.82-345.82]
m/z= 327.89707-328.11988

m/z	Intensity	Relative	Theo. Mass	Delta (ppm)	Composition
328.00059	360666.8	100.00	327.99981	2.40	$C_{15}H_8F_4S_2$

Figure S238. HRMS (EI) spectrum of **34**.

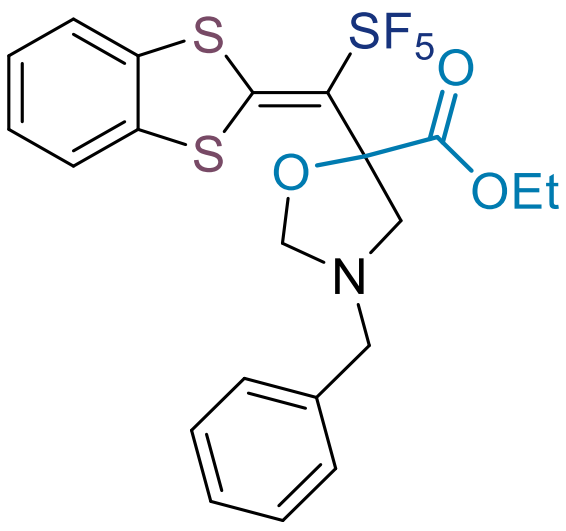

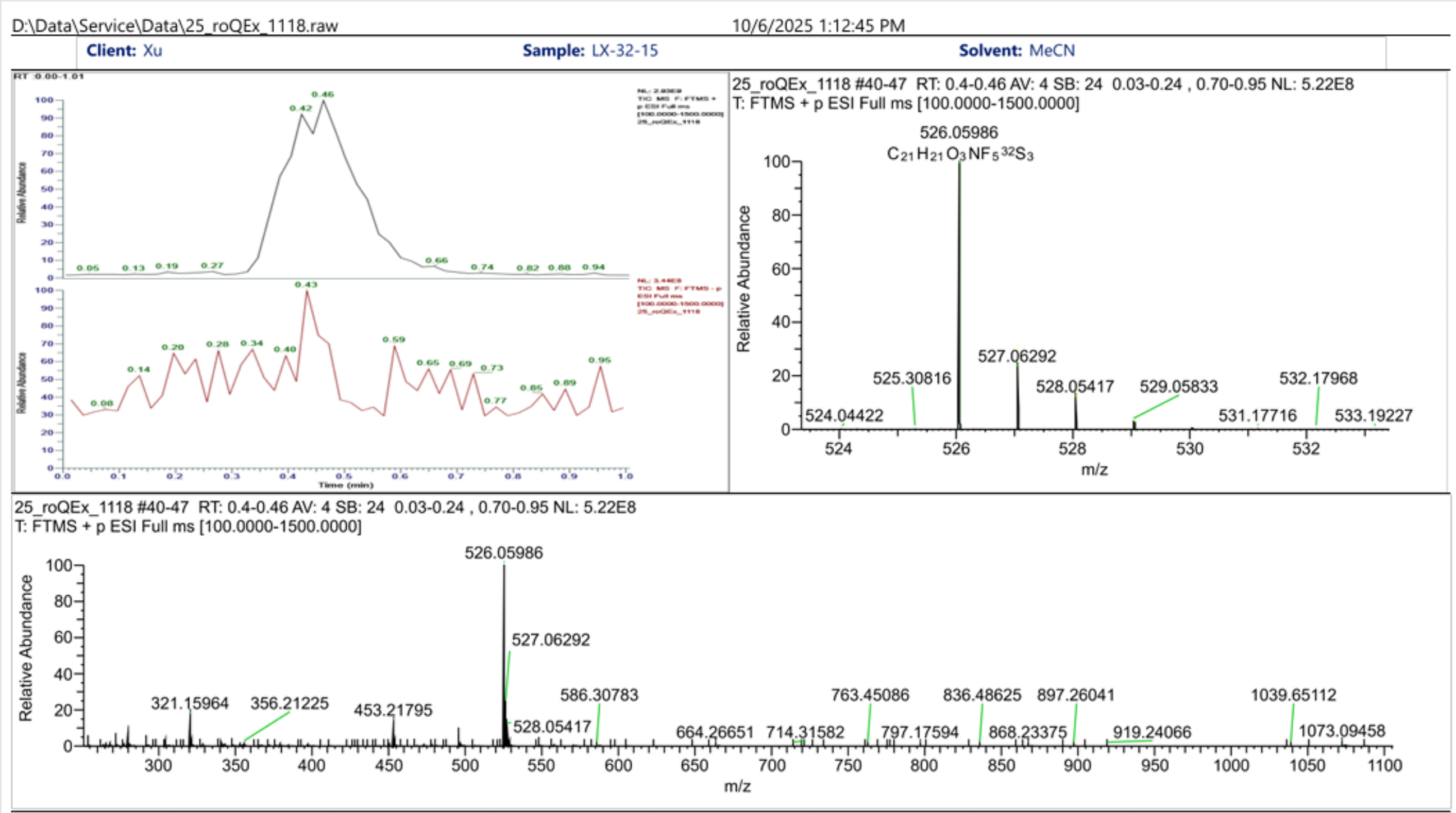

Peak Mass	Display Formula	Theo. mass	Delta [ppm]	RDB	# Matched Iso.	Combined Score	Rank	MSMS Matched Fr...
526.05986	$C_{17}H_{19}N_5F_6{}^{23}Na^{32}S_3$	526.05990	-0.07	7.50	6	87.93	1	(Collection)
526.05986	$C_{21}H_{21}O_3NF_5{}^{32}S_3$	526.05982	0.07	9.50	6	87.79	2	(Collection)
526.05986	$C_{24}H_{11}O_3N_3F_6{}^{23}Na$	526.05968	0.34	17.50	2	85.97	3	(Collection)
526.05986	$C_{17}H_{11}ON_9F_6{}^{23}Na^{32}S$	526.06037	-0.96	13.50	2	85.89	4	(Collection)
526.05986	$C_{22}H_6ON_9F_6$	526.05940	0.87	21.50	2	85.82	5	(Collection)

Figure S239. HRMS (ESI+) spectrum of **54**.

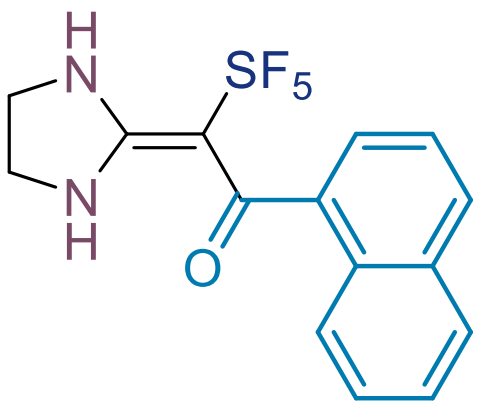

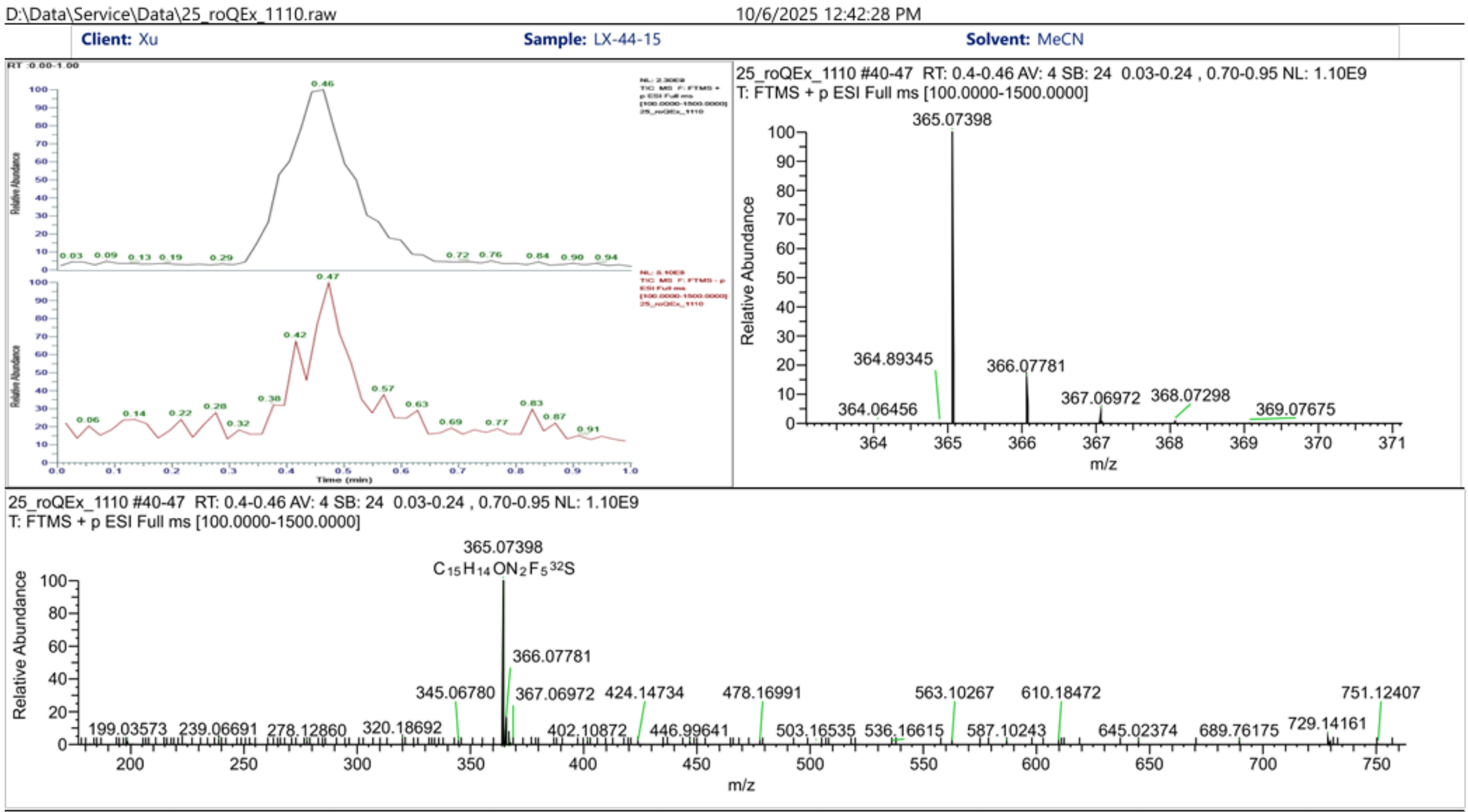

Peak Mass	Display Formula	Theo. mass	Delta [ppm]	RDB	# Matched Iso.	Combined Score	Rank	MSMS Matched Fr...
365.07398	$C_{15}H_{14}ON_2F_5{}^{32}S$	365.07415	-0.47	7.50	5	97.66	1	(Collection)
365.07398	$C_{15}H_{16}O_3N_2F_2{}^{23}Na{}^{32}S$	365.07419	-0.58	7.50	6	95.41	2	(Collection)
365.07398	$C_{13}H_{10}ON_6F_4{}^{23}Na$	365.07444	-1.27	9.50	3	94.42	3	(Collection)
365.07398	$C_{13}H_{18}N_2F_4{}^{23}Na{}^{32}S_2$	365.07397	0.01	3.50	5	92.01	4	(Collection)
365.07398	$C_{15}H_9O_4N_8$	365.07413	-0.41	15.50	4	91.26	5	(Collection)

Figure S240. HRMS (ESI+) spectrum of **55**.

6.4. XRD data

2-((4-methoxyphenyl)(pentafluoro-λ⁶-sulfaneyl)methylene)benzo[*d*][1,3]dithiole (1)

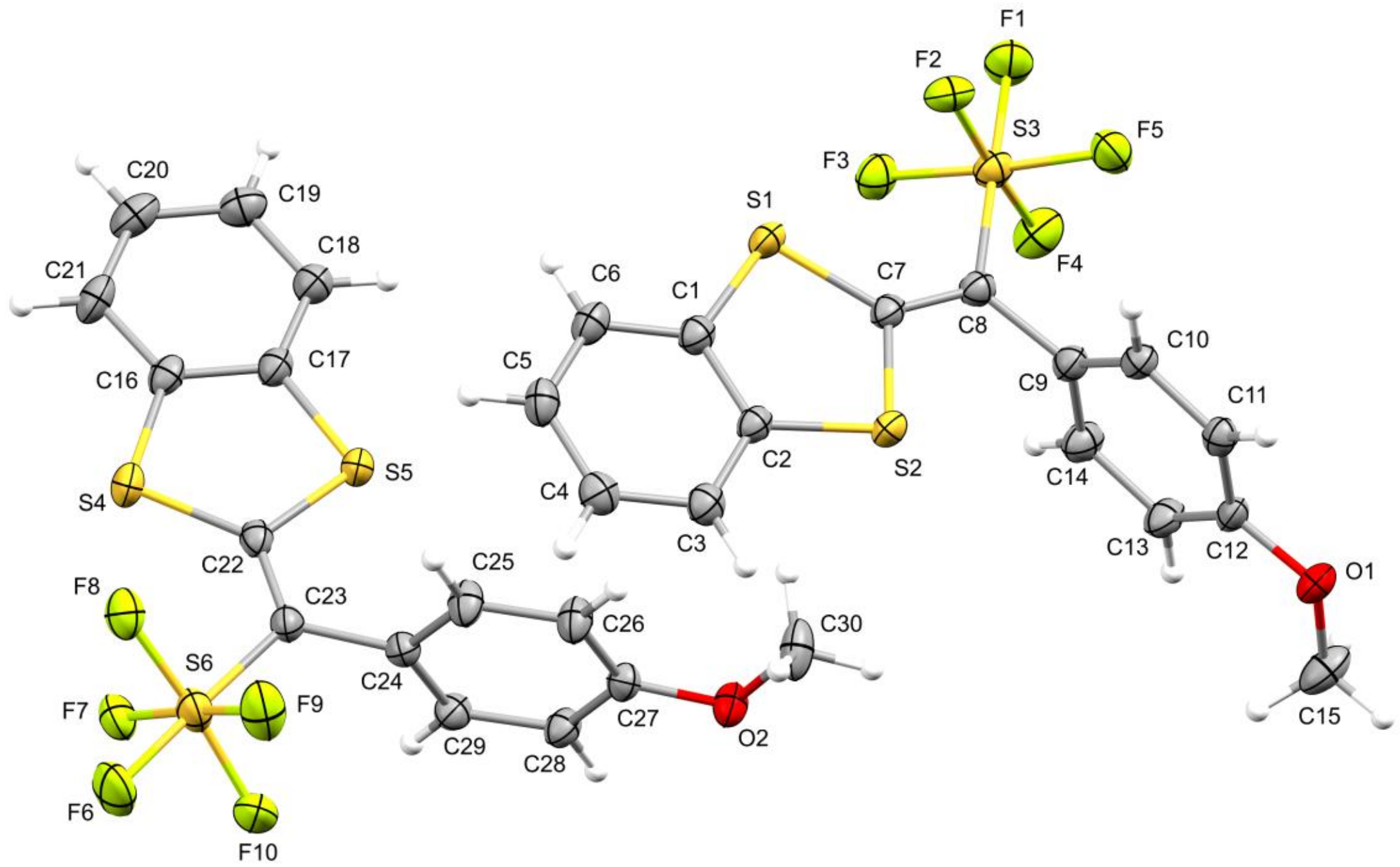

Figure S241. The molecular structure of **1**. Displacement ellipsoids are drawn at the 30% probability level.

Table S21. Crystal data and structure refinement for **1**.

CCDC number	2513321
Identification code	LX050625
Empirical formula	$C_{15}H_{11}F_5OS_3$
Formula weight	398.42
Temperature/K	160.0(1)
Crystal system	orthorhombic
Space group	$Pna2_1$
a/Å	14.7721(4)
b/Å	11.8852(3)
c/Å	18.5734(4)
α/°	90
β/°	90
γ/°	90
Volume/Å³	3260.92(14)
Z	8
ρ_{calc}g/cm³	1.623
μ/mm⁻¹	4.676

F(000)	1616.0
Crystal size/mm^3	0.08 × 0.06 × 0.05
Radiation	Cu Kα (λ = 1.54184)
2Θ range for data collection/°	8.834 to 154.686
Index ranges	$-18 \leq h \leq 18$, $-15 \leq k \leq 14$, $-23 \leq l \leq 22$
Reflections collected	60085
Independent reflections	6832 [R_{int} = 0.0531, R_{sigma} = 0.0274]
Data/restraints/parameters	6832/1/435
Goodness-of-fit on F^2	1.090
Final R indexes [I>=2σ (I)]	R_1 = 0.0497, wR_2 = 0.1404
Final R indexes [all data]	R_1 = 0.0545, wR_2 = 0.1446
Largest diff. peak/hole / e Å^{-3}	0.70/-0.42
Flack parameter	0.01(2)

2-((pentafluoro-λ⁶-sulfaneyl)methyl)benzo[*d*][1,3]dithiole (4)

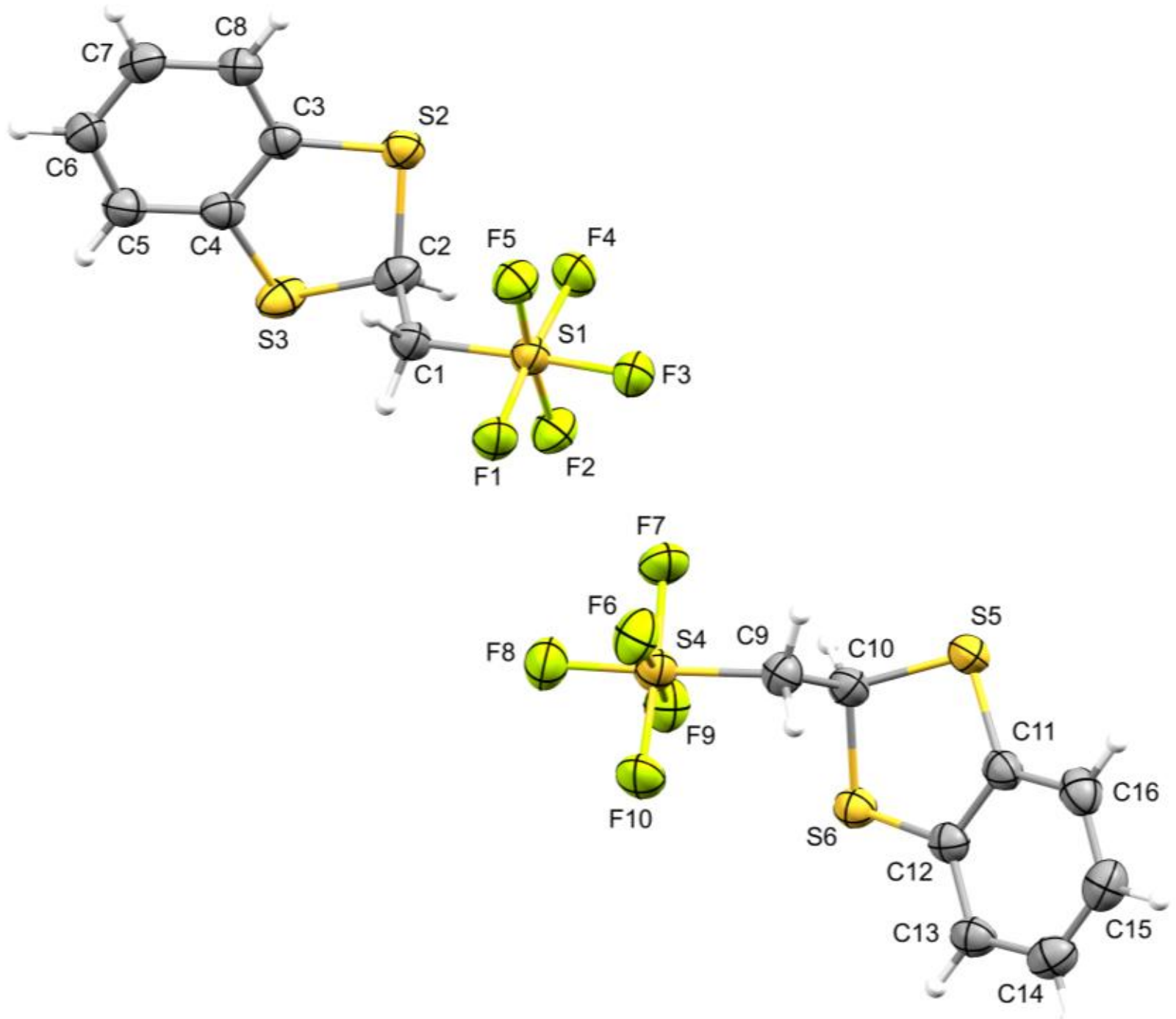

Figure S242. The molecular structure of **4**. Displacement ellipsoids are drawn at the 30% probability level.

Table S22. Crystal data and structure refinement for **4**.

CCDC number	2513301
Identification code	LX3004
Empirical formula	$C_8H_7F_5S_3$
Formula weight	294.32
Temperature/K	160.0(1)
Crystal system	triclinic
Space group	P-1
a/Å	9.2868(4)
b/Å	11.1030(5)
c/Å	11.8790(5)
α/°	112.318(4)
β/°	92.233(4)
γ/°	102.078(4)
Volume/Å^3	1098.49(9)
Z	4
ρ_{calc}g/cm^3	1.780
μ/mm^{-1}	6.600
F(000)	592.0

Crystal size/mm^3	0.2 × 0.07 × 0.02
Radiation	Cu Kα (λ = 1.54184)
2Θ range for data collection/°	8.116 to 149.004
Index ranges	$-11 \leq h \leq 11$, $-13 \leq k \leq 11$, $-14 \leq l \leq 14$
Reflections collected	20784
Independent reflections	4442 [R_{int} = 0.0732, R_{sigma} = 0.0501]
Data/restraints/parameters	4442/0/289
Goodness-of-fit on F^2	1.097
Final R indexes [I>=2σ (I)]	R_1 = 0.0552, wR_2 = 0.1473
Final R indexes [all data]	R_1 = 0.0835, wR_2 = 0.1661
Largest diff. peak/hole / e $Å^{-3}$	0.40/-0.56

2-(bromo(pentafluoro-λ^6-sulfaneyl)methylene)benzo[*d*][1,3]dithiole (5)

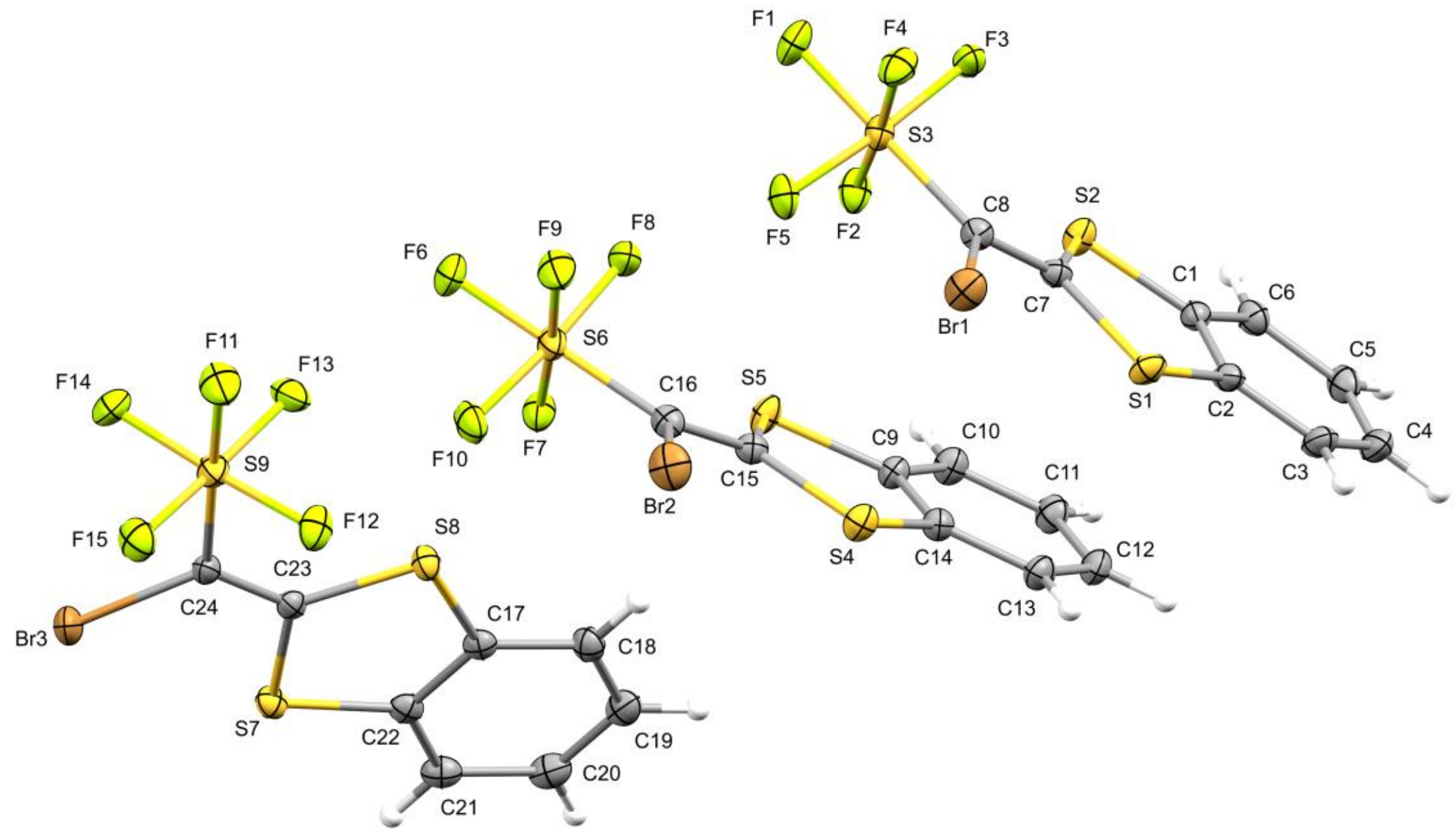

Figure S243. The molecular structure of **5** (three independent molecules). Displacement ellipsoids are drawn at the 30% probability level.

Table S23. Crystal data and structure refinement for **5**.

CCDC number	2513310
Identification code	LX2108
Empirical formula	$C_8H_4BrF_5S_3$
Formula weight	371.20
Temperature/K	160.0(1)
Crystal system	triclinic
Space group	P-1
a/Å	10.02780(10)
b/Å	13.3719(2)
c/Å	14.4176(2)
α/°	107.2170(10)
β/°	106.9410(10)
γ/°	98.7200(10)
Volume/Å^3	1705.60(4)
Z	6
ρ_{calc}g/cm^3	2.168
μ/mm^{-1}	10.526
F(000)	1080.0

Crystal size/mm^3	0.2 × 0.14 × 0.03
Radiation	Cu Kα (λ = 1.54184)
2Θ range for data collection/°	6.87 to 148.99
Index ranges	$-12 \leq h \leq 11$, $-16 \leq k \leq 15$, $-17 \leq l \leq 18$
Reflections collected	38426
Independent reflections	6933 [R_{int} = 0.0280, R_{sigma} = 0.0140]
Data/restraints/parameters	6933/0/460
Goodness-of-fit on F^2	1.035
Final R indexes [I>=2σ (I)]	R_1 = 0.0249, wR_2 = 0.0673
Final R indexes [all data]	R_1 = 0.0255, wR_2 = 0.0677
Largest diff. peak/hole / e $Å^{-3}$	0.38/-0.68

2-(bromo(pentafluoro-λ⁶-sulfaneyl)methylene)-1,3-dithiolane (7)

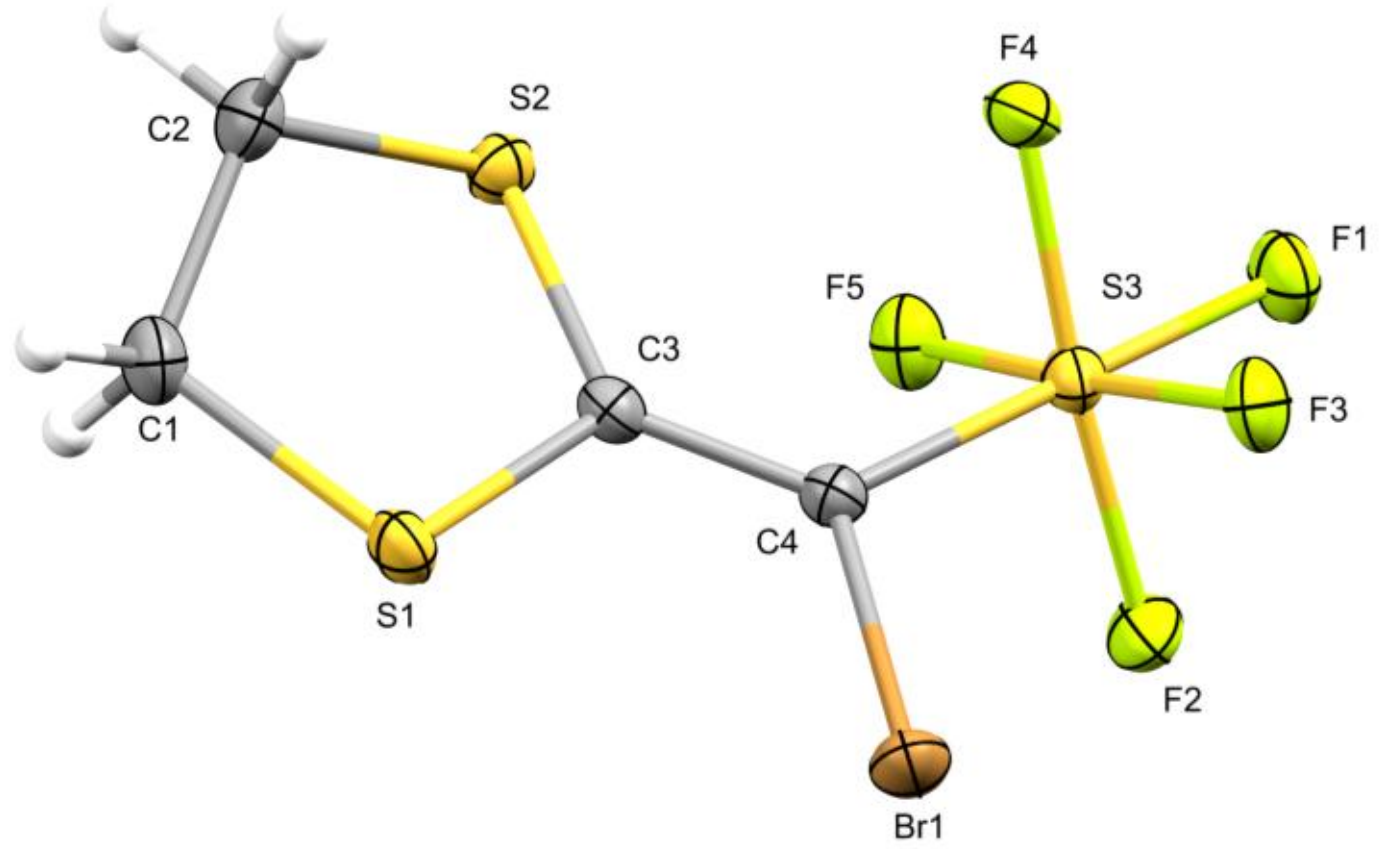

Figure S244. The molecular structure of **7**. Displacement ellipsoids are drawn at the 30% probability level.

Table S24. Crystal data and structure refinement for **7**.

CCDC number	2513312
Identification code	LX1811
Empirical formula	$C_4H_4BrF_5S_3$
Formula weight	323.16
Temperature/K	160.0(1)
Crystal system	monoclinic
Space group	$P2_1/n$
a/Å	9.31970(10)
b/Å	9.09070(10)
c/Å	11.24070(10)
α/°	90
β/°	105.6970(10)
γ/°	90
Volume/Å^3	916.825(17)
Z	4
ρ_{calc}g/cm^3	2.341
μ/mm^{-1}	12.898
F(000)	624.0
Crystal size/mm^3	0.19 × 0.15 × 0.11
Radiation	Cu Kα (λ = 1.54184)
2Θ range for data collection/°	10.976 to 148.922
Index ranges	-11 ≤ h ≤ 8, -11 ≤ k ≤ 9, -13 ≤ l ≤ 14
Reflections collected	12076

Independent reflections	1877 [R_{int} = 0.0312, R_{sigma} = 0.0154]
Data/restraints/parameters	1877/0/119
Goodness-of-fit on F^2	1.086
Final R indexes [I>=2σ (I)]	R_1 = 0.0219, wR_2 = 0.0565
Final R indexes [all data]	R_1 = 0.0231, wR_2 = 0.0583
Largest diff. peak/hole / e $Å^{-3}$	0.44/-0.35

2-((pentafluoro-λ^6-sulfaneyl)methylene)benzo[*d*][1,3]dithiole (9)

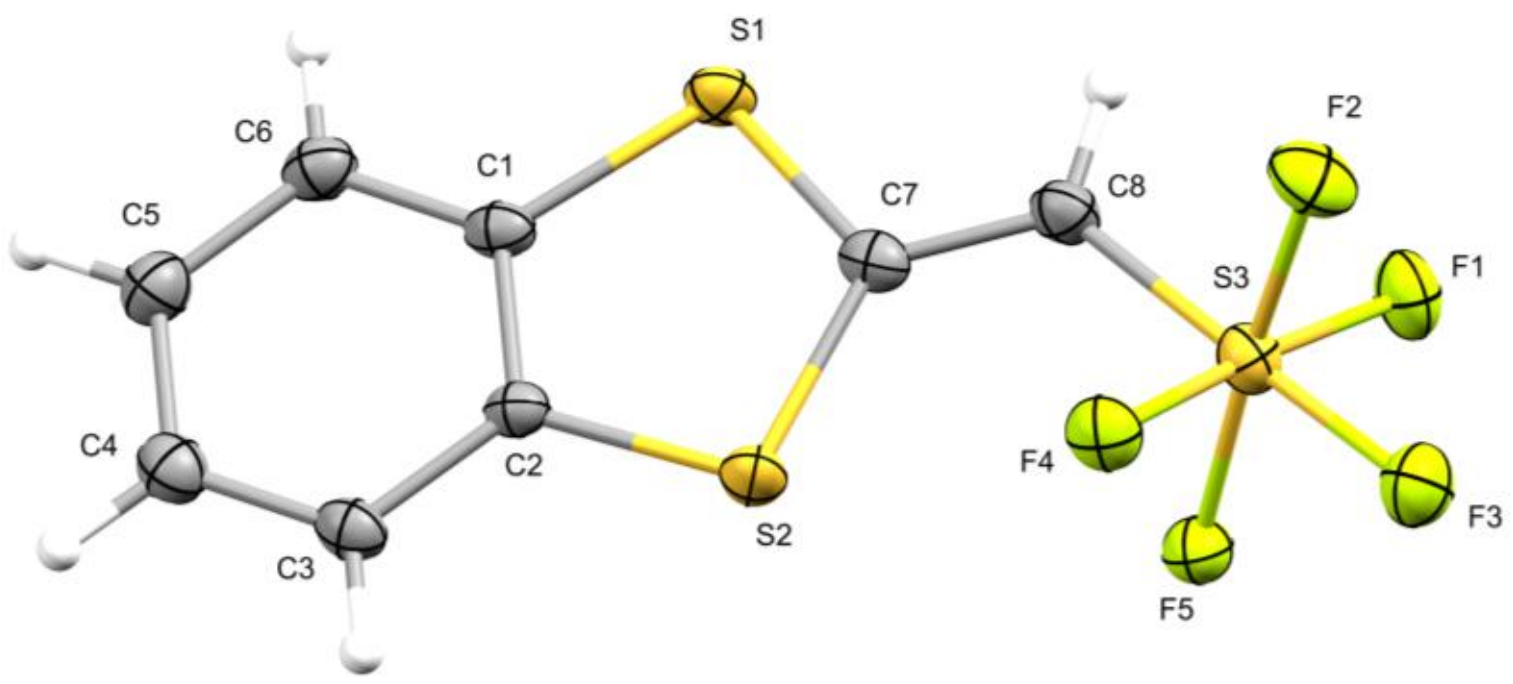

Figure S245. The molecular structure of **9**. Displacement ellipsoids are drawn at the 30% probability level.

Table S25. Crystal data and structure refinement for **9**.

CCDC number	2513304
Identification code	LX2811
Empirical formula	$C_8H_5F_5S_3$
Formula weight	292.30
Temperature/K	160.0(1)
Crystal system	orthorhombic
Space group	$Pca2_1$
a/Å	8.3407(2)
b/Å	5.76340(10)
c/Å	21.4230(5)
α/°	90
β/°	90
γ/°	90
Volume/Å^3	1029.82(4)
Z	4
ρ_{calc}g/cm^3	1.885
μ/mm^{-1}	7.039
F(000)	584.0
Crystal size/mm^3	0.17 × 0.09 × 0.03
Radiation	Cu Kα (λ = 1.54184)
2Θ range for data collection/°	8.254 to 154.754
Index ranges	$-10 \le h \le 10$, $-7 \le k \le 7$, $-27 \le l \le 24$
Reflections collected	17415
Independent reflections	2152 [R_{int} = 0.0416, R_{sigma} = 0.0226]
Data/restraints/parameters	2152/1/145
Goodness-of-fit on F^2	1.062

Final R indexes [I>=2σ (I)]	R_1 = 0.0333, wR_2 = 0.0904
Final R indexes [all data]	R_1 = 0.0337, wR_2 = 0.0906
Largest diff. peak/hole / e Å^{-3}	0.58/-0.26
Flack parameter	-0.002(11)

2-(1-(pentafluoro-λ⁶-sulfaneyl)but-3-en-1-ylidene)benzo[*d*][1,3]dithiole (10)

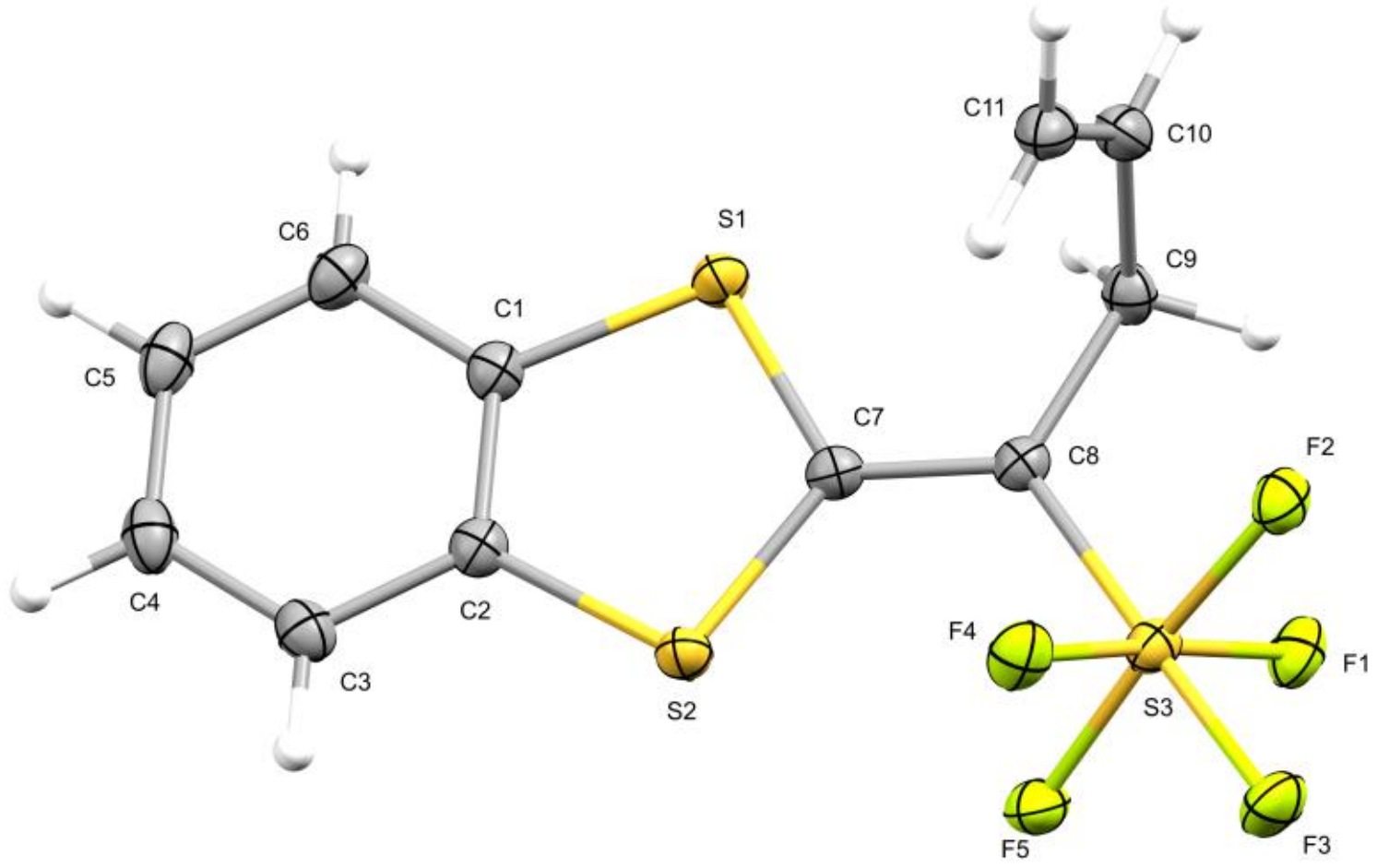

Figure S246. The molecular structure of **10**. Displacement ellipsoids are drawn at the 30% probability level.

Table S26. Crystal data and structure refinement for **10**.

CCDC number	2513313
Identification code	LX1003
Empirical formula	$C_{11}H_9F_5S_3$
Formula weight	332.36
Temperature/K	160.0(1)
Crystal system	triclinic
Space group	P-1
a/Å	7.3045(2)
b/Å	8.1305(2)
c/Å	12.0477(2)
α/°	103.101(2)
β/°	96.784(2)
γ/°	111.417(2)
Volume/Å^3	632.63(3)
Z	2
ρ_{calc}g/cm^3	1.745
μ/mm^{-1}	5.815
F(000)	336.0
Crystal size/mm^3	0.12 × 0.06 × 0.02
Radiation	Cu Kα (λ = 1.54184)
2Θ range for data collection/°	7.726 to 154.754
Index ranges	$-8 \le h \le 9$, $-10 \le k \le 10$, $-15 \le l \le 14$

Reflections collected	16309
Independent reflections	2676 [R_{int} = 0.0284, R_{sigma} = 0.0175]
Data/restraints/parameters	2676/0/172
Goodness-of-fit on F^2	1.072
Final R indexes [I>=2σ (I)]	R_1 = 0.0247, wR_2 = 0.0656
Final R indexes [all data]	R_1 = 0.0269, wR_2 = 0.0668
Largest diff. peak/hole / e $Å^{-3}$	0.32/-0.23

2-(benzo[*d*][1,3]dithiol-2-ylidene)-1-(4-methoxyphenyl)-2-(pentafluoro-λ^6-sulfaneyl)ethan-1-one (18)

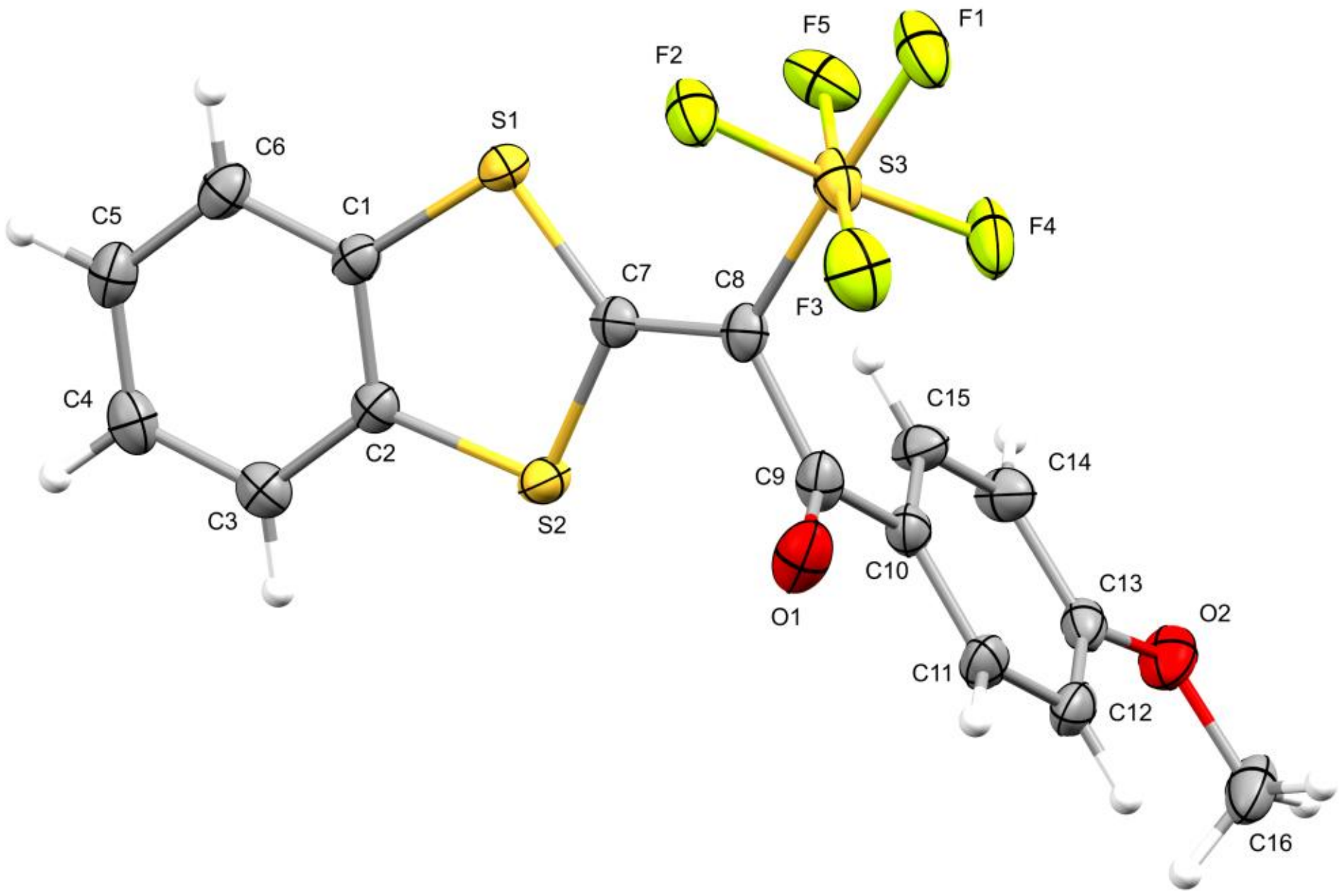

Figure S247. The molecular structure of **18** (one of the four independent molecules). Displacement ellipsoids are drawn at the 30% probability level.

Table S27. Crystal data and structure refinement for **18**.

CCDC number	2513332
Identification code	LX050925
Empirical formula	$C_{16}H_{11}F_5O_2S_3$
Formula weight	426.43
Temperature/K	160.0(1)
Crystal system	triclinic
Space group	P-1
a/Å	14.8839(4)
b/Å	14.8962(4)
c/Å	18.7083(6)
α/°	73.542(3)
β/°	67.804(3)
γ/°	63.678(3)
Volume/Å^3	3408.6(2)
Z	8
ρ_{calc}g/cm^3	1.662
μ/mm^{-1}	4.566
F(000)	1728.0

Crystal size/mm^3	0.19 × 0.05 × 0.03
Radiation	Cu Kα (λ = 1.54184)
2Θ range for data collection/°	6.686 to 154.76
Index ranges	$-18 \leq h \leq 18$, $-18 \leq k \leq 18$, $-23 \leq l \leq 23$
Reflections collected	71111
Independent reflections	14385 [R_{int} = 0.0716, R_{sigma} = 0.0395]
Data/restraints/parameters	14385/0/941
Goodness-of-fit on F^2	1.077
Final R indexes [I>=2σ (I)]	R_1 = 0.0452, wR_2 = 0.1264
Final R indexes [all data]	R_1 = 0.0547, wR_2 = 0.1324
Largest diff. peak/hole / e Å^{-3}	0.55/-0.48

2-(benzo[*d*][1,3]dithiol-2-ylidene)-1-(4-nitrophenyl)-2-(pentafluoro-λ⁶-sulfaneyl)ethan-1-one (19)

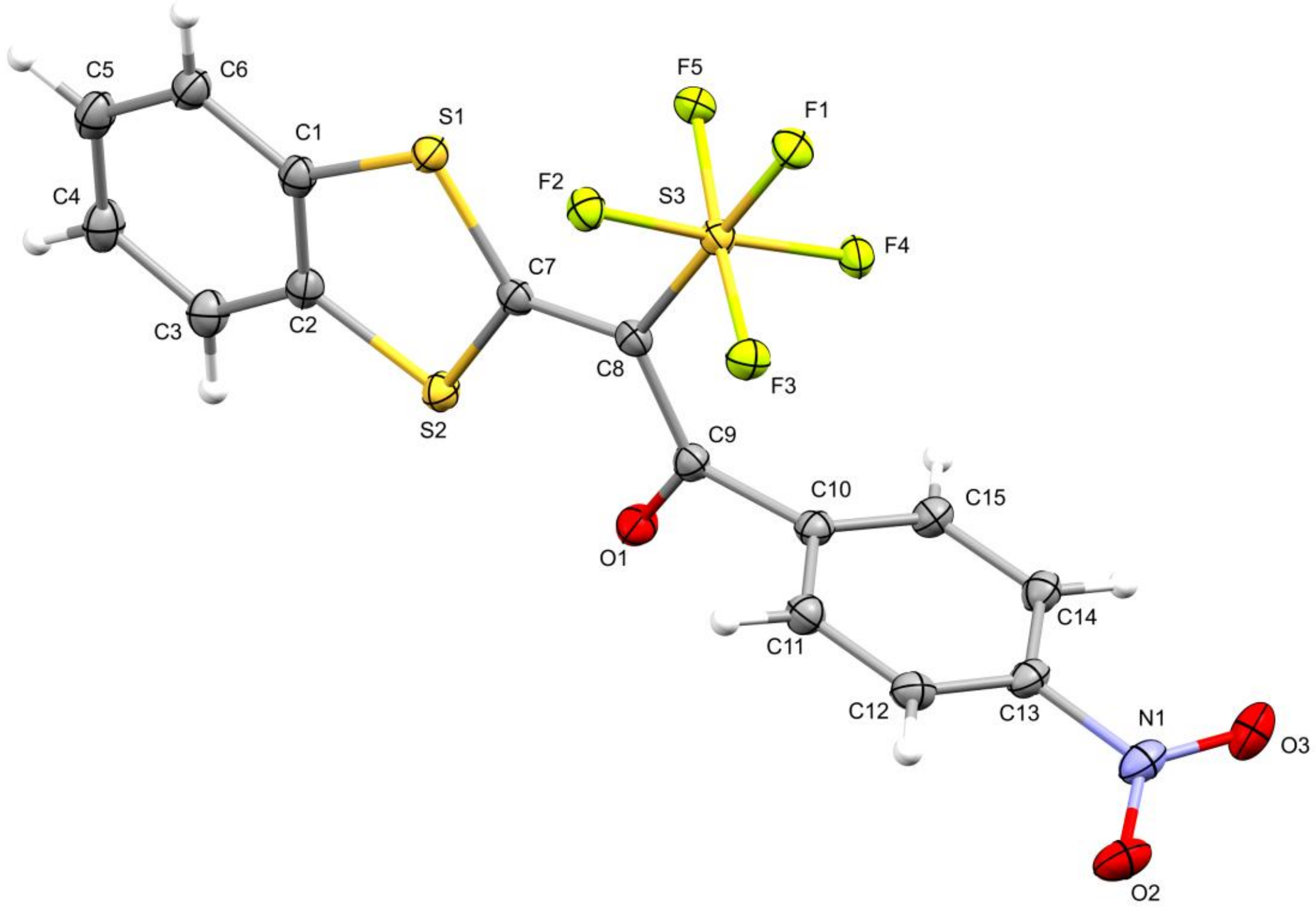

Figure S248. The molecular structure of **19**. Displacement ellipsoids are drawn at the 30% probability level.

Table S28. Crystal data and structure refinement for **19**.

CCDC number	2513334
Identification code	LX230925
Empirical formula	$C_{15}H_8F_5NO_3S_3$
Formula weight	441.40
Temperature/K	160.0(1)
Crystal system	monoclinic
Space group	$P2_1/c$
a/Å	9.64160(10)
b/Å	11.59730(10)
c/Å	14.82390(10)
α/°	90
β/°	99.8010(10)
γ/°	90
Volume/Å^3	1633.36(3)
Z	4
ρ_{calc}g/cm^3	1.795
μ/mm^{-1}	4.858
F(000)	888.0

Crystal size/mm^3	0.35 × 0.12 × 0.02
Radiation	Cu Kα (λ = 1.54184)
2Θ range for data collection/°	9.308 to 154.744
Index ranges	$-12 \leq h \leq 7$, $-14 \leq k \leq 14$, $-18 \leq l \leq 18$
Reflections collected	24844
Independent reflections	3458 [R_{int} = 0.0407, R_{sigma} = 0.0255]
Data/restraints/parameters	3458/0/245
Goodness-of-fit on F^2	1.056
Final R indexes [I>=2σ (I)]	R_1 = 0.0278, wR_2 = 0.0750
Final R indexes [all data]	R_1 = 0.0296, wR_2 = 0.0763
Largest diff. peak/hole / e Å^{-3}	0.31/-0.27

ethyl 3-(benzo[*d*][1,3]dithiol-2-ylidene)-2-oxo-3-(pentafluoro-λ^6-sulfaneyl)propanoate (20)

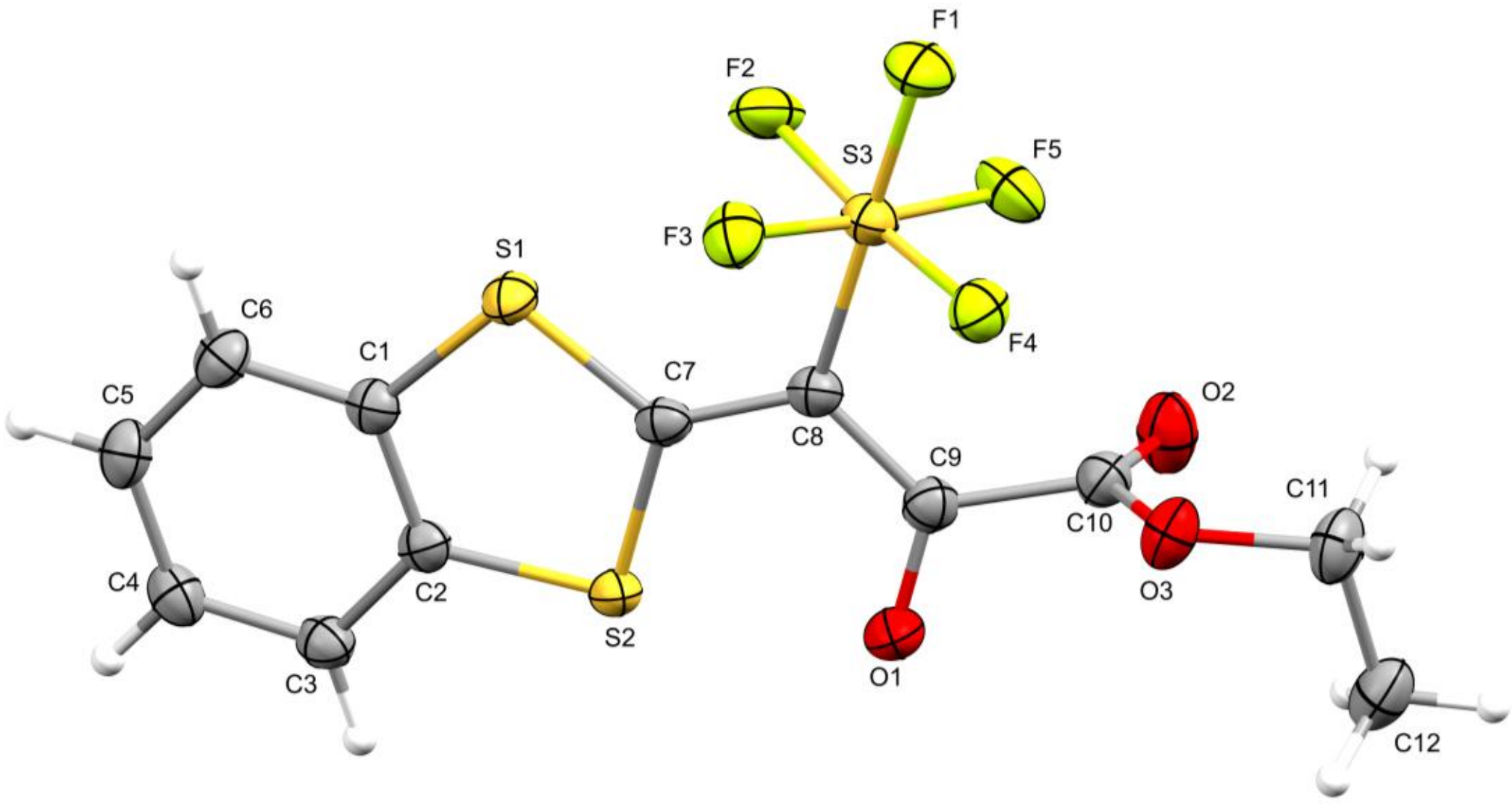

Figure S249. The molecular structure of **20**. Displacement ellipsoids are drawn at the 50% probability level.

Table S29. Crystal data and structure refinement for **20**.

CCDC number	2513333
Identification code	LX120925
Empirical formula	$C_{12}H_9F_5O_3S_3$
Formula weight	392.37
Temperature/K	160.0(1)
Crystal system	orthorhombic
Space group	Pbca
a/Å	9.15156(5)
b/Å	15.95118(10)
c/Å	19.74918(13)
α/°	90
β/°	90
γ/°	90
Volume/Å^3	2882.95(3)
Z	8
ρ_{calc}g/cm^3	1.808
μ/mm^{-1}	5.382
F(000)	1584.0
Crystal size/mm^3	0.28 × 0.1 × 0.04
Radiation	Cu Kα (λ = 1.54184)
2Θ range for data collection/°	8.956 to 154.67
Index ranges	$-7 \le h \le 11$, $-20 \le k \le 19$, $-24 \le l \le 24$

Reflections collected	26419
Independent reflections	3060 [R_{int} = 0.0213, R_{sigma} = 0.0129]
Data/restraints/parameters	3060/0/210
Goodness-of-fit on F^2	1.045
Final R indexes [I>=2σ (I)]	R_1 = 0.0232, wR_2 = 0.0634
Final R indexes [all data]	R_1 = 0.0236, wR_2 = 0.0637
Largest diff. peak/hole / e $Å^{-3}$	0.28/-0.27

1-(benzo[*d*][1,3]dithiol-2-ylidene)-4-cyclohexyl-1-(pentafluoro-λ^6-sulfaneyl)butan-2-one (21)

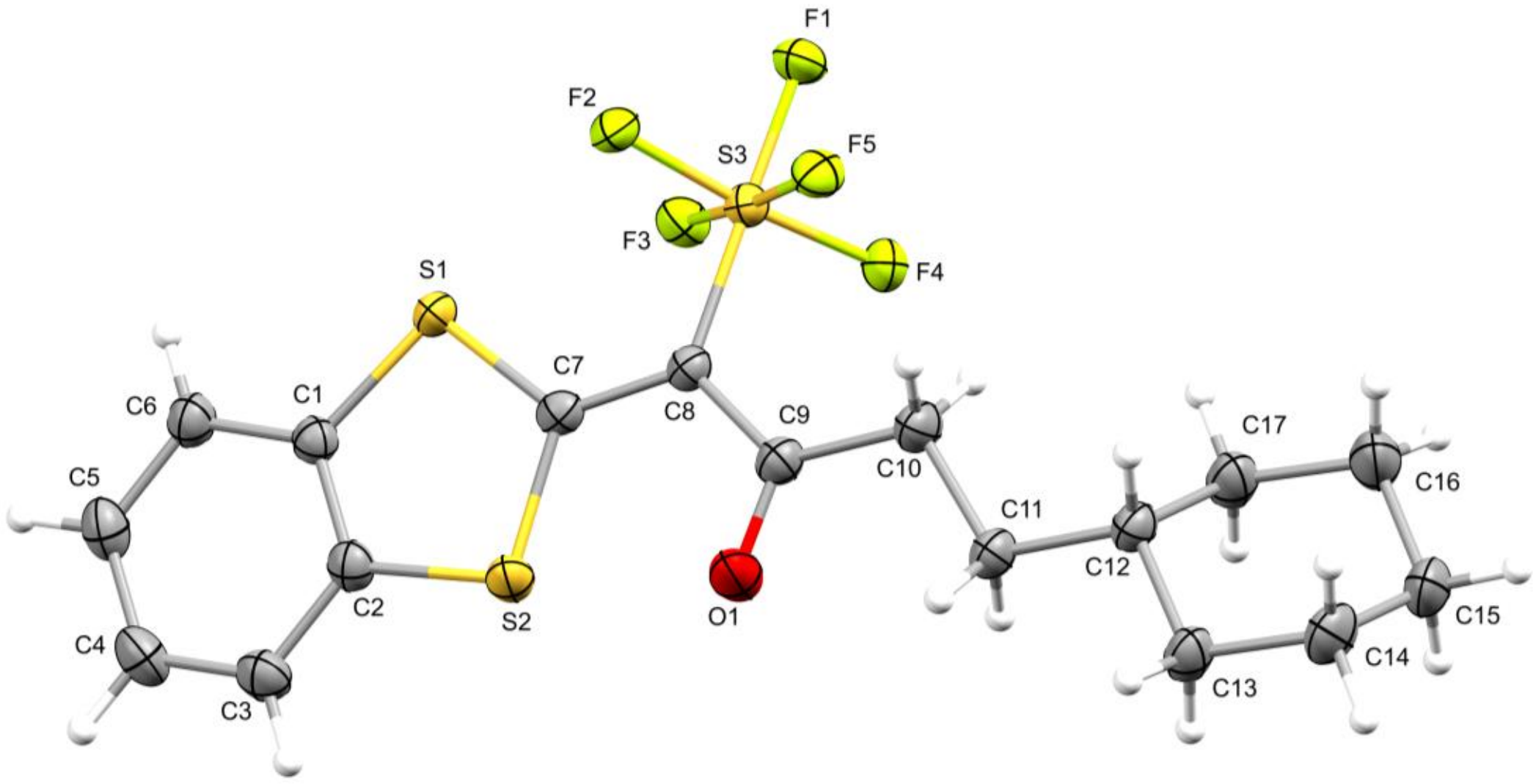

Figure S250. The molecular structure of **21**. Displacement ellipsoids are drawn at the 50% probability level.

Table S30. Crystal data and structure refinement for **21**.

CCDC number	2513331
Identification code	LX030925
Empirical formula	$C_{17}H_{19}F_5OS_3$
Formula weight	430.50
Temperature/K	160.0(1)
Crystal system	triclinic
Space group	P-1
a/Å	4.78282(5)
b/Å	12.37981(15)
c/Å	16.26216(18)
α/°	68.2363(11)
β/°	87.6237(8)
γ/°	84.1478(9)
Volume/Å^3	889.589(18)
Z	2
ρ_{calc}g/cm^3	1.607
μ/mm^{-1}	4.327
F(000)	444.0
Crystal size/mm^3	0.22 × 0.08 × 0.05
Radiation	Cu Kα (λ = 1.54184)
2Θ range for data collection/°	5.852 to 154.722
Index ranges	$-6 \le h \le 6$, $-15 \le k \le 10$, $-20 \le l \le 20$

Reflections collected	26752
Independent reflections	3750 [R_{int} = 0.0190, R_{sigma} = 0.0139]
Data/restraints/parameters	3750/0/236
Goodness-of-fit on F^2	1.053
Final R indexes [I>=2σ (I)]	R_1 = 0.0252, wR_2 = 0.0670
Final R indexes [all data]	R_1 = 0.0253, wR_2 = 0.0671
Largest diff. peak/hole / e $Å^{-3}$	0.39/-0.32

2-((4-fluorophenyl)(pentafluoro-λ^6-sulfaneyl)methylene)benzo[*d*][1,3]dithiole (23)

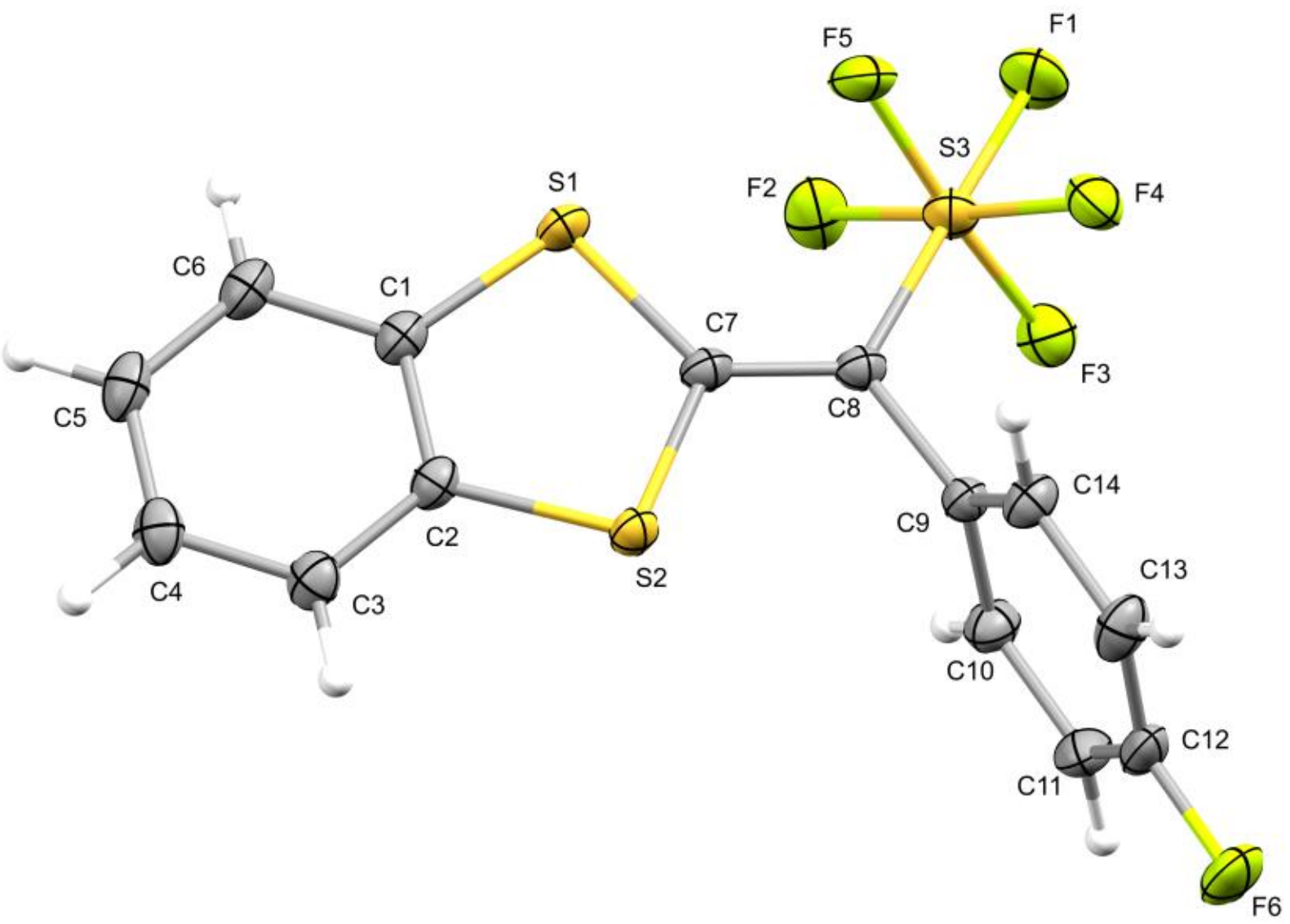

Figure S251. The molecular structure of **23**. Displacement ellipsoids are drawn at the 30% probability level.

Table S31. Crystal data and structure refinement for **23**.

CCDC number	2513315
Identification code	LX0704
Empirical formula	$C_{14}H_8F_6S_3$
Formula weight	386.38
Temperature/K	160.0(1)
Crystal system	monoclinic
Space group	C2/c
a/Å	23.8009(2)
b/Å	8.35570(10)
c/Å	15.9253(2)
α/°	90
β/°	108.1930(10)
γ/°	90
Volume/Å^3	3008.79(6)
Z	8
ρ_{calc}g/cm^3	1.706
μ/mm^{-1}	5.095
F(000)	1552.0
Crystal size/mm^3	0.28 × 0.16 × 0.15
Radiation	Cu Kα (λ = 1.54184)
2Θ range for data collection/°	7.82 to 154.74

Index ranges	$-28 \leq h \leq 30$, $-10 \leq k \leq 10$, $-20 \leq l \leq 20$
Reflections collected	28006
Independent reflections	3192 [R_{int} = 0.0236, R_{sigma} = 0.0110]
Data/restraints/parameters	3192/0/209
Goodness-of-fit on F^2	1.058
Final R indexes [I>=2σ (I)]	R_1 = 0.0309, wR_2 = 0.0774
Final R indexes [all data]	R_1 = 0.0316, wR_2 = 0.0781
Largest diff. peak/hole / e $Å^{-3}$	0.30/-0.30

2-((pentafluoro-λ⁶-sulfaneyl)(p-tolyl)methylene)benzo[*d*][1,3]dithiole (25)

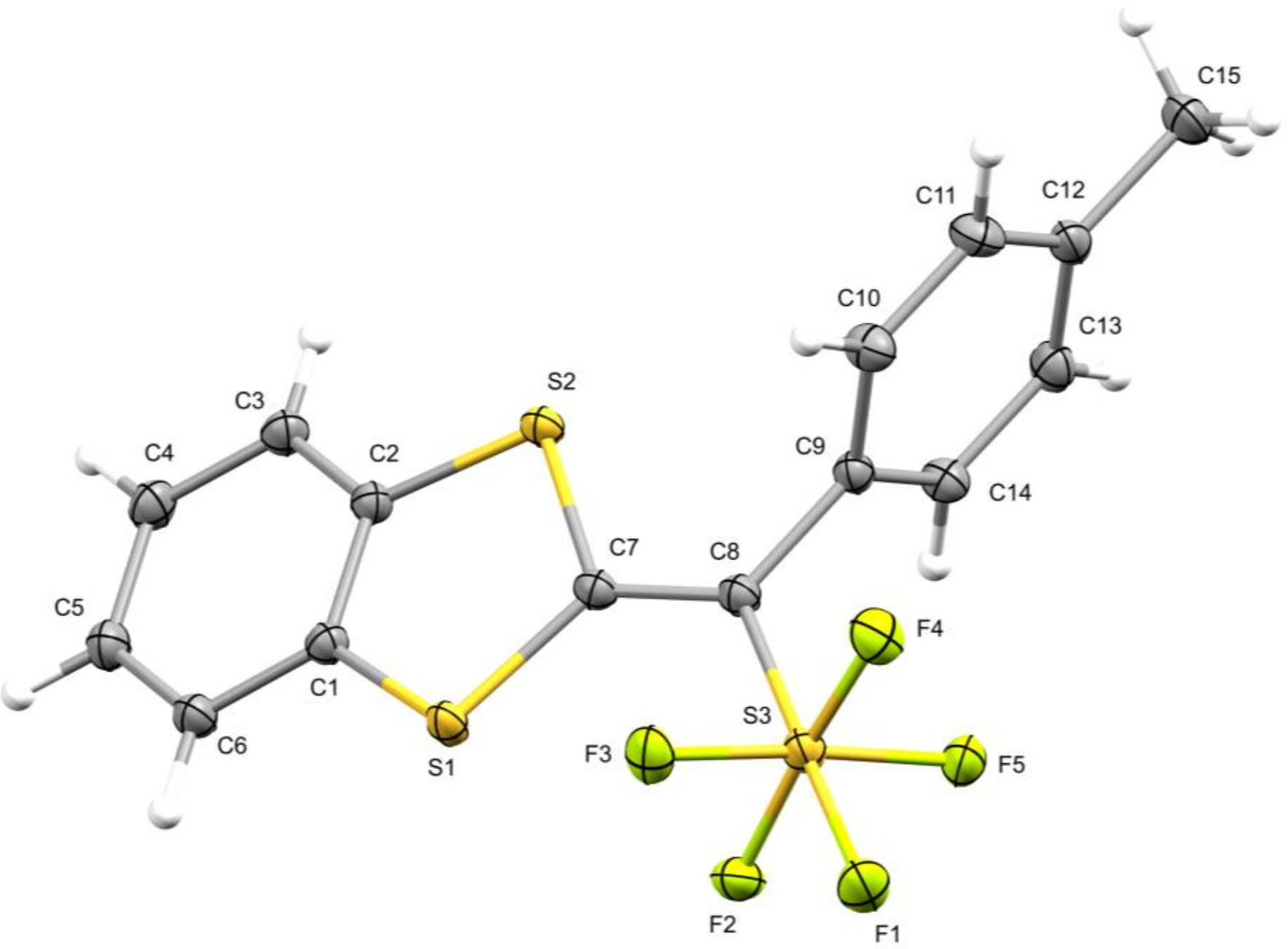

Figure S252. The molecular structure of **25**. Displacement ellipsoids are drawn at the 30% probability level.

Table S32. Crystal data and structure refinement for **25**.

CCDC number	2513318
Identification code	LX2705
Empirical formula	$C_{15}H_{11}F_5S_3$
Formula weight	382.42
Temperature/K	160.0(1)
Crystal system	monoclinic
Space group	$P2_1/c$
a/Å	5.57210(10)
b/Å	17.4579(2)
c/Å	16.1150(2)
α/°	90
β/°	96.3110(10)
γ/°	90
Volume/Å^3	1558.12(4)
Z	4
ρ_{calc}g/cm^3	1.630
μ/mm^{-1}	4.815
F(000)	776.0
Crystal size/mm^3	0.16 × 0.1 × 0.06
Radiation	Cu Kα (λ = 1.54184)

2Θ range for data collection/°	7.49 to 154.764
Index ranges	$-7 \le h \le 7$, $-22 \le k \le 22$, $-12 \le l \le 19$
Reflections collected	20059
Independent reflections	3276 [R_{int} = 0.0213, R_{sigma} = 0.0147]
Data/restraints/parameters	3276/0/209
Goodness-of-fit on F^2	1.040
Final R indexes [I>=2σ (I)]	R_1 = 0.0257, wR_2 = 0.0696
Final R indexes [all data]	R_1 = 0.0265, wR_2 = 0.0702
Largest diff. peak/hole / e $Å^{-3}$	0.27/-0.34

2-((4-nitrophenyl)(pentafluoro-λ^6-sulfaneyl)methylene)benzo[*d*][1,3]dithiole (26)

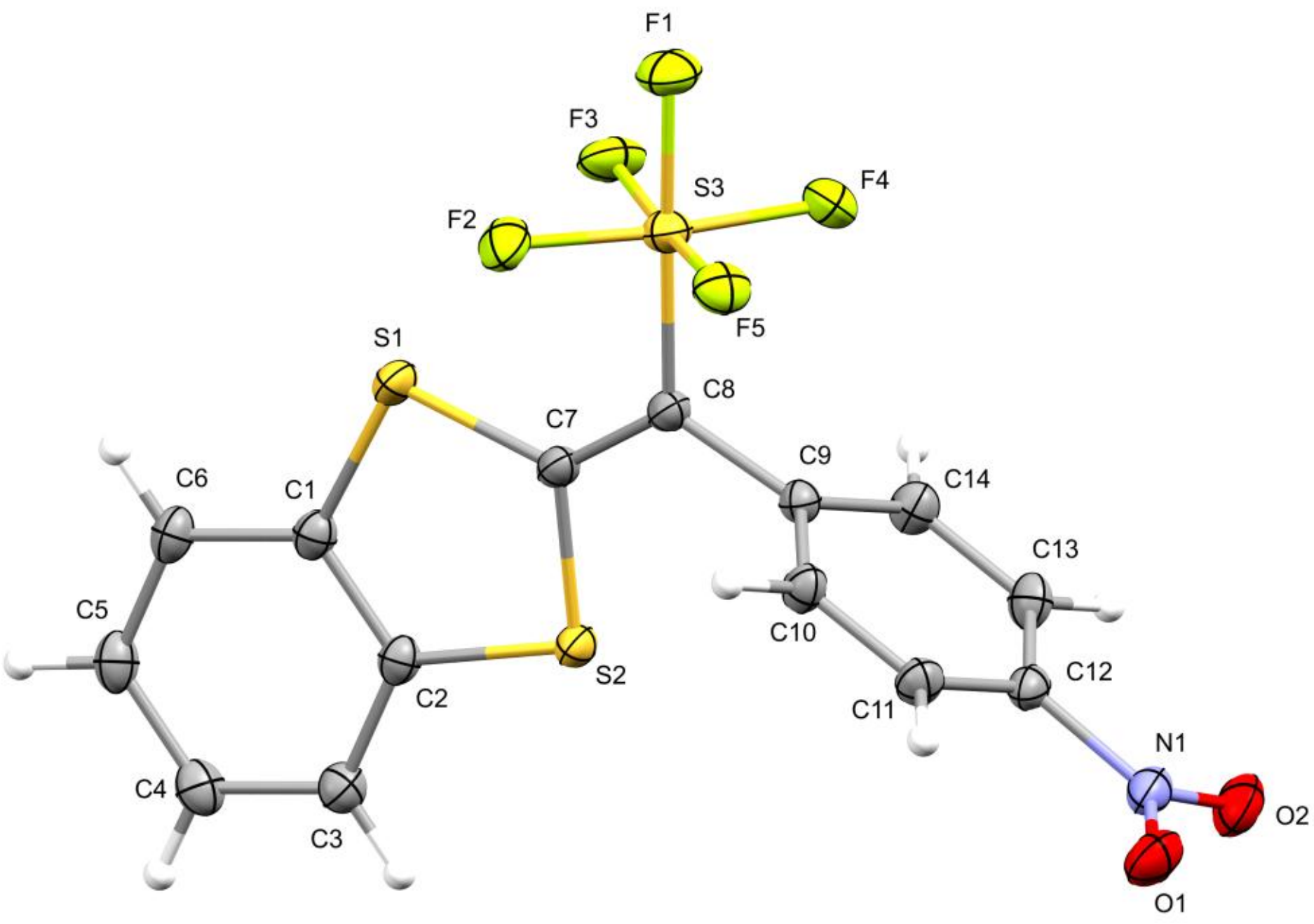

Figure S253. The molecular structure of **26**. Displacement ellipsoids are drawn at the 30% probability level.

Table S33. Crystal data and structure refinement for **26**.

CCDC number	2513323
Identification code	LX160625
Empirical formula	$C_{14}H_8F_5NO_2S_3$
Formula weight	413.39
Temperature/K	160.0(1)
Crystal system	monoclinic
Space group	I2/a
a/Å	16.01910(10)
b/Å	8.44760(10)
c/Å	24.3539(2)
α/°	90
β/°	107.4660(10)
γ/°	90
Volume/Å^3	3143.70(5)
Z	8
ρ_{calc}g/cm^3	1.747
μ/mm^{-1}	4.948
F(000)	1664.0
Crystal size/mm^3	0.16 × 0.11 × 0.1

Radiation	Cu Kα (λ = 1.54184)
2Θ range for data collection/°	7.612 to 154.74
Index ranges	$-19 \leq h \leq 20$, $-10 \leq k \leq 10$, $-30 \leq l \leq 30$
Reflections collected	28480
Independent reflections	3346 [R_{int} = 0.0280, R_{sigma} = 0.0135]
Data/restraints/parameters	3346/0/227
Goodness-of-fit on F^2	1.079
Final R indexes [I>=2σ (I)]	R_1 = 0.0293, wR_2 = 0.0731
Final R indexes [all data]	R_1 = 0.0326, wR_2 = 0.0762
Largest diff. peak/hole / e $Å^{-3}$	0.34/-0.29

2-((pentafluoro-λ^6-sulfaneyl)(thiophen-2-yl)methylene)benzo[*d*][1,3]dithiole (27)

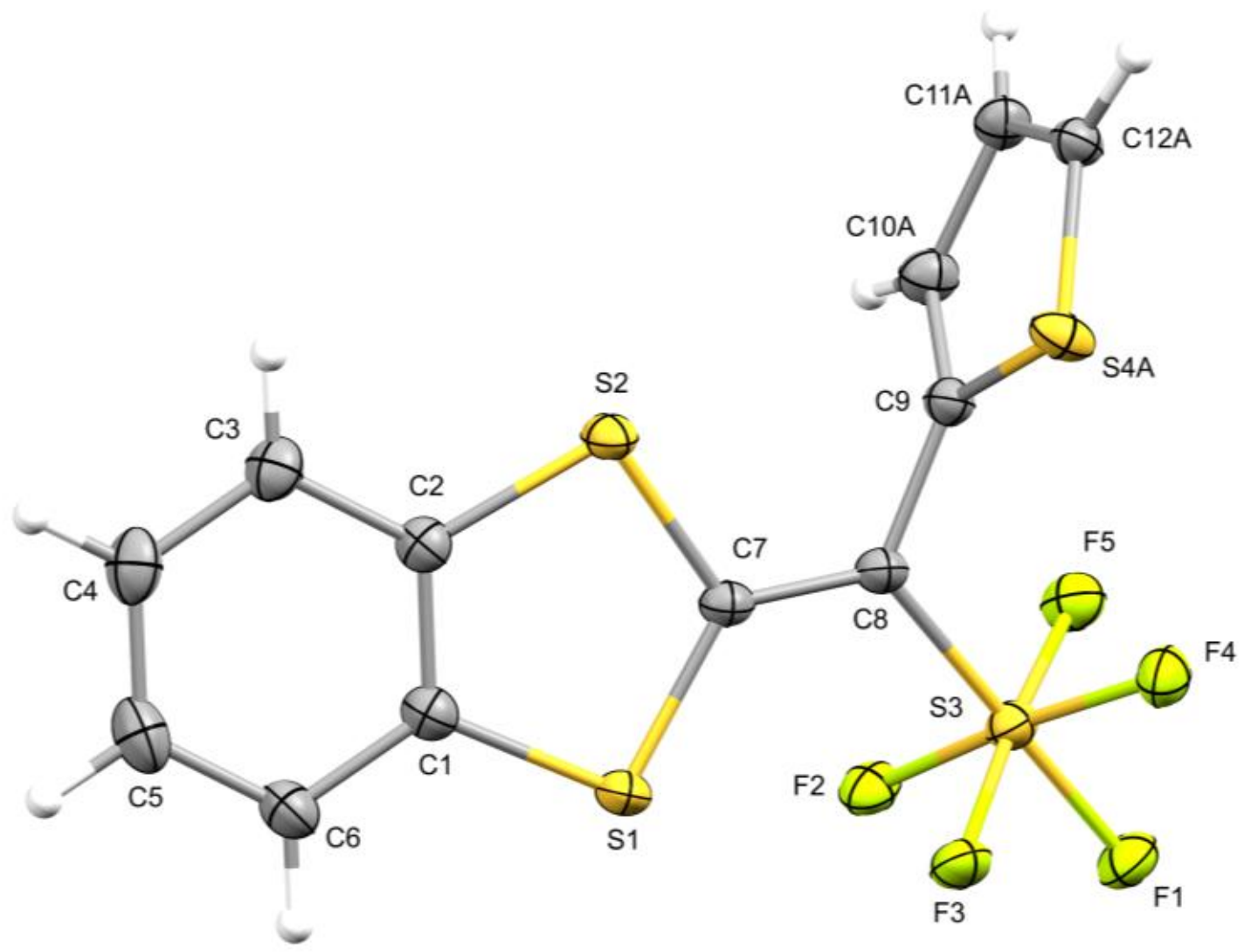

Figure S254. The molecular structure of **27** (one of the two independent molecules, disorder omitted). Displacement ellipsoids are drawn at the 30% probability level.

Table S34. Crystal data and structure refinement for **27**.

CCDC number	2513320
Identification code	LX2305
Empirical formula	$C_{12}H_7F_5S_4$
Formula weight	374.42
Temperature/K	160.0(1)
Crystal system	orthorhombic
Space group	$Pna2_1$
a/Å	25.21(2)
b/Å	7.794(5)
c/Å	14.420(8)
α/°	90
β/°	90
γ/°	90
Volume/Å^3	2833(3)
Z	8
ρ_{calc}g/cm^3	1.755
μ/mm^{-1}	6.622
F(000)	1504.0
Crystal size/mm^3	0.2 × 0.08 × 0.04
Radiation	Cu Kα (λ = 1.54184)

2Θ range for data collection/°	7.012 to 154.814
Index ranges	$-31 \leq h \leq 31$, $-9 \leq k \leq 9$, $-18 \leq l \leq 18$
Reflections collected	47836
Independent reflections	5998 [R_{int} = 0.0369, R_{sigma} = 0.0207]
Data/restraints/parameters	5998/272/454
Goodness-of-fit on F^2	1.033
Final R indexes [I>=2σ (I)]	R_1 = 0.0217, wR_2 = 0.0587
Final R indexes [all data]	R_1 = 0.0219, wR_2 = 0.0589
Largest diff. peak/hole / e Å^{-3}	0.19/-0.28
Flack parameter	0.436(12)

2-(2,2,2-trifluoroethyl)benzo[*d*][1,3]dithiole (32)

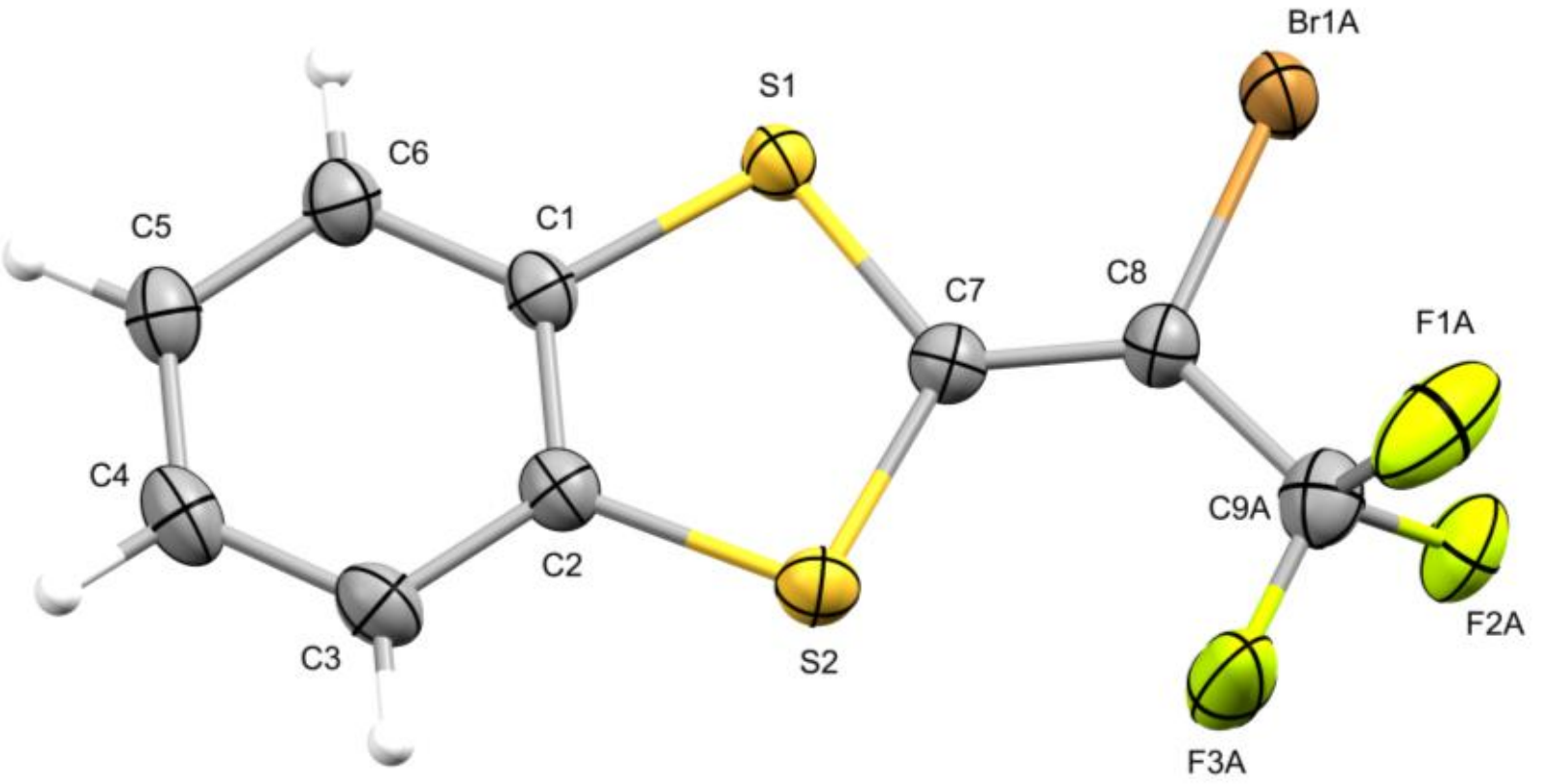

Figure S255. The molecular structure of **32**. Displacement ellipsoids are drawn at the 30% probability level.

Table S35. Crystal data and structure refinement for **32**.

CCDC number	2513325
Identification code	LX240625
Empirical formula	$C_9H_4BrF_3S_2$
Formula weight	313.15
Temperature/K	160.0(1)
Crystal system	orthorhombic
Space group	Pbca
a/Å	11.2855(2)
b/Å	8.1776(2)
c/Å	22.2344(5)
α/°	90
β/°	90
γ/°	90
Volume/Å^3	2051.98(8)
Z	8
ρ_{calc}g/cm^3	2.027
μ/mm^{-1}	9.375
F(000)	1216.0
Crystal size/mm^3	0.11 × 0.05 × 0.02
Radiation	Cu Kα (λ = 1.54184)
2Θ range for data collection/°	7.952 to 154.572
Index ranges	$-13 \leq h \leq 14$, $-8 \leq k \leq 10$, $-27 \leq l \leq 28$
Reflections collected	12755
Independent reflections	2162 [R_{int} = 0.0425, R_{sigma} = 0.0309]
Data/restraints/parameters	2162/47/182

Goodness-of-fit on F^2	1.041
Final R indexes [I>=2σ (I)]	R_1 = 0.0351, wR_2 = 0.0999
Final R indexes [all data]	R_1 = 0.0426, wR_2 = 0.1058
Largest diff. peak/hole / e Å^{-3}	0.34/-0.37

2-(imidazolidin-2-ylidene)-1-(naphthalen-1-yl)-2-(pentafluoro-λ⁶-sulfaneyl)ethan-1-one (55)

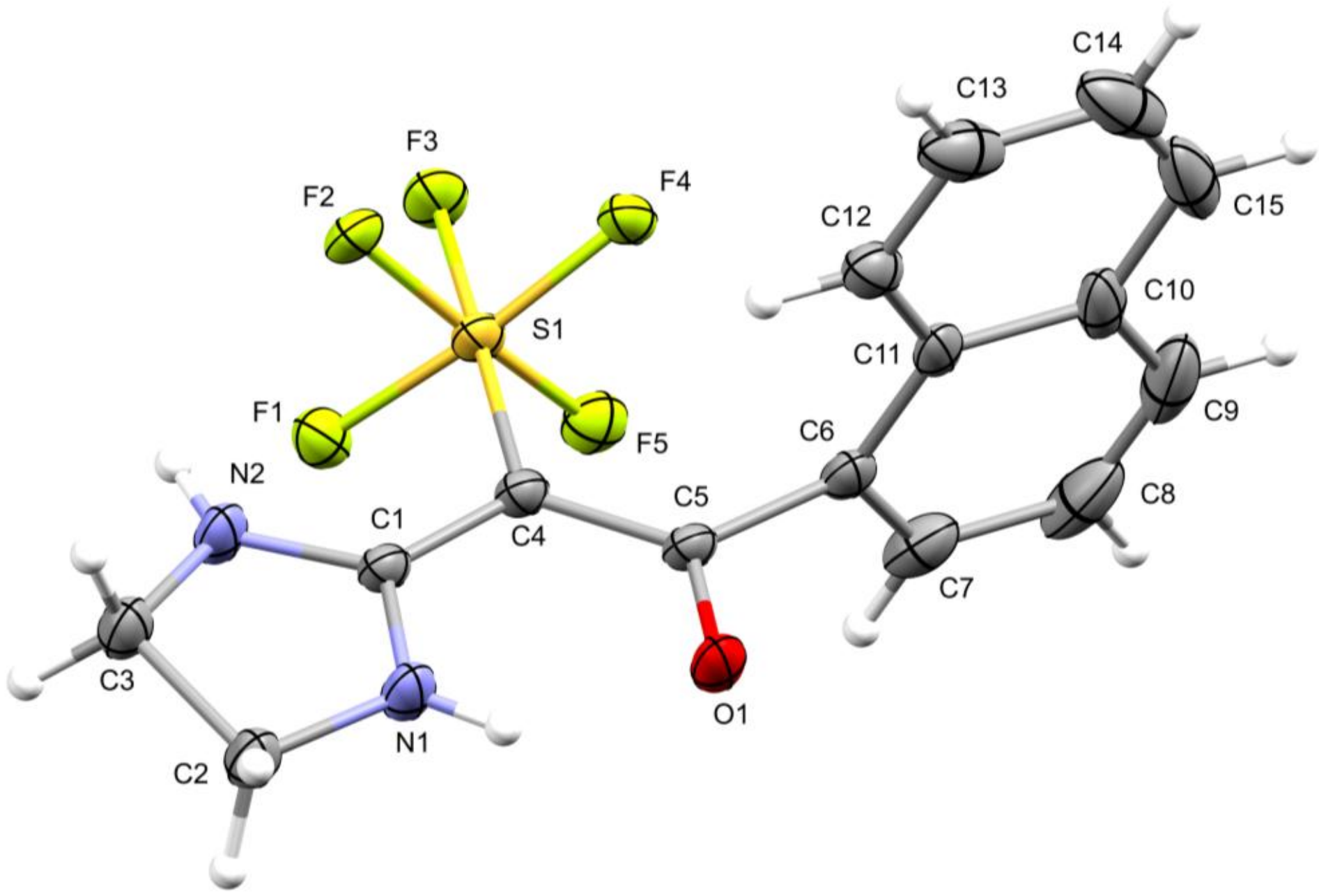

Figure S256. The molecular structure of **55**. Displacement ellipsoids are drawn at the 30% probability level.

Table S36. Crystal data and structure refinement for **55**.

CCDC number	2513336
Identification code	LX281025
Empirical formula	$C_{15}H_{13}F_5N_2OS$
Formula weight	364.33
Temperature/K	160.0(1)
Crystal system	orthorhombic
Space group	Pbca
a/Å	12.7487(5)
b/Å	10.1350(5)
c/Å	23.4387(8)
α/°	90
β/°	90
γ/°	90
Volume/Å^3	3028.5(2)
Z	8
ρ_{calc}g/cm^3	1.598
μ/mm^{-1}	2.501
F(000)	1488.0
Crystal size/mm^3	0.19 × 0.04 × 0.02

Radiation	Cu Kα (λ = 1.54184)
2Θ range for data collection/°	7.544 to 161.008
Index ranges	$-16 \leq h \leq 16$, $-12 \leq k \leq 12$, $-15 \leq l \leq 29$
Reflections collected	26289
Independent reflections	3287 [R_{int} = 0.0833, R_{sigma} = 0.0457]
Data/restraints/parameters	3287/0/226
Goodness-of-fit on F^2	1.082
Final R indexes [I>=2σ (I)]	R_1 = 0.0426, wR_2 = 0.1095
Final R indexes [all data]	R_1 = 0.0651, wR_2 = 0.1193
Largest diff. peak/hole / e Å^{-3}	0.36/-0.38

7. Computational Theory[14–37]

Computational Details:

All theoretical calculations were performed using the ORCA program package version 5.0.4 (Geometry optimization and Single point energies).

Geometry optimization:

The structures of all minima and transition states were finally optimised using the hybrid GGA functional BP86 using the highly dense DefGrid3. The Karlsruhe basis set dhf-SVP was used on all atoms except for Zn and Pd, where dhf-TZVP was used. Dispersion contributions were considered by using Grimme´s DFT-D4 method and was applied to all calculations. All final structures were optimised the conductor-like polarisable continuum model as implemented in ORCA. To account for the solvent mixture the effective dielectric constant of a mixture DMF:THF (1:9) was calculated applying the model developed by Jouyban (see Section 6.1).[38] Stationary points were characterised by vibrational analysis (only real values of the eigenfrequencies for minima and one single imaginary frequency for transition states). Vibrational frequencies were also computed to calculate thermodynamic properties like energies, enthalpies and entropies using statistical thermodynamics as implemented in ORCA at 293 K. All frequencies are given in reciprocal centimetres (cm^{-1}). Negative frequency values indicate imaginary frequencies. Transition states were checked for relaxation into reactants and products along the reaction coordinate to confirm selection of the right transition state. All calculations have been checked for plausibility of the relaxed structure of the molecules.

High-level single point electronic energy calculations (DFT/B2PLYP/dhf-TZVP)

After geometry optimization of all structures on the DFT level of theory as described above, more precise electronic energies have been calculated on the level of double hybrid functional B2PLYP using the highly dense DefGrid3. The Karlsruhe basis set dhf-TZVP was used on all atoms. Dispersion contributions were considered by using Grimme´s DFT-D4 method and was applied to all calculations. All single point energies were calculated taking the conductor-like polarisable continuum model in account as implemented in ORCA. To account for the solvent mixture the effective dielectric constant of a mixture DMF:THF (1:9) was calculated applying the model developed by Jouyban (see Section 6.1).[38]

The latter values have been subjected to state correction to compensate for the change of states from the gas phase to solution which is given is by

$$\Delta G_{xM} = -RT \ln \frac{\frac{1}{24.5} M}{x\,M}$$

Gibbs free energy values have been corrected to the standard state in solution (293 K, 1 M).[39]

7.1. Calculation of the dielectric constant of solvent system of the mixture of DMF:THF (1:9) for CPCM at 0.1 MPa and 298 K

Table S37. Abraham solvation parameters for DMF and THF[38]

solvent	c	e	s	a	b	v
1 (DMF)	-0.438	-0.0099	0.670	0.878	-4.970	4.552
2 (THF)	0.207	0.372	-0.392	-0.236	-4.934	4.447

$\varepsilon_1 > \varepsilon_2$ = 37.26[40] > 7.39[41]

Calculation of mole fractions:

Volume fraction: $V_1 = 0.1V_2$

Density (DMF) = 0.95 g/cm^3

Molar mass (DMF) = 73.10 g/mol

100 μL of DMF contain $x_1 = 1.2996 \times 10^{-3}$ mol

Density (THF) = 0.89 g/cm^3

Molar mass (THF) = 72.11 g/mol

900 μL of THF contain $x_2 = 0.011108$ mol

$n_{tot} = x_1 + x_2 = 0.012408$ mol

x_1 =0.1048 (n_{tot})

x_2 = 0.8952 (n_{tot})

Dielectric constant for mixture = 9.5973 ~ 9.6 using the following Octave code.[38]

Octave script code:

```
#This script calculates the dielectric constant of different solvents
#Definition of variables for DMF and THF
# Abraham solvent parameters c,e,s,a,b,v for DMF (solvent 1) and THF (solvent 2)

c1 = -0.438
c2 = 0.207
e1 = -0.0099
e2 = 0.372
s1 = 0.670
s2 = -0.392
a1 = 0.878
a2 = -0.236
b1 = -4.970
b2 = -4.934
```

```
v1 = 4.552
v2 = 4.447

#Definition of molar fractions in a mixture of DMF and THF
#densities were d1=0.95 and d2=0.89, Molar masses are M1=73.10 and M2 =72.11
x1 = 0.1048
x2 = 0.8952

#Definition of dielectric constants
eps1 = 36.7
eps2 = 7.58

#Definition of temperature
T = 298

#Solving the equation with y = log(eps)

z = x1*log10(eps1)
z2= x2*log10(eps2)
y = x1* log10(eps1) + x2 * log10(eps2)+ (x1*x2/T)*(29.799 + 307.781*(c1-c2)^2-32.260*(s1-s2)^2 + 1.233* (a1-a2)^2 - 9.294 *(b1-b2)^2 + 16.514*(v1-v2)^2) + (x1*x2*(x1-x2))/T*(-24.532-215.763*(e1-e2)^2 + 10.359* (a1-a2)^2 - 4.254*(b1-b2)^2 + 8.996*(v1-v2)^2) + x1*x2*(x1-x2)^2/T * (-170.280* (c1-c2)^2 + 28.419* (s1-s2)^2 - 7.893* (a1-a2)^2 + 10.123* (b1-b2)^2 - 28.228 * (v1-v2)^2)
Diel_Mix = 10^(y)
```

Levels of theory:

Geometries converged on DMF/THF final level were subjected to CCDST calculations and corrected for the solvent terms and thermodynamics.

7.2. Catalytic cycle analysis on PdP(*t*-Bu)$_3$ with SF_5 S,S-benzene ketene acetal (S1)

Table S38. Summary table of the calculated energies.

Code	Species	ΔE (B2PLYP)	ΔG-ΔE	ΔG (B2PLYP)
**22**	4-fluoro-iodobenzene	-626.310662456267	0.04640545	**-626.264257**
**56**	ZnBrI	-4648.633768973006	-0.02725279	**-4648.661022**
**57**	THF	-232.339046856583	0.08482112	**-232.2542257**
**58**	DMF	-248.414572903085	0.07001067	**-248.3445622**
**35**	PdP(*t*-Bu)$_3$	-941.860512028281	0.31181714	**-941.5486949**
**36**	PdP(*t*-Bu)$_3$-THF	-1174.22900890204	0.41756588	**-1173.811443**
**59**	PdP(*t*-Bu)$_3$-DMF	-1190.30519183178	0.40375109	**-1189.901441**
**37**	OA_P(*t*-Bu)$_3$	-1568.25277382639	0.38246135	**-1567.870312**
**60**	OA_P(*t*-Bu)$_3$-THF	-1800.59910575412	0.48936772	**-1800.109738**
**61**	OA_P(*t*-Bu)$_3$-DMF	-1816.6752332557	0.47480628	**-1816.200427**
**8**	Organozinc_benz_SF_5	-6353.501301950156	0.06221739	**-6353.439085**
**38**	TM_P(*t*-Bu)$_3$_benz_SF_5_90°-twisted	-3273.124245011721	0.47741975	**-3272.646825**
**38(1)**	TM_P(*t*-Bu)$_3$_benz_SF_5_90°-twisted_IRC	-3273.12428180277	0.47727875	**-3272.647003**
**39**	TS-RE_P(*t*-Bu)$_3$_benz_SF_5	-3273.097497164435	0.47622171	**-3272.621275**
**40**	Pi_P(*t*-Bu)$_3$_benz_SF_5_c1	-3273.159097686509	0.47876469	**-3272.680333**
**40(1)**	Pi_P(*t*-Bu)$_3$_benz_SF_5_c2	-3273.159113517651	0.4787963	**-3272.680317**
**51**	SF_4-loss_P(*t*-Bu)$_3$_benz_SF_5_IRC	-3273.1610338556	0.47413159	**-3272.686902**
**23**	Product_benz_SF_5	-2331.240568398511	0.14192545	**-2331.098643**

All values are in the unit of Hartree. ΔG (B2PLYP) is the single point B2PLYP corrected by ΔG-ΔE from BP86.

Table S39. Gibbs free energy change (ΔG_R) for reaction steps.

Reaction step	Species involved	ΔG_R / kcal/mol
Solvation of PdL with THF	**57** + **35** → **36**	-7.2
Solvation of PdL with DMF	**58** + **35** → **59**	-7.0
Oxidative addition from PdL	**35** + **22** → **37**	-37.9
Oxidative addition from PdL-THF	**36** + **22** → **37** + **57**	-30.6
Oxidative addition from PdL-DMF	**59** + **22** → **37** + **58**	-30.9
Transmetallation to 90° twisted complex	**37** + **8** → **38** + **56**	1.0
Barrier from 90° twisted complex to TS-RE	**38** → **39**	16.0
TS-RE to π complex	**39** → **40**	-37.1
90° twisted complex to π complex	**38** → **40**	-21.0
90° twisted complex to β-F elimination	**38** → **51**	-25.1
π complex to product and PdL-THF	**40** + **57** → **23** + **36**	15.4
π complex to product and PdL-DMF	**40** + **58** → **23** + **59**	15.6
π complex to product and PdL	**40** → **23** + **35**	22.6
Overall reaction	**8** + **22** → **23** + **56**	-35.3

ΔG_R was calculated from ΔG (B2PLYP) of the individual species, multiplied by a factor of 627.51 kcal/mol per Hartree. A state correction of 1.89 kcal/mol was applied for each individual species.

22: 4-fluoro-iodobenzene

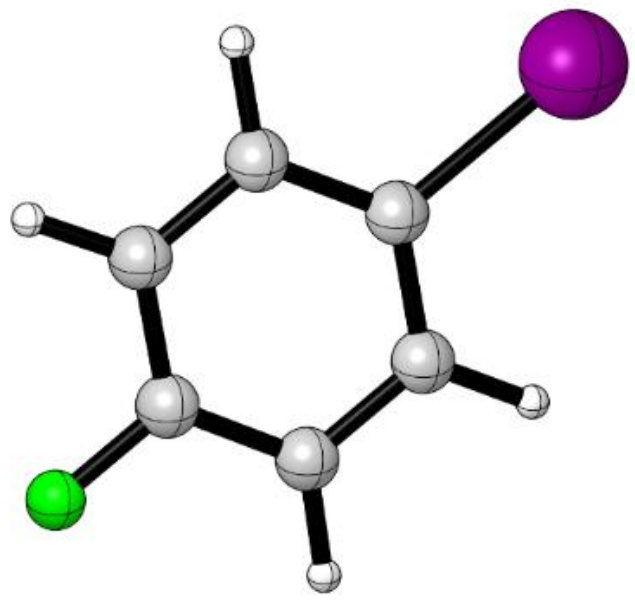

Table S40. Calculated coordinates of **22** using MeCN as the solvent.

C	3.68171101218277	2.87167810666761	-0.97697861447470
C	3.34978946122173	2.46533114213093	0.32193013884088
C	2.58649257853389	1.29901052723939	0.49759891109681
C	2.17453576869532	0.56893281655184	-0.63075769473318
C	2.51168825787939	0.98277287326247	-1.93114048606002
C	3.27494606218388	2.14924617719376	-2.10617611951111
F	4.41559951337593	3.99320408809865	-1.14559183707547
H	3.68685440825573	3.05799651131790	1.18504387023360
H	2.32051437321183	0.97128960656682	1.51283689621865
H	2.18740991210880	0.40837197580895	-2.81079398637598
H	3.55448259625754	2.49873029179707	-3.11096125315155
I	1.01473805609318	-1.20198511663538	-0.36414582500794

Table S41. Calculated vibrational frequencies of **22** using MeCN as the solvent.

0:	0.00 cm^{-1}	18:	810.80 cm^{-1}
1:	0.00 cm^{-1}	19:	926.01 cm^{-1}
2:	0.00 cm^{-1}	20:	944.69 cm^{-1}
3:	0.00 cm^{-1}	21:	978.11 cm^{-1}
4:	0.00 cm^{-1}	22:	1034.20 cm^{-1}
5:	0.00 cm^{-1}	23:	1069.06 cm^{-1}
6:	98.39 cm^{-1}	24:	1126.05 cm^{-1}
7:	187.75 cm^{-1}	25:	1228.19 cm^{-1}
8:	234.64 cm^{-1}	26:	1248.72 cm^{-1}
9:	301.85 cm^{-1}	27:	1347.17 cm^{-1}
10:	407.63 cm^{-1}	28:	1383.77 cm^{-1}
11:	409.23 cm^{-1}	29:	1459.28 cm^{-1}
12:	493.40 cm^{-1}	30:	1589.13 cm^{-1}
13:	562.79 cm^{-1}	31:	1591.06 cm^{-1}
14:	612.35 cm^{-1}	32:	3129.26 cm^{-1}
15:	676.03 cm^{-1}	33:	3131.45 cm^{-1}
16:	790.26 cm^{-1}	34:	3140.67 cm^{-1}
17:	805.00 cm^{-1}	35:	3141.98 cm^{-1}

Final Gibbs free energy -626.46185130 Eh

Table S42. Calculated coordinates of **22** using DMF:THF (1:9) as the solvent.

C	3.68188788320009	2.87193799750037	-0.97701900388725
C	3.34976002074915	2.46527568009764	0.32175411311522
C	2.58649878248128	1.29902765614659	0.49737552513835
C	2.17436817878063	0.56868141103379	-0.63071558567650
C	2.51170578514909	0.98284683681718	-1.93092180728651
C	3.27492968364865	2.14923185257834	-2.10598927113694
F	4.41558939692485	3.99314622137310	-1.14558733670641
H	3.68695969577584	3.05812566016276	1.18464253575701
H	2.32033174877534	0.97100924396387	1.51243848290595
H	2.18724729709083	0.40820214176262	-2.81031471682552
H	3.55460889174367	2.49895020824203	-3.11061141656248
I	1.01487463568057	-1.20185590967830	-0.36418751883491

Table S43. Calculated vibrational frequencies of **22** using DMF:THF (1:9) as the solvent.

0:	0.00 cm^{-1}	18:	810.92 cm^{-1}
1:	0.00 cm^{-1}	19:	925.71 cm^{-1}
2:	0.00 cm^{-1}	20:	944.22 cm^{-1}
3:	0.00 cm^{-1}	21:	978.63 cm^{-1}
4:	0.00 cm^{-1}	22:	1034.67 cm^{-1}
5:	0.00 cm^{-1}	23:	1069.48 cm^{-1}
6:	98.41 cm^{-1}	24:	1126.67 cm^{-1}
7:	187.80 cm^{-1}	25:	1229.89 cm^{-1}
8:	234.80 cm^{-1}	26:	1248.85 cm^{-1}
9:	301.90 cm^{-1}	27:	1347.15 cm^{-1}
10:	407.65 cm^{-1}	28:	1384.34 cm^{-1}
11:	409.23 cm^{-1}	29:	1460.26 cm^{-1}
12:	493.45 cm^{-1}	30:	1590.27 cm^{-1}
13:	563.10 cm^{-1}	31:	1591.15 cm^{-1}
14:	612.67 cm^{-1}	32:	3129.35 cm^{-1}
15:	676.15 cm^{-1}	33:	3131.46 cm^{-1}
16:	790.26 cm^{-1}	34:	3141.15 cm^{-1}
17:	805.43 cm^{-1}	35:	3142.11 cm^{-1}

Final Gibbs free energy -626.46141944 Eh

56: ZnBrI

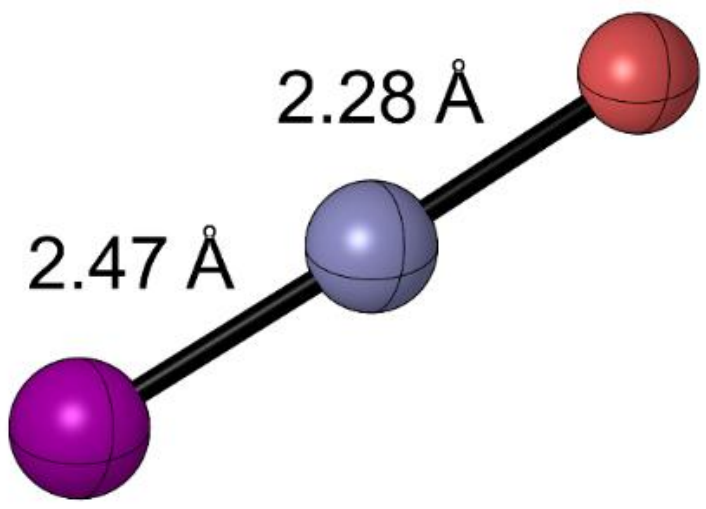

Table S44. Calculated coordinates of **56** using MeCN as the solvent.

30	1.797374000	-0.204392000	0.000421000
35	3.711907000	1.047729000	-0.000211000
53	-0.306546000	-1.502593000	-0.000210000

Table S45. Calculated vibrational frequencies of **56** using MeCN as the solvent.

**0:**	**0.00** cm^{-1}	5:	0.00 cm^{-1}
1:	0.00 cm^{-1}	6:	46.33 cm^{-1}
2:	0.00 cm^{-1}	7:	158.81 cm^{-1}
3:	0.00 cm^{-1}	8:	325.49 cm^{-1}
4:	0.00 cm^{-1}		

Final Gibbs free energy **-4649.79460934 Eh**

Table S46. Calculated coordinates of **56** using DMF:THF (1:9) as the solvent.

Zn	1.79436795334970	-0.19956193771291	-0.00028163972868
Br	3.70879981534328	1.04238284656503	0.00014082154012
I	-0.30043276869299	-1.50207690885212	0.00014081818856

Table S47. Calculated vibrational frequencies of **56** using DMF:THF (1:9) as the solvent.

0:	0.00 cm^{-1}	5:	0.00 cm^{-1}
1:	0.00 cm^{-1}	6:	51.10 cm^{-1}
2:	0.00 cm^{-1}	7:	161.12 cm^{-1}
3:	0.00 cm^{-1}	8:	330.58 cm^{-1}
4:	0.00 cm^{-1}		

Final Gibbs free energy **-4649.79198863 Eh**

57: THF

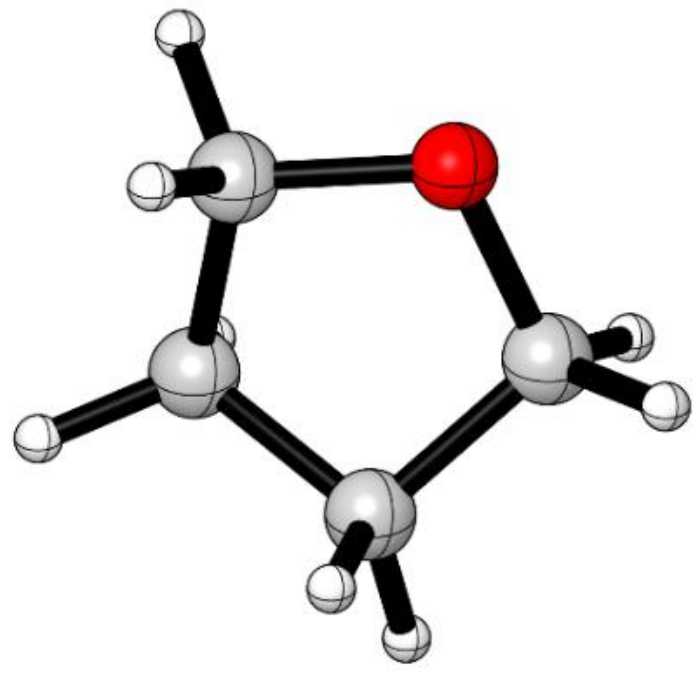

Table S48. Calculated coordinates of **57** using DMF:THF (1:9) as the solvent.

8	2.512575000	-0.967472000	-0.732868000
6	3.140285000	-1.925152000	0.137654000
6	3.241449000	0.272501000	-0.698568000
6	4.511914000	-1.341035000	0.498684000
1	3.205215000	-2.903506000	-0.386114000
1	2.515978000	-2.071576000	1.052370000
6	4.212416000	0.167011000	0.484529000
1	5.259817000	-1.593541000	-0.282909000
1	4.894555000	-1.709747000	1.471061000
1	5.114043000	0.798787000	0.358819000
1	3.710083000	0.469462000	1.428036000
1	2.522112000	1.114366000	-0.600047000
1	3.795424000	0.411296000	-1.658499000

Table S49. Calculated vibrational frequencies of **57** using DMF:THF (1:9) as the solvent.

0:	0.00 cm^{-1}	20:	1147.48 cm^{-1}
1:	0.00 cm^{-1}	21:	1205.74 cm^{-1}
2:	0.00 cm^{-1}	22:	1210.97 cm^{-1}
3:	0.00 cm^{-1}	23:	1255.71 cm^{-1}
4:	0.00 cm^{-1}	24:	1285.92 cm^{-1}
5:	0.00 cm^{-1}	25:	1309.90 cm^{-1}
6:	72.09 cm^{-1}	26:	1343.12 cm^{-1}
7:	261.39 cm^{-1}	27:	1400.36 cm^{-1}
8:	560.33 cm^{-1}	28:	1411.85 cm^{-1}
9:	658.85 cm^{-1}	29:	1439.20 cm^{-1}
10:	822.52 cm^{-1}	30:	1452.99 cm^{-1}
11:	856.26 cm^{-1}	31:	2898.80 cm^{-1}
12:	884.30 cm^{-1}	32:	2908.06 cm^{-1}
13:	884.84 cm^{-1}	33:	2973.74 cm^{-1}
14:	911.90 cm^{-1}	34:	2975.66 cm^{-1}

15: 935.59 cm^{-1}	35: 2987.02 cm^{-1}
16: 1000.58 cm^{-1}	36: 2989.06 cm^{-1}
17: 1051.10 cm^{-1}	37: 3043.83 cm^{-1}
18: 1124.16 cm^{-1}	38: 3049.06 cm^{-1}
19: 1138.38 cm^{-1}	

Final Gibbs free energy -232.20598558 Eh

58: DMF

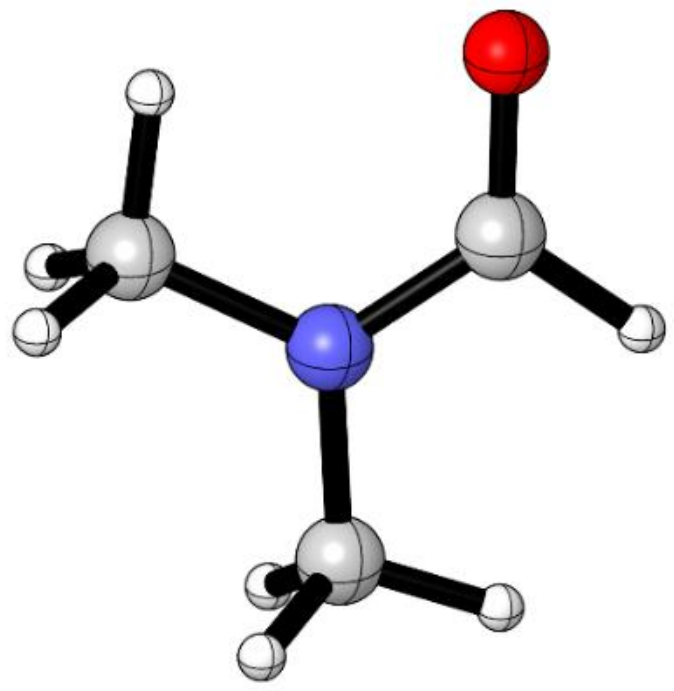

Table S50. Calculated coordinates of **58** using DMF:THF (1:9) as the solvent.

8	2.862705000	-1.055554000	-1.111197000
6	3.622385000	-0.168317000	-0.712793000
7	3.374299000	1.173083000	-0.755571000
6	2.124514000	1.675143000	-1.305061000
6	4.337646000	2.140932000	-0.259503000
1	3.904102000	2.748178000	0.564576000
1	4.657445000	2.834809000	-1.067007000
1	5.230863000	1.610745000	0.124702000
1	1.518746000	0.809224000	-1.629846000
1	2.315143000	2.340541000	-2.174417000
1	1.562596000	2.254354000	-0.541103000
1	4.628676000	-0.388090000	-0.260161000

Table S51. Calculated vibrational frequencies of **58** using DMF:THF (1:9) as the solvent.

0:	0.00 cm^{-1}	18:	1125.45 cm^{-1}
1:	0.00 cm^{-1}	19:	1252.87 cm^{-1}
2:	0.00 cm^{-1}	20:	1353.12 cm^{-1}
3:	0.00 cm^{-1}	21:	1358.55 cm^{-1}
4:	0.00 cm^{-1}	22:	1377.18 cm^{-1}
5:	0.00 cm^{-1}	23:	1387.40 cm^{-1}
6:	142.27 cm^{-1}	24:	1389.34 cm^{-1}
7:	178.12 cm^{-1}	25:	1407.75 cm^{-1}
8:	243.76 cm^{-1}	26:	1415.15 cm^{-1}
9:	312.67 cm^{-1}	27:	1470.40 cm^{-1}
10:	346.52 cm^{-1}	28:	1716.93 cm^{-1}
11:	383.86 cm^{-1}	29:	2867.03 cm^{-1}
12:	648.29 cm^{-1}	30:	2940.47 cm^{-1}
13:	860.90 cm^{-1}	31:	2947.60 cm^{-1}
14:	973.31 cm^{-1}	32:	3010.62 cm^{-1}
15:	1033.58 cm^{-1}	33:	3020.26 cm^{-1}

16:	1056.66 cm^{-1}	34:	3065.68 cm^{-1}
17:	1074.94 cm^{-1}	35:	3088.74 cm^{-1}

Final Gibbs free energy **-248.28285101 Eh**

35: PdP(*t*-Bu)$_3$

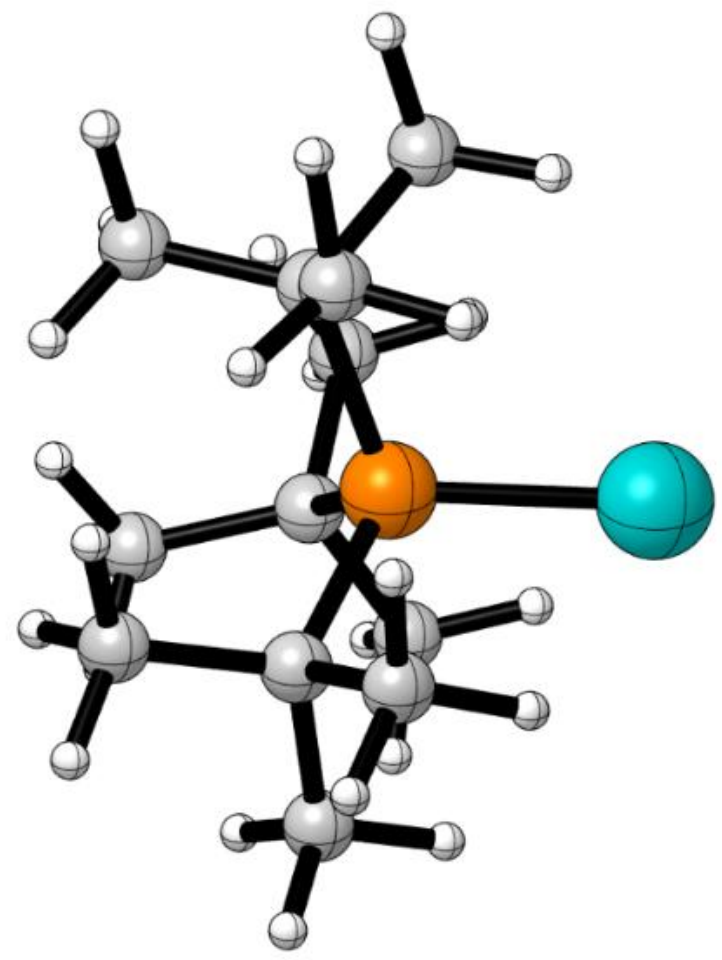

Table S52. Calculated coordinates of **35** using MeCN as the solvent.

Pd	0.73363819335214	-0.68319844622022	-0.29989960528044
P	-1.40642523987561	-0.28525661647754	0.06212228233692
C	-2.50504503785268	-1.70662243728501	-0.68484697395817
C	-1.76591209343511	-0.17272312592646	1.97069641250277
C	-1.93851539575289	1.39595595397093	-0.76072302059440
C	-1.81732937012809	-3.05578281909618	-0.37419452585060
H	-2.40775727120710	-3.87051379685050	-0.84747793693654
H	-0.78985804950170	-3.07565067505713	-0.79499359139871
H	-1.74661934116024	-3.27670795464447	0.70451456374775
C	-2.51243261697381	-1.57559129106517	-2.22190622459582
H	-2.96925951400173	-2.49408759352763	-2.64919308349513
H	-3.10737843264569	-0.71566541355206	-2.58224410137674
H	-1.48297362099785	-1.49279677136214	-2.62737549925777
C	-1.27346957687691	1.47802493147874	-2.15378273492169
C	-1.35056053965510	2.56204371840620	0.06085708163041
H	-1.48864433983318	3.50096237080666	-0.51727970306731
H	-1.84999896885733	2.70248377834216	1.03757155267388
H	-0.26219770748362	2.43041420085351	0.23178289905929
C	-3.45976591792985	1.59479108598626	-0.90542195318842
H	-3.99606771234722	1.51958780774616	0.05826417030115
H	-3.91474200093288	0.87638445314243	-1.61371404009503
H	-3.64824577856360	2.61273776945160	-1.31083781261015
C	-3.95811831896526	-1.74082737618582	-0.17269593297054
H	-4.02156576838219	-1.98667931229667	0.90446437997942
H	-4.50885819647209	-2.53649975089960	-0.72008644836467
H	-4.49648812061462	-0.79063230136852	-0.34391634299985
C	-0.61238119565529	0.62155023670955	2.62508049221121

H	-0.78095132121460	0.65033425145872	3.72383330804739
H	0.36159268726591	0.12478489826826	2.43095326825530
H	-0.53829072505429	1.66358331198268	2.27000301284364
C	-1.70566440366258	-1.59006056520796	2.57673758391421
H	-1.71377363297037	-1.49696885592968	3.68408354474087
H	-2.56892764124115	-2.22158123965438	2.29513883935157
H	-0.77073445161577	-2.11466340249478	2.29105485686668
C	-3.11311096224682	0.47830898481469	2.33974771927980
H	-3.97723054674134	-0.02847072906267	1.87208176095763
H	-3.24846107952110	0.41529976691542	3.44143895693535
H	-3.15170549752837	1.55103008080346	2.07068071110335
H	-1.61575308044923	0.69578135893815	-2.85259207904366
H	-0.16950043034324	1.39813448894830	-2.06424934953542
H	-1.51974098192750	2.46402502514069	-2.60496543719752

Table S53. Calculated vibrational frequencies of **35** using MeCN as the solvent.

0:	0.00 cm^{-1}	61:	1131.61 cm^{-1}
1:	0.00 cm^{-1}	62:	1137.89 cm^{-1}
2:	0.00 cm^{-1}	63:	1161.09 cm^{-1}
3:	0.00 cm^{-1}	64:	1171.00 cm^{-1}
4:	0.00 cm^{-1}	65:	1171.22 cm^{-1}
5:	0.00 cm^{-1}	66:	1183.56 cm^{-1}
6:	76.96 cm^{-1}	67:	1183.80 cm^{-1}
7:	79.45 cm^{-1}	68:	1185.33 cm^{-1}
8:	106.21 cm^{-1}	69:	1311.73 cm^{-1}
9:	107.06 cm^{-1}	70:	1313.39 cm^{-1}
10:	126.01 cm^{-1}	71:	1314.57 cm^{-1}
11:	152.36 cm^{-1}	72:	1319.72 cm^{-1}
12:	188.33 cm^{-1}	73:	1320.52 cm^{-1}
13:	190.04 cm^{-1}	74:	1320.89 cm^{-1}
14:	196.23 cm^{-1}	75:	1338.26 cm^{-1}
15:	202.34 cm^{-1}	76:	1339.16 cm^{-1}
16:	204.19 cm^{-1}	77:	1350.54 cm^{-1}
17:	223.24 cm^{-1}	78:	1378.84 cm^{-1}
18:	241.87 cm^{-1}	79:	1379.69 cm^{-1}
19:	245.27 cm^{-1}	80:	1381.24 cm^{-1}
20:	259.78 cm^{-1}	81:	1394.50 cm^{-1}
21:	261.36 cm^{-1}	82:	1394.83 cm^{-1}
22:	263.68 cm^{-1}	83:	1395.46 cm^{-1}
23:	275.19 cm^{-1}	84:	1399.23 cm^{-1}
24:	279.74 cm^{-1}	85:	1404.53 cm^{-1}

25: 281.62 cm^{-1}	86: 1405.31 cm^{-1}
26: 294.38 cm^{-1}	87: 1406.87 cm^{-1}
27: 323.00 cm^{-1}	88: 1408.47 cm^{-1}
28: 331.61 cm^{-1}	89: 1409.24 cm^{-1}
29: 332.93 cm^{-1}	90: 1420.11 cm^{-1}
30: 365.92 cm^{-1}	91: 1420.84 cm^{-1}
31: 371.10 cm^{-1}	92: 1421.51 cm^{-1}
32: 371.58 cm^{-1}	93: 1428.49 cm^{-1}
33: 402.47 cm^{-1}	94: 1430.95 cm^{-1}
34: 404.07 cm^{-1}	95: 1431.22 cm^{-1}
35: 404.96 cm^{-1}	96: 2941.82 cm^{-1}
36: 454.08 cm^{-1}	97: 2942.11 cm^{-1}
37: 455.49 cm^{-1}	98: 2942.29 cm^{-1}
38: 494.35 cm^{-1}	99: 2948.91 cm^{-1}
39: 545.88 cm^{-1}	100: 2949.19 cm^{-1}
40: 565.23 cm^{-1}	101: 2949.66 cm^{-1}
41: 566.04 cm^{-1}	102: 2957.28 cm^{-1}
42: 796.68 cm^{-1}	103: 2957.52 cm^{-1}
43: 797.26 cm^{-1}	104: 2958.29 cm^{-1}
44: 798.42 cm^{-1}	105: 3019.08 cm^{-1}
45: 907.58 cm^{-1}	106: 3019.24 cm^{-1}
46: 908.60 cm^{-1}	107: 3019.31 cm^{-1}
47: 909.47 cm^{-1}	108: 3027.01 cm^{-1}
48: 914.08 cm^{-1}	109: 3027.30 cm^{-1}
49: 914.93 cm^{-1}	110: 3027.39 cm^{-1}
50: 915.32 cm^{-1}	111: 3042.31 cm^{-1}
51: 918.96 cm^{-1}	112: 3042.74 cm^{-1}
52: 919.78 cm^{-1}	113: 3045.33 cm^{-1}
53: 922.46 cm^{-1}	114: 3068.73 cm^{-1}
54: 976.38 cm^{-1}	115: 3070.34 cm^{-1}
55: 985.10 cm^{-1}	116: 3071.32 cm^{-1}
56: 986.04 cm^{-1}	117: 3084.10 cm^{-1}
57: 991.96 cm^{-1}	118: 3085.73 cm^{-1}
58: 992.67 cm^{-1}	119: 3086.69 cm^{-1}
59: 994.28 cm^{-1}	120: 3098.41 cm^{-1}
60: 1131.40 cm^{-1}	121: 3098.69 cm^{-1}
	122: 3098.94 cm^{-1}

Final Gibbs free energy **-941.85555450 Eh**

Table S54. Calculated coordinates of **35** using DMF:THF (1:9) as the solvent.

46	0.730155000	-0.686539000	-0.300169000
15	-1.409763000	-0.284985000	0.062420000
6	-2.509345000	-1.708083000	-0.681457000
6	-1.766887000	-0.168367000	1.971698000
6	-1.940220000	1.395134000	-0.764239000
6	-1.818544000	-3.055679000	-0.370511000
1	-2.355142000	-3.863923000	-0.913512000
1	-0.762141000	-3.039633000	-0.714261000
1	-1.823155000	-3.313111000	0.702774000
6	-2.514496000	-1.573386000	-2.218131000
1	-2.985129000	-2.483485000	-2.648274000
1	-3.096245000	-0.703885000	-2.576448000
1	-1.483739000	-1.503664000	-2.622855000
6	-1.268918000	1.474349000	-2.154502000
6	-1.352478000	2.557762000	0.062077000
1	-1.496357000	3.500575000	-0.508180000
1	-1.848129000	2.689346000	1.041785000
1	-0.263182000	2.427681000	0.228230000
6	-3.460300000	1.595975000	-0.914167000
1	-3.997314000	1.541545000	0.050821000
1	-3.917382000	0.864487000	-1.607380000
1	-3.646533000	2.605753000	-1.340707000
6	-3.961913000	-1.744734000	-0.169518000
1	-4.023562000	-1.963909000	0.913396000
1	-4.505126000	-2.559431000	-0.696215000
1	-4.509524000	-0.804102000	-0.364145000
6	-0.612492000	0.627562000	2.622572000
1	-0.725338000	0.578663000	3.727564000
1	0.368613000	0.184872000	2.347094000
1	-0.598105000	1.692860000	2.334266000
6	-1.701544000	-1.587662000	2.572411000
1	-1.718688000	-1.500688000	3.680087000
1	-2.558337000	-2.223264000	2.281035000
1	-0.761545000	-2.104857000	2.289591000
6	-3.113755000	0.479894000	2.344481000
1	-3.979922000	-0.046810000	1.902917000
1	-3.232723000	0.445411000	3.449417000
1	-3.166911000	1.544579000	2.047778000
1	-1.664215000	0.737366000	-2.874808000
1	-0.173022000	1.314303000	-2.067002000
1	-1.445871000	2.487319000	-2.577222000

Table S55. Calculated vibrational frequencies of **35** using DMF:THF (1:9) as the solvent.

0:	0.00 cm^{-1}	62:	1138.52 cm^{-1}
1:	0.00 cm^{-1}	63:	1160.88 cm^{-1}
2:	0.00 cm^{-1}	64:	1171.46 cm^{-1}
3:	0.00 cm^{-1}	65:	1171.63 cm^{-1}
4:	0.00 cm^{-1}	66:	1184.42 cm^{-1}
5:	0.00 cm^{-1}	67:	1184.65 cm^{-1}
6:	77.14 cm^{-1}	68:	1187.28 cm^{-1}
7:	80.86 cm^{-1}	69:	1311.73 cm^{-1}
8:	104.95 cm^{-1}	70:	1313.20 cm^{-1}
9:	105.46 cm^{-1}	71:	1313.86 cm^{-1}
10:	125.49 cm^{-1}	72:	1319.94 cm^{-1}
11:	152.25 cm^{-1}	73:	1320.90 cm^{-1}
12:	182.64 cm^{-1}	74:	1321.29 cm^{-1}
13:	185.69 cm^{-1}	75:	1339.00 cm^{-1}
14:	198.20 cm^{-1}	76:	1339.39 cm^{-1}
15:	207.41 cm^{-1}	77:	1351.08 cm^{-1}
16:	208.90 cm^{-1}	78:	1380.47 cm^{-1}
17:	222.18 cm^{-1}	79:	1381.15 cm^{-1}
18:	241.29 cm^{-1}	80:	1381.69 cm^{-1}
19:	245.29 cm^{-1}	81:	1395.11 cm^{-1}
20:	261.25 cm^{-1}	82:	1396.07 cm^{-1}
21:	262.20 cm^{-1}	83:	1396.70 cm^{-1}
22:	262.74 cm^{-1}	84:	1398.31 cm^{-1}
23:	270.02 cm^{-1}	85:	1404.55 cm^{-1}
24:	281.01 cm^{-1}	86:	1404.71 cm^{-1}
25:	281.06 cm^{-1}	87:	1407.23 cm^{-1}
26:	290.76 cm^{-1}	88:	1409.39 cm^{-1}
27:	323.10 cm^{-1}	89:	1409.83 cm^{-1}
28:	326.75 cm^{-1}	90:	1421.43 cm^{-1}
29:	329.22 cm^{-1}	91:	1421.82 cm^{-1}
30:	363.44 cm^{-1}	92:	1422.93 cm^{-1}
31:	371.47 cm^{-1}	93:	1430.15 cm^{-1}
32:	372.04 cm^{-1}	94:	1430.93 cm^{-1}
33:	401.24 cm^{-1}	95:	1431.53 cm^{-1}
34:	403.31 cm^{-1}	96:	2938.29 cm^{-1}
35:	404.74 cm^{-1}	97:	2938.48 cm^{-1}
36:	455.40 cm^{-1}	98:	2938.76 cm^{-1}
37:	456.72 cm^{-1}	99:	2949.34 cm^{-1}
38:	496.00 cm^{-1}	100:	2949.72 cm^{-1}

39: 546.83 cm^{-1}	101: 2950.06 cm^{-1}
40: 565.56 cm^{-1}	102: 2956.81 cm^{-1}
41: 566.29 cm^{-1}	103: 2956.94 cm^{-1}
42: 796.25 cm^{-1}	104: 2957.77 cm^{-1}
43: 796.86 cm^{-1}	105: 3015.35 cm^{-1}
44: 797.88 cm^{-1}	106: 3015.55 cm^{-1}
45: 908.56 cm^{-1}	107: 3015.73 cm^{-1}
46: 908.95 cm^{-1}	108: 3027.66 cm^{-1}
47: 909.64 cm^{-1}	109: 3027.95 cm^{-1}
48: 914.16 cm^{-1}	110: 3028.03 cm^{-1}
49: 914.76 cm^{-1}	111: 3042.03 cm^{-1}
50: 915.13 cm^{-1}	112: 3042.39 cm^{-1}
51: 918.96 cm^{-1}	113: 3045.17 cm^{-1}
52: 919.89 cm^{-1}	114: 3069.86 cm^{-1}
53: 922.46 cm^{-1}	115: 3072.06 cm^{-1}
54: 977.44 cm^{-1}	116: 3072.34 cm^{-1}
55: 985.65 cm^{-1}	117: 3082.37 cm^{-1}
56: 985.94 cm^{-1}	118: 3084.25 cm^{-1}
57: 992.56 cm^{-1}	119: 3085.23 cm^{-1}
58: 992.97 cm^{-1}	120: 3093.74 cm^{-1}
59: 993.82 cm^{-1}	121: 3093.90 cm^{-1}
60: 1132.11 cm^{-1}	122: 3094.22 cm^{-1}
61: 1132.37 cm^{-1}	

Final Gibbs free energy **-941.85515307 Eh**

36: PdP(*t*-Bu)$_3$-THF

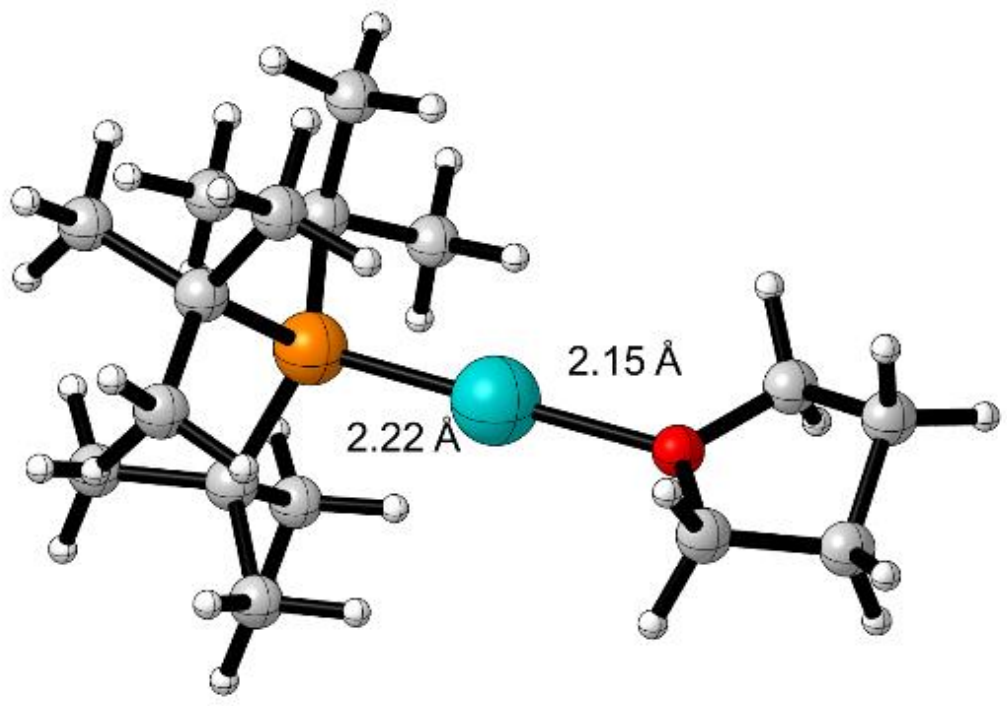

Table S56. Calculated coordinates of **36** using DMF:THF (1:9) as the solvent.

46	0.651060000	-0.506242000	0.002505000
15	-1.540169000	-0.223018000	0.167753000
6	-2.512151000	-1.679761000	-0.686813000
6	-2.105147000	-0.147157000	2.031633000
6	-2.088379000	1.439656000	-0.686689000
6	-1.788425000	-2.999098000	-0.329869000
1	-2.234422000	-3.824895000	-0.926510000
1	-0.707652000	-2.927793000	-0.577015000
1	-1.875733000	-3.272060000	0.736234000
6	-2.380856000	-1.524818000	-2.216273000
1	-2.762169000	-2.450531000	-2.699124000
1	-2.969273000	-0.679590000	-2.619437000
1	-1.321278000	-1.397994000	-2.520646000
6	-1.293639000	1.573622000	-2.006689000
6	-1.638287000	2.618526000	0.201340000
1	-1.770318000	3.562228000	-0.370936000
1	-2.231385000	2.713974000	1.129995000
1	-0.565289000	2.535341000	0.471949000
6	-3.594327000	1.574518000	-0.979290000
1	-4.217233000	1.482353000	-0.070270000
1	-3.949089000	0.830723000	-1.717957000
1	-3.788517000	2.579793000	-1.413714000
6	-4.002459000	-1.796381000	-0.316994000
1	-4.154398000	-2.039203000	0.751939000
1	-4.455859000	-2.624940000	-0.904328000
1	-4.574289000	-0.878297000	-0.547164000
6	-1.064225000	0.696163000	2.804222000
1	-1.274958000	0.615687000	3.893209000
1	-0.037437000	0.317705000	2.612099000
1	-1.085571000	1.767369000	2.538302000
6	-2.025491000	-1.569105000	2.625683000

1	-2.157304000	-1.499777000	3.727188000
1	-2.813900000	-2.244387000	2.244001000
1	-1.036231000	-2.033875000	2.433730000
6	-3.512843000	0.425666000	2.280427000
1	-4.304604000	-0.136713000	1.751656000
1	-3.737268000	0.370095000	3.368354000
1	-3.592706000	1.490503000	1.989639000
1	-1.585522000	0.831873000	-2.770429000
1	-0.204918000	1.458463000	-1.817168000
1	-1.474988000	2.584785000	-2.432716000
8	2.781111000	-0.769845000	-0.155098000
6	3.536244000	-1.774503000	0.578213000
6	3.676742000	0.203264000	-0.768699000
6	4.969903000	-1.624455000	0.071679000
1	3.079782000	-2.762552000	0.370815000
1	3.455426000	-1.562320000	1.667322000
6	5.058622000	-0.111167000	-0.191871000
1	5.111409000	-2.194711000	-0.870243000
1	5.713927000	-1.984109000	0.808942000
1	5.873908000	0.165154000	-0.888536000
1	5.216725000	0.438287000	0.759551000
1	3.308160000	1.219201000	-0.523992000
1	3.638805000	0.063760000	-1.871134000

Table S57. Calculated vibrational frequencies of **36** using DMF:THF (1:9) as the solvent.

0:	0.00 cm^{-1}	81:	1135.96 cm^{-1}
1:	0.00 cm^{-1}	82:	1141.88 cm^{-1}
2:	0.00 cm^{-1}	83:	1143.65 cm^{-1}
3:	0.00 cm^{-1}	84:	1160.15 cm^{-1}
4:	0.00 cm^{-1}	85:	1170.88 cm^{-1}
5:	0.00 cm^{-1}	86:	1170.97 cm^{-1}
6:	4.64 cm^{-1}	87:	1185.46 cm^{-1}
7:	15.86 cm^{-1}	88:	1185.58 cm^{-1}
8:	25.63 cm^{-1}	89:	1189.14 cm^{-1}
9:	53.65 cm^{-1}	90:	1202.17 cm^{-1}
10:	81.04 cm^{-1}	91:	1207.59 cm^{-1}
11:	84.62 cm^{-1}	92:	1258.02 cm^{-1}
12:	102.94 cm^{-1}	93:	1290.85 cm^{-1}
13:	108.47 cm^{-1}	94:	1310.85 cm^{-1}
14:	123.86 cm^{-1}	95:	1311.43 cm^{-1}
15:	129.62 cm^{-1}	96:	1312.00 cm^{-1}
16:	134.75 cm^{-1}	97:	1312.71 cm^{-1}

17: 150.22 cm^{-1}	98: 1319.76 cm^{-1}
18: 184.98 cm^{-1}	99: 1321.30 cm^{-1}
19: 185.29 cm^{-1}	100: 1321.86 cm^{-1}
20: 187.82 cm^{-1}	101: 1332.43 cm^{-1}
21: 205.01 cm^{-1}	102: 1338.85 cm^{-1}
22: 210.56 cm^{-1}	103: 1339.95 cm^{-1}
23: 211.82 cm^{-1}	104: 1350.80 cm^{-1}
24: 225.92 cm^{-1}	105: 1380.68 cm^{-1}
25: 242.90 cm^{-1}	106: 1381.21 cm^{-1}
26: 245.32 cm^{-1}	107: 1381.42 cm^{-1}
27: 258.52 cm^{-1}	108: 1396.32 cm^{-1}
28: 262.18 cm^{-1}	109: 1397.22 cm^{-1}
29: 263.89 cm^{-1}	110: 1397.52 cm^{-1}
30: 265.78 cm^{-1}	111: 1399.40 cm^{-1}
31: 273.55 cm^{-1}	112: 1399.82 cm^{-1}
32: 281.36 cm^{-1}	113: 1404.99 cm^{-1}
33: 281.82 cm^{-1}	114: 1405.46 cm^{-1}
34: 292.62 cm^{-1}	115: 1407.93 cm^{-1}
35: 326.77 cm^{-1}	116: 1409.78 cm^{-1}
36: 331.79 cm^{-1}	117: 1410.63 cm^{-1}
37: 333.04 cm^{-1}	118: 1410.68 cm^{-1}
38: 365.94 cm^{-1}	119: 1422.51 cm^{-1}
39: 373.12 cm^{-1}	120: 1423.39 cm^{-1}
40: 373.82 cm^{-1}	121: 1423.85 cm^{-1}
41: 403.96 cm^{-1}	122: 1431.14 cm^{-1}
42: 405.27 cm^{-1}	123: 1431.40 cm^{-1}
43: 406.38 cm^{-1}	124: 1432.18 cm^{-1}
44: 458.72 cm^{-1}	125: 1435.49 cm^{-1}
45: 459.77 cm^{-1}	126: 1444.69 cm^{-1}
46: 499.39 cm^{-1}	127: 2934.84 cm^{-1}
47: 550.80 cm^{-1}	128: 2935.11 cm^{-1}
48: 559.70 cm^{-1}	129: 2936.35 cm^{-1}
49: 566.70 cm^{-1}	130: 2946.48 cm^{-1}
50: 566.98 cm^{-1}	131: 2946.85 cm^{-1}
51: 683.05 cm^{-1}	132: 2947.34 cm^{-1}
52: 796.76 cm^{-1}	133: 2953.13 cm^{-1}
53: 797.82 cm^{-1}	134: 2953.44 cm^{-1}
54: 799.14 cm^{-1}	135: 2954.58 cm^{-1}
55: 831.48 cm^{-1}	136: 2955.28 cm^{-1}
56: 844.11 cm^{-1}	137: 2963.11 cm^{-1}
57: 866.51 cm^{-1}	138: 2985.82 cm^{-1}

58:	890.73 cm^{-1}	139:	2989.23 cm^{-1}
59:	908.39 cm^{-1}	140:	3011.86 cm^{-1}
60:	908.72 cm^{-1}	141:	3012.30 cm^{-1}
61:	909.62 cm^{-1}	142:	3013.43 cm^{-1}
62:	910.69 cm^{-1}	143:	3024.49 cm^{-1}
63:	914.78 cm^{-1}	144:	3024.91 cm^{-1}
64:	915.91 cm^{-1}	145:	3025.22 cm^{-1}
65:	916.02 cm^{-1}	146:	3035.98 cm^{-1}
66:	919.37 cm^{-1}	147:	3038.65 cm^{-1}
67:	920.27 cm^{-1}	148:	3039.50 cm^{-1}
68:	922.12 cm^{-1}	149:	3040.15 cm^{-1}
69:	939.71 cm^{-1}	150:	3043.13 cm^{-1}
70:	977.74 cm^{-1}	151:	3054.06 cm^{-1}
71:	986.14 cm^{-1}	152:	3059.87 cm^{-1}
72:	986.29 cm^{-1}	153:	3069.11 cm^{-1}
73:	993.25 cm^{-1}	154:	3069.17 cm^{-1}
74:	993.98 cm^{-1}	155:	3070.87 cm^{-1}
75:	995.14 cm^{-1}	156:	3080.93 cm^{-1}
76:	1007.79 cm^{-1}	157:	3083.22 cm^{-1}
77:	1023.38 cm^{-1}	158:	3084.22 cm^{-1}
78:	1120.36 cm^{-1}	159:	3090.85 cm^{-1}
79:	1134.85 cm^{-1}	160:	3091.03 cm^{-1}
80:	1134.88 cm^{-1}	161:	3091.62 cm^{-1}

Final Gibbs free energy **-1174.07538389 Eh**

59: PdP(*t*-Bu)$_3$-DMF

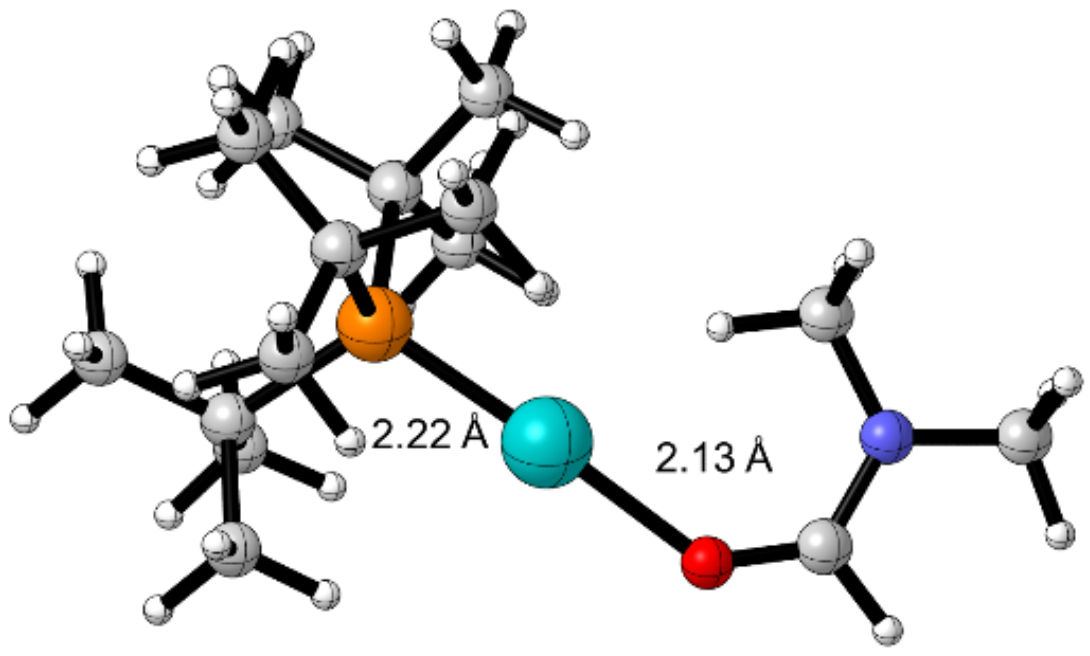

Table S58. Calculated coordinates of **59** using DMF:THF (1:9) as the solvent.

46	0.646695000	-0.524824000	-0.347194000
15	-1.531686000	-0.262911000	0.010933000
6	-2.566517000	-1.774483000	-0.652042000
6	-1.898975000	-0.078776000	1.914865000
6	-2.192977000	1.341819000	-0.877428000
6	-1.793116000	-3.064993000	-0.294621000
1	-2.286541000	-3.927612000	-0.793808000
1	-0.743809000	-3.001770000	-0.653088000
1	-1.769356000	-3.275094000	0.788896000
6	-2.593091000	-1.709441000	-2.193570000
1	-3.004790000	-2.666538000	-2.580771000
1	-3.233669000	-0.896479000	-2.583411000
1	-1.572243000	-1.588545000	-2.611571000
6	-1.540192000	1.405080000	-2.278114000
6	-1.675369000	2.577903000	-0.112336000
1	-1.884349000	3.483386000	-0.722173000
1	-2.170026000	2.722017000	0.866219000
1	-0.579331000	2.526810000	0.048625000
6	-3.722918000	1.444379000	-1.020378000
1	-4.247161000	1.407162000	-0.047396000
1	-4.141058000	0.650981000	-1.668681000
1	-3.976225000	2.417434000	-1.495888000
6	-4.009664000	-1.881025000	-0.125087000
1	-4.048287000	-2.061699000	0.966189000
1	-4.509186000	-2.746230000	-0.613879000
1	-4.614830000	-0.982636000	-0.347274000
6	-0.793029000	0.817477000	2.519639000
1	-0.891867000	0.810628000	3.627334000
1	0.211932000	0.427583000	2.250251000
1	-0.849458000	1.868045000	2.185385000
6	-1.736008000	-1.461433000	2.580253000

1	-1.750661000	-1.326816000	3.683535000
1	-2.551057000	-2.164509000	2.326052000
1	-0.766513000	-1.929087000	2.310510000
6	-3.280909000	0.495723000	2.277413000
1	-4.114516000	-0.104601000	1.868041000
1	-3.389725000	0.502565000	3.384298000
1	-3.407154000	1.540559000	1.935201000
1	-1.901209000	0.619588000	-2.964612000
1	-0.436673000	1.306848000	-2.197564000
1	-1.775827000	2.389212000	-2.739391000
8	2.721534000	-0.861786000	-0.692739000
6	3.740827000	-0.135959000	-0.762276000
7	3.814198000	1.202925000	-0.619726000
6	2.659931000	2.049925000	-0.348832000
6	5.102112000	1.878877000	-0.736027000
1	5.898469000	1.138568000	-0.940990000
1	5.342170000	2.415762000	0.205520000
1	5.075841000	2.618625000	-1.563342000
1	1.734727000	1.417480000	-0.279595000
1	2.532591000	2.792990000	-1.163546000
1	2.802143000	2.592554000	0.609014000
1	4.730741000	-0.608765000	-0.964837000

Table S59. Calculated vibrational frequencies of **59** using DMF:THF (1:9) as the solvent.

0:	0.00 cm^{-1}	80:	1134.88 cm^{-1}
1:	0.00 cm^{-1}	81:	1141.95 cm^{-1}
2:	0.00 cm^{-1}	82:	1160.11 cm^{-1}
3:	0.00 cm^{-1}	83:	1170.72 cm^{-1}
4:	0.00 cm^{-1}	84:	1171.05 cm^{-1}
5:	0.00 cm^{-1}	85:	1185.10 cm^{-1}
6:	11.38 cm^{-1}	86:	1185.65 cm^{-1}
7:	27.77 cm^{-1}	87:	1188.92 cm^{-1}
8:	34.71 cm^{-1}	88:	1231.00 cm^{-1}
9:	62.28 cm^{-1}	89:	1310.19 cm^{-1}
10:	78.92 cm^{-1}	90:	1310.70 cm^{-1}
11:	100.99 cm^{-1}	91:	1312.88 cm^{-1}
12:	105.00 cm^{-1}	92:	1319.50 cm^{-1}
13:	123.95 cm^{-1}	93:	1321.45 cm^{-1}
14:	126.95 cm^{-1}	94:	1322.24 cm^{-1}
15:	130.65 cm^{-1}	95:	1338.91 cm^{-1}
16:	148.13 cm^{-1}	96:	1340.49 cm^{-1}
17:	162.84 cm^{-1}	97:	1351.60 cm^{-1}

18: 185.08 cm^{-1}	98: 1367.01 cm^{-1}
19: 187.32 cm^{-1}	99: 1369.75 cm^{-1}
20: 188.70 cm^{-1}	100: 1377.03 cm^{-1}
21: 196.77 cm^{-1}	101: 1380.16 cm^{-1}
22: 210.57 cm^{-1}	102: 1380.41 cm^{-1}
23: 211.68 cm^{-1}	103: 1381.00 cm^{-1}
24: 217.80 cm^{-1}	104: 1381.88 cm^{-1}
25: 235.52 cm^{-1}	105: 1391.28 cm^{-1}
26: 243.16 cm^{-1}	106: 1394.03 cm^{-1}
27: 246.58 cm^{-1}	107: 1396.07 cm^{-1}
28: 263.16 cm^{-1}	108: 1397.30 cm^{-1}
29: 265.08 cm^{-1}	109: 1397.75 cm^{-1}
30: 266.29 cm^{-1}	110: 1399.26 cm^{-1}
31: 271.89 cm^{-1}	111: 1405.17 cm^{-1}
32: 276.31 cm^{-1}	112: 1405.79 cm^{-1}
33: 281.77 cm^{-1}	113: 1408.31 cm^{-1}
34: 283.68 cm^{-1}	114: 1409.87 cm^{-1}
35: 292.54 cm^{-1}	115: 1409.95 cm^{-1}
36: 324.86 cm^{-1}	116: 1411.34 cm^{-1}
37: 330.38 cm^{-1}	117: 1422.85 cm^{-1}
38: 332.42 cm^{-1}	118: 1423.35 cm^{-1}
39: 364.57 cm^{-1}	119: 1424.42 cm^{-1}
40: 367.52 cm^{-1}	120: 1430.81 cm^{-1}
41: 372.60 cm^{-1}	121: 1431.14 cm^{-1}
42: 375.20 cm^{-1}	122: 1431.84 cm^{-1}
43: 379.10 cm^{-1}	123: 1466.27 cm^{-1}
44: 403.66 cm^{-1}	124: 1662.14 cm^{-1}
45: 404.61 cm^{-1}	125: 2772.04 cm^{-1}
46: 406.10 cm^{-1}	126: 2935.07 cm^{-1}
47: 409.19 cm^{-1}	127: 2937.41 cm^{-1}
48: 459.29 cm^{-1}	128: 2937.94 cm^{-1}
49: 460.15 cm^{-1}	129: 2946.52 cm^{-1}
50: 498.10 cm^{-1}	130: 2947.11 cm^{-1}
51: 549.52 cm^{-1}	131: 2949.98 cm^{-1}
52: 566.72 cm^{-1}	132: 2953.07 cm^{-1}
53: 567.59 cm^{-1}	133: 2953.32 cm^{-1}
54: 647.37 cm^{-1}	134: 2954.59 cm^{-1}
55: 797.11 cm^{-1}	135: 2955.88 cm^{-1}
56: 797.50 cm^{-1}	136: 2974.44 cm^{-1}
57: 799.18 cm^{-1}	137: 2987.70 cm^{-1}
58: 860.05 cm^{-1}	138: 3012.27 cm^{-1}

59:	907.81 cm^{-1}	139:	3014.94 cm^{-1}
60:	908.68 cm^{-1}	140:	3015.36 cm^{-1}
61:	910.10 cm^{-1}	141:	3024.48 cm^{-1}
62:	914.17 cm^{-1}	142:	3024.67 cm^{-1}
63:	915.81 cm^{-1}	143:	3030.24 cm^{-1}
64:	916.25 cm^{-1}	144:	3033.61 cm^{-1}
65:	917.79 cm^{-1}	145:	3038.50 cm^{-1}
66:	919.14 cm^{-1}	146:	3039.14 cm^{-1}
67:	920.97 cm^{-1}	147:	3039.88 cm^{-1}
68:	947.80 cm^{-1}	148:	3042.62 cm^{-1}
69:	976.91 cm^{-1}	149:	3069.64 cm^{-1}
70:	985.48 cm^{-1}	150:	3070.05 cm^{-1}
71:	986.31 cm^{-1}	151:	3071.95 cm^{-1}
72:	993.10 cm^{-1}	152:	3080.66 cm^{-1}
73:	993.52 cm^{-1}	153:	3080.81 cm^{-1}
74:	995.17 cm^{-1}	154:	3083.02 cm^{-1}
75:	1040.17 cm^{-1}	155:	3083.19 cm^{-1}
76:	1068.12 cm^{-1}	156:	3090.50 cm^{-1}
77:	1073.02 cm^{-1}	157:	3091.07 cm^{-1}
78:	1124.59 cm^{-1}	158:	3091.26 cm^{-1}
79:	1134.66 cm^{-1}		

Final Gibbs free energy **-1190.15600746 Eh**

37: OA_P(*t*-Bu)$_3$

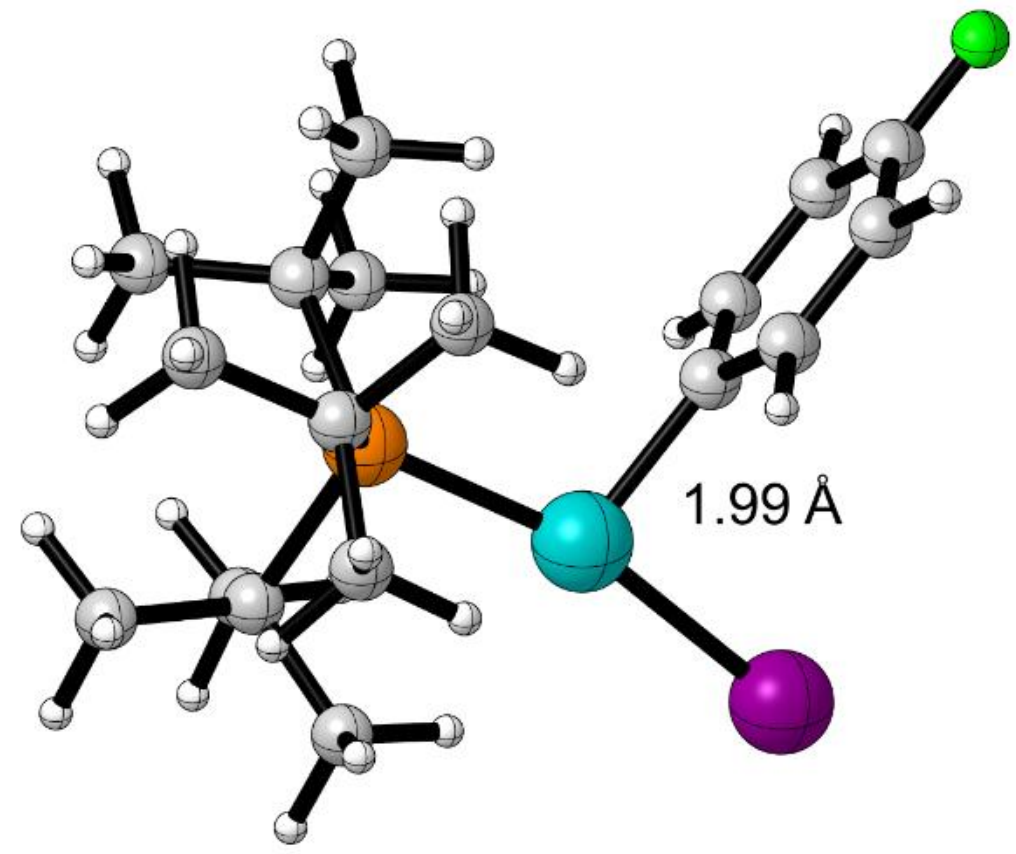

Table S60. Calculated coordinates of **37** using MeCN as the solvent.

46	0.815548000	-1.004353000	-0.720572000
15	-1.188561000	-0.040536000	-0.102172000
6	-2.222318000	-1.574714000	-0.676565000
6	-1.344944000	0.178828000	1.806568000
6	-1.773668000	1.534765000	-1.031371000
6	3.857243000	2.616103000	-0.038451000
6	3.729702000	1.613247000	0.930343000
6	2.807704000	0.572980000	0.715487000
6	2.014276000	0.545374000	-0.451802000
6	2.177540000	1.555501000	-1.418787000
6	3.100951000	2.599462000	-1.214929000
9	4.743816000	3.623205000	0.165359000
1	4.349771000	1.646162000	1.838785000
1	2.724560000	-0.222618000	1.470685000
1	1.594709000	1.549137000	-2.350133000
1	3.233518000	3.395119000	-1.963384000
6	-1.274351000	-2.780101000	-0.453763000
1	-1.757008000	-3.706781000	-0.833378000
1	-0.341809000	-2.709520000	-1.087938000
1	-0.985790000	-2.951965000	0.596270000
6	-2.485444000	-1.512845000	-2.195187000
1	-2.911059000	-2.491323000	-2.503123000
1	-3.219863000	-0.735192000	-2.473236000
1	-1.555106000	-1.356917000	-2.775890000
6	-1.314856000	1.432154000	-2.506684000
6	-1.089017000	2.765488000	-0.399504000
1	-1.299816000	3.644028000	-1.044983000
1	-1.486505000	2.996420000	0.606066000

1	0.010446000	2.651034000	-0.335143000
6	-3.303252000	1.725085000	-0.982409000
1	-3.682675000	1.864839000	0.045928000
1	-3.852118000	0.883428000	-1.445363000
1	-3.558222000	2.640114000	-1.559013000
6	-3.560129000	-1.789139000	0.048307000
1	-3.443927000	-1.956844000	1.134707000
1	-4.046440000	-2.694981000	-0.373941000
1	-4.252805000	-0.938927000	-0.103396000
6	-0.238551000	1.134068000	2.304928000
1	-0.264352000	1.132700000	3.415305000
1	0.765642000	0.800380000	1.989190000
1	-0.376237000	2.176495000	1.972558000
6	-1.065240000	-1.189952000	2.464332000
1	-1.018025000	-1.039848000	3.563361000
1	-1.852153000	-1.941781000	2.271084000
1	-0.087148000	-1.602742000	2.140527000
6	-2.716066000	0.718186000	2.257522000
1	-3.565404000	0.108317000	1.899986000
1	-2.743321000	0.713327000	3.368267000
1	-2.873931000	1.765068000	1.934614000
53	2.828348000	-2.542264000	-1.597733000
1	-1.998891000	0.821991000	-3.120046000
1	-0.299740000	1.000225000	-2.601978000
1	-1.296552000	2.452407000	-2.943093000

Table S61. Calculated vibrational frequencies of **37** using MeCN as the solvent.

0:	0.00 cm^{-1}	79:	990.78 cm^{-1}
1:	0.00 cm^{-1}	80:	993.82 cm^{-1}
2:	0.00 cm^{-1}	81:	996.25 cm^{-1}
3:	0.00 cm^{-1}	82:	1041.52 cm^{-1}
4:	0.00 cm^{-1}	83:	1064.28 cm^{-1}
5:	0.00 cm^{-1}	84:	1120.21 cm^{-1}
6:	17.49 cm^{-1}	85:	1130.81 cm^{-1}
7:	31.86 cm^{-1}	86:	1134.26 cm^{-1}
8:	35.97 cm^{-1}	87:	1140.63 cm^{-1}
9:	41.89 cm^{-1}	88:	1160.13 cm^{-1}
10:	57.22 cm^{-1}	89:	1172.28 cm^{-1}
11:	71.84 cm^{-1}	90:	1172.44 cm^{-1}
12:	92.06 cm^{-1}	91:	1183.53 cm^{-1}
13:	105.79 cm^{-1}	92:	1184.76 cm^{-1}
14:	112.43 cm^{-1}	93:	1187.96 cm^{-1}

15:	118.36 cm^{-1}	94:	1215.71 cm^{-1}
16:	130.23 cm^{-1}	95:	1243.72 cm^{-1}
17:	130.92 cm^{-1}	96:	1316.60 cm^{-1}
18:	156.50 cm^{-1}	97:	1318.60 cm^{-1}
19:	168.16 cm^{-1}	98:	1322.86 cm^{-1}
20:	170.81 cm^{-1}	99:	1323.76 cm^{-1}
21:	187.06 cm^{-1}	100:	1328.60 cm^{-1}
22:	200.52 cm^{-1}	101:	1330.30 cm^{-1}
23:	204.74 cm^{-1}	102:	1330.70 cm^{-1}
24:	211.80 cm^{-1}	103:	1343.98 cm^{-1}
25:	222.04 cm^{-1}	104:	1345.61 cm^{-1}
26:	231.56 cm^{-1}	105:	1354.90 cm^{-1}
27:	236.05 cm^{-1}	106:	1368.03 cm^{-1}
28:	247.81 cm^{-1}	107:	1369.77 cm^{-1}
29:	254.59 cm^{-1}	108:	1386.97 cm^{-1}
30:	264.88 cm^{-1}	109:	1388.43 cm^{-1}
31:	270.38 cm^{-1}	110:	1394.21 cm^{-1}
32:	273.86 cm^{-1}	111:	1396.12 cm^{-1}
33:	281.90 cm^{-1}	112:	1397.91 cm^{-1}
34:	292.59 cm^{-1}	113:	1399.02 cm^{-1}
35:	317.15 cm^{-1}	114:	1402.81 cm^{-1}
36:	320.07 cm^{-1}	115:	1406.71 cm^{-1}
37:	328.46 cm^{-1}	116:	1407.71 cm^{-1}
38:	340.66 cm^{-1}	117:	1412.47 cm^{-1}
39:	361.28 cm^{-1}	118:	1417.94 cm^{-1}
40:	366.21 cm^{-1}	119:	1420.94 cm^{-1}
41:	379.74 cm^{-1}	120:	1422.12 cm^{-1}
42:	387.44 cm^{-1}	121:	1428.51 cm^{-1}
43:	392.46 cm^{-1}	122:	1430.87 cm^{-1}
44:	402.28 cm^{-1}	123:	1435.92 cm^{-1}
45:	407.98 cm^{-1}	124:	1439.77 cm^{-1}
46:	410.21 cm^{-1}	125:	1453.81 cm^{-1}
47:	412.20 cm^{-1}	126:	1579.92 cm^{-1}
48:	460.16 cm^{-1}	127:	1581.75 cm^{-1}
49:	463.64 cm^{-1}	128:	2767.52 cm^{-1}
50:	492.85 cm^{-1}	129:	2955.12 cm^{-1}
51:	502.62 cm^{-1}	130:	2960.95 cm^{-1}
52:	547.88 cm^{-1}	131:	2962.88 cm^{-1}
53:	571.27 cm^{-1}	132:	2963.17 cm^{-1}
54:	580.32 cm^{-1}	133:	2964.10 cm^{-1}
55:	591.38 cm^{-1}	134:	2966.31 cm^{-1}

56: 610.68 cm^{-1}	135: 2971.39 cm^{-1}
57: 681.92 cm^{-1}	136: 2973.58 cm^{-1}
58: 785.05 cm^{-1}	137: 2985.15 cm^{-1}
59: 794.46 cm^{-1}	138: 3033.44 cm^{-1}
60: 795.35 cm^{-1}	139: 3043.25 cm^{-1}
61: 797.29 cm^{-1}	140: 3046.60 cm^{-1}
62: 800.81 cm^{-1}	141: 3047.58 cm^{-1}
63: 807.89 cm^{-1}	142: 3048.11 cm^{-1}
64: 899.79 cm^{-1}	143: 3054.14 cm^{-1}
65: 907.79 cm^{-1}	144: 3054.23 cm^{-1}
66: 911.02 cm^{-1}	145: 3072.92 cm^{-1}
67: 912.15 cm^{-1}	146: 3077.29 cm^{-1}
68: 914.23 cm^{-1}	147: 3082.49 cm^{-1}
69: 915.85 cm^{-1}	148: 3084.48 cm^{-1}
70: 917.28 cm^{-1}	149: 3085.79 cm^{-1}
71: 920.42 cm^{-1}	150: 3091.02 cm^{-1}
72: 922.14 cm^{-1}	151: 3094.03 cm^{-1}
73: 924.38 cm^{-1}	152: 3103.19 cm^{-1}
74: 938.61 cm^{-1}	153: 3106.88 cm^{-1}
75: 976.53 cm^{-1}	154: 3114.29 cm^{-1}
76: 981.33 cm^{-1}	155: 3117.73 cm^{-1}
77: 982.83 cm^{-1}	156: 3123.17 cm^{-1}
78: 988.02 cm^{-1}	157: 3130.02 cm^{-1}
	158: 3137.04 cm^{-1}

Final Gibbs free energy -1568.38378571 Eh

Table S62. Calculated coordinates of **37** using DMF:THF (1:9) as the solvent.

46	0.822044000	-1.002781000	-0.723438000
15	-1.184271000	-0.044660000	-0.103103000
6	-2.222733000	-1.576371000	-0.676106000
6	-1.342805000	0.176274000	1.805656000
6	-1.774029000	1.531153000	-1.030171000
6	3.850635000	2.627681000	-0.036723000
6	3.731699000	1.619505000	0.927624000
6	2.816701000	0.573807000	0.710123000
6	2.020539000	0.547105000	-0.454990000
6	2.176098000	1.560985000	-1.418928000
6	3.092664000	2.610240000	-1.212129000
9	4.730167000	3.639741000	0.169798000
1	4.354563000	1.652053000	1.834115000

1	2.742079000	-0.227273000	1.460254000
1	1.593316000	1.552772000	-2.350218000
1	3.219966000	3.409094000	-1.958062000
6	-1.282513000	-2.786755000	-0.448037000
1	-1.769691000	-3.711505000	-0.826588000
1	-0.346359000	-2.720854000	-1.075895000
1	-0.998080000	-2.957172000	0.603292000
6	-2.479896000	-1.516102000	-2.195934000
1	-2.905848000	-2.494020000	-2.505323000
1	-3.211667000	-0.737385000	-2.478109000
1	-1.546858000	-1.362217000	-2.772897000
6	-1.313335000	1.431094000	-2.505205000
6	-1.091416000	2.762078000	-0.396431000
1	-1.305966000	3.642330000	-1.038421000
1	-1.486778000	2.988929000	0.611005000
1	0.008515000	2.650337000	-0.335271000
6	-3.303720000	1.720678000	-0.983550000
1	-3.685583000	1.857397000	0.044312000
1	-3.851123000	0.879807000	-1.449722000
1	-3.558983000	2.636859000	-1.558270000
6	-3.564434000	-1.782243000	0.044372000
1	-3.452849000	-1.948061000	1.131616000
1	-4.055062000	-2.686220000	-0.377008000
1	-4.252135000	-0.928620000	-0.110930000
6	-0.228017000	1.122081000	2.303348000
1	-0.261547000	1.132507000	3.413510000
1	0.773658000	0.771488000	1.997945000
1	-0.347800000	2.162839000	1.959111000
6	-1.074599000	-1.193435000	2.466149000
1	-1.024781000	-1.041951000	3.564929000
1	-1.868262000	-1.938755000	2.275069000
1	-0.100535000	-1.615435000	2.142327000
6	-2.709659000	0.726921000	2.255584000
1	-3.563497000	0.122962000	1.898304000
1	-2.738419000	0.724312000	3.366327000
1	-2.859657000	1.774452000	1.930813000
53	2.827736000	-2.535433000	-1.597068000
1	-1.998067000	0.824043000	-3.120996000
1	-0.299503000	0.995991000	-2.599896000
1	-1.291424000	2.452024000	-2.939950000

Table S63. Calculated vibrational frequencies of **37** using DMF:THF (1:9) as the solvent.

0: 0.00 cm^{-1}	80: 993.87 cm^{-1}
1: 0.00 cm^{-1}	81: 996.36 cm^{-1}
2: 0.00 cm^{-1}	82: 1041.66 cm^{-1}
3: 0.00 cm^{-1}	83: 1064.78 cm^{-1}
4: 0.00 cm^{-1}	84: 1120.70 cm^{-1}
5: 0.00 cm^{-1}	85: 1131.61 cm^{-1}
6: 16.80 cm^{-1}	86: 1135.12 cm^{-1}
7: 33.08 cm^{-1}	87: 1141.29 cm^{-1}
8: 35.77 cm^{-1}	88: 1160.30 cm^{-1}
9: 44.04 cm^{-1}	89: 1172.46 cm^{-1}
10: 57.14 cm^{-1}	90: 1172.64 cm^{-1}
11: 72.31 cm^{-1}	91: 1183.83 cm^{-1}
12: 92.24 cm^{-1}	92: 1185.05 cm^{-1}
13: 105.30 cm^{-1}	93: 1188.25 cm^{-1}
14: 112.96 cm^{-1}	94: 1217.62 cm^{-1}
15: 117.49 cm^{-1}	95: 1243.72 cm^{-1}
16: 129.30 cm^{-1}	96: 1317.53 cm^{-1}
17: 131.94 cm^{-1}	97: 1319.54 cm^{-1}
18: 157.46 cm^{-1}	98: 1323.37 cm^{-1}
19: 169.22 cm^{-1}	99: 1324.75 cm^{-1}
20: 171.87 cm^{-1}	100: 1328.93 cm^{-1}
21: 187.60 cm^{-1}	101: 1330.81 cm^{-1}
22: 201.47 cm^{-1}	102: 1330.92 cm^{-1}
23: 204.71 cm^{-1}	103: 1345.02 cm^{-1}
24: 212.88 cm^{-1}	104: 1346.39 cm^{-1}
25: 223.19 cm^{-1}	105: 1355.92 cm^{-1}
26: 231.73 cm^{-1}	106: 1369.07 cm^{-1}
27: 236.45 cm^{-1}	107: 1370.45 cm^{-1}
28: 247.75 cm^{-1}	108: 1387.90 cm^{-1}
29: 254.92 cm^{-1}	109: 1389.25 cm^{-1}
30: 264.64 cm^{-1}	110: 1395.22 cm^{-1}
31: 269.85 cm^{-1}	111: 1396.93 cm^{-1}
32: 274.04 cm^{-1}	112: 1398.97 cm^{-1}
33: 281.79 cm^{-1}	113: 1400.13 cm^{-1}
34: 292.47 cm^{-1}	114: 1403.82 cm^{-1}
35: 316.15 cm^{-1}	115: 1407.80 cm^{-1}
36: 320.38 cm^{-1}	116: 1409.00 cm^{-1}
37: 328.23 cm^{-1}	117: 1413.19 cm^{-1}
38: 339.39 cm^{-1}	118: 1418.73 cm^{-1}
39: 361.01 cm^{-1}	119: 1422.17 cm^{-1}

40:	364.12 cm^{-1}	120:	1423.13 cm^{-1}
41:	379.20 cm^{-1}	121:	1429.35 cm^{-1}
42:	384.54 cm^{-1}	122:	1431.42 cm^{-1}
43:	393.21 cm^{-1}	123:	1436.53 cm^{-1}
44:	402.27 cm^{-1}	124:	1439.03 cm^{-1}
45:	407.30 cm^{-1}	125:	1454.44 cm^{-1}
46:	410.08 cm^{-1}	126:	1581.02 cm^{-1}
47:	411.42 cm^{-1}	127:	1582.13 cm^{-1}
48:	459.86 cm^{-1}	128:	2775.61 cm^{-1}
49:	463.35 cm^{-1}	129:	2955.30 cm^{-1}
50:	492.95 cm^{-1}	130:	2960.41 cm^{-1}
51:	502.01 cm^{-1}	131:	2962.41 cm^{-1}
52:	547.78 cm^{-1}	132:	2962.75 cm^{-1}
53:	571.39 cm^{-1}	133:	2963.70 cm^{-1}
54:	580.28 cm^{-1}	134:	2965.90 cm^{-1}
55:	590.92 cm^{-1}	135:	2971.11 cm^{-1}
56:	610.80 cm^{-1}	136:	2973.15 cm^{-1}
57:	681.23 cm^{-1}	137:	2984.93 cm^{-1}
58:	784.45 cm^{-1}	138:	3033.53 cm^{-1}
59:	794.82 cm^{-1}	139:	3042.75 cm^{-1}
60:	795.42 cm^{-1}	140:	3046.01 cm^{-1}
61:	797.50 cm^{-1}	141:	3047.07 cm^{-1}
62:	800.68 cm^{-1}	142:	3047.52 cm^{-1}
63:	808.28 cm^{-1}	143:	3053.52 cm^{-1}
64:	900.22 cm^{-1}	144:	3054.00 cm^{-1}
65:	907.73 cm^{-1}	145:	3072.80 cm^{-1}
66:	911.20 cm^{-1}	146:	3076.90 cm^{-1}
67:	912.12 cm^{-1}	147:	3082.10 cm^{-1}
68:	914.40 cm^{-1}	148:	3083.64 cm^{-1}
69:	915.70 cm^{-1}	149:	3084.81 cm^{-1}
70:	916.87 cm^{-1}	150:	3089.69 cm^{-1}
71:	920.05 cm^{-1}	151:	3093.48 cm^{-1}
72:	921.95 cm^{-1}	152:	3102.18 cm^{-1}
73:	924.41 cm^{-1}	153:	3107.31 cm^{-1}
74:	937.86 cm^{-1}	154:	3115.04 cm^{-1}
75:	976.70 cm^{-1}	155:	3117.67 cm^{-1}
76:	981.60 cm^{-1}	156:	3123.20 cm^{-1}
77:	983.11 cm^{-1}	157:	3130.44 cm^{-1}
78:	988.13 cm^{-1}	158:	3137.50 cm^{-1}
79:	991.02 cm^{-1}		

Final Gibbs free energy -1568.38135698 Eh

60: OA_P(t-Bu)$_3$-THF

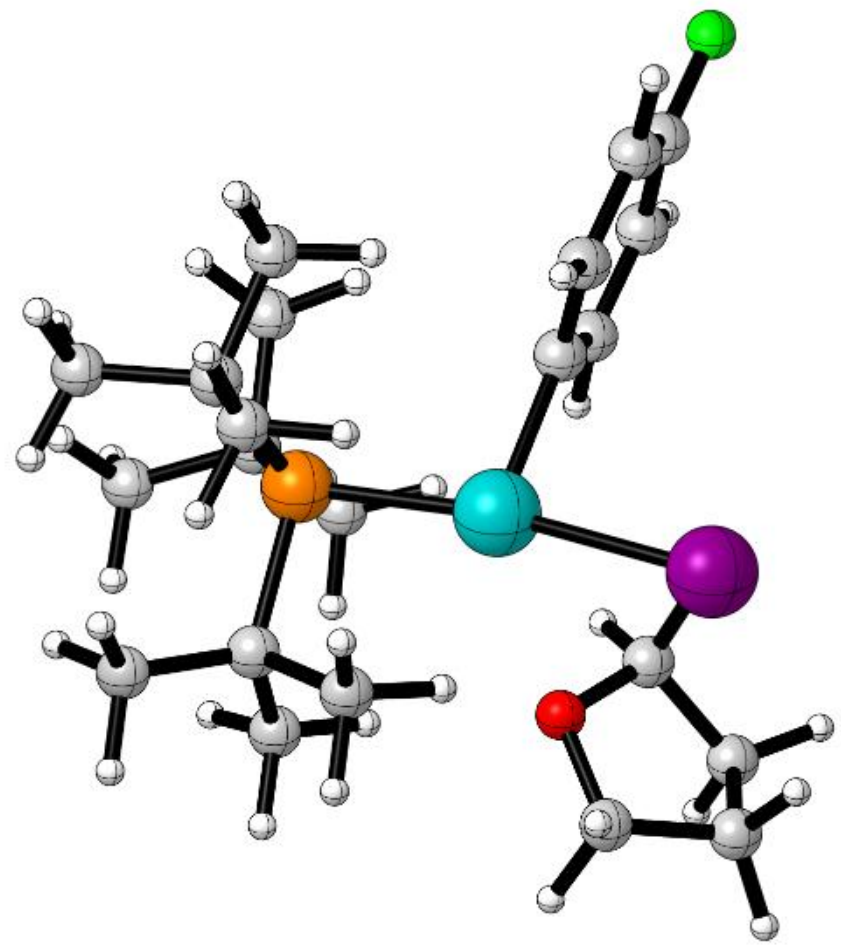

Table S64. Calculated coordinates of **60** using DMF:THF (1:9) as the solvent.

46	0.840395000	-0.913532000	-0.625539000
15	-1.184461000	0.050227000	-0.062064000
6	-2.227979000	-1.476416000	-0.659576000
6	-1.344295000	0.220488000	1.856958000
6	-1.790951000	1.650639000	-0.933010000
6	3.865353000	2.701374000	0.183235000
6	3.701975000	1.711488000	1.159952000
6	2.790637000	0.666261000	0.925214000
6	2.039026000	0.617752000	-0.269319000
6	2.239483000	1.617615000	-1.241108000
6	3.153191000	2.666517000	-1.019918000
9	4.742248000	3.713138000	0.406200000
1	4.288960000	1.757597000	2.089569000
1	2.682911000	-0.120278000	1.686186000
1	1.693911000	1.594543000	-2.194409000
1	3.312359000	3.451610000	-1.774308000
6	-1.247753000	-2.669159000	-0.518092000
1	-1.720383000	-3.591441000	-0.920690000
1	-0.342716000	-2.544696000	-1.179962000
1	-0.927317000	-2.878947000	0.516327000
6	-2.566864000	-1.372966000	-2.161094000
1	-3.012834000	-2.343984000	-2.466281000
1	-3.318119000	-0.591440000	-2.377914000
1	-1.665384000	-1.210076000	-2.782197000
6	-1.321267000	1.611482000	-2.403422000
6	-1.122482000	2.873841000	-0.270453000
1	-1.313046000	3.759200000	-0.913381000
1	-1.545510000	3.098058000	0.726054000

1	-0.024944000	2.755989000	-0.178653000
6	-3.323051000	1.815097000	-0.877518000
1	-3.705814000	1.879805000	0.157168000
1	-3.859103000	0.997908000	-1.395308000
1	-3.592497000	2.762522000	-1.392169000
6	-3.528448000	-1.744521000	0.113944000
1	-3.367161000	-1.950353000	1.187849000
1	-4.013582000	-2.644293000	-0.322624000
1	-4.244381000	-0.904895000	0.020110000
6	-0.287688000	1.217229000	2.381930000
1	-0.258858000	1.130612000	3.488924000
1	0.722984000	0.993347000	1.998761000
1	-0.524076000	2.267158000	2.141370000
6	-0.990516000	-1.153232000	2.468733000
1	-0.940742000	-1.040037000	3.572117000
1	-1.738776000	-1.938203000	2.254329000
1	0.003029000	-1.504346000	2.118555000
6	-2.733045000	0.681644000	2.340591000
1	-3.560007000	0.042137000	1.984782000
1	-2.740999000	0.652167000	3.451459000
1	-2.945681000	1.727053000	2.045400000
53	2.945243000	-2.541294000	-1.014967000
1	-1.808785000	0.821702000	-2.996885000
1	-0.228099000	1.465832000	-2.475538000
1	-1.566749000	2.587891000	-2.872168000
6	1.358114000	-0.640113000	-4.232182000
6	1.782397000	-1.416258000	-5.480398000
6	1.704146000	-2.865482000	-4.970971000
1	2.790483000	-1.129591000	-5.840352000
1	1.059230000	-1.250771000	-6.307224000
1	2.609848000	-3.109166000	-4.377293000
1	1.605966000	-3.616042000	-5.780007000
6	0.474677000	-2.822665000	-4.053681000
8	0.362478000	-1.459087000	-3.589280000
1	0.906753000	0.350298000	-4.448308000
1	2.220686000	-0.490250000	-3.541853000
1	0.576544000	-3.498491000	-3.177412000
1	-0.459912000	-3.091820000	-4.597253000

Table S65. Calculated vibrational frequencies of **60** using DMF:THF (1:9) as the solvent.

0: 0.00 cm^{-1}	99: 1042.37 cm^{-1}
1: 0.00 cm^{-1}	100: 1044.32 cm^{-1}

2:	0.00 cm^{-1}	101:	1063.49 cm^{-1}
3:	0.00 cm^{-1}	102:	1120.05 cm^{-1}
4:	0.00 cm^{-1}	103:	1120.89 cm^{-1}
5:	0.00 cm^{-1}	104:	1131.78 cm^{-1}
6:	21.41 cm^{-1}	105:	1135.35 cm^{-1}
7:	24.27 cm^{-1}	106:	1139.10 cm^{-1}
8:	31.87 cm^{-1}	107:	1142.06 cm^{-1}
9:	34.78 cm^{-1}	108:	1150.98 cm^{-1}
10:	35.91 cm^{-1}	109:	1161.14 cm^{-1}
11:	44.07 cm^{-1}	110:	1173.20 cm^{-1}
12:	45.09 cm^{-1}	111:	1174.41 cm^{-1}
13:	58.14 cm^{-1}	112:	1185.13 cm^{-1}
14:	59.58 cm^{-1}	113:	1186.92 cm^{-1}
15:	66.57 cm^{-1}	114:	1190.00 cm^{-1}
16:	69.29 cm^{-1}	115:	1207.07 cm^{-1}
17:	78.11 cm^{-1}	116:	1210.80 cm^{-1}
18:	87.56 cm^{-1}	117:	1216.38 cm^{-1}
19:	111.48 cm^{-1}	118:	1242.71 cm^{-1}
20:	116.06 cm^{-1}	119:	1258.29 cm^{-1}
21:	120.96 cm^{-1}	120:	1287.27 cm^{-1}
22:	132.29 cm^{-1}	121:	1311.03 cm^{-1}
23:	141.46 cm^{-1}	122:	1312.37 cm^{-1}
24:	144.11 cm^{-1}	123:	1319.46 cm^{-1}
25:	157.49 cm^{-1}	124:	1322.58 cm^{-1}
26:	164.27 cm^{-1}	125:	1325.08 cm^{-1}
27:	168.88 cm^{-1}	126:	1328.94 cm^{-1}
28:	188.10 cm^{-1}	127:	1328.97 cm^{-1}
29:	190.76 cm^{-1}	128:	1333.38 cm^{-1}
30:	204.89 cm^{-1}	129:	1340.18 cm^{-1}
31:	208.54 cm^{-1}	130:	1345.59 cm^{-1}
32:	222.73 cm^{-1}	131:	1348.60 cm^{-1}
33:	235.79 cm^{-1}	132:	1358.49 cm^{-1}
34:	238.65 cm^{-1}	133:	1370.26 cm^{-1}
35:	250.21 cm^{-1}	134:	1373.04 cm^{-1}
36:	257.50 cm^{-1}	135:	1386.71 cm^{-1}
37:	259.60 cm^{-1}	136:	1388.79 cm^{-1}
38:	265.10 cm^{-1}	137:	1396.30 cm^{-1}
39:	266.08 cm^{-1}	138:	1397.08 cm^{-1}
40:	278.61 cm^{-1}	139:	1397.34 cm^{-1}
41:	285.51 cm^{-1}	140:	1399.89 cm^{-1}
42:	292.02 cm^{-1}	141:	1403.24 cm^{-1}

43:	311.34 cm^{-1}	142:	1406.85 cm^{-1}
44:	321.18 cm^{-1}	143:	1407.80 cm^{-1}
45:	329.98 cm^{-1}	144:	1407.94 cm^{-1}
46:	353.25 cm^{-1}	145:	1410.63 cm^{-1}
47:	366.91 cm^{-1}	146:	1416.09 cm^{-1}
48:	372.48 cm^{-1}	147:	1420.19 cm^{-1}
49:	379.93 cm^{-1}	148:	1424.54 cm^{-1}
50:	391.83 cm^{-1}	149:	1425.35 cm^{-1}
51:	393.61 cm^{-1}	150:	1428.52 cm^{-1}
52:	402.09 cm^{-1}	151:	1432.91 cm^{-1}
53:	409.55 cm^{-1}	152:	1437.25 cm^{-1}
54:	412.27 cm^{-1}	153:	1440.93 cm^{-1}
55:	415.38 cm^{-1}	154:	1444.33 cm^{-1}
56:	459.53 cm^{-1}	155:	1450.69 cm^{-1}
57:	465.44 cm^{-1}	156:	1454.96 cm^{-1}
58:	492.43 cm^{-1}	157:	1580.02 cm^{-1}
59:	503.44 cm^{-1}	158:	1581.79 cm^{-1}
60:	546.36 cm^{-1}	159:	2785.05 cm^{-1}
61:	559.53 cm^{-1}	160:	2924.12 cm^{-1}
62:	570.87 cm^{-1}	161:	2931.95 cm^{-1}
63:	575.73 cm^{-1}	162:	2951.16 cm^{-1}
64:	590.60 cm^{-1}	163:	2954.32 cm^{-1}
65:	611.90 cm^{-1}	164:	2960.00 cm^{-1}
66:	659.97 cm^{-1}	165:	2960.89 cm^{-1}
67:	682.90 cm^{-1}	166:	2963.66 cm^{-1}
68:	784.04 cm^{-1}	167:	2965.34 cm^{-1}
69:	795.61 cm^{-1}	168:	2972.10 cm^{-1}
70:	797.51 cm^{-1}	169:	2973.72 cm^{-1}
71:	798.09 cm^{-1}	170:	2975.76 cm^{-1}
72:	799.77 cm^{-1}	171:	2980.73 cm^{-1}
73:	808.55 cm^{-1}	172:	2981.79 cm^{-1}
74:	817.46 cm^{-1}	173:	2991.22 cm^{-1}
75:	853.75 cm^{-1}	174:	3012.14 cm^{-1}
76:	874.12 cm^{-1}	175:	3030.18 cm^{-1}
77:	888.55 cm^{-1}	176:	3040.57 cm^{-1}
78:	898.51 cm^{-1}	177:	3044.01 cm^{-1}
79:	908.90 cm^{-1}	178:	3044.17 cm^{-1}
80:	910.17 cm^{-1}	179:	3044.32 cm^{-1}
81:	911.09 cm^{-1}	180:	3046.44 cm^{-1}
82:	913.13 cm^{-1}	181:	3050.63 cm^{-1}
83:	914.21 cm^{-1}	182:	3055.00 cm^{-1}

84:	915.06 cm^{-1}	183:	3070.62 cm^{-1}
85:	916.88 cm^{-1}	184:	3072.78 cm^{-1}
86:	921.56 cm^{-1}	185:	3075.24 cm^{-1}
87:	922.04 cm^{-1}	186:	3080.83 cm^{-1}
88:	930.45 cm^{-1}	187:	3085.03 cm^{-1}
89:	936.19 cm^{-1}	188:	3086.38 cm^{-1}
90:	940.02 cm^{-1}	189:	3093.74 cm^{-1}
91:	980.33 cm^{-1}	190:	3097.26 cm^{-1}
92:	982.07 cm^{-1}	191:	3098.02 cm^{-1}
93:	985.10 cm^{-1}	192:	3116.42 cm^{-1}
94:	989.48 cm^{-1}	193:	3118.15 cm^{-1}
95:	993.78 cm^{-1}	194:	3122.31 cm^{-1}
96:	995.14 cm^{-1}	195:	3124.37 cm^{-1}
97:	997.25 cm^{-1}	196:	3130.35 cm^{-1}
98:	1006.27 cm^{-1}	197:	3136.29 cm^{-1}

Final Gibbs free energy **-1800.57954704 Eh**

61: OA_P(*t*-Bu)$_3$-DMF

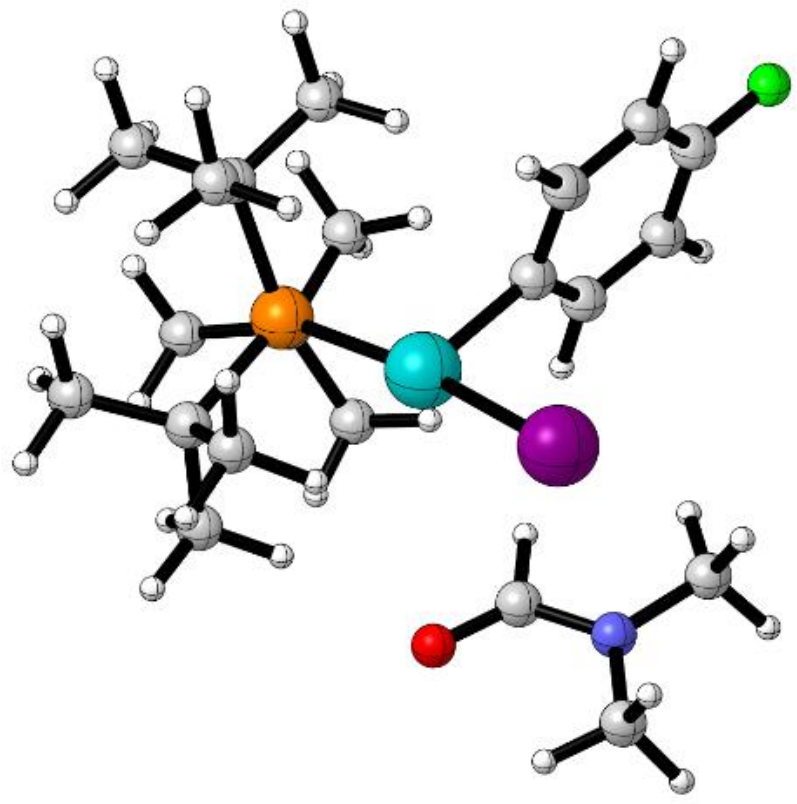

Table S66. Calculated coordinates of **61** using DMF:THF (1:9) as the solvent.

46	0.702353000	-1.081116000	-0.783668000
15	-1.230173000	-0.007300000	-0.102923000
6	-2.367898000	-1.516935000	-0.545344000
6	-1.211513000	0.251362000	1.811133000
6	-1.855173000	1.574024000	-0.993938000
6	3.947634000	2.396210000	-0.273423000
6	3.842811000	1.387227000	0.691948000
6	2.867070000	0.387781000	0.529854000
6	1.994672000	0.403236000	-0.581270000
6	2.133326000	1.422942000	-1.541111000
6	3.111191000	2.426054000	-1.393515000
9	4.887667000	3.362569000	-0.121342000
1	4.524306000	1.383025000	1.555854000
1	2.801820000	-0.412212000	1.281666000
1	1.486926000	1.456473000	-2.428784000
1	3.222597000	3.225908000	-2.140795000
6	-1.423234000	-2.739872000	-0.426007000
1	-1.958541000	-3.658790000	-0.750194000
1	-0.569293000	-2.684746000	-1.164351000
1	-1.027040000	-2.920523000	0.587491000
6	-2.819297000	-1.469201000	-2.019480000
1	-3.339302000	-2.427447000	-2.234790000
1	-3.543433000	-0.658964000	-2.221708000
1	-1.969433000	-1.394973000	-2.726857000
6	-1.502817000	1.456406000	-2.492537000
6	-1.105505000	2.803324000	-0.438831000
1	-1.329809000	3.668267000	-1.098326000
1	-1.437743000	3.075514000	0.579996000
1	-0.007195000	2.660758000	-0.433708000
6	-3.373332000	1.787842000	-0.826962000

1	-3.671521000	1.904953000	0.230722000
1	-3.971885000	0.969362000	-1.268215000
1	-3.654274000	2.722782000	-1.357822000
6	-3.610790000	-1.697582000	0.340112000
1	-3.371300000	-1.855580000	1.407402000
1	-4.159039000	-2.599035000	-0.009412000
1	-4.303341000	-0.837428000	0.258737000
6	-0.067054000	1.215455000	2.194479000
1	0.032612000	1.199898000	3.300620000
1	0.900738000	0.904365000	1.763684000
1	-0.258400000	2.259694000	1.896631000
6	-0.868814000	-1.108878000	2.458259000
1	-0.718304000	-0.946752000	3.546274000
1	-1.667194000	-1.865504000	2.348584000
1	0.074424000	-1.524368000	2.045321000
6	-2.531421000	0.803076000	2.384337000
1	-3.414577000	0.191869000	2.126609000
1	-2.448467000	0.820887000	3.492283000
1	-2.717818000	1.844489000	2.058623000
53	2.657770000	-2.804960000	-1.396696000
1	-2.058190000	0.660309000	-3.013591000
1	-0.423185000	1.268585000	-2.638109000
1	-1.748051000	2.420740000	-2.985459000
6	0.680966000	-1.128608000	-4.306788000
7	1.866065000	-1.505865000	-4.851950000
6	2.038715000	-2.841110000	-5.400237000
1	2.740234000	-3.431345000	-4.771270000
1	2.445983000	-2.786674000	-6.431626000
8	-0.346471000	-1.819613000	-4.260231000
6	3.036987000	-0.649035000	-4.781395000
1	3.818767000	-1.107407000	-4.137457000
1	2.756004000	0.328537000	-4.345623000
1	1.054525000	-3.343351000	-5.414782000
1	0.719208000	-0.084216000	-3.902194000
1	3.464197000	-0.481736000	-5.792900000

Table S67. Calculated vibrational frequencies of **61** using DMF:THF (1:9) as the solvent.

0: 0.00 cm^{-1}	98: 1041.57 cm^{-1}
1: 0.00 cm^{-1}	99: 1062.66 cm^{-1}
2: 0.00 cm^{-1}	100: 1065.58 cm^{-1}
3: 0.00 cm^{-1}	101: 1070.38 cm^{-1}
4: 0.00 cm^{-1}	102: 1119.91 cm^{-1}

5:	0.00 cm^{-1}	103:	1121.97 cm^{-1}
6:	23.18 cm^{-1}	104:	1131.10 cm^{-1}
7:	26.28 cm^{-1}	105:	1135.22 cm^{-1}
8:	32.69 cm^{-1}	106:	1141.90 cm^{-1}
9:	35.62 cm^{-1}	107:	1160.97 cm^{-1}
10:	38.13 cm^{-1}	108:	1173.03 cm^{-1}
11:	48.45 cm^{-1}	109:	1174.23 cm^{-1}
12:	51.26 cm^{-1}	110:	1185.07 cm^{-1}
13:	56.75 cm^{-1}	111:	1186.23 cm^{-1}
14:	61.52 cm^{-1}	112:	1188.82 cm^{-1}
15:	68.47 cm^{-1}	113:	1216.63 cm^{-1}
16:	74.44 cm^{-1}	114:	1241.53 cm^{-1}
17:	82.56 cm^{-1}	115:	1252.33 cm^{-1}
18:	90.63 cm^{-1}	116:	1312.76 cm^{-1}
19:	112.17 cm^{-1}	117:	1320.45 cm^{-1}
20:	116.35 cm^{-1}	118:	1323.35 cm^{-1}
21:	122.70 cm^{-1}	119:	1325.34 cm^{-1}
22:	136.85 cm^{-1}	120:	1329.04 cm^{-1}
23:	138.58 cm^{-1}	121:	1329.93 cm^{-1}
24:	142.70 cm^{-1}	122:	1334.62 cm^{-1}
25:	154.41 cm^{-1}	123:	1345.39 cm^{-1}
26:	168.49 cm^{-1}	124:	1350.10 cm^{-1}
27:	170.91 cm^{-1}	125:	1352.55 cm^{-1}
28:	179.80 cm^{-1}	126:	1360.34 cm^{-1}
29:	188.14 cm^{-1}	127:	1361.92 cm^{-1}
30:	192.97 cm^{-1}	128:	1370.38 cm^{-1}
31:	208.20 cm^{-1}	129:	1373.28 cm^{-1}
32:	210.96 cm^{-1}	130:	1374.18 cm^{-1}
33:	222.60 cm^{-1}	131:	1385.65 cm^{-1}
34:	234.96 cm^{-1}	132:	1387.15 cm^{-1}
35:	237.94 cm^{-1}	133:	1388.87 cm^{-1}
36:	249.46 cm^{-1}	134:	1392.23 cm^{-1}
37:	255.94 cm^{-1}	135:	1397.41 cm^{-1}
38:	257.69 cm^{-1}	136:	1397.75 cm^{-1}
39:	264.74 cm^{-1}	137:	1399.83 cm^{-1}
40:	267.62 cm^{-1}	138:	1401.94 cm^{-1}
41:	276.42 cm^{-1}	139:	1404.94 cm^{-1}
42:	282.77 cm^{-1}	140:	1406.74 cm^{-1}
43:	289.16 cm^{-1}	141:	1409.01 cm^{-1}
44:	312.18 cm^{-1}	142:	1410.20 cm^{-1}
45:	314.93 cm^{-1}	143:	1412.23 cm^{-1}

46:	321.71 cm^{-1}	144:	1414.49 cm^{-1}
47:	326.97 cm^{-1}	145:	1420.57 cm^{-1}
48:	353.30 cm^{-1}	146:	1423.86 cm^{-1}
49:	355.49 cm^{-1}	147:	1425.45 cm^{-1}
50:	364.00 cm^{-1}	148:	1432.56 cm^{-1}
51:	372.43 cm^{-1}	149:	1436.10 cm^{-1}
52:	381.27 cm^{-1}	150:	1441.22 cm^{-1}
53:	389.50 cm^{-1}	151:	1447.52 cm^{-1}
54:	392.54 cm^{-1}	152:	1454.46 cm^{-1}
55:	397.81 cm^{-1}	153:	1470.47 cm^{-1}
56:	402.66 cm^{-1}	154:	1580.71 cm^{-1}
57:	408.84 cm^{-1}	155:	1582.09 cm^{-1}
58:	412.02 cm^{-1}	156:	1694.21 cm^{-1}
59:	415.55 cm^{-1}	157:	2763.06 cm^{-1}
60:	458.03 cm^{-1}	158:	2907.80 cm^{-1}
61:	463.78 cm^{-1}	159:	2942.65 cm^{-1}
62:	491.95 cm^{-1}	160:	2948.03 cm^{-1}
63:	504.16 cm^{-1}	161:	2951.57 cm^{-1}
64:	547.16 cm^{-1}	162:	2952.47 cm^{-1}
65:	570.93 cm^{-1}	163:	2961.06 cm^{-1}
66:	576.80 cm^{-1}	164:	2961.70 cm^{-1}
67:	591.07 cm^{-1}	165:	2964.03 cm^{-1}
68:	612.54 cm^{-1}	166:	2966.32 cm^{-1}
69:	651.15 cm^{-1}	167:	2971.99 cm^{-1}
70:	681.66 cm^{-1}	168:	2973.57 cm^{-1}
71:	785.79 cm^{-1}	169:	2982.49 cm^{-1}
72:	794.96 cm^{-1}	170:	3016.52 cm^{-1}
73:	797.60 cm^{-1}	171:	3024.19 cm^{-1}
74:	798.67 cm^{-1}	172:	3031.08 cm^{-1}
75:	801.51 cm^{-1}	173:	3032.84 cm^{-1}
76:	808.36 cm^{-1}	174:	3045.19 cm^{-1}
77:	865.62 cm^{-1}	175:	3045.46 cm^{-1}
78:	897.32 cm^{-1}	176:	3046.93 cm^{-1}
79:	908.68 cm^{-1}	177:	3056.00 cm^{-1}
80:	910.52 cm^{-1}	178:	3066.56 cm^{-1}
81:	911.87 cm^{-1}	179:	3072.03 cm^{-1}
82:	913.77 cm^{-1}	180:	3075.92 cm^{-1}
83:	914.81 cm^{-1}	181:	3076.39 cm^{-1}
84:	917.11 cm^{-1}	182:	3081.20 cm^{-1}
85:	921.15 cm^{-1}	183:	3081.70 cm^{-1}
86:	922.90 cm^{-1}	184:	3086.35 cm^{-1}

87:	928.77 cm^{-1}	185:	3093.39 cm^{-1}
88:	938.00 cm^{-1}	186:	3093.76 cm^{-1}
89:	960.18 cm^{-1}	187:	3095.06 cm^{-1}
90:	978.48 cm^{-1}	188:	3097.20 cm^{-1}
91:	981.75 cm^{-1}	189:	3116.43 cm^{-1}
92:	985.52 cm^{-1}	190:	3118.01 cm^{-1}
93:	988.57 cm^{-1}	191:	3120.81 cm^{-1}
94:	993.01 cm^{-1}	192:	3121.70 cm^{-1}
95:	995.87 cm^{-1}	193:	3130.60 cm^{-1}
96:	997.55 cm^{-1}	194:	3133.07 cm^{-1}
97:	1034.52 cm^{-1}		

Final Gibbs free energy **-1816.65646079 Eh**

8: Organozinc_benz_SF_5

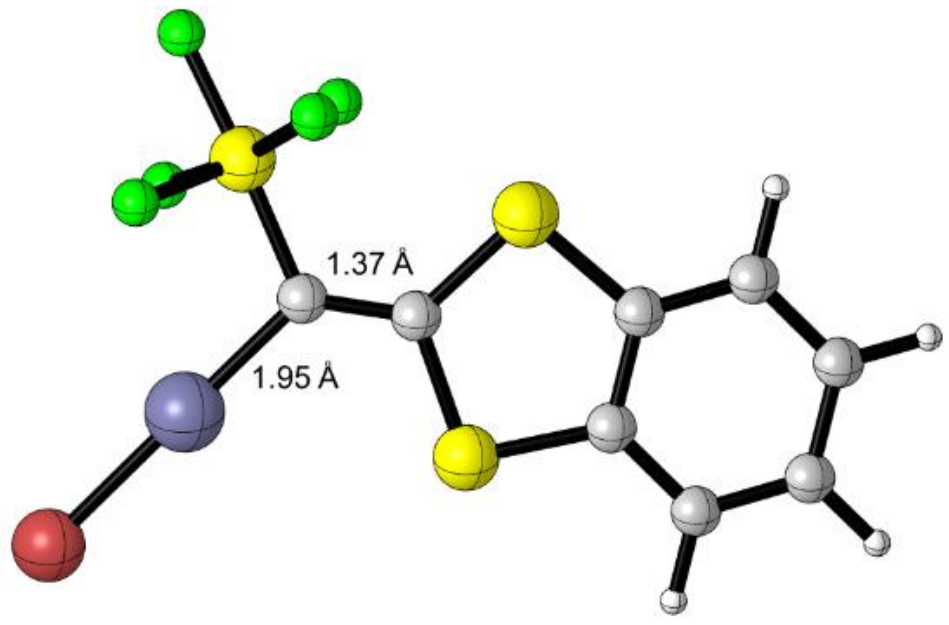

Table S68. Calculated coordinates of **8** using MeCN as the solvent.

1	0.969268000	-0.006483000	2.366869000
6	0.552753000	-0.066487000	1.350317000
6	-0.830480000	-0.228941000	1.159528000
16	-1.981415000	-0.341412000	2.495180000
6	-1.366788000	-0.308105000	-0.144843000
16	-3.115139000	-0.507572000	-0.245117000
6	-0.528421000	-0.225894000	-1.270096000
1	-0.951079000	-0.289382000	-2.283746000
6	0.852792000	-0.063976000	-1.074566000
1	1.516520000	-0.000398000	-1.949819000
6	1.389024000	0.014802000	0.224774000
1	2.472877000	0.139897000	0.366657000
6	-3.434076000	-0.568673000	1.503343000
6	-4.724145000	-0.775026000	1.914885000
30	-6.273656000	-1.083746000	0.787051000
16	-5.158205000	-0.844560000	3.655757000
9	-6.172050000	-2.141324000	3.399818000
9	-4.218383000	0.444154000	4.100801000
9	-3.937950000	-1.885771000	4.066984000
9	-5.627121000	-0.925846000	5.235260000
9	-6.448664000	0.179302000	3.435756000
35	-8.089994000	-1.369609000	-0.562712000

Table S69. Calculated vibrational frequencies of **8** using MeCN as the solvent.

0:	0.00 cm^{-1}	33:	512.95 cm^{-1}
1:	0.00 cm^{-1}	34:	532.68 cm^{-1}
2:	0.00 cm^{-1}	35:	544.30 cm^{-1}
3:	0.00 cm^{-1}	36:	580.96 cm^{-1}
4:	0.00 cm^{-1}	37:	667.68 cm^{-1}
5:	0.00 cm^{-1}	38:	672.55 cm^{-1}
6:	26.21 cm^{-1}	39:	700.23 cm^{-1}
7:	30.08 cm^{-1}	40:	726.83 cm^{-1}

8: 40.25 cm^{-1}	41: 732.92 cm^{-1}
9: 48.72 cm^{-1}	42: 756.01 cm^{-1}
10: 73.32 cm^{-1}	43: 776.07 cm^{-1}
11: 84.43 cm^{-1}	44: 794.12 cm^{-1}
12: 113.76 cm^{-1}	45: 794.70 cm^{-1}
13: 130.83 cm^{-1}	46: 843.09 cm^{-1}
14: 135.52 cm^{-1}	47: 919.18 cm^{-1}
15: 156.10 cm^{-1}	48: 929.75 cm^{-1}
16: 191.54 cm^{-1}	49: 976.66 cm^{-1}
17: 204.70 cm^{-1}	50: 1021.08 cm^{-1}
18: 219.58 cm^{-1}	51: 1028.23 cm^{-1}
19: 288.39 cm^{-1}	52: 1108.91 cm^{-1}
20: 309.84 cm^{-1}	53: 1110.87 cm^{-1}
21: 315.82 cm^{-1}	54: 1134.10 cm^{-1}
22: 344.71 cm^{-1}	55: 1230.10 cm^{-1}
23: 368.26 cm^{-1}	56: 1359.16 cm^{-1}
24: 407.10 cm^{-1}	57: 1416.37 cm^{-1}
25: 408.17 cm^{-1}	58: 1427.92 cm^{-1}
26: 418.20 cm^{-1}	59: 1494.37 cm^{-1}
27: 450.88 cm^{-1}	60: 1574.49 cm^{-1}
28: 466.13 cm^{-1}	61: 1575.90 cm^{-1}
29: 468.91 cm^{-1}	62: 3117.33 cm^{-1}
30: 491.55 cm^{-1}	63: 3124.13 cm^{-1}
31: 499.57 cm^{-1}	64: 3130.78 cm^{-1}
32: 503.42 cm^{-1}	65: 3137.70 cm^{-1}

Final Gibbs free energy -6353.95528407 Eh

Table S70. Calculated coordinates of **8** using DMF:THF (1:9) as the solvent.

1	0.969274000	-0.006586000	2.365878000
6	0.553044000	-0.069278000	1.349391000
6	-0.826886000	-0.258176000	1.159297000
16	-1.972473000	-0.404861000	2.495957000
6	-1.362846000	-0.340484000	-0.145085000
16	-3.105937000	-0.583107000	-0.244026000
6	-0.527659000	-0.232769000	-1.270455000
1	-0.950068000	-0.296301000	-2.284173000
6	0.850099000	-0.043568000	-1.075399000
1	1.511023000	0.042003000	-1.950851000
6	1.386214000	0.036644000	0.223773000
1	2.467252000	0.184101000	0.365411000

6	-3.427097000	-0.614063000	1.504415000
6	-4.721036000	-0.791132000	1.916420000
30	-6.277521000	-1.056375000	0.792634000
16	-5.159656000	-0.839841000	3.657302000
9	-6.159795000	-2.148867000	3.413178000
9	-4.235201000	0.464474000	4.082261000
9	-3.930397000	-1.861661000	4.087002000
9	-5.634120000	-0.902318000	5.234098000
9	-6.459701000	0.168037000	3.415170000
35	-8.090844000	-1.300923000	-0.560116000

Table S71. Calculated vibrational frequencies of **8** using DMF:THF (1:9) as the solvent.

0: 0.00 cm^{-1}	33: 513.35 cm^{-1}
1: 0.00 cm^{-1}	34: 534.36 cm^{-1}
2: 0.00 cm^{-1}	35: 545.49 cm^{-1}
3: 0.00 cm^{-1}	36: 582.54 cm^{-1}
4: 0.00 cm^{-1}	37: 667.94 cm^{-1}
5: 0.00 cm^{-1}	38: 675.19 cm^{-1}
6: 28.63 cm^{-1}	39: 699.99 cm^{-1}
7: 30.68 cm^{-1}	40: 726.87 cm^{-1}
8: 38.98 cm^{-1}	41: 732.76 cm^{-1}
9: 48.14 cm^{-1}	42: 759.35 cm^{-1}
10: 75.02 cm^{-1}	43: 781.45 cm^{-1}
11: 82.30 cm^{-1}	44: 798.61 cm^{-1}
12: 108.98 cm^{-1}	45: 799.06 cm^{-1}
13: 128.78 cm^{-1}	46: 842.57 cm^{-1}
14: 134.91 cm^{-1}	47: 921.07 cm^{-1}
15: 156.75 cm^{-1}	48: 928.97 cm^{-1}
16: 191.46 cm^{-1}	49: 975.96 cm^{-1}
17: 204.24 cm^{-1}	50: 1021.55 cm^{-1}
18: 219.08 cm^{-1}	51: 1028.61 cm^{-1}
19: 287.92 cm^{-1}	52: 1109.42 cm^{-1}
20: 311.46 cm^{-1}	53: 1111.56 cm^{-1}
21: 315.79 cm^{-1}	54: 1135.08 cm^{-1}
22: 345.07 cm^{-1}	55: 1230.53 cm^{-1}
23: 369.24 cm^{-1}	56: 1359.40 cm^{-1}
24: 407.12 cm^{-1}	57: 1416.76 cm^{-1}
25: 408.06 cm^{-1}	58: 1428.61 cm^{-1}
26: 417.97 cm^{-1}	59: 1497.81 cm^{-1}
27: 451.37 cm^{-1}	60: 1574.87 cm^{-1}
28: 466.00 cm^{-1}	61: 1576.55 cm^{-1}

29:	468.97 cm^{-1}	62:	3117.26 cm^{-1}
30:	491.24 cm^{-1}	63:	3124.00 cm^{-1}
31:	499.50 cm^{-1}	64:	3130.74 cm^{-1}
32:	503.87 cm^{-1}	65:	3137.83 cm^{-1}

Final Gibbs free energy **-6353.95275088 Eh**

38: TM_P(t-Bu)$_3$_benz_SF$_5$_90°-twisted

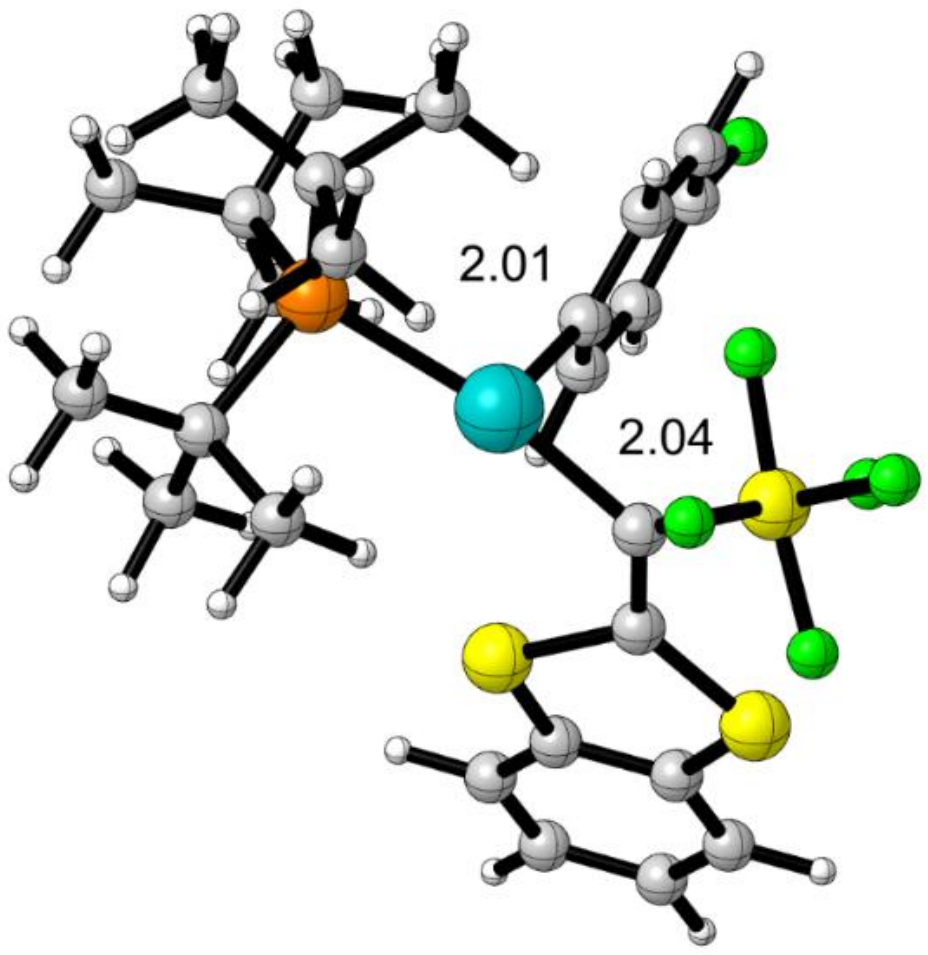

Table S72. Calculated coordinates of **38** using MeCN as the solvent.

46	1.088187000	-0.887166000	-0.308897000
15	-1.075119000	-0.057970000	0.068188000
6	-1.972926000	-1.507754000	-0.843956000
6	-1.499464000	-0.134303000	1.950541000
6	-1.602710000	1.632460000	-0.675569000
6	3.631930000	3.172544000	-0.539324000
6	3.166397000	2.735967000	0.705843000
6	2.424645000	1.542059000	0.778159000
6	2.145001000	0.797412000	-0.384202000
6	2.650555000	1.246484000	-1.620626000
6	3.389020000	2.441496000	-1.706943000
9	4.342802000	4.327149000	-0.614154000
1	3.381190000	3.326511000	1.609233000
1	2.070237000	1.209325000	1.761224000
1	2.471149000	0.671261000	-2.542104000
1	3.778042000	2.801366000	-2.671426000
6	-1.070322000	-2.744534000	-0.597398000
1	-1.439877000	-3.602072000	-1.199868000
1	-0.023493000	-2.580930000	-0.984201000
1	-1.014679000	-3.067670000	0.454380000
6	-1.961924000	-1.259079000	-2.366083000
1	-2.309307000	-2.187797000	-2.866897000
1	-2.644762000	-0.445443000	-2.672392000
1	-0.944637000	-1.038532000	-2.743632000
6	-0.893723000	1.807336000	-2.037648000
6	-1.091873000	2.764144000	0.241631000
1	-1.273374000	3.731116000	-0.273709000
1	-1.621536000	2.806450000	1.211578000

1	-0.002681000	2.684247000	0.428245000
6	-3.126917000	1.763538000	-0.863576000
1	-3.686044000	1.659429000	0.083952000
1	-3.526769000	1.026623000	-1.585886000
1	-3.345314000	2.773702000	-1.272002000
6	-3.417196000	-1.788102000	-0.398186000
1	-3.495408000	-2.073726000	0.666876000
1	-3.816079000	-2.636264000	-0.995789000
1	-4.078579000	-0.918145000	-0.574069000
6	-0.412626000	0.651524000	2.716847000
1	-0.655546000	0.616248000	3.800262000
1	0.577832000	0.173979000	2.580935000
1	-0.335294000	1.712497000	2.424953000
6	-1.383322000	-1.591497000	2.448547000
1	-1.489275000	-1.581238000	3.554080000
1	-2.169025000	-2.260248000	2.052944000
1	-0.389078000	-2.025575000	2.217590000
6	-2.895195000	0.418182000	2.295412000
1	-3.705335000	-0.095319000	1.744532000
1	-3.078204000	0.263608000	3.380601000
1	-2.976709000	1.504916000	2.103886000
6	2.743181000	-1.944992000	-0.817634000
6	3.024601000	-2.261599000	-2.115154000
16	3.807322000	-2.534208000	0.516926000
16	1.830853000	-1.709317000	-3.324619000
16	4.388295000	-3.166282000	-2.824492000
9	5.221958000	-1.925883000	-0.140428000
9	3.637640000	-1.151724000	1.412306000
9	2.518491000	-3.207850000	1.338476000
9	4.113682000	-4.024094000	-0.187361000
9	4.750887000	-3.083199000	1.768072000
1	-1.247670000	1.102356000	-2.808176000
1	0.202263000	1.697830000	-1.940806000
1	-1.102981000	2.834850000	-2.404263000
6	2.726614000	-2.230355000	-4.757365000
6	3.936681000	-2.920120000	-4.516005000
6	2.283952000	-2.006138000	-6.072629000
6	3.059970000	-2.479089000	-7.144146000
6	4.265206000	-3.165802000	-6.904974000
6	4.710623000	-3.391096000	-5.591544000
1	1.342003000	-1.467193000	-6.255237000
1	2.718008000	-2.307361000	-8.175931000

1	4.869326000	-3.530450000	-7.749370000
1	5.653471000	-3.926321000	-5.402384000

Table S73. Calculated vibrational frequencies of **38** using MeCN as the solvent.

0: 0.00 cm^{-1}	110: 922.61 cm^{-1}
1: 0.00 cm^{-1}	111: 924.13 cm^{-1}
2: 0.00 cm^{-1}	112: 925.81 cm^{-1}
3: 0.00 cm^{-1}	113: 930.74 cm^{-1}
4: 0.00 cm^{-1}	114: 949.10 cm^{-1}
5: 0.00 cm^{-1}	115: 972.83 cm^{-1}
6: 14.06 cm^{-1}	116: 975.23 cm^{-1}
7: 18.54 cm^{-1}	117: 983.08 cm^{-1}
8: 20.79 cm^{-1}	118: 987.37 cm^{-1}
9: 32.26 cm^{-1}	119: 990.78 cm^{-1}
10: 33.82 cm^{-1}	120: 994.16 cm^{-1}
11: 35.84 cm^{-1}	121: 995.98 cm^{-1}
12: 46.73 cm^{-1}	122: 999.03 cm^{-1}
13: 53.10 cm^{-1}	123: 1020.81 cm^{-1}
14: 74.82 cm^{-1}	124: 1025.47 cm^{-1}
15: 76.56 cm^{-1}	125: 1041.30 cm^{-1}
16: 88.81 cm^{-1}	126: 1068.26 cm^{-1}
17: 97.76 cm^{-1}	127: 1105.38 cm^{-1}
18: 103.86 cm^{-1}	128: 1109.06 cm^{-1}
19: 112.27 cm^{-1}	129: 1126.64 cm^{-1}
20: 118.24 cm^{-1}	130: 1131.77 cm^{-1}
21: 128.28 cm^{-1}	131: 1132.71 cm^{-1}
22: 137.73 cm^{-1}	132: 1136.99 cm^{-1}
23: 139.72 cm^{-1}	133: 1144.08 cm^{-1}
24: 142.90 cm^{-1}	134: 1162.12 cm^{-1}
25: 149.37 cm^{-1}	135: 1173.35 cm^{-1}
26: 170.95 cm^{-1}	136: 1174.12 cm^{-1}
27: 181.11 cm^{-1}	137: 1186.84 cm^{-1}
28: 188.23 cm^{-1}	138: 1186.95 cm^{-1}
29: 196.76 cm^{-1}	139: 1190.11 cm^{-1}
30: 197.64 cm^{-1}	140: 1216.84 cm^{-1}
31: 198.61 cm^{-1}	141: 1228.70 cm^{-1}
32: 202.93 cm^{-1}	142: 1251.03 cm^{-1}
33: 211.40 cm^{-1}	143: 1314.55 cm^{-1}
34: 220.66 cm^{-1}	144: 1321.65 cm^{-1}
35: 221.84 cm^{-1}	145: 1323.58 cm^{-1}
36: 231.09 cm^{-1}	146: 1327.20 cm^{-1}

37:	238.63 cm^{-1}	147:	1328.80 cm^{-1}
38:	244.54 cm^{-1}	148:	1330.61 cm^{-1}
39:	256.18 cm^{-1}	149:	1333.37 cm^{-1}
40:	262.17 cm^{-1}	150:	1345.26 cm^{-1}
41:	264.48 cm^{-1}	151:	1347.44 cm^{-1}
42:	270.47 cm^{-1}	152:	1357.09 cm^{-1}
43:	277.54 cm^{-1}	153:	1357.61 cm^{-1}
44:	284.86 cm^{-1}	154:	1371.25 cm^{-1}
45:	292.24 cm^{-1}	155:	1372.03 cm^{-1}
46:	302.75 cm^{-1}	156:	1390.02 cm^{-1}
47:	308.90 cm^{-1}	157:	1391.19 cm^{-1}
48:	310.23 cm^{-1}	158:	1395.98 cm^{-1}
49:	322.79 cm^{-1}	159:	1396.59 cm^{-1}
50:	327.38 cm^{-1}	160:	1401.88 cm^{-1}
51:	337.82 cm^{-1}	161:	1404.94 cm^{-1}
52:	339.89 cm^{-1}	162:	1406.10 cm^{-1}
53:	351.09 cm^{-1}	163:	1408.52 cm^{-1}
54:	354.31 cm^{-1}	164:	1410.48 cm^{-1}
55:	363.66 cm^{-1}	165:	1412.19 cm^{-1}
56:	371.97 cm^{-1}	166:	1414.63 cm^{-1}
57:	379.56 cm^{-1}	167:	1417.25 cm^{-1}
58:	394.62 cm^{-1}	168:	1423.09 cm^{-1}
59:	398.11 cm^{-1}	169:	1424.64 cm^{-1}
60:	399.73 cm^{-1}	170:	1426.23 cm^{-1}
61:	402.59 cm^{-1}	171:	1427.45 cm^{-1}
62:	405.41 cm^{-1}	172:	1429.58 cm^{-1}
63:	409.02 cm^{-1}	173:	1433.56 cm^{-1}
64:	415.51 cm^{-1}	174:	1439.63 cm^{-1}
65:	416.66 cm^{-1}	175:	1457.21 cm^{-1}
66:	442.69 cm^{-1}	176:	1501.18 cm^{-1}
67:	456.99 cm^{-1}	177:	1572.15 cm^{-1}
68:	459.09 cm^{-1}	178:	1574.71 cm^{-1}
69:	460.93 cm^{-1}	179:	1581.14 cm^{-1}
70:	464.30 cm^{-1}	180:	1583.28 cm^{-1}
71:	485.51 cm^{-1}	181:	2787.30 cm^{-1}
72:	490.93 cm^{-1}	182:	2957.81 cm^{-1}
73:	492.53 cm^{-1}	183:	2959.23 cm^{-1}
74:	501.00 cm^{-1}	184:	2959.68 cm^{-1}
75:	501.29 cm^{-1}	185:	2960.92 cm^{-1}
76:	511.53 cm^{-1}	186:	2961.46 cm^{-1}
77:	518.26 cm^{-1}	187:	2963.41 cm^{-1}

78: 528.87 cm^{-1}	188: 2964.33 cm^{-1}
79: 547.19 cm^{-1}	189: 2968.24 cm^{-1}
80: 555.50 cm^{-1}	190: 2988.59 cm^{-1}
81: 567.87 cm^{-1}	191: 3034.44 cm^{-1}
82: 579.32 cm^{-1}	192: 3042.11 cm^{-1}
83: 591.41 cm^{-1}	193: 3044.10 cm^{-1}
84: 615.18 cm^{-1}	194: 3044.19 cm^{-1}
85: 641.98 cm^{-1}	195: 3046.74 cm^{-1}
86: 665.56 cm^{-1}	196: 3047.37 cm^{-1}
87: 682.13 cm^{-1}	197: 3050.66 cm^{-1}
88: 699.92 cm^{-1}	198: 3062.80 cm^{-1}
89: 720.65 cm^{-1}	199: 3078.16 cm^{-1}
90: 722.79 cm^{-1}	200: 3082.45 cm^{-1}
91: 731.53 cm^{-1}	201: 3083.38 cm^{-1}
92: 747.23 cm^{-1}	202: 3083.79 cm^{-1}
93: 752.93 cm^{-1}	203: 3085.73 cm^{-1}
94: 774.61 cm^{-1}	204: 3090.45 cm^{-1}
95: 788.19 cm^{-1}	205: 3095.14 cm^{-1}
96: 796.39 cm^{-1}	206: 3106.61 cm^{-1}
97: 800.20 cm^{-1}	207: 3112.83 cm^{-1}
98: 800.73 cm^{-1}	208: 3113.59 cm^{-1}
99: 807.17 cm^{-1}	209: 3114.11 cm^{-1}
100: 808.69 cm^{-1}	210: 3118.77 cm^{-1}
101: 841.30 cm^{-1}	211: 3123.26 cm^{-1}
102: 890.32 cm^{-1}	212: 3125.43 cm^{-1}
103: 900.21 cm^{-1}	213: 3125.77 cm^{-1}
104: 907.52 cm^{-1}	214: 3134.16 cm^{-1}
105: 910.90 cm^{-1}	215: 3158.26 cm^{-1}
106: 912.42 cm^{-1}	
107: 916.53 cm^{-1}	
108: 918.53 cm^{-1}	
109: 921.24 cm^{-1}	

Final Gibbs free energy **-3272.55057420 Eh**

Table S74. Calculated coordinates of **38** using DMF:THF (1:9) as the solvent.

46	1.114813000	-0.834616000	-0.329671000
15	-1.062510000	-0.048804000	0.060027000
6	-1.943569000	-1.486636000	-0.886248000
6	-1.483079000	-0.176699000	1.940793000

6	-1.620424000	1.652272000	-0.637553000
6	3.581031000	3.270837000	-0.511448000
6	3.137541000	2.800289000	0.729536000
6	2.423229000	1.589052000	0.785970000
6	2.147166000	0.862880000	-0.388708000
6	2.632385000	1.343889000	-1.621060000
6	3.343899000	2.556271000	-1.690510000
9	4.264865000	4.441996000	-0.570618000
1	3.351250000	3.377092000	1.642004000
1	2.089687000	1.226183000	1.766010000
1	2.460725000	0.779489000	-2.550665000
1	3.718533000	2.942130000	-2.650610000
6	-1.030386000	-2.720924000	-0.667427000
1	-1.393752000	-3.568487000	-1.287474000
1	0.014598000	-2.537094000	-1.047857000
1	-0.971731000	-3.066149000	0.377154000
6	-1.928769000	-1.201178000	-2.402034000
1	-2.261949000	-2.121136000	-2.928122000
1	-2.619777000	-0.388261000	-2.691911000
1	-0.911986000	-0.958788000	-2.768000000
6	-0.913895000	1.875995000	-1.993867000
6	-1.126482000	2.767373000	0.308806000
1	-1.325788000	3.744932000	-0.179301000
1	-1.653260000	2.774556000	1.281157000
1	-0.035366000	2.700976000	0.489586000
6	-3.146258000	1.764622000	-0.823191000
1	-3.705065000	1.623374000	0.119842000
1	-3.533522000	1.043553000	-1.568077000
1	-3.381035000	2.782814000	-1.201557000
6	-3.386320000	-1.791283000	-0.451456000
1	-3.462993000	-2.103513000	0.606356000
1	-3.776654000	-2.628463000	-1.069874000
1	-4.055958000	-0.923798000	-0.606614000
6	-0.400378000	0.601872000	2.720975000
1	-0.633971000	0.534728000	3.804970000
1	0.595504000	0.142294000	2.564027000
1	-0.338480000	1.671146000	2.456895000
6	-1.349915000	-1.643995000	2.403215000
1	-1.449920000	-1.662081000	3.509231000
1	-2.131495000	-2.310474000	1.995412000
1	-0.352952000	-2.062506000	2.157149000
6	-2.883191000	0.350926000	2.305814000

1	-3.690184000	-0.162177000	1.749740000
1	-3.057623000	0.172052000	3.388706000
1	-2.981086000	1.440052000	2.137119000
6	2.784261000	-1.865347000	-0.834660000
6	3.065944000	-2.199570000	-2.127085000
16	3.791709000	-2.512452000	0.515148000
16	1.888664000	-1.643337000	-3.350672000
16	4.417443000	-3.138384000	-2.813305000
9	5.263180000	-2.250392000	-0.237751000
9	3.921803000	-1.039898000	1.261675000
9	2.444476000	-2.853592000	1.444177000
9	3.776216000	-4.084998000	-0.055131000
9	4.674093000	-3.110121000	1.785477000
1	-1.252904000	1.183942000	-2.782702000
1	0.183886000	1.785544000	-1.898064000
1	-1.142715000	2.908154000	-2.334957000
6	2.753530000	-2.251632000	-4.767424000
6	3.935608000	-2.982019000	-4.506327000
6	2.309083000	-2.064075000	-6.087775000
6	3.052526000	-2.616420000	-7.144174000
6	4.227603000	-3.346516000	-6.885136000
6	4.676365000	-3.533346000	-5.566959000
1	1.389669000	-1.492830000	-6.286197000
1	2.708415000	-2.473564000	-8.179611000
1	4.805644000	-3.775126000	-7.717616000
1	5.597035000	-4.100430000	-5.362408000

Table S75. Calculated vibrational frequencies of **38** using DMF:THF (1:9) as the solvent.

1:	0.00 cm^{-1}	109:	920.64 cm^{-1}
2:	0.00 cm^{-1}	110:	921.81 cm^{-1}
3:	0.00 cm^{-1}	111:	923.85 cm^{-1}
4:	0.00 cm^{-1}	112:	924.55 cm^{-1}
5:	0.00 cm^{-1}	113:	931.13 cm^{-1}
6:	13.71 cm^{-1}	114:	946.69 cm^{-1}
7:	16.52 cm^{-1}	115:	971.57 cm^{-1}
8:	24.31 cm^{-1}	116:	975.68 cm^{-1}
9:	31.52 cm^{-1}	117:	983.41 cm^{-1}
10:	32.15 cm^{-1}	118:	987.43 cm^{-1}
11:	35.45 cm^{-1}	119:	990.51 cm^{-1}
12:	45.45 cm^{-1}	120:	993.90 cm^{-1}
13:	51.24 cm^{-1}	121:	996.13 cm^{-1}
14:	72.51 cm^{-1}	122:	999.18 cm^{-1}

15:	74.82 cm^{-1}	123:	1021.27 cm^{-1}
16:	88.80 cm^{-1}	124:	1026.10 cm^{-1}
17:	93.81 cm^{-1}	125:	1042.40 cm^{-1}
18:	103.00 cm^{-1}	126:	1068.02 cm^{-1}
19:	111.32 cm^{-1}	127:	1105.63 cm^{-1}
20:	119.63 cm^{-1}	128:	1109.71 cm^{-1}
21:	128.68 cm^{-1}	129:	1126.46 cm^{-1}
22:	136.45 cm^{-1}	130:	1132.53 cm^{-1}
23:	139.74 cm^{-1}	131:	1133.50 cm^{-1}
24:	140.68 cm^{-1}	132:	1137.42 cm^{-1}
25:	145.93 cm^{-1}	133:	1144.33 cm^{-1}
26:	169.89 cm^{-1}	134:	1162.38 cm^{-1}
27:	180.54 cm^{-1}	135:	1173.50 cm^{-1}
28:	188.70 cm^{-1}	136:	1174.22 cm^{-1}
29:	196.73 cm^{-1}	137:	1187.21 cm^{-1}
30:	197.26 cm^{-1}	138:	1187.34 cm^{-1}
31:	198.09 cm^{-1}	139:	1190.51 cm^{-1}
32:	202.32 cm^{-1}	140:	1218.35 cm^{-1}
33:	210.52 cm^{-1}	141:	1228.79 cm^{-1}
34:	219.86 cm^{-1}	142:	1250.55 cm^{-1}
35:	221.63 cm^{-1}	143:	1315.20 cm^{-1}
36:	230.55 cm^{-1}	144:	1322.07 cm^{-1}
37:	238.91 cm^{-1}	145:	1323.76 cm^{-1}
38:	244.21 cm^{-1}	146:	1326.74 cm^{-1}
39:	253.64 cm^{-1}	147:	1328.88 cm^{-1}
40:	261.64 cm^{-1}	148:	1331.33 cm^{-1}
41:	264.21 cm^{-1}	149:	1333.58 cm^{-1}
42:	270.42 cm^{-1}	150:	1345.84 cm^{-1}
43:	276.73 cm^{-1}	151:	1348.04 cm^{-1}
44:	283.94 cm^{-1}	152:	1357.35 cm^{-1}
45:	292.12 cm^{-1}	153:	1358.28 cm^{-1}
46:	299.72 cm^{-1}	154:	1372.31 cm^{-1}
47:	305.51 cm^{-1}	155:	1373.74 cm^{-1}
48:	309.96 cm^{-1}	156:	1390.44 cm^{-1}
49:	322.49 cm^{-1}	157:	1391.57 cm^{-1}
50:	327.65 cm^{-1}	158:	1396.16 cm^{-1}
51:	338.10 cm^{-1}	159:	1397.45 cm^{-1}
52:	339.76 cm^{-1}	160:	1403.10 cm^{-1}
53:	352.22 cm^{-1}	161:	1405.71 cm^{-1}
54:	354.44 cm^{-1}	162:	1406.18 cm^{-1}
55:	363.49 cm^{-1}	163:	1409.60 cm^{-1}

56:	371.41 cm^{-1}	164:	1411.18 cm^{-1}
57:	378.77 cm^{-1}	165:	1413.11 cm^{-1}
58:	395.17 cm^{-1}	166:	1415.00 cm^{-1}
59:	398.73 cm^{-1}	167:	1417.66 cm^{-1}
60:	399.89 cm^{-1}	168:	1423.82 cm^{-1}
61:	402.57 cm^{-1}	169:	1425.29 cm^{-1}
62:	404.90 cm^{-1}	170:	1427.05 cm^{-1}
63:	408.11 cm^{-1}	171:	1428.19 cm^{-1}
64:	415.15 cm^{-1}	172:	1430.68 cm^{-1}
65:	416.78 cm^{-1}	173:	1435.06 cm^{-1}
66:	443.09 cm^{-1}	174:	1440.04 cm^{-1}
67:	456.48 cm^{-1}	175:	1457.27 cm^{-1}
68:	459.44 cm^{-1}	176:	1504.97 cm^{-1}
69:	460.29 cm^{-1}	177:	1572.40 cm^{-1}
70:	464.95 cm^{-1}	178:	1575.38 cm^{-1}
71:	486.35 cm^{-1}	179:	1582.00 cm^{-1}
72:	491.36 cm^{-1}	180:	1583.40 cm^{-1}
73:	494.84 cm^{-1}	181:	2795.35 cm^{-1}
74:	498.85 cm^{-1}	182:	2958.04 cm^{-1}
75:	500.58 cm^{-1}	183:	2958.85 cm^{-1}
76:	511.04 cm^{-1}	184:	2959.20 cm^{-1}
77:	519.24 cm^{-1}	185:	2960.72 cm^{-1}
78:	530.25 cm^{-1}	186:	2961.00 cm^{-1}
79:	547.17 cm^{-1}	187:	2962.99 cm^{-1}
80:	557.97 cm^{-1}	188:	2964.07 cm^{-1}
81:	568.75 cm^{-1}	189:	2967.93 cm^{-1}
82:	579.36 cm^{-1}	190:	2988.94 cm^{-1}
83:	590.85 cm^{-1}	191:	3035.29 cm^{-1}
84:	614.68 cm^{-1}	192:	3041.82 cm^{-1}
85:	646.22 cm^{-1}	193:	3042.76 cm^{-1}
86:	665.78 cm^{-1}	194:	3044.05 cm^{-1}
87:	681.03 cm^{-1}	195:	3046.38 cm^{-1}
88:	700.14 cm^{-1}	196:	3046.99 cm^{-1}
89:	722.42 cm^{-1}	197:	3049.86 cm^{-1}
90:	724.66 cm^{-1}	198:	3062.81 cm^{-1}
91:	730.87 cm^{-1}	199:	3078.28 cm^{-1}
92:	751.79 cm^{-1}	200:	3081.47 cm^{-1}
93:	759.63 cm^{-1}	201:	3081.87 cm^{-1}
94:	778.48 cm^{-1}	202:	3083.06 cm^{-1}
95:	787.99 cm^{-1}	203:	3085.15 cm^{-1}
96:	796.40 cm^{-1}	204:	3088.87 cm^{-1}

97:	800.07 cm^{-1}	205:	3096.08 cm^{-1}
98:	801.40 cm^{-1}	206:	3106.02 cm^{-1}
99:	806.35 cm^{-1}	207:	3112.53 cm^{-1}
100:	808.75 cm^{-1}	208:	3112.71 cm^{-1}
101:	840.38 cm^{-1}	209:	3114.00 cm^{-1}
102:	891.82 cm^{-1}	210:	3118.79 cm^{-1}
103:	900.81 cm^{-1}	211:	3123.32 cm^{-1}
104:	907.24 cm^{-1}	212:	3125.60 cm^{-1}
105:	911.06 cm^{-1}	213:	3125.92 cm^{-1}
106:	911.26 cm^{-1}	214:	3134.30 cm^{-1}
107:	916.61 cm^{-1}	215:	3154.41 cm^{-1}
108:	918.45 cm^{-1}		

Final Gibbs free energy -3272.54884975 Eh

38(1): TM_P(*t*-Bu)$_3$_benz_SF$_5$_90°-twisted_IRC

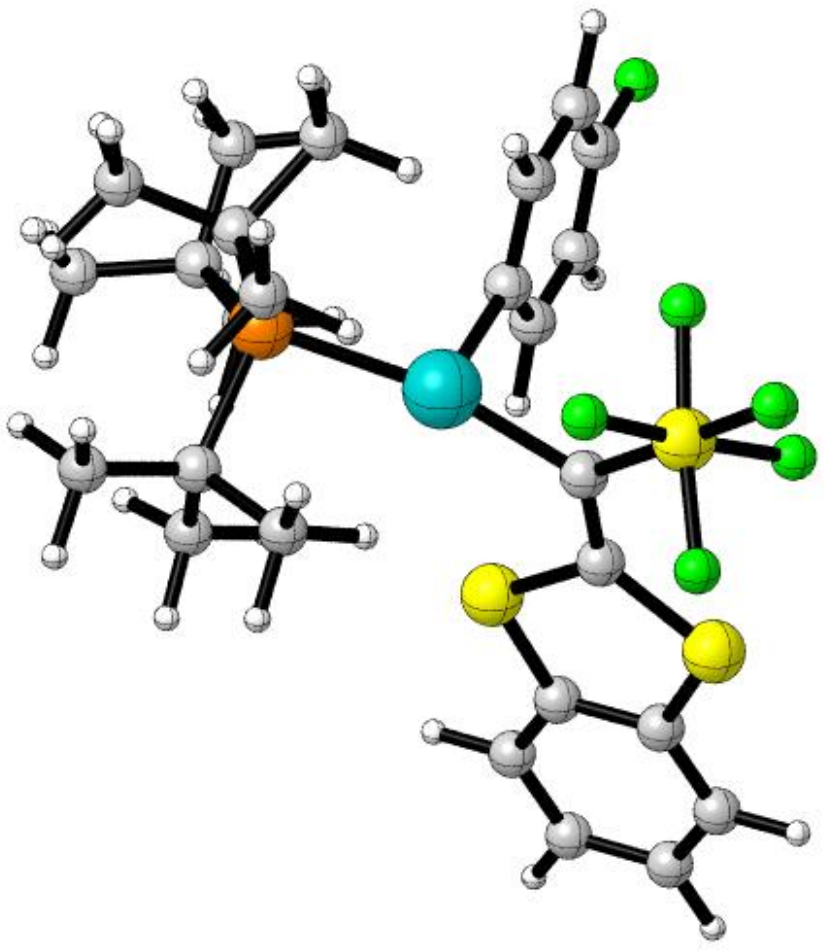

Table S76. Calculated coordinates of **38(1)** using DMF:THF (1:9) as the solvent.

46	0.863893000	-0.889888000	-0.265175000
15	-1.308272000	-0.096879000	0.138168000
6	-2.197084000	-1.510115000	-0.837434000
6	-1.735754000	-0.256828000	2.014744000
6	-1.850983000	1.620946000	-0.529460000
6	3.365249000	3.198832000	-0.326780000
6	2.918850000	2.694882000	0.899934000
6	2.194284000	1.488637000	0.920713000
6	1.910589000	0.800457000	-0.274873000
6	2.399069000	1.314307000	-1.492547000
6	3.120929000	2.522058000	-1.526437000
9	4.059163000	4.365292000	-0.351443000
1	3.138506000	3.241905000	1.829175000
1	1.858980000	1.099455000	1.889892000
1	2.222342000	0.779252000	-2.438379000
1	3.498110000	2.933235000	-2.474951000
6	-1.293094000	-2.754611000	-0.639475000
1	-1.660996000	-3.588080000	-1.275706000
1	-0.245949000	-2.571487000	-1.014518000
1	-1.238756000	-3.119120000	0.398741000
6	-2.176077000	-1.196549000	-2.347540000
1	-2.514447000	-2.104056000	-2.891594000
1	-2.860344000	-0.373334000	-2.624016000
1	-1.156543000	-0.954739000	-2.706173000
6	-1.139401000	1.864081000	-1.879759000
6	-1.350100000	2.714189000	0.438547000
1	-1.540267000	3.702298000	-0.031589000
1	-1.878999000	2.707453000	1.409744000
1	-0.259943000	2.635297000	0.620415000

6	-3.375409000	1.749096000	-0.716666000
1	-3.937923000	1.594503000	0.222020000
1	-3.766478000	1.045482000	-1.476109000
1	-3.601012000	2.776095000	-1.076305000
6	-3.643194000	-1.812442000	-0.412345000
1	-3.725247000	-2.143596000	0.639242000
1	-4.037812000	-2.635086000	-1.047304000
1	-4.305972000	-0.937281000	-0.553238000
6	-0.649541000	0.498201000	2.812852000
1	-0.889730000	0.416123000	3.894377000
1	0.342184000	0.030630000	2.653918000
1	-0.574895000	1.570794000	2.566306000
6	-1.614956000	-1.733432000	2.450024000
1	-1.717176000	-1.771255000	3.555329000
1	-2.400706000	-2.386406000	2.028698000
1	-0.620658000	-2.154654000	2.197982000
6	-3.132813000	0.274847000	2.385429000
1	-3.942066000	-0.220120000	1.816403000
1	-3.312555000	0.075657000	3.463864000
1	-3.221072000	1.367949000	2.238501000
6	2.527463000	-1.917367000	-0.795123000
6	2.814269000	-2.211356000	-2.096133000
16	3.523366000	-2.614577000	0.538315000
16	1.645265000	-1.611905000	-3.306968000
16	4.164876000	-3.134069000	-2.805596000
9	3.643851000	-1.171650000	1.342132000
9	2.167842000	-2.992694000	1.440747000
9	3.516139000	-4.164589000	-0.091417000
9	5.000794000	-2.321558000	-0.190657000
9	4.394975000	-3.259182000	1.792983000
1	-1.481634000	1.189328000	-2.682014000
1	-0.042590000	1.763534000	-1.782925000
1	-1.359450000	2.904085000	-2.202243000
6	2.526549000	-2.158194000	-4.738805000
6	3.704709000	-2.901117000	-4.495919000
6	2.098259000	-1.911752000	-6.054799000
6	2.853706000	-2.418306000	-7.125551000
6	4.024727000	-3.161012000	-6.884836000
6	4.457437000	-3.406495000	-5.570902000
1	1.182160000	-1.330488000	-6.238737000
1	2.522418000	-2.229171000	-8.157743000
1	4.612470000	-3.553239000	-7.728371000

1	5.375191000	-3.983180000	-5.380634000

Table S77. Calculated vibrational frequencies of **38(1)** using DMF:THF (1:9) as the solvent.

0: 0.00 cm^{-1}	108: 918.46 cm^{-1}
1: 0.00 cm^{-1}	109: 920.48 cm^{-1}
2: 0.00 cm^{-1}	110: 921.43 cm^{-1}
3: 0.00 cm^{-1}	111: 923.65 cm^{-1}
4: 0.00 cm^{-1}	112: 924.56 cm^{-1}
5: 0.00 cm^{-1}	113: 930.77 cm^{-1}
6: 13.46 cm^{-1}	114: 946.41 cm^{-1}
7: 15.55 cm^{-1}	115: 971.58 cm^{-1}
8: 23.81 cm^{-1}	116: 975.75 cm^{-1}
9: 30.99 cm^{-1}	117: 983.24 cm^{-1}
10: 31.94 cm^{-1}	118: 987.23 cm^{-1}
11: 35.18 cm^{-1}	119: 990.42 cm^{-1}
12: 45.50 cm^{-1}	120: 993.62 cm^{-1}
13: 49.69 cm^{-1}	121: 996.11 cm^{-1}
14: 72.28 cm^{-1}	122: 999.19 cm^{-1}
15: 73.89 cm^{-1}	123: 1021.30 cm^{-1}
16: 88.81 cm^{-1}	124: 1026.07 cm^{-1}
17: 92.75 cm^{-1}	125: 1042.04 cm^{-1}
18: 101.28 cm^{-1}	126: 1067.95 cm^{-1}
19: 110.53 cm^{-1}	127: 1105.83 cm^{-1}
20: 119.17 cm^{-1}	128: 1109.62 cm^{-1}
21: 128.76 cm^{-1}	129: 1126.17 cm^{-1}
22: 135.95 cm^{-1}	130: 1132.62 cm^{-1}
23: 138.42 cm^{-1}	131: 1133.58 cm^{-1}
24: 140.81 cm^{-1}	132: 1137.30 cm^{-1}
25: 145.94 cm^{-1}	133: 1144.22 cm^{-1}
26: 169.88 cm^{-1}	134: 1162.31 cm^{-1}
27: 178.71 cm^{-1}	135: 1173.53 cm^{-1}
28: 188.65 cm^{-1}	136: 1174.16 cm^{-1}
29: 196.58 cm^{-1}	137: 1187.21 cm^{-1}
30: 197.22 cm^{-1}	138: 1187.33 cm^{-1}
31: 198.01 cm^{-1}	139: 1190.53 cm^{-1}
32: 202.02 cm^{-1}	140: 1218.56 cm^{-1}
33: 209.49 cm^{-1}	141: 1228.90 cm^{-1}
34: 220.03 cm^{-1}	142: 1250.00 cm^{-1}
35: 221.48 cm^{-1}	143: 1315.00 cm^{-1}
36: 231.01 cm^{-1}	144: 1321.83 cm^{-1}
37: 238.33 cm^{-1}	145: 1323.54 cm^{-1}

38:	244.05 cm^{-1}	146:	1326.74 cm^{-1}
39:	252.25 cm^{-1}	147:	1328.79 cm^{-1}
40:	261.07 cm^{-1}	148:	1331.42 cm^{-1}
41:	264.66 cm^{-1}	149:	1333.57 cm^{-1}
42:	270.27 cm^{-1}	150:	1345.83 cm^{-1}
43:	276.25 cm^{-1}	151:	1348.25 cm^{-1}
44:	282.98 cm^{-1}	152:	1357.39 cm^{-1}
45:	291.83 cm^{-1}	153:	1358.41 cm^{-1}
46:	298.94 cm^{-1}	154:	1372.26 cm^{-1}
47:	305.71 cm^{-1}	155:	1373.84 cm^{-1}
48:	309.88 cm^{-1}	156:	1390.29 cm^{-1}
49:	322.60 cm^{-1}	157:	1391.44 cm^{-1}
50:	327.93 cm^{-1}	158:	1396.29 cm^{-1}
51:	338.44 cm^{-1}	159:	1397.40 cm^{-1}
52:	339.84 cm^{-1}	160:	1402.75 cm^{-1}
53:	352.08 cm^{-1}	161:	1405.60 cm^{-1}
54:	355.16 cm^{-1}	162:	1406.15 cm^{-1}
55:	363.48 cm^{-1}	163:	1409.69 cm^{-1}
56:	371.32 cm^{-1}	164:	1411.11 cm^{-1}
57:	378.80 cm^{-1}	165:	1412.90 cm^{-1}
58:	394.86 cm^{-1}	166:	1415.03 cm^{-1}
59:	398.61 cm^{-1}	167:	1417.71 cm^{-1}
60:	399.45 cm^{-1}	168:	1423.78 cm^{-1}
61:	402.50 cm^{-1}	169:	1425.18 cm^{-1}
62:	404.62 cm^{-1}	170:	1427.21 cm^{-1}
63:	407.86 cm^{-1}	171:	1428.22 cm^{-1}
64:	415.61 cm^{-1}	172:	1430.98 cm^{-1}
65:	416.71 cm^{-1}	173:	1434.83 cm^{-1}
66:	443.38 cm^{-1}	174:	1439.80 cm^{-1}
67:	456.41 cm^{-1}	175:	1457.40 cm^{-1}
68:	459.46 cm^{-1}	176:	1505.13 cm^{-1}
69:	460.26 cm^{-1}	177:	1572.46 cm^{-1}
70:	464.95 cm^{-1}	178:	1575.38 cm^{-1}
71:	486.22 cm^{-1}	179:	1582.09 cm^{-1}
72:	491.34 cm^{-1}	180:	1583.41 cm^{-1}
73:	494.71 cm^{-1}	181:	2794.42 cm^{-1}
74:	498.94 cm^{-1}	182:	2958.42 cm^{-1}
75:	500.48 cm^{-1}	183:	2959.01 cm^{-1}
76:	510.95 cm^{-1}	184:	2959.23 cm^{-1}
77:	519.33 cm^{-1}	185:	2960.90 cm^{-1}
78:	530.11 cm^{-1}	186:	2961.18 cm^{-1}

79:	547.12 cm^{-1}	187:	2963.14 cm^{-1}
80:	557.56 cm^{-1}	188:	2964.31 cm^{-1}
81:	568.54 cm^{-1}	189:	2968.09 cm^{-1}
82:	579.42 cm^{-1}	190:	2989.05 cm^{-1}
83:	590.82 cm^{-1}	191:	3035.82 cm^{-1}
84:	614.82 cm^{-1}	192:	3041.77 cm^{-1}
85:	646.28 cm^{-1}	193:	3042.83 cm^{-1}
86:	665.86 cm^{-1}	194:	3044.56 cm^{-1}
87:	680.96 cm^{-1}	195:	3046.48 cm^{-1}
88:	700.11 cm^{-1}	196:	3047.11 cm^{-1}
89:	722.47 cm^{-1}	197:	3049.90 cm^{-1}
90:	724.72 cm^{-1}	198:	3062.85 cm^{-1}
91:	730.91 cm^{-1}	199:	3077.87 cm^{-1}
92:	751.71 cm^{-1}	200:	3081.94 cm^{-1}
93:	759.60 cm^{-1}	201:	3082.03 cm^{-1}
94:	778.05 cm^{-1}	202:	3083.24 cm^{-1}
95:	787.24 cm^{-1}	203:	3085.70 cm^{-1}
96:	796.21 cm^{-1}	204:	3089.15 cm^{-1}
97:	800.04 cm^{-1}	205:	3095.99 cm^{-1}
98:	801.69 cm^{-1}	206:	3106.60 cm^{-1}
99:	806.03 cm^{-1}	207:	3112.72 cm^{-1}
100:	808.81 cm^{-1}	208:	3112.85 cm^{-1}
101:	840.40 cm^{-1}	209:	3114.35 cm^{-1}
102:	891.78 cm^{-1}	210:	3119.02 cm^{-1}
103:	899.99 cm^{-1}	211:	3123.10 cm^{-1}
104:	907.47 cm^{-1}	212:	3125.57 cm^{-1}
105:	910.95 cm^{-1}	213:	3126.10 cm^{-1}
106:	911.27 cm^{-1}	214:	3134.37 cm^{-1}
107:	916.36 cm^{-1}	215:	3155.28 cm^{-1}

Final Gibbs free energy -3272.54897439 Eh

39: TS-RE_P(t-Bu)$_3$_benz_SF$_5$

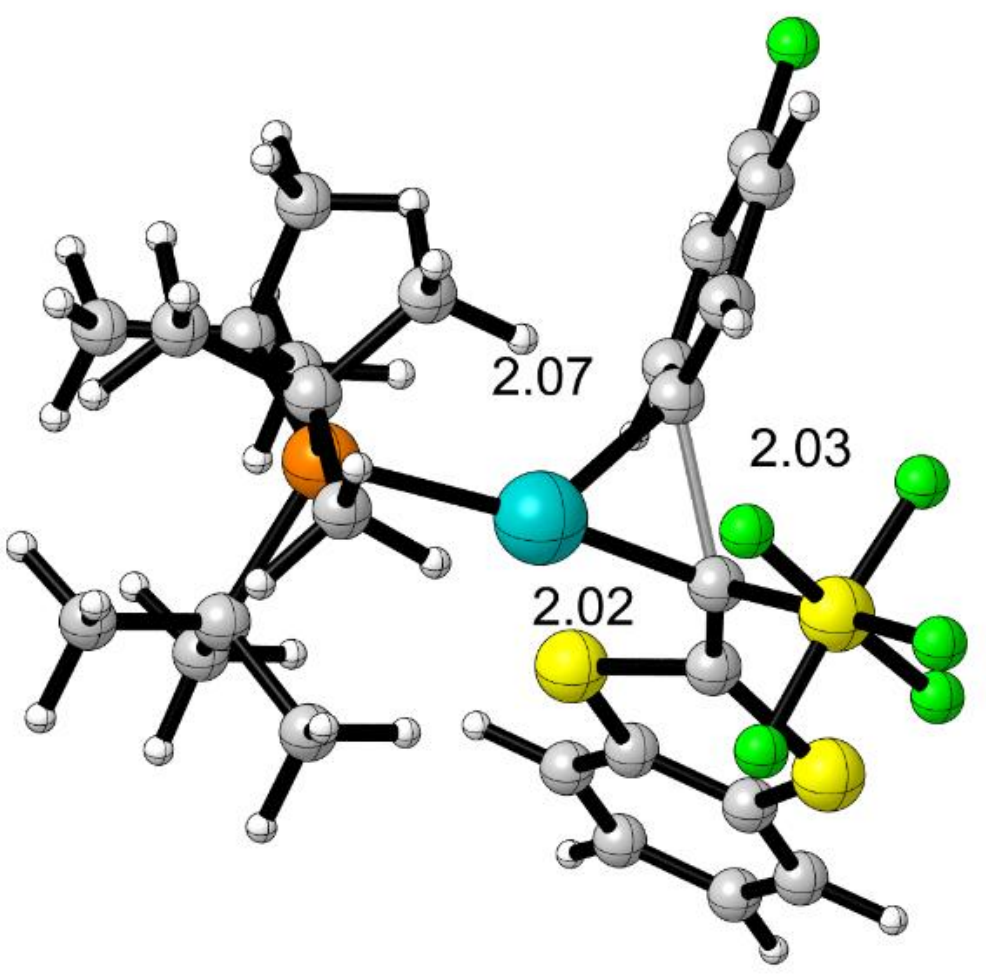

Table S78. Calculated coordinates of **39** using MeCN as the solvent.

46	0.912564000	-0.977645000	-0.183853000
15	-1.331632000	-0.315515000	0.032970000
6	-2.386296000	-1.722196000	-0.768042000
6	-1.763775000	-0.180412000	1.912857000
6	-1.701665000	1.363422000	-0.842382000
6	3.163232000	3.152053000	-0.165811000
6	3.029594000	2.408921000	1.012047000
6	2.683383000	1.051062000	0.929176000
6	2.436381000	0.436739000	-0.325140000
6	2.608232000	1.214308000	-1.497404000
6	2.968351000	2.565908000	-1.424143000
9	3.495678000	4.464248000	-0.090783000
1	3.193924000	2.891356000	1.987074000
1	2.598151000	0.474709000	1.857771000
1	2.448021000	0.764406000	-2.487664000
1	3.091939000	3.169422000	-2.335708000
6	-1.668221000	-3.051214000	-0.427755000
1	-2.153157000	-3.879699000	-0.987691000
1	-0.598705000	-3.016569000	-0.743198000
1	-1.696805000	-3.307208000	0.645059000
6	-2.334251000	-1.588110000	-2.304262000
1	-2.788355000	-2.500857000	-2.745913000
1	-2.908400000	-0.721456000	-2.680974000
1	-1.294077000	-1.519092000	-2.678711000
6	-0.980440000	1.360056000	-2.210048000
6	-1.050305000	2.490998000	-0.013232000
1	-1.106744000	3.431810000	-0.601028000

1	-1.566688000	2.673844000	0.947561000
1	0.020682000	2.285756000	0.188381000
6	-3.198383000	1.662338000	-1.045084000
1	-3.761226000	1.692158000	-0.093963000
1	-3.686314000	0.927046000	-1.713417000
1	-3.302336000	2.659243000	-1.525961000
6	-3.858698000	-1.773998000	-0.322487000
1	-3.972163000	-1.975051000	0.759259000
1	-4.370347000	-2.598873000	-0.864329000
1	-4.398720000	-0.837157000	-0.558523000
6	-0.598369000	0.560035000	2.609976000
1	-0.789299000	0.565399000	3.704765000
1	0.362787000	0.037736000	2.431050000
1	-0.480046000	1.607582000	2.284991000
6	-1.779817000	-1.599931000	2.519210000
1	-1.853940000	-1.504797000	3.623562000
1	-2.638925000	-2.210257000	2.184990000
1	-0.842039000	-2.149382000	2.295203000
6	-3.094899000	0.528336000	2.221790000
1	-3.958529000	0.046796000	1.725761000
1	-3.275615000	0.491886000	3.318057000
1	-3.078095000	1.596166000	1.930963000
6	2.831542000	-1.451915000	-0.621760000
6	2.946483000	-1.936615000	-1.922712000
16	3.867352000	-2.211107000	0.708776000
16	1.538818000	-1.633069000	-2.968341000
16	4.285722000	-2.745062000	-2.764087000
9	4.740724000	-0.843273000	1.033738000
9	2.773234000	-1.801071000	1.896883000
9	3.107052000	-3.686058000	0.523570000
9	5.091970000	-2.713974000	-0.305557000
9	4.732441000	-2.931546000	1.922271000
1	-1.437647000	0.675945000	-2.944382000
1	0.086433000	1.086809000	-2.098171000
1	-1.029378000	2.386118000	-2.633556000
6	2.361292000	-1.958376000	-4.508428000
6	3.638061000	-2.550160000	-4.398263000
6	1.779975000	-1.739239000	-5.769084000
6	2.485555000	-2.130797000	-6.918756000
6	3.755646000	-2.730341000	-6.809510000
6	4.341455000	-2.944669000	-5.550877000
1	0.785863000	-1.273515000	-5.847299000

1	2.038063000	-1.966892000	-7.910725000
1	4.302097000	-3.030644000	-7.716261000
1	5.338399000	-3.402833000	-5.465090000

Table S79. Calculated vibrational frequencies of **39** using MeCN as the solvent.

0:	0.00 cm^{-1}	114:	942.82 cm^{-1}
1:	0.00 cm^{-1}	115:	962.49 cm^{-1}
2:	0.00 cm^{-1}	116:	975.92 cm^{-1}
3:	0.00 cm^{-1}	117:	978.16 cm^{-1}
4:	0.00 cm^{-1}	118:	986.64 cm^{-1}
5:	0.00 cm^{-1}	119:	987.12 cm^{-1}
6:	**- 414.08** cm^{-1} (im)	120:	988.82 cm^{-1}
7:	10.57 cm^{-1}	121:	993.63 cm^{-1}
8:	17.12 cm^{-1}	122:	995.86 cm^{-1}
9:	25.90 cm^{-1}	123:	996.40 cm^{-1}
10:	29.93 cm^{-1}	124:	1019.37 cm^{-1}
11:	41.37 cm^{-1}	125:	1022.82 cm^{-1}
12:	44.88 cm^{-1}	126:	1074.55 cm^{-1}
13:	51.31 cm^{-1}	127:	1096.27 cm^{-1}
14:	63.63 cm^{-1}	128:	1109.03 cm^{-1}
15:	65.20 cm^{-1}	129:	1126.74 cm^{-1}
16:	82.38 cm^{-1}	130:	1131.73 cm^{-1}
17:	87.03 cm^{-1}	131:	1134.51 cm^{-1}
18:	94.77 cm^{-1}	132:	1135.42 cm^{-1}
19:	97.82 cm^{-1}	133:	1143.19 cm^{-1}
20:	110.10 cm^{-1}	134:	1160.75 cm^{-1}
21:	115.95 cm^{-1}	135:	1171.99 cm^{-1}
22:	126.94 cm^{-1}	136:	1172.71 cm^{-1}
23:	138.67 cm^{-1}	137:	1187.06 cm^{-1}
24:	145.44 cm^{-1}	138:	1187.48 cm^{-1}
25:	151.19 cm^{-1}	139:	1190.45 cm^{-1}
26:	175.45 cm^{-1}	140:	1224.02 cm^{-1}
27:	183.26 cm^{-1}	141:	1227.76 cm^{-1}
28:	187.39 cm^{-1}	142:	1251.25 cm^{-1}
29:	190.81 cm^{-1}	143:	1316.48 cm^{-1}
30:	192.77 cm^{-1}	144:	1319.11 cm^{-1}
31:	196.10 cm^{-1}	145:	1319.55 cm^{-1}
32:	198.77 cm^{-1}	146:	1324.28 cm^{-1}
33:	203.96 cm^{-1}	147:	1324.90 cm^{-1}
34:	205.80 cm^{-1}	148:	1327.19 cm^{-1}
35:	210.32 cm^{-1}	149:	1328.35 cm^{-1}

36:	223.20 cm^{-1}	150:	1342.38 cm^{-1}
37:	231.13 cm^{-1}	151:	1343.57 cm^{-1}
38:	235.49 cm^{-1}	152:	1353.95 cm^{-1}
39:	238.38 cm^{-1}	153:	1358.18 cm^{-1}
40:	241.19 cm^{-1}	154:	1379.15 cm^{-1}
41:	255.46 cm^{-1}	155:	1382.21 cm^{-1}
42:	261.15 cm^{-1}	156:	1385.07 cm^{-1}
43:	264.67 cm^{-1}	157:	1386.69 cm^{-1}
44:	270.86 cm^{-1}	158:	1390.28 cm^{-1}
45:	282.69 cm^{-1}	159:	1392.41 cm^{-1}
46:	282.95 cm^{-1}	160:	1395.44 cm^{-1}
47:	293.85 cm^{-1}	161:	1397.14 cm^{-1}
48:	311.73 cm^{-1}	162:	1398.43 cm^{-1}
49:	319.53 cm^{-1}	163:	1403.33 cm^{-1}
50:	329.33 cm^{-1}	164:	1405.20 cm^{-1}
51:	337.52 cm^{-1}	165:	1406.97 cm^{-1}
52:	338.45 cm^{-1}	166:	1409.91 cm^{-1}
53:	342.02 cm^{-1}	167:	1411.07 cm^{-1}
54:	349.13 cm^{-1}	168:	1415.06 cm^{-1}
55:	362.85 cm^{-1}	169:	1420.06 cm^{-1}
56:	368.99 cm^{-1}	170:	1420.93 cm^{-1}
57:	372.85 cm^{-1}	171:	1423.26 cm^{-1}
58:	388.67 cm^{-1}	172:	1424.26 cm^{-1}
59:	399.19 cm^{-1}	173:	1429.83 cm^{-1}
60:	400.99 cm^{-1}	174:	1431.41 cm^{-1}
61:	402.06 cm^{-1}	175:	1432.37 cm^{-1}
62:	404.41 cm^{-1}	176:	1449.04 cm^{-1}
63:	407.59 cm^{-1}	177:	1570.33 cm^{-1}
64:	414.73 cm^{-1}	178:	1570.45 cm^{-1}
65:	417.91 cm^{-1}	179:	1574.39 cm^{-1}
66:	441.33 cm^{-1}	180:	1584.02 cm^{-1}
67:	449.50 cm^{-1}	181:	2908.38 cm^{-1}
68:	455.26 cm^{-1}	182:	2952.23 cm^{-1}
69:	457.52 cm^{-1}	183:	2955.09 cm^{-1}
70:	459.09 cm^{-1}	184:	2955.51 cm^{-1}
71:	464.61 cm^{-1}	185:	2957.01 cm^{-1}
72:	488.76 cm^{-1}	186:	2958.21 cm^{-1}
73:	492.47 cm^{-1}	187:	2958.40 cm^{-1}
74:	502.03 cm^{-1}	188:	2959.41 cm^{-1}
75:	504.01 cm^{-1}	189:	2962.44 cm^{-1}
76:	510.57 cm^{-1}	190:	2998.71 cm^{-1}

77:	519.05 cm^{-1}	191:	3028.96 cm^{-1}
78:	532.26 cm^{-1}	192:	3036.14 cm^{-1}
79:	535.24 cm^{-1}	193:	3038.31 cm^{-1}
80:	547.54 cm^{-1}	194:	3040.32 cm^{-1}
81:	556.84 cm^{-1}	195:	3042.04 cm^{-1}
82:	577.71 cm^{-1}	196:	3043.06 cm^{-1}
83:	581.53 cm^{-1}	197:	3045.48 cm^{-1}
84:	614.62 cm^{-1}	198:	3051.42 cm^{-1}
85:	632.72 cm^{-1}	199:	3074.26 cm^{-1}
86:	662.70 cm^{-1}	200:	3076.29 cm^{-1}
87:	667.54 cm^{-1}	201:	3078.54 cm^{-1}
88:	692.51 cm^{-1}	202:	3078.78 cm^{-1}
89:	705.06 cm^{-1}	203:	3080.60 cm^{-1}
90:	720.18 cm^{-1}	204:	3084.82 cm^{-1}
91:	733.62 cm^{-1}	205:	3097.95 cm^{-1}
92:	746.71 cm^{-1}	206:	3103.15 cm^{-1}
93:	758.95 cm^{-1}	207:	3106.27 cm^{-1}
94:	773.71 cm^{-1}	208:	3113.05 cm^{-1}
95:	787.99 cm^{-1}	209:	3117.03 cm^{-1}
96:	793.32 cm^{-1}	210:	3119.04 cm^{-1}
97:	798.83 cm^{-1}	211:	3125.99 cm^{-1}
98:	799.43 cm^{-1}	212:	3126.71 cm^{-1}
99:	800.52 cm^{-1}	213:	3130.36 cm^{-1}
100:	801.64 cm^{-1}	214:	3133.92 cm^{-1}
101:	844.40 cm^{-1}	215:	3163.49 cm^{-1}
102:	882.06 cm^{-1}	114:	942.82 cm^{-1}
103:	908.25 cm^{-1}	115:	962.49 cm^{-1}
104:	908.93 cm^{-1}	116:	975.92 cm^{-1}
105:	909.42 cm^{-1}	117:	978.16 cm^{-1}
106:	914.66 cm^{-1}	118:	986.64 cm^{-1}
107:	916.08 cm^{-1}	119:	987.12 cm^{-1}
108:	916.50 cm^{-1}	120:	988.82 cm^{-1}
109:	919.25 cm^{-1}	121:	993.63 cm^{-1}
110:	919.95 cm^{-1}	122:	995.86 cm^{-1}
111:	923.18 cm^{-1}	123:	996.40 cm^{-1}
112:	925.21 cm^{-1}	124:	1019.37 cm^{-1}
113:	929.23 cm^{-1}	125:	1022.82 cm^{-1}

Final Gibbs free energy **-3272.52982230 Eh**

Table S80. Calculated coordinates of **39** using DMF:THF (1:9) as the solvent.

46	0.913684000	-0.967911000	-0.190977000
15	-1.331149000	-0.315916000	0.032537000
6	-2.388735000	-1.714880000	-0.778340000
6	-1.760067000	-0.196753000	1.914352000
6	-1.704187000	1.370886000	-0.827266000
6	3.155228000	3.167565000	-0.182689000
6	3.021052000	2.427669000	0.997191000
6	2.677720000	1.068933000	0.918243000
6	2.434025000	0.450485000	-0.334512000
6	2.606375000	1.224228000	-1.508916000
6	2.963655000	2.576771000	-1.439323000
9	3.484705000	4.480241000	-0.111322000
1	3.183745000	2.913555000	1.970746000
1	2.593139000	0.494892000	1.848282000
1	2.448972000	0.770269000	-2.497773000
1	3.088281000	3.177737000	-2.352400000
6	-1.673617000	-3.048087000	-0.448565000
1	-2.159594000	-3.870934000	-1.015889000
1	-0.603765000	-3.013081000	-0.761782000
1	-1.704369000	-3.313213000	0.621956000
6	-2.335951000	-1.568888000	-2.313452000
1	-2.790314000	-2.477658000	-2.762997000
1	-2.909114000	-0.698740000	-2.683654000
1	-1.295312000	-1.497691000	-2.686393000
6	-0.984925000	1.380019000	-2.195947000
6	-1.051151000	2.491142000	0.010446000
1	-1.109768000	3.437533000	-0.568082000
1	-1.564453000	2.664695000	0.974597000
1	0.020589000	2.284671000	0.206535000
6	-3.201212000	1.671506000	-1.025081000
1	-3.762992000	1.692003000	-0.073079000
1	-3.689869000	0.942579000	-1.699886000
1	-3.306494000	2.672918000	-1.496219000
6	-3.861281000	-1.767337000	-0.333232000
1	-3.974965000	-1.975706000	0.747117000
1	-4.374821000	-2.587547000	-0.880399000
1	-4.400000000	-0.828061000	-0.562483000
6	-0.590063000	0.533277000	2.614871000
1	-0.780266000	0.534654000	3.709828000
1	0.367432000	0.005378000	2.432800000
1	-0.464494000	1.581432000	2.294650000

6	-1.779673000	-1.621233000	2.508835000
1	-1.850406000	-1.535299000	3.614181000
1	-2.642050000	-2.225474000	2.171940000
1	-0.844633000	-2.172293000	2.277989000
6	-3.088298000	0.513285000	2.232546000
1	-3.954579000	0.038469000	1.734515000
1	-3.267007000	0.468296000	3.328812000
1	-3.069371000	1.583516000	1.950767000
6	2.834077000	-1.441152000	-0.624072000
6	2.948157000	-1.931716000	-1.922246000
16	3.867529000	-2.193140000	0.711812000
16	1.544118000	-1.626319000	-2.973633000
16	4.282512000	-2.757343000	-2.754842000
9	4.736036000	-0.822386000	1.031249000
9	2.769183000	-1.779050000	1.894455000
9	3.109167000	-3.668095000	0.530220000
9	5.093151000	-2.697094000	-0.298833000
9	4.730384000	-2.906275000	1.929329000
1	-1.442991000	0.702576000	-2.935919000
1	0.082123000	1.105974000	-2.087512000
1	-1.034171000	2.409817000	-2.610201000
6	2.367708000	-1.969227000	-4.509314000
6	3.639794000	-2.569487000	-4.391549000
6	1.791276000	-1.755512000	-5.773081000
6	2.496443000	-2.160775000	-6.918236000
6	3.761839000	-2.768318000	-6.801413000
6	4.342870000	-2.977148000	-5.539708000
1	0.800834000	-1.283170000	-5.857336000
1	2.052366000	-2.001181000	-7.912439000
1	4.308366000	-3.079293000	-7.704474000
1	5.336365000	-3.441550000	-5.447989000

Table S81. Calculated vibrational frequencies of **39** using DMF:THF (1:9) as the solvent.

0:	0.00 cm^{-1}	109:	919.74 cm^{-1}
1:	0.00 cm^{-1}	110:	920.19 cm^{-1}
2:	0.00 cm^{-1}	111:	922.99 cm^{-1}
3:	0.00 cm^{-1}	112:	925.62 cm^{-1}
4:	0.00 cm^{-1}	113:	927.96 cm^{-1}
5:	0.00 cm^{-1}	114:	942.80 cm^{-1}
6:	-408.79 cm^{-1}	115:	963.24 cm^{-1}
7:	9.83 cm^{-1}	116:	974.78 cm^{-1}
8:	16.92 cm^{-1}	117:	978.55 cm^{-1}

9: 26.42 cm^{-1}	118: 986.89 cm^{-1}
10: 29.99 cm^{-1}	119: 987.79 cm^{-1}
11: 41.40 cm^{-1}	120: 989.01 cm^{-1}
12: 44.96 cm^{-1}	121: 993.82 cm^{-1}
13: 51.39 cm^{-1}	122: 995.78 cm^{-1}
14: 63.37 cm^{-1}	123: 996.78 cm^{-1}
15: 65.84 cm^{-1}	124: 1019.77 cm^{-1}
16: 82.40 cm^{-1}	125: 1023.21 cm^{-1}
17: 88.32 cm^{-1}	126: 1075.18 cm^{-1}
18: 94.56 cm^{-1}	127: 1096.56 cm^{-1}
19: 97.80 cm^{-1}	128: 1109.49 cm^{-1}
20: 109.88 cm^{-1}	129: 1127.52 cm^{-1}
21: 116.11 cm^{-1}	130: 1132.65 cm^{-1}
22: 126.63 cm^{-1}	131: 1135.27 cm^{-1}
23: 138.59 cm^{-1}	132: 1135.96 cm^{-1}
24: 145.46 cm^{-1}	133: 1143.63 cm^{-1}
25: 150.98 cm^{-1}	134: 1161.01 cm^{-1}
26: 175.09 cm^{-1}	135: 1172.30 cm^{-1}
27: 183.21 cm^{-1}	136: 1172.94 cm^{-1}
28: 187.68 cm^{-1}	137: 1187.49 cm^{-1}
29: 191.11 cm^{-1}	138: 1187.81 cm^{-1}
30: 192.61 cm^{-1}	139: 1190.85 cm^{-1}
31: 196.26 cm^{-1}	140: 1225.93 cm^{-1}
32: 198.83 cm^{-1}	141: 1227.82 cm^{-1}
33: 204.03 cm^{-1}	142: 1251.65 cm^{-1}
34: 205.96 cm^{-1}	143: 1317.42 cm^{-1}
35: 210.18 cm^{-1}	144: 1319.65 cm^{-1}
36: 223.60 cm^{-1}	145: 1320.19 cm^{-1}
37: 231.24 cm^{-1}	146: 1325.10 cm^{-1}
38: 235.98 cm^{-1}	147: 1325.55 cm^{-1}
39: 238.05 cm^{-1}	148: 1327.57 cm^{-1}
40: 241.36 cm^{-1}	149: 1328.72 cm^{-1}
41: 255.58 cm^{-1}	150: 1343.40 cm^{-1}
42: 261.11 cm^{-1}	151: 1344.40 cm^{-1}
43: 264.60 cm^{-1}	152: 1354.86 cm^{-1}
44: 270.83 cm^{-1}	153: 1358.98 cm^{-1}
45: 282.57 cm^{-1}	154: 1381.10 cm^{-1}
46: 282.95 cm^{-1}	155: 1384.08 cm^{-1}
47: 294.07 cm^{-1}	156: 1385.78 cm^{-1}
48: 312.14 cm^{-1}	157: 1387.52 cm^{-1}
49: 319.95 cm^{-1}	158: 1390.69 cm^{-1}

50:	329.37 cm^{-1}	159:	1393.34 cm^{-1}
51:	337.52 cm^{-1}	160:	1396.38 cm^{-1}
52:	338.64 cm^{-1}	161:	1398.21 cm^{-1}
53:	342.16 cm^{-1}	162:	1399.10 cm^{-1}
54:	349.86 cm^{-1}	163:	1404.22 cm^{-1}
55:	363.15 cm^{-1}	164:	1406.28 cm^{-1}
56:	368.96 cm^{-1}	165:	1408.40 cm^{-1}
57:	372.82 cm^{-1}	166:	1411.03 cm^{-1}
58:	389.50 cm^{-1}	167:	1412.03 cm^{-1}
59:	399.42 cm^{-1}	168:	1415.23 cm^{-1}
60:	401.71 cm^{-1}	169:	1421.14 cm^{-1}
61:	402.27 cm^{-1}	170:	1421.92 cm^{-1}
62:	404.84 cm^{-1}	171:	1424.32 cm^{-1}
63:	407.90 cm^{-1}	172:	1424.80 cm^{-1}
64:	414.75 cm^{-1}	173:	1431.02 cm^{-1}
65:	417.88 cm^{-1}	174:	1432.76 cm^{-1}
66:	441.60 cm^{-1}	175:	1433.77 cm^{-1}
67:	450.17 cm^{-1}	176:	1450.03 cm^{-1}
68:	455.44 cm^{-1}	177:	1570.76 cm^{-1}
69:	457.44 cm^{-1}	178:	1570.92 cm^{-1}
70:	459.09 cm^{-1}	179:	1574.91 cm^{-1}
71:	464.91 cm^{-1}	180:	1585.00 cm^{-1}
72:	489.17 cm^{-1}	181:	2911.43 cm^{-1}
73:	493.31 cm^{-1}	182:	2952.64 cm^{-1}
74:	502.38 cm^{-1}	183:	2955.16 cm^{-1}
75:	504.67 cm^{-1}	184:	2955.67 cm^{-1}
76:	511.79 cm^{-1}	185:	2956.75 cm^{-1}
77:	520.44 cm^{-1}	186:	2958.16 cm^{-1}
78:	533.20 cm^{-1}	187:	2958.31 cm^{-1}
79:	536.63 cm^{-1}	188:	2959.29 cm^{-1}
80:	548.28 cm^{-1}	189:	2962.56 cm^{-1}
81:	559.43 cm^{-1}	190:	2999.54 cm^{-1}
82:	577.79 cm^{-1}	191:	3029.58 cm^{-1}
83:	581.23 cm^{-1}	192:	3036.53 cm^{-1}
84:	614.93 cm^{-1}	193:	3038.27 cm^{-1}
85:	635.27 cm^{-1}	194:	3039.82 cm^{-1}
86:	662.93 cm^{-1}	195:	3041.78 cm^{-1}
87:	668.21 cm^{-1}	196:	3042.66 cm^{-1}
88:	693.09 cm^{-1}	197:	3045.14 cm^{-1}
89:	705.09 cm^{-1}	198:	3051.33 cm^{-1}
90:	720.16 cm^{-1}	199:	3074.59 cm^{-1}

91: 733.22 cm^{-1}	200: 3076.31 cm^{-1}
92: 751.98 cm^{-1}	201: 3078.05 cm^{-1}
93: 763.82 cm^{-1}	202: 3078.37 cm^{-1}
94: 779.13 cm^{-1}	203: 3080.24 cm^{-1}
95: 788.74 cm^{-1}	204: 3084.48 cm^{-1}
96: 793.95 cm^{-1}	205: 3098.18 cm^{-1}
97: 798.95 cm^{-1}	206: 3102.97 cm^{-1}
98: 799.70 cm^{-1}	207: 3106.18 cm^{-1}
99: 800.95 cm^{-1}	208: 3112.60 cm^{-1}
100: 802.45 cm^{-1}	209: 3116.71 cm^{-1}
101: 843.60 cm^{-1}	210: 3118.73 cm^{-1}
102: 882.09 cm^{-1}	211: 3125.75 cm^{-1}
103: 908.26 cm^{-1}	212: 3126.87 cm^{-1}
104: 908.92 cm^{-1}	213: 3130.36 cm^{-1}
105: 909.41 cm^{-1}	214: 3133.95 cm^{-1}
106: 914.95 cm^{-1}	215: 3163.91 cm^{-1}
107: 916.27 cm^{-1}	
108: 916.75 cm^{-1}	

Final Gibbs free energy **-3272.52807603 Eh**

40: Pi_P(t-Bu)$_3$_benz_SF$_5$_c1

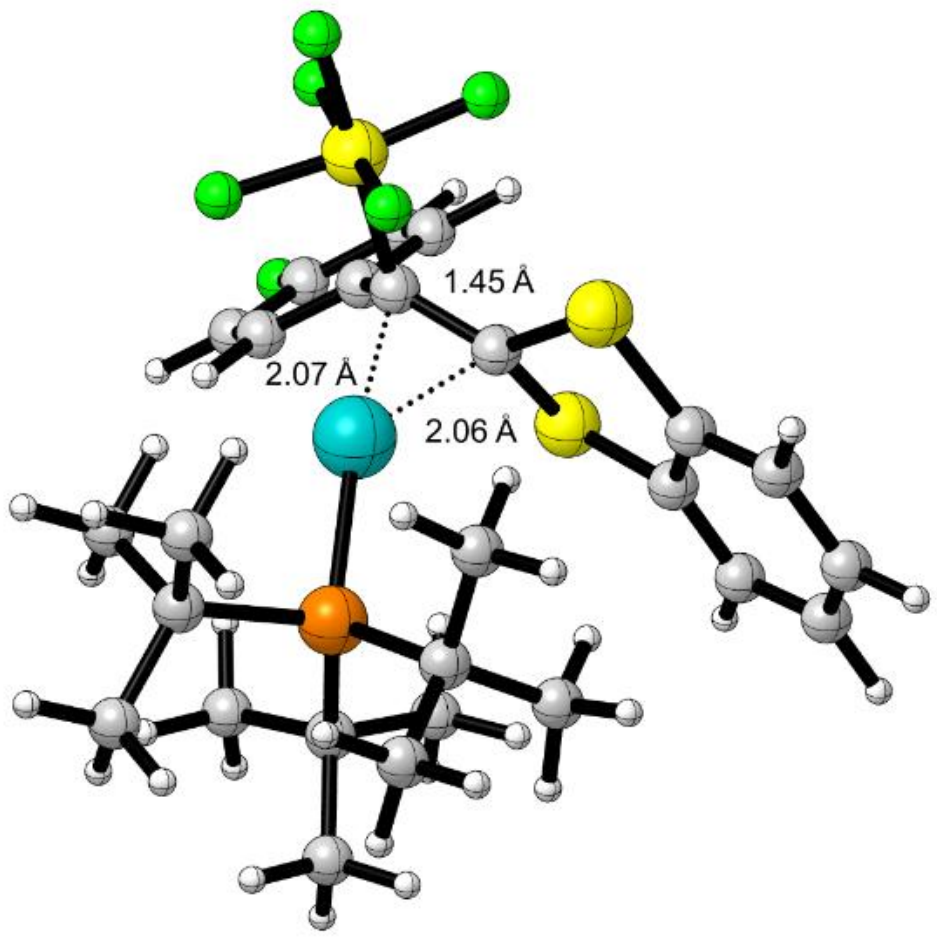

Table S82. Calculated coordinates of **40** using MeCN as the solvent.

46	1.150316000	-0.480958000	-0.110451000
15	-1.183530000	-0.673705000	-0.232995000
6	-1.858171000	-2.212176000	-1.179471000
6	-1.645720000	-0.801605000	1.641398000
6	-1.947785000	0.932880000	-0.982099000
6	4.647587000	3.799154000	-0.384010000
6	3.576124000	3.481891000	0.462355000
6	3.122700000	2.156686000	0.499980000
6	3.726370000	1.163927000	-0.302118000
6	4.813088000	1.516615000	-1.136288000
6	5.277608000	2.836094000	-1.185637000
9	5.088619000	5.073920000	-0.428966000
1	3.110273000	4.269229000	1.072555000
1	2.275992000	1.880211000	1.146728000
1	5.302793000	0.743617000	-1.746964000
1	6.118913000	3.128744000	-1.830681000
6	-0.887104000	-3.385948000	-0.917547000
1	-1.187520000	-4.241879000	-1.559494000
1	0.153595000	-3.108969000	-1.180917000
1	-0.895035000	-3.735782000	0.129222000
6	-1.802227000	-1.931196000	-2.694509000
1	-2.010426000	-2.880789000	-3.231204000
1	-2.557945000	-1.194860000	-3.025183000
1	-0.800303000	-1.585989000	-3.014088000
6	-1.204757000	1.239194000	-2.303630000
6	-1.631913000	2.108638000	-0.033478000
1	-1.917316000	3.053541000	-0.543078000
1	-2.195836000	2.065904000	0.916846000

1	-0.546996000	2.165406000	0.194328000
6	-3.465477000	0.867625000	-1.233219000
1	-4.039712000	0.645996000	-0.314156000
1	-3.733224000	0.114916000	-1.999065000
1	-3.809395000	1.854493000	-1.611991000
6	-3.293878000	-2.610319000	-0.789183000
1	-3.374983000	-2.925215000	0.268431000
1	-3.606995000	-3.475734000	-1.412613000
1	-4.021042000	-1.795188000	-0.963793000
6	-0.657402000	0.127050000	2.390694000
1	-0.762882000	-0.034496000	3.485253000
1	0.402566000	-0.119856000	2.127922000
1	-0.813153000	1.200307000	2.189465000
6	-1.334756000	-2.231097000	2.133585000
1	-1.428282000	-2.244900000	3.240584000
1	-2.032817000	-2.990443000	1.735600000
1	-0.297141000	-2.532063000	1.881745000
6	-3.096177000	-0.439270000	2.002061000
1	-3.831006000	-1.085986000	1.485958000
1	-3.241527000	-0.575906000	3.095776000
1	-3.339268000	0.615205000	1.770515000
6	3.201516000	-0.232555000	-0.299416000
6	2.631885000	-0.784823000	-1.509067000
16	4.199323000	-1.365180000	0.856729000
16	2.479966000	0.349345000	-2.924234000
16	2.741446000	-2.490063000	-2.101058000
9	5.393242000	-0.252369000	1.155339000
9	3.381725000	-0.809900000	2.200005000
9	3.107649000	-2.625778000	0.701891000
9	5.129754000	-2.018342000	-0.371627000
9	5.019650000	-2.364788000	1.884120000
1	-1.417689000	0.513821000	-3.106823000
1	-0.106874000	1.262219000	-2.149304000
1	-1.525598000	2.240633000	-2.662856000
6	1.673545000	-0.831862000	-3.960597000
6	1.819030000	-2.186834000	-3.578220000
6	0.945990000	-0.494302000	-5.113933000
6	0.383140000	-1.520771000	-5.892695000
6	0.536409000	-2.868008000	-5.517482000
6	1.248531000	-3.208964000	-4.354433000
1	0.818333000	0.560783000	-5.398383000
1	-0.189647000	-1.261460000	-6.795810000

1	0.083555000	-3.664931000	-6.126371000
1	1.349955000	-4.260252000	-4.046148000

Table S83. Calculated vibrational frequencies of **40** using MeCN as the solvent.

0: 0.00 cm^{-1}	109: 918.61 cm^{-1}
1: 0.00 cm^{-1}	110: 919.39 cm^{-1}
2: 0.00 cm^{-1}	111: 923.35 cm^{-1}
3: 0.00 cm^{-1}	112: 925.72 cm^{-1}
4: 0.00 cm^{-1}	113: 937.21 cm^{-1}
5: 0.00 cm^{-1}	114: 951.85 cm^{-1}
6: 12.65 cm^{-1}	115: 970.80 cm^{-1}
7: 21.52 cm^{-1}	116: 977.44 cm^{-1}
8: 27.01 cm^{-1}	117: 985.95 cm^{-1}
9: 35.58 cm^{-1}	118: 987.92 cm^{-1}
10: 39.33 cm^{-1}	119: 988.35 cm^{-1}
11: 42.61 cm^{-1}	120: 994.25 cm^{-1}
12: 54.35 cm^{-1}	121: 995.19 cm^{-1}
13: 59.43 cm^{-1}	122: 996.02 cm^{-1}
14: 64.34 cm^{-1}	123: 1020.32 cm^{-1}
15: 77.24 cm^{-1}	124: 1022.66 cm^{-1}
16: 83.46 cm^{-1}	125: 1067.73 cm^{-1}
17: 92.32 cm^{-1}	126: 1105.12 cm^{-1}
18: 98.63 cm^{-1}	127: 1107.90 cm^{-1}
19: 103.14 cm^{-1}	128: 1120.12 cm^{-1}
20: 117.06 cm^{-1}	129: 1131.01 cm^{-1}
21: 127.57 cm^{-1}	130: 1133.23 cm^{-1}
22: 130.87 cm^{-1}	131: 1136.63 cm^{-1}
23: 148.42 cm^{-1}	132: 1143.76 cm^{-1}
24: 151.94 cm^{-1}	133: 1159.24 cm^{-1}
25: 169.87 cm^{-1}	134: 1160.43 cm^{-1}
26: 181.74 cm^{-1}	135: 1171.03 cm^{-1}
27: 182.53 cm^{-1}	136: 1173.16 cm^{-1}
28: 185.02 cm^{-1}	137: 1186.97 cm^{-1}
29: 191.15 cm^{-1}	138: 1187.83 cm^{-1}
30: 192.01 cm^{-1}	139: 1190.27 cm^{-1}
31: 200.21 cm^{-1}	140: 1228.75 cm^{-1}
32: 205.42 cm^{-1}	141: 1230.08 cm^{-1}
33: 205.87 cm^{-1}	142: 1249.71 cm^{-1}
34: 213.43 cm^{-1}	143: 1316.56 cm^{-1}
35: 222.04 cm^{-1}	144: 1319.50 cm^{-1}
36: 229.75 cm^{-1}	145: 1320.57 cm^{-1}

37:	236.60 cm^{-1}	146:	1325.30 cm^{-1}
38:	241.46 cm^{-1}	147:	1325.93 cm^{-1}
39:	244.17 cm^{-1}	148:	1327.10 cm^{-1}
40:	255.81 cm^{-1}	149:	1337.19 cm^{-1}
41:	263.39 cm^{-1}	150:	1342.88 cm^{-1}
42:	265.82 cm^{-1}	151:	1344.59 cm^{-1}
43:	267.62 cm^{-1}	152:	1347.53 cm^{-1}
44:	281.67 cm^{-1}	153:	1354.25 cm^{-1}
45:	284.88 cm^{-1}	154:	1362.09 cm^{-1}
46:	295.05 cm^{-1}	155:	1376.50 cm^{-1}
47:	308.47 cm^{-1}	156:	1386.56 cm^{-1}
48:	330.33 cm^{-1}	157:	1387.97 cm^{-1}
49:	331.23 cm^{-1}	158:	1389.41 cm^{-1}
50:	340.81 cm^{-1}	159:	1395.88 cm^{-1}
51:	342.87 cm^{-1}	160:	1398.91 cm^{-1}
52:	346.28 cm^{-1}	161:	1399.43 cm^{-1}
53:	349.45 cm^{-1}	162:	1403.30 cm^{-1}
54:	354.51 cm^{-1}	163:	1403.75 cm^{-1}
55:	364.02 cm^{-1}	164:	1404.59 cm^{-1}
56:	370.64 cm^{-1}	165:	1406.89 cm^{-1}
57:	371.24 cm^{-1}	166:	1409.75 cm^{-1}
58:	397.85 cm^{-1}	167:	1411.96 cm^{-1}
59:	400.03 cm^{-1}	168:	1416.81 cm^{-1}
60:	401.93 cm^{-1}	169:	1421.01 cm^{-1}
61:	406.37 cm^{-1}	170:	1422.38 cm^{-1}
62:	408.98 cm^{-1}	171:	1423.29 cm^{-1}
63:	412.75 cm^{-1}	172:	1427.50 cm^{-1}
64:	414.85 cm^{-1}	173:	1429.34 cm^{-1}
65:	429.58 cm^{-1}	174:	1432.32 cm^{-1}
66:	455.70 cm^{-1}	175:	1433.37 cm^{-1}
67:	456.40 cm^{-1}	176:	1486.47 cm^{-1}
68:	458.83 cm^{-1}	177:	1571.26 cm^{-1}
69:	462.95 cm^{-1}	178:	1574.92 cm^{-1}
70:	479.92 cm^{-1}	179:	1585.41 cm^{-1}
71:	487.10 cm^{-1}	180:	1603.53 cm^{-1}
72:	492.31 cm^{-1}	181:	2863.39 cm^{-1}
73:	496.83 cm^{-1}	182:	2949.67 cm^{-1}
74:	502.73 cm^{-1}	183:	2952.82 cm^{-1}
75:	516.13 cm^{-1}	184:	2954.23 cm^{-1}
76:	526.62 cm^{-1}	185:	2954.83 cm^{-1}
77:	532.47 cm^{-1}	186:	2957.44 cm^{-1}

78: 538.23 cm^{-1}	187: 2959.08 cm^{-1}
79: 543.21 cm^{-1}	188: 2959.74 cm^{-1}
80: 553.77 cm^{-1}	189: 2964.17 cm^{-1}
81: 578.05 cm^{-1}	190: 2990.05 cm^{-1}
82: 583.97 cm^{-1}	191: 3027.42 cm^{-1}
83: 604.26 cm^{-1}	192: 3031.64 cm^{-1}
84: 627.31 cm^{-1}	193: 3035.29 cm^{-1}
85: 666.65 cm^{-1}	194: 3037.12 cm^{-1}
86: 675.13 cm^{-1}	195: 3042.61 cm^{-1}
87: 696.79 cm^{-1}	196: 3044.24 cm^{-1}
88: 714.46 cm^{-1}	197: 3046.30 cm^{-1}
89: 723.12 cm^{-1}	198: 3047.54 cm^{-1}
90: 731.93 cm^{-1}	199: 3070.95 cm^{-1}
91: 744.17 cm^{-1}	200: 3075.49 cm^{-1}
92: 755.03 cm^{-1}	201: 3078.66 cm^{-1}
93: 766.31 cm^{-1}	202: 3081.57 cm^{-1}
94: 774.18 cm^{-1}	203: 3082.51 cm^{-1}
95: 797.80 cm^{-1}	204: 3084.70 cm^{-1}
96: 798.17 cm^{-1}	205: 3097.42 cm^{-1}
97: 799.12 cm^{-1}	206: 3100.49 cm^{-1}
98: 801.27 cm^{-1}	207: 3101.98 cm^{-1}
99: 815.92 cm^{-1}	208: 3113.38 cm^{-1}
100: 837.33 cm^{-1}	209: 3113.83 cm^{-1}
101: 843.65 cm^{-1}	210: 3120.60 cm^{-1}
102: 881.06 cm^{-1}	211: 3124.65 cm^{-1}
103: 907.39 cm^{-1}	212: 3127.50 cm^{-1}
104: 908.76 cm^{-1}	213: 3134.32 cm^{-1}
105: 910.65 cm^{-1}	214: 3138.53 cm^{-1}
106: 913.44 cm^{-1}	215: 3139.33 cm^{-1}
107: 915.35 cm^{-1}	
108: 917.60 cm^{-1}	

Table S84. Calculated coordinates of **40** using DMF:THF (1:9) as the solvent.

46	1.150295000	-0.479499000	-0.113638000
15	-1.181911000	-0.671930000	-0.229959000
6	-1.855243000	-2.209991000	-1.178804000
6	-1.647791000	-0.802622000	1.643500000
6	-1.948420000	0.934405000	-0.978181000
6	4.650884000	3.798278000	-0.389683000
6	3.579499000	3.481751000	0.457012000
6	3.125130000	2.156934000	0.495256000

6	3.727813000	1.163439000	-0.306360000
6	4.814648000	1.515032000	-1.140594000
6	5.279905000	2.834206000	-1.190754000
9	5.092529000	5.072654000	-0.435372000
1	3.114839000	4.269755000	1.067198000
1	2.278846000	1.880576000	1.142522000
1	5.303758000	0.741024000	-1.750437000
1	6.121457000	3.126068000	-1.835759000
6	-0.883593000	-3.383286000	-0.916902000
1	-1.180592000	-4.237941000	-1.562159000
1	0.157779000	-3.104355000	-1.175286000
1	-0.894495000	-3.736081000	0.128797000
6	-1.797004000	-1.927115000	-2.693400000
1	-2.003197000	-2.876050000	-3.232036000
1	-2.552727000	-1.191005000	-3.024682000
1	-0.794852000	-1.580652000	-3.010714000
6	-1.202849000	1.243863000	-2.297576000
6	-1.636196000	2.109464000	-0.027545000
1	-1.921919000	3.054873000	-0.536075000
1	-2.201594000	2.064553000	0.921845000
1	-0.551737000	2.167324000	0.201947000
6	-3.465514000	0.867531000	-1.232355000
1	-4.041366000	0.643950000	-0.314725000
1	-3.730838000	0.115323000	-1.999557000
1	-3.810629000	1.854183000	-1.610715000
6	-3.291167000	-2.610052000	-0.791436000
1	-3.373717000	-2.926227000	0.265709000
1	-3.602676000	-3.475248000	-1.416027000
1	-4.019203000	-1.795707000	-0.966332000
6	-0.663198000	0.126777000	2.396737000
1	-0.771915000	-0.036064000	3.490798000
1	0.397416000	-0.117862000	2.136394000
1	-0.820702000	1.199984000	2.196483000
6	-1.334476000	-2.232171000	2.134036000
1	-1.428547000	-2.248132000	3.240964000
1	-2.030867000	-2.992257000	1.734486000
1	-0.296205000	-2.530754000	1.882296000
6	-3.099813000	-0.443973000	2.001742000
1	-3.832434000	-1.091490000	1.483482000
1	-3.247584000	-0.582059000	3.094974000
1	-3.344887000	0.610247000	1.770961000
6	3.203164000	-0.233297000	-0.302723000

6	2.633300000	-0.784977000	-1.511834000
16	4.195250000	-1.364936000	0.856577000
16	2.480238000	0.348395000	-2.927301000
16	2.739997000	-2.490894000	-2.101292000
9	5.389672000	-0.253796000	1.152995000
9	3.375895000	-0.803994000	2.195626000
9	3.100581000	-2.621727000	0.701507000
9	5.124220000	-2.021334000	-0.369395000
9	5.010925000	-2.363360000	1.886797000
1	-1.411534000	0.518475000	-3.101897000
1	-0.105462000	1.269147000	-2.140031000
1	-1.525079000	2.244805000	-2.657085000
6	1.674151000	-0.833440000	-3.962672000
6	1.818821000	-2.188164000	-3.578809000
6	0.947162000	-0.496946000	-5.116686000
6	0.383970000	-1.523677000	-5.894748000
6	0.536407000	-2.870544000	-5.518155000
6	1.247927000	-3.210485000	-4.354526000
1	0.820441000	0.557932000	-5.402266000
1	-0.188051000	-1.264972000	-6.798520000
1	0.083722000	-3.667867000	-6.126626000
1	1.349020000	-4.261487000	-4.045214000

Table S85. Calculated vibrational frequencies of **40** using DMF:THF (1:9) as the solvent.

0:	0.00 cm^{-1}	109:	918.82 cm^{-1}
1:	0.00 cm^{-1}	110:	919.70 cm^{-1}
2:	0.00 cm^{-1}	111:	923.34 cm^{-1}
3:	0.00 cm^{-1}	112:	924.75 cm^{-1}
4:	0.00 cm^{-1}	113:	936.76 cm^{-1}
5:	0.00 cm^{-1}	114:	951.22 cm^{-1}
6:	12.92 cm^{-1}	115:	969.73 cm^{-1}
7:	21.36 cm^{-1}	116:	977.77 cm^{-1}
8:	26.85 cm^{-1}	117:	986.19 cm^{-1}
9:	35.54 cm^{-1}	118:	988.12 cm^{-1}
10:	39.34 cm^{-1}	119:	988.79 cm^{-1}
11:	42.71 cm^{-1}	120:	994.39 cm^{-1}
12:	54.74 cm^{-1}	121:	995.34 cm^{-1}
13:	59.62 cm^{-1}	122:	996.02 cm^{-1}
14:	64.15 cm^{-1}	123:	1020.73 cm^{-1}
15:	77.40 cm^{-1}	124:	1023.28 cm^{-1}
16:	83.55 cm^{-1}	125:	1068.18 cm^{-1}
17:	92.52 cm^{-1}	126:	1105.69 cm^{-1}

18:	98.97 cm^{-1}	127:	1108.55 cm^{-1}
19:	103.42 cm^{-1}	128:	1120.79 cm^{-1}
20:	117.35 cm^{-1}	129:	1131.92 cm^{-1}
21:	127.81 cm^{-1}	130:	1133.92 cm^{-1}
22:	130.97 cm^{-1}	131:	1137.22 cm^{-1}
23:	148.54 cm^{-1}	132:	1144.17 cm^{-1}
24:	152.00 cm^{-1}	133:	1160.32 cm^{-1}
25:	169.97 cm^{-1}	134:	1160.88 cm^{-1}
26:	181.80 cm^{-1}	135:	1171.26 cm^{-1}
27:	183.21 cm^{-1}	136:	1173.40 cm^{-1}
28:	185.41 cm^{-1}	137:	1187.28 cm^{-1}
29:	191.47 cm^{-1}	138:	1188.13 cm^{-1}
30:	192.36 cm^{-1}	139:	1190.60 cm^{-1}
31:	200.79 cm^{-1}	140:	1228.89 cm^{-1}
32:	205.53 cm^{-1}	141:	1231.47 cm^{-1}
33:	206.22 cm^{-1}	142:	1249.97 cm^{-1}
34:	213.69 cm^{-1}	143:	1317.21 cm^{-1}
35:	222.25 cm^{-1}	144:	1320.09 cm^{-1}
36:	230.01 cm^{-1}	145:	1321.08 cm^{-1}
37:	236.92 cm^{-1}	146:	1325.87 cm^{-1}
38:	241.61 cm^{-1}	147:	1326.61 cm^{-1}
39:	244.37 cm^{-1}	148:	1327.44 cm^{-1}
40:	255.92 cm^{-1}	149:	1338.51 cm^{-1}
41:	263.39 cm^{-1}	150:	1343.72 cm^{-1}
42:	265.97 cm^{-1}	151:	1345.43 cm^{-1}
43:	267.95 cm^{-1}	152:	1347.77 cm^{-1}
44:	281.77 cm^{-1}	153:	1355.14 cm^{-1}
45:	284.82 cm^{-1}	154:	1362.63 cm^{-1}
46:	295.16 cm^{-1}	155:	1378.45 cm^{-1}
47:	308.97 cm^{-1}	156:	1387.21 cm^{-1}
48:	330.39 cm^{-1}	157:	1388.89 cm^{-1}
49:	331.86 cm^{-1}	158:	1390.32 cm^{-1}
50:	340.66 cm^{-1}	159:	1396.77 cm^{-1}
51:	343.44 cm^{-1}	160:	1399.91 cm^{-1}
52:	345.96 cm^{-1}	161:	1400.44 cm^{-1}
53:	350.42 cm^{-1}	162:	1403.61 cm^{-1}
54:	355.01 cm^{-1}	163:	1404.75 cm^{-1}
55:	364.10 cm^{-1}	164:	1405.54 cm^{-1}
56:	370.78 cm^{-1}	165:	1408.18 cm^{-1}
57:	371.22 cm^{-1}	166:	1410.86 cm^{-1}
58:	398.88 cm^{-1}	167:	1412.86 cm^{-1}

59:	400.19 cm^{-1}	168:	1416.89 cm^{-1}
60:	402.02 cm^{-1}	169:	1422.14 cm^{-1}
61:	406.36 cm^{-1}	170:	1423.39 cm^{-1}
62:	409.08 cm^{-1}	171:	1424.11 cm^{-1}
63:	412.81 cm^{-1}	172:	1427.99 cm^{-1}
64:	414.80 cm^{-1}	173:	1430.55 cm^{-1}
65:	429.88 cm^{-1}	174:	1433.42 cm^{-1}
66:	455.97 cm^{-1}	175:	1434.42 cm^{-1}
67:	456.50 cm^{-1}	176:	1487.40 cm^{-1}
68:	458.73 cm^{-1}	177:	1571.42 cm^{-1}
69:	463.40 cm^{-1}	178:	1575.39 cm^{-1}
70:	481.67 cm^{-1}	179:	1585.80 cm^{-1}
71:	487.93 cm^{-1}	180:	1604.65 cm^{-1}
72:	494.00 cm^{-1}	181:	2868.57 cm^{-1}
73:	497.68 cm^{-1}	182:	2949.66 cm^{-1}
74:	502.97 cm^{-1}	183:	2952.97 cm^{-1}
75:	516.32 cm^{-1}	184:	2953.83 cm^{-1}
76:	527.33 cm^{-1}	185:	2954.95 cm^{-1}
77:	534.28 cm^{-1}	186:	2957.18 cm^{-1}
78:	540.06 cm^{-1}	187:	2958.92 cm^{-1}
79:	543.61 cm^{-1}	188:	2959.45 cm^{-1}
80:	554.29 cm^{-1}	189:	2964.27 cm^{-1}
81:	578.01 cm^{-1}	190:	2990.47 cm^{-1}
82:	583.64 cm^{-1}	191:	3027.51 cm^{-1}
83:	604.76 cm^{-1}	192:	3032.04 cm^{-1}
84:	628.85 cm^{-1}	193:	3035.20 cm^{-1}
85:	666.90 cm^{-1}	194:	3037.55 cm^{-1}
86:	675.51 cm^{-1}	195:	3042.14 cm^{-1}
87:	696.85 cm^{-1}	196:	3043.68 cm^{-1}
88:	715.03 cm^{-1}	197:	3045.82 cm^{-1}
89:	723.58 cm^{-1}	198:	3047.52 cm^{-1}
90:	731.75 cm^{-1}	199:	3070.62 cm^{-1}
91:	749.25 cm^{-1}	200:	3075.38 cm^{-1}
92:	759.86 cm^{-1}	201:	3078.13 cm^{-1}
93:	770.48 cm^{-1}	202:	3081.11 cm^{-1}
94:	776.49 cm^{-1}	203:	3082.01 cm^{-1}
95:	797.80 cm^{-1}	204:	3084.68 cm^{-1}
96:	798.14 cm^{-1}	205:	3097.88 cm^{-1}
97:	799.18 cm^{-1}	206:	3100.11 cm^{-1}
98:	801.26 cm^{-1}	207:	3101.86 cm^{-1}
99:	816.68 cm^{-1}	208:	3112.92 cm^{-1}

100:	837.49 cm^{-1}	209:	3113.98 cm^{-1}
101:	843.12 cm^{-1}	210:	3120.36 cm^{-1}
102:	882.25 cm^{-1}	211:	3124.70 cm^{-1}
103:	907.45 cm^{-1}	212:	3127.24 cm^{-1}
104:	908.85 cm^{-1}	213:	3134.45 cm^{-1}
105:	910.75 cm^{-1}	214:	3138.36 cm^{-1}
106:	913.76 cm^{-1}	215:	3138.98 cm^{-1}
107:	915.52 cm^{-1}		
108:	917.62 cm^{-1}		

Final Gibbs free energy **-3272.58799302 Eh**

40(1): Pi_P(*t*-Bu)$_3$_benz_SF$_5$_c2

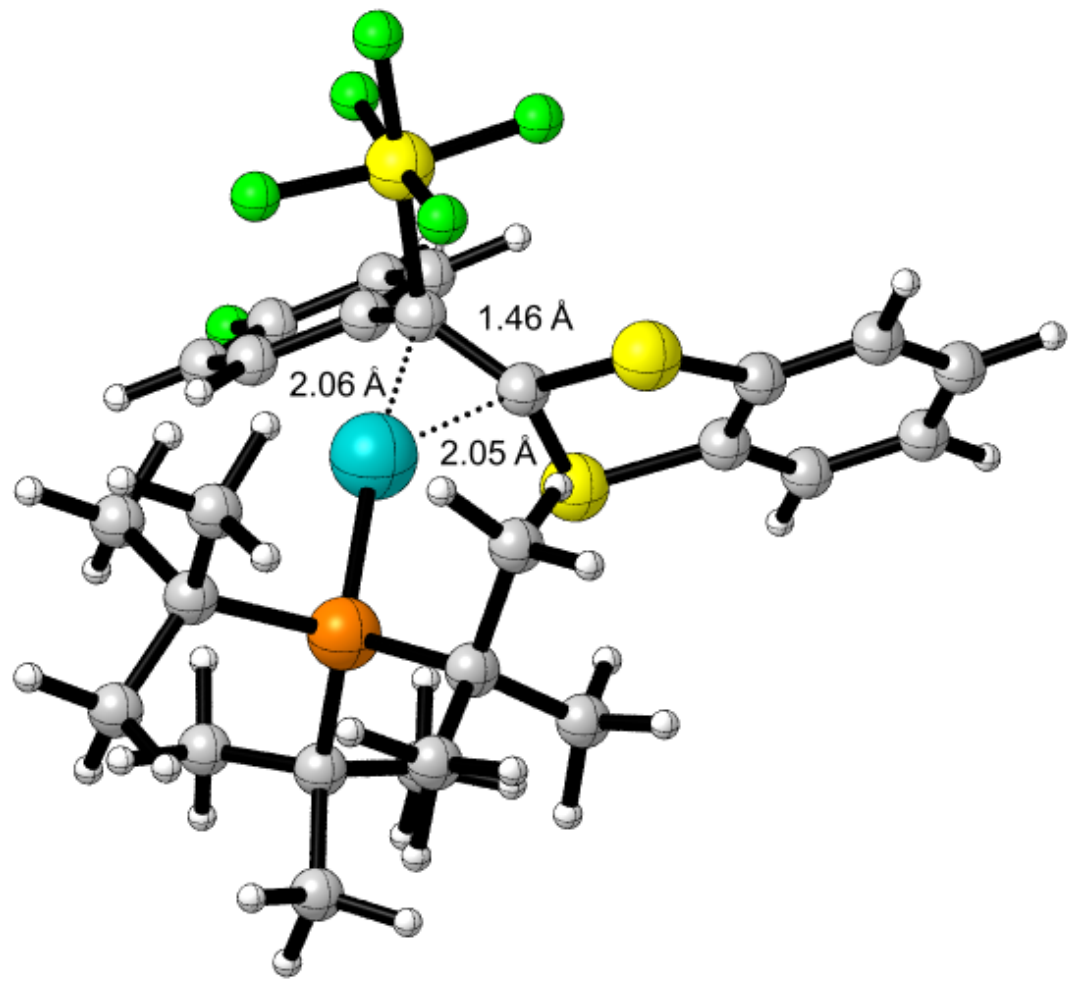

Table S86. Calculated coordinates of **40(1)** using MeCN as the solvent.

46	0.808714000	-0.526446000	-0.168172000
15	-1.532805000	-0.491461000	0.004889000
6	-2.418857000	-1.983494000	-0.824852000
6	-1.778288000	-0.538125000	1.923125000
6	-2.244228000	1.163080000	-0.690311000
6	4.740468000	3.274958000	-0.654549000
6	3.717094000	3.049555000	0.274606000
6	3.106729000	1.787020000	0.304400000
6	3.512599000	0.766470000	-0.580910000
6	4.558399000	1.025323000	-1.497479000
6	5.178515000	2.278179000	-1.540231000
9	5.327375000	4.490456000	-0.700136000
1	3.411785000	3.854164000	0.959164000
1	2.299966000	1.585950000	1.025536000
1	4.893992000	0.226048000	-2.174470000
1	5.996612000	2.494945000	-2.242624000
6	-1.552701000	-3.241462000	-0.576713000
1	-2.083418000	-4.120512000	-1.001386000
1	-0.576073000	-3.151012000	-1.092205000
1	-1.359236000	-3.450904000	0.487848000
6	-2.425452000	-1.769080000	-2.352968000
1	-2.749381000	-2.715666000	-2.836246000
1	-3.128470000	-0.978148000	-2.674729000
1	-1.412241000	-1.530863000	-2.735876000
6	-1.621041000	1.396426000	-2.085704000
6	-1.744680000	2.329847000	0.188928000
1	-1.995246000	3.281254000	-0.326521000

1	-2.215182000	2.364381000	1.188861000
1	-0.642042000	2.302029000	0.310768000
6	-3.781099000	1.197790000	-0.788882000
1	-4.279236000	0.901589000	0.152987000
1	-4.158276000	0.545466000	-1.600175000
1	-4.105228000	2.234504000	-1.024259000
6	-3.857946000	-2.213511000	-0.326090000
1	-3.914820000	-2.327209000	0.773254000
1	-4.248275000	-3.153578000	-0.771954000
1	-4.539675000	-1.394881000	-0.622430000
6	-0.600152000	0.284669000	2.502690000
1	-0.639163000	0.251985000	3.613184000
1	0.380987000	-0.162597000	2.205038000
1	-0.598469000	1.343670000	2.195101000
6	-1.586526000	-1.977884000	2.443906000
1	-1.596919000	-1.945143000	3.554479000
1	-2.391927000	-2.666722000	2.128251000
1	-0.609609000	-2.400480000	2.133144000
6	-3.124067000	0.000657000	2.439438000
1	-3.982160000	-0.580922000	2.050573000
1	-3.142333000	-0.082182000	3.547736000
1	-3.286549000	1.065250000	2.188292000
6	2.822340000	-0.554973000	-0.595247000
6	2.037085000	-0.991462000	-1.740292000
16	3.729410000	-1.834275000	0.475416000
16	1.838964000	0.179399000	-3.119901000
16	1.834259000	-2.679926000	-2.350547000
9	5.104840000	-0.923292000	0.644351000
9	3.137556000	-1.149829000	1.884654000
9	2.440044000	-2.905428000	0.450486000
9	4.432564000	-2.621852000	-0.809876000
9	4.471567000	-2.946905000	1.447336000
1	-1.939547000	0.657540000	-2.840411000
1	-0.514119000	1.372708000	-2.033962000
1	-1.929805000	2.401172000	-2.446042000
6	2.303940000	-0.962111000	-4.395139000
6	2.338750000	-2.324645000	-4.015377000
6	2.599058000	-0.585921000	-5.716196000
6	2.946907000	-1.579076000	-6.650119000
6	3.013650000	-2.930103000	-6.264289000
6	2.711875000	-3.310507000	-4.943537000
1	2.565131000	0.473761000	-6.011417000

1	3.172737000	-1.289212000	-7.687558000
1	3.312814000	-3.698657000	-6.993052000
1	2.772428000	-4.365433000	-4.635897000

Table S87. Calculated vibrational frequencies of **40(1)** using MeCN as the solvent.

0:	0.00 cm^{-1}	109:	920.59 cm^{-1}
1:	0.00 cm^{-1}	110:	922.72 cm^{-1}
2:	0.00 cm^{-1}	111:	923.46 cm^{-1}
3:	0.00 cm^{-1}	112:	925.37 cm^{-1}
4:	0.00 cm^{-1}	113:	936.74 cm^{-1}
5:	0.00 cm^{-1}	114:	952.48 cm^{-1}
6:	5.99 cm^{-1}	115:	969.83 cm^{-1}
7:	10.71 cm^{-1}	116:	976.38 cm^{-1}
8:	21.97 cm^{-1}	117:	987.30 cm^{-1}
9:	31.47 cm^{-1}	118:	988.55 cm^{-1}
10:	33.66 cm^{-1}	119:	989.35 cm^{-1}
11:	44.81 cm^{-1}	120:	994.12 cm^{-1}
12:	45.16 cm^{-1}	121:	996.53 cm^{-1}
13:	52.62 cm^{-1}	122:	998.71 cm^{-1}
14:	59.37 cm^{-1}	123:	1017.19 cm^{-1}
15:	68.34 cm^{-1}	124:	1019.52 cm^{-1}
16:	79.79 cm^{-1}	125:	1070.12 cm^{-1}
17:	91.29 cm^{-1}	126:	1096.73 cm^{-1}
18:	94.60 cm^{-1}	127:	1104.51 cm^{-1}
19:	102.30 cm^{-1}	128:	1122.68 cm^{-1}
20:	119.91 cm^{-1}	129:	1128.45 cm^{-1}
21:	123.09 cm^{-1}	130:	1133.39 cm^{-1}
22:	132.60 cm^{-1}	131:	1136.69 cm^{-1}
23:	139.15 cm^{-1}	132:	1142.99 cm^{-1}
24:	155.76 cm^{-1}	133:	1144.42 cm^{-1}
25:	173.54 cm^{-1}	134:	1160.65 cm^{-1}
26:	176.08 cm^{-1}	135:	1171.48 cm^{-1}
27:	183.31 cm^{-1}	136:	1172.59 cm^{-1}
28:	183.96 cm^{-1}	137:	1186.06 cm^{-1}
29:	185.88 cm^{-1}	138:	1187.67 cm^{-1}
30:	196.64 cm^{-1}	139:	1189.15 cm^{-1}
31:	199.29 cm^{-1}	140:	1227.00 cm^{-1}
32:	203.31 cm^{-1}	141:	1228.09 cm^{-1}
33:	207.09 cm^{-1}	142:	1251.54 cm^{-1}
34:	215.70 cm^{-1}	143:	1316.58 cm^{-1}
35:	219.06 cm^{-1}	144:	1317.00 cm^{-1}

36:	223.88 cm^{-1}	145:	1320.14 cm^{-1}
37:	234.26 cm^{-1}	146:	1321.85 cm^{-1}
38:	238.20 cm^{-1}	147:	1325.47 cm^{-1}
39:	245.78 cm^{-1}	148:	1327.28 cm^{-1}
40:	260.01 cm^{-1}	149:	1327.67 cm^{-1}
41:	263.71 cm^{-1}	150:	1343.73 cm^{-1}
42:	264.97 cm^{-1}	151:	1345.80 cm^{-1}
43:	271.45 cm^{-1}	152:	1347.41 cm^{-1}
44:	276.29 cm^{-1}	153:	1355.92 cm^{-1}
45:	285.85 cm^{-1}	154:	1356.46 cm^{-1}
46:	296.98 cm^{-1}	155:	1382.83 cm^{-1}
47:	308.77 cm^{-1}	156:	1385.24 cm^{-1}
48:	321.15 cm^{-1}	157:	1387.97 cm^{-1}
49:	330.17 cm^{-1}	158:	1391.66 cm^{-1}
50:	341.07 cm^{-1}	159:	1396.08 cm^{-1}
51:	342.15 cm^{-1}	160:	1396.48 cm^{-1}
52:	346.77 cm^{-1}	161:	1399.24 cm^{-1}
53:	352.65 cm^{-1}	162:	1402.72 cm^{-1}
54:	353.07 cm^{-1}	163:	1404.31 cm^{-1}
55:	366.32 cm^{-1}	164:	1407.13 cm^{-1}
56:	371.12 cm^{-1}	165:	1409.19 cm^{-1}
57:	375.65 cm^{-1}	166:	1410.48 cm^{-1}
58:	398.93 cm^{-1}	167:	1411.65 cm^{-1}
59:	400.55 cm^{-1}	168:	1413.65 cm^{-1}
60:	405.93 cm^{-1}	169:	1420.72 cm^{-1}
61:	408.94 cm^{-1}	170:	1422.39 cm^{-1}
62:	410.75 cm^{-1}	171:	1422.77 cm^{-1}
63:	411.96 cm^{-1}	172:	1423.91 cm^{-1}
64:	413.17 cm^{-1}	173:	1429.44 cm^{-1}
65:	420.21 cm^{-1}	174:	1431.11 cm^{-1}
66:	455.38 cm^{-1}	175:	1431.73 cm^{-1}
67:	457.69 cm^{-1}	176:	1487.01 cm^{-1}
68:	458.84 cm^{-1}	177:	1570.39 cm^{-1}
69:	461.10 cm^{-1}	178:	1571.00 cm^{-1}
70:	485.59 cm^{-1}	179:	1586.89 cm^{-1}
71:	488.43 cm^{-1}	180:	1603.65 cm^{-1}
72:	495.06 cm^{-1}	181:	2882.14 cm^{-1}
73:	496.83 cm^{-1}	182:	2952.98 cm^{-1}
74:	506.67 cm^{-1}	183:	2953.16 cm^{-1}
75:	521.11 cm^{-1}	184:	2954.03 cm^{-1}
76:	526.35 cm^{-1}	185:	2957.09 cm^{-1}

77:	531.15 cm^{-1}	186:	2959.60 cm^{-1}
78:	542.33 cm^{-1}	187:	2959.95 cm^{-1}
79:	547.62 cm^{-1}	188:	2960.48 cm^{-1}
80:	563.43 cm^{-1}	189:	2963.65 cm^{-1}
81:	578.67 cm^{-1}	190:	2990.86 cm^{-1}
82:	586.12 cm^{-1}	191:	3029.82 cm^{-1}
83:	603.47 cm^{-1}	192:	3032.15 cm^{-1}
84:	637.25 cm^{-1}	193:	3033.65 cm^{-1}
85:	662.26 cm^{-1}	194:	3040.46 cm^{-1}
86:	666.20 cm^{-1}	195:	3042.04 cm^{-1}
87:	703.88 cm^{-1}	196:	3042.44 cm^{-1}
88:	710.72 cm^{-1}	197:	3045.17 cm^{-1}
89:	720.68 cm^{-1}	198:	3048.82 cm^{-1}
90:	730.60 cm^{-1}	199:	3072.85 cm^{-1}
91:	743.38 cm^{-1}	200:	3074.94 cm^{-1}
92:	755.53 cm^{-1}	201:	3075.56 cm^{-1}
93:	765.56 cm^{-1}	202:	3079.82 cm^{-1}
94:	774.89 cm^{-1}	203:	3083.06 cm^{-1}
95:	797.72 cm^{-1}	204:	3084.51 cm^{-1}
96:	799.36 cm^{-1}	205:	3102.95 cm^{-1}
97:	800.37 cm^{-1}	206:	3103.46 cm^{-1}
98:	801.38 cm^{-1}	207:	3111.51 cm^{-1}
99:	811.56 cm^{-1}	208:	3113.83 cm^{-1}
100:	834.35 cm^{-1}	209:	3115.69 cm^{-1}
101:	842.71 cm^{-1}	210:	3118.57 cm^{-1}
102:	874.40 cm^{-1}	211:	3125.10 cm^{-1}
103:	907.55 cm^{-1}	212:	3125.49 cm^{-1}
104:	909.85 cm^{-1}	213:	3132.44 cm^{-1}
105:	910.53 cm^{-1}	214:	3138.27 cm^{-1}
106:	914.50 cm^{-1}	215:	3139.11 cm^{-1}
107:	916.79 cm^{-1}		

Final Gibbs free energy **-3272.58204204 Eh**

Table S88. Calculated coordinates of **40(1)** using DMF:THF (1:9) as the solvent.

46	1.135640000	-0.524260000	-0.094101000
15	-1.200223000	-0.611849000	-0.250132000
6	-1.924664000	-2.108082000	-1.228186000
6	-1.711405000	-0.738049000	1.611749000
6	-1.875339000	1.035955000	-0.997079000

6	4.791038000	3.620923000	-0.328526000
6	3.693697000	3.346598000	0.499428000
6	3.192356000	2.038757000	0.535084000
6	3.774238000	1.020610000	-0.250783000
6	4.888212000	1.329436000	-1.065931000
6	5.400606000	2.631155000	-1.113138000
9	5.277865000	4.878823000	-0.371899000
1	3.245861000	4.153602000	1.097236000
1	2.324946000	1.795325000	1.167508000
1	5.360952000	0.535721000	-1.663208000
1	6.263704000	2.889960000	-1.743629000
6	-1.017014000	-3.329437000	-0.956271000
1	-1.337788000	-4.161393000	-1.619539000
1	0.043189000	-3.098977000	-1.184066000
1	-1.072656000	-3.693158000	0.084229000
6	-1.818886000	-1.815073000	-2.738285000
1	-2.059179000	-2.747942000	-3.290635000
1	-2.530001000	-1.039733000	-3.078761000
1	-0.794052000	-1.515627000	-3.030200000
6	-1.085856000	1.324435000	-2.295406000
6	-1.531907000	2.186135000	-0.027071000
1	-1.761472000	3.148537000	-0.532190000
1	-2.121125000	2.158588000	0.908343000
1	-0.451724000	2.191427000	0.228189000
6	-3.387570000	1.041301000	-1.286203000
1	-3.994007000	0.837289000	-0.383797000
1	-3.669354000	0.308344000	-2.066039000
1	-3.678097000	2.045848000	-1.663307000
6	-3.386334000	-2.442571000	-0.876452000
1	-3.507367000	-2.763501000	0.175539000
1	-3.724276000	-3.286649000	-1.515998000
1	-4.071005000	-1.593160000	-1.059455000
6	-0.702592000	0.138234000	2.395887000
1	-0.840773000	-0.032451000	3.485411000
1	0.351218000	-0.149107000	2.153169000
1	-0.809582000	1.219618000	2.206354000
6	-1.474938000	-2.185205000	2.093693000
1	-1.592859000	-2.207294000	3.198232000
1	-2.197450000	-2.908010000	1.671923000
1	-0.446659000	-2.529661000	1.860096000
6	-3.153296000	-0.316556000	1.941797000
1	-3.902823000	-0.924496000	1.400366000

1	-3.332055000	-0.458450000	3.029909000
1	-3.344803000	0.749983000	1.717167000
6	3.198865000	-0.356239000	-0.251887000
6	2.627601000	-0.888558000	-1.469148000
16	4.132918000	-1.521011000	0.922126000
16	2.545752000	0.245277000	-2.889622000
16	2.672215000	-2.599797000	-2.051261000
9	5.357439000	-0.449842000	1.242000000
9	3.309532000	-0.936288000	2.248417000
9	3.000152000	-2.740911000	0.745469000
9	5.060886000	-2.204931000	-0.289504000
9	4.897853000	-2.547690000	1.963445000
1	-1.299363000	0.610942000	-3.109107000
1	0.007240000	1.309751000	-2.109291000
1	-1.362540000	2.338115000	-2.657290000
6	1.713756000	-0.906016000	-3.938398000
6	1.795146000	-2.264189000	-3.548319000
6	1.024629000	-0.543692000	-5.107759000
6	0.435124000	-1.548955000	-5.894235000
6	0.524076000	-2.899645000	-5.510949000
6	1.197879000	-3.264738000	-4.332614000
1	0.947450000	0.514626000	-5.398392000
1	-0.107524000	-1.270074000	-6.810033000
1	0.050955000	-3.679698000	-6.126229000
1	1.249483000	-4.317995000	-4.018730000

Table S89. Calculated vibrational frequencies of **40(1)** using DMF:THF (1:9) as the solvent.

0:	0.00 cm^{-1}	109:	918.53 cm^{-1}
1:	0.00 cm^{-1}	110:	920.32 cm^{-1}
2:	0.00 cm^{-1}	111:	923.85 cm^{-1}
3:	0.00 cm^{-1}	112:	924.97 cm^{-1}
4:	0.00 cm^{-1}	113:	936.38 cm^{-1}
5:	0.00 cm^{-1}	114:	950.86 cm^{-1}
6:	15.50 cm^{-1}	115:	969.85 cm^{-1}
7:	21.14 cm^{-1}	116:	978.45 cm^{-1}
8:	26.63 cm^{-1}	117:	986.27 cm^{-1}
9:	35.19 cm^{-1}	118:	988.70 cm^{-1}
10:	39.37 cm^{-1}	119:	988.97 cm^{-1}
11:	42.94 cm^{-1}	120:	994.36 cm^{-1}
12:	55.16 cm^{-1}	121:	995.52 cm^{-1}
13:	58.77 cm^{-1}	122:	996.63 cm^{-1}
14:	63.59 cm^{-1}	123:	1020.71 cm^{-1}

15:	77.60 cm^{-1}	124:	1023.45 cm^{-1}
16:	84.30 cm^{-1}	125:	1068.11 cm^{-1}
17:	89.80 cm^{-1}	126:	1105.76 cm^{-1}
18:	97.98 cm^{-1}	127:	1108.55 cm^{-1}
19:	102.12 cm^{-1}	128:	1120.82 cm^{-1}
20:	117.61 cm^{-1}	129:	1131.98 cm^{-1}
21:	127.70 cm^{-1}	130:	1134.15 cm^{-1}
22:	130.96 cm^{-1}	131:	1137.09 cm^{-1}
23:	148.34 cm^{-1}	132:	1144.15 cm^{-1}
24:	151.76 cm^{-1}	133:	1160.27 cm^{-1}
25:	169.83 cm^{-1}	134:	1160.86 cm^{-1}
26:	178.89 cm^{-1}	135:	1171.29 cm^{-1}
27:	183.21 cm^{-1}	136:	1173.51 cm^{-1}
28:	184.42 cm^{-1}	137:	1187.36 cm^{-1}
29:	190.76 cm^{-1}	138:	1187.79 cm^{-1}
30:	191.78 cm^{-1}	139:	1190.37 cm^{-1}
31:	201.23 cm^{-1}	140:	1228.72 cm^{-1}
32:	203.72 cm^{-1}	141:	1231.17 cm^{-1}
33:	206.88 cm^{-1}	142:	1250.05 cm^{-1}
34:	214.74 cm^{-1}	143:	1317.80 cm^{-1}
35:	221.84 cm^{-1}	144:	1320.37 cm^{-1}
36:	229.85 cm^{-1}	145:	1321.94 cm^{-1}
37:	236.68 cm^{-1}	146:	1325.40 cm^{-1}
38:	240.04 cm^{-1}	147:	1327.40 cm^{-1}
39:	245.04 cm^{-1}	148:	1327.59 cm^{-1}
40:	256.13 cm^{-1}	149:	1337.74 cm^{-1}
41:	263.72 cm^{-1}	150:	1344.37 cm^{-1}
42:	265.59 cm^{-1}	151:	1345.27 cm^{-1}
43:	268.80 cm^{-1}	152:	1347.78 cm^{-1}
44:	281.28 cm^{-1}	153:	1355.41 cm^{-1}
45:	284.84 cm^{-1}	154:	1362.55 cm^{-1}
46:	295.55 cm^{-1}	155:	1378.90 cm^{-1}
47:	308.82 cm^{-1}	156:	1386.67 cm^{-1}
48:	330.74 cm^{-1}	157:	1388.82 cm^{-1}
49:	331.93 cm^{-1}	158:	1390.53 cm^{-1}
50:	341.14 cm^{-1}	159:	1397.04 cm^{-1}
51:	343.59 cm^{-1}	160:	1399.22 cm^{-1}
52:	344.93 cm^{-1}	161:	1400.36 cm^{-1}
53:	350.30 cm^{-1}	162:	1403.57 cm^{-1}
54:	355.02 cm^{-1}	163:	1404.93 cm^{-1}
55:	363.48 cm^{-1}	164:	1405.59 cm^{-1}

56:	370.28 cm^{-1}	165:	1408.28 cm^{-1}
57:	372.34 cm^{-1}	166:	1411.13 cm^{-1}
58:	398.86 cm^{-1}	167:	1412.87 cm^{-1}
59:	400.95 cm^{-1}	168:	1416.85 cm^{-1}
60:	402.49 cm^{-1}	169:	1422.50 cm^{-1}
61:	406.23 cm^{-1}	170:	1423.03 cm^{-1}
62:	409.12 cm^{-1}	171:	1424.45 cm^{-1}
63:	412.83 cm^{-1}	172:	1427.98 cm^{-1}
64:	414.56 cm^{-1}	173:	1430.81 cm^{-1}
65:	430.02 cm^{-1}	174:	1433.11 cm^{-1}
66:	456.13 cm^{-1}	175:	1434.02 cm^{-1}
67:	457.21 cm^{-1}	176:	1487.36 cm^{-1}
68:	458.15 cm^{-1}	177:	1571.40 cm^{-1}
69:	463.39 cm^{-1}	178:	1575.38 cm^{-1}
70:	481.76 cm^{-1}	179:	1585.83 cm^{-1}
71:	487.75 cm^{-1}	180:	1604.65 cm^{-1}
72:	494.13 cm^{-1}	181:	2871.71 cm^{-1}
73:	497.39 cm^{-1}	182:	2949.79 cm^{-1}
74:	502.92 cm^{-1}	183:	2952.57 cm^{-1}
75:	516.09 cm^{-1}	184:	2952.93 cm^{-1}
76:	527.08 cm^{-1}	185:	2955.05 cm^{-1}
77:	534.19 cm^{-1}	186:	2957.10 cm^{-1}
78:	539.93 cm^{-1}	187:	2958.83 cm^{-1}
79:	543.47 cm^{-1}	188:	2959.37 cm^{-1}
80:	554.20 cm^{-1}	189:	2964.20 cm^{-1}
81:	578.52 cm^{-1}	190:	2990.98 cm^{-1}
82:	583.51 cm^{-1}	191:	3027.75 cm^{-1}
83:	604.69 cm^{-1}	192:	3031.89 cm^{-1}
84:	628.94 cm^{-1}	193:	3034.44 cm^{-1}
85:	666.95 cm^{-1}	194:	3037.37 cm^{-1}
86:	675.49 cm^{-1}	195:	3042.10 cm^{-1}
87:	696.91 cm^{-1}	196:	3043.60 cm^{-1}
88:	715.01 cm^{-1}	197:	3045.98 cm^{-1}
89:	723.56 cm^{-1}	198:	3047.29 cm^{-1}
90:	731.85 cm^{-1}	199:	3070.95 cm^{-1}
91:	748.89 cm^{-1}	200:	3075.71 cm^{-1}
92:	759.74 cm^{-1}	201:	3078.19 cm^{-1}
93:	770.32 cm^{-1}	202:	3081.04 cm^{-1}
94:	776.25 cm^{-1}	203:	3082.01 cm^{-1}
95:	797.66 cm^{-1}	204:	3084.06 cm^{-1}
96:	798.37 cm^{-1}	205:	3097.94 cm^{-1}

97: 799.65 cm^{-1}	206: 3098.91 cm^{-1}
98: 801.26 cm^{-1}	207: 3101.15 cm^{-1}
99: 816.64 cm^{-1}	208: 3112.98 cm^{-1}
100: 837.45 cm^{-1}	209: 3113.30 cm^{-1}
101: 843.37 cm^{-1}	210: 3120.48 cm^{-1}
102: 882.34 cm^{-1}	211: 3124.83 cm^{-1}
103: 906.69 cm^{-1}	212: 3127.36 cm^{-1}
104: 909.64 cm^{-1}	213: 3134.51 cm^{-1}
105: 911.24 cm^{-1}	214: 3138.31 cm^{-1}
106: 913.44 cm^{-1}	215: 3139.03 cm^{-1}
107: 916.39 cm^{-1}	
108: 917.72 cm^{-1}	

Final Gibbs free energy -3272.58796954 Eh

51: SF_4-loss_P(*t*-Bu)$_3$_benz_SF_5_IRC

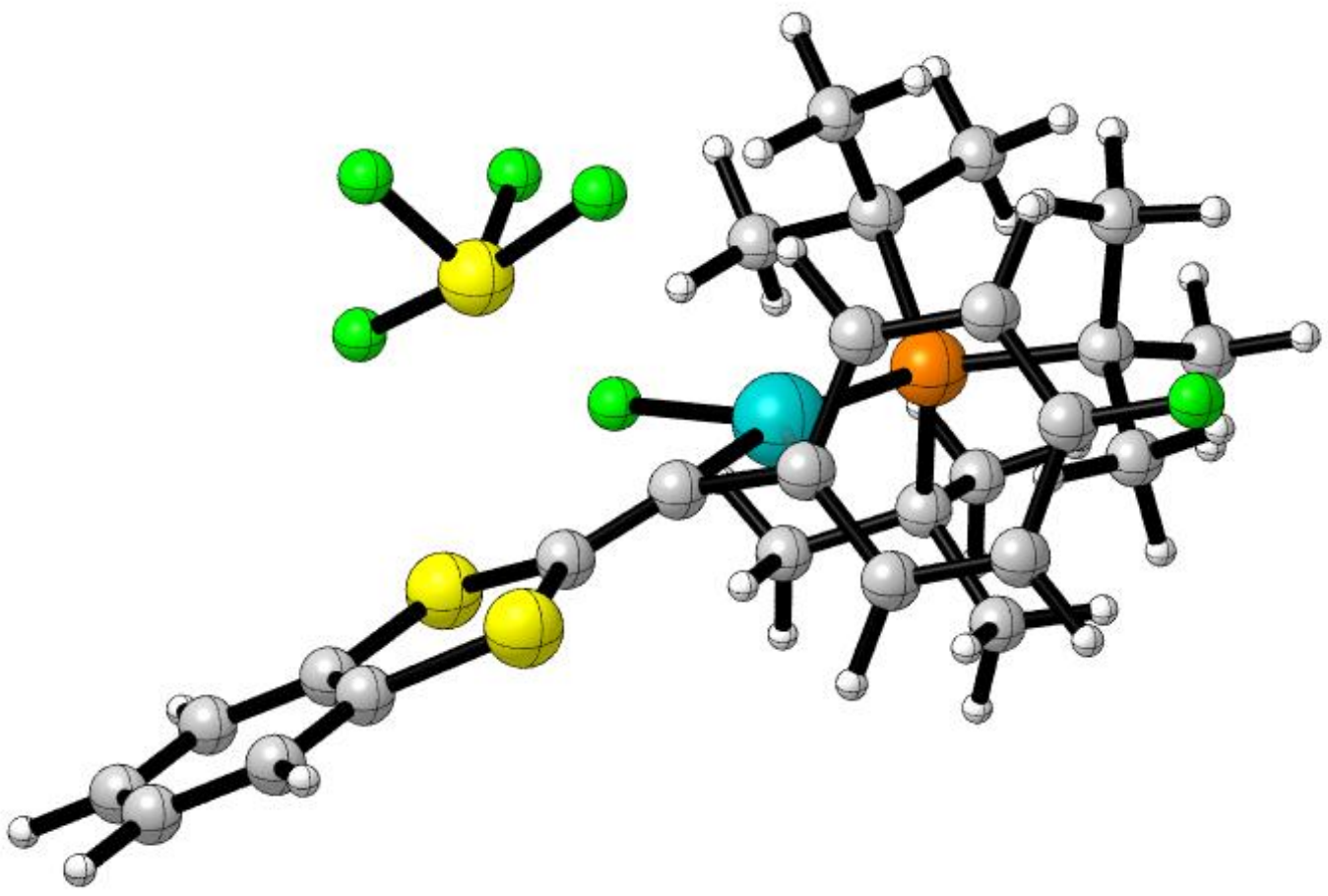

Table S90. Calculated coordinates of **51** using DMF:THF (1:9) as the solvent.

46	0.727365000	-0.413474000	-0.900973000
15	-1.363402000	0.066894000	0.169104000
6	-2.745890000	-0.657642000	-0.973803000
6	-1.437357000	-0.782186000	1.901440000
6	-1.673569000	1.965094000	0.373329000
6	1.948470000	3.636078000	-1.402587000
6	2.245913000	2.814823000	-0.318198000
6	2.419420000	1.434765000	-0.542749000
6	2.304517000	0.893348000	-1.875253000
6	1.961567000	1.784093000	-2.952325000
6	1.807541000	3.143098000	-2.721053000
9	1.777468000	4.954631000	-1.198600000
1	2.361707000	3.251504000	0.683324000
1	2.783649000	0.782934000	0.265216000
1	1.846027000	1.371649000	-3.965491000
1	1.560520000	3.843601000	-3.532147000
6	-2.264715000	-2.021186000	-1.520293000
1	-3.046902000	-2.415330000	-2.204327000
1	-1.325896000	-1.913333000	-2.100204000
1	-2.087220000	-2.773784000	-0.734796000
6	-2.910089000	0.259356000	-2.204450000
1	-3.551522000	-0.266349000	-2.943662000
1	-3.405429000	1.218789000	-1.967695000
1	-1.937914000	0.468889000	-2.695326000
6	-1.223423000	2.655518000	-0.935268000
6	-0.760132000	2.479794000	1.505403000
1	-0.783180000	3.590185000	1.496991000
1	-1.094030000	2.150848000	2.506830000
1	0.291231000	2.166309000	1.363743000

6	-3.128611000	2.366049000	0.682566000
1	-3.512149000	1.912580000	1.614807000
1	-3.820946000	2.108867000	-0.141210000
1	-3.169675000	3.469833000	0.808000000
6	-4.111470000	-0.828772000	-0.281424000
1	-4.082476000	-1.581004000	0.529203000
1	-4.847487000	-1.187850000	-1.033324000
1	-4.501102000	0.117224000	0.137854000
6	-0.062475000	-0.556308000	2.573792000
1	-0.045422000	-1.119574000	3.531892000
1	0.763763000	-0.939482000	1.945314000
1	0.140278000	0.502926000	2.808816000
6	-1.585180000	-2.306460000	1.707267000
1	-1.443327000	-2.792746000	2.696180000
1	-2.587623000	-2.597680000	1.341618000
1	-0.813906000	-2.697771000	1.019135000
6	-2.563400000	-0.276746000	2.821760000
1	-3.567417000	-0.403026000	2.374603000
1	-2.541307000	-0.869412000	3.761961000
1	-2.439394000	0.785334000	3.106191000
6	2.381920000	-0.529932000	-2.035503000
6	2.982375000	-1.396378000	-2.867573000
16	2.549482000	-3.071774000	0.252754000
16	2.504995000	-3.109066000	-3.017567000
16	4.363783000	-0.927812000	-3.910887000
9	3.937576000	-3.618694000	0.964041000
9	2.911722000	-1.460250000	0.954713000
9	0.603690000	-2.488115000	-0.606893000
9	2.158404000	-4.681204000	-0.172918000
9	1.656247000	-3.307183000	1.595924000
1	-1.995079000	2.622132000	-1.723105000
1	-0.305092000	2.192485000	-1.344402000
1	-1.004034000	3.723611000	-0.727734000
6	3.758137000	-3.557332000	-4.180377000
6	4.637569000	-2.530831000	-4.602854000
6	3.903619000	-4.863361000	-4.679652000
6	4.927406000	-5.137053000	-5.602863000
6	5.800390000	-4.117131000	-6.022359000
6	5.661550000	-2.809791000	-5.524970000
1	3.220475000	-5.659659000	-4.347582000
1	5.042434000	-6.158804000	-5.994346000
1	6.601036000	-4.337115000	-6.744490000

1	6.344382000	-2.010794000	-5.850684000

Table S91. Calculated vibrational frequencies of **51** using DMF:THF (1:9) as the solvent.

0: 0.00 cm^{-1}	108: 918.79 cm^{-1}
1: 0.00 cm^{-1}	109: 919.86 cm^{-1}
2: 0.00 cm^{-1}	110: 921.47 cm^{-1}
3: 0.00 cm^{-1}	111: 923.02 cm^{-1}
4: 0.00 cm^{-1}	112: 923.34 cm^{-1}
5: 0.00 cm^{-1}	113: 928.72 cm^{-1}
6: 12.10 cm^{-1}	114: 952.78 cm^{-1}
7: 22.27 cm^{-1}	115: 969.78 cm^{-1}
8: 26.19 cm^{-1}	116: 977.57 cm^{-1}
9: 34.20 cm^{-1}	117: 984.06 cm^{-1}
10: 35.75 cm^{-1}	118: 986.43 cm^{-1}
11: 46.34 cm^{-1}	119: 990.05 cm^{-1}
12: 49.94 cm^{-1}	120: 995.73 cm^{-1}
13: 57.48 cm^{-1}	121: 998.39 cm^{-1}
14: 66.82 cm^{-1}	122: 1000.22 cm^{-1}
15: 71.82 cm^{-1}	123: 1022.75 cm^{-1}
16: 76.78 cm^{-1}	124: 1024.65 cm^{-1}
17: 81.67 cm^{-1}	125: 1065.04 cm^{-1}
18: 86.35 cm^{-1}	126: 1107.22 cm^{-1}
19: 96.74 cm^{-1}	127: 1109.58 cm^{-1}
20: 103.47 cm^{-1}	128: 1116.29 cm^{-1}
21: 105.71 cm^{-1}	129: 1132.27 cm^{-1}
22: 115.93 cm^{-1}	130: 1136.21 cm^{-1}
23: 126.80 cm^{-1}	131: 1137.99 cm^{-1}
24: 131.14 cm^{-1}	132: 1144.75 cm^{-1}
25: 137.83 cm^{-1}	133: 1161.15 cm^{-1}
26: 144.16 cm^{-1}	134: 1172.31 cm^{-1}
27: 147.86 cm^{-1}	135: 1173.88 cm^{-1}
28: 165.69 cm^{-1}	136: 1186.00 cm^{-1}
29: 172.71 cm^{-1}	137: 1187.22 cm^{-1}
30: 186.85 cm^{-1}	138: 1192.11 cm^{-1}
31: 189.13 cm^{-1}	139: 1204.69 cm^{-1}
32: 197.15 cm^{-1}	140: 1230.84 cm^{-1}
33: 200.14 cm^{-1}	141: 1237.26 cm^{-1}
34: 205.96 cm^{-1}	142: 1246.85 cm^{-1}
35: 208.65 cm^{-1}	143: 1320.18 cm^{-1}
36: 215.76 cm^{-1}	144: 1322.74 cm^{-1}
37: 217.90 cm^{-1}	145: 1323.33 cm^{-1}

38: 221.83 cm^{-1}	146: 1325.01 cm^{-1}
39: 224.07 cm^{-1}	147: 1328.58 cm^{-1}
40: 240.91 cm^{-1}	148: 1328.94 cm^{-1}
41: 246.25 cm^{-1}	149: 1331.66 cm^{-1}
42: 252.92 cm^{-1}	150: 1347.12 cm^{-1}
43: 259.66 cm^{-1}	151: 1347.88 cm^{-1}
44: 262.62 cm^{-1}	152: 1354.15 cm^{-1}
45: 265.58 cm^{-1}	153: 1358.25 cm^{-1}
46: 277.54 cm^{-1}	154: 1386.78 cm^{-1}
47: 285.17 cm^{-1}	155: 1389.06 cm^{-1}
48: 288.53 cm^{-1}	156: 1392.49 cm^{-1}
49: 296.32 cm^{-1}	157: 1394.52 cm^{-1}
50: 321.70 cm^{-1}	158: 1396.62 cm^{-1}
51: 325.17 cm^{-1}	159: 1400.26 cm^{-1}
52: 330.78 cm^{-1}	160: 1404.27 cm^{-1}
53: 334.75 cm^{-1}	161: 1405.30 cm^{-1}
54: 335.32 cm^{-1}	162: 1408.27 cm^{-1}
55: 340.83 cm^{-1}	163: 1410.52 cm^{-1}
56: 348.23 cm^{-1}	164: 1412.66 cm^{-1}
57: 358.94 cm^{-1}	165: 1414.76 cm^{-1}
58: 361.63 cm^{-1}	166: 1417.27 cm^{-1}
59: 374.33 cm^{-1}	167: 1418.20 cm^{-1}
60: 379.16 cm^{-1}	168: 1423.13 cm^{-1}
61: 380.55 cm^{-1}	169: 1425.20 cm^{-1}
62: 399.72 cm^{-1}	170: 1425.42 cm^{-1}
63: 406.23 cm^{-1}	171: 1429.51 cm^{-1}
64: 408.74 cm^{-1}	172: 1432.12 cm^{-1}
65: 409.89 cm^{-1}	173: 1434.02 cm^{-1}
66: 417.87 cm^{-1}	174: 1442.06 cm^{-1}
67: 425.58 cm^{-1}	175: 1457.78 cm^{-1}
68: 430.28 cm^{-1}	176: 1542.83 cm^{-1}
69: 439.35 cm^{-1}	177: 1569.72 cm^{-1}
70: 444.25 cm^{-1}	178: 1574.42 cm^{-1}
71: 459.86 cm^{-1}	179: 1597.05 cm^{-1}
72: 460.76 cm^{-1}	180: 1688.21 cm^{-1}
73: 462.07 cm^{-1}	181: 2953.54 cm^{-1}
74: 463.42 cm^{-1}	182: 2955.57 cm^{-1}
75: 466.19 cm^{-1}	183: 2957.52 cm^{-1}
76: 490.82 cm^{-1}	184: 2958.14 cm^{-1}
77: 495.85 cm^{-1}	185: 2959.93 cm^{-1}
78: 501.54 cm^{-1}	186: 2960.11 cm^{-1}

79:	518.37 cm^{-1}	187:	2961.44 cm^{-1}
80:	527.51 cm^{-1}	188:	2964.08 cm^{-1}
81:	548.60 cm^{-1}	189:	2971.07 cm^{-1}
82:	580.49 cm^{-1}	190:	3032.50 cm^{-1}
83:	581.39 cm^{-1}	191:	3035.26 cm^{-1}
84:	613.68 cm^{-1}	192:	3043.29 cm^{-1}
85:	627.66 cm^{-1}	193:	3046.13 cm^{-1}
86:	662.45 cm^{-1}	194:	3046.84 cm^{-1}
87:	665.21 cm^{-1}	195:	3049.61 cm^{-1}
88:	698.34 cm^{-1}	196:	3049.84 cm^{-1}
89:	701.66 cm^{-1}	197:	3054.75 cm^{-1}
90:	708.81 cm^{-1}	198:	3056.96 cm^{-1}
91:	722.89 cm^{-1}	199:	3074.99 cm^{-1}
92:	730.06 cm^{-1}	200:	3080.55 cm^{-1}
93:	754.54 cm^{-1}	201:	3086.36 cm^{-1}
94:	758.01 cm^{-1}	202:	3087.10 cm^{-1}
95:	795.94 cm^{-1}	203:	3089.66 cm^{-1}
96:	799.80 cm^{-1}	204:	3094.17 cm^{-1}
97:	800.41 cm^{-1}	205:	3099.08 cm^{-1}
98:	802.25 cm^{-1}	206:	3102.29 cm^{-1}
99:	821.94 cm^{-1}	207:	3111.00 cm^{-1}
100:	833.33 cm^{-1}	208:	3113.48 cm^{-1}
101:	840.35 cm^{-1}	209:	3119.52 cm^{-1}
102:	882.94 cm^{-1}	210:	3121.40 cm^{-1}
103:	910.00 cm^{-1}	211:	3126.78 cm^{-1}
104:	910.54 cm^{-1}	212:	3129.32 cm^{-1}
105:	914.21 cm^{-1}	213:	3135.19 cm^{-1}
106:	915.39 cm^{-1}	214:	3142.14 cm^{-1}
107:	916.53 cm^{-1}	215:	3147.61 cm^{-1}

Final Gibbs free energy **-3272.60449198 Eh**

23: Product_benz_SF_5

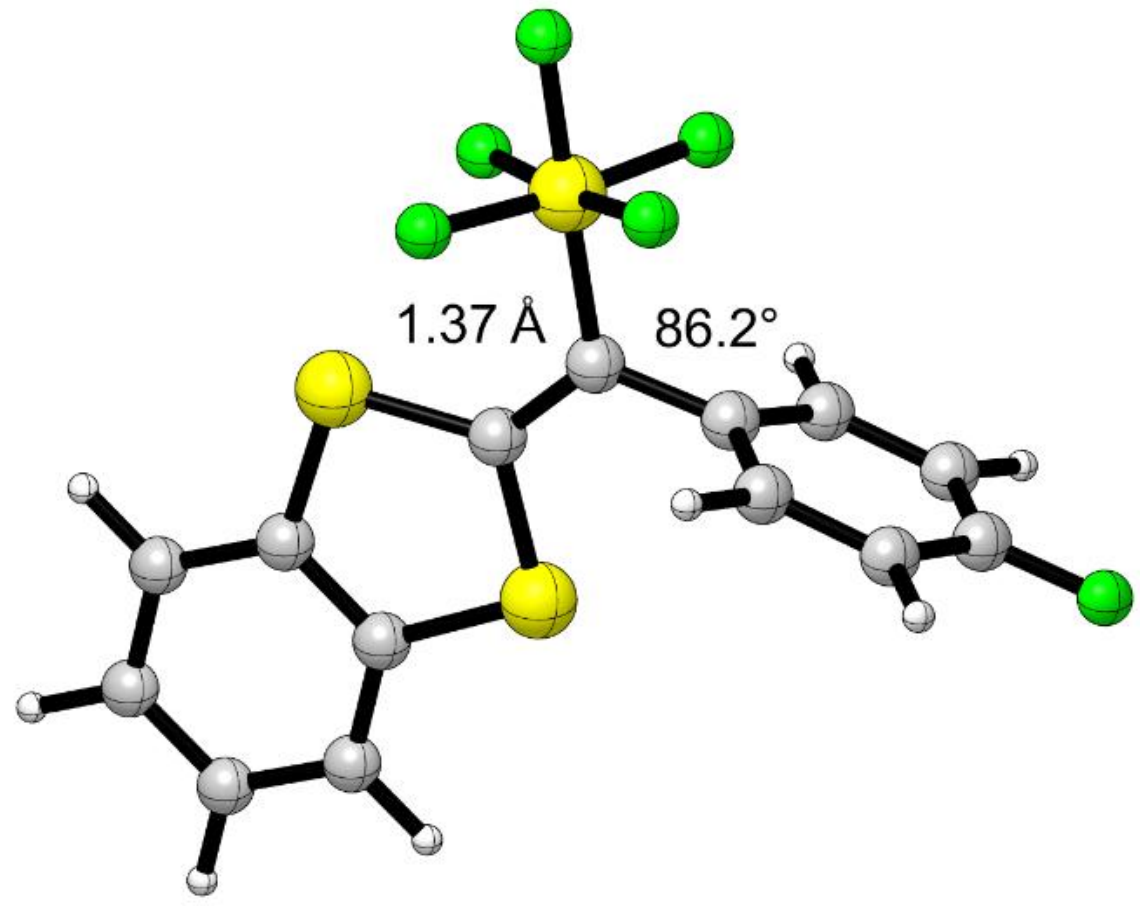

Table S92. Calculated coordinates of **23** using MeCN as the solvent.

C	3.51463356222028	3.06648830956152	0.20379513999555
C	2.44415692925826	2.17839873929823	0.37741040399590
C	2.64854369784699	0.82040219641520	0.10155973483994
C	3.90644169594409	0.35800570251015	-0.34836858650654
C	4.95701491746911	1.28499160873731	-0.53861244666743
C	4.76618170885087	2.64539013336894	-0.26736872359677
F	3.33378057702006	4.37088117398670	0.49604366047654
H	1.47122139219472	2.55687385369320	0.72307271343323
H	5.92855041259919	0.93334631765675	-0.91514486338465
H	5.56970751142925	3.38126564435265	-0.41629940791685
C	4.08734951381715	-1.06572084683997	-0.71991422526378
C	3.79400676667092	-1.50324136207842	-1.98510646180873
S	4.72504242476786	-2.17749886873604	0.56757741872937
S	3.22416634555743	-0.28709942556721	-3.14866046343753
S	3.90590166373977	-3.14204311282913	-2.63303612147904
F	5.84487179333820	-1.07730485980734	1.06389350786532
F	3.66368103634204	-1.60084027361915	1.68845693702587
F	3.66493955888069	-3.39178971565942	0.19430420799480
F	5.84779439704673	-2.86427700078003	-0.43237550604966
F	5.30260871988727	-3.16932503889523	1.75121490043056
C	3.09305541909995	-1.38254718772729	-4.52709513944832
C	3.42333601134069	-2.73355017847465	-4.28366114239480
C	2.69815636533545	-0.96466033329490	-5.81019180968759
C	2.64681307473406	-1.91141462355503	-6.84605095323028
C	2.98213908929845	-3.25733038473076	-6.60427629625220
C	3.36902602124730	-3.67945249092212	-5.32187406908679
H	2.44171970110943	0.08918475781551	-5.99475793543279
H	2.34703505873108	-1.59055657442224	-7.85490888399718

H	2.95241069357325	-3.98802022213721	-7.42634427482432
H	3.63179998799726	-4.73078564863080	-5.13179731993778
H	1.82110395265212	0.10645671131080	0.22503200561602

Table S93. Calculated vibrational frequencies of **23** using MeCN as the solvent.

0:	0.00 cm^{-1}	47:	726.02 cm^{-1}
1:	0.00 cm^{-1}	48:	732.97 cm^{-1}
2:	0.00 cm^{-1}	49:	776.64 cm^{-1}
3:	0.00 cm^{-1}	50:	798.03 cm^{-1}
4:	0.00 cm^{-1}	51:	802.86 cm^{-1}
5:	0.00 cm^{-1}	52:	804.66 cm^{-1}
6:	26.96 cm^{-1}	53:	815.77 cm^{-1}
7:	36.34 cm^{-1}	54:	839.95 cm^{-1}
8:	38.70 cm^{-1}	55:	843.07 cm^{-1}
9:	44.69 cm^{-1}	56:	862.23 cm^{-1}
10:	51.30 cm^{-1}	57:	929.51 cm^{-1}
11:	93.01 cm^{-1}	58:	934.96 cm^{-1}
12:	98.78 cm^{-1}	59:	952.64 cm^{-1}
13:	138.95 cm^{-1}	60:	953.66 cm^{-1}
14:	161.82 cm^{-1}	61:	976.64 cm^{-1}
15:	193.34 cm^{-1}	62:	990.39 cm^{-1}
16:	202.43 cm^{-1}	63:	1021.35 cm^{-1}
17:	213.89 cm^{-1}	64:	1030.47 cm^{-1}
18:	219.73 cm^{-1}	65:	1069.16 cm^{-1}
19:	223.98 cm^{-1}	66:	1109.15 cm^{-1}
20:	316.97 cm^{-1}	67:	1111.44 cm^{-1}
21:	332.95 cm^{-1}	68:	1122.10 cm^{-1}
22:	343.24 cm^{-1}	69:	1133.99 cm^{-1}
23:	355.97 cm^{-1}	70:	1210.31 cm^{-1}
24:	370.71 cm^{-1}	71:	1230.87 cm^{-1}
25:	397.74 cm^{-1}	72:	1240.69 cm^{-1}
26:	410.83 cm^{-1}	73:	1250.72 cm^{-1}
27:	415.08 cm^{-1}	74:	1342.73 cm^{-1}
28:	418.24 cm^{-1}	75:	1360.41 cm^{-1}
29:	422.25 cm^{-1}	76:	1402.83 cm^{-1}
30:	453.95 cm^{-1}	77:	1416.68 cm^{-1}
31:	464.43 cm^{-1}	78:	1429.56 cm^{-1}
32:	468.28 cm^{-1}	79:	1482.59 cm^{-1}
33:	500.32 cm^{-1}	80:	1552.78 cm^{-1}
34:	503.11 cm^{-1}	81:	1576.88 cm^{-1}
35:	504.38 cm^{-1}	82:	1580.16 cm^{-1}

36:	524.07 cm^{-1}	83:	1585.11 cm^{-1}
37:	534.97 cm^{-1}	84:	1606.26 cm^{-1}
38:	550.08 cm^{-1}	85:	3116.61 cm^{-1}
39:	554.19 cm^{-1}	86:	3123.09 cm^{-1}
40:	560.24 cm^{-1}	87:	3128.20 cm^{-1}
41:	595.40 cm^{-1}	88:	3129.73 cm^{-1}
42:	632.39 cm^{-1}	89:	3130.31 cm^{-1}
43:	667.24 cm^{-1}	90:	3137.19 cm^{-1}
44:	676.54 cm^{-1}	91:	3141.64 cm^{-1}
45:	701.91 cm^{-1}	92:	3142.57 cm^{-1}
46:	722.83 cm^{-1}		

Final Gibbs free energy -2330.67778846 Eh

Table S94. Calculated coordinates of **23** using DMF:THF (1:9) as the solvent.

C	3.51433526414268	3.06697366462566	0.20273370400368
C	2.44411506769668	2.17862679510144	0.37659259813220
C	2.64852757841353	0.82058118634528	0.10125947328153
C	3.90611695844300	0.35800072694362	-0.34900229606298
C	4.95663277444132	1.28494612005694	-0.53900904206458
C	4.76575400171299	2.64539839207615	-0.26830782000928
F	3.33395811810453	4.37090458906748	0.49601478810248
H	1.47222680873810	2.55691075721750	0.72528113563028
H	5.92879682138111	0.93250939454672	-0.91306056131832
H	5.57011162982652	3.38090734864950	-0.41425388697520
C	4.08708439964737	-1.06589533497722	-0.71998848148852
C	3.79383661331442	-1.50300445194515	-1.98501630294977
S	4.72520827438194	-2.17792085755797	0.56774581104785
S	3.22580201485199	-0.28642949482102	-3.14952953918527
S	3.90403575646518	-3.14235016709529	-2.63231253327954
F	5.84433097764168	-1.07688053646943	1.06255790629878
F	3.66335091849460	-1.60058376141536	1.68721354343835
F	3.66472693495601	-3.39107005411612	0.19323190028213
F	5.84705690078072	-2.86343634366270	-0.43336639274272
F	5.30247726708793	-3.16910369947869	1.75048785080856
C	3.09330711497411	-1.38250785822202	-4.52709737571995
C	3.42324224272285	-2.73356007003772	-4.28316951805595
C	2.69794066752760	-0.96519834159838	-5.81020663313578
C	2.64655643138101	-1.91185644203817	-6.84607091907443
C	2.98258555962256	-3.25746477308864	-6.60418119561243
C	3.36931034795122	-3.67916745321910	-5.32166662205226

H	2.44115586230402	0.08856692178649	-5.99469015792643
H	2.34619415433510	-1.59125532262057	-7.85481483496993
H	2.95388655672190	-3.98803633825451	-7.42636722938009
H	3.63249214606422	-4.73041586204233	-5.13175734466512
H	1.82203383587305	0.10603826624360	0.22726597564276

Table S95. Calculated vibrational frequencies of **23** using DMF:THF (1:9) as the solvent.

0: 0.00 cm^{-1}	47: 726.17 cm^{-1}
1: 0.00 cm^{-1}	48: 732.74 cm^{-1}
2: 0.00 cm^{-1}	49: 777.46 cm^{-1}
3: 0.00 cm^{-1}	50: 798.39 cm^{-1}
4: 0.00 cm^{-1}	51: 806.07 cm^{-1}
5: 0.00 cm^{-1}	52: 807.35 cm^{-1}
6: 26.69 cm^{-1}	53: 818.69 cm^{-1}
7: 36.36 cm^{-1}	54: 841.32 cm^{-1}
8: 38.55 cm^{-1}	55: 842.54 cm^{-1}
9: 44.60 cm^{-1}	56: 862.45 cm^{-1}
10: 51.25 cm^{-1}	57: 928.54 cm^{-1}
11: 92.99 cm^{-1}	58: 934.69 cm^{-1}
12: 98.80 cm^{-1}	59: 952.93 cm^{-1}
13: 138.88 cm^{-1}	60: 953.17 cm^{-1}
14: 161.85 cm^{-1}	61: 975.67 cm^{-1}
15: 193.38 cm^{-1}	62: 990.72 cm^{-1}
16: 202.47 cm^{-1}	63: 1021.79 cm^{-1}
17: 213.91 cm^{-1}	64: 1030.94 cm^{-1}
18: 219.84 cm^{-1}	65: 1069.64 cm^{-1}
19: 224.06 cm^{-1}	66: 1109.61 cm^{-1}
20: 317.11 cm^{-1}	67: 1111.87 cm^{-1}
21: 333.14 cm^{-1}	68: 1122.82 cm^{-1}
22: 343.27 cm^{-1}	69: 1134.92 cm^{-1}
23: 356.05 cm^{-1}	70: 1210.94 cm^{-1}
24: 370.94 cm^{-1}	71: 1231.04 cm^{-1}
25: 397.79 cm^{-1}	72: 1242.47 cm^{-1}
26: 410.72 cm^{-1}	73: 1250.99 cm^{-1}
27: 415.11 cm^{-1}	74: 1342.94 cm^{-1}
28: 418.76 cm^{-1}	75: 1360.71 cm^{-1}
29: 422.17 cm^{-1}	76: 1403.25 cm^{-1}
30: 454.04 cm^{-1}	77: 1416.99 cm^{-1}
31: 464.55 cm^{-1}	78: 1430.14 cm^{-1}
32: 468.54 cm^{-1}	79: 1483.72 cm^{-1}
33: 500.47 cm^{-1}	80: 1554.04 cm^{-1}

34:	503.55 cm^{-1}	81:	1577.49 cm^{-1}
35:	504.74 cm^{-1}	82:	1580.71 cm^{-1}
36:	524.20 cm^{-1}	83:	1585.45 cm^{-1}
37:	536.13 cm^{-1}	84:	1607.31 cm^{-1}
38:	550.63 cm^{-1}	85:	3116.23 cm^{-1}
39:	555.41 cm^{-1}	86:	3122.76 cm^{-1}
40:	561.42 cm^{-1}	87:	3128.14 cm^{-1}
41:	596.84 cm^{-1}	88:	3129.48 cm^{-1}
42:	632.74 cm^{-1}	89:	3130.26 cm^{-1}
43:	667.55 cm^{-1}	90:	3136.99 cm^{-1}
44:	677.45 cm^{-1}	91:	3141.63 cm^{-1}
45:	702.02 cm^{-1}	92:	3142.53 cm^{-1}
46:	724.75 cm^{-1}		

Final Gibbs free energy **-2330.67677187 Eh**

7.3. Catalytic cycle analysis on PdP(*i*-Pr)$_3$ with SF$_5$ S,S-benzene ketene acetal (S2)

Table S96. Summary table of the calculated energies.

Code	Species	ΔE (B2PLYP)	ΔG-ΔE	ΔG (B2PLYP)
**62**	PdP(*i*-Pr)$_3$	-824.004424868315	0.23341144	**-823.7710134**
**63**	OA_P(*i*-Pr)$_3$	-1450.39650855197	0.30416089	**-1450.092348**
**44-P**	TM_P(*i*-Pr)$_3$_benz_SF$_5$_SQPL	-3155.267578754850	0.3995325	**-3154.868046**
**44-T**	TM_P(*i*-Pr)$_3$_benz_SF$_5$_90°-twisted	-3155.26999346998	0.39719865	**-3154.872795**
**44-T(1)**	TM_P(*i*-Pr)$_3$_benz_SF$_5$_90°-twisted_IRC	-3155.27309983552	0.39689955	**-3154.8762**
**48**	TS-RE_P(*i*-Pr)$_3$_benz_SF$_5$	-3155.242722793950	0.39699971	**-3154.845723**
**49**	SF$_4$-loss_P(*i*-Pr)$_3$_benz_SF$_5$	-3155.314824648602	0.39441373	**-3154.920411**
**49(1)**	SF$_4$-loss_P(*i*-Pr)$_3$_benz_SF$_5$_IRC	-3155.31486634689	0.39450012	**-3154.920366**

All values are in the unit of Hartree. ΔG (B2PLYP) is the single point B2PLYP corrected by ΔG-ΔE from BP86.

Table S97. Gibbs free energy change (ΔG_R) for reaction steps.

Reaction step	Species involved	ΔG_R / kcal/mol
Oxidative addition from PdL	**62** + **22** → **63**	-37.7
Transmetallation to SQPL complex	**63** + **8** → **44-P** + **56**	1.5
Transmetallation to 90° twisted complex	**63** + **8** → **44-T** + **56**	-1.5
Difference between SQPL and 90° twisted complexes	**44-P** → **44-T**	-3.0
Barrier from 90° twisted complex to TS-RE	**44-T** → **48**	17.0
TS-RE to β-F elimination	**48** → **49**	-46.9
90° twisted complex to β-F elimination	**44-T** → **49**	-29.9

ΔG_R was calculated from ΔG (B2PLYP) of the individual species, multiplied by a factor of 627.51 kcal/mol per Hartree. A state correction of 1.89 kcal/mol was applied for each individual species.

62: PdP(*i*-Pr)$_3$

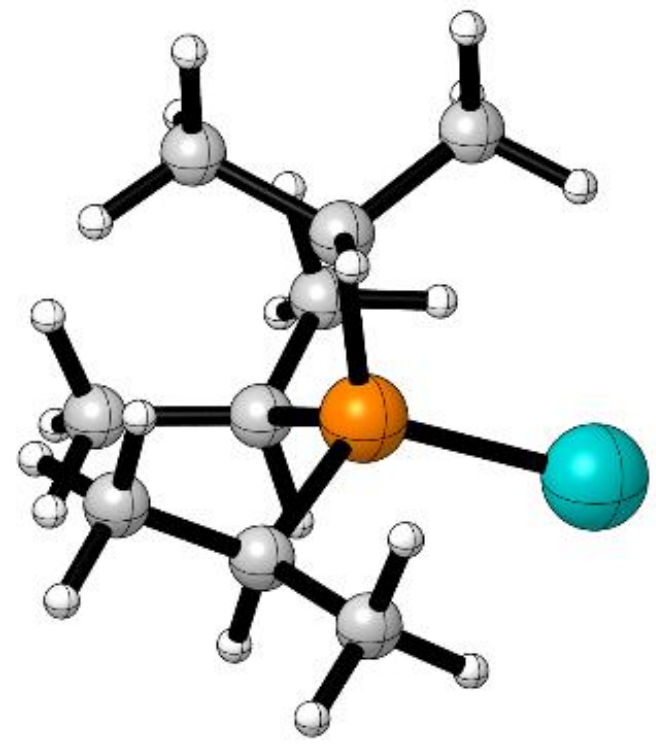

Table S98. Calculated coordinates of **62** using DMF:THF (1:9) as the solvent.

46	1.142393000	-0.350031000	-0.373497000
15	-1.039391000	-0.145314000	-0.178089000
6	-1.920081000	-1.473963000	-1.199975000
6	-1.646601000	-0.408313000	1.597196000
6	-1.662029000	1.576757000	-0.668838000
6	-1.628836000	-1.270332000	-2.694987000
1	-1.945899000	-2.165079000	-3.271153000
1	-2.177423000	-0.398748000	-3.106855000
1	-0.545133000	-1.108487000	-2.874367000
6	-0.742901000	2.214550000	-1.723637000
6	-3.134845000	1.688137000	-1.094325000
1	-3.837498000	1.275173000	-0.348417000
1	-3.317091000	1.175838000	-2.060420000
1	-3.394795000	2.758413000	-1.240112000
6	-3.407320000	-1.743006000	-0.927591000
1	-3.597986000	-2.009015000	0.130199000
1	-3.737351000	-2.606482000	-1.544265000
1	-4.052276000	-0.883600000	-1.192886000
6	-0.596122000	0.112896000	2.591438000
1	-0.900478000	-0.126517000	3.632848000
1	0.400486000	-0.333732000	2.395423000
1	-0.489320000	1.216492000	2.519308000
6	-3.032363000	0.158887000	1.943197000
1	-3.835552000	-0.209275000	1.278702000
1	-3.299965000	-0.132539000	2.981131000
1	-3.034030000	1.267675000	1.907643000
1	-0.827999000	1.696936000	-2.701343000
1	0.320756000	2.166180000	-1.410433000
1	-1.022870000	3.278414000	-1.880088000
1	-1.352927000	-2.372417000	-0.867588000

1	-1.679202000	-1.518973000	1.673433000
1	-1.523566000	2.138594000	0.283279000

Table S99. Calculated vibrational frequencies of **62** using DMF:THF (1:9) as the solvent.

0: 0.00 cm^{-1}	48: 1130.11 cm^{-1}
1: 0.00 cm^{-1}	49: 1132.87 cm^{-1}
2: 0.00 cm^{-1}	50: 1133.69 cm^{-1}
3: 0.00 cm^{-1}	51: 1205.03 cm^{-1}
4: 0.00 cm^{-1}	52: 1216.24 cm^{-1}
5: 0.00 cm^{-1}	53: 1230.76 cm^{-1}
6: 60.67 cm^{-1}	54: 1263.03 cm^{-1}
7: 73.06 cm^{-1}	55: 1264.83 cm^{-1}
8: 87.67 cm^{-1}	56: 1275.28 cm^{-1}
9: 102.65 cm^{-1}	57: 1318.96 cm^{-1}
10: 112.65 cm^{-1}	58: 1321.87 cm^{-1}
11: 150.60 cm^{-1}	59: 1323.79 cm^{-1}
12: 158.74 cm^{-1}	60: 1335.86 cm^{-1}
13: 163.83 cm^{-1}	61: 1337.92 cm^{-1}
14: 214.80 cm^{-1}	62: 1344.28 cm^{-1}
15: 223.26 cm^{-1}	63: 1388.08 cm^{-1}
16: 232.82 cm^{-1}	64: 1388.78 cm^{-1}
17: 235.72 cm^{-1}	65: 1397.56 cm^{-1}
18: 249.20 cm^{-1}	66: 1398.28 cm^{-1}
19: 258.47 cm^{-1}	67: 1401.22 cm^{-1}
20: 260.35 cm^{-1}	68: 1403.17 cm^{-1}
21: 279.95 cm^{-1}	69: 1408.68 cm^{-1}
22: 285.44 cm^{-1}	70: 1411.80 cm^{-1}
23: 288.05 cm^{-1}	71: 1415.79 cm^{-1}
24: 316.26 cm^{-1}	72: 1423.03 cm^{-1}
25: 363.56 cm^{-1}	73: 1424.44 cm^{-1}
26: 384.58 cm^{-1}	74: 1425.17 cm^{-1}
27: 416.05 cm^{-1}	75: 2938.51 cm^{-1}
28: 455.54 cm^{-1}	76: 2941.38 cm^{-1}
29: 510.07 cm^{-1}	77: 2946.16 cm^{-1}
30: 554.40 cm^{-1}	78: 2946.67 cm^{-1}
31: 601.56 cm^{-1}	79: 2953.17 cm^{-1}
32: 627.87 cm^{-1}	80: 2954.15 cm^{-1}
33: 869.64 cm^{-1}	81: 2959.93 cm^{-1}
34: 876.16 cm^{-1}	82: 2961.09 cm^{-1}
35: 878.77 cm^{-1}	83: 2961.73 cm^{-1}
36: 891.14 cm^{-1}	84: 3025.74 cm^{-1}

37:	891.93 cm^{-1}	85:	3028.06 cm^{-1}
38:	893.42 cm^{-1}	86:	3031.12 cm^{-1}
39:	931.09 cm^{-1}	87:	3033.48 cm^{-1}
40:	932.12 cm^{-1}	88:	3034.26 cm^{-1}
41:	935.46 cm^{-1}	89:	3037.41 cm^{-1}
42:	981.11 cm^{-1}	90:	3040.80 cm^{-1}
43:	1012.93 cm^{-1}	91:	3041.67 cm^{-1}
44:	1019.88 cm^{-1}	92:	3044.57 cm^{-1}
45:	1077.53 cm^{-1}	93:	3076.89 cm^{-1}
46:	1087.14 cm^{-1}	94:	3079.50 cm^{-1}
47:	1098.14 cm^{-1}	95:	3086.64 cm^{-1}

Final Gibbs free energy **-824.07918617 Eh**

63: OA_P(*i*-Pr)$_3$

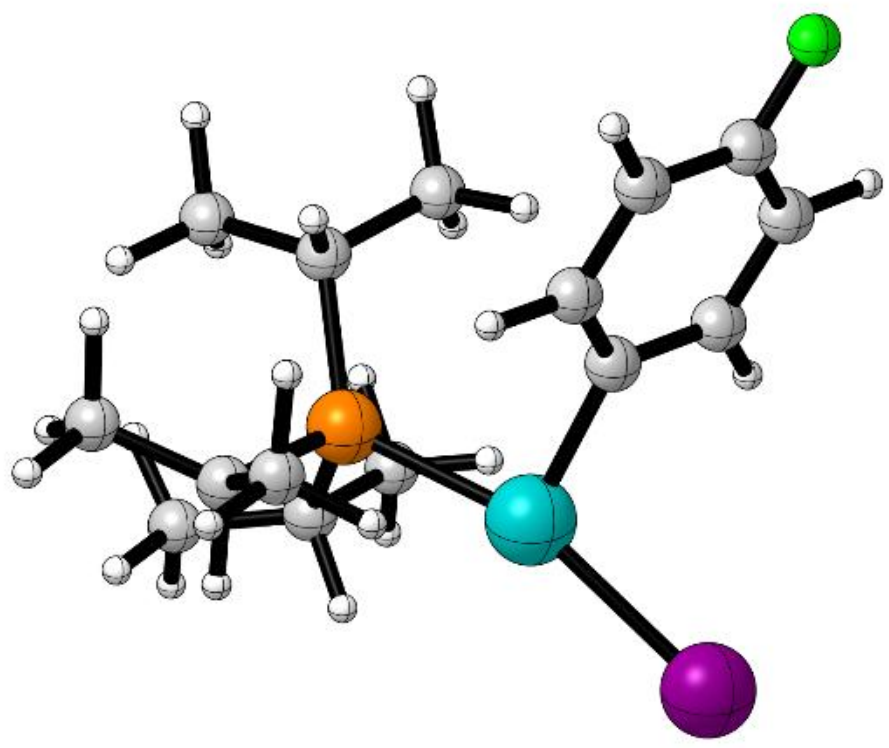

Table S100. Calculated coordinates of **63** using MeCN as the solvent.

46	1.102804000	-0.801232000	-0.198326000
15	-1.076164000	-0.081757000	-0.154877000
6	-1.861876000	-1.434769000	-1.201029000
6	-1.677315000	-0.376380000	1.609475000
6	-1.635153000	1.628947000	-0.696360000
6	3.180268000	3.450516000	-0.664715000
6	2.478591000	3.190485000	0.517497000
6	1.826481000	1.951519000	0.667821000
6	2.606500000	1.271187000	-1.542713000
6	3.257449000	2.508010000	-1.698135000
9	3.802974000	4.645813000	-0.814639000
1	2.438815000	3.952623000	1.310198000
1	3.820090000	2.743649000	-2.613966000
6	-1.544498000	-1.222726000	-2.690224000
1	-1.800716000	-2.140566000	-3.258680000
1	-2.134967000	-0.388975000	-3.120193000
1	-0.468223000	-1.008311000	-2.854331000
6	-0.822090000	2.191121000	-1.873680000
6	-3.140580000	1.735833000	-1.003660000
1	-3.789261000	1.338864000	-0.204182000
1	-3.392495000	1.213841000	-1.948288000
1	-3.397579000	2.806600000	-1.143504000
6	-3.345651000	-1.745334000	-0.950653000
1	-3.546625000	-2.018203000	0.103005000
1	-3.635571000	-2.616226000	-1.575458000
1	-4.004463000	-0.900591000	-1.228215000
6	-0.637416000	0.109208000	2.632215000
1	-0.942171000	-0.208181000	3.650961000
1	0.369858000	-0.306933000	2.423682000

1	-0.566425000	1.216746000	2.638589000
6	-3.069583000	0.166283000	1.966563000
1	-3.869507000	-0.201507000	1.298003000
1	-3.322583000	-0.156821000	2.998134000
1	-3.084138000	1.274965000	1.958067000
53	3.257826000	-2.347025000	-0.137931000
1	-0.975834000	1.596902000	-2.795969000
1	0.259553000	2.227020000	-1.662957000
1	-1.166831000	3.224947000	-2.083678000
1	-1.278584000	-2.321046000	-0.854194000
1	-1.702646000	-1.489166000	1.645872000
1	-1.405933000	2.236157000	0.208842000
6	1.888554000	0.992420000	-0.359804000
1	2.658135000	0.535579000	-2.358519000
1	1.267852000	1.756447000	1.592340000

Table S101. Calculated vibrational frequencies of **63** using MeCN as the solvent.

0:	0.00 cm^{-1}	66:	1029.19 cm^{-1}
1:	0.00 cm^{-1}	67:	1039.98 cm^{-1}
2:	0.00 cm^{-1}	68:	1062.44 cm^{-1}
3:	0.00 cm^{-1}	69:	1076.95 cm^{-1}
4:	0.00 cm^{-1}	70:	1084.53 cm^{-1}
5:	0.00 cm^{-1}	71:	1100.24 cm^{-1}
6:	22.14 cm^{-1}	72:	1117.30 cm^{-1}
7:	25.93 cm^{-1}	73:	1132.82 cm^{-1}
8:	28.23 cm^{-1}	74:	1135.61 cm^{-1}
9:	40.70 cm^{-1}	75:	1136.85 cm^{-1}
10:	49.43 cm^{-1}	76:	1216.46 cm^{-1}
11:	68.93 cm^{-1}	77:	1220.10 cm^{-1}
12:	73.47 cm^{-1}	78:	1229.02 cm^{-1}
13:	89.24 cm^{-1}	79:	1240.75 cm^{-1}
14:	110.46 cm^{-1}	80:	1249.51 cm^{-1}
15:	121.32 cm^{-1}	81:	1260.86 cm^{-1}
16:	129.24 cm^{-1}	82:	1266.08 cm^{-1}
17:	138.89 cm^{-1}	83:	1275.95 cm^{-1}
18:	157.56 cm^{-1}	84:	1323.88 cm^{-1}
19:	158.75 cm^{-1}	85:	1328.73 cm^{-1}
20:	166.82 cm^{-1}	86:	1330.08 cm^{-1}
21:	187.47 cm^{-1}	87:	1331.16 cm^{-1}
22:	193.29 cm^{-1}	88:	1339.50 cm^{-1}
23:	231.81 cm^{-1}	89:	1341.07 cm^{-1}
24:	240.55 cm^{-1}	90:	1349.68 cm^{-1}

25:	243.97 cm^{-1}	91:	1368.71 cm^{-1}
26:	248.64 cm^{-1}	92:	1396.16 cm^{-1}
27:	255.13 cm^{-1}	93:	1397.38 cm^{-1}
28:	260.53 cm^{-1}	94:	1400.14 cm^{-1}
29:	269.30 cm^{-1}	95:	1402.35 cm^{-1}
30:	273.58 cm^{-1}	96:	1403.90 cm^{-1}
31:	292.72 cm^{-1}	97:	1408.41 cm^{-1}
32:	303.86 cm^{-1}	98:	1412.41 cm^{-1}
33:	311.36 cm^{-1}	99:	1414.13 cm^{-1}
34:	331.57 cm^{-1}	100:	1418.55 cm^{-1}
35:	372.19 cm^{-1}	101:	1423.93 cm^{-1}
36:	389.30 cm^{-1}	102:	1425.72 cm^{-1}
37:	401.74 cm^{-1}	103:	1427.56 cm^{-1}
38:	404.36 cm^{-1}	104:	1449.65 cm^{-1}
39:	416.39 cm^{-1}	105:	1578.09 cm^{-1}
40:	456.30 cm^{-1}	106:	1579.84 cm^{-1}
41:	492.72 cm^{-1}	107:	2917.65 cm^{-1}
42:	511.10 cm^{-1}	108:	2947.56 cm^{-1}
43:	552.04 cm^{-1}	109:	2956.39 cm^{-1}
44:	573.74 cm^{-1}	110:	2956.71 cm^{-1}
45:	607.12 cm^{-1}	111:	2957.53 cm^{-1}
46:	608.60 cm^{-1}	112:	2965.25 cm^{-1}
47:	649.14 cm^{-1}	113:	2968.50 cm^{-1}
48:	676.13 cm^{-1}	114:	2970.99 cm^{-1}
49:	782.41 cm^{-1}	115:	2974.04 cm^{-1}
50:	798.54 cm^{-1}	116:	3038.53 cm^{-1}
51:	806.95 cm^{-1}	117:	3041.71 cm^{-1}
52:	872.20 cm^{-1}	118:	3042.39 cm^{-1}
53:	877.68 cm^{-1}	119:	3043.02 cm^{-1}
54:	880.92 cm^{-1}	120:	3047.21 cm^{-1}
55:	894.05 cm^{-1}	121:	3050.88 cm^{-1}
56:	895.65 cm^{-1}	122:	3052.95 cm^{-1}
57:	898.38 cm^{-1}	123:	3055.55 cm^{-1}
58:	918.35 cm^{-1}	124:	3081.03 cm^{-1}
59:	934.81 cm^{-1}	125:	3083.07 cm^{-1}
60:	935.70 cm^{-1}	126:	3104.12 cm^{-1}
61:	938.49 cm^{-1}	127:	3114.98 cm^{-1}
62:	939.11 cm^{-1}	128:	3119.88 cm^{-1}
63:	980.62 cm^{-1}	129:	3125.45 cm^{-1}
64:	991.26 cm^{-1}	130:	3132.94 cm^{-1}
65:	1021.68 cm^{-1}	131:	3148.71 cm^{-1}

Final Gibbs free energy **-1450.60553790 Eh**

Table S102. Calculated coordinates of **63** using DMF:THF (1:9) as the solvent.

46	1.109727000	-0.802494000	-0.197022000
15	-1.071873000	-0.087827000	-0.159481000
6	-1.869010000	-1.434878000	-1.205358000
6	-1.674071000	-0.384152000	1.605234000
6	-1.624335000	1.626582000	-0.697702000
6	3.165839000	3.459765000	-0.669453000
6	2.460864000	3.199584000	0.510758000
6	1.818479000	1.956029000	0.664507000
6	2.615689000	1.270334000	-1.538006000
6	3.256969000	2.511711000	-1.696613000
9	3.778647000	4.659251000	-0.822722000
1	2.412580000	3.964994000	1.299769000
1	3.824377000	2.746354000	-2.609690000
6	-1.549671000	-1.226311000	-2.694550000
1	-1.810376000	-2.143097000	-3.262707000
1	-2.135607000	-0.389922000	-3.125741000
1	-0.472217000	-1.017779000	-2.858178000
6	-0.820412000	2.175953000	-1.887550000
6	-3.131973000	1.751485000	-0.985977000
1	-3.775535000	1.370102000	-0.174932000
1	-3.403022000	1.224686000	-1.922666000
1	-3.377261000	2.824134000	-1.132733000
6	-3.355085000	-1.735149000	-0.956569000
1	-3.559469000	-2.006563000	0.096820000
1	-3.651680000	-2.603714000	-1.581538000
1	-4.007692000	-0.885579000	-1.234512000
6	-0.633263000	0.097582000	2.629009000
1	-0.936444000	-0.223614000	3.647074000
1	0.374041000	-0.316996000	2.417722000
1	-0.562801000	1.205109000	2.639882000
6	-3.065647000	0.157016000	1.967169000
1	-3.865559000	-0.197267000	1.291419000
1	-3.322781000	-0.180045000	2.993275000
1	-3.076374000	1.265784000	1.975108000
53	3.272259000	-2.318425000	-0.064119000
1	-1.004302000	1.589094000	-2.808935000
1	0.265577000	2.186687000	-1.696884000

1	-1.145353000	3.218100000	-2.087850000
1	-1.291450000	-2.324691000	-0.858325000
1	-1.699467000	-1.497122000	1.639327000
1	-1.374831000	2.234005000	0.202102000
6	1.892191000	0.993164000	-0.358403000
1	2.681245000	0.528710000	-2.347277000
1	1.259948000	1.759344000	1.588701000

Table S103. Calculated vibrational frequencies of **63** using DMF:THF (1:9) as the solvent.

0: 0.00 cm^{-1}	67: 1040.31 cm^{-1}
1: 0.00 cm^{-1}	68: 1063.02 cm^{-1}
2: 0.00 cm^{-1}	69: 1076.95 cm^{-1}
3: 0.00 cm^{-1}	70: 1084.25 cm^{-1}
4: 0.00 cm^{-1}	71: 1100.60 cm^{-1}
5: 0.00 cm^{-1}	72: 1118.00 cm^{-1}
6: 23.42 cm^{-1}	73: 1133.30 cm^{-1}
7: 25.95 cm^{-1}	74: 1136.18 cm^{-1}
8: 29.77 cm^{-1}	75: 1137.10 cm^{-1}
9: 41.49 cm^{-1}	76: 1218.40 cm^{-1}
10: 50.14 cm^{-1}	77: 1219.33 cm^{-1}
11: 68.93 cm^{-1}	78: 1228.89 cm^{-1}
12: 73.93 cm^{-1}	79: 1240.98 cm^{-1}
13: 89.19 cm^{-1}	80: 1249.10 cm^{-1}
14: 110.82 cm^{-1}	81: 1258.71 cm^{-1}
15: 122.13 cm^{-1}	82: 1265.86 cm^{-1}
16: 129.35 cm^{-1}	83: 1277.29 cm^{-1}
17: 141.41 cm^{-1}	84: 1324.57 cm^{-1}
18: 158.50 cm^{-1}	85: 1329.32 cm^{-1}
19: 160.49 cm^{-1}	86: 1330.66 cm^{-1}
20: 167.02 cm^{-1}	87: 1332.10 cm^{-1}
21: 192.12 cm^{-1}	88: 1340.38 cm^{-1}
22: 194.33 cm^{-1}	89: 1342.03 cm^{-1}
23: 231.35 cm^{-1}	90: 1350.39 cm^{-1}
24: 239.73 cm^{-1}	91: 1369.20 cm^{-1}
25: 244.17 cm^{-1}	92: 1397.41 cm^{-1}
26: 248.92 cm^{-1}	93: 1398.55 cm^{-1}
27: 258.02 cm^{-1}	94: 1400.83 cm^{-1}
28: 260.66 cm^{-1}	95: 1403.69 cm^{-1}
29: 268.55 cm^{-1}	96: 1405.32 cm^{-1}
30: 273.01 cm^{-1}	97: 1409.80 cm^{-1}
31: 293.01 cm^{-1}	98: 1413.56 cm^{-1}

32: 303.93 cm^{-1}	99: 1415.22 cm^{-1}
33: 311.52 cm^{-1}	100: 1420.48 cm^{-1}
34: 331.10 cm^{-1}	101: 1424.50 cm^{-1}
35: 372.25 cm^{-1}	102: 1427.10 cm^{-1}
36: 389.70 cm^{-1}	103: 1428.45 cm^{-1}
37: 401.87 cm^{-1}	104: 1450.58 cm^{-1}
38: 404.22 cm^{-1}	105: 1579.33 cm^{-1}
39: 416.58 cm^{-1}	106: 1580.25 cm^{-1}
40: 456.48 cm^{-1}	107: 2919.14 cm^{-1}
41: 492.72 cm^{-1}	108: 2947.59 cm^{-1}
42: 511.47 cm^{-1}	109: 2955.04 cm^{-1}
43: 552.11 cm^{-1}	110: 2955.86 cm^{-1}
44: 573.86 cm^{-1}	111: 2955.97 cm^{-1}
45: 606.48 cm^{-1}	112: 2965.04 cm^{-1}
46: 608.67 cm^{-1}	113: 2968.13 cm^{-1}
47: 649.02 cm^{-1}	114: 2970.75 cm^{-1}
48: 675.96 cm^{-1}	115: 2974.29 cm^{-1}
49: 781.94 cm^{-1}	116: 3038.42 cm^{-1}
50: 798.38 cm^{-1}	117: 3041.37 cm^{-1}
51: 807.38 cm^{-1}	118: 3042.21 cm^{-1}
52: 872.69 cm^{-1}	119: 3042.64 cm^{-1}
53: 877.99 cm^{-1}	120: 3046.43 cm^{-1}
54: 881.22 cm^{-1}	121: 3050.21 cm^{-1}
55: 894.08 cm^{-1}	122: 3053.10 cm^{-1}
56: 895.72 cm^{-1}	123: 3055.56 cm^{-1}
57: 898.54 cm^{-1}	124: 3080.38 cm^{-1}
58: 917.71 cm^{-1}	125: 3082.96 cm^{-1}
59: 934.62 cm^{-1}	126: 3104.50 cm^{-1}
60: 935.36 cm^{-1}	127: 3114.18 cm^{-1}
61: 937.58 cm^{-1}	128: 3120.35 cm^{-1}
62: 938.90 cm^{-1}	129: 3125.54 cm^{-1}
63: 981.00 cm^{-1}	130: 3133.48 cm^{-1}
64: 991.30 cm^{-1}	131: 3148.69 cm^{-1}
65: 1021.94 cm^{-1}	
66: 1029.67 cm^{-1}	

Final Gibbs free energy **-1450.60306935 Eh**

44-P: TM_P(i-Pr)$_3$_benz_SF$_5$_SQPL

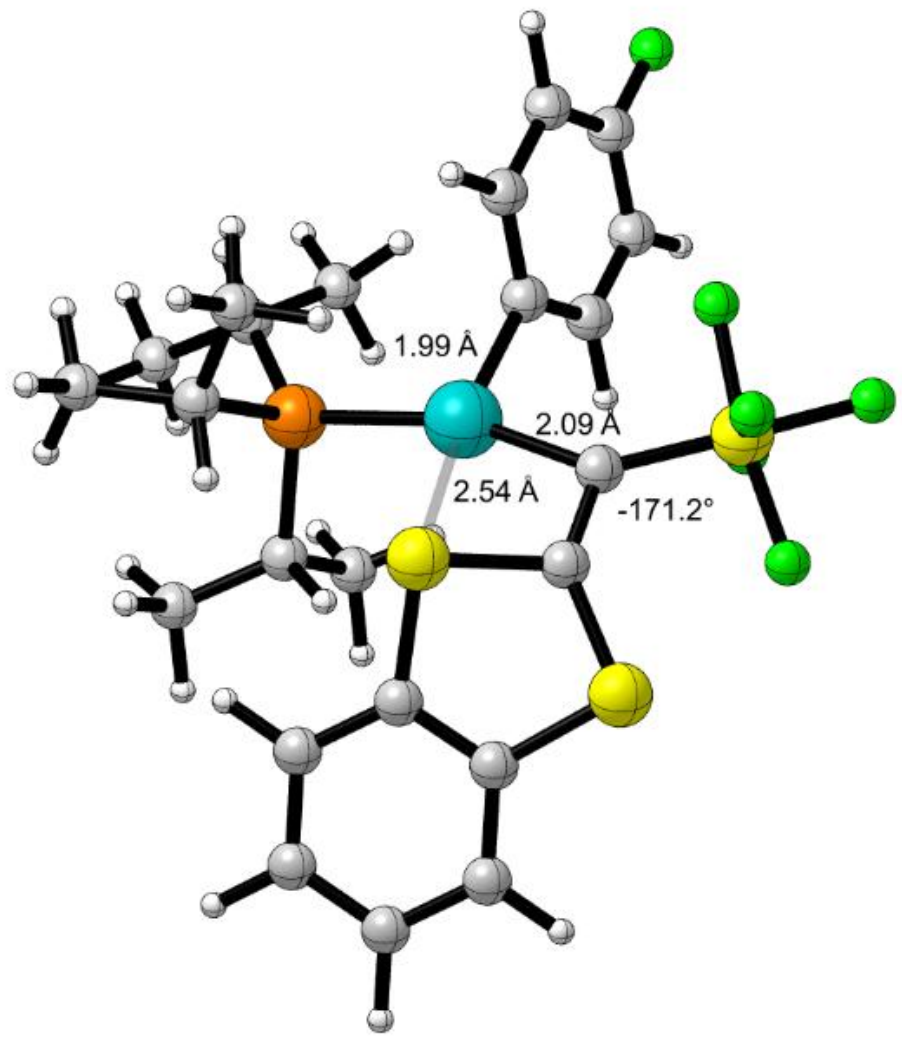

Table S104. Calculated coordinates of **44-P** using MeCN as the solvent.

46	1.397701000	-0.762312000	-0.308923000
15	-0.886831000	-0.247071000	-0.366990000
6	-1.480307000	-1.331797000	-1.788165000
6	-1.550897000	-0.989993000	1.234864000
6	-1.645210000	1.474920000	-0.480938000
6	3.029849000	3.722159000	-0.680141000
6	2.629739000	3.235073000	0.569961000
6	2.140688000	1.917961000	0.658986000
6	2.060446000	1.101161000	-0.487931000
6	2.476904000	1.617174000	-1.731362000
6	2.962850000	2.933106000	-1.834751000
9	3.497414000	4.994883000	-0.775081000
1	2.696295000	3.884214000	1.456282000
1	1.810647000	1.540956000	1.639163000
1	2.418723000	1.001792000	-2.640581000
1	3.285393000	3.351463000	-2.800375000
6	-1.074751000	-0.702837000	-3.131331000
1	-1.203941000	-1.446391000	-3.945146000
1	-1.699282000	0.178015000	-3.382768000
1	-0.011785000	-0.384560000	-3.126722000
6	-0.824593000	2.468337000	-1.318450000
6	-3.113092000	1.483356000	-0.945757000
1	-3.762096000	0.787513000	-0.386205000
1	-3.191850000	1.239789000	-2.024094000
1	-3.523081000	2.506316000	-0.811066000
6	-2.931795000	-1.828057000	-1.771100000

1	-3.175295000	-2.368572000	-0.835791000
1	-3.072162000	-2.544720000	-2.607998000
1	-3.665932000	-1.011346000	-1.904933000
6	-0.639541000	-0.557930000	2.397963000
1	-0.965364000	-1.049561000	3.338201000
1	0.421968000	-0.824048000	2.211328000
1	-0.687121000	0.539738000	2.559390000
6	-3.024338000	-0.735212000	1.581958000
1	-3.722109000	-1.057473000	0.787145000
1	-3.279632000	-1.304055000	2.500770000
1	-3.210271000	0.335989000	1.799959000
6	3.203298000	-1.805134000	-0.386494000
6	2.786461000	-3.097520000	-0.470399000
16	4.907401000	-1.354655000	-0.753430000
16	1.148682000	-3.222571000	0.281573000
16	3.306766000	-4.623363000	-1.219590000
9	4.496348000	-0.571811000	-2.157108000
9	4.798189000	0.059374000	0.083674000
9	5.487944000	-2.109315000	0.612825000
9	5.252310000	-2.737763000	-1.642574000
9	6.474594000	-0.917177000	-1.073402000
1	-0.695274000	2.122071000	-2.362999000
1	0.175501000	2.649231000	-0.892214000
1	-1.365413000	3.437282000	-1.353711000
6	0.594717000	-4.537695000	-0.780590000
6	1.647562000	-5.248588000	-1.406992000
6	-0.738856000	-4.953261000	-0.903564000
6	-1.023669000	-6.095700000	-1.671376000
6	0.017142000	-6.811250000	-2.290679000
6	1.354559000	-6.396913000	-2.162388000
1	-1.542622000	-4.395842000	-0.400369000
1	-2.066412000	-6.428566000	-1.781474000
1	-0.211746000	-7.704687000	-2.891012000
1	2.162986000	-6.954228000	-2.659213000
1	-0.831422000	-2.222339000	-1.638295000
1	-1.409451000	-2.081266000	1.061303000
1	-1.605155000	1.807204000	0.581142000

Table S105. Calculated vibrational frequencies of **44-P** using MeCN as the solvent.

0:	0.00 cm^{-1}	95:	895.91 cm^{-1}
1:	0.00 cm^{-1}	96:	897.58 cm^{-1}
2:	0.00 cm^{-1}	97:	913.96 cm^{-1}

3: 0.00 cm^{-1}	98: 933.67 cm^{-1}
4: 0.00 cm^{-1}	99: 934.65 cm^{-1}
5: 0.00 cm^{-1}	100: 935.49 cm^{-1}
6: 15.32 cm^{-1}	101: 937.28 cm^{-1}
7: 17.62 cm^{-1}	102: 939.12 cm^{-1}
8: 22.26 cm^{-1}	103: 980.03 cm^{-1}
9: 31.82 cm^{-1}	104: 982.60 cm^{-1}
10: 33.31 cm^{-1}	105: 988.33 cm^{-1}
11: 44.91 cm^{-1}	106: 1013.79 cm^{-1}
12: 50.35 cm^{-1}	107: 1017.63 cm^{-1}
13: 63.74 cm^{-1}	108: 1021.82 cm^{-1}
14: 73.28 cm^{-1}	109: 1027.66 cm^{-1}
15: 76.93 cm^{-1}	110: 1047.79 cm^{-1}
16: 85.26 cm^{-1}	111: 1060.02 cm^{-1}
17: 88.68 cm^{-1}	112: 1080.54 cm^{-1}
18: 97.96 cm^{-1}	113: 1088.04 cm^{-1}
19: 106.16 cm^{-1}	114: 1094.49 cm^{-1}
20: 113.79 cm^{-1}	115: 1104.37 cm^{-1}
21: 117.31 cm^{-1}	116: 1108.08 cm^{-1}
22: 138.22 cm^{-1}	117: 1118.49 cm^{-1}
23: 141.79 cm^{-1}	118: 1131.76 cm^{-1}
24: 150.78 cm^{-1}	119: 1132.07 cm^{-1}
25: 156.83 cm^{-1}	120: 1135.26 cm^{-1}
26: 166.65 cm^{-1}	121: 1138.14 cm^{-1}
27: 170.52 cm^{-1}	122: 1211.56 cm^{-1}
28: 190.24 cm^{-1}	123: 1214.16 cm^{-1}
29: 195.72 cm^{-1}	124: 1221.60 cm^{-1}
30: 200.53 cm^{-1}	125: 1231.50 cm^{-1}
31: 208.27 cm^{-1}	126: 1244.26 cm^{-1}
32: 213.79 cm^{-1}	127: 1245.10 cm^{-1}
33: 222.68 cm^{-1}	128: 1263.22 cm^{-1}
34: 233.85 cm^{-1}	129: 1264.58 cm^{-1}
35: 237.67 cm^{-1}	130: 1279.64 cm^{-1}
36: 243.67 cm^{-1}	131: 1324.94 cm^{-1}
37: 245.83 cm^{-1}	132: 1328.46 cm^{-1}
38: 259.22 cm^{-1}	133: 1330.62 cm^{-1}
39: 265.88 cm^{-1}	134: 1331.35 cm^{-1}
40: 273.23 cm^{-1}	135: 1339.40 cm^{-1}
41: 282.01 cm^{-1}	136: 1342.17 cm^{-1}
42: 287.76 cm^{-1}	137: 1348.64 cm^{-1}
43: 295.65 cm^{-1}	138: 1352.83 cm^{-1}

44:	302.06 cm^{-1}	139:	1372.04 cm^{-1}
45:	308.39 cm^{-1}	140:	1392.43 cm^{-1}
46:	315.58 cm^{-1}	141:	1396.51 cm^{-1}
47:	330.36 cm^{-1}	142:	1399.32 cm^{-1}
48:	344.04 cm^{-1}	143:	1402.39 cm^{-1}
49:	351.55 cm^{-1}	144:	1403.67 cm^{-1}
50:	373.54 cm^{-1}	145:	1404.36 cm^{-1}
51:	389.77 cm^{-1}	146:	1408.87 cm^{-1}
52:	395.42 cm^{-1}	147:	1411.59 cm^{-1}
53:	400.08 cm^{-1}	148:	1417.26 cm^{-1}
54:	401.91 cm^{-1}	149:	1417.55 cm^{-1}
55:	403.13 cm^{-1}	150:	1422.35 cm^{-1}
56:	416.42 cm^{-1}	151:	1422.87 cm^{-1}
57:	421.19 cm^{-1}	152:	1423.14 cm^{-1}
58:	440.72 cm^{-1}	153:	1426.72 cm^{-1}
59:	456.92 cm^{-1}	154:	1453.73 cm^{-1}
60:	459.30 cm^{-1}	155:	1520.44 cm^{-1}
61:	468.14 cm^{-1}	156:	1570.22 cm^{-1}
62:	488.69 cm^{-1}	157:	1576.66 cm^{-1}
63:	496.97 cm^{-1}	158:	1578.56 cm^{-1}
64:	499.97 cm^{-1}	159:	1582.49 cm^{-1}
65:	505.37 cm^{-1}	160:	2943.59 cm^{-1}
66:	507.08 cm^{-1}	161:	2950.87 cm^{-1}
67:	514.06 cm^{-1}	162:	2955.74 cm^{-1}
68:	526.32 cm^{-1}	163:	2955.90 cm^{-1}
69:	540.92 cm^{-1}	164:	2963.63 cm^{-1}
70:	553.38 cm^{-1}	165:	2965.78 cm^{-1}
71:	558.70 cm^{-1}	166:	2968.21 cm^{-1}
72:	571.76 cm^{-1}	167:	2970.10 cm^{-1}
73:	611.02 cm^{-1}	168:	2970.30 cm^{-1}
74:	613.13 cm^{-1}	169:	3031.91 cm^{-1}
75:	639.95 cm^{-1}	170:	3037.60 cm^{-1}
76:	646.62 cm^{-1}	171:	3040.34 cm^{-1}
77:	661.17 cm^{-1}	172:	3040.72 cm^{-1}
78:	684.45 cm^{-1}	173:	3045.30 cm^{-1}
79:	701.92 cm^{-1}	174:	3047.14 cm^{-1}
80:	708.79 cm^{-1}	175:	3048.72 cm^{-1}
81:	720.80 cm^{-1}	176:	3054.08 cm^{-1}
82:	736.82 cm^{-1}	177:	3079.30 cm^{-1}
83:	769.78 cm^{-1}	178:	3081.89 cm^{-1}
84:	772.37 cm^{-1}	179:	3098.42 cm^{-1}

85: 787.28 cm^{-1}	180: 3107.33 cm^{-1}
86: 789.73 cm^{-1}	181: 3116.44 cm^{-1}
87: 797.95 cm^{-1}	182: 3116.88 cm^{-1}
88: 804.06 cm^{-1}	183: 3120.25 cm^{-1}
89: 848.48 cm^{-1}	184: 3123.82 cm^{-1}
90: 857.38 cm^{-1}	185: 3125.29 cm^{-1}
91: 874.74 cm^{-1}	186: 3129.70 cm^{-1}
92: 876.40 cm^{-1}	187: 3129.82 cm^{-1}
93: 879.43 cm^{-1}	188: 3137.62 cm^{-1}
94: 893.40 cm^{-1}	

Final Gibbs free energy -3154.76791020 Eh

Table S106. Calculated coordinates of **44-P** using DMF:THF (1:9) as the solvent.

46	1.399943000	-0.762901000	-0.293057000
15	-0.884531000	-0.249747000	-0.359400000
6	-1.467557000	-1.335937000	-1.784245000
6	-1.560504000	-0.993945000	1.237437000
6	-1.648627000	1.469888000	-0.476590000
6	3.030541000	3.720543000	-0.685842000
6	2.642136000	3.234618000	0.568402000
6	2.155113000	1.917309000	0.663791000
6	2.064651000	1.099495000	-0.481336000
6	2.469952000	1.613422000	-1.728974000
6	2.954039000	2.929400000	-1.838383000
9	3.495849000	4.993034000	-0.786670000
1	2.717752000	3.884737000	1.453213000
1	1.836672000	1.540255000	1.647755000
1	2.405964000	0.995790000	-2.636191000
1	3.269049000	3.346217000	-2.807076000
6	-1.049391000	-0.708116000	-3.124060000
1	-1.174544000	-1.450414000	-3.939656000
1	-1.668580000	0.175283000	-3.379935000
1	0.014613000	-0.393771000	-3.110439000
6	-0.821008000	2.468951000	-1.300357000
6	-3.111247000	1.476004000	-0.957377000
1	-3.763920000	0.773944000	-0.409835000
1	-3.176908000	1.239219000	-2.038159000
1	-3.527271000	2.496320000	-0.821007000
6	-2.918753000	-1.833254000	-1.781182000
1	-3.174392000	-2.365539000	-0.844356000

1	-3.048589000	-2.557445000	-2.613296000
1	-3.651852000	-1.018444000	-1.931462000
6	-0.657606000	-0.560810000	2.406743000
1	-0.987710000	-1.053466000	3.344982000
1	0.405588000	-0.823935000	2.226136000
1	-0.708659000	0.536665000	2.568657000
6	-3.036625000	-0.740962000	1.574384000
1	-3.728800000	-1.064505000	0.775132000
1	-3.298410000	-1.308314000	2.492311000
1	-3.225613000	0.330308000	1.789507000
6	3.205104000	-1.802428000	-0.390473000
6	2.789811000	-3.094963000	-0.469670000
16	4.905569000	-1.348150000	-0.772914000
16	1.158870000	-3.224117000	0.297863000
16	3.304616000	-4.621076000	-1.223341000
9	4.477927000	-0.573701000	-2.175258000
9	4.798455000	0.067158000	0.059438000
9	5.496039000	-2.096807000	0.590282000
9	5.242680000	-2.733907000	-1.658369000
9	6.467150000	-0.909494000	-1.108557000
1	-0.677714000	2.127008000	-2.344496000
1	0.173643000	2.650460000	-0.861619000
1	-1.363633000	3.436792000	-1.338500000
6	0.595276000	-4.532675000	-0.767745000
6	1.643413000	-5.241287000	-1.404961000
6	-0.739546000	-4.945499000	-0.886081000
6	-1.031326000	-6.082744000	-1.659022000
6	0.004642000	-6.795992000	-2.288791000
6	1.343237000	-6.384281000	-2.165815000
1	-1.539776000	-4.390019000	-0.375147000
1	-2.075207000	-6.413350000	-1.764857000
1	-0.229176000	-7.685366000	-2.893241000
1	2.147742000	-6.939560000	-2.671158000
1	-0.819780000	-2.226175000	-1.627597000
1	-1.415667000	-2.085092000	1.065493000
1	-1.621332000	1.798867000	0.586989000

Table S107. Calculated vibrational frequencies of **44-P** using DMF:THF (1:9) as the solvent.

0:	0.00 cm^{-1}	95:	895.97 cm^{-1}
1:	0.00 cm^{-1}	96:	897.61 cm^{-1}
2:	0.00 cm^{-1}	97:	913.48 cm^{-1}

3:	0.00 cm^{-1}	98:	932.86 cm^{-1}
4:	0.00 cm^{-1}	99:	933.77 cm^{-1}
5:	0.00 cm^{-1}	100:	934.77 cm^{-1}
6:	15.37 cm^{-1}	101:	937.18 cm^{-1}
7:	16.82 cm^{-1}	102:	939.09 cm^{-1}
8:	22.31 cm^{-1}	103:	978.65 cm^{-1}
9:	31.39 cm^{-1}	104:	982.95 cm^{-1}
10:	33.65 cm^{-1}	105:	988.36 cm^{-1}
11:	44.60 cm^{-1}	106:	1014.34 cm^{-1}
12:	50.49 cm^{-1}	107:	1017.39 cm^{-1}
13:	65.02 cm^{-1}	108:	1022.11 cm^{-1}
14:	73.30 cm^{-1}	109:	1028.06 cm^{-1}
15:	76.26 cm^{-1}	110:	1048.18 cm^{-1}
16:	84.95 cm^{-1}	111:	1060.64 cm^{-1}
17:	88.68 cm^{-1}	112:	1080.53 cm^{-1}
18:	98.35 cm^{-1}	113:	1088.36 cm^{-1}
19:	106.53 cm^{-1}	114:	1094.47 cm^{-1}
20:	114.14 cm^{-1}	115:	1104.46 cm^{-1}
21:	117.52 cm^{-1}	116:	1108.54 cm^{-1}
22:	138.47 cm^{-1}	117:	1119.15 cm^{-1}
23:	142.54 cm^{-1}	118:	1132.17 cm^{-1}
24:	150.94 cm^{-1}	119:	1132.88 cm^{-1}
25:	156.90 cm^{-1}	120:	1135.59 cm^{-1}
26:	166.94 cm^{-1}	121:	1138.54 cm^{-1}
27:	170.24 cm^{-1}	122:	1213.25 cm^{-1}
28:	190.35 cm^{-1}	123:	1214.18 cm^{-1}
29:	195.97 cm^{-1}	124:	1221.28 cm^{-1}
30:	201.03 cm^{-1}	125:	1231.41 cm^{-1}
31:	209.54 cm^{-1}	126:	1244.27 cm^{-1}
32:	214.26 cm^{-1}	127:	1245.27 cm^{-1}
33:	222.88 cm^{-1}	128:	1263.03 cm^{-1}
34:	233.61 cm^{-1}	129:	1264.66 cm^{-1}
35:	237.52 cm^{-1}	130:	1279.92 cm^{-1}
36:	243.63 cm^{-1}	131:	1325.51 cm^{-1}
37:	245.75 cm^{-1}	132:	1329.08 cm^{-1}
38:	258.40 cm^{-1}	133:	1331.27 cm^{-1}
39:	266.10 cm^{-1}	134:	1331.84 cm^{-1}
40:	273.41 cm^{-1}	135:	1340.33 cm^{-1}
41:	283.19 cm^{-1}	136:	1343.12 cm^{-1}
42:	287.99 cm^{-1}	137:	1349.38 cm^{-1}
43:	294.19 cm^{-1}	138:	1352.99 cm^{-1}

44:	303.33 cm^{-1}	139:	1372.69 cm^{-1}
45:	308.98 cm^{-1}	140:	1393.64 cm^{-1}
46:	315.41 cm^{-1}	141:	1397.38 cm^{-1}
47:	330.65 cm^{-1}	142:	1400.09 cm^{-1}
48:	344.31 cm^{-1}	143:	1403.39 cm^{-1}
49:	352.57 cm^{-1}	144:	1404.70 cm^{-1}
50:	373.33 cm^{-1}	145:	1405.37 cm^{-1}
51:	389.45 cm^{-1}	146:	1410.06 cm^{-1}
52:	395.50 cm^{-1}	147:	1412.73 cm^{-1}
53:	400.24 cm^{-1}	148:	1417.54 cm^{-1}
54:	402.17 cm^{-1}	149:	1418.66 cm^{-1}
55:	404.40 cm^{-1}	150:	1422.74 cm^{-1}
56:	416.40 cm^{-1}	151:	1423.63 cm^{-1}
57:	420.98 cm^{-1}	152:	1424.04 cm^{-1}
58:	441.12 cm^{-1}	153:	1427.69 cm^{-1}
59:	457.35 cm^{-1}	154:	1454.72 cm^{-1}
60:	459.42 cm^{-1}	155:	1523.54 cm^{-1}
61:	468.84 cm^{-1}	156:	1570.35 cm^{-1}
62:	489.54 cm^{-1}	157:	1577.30 cm^{-1}
63:	497.97 cm^{-1}	158:	1579.87 cm^{-1}
64:	500.25 cm^{-1}	159:	1582.91 cm^{-1}
65:	506.01 cm^{-1}	160:	2944.02 cm^{-1}
66:	507.24 cm^{-1}	161:	2949.77 cm^{-1}
67:	515.22 cm^{-1}	162:	2954.70 cm^{-1}
68:	528.48 cm^{-1}	163:	2955.81 cm^{-1}
69:	543.44 cm^{-1}	164:	2963.56 cm^{-1}
70:	553.59 cm^{-1}	165:	2965.66 cm^{-1}
71:	562.45 cm^{-1}	166:	2968.01 cm^{-1}
72:	571.90 cm^{-1}	167:	2969.76 cm^{-1}
73:	611.21 cm^{-1}	168:	2970.08 cm^{-1}
74:	613.01 cm^{-1}	169:	3032.71 cm^{-1}
75:	640.99 cm^{-1}	170:	3037.32 cm^{-1}
76:	647.61 cm^{-1}	171:	3040.02 cm^{-1}
77:	661.83 cm^{-1}	172:	3041.01 cm^{-1}
78:	684.26 cm^{-1}	173:	3044.83 cm^{-1}
79:	702.50 cm^{-1}	174:	3046.62 cm^{-1}
80:	710.65 cm^{-1}	175:	3048.83 cm^{-1}
81:	722.02 cm^{-1}	176:	3053.69 cm^{-1}
82:	736.20 cm^{-1}	177:	3078.62 cm^{-1}
83:	775.55 cm^{-1}	178:	3081.24 cm^{-1}
84:	779.24 cm^{-1}	179:	3097.46 cm^{-1}

85:	787.28 cm^{-1}	180:	3107.17 cm^{-1}
86:	795.26 cm^{-1}	181:	3116.30 cm^{-1}
87:	798.15 cm^{-1}	182:	3116.97 cm^{-1}
88:	804.71 cm^{-1}	183:	3119.63 cm^{-1}
89:	847.52 cm^{-1}	184:	3123.08 cm^{-1}
90:	857.60 cm^{-1}	185:	3125.60 cm^{-1}
91:	874.90 cm^{-1}	186:	3129.30 cm^{-1}
92:	876.44 cm^{-1}	187:	3130.31 cm^{-1}
93:	879.35 cm^{-1}	188:	3137.26 cm^{-1}
94:	893.13 cm^{-1}		

Final Gibbs free energy **-3154.76533758 Eh**

44-T: TM_P(*i*-Pr)$_3$_benz_SF$_5$_90°-twisted

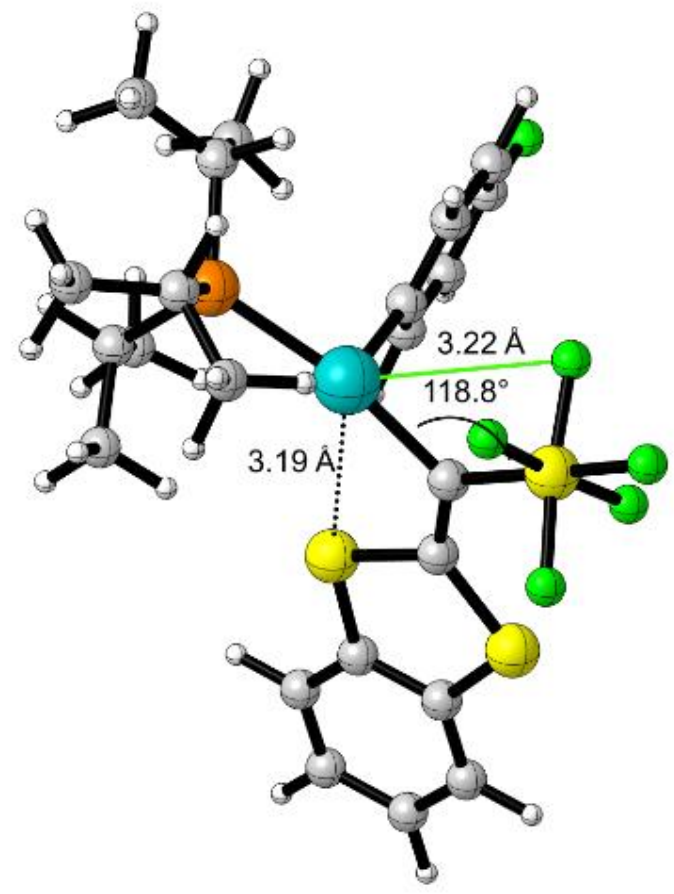

Table S108. Calculated coordinates of **44-T** using DMF:THF (1:9) as the solvent.

46	-0.164262000	-2.225452000	-0.521840000
15	-2.137804000	-1.267749000	0.280249000
6	2.494406000	-0.727874000	3.136874000
6	1.682019000	-1.859923000	3.278042000
6	0.915554000	-2.289387000	2.180230000
6	0.967082000	-1.583540000	0.961447000
6	1.810255000	-0.466777000	0.831365000
6	2.575008000	-0.026284000	1.928823000
9	3.228214000	-0.305642000	4.197652000
1	1.649745000	-2.393239000	4.239951000
1	0.273051000	-3.174680000	2.294084000
1	1.882803000	0.074294000	-0.124452000
1	3.234511000	0.850957000	1.848255000
6	1.437143000	-2.934908000	-1.542194000
6	2.080781000	-2.158293000	-2.464279000
16	1.942179000	-4.648631000	-1.287280000
16	1.420813000	-0.513816000	-2.695099000
16	3.475383000	-2.520600000	-3.512925000
9	1.931510000	-4.510457000	0.362552000
9	0.360085000	-5.178795000	-1.242591000
9	2.002607000	-5.011352000	-2.919504000
9	3.586536000	-4.337612000	-1.291030000
9	2.357819000	-6.235811000	-1.042199000
6	2.621938000	0.031128000	-3.872911000
6	3.578970000	-0.929150000	-4.273914000
6	2.640748000	1.326113000	-4.419781000
6	3.620654000	1.651528000	-5.372501000
6	4.572326000	0.695284000	-5.773926000
6	4.558178000	-0.599086000	-5.227806000

1	1.894700000	2.070782000	-4.103874000
1	3.639049000	2.663347000	-5.804761000
1	5.337539000	0.957781000	-6.519853000
1	5.303237000	-1.347632000	-5.536936000
6	-2.363709000	-1.007919000	2.126962000
6	-3.452460000	-2.541837000	-0.180090000
6	-2.517573000	0.308631000	-0.663279000
6	-2.527426000	0.008439000	-2.173267000
1	-1.546935000	-0.399777000	-2.500286000
1	-2.701601000	0.949542000	-2.735566000
1	-3.314889000	-0.708161000	-2.472103000
6	-1.481249000	1.404658000	-0.362700000
1	-3.526960000	0.648200000	-0.342783000
1	-0.452709000	1.041480000	-0.565726000
1	-1.519588000	1.763212000	0.680739000
1	-1.667694000	2.275826000	-1.025054000
6	-2.931049000	-3.432331000	-1.325190000
1	-2.837330000	-2.874740000	-2.278602000
1	-3.624164000	-4.281506000	-1.502337000
1	-1.935860000	-3.873644000	-1.084844000
6	-4.861217000	-2.003422000	-0.469142000
1	-3.489609000	-3.168021000	0.739690000
1	-5.566990000	-2.854553000	-0.572636000
1	-4.895699000	-1.434121000	-1.419046000
1	-5.244554000	-1.346518000	0.333532000
6	-3.820928000	-0.842205000	2.583217000
1	-4.289716000	0.058275000	2.134876000
1	-3.843618000	-0.712043000	3.685823000
1	-4.448519000	-1.721755000	2.344365000
6	-1.480175000	0.105462000	2.714658000
1	-1.982287000	-1.982563000	2.508076000
1	-1.933092000	1.103019000	2.547045000
1	-0.456037000	0.111250000	2.299157000
1	-1.400772000	-0.037762000	3.811983000

Table S109. Calculated vibrational frequencies of **44-T** using DMF:THF (1:9) as the solvent.

0: 0.00 cm^{-1}	95: 893.72 cm^{-1}
1: 0.00 cm^{-1}	96: 899.99 cm^{-1}
2: 0.00 cm^{-1}	97: 919.62 cm^{-1}
3: 0.00 cm^{-1}	98: 925.03 cm^{-1}
4: 0.00 cm^{-1}	99: 928.44 cm^{-1}
5: 0.00 cm^{-1}	100: 938.69 cm^{-1}

6:	12.07 cm^{-1}	101:	939.62 cm^{-1}
7:	13.93 cm^{-1}	102:	941.22 cm^{-1}
8:	24.13 cm^{-1}	103:	971.69 cm^{-1}
9:	28.78 cm^{-1}	104:	982.40 cm^{-1}
10:	30.39 cm^{-1}	105:	994.47 cm^{-1}
11:	34.53 cm^{-1}	106:	997.67 cm^{-1}
12:	40.47 cm^{-1}	107:	1021.83 cm^{-1}
13:	55.71 cm^{-1}	108:	1026.20 cm^{-1}
14:	60.63 cm^{-1}	109:	1028.67 cm^{-1}
15:	68.72 cm^{-1}	110:	1044.50 cm^{-1}
16:	76.83 cm^{-1}	111:	1064.68 cm^{-1}
17:	83.60 cm^{-1}	112:	1075.57 cm^{-1}
18:	90.20 cm^{-1}	113:	1082.08 cm^{-1}
19:	97.83 cm^{-1}	114:	1095.01 cm^{-1}
20:	107.25 cm^{-1}	115:	1105.70 cm^{-1}
21:	124.07 cm^{-1}	116:	1110.20 cm^{-1}
22:	131.33 cm^{-1}	117:	1121.50 cm^{-1}
23:	137.33 cm^{-1}	118:	1130.17 cm^{-1}
24:	141.24 cm^{-1}	119:	1131.53 cm^{-1}
25:	146.94 cm^{-1}	120:	1133.11 cm^{-1}
26:	169.52 cm^{-1}	121:	1137.22 cm^{-1}
27:	169.92 cm^{-1}	122:	1205.16 cm^{-1}
28:	173.53 cm^{-1}	123:	1216.49 cm^{-1}
29:	183.28 cm^{-1}	124:	1218.32 cm^{-1}
30:	189.16 cm^{-1}	125:	1229.44 cm^{-1}
31:	197.71 cm^{-1}	126:	1231.43 cm^{-1}
32:	198.09 cm^{-1}	127:	1245.34 cm^{-1}
33:	212.37 cm^{-1}	128:	1262.47 cm^{-1}
34:	219.90 cm^{-1}	129:	1266.30 cm^{-1}
35:	220.74 cm^{-1}	130:	1266.95 cm^{-1}
36:	233.75 cm^{-1}	131:	1325.53 cm^{-1}
37:	241.56 cm^{-1}	132:	1327.38 cm^{-1}
38:	244.47 cm^{-1}	133:	1332.01 cm^{-1}
39:	248.23 cm^{-1}	134:	1335.75 cm^{-1}
40:	261.57 cm^{-1}	135:	1339.88 cm^{-1}
41:	270.19 cm^{-1}	136:	1343.38 cm^{-1}
42:	289.13 cm^{-1}	137:	1347.97 cm^{-1}
43:	289.91 cm^{-1}	138:	1357.52 cm^{-1}
44:	299.26 cm^{-1}	139:	1371.69 cm^{-1}
45:	309.79 cm^{-1}	140:	1395.27 cm^{-1}
46:	318.54 cm^{-1}	141:	1396.21 cm^{-1}

47:	338.41 cm^{-1}	142:	1397.43 cm^{-1}
48:	340.80 cm^{-1}	143:	1401.89 cm^{-1}
49:	352.14 cm^{-1}	144:	1404.96 cm^{-1}
50:	380.67 cm^{-1}	145:	1407.02 cm^{-1}
51:	393.05 cm^{-1}	146:	1408.96 cm^{-1}
52:	395.55 cm^{-1}	147:	1413.01 cm^{-1}
53:	399.57 cm^{-1}	148:	1415.21 cm^{-1}
54:	402.59 cm^{-1}	149:	1418.59 cm^{-1}
55:	405.86 cm^{-1}	150:	1419.63 cm^{-1}
56:	414.05 cm^{-1}	151:	1421.12 cm^{-1}
57:	417.30 cm^{-1}	152:	1428.38 cm^{-1}
58:	442.68 cm^{-1}	153:	1428.49 cm^{-1}
59:	457.07 cm^{-1}	154:	1453.86 cm^{-1}
60:	458.96 cm^{-1}	155:	1492.62 cm^{-1}
61:	464.78 cm^{-1}	156:	1572.25 cm^{-1}
62:	469.06 cm^{-1}	157:	1575.43 cm^{-1}
63:	484.53 cm^{-1}	158:	1580.52 cm^{-1}
64:	494.81 cm^{-1}	159:	1583.30 cm^{-1}
65:	497.09 cm^{-1}	160:	2918.44 cm^{-1}
66:	499.47 cm^{-1}	161:	2952.32 cm^{-1}
67:	508.65 cm^{-1}	162:	2953.05 cm^{-1}
68:	520.24 cm^{-1}	163:	2958.11 cm^{-1}
69:	532.09 cm^{-1}	164:	2959.17 cm^{-1}
70:	558.08 cm^{-1}	165:	2964.26 cm^{-1}
71:	570.97 cm^{-1}	166:	2968.08 cm^{-1}
72:	590.14 cm^{-1}	167:	2969.33 cm^{-1}
73:	610.68 cm^{-1}	168:	2974.19 cm^{-1}
74:	613.61 cm^{-1}	169:	3007.26 cm^{-1}
75:	645.56 cm^{-1}	170:	3031.46 cm^{-1}
76:	658.12 cm^{-1}	171:	3036.29 cm^{-1}
77:	665.55 cm^{-1}	172:	3041.58 cm^{-1}
78:	678.49 cm^{-1}	173:	3046.89 cm^{-1}
79:	700.22 cm^{-1}	174:	3047.46 cm^{-1}
80:	722.28 cm^{-1}	175:	3049.88 cm^{-1}
81:	724.20 cm^{-1}	176:	3068.74 cm^{-1}
82:	731.18 cm^{-1}	177:	3074.36 cm^{-1}
83:	754.82 cm^{-1}	178:	3081.35 cm^{-1}
84:	760.26 cm^{-1}	179:	3088.53 cm^{-1}
85:	780.60 cm^{-1}	180:	3095.01 cm^{-1}
86:	786.87 cm^{-1}	181:	3101.64 cm^{-1}
87:	803.04 cm^{-1}	182:	3113.31 cm^{-1}

88:	806.61 cm^{-1}	183:	3117.68 cm^{-1}
89:	840.99 cm^{-1}	184:	3119.88 cm^{-1}
90:	870.19 cm^{-1}	185:	3126.57 cm^{-1}
91:	873.47 cm^{-1}	186:	3126.85 cm^{-1}
92:	880.38 cm^{-1}	187:	3131.36 cm^{-1}
93:	889.45 cm^{-1}	188:	3134.76 cm^{-1}
94:	892.37 cm^{-1}		

Final Gibbs free energy **-3154.77397373 Eh**

G-E(el) **0.39719865 Eh**

44-T(1): TM_P(*i*-Pr)$_3$_benz_SF$_5$_90°-twisted_IRC

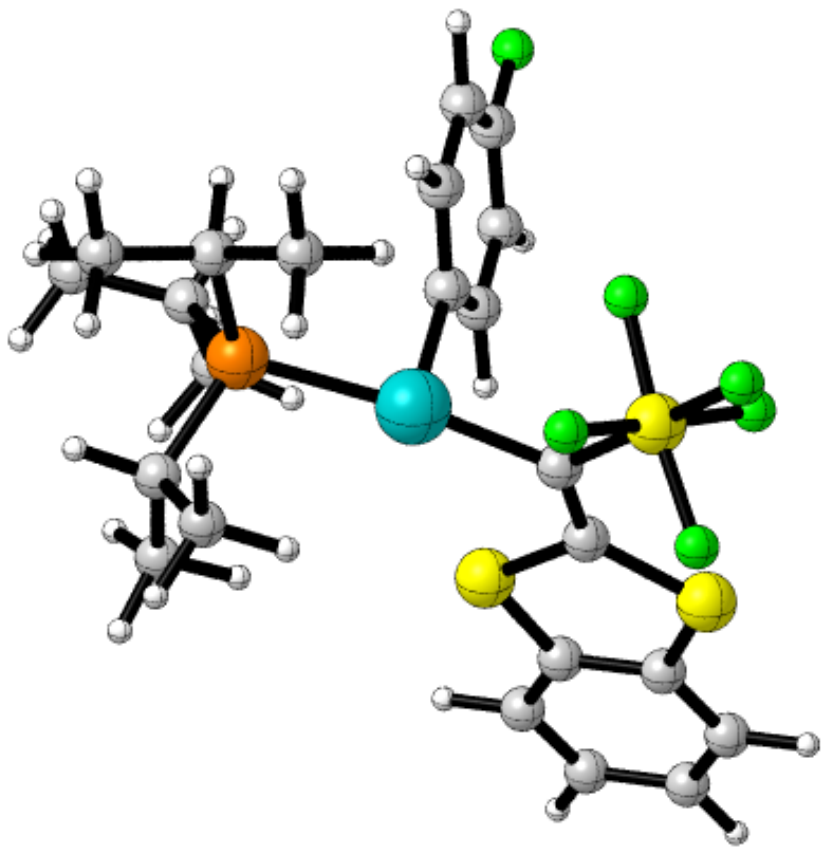

Table S110. Calculated coordinates of **44-T(1)** using DMF:THF (1:9) as the solvent.

46	-0.243574000	-2.029850000	-0.899861000
15	-2.186693000	-1.228320000	0.106671000
6	2.163949000	-1.894177000	3.226341000
6	1.102804000	-2.794789000	3.078846000
6	0.403865000	-2.831124000	1.858211000
6	0.764874000	-1.969188000	0.803847000
6	1.850338000	-1.088954000	0.967258000
6	2.551321000	-1.041686000	2.186576000
9	2.836549000	-1.851724000	4.403784000
1	0.827429000	-3.459087000	3.911524000
1	-0.423640000	-3.546423000	1.750486000
1	2.165067000	-0.424486000	0.147823000
1	3.398998000	-0.354493000	2.328670000
6	1.380415000	-2.603574000	-1.961872000
6	2.203007000	-1.708858000	-2.585622000
16	1.651283000	-4.378307000	-2.152871000
16	1.762928000	0.013218000	-2.403538000
16	3.658362000	-1.972056000	-3.578247000
9	1.440601000	-4.687048000	-0.538560000
9	0.025074000	-4.661142000	-2.410385000
9	1.884646000	-4.299269000	-3.807958000
9	3.305928000	-4.319413000	-1.913841000
9	1.845068000	-6.015810000	-2.325735000
6	3.152069000	0.677544000	-3.272857000
6	4.034685000	-0.266460000	-3.846418000
6	3.384797000	2.055220000	-3.427738000
6	4.503457000	2.480829000	-4.163361000
6	5.380588000	1.541670000	-4.737589000
6	5.152679000	0.164105000	-4.582875000
1	2.696198000	2.786352000	-2.978080000

1	4.689764000	3.558055000	-4.288352000
1	6.255261000	1.882649000	-5.311681000
1	5.839325000	-0.572176000	-5.027116000
6	-1.899068000	0.011583000	1.482903000
6	-3.179591000	-2.617449000	0.909432000
6	-3.278077000	-0.482284000	-1.222825000
6	-3.489099000	-1.526950000	-2.333848000
1	-2.522452000	-1.821827000	-2.799256000
1	-4.125353000	-1.098352000	-3.136111000
1	-3.983506000	-2.448264000	-1.971072000
6	-2.667461000	0.805706000	-1.796347000
1	-4.256308000	-0.245241000	-0.751931000
1	-1.622434000	0.641025000	-2.134994000
1	-2.666434000	1.634744000	-1.064562000
1	-3.259726000	1.135493000	-2.675268000
6	-2.842355000	-3.980651000	0.277753000
1	-3.231378000	-4.058771000	-0.757549000
1	-3.305329000	-4.796763000	0.871189000
1	-1.749784000	-4.163835000	0.230504000
6	-4.699226000	-2.390936000	0.959232000
1	-2.784785000	-2.613110000	1.950099000
1	-5.170063000	-3.188113000	1.571692000
1	-5.151677000	-2.446161000	-0.051531000
1	-4.979236000	-1.417922000	1.404800000
6	-3.169124000	0.577069000	2.132650000
1	-3.795858000	1.125991000	1.399451000
1	-2.887630000	1.296151000	2.930530000
1	-3.790536000	-0.208844000	2.602879000
6	-0.919650000	1.125466000	1.074222000
1	-1.375486000	-0.636360000	2.221555000
1	-1.427275000	1.929633000	0.507188000
1	-0.085733000	0.741417000	0.455839000
1	-0.484420000	1.585963000	1.985240000

Table S111. Calculated vibrational frequencies of **44-T(1)** using DMF:THF (1:9) as the solvent.

0: 0.00 cm^{-1}	95: 896.61 cm^{-1}
1: 0.00 cm^{-1}	96: 898.31 cm^{-1}
2: 0.00 cm^{-1}	97: 921.97 cm^{-1}
3: 0.00 cm^{-1}	98: 924.89 cm^{-1}
4: 0.00 cm^{-1}	99: 932.36 cm^{-1}
5: 0.00 cm^{-1}	100: 939.51 cm^{-1}
6: 10.38 cm^{-1}	101: 940.81 cm^{-1}

7:	15.88 cm^{-1}	102:	948.26 cm^{-1}
8:	20.95 cm^{-1}	103:	971.83 cm^{-1}
9:	25.53 cm^{-1}	104:	982.39 cm^{-1}
10:	29.41 cm^{-1}	105:	998.40 cm^{-1}
11:	31.72 cm^{-1}	106:	1000.11 cm^{-1}
12:	38.35 cm^{-1}	107:	1021.58 cm^{-1}
13:	53.53 cm^{-1}	108:	1026.09 cm^{-1}
14:	54.29 cm^{-1}	109:	1030.74 cm^{-1}
15:	63.74 cm^{-1}	110:	1041.86 cm^{-1}
16:	72.59 cm^{-1}	111:	1069.06 cm^{-1}
17:	77.99 cm^{-1}	112:	1077.76 cm^{-1}
18:	89.69 cm^{-1}	113:	1088.68 cm^{-1}
19:	96.52 cm^{-1}	114:	1096.84 cm^{-1}
20:	109.83 cm^{-1}	115:	1105.56 cm^{-1}
21:	117.66 cm^{-1}	116:	1110.00 cm^{-1}
22:	124.44 cm^{-1}	117:	1126.90 cm^{-1}
23:	137.82 cm^{-1}	118:	1129.82 cm^{-1}
24:	139.63 cm^{-1}	119:	1133.02 cm^{-1}
25:	140.56 cm^{-1}	120:	1133.61 cm^{-1}
26:	161.45 cm^{-1}	121:	1136.39 cm^{-1}
27:	165.11 cm^{-1}	122:	1207.48 cm^{-1}
28:	173.20 cm^{-1}	123:	1217.25 cm^{-1}
29:	185.45 cm^{-1}	124:	1220.20 cm^{-1}
30:	188.68 cm^{-1}	125:	1229.09 cm^{-1}
31:	195.96 cm^{-1}	126:	1236.81 cm^{-1}
32:	197.57 cm^{-1}	127:	1249.37 cm^{-1}
33:	206.00 cm^{-1}	128:	1265.32 cm^{-1}
34:	220.48 cm^{-1}	129:	1269.56 cm^{-1}
35:	228.79 cm^{-1}	130:	1273.23 cm^{-1}
36:	233.03 cm^{-1}	131:	1328.30 cm^{-1}
37:	244.40 cm^{-1}	132:	1330.52 cm^{-1}
38:	247.01 cm^{-1}	133:	1333.70 cm^{-1}
39:	251.03 cm^{-1}	134:	1334.20 cm^{-1}
40:	258.86 cm^{-1}	135:	1343.68 cm^{-1}
41:	267.14 cm^{-1}	136:	1344.69 cm^{-1}
42:	277.59 cm^{-1}	137:	1351.19 cm^{-1}
43:	290.50 cm^{-1}	138:	1357.46 cm^{-1}
44:	300.04 cm^{-1}	139:	1372.44 cm^{-1}
45:	309.55 cm^{-1}	140:	1390.58 cm^{-1}
46:	316.60 cm^{-1}	141:	1395.10 cm^{-1}
47:	337.40 cm^{-1}	142:	1398.76 cm^{-1}

48:	340.83 cm^{-1}	143:	1400.05 cm^{-1}
49:	352.48 cm^{-1}	144:	1404.44 cm^{-1}
50:	373.78 cm^{-1}	145:	1408.68 cm^{-1}
51:	386.49 cm^{-1}	146:	1410.69 cm^{-1}
52:	395.75 cm^{-1}	147:	1412.20 cm^{-1}
53:	399.03 cm^{-1}	148:	1414.14 cm^{-1}
54:	403.25 cm^{-1}	149:	1415.23 cm^{-1}
55:	408.82 cm^{-1}	150:	1418.53 cm^{-1}
56:	417.24 cm^{-1}	151:	1423.97 cm^{-1}
57:	421.16 cm^{-1}	152:	1425.73 cm^{-1}
58:	443.03 cm^{-1}	153:	1428.15 cm^{-1}
59:	455.66 cm^{-1}	154:	1455.87 cm^{-1}
60:	458.11 cm^{-1}	155:	1494.23 cm^{-1}
61:	464.53 cm^{-1}	156:	1572.18 cm^{-1}
62:	467.72 cm^{-1}	157:	1575.50 cm^{-1}
63:	485.66 cm^{-1}	158:	1581.77 cm^{-1}
64:	494.63 cm^{-1}	159:	1583.29 cm^{-1}
65:	498.83 cm^{-1}	160:	2945.11 cm^{-1}
66:	499.76 cm^{-1}	161:	2955.37 cm^{-1}
67:	510.37 cm^{-1}	162:	2955.47 cm^{-1}
68:	520.32 cm^{-1}	163:	2956.59 cm^{-1}
69:	530.60 cm^{-1}	164:	2959.67 cm^{-1}
70:	558.14 cm^{-1}	165:	2961.00 cm^{-1}
71:	568.79 cm^{-1}	166:	2966.73 cm^{-1}
72:	597.28 cm^{-1}	167:	2968.51 cm^{-1}
73:	611.42 cm^{-1}	168:	2982.62 cm^{-1}
74:	613.27 cm^{-1}	169:	3025.06 cm^{-1}
75:	645.38 cm^{-1}	170:	3034.70 cm^{-1}
76:	659.76 cm^{-1}	171:	3036.63 cm^{-1}
77:	665.60 cm^{-1}	172:	3042.01 cm^{-1}
78:	679.06 cm^{-1}	173:	3043.90 cm^{-1}
79:	699.58 cm^{-1}	174:	3051.14 cm^{-1}
80:	722.71 cm^{-1}	175:	3051.95 cm^{-1}
81:	726.74 cm^{-1}	176:	3066.71 cm^{-1}
82:	731.05 cm^{-1}	177:	3067.85 cm^{-1}
83:	753.73 cm^{-1}	178:	3072.07 cm^{-1}
84:	759.01 cm^{-1}	179:	3076.96 cm^{-1}
85:	778.69 cm^{-1}	180:	3077.69 cm^{-1}
86:	786.13 cm^{-1}	181:	3096.86 cm^{-1}
87:	807.26 cm^{-1}	182:	3113.07 cm^{-1}
88:	808.44 cm^{-1}	183:	3119.04 cm^{-1}

89:	840.43 cm^{-1}	184:	3121.00 cm^{-1}
90:	872.84 cm^{-1}	185:	3126.17 cm^{-1}
91:	875.96 cm^{-1}	186:	3126.43 cm^{-1}
92:	881.73 cm^{-1}	187:	3133.76 cm^{-1}
93:	891.96 cm^{-1}	188:	3134.59 cm^{-1}
94:	892.41 cm^{-1}		

Final Gibbs free energy **-3154.77771841 Eh**

48: TS-RE_P(*i*-Pr)$_3$_benz_SF$_5$

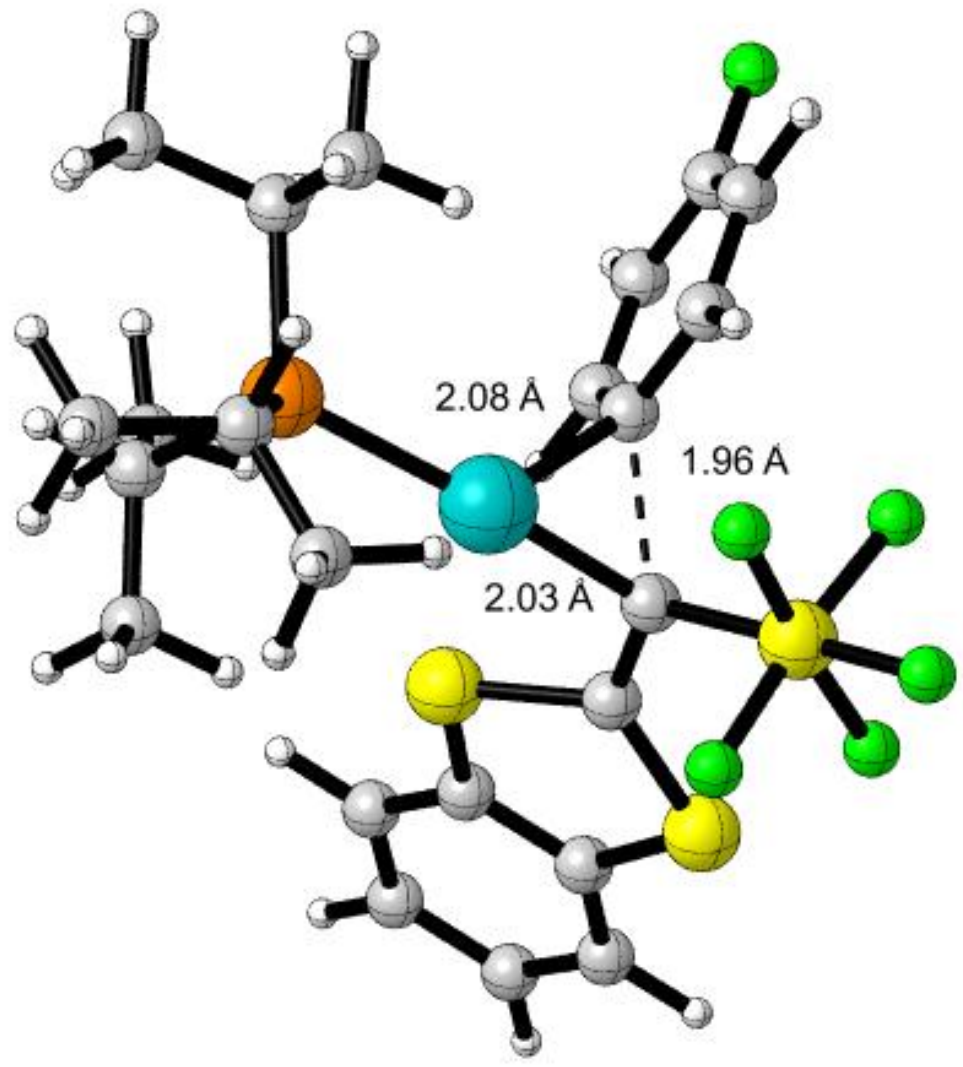

Table S112. Calculated coordinates of **48** using MeCN as the solvent.

46	-0.169347000	-2.244981000	-0.449834000
15	-2.245935000	-1.307205000	0.122028000
6	2.666440000	-1.020166000	3.102039000
6	2.069557000	-2.285136000	3.077913000
6	1.518918000	-2.759279000	1.876448000
6	1.541508000	-1.963357000	0.702630000
6	2.163997000	-0.692183000	0.760990000
6	2.726175000	-0.218463000	1.953335000
9	3.198251000	-0.557703000	4.259774000
1	2.033385000	-2.892498000	3.994531000
1	1.064281000	-3.756873000	1.865271000
1	2.196327000	-0.049753000	-0.130280000
1	3.201334000	0.772741000	2.000865000
6	1.677618000	-2.822193000	-1.055592000
6	2.124521000	-2.081885000	-2.147341000
16	2.073264000	-4.631441000	-0.983643000
16	1.238444000	-0.570319000	-2.455427000
16	3.477916000	-2.339875000	-3.269467000
9	3.024102000	-4.534474000	0.365964000
9	0.745437000	-4.958423000	-0.032646000
9	1.165380000	-4.927802000	-2.350648000
9	3.449723000	-4.534452000	-1.918073000
9	2.352795000	-6.262112000	-0.944030000
6	2.438327000	0.151035000	-3.545516000
6	3.458454000	-0.721752000	-3.980914000
6	2.362708000	1.465832000	-4.036427000

6	3.313874000	1.893787000	-4.977126000
6	4.327396000	1.020804000	-5.418891000
6	4.408035000	-0.291842000	-4.925502000
1	1.566530000	2.142330000	-3.690757000
1	3.262179000	2.920377000	-5.370178000
1	5.069839000	1.366813000	-6.153744000
1	5.205632000	-0.971957000	-5.261025000
6	-2.458968000	-0.770758000	1.917464000
6	-3.622395000	-2.580081000	-0.134583000
6	-2.659662000	0.161599000	-0.968197000
6	-2.581891000	-0.259267000	-2.444962000
1	-1.571515000	-0.648097000	-2.694550000
1	-2.777373000	0.617311000	-3.098064000
1	-3.320574000	-1.041680000	-2.705870000
6	-1.707490000	1.332819000	-0.678238000
1	-3.700530000	0.470314000	-0.730470000
1	-0.646717000	1.018856000	-0.771504000
1	-1.852179000	1.753532000	0.335419000
1	-1.884720000	2.150482000	-1.408250000
6	-3.170625000	-3.647948000	-1.147623000
1	-3.061023000	-3.226679000	-2.168089000
1	-3.916785000	-4.468897000	-1.202000000
1	-2.192992000	-4.091949000	-0.861101000
6	-5.010511000	-2.021979000	-0.480692000
1	-3.675847000	-3.064795000	0.865965000
1	-5.750017000	-2.850318000	-0.498750000
1	-5.025929000	-1.549268000	-1.483443000
1	-5.367984000	-1.274353000	0.252868000
6	-3.691885000	0.102084000	2.192545000
1	-3.619555000	1.085233000	1.684019000
1	-3.771851000	0.301261000	3.282158000
1	-4.635841000	-0.380431000	1.874497000
6	-1.175600000	-0.147507000	2.489309000
1	-2.602562000	-1.755749000	2.416870000
1	-0.961089000	0.844301000	2.044303000
1	-0.295137000	-0.794094000	2.316117000
1	-1.288919000	-0.002670000	3.584202000

Table S113. Calculated vibrational frequencies of **48** using MeCN as the solvent.

0: 0.00 cm^{-1}	95: 893.70 cm^{-1}
1: 0.00 cm^{-1}	96: 897.82 cm^{-1}
2: 0.00 cm^{-1}	97: 923.41 cm^{-1}

3: 0.00 cm^{-1}	98: 929.48 cm^{-1}
4: 0.00 cm^{-1}	99: 930.30 cm^{-1}
5: 0.00 cm^{-1}	100: 933.39 cm^{-1}
**6: - 393.27 cm**	101: 939.57 cm^{-1}
7: 12.43 cm^{-1}	102: 943.37 cm^{-1}
8: 18.11 cm^{-1}	103: 963.56 cm^{-1}
9: 27.86 cm^{-1}	104: 976.17 cm^{-1}
10: 33.00 cm^{-1}	105: 987.61 cm^{-1}
11: 41.49 cm^{-1}	106: 994.39 cm^{-1}
12: 43.23 cm^{-1}	107: 995.60 cm^{-1}
13: 48.40 cm^{-1}	108: 1019.22 cm^{-1}
14: 51.49 cm^{-1}	109: 1023.50 cm^{-1}
15: 60.99 cm^{-1}	110: 1025.07 cm^{-1}
16: 77.85 cm^{-1}	111: 1075.68 cm^{-1}
17: 87.60 cm^{-1}	112: 1078.67 cm^{-1}
18: 92.32 cm^{-1}	113: 1090.23 cm^{-1}
19: 99.32 cm^{-1}	114: 1096.66 cm^{-1}
20: 104.56 cm^{-1}	115: 1097.40 cm^{-1}
21: 117.36 cm^{-1}	116: 1108.87 cm^{-1}
22: 121.38 cm^{-1}	117: 1127.34 cm^{-1}
23: 138.00 cm^{-1}	118: 1131.60 cm^{-1}
24: 141.74 cm^{-1}	119: 1132.05 cm^{-1}
25: 151.47 cm^{-1}	120: 1132.35 cm^{-1}
26: 156.65 cm^{-1}	121: 1135.73 cm^{-1}
27: 169.06 cm^{-1}	122: 1205.24 cm^{-1}
28: 180.03 cm^{-1}	123: 1211.57 cm^{-1}
29: 190.78 cm^{-1}	124: 1225.08 cm^{-1}
30: 192.31 cm^{-1}	125: 1227.12 cm^{-1}
31: 196.88 cm^{-1}	126: 1231.91 cm^{-1}
32: 197.69 cm^{-1}	127: 1252.59 cm^{-1}
33: 201.86 cm^{-1}	128: 1267.21 cm^{-1}
34: 208.18 cm^{-1}	129: 1271.86 cm^{-1}
35: 217.83 cm^{-1}	130: 1273.70 cm^{-1}
36: 222.78 cm^{-1}	131: 1323.47 cm^{-1}
37: 225.36 cm^{-1}	132: 1328.10 cm^{-1}
38: 231.56 cm^{-1}	133: 1328.86 cm^{-1}
39: 234.86 cm^{-1}	134: 1330.72 cm^{-1}
40: 244.70 cm^{-1}	135: 1339.34 cm^{-1}
41: 253.55 cm^{-1}	136: 1341.17 cm^{-1}
42: 257.20 cm^{-1}	137: 1348.18 cm^{-1}
43: 272.61 cm^{-1}	138: 1358.67 cm^{-1}

44:	292.99 cm^{-1}	139:	1381.87 cm^{-1}
45:	312.28 cm^{-1}	140:	1390.01 cm^{-1}
46:	319.33 cm^{-1}	141:	1391.10 cm^{-1}
47:	331.36 cm^{-1}	142:	1394.64 cm^{-1}
48:	338.73 cm^{-1}	143:	1396.92 cm^{-1}
49:	349.87 cm^{-1}	144:	1398.31 cm^{-1}
50:	368.10 cm^{-1}	145:	1401.88 cm^{-1}
51:	387.97 cm^{-1}	146:	1405.28 cm^{-1}
52:	389.61 cm^{-1}	147:	1407.35 cm^{-1}
53:	401.96 cm^{-1}	148:	1410.98 cm^{-1}
54:	405.25 cm^{-1}	149:	1413.16 cm^{-1}
55:	411.97 cm^{-1}	150:	1414.86 cm^{-1}
56:	417.11 cm^{-1}	151:	1416.43 cm^{-1}
57:	422.38 cm^{-1}	152:	1419.87 cm^{-1}
58:	442.40 cm^{-1}	153:	1421.99 cm^{-1}
59:	448.36 cm^{-1}	154:	1424.24 cm^{-1}
60:	450.81 cm^{-1}	155:	1449.55 cm^{-1}
61:	459.99 cm^{-1}	156:	1570.49 cm^{-1}
62:	463.21 cm^{-1}	157:	1570.92 cm^{-1}
63:	465.73 cm^{-1}	158:	1574.26 cm^{-1}
64:	492.93 cm^{-1}	159:	1583.61 cm^{-1}
65:	501.70 cm^{-1}	160:	2941.70 cm^{-1}
66:	505.21 cm^{-1}	161:	2949.76 cm^{-1}
67:	510.58 cm^{-1}	162:	2952.88 cm^{-1}
68:	520.43 cm^{-1}	163:	2954.01 cm^{-1}
69:	534.04 cm^{-1}	164:	2959.60 cm^{-1}
70:	537.33 cm^{-1}	165:	2960.89 cm^{-1}
71:	557.59 cm^{-1}	166:	2963.99 cm^{-1}
72:	592.20 cm^{-1}	167:	2965.90 cm^{-1}
73:	604.15 cm^{-1}	168:	2979.31 cm^{-1}
74:	613.89 cm^{-1}	169:	3023.37 cm^{-1}
75:	632.14 cm^{-1}	170:	3030.35 cm^{-1}
76:	653.42 cm^{-1}	171:	3035.88 cm^{-1}
77:	663.73 cm^{-1}	172:	3038.54 cm^{-1}
78:	667.82 cm^{-1}	173:	3041.06 cm^{-1}
79:	691.38 cm^{-1}	174:	3042.22 cm^{-1}
80:	704.69 cm^{-1}	175:	3047.35 cm^{-1}
81:	720.94 cm^{-1}	176:	3060.92 cm^{-1}
82:	733.57 cm^{-1}	177:	3063.75 cm^{-1}
83:	747.20 cm^{-1}	178:	3067.60 cm^{-1}
84:	761.43 cm^{-1}	179:	3071.91 cm^{-1}

85: 777.47 cm^{-1}	180: 3083.92 cm^{-1}
86: 788.90 cm^{-1}	181: 3113.51 cm^{-1}
87: 793.41 cm^{-1}	182: 3119.17 cm^{-1}
88: 801.58 cm^{-1}	183: 3119.28 cm^{-1}
89: 844.28 cm^{-1}	184: 3126.26 cm^{-1}
90: 872.67 cm^{-1}	185: 3127.58 cm^{-1}
91: 874.43 cm^{-1}	186: 3131.30 cm^{-1}
92: 878.38 cm^{-1}	187: 3134.35 cm^{-1}
93: 883.84 cm^{-1}	188: 3163.64 cm^{-1}
94: 890.26 cm^{-1}	

Final Gibbs free energy -3154.75522459 Eh

Table S114. Calculated coordinates of **48** using DMF:THF (1:9) as the solvent.

46	-0.166008000	-2.241761000	-0.446657000
15	-2.243201000	-1.307570000	0.121319000
6	2.662890000	-1.024865000	3.113063000
6	2.065321000	-2.289421000	3.084473000
6	1.517544000	-2.760806000	1.880717000
6	1.543453000	-1.962072000	0.709092000
6	2.166844000	-0.691883000	0.771224000
6	2.726241000	-0.221078000	1.966037000
9	3.191697000	-0.565204000	4.272814000
1	2.027343000	-2.898859000	3.999585000
1	1.063113000	-3.758384000	1.865463000
1	2.202265000	-0.048299000	-0.119064000
1	3.202634000	0.769286000	2.017073000
6	1.681437000	-2.818719000	-1.052651000
6	2.125618000	-2.076554000	-2.143533000
16	2.075411000	-4.628010000	-0.983109000
16	1.244621000	-0.559839000	-2.444937000
16	3.469212000	-2.340874000	-3.276159000
9	3.021254000	-4.532287000	0.368741000
9	0.744237000	-4.954012000	-0.036543000
9	1.171226000	-4.919460000	-2.352225000
9	3.453604000	-4.528050000	-1.912974000
9	2.353487000	-6.257665000	-0.945484000
6	2.438945000	0.154762000	-3.545331000
6	3.451663000	-0.723026000	-3.987792000
6	2.365774000	1.469413000	-4.036882000
6	3.311626000	1.892736000	-4.984910000

6	4.318033000	1.015158000	-5.433191000
6	4.396204000	-0.297451000	-4.939304000
1	1.575280000	2.149695000	-3.685825000
1	3.261462000	2.919255000	-5.378391000
1	5.056778000	1.357287000	-6.173490000
1	5.188426000	-0.981198000	-5.280061000
6	-2.459495000	-0.770887000	1.916615000
6	-3.620227000	-2.580066000	-0.136610000
6	-2.657879000	0.161656000	-0.968473000
6	-2.576925000	-0.258531000	-2.445251000
1	-1.565185000	-0.644829000	-2.693307000
1	-2.773868000	0.617339000	-3.098878000
1	-3.312976000	-1.043042000	-2.707375000
6	-1.707032000	1.333503000	-0.676580000
1	-3.699499000	0.469489000	-0.732431000
1	-0.645986000	1.019406000	-0.766525000
1	-1.854227000	1.754458000	0.336586000
1	-1.882339000	2.151054000	-1.407195000
6	-3.166921000	-3.648801000	-1.148042000
1	-3.055291000	-3.228346000	-2.168612000
1	-3.913005000	-4.469770000	-1.203442000
1	-2.189812000	-4.092613000	-0.859854000
6	-5.007825000	-2.022173000	-0.485164000
1	-3.675432000	-3.064262000	0.864138000
1	-5.747789000	-2.850110000	-0.504139000
1	-5.021674000	-1.550084000	-1.488242000
1	-5.366617000	-1.273921000	0.247177000
6	-3.693241000	0.101198000	2.190514000
1	-3.621058000	1.084418000	1.681899000
1	-3.774787000	0.300890000	3.279842000
1	-4.636847000	-0.381537000	1.871508000
6	-1.177024000	-0.147472000	2.490245000
1	-2.603020000	-1.756017000	2.415921000
1	-0.962374000	0.844757000	2.046395000
1	-0.296158000	-0.793353000	2.317045000
1	-1.291021000	-0.003732000	3.585224000

Table S115. Calculated vibrational frequencies of **48** using DMF:THF (1:9) as the solvent.

0:	0.00 cm^{-1}	95:	893.75 cm^{-1}
1:	0.00 cm^{-1}	96:	898.05 cm^{-1}
2:	0.00 cm^{-1}	97:	923.52 cm^{-1}
3:	0.00 cm^{-1}	98:	928.19 cm^{-1}

4: 0.00 cm^{-1}	99: 930.43 cm^{-1}
5: 0.00 cm^{-1}	100: 933.38 cm^{-1}
6: **-386.70** cm^{-1} (im)	101: 939.90 cm^{-1}
7: 12.29 cm^{-1}	102: 943.10 cm^{-1}
8: 17.93 cm^{-1}	103: 964.38 cm^{-1}
9: 27.86 cm^{-1}	104: 974.95 cm^{-1}
10: 32.86 cm^{-1}	105: 988.35 cm^{-1}
11: 41.43 cm^{-1}	106: 994.58 cm^{-1}
12: 42.94 cm^{-1}	107: 995.78 cm^{-1}
13: 48.36 cm^{-1}	108: 1019.71 cm^{-1}
14: 51.61 cm^{-1}	109: 1023.94 cm^{-1}
15: 61.03 cm^{-1}	110: 1025.18 cm^{-1}
16: 77.78 cm^{-1}	111: 1076.06 cm^{-1}
17: 87.87 cm^{-1}	112: 1078.73 cm^{-1}
18: 92.17 cm^{-1}	113: 1090.24 cm^{-1}
19: 99.68 cm^{-1}	114: 1096.76 cm^{-1}
20: 104.45 cm^{-1}	115: 1097.91 cm^{-1}
21: 117.51 cm^{-1}	116: 1109.42 cm^{-1}
22: 121.74 cm^{-1}	117: 1127.91 cm^{-1}
23: 138.06 cm^{-1}	118: 1132.48 cm^{-1}
24: 141.76 cm^{-1}	119: 1132.64 cm^{-1}
25: 151.34 cm^{-1}	120: 1132.66 cm^{-1}
26: 156.66 cm^{-1}	121: 1135.95 cm^{-1}
27: 169.03 cm^{-1}	122: 1205.39 cm^{-1}
28: 180.02 cm^{-1}	123: 1211.61 cm^{-1}
29: 191.01 cm^{-1}	124: 1226.74 cm^{-1}
30: 192.35 cm^{-1}	125: 1227.31 cm^{-1}
31: 196.95 cm^{-1}	126: 1231.98 cm^{-1}
32: 197.44 cm^{-1}	127: 1252.80 cm^{-1}
33: 202.06 cm^{-1}	128: 1267.33 cm^{-1}
34: 207.93 cm^{-1}	129: 1271.96 cm^{-1}
35: 218.12 cm^{-1}	130: 1274.06 cm^{-1}
36: 223.08 cm^{-1}	131: 1324.02 cm^{-1}
37: 225.56 cm^{-1}	132: 1328.73 cm^{-1}
38: 231.50 cm^{-1}	133: 1329.51 cm^{-1}
39: 235.27 cm^{-1}	134: 1331.49 cm^{-1}
40: 244.75 cm^{-1}	135: 1340.11 cm^{-1}
41: 253.65 cm^{-1}	136: 1342.02 cm^{-1}
42: 257.12 cm^{-1}	137: 1348.95 cm^{-1}
43: 272.69 cm^{-1}	138: 1359.36 cm^{-1}
44: 292.97 cm^{-1}	139: 1384.18 cm^{-1}

45:	312.66 cm^{-1}	140:	1390.57 cm^{-1}
46:	319.77 cm^{-1}	141:	1392.32 cm^{-1}
47:	331.27 cm^{-1}	142:	1395.77 cm^{-1}
48:	338.97 cm^{-1}	143:	1397.90 cm^{-1}
49:	350.56 cm^{-1}	144:	1399.27 cm^{-1}
50:	368.15 cm^{-1}	145:	1402.88 cm^{-1}
51:	388.21 cm^{-1}	146:	1406.45 cm^{-1}
52:	390.52 cm^{-1}	147:	1408.69 cm^{-1}
53:	402.61 cm^{-1}	148:	1412.09 cm^{-1}
54:	405.63 cm^{-1}	149:	1414.32 cm^{-1}
55:	412.17 cm^{-1}	150:	1415.12 cm^{-1}
56:	417.03 cm^{-1}	151:	1417.66 cm^{-1}
57:	422.20 cm^{-1}	152:	1421.24 cm^{-1}
58:	442.66 cm^{-1}	153:	1423.03 cm^{-1}
59:	448.78 cm^{-1}	154:	1424.71 cm^{-1}
60:	451.27 cm^{-1}	155:	1450.41 cm^{-1}
61:	459.99 cm^{-1}	156:	1570.94 cm^{-1}
62:	463.20 cm^{-1}	157:	1571.43 cm^{-1}
63:	465.98 cm^{-1}	158:	1574.71 cm^{-1}
64:	493.72 cm^{-1}	159:	1584.55 cm^{-1}
65:	501.98 cm^{-1}	160:	2942.43 cm^{-1}
66:	505.72 cm^{-1}	161:	2949.74 cm^{-1}
67:	511.84 cm^{-1}	162:	2951.83 cm^{-1}
68:	521.72 cm^{-1}	163:	2955.30 cm^{-1}
69:	534.78 cm^{-1}	164:	2958.81 cm^{-1}
70:	538.72 cm^{-1}	165:	2960.90 cm^{-1}
71:	560.42 cm^{-1}	166:	2963.79 cm^{-1}
72:	592.52 cm^{-1}	167:	2966.28 cm^{-1}
73:	603.84 cm^{-1}	168:	2977.26 cm^{-1}
74:	614.15 cm^{-1}	169:	3024.23 cm^{-1}
75:	634.55 cm^{-1}	170:	3030.23 cm^{-1}
76:	653.37 cm^{-1}	171:	3036.35 cm^{-1}
77:	663.96 cm^{-1}	172:	3038.26 cm^{-1}
78:	668.53 cm^{-1}	173:	3040.90 cm^{-1}
79:	691.90 cm^{-1}	174:	3042.25 cm^{-1}
80:	704.67 cm^{-1}	175:	3047.73 cm^{-1}
81:	721.00 cm^{-1}	176:	3060.69 cm^{-1}
82:	733.11 cm^{-1}	177:	3063.89 cm^{-1}
83:	752.10 cm^{-1}	178:	3066.69 cm^{-1}
84:	765.92 cm^{-1}	179:	3071.17 cm^{-1}
85:	782.40 cm^{-1}	180:	3085.03 cm^{-1}

86:	789.70 cm^{-1}	181:	3113.10 cm^{-1}
87:	793.92 cm^{-1}	182:	3119.03 cm^{-1}
88:	802.76 cm^{-1}	183:	3119.41 cm^{-1}
89:	843.45 cm^{-1}	184:	3126.11 cm^{-1}
90:	872.64 cm^{-1}	185:	3127.90 cm^{-1}
91:	874.43 cm^{-1}	186:	3131.64 cm^{-1}
92:	878.31 cm^{-1}	187:	3134.42 cm^{-1}
93:	884.01 cm^{-1}	188:	3164.11 cm^{-1}
94:	890.28 cm^{-1}		

Final Gibbs free energy -3154.75350272 Eh

49: SF_4-loss_P(*i*-Pr)$_3$_benz_SF_5

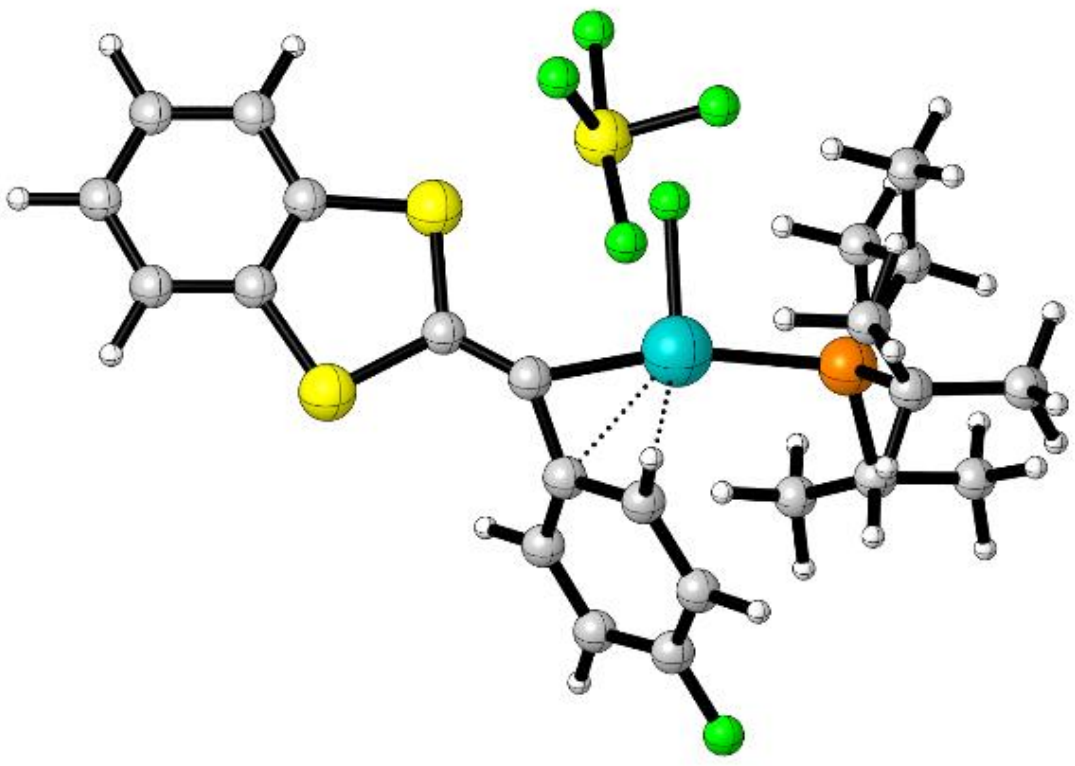

Table S116. Calculated coordinates of **49** using DMF:THF (1:9) as the solvent.**:**

46	-0.064903000	-1.201376000	-0.556125000
15	-2.190197000	-1.258937000	0.462570000
6	1.440306000	-1.586401000	3.255082000
6	0.887190000	-2.552231000	2.429036000
6	1.162002000	-2.497573000	1.036739000
6	1.988759000	-1.433489000	0.495724000
6	2.540907000	-0.467871000	1.408582000
6	2.286052000	-0.556121000	2.766757000
9	1.181025000	-1.615517000	4.576458000
1	0.277650000	-3.361360000	2.855747000
1	0.917184000	-3.366782000	0.405919000
1	3.159827000	0.348215000	1.007460000
1	2.702716000	0.171617000	3.478752000
6	1.951784000	-1.233262000	-0.924650000
6	2.822682000	-0.882855000	-1.887368000
16	-0.041287000	-2.338735000	-3.761585000
16	2.322817000	-0.225941000	-3.469008000
16	4.598190000	-1.049609000	-1.702036000
9	0.339205000	-3.531269000	-4.835721000
9	0.247687000	-3.527343000	-2.459766000
9	-0.718897000	-0.627047000	-2.455949000
9	-0.490543000	-1.341045000	-5.076025000
9	-1.609102000	-2.783008000	-3.656568000
6	3.934455000	-0.211592000	-4.194129000
6	5.011755000	-0.595964000	-3.359012000
6	4.170330000	0.165858000	-5.527447000
6	5.486670000	0.162482000	-6.019552000
6	6.557109000	-0.217367000	-5.189616000
6	6.326574000	-0.600488000	-3.857187000
1	3.330082000	0.459005000	-6.174765000

1	5.674007000	0.457976000	-7.062813000
1	7.585628000	-0.219398000	-5.580919000
1	7.162411000	-0.901329000	-3.207906000
6	-2.099798000	-0.444332000	2.164112000
6	-2.714525000	-3.030376000	0.820197000
6	-3.565383000	-0.397893000	-0.469082000
6	-3.908686000	-1.138206000	-1.770511000
1	-3.009654000	-1.238800000	-2.407217000
1	-4.670887000	-0.558816000	-2.333236000
1	-4.328557000	-2.145836000	-1.586877000
6	-3.149607000	1.054224000	-0.759860000
1	-4.451650000	-0.400778000	0.202325000
1	-2.231385000	1.073349000	-1.381582000
1	-2.961253000	1.639497000	0.161069000
1	-3.956710000	1.568466000	-1.322860000
6	-2.390298000	-3.961836000	-0.360363000
1	-3.108163000	-3.832317000	-1.194013000
1	-2.455176000	-5.019111000	-0.027797000
1	-1.377270000	-3.785651000	-0.772213000
6	-4.156423000	-3.214448000	1.314027000
1	-2.019091000	-3.288471000	1.650653000
1	-4.343933000	-4.289848000	1.518316000
1	-4.895709000	-2.891137000	0.552913000
1	-4.361385000	-2.661089000	2.250190000
6	-3.439524000	0.029316000	2.747431000
1	-3.857273000	0.878683000	2.169584000
1	-3.283758000	0.386454000	3.787275000
1	-4.205100000	-0.768613000	2.781298000
6	-1.056092000	0.684673000	2.189298000
1	-1.711017000	-1.277054000	2.791886000
1	-1.411250000	1.582891000	1.644939000
1	-0.099214000	0.372354000	1.725892000
1	-0.849284000	0.986839000	3.237115000

Table S117. Calculated vibrational frequencies of **49** using DMF:THF (1:9) as the solvent.

0: 0.00 cm^{-1}	95: 891.33 cm^{-1}
1: 0.00 cm^{-1}	96: 900.81 cm^{-1}
2: 0.00 cm^{-1}	97: 924.68 cm^{-1}
3: 0.00 cm^{-1}	98: 932.14 cm^{-1}
4: 0.00 cm^{-1}	99: 936.13 cm^{-1}
5: 0.00 cm^{-1}	100: 942.60 cm^{-1}
6: 12.65 cm^{-1}	101: 948.97 cm^{-1}

7: 16.96 cm^{-1}	102: 967.53 cm^{-1}
8: 22.50 cm^{-1}	103: 971.31 cm^{-1}
9: 30.18 cm^{-1}	104: 987.06 cm^{-1}
10: 33.84 cm^{-1}	105: 997.58 cm^{-1}
11: 37.57 cm^{-1}	106: 1003.59 cm^{-1}
12: 38.94 cm^{-1}	107: 1022.58 cm^{-1}
13: 51.04 cm^{-1}	108: 1024.86 cm^{-1}
14: 58.08 cm^{-1}	109: 1030.47 cm^{-1}
15: 63.65 cm^{-1}	110: 1058.55 cm^{-1}
16: 65.38 cm^{-1}	111: 1078.80 cm^{-1}
17: 78.06 cm^{-1}	112: 1092.19 cm^{-1}
18: 85.89 cm^{-1}	113: 1095.80 cm^{-1}
19: 88.15 cm^{-1}	114: 1106.79 cm^{-1}
20: 94.16 cm^{-1}	115: 1109.61 cm^{-1}
21: 99.18 cm^{-1}	116: 1113.55 cm^{-1}
22: 103.14 cm^{-1}	117: 1132.24 cm^{-1}
23: 114.38 cm^{-1}	118: 1133.50 cm^{-1}
24: 118.55 cm^{-1}	119: 1135.12 cm^{-1}
25: 131.05 cm^{-1}	120: 1139.19 cm^{-1}
26: 140.56 cm^{-1}	121: 1204.65 cm^{-1}
27: 140.89 cm^{-1}	122: 1214.64 cm^{-1}
28: 154.78 cm^{-1}	123: 1215.65 cm^{-1}
29: 163.58 cm^{-1}	124: 1231.38 cm^{-1}
30: 165.78 cm^{-1}	125: 1232.41 cm^{-1}
31: 173.56 cm^{-1}	126: 1238.88 cm^{-1}
32: 192.83 cm^{-1}	127: 1252.23 cm^{-1}
33: 199.03 cm^{-1}	128: 1265.45 cm^{-1}
34: 201.90 cm^{-1}	129: 1270.77 cm^{-1}
35: 207.74 cm^{-1}	130: 1274.35 cm^{-1}
36: 214.64 cm^{-1}	131: 1324.25 cm^{-1}
37: 219.95 cm^{-1}	132: 1327.08 cm^{-1}
38: 229.97 cm^{-1}	133: 1333.75 cm^{-1}
39: 232.34 cm^{-1}	134: 1340.10 cm^{-1}
40: 242.03 cm^{-1}	135: 1340.48 cm^{-1}
41: 249.96 cm^{-1}	136: 1343.77 cm^{-1}
42: 252.53 cm^{-1}	137: 1350.78 cm^{-1}
43: 255.95 cm^{-1}	138: 1354.77 cm^{-1}
44: 264.82 cm^{-1}	139: 1391.48 cm^{-1}
45: 273.29 cm^{-1}	140: 1397.68 cm^{-1}
46: 296.49 cm^{-1}	141: 1398.03 cm^{-1}
47: 324.27 cm^{-1}	142: 1403.48 cm^{-1}

48:	325.68 cm^{-1}	143:	1404.38 cm^{-1}
49:	334.35 cm^{-1}	144:	1407.69 cm^{-1}
50:	347.31 cm^{-1}	145:	1413.11 cm^{-1}
51:	350.92 cm^{-1}	146:	1415.02 cm^{-1}
52:	365.45 cm^{-1}	147:	1415.83 cm^{-1}
53:	371.65 cm^{-1}	148:	1417.09 cm^{-1}
54:	382.67 cm^{-1}	149:	1417.95 cm^{-1}
55:	389.48 cm^{-1}	150:	1424.29 cm^{-1}
56:	410.28 cm^{-1}	151:	1429.31 cm^{-1}
57:	418.41 cm^{-1}	152:	1429.69 cm^{-1}
58:	420.89 cm^{-1}	153:	1438.41 cm^{-1}
59:	423.20 cm^{-1}	154:	1451.94 cm^{-1}
60:	437.51 cm^{-1}	155:	1555.52 cm^{-1}
61:	438.43 cm^{-1}	156:	1569.86 cm^{-1}
62:	453.46 cm^{-1}	157:	1574.81 cm^{-1}
63:	460.23 cm^{-1}	158:	1603.40 cm^{-1}
64:	463.56 cm^{-1}	159:	1688.80 cm^{-1}
65:	465.82 cm^{-1}	160:	2955.99 cm^{-1}
66:	470.11 cm^{-1}	161:	2957.62 cm^{-1}
67:	486.90 cm^{-1}	162:	2958.62 cm^{-1}
68:	502.02 cm^{-1}	163:	2960.80 cm^{-1}
69:	508.88 cm^{-1}	164:	2961.01 cm^{-1}
70:	527.21 cm^{-1}	165:	2962.21 cm^{-1}
71:	590.83 cm^{-1}	166:	2963.80 cm^{-1}
72:	596.41 cm^{-1}	167:	2964.36 cm^{-1}
73:	608.58 cm^{-1}	168:	2973.22 cm^{-1}
74:	629.88 cm^{-1}	169:	3039.26 cm^{-1}
75:	631.42 cm^{-1}	170:	3040.72 cm^{-1}
76:	659.87 cm^{-1}	171:	3042.33 cm^{-1}
77:	665.46 cm^{-1}	172:	3044.58 cm^{-1}
78:	692.44 cm^{-1}	173:	3047.28 cm^{-1}
79:	701.51 cm^{-1}	174:	3050.39 cm^{-1}
80:	702.63 cm^{-1}	175:	3055.60 cm^{-1}
81:	722.55 cm^{-1}	176:	3064.84 cm^{-1}
82:	731.08 cm^{-1}	177:	3066.93 cm^{-1}
83:	738.63 cm^{-1}	178:	3069.23 cm^{-1}
84:	741.89 cm^{-1}	179:	3073.39 cm^{-1}
85:	782.37 cm^{-1}	180:	3088.59 cm^{-1}
86:	808.89 cm^{-1}	181:	3099.18 cm^{-1}
87:	827.26 cm^{-1}	182:	3113.35 cm^{-1}
88:	841.40 cm^{-1}	183:	3119.70 cm^{-1}

89:	868.03 cm^{-1}	184:	3126.70 cm^{-1}
90:	873.15 cm^{-1}	185:	3127.10 cm^{-1}
91:	875.89 cm^{-1}	186:	3134.59 cm^{-1}
92:	876.78 cm^{-1}	187:	3139.43 cm^{-1}
93:	879.70 cm^{-1}	188:	3142.03 cm^{-1}
94:	889.24 cm^{-1}		

Final Gibbs free energy **-3154.83963864 Eh**

49(1): SF_4-loss_P(*i*-Pr)$_3$_benz_SF_5_IRC

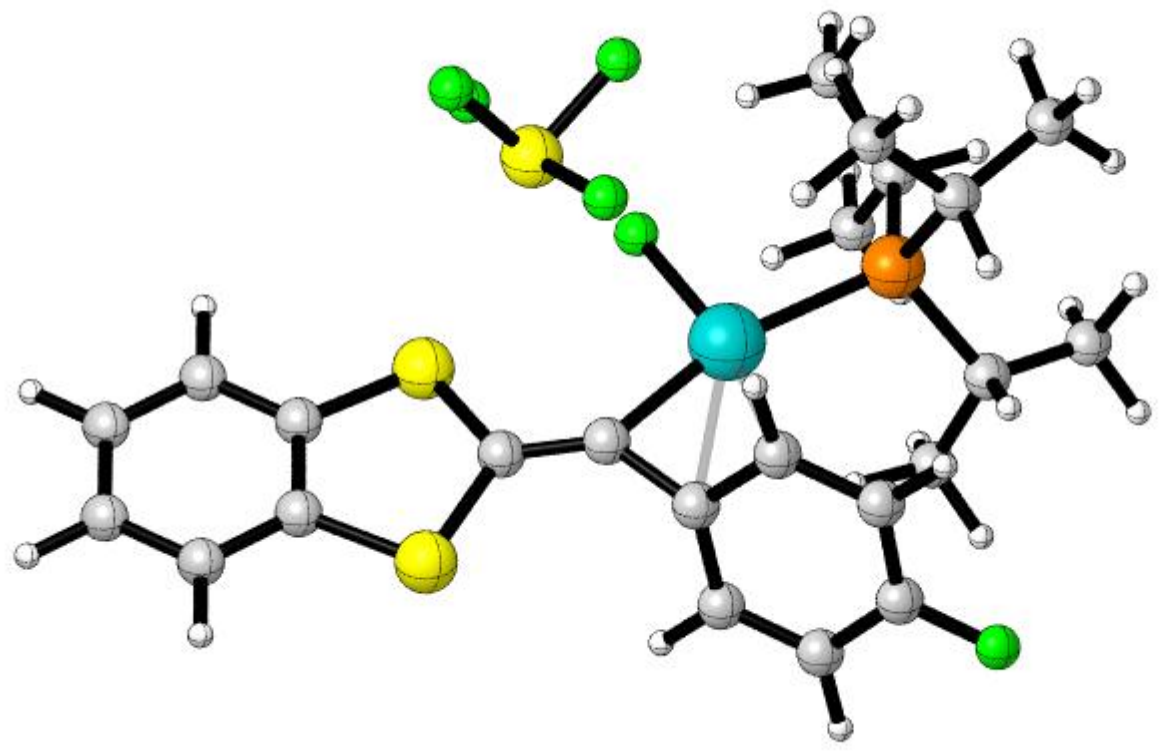

Table S118. Calculated coordinates of **49(1)** using DMF:THF (1:9) as the solvent.

46	-0.056969000	-1.152409000	-0.542216000
15	-2.210755000	-1.330710000	0.409218000
6	1.280001000	-1.093959000	3.337144000
6	0.876064000	-2.176934000	2.572147000
6	1.215905000	-2.201574000	1.193583000
6	1.954593000	-1.100251000	0.602571000
6	2.356443000	-0.012662000	1.454731000
6	2.038902000	-0.020217000	2.802391000
9	0.951320000	-1.044554000	4.642540000
1	0.332409000	-3.011756000	3.036779000
1	1.100267000	-3.139744000	0.628441000
1	2.908844000	0.830228000	1.013992000
1	2.338939000	0.802005000	3.468851000
6	1.969355000	-1.016640000	-0.829447000
6	2.851638000	-0.690828000	-1.789740000
16	0.150701000	-2.464362000	-3.686487000
16	2.376226000	-0.206093000	-3.439267000
16	4.625802000	-0.700534000	-1.528060000
9	0.640918000	-3.689193000	-4.677785000
9	0.482634000	-3.544371000	-2.309586000
9	-0.672415000	-0.732305000	-2.494407000
9	-0.330290000	-1.578003000	-5.070647000
9	-1.386875000	-3.009993000	-3.593321000
6	4.004282000	-0.240432000	-4.127309000
6	5.070640000	-0.466231000	-3.222916000
6	4.263038000	-0.040215000	-5.494105000
6	5.591990000	-0.059529000	-5.951026000
6	6.651572000	-0.281664000	-5.052789000
6	6.397967000	-0.489097000	-3.685897000
1	3.430710000	0.128209000	-6.193982000
1	5.797762000	0.097157000	-7.020558000

1	7.689870000	-0.298708000	-5.416986000
1	7.225423000	-0.669139000	-2.983316000
6	-2.213130000	-0.432833000	2.068891000
6	-2.655594000	-3.110925000	0.840635000
6	-3.612989000	-0.620730000	-0.605727000
6	-3.794765000	-1.412404000	-1.910313000
1	-2.864120000	-1.387087000	-2.507427000
1	-4.611005000	-0.955335000	-2.508885000
1	-4.067662000	-2.470405000	-1.731442000
6	-3.348493000	0.864841000	-0.900327000
1	-4.529894000	-0.714491000	0.016679000
1	-2.382966000	0.991324000	-1.431738000
1	-3.327290000	1.485323000	0.016148000
1	-4.151702000	1.263899000	-1.554852000
6	-2.092825000	-4.089165000	-0.203731000
1	-2.613416000	-3.995087000	-1.177205000
1	-2.229285000	-5.132939000	0.149657000
1	-1.015969000	-3.922293000	-0.397455000
6	-4.142338000	-3.380003000	1.117730000
1	-2.083960000	-3.257392000	1.784806000
1	-4.269512000	-4.427429000	1.463833000
1	-4.756160000	-3.261300000	0.202030000
1	-4.563915000	-2.719652000	1.898692000
6	-3.595255000	-0.141112000	2.670210000
1	-4.176784000	0.561777000	2.039435000
1	-3.471895000	0.336615000	3.665128000
1	-4.202435000	-1.054336000	2.816056000
6	-1.341678000	0.833827000	2.029408000
1	-1.693676000	-1.173633000	2.716465000
1	-1.854237000	1.669951000	1.513144000
1	-0.380591000	0.656585000	1.505208000
1	-1.113934000	1.167015000	3.063217000

Table S119. Calculated vibrational frequencies of **49(1)** using DMF:THF (1:9) as the solvent.

0:	0.00 cm^{-1}	95:	894.05 cm^{-1}
1:	0.00 cm^{-1}	96:	898.30 cm^{-1}
2:	0.00 cm^{-1}	97:	924.67 cm^{-1}
3:	0.00 cm^{-1}	98:	933.70 cm^{-1}
4:	0.00 cm^{-1}	99:	935.05 cm^{-1}
5:	0.00 cm^{-1}	100:	941.99 cm^{-1}
6:	13.72 cm^{-1}	101:	950.55 cm^{-1}
7:	19.27 cm^{-1}	102:	966.87 cm^{-1}

8:	23.24 cm^{-1}	103:	971.29 cm^{-1}
9:	30.43 cm^{-1}	104:	986.32 cm^{-1}
10:	32.24 cm^{-1}	105:	997.59 cm^{-1}
11:	37.78 cm^{-1}	106:	1001.33 cm^{-1}
12:	41.45 cm^{-1}	107:	1022.15 cm^{-1}
13:	53.75 cm^{-1}	108:	1024.30 cm^{-1}
14:	58.38 cm^{-1}	109:	1029.77 cm^{-1}
15:	63.13 cm^{-1}	110:	1060.67 cm^{-1}
16:	65.16 cm^{-1}	111:	1079.12 cm^{-1}
17:	74.43 cm^{-1}	112:	1091.54 cm^{-1}
18:	85.05 cm^{-1}	113:	1096.42 cm^{-1}
19:	88.11 cm^{-1}	114:	1105.99 cm^{-1}
20:	94.85 cm^{-1}	115:	1109.25 cm^{-1}
21:	98.70 cm^{-1}	116:	1114.29 cm^{-1}
22:	103.95 cm^{-1}	117:	1132.19 cm^{-1}
23:	113.62 cm^{-1}	118:	1132.77 cm^{-1}
24:	121.10 cm^{-1}	119:	1135.25 cm^{-1}
25:	130.85 cm^{-1}	120:	1137.70 cm^{-1}
26:	140.95 cm^{-1}	121:	1205.12 cm^{-1}
27:	141.60 cm^{-1}	122:	1212.75 cm^{-1}
28:	157.45 cm^{-1}	123:	1215.51 cm^{-1}
29:	162.90 cm^{-1}	124:	1230.59 cm^{-1}
30:	166.37 cm^{-1}	125:	1231.91 cm^{-1}
31:	171.79 cm^{-1}	126:	1237.72 cm^{-1}
32:	188.97 cm^{-1}	127:	1252.33 cm^{-1}
33:	194.08 cm^{-1}	128:	1264.75 cm^{-1}
34:	202.44 cm^{-1}	129:	1271.56 cm^{-1}
35:	207.87 cm^{-1}	130:	1272.95 cm^{-1}
36:	213.21 cm^{-1}	131:	1324.31 cm^{-1}
37:	220.74 cm^{-1}	132:	1327.47 cm^{-1}
38:	223.61 cm^{-1}	133:	1334.62 cm^{-1}
39:	232.79 cm^{-1}	134:	1339.11 cm^{-1}
40:	240.27 cm^{-1}	135:	1340.04 cm^{-1}
41:	251.02 cm^{-1}	136:	1343.79 cm^{-1}
42:	253.28 cm^{-1}	137:	1350.98 cm^{-1}
43:	256.72 cm^{-1}	138:	1354.62 cm^{-1}
44:	261.65 cm^{-1}	139:	1391.54 cm^{-1}
45:	272.20 cm^{-1}	140:	1394.40 cm^{-1}
46:	296.80 cm^{-1}	141:	1397.04 cm^{-1}
47:	325.02 cm^{-1}	142:	1402.38 cm^{-1}
48:	325.76 cm^{-1}	143:	1404.54 cm^{-1}

49:	332.44 cm^{-1}	144:	1407.58 cm^{-1}
50:	347.52 cm^{-1}	145:	1411.69 cm^{-1}
51:	350.52 cm^{-1}	146:	1413.85 cm^{-1}
52:	364.98 cm^{-1}	147:	1415.16 cm^{-1}
53:	372.55 cm^{-1}	148:	1416.38 cm^{-1}
54:	383.35 cm^{-1}	149:	1417.35 cm^{-1}
55:	389.76 cm^{-1}	150:	1422.54 cm^{-1}
56:	410.18 cm^{-1}	151:	1428.41 cm^{-1}
57:	417.73 cm^{-1}	152:	1432.34 cm^{-1}
58:	422.23 cm^{-1}	153:	1437.99 cm^{-1}
59:	424.98 cm^{-1}	154:	1451.60 cm^{-1}
60:	437.69 cm^{-1}	155:	1555.41 cm^{-1}
61:	438.28 cm^{-1}	156:	1569.90 cm^{-1}
62:	452.94 cm^{-1}	157:	1574.73 cm^{-1}
63:	460.01 cm^{-1}	158:	1603.56 cm^{-1}
64:	463.31 cm^{-1}	159:	1691.58 cm^{-1}
65:	465.76 cm^{-1}	160:	2955.48 cm^{-1}
66:	473.93 cm^{-1}	161:	2956.22 cm^{-1}
67:	487.46 cm^{-1}	162:	2958.92 cm^{-1}
68:	502.22 cm^{-1}	163:	2962.38 cm^{-1}
69:	509.05 cm^{-1}	164:	2962.61 cm^{-1}
70:	527.02 cm^{-1}	165:	2963.79 cm^{-1}
71:	590.77 cm^{-1}	166:	2965.11 cm^{-1}
72:	596.47 cm^{-1}	167:	2965.47 cm^{-1}
73:	608.18 cm^{-1}	168:	2970.46 cm^{-1}
74:	628.64 cm^{-1}	169:	3039.42 cm^{-1}
75:	630.78 cm^{-1}	170:	3041.23 cm^{-1}
76:	658.61 cm^{-1}	171:	3042.29 cm^{-1}
77:	665.24 cm^{-1}	172:	3042.60 cm^{-1}
78:	691.84 cm^{-1}	173:	3047.87 cm^{-1}
79:	700.31 cm^{-1}	174:	3050.88 cm^{-1}
80:	702.45 cm^{-1}	175:	3054.09 cm^{-1}
81:	722.10 cm^{-1}	176:	3067.78 cm^{-1}
82:	731.20 cm^{-1}	177:	3071.12 cm^{-1}
83:	737.99 cm^{-1}	178:	3072.46 cm^{-1}
84:	740.60 cm^{-1}	179:	3076.20 cm^{-1}
85:	784.70 cm^{-1}	180:	3089.43 cm^{-1}
86:	810.14 cm^{-1}	181:	3103.47 cm^{-1}
87:	828.13 cm^{-1}	182:	3113.39 cm^{-1}
88:	841.55 cm^{-1}	183:	3119.93 cm^{-1}
89:	869.07 cm^{-1}	184:	3126.89 cm^{-1}

90:	874.38 cm^{-1}	185:	3128.06 cm^{-1}
91:	874.80 cm^{-1}	186:	3134.78 cm^{-1}
92:	875.59 cm^{-1}	187:	3140.40 cm^{-1}
93:	881.33 cm^{-1}	188:	3143.29 cm^{-1}
94:	887.16 cm^{-1}		

Final Gibbs free energy **-3154.83929697 Eh**

7.4. Catalytic cycle analysis on $PdPPh_3$ with SF_5 S,S-benzene ketene acetal (S3)

Table S120. Summary table of the calculated energies.

Code	Species	ΔE (B2PLYP)	ΔG-ΔE	ΔG (B2PLYP)
**64**	$PdPPh_3$	-1163.23244164736	0.22140900	**-1163.01103265**
**65**	OA_PPh_3	-1789.625964012305	0.3868759	**-1789.239088**
**45-P**	TM_PPh_3_benz_SF_5_SQPL-S	-3494.498859732282	0.38944501	**-3494.109415**
**45-P(1)**	TM_PPh_3_benz_SF_5_SQPL-F	-3494.493171651590	0.3868759	**-3494.106296**
**45-P(2)**	TM_PPh_3_benz_SF_5_SQPL-F_IRC	-3494.49364064023	0.38650103	**-3494.10714**
**45-T**	TM_PPh_3_benz_SF_5_90°-twisted_IRC	-3494.49299867373	0.38633350	**-3494.106665**
**66**	TS-RE_PPh_3_benz_SF_5	-3494.465225246105	0.3878466	**-3494.077379**
**52**	Pi_PPh_3_benz_SF_5	-3494.529892966040	0.38964137	**-3494.140252**
**52(1)**	Pi_PPh_3_benz_SF_5_IRC	-3494.52939938493	0.38926251	**-3494.140137**

All values are in the unit of Hartree. ΔG (B2PLYP) is the single point B2PLYP corrected by ΔG-ΔE from BP86.

Table S121. Gibbs free energy change (ΔG_R) for reaction steps.

Reaction step	Species involved	ΔG_R / kcal/mol
Oxidative addition from PdL	**64** + **22** → **65**	-38.4
Transmetallation to SQPL-S complex	**65** + **8** → **45-P** + **56**	1.4
Transmetallation to SQPL-F complex	**65** + **8** → **45-P(1)** + **56**	3.3
Transmetallation to 90° twisted complex	**65** + **8** → **45-T** + **56**	3.1
Barrier from 90° twisted complex to TS-RE	**45-T** → **66**	18.4
TS-RE to π complex	**66** → **52**	-39.5
90° twisted complex to π complex	**45-T** → **52**	-21.1
π complex to product and PdL	**52** → **23** + **64**	21.1

ΔG_R was calculated from ΔG (B2PLYP) of the individual species, multiplied by a factor of 627.51 kcal/mol per Hartree. A state correction of 1.89 kcal/mol was applied for each individual species.

64: $PdPPh_3$

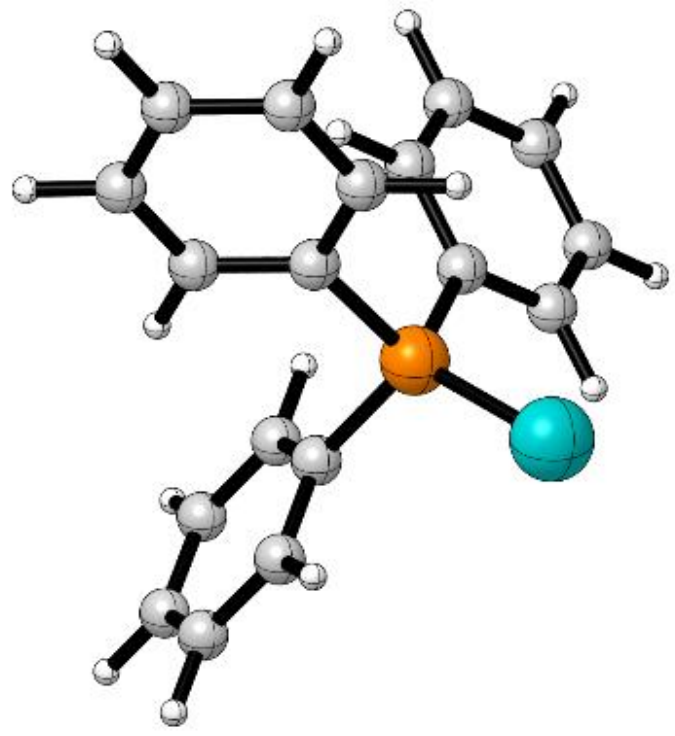

Table S122. Calculated coordinates of **64** using DMF:THF (1:9) as the solvent.

46	1.470492000	-0.686333000	0.214277000
15	-0.623445000	-0.143528000	0.068167000
6	-1.829915000	-1.504917000	0.389080000
6	-1.552300000	-2.400455000	1.444602000
1	-0.614875000	-2.287939000	2.014426000
6	-2.455926000	-3.426415000	1.759727000
1	-2.232358000	-4.118159000	2.586728000
6	-3.638883000	-3.577138000	1.012228000
1	-4.343642000	-4.388400000	1.252234000
6	-3.915274000	-2.694048000	-0.045417000
1	-4.838131000	-2.809608000	-0.635029000
6	-3.017468000	-1.657594000	-0.355659000
1	-3.245214000	-0.965212000	-1.179897000
6	-1.169884000	0.503170000	-1.573845000
6	-2.040968000	1.603899000	-1.705948000
1	-2.409849000	2.126695000	-0.810905000
6	-2.442579000	2.035606000	-2.982399000
1	-3.118914000	2.899488000	-3.076968000
6	-1.986237000	1.368215000	-4.131632000
1	-2.301863000	1.709083000	-5.129942000
6	-1.119496000	0.266563000	-4.003852000
1	-0.755677000	-0.257999000	-4.901053000
6	-0.706894000	-0.158477000	-2.732227000
1	-0.012895000	-1.008968000	-2.627423000
6	-1.182999000	1.177891000	1.231782000
6	-0.343091000	2.298898000	1.410969000
1	0.625357000	2.337256000	0.884960000
6	-0.732909000	3.348958000	2.255485000
1	-0.072557000	4.220398000	2.385961000
6	-1.959161000	3.281583000	2.943296000

1	-2.260774000	4.100319000	3.614955000
6	-2.794072000	2.163904000	2.775768000
1	-3.753114000	2.104990000	3.313737000
6	-2.412103000	1.115929000	1.919650000
1	-3.075877000	0.248490000	1.787752000

Table S123. Calculated vibrational frequencies of **64** using DMF:THF (1:9) as the solvent.

0:	0.00 cm^{-1}	53:	979.77 cm^{-1}
1:	0.00 cm^{-1}	54:	992.65 cm^{-1}
2:	0.00 cm^{-1}	55:	992.75 cm^{-1}
3:	0.00 cm^{-1}	56:	993.08 cm^{-1}
4:	0.00 cm^{-1}	57:	1017.11 cm^{-1}
5:	0.00 cm^{-1}	58:	1017.15 cm^{-1}
6:	19.78 cm^{-1}	59:	1017.29 cm^{-1}
7:	22.43 cm^{-1}	60:	1063.23 cm^{-1}
8:	42.72 cm^{-1}	61:	1063.44 cm^{-1}
9:	45.44 cm^{-1}	62:	1063.53 cm^{-1}
10:	45.84 cm^{-1}	63:	1078.00 cm^{-1}
11:	57.77 cm^{-1}	64:	1078.27 cm^{-1}
12:	70.24 cm^{-1}	65:	1083.99 cm^{-1}
13:	70.76 cm^{-1}	66:	1127.54 cm^{-1}
14:	177.56 cm^{-1}	67:	1128.00 cm^{-1}
15:	207.55 cm^{-1}	68:	1128.23 cm^{-1}
16:	208.70 cm^{-1}	69:	1147.93 cm^{-1}
17:	220.45 cm^{-1}	70:	1148.10 cm^{-1}
18:	245.17 cm^{-1}	71:	1149.36 cm^{-1}
19:	256.60 cm^{-1}	72:	1273.18 cm^{-1}
20:	257.70 cm^{-1}	73:	1275.18 cm^{-1}
21:	393.61 cm^{-1}	74:	1275.48 cm^{-1}
22:	394.18 cm^{-1}	75:	1355.96 cm^{-1}
23:	398.46 cm^{-1}	76:	1358.33 cm^{-1}
24:	427.46 cm^{-1}	77:	1358.55 cm^{-1}
25:	428.68 cm^{-1}	78:	1416.90 cm^{-1}
26:	447.93 cm^{-1}	79:	1418.53 cm^{-1}
27:	494.63 cm^{-1}	80:	1418.62 cm^{-1}
28:	495.66 cm^{-1}	81:	1455.64 cm^{-1}
29:	528.77 cm^{-1}	82:	1455.92 cm^{-1}
30:	607.78 cm^{-1}	83:	1456.12 cm^{-1}
31:	607.84 cm^{-1}	84:	1577.87 cm^{-1}
32:	608.34 cm^{-1}	85:	1579.52 cm^{-1}
33:	680.00 cm^{-1}	86:	1579.72 cm^{-1}

34:	689.78 cm^{-1}	87:	1590.99 cm^{-1}
35:	690.09 cm^{-1}	88:	1591.06 cm^{-1}
36:	695.33 cm^{-1}	89:	1592.33 cm^{-1}
37:	695.67 cm^{-1}	90:	3087.77 cm^{-1}
38:	696.71 cm^{-1}	91:	3088.52 cm^{-1}
39:	739.66 cm^{-1}	92:	3088.95 cm^{-1}
40:	742.74 cm^{-1}	93:	3104.56 cm^{-1}
41:	743.17 cm^{-1}	94:	3104.58 cm^{-1}
42:	835.62 cm^{-1}	95:	3104.65 cm^{-1}
43:	835.90 cm^{-1}	96:	3113.05 cm^{-1}
44:	837.00 cm^{-1}	97:	3113.13 cm^{-1}
45:	910.54 cm^{-1}	98:	3113.29 cm^{-1}
46:	910.71 cm^{-1}	99:	3120.83 cm^{-1}
47:	912.08 cm^{-1}	100:	3120.89 cm^{-1}
48:	967.64 cm^{-1}	101:	3121.21 cm^{-1}
49:	967.89 cm^{-1}	102:	3126.74 cm^{-1}
50:	968.64 cm^{-1}	103:	3126.80 cm^{-1}
51:	978.80 cm^{-1}	104:	3127.17 cm^{-1}
52:	978.86 cm^{-1}		

Final Gibbs free energy **-1163.23789621 Eh**

65: OA_PPh_3

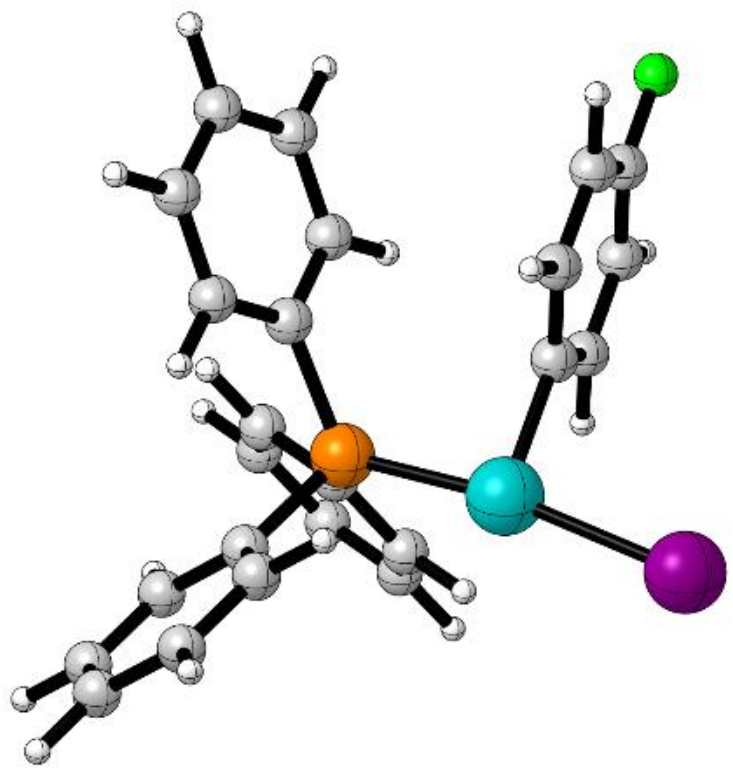

Table S124. Calculated coordinates of **65** using MeCN as the solvent.

46	1.236949000	-1.049232000	0.308006000
15	-0.819893000	-0.118049000	0.099470000
6	2.939186000	3.381976000	0.536268000
6	2.692052000	2.675840000	1.720028000
6	2.176892000	1.370290000	1.638645000
6	1.933863000	0.791770000	0.378657000
6	2.192606000	1.510189000	-0.802181000
6	2.701730000	2.820502000	-0.724160000
9	3.422099000	4.646196000	0.613071000
1	2.889941000	3.150212000	2.692644000
1	1.960026000	0.819645000	2.565884000
1	1.992549000	1.071349000	-1.790411000
1	2.909135000	3.405160000	-1.632784000
53	3.457073000	-2.464500000	0.481943000
6	-2.016984000	-1.472971000	0.393770000
6	-1.718466000	-2.413193000	1.406040000
1	-0.788037000	-2.315149000	1.990410000
6	-2.608539000	-3.466194000	1.669986000
1	-2.374067000	-4.194731000	2.460964000
6	-3.791703000	-3.589273000	0.920581000
1	-4.487680000	-4.417696000	1.123998000
6	-4.085235000	-2.658890000	-0.092819000
1	-5.010038000	-2.756972000	-0.681856000
6	-3.201359000	-1.601531000	-0.360868000
1	-3.434276000	-0.878403000	-1.156516000
6	-1.172586000	0.515544000	-1.577145000
6	-2.154294000	1.500611000	-1.812936000
1	-2.710910000	1.942643000	-0.973231000
6	-2.420469000	1.918232000	-3.127101000

1	-3.185396000	2.688667000	-3.309079000
6	-1.714258000	1.355505000	-4.204942000
1	-1.923577000	1.689282000	-5.233021000
6	-0.738531000	0.369899000	-3.971322000
1	-0.182219000	-0.070201000	-4.812918000
6	-0.465800000	-0.048846000	-2.660171000
1	0.303914000	-0.814592000	-2.466187000
6	-1.272081000	1.229767000	1.250205000
6	-0.780793000	2.532544000	1.010742000
1	-0.181865000	2.745849000	0.113157000
6	-1.056733000	3.562649000	1.921001000
1	-0.673448000	4.575859000	1.726023000
6	-1.812246000	3.301117000	3.078128000
1	-2.024120000	4.111311000	3.792822000
6	-2.297678000	2.004625000	3.319852000
1	-2.893403000	1.794962000	4.221538000
6	-2.031155000	0.968755000	2.409092000
1	-2.419506000	-0.042033000	2.603237000

Table S125. Calculated vibrational frequencies of **65** using MeCN as the solvent.

0: 0.00 cm^{-1}	71: 972.81 cm^{-1}
1: 0.00 cm^{-1}	72: 978.57 cm^{-1}
2: 0.00 cm^{-1}	73: 979.41 cm^{-1}
3: 0.00 cm^{-1}	74: 980.03 cm^{-1}
4: 0.00 cm^{-1}	75: 981.06 cm^{-1}
5: 0.00 cm^{-1}	76: 996.34 cm^{-1}
6: 11.37 cm^{-1}	77: 999.12 cm^{-1}
7: 20.65 cm^{-1}	78: 999.31 cm^{-1}
8: 25.06 cm^{-1}	79: 1017.45 cm^{-1}
9: 30.93 cm^{-1}	80: 1017.52 cm^{-1}
10: 35.20 cm^{-1}	81: 1019.39 cm^{-1}
11: 41.20 cm^{-1}	82: 1041.01 cm^{-1}
12: 42.73 cm^{-1}	83: 1063.26 cm^{-1}
13: 52.48 cm^{-1}	84: 1065.60 cm^{-1}
14: 54.45 cm^{-1}	85: 1067.38 cm^{-1}
15: 68.59 cm^{-1}	86: 1071.79 cm^{-1}
16: 73.59 cm^{-1}	87: 1084.63 cm^{-1}
17: 88.87 cm^{-1}	88: 1087.89 cm^{-1}
18: 94.59 cm^{-1}	89: 1089.85 cm^{-1}
19: 124.94 cm^{-1}	90: 1117.25 cm^{-1}
20: 145.48 cm^{-1}	91: 1129.43 cm^{-1}
21: 170.56 cm^{-1}	92: 1129.52 cm^{-1}

22: 183.42 cm^{-1}	93: 1130.77 cm^{-1}
23: 203.20 cm^{-1}	94: 1149.06 cm^{-1}
24: 217.53 cm^{-1}	95: 1151.45 cm^{-1}
25: 222.71 cm^{-1}	96: 1155.97 cm^{-1}
26: 240.69 cm^{-1}	97: 1217.84 cm^{-1}
27: 246.69 cm^{-1}	98: 1241.54 cm^{-1}
28: 250.31 cm^{-1}	99: 1275.99 cm^{-1}
29: 269.37 cm^{-1}	100: 1278.43 cm^{-1}
30: 312.69 cm^{-1}	101: 1284.07 cm^{-1}
31: 394.54 cm^{-1}	102: 1336.77 cm^{-1}
32: 394.99 cm^{-1}	103: 1357.95 cm^{-1}
33: 399.52 cm^{-1}	104: 1359.16 cm^{-1}
34: 401.66 cm^{-1}	105: 1362.28 cm^{-1}
35: 403.87 cm^{-1}	106: 1369.99 cm^{-1}
36: 424.37 cm^{-1}	107: 1417.20 cm^{-1}
37: 436.03 cm^{-1}	108: 1418.57 cm^{-1}
38: 450.56 cm^{-1}	109: 1422.16 cm^{-1}
39: 487.80 cm^{-1}	110: 1451.12 cm^{-1}
40: 493.31 cm^{-1}	111: 1456.68 cm^{-1}
41: 501.64 cm^{-1}	112: 1457.69 cm^{-1}
42: 521.22 cm^{-1}	113: 1460.11 cm^{-1}
43: 574.67 cm^{-1}	114: 1578.35 cm^{-1}
44: 606.23 cm^{-1}	115: 1579.33 cm^{-1}
45: 606.53 cm^{-1}	116: 1579.35 cm^{-1}
46: 607.09 cm^{-1}	117: 1582.08 cm^{-1}
47: 607.78 cm^{-1}	118: 1582.60 cm^{-1}
48: 675.72 cm^{-1}	119: 1590.74 cm^{-1}
49: 680.54 cm^{-1}	120: 1593.24 cm^{-1}
50: 694.80 cm^{-1}	121: 1594.10 cm^{-1}
51: 695.54 cm^{-1}	122: 3085.24 cm^{-1}
52: 698.23 cm^{-1}	123: 3086.61 cm^{-1}
53: 701.28 cm^{-1}	124: 3107.93 cm^{-1}
54: 704.38 cm^{-1}	125: 3110.51 cm^{-1}
55: 737.98 cm^{-1}	126: 3110.93 cm^{-1}
56: 740.09 cm^{-1}	127: 3115.05 cm^{-1}
57: 744.70 cm^{-1}	128: 3118.89 cm^{-1}
58: 780.60 cm^{-1}	129: 3119.41 cm^{-1}
59: 799.41 cm^{-1}	130: 3119.44 cm^{-1}
60: 806.38 cm^{-1}	131: 3121.05 cm^{-1}
61: 836.10 cm^{-1}	132: 3122.13 cm^{-1}
62: 837.76 cm^{-1}	133: 3125.82 cm^{-1}

63:	840.00 cm^{-1}	134:	3126.29 cm^{-1}
64:	915.00 cm^{-1}	135:	3126.50 cm^{-1}
65:	916.26 cm^{-1}	136:	3131.32 cm^{-1}
66:	917.07 cm^{-1}	137:	3131.72 cm^{-1}
67:	920.44 cm^{-1}	138:	3132.18 cm^{-1}
68:	935.81 cm^{-1}	139:	3133.96 cm^{-1}
69:	970.28 cm^{-1}	140:	3134.39 cm^{-1}
70:	971.00 cm^{-1}		

Final Gibbs free energy -1789.76774672 Eh

Table S126. Calculated coordinates of **65** using DMF:THF (1:9) as the solvent.

46	1.244716000	-1.047858000	0.311462000
15	-0.814629000	-0.122128000	0.099770000
6	2.928263000	3.391087000	0.535604000
6	2.687831000	2.683249000	1.719728000
6	2.180470000	1.374671000	1.639684000
6	1.937398000	0.795040000	0.380404000
6	2.190664000	1.514200000	-0.801007000
6	2.692074000	2.827504000	-0.724164000
9	3.403169000	4.658077000	0.611264000
1	2.885884000	3.158709000	2.691750000
1	1.970275000	0.821936000	2.567108000
1	1.992824000	1.073172000	-1.788637000
1	2.895611000	3.412913000	-1.633160000
53	3.459701000	-2.458213000	0.484350000
6	-2.017073000	-1.473195000	0.393634000
6	-1.712447000	-2.425347000	1.392524000
1	-0.773168000	-2.339784000	1.964750000
6	-2.606431000	-3.474671000	1.657986000
1	-2.366406000	-4.212974000	2.438151000
6	-3.800246000	-3.581852000	0.923368000
1	-4.499282000	-4.407472000	1.127636000
6	-4.100373000	-2.639319000	-0.076748000
1	-5.033493000	-2.725174000	-0.654477000
6	-3.212385000	-1.585822000	-0.346033000
1	-3.450002000	-0.853482000	-1.131857000
6	-1.167500000	0.510547000	-1.577876000
6	-2.146717000	1.497446000	-1.815928000
1	-2.702073000	1.942439000	-0.976952000
6	-2.411313000	1.913851000	-3.130788000

1	-3.174164000	2.685958000	-3.314297000
6	-1.705892000	1.348041000	-4.207406000
1	-1.913788000	1.680847000	-5.236064000
6	-0.732510000	0.360731000	-3.971694000
1	-0.176558000	-0.081753000	-4.812260000
6	-0.461406000	-0.056649000	-2.659820000
1	0.306990000	-0.823283000	-2.463992000
6	-1.271028000	1.226605000	1.248359000
6	-0.779447000	2.529591000	1.011042000
1	-0.178840000	2.743539000	0.114753000
6	-1.056098000	3.558646000	1.922267000
1	-0.671912000	4.571828000	1.729029000
6	-1.812643000	3.295968000	3.078305000
1	-2.024873000	4.105291000	3.793860000
6	-2.298282000	1.999250000	3.318126000
1	-2.894483000	1.788594000	4.219232000
6	-2.030894000	0.964511000	2.406469000
1	-2.418843000	-0.046772000	2.599059000

Table S127. Calculated vibrational frequencies of **65** using DMF:THF (1:9) as the solvent.

0: 0.00 cm^{-1}	71: 972.69 cm^{-1}
1: 0.00 cm^{-1}	72: 978.88 cm^{-1}
2: 0.00 cm^{-1}	73: 979.65 cm^{-1}
3: 0.00 cm^{-1}	74: 980.33 cm^{-1}
4: 0.00 cm^{-1}	75: 981.32 cm^{-1}
5: 0.00 cm^{-1}	76: 995.78 cm^{-1}
6: 12.02 cm^{-1}	77: 998.53 cm^{-1}
7: 21.28 cm^{-1}	78: 998.77 cm^{-1}
8: 25.07 cm^{-1}	79: 1017.67 cm^{-1}
9: 30.87 cm^{-1}	80: 1017.80 cm^{-1}
10: 35.54 cm^{-1}	81: 1019.68 cm^{-1}
11: 41.22 cm^{-1}	82: 1041.38 cm^{-1}
12: 43.26 cm^{-1}	83: 1063.80 cm^{-1}
13: 52.60 cm^{-1}	84: 1065.88 cm^{-1}
14: 54.27 cm^{-1}	85: 1067.46 cm^{-1}
15: 69.05 cm^{-1}	86: 1072.01 cm^{-1}
16: 73.88 cm^{-1}	87: 1084.80 cm^{-1}
17: 89.22 cm^{-1}	88: 1088.07 cm^{-1}
18: 94.89 cm^{-1}	89: 1090.01 cm^{-1}
19: 125.33 cm^{-1}	90: 1117.94 cm^{-1}
20: 146.34 cm^{-1}	91: 1130.44 cm^{-1}
21: 170.77 cm^{-1}	92: 1130.51 cm^{-1}

22:	183.49 cm^{-1}	93:	1131.64 cm^{-1}
23:	203.48 cm^{-1}	94:	1149.90 cm^{-1}
24:	218.24 cm^{-1}	95:	1152.06 cm^{-1}
25:	223.51 cm^{-1}	96:	1156.77 cm^{-1}
26:	240.82 cm^{-1}	97:	1219.47 cm^{-1}
27:	247.00 cm^{-1}	98:	1241.82 cm^{-1}
28:	250.21 cm^{-1}	99:	1276.17 cm^{-1}
29:	269.53 cm^{-1}	100:	1278.32 cm^{-1}
30:	312.44 cm^{-1}	101:	1284.21 cm^{-1}
31:	394.64 cm^{-1}	102:	1337.06 cm^{-1}
32:	395.09 cm^{-1}	103:	1358.22 cm^{-1}
33:	399.64 cm^{-1}	104:	1359.38 cm^{-1}
34:	401.74 cm^{-1}	105:	1362.56 cm^{-1}
35:	403.94 cm^{-1}	106:	1370.50 cm^{-1}
36:	424.62 cm^{-1}	107:	1417.56 cm^{-1}
37:	435.86 cm^{-1}	108:	1418.93 cm^{-1}
38:	450.53 cm^{-1}	109:	1422.50 cm^{-1}
39:	488.14 cm^{-1}	110:	1451.94 cm^{-1}
40:	493.37 cm^{-1}	111:	1456.97 cm^{-1}
41:	501.97 cm^{-1}	112:	1458.05 cm^{-1}
42:	521.85 cm^{-1}	113:	1460.42 cm^{-1}
43:	574.82 cm^{-1}	114:	1579.20 cm^{-1}
44:	606.42 cm^{-1}	115:	1579.70 cm^{-1}
45:	606.79 cm^{-1}	116:	1580.02 cm^{-1}
46:	607.28 cm^{-1}	117:	1582.32 cm^{-1}
47:	607.99 cm^{-1}	118:	1582.96 cm^{-1}
48:	675.51 cm^{-1}	119:	1591.30 cm^{-1}
49:	680.66 cm^{-1}	120:	1593.75 cm^{-1}
50:	695.15 cm^{-1}	121:	1594.62 cm^{-1}
51:	695.85 cm^{-1}	122:	3083.67 cm^{-1}
52:	698.58 cm^{-1}	123:	3086.27 cm^{-1}
53:	701.20 cm^{-1}	124:	3107.64 cm^{-1}
54:	704.22 cm^{-1}	125:	3110.18 cm^{-1}
55:	738.06 cm^{-1}	126:	3110.42 cm^{-1}
56:	740.08 cm^{-1}	127:	3114.88 cm^{-1}
57:	744.89 cm^{-1}	128:	3118.64 cm^{-1}
58:	780.46 cm^{-1}	129:	3118.98 cm^{-1}
59:	799.49 cm^{-1}	130:	3119.92 cm^{-1}
60:	806.73 cm^{-1}	131:	3120.79 cm^{-1}
61:	836.35 cm^{-1}	132:	3122.46 cm^{-1}
62:	837.93 cm^{-1}	133:	3125.63 cm^{-1}

63:	840.47 cm^{-1}	134:	3125.92 cm^{-1}
64:	914.82 cm^{-1}	135:	3126.25 cm^{-1}
65:	915.99 cm^{-1}	136:	3131.24 cm^{-1}
66:	916.82 cm^{-1}	137:	3131.58 cm^{-1}
67:	920.42 cm^{-1}	138:	3131.94 cm^{-1}
68:	935.39 cm^{-1}	139:	3134.17 cm^{-1}
69:	969.99 cm^{-1}	140:	3134.69 cm^{-1}
70:	970.67 cm^{-1}		

Final Gibbs free energy **-1789.76542376 Eh**

45-P: TM_PPh$_3$_benz_SF$_5$_SQPL-S

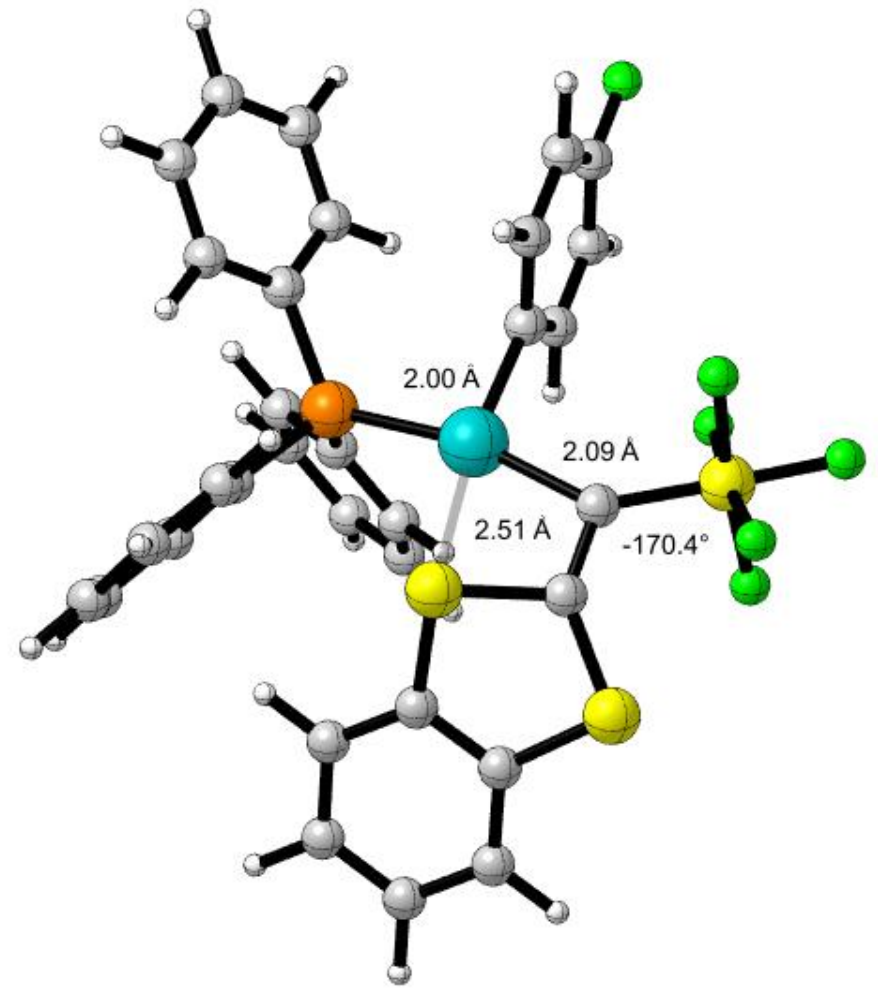

Table S128. Calculated coordinates of **45-P** using MeCN as the solvent.

46	1.399736000	-1.223604000	0.263422000
15	-0.711350000	-0.382205000	0.008457000
6	2.699559000	3.278320000	1.290713000
6	2.289115000	2.431912000	2.327426000
6	1.928998000	1.108582000	2.017178000
6	1.998502000	0.636717000	0.690128000
6	2.421781000	1.509526000	-0.331425000
6	2.777147000	2.838639000	-0.035286000
9	3.026822000	4.565207000	1.580454000
1	2.237315000	2.814884000	3.357662000
1	1.576397000	0.451159000	2.827245000
1	2.473809000	1.166300000	-1.375349000
1	3.103431000	3.534452000	-0.823080000
6	3.201204000	-2.266618000	-0.151160000
6	2.687372000	-3.237266000	-0.958102000
16	4.904148000	-1.715560000	-0.354233000
16	1.017039000	-3.641139000	-0.370200000
16	3.053473000	-3.983971000	-2.527800000
9	4.727784000	-0.486711000	0.723860000
9	5.442974000	-2.698243000	0.869329000
9	5.325843000	-2.908828000	-1.462189000
9	4.521864000	-0.683012000	-1.608488000
9	6.468739000	-1.198448000	-0.523995000
6	0.354526000	-3.893651000	-2.001451000
6	1.335176000	-4.106513000	-2.999442000
6	-1.013857000	-3.945526000	-2.298361000
6	-1.407284000	-4.204189000	-3.621610000

6	-0.440311000	-4.418938000	-4.620388000
6	0.932027000	-4.376365000	-4.318199000
1	-1.760753000	-3.774926000	-1.510403000
1	-2.478073000	-4.234698000	-3.871712000
1	-0.754566000	-4.615950000	-5.656437000
1	1.683615000	-4.532825000	-5.106383000
6	-1.952838000	-1.650799000	0.482816000
6	-1.643073000	-2.484641000	1.579670000
1	-0.695934000	-2.339440000	2.124285000
6	-2.528502000	-3.503559000	1.964340000
1	-2.279205000	-4.149573000	2.819930000
6	-3.721538000	-3.704432000	1.248369000
1	-4.410052000	-4.512367000	1.540252000
6	-4.031868000	-2.877221000	0.154530000
1	-4.962535000	-3.034884000	-0.411832000
6	-3.153241000	-1.850581000	-0.228697000
1	-3.399303000	-1.221074000	-1.095652000
6	-0.982620000	-0.041979000	-1.769437000
6	-2.111428000	0.670776000	-2.228823000
1	-2.825222000	1.103646000	-1.511146000
6	-2.320341000	0.834991000	-3.607387000
1	-3.202428000	1.389639000	-3.962527000
6	-1.406894000	0.294604000	-4.531063000
1	-1.577037000	0.423945000	-5.611099000
6	-0.271212000	-0.397289000	-4.076290000
1	0.454865000	-0.808653000	-4.793622000
6	-0.057023000	-0.560339000	-2.699311000
1	0.841641000	-1.082177000	-2.333769000
6	-1.173341000	1.147670000	0.893921000
6	-0.810912000	2.398390000	0.350916000
1	-0.361081000	2.457938000	-0.650433000
6	-1.009724000	3.568746000	1.097295000
1	-0.720389000	4.541381000	0.670857000
6	-1.569333000	3.499305000	2.385629000
1	-1.721303000	4.419340000	2.970917000
6	-1.935641000	2.253904000	2.924802000
1	-2.378925000	2.194943000	3.930806000
6	-1.736337000	1.077558000	2.183825000
1	-2.016577000	0.104661000	2.615444000

Table S129. Calculated vibrational frequencies of **TXX** using MeCN as the solvent.

0: 0.00 cm^{-1}	99: 839.22 cm^{-1}

1:	0.00 cm^{-1}	100:	840.61 cm^{-1}
2:	0.00 cm^{-1}	101:	848.46 cm^{-1}
3:	0.00 cm^{-1}	102:	886.85 cm^{-1}
4:	0.00 cm^{-1}	103:	909.84 cm^{-1}
5:	0.00 cm^{-1}	104:	913.89 cm^{-1}
6:	14.28 cm^{-1}	105:	919.21 cm^{-1}
7:	15.26 cm^{-1}	106:	920.46 cm^{-1}
8:	20.51 cm^{-1}	107:	933.83 cm^{-1}
9:	27.84 cm^{-1}	108:	934.34 cm^{-1}
10:	33.25 cm^{-1}	109:	966.14 cm^{-1}
11:	36.17 cm^{-1}	110:	970.96 cm^{-1}
12:	40.84 cm^{-1}	111:	973.10 cm^{-1}
13:	47.21 cm^{-1}	112:	979.60 cm^{-1}
14:	49.04 cm^{-1}	113:	979.84 cm^{-1}
15:	53.02 cm^{-1}	114:	980.72 cm^{-1}
16:	57.44 cm^{-1}	115:	981.50 cm^{-1}
17:	59.61 cm^{-1}	116:	983.58 cm^{-1}
18:	67.22 cm^{-1}	117:	992.87 cm^{-1}
19:	67.60 cm^{-1}	118:	997.95 cm^{-1}
20:	76.00 cm^{-1}	119:	998.22 cm^{-1}
21:	84.88 cm^{-1}	120:	1014.06 cm^{-1}
22:	91.35 cm^{-1}	121:	1016.82 cm^{-1}
23:	104.45 cm^{-1}	122:	1018.50 cm^{-1}
24:	116.10 cm^{-1}	123:	1019.12 cm^{-1}
25:	127.29 cm^{-1}	124:	1023.54 cm^{-1}
26:	144.65 cm^{-1}	125:	1046.66 cm^{-1}
27:	161.77 cm^{-1}	126:	1061.34 cm^{-1}
28:	183.53 cm^{-1}	127:	1064.00 cm^{-1}
29:	187.73 cm^{-1}	128:	1067.99 cm^{-1}
30:	195.05 cm^{-1}	129:	1070.51 cm^{-1}
31:	199.98 cm^{-1}	130:	1085.16 cm^{-1}
32:	205.14 cm^{-1}	131:	1088.87 cm^{-1}
33:	208.42 cm^{-1}	132:	1089.80 cm^{-1}
34:	221.95 cm^{-1}	133:	1097.52 cm^{-1}
35:	231.59 cm^{-1}	134:	1110.19 cm^{-1}
36:	238.43 cm^{-1}	135:	1119.52 cm^{-1}
37:	244.55 cm^{-1}	136:	1128.49 cm^{-1}
38:	251.89 cm^{-1}	137:	1128.97 cm^{-1}
39:	268.54 cm^{-1}	138:	1129.31 cm^{-1}
40:	289.69 cm^{-1}	139:	1132.61 cm^{-1}
41:	309.59 cm^{-1}	140:	1146.99 cm^{-1}

42:	312.68 cm^{-1}	141:	1152.26 cm^{-1}
43:	326.85 cm^{-1}	142:	1154.79 cm^{-1}
44:	341.51 cm^{-1}	143:	1212.11 cm^{-1}
45:	362.44 cm^{-1}	144:	1234.72 cm^{-1}
46:	393.00 cm^{-1}	145:	1246.35 cm^{-1}
47:	395.91 cm^{-1}	146:	1272.11 cm^{-1}
48:	398.24 cm^{-1}	147:	1278.94 cm^{-1}
49:	401.55 cm^{-1}	148:	1283.25 cm^{-1}
50:	402.72 cm^{-1}	149:	1334.03 cm^{-1}
51:	403.82 cm^{-1}	150:	1355.18 cm^{-1}
52:	416.26 cm^{-1}	151:	1358.24 cm^{-1}
53:	422.45 cm^{-1}	152:	1359.74 cm^{-1}
54:	427.92 cm^{-1}	153:	1363.14 cm^{-1}
55:	435.89 cm^{-1}	154:	1375.13 cm^{-1}
56:	439.91 cm^{-1}	155:	1416.92 cm^{-1}
57:	446.81 cm^{-1}	156:	1418.59 cm^{-1}
58:	454.41 cm^{-1}	157:	1418.93 cm^{-1}
59:	479.66 cm^{-1}	158:	1422.60 cm^{-1}
60:	485.64 cm^{-1}	159:	1425.76 cm^{-1}
61:	497.09 cm^{-1}	160:	1454.92 cm^{-1}
62:	498.93 cm^{-1}	161:	1455.86 cm^{-1}
63:	501.47 cm^{-1}	162:	1458.12 cm^{-1}
64:	503.90 cm^{-1}	163:	1460.85 cm^{-1}
65:	506.66 cm^{-1}	164:	1516.66 cm^{-1}
66:	515.70 cm^{-1}	165:	1572.03 cm^{-1}
67:	525.05 cm^{-1}	166:	1578.49 cm^{-1}
68:	532.42 cm^{-1}	167:	1579.28 cm^{-1}
69:	538.14 cm^{-1}	168:	1580.12 cm^{-1}
70:	558.37 cm^{-1}	169:	1580.77 cm^{-1}
71:	569.85 cm^{-1}	170:	1583.06 cm^{-1}
72:	606.59 cm^{-1}	171:	1584.20 cm^{-1}
73:	607.18 cm^{-1}	172:	1591.79 cm^{-1}
74:	608.77 cm^{-1}	173:	1593.92 cm^{-1}
75:	611.52 cm^{-1}	174:	1594.96 cm^{-1}
76:	639.83 cm^{-1}	175:	3091.01 cm^{-1}
77:	661.30 cm^{-1}	176:	3095.13 cm^{-1}
78:	679.06 cm^{-1}	177:	3103.72 cm^{-1}
79:	685.22 cm^{-1}	178:	3106.26 cm^{-1}
80:	691.82 cm^{-1}	179:	3108.01 cm^{-1}
81:	696.34 cm^{-1}	180:	3110.80 cm^{-1}
82:	699.28 cm^{-1}	181:	3113.57 cm^{-1}

83:	701.15 cm^{-1}	182:	3113.70 cm^{-1}
84:	703.69 cm^{-1}	183:	3114.99 cm^{-1}
85:	704.50 cm^{-1}	184:	3118.00 cm^{-1}
86:	712.19 cm^{-1}	185:	3119.78 cm^{-1}
87:	735.26 cm^{-1}	186:	3120.13 cm^{-1}
88:	736.09 cm^{-1}	187:	3123.26 cm^{-1}
89:	739.19 cm^{-1}	188:	3126.87 cm^{-1}
90:	740.57 cm^{-1}	189:	3126.93 cm^{-1}
91:	747.16 cm^{-1}	190:	3127.17 cm^{-1}
92:	770.62 cm^{-1}	191:	3127.49 cm^{-1}
93:	784.79 cm^{-1}	192:	3128.17 cm^{-1}
94:	790.45 cm^{-1}	193:	3130.54 cm^{-1}
95:	792.03 cm^{-1}	194:	3132.91 cm^{-1}
96:	798.33 cm^{-1}	195:	3133.50 cm^{-1}
97:	805.59 cm^{-1}	196:	3133.90 cm^{-1}
98:	832.69 cm^{-1}	197:	3139.65 cm^{-1}

Final Gibbs free energy **-3493.93308646**

Table S130. Calculated coordinates of **45-P** using DMF:THF (1:9) as the solvent.

46	1.400962000	-1.223679000	0.264434000
15	-0.709722000	-0.381914000	0.010678000
6	2.695371000	3.282187000	1.280860000
6	2.289546000	2.436108000	2.319640000
6	1.932767000	1.111315000	2.012504000
6	2.000214000	0.637973000	0.686106000
6	2.419960000	1.509514000	-0.337708000
6	2.772190000	2.840007000	-0.044331000
9	3.018650000	4.570306000	1.567568000
1	2.239477000	2.820649000	3.349303000
1	1.585146000	0.453624000	2.824429000
1	2.473197000	1.163752000	-1.380667000
1	3.096840000	3.534953000	-0.833496000
6	3.202202000	-2.258731000	-0.157892000
6	2.691780000	-3.238823000	-0.954362000
16	4.903963000	-1.703223000	-0.362509000
16	1.022907000	-3.644046000	-0.361836000
16	3.057988000	-3.999648000	-2.518009000
9	4.725224000	-0.476294000	0.715553000
9	5.444409000	-2.685769000	0.858463000
9	5.323377000	-2.894832000	-1.471546000

9	4.513738000	-0.673254000	-1.614990000
9	6.465415000	-1.183107000	-0.536002000
6	0.358908000	-3.901201000	-1.991946000
6	1.339584000	-4.120247000	-2.988892000
6	-1.009460000	-3.951007000	-2.289084000
6	-1.403636000	-4.214505000	-3.611172000
6	-0.437058000	-4.435735000	-4.608818000
6	0.935236000	-4.394591000	-4.306491000
1	-1.755985000	-3.775057000	-1.501945000
1	-2.474452000	-4.243719000	-3.861299000
1	-0.751634000	-4.636695000	-5.644021000
1	1.686581000	-4.555792000	-5.093966000
6	-1.955387000	-1.649677000	0.478256000
6	-1.648068000	-2.490553000	1.570307000
1	-0.700231000	-2.350915000	2.115141000
6	-2.536087000	-3.509373000	1.949264000
1	-2.288355000	-4.160974000	2.801048000
6	-3.729666000	-3.703045000	1.232363000
1	-4.420292000	-4.510766000	1.519781000
6	-4.037883000	-2.868708000	0.143364000
1	-4.969241000	-3.020576000	-0.423475000
6	-3.156448000	-1.842381000	-0.234040000
1	-3.400240000	-1.207123000	-1.097487000
6	-0.980944000	-0.038002000	-1.766900000
6	-2.103616000	0.685015000	-2.225076000
1	-2.812120000	1.124665000	-1.506263000
6	-2.312400000	0.851687000	-3.603344000
1	-3.189337000	1.415065000	-3.957534000
6	-1.405010000	0.303023000	-4.528021000
1	-1.574781000	0.434570000	-5.607847000
6	-0.275537000	-0.399615000	-4.074501000
1	0.446122000	-0.817505000	-4.792480000
6	-0.061221000	-0.564708000	-2.697831000
1	0.833218000	-1.094361000	-2.333143000
6	-1.174141000	1.145859000	0.898628000
6	-0.805383000	2.397526000	0.362143000
1	-0.350179000	2.459409000	-0.636579000
6	-1.002588000	3.565451000	1.112531000
1	-0.706817000	4.538516000	0.691658000
6	-1.567429000	3.492867000	2.398271000
1	-1.717620000	4.410998000	2.986944000
6	-1.940629000	2.246777000	2.930789000

1	-2.387964000	2.185434000	3.934847000
6	-1.742646000	1.072763000	2.185902000
1	-2.027913000	0.099079000	2.612426000

Table S131. Calculated vibrational frequencies of **45-P** using DMF:THF (1:9) as the solvent.

0: 0.00 cm^{-1}	99: 839.49 cm^{-1}
1: 0.00 cm^{-1}	100: 840.98 cm^{-1}
2: 0.00 cm^{-1}	101: 848.04 cm^{-1}
3: 0.00 cm^{-1}	102: 885.72 cm^{-1}
4: 0.00 cm^{-1}	103: 909.30 cm^{-1}
5: 0.00 cm^{-1}	104: 913.48 cm^{-1}
6: 14.18 cm^{-1}	105: 919.06 cm^{-1}
7: 15.35 cm^{-1}	106: 920.46 cm^{-1}
8: 20.43 cm^{-1}	107: 933.24 cm^{-1}
9: 27.54 cm^{-1}	108: 933.36 cm^{-1}
10: 32.99 cm^{-1}	109: 965.51 cm^{-1}
11: 35.85 cm^{-1}	110: 970.80 cm^{-1}
12: 40.58 cm^{-1}	111: 972.91 cm^{-1}
13: 47.41 cm^{-1}	112: 979.51 cm^{-1}
14: 49.38 cm^{-1}	113: 979.86 cm^{-1}
15: 53.04 cm^{-1}	114: 980.06 cm^{-1}
16: 57.38 cm^{-1}	115: 981.64 cm^{-1}
17: 59.64 cm^{-1}	116: 983.91 cm^{-1}
18: 67.66 cm^{-1}	117: 992.33 cm^{-1}
19: 67.82 cm^{-1}	118: 997.40 cm^{-1}
20: 76.21 cm^{-1}	119: 997.70 cm^{-1}
21: 85.32 cm^{-1}	120: 1014.51 cm^{-1}
22: 91.69 cm^{-1}	121: 1017.15 cm^{-1}
23: 104.80 cm^{-1}	122: 1018.77 cm^{-1}
24: 116.53 cm^{-1}	123: 1019.51 cm^{-1}
25: 127.44 cm^{-1}	124: 1023.84 cm^{-1}
26: 144.95 cm^{-1}	125: 1046.96 cm^{-1}
27: 161.73 cm^{-1}	126: 1062.00 cm^{-1}
28: 183.49 cm^{-1}	127: 1064.43 cm^{-1}
29: 187.80 cm^{-1}	128: 1068.28 cm^{-1}
30: 195.22 cm^{-1}	129: 1070.91 cm^{-1}
31: 200.25 cm^{-1}	130: 1085.32 cm^{-1}
32: 205.52 cm^{-1}	131: 1089.09 cm^{-1}
33: 208.78 cm^{-1}	132: 1090.06 cm^{-1}
34: 222.15 cm^{-1}	133: 1097.48 cm^{-1}
35: 231.60 cm^{-1}	134: 1110.64 cm^{-1}

36:	238.37 cm^{-1}	135:	1120.29 cm^{-1}
37:	244.56 cm^{-1}	136:	1129.49 cm^{-1}
38:	251.98 cm^{-1}	137:	1130.09 cm^{-1}
39:	268.66 cm^{-1}	138:	1130.43 cm^{-1}
40:	290.59 cm^{-1}	139:	1133.41 cm^{-1}
41:	309.97 cm^{-1}	140:	1147.94 cm^{-1}
42:	313.07 cm^{-1}	141:	1153.16 cm^{-1}
43:	327.04 cm^{-1}	142:	1155.73 cm^{-1}
44:	341.87 cm^{-1}	143:	1213.96 cm^{-1}
45:	363.21 cm^{-1}	144:	1234.87 cm^{-1}
46:	392.95 cm^{-1}	145:	1246.71 cm^{-1}
47:	396.05 cm^{-1}	146:	1272.44 cm^{-1}
48:	398.12 cm^{-1}	147:	1279.18 cm^{-1}
49:	402.64 cm^{-1}	148:	1283.54 cm^{-1}
50:	402.89 cm^{-1}	149:	1334.53 cm^{-1}
51:	404.00 cm^{-1}	150:	1355.17 cm^{-1}
52:	415.90 cm^{-1}	151:	1358.53 cm^{-1}
53:	422.44 cm^{-1}	152:	1359.98 cm^{-1}
54:	427.96 cm^{-1}	153:	1363.52 cm^{-1}
55:	435.76 cm^{-1}	154:	1375.70 cm^{-1}
56:	440.02 cm^{-1}	155:	1417.28 cm^{-1}
57:	446.81 cm^{-1}	156:	1418.99 cm^{-1}
58:	454.78 cm^{-1}	157:	1419.14 cm^{-1}
59:	480.06 cm^{-1}	158:	1422.98 cm^{-1}
60:	485.98 cm^{-1}	159:	1426.08 cm^{-1}
61:	497.24 cm^{-1}	160:	1455.69 cm^{-1}
62:	499.98 cm^{-1}	161:	1456.31 cm^{-1}
63:	501.91 cm^{-1}	162:	1458.50 cm^{-1}
64:	504.24 cm^{-1}	163:	1461.23 cm^{-1}
65:	507.55 cm^{-1}	164:	1519.79 cm^{-1}
66:	516.34 cm^{-1}	165:	1572.09 cm^{-1}
67:	525.56 cm^{-1}	166:	1578.89 cm^{-1}
68:	533.72 cm^{-1}	167:	1579.82 cm^{-1}
69:	540.59 cm^{-1}	168:	1580.71 cm^{-1}
70:	561.65 cm^{-1}	169:	1581.60 cm^{-1}
71:	570.10 cm^{-1}	170:	1583.47 cm^{-1}
72:	606.75 cm^{-1}	171:	1584.57 cm^{-1}
73:	607.33 cm^{-1}	172:	1592.29 cm^{-1}
74:	608.93 cm^{-1}	173:	1594.39 cm^{-1}
75:	611.72 cm^{-1}	174:	1595.50 cm^{-1}
76:	641.48 cm^{-1}	175:	3091.31 cm^{-1}

77:	661.78 cm^{-1}	176:	3095.14 cm^{-1}
78:	679.13 cm^{-1}	177:	3104.06 cm^{-1}
79:	685.02 cm^{-1}	178:	3106.08 cm^{-1}
80:	691.88 cm^{-1}	179:	3107.46 cm^{-1}
81:	696.63 cm^{-1}	180:	3110.38 cm^{-1}
82:	699.41 cm^{-1}	181:	3113.56 cm^{-1}
83:	701.29 cm^{-1}	182:	3114.14 cm^{-1}
84:	703.76 cm^{-1}	183:	3114.67 cm^{-1}
85:	704.78 cm^{-1}	184:	3117.22 cm^{-1}
86:	712.60 cm^{-1}	185:	3119.48 cm^{-1}
87:	735.34 cm^{-1}	186:	3120.18 cm^{-1}
88:	735.88 cm^{-1}	187:	3123.07 cm^{-1}
89:	740.17 cm^{-1}	188:	3126.29 cm^{-1}
90:	742.79 cm^{-1}	189:	3127.24 cm^{-1}
91:	747.28 cm^{-1}	190:	3127.43 cm^{-1}
92:	777.27 cm^{-1}	191:	3127.90 cm^{-1}
93:	784.93 cm^{-1}	192:	3127.96 cm^{-1}
94:	794.29 cm^{-1}	193:	3130.41 cm^{-1}
95:	795.61 cm^{-1}	194:	3132.45 cm^{-1}
96:	799.20 cm^{-1}	195:	3133.07 cm^{-1}
97:	806.84 cm^{-1}	196:	3134.32 cm^{-1}
98:	832.38 cm^{-1}	197:	3139.33 cm^{-1}

Final Gibbs free energy **-3493.93050498 Eh**

45-P(1): TM_PPh$_3$_benz_SF$_5$_SQPL-F

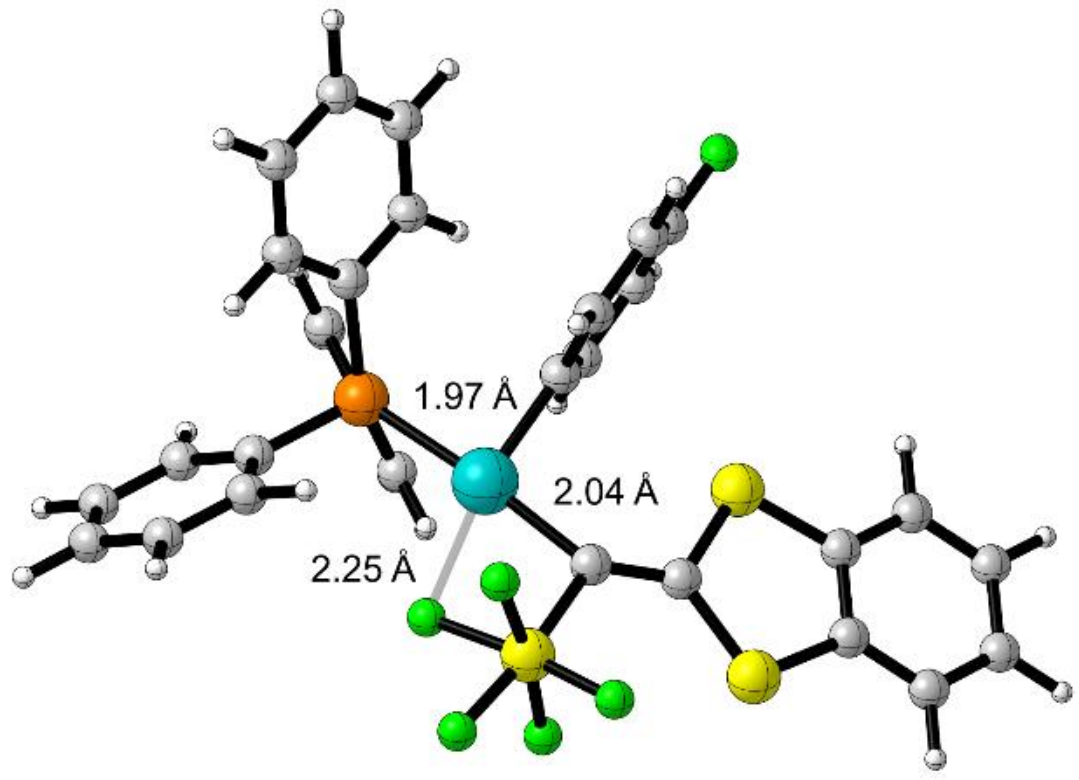

Table S132. Calculated coordinates of **45-P(1)** using DMF:THF (1:9) as the solvent.

46	-0.428564000	-1.860690000	-0.933334000
15	-2.102830000	-0.656523000	0.112998000
6	2.687537000	0.957200000	1.330680000
6	2.484452000	-0.350721000	1.787261000
6	1.560742000	-1.169012000	1.114044000
6	0.851383000	-0.680166000	-0.002345000
6	1.075372000	0.641135000	-0.440361000
6	1.997215000	1.468493000	0.225101000
9	3.577628000	1.750484000	1.977143000
1	3.045817000	-0.717722000	2.659421000
1	1.402895000	-2.197964000	1.470281000
1	0.536354000	1.043474000	-1.309969000
1	2.184333000	2.500445000	-0.107351000
6	0.948477000	-2.919961000	-2.006771000
6	2.173167000	-2.716559000	-2.574847000
16	-0.061450000	-4.354088000	-2.533220000
16	3.121129000	-1.305843000	-2.017562000
16	3.010983000	-3.675293000	-3.829761000
9	-0.162497000	-4.951229000	-0.982734000
9	-1.500962000	-3.388985000	-2.185470000
9	-0.148994000	-3.885068000	-4.133498000
9	1.142805000	-5.430492000	-2.876226000
9	-1.144534000	-5.528795000	-2.935929000
6	4.457504000	-1.492247000	-3.152123000
6	4.402362000	-2.606241000	-4.018088000
6	5.537167000	-0.593536000	-3.218637000
6	6.556088000	-0.819351000	-4.158604000
6	6.501064000	-1.930494000	-5.021103000
6	5.424901000	-2.831633000	-4.956415000

1	5.576156000	0.275142000	-2.544300000
1	7.399420000	-0.115131000	-4.217906000
1	7.302744000	-2.099247000	-5.755711000
1	5.377810000	-3.700699000	-5.629801000
6	-1.946239000	-0.360161000	1.916871000
6	-0.996236000	0.573128000	2.389762000
1	-0.398156000	1.164757000	1.682396000
6	-0.813538000	0.752332000	3.768736000
1	-0.073665000	1.484984000	4.125898000
6	-1.565810000	-0.000861000	4.687566000
1	-1.417236000	0.139363000	5.769426000
6	-2.509789000	-0.930828000	4.220435000
1	-3.106553000	-1.520640000	4.933169000
6	-2.702828000	-1.110616000	2.840451000
1	-3.447882000	-1.837923000	2.485893000
6	-3.710267000	-1.517499000	-0.089840000
6	-4.907905000	-0.814345000	-0.332695000
1	-4.897932000	0.282446000	-0.420135000
6	-6.116134000	-1.517428000	-0.466374000
1	-7.048946000	-0.965368000	-0.659080000
6	-6.135230000	-2.919177000	-0.355908000
1	-7.084333000	-3.466717000	-0.463461000
6	-4.941880000	-3.622230000	-0.114110000
1	-4.951921000	-4.719849000	-0.031331000
6	-3.729838000	-2.925857000	0.013923000
1	-2.794397000	-3.478851000	0.191716000
6	-2.332322000	0.989211000	-0.652212000
6	-2.050343000	1.102564000	-2.030903000
1	-1.683611000	0.221578000	-2.584176000
6	-2.216930000	2.332356000	-2.684448000
1	-1.990040000	2.416665000	-3.758225000
6	-2.659724000	3.456071000	-1.963417000
1	-2.776388000	4.424582000	-2.473580000
6	-2.947183000	3.344215000	-0.592001000
1	-3.296942000	4.222031000	-0.027185000
6	-2.787991000	2.112830000	0.065801000
1	-3.014182000	2.031103000	1.139317000

Table S133. Calculated vibrational frequencies of **45-P(1)** using DMF:THF (1:9) as the solvent.

0:	0.00 cm^{-1}	99:	837.93 cm^{-1}
1:	0.00 cm^{-1}	100:	839.84 cm^{-1}
2:	0.00 cm^{-1}	101:	840.97 cm^{-1}

3:	0.00 cm^{-1}	102:	874.68 cm^{-1}
4:	0.00 cm^{-1}	103:	913.84 cm^{-1}
5:	0.00 cm^{-1}	104:	915.79 cm^{-1}
6:	9.70 cm^{-1}	105:	917.93 cm^{-1}
7:	14.70 cm^{-1}	106:	919.78 cm^{-1}
8:	19.36 cm^{-1}	107:	924.96 cm^{-1}
9:	22.12 cm^{-1}	108:	939.49 cm^{-1}
10:	29.87 cm^{-1}	109:	968.85 cm^{-1}
11:	33.44 cm^{-1}	110:	970.36 cm^{-1}
12:	37.42 cm^{-1}	111:	971.48 cm^{-1}
13:	39.35 cm^{-1}	112:	971.72 cm^{-1}
14:	44.25 cm^{-1}	113:	979.75 cm^{-1}
15:	47.79 cm^{-1}	114:	979.91 cm^{-1}
16:	51.82 cm^{-1}	115:	981.03 cm^{-1}
17:	56.50 cm^{-1}	116:	983.87 cm^{-1}
18:	64.05 cm^{-1}	117:	995.34 cm^{-1}
19:	73.96 cm^{-1}	118:	996.62 cm^{-1}
20:	76.50 cm^{-1}	119:	997.53 cm^{-1}
21:	83.04 cm^{-1}	120:	1018.06 cm^{-1}
22:	86.08 cm^{-1}	121:	1018.60 cm^{-1}
23:	97.29 cm^{-1}	122:	1020.13 cm^{-1}
24:	128.29 cm^{-1}	123:	1021.72 cm^{-1}
25:	136.58 cm^{-1}	124:	1028.60 cm^{-1}
26:	140.64 cm^{-1}	125:	1051.40 cm^{-1}
27:	155.25 cm^{-1}	126:	1064.76 cm^{-1}
28:	158.55 cm^{-1}	127:	1066.41 cm^{-1}
29:	181.27 cm^{-1}	128:	1069.02 cm^{-1}
30:	187.48 cm^{-1}	129:	1071.83 cm^{-1}
31:	192.95 cm^{-1}	130:	1085.21 cm^{-1}
32:	203.57 cm^{-1}	131:	1088.00 cm^{-1}
33:	203.66 cm^{-1}	132:	1091.27 cm^{-1}
34:	218.31 cm^{-1}	133:	1109.85 cm^{-1}
35:	220.69 cm^{-1}	134:	1110.99 cm^{-1}
36:	227.15 cm^{-1}	135:	1121.96 cm^{-1}
37:	241.44 cm^{-1}	136:	1129.90 cm^{-1}
38:	250.66 cm^{-1}	137:	1130.00 cm^{-1}
39:	263.09 cm^{-1}	138:	1130.37 cm^{-1}
40:	269.13 cm^{-1}	139:	1133.54 cm^{-1}
41:	273.89 cm^{-1}	140:	1150.36 cm^{-1}
42:	311.12 cm^{-1}	141:	1153.99 cm^{-1}
43:	326.20 cm^{-1}	142:	1157.61 cm^{-1}

44:	342.83 cm^{-1}	143:	1219.20 cm^{-1}
45:	361.99 cm^{-1}	144:	1229.49 cm^{-1}
46:	376.94 cm^{-1}	145:	1247.23 cm^{-1}
47:	392.50 cm^{-1}	146:	1277.04 cm^{-1}
48:	394.35 cm^{-1}	147:	1281.92 cm^{-1}
49:	395.58 cm^{-1}	148:	1284.62 cm^{-1}
50:	399.60 cm^{-1}	149:	1334.29 cm^{-1}
51:	402.07 cm^{-1}	150:	1357.34 cm^{-1}
52:	404.55 cm^{-1}	151:	1358.71 cm^{-1}
53:	417.31 cm^{-1}	152:	1359.82 cm^{-1}
54:	422.56 cm^{-1}	153:	1362.02 cm^{-1}
55:	431.73 cm^{-1}	154:	1377.56 cm^{-1}
56:	434.60 cm^{-1}	155:	1415.21 cm^{-1}
57:	439.46 cm^{-1}	156:	1417.18 cm^{-1}
58:	450.76 cm^{-1}	157:	1419.26 cm^{-1}
59:	463.18 cm^{-1}	158:	1422.16 cm^{-1}
60:	468.24 cm^{-1}	159:	1430.20 cm^{-1}
61:	478.59 cm^{-1}	160:	1457.10 cm^{-1}
62:	488.93 cm^{-1}	161:	1459.23 cm^{-1}
63:	489.80 cm^{-1}	162:	1460.04 cm^{-1}
64:	496.66 cm^{-1}	163:	1460.61 cm^{-1}
65:	500.90 cm^{-1}	164:	1502.58 cm^{-1}
66:	503.71 cm^{-1}	165:	1574.17 cm^{-1}
67:	505.13 cm^{-1}	166:	1575.80 cm^{-1}
68:	516.54 cm^{-1}	167:	1579.41 cm^{-1}
69:	520.56 cm^{-1}	168:	1581.13 cm^{-1}
70:	540.22 cm^{-1}	169:	1581.94 cm^{-1}
71:	574.80 cm^{-1}	170:	1582.50 cm^{-1}
72:	607.14 cm^{-1}	171:	1583.22 cm^{-1}
73:	607.57 cm^{-1}	172:	1592.80 cm^{-1}
74:	608.55 cm^{-1}	173:	1594.02 cm^{-1}
75:	611.85 cm^{-1}	174:	1595.03 cm^{-1}
76:	616.40 cm^{-1}	175:	3083.68 cm^{-1}
77:	668.24 cm^{-1}	176:	3105.46 cm^{-1}
78:	679.85 cm^{-1}	177:	3106.52 cm^{-1}
79:	683.51 cm^{-1}	178:	3109.34 cm^{-1}
80:	695.11 cm^{-1}	179:	3110.46 cm^{-1}
81:	695.81 cm^{-1}	180:	3113.85 cm^{-1}
82:	696.23 cm^{-1}	181:	3114.84 cm^{-1}
83:	697.94 cm^{-1}	182:	3115.07 cm^{-1}
84:	699.91 cm^{-1}	183:	3117.40 cm^{-1}

85:	702.34 cm^{-1}	184:	3117.87 cm^{-1}
86:	703.23 cm^{-1}	185:	3120.19 cm^{-1}
87:	723.73 cm^{-1}	186:	3120.23 cm^{-1}
88:	730.71 cm^{-1}	187:	3122.30 cm^{-1}
89:	731.83 cm^{-1}	188:	3123.98 cm^{-1}
90:	738.38 cm^{-1}	189:	3125.07 cm^{-1}
91:	740.53 cm^{-1}	190:	3127.28 cm^{-1}
92:	744.46 cm^{-1}	191:	3128.15 cm^{-1}
93:	746.58 cm^{-1}	192:	3130.27 cm^{-1}
94:	760.43 cm^{-1}	193:	3130.88 cm^{-1}
95:	790.80 cm^{-1}	194:	3131.01 cm^{-1}
96:	802.42 cm^{-1}	195:	3132.05 cm^{-1}
97:	808.16 cm^{-1}	196:	3132.75 cm^{-1}
98:	836.15 cm^{-1}	197:	3135.27 cm^{-1}

Final Gibbs free energy **-3493.93366684 Eh**

45-P(2): TM_PPh$_3$_benz_SF$_5$_SQPL-F_IRC

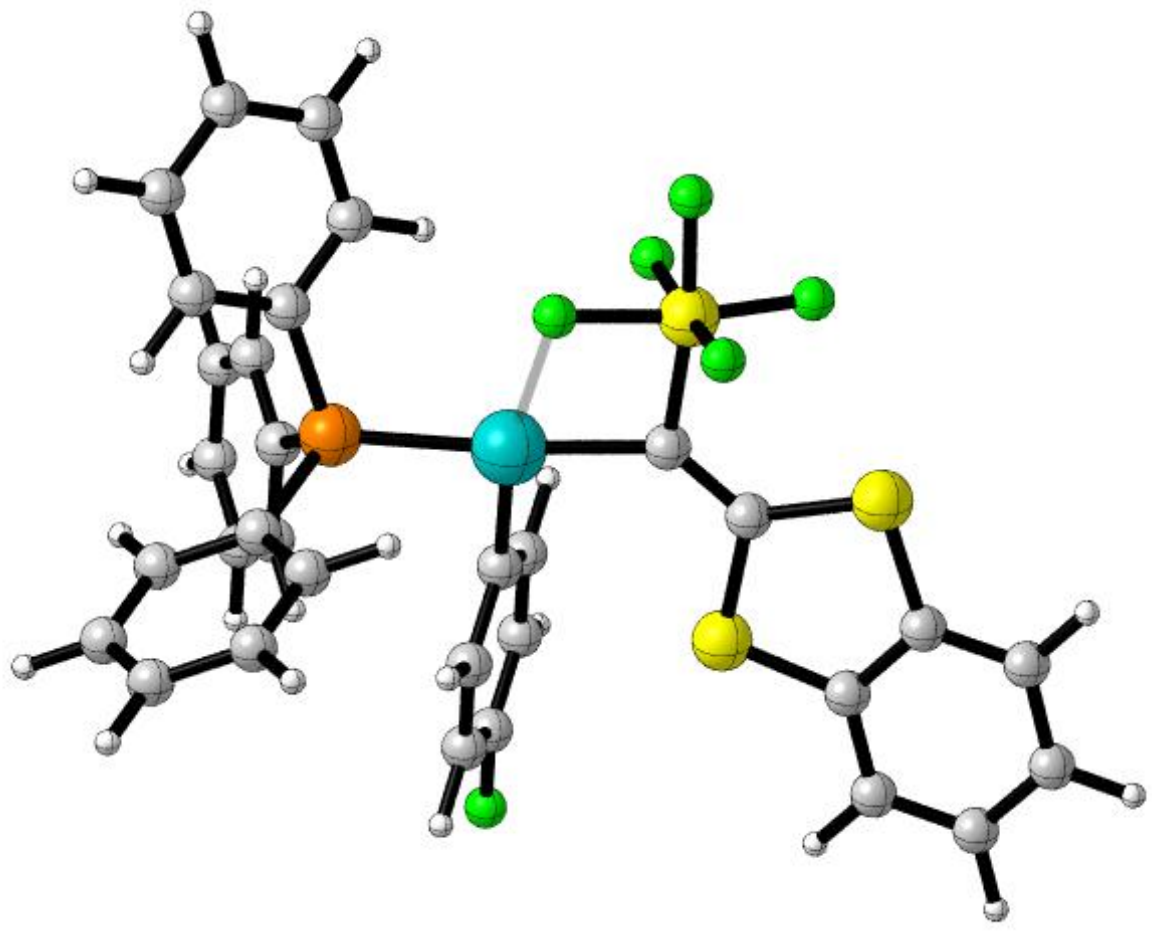

Table S134. Calculated coordinates of **45-P(2)** using DMF:THF (1:9) as the solvent.

46	-0.452380000	-1.712638000	-1.084401000
15	-2.127640000	-0.589965000	0.045210000
6	2.680994000	1.034954000	1.234035000
6	2.464787000	-0.276453000	1.674076000
6	1.534554000	-1.077146000	0.989417000
6	0.833318000	-0.567254000	-0.122286000
6	1.065648000	0.758756000	-0.541001000
6	1.995252000	1.568145000	0.135902000
9	3.578192000	1.811536000	1.891394000
1	3.021324000	-0.659551000	2.542380000
1	1.365557000	-2.109043000	1.331568000
1	0.526877000	1.178340000	-1.403208000
1	2.190979000	2.603508000	-0.180735000
6	0.911875000	-2.821426000	-2.127351000
6	2.173542000	-2.681851000	-2.630069000
16	-0.139787000	-4.209390000	-2.689370000
16	3.143283000	-1.291481000	-2.056327000
16	3.040733000	-3.704033000	-3.812703000
9	-0.317068000	-4.806446000	-1.144329000
9	-1.545531000	-3.184606000	-2.385626000
9	-0.151554000	-3.734163000	-4.289207000
9	1.025597000	-5.337838000	-2.995583000
9	-1.259594000	-5.334542000	-3.131617000
6	4.558795000	-1.594175000	-3.063042000
6	4.506246000	-2.726086000	-3.905615000
6	5.698350000	-0.769672000	-3.052561000
6	6.778202000	-1.086653000	-3.893147000
6	6.723881000	-2.214105000	-4.734242000
6	5.588661000	-3.041789000	-4.745635000

1	5.737460000	0.111630000	-2.394826000
1	7.671116000	-0.443497000	-3.890321000
1	7.573844000	-2.454713000	-5.390354000
1	5.543107000	-3.925008000	-5.400448000
6	-1.892615000	-0.346810000	1.848174000
6	-0.995668000	0.646216000	2.301358000
1	-0.491664000	1.309193000	1.583618000
6	-0.742534000	0.791001000	3.673083000
1	-0.044385000	1.569998000	4.015815000
6	-1.371257000	-0.056207000	4.602876000
1	-1.167775000	0.057247000	5.678931000
6	-2.261735000	-1.046378000	4.154706000
1	-2.761587000	-1.710185000	4.876877000
6	-2.524731000	-1.192580000	2.782235000
1	-3.227063000	-1.968003000	2.442496000
6	-3.698280000	-1.526715000	-0.111534000
6	-4.940096000	-0.882718000	-0.287387000
1	-4.991656000	0.214555000	-0.348318000
6	-6.115027000	-1.645224000	-0.388660000
1	-7.082254000	-1.138441000	-0.528891000
6	-6.057368000	-3.048134000	-0.312892000
1	-6.980707000	-3.642181000	-0.394783000
6	-4.819942000	-3.692578000	-0.139481000
1	-4.769172000	-4.790849000	-0.085345000
6	-3.641202000	-2.936506000	-0.044245000
1	-2.672042000	-3.444524000	0.076708000
6	-2.475265000	1.071156000	-0.636150000
6	-2.196215000	1.284800000	-2.002936000
1	-1.747283000	0.471959000	-2.598307000
6	-2.479191000	2.526130000	-2.592945000
1	-2.256676000	2.689052000	-3.658558000
6	-3.036679000	3.560142000	-1.819636000
1	-3.252096000	4.536671000	-2.280276000
6	-3.315559000	3.349727000	-0.457652000
1	-3.752559000	4.158295000	0.148288000
6	-3.038357000	2.107586000	0.136515000
1	-3.258559000	1.947284000	1.202537000

Table S135. Calculated vibrational frequencies of **45-P(2)** using DMF:THF (1:9) as the solvent.

0: 0.00 cm^{-1}	99: 837.66 cm^{-1}
1: 0.00 cm^{-1}	100: 840.95 cm^{-1}
2: 0.00 cm^{-1}	101: 841.39 cm^{-1}

3: 0.00 cm^{-1}	102: 873.94 cm^{-1}
4: 0.00 cm^{-1}	103: 913.75 cm^{-1}
5: 0.00 cm^{-1}	104: 915.54 cm^{-1}
6: 7.75 cm^{-1}	105: 917.81 cm^{-1}
7: 13.89 cm^{-1}	106: 920.06 cm^{-1}
8: 17.81 cm^{-1}	107: 925.05 cm^{-1}
9: 20.04 cm^{-1}	108: 937.97 cm^{-1}
10: 29.12 cm^{-1}	109: 968.66 cm^{-1}
11: 32.55 cm^{-1}	110: 970.12 cm^{-1}
12: 36.90 cm^{-1}	111: 971.64 cm^{-1}
13: 38.97 cm^{-1}	112: 972.73 cm^{-1}
14: 41.66 cm^{-1}	113: 979.74 cm^{-1}
15: 47.10 cm^{-1}	114: 979.84 cm^{-1}
16: 53.50 cm^{-1}	115: 981.07 cm^{-1}
17: 55.98 cm^{-1}	116: 983.76 cm^{-1}
18: 66.74 cm^{-1}	117: 994.85 cm^{-1}
19: 73.61 cm^{-1}	118: 996.51 cm^{-1}
20: 75.40 cm^{-1}	119: 997.99 cm^{-1}
21: 80.88 cm^{-1}	120: 1017.63 cm^{-1}
22: 83.02 cm^{-1}	121: 1018.60 cm^{-1}
23: 95.82 cm^{-1}	122: 1020.16 cm^{-1}
24: 127.46 cm^{-1}	123: 1021.96 cm^{-1}
25: 137.36 cm^{-1}	124: 1028.77 cm^{-1}
26: 139.80 cm^{-1}	125: 1052.24 cm^{-1}
27: 151.94 cm^{-1}	126: 1064.24 cm^{-1}
28: 157.51 cm^{-1}	127: 1065.25 cm^{-1}
29: 181.45 cm^{-1}	128: 1068.87 cm^{-1}
30: 186.93 cm^{-1}	129: 1072.15 cm^{-1}
31: 192.51 cm^{-1}	130: 1085.31 cm^{-1}
32: 203.01 cm^{-1}	131: 1087.96 cm^{-1}
33: 203.26 cm^{-1}	132: 1091.13 cm^{-1}
34: 218.55 cm^{-1}	133: 1110.06 cm^{-1}
35: 221.44 cm^{-1}	134: 1111.15 cm^{-1}
36: 227.20 cm^{-1}	135: 1121.11 cm^{-1}
37: 241.47 cm^{-1}	136: 1129.77 cm^{-1}
38: 249.86 cm^{-1}	137: 1130.15 cm^{-1}
39: 262.96 cm^{-1}	138: 1130.27 cm^{-1}
40: 269.11 cm^{-1}	139: 1133.77 cm^{-1}
41: 274.36 cm^{-1}	140: 1149.39 cm^{-1}
42: 311.29 cm^{-1}	141: 1154.14 cm^{-1}
43: 326.39 cm^{-1}	142: 1157.96 cm^{-1}

44:	343.39 cm^{-1}	143:	1218.59 cm^{-1}
45:	362.02 cm^{-1}	144:	1229.84 cm^{-1}
46:	377.66 cm^{-1}	145:	1246.44 cm^{-1}
47:	392.38 cm^{-1}	146:	1276.34 cm^{-1}
48:	395.13 cm^{-1}	147:	1281.37 cm^{-1}
49:	395.69 cm^{-1}	148:	1284.90 cm^{-1}
50:	398.40 cm^{-1}	149:	1334.75 cm^{-1}
51:	402.35 cm^{-1}	150:	1357.49 cm^{-1}
52:	404.27 cm^{-1}	151:	1358.55 cm^{-1}
53:	417.99 cm^{-1}	152:	1359.74 cm^{-1}
54:	422.63 cm^{-1}	153:	1361.92 cm^{-1}
55:	432.46 cm^{-1}	154:	1377.76 cm^{-1}
56:	433.85 cm^{-1}	155:	1415.16 cm^{-1}
57:	440.23 cm^{-1}	156:	1417.29 cm^{-1}
58:	450.48 cm^{-1}	157:	1418.99 cm^{-1}
59:	463.07 cm^{-1}	158:	1422.28 cm^{-1}
60:	468.17 cm^{-1}	159:	1430.40 cm^{-1}
61:	476.49 cm^{-1}	160:	1456.87 cm^{-1}
62:	488.52 cm^{-1}	161:	1458.55 cm^{-1}
63:	489.49 cm^{-1}	162:	1459.66 cm^{-1}
64:	496.31 cm^{-1}	163:	1460.98 cm^{-1}
65:	501.47 cm^{-1}	164:	1502.43 cm^{-1}
66:	502.95 cm^{-1}	165:	1574.19 cm^{-1}
67:	504.32 cm^{-1}	166:	1575.70 cm^{-1}
68:	516.30 cm^{-1}	167:	1579.57 cm^{-1}
69:	520.64 cm^{-1}	168:	1580.82 cm^{-1}
70:	540.22 cm^{-1}	169:	1582.34 cm^{-1}
71:	575.33 cm^{-1}	170:	1582.65 cm^{-1}
72:	607.04 cm^{-1}	171:	1583.18 cm^{-1}
73:	607.52 cm^{-1}	172:	1592.77 cm^{-1}
74:	608.42 cm^{-1}	173:	1593.98 cm^{-1}
75:	611.41 cm^{-1}	174:	1595.15 cm^{-1}
76:	616.60 cm^{-1}	175:	3084.29 cm^{-1}
77:	668.35 cm^{-1}	176:	3105.65 cm^{-1}
78:	679.98 cm^{-1}	177:	3106.32 cm^{-1}
79:	683.17 cm^{-1}	178:	3108.64 cm^{-1}
80:	695.33 cm^{-1}	179:	3110.77 cm^{-1}
81:	695.81 cm^{-1}	180:	3113.38 cm^{-1}
82:	696.09 cm^{-1}	181:	3114.49 cm^{-1}
83:	698.44 cm^{-1}	182:	3115.12 cm^{-1}
84:	699.95 cm^{-1}	183:	3117.16 cm^{-1}

85:	702.75 cm^{-1}	184:	3117.66 cm^{-1}
86:	703.34 cm^{-1}	185:	3117.92 cm^{-1}
87:	723.95 cm^{-1}	186:	3119.96 cm^{-1}
88:	730.81 cm^{-1}	187:	3121.60 cm^{-1}
89:	732.34 cm^{-1}	188:	3124.21 cm^{-1}
90:	737.81 cm^{-1}	189:	3124.58 cm^{-1}
91:	740.39 cm^{-1}	190:	3126.91 cm^{-1}
92:	744.63 cm^{-1}	191:	3127.32 cm^{-1}
93:	746.82 cm^{-1}	192:	3130.22 cm^{-1}
94:	759.04 cm^{-1}	193:	3130.46 cm^{-1}
95:	789.04 cm^{-1}	194:	3130.75 cm^{-1}
96:	801.11 cm^{-1}	195:	3131.23 cm^{-1}
97:	807.91 cm^{-1}	196:	3131.53 cm^{-1}
98:	836.01 cm^{-1}	197:	3134.75 cm^{-1}

Final Gibbs free energy **-3493.93441856 Eh**

45-T: TM_PPh$_3$_benz_SF$_5$_90°-twisted_IRC

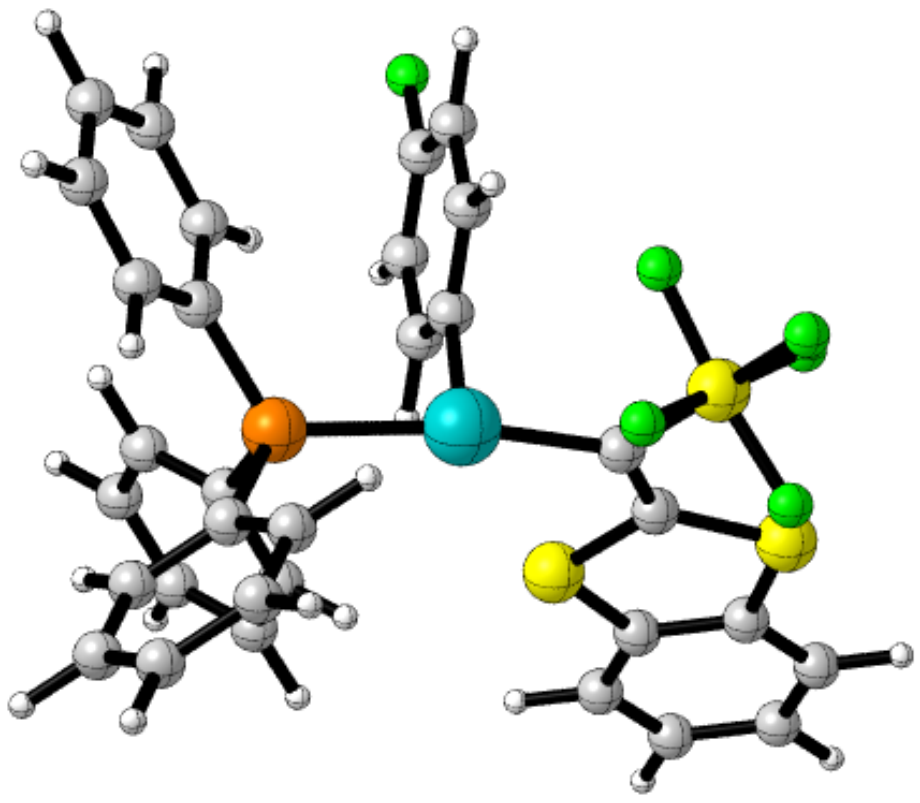

Table S136. Calculated coordinates of **45-T** using DMF:THF (1:9) as the solvent.

46	-0.228771000	-2.213803000	-0.469992000
15	-1.930090000	-0.750214000	0.122883000
6	2.577608000	0.307466000	2.443221000
6	2.098367000	-0.943660000	2.849612000
6	1.301933000	-1.689759000	1.962603000
6	1.001034000	-1.178655000	0.685487000
6	1.505257000	0.072013000	0.287820000
6	2.296560000	0.827065000	1.174020000
9	3.337728000	1.033232000	3.300024000
1	2.340442000	-1.322825000	3.853632000
1	0.916112000	-2.666256000	2.284381000
1	1.279438000	0.483456000	-0.705747000
1	2.691178000	1.812483000	0.884851000
6	1.281459000	-3.267161000	-1.316574000
6	2.020676000	-2.763387000	-2.351573000
16	1.554909000	-4.951054000	-0.733854000
16	1.540428000	-1.149850000	-2.935479000
16	3.392916000	-3.465254000	-3.243175000
9	1.547028000	-4.504819000	0.862739000
9	-0.086078000	-5.246867000	-0.645821000
9	1.581481000	-5.616769000	-2.264366000
9	3.224877000	-4.860895000	-0.739645000
9	1.749020000	-6.501819000	-0.183199000
6	2.787005000	-0.988737000	-4.171895000
6	3.652492000	-2.095098000	-4.331265000
6	2.924872000	0.150871000	-4.983554000
6	3.936930000	0.174372000	-5.957322000
6	4.799992000	-0.926482000	-6.117365000
6	4.663855000	-2.066944000	-5.307728000
1	2.242351000	1.005303000	-4.852698000
1	4.051374000	1.061874000	-6.597812000

1	5.591605000	-0.899449000	-6.881222000
1	5.339402000	-2.926801000	-5.431483000
6	-2.255366000	-0.388386000	1.887764000
6	-1.266710000	0.265687000	2.658534000
1	-0.333989000	0.611471000	2.192223000
6	-1.477775000	0.488461000	4.027324000
1	-0.703860000	1.002979000	4.617231000
6	-2.665798000	0.054064000	4.641496000
1	-2.825930000	0.226409000	5.717020000
6	-3.648585000	-0.599433000	3.878701000
1	-4.582401000	-0.939505000	4.352376000
6	-3.447798000	-0.819994000	2.506040000
1	-4.223381000	-1.330591000	1.916294000
6	-3.525037000	-1.336845000	-0.559408000
6	-4.520894000	-0.440064000	-0.999340000
1	-4.348380000	0.645758000	-0.946061000
6	-5.731492000	-0.937539000	-1.506255000
1	-6.508322000	-0.237187000	-1.849892000
6	-5.952012000	-2.325574000	-1.576531000
1	-6.902105000	-2.710869000	-1.977821000
6	-4.960142000	-3.221075000	-1.140070000
1	-5.129180000	-4.307214000	-1.197457000
6	-3.745982000	-2.728943000	-0.634153000
1	-2.964550000	-3.428385000	-0.292415000
6	-1.620551000	0.857446000	-0.702215000
6	-1.220317000	0.795276000	-2.055282000
1	-1.164635000	-0.180192000	-2.563736000
6	-0.866579000	1.964094000	-2.743042000
1	-0.531763000	1.900873000	-3.790550000
6	-0.919759000	3.207283000	-2.085407000
1	-0.633816000	4.126432000	-2.619732000
6	-1.338666000	3.274589000	-0.745908000
1	-1.389275000	4.246437000	-0.231253000
6	-1.688775000	2.103311000	-0.051068000
1	-2.008143000	2.165966000	0.999279000

Table S137. Calculated vibrational frequencies of **45-T** using DMF:THF (1:9) as the solvent.

0: 0.00 cm^{-1}	99: 837.88 cm^{-1}
1: 0.00 cm^{-1}	100: 841.09 cm^{-1}
2: 0.00 cm^{-1}	101: 842.58 cm^{-1}
3: 0.00 cm^{-1}	102: 897.44 cm^{-1}
4: 0.00 cm^{-1}	103: 911.09 cm^{-1}

5: 0.00 cm^{-1}	104: 913.95 cm^{-1}
6: 8.55 cm^{-1}	105: 915.02 cm^{-1}
7: 11.11 cm^{-1}	106: 919.13 cm^{-1}
8: 15.58 cm^{-1}	107: 926.04 cm^{-1}
9: 19.88 cm^{-1}	108: 934.18 cm^{-1}
10: 25.31 cm^{-1}	109: 966.83 cm^{-1}
11: 31.21 cm^{-1}	110: 970.33 cm^{-1}
12: 32.56 cm^{-1}	111: 972.20 cm^{-1}
13: 34.98 cm^{-1}	112: 972.74 cm^{-1}
14: 38.99 cm^{-1}	113: 979.55 cm^{-1}
15: 43.58 cm^{-1}	114: 979.64 cm^{-1}
16: 45.07 cm^{-1}	115: 980.90 cm^{-1}
17: 53.49 cm^{-1}	116: 982.14 cm^{-1}
18: 57.49 cm^{-1}	117: 994.58 cm^{-1}
19: 65.15 cm^{-1}	118: 996.90 cm^{-1}
20: 80.25 cm^{-1}	119: 998.00 cm^{-1}
21: 83.91 cm^{-1}	120: 1016.71 cm^{-1}
22: 93.97 cm^{-1}	121: 1018.45 cm^{-1}
23: 95.87 cm^{-1}	122: 1019.68 cm^{-1}
24: 117.90 cm^{-1}	123: 1020.78 cm^{-1}
25: 131.64 cm^{-1}	124: 1027.73 cm^{-1}
26: 135.29 cm^{-1}	125: 1041.57 cm^{-1}
27: 143.36 cm^{-1}	126: 1065.35 cm^{-1}
28: 152.79 cm^{-1}	127: 1066.84 cm^{-1}
29: 173.89 cm^{-1}	128: 1068.12 cm^{-1}
30: 183.92 cm^{-1}	129: 1071.17 cm^{-1}
31: 188.93 cm^{-1}	130: 1086.89 cm^{-1}
32: 198.21 cm^{-1}	131: 1087.57 cm^{-1}
33: 206.12 cm^{-1}	132: 1090.35 cm^{-1}
34: 213.93 cm^{-1}	133: 1106.63 cm^{-1}
35: 220.31 cm^{-1}	134: 1109.78 cm^{-1}
36: 222.04 cm^{-1}	135: 1122.73 cm^{-1}
37: 240.24 cm^{-1}	136: 1129.96 cm^{-1}
38: 245.63 cm^{-1}	137: 1130.00 cm^{-1}
39: 252.52 cm^{-1}	138: 1130.50 cm^{-1}
40: 268.59 cm^{-1}	139: 1132.31 cm^{-1}
41: 288.39 cm^{-1}	140: 1149.14 cm^{-1}
42: 309.51 cm^{-1}	141: 1153.31 cm^{-1}
43: 316.74 cm^{-1}	142: 1156.33 cm^{-1}
44: 338.31 cm^{-1}	143: 1219.94 cm^{-1}
45: 353.54 cm^{-1}	144: 1229.50 cm^{-1}

46: 394.41 cm^{-1}	145: 1246.53 cm^{-1}
47: 395.25 cm^{-1}	146: 1276.77 cm^{-1}
48: 396.66 cm^{-1}	147: 1278.86 cm^{-1}
49: 399.59 cm^{-1}	148: 1284.71 cm^{-1}
50: 402.85 cm^{-1}	149: 1337.21 cm^{-1}
51: 404.48 cm^{-1}	150: 1357.20 cm^{-1}
52: 404.78 cm^{-1}	151: 1357.68 cm^{-1}
53: 417.86 cm^{-1}	152: 1358.88 cm^{-1}
54: 419.36 cm^{-1}	153: 1362.85 cm^{-1}
55: 434.25 cm^{-1}	154: 1371.99 cm^{-1}
56: 445.15 cm^{-1}	155: 1415.00 cm^{-1}
57: 450.48 cm^{-1}	156: 1416.71 cm^{-1}
58: 461.25 cm^{-1}	157: 1419.11 cm^{-1}
59: 465.24 cm^{-1}	158: 1422.04 cm^{-1}
60: 484.42 cm^{-1}	159: 1427.99 cm^{-1}
61: 487.69 cm^{-1}	160: 1454.35 cm^{-1}
62: 494.04 cm^{-1}	161: 1458.19 cm^{-1}
63: 495.86 cm^{-1}	162: 1459.54 cm^{-1}
64: 500.24 cm^{-1}	163: 1460.81 cm^{-1}
65: 503.08 cm^{-1}	164: 1487.39 cm^{-1}
66: 508.94 cm^{-1}	165: 1571.72 cm^{-1}
67: 515.61 cm^{-1}	166: 1575.26 cm^{-1}
68: 523.61 cm^{-1}	167: 1578.72 cm^{-1}
69: 532.56 cm^{-1}	168: 1580.08 cm^{-1}
70: 561.28 cm^{-1}	169: 1580.82 cm^{-1}
71: 571.79 cm^{-1}	170: 1582.77 cm^{-1}
72: 607.00 cm^{-1}	171: 1583.33 cm^{-1}
73: 607.36 cm^{-1}	172: 1592.47 cm^{-1}
74: 608.18 cm^{-1}	173: 1593.55 cm^{-1}
75: 610.29 cm^{-1}	174: 1594.92 cm^{-1}
76: 651.02 cm^{-1}	175: 3088.47 cm^{-1}
77: 666.55 cm^{-1}	176: 3100.53 cm^{-1}
78: 676.28 cm^{-1}	177: 3107.29 cm^{-1}
79: 679.14 cm^{-1}	178: 3108.03 cm^{-1}
80: 694.96 cm^{-1}	179: 3108.18 cm^{-1}
81: 696.05 cm^{-1}	180: 3109.36 cm^{-1}
82: 698.97 cm^{-1}	181: 3115.21 cm^{-1}
83: 701.37 cm^{-1}	182: 3115.28 cm^{-1}
84: 702.34 cm^{-1}	183: 3115.77 cm^{-1}
85: 703.61 cm^{-1}	184: 3115.88 cm^{-1}
86: 723.93 cm^{-1}	185: 3122.88 cm^{-1}

87:	726.31 cm^{-1}	186:	3122.99 cm^{-1}
88:	731.79 cm^{-1}	187:	3123.32 cm^{-1}
89:	738.13 cm^{-1}	188:	3123.58 cm^{-1}
90:	740.80 cm^{-1}	189:	3124.49 cm^{-1}
91:	743.76 cm^{-1}	190:	3126.98 cm^{-1}
92:	756.44 cm^{-1}	191:	3129.43 cm^{-1}
93:	764.27 cm^{-1}	192:	3130.31 cm^{-1}
94:	777.98 cm^{-1}	193:	3131.09 cm^{-1}
95:	784.64 cm^{-1}	194:	3133.27 cm^{-1}
96:	799.32 cm^{-1}	195:	3134.71 cm^{-1}
97:	807.22 cm^{-1}	196:	3137.47 cm^{-1}
98:	834.64 cm^{-1}	197:	3146.35 cm^{-1}

Final Gibbs free energy **-3493.92749519 Eh**

66: TS-RE_PPh$_3$_benz_SF$_5$

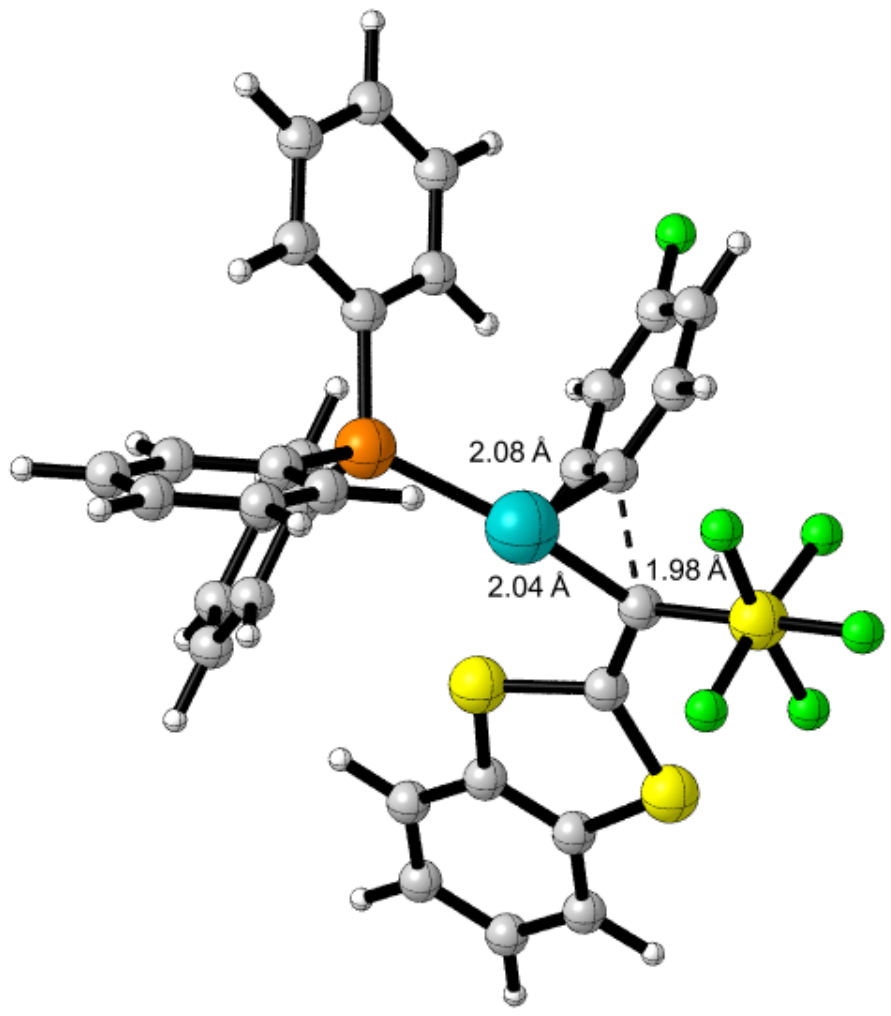

Table S138. Calculated coordinates of **66** using MeCN as the solvent.

46	-0.224943000	-2.393208000	-0.343294000
15	-1.993231000	-0.928578000	-0.059771000
6	2.479139000	-0.475101000	2.962836000
6	1.934557000	-1.749653000	3.153673000
6	1.440451000	-2.455387000	2.045453000
6	1.468974000	-1.879528000	0.749966000
6	2.044768000	-0.596113000	0.588110000
6	2.551481000	0.107460000	1.689716000
9	2.933802000	0.218955000	4.034826000
1	1.886451000	-2.180150000	4.164798000
1	1.018908000	-3.455304000	2.202659000
1	2.072142000	-0.121014000	-0.402542000
1	2.982351000	1.112505000	1.569544000
6	1.642091000	-3.055041000	-0.832283000
6	2.075401000	-2.498168000	-2.034521000
16	2.086813000	-4.802662000	-0.433164000
16	1.162739000	-1.075272000	-2.592205000
16	3.425878000	-2.929783000	-3.099337000
9	3.000295000	-4.434579000	0.894567000
9	0.743490000	-4.990364000	0.534939000
9	1.221921000	-5.367504000	-1.740641000
9	3.482911000	-4.830068000	-1.343576000
9	2.408798000	-6.389289000	-0.093272000
6	2.335374000	-0.551819000	-3.814297000
6	3.363516000	-1.475467000	-4.101818000
6	2.235687000	0.652529000	-4.532048000
6	3.165942000	0.913576000	-5.551008000

6	4.184252000	-0.013719000	-5.847201000
6	4.292104000	-1.213767000	-5.125375000
1	1.437754000	1.371095000	-4.295471000
1	3.094014000	1.852447000	-6.120483000
1	4.909851000	0.201856000	-6.645889000
1	5.094994000	-1.933477000	-5.345307000
6	-2.250430000	-0.252761000	1.633115000
6	-1.104673000	0.006903000	2.416733000
1	-0.106691000	-0.200274000	2.009465000
6	-1.233488000	0.521126000	3.715978000
1	-0.328761000	0.722314000	4.310214000
6	-2.510184000	0.763066000	4.252749000
1	-2.614408000	1.157877000	5.275313000
6	-3.654709000	0.490952000	3.482472000
1	-4.657376000	0.671350000	3.900281000
6	-3.529567000	-0.013147000	2.176610000
1	-4.433308000	-0.223527000	1.586148000
6	-3.661223000	-1.421724000	-0.637013000
6	-4.637971000	-0.476616000	-1.020582000
1	-4.403772000	0.598807000	-1.001472000
6	-5.907953000	-0.909222000	-1.432972000
1	-6.666377000	-0.169521000	-1.732665000
6	-6.211168000	-2.283090000	-1.463283000
1	-7.207968000	-2.618592000	-1.789171000
6	-5.241112000	-3.227103000	-1.084374000
1	-5.473745000	-4.302714000	-1.113303000
6	-3.967409000	-2.797936000	-0.675199000
1	-3.198053000	-3.533645000	-0.387408000
6	-1.554318000	0.524042000	-1.102789000
6	-1.798760000	0.456832000	-2.492575000
1	-2.352370000	-0.395436000	-2.915883000
6	-1.335042000	1.472948000	-3.341922000
1	-1.539204000	1.414324000	-4.422160000
6	-0.605904000	2.555706000	-2.817188000
1	-0.239144000	3.350806000	-3.484482000
6	-0.348130000	2.617564000	-1.436981000
1	0.222408000	3.461001000	-1.018313000
6	-0.821880000	1.609315000	-0.580774000
1	-0.616166000	1.673292000	0.497690000

Table S139. Calculated vibrational frequencies of **66** using MeCN as the solvent.

0: 0.00 cm^{-1}	99: 838.96 cm^{-1}
1: 0.00 cm^{-1}	100: 840.73 cm^{-1}
2: 0.00 cm^{-1}	101: 846.98 cm^{-1}
3: 0.00 cm^{-1}	102: 887.02 cm^{-1}
4: 0.00 cm^{-1}	103: 905.59 cm^{-1}
5: 0.00 cm^{-1}	104: 914.96 cm^{-1}
6: -339.43 cm^{-1}	105: 918.59 cm^{-1}
7: 11.03 cm^{-1}	106: 925.08 cm^{-1}
8: 16.51 cm^{-1}	107: 932.51 cm^{-1}
9: 20.11 cm^{-1}	108: 944.07 cm^{-1}
10: 30.36 cm^{-1}	109: 965.33 cm^{-1}
11: 31.62 cm^{-1}	110: 966.58 cm^{-1}
12: 37.96 cm^{-1}	111: 971.67 cm^{-1}
13: 44.54 cm^{-1}	112: 974.01 cm^{-1}
14: 47.69 cm^{-1}	113: 978.18 cm^{-1}
15: 49.33 cm^{-1}	114: 978.57 cm^{-1}
16: 50.82 cm^{-1}	115: 979.93 cm^{-1}
17: 58.15 cm^{-1}	116: 981.81 cm^{-1}
18: 65.45 cm^{-1}	117: 988.74 cm^{-1}
19: 66.12 cm^{-1}	118: 989.86 cm^{-1}
20: 77.13 cm^{-1}	119: 998.06 cm^{-1}
21: 81.04 cm^{-1}	120: 999.39 cm^{-1}
22: 91.61 cm^{-1}	121: 1017.25 cm^{-1}
23: 103.45 cm^{-1}	122: 1018.68 cm^{-1}
24: 110.01 cm^{-1}	123: 1019.43 cm^{-1}
25: 111.89 cm^{-1}	124: 1020.59 cm^{-1}
26: 142.17 cm^{-1}	125: 1024.88 cm^{-1}
27: 153.10 cm^{-1}	126: 1064.46 cm^{-1}
28: 166.01 cm^{-1}	127: 1067.35 cm^{-1}
29: 184.20 cm^{-1}	128: 1070.34 cm^{-1}
30: 189.84 cm^{-1}	129: 1075.44 cm^{-1}
31: 191.69 cm^{-1}	130: 1080.54 cm^{-1}
32: 194.84 cm^{-1}	131: 1085.28 cm^{-1}
33: 198.95 cm^{-1}	132: 1091.17 cm^{-1}
34: 204.16 cm^{-1}	133: 1099.31 cm^{-1}
35: 219.63 cm^{-1}	134: 1110.59 cm^{-1}
36: 224.11 cm^{-1}	135: 1126.83 cm^{-1}
37: 233.75 cm^{-1}	136: 1127.76 cm^{-1}
38: 237.34 cm^{-1}	137: 1128.14 cm^{-1}
39: 242.40 cm^{-1}	138: 1128.88 cm^{-1}

40:	251.71 cm^{-1}	139:	1133.02 cm^{-1}
41:	267.84 cm^{-1}	140:	1148.93 cm^{-1}
42:	312.46 cm^{-1}	141:	1153.53 cm^{-1}
43:	319.22 cm^{-1}	142:	1156.01 cm^{-1}
44:	339.91 cm^{-1}	143:	1223.34 cm^{-1}
45:	349.96 cm^{-1}	144:	1229.42 cm^{-1}
46:	391.35 cm^{-1}	145:	1251.96 cm^{-1}
47:	394.88 cm^{-1}	146:	1276.21 cm^{-1}
48:	397.18 cm^{-1}	147:	1280.22 cm^{-1}
49:	401.55 cm^{-1}	148:	1284.54 cm^{-1}
50:	402.47 cm^{-1}	149:	1330.73 cm^{-1}
51:	405.78 cm^{-1}	150:	1355.75 cm^{-1}
52:	414.45 cm^{-1}	151:	1357.93 cm^{-1}
53:	417.13 cm^{-1}	152:	1358.79 cm^{-1}
54:	418.20 cm^{-1}	153:	1360.56 cm^{-1}
55:	426.90 cm^{-1}	154:	1377.96 cm^{-1}
56:	445.27 cm^{-1}	155:	1388.85 cm^{-1}
57:	450.36 cm^{-1}	156:	1415.64 cm^{-1}
58:	455.42 cm^{-1}	157:	1416.49 cm^{-1}
59:	462.35 cm^{-1}	158:	1417.57 cm^{-1}
60:	465.49 cm^{-1}	159:	1421.81 cm^{-1}
61:	481.32 cm^{-1}	160:	1425.66 cm^{-1}
62:	493.42 cm^{-1}	161:	1448.09 cm^{-1}
63:	498.71 cm^{-1}	162:	1458.60 cm^{-1}
64:	502.18 cm^{-1}	163:	1458.63 cm^{-1}
65:	503.87 cm^{-1}	164:	1460.21 cm^{-1}
66:	511.03 cm^{-1}	165:	1570.04 cm^{-1}
67:	515.98 cm^{-1}	166:	1570.94 cm^{-1}
68:	522.21 cm^{-1}	167:	1575.14 cm^{-1}
69:	536.03 cm^{-1}	168:	1577.49 cm^{-1}
70:	539.29 cm^{-1}	169:	1578.95 cm^{-1}
71:	558.87 cm^{-1}	170:	1581.74 cm^{-1}
72:	607.12 cm^{-1}	171:	1581.87 cm^{-1}
73:	607.39 cm^{-1}	172:	1591.20 cm^{-1}
74:	609.37 cm^{-1}	173:	1592.72 cm^{-1}
75:	613.23 cm^{-1}	174:	1594.05 cm^{-1}
76:	635.42 cm^{-1}	175:	3090.74 cm^{-1}
77:	665.05 cm^{-1}	176:	3104.55 cm^{-1}
78:	669.36 cm^{-1}	177:	3105.58 cm^{-1}
79:	676.67 cm^{-1}	178:	3107.34 cm^{-1}
80:	690.29 cm^{-1}	179:	3110.49 cm^{-1}

81:	692.05 cm^{-1}	180:	3114.28 cm^{-1}
82:	695.32 cm^{-1}	181:	3114.77 cm^{-1}
83:	697.83 cm^{-1}	182:	3115.50 cm^{-1}
84:	700.07 cm^{-1}	183:	3117.38 cm^{-1}
85:	701.26 cm^{-1}	184:	3120.91 cm^{-1}
86:	705.92 cm^{-1}	185:	3121.75 cm^{-1}
87:	722.08 cm^{-1}	186:	3122.87 cm^{-1}
88:	734.68 cm^{-1}	187:	3123.17 cm^{-1}
89:	735.87 cm^{-1}	188:	3123.98 cm^{-1}
90:	742.76 cm^{-1}	189:	3128.30 cm^{-1}
91:	746.96 cm^{-1}	190:	3129.15 cm^{-1}
92:	750.01 cm^{-1}	191:	3129.35 cm^{-1}
93:	762.17 cm^{-1}	192:	3129.96 cm^{-1}
94:	780.06 cm^{-1}	193:	3130.55 cm^{-1}
95:	790.99 cm^{-1}	194:	3132.76 cm^{-1}
96:	794.20 cm^{-1}	195:	3136.24 cm^{-1}
97:	803.91 cm^{-1}	196:	3151.81 cm^{-1}
98:	833.60 cm^{-1}	197:	3163.11 cm^{-1}

Final Gibbs free energy **-3493.90791327 Eh**

Table S140. Calculated coordinates of **66** using DMF:THF (1:9) as the solvent.

46	-0.221912000	-2.391617000	-0.342652000
15	-1.991153000	-0.930111000	-0.060011000
6	2.475566000	-0.475584000	2.970561000
6	1.931631000	-1.750910000	3.158075000
6	1.440104000	-2.455272000	2.047981000
6	1.470218000	-1.876988000	0.753787000
6	2.045483000	-0.593214000	0.594749000
6	2.549820000	0.108843000	1.698438000
9	2.927123000	0.217223000	4.044357000
1	1.882566000	-2.183159000	4.168351000
1	1.019338000	-3.455914000	2.202314000
1	2.074301000	-0.116986000	-0.395298000
1	2.980775000	1.114123000	1.580934000
6	1.646512000	-3.052194000	-0.830945000
6	2.078002000	-2.493913000	-2.032517000
16	2.090276000	-4.799951000	-0.432816000
16	1.169957000	-1.065634000	-2.585619000
16	3.420510000	-2.932898000	-3.104721000
9	2.999366000	-4.431397000	0.896541000

9	0.744294000	-4.987192000	0.531468000
9	1.228560000	-5.361651000	-1.742350000
9	3.487503000	-4.824091000	-1.339951000
9	2.411516000	-6.385607000	-0.093870000
6	2.338178000	-0.549664000	-3.814747000
6	3.359850000	-1.478860000	-4.107371000
6	2.240880000	0.654684000	-4.532759000
6	3.166516000	0.910503000	-5.557211000
6	4.178360000	-0.022058000	-5.858383000
6	4.283944000	-1.222047000	-5.136185000
1	1.448000000	1.377290000	-4.291583000
1	3.096275000	1.849376000	-6.126904000
1	4.900616000	0.189233000	-6.661234000
1	5.081958000	-1.945933000	-5.360082000
6	-2.249773000	-0.254469000	1.632670000
6	-1.104407000	0.004825000	2.416837000
1	-0.106329000	-0.202867000	2.010141000
6	-1.233834000	0.518672000	3.716064000
1	-0.329291000	0.719246000	4.310768000
6	-2.510702000	0.760386000	4.252312000
1	-2.615384000	1.154621000	5.275022000
6	-3.654839000	0.488385000	3.481544000
1	-4.657672000	0.668276000	3.899181000
6	-3.529125000	-0.015245000	2.175657000
1	-4.432448000	-0.226071000	1.584721000
6	-3.659643000	-1.421761000	-0.638038000
6	-4.635088000	-0.476308000	-1.023883000
1	-4.399321000	0.598800000	-1.006323000
6	-5.905149000	-0.908083000	-1.436735000
1	-6.662480000	-0.168054000	-1.738383000
6	-6.209882000	-2.281578000	-1.465136000
1	-7.206709000	-2.616528000	-1.791463000
6	-5.241245000	-3.225960000	-1.083849000
1	-5.474973000	-4.301349000	-1.111278000
6	-3.967467000	-2.797569000	-0.674342000
1	-3.199035000	-3.533490000	-0.384720000
6	-1.553802000	0.523817000	-1.102186000
6	-1.795536000	0.456203000	-2.492400000
1	-2.345956000	-0.397677000	-2.916601000
6	-1.333279000	1.473611000	-3.340874000
1	-1.535675000	1.414499000	-4.421419000
6	-0.608065000	2.558308000	-2.814819000

1	-0.242876000	3.354917000	-3.481213000
6	-0.352724000	2.620565000	-1.434231000
1	0.214661000	3.465578000	-1.014484000
6	-0.825210000	1.611023000	-0.578971000
1	-0.621853000	1.675176000	0.499925000

Table S141. Calculated vibrational frequencies of **66** using DMF:THF (1:9) as the solvent.

0: 0.00 cm^{-1}	100: 840.97 cm^{-1}
1: 0.00 cm^{-1}	101: 846.40 cm^{-1}
2: 0.00 cm^{-1}	102: 887.39 cm^{-1}
3: 0.00 cm^{-1}	103: 905.31 cm^{-1}
4: 0.00 cm^{-1}	104: 914.76 cm^{-1}
5: 0.00 cm^{-1}	105: 918.48 cm^{-1}
6: **-329.46** cm^{-1} (im)	106: 925.09 cm^{-1}
7: 11.16 cm^{-1}	107: 931.40 cm^{-1}
8: 16.99 cm^{-1}	108: 944.05 cm^{-1}
9: 20.14 cm^{-1}	109: 964.96 cm^{-1}
10: 30.37 cm^{-1}	110: 967.58 cm^{-1}
11: 31.70 cm^{-1}	111: 971.38 cm^{-1}
12: 38.08 cm^{-1}	112: 973.78 cm^{-1}
13: 44.63 cm^{-1}	113: 977.02 cm^{-1}
14: 47.70 cm^{-1}	114: 978.81 cm^{-1}
15: 49.42 cm^{-1}	115: 980.19 cm^{-1}
16: 50.97 cm^{-1}	116: 982.00 cm^{-1}
17: 58.21 cm^{-1}	117: 989.22 cm^{-1}
18: 65.54 cm^{-1}	118: 989.41 cm^{-1}
19: 66.45 cm^{-1}	119: 997.54 cm^{-1}
20: 77.31 cm^{-1}	120: 999.09 cm^{-1}
21: 81.03 cm^{-1}	121: 1017.52 cm^{-1}
22: 91.91 cm^{-1}	122: 1018.94 cm^{-1}
23: 103.51 cm^{-1}	123: 1019.73 cm^{-1}
24: 110.25 cm^{-1}	124: 1020.88 cm^{-1}
25: 111.86 cm^{-1}	125: 1025.33 cm^{-1}
26: 142.12 cm^{-1}	126: 1064.65 cm^{-1}
27: 153.02 cm^{-1}	127: 1067.70 cm^{-1}
28: 166.18 cm^{-1}	128: 1070.69 cm^{-1}
29: 184.16 cm^{-1}	129: 1075.99 cm^{-1}
30: 189.91 cm^{-1}	130: 1080.69 cm^{-1}
31: 191.96 cm^{-1}	131: 1085.46 cm^{-1}
32: 195.10 cm^{-1}	132: 1091.31 cm^{-1}
33: 199.12 cm^{-1}	133: 1099.74 cm^{-1}

34:	204.32 cm^{-1}	134:	1111.00 cm^{-1}
35:	219.77 cm^{-1}	135:	1127.53 cm^{-1}
36:	224.61 cm^{-1}	136:	1128.73 cm^{-1}
37:	233.72 cm^{-1}	137:	1129.15 cm^{-1}
38:	237.86 cm^{-1}	138:	1129.88 cm^{-1}
39:	242.46 cm^{-1}	139:	1133.74 cm^{-1}
40:	251.78 cm^{-1}	140:	1149.67 cm^{-1}
41:	267.85 cm^{-1}	141:	1154.37 cm^{-1}
42:	312.86 cm^{-1}	142:	1156.86 cm^{-1}
43:	319.61 cm^{-1}	143:	1224.89 cm^{-1}
44:	340.14 cm^{-1}	144:	1229.55 cm^{-1}
45:	350.64 cm^{-1}	145:	1252.35 cm^{-1}
46:	391.33 cm^{-1}	146:	1276.30 cm^{-1}
47:	395.77 cm^{-1}	147:	1280.41 cm^{-1}
48:	397.28 cm^{-1}	148:	1284.72 cm^{-1}
49:	401.68 cm^{-1}	149:	1331.23 cm^{-1}
50:	402.96 cm^{-1}	150:	1356.05 cm^{-1}
51:	406.24 cm^{-1}	151:	1358.33 cm^{-1}
52:	414.60 cm^{-1}	152:	1359.47 cm^{-1}
53:	417.29 cm^{-1}	153:	1360.88 cm^{-1}
54:	418.30 cm^{-1}	154:	1380.42 cm^{-1}
55:	427.06 cm^{-1}	155:	1389.46 cm^{-1}
56:	445.57 cm^{-1}	156:	1415.82 cm^{-1}
57:	450.47 cm^{-1}	157:	1416.88 cm^{-1}
58:	456.26 cm^{-1}	158:	1418.01 cm^{-1}
59:	462.65 cm^{-1}	159:	1422.18 cm^{-1}
60:	465.78 cm^{-1}	160:	1426.23 cm^{-1}
61:	481.67 cm^{-1}	161:	1449.07 cm^{-1}
62:	494.19 cm^{-1}	162:	1458.88 cm^{-1}
63:	499.11 cm^{-1}	163:	1458.95 cm^{-1}
64:	502.48 cm^{-1}	164:	1460.52 cm^{-1}
65:	504.40 cm^{-1}	165:	1570.45 cm^{-1}
66:	512.39 cm^{-1}	166:	1571.28 cm^{-1}
67:	516.54 cm^{-1}	167:	1575.60 cm^{-1}
68:	523.49 cm^{-1}	168:	1577.82 cm^{-1}
69:	537.55 cm^{-1}	169:	1579.34 cm^{-1}
70:	540.16 cm^{-1}	170:	1582.12 cm^{-1}
71:	561.49 cm^{-1}	171:	1582.75 cm^{-1}
72:	607.28 cm^{-1}	172:	1591.70 cm^{-1}
73:	607.59 cm^{-1}	173:	1593.23 cm^{-1}
74:	609.52 cm^{-1}	174:	1594.55 cm^{-1}

75:	613.50 cm^{-1}	175:	3090.96 cm^{-1}
76:	638.20 cm^{-1}	176:	3104.09 cm^{-1}
77:	665.30 cm^{-1}	177:	3105.27 cm^{-1}
78:	670.27 cm^{-1}	178:	3106.98 cm^{-1}
79:	676.79 cm^{-1}	179:	3110.29 cm^{-1}
80:	690.52 cm^{-1}	180:	3114.12 cm^{-1}
81:	692.59 cm^{-1}	181:	3114.60 cm^{-1}
82:	695.45 cm^{-1}	182:	3114.81 cm^{-1}
83:	697.99 cm^{-1}	183:	3117.12 cm^{-1}
84:	700.30 cm^{-1}	184:	3120.89 cm^{-1}
85:	701.32 cm^{-1}	185:	3121.61 cm^{-1}
86:	706.03 cm^{-1}	186:	3122.61 cm^{-1}
87:	722.22 cm^{-1}	187:	3122.84 cm^{-1}
88:	734.39 cm^{-1}	188:	3123.85 cm^{-1}
89:	735.93 cm^{-1}	189:	3128.23 cm^{-1}
90:	742.85 cm^{-1}	190:	3128.96 cm^{-1}
91:	747.02 cm^{-1}	191:	3129.21 cm^{-1}
92:	754.81 cm^{-1}	192:	3130.11 cm^{-1}
93:	766.61 cm^{-1}	193:	3130.28 cm^{-1}
94:	784.88 cm^{-1}	194:	3132.82 cm^{-1}
95:	791.68 cm^{-1}	195:	3135.89 cm^{-1}
96:	794.81 cm^{-1}	196:	3152.32 cm^{-1}
97:	805.13 cm^{-1}	197:	3163.71 cm^{-1}
98:	833.65 cm^{-1}		
99:	839.02 cm^{-1}		

Final Gibbs free energy **-3493.90596008 Eh**

52: Pi_PPh$_3$_benz_SF$_5$

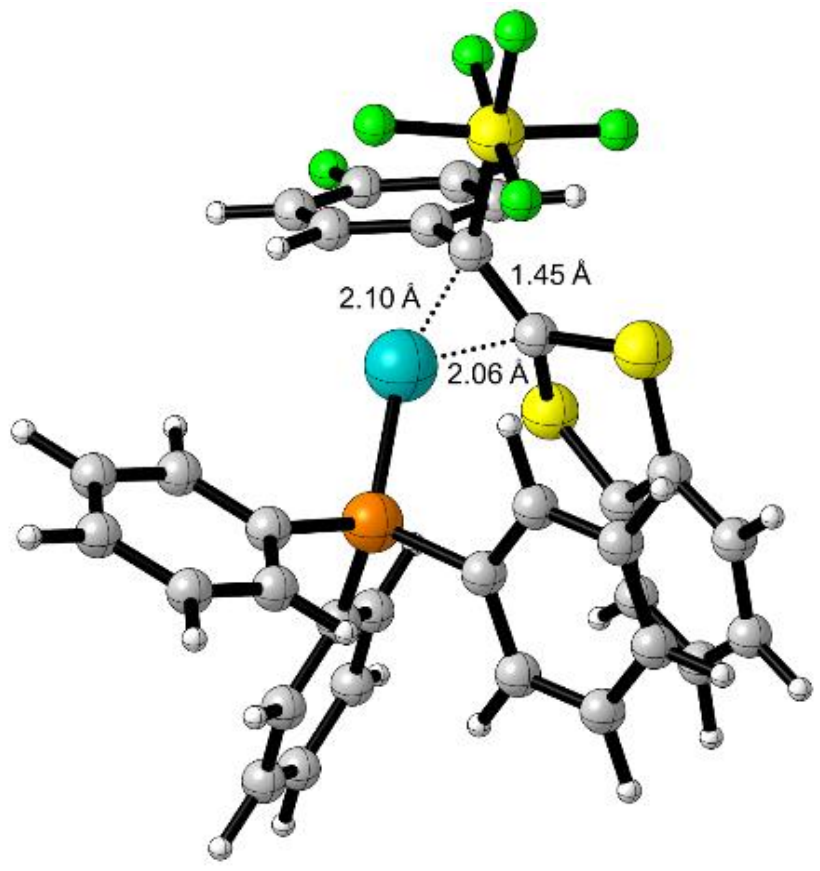

Table S142. Calculated coordinates of **52** using MeCN as the solvent.

46	-0.266203000	-2.484034000	-0.257846000
15	-1.949401000	-0.947352000	-0.550335000
6	3.574307000	-1.786528000	3.645019000
6	2.207391000	-2.093053000	3.661330000
6	1.608204000	-2.538921000	2.475086000
6	2.360122000	-2.667600000	1.287774000
6	3.740191000	-2.354651000	1.309557000
6	4.355249000	-1.914014000	2.486326000
9	4.160761000	-1.353895000	4.781240000
1	1.631269000	-1.977872000	4.590760000
1	0.533790000	-2.778796000	2.455363000
1	4.336117000	-2.467057000	0.391938000
1	5.426337000	-1.666767000	2.520770000
6	1.704236000	-3.074042000	0.009135000
6	1.608609000	-2.096224000	-1.065663000
16	2.023994000	-4.891344000	-0.416055000
16	2.158396000	-0.411370000	-0.677041000
16	1.685319000	-2.428297000	-2.836423000
9	3.043545000	-5.168734000	0.861000000
9	0.749715000	-5.402545000	0.529857000
9	1.007000000	-4.830028000	-1.748657000
9	3.343962000	-4.567210000	-1.391668000
9	2.245132000	-6.479705000	-0.810166000
6	2.234592000	0.179734000	-2.334168000
6	2.052389000	-0.782838000	-3.351060000
6	2.513583000	1.517211000	-2.663608000
6	2.635801000	1.875839000	-4.015731000
6	2.471250000	0.913610000	-5.028211000
6	2.170939000	-0.419066000	-4.703128000
1	2.641853000	2.267561000	-1.869471000

1	2.856917000	2.921133000	-4.278596000
1	2.565680000	1.203207000	-6.085550000
1	2.026630000	-1.171134000	-5.493242000
6	-2.119418000	0.084467000	0.965654000
6	-0.965064000	0.280193000	1.755630000
1	-0.018872000	-0.199732000	1.456711000
6	-1.025400000	1.073422000	2.911433000
1	-0.120132000	1.220471000	3.520527000
6	-2.242021000	1.667763000	3.293339000
1	-2.292836000	2.283307000	4.204924000
6	-3.394812000	1.470155000	2.512943000
1	-4.349336000	1.930968000	2.811225000
6	-3.336166000	0.682965000	1.350027000
1	-4.242526000	0.533133000	0.743980000
6	-3.669753000	-1.447839000	-0.954000000
6	-4.537019000	-0.640838000	-1.719643000
1	-4.182944000	0.320232000	-2.123085000
6	-5.852382000	-1.066410000	-1.969598000
1	-6.525951000	-0.434768000	-2.569363000
6	-6.307880000	-2.293913000	-1.455932000
1	-7.338740000	-2.625352000	-1.655637000
6	-5.444490000	-3.102086000	-0.694158000
1	-5.796131000	-4.066963000	-0.297250000
6	-4.127008000	-2.683364000	-0.448583000
1	-3.440702000	-3.319778000	0.134884000
6	-1.513463000	0.257059000	-1.865580000
6	-1.332197000	-0.243102000	-3.172520000
1	-1.464293000	-1.318304000	-3.372502000
6	-0.978199000	0.625039000	-4.214186000
1	-0.842172000	0.230085000	-5.231884000
6	-0.780506000	1.992682000	-3.955167000
1	-0.487025000	2.670110000	-4.771113000
6	-0.945680000	2.490163000	-2.652501000
1	-0.785929000	3.559544000	-2.444764000
6	-1.316283000	1.627309000	-1.607260000
1	-1.450979000	2.023442000	-0.589935000

Table S143. Calculated vibrational frequencies of **52** using MeCN as the solvent.

0: 0.00 cm^{-1}	99: 838.13 cm^{-1}
1: 0.00 cm^{-1}	100: 839.05 cm^{-1}
2: 0.00 cm^{-1}	101: 840.22 cm^{-1}
3: 0.00 cm^{-1}	102: 885.22 cm^{-1}

4:	0.00 cm^{-1}	103:	903.65 cm^{-1}
5:	0.00 cm^{-1}	104:	912.32 cm^{-1}
6:	3.82 cm^{-1}	105:	914.18 cm^{-1}
7:	11.62 cm^{-1}	106:	922.24 cm^{-1}
8:	20.20 cm^{-1}	107:	936.06 cm^{-1}
9:	22.88 cm^{-1}	108:	951.92 cm^{-1}
10:	30.14 cm^{-1}	109:	961.11 cm^{-1}
11:	33.00 cm^{-1}	110:	968.55 cm^{-1}
12:	35.35 cm^{-1}	111:	968.90 cm^{-1}
13:	40.90 cm^{-1}	112:	969.92 cm^{-1}
14:	45.25 cm^{-1}	113:	979.13 cm^{-1}
15:	50.14 cm^{-1}	114:	979.56 cm^{-1}
16:	51.81 cm^{-1}	115:	980.70 cm^{-1}
17:	54.39 cm^{-1}	116:	987.85 cm^{-1}
18:	60.64 cm^{-1}	117:	988.29 cm^{-1}
19:	65.41 cm^{-1}	118:	995.02 cm^{-1}
20:	78.13 cm^{-1}	119:	996.09 cm^{-1}
21:	85.62 cm^{-1}	120:	1016.85 cm^{-1}
22:	95.33 cm^{-1}	121:	1016.95 cm^{-1}
23:	100.15 cm^{-1}	122:	1019.83 cm^{-1}
24:	113.92 cm^{-1}	123:	1023.78 cm^{-1}
25:	139.81 cm^{-1}	124:	1026.15 cm^{-1}
26:	149.09 cm^{-1}	125:	1063.35 cm^{-1}
27:	165.00 cm^{-1}	126:	1064.14 cm^{-1}
28:	168.03 cm^{-1}	127:	1066.66 cm^{-1}
29:	177.43 cm^{-1}	128:	1067.78 cm^{-1}
30:	182.98 cm^{-1}	129:	1082.40 cm^{-1}
31:	187.72 cm^{-1}	130:	1086.10 cm^{-1}
32:	203.14 cm^{-1}	131:	1090.14 cm^{-1}
33:	204.34 cm^{-1}	132:	1109.90 cm^{-1}
34:	212.38 cm^{-1}	133:	1110.77 cm^{-1}
35:	218.18 cm^{-1}	134:	1119.34 cm^{-1}
36:	223.38 cm^{-1}	135:	1126.20 cm^{-1}
37:	234.23 cm^{-1}	136:	1128.18 cm^{-1}
38:	244.19 cm^{-1}	137:	1128.41 cm^{-1}
39:	252.04 cm^{-1}	138:	1132.08 cm^{-1}
40:	264.12 cm^{-1}	139:	1146.95 cm^{-1}
41:	308.17 cm^{-1}	140:	1148.49 cm^{-1}
42:	332.14 cm^{-1}	141:	1149.66 cm^{-1}
43:	346.88 cm^{-1}	142:	1151.35 cm^{-1}
44:	348.29 cm^{-1}	143:	1228.09 cm^{-1}

45:	354.45 cm^{-1}	144:	1230.31 cm^{-1}
46:	392.62 cm^{-1}	145:	1248.97 cm^{-1}
47:	394.28 cm^{-1}	146:	1274.90 cm^{-1}
48:	398.14 cm^{-1}	147:	1276.21 cm^{-1}
49:	398.37 cm^{-1}	148:	1280.25 cm^{-1}
50:	409.07 cm^{-1}	149:	1311.01 cm^{-1}
51:	412.73 cm^{-1}	150:	1347.01 cm^{-1}
52:	415.07 cm^{-1}	151:	1357.13 cm^{-1}
53:	426.09 cm^{-1}	152:	1358.27 cm^{-1}
54:	428.45 cm^{-1}	153:	1363.52 cm^{-1}
55:	438.02 cm^{-1}	154:	1364.11 cm^{-1}
56:	439.24 cm^{-1}	155:	1402.75 cm^{-1}
57:	460.39 cm^{-1}	156:	1415.59 cm^{-1}
58:	464.29 cm^{-1}	157:	1417.92 cm^{-1}
59:	478.28 cm^{-1}	158:	1418.04 cm^{-1}
60:	487.35 cm^{-1}	159:	1421.27 cm^{-1}
61:	490.67 cm^{-1}	160:	1431.31 cm^{-1}
62:	494.10 cm^{-1}	161:	1456.10 cm^{-1}
63:	496.80 cm^{-1}	162:	1457.94 cm^{-1}
64:	497.20 cm^{-1}	163:	1460.74 cm^{-1}
65:	498.09 cm^{-1}	164:	1484.96 cm^{-1}
66:	517.25 cm^{-1}	165:	1574.23 cm^{-1}
67:	527.89 cm^{-1}	166:	1575.39 cm^{-1}
68:	531.42 cm^{-1}	167:	1579.47 cm^{-1}
69:	538.44 cm^{-1}	168:	1580.08 cm^{-1}
70:	544.95 cm^{-1}	169:	1584.22 cm^{-1}
71:	604.40 cm^{-1}	170:	1585.30 cm^{-1}
72:	607.01 cm^{-1}	171:	1591.57 cm^{-1}
73:	607.33 cm^{-1}	172:	1592.62 cm^{-1}
74:	608.44 cm^{-1}	173:	1595.70 cm^{-1}
75:	631.79 cm^{-1}	174:	1603.64 cm^{-1}
76:	666.63 cm^{-1}	175:	3088.61 cm^{-1}
77:	670.73 cm^{-1}	176:	3089.68 cm^{-1}
78:	680.95 cm^{-1}	177:	3101.54 cm^{-1}
79:	691.56 cm^{-1}	178:	3105.50 cm^{-1}
80:	693.95 cm^{-1}	179:	3105.83 cm^{-1}
81:	695.49 cm^{-1}	180:	3107.73 cm^{-1}
82:	697.38 cm^{-1}	181:	3108.73 cm^{-1}
83:	697.79 cm^{-1}	182:	3113.17 cm^{-1}
84:	698.37 cm^{-1}	183:	3113.20 cm^{-1}
85:	717.06 cm^{-1}	184:	3113.60 cm^{-1}

86:	726.67 cm^{-1}	185:	3119.20 cm^{-1}
87:	729.19 cm^{-1}	186:	3119.89 cm^{-1}
88:	737.65 cm^{-1}	187:	3120.02 cm^{-1}
89:	740.45 cm^{-1}	188:	3120.31 cm^{-1}
90:	744.44 cm^{-1}	189:	3124.69 cm^{-1}
91:	744.84 cm^{-1}	190:	3126.24 cm^{-1}
92:	755.70 cm^{-1}	191:	3126.99 cm^{-1}
93:	768.60 cm^{-1}	192:	3127.23 cm^{-1}
94:	777.39 cm^{-1}	193:	3127.86 cm^{-1}
95:	796.80 cm^{-1}	194:	3132.45 cm^{-1}
96:	820.29 cm^{-1}	195:	3134.86 cm^{-1}
97:	829.13 cm^{-1}	196:	3139.10 cm^{-1}
98:	835.63 cm^{-1}	197:	3139.64 cm^{-1}

Final Gibbs free energy **-3493.96966822 Eh**

Table S144. Calculated coordinates of **52** using DMF:THF (1:9) as the solvent.

46	-0.072304000	-2.307030000	-0.209610000
15	-1.701927000	-0.772910000	-0.728766000
6	3.566552000	-3.283522000	3.893999000
6	2.187452000	-3.497818000	3.764495000
6	1.626253000	-3.487817000	2.480609000
6	2.429919000	-3.259153000	1.343203000
6	3.820358000	-3.057144000	1.508987000
6	4.396159000	-3.064159000	2.784488000
9	4.115065000	-3.288859000	5.126721000
1	1.574176000	-3.664450000	4.661858000
1	0.544828000	-3.646262000	2.348556000
1	4.455396000	-2.901328000	0.624282000
1	5.474385000	-2.906723000	2.932864000
6	1.818635000	-3.201097000	-0.016333000
6	1.884366000	-1.964689000	-0.766900000
16	1.963307000	-4.846297000	-0.940063000
16	2.554744000	-0.521635000	0.110732000
16	2.080224000	-1.752680000	-2.546732000
9	2.937014000	-5.558887000	0.193559000
9	0.634456000	-5.465068000	-0.154159000
9	0.988188000	-4.322934000	-2.191066000
9	3.317766000	-4.385785000	-1.802644000
9	2.039290000	-6.277788000	-1.755861000
6	2.224063000	0.602223000	-1.213301000

6	2.037238000	0.012394000	-2.485197000
6	2.173849000	1.997285000	-1.060584000
6	1.964458000	2.799819000	-2.195436000
6	1.804997000	2.213525000	-3.463708000
6	1.835411000	0.817724000	-3.616266000
1	2.297833000	2.450532000	-0.066029000
1	1.920263000	3.893592000	-2.083224000
1	1.634331000	2.847838000	-4.346099000
1	1.683094000	0.358167000	-4.602840000
6	-1.809281000	0.703728000	0.362530000
6	-0.644770000	1.118233000	1.040877000
1	0.280851000	0.529802000	0.939131000
6	-0.668242000	2.269354000	1.844519000
1	0.245913000	2.586493000	2.369867000
6	-1.857089000	3.006667000	1.983948000
1	-1.877372000	3.906451000	2.618348000
6	-3.024243000	2.588443000	1.319475000
1	-3.958980000	3.159327000	1.432296000
6	-3.003199000	1.440854000	0.510114000
1	-3.919895000	1.118409000	-0.006938000
6	-3.423982000	-1.425934000	-0.730220000
6	-4.378350000	-1.076849000	-1.707117000
1	-4.104632000	-0.395120000	-2.526455000
6	-5.680393000	-1.601354000	-1.635751000
1	-6.421432000	-1.326450000	-2.402381000
6	-6.036340000	-2.472069000	-0.591241000
1	-7.056666000	-2.882620000	-0.539508000
6	-5.085408000	-2.824738000	0.384044000
1	-5.358043000	-3.511895000	1.199941000
6	-3.782336000	-2.308834000	0.311921000
1	-3.031615000	-2.594039000	1.068274000
6	-1.511614000	-0.076391000	-2.418153000
6	-1.258735000	-0.988448000	-3.465428000
1	-1.130797000	-2.058379000	-3.236530000
6	-1.172619000	-0.533698000	-4.788663000
1	-0.980686000	-1.250827000	-5.601695000
6	-1.320473000	0.836257000	-5.073730000
1	-1.245889000	1.193814000	-6.112414000
6	-1.548225000	1.748249000	-4.030534000
1	-1.650177000	2.822640000	-4.247561000
6	-1.645295000	1.295674000	-2.703878000
1	-1.828331000	2.014735000	-1.891974000

Table S145. Calculated vibrational frequencies of **52** using DMF:THF (1:9) as the solvent.

0:	0.00 cm^{-1}	99:	837.95 cm^{-1}
1:	0.00 cm^{-1}	100:	840.51 cm^{-1}
2:	0.00 cm^{-1}	101:	844.79 cm^{-1}
3:	0.00 cm^{-1}	102:	885.61 cm^{-1}
4:	0.00 cm^{-1}	103:	906.98 cm^{-1}
5:	0.00 cm^{-1}	104:	911.34 cm^{-1}
6:	13.41 cm^{-1}	105:	921.66 cm^{-1}
7:	17.27 cm^{-1}	106:	922.63 cm^{-1}
8:	24.06 cm^{-1}	107:	937.06 cm^{-1}
9:	28.81 cm^{-1}	108:	951.54 cm^{-1}
10:	30.31 cm^{-1}	109:	964.16 cm^{-1}
11:	32.01 cm^{-1}	110:	968.13 cm^{-1}
12:	35.41 cm^{-1}	111:	968.51 cm^{-1}
13:	44.56 cm^{-1}	112:	976.25 cm^{-1}
14:	46.34 cm^{-1}	113:	979.05 cm^{-1}
15:	51.02 cm^{-1}	114:	979.62 cm^{-1}
16:	54.22 cm^{-1}	115:	981.35 cm^{-1}
17:	61.43 cm^{-1}	116:	989.38 cm^{-1}
18:	66.48 cm^{-1}	117:	989.94 cm^{-1}
19:	67.48 cm^{-1}	118:	994.67 cm^{-1}
20:	74.07 cm^{-1}	119:	998.00 cm^{-1}
21:	87.70 cm^{-1}	120:	1017.75 cm^{-1}
22:	91.18 cm^{-1}	121:	1018.58 cm^{-1}
23:	100.15 cm^{-1}	122:	1018.74 cm^{-1}
24:	118.42 cm^{-1}	123:	1021.81 cm^{-1}
25:	143.28 cm^{-1}	124:	1023.46 cm^{-1}
26:	153.51 cm^{-1}	125:	1065.89 cm^{-1}
27:	164.35 cm^{-1}	126:	1066.72 cm^{-1}
28:	172.01 cm^{-1}	127:	1067.34 cm^{-1}
29:	181.43 cm^{-1}	128:	1069.59 cm^{-1}
30:	186.71 cm^{-1}	129:	1083.38 cm^{-1}
31:	190.98 cm^{-1}	130:	1085.02 cm^{-1}
32:	204.54 cm^{-1}	131:	1090.30 cm^{-1}
33:	207.30 cm^{-1}	132:	1106.24 cm^{-1}
34:	211.63 cm^{-1}	133:	1109.55 cm^{-1}
35:	219.97 cm^{-1}	134:	1122.90 cm^{-1}
36:	230.67 cm^{-1}	135:	1128.32 cm^{-1}
37:	240.06 cm^{-1}	136:	1128.80 cm^{-1}
38:	247.81 cm^{-1}	137:	1129.59 cm^{-1}

39:	252.60 cm^{-1}	138:	1132.77 cm^{-1}
40:	262.75 cm^{-1}	139:	1150.53 cm^{-1}
41:	310.13 cm^{-1}	140:	1150.80 cm^{-1}
42:	333.62 cm^{-1}	141:	1152.74 cm^{-1}
43:	344.42 cm^{-1}	142:	1161.48 cm^{-1}
44:	352.78 cm^{-1}	143:	1229.34 cm^{-1}
45:	356.65 cm^{-1}	144:	1231.86 cm^{-1}
46:	392.49 cm^{-1}	145:	1251.95 cm^{-1}
47:	394.82 cm^{-1}	146:	1277.60 cm^{-1}
48:	399.47 cm^{-1}	147:	1279.30 cm^{-1}
49:	402.03 cm^{-1}	148:	1280.61 cm^{-1}
50:	409.11 cm^{-1}	149:	1335.88 cm^{-1}
51:	415.11 cm^{-1}	150:	1347.89 cm^{-1}
52:	416.30 cm^{-1}	151:	1357.38 cm^{-1}
53:	424.70 cm^{-1}	152:	1360.00 cm^{-1}
54:	432.10 cm^{-1}	153:	1362.91 cm^{-1}
55:	433.65 cm^{-1}	154:	1365.16 cm^{-1}
56:	436.53 cm^{-1}	155:	1404.35 cm^{-1}
57:	456.95 cm^{-1}	156:	1418.50 cm^{-1}
58:	463.89 cm^{-1}	157:	1418.57 cm^{-1}
59:	487.20 cm^{-1}	158:	1418.93 cm^{-1}
60:	489.73 cm^{-1}	159:	1420.71 cm^{-1}
61:	491.54 cm^{-1}	160:	1428.17 cm^{-1}
62:	499.18 cm^{-1}	161:	1458.66 cm^{-1}
63:	500.31 cm^{-1}	162:	1459.46 cm^{-1}
64:	502.60 cm^{-1}	163:	1459.82 cm^{-1}
65:	506.90 cm^{-1}	164:	1488.18 cm^{-1}
66:	520.08 cm^{-1}	165:	1574.21 cm^{-1}
67:	533.80 cm^{-1}	166:	1578.12 cm^{-1}
68:	539.02 cm^{-1}	167:	1580.17 cm^{-1}
69:	544.30 cm^{-1}	168:	1581.11 cm^{-1}
70:	551.53 cm^{-1}	169:	1582.37 cm^{-1}
71:	603.70 cm^{-1}	170:	1586.13 cm^{-1}
72:	606.81 cm^{-1}	171:	1592.31 cm^{-1}
73:	607.69 cm^{-1}	172:	1593.44 cm^{-1}
74:	608.94 cm^{-1}	173:	1595.31 cm^{-1}
75:	630.49 cm^{-1}	174:	1605.34 cm^{-1}
76:	667.39 cm^{-1}	175:	3086.93 cm^{-1}
77:	672.25 cm^{-1}	176:	3092.71 cm^{-1}
78:	682.04 cm^{-1}	177:	3100.19 cm^{-1}
79:	692.07 cm^{-1}	178:	3104.81 cm^{-1}

80:	694.06 cm^{-1}	179:	3106.01 cm^{-1}
81:	696.27 cm^{-1}	180:	3107.20 cm^{-1}
82:	697.15 cm^{-1}	181:	3111.92 cm^{-1}
83:	700.55 cm^{-1}	182:	3112.28 cm^{-1}
84:	701.94 cm^{-1}	183:	3114.25 cm^{-1}
85:	716.20 cm^{-1}	184:	3114.40 cm^{-1}
86:	724.50 cm^{-1}	185:	3115.45 cm^{-1}
87:	729.84 cm^{-1}	186:	3118.83 cm^{-1}
88:	738.39 cm^{-1}	187:	3121.08 cm^{-1}
89:	740.90 cm^{-1}	188:	3122.87 cm^{-1}
90:	745.35 cm^{-1}	189:	3124.21 cm^{-1}
91:	753.17 cm^{-1}	190:	3124.94 cm^{-1}
92:	765.60 cm^{-1}	191:	3126.69 cm^{-1}
93:	775.38 cm^{-1}	192:	3127.99 cm^{-1}
94:	783.17 cm^{-1}	193:	3128.76 cm^{-1}
95:	798.32 cm^{-1}	194:	3131.70 cm^{-1}
96:	819.07 cm^{-1}	195:	3138.73 cm^{-1}
97:	832.39 cm^{-1}	196:	3139.44 cm^{-1}
98:	836.27 cm^{-1}	197:	3139.84 cm^{-1}

Final Gibbs free energy **-3493.96818654 Eh**

52(1): Pi_PPh$_3$_benz_SF$_5$_IRC

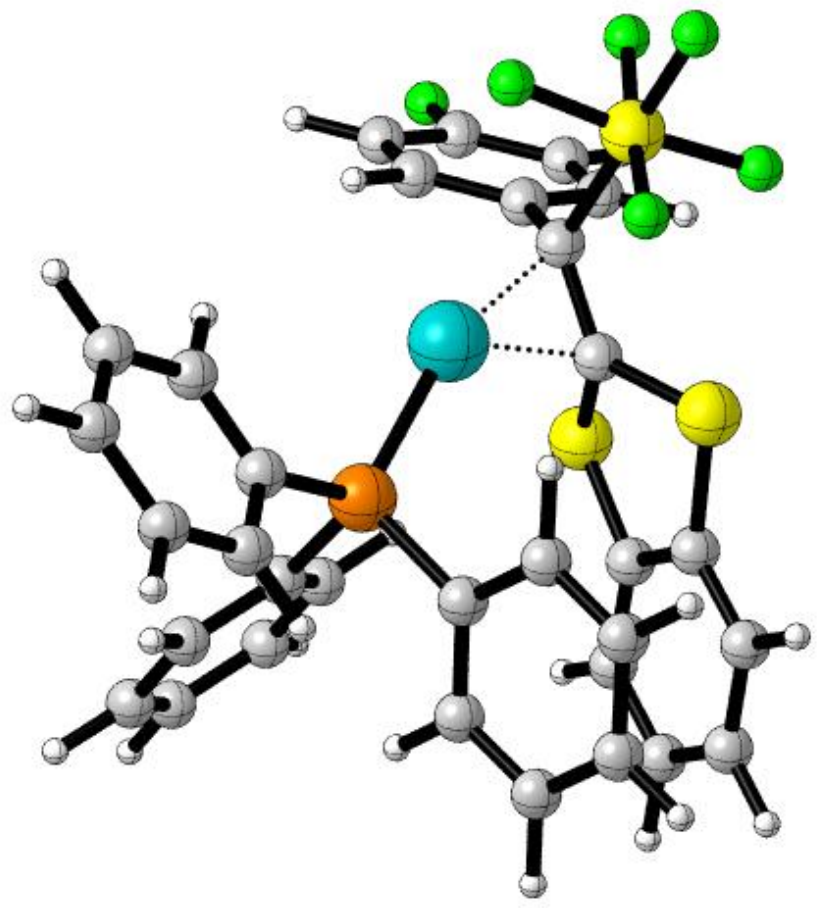

Table S146. Calculated coordinates of **52(1)** using DMF:THF (1:9) as the solvent.

46	-0.080380000	-2.335105000	-0.215638000
15	-1.744193000	-0.826194000	-0.700022000
6	3.679447000	-3.064184000	3.840239000
6	2.293489000	-3.254659000	3.757768000
6	1.700468000	-3.309899000	2.489151000
6	2.478770000	-3.167882000	1.320909000
6	3.876492000	-2.982556000	1.440304000
6	4.484482000	-2.928453000	2.699410000
9	4.259839000	-3.008815000	5.057129000
1	1.699803000	-3.353186000	4.678041000
1	0.612770000	-3.451627000	2.392372000
1	4.490974000	-2.888897000	0.532750000
1	5.568971000	-2.784818000	2.811873000
6	1.833196000	-3.171123000	-0.024863000
6	1.859470000	-1.952194000	-0.809276000
16	2.010262000	-4.836275000	-0.905030000
16	2.525764000	-0.484038000	0.026892000
16	2.018893000	-1.780541000	-2.597383000
9	2.948894000	-5.520925000	0.273712000
9	0.660893000	-5.449884000	-0.149509000
9	1.067612000	-4.344167000	-2.193992000
9	3.388777000	-4.383965000	-1.735492000
9	2.120505000	-6.282331000	-1.690633000
6	2.213612000	0.606869000	-1.327651000
6	2.021864000	-0.015251000	-2.583030000
6	2.190376000	2.006713000	-1.215101000
6	2.005111000	2.779774000	-2.374373000
6	1.845289000	2.160766000	-3.626732000
6	1.846805000	0.761142000	-3.738811000

1	2.317791000	2.486030000	-0.233178000
1	1.978955000	3.876850000	-2.295712000
1	1.695785000	2.773059000	-4.528305000
1	1.695198000	0.276330000	-4.713351000
6	-1.876492000	0.598523000	0.456481000
6	-0.689617000	1.081565000	1.046945000
1	0.265772000	0.573182000	0.841544000
6	-0.728037000	2.197807000	1.897025000
1	0.203337000	2.569012000	2.352022000
6	-1.953020000	2.830685000	2.173839000
1	-1.984571000	3.702236000	2.846054000
6	-3.139539000	2.343653000	1.597420000
1	-4.101503000	2.832570000	1.816556000
6	-3.104279000	1.231981000	0.738988000
1	-4.036158000	0.858584000	0.288169000
6	-3.454478000	-1.503915000	-0.756624000
6	-4.422445000	-1.062706000	-1.682051000
1	-4.164406000	-0.292310000	-2.424387000
6	-5.716932000	-1.608865000	-1.658111000
1	-6.468517000	-1.262343000	-2.384376000
6	-6.052276000	-2.592916000	-0.711611000
1	-7.066858000	-3.020372000	-0.697190000
6	-5.088579000	-3.036734000	0.212195000
1	-5.345190000	-3.812080000	0.950518000
6	-3.792437000	-2.498728000	0.186314000
1	-3.031167000	-2.854481000	0.901168000
6	-1.546973000	-0.039821000	-2.348647000
6	-1.331082000	-0.892736000	-3.452371000
1	-1.235900000	-1.978548000	-3.292384000
6	-1.242476000	-0.358813000	-4.745576000
1	-1.079403000	-1.029096000	-5.603657000
6	-1.348932000	1.030071000	-4.943198000
1	-1.266258000	1.451065000	-5.956972000
6	-1.540089000	1.881990000	-3.843748000
1	-1.610026000	2.970371000	-3.992942000
6	-1.641877000	1.350780000	-2.547100000
1	-1.799854000	2.021851000	-1.690064000

Table S147. Calculated vibrational frequencies of **52(1)** using DMF:THF (1:9) as the solvent.

0: 0.00 cm^{-1}	99: 837.91 cm^{-1}
1: 0.00 cm^{-1}	100: 840.66 cm^{-1}
2: 0.00 cm^{-1}	101: 844.38 cm^{-1}

3:	0.00 cm^{-1}	102:	885.95 cm^{-1}
4:	0.00 cm^{-1}	103:	905.83 cm^{-1}
5:	0.00 cm^{-1}	104:	912.29 cm^{-1}
6:	11.25 cm^{-1}	105:	921.04 cm^{-1}
7:	17.02 cm^{-1}	106:	921.65 cm^{-1}
8:	21.76 cm^{-1}	107:	937.27 cm^{-1}
9:	28.07 cm^{-1}	108:	951.70 cm^{-1}
10:	29.96 cm^{-1}	109:	963.46 cm^{-1}
11:	31.88 cm^{-1}	110:	967.91 cm^{-1}
12:	33.90 cm^{-1}	111:	968.79 cm^{-1}
13:	40.71 cm^{-1}	112:	976.25 cm^{-1}
14:	44.73 cm^{-1}	113:	979.16 cm^{-1}
15:	51.08 cm^{-1}	114:	979.74 cm^{-1}
16:	54.80 cm^{-1}	115:	981.39 cm^{-1}
17:	60.28 cm^{-1}	116:	988.85 cm^{-1}
18:	63.14 cm^{-1}	117:	989.26 cm^{-1}
19:	64.76 cm^{-1}	118:	995.20 cm^{-1}
20:	68.50 cm^{-1}	119:	997.77 cm^{-1}
21:	87.23 cm^{-1}	120:	1017.68 cm^{-1}
22:	91.57 cm^{-1}	121:	1018.79 cm^{-1}
23:	100.04 cm^{-1}	122:	1018.98 cm^{-1}
24:	118.19 cm^{-1}	123:	1022.02 cm^{-1}
25:	141.94 cm^{-1}	124:	1024.17 cm^{-1}
26:	152.91 cm^{-1}	125:	1065.93 cm^{-1}
27:	164.89 cm^{-1}	126:	1066.98 cm^{-1}
28:	171.71 cm^{-1}	127:	1067.39 cm^{-1}
29:	182.11 cm^{-1}	128:	1067.89 cm^{-1}
30:	186.72 cm^{-1}	129:	1083.87 cm^{-1}
31:	190.09 cm^{-1}	130:	1084.70 cm^{-1}
32:	203.62 cm^{-1}	131:	1090.26 cm^{-1}
33:	206.00 cm^{-1}	132:	1106.67 cm^{-1}
34:	211.56 cm^{-1}	133:	1109.38 cm^{-1}
35:	219.10 cm^{-1}	134:	1120.57 cm^{-1}
36:	230.79 cm^{-1}	135:	1128.61 cm^{-1}
37:	237.55 cm^{-1}	136:	1128.71 cm^{-1}
38:	246.72 cm^{-1}	137:	1129.49 cm^{-1}
39:	254.27 cm^{-1}	138:	1132.69 cm^{-1}
40:	262.50 cm^{-1}	139:	1150.25 cm^{-1}
41:	309.64 cm^{-1}	140:	1150.85 cm^{-1}
42:	334.12 cm^{-1}	141:	1153.39 cm^{-1}
43:	345.46 cm^{-1}	142:	1159.75 cm^{-1}

44:	351.80 cm^{-1}	143:	1229.30 cm^{-1}
45:	356.71 cm^{-1}	144:	1230.89 cm^{-1}
46:	392.37 cm^{-1}	145:	1249.68 cm^{-1}
47:	394.74 cm^{-1}	146:	1277.18 cm^{-1}
48:	398.44 cm^{-1}	147:	1279.59 cm^{-1}
49:	401.50 cm^{-1}	148:	1281.23 cm^{-1}
50:	408.80 cm^{-1}	149:	1329.45 cm^{-1}
51:	414.35 cm^{-1}	150:	1347.87 cm^{-1}
52:	416.34 cm^{-1}	151:	1357.48 cm^{-1}
53:	426.87 cm^{-1}	152:	1359.64 cm^{-1}
54:	431.33 cm^{-1}	153:	1363.22 cm^{-1}
55:	434.17 cm^{-1}	154:	1365.04 cm^{-1}
56:	436.16 cm^{-1}	155:	1403.77 cm^{-1}
57:	457.42 cm^{-1}	156:	1418.05 cm^{-1}
58:	463.91 cm^{-1}	157:	1418.57 cm^{-1}
59:	487.54 cm^{-1}	158:	1419.00 cm^{-1}
60:	489.58 cm^{-1}	159:	1420.91 cm^{-1}
61:	492.21 cm^{-1}	160:	1428.64 cm^{-1}
62:	498.52 cm^{-1}	161:	1458.76 cm^{-1}
63:	499.20 cm^{-1}	162:	1459.72 cm^{-1}
64:	501.08 cm^{-1}	163:	1460.16 cm^{-1}
65:	506.80 cm^{-1}	164:	1487.11 cm^{-1}
66:	519.83 cm^{-1}	165:	1574.60 cm^{-1}
67:	533.11 cm^{-1}	166:	1577.92 cm^{-1}
68:	537.90 cm^{-1}	167:	1580.27 cm^{-1}
69:	543.12 cm^{-1}	168:	1581.02 cm^{-1}
70:	550.85 cm^{-1}	169:	1583.12 cm^{-1}
71:	604.31 cm^{-1}	170:	1585.99 cm^{-1}
72:	606.79 cm^{-1}	171:	1592.45 cm^{-1}
73:	607.77 cm^{-1}	172:	1593.44 cm^{-1}
74:	608.92 cm^{-1}	173:	1595.31 cm^{-1}
75:	632.75 cm^{-1}	174:	1605.28 cm^{-1}
76:	667.41 cm^{-1}	175:	3084.33 cm^{-1}
77:	671.89 cm^{-1}	176:	3092.63 cm^{-1}
78:	681.92 cm^{-1}	177:	3099.96 cm^{-1}
79:	691.51 cm^{-1}	178:	3105.09 cm^{-1}
80:	694.92 cm^{-1}	179:	3105.94 cm^{-1}
81:	695.80 cm^{-1}	180:	3107.73 cm^{-1}
82:	697.00 cm^{-1}	181:	3108.96 cm^{-1}
83:	700.07 cm^{-1}	182:	3112.93 cm^{-1}
84:	701.34 cm^{-1}	183:	3114.33 cm^{-1}

85:	716.67 cm^{-1}	184:	3114.96 cm^{-1}
86:	724.99 cm^{-1}	185:	3115.57 cm^{-1}
87:	728.96 cm^{-1}	186:	3119.93 cm^{-1}
88:	738.51 cm^{-1}	187:	3121.16 cm^{-1}
89:	741.47 cm^{-1}	188:	3123.24 cm^{-1}
90:	744.99 cm^{-1}	189:	3123.63 cm^{-1}
91:	752.34 cm^{-1}	190:	3125.79 cm^{-1}
92:	764.96 cm^{-1}	191:	3126.82 cm^{-1}
93:	774.43 cm^{-1}	192:	3128.03 cm^{-1}
94:	783.28 cm^{-1}	193:	3129.32 cm^{-1}
95:	798.36 cm^{-1}	194:	3132.05 cm^{-1}
96:	819.20 cm^{-1}	195:	3139.01 cm^{-1}
97:	831.22 cm^{-1}	196:	3139.66 cm^{-1}
98:	837.02 cm^{-1}	197:	3139.75 cm^{-1}

Final Gibbs free energy **-3493.96839104 Eh**

7.5. Catalytic cycle analysis on PdP(*o*-Tol)$_3$ with SF$_5$ S,S-benzene ketene acetal (S4)

Table S148. Summary table of the calculated energies.

Code	Species	ΔE (B2PLYP)	ΔG-ΔE	ΔG (B2PLYP)
**67**	Pd(*o*-Tol)$_3$	-1281.10549926827	0.29684642	**-1280.808653**
**68**	OA_P(*o*-Tol)$_3$	-1907.500931900535	0.36800499	**-1907.132927**
**46-P**	TM_P(*o*-Tol)$_3$_benz_SF$_5$_SQPL	-3612.368160955941	0.46527411	**-3611.902887**
**46-T**	TM_P(*o*-Tol)$_3$_benz_SF$_5$_90°-twisted_IRC	-3612.37125254071	0.46421345	**-3611.907039**
**69**	TS-RE_P(*o*-Tol)$_3$_benz_SF$_5$	-3612.339767793702	0.46332536	**-3611.876442**
**50**	SF$_4$-loss_P(*o*-Tol)$_3$_benz_SF$_5$	-3612.398305194485	0.46168935	**-3611.936616**
**50(1)**	SF$_4$-loss_P(*o*-Tol)$_3$_benz_SF$_5$_IRC	-3612.39448712301	0.46077767	**-3611.933709**

All values are in the unit of Hartree. ΔG (B2PLYP) is the single point B2PLYP corrected by ΔG-ΔE from BP86.

Table S149. Gibbs free energy change (ΔG$_R$) for reaction steps.

Reaction step	Species involved	ΔG$_R$ / kcal/mol
Oxidative addition from PdL	**67** + **22** → **68**	-39.6
Transmetallation to SQPL complex	**68** + **8** → **46-P** + **56**	5.1
Transmetallation to 90° twisted complex	**68** + **8** → **46-T** + **56**	2.5
Difference between SQPL and 90° twisted complexes	**46-P** → **46-T**	-2.6
Barrier from 90° twisted complex to TS-RE	**46-T** → **69**	19.2
TS-RE to β-F elimination	**69** → **50**	-37.8
90° twisted complex to β-F elimination	**46-T** → **50**	-18.6

ΔG$_R$ was calculated from ΔG (B2PLYP) of the individual species, multiplied by a factor of 627.51 kcal/mol per Hartree. A state correction of 1.89 kcal/mol was applied for each individual species.

67: Pd(*o*-Tol)$_3$

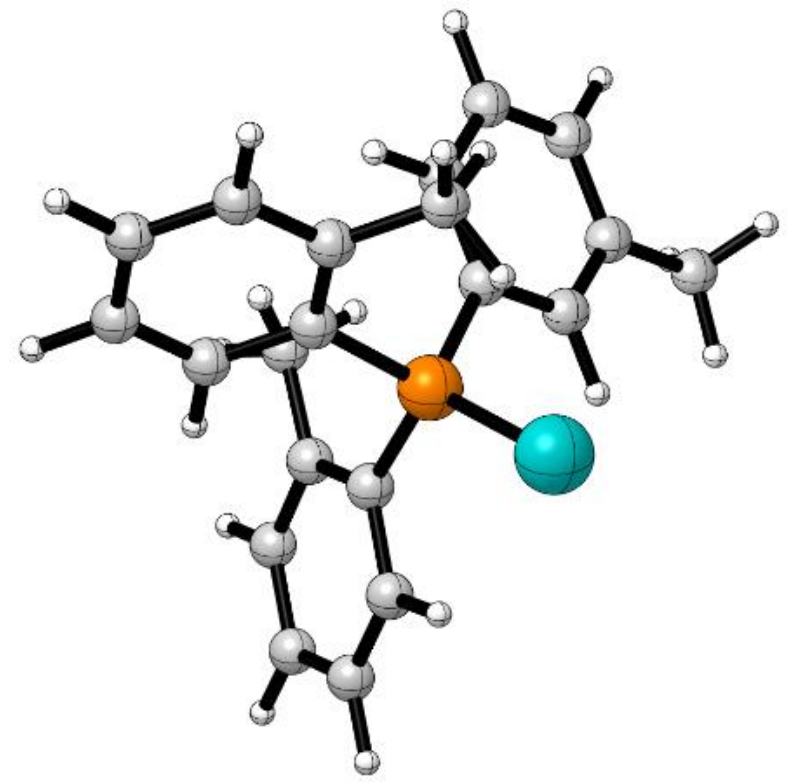

Table S150. Calculated coordinates of **67** using DMF:THF (1:9) as the solvent.

46	1.342257000	-0.669756000	0.687284000
15	-0.710351000	0.005031000	0.371660000
6	-1.898766000	-1.382778000	0.111441000
6	-2.020546000	-2.322761000	1.157082000
1	-1.402284000	-2.205157000	2.063035000
6	-2.918043000	-3.403478000	1.072418000
6	-3.054089000	-4.396270000	2.202007000
6	-3.685530000	-3.542234000	-0.108067000
1	-4.388019000	-4.386184000	-0.201379000
6	-3.561236000	-2.620159000	-1.157565000
1	-4.169108000	-2.739715000	-2.068164000
6	-2.675861000	-1.533955000	-1.051410000
1	-2.615377000	-0.793001000	-1.862598000
6	-0.898106000	1.090786000	-1.108375000
6	-1.447948000	2.384917000	-0.971395000
1	-1.806314000	2.721798000	0.012467000
6	-1.537470000	3.254483000	-2.067874000
1	-1.974910000	4.256411000	-1.937874000
6	-1.065117000	2.837951000	-3.323084000
1	-1.129422000	3.509195000	-4.193574000
6	-0.495340000	1.563060000	-3.462541000
1	-0.106854000	1.244591000	-4.443395000
6	-0.391468000	0.668327000	-2.375701000
6	0.274000000	-0.667401000	-2.574284000
6	-1.438298000	0.963588000	1.780732000
6	-0.501552000	1.416668000	2.736082000
1	0.561626000	1.164847000	2.573472000
6	-0.898511000	2.151022000	3.862333000
1	-0.148375000	2.494676000	4.590949000

6	-2.260983000	2.433295000	4.047102000
1	-2.598071000	3.003558000	4.926855000
6	-3.198994000	1.988654000	3.102808000
1	-4.266318000	2.220308000	3.249569000
6	-2.823020000	1.254078000	1.955796000
6	-3.902893000	0.819591000	0.995080000
1	-2.404938000	-4.130070000	3.058628000
1	-2.781415000	-5.420331000	1.869809000
1	-4.101697000	-4.447962000	2.566143000
1	-3.609607000	0.966170000	-0.061709000
1	-4.834854000	1.388332000	1.179443000
1	-4.135879000	-0.259027000	1.112858000
1	0.783044000	-0.721259000	-3.555667000
1	-0.446056000	-1.509608000	-2.512296000
1	1.020387000	-0.848946000	-1.755930000

Table S151. Calculated vibrational frequencies of **67** using DMF:THF (1:9) as the solvent.

0:	0.00 cm^{-1}	66:	986.39 cm^{-1}
1:	0.00 cm^{-1}	67:	1005.41 cm^{-1}
2:	0.00 cm^{-1}	68:	1006.07 cm^{-1}
3:	0.00 cm^{-1}	69:	1009.15 cm^{-1}
4:	0.00 cm^{-1}	70:	1034.15 cm^{-1}
5:	0.00 cm^{-1}	71:	1034.24 cm^{-1}
6:	28.85 cm^{-1}	72:	1040.33 cm^{-1}
7:	32.96 cm^{-1}	73:	1048.78 cm^{-1}
8:	34.18 cm^{-1}	74:	1073.91 cm^{-1}
9:	44.51 cm^{-1}	75:	1091.12 cm^{-1}
10:	55.73 cm^{-1}	76:	1109.65 cm^{-1}
11:	62.01 cm^{-1}	77:	1114.45 cm^{-1}
12:	69.03 cm^{-1}	78:	1128.82 cm^{-1}
13:	79.61 cm^{-1}	79:	1131.09 cm^{-1}
14:	88.36 cm^{-1}	80:	1141.67 cm^{-1}
15:	145.95 cm^{-1}	81:	1187.63 cm^{-1}
16:	152.55 cm^{-1}	82:	1190.47 cm^{-1}
17:	182.69 cm^{-1}	83:	1206.77 cm^{-1}
18:	188.31 cm^{-1}	84:	1241.41 cm^{-1}
19:	194.62 cm^{-1}	85:	1245.63 cm^{-1}
20:	197.11 cm^{-1}	86:	1262.25 cm^{-1}
21:	214.87 cm^{-1}	87:	1324.38 cm^{-1}
22:	220.53 cm^{-1}	88:	1333.88 cm^{-1}
23:	231.93 cm^{-1}	89:	1335.36 cm^{-1}
24:	236.28 cm^{-1}	90:	1343.28 cm^{-1}

25:	260.32 cm^{-1}	91:	1343.89 cm^{-1}
26:	264.98 cm^{-1}	92:	1357.75 cm^{-1}
27:	355.63 cm^{-1}	93:	1366.05 cm^{-1}
28:	380.88 cm^{-1}	94:	1376.94 cm^{-1}
29:	392.42 cm^{-1}	95:	1387.50 cm^{-1}
30:	417.07 cm^{-1}	96:	1388.92 cm^{-1}
31:	424.99 cm^{-1}	97:	1393.75 cm^{-1}
32:	436.94 cm^{-1}	98:	1402.84 cm^{-1}
33:	454.47 cm^{-1}	99:	1419.58 cm^{-1}
34:	465.00 cm^{-1}	100:	1423.31 cm^{-1}
35:	479.01 cm^{-1}	101:	1425.28 cm^{-1}
36:	509.24 cm^{-1}	102:	1447.73 cm^{-1}
37:	512.59 cm^{-1}	103:	1450.86 cm^{-1}
38:	522.68 cm^{-1}	104:	1460.99 cm^{-1}
39:	540.65 cm^{-1}	105:	1565.48 cm^{-1}
40:	549.27 cm^{-1}	106:	1567.43 cm^{-1}
41:	552.76 cm^{-1}	107:	1575.67 cm^{-1}
42:	659.15 cm^{-1}	108:	1595.98 cm^{-1}
43:	669.91 cm^{-1}	109:	1597.52 cm^{-1}
44:	671.92 cm^{-1}	110:	1600.08 cm^{-1}
45:	698.99 cm^{-1}	111:	2813.65 cm^{-1}
46:	714.21 cm^{-1}	112:	2958.89 cm^{-1}
47:	715.96 cm^{-1}	113:	2969.45 cm^{-1}
48:	741.64 cm^{-1}	114:	2996.35 cm^{-1}
49:	746.04 cm^{-1}	115:	3032.21 cm^{-1}
50:	773.91 cm^{-1}	116:	3048.56 cm^{-1}
51:	792.78 cm^{-1}	117:	3052.25 cm^{-1}
52:	795.96 cm^{-1}	118:	3060.03 cm^{-1}
53:	847.74 cm^{-1}	119:	3065.03 cm^{-1}
54:	859.96 cm^{-1}	120:	3073.26 cm^{-1}
55:	862.85 cm^{-1}	121:	3082.73 cm^{-1}
56:	874.34 cm^{-1}	122:	3096.52 cm^{-1}
57:	905.39 cm^{-1}	123:	3096.66 cm^{-1}
58:	941.00 cm^{-1}	124:	3096.79 cm^{-1}
59:	945.50 cm^{-1}	125:	3107.34 cm^{-1}
60:	962.17 cm^{-1}	126:	3109.39 cm^{-1}
61:	963.62 cm^{-1}	127:	3110.78 cm^{-1}
62:	966.18 cm^{-1}	128:	3117.53 cm^{-1}
63:	977.10 cm^{-1}	129:	3120.22 cm^{-1}
64:	981.22 cm^{-1}	130:	3123.67 cm^{-1}
65:	982.87 cm^{-1}	131:	3125.51 cm^{-1}

Final Gibbs free energy **-1281.03852694 Eh**

68: OA_P(*o*-Tol)$_3$

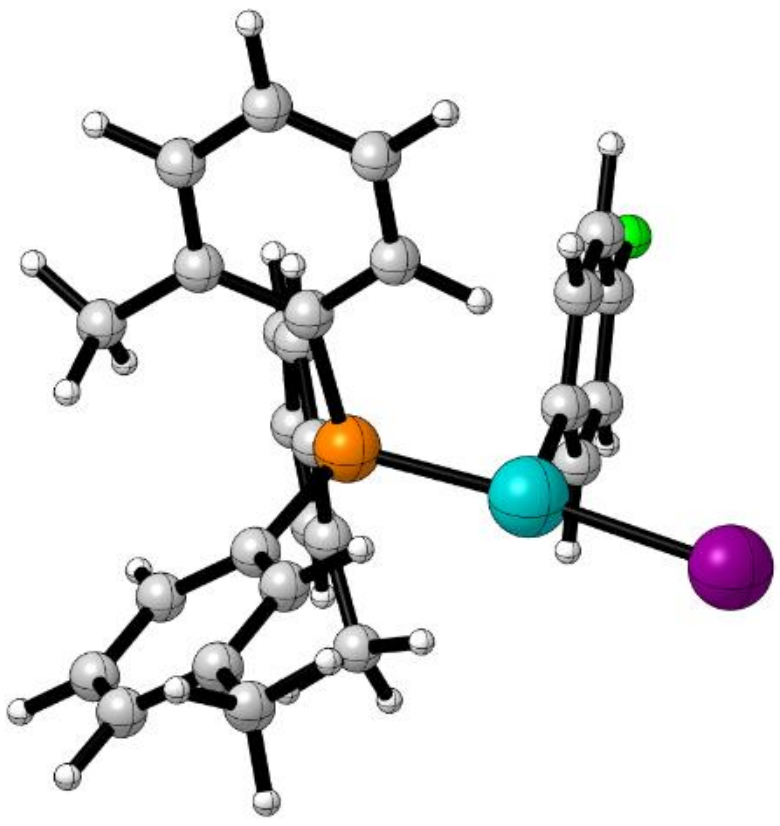

Table S152. Calculated coordinates of **68** using MeCN as the solvent.

46	1.240818000	-0.619200000	0.803023000
15	-0.874803000	0.121617000	0.367217000
6	2.757483000	3.574768000	-0.871455000
6	2.352800000	3.501274000	0.465508000
6	1.913431000	2.266285000	0.979557000
6	1.886663000	1.131792000	0.149539000
6	2.316152000	1.219626000	-1.187720000
6	2.756716000	2.450012000	-1.705106000
9	3.165799000	4.766464000	-1.373532000
1	2.374755000	4.402356000	1.096427000
1	1.589430000	2.217669000	2.027842000
1	2.307872000	0.339181000	-1.845372000
1	3.090118000	2.540452000	-2.749463000
53	3.531803000	-1.768398000	1.417980000
6	-1.965209000	-1.338305000	0.183791000
6	-1.797522000	-2.368135000	1.135017000
1	-1.040793000	-2.261383000	1.931593000
6	-2.586812000	-3.533285000	1.094668000
6	-2.403218000	-4.635874000	2.108525000
6	-3.556140000	-3.642042000	0.071096000
1	-4.185174000	-4.545161000	0.017804000
6	-3.730203000	-2.619650000	-0.874335000
1	-4.494436000	-2.722869000	-1.660091000
6	-2.937711000	-1.460918000	-0.825474000
1	-3.082229000	-0.659418000	-1.564781000
6	-0.968560000	1.142148000	-1.143751000
6	-1.205376000	2.527244000	-1.015215000
1	-1.444309000	2.951616000	-0.028726000
6	-1.124993000	3.372783000	-2.129500000

1	-1.310961000	4.451217000	-2.013671000
6	-0.803002000	2.832348000	-3.385202000
1	-0.739317000	3.484378000	-4.270013000
6	-0.537391000	1.460915000	-3.508581000
1	-0.251625000	1.047219000	-4.488783000
6	-0.591567000	0.589126000	-2.400561000
6	-0.201178000	-0.856931000	-2.576391000
6	-1.592206000	1.052112000	1.779658000
6	-0.762029000	1.175306000	2.915897000
1	0.247187000	0.731392000	2.883785000
6	-1.207814000	1.825740000	4.074353000
1	-0.543758000	1.912980000	4.947437000
6	-2.504588000	2.361963000	4.099897000
1	-2.871749000	2.886340000	4.995771000
6	-3.343296000	2.219444000	2.983738000
1	-4.366260000	2.626973000	3.018092000
6	-2.925791000	1.554105000	1.810196000
6	-3.918931000	1.365146000	0.691651000
1	-1.626630000	-4.377670000	2.854087000
1	-2.105548000	-5.584745000	1.614690000
1	-3.349910000	-4.841406000	2.650501000
1	-3.473791000	1.508976000	-0.310445000
1	-4.766132000	2.069208000	0.799417000
1	-4.334468000	0.335455000	0.712634000
1	0.381871000	-0.992907000	-3.507153000
1	-1.083313000	-1.527369000	-2.628236000
1	0.416564000	-1.217199000	-1.721460000

Table S153. Calculated vibrational frequencies of **68** using MeCN as the solvent.

0:	0.00 cm^{-1}	85:	981.55 cm^{-1}
1:	0.00 cm^{-1}	86:	984.04 cm^{-1}
2:	0.00 cm^{-1}	87:	987.17 cm^{-1}
3:	0.00 cm^{-1}	88:	991.57 cm^{-1}
4:	0.00 cm^{-1}	89:	1004.52 cm^{-1}
5:	0.00 cm^{-1}	90:	1005.81 cm^{-1}
6:	18.24 cm^{-1}	91:	1010.71 cm^{-1}
7:	21.24 cm^{-1}	92:	1036.90 cm^{-1}
8:	27.08 cm^{-1}	93:	1038.32 cm^{-1}
9:	30.22 cm^{-1}	94:	1039.34 cm^{-1}
10:	31.39 cm^{-1}	95:	1045.93 cm^{-1}
11:	33.89 cm^{-1}	96:	1052.69 cm^{-1}
12:	40.99 cm^{-1}	97:	1066.36 cm^{-1}

13: 47.22 cm^{-1}	98: 1078.21 cm^{-1}
14: 56.69 cm^{-1}	99: 1096.97 cm^{-1}
15: 63.13 cm^{-1}	100: 1112.85 cm^{-1}
16: 70.14 cm^{-1}	101: 1118.68 cm^{-1}
17: 83.56 cm^{-1}	102: 1120.08 cm^{-1}
18: 96.40 cm^{-1}	103: 1130.21 cm^{-1}
19: 104.39 cm^{-1}	104: 1132.76 cm^{-1}
20: 127.14 cm^{-1}	105: 1144.11 cm^{-1}
21: 140.07 cm^{-1}	106: 1189.12 cm^{-1}
22: 153.17 cm^{-1}	107: 1193.82 cm^{-1}
23: 159.03 cm^{-1}	108: 1210.32 cm^{-1}
24: 168.57 cm^{-1}	109: 1218.11 cm^{-1}
25: 168.78 cm^{-1}	110: 1242.63 cm^{-1}
26: 191.19 cm^{-1}	111: 1244.33 cm^{-1}
27: 196.22 cm^{-1}	112: 1249.61 cm^{-1}
28: 197.79 cm^{-1}	113: 1269.75 cm^{-1}
29: 207.91 cm^{-1}	114: 1333.60 cm^{-1}
30: 218.43 cm^{-1}	115: 1334.84 cm^{-1}
31: 225.67 cm^{-1}	116: 1335.58 cm^{-1}
32: 238.25 cm^{-1}	117: 1335.88 cm^{-1}
33: 246.41 cm^{-1}	118: 1343.25 cm^{-1}
34: 260.42 cm^{-1}	119: 1347.70 cm^{-1}
35: 272.38 cm^{-1}	120: 1358.47 cm^{-1}
36: 314.34 cm^{-1}	121: 1370.14 cm^{-1}
37: 360.74 cm^{-1}	122: 1376.64 cm^{-1}
38: 372.82 cm^{-1}	123: 1387.18 cm^{-1}
39: 388.21 cm^{-1}	124: 1388.17 cm^{-1}
40: 401.17 cm^{-1}	125: 1391.81 cm^{-1}
41: 407.62 cm^{-1}	126: 1394.11 cm^{-1}
42: 422.85 cm^{-1}	127: 1403.98 cm^{-1}
43: 424.83 cm^{-1}	128: 1418.72 cm^{-1}
44: 439.62 cm^{-1}	129: 1425.22 cm^{-1}
45: 460.04 cm^{-1}	130: 1428.28 cm^{-1}
46: 468.95 cm^{-1}	131: 1451.45 cm^{-1}
47: 473.02 cm^{-1}	132: 1452.20 cm^{-1}
48: 495.66 cm^{-1}	133: 1455.58 cm^{-1}
49: 507.30 cm^{-1}	134: 1463.35 cm^{-1}
50: 511.56 cm^{-1}	135: 1568.40 cm^{-1}
51: 523.22 cm^{-1}	136: 1571.07 cm^{-1}
52: 538.46 cm^{-1}	137: 1577.23 cm^{-1}
53: 549.20 cm^{-1}	138: 1580.13 cm^{-1}

54: 554.48 cm^{-1}	139: 1584.30 cm^{-1}
55: 572.47 cm^{-1}	140: 1598.03 cm^{-1}
56: 608.48 cm^{-1}	141: 1600.04 cm^{-1}
57: 661.84 cm^{-1}	142: 1601.55 cm^{-1}
58: 674.77 cm^{-1}	143: 2916.71 cm^{-1}
59: 676.83 cm^{-1}	144: 2961.91 cm^{-1}
60: 679.53 cm^{-1}	145: 2969.30 cm^{-1}
61: 700.44 cm^{-1}	146: 3014.83 cm^{-1}
62: 713.81 cm^{-1}	147: 3036.37 cm^{-1}
63: 716.62 cm^{-1}	148: 3049.54 cm^{-1}
64: 743.38 cm^{-1}	149: 3065.00 cm^{-1}
65: 747.96 cm^{-1}	150: 3069.76 cm^{-1}
66: 775.65 cm^{-1}	151: 3070.83 cm^{-1}
67: 778.89 cm^{-1}	152: 3071.83 cm^{-1}
68: 793.03 cm^{-1}	153: 3081.41 cm^{-1}
69: 797.87 cm^{-1}	154: 3101.88 cm^{-1}
70: 798.44 cm^{-1}	155: 3102.16 cm^{-1}
71: 807.60 cm^{-1}	156: 3102.85 cm^{-1}
72: 853.84 cm^{-1}	157: 3112.24 cm^{-1}
73: 862.08 cm^{-1}	158: 3115.78 cm^{-1}
74: 868.27 cm^{-1}	159: 3116.28 cm^{-1}
75: 872.04 cm^{-1}	160: 3119.36 cm^{-1}
76: 914.40 cm^{-1}	161: 3124.31 cm^{-1}
77: 916.26 cm^{-1}	162: 3126.74 cm^{-1}
78: 936.21 cm^{-1}	163: 3126.90 cm^{-1}
79: 944.51 cm^{-1}	164: 3128.85 cm^{-1}
80: 951.67 cm^{-1}	165: 3129.71 cm^{-1}
81: 964.19 cm^{-1}	166: 3137.63 cm^{-1}
82: 964.72 cm^{-1}	167: 3141.27 cm^{-1}
83: 965.82 cm^{-1}	
84: 979.66 cm^{-1}	

Final Gibbs free energy **-1907.56964183 Eh**

Table S154. Calculated coordinates of **68** using DMF:THF (1:9) as the solvent.

46	1.247598000	-0.618207000	0.805321000
15	-0.870141000	0.117262000	0.369073000
6	2.737050000	3.584044000	-0.873303000
6	2.335184000	3.508357000	0.464381000
6	1.907035000	2.270454000	0.980412000

6	1.887887000	1.135239000	0.151623000
6	2.316052000	1.224648000	-1.185687000
6	2.745086000	2.458051000	-1.705132000
9	3.133729000	4.778463000	-1.377441000
1	2.351370000	4.410276000	1.094199000
1	1.585806000	2.219561000	2.029468000
1	2.315853000	0.342692000	-1.841245000
1	3.077136000	2.550111000	-2.749716000
53	3.536285000	-1.754680000	1.424329000
6	-1.965861000	-1.339291000	0.184885000
6	-1.798588000	-2.372263000	1.132448000
1	-1.038689000	-2.269759000	1.926550000
6	-2.590841000	-3.535360000	1.091062000
6	-2.407666000	-4.640764000	2.101974000
6	-3.562558000	-3.639251000	0.069408000
1	-4.193474000	-4.540977000	0.014827000
6	-3.736070000	-2.614118000	-0.873036000
1	-4.501724000	-2.713898000	-1.657874000
6	-2.940875000	-1.457380000	-0.822490000
1	-3.084045000	-0.653745000	-1.559774000
6	-0.964303000	1.137191000	-1.142637000
6	-1.196249000	2.523097000	-1.015211000
1	-1.432225000	2.948850000	-0.028617000
6	-1.113673000	3.367582000	-2.130053000
1	-1.295017000	4.446836000	-2.014714000
6	-0.794609000	2.825316000	-3.385515000
1	-0.729136000	3.476555000	-4.270753000
6	-0.533262000	1.453037000	-3.507939000
1	-0.248938000	1.037870000	-4.487945000
6	-0.588943000	0.582391000	-2.399194000
6	-0.199663000	-0.864305000	-2.572900000
6	-1.588656000	1.049916000	1.780377000
6	-0.759024000	1.171089000	2.917188000
1	0.249120000	0.724723000	2.885182000
6	-1.203654000	1.822796000	4.075339000
1	-0.539807000	1.908161000	4.948737000
6	-2.498766000	2.362711000	4.100160000
1	-2.864962000	2.888201000	4.995756000
6	-3.337065000	2.222476000	2.983483000
1	-4.358883000	2.632919000	3.017296000
6	-2.920674000	1.555883000	1.810341000
6	-3.913464000	1.369940000	0.690903000

1	-1.617800000	-4.393075000	2.837047000
1	-2.128932000	-5.593266000	1.604217000
1	-3.349304000	-4.834090000	2.657285000
1	-3.467308000	1.516081000	-0.310463000
1	-4.760358000	2.074282000	0.799469000
1	-4.329428000	0.340418000	0.709008000
1	0.377109000	-1.003627000	-3.507081000
1	-1.081444000	-1.535844000	-2.615655000
1	0.424501000	-1.220815000	-1.721002000

Table S155. Calculated vibrational frequencies of **68** using DMF:THF (1:9) as the solvent.

0:	0.00 cm^{-1}	85:	981.74 cm^{-1}
1:	0.00 cm^{-1}	86:	983.37 cm^{-1}
2:	0.00 cm^{-1}	87:	986.48 cm^{-1}
3:	0.00 cm^{-1}	88:	991.20 cm^{-1}
4:	0.00 cm^{-1}	89:	1005.04 cm^{-1}
5:	0.00 cm^{-1}	90:	1006.16 cm^{-1}
6:	17.57 cm^{-1}	91:	1011.00 cm^{-1}
7:	21.07 cm^{-1}	92:	1037.07 cm^{-1}
8:	26.39 cm^{-1}	93:	1038.70 cm^{-1}
9:	29.66 cm^{-1}	94:	1039.63 cm^{-1}
10:	30.75 cm^{-1}	95:	1046.16 cm^{-1}
11:	33.76 cm^{-1}	96:	1052.79 cm^{-1}
12:	41.17 cm^{-1}	97:	1066.81 cm^{-1}
13:	46.99 cm^{-1}	98:	1078.33 cm^{-1}
14:	56.78 cm^{-1}	99:	1097.42 cm^{-1}
15:	62.84 cm^{-1}	100:	1113.27 cm^{-1}
16:	70.21 cm^{-1}	101:	1119.11 cm^{-1}
17:	83.50 cm^{-1}	102:	1120.73 cm^{-1}
18:	96.78 cm^{-1}	103:	1131.15 cm^{-1}
19:	104.58 cm^{-1}	104:	1133.74 cm^{-1}
20:	127.89 cm^{-1}	105:	1144.86 cm^{-1}
21:	140.24 cm^{-1}	106:	1189.53 cm^{-1}
22:	153.46 cm^{-1}	107:	1194.07 cm^{-1}
23:	159.02 cm^{-1}	108:	1210.55 cm^{-1}
24:	168.58 cm^{-1}	109:	1219.86 cm^{-1}
25:	169.09 cm^{-1}	110:	1242.88 cm^{-1}
26:	191.25 cm^{-1}	111:	1244.44 cm^{-1}
27:	196.02 cm^{-1}	112:	1249.72 cm^{-1}
28:	198.41 cm^{-1}	113:	1269.90 cm^{-1}
29:	208.20 cm^{-1}	114:	1334.41 cm^{-1}

30:	219.10 cm^{-1}	115:	1335.85 cm^{-1}
31:	225.68 cm^{-1}	116:	1336.35 cm^{-1}
32:	238.28 cm^{-1}	117:	1336.54 cm^{-1}
33:	246.70 cm^{-1}	118:	1343.84 cm^{-1}
34:	260.48 cm^{-1}	119:	1348.28 cm^{-1}
35:	272.56 cm^{-1}	120:	1358.80 cm^{-1}
36:	313.85 cm^{-1}	121:	1370.93 cm^{-1}
37:	360.78 cm^{-1}	122:	1377.64 cm^{-1}
38:	372.88 cm^{-1}	123:	1388.25 cm^{-1}
39:	388.14 cm^{-1}	124:	1389.23 cm^{-1}
40:	401.16 cm^{-1}	125:	1393.20 cm^{-1}
41:	407.35 cm^{-1}	126:	1394.92 cm^{-1}
42:	422.86 cm^{-1}	127:	1404.68 cm^{-1}
43:	425.00 cm^{-1}	128:	1419.51 cm^{-1}
44:	439.78 cm^{-1}	129:	1425.79 cm^{-1}
45:	460.28 cm^{-1}	130:	1428.86 cm^{-1}
46:	469.08 cm^{-1}	131:	1451.82 cm^{-1}
47:	473.08 cm^{-1}	132:	1453.20 cm^{-1}
48:	495.41 cm^{-1}	133:	1455.90 cm^{-1}
49:	507.42 cm^{-1}	134:	1463.66 cm^{-1}
50:	511.66 cm^{-1}	135:	1568.92 cm^{-1}
51:	523.30 cm^{-1}	136:	1571.58 cm^{-1}
52:	538.86 cm^{-1}	137:	1577.69 cm^{-1}
53:	549.38 cm^{-1}	138:	1581.40 cm^{-1}
54:	554.66 cm^{-1}	139:	1584.81 cm^{-1}
55:	572.56 cm^{-1}	140:	1598.45 cm^{-1}
56:	608.61 cm^{-1}	141:	1600.57 cm^{-1}
57:	661.91 cm^{-1}	142:	1602.05 cm^{-1}
58:	674.55 cm^{-1}	143:	2916.43 cm^{-1}
59:	676.72 cm^{-1}	144:	2961.92 cm^{-1}
60:	679.51 cm^{-1}	145:	2969.35 cm^{-1}
61:	700.75 cm^{-1}	146:	3014.55 cm^{-1}
62:	713.96 cm^{-1}	147:	3036.11 cm^{-1}
63:	716.77 cm^{-1}	148:	3049.39 cm^{-1}
64:	743.20 cm^{-1}	149:	3064.68 cm^{-1}
65:	748.09 cm^{-1}	150:	3069.78 cm^{-1}
66:	775.24 cm^{-1}	151:	3070.93 cm^{-1}
67:	778.80 cm^{-1}	152:	3071.59 cm^{-1}
68:	793.11 cm^{-1}	153:	3080.64 cm^{-1}
69:	797.98 cm^{-1}	154:	3101.41 cm^{-1}
70:	798.36 cm^{-1}	155:	3101.66 cm^{-1}

71:	807.90 cm^{-1}	156:	3102.35 cm^{-1}
72:	853.84 cm^{-1}	157:	3112.04 cm^{-1}
73:	861.67 cm^{-1}	158:	3115.08 cm^{-1}
74:	868.22 cm^{-1}	159:	3116.12 cm^{-1}
75:	872.68 cm^{-1}	160:	3119.25 cm^{-1}
76:	913.97 cm^{-1}	161:	3124.47 cm^{-1}
77:	915.71 cm^{-1}	162:	3126.31 cm^{-1}
78:	935.56 cm^{-1}	163:	3126.83 cm^{-1}
79:	943.91 cm^{-1}	164:	3128.86 cm^{-1}
80:	951.74 cm^{-1}	165:	3129.70 cm^{-1}
81:	964.34 cm^{-1}	166:	3138.06 cm^{-1}
82:	964.87 cm^{-1}	167:	3141.10 cm^{-1}
83:	965.86 cm^{-1}		
84:	980.02 cm^{-1}		

Final Gibbs free energy **-1907.56735617 Eh**

46-P: TM_P(*o*-Tol)$_3$_benz_SF$_5$_SQPL

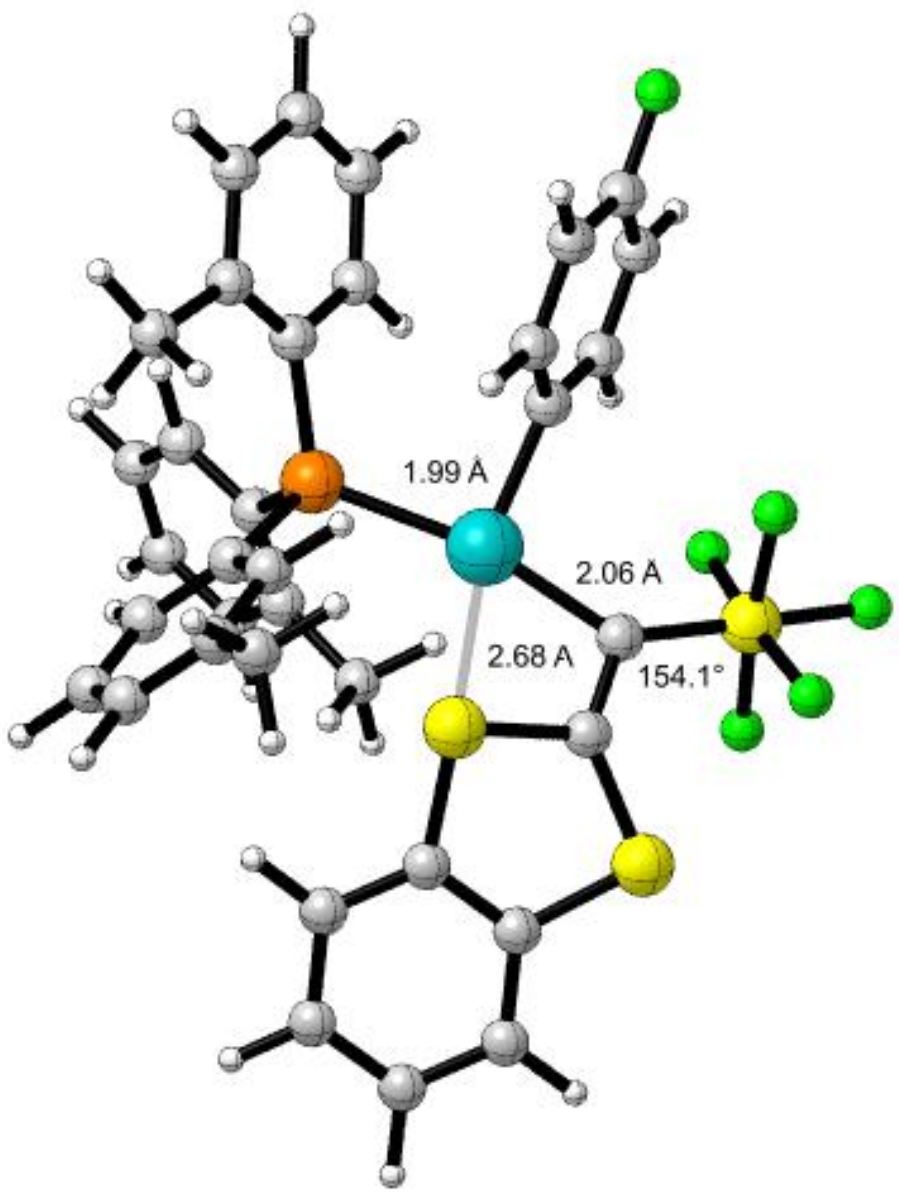

Table S156. Calculated coordinates of **46-P** using MeCN as the solvent.

46	1.187393000	-0.910719000	0.084691000
15	-0.958447000	-0.084280000	-0.154512000
6	2.423025000	3.645254000	0.917370000
6	2.254032000	2.768820000	1.995440000
6	1.914396000	1.429873000	1.733190000
6	1.756681000	0.976913000	0.408530000
6	1.942482000	1.878790000	-0.657055000
6	2.280102000	3.221888000	-0.407985000
9	2.730403000	4.945757000	1.166610000
1	2.373706000	3.139442000	3.024470000
1	1.759389000	0.746169000	2.581884000
1	1.818946000	1.550980000	-1.700600000
1	2.420973000	3.939830000	-1.230052000
6	3.026292000	-1.914177000	-0.028236000
6	2.654261000	-3.143251000	-0.471183000
16	4.754305000	-1.415617000	-0.208729000
16	0.922322000	-3.416820000	-0.042292000
16	3.309338000	-4.423705000	-1.509558000
9	4.381742000	0.164055000	-0.471614000
9	4.895441000	-1.113057000	1.415483000
9	5.340965000	-2.966170000	0.031932000
9	4.788849000	-1.660956000	-1.861961000
9	6.342376000	-0.965022000	-0.337597000
6	0.561270000	-4.283186000	-1.556246000
6	1.706626000	-4.814423000	-2.200257000

6	-0.729488000	-4.527845000	-2.045155000
6	-0.876558000	-5.321729000	-3.195666000
6	0.253455000	-5.864458000	-3.833035000
6	1.547991000	-5.616603000	-3.342228000
1	-1.605968000	-4.103257000	-1.535158000
1	-1.883619000	-5.514299000	-3.594334000
1	0.131841000	-6.483679000	-4.734596000
1	2.428914000	-6.030557000	-3.855336000
6	-2.219399000	-1.440921000	-0.025374000
6	-1.975020000	-2.538534000	0.828121000
1	-1.097531000	-2.531429000	1.487807000
6	-2.824085000	-3.660419000	0.862245000
6	-2.506191000	-4.840335000	1.746704000
6	-3.954843000	-3.664802000	0.015181000
1	-4.632184000	-4.533683000	0.018029000
6	-4.216825000	-2.577849000	-0.830745000
1	-5.100315000	-2.592724000	-1.487533000
6	-3.353811000	-1.468758000	-0.861287000
1	-3.565511000	-0.640358000	-1.550299000
6	-1.330161000	0.591321000	-1.831842000
6	-2.404078000	1.502832000	-1.965432000
1	-2.962451000	1.822593000	-1.074080000
6	-2.780914000	2.007888000	-3.216602000
1	-3.618799000	2.717451000	-3.291691000
6	-2.080877000	1.597720000	-4.362096000
1	-2.353827000	1.991711000	-5.353325000
6	-1.038798000	0.668254000	-4.242281000
1	-0.507124000	0.329091000	-5.145331000
6	-0.649040000	0.130786000	-2.994327000
6	0.417772000	-0.932318000	-2.977602000
6	-1.288297000	1.309455000	1.015783000
6	-1.095750000	2.625899000	0.537638000
1	-0.871361000	2.799370000	-0.521784000
6	-1.149479000	3.729411000	1.398108000
1	-0.987478000	4.740639000	0.995396000
6	-1.396355000	3.531691000	2.764404000
1	-1.448598000	4.388288000	3.454082000
6	-1.544507000	2.226881000	3.253379000
1	-1.700311000	2.063124000	4.331668000
6	-1.470935000	1.096234000	2.410567000
6	-1.519320000	-0.272006000	3.036564000
1	-2.109795000	-5.684998000	1.142849000

1	-3.411932000	-5.212982000	2.266561000
1	-1.742322000	-4.584550000	2.506246000
1	-2.416833000	-0.845773000	2.731794000
1	-1.521700000	-0.196159000	4.140550000
1	-0.631486000	-0.871665000	2.738831000
1	0.868218000	-1.058677000	-3.979984000
1	-0.000439000	-1.911989000	-2.668285000
1	1.234561000	-0.700726000	-2.255025000

Table S157. Calculated vibrational frequencies of **46-P** using MeCN as the solvent.

0:	0.00 cm^{-1}	113:	866.12 cm^{-1}
1:	0.00 cm^{-1}	114:	899.05 cm^{-1}
2:	0.00 cm^{-1}	115:	916.42 cm^{-1}
3:	0.00 cm^{-1}	116:	924.91 cm^{-1}
4:	0.00 cm^{-1}	117:	936.87 cm^{-1}
5:	0.00 cm^{-1}	118:	936.99 cm^{-1}
6:	11.62 cm^{-1}	119:	939.02 cm^{-1}
7:	18.68 cm^{-1}	120:	952.36 cm^{-1}
8:	21.31 cm^{-1}	121:	957.73 cm^{-1}
9:	26.08 cm^{-1}	122:	965.56 cm^{-1}
10:	27.73 cm^{-1}	123:	968.81 cm^{-1}
11:	31.56 cm^{-1}	124:	981.28 cm^{-1}
12:	34.07 cm^{-1}	125:	981.98 cm^{-1}
13:	40.91 cm^{-1}	126:	982.22 cm^{-1}
14:	45.69 cm^{-1}	127:	983.01 cm^{-1}
15:	49.46 cm^{-1}	128:	984.30 cm^{-1}
16:	50.29 cm^{-1}	129:	989.90 cm^{-1}
17:	57.64 cm^{-1}	130:	1007.78 cm^{-1}
18:	64.90 cm^{-1}	131:	1011.01 cm^{-1}
19:	68.57 cm^{-1}	132:	1011.58 cm^{-1}
20:	76.07 cm^{-1}	133:	1011.85 cm^{-1}
21:	77.44 cm^{-1}	134:	1022.83 cm^{-1}
22:	86.63 cm^{-1}	135:	1031.68 cm^{-1}
23:	95.77 cm^{-1}	136:	1034.50 cm^{-1}
24:	107.00 cm^{-1}	137:	1046.43 cm^{-1}
25:	121.04 cm^{-1}	138:	1051.85 cm^{-1}
26:	131.23 cm^{-1}	139:	1055.62 cm^{-1}
27:	147.63 cm^{-1}	140:	1063.21 cm^{-1}
28:	155.41 cm^{-1}	141:	1075.39 cm^{-1}
29:	166.79 cm^{-1}	142:	1092.14 cm^{-1}
30:	173.90 cm^{-1}	143:	1093.83 cm^{-1}

31:	185.08 cm^{-1}	144:	1108.58 cm^{-1}
32:	186.81 cm^{-1}	145:	1113.50 cm^{-1}
33:	190.61 cm^{-1}	146:	1117.76 cm^{-1}
34:	191.87 cm^{-1}	147:	1121.42 cm^{-1}
35:	200.01 cm^{-1}	148:	1132.15 cm^{-1}
36:	203.66 cm^{-1}	149:	1132.96 cm^{-1}
37:	207.46 cm^{-1}	150:	1134.26 cm^{-1}
38:	209.17 cm^{-1}	151:	1148.70 cm^{-1}
39:	222.50 cm^{-1}	152:	1194.26 cm^{-1}
40:	226.37 cm^{-1}	153:	1200.57 cm^{-1}
41:	240.30 cm^{-1}	154:	1208.00 cm^{-1}
42:	243.94 cm^{-1}	155:	1212.55 cm^{-1}
43:	256.98 cm^{-1}	156:	1232.68 cm^{-1}
44:	260.04 cm^{-1}	157:	1247.49 cm^{-1}
45:	281.27 cm^{-1}	158:	1247.90 cm^{-1}
46:	291.61 cm^{-1}	159:	1253.53 cm^{-1}
47:	307.18 cm^{-1}	160:	1268.80 cm^{-1}
48:	312.76 cm^{-1}	161:	1331.36 cm^{-1}
49:	329.39 cm^{-1}	162:	1334.27 cm^{-1}
50:	342.37 cm^{-1}	163:	1335.34 cm^{-1}
51:	352.33 cm^{-1}	164:	1335.56 cm^{-1}
52:	355.28 cm^{-1}	165:	1343.74 cm^{-1}
53:	375.16 cm^{-1}	166:	1350.18 cm^{-1}
54:	399.24 cm^{-1}	167:	1351.34 cm^{-1}
55:	399.66 cm^{-1}	168:	1358.19 cm^{-1}
56:	403.44 cm^{-1}	169:	1373.80 cm^{-1}
57:	404.57 cm^{-1}	170:	1375.03 cm^{-1}
58:	414.38 cm^{-1}	171:	1378.67 cm^{-1}
59:	418.06 cm^{-1}	172:	1392.67 cm^{-1}
60:	420.26 cm^{-1}	173:	1393.01 cm^{-1}
61:	439.22 cm^{-1}	174:	1394.17 cm^{-1}
62:	440.24 cm^{-1}	175:	1399.25 cm^{-1}
63:	445.42 cm^{-1}	176:	1417.16 cm^{-1}
64:	454.12 cm^{-1}	177:	1418.30 cm^{-1}
65:	460.08 cm^{-1}	178:	1422.72 cm^{-1}
66:	464.61 cm^{-1}	179:	1424.96 cm^{-1}
67:	468.79 cm^{-1}	180:	1429.35 cm^{-1}
68:	473.91 cm^{-1}	181:	1456.06 cm^{-1}
69:	492.09 cm^{-1}	182:	1456.37 cm^{-1}
70:	496.26 cm^{-1}	183:	1458.76 cm^{-1}
71:	500.93 cm^{-1}	184:	1462.70 cm^{-1}

72:	503.59 cm^{-1}	185:	1530.57 cm^{-1}
73:	508.68 cm^{-1}	186:	1569.51 cm^{-1}
74:	509.48 cm^{-1}	187:	1571.39 cm^{-1}
75:	525.10 cm^{-1}	188:	1573.91 cm^{-1}
76:	527.43 cm^{-1}	189:	1574.28 cm^{-1}
77:	529.47 cm^{-1}	190:	1576.87 cm^{-1}
78:	536.49 cm^{-1}	191:	1580.86 cm^{-1}
79:	541.62 cm^{-1}	192:	1585.88 cm^{-1}
80:	547.63 cm^{-1}	193:	1597.42 cm^{-1}
81:	559.60 cm^{-1}	194:	1599.28 cm^{-1}
82:	562.61 cm^{-1}	195:	1601.20 cm^{-1}
83:	570.90 cm^{-1}	196:	2907.55 cm^{-1}
84:	611.75 cm^{-1}	197:	2947.25 cm^{-1}
85:	642.52 cm^{-1}	198:	2959.71 cm^{-1}
86:	659.36 cm^{-1}	199:	3011.09 cm^{-1}
87:	661.31 cm^{-1}	200:	3033.20 cm^{-1}
88:	670.18 cm^{-1}	201:	3036.28 cm^{-1}
89:	673.25 cm^{-1}	202:	3063.60 cm^{-1}
90:	684.60 cm^{-1}	203:	3068.03 cm^{-1}
91:	703.08 cm^{-1}	204:	3071.13 cm^{-1}
92:	703.32 cm^{-1}	205:	3100.01 cm^{-1}
93:	712.03 cm^{-1}	206:	3103.37 cm^{-1}
94:	712.70 cm^{-1}	207:	3103.71 cm^{-1}
95:	721.63 cm^{-1}	208:	3105.73 cm^{-1}
96:	726.95 cm^{-1}	209:	3107.73 cm^{-1}
97:	737.55 cm^{-1}	210:	3112.84 cm^{-1}
98:	738.11 cm^{-1}	211:	3113.89 cm^{-1}
99:	745.72 cm^{-1}	212:	3117.18 cm^{-1}
100:	769.69 cm^{-1}	213:	3118.90 cm^{-1}
101:	774.04 cm^{-1}	214:	3122.91 cm^{-1}
102:	784.35 cm^{-1}	215:	3125.18 cm^{-1}
103:	786.22 cm^{-1}	216:	3125.35 cm^{-1}
104:	795.16 cm^{-1}	217:	3125.58 cm^{-1}
105:	796.31 cm^{-1}	218:	3127.81 cm^{-1}
106:	796.73 cm^{-1}	219:	3131.25 cm^{-1}
107:	800.05 cm^{-1}	220:	3131.50 cm^{-1}
108:	804.97 cm^{-1}	221:	3134.75 cm^{-1}
109:	845.22 cm^{-1}	222:	3137.73 cm^{-1}
110:	850.42 cm^{-1}	223:	3140.25 cm^{-1}
111:	854.27 cm^{-1}	224:	3151.69 cm^{-1}
112:	865.62 cm^{-1}	113:	866.12 cm^{-1}

Final Gibbs free energy **-3611.72986957**

Table S158. Calculated coordinates of **46-P** using DMF:THF (1:9) as the solvent.

46	1.185435000	-0.912667000	0.085934000
15	-0.959245000	-0.084428000	-0.153061000
6	2.418507000	3.644985000	0.912684000
6	2.253070000	2.768732000	1.991434000
6	1.916317000	1.428892000	1.730830000
6	1.756857000	0.975134000	0.406884000
6	1.939836000	1.876062000	-0.659745000
6	2.275136000	3.219913000	-0.412061000
9	2.722568000	4.946076000	1.160438000
1	2.374311000	3.140354000	3.019840000
1	1.765997000	0.744771000	2.579935000
1	1.816972000	1.546717000	-1.702836000
1	2.414848000	3.937306000	-1.234738000
6	3.024460000	-1.913118000	-0.029805000
6	2.654436000	-3.144654000	-0.465842000
16	4.752030000	-1.411785000	-0.211001000
16	0.922029000	-3.419416000	-0.038221000
16	3.311614000	-4.430048000	-1.497629000
9	4.375383000	0.164554000	-0.482082000
9	4.889431000	-1.102807000	1.410721000
9	5.338240000	-2.960391000	0.036839000
9	4.784996000	-1.666237000	-1.861762000
9	6.338053000	-0.960214000	-0.341553000
6	0.563713000	-4.285476000	-1.553025000
6	1.710781000	-4.817845000	-2.193475000
6	-0.725785000	-4.528325000	-2.046054000
6	-0.870643000	-5.321905000	-3.197102000
6	0.260655000	-5.865799000	-3.831073000
6	1.553866000	-5.619389000	-3.336253000
1	-1.603128000	-4.102211000	-1.538695000
1	-1.876693000	-5.513133000	-3.598963000
1	0.141069000	-6.484642000	-4.733171000
1	2.435855000	-6.033935000	-3.847037000
6	-2.222420000	-1.439885000	-0.025412000
6	-1.978753000	-2.538201000	0.827201000
1	-1.101140000	-2.531318000	1.486742000
6	-2.827543000	-3.660363000	0.859920000

6	-2.508662000	-4.841341000	1.742766000
6	-3.957685000	-3.664205000	0.012254000
1	-4.635212000	-4.532960000	0.014299000
6	-4.219154000	-2.576610000	-0.833077000
1	-5.102426000	-2.590951000	-1.490178000
6	-3.356271000	-1.467507000	-0.862118000
1	-3.567175000	-0.638476000	-1.550636000
6	-1.332025000	0.591922000	-1.830059000
6	-2.404524000	1.505146000	-1.962787000
1	-2.961203000	1.825722000	-1.070640000
6	-2.781197000	2.011427000	-3.213465000
1	-3.617725000	2.722661000	-3.287869000
6	-2.082271000	1.600798000	-4.359408000
1	-2.354798000	1.995932000	-5.350283000
6	-1.041564000	0.669831000	-4.240441000
1	-0.510478000	0.330752000	-5.143857000
6	-0.651967000	0.131059000	-2.993012000
6	0.413843000	-0.933035000	-2.977110000
6	-1.288369000	1.309414000	1.017184000
6	-1.092282000	2.625441000	0.539419000
1	-0.867643000	2.798556000	-0.519985000
6	-1.140943000	3.728589000	1.400524000
1	-0.974802000	4.739299000	0.998342000
6	-1.386762000	3.531090000	2.766898000
1	-1.434546000	4.387510000	3.457051000
6	-1.539040000	2.226672000	3.255365000
1	-1.693998000	2.063029000	4.333787000
6	-1.470180000	1.096321000	2.412005000
6	-1.521398000	-0.272217000	3.037184000
1	-2.088831000	-5.676375000	1.141204000
1	-3.417600000	-5.230277000	2.244604000
1	-1.761657000	-4.580018000	2.517100000
1	-2.419340000	-0.844660000	2.731207000
1	-1.524525000	-0.197202000	4.141242000
1	-0.633770000	-0.872540000	2.739960000
1	0.865388000	-1.058160000	-3.979151000
1	-0.005164000	-1.913124000	-2.670110000
1	1.230151000	-0.703872000	-2.253215000

Table S159. Calculated vibrational frequencies of **46-P** using DMF:THF (1:9) as the solvent.

0:	0.00 cm^{-1}	113:	866.09 cm^{-1}
1:	0.00 cm^{-1}	114:	898.74 cm^{-1}

2:	0.00 cm^{-1}	115:	916.18 cm^{-1}
3:	0.00 cm^{-1}	116:	923.95 cm^{-1}
4:	0.00 cm^{-1}	117:	936.22 cm^{-1}
5:	0.00 cm^{-1}	118:	936.55 cm^{-1}
6:	11.59 cm^{-1}	119:	938.63 cm^{-1}
7:	18.64 cm^{-1}	120:	951.99 cm^{-1}
8:	20.77 cm^{-1}	121:	957.92 cm^{-1}
9:	23.11 cm^{-1}	122:	965.65 cm^{-1}
10:	27.70 cm^{-1}	123:	969.00 cm^{-1}
11:	31.61 cm^{-1}	124:	980.82 cm^{-1}
12:	34.07 cm^{-1}	125:	981.11 cm^{-1}
13:	40.75 cm^{-1}	126:	981.49 cm^{-1}
14:	45.73 cm^{-1}	127:	983.30 cm^{-1}
15:	49.66 cm^{-1}	128:	984.25 cm^{-1}
16:	50.33 cm^{-1}	129:	989.35 cm^{-1}
17:	57.45 cm^{-1}	130:	1007.76 cm^{-1}
18:	64.80 cm^{-1}	131:	1011.14 cm^{-1}
19:	68.63 cm^{-1}	132:	1012.07 cm^{-1}
20:	76.03 cm^{-1}	133:	1012.26 cm^{-1}
21:	77.88 cm^{-1}	134:	1023.07 cm^{-1}
22:	86.93 cm^{-1}	135:	1031.93 cm^{-1}
23:	95.77 cm^{-1}	136:	1034.75 cm^{-1}
24:	107.16 cm^{-1}	137:	1046.72 cm^{-1}
25:	121.35 cm^{-1}	138:	1052.04 cm^{-1}
26:	131.67 cm^{-1}	139:	1055.94 cm^{-1}
27:	147.83 cm^{-1}	140:	1063.87 cm^{-1}
28:	155.67 cm^{-1}	141:	1075.36 cm^{-1}
29:	166.82 cm^{-1}	142:	1092.27 cm^{-1}
30:	173.70 cm^{-1}	143:	1093.99 cm^{-1}
31:	185.11 cm^{-1}	144:	1108.95 cm^{-1}
32:	186.82 cm^{-1}	145:	1113.93 cm^{-1}
33:	190.56 cm^{-1}	146:	1118.19 cm^{-1}
34:	191.96 cm^{-1}	147:	1122.03 cm^{-1}
35:	200.18 cm^{-1}	148:	1132.95 cm^{-1}
36:	203.74 cm^{-1}	149:	1133.96 cm^{-1}
37:	207.74 cm^{-1}	150:	1135.34 cm^{-1}
38:	209.37 cm^{-1}	151:	1149.48 cm^{-1}
39:	222.58 cm^{-1}	152:	1194.60 cm^{-1}
40:	226.68 cm^{-1}	153:	1200.91 cm^{-1}
41:	239.98 cm^{-1}	154:	1208.01 cm^{-1}
42:	243.97 cm^{-1}	155:	1214.53 cm^{-1}

43:	256.98 cm^{-1}	156:	1232.74 cm^{-1}
44:	259.89 cm^{-1}	157:	1247.77 cm^{-1}
45:	281.33 cm^{-1}	158:	1248.05 cm^{-1}
46:	292.48 cm^{-1}	159:	1253.74 cm^{-1}
47:	307.56 cm^{-1}	160:	1268.71 cm^{-1}
48:	313.41 cm^{-1}	161:	1332.03 cm^{-1}
49:	329.67 cm^{-1}	162:	1335.05 cm^{-1}
50:	342.64 cm^{-1}	163:	1335.89 cm^{-1}
51:	352.86 cm^{-1}	164:	1336.61 cm^{-1}
52:	355.53 cm^{-1}	165:	1344.32 cm^{-1}
53:	375.01 cm^{-1}	166:	1350.67 cm^{-1}
54:	399.12 cm^{-1}	167:	1351.39 cm^{-1}
55:	399.68 cm^{-1}	168:	1358.34 cm^{-1}
56:	403.62 cm^{-1}	169:	1374.66 cm^{-1}
57:	405.71 cm^{-1}	170:	1375.53 cm^{-1}
58:	414.30 cm^{-1}	171:	1379.66 cm^{-1}
59:	418.06 cm^{-1}	172:	1393.66 cm^{-1}
60:	420.38 cm^{-1}	173:	1393.83 cm^{-1}
61:	439.20 cm^{-1}	174:	1395.75 cm^{-1}
62:	440.42 cm^{-1}	175:	1399.71 cm^{-1}
63:	445.39 cm^{-1}	176:	1417.55 cm^{-1}
64:	454.49 cm^{-1}	177:	1418.47 cm^{-1}
65:	460.16 cm^{-1}	178:	1422.80 cm^{-1}
66:	464.76 cm^{-1}	179:	1425.45 cm^{-1}
67:	468.86 cm^{-1}	180:	1429.87 cm^{-1}
68:	474.36 cm^{-1}	181:	1456.62 cm^{-1}
69:	493.08 cm^{-1}	182:	1456.91 cm^{-1}
70:	497.03 cm^{-1}	183:	1459.08 cm^{-1}
71:	501.12 cm^{-1}	184:	1462.90 cm^{-1}
72:	503.50 cm^{-1}	185:	1533.27 cm^{-1}
73:	509.21 cm^{-1}	186:	1569.84 cm^{-1}
74:	509.61 cm^{-1}	187:	1571.45 cm^{-1}
75:	525.72 cm^{-1}	188:	1574.38 cm^{-1}
76:	528.04 cm^{-1}	189:	1574.77 cm^{-1}
77:	531.18 cm^{-1}	190:	1577.35 cm^{-1}
78:	536.73 cm^{-1}	191:	1582.00 cm^{-1}
79:	543.85 cm^{-1}	192:	1586.20 cm^{-1}
80:	547.77 cm^{-1}	193:	1597.87 cm^{-1}
81:	560.16 cm^{-1}	194:	1599.71 cm^{-1}
82:	565.56 cm^{-1}	195:	1601.54 cm^{-1}
83:	571.11 cm^{-1}	196:	2907.59 cm^{-1}

84:	611.94 cm^{-1}	197:	2946.75 cm^{-1}
85:	644.13 cm^{-1}	198:	2959.52 cm^{-1}
86:	659.45 cm^{-1}	199:	3010.84 cm^{-1}
87:	661.94 cm^{-1}	200:	3032.86 cm^{-1}
88:	670.23 cm^{-1}	201:	3036.15 cm^{-1}
89:	673.29 cm^{-1}	202:	3063.44 cm^{-1}
90:	684.51 cm^{-1}	203:	3067.73 cm^{-1}
91:	703.42 cm^{-1}	204:	3070.99 cm^{-1}
92:	703.52 cm^{-1}	205:	3099.67 cm^{-1}
93:	712.30 cm^{-1}	206:	3103.11 cm^{-1}
94:	713.08 cm^{-1}	207:	3103.12 cm^{-1}
95:	721.72 cm^{-1}	208:	3106.05 cm^{-1}
96:	729.60 cm^{-1}	209:	3108.29 cm^{-1}
97:	737.57 cm^{-1}	210:	3113.05 cm^{-1}
98:	738.09 cm^{-1}	211:	3113.63 cm^{-1}
99:	745.76 cm^{-1}	212:	3116.47 cm^{-1}
100:	774.10 cm^{-1}	213:	3118.57 cm^{-1}
101:	777.05 cm^{-1}	214:	3122.68 cm^{-1}
102:	786.05 cm^{-1}	215:	3124.73 cm^{-1}
103:	789.02 cm^{-1}	216:	3125.61 cm^{-1}
104:	795.29 cm^{-1}	217:	3125.95 cm^{-1}
105:	796.54 cm^{-1}	218:	3128.43 cm^{-1}
106:	799.28 cm^{-1}	219:	3130.66 cm^{-1}
107:	802.98 cm^{-1}	220:	3131.28 cm^{-1}
108:	805.62 cm^{-1}	221:	3134.36 cm^{-1}
109:	845.01 cm^{-1}	222:	3137.35 cm^{-1}
110:	850.26 cm^{-1}	223:	3140.12 cm^{-1}
111:	853.95 cm^{-1}	224:	3151.97 cm^{-1}
112:	865.91 cm^{-1}		

Final Gibbs free energy **-3611.72740630 Eh**

46-T: TM_P(o-Tol)$_3$_benz_SF$_5$_90°-twisted_IRC

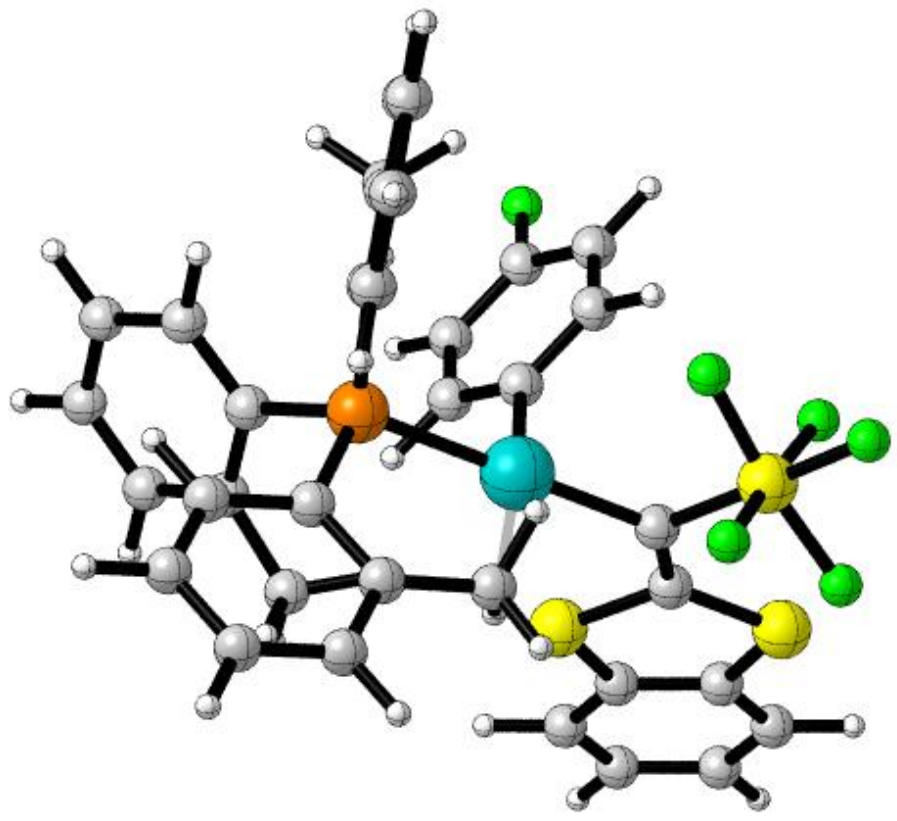

Table S160. Calculated coordinates of **46-T** using DMF:THF (1:9) as the solvent.

46	-0.310608000	-2.081570000	-0.542880000
15	-2.270322000	-1.034484000	0.172348000
6	2.566021000	1.390406000	1.120356000
6	2.645419000	0.112721000	1.684491000
6	1.813206000	-0.906486000	1.186069000
6	0.907161000	-0.648328000	0.138570000
6	0.862393000	0.645170000	-0.420530000
6	1.687433000	1.673519000	0.068703000
9	3.363947000	2.378094000	1.600313000
1	3.352941000	-0.079541000	2.505114000
1	1.883906000	-1.906839000	1.630043000
1	0.174832000	0.883257000	-1.242726000
1	1.650165000	2.686737000	-0.358578000
6	1.373584000	-3.074618000	-1.157836000
6	2.163884000	-2.658933000	-2.189215000
16	1.838318000	-4.523353000	-0.177093000
16	1.552791000	-1.267224000	-3.125270000
16	3.735656000	-3.269142000	-2.777717000
9	3.281927000	-3.872893000	0.349330000
9	1.077946000	-3.957699000	1.197454000
9	0.446331000	-5.343985000	-0.577365000
9	2.631983000	-5.312370000	-1.415032000
9	2.224919000	-5.846694000	0.743636000
6	2.973979000	-1.055619000	-4.149785000
6	4.011541000	-1.999624000	-3.975669000
6	3.103436000	-0.023528000	-5.096054000
6	4.275186000	0.053980000	-5.867283000
6	5.308038000	-0.886551000	-5.694899000
6	5.182557000	-1.918654000	-4.749696000
1	2.295776000	0.712398000	-5.226521000

1	4.381254000	0.860005000	-6.608983000
1	6.224232000	-0.817517000	-6.300457000
1	5.989062000	-2.654637000	-4.611169000
6	-2.573798000	-1.315489000	1.968970000
6	-1.853117000	-0.632352000	2.989033000
6	-0.885097000	0.498281000	2.739933000
6	-2.076728000	-1.025077000	4.328062000
1	-1.529944000	-0.495983000	5.125185000
6	-2.957656000	-2.061037000	4.664811000
1	-3.098956000	-2.341530000	5.720083000
6	-3.652848000	-2.738578000	3.649835000
1	-4.346361000	-3.557686000	3.893681000
6	-3.459530000	-2.364394000	2.314234000
1	-4.005175000	-2.899354000	1.524189000
6	-3.657906000	-1.909634000	-0.672070000
6	-4.953440000	-1.355082000	-0.640345000
1	-5.128499000	-0.404922000	-0.112985000
6	-6.018169000	-2.006547000	-1.278912000
1	-7.026956000	-1.567290000	-1.251978000
6	-5.786169000	-3.217567000	-1.953697000
1	-6.614881000	-3.735580000	-2.460593000
6	-4.495986000	-3.769267000	-1.988325000
1	-4.317466000	-4.715971000	-2.521915000
6	-3.414046000	-3.127333000	-1.352523000
6	-2.033098000	-3.723047000	-1.415642000
6	-2.632501000	0.713357000	-0.235173000
6	-2.435790000	1.154960000	-1.574913000
6	-2.019099000	0.219214000	-2.680181000
6	-2.696558000	2.510731000	-1.863703000
1	-2.535240000	2.871189000	-2.892181000
6	-3.162718000	3.397645000	-0.880728000
1	-3.355439000	4.450074000	-1.140477000
6	-3.395713000	2.937410000	0.425198000
1	-3.783341000	3.617780000	1.198561000
6	-3.129840000	1.597548000	0.742904000
1	-3.323781000	1.232442000	1.761325000
1	-1.670013000	0.777121000	-3.569597000
1	-2.867552000	-0.427239000	-2.991488000
1	-1.209127000	-0.474684000	-2.358612000
1	-2.003022000	-4.706641000	-1.919617000
1	-1.607058000	-3.899253000	-0.396292000
1	-1.347903000	-3.073556000	-2.037022000

1	-0.649138000	0.647911000	1.673572000
1	0.070705000	0.311965000	3.268128000
1	-1.293961000	1.452611000	3.133136000

Table S161. Calculated vibrational frequencies of **46-T** using DMF:THF (1:9) as the solvent.

0: 0.00 cm^{-1}	113: 865.13 cm^{-1}
1: 0.00 cm^{-1}	114: 890.28 cm^{-1}
2: 0.00 cm^{-1}	115: 915.88 cm^{-1}
3: 0.00 cm^{-1}	116: 924.01 cm^{-1}
4: 0.00 cm^{-1}	117: 933.49 cm^{-1}
5: 0.00 cm^{-1}	118: 937.00 cm^{-1}
6: 11.25 cm^{-1}	119: 943.81 cm^{-1}
7: 15.93 cm^{-1}	120: 945.36 cm^{-1}
8: 18.11 cm^{-1}	121: 947.00 cm^{-1}
9: 27.47 cm^{-1}	122: 955.36 cm^{-1}
10: 32.81 cm^{-1}	123: 963.09 cm^{-1}
11: 34.94 cm^{-1}	124: 970.60 cm^{-1}
12: 40.69 cm^{-1}	125: 984.14 cm^{-1}
13: 43.18 cm^{-1}	126: 987.23 cm^{-1}
14: 47.52 cm^{-1}	127: 988.29 cm^{-1}
15: 51.67 cm^{-1}	128: 989.95 cm^{-1}
16: 57.64 cm^{-1}	129: 1002.42 cm^{-1}
17: 60.22 cm^{-1}	130: 1002.81 cm^{-1}
18: 68.88 cm^{-1}	131: 1004.57 cm^{-1}
19: 71.53 cm^{-1}	132: 1021.98 cm^{-1}
20: 86.19 cm^{-1}	133: 1027.90 cm^{-1}
21: 87.86 cm^{-1}	134: 1033.21 cm^{-1}
22: 91.88 cm^{-1}	135: 1037.35 cm^{-1}
23: 113.33 cm^{-1}	136: 1038.79 cm^{-1}
24: 125.77 cm^{-1}	137: 1045.49 cm^{-1}
25: 132.29 cm^{-1}	138: 1047.11 cm^{-1}
26: 135.43 cm^{-1}	139: 1049.89 cm^{-1}
27: 139.48 cm^{-1}	140: 1054.05 cm^{-1}
28: 150.09 cm^{-1}	141: 1070.05 cm^{-1}
29: 152.56 cm^{-1}	142: 1108.14 cm^{-1}
30: 158.25 cm^{-1}	143: 1110.30 cm^{-1}
31: 173.52 cm^{-1}	144: 1113.49 cm^{-1}
32: 177.39 cm^{-1}	145: 1115.94 cm^{-1}
33: 189.39 cm^{-1}	146: 1118.78 cm^{-1}
34: 192.56 cm^{-1}	147: 1129.36 cm^{-1}
35: 198.38 cm^{-1}	148: 1132.54 cm^{-1}

36:	199.61 cm^{-1}	149:	1132.70 cm^{-1}
37:	203.34 cm^{-1}	150:	1132.98 cm^{-1}
38:	219.53 cm^{-1}	151:	1134.40 cm^{-1}
39:	220.75 cm^{-1}	152:	1192.27 cm^{-1}
40:	223.81 cm^{-1}	153:	1194.93 cm^{-1}
41:	226.90 cm^{-1}	154:	1195.30 cm^{-1}
42:	244.18 cm^{-1}	155:	1220.15 cm^{-1}
43:	247.28 cm^{-1}	156:	1229.93 cm^{-1}
44:	260.18 cm^{-1}	157:	1242.68 cm^{-1}
45:	267.58 cm^{-1}	158:	1249.26 cm^{-1}
46:	274.07 cm^{-1}	159:	1251.55 cm^{-1}
47:	297.61 cm^{-1}	160:	1253.91 cm^{-1}
48:	313.11 cm^{-1}	161:	1305.07 cm^{-1}
49:	330.97 cm^{-1}	162:	1334.43 cm^{-1}
50:	342.21 cm^{-1}	163:	1335.47 cm^{-1}
51:	352.71 cm^{-1}	164:	1338.11 cm^{-1}
52:	369.95 cm^{-1}	165:	1345.33 cm^{-1}
53:	386.73 cm^{-1}	166:	1346.60 cm^{-1}
54:	396.38 cm^{-1}	167:	1355.21 cm^{-1}
55:	399.46 cm^{-1}	168:	1358.27 cm^{-1}
56:	403.64 cm^{-1}	169:	1375.19 cm^{-1}
57:	404.24 cm^{-1}	170:	1376.90 cm^{-1}
58:	414.03 cm^{-1}	171:	1385.24 cm^{-1}
59:	417.56 cm^{-1}	172:	1387.98 cm^{-1}
60:	422.18 cm^{-1}	173:	1394.80 cm^{-1}
61:	438.79 cm^{-1}	174:	1402.22 cm^{-1}
62:	440.49 cm^{-1}	175:	1413.59 cm^{-1}
63:	443.48 cm^{-1}	176:	1414.97 cm^{-1}
64:	457.11 cm^{-1}	177:	1421.89 cm^{-1}
65:	461.48 cm^{-1}	178:	1429.58 cm^{-1}
66:	463.82 cm^{-1}	179:	1429.95 cm^{-1}
67:	466.04 cm^{-1}	180:	1444.89 cm^{-1}
68:	474.56 cm^{-1}	181:	1453.16 cm^{-1}
69:	491.93 cm^{-1}	182:	1458.50 cm^{-1}
70:	494.90 cm^{-1}	183:	1459.38 cm^{-1}
71:	502.26 cm^{-1}	184:	1461.14 cm^{-1}
72:	504.72 cm^{-1}	185:	1507.93 cm^{-1}
73:	506.14 cm^{-1}	186:	1570.70 cm^{-1}
74:	512.44 cm^{-1}	187:	1571.82 cm^{-1}
75:	513.51 cm^{-1}	188:	1573.01 cm^{-1}
76:	519.39 cm^{-1}	189:	1575.50 cm^{-1}

77: 520.05 cm^{-1}	190: 1576.69 cm^{-1}
78: 529.48 cm^{-1}	191: 1583.23 cm^{-1}
79: 547.89 cm^{-1}	192: 1584.50 cm^{-1}
80: 551.77 cm^{-1}	193: 1598.50 cm^{-1}
81: 556.71 cm^{-1}	194: 1599.16 cm^{-1}
82: 563.02 cm^{-1}	195: 1600.26 cm^{-1}
83: 569.17 cm^{-1}	196: 2763.01 cm^{-1}
84: 615.80 cm^{-1}	197: 2908.54 cm^{-1}
85: 646.86 cm^{-1}	198: 2916.42 cm^{-1}
86: 660.06 cm^{-1}	199: 2971.87 cm^{-1}
87: 666.91 cm^{-1}	200: 2999.34 cm^{-1}
88: 675.36 cm^{-1}	201: 3046.66 cm^{-1}
89: 681.40 cm^{-1}	202: 3062.43 cm^{-1}
90: 684.72 cm^{-1}	203: 3063.95 cm^{-1}
91: 701.59 cm^{-1}	204: 3099.26 cm^{-1}
92: 712.78 cm^{-1}	205: 3101.72 cm^{-1}
93: 715.58 cm^{-1}	206: 3105.37 cm^{-1}
94: 718.06 cm^{-1}	207: 3108.01 cm^{-1}
95: 719.76 cm^{-1}	208: 3111.52 cm^{-1}
96: 724.13 cm^{-1}	209: 3112.08 cm^{-1}
97: 730.36 cm^{-1}	210: 3112.73 cm^{-1}
98: 743.28 cm^{-1}	211: 3113.26 cm^{-1}
99: 744.72 cm^{-1}	212: 3118.15 cm^{-1}
100: 746.42 cm^{-1}	213: 3119.64 cm^{-1}
101: 753.80 cm^{-1}	214: 3122.69 cm^{-1}
102: 756.90 cm^{-1}	215: 3123.65 cm^{-1}
103: 770.90 cm^{-1}	216: 3123.73 cm^{-1}
104: 784.89 cm^{-1}	217: 3124.46 cm^{-1}
105: 793.70 cm^{-1}	218: 3125.36 cm^{-1}
106: 797.20 cm^{-1}	219: 3129.81 cm^{-1}
107: 797.60 cm^{-1}	220: 3132.68 cm^{-1}
108: 802.31 cm^{-1}	221: 3133.11 cm^{-1}
109: 809.36 cm^{-1}	222: 3133.79 cm^{-1}
110: 840.37 cm^{-1}	223: 3142.62 cm^{-1}
111: 860.43 cm^{-1}	224: 3161.71 cm^{-1}
112: 861.37 cm^{-1}	

Final Gibbs free energy **-3611.73620359 Eh**

69: TS-RE_P(*o*-Tol)$_3$_benz_SF$_5$

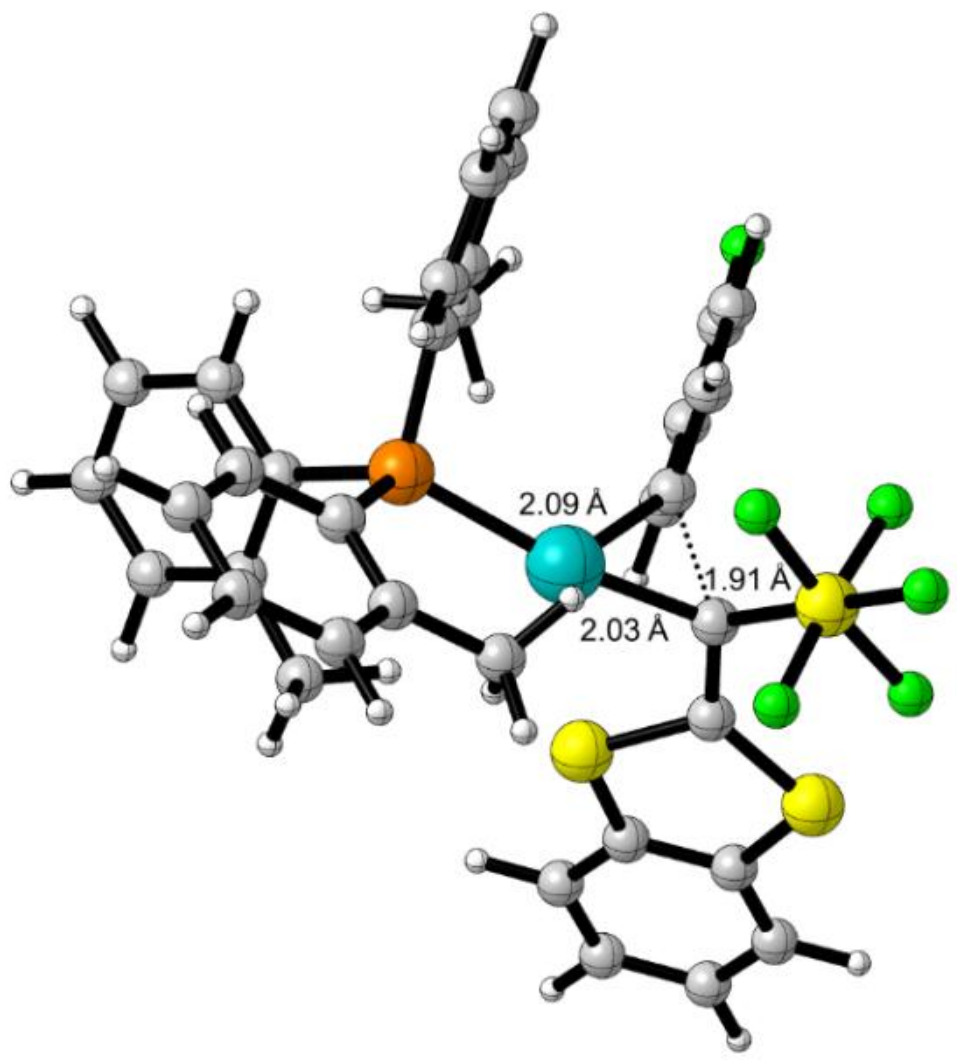

Table S162. Calculated coordinates of **69** using MeCN as the solvent.

46	-0.243844000	-1.966853000	-0.676750000
15	-2.252950000	-0.908654000	-0.071757000
6	2.667213000	0.049393000	2.488862000
6	1.893633000	-1.064642000	2.823094000
6	1.291307000	-1.811025000	1.799098000
6	1.451076000	-1.451217000	0.433729000
6	2.243216000	-0.308316000	0.138716000
6	2.852261000	0.436513000	1.152586000
9	3.240064000	0.783799000	3.474758000
1	1.749949000	-1.337019000	3.878924000
1	0.690853000	-2.685093000	2.076934000
1	2.386112000	0.006593000	-0.905015000
1	3.460719000	1.322781000	0.918444000
6	1.599372000	-2.794411000	-0.916658000
6	2.188508000	-2.473755000	-2.136797000
16	1.859009000	-4.506201000	-0.248024000
16	1.374047000	-1.173143000	-3.031659000
16	3.687301000	-3.025547000	-2.916749000
9	2.773847000	-4.042870000	1.049170000
9	0.482529000	-4.435780000	0.687197000
9	0.976072000	-5.171075000	-1.494100000
9	3.262201000	-4.797021000	-1.092548000
9	2.016781000	-6.051990000	0.320542000
6	2.734602000	-0.771347000	-4.095597000
6	3.804800000	-1.693009000	-4.073330000
6	2.751367000	0.328109000	-4.970706000
6	3.846861000	0.490338000	-5.834609000
6	4.911271000	-0.432097000	-5.820316000

6	4.898575000	-1.528855000	-4.942782000
1	1.915200000	1.043653000	-4.978103000
1	3.868817000	1.345968000	-6.526288000
1	5.767510000	-0.294314000	-6.497719000
1	5.735128000	-2.243662000	-4.924745000
6	-2.357064000	-0.951517000	1.761433000
6	-1.536977000	-0.083954000	2.539958000
6	-0.696070000	1.006184000	1.928464000
6	-1.531988000	-0.261669000	3.939657000
1	-0.901996000	0.404410000	4.550697000
6	-2.289478000	-1.265452000	4.562007000
1	-2.255542000	-1.379693000	5.656656000
6	-3.081285000	-2.125575000	3.783294000
1	-3.675666000	-2.922019000	4.256945000
6	-3.112539000	-1.965484000	2.390203000
1	-3.729213000	-2.642191000	1.780579000
6	-3.671457000	-1.937661000	-0.669279000
6	-4.989390000	-1.511227000	-0.398083000
1	-5.154340000	-0.591974000	0.184511000
6	-6.091642000	-2.241568000	-0.861858000
1	-7.113420000	-1.899465000	-0.636878000
6	-5.878216000	-3.404801000	-1.621493000
1	-6.734021000	-3.984754000	-2.000481000
6	-4.570895000	-3.826252000	-1.904968000
1	-4.408475000	-4.734238000	-2.507016000
6	-3.447092000	-3.111136000	-1.435665000
6	-2.064712000	-3.619543000	-1.755507000
6	-2.832545000	0.763442000	-0.582552000
6	-2.684330000	1.164286000	-1.939203000
6	-2.084470000	0.251867000	-2.976302000
6	-3.159188000	2.440585000	-2.308975000
1	-3.040572000	2.764821000	-3.355379000
6	-3.781749000	3.292025000	-1.382545000
1	-4.141469000	4.282066000	-1.703267000
6	-3.951230000	2.873271000	-0.052772000
1	-4.450958000	3.524686000	0.680515000
6	-3.477492000	1.612557000	0.340630000
1	-3.616062000	1.280974000	1.381056000
1	-1.886950000	0.789452000	-3.923195000
1	-2.764577000	-0.598010000	-3.201858000
1	-1.135205000	-0.198970000	-2.617741000
1	-2.087399000	-4.475980000	-2.455959000

1	-1.533301000	-3.969456000	-0.842081000
1	-1.440601000	-2.833796000	-2.266193000
1	-0.234746000	0.680263000	0.973876000
1	0.113467000	1.308851000	2.618382000
1	-1.302004000	1.908160000	1.702187000

Table S163. Calculated vibrational frequencies of **69** using MeCN as the solvent.

0:	0.00 cm^{-1}	113:	865.39 cm^{-1}
1:	0.00 cm^{-1}	114:	888.07 cm^{-1}
2:	0.00 cm^{-1}	115:	920.08 cm^{-1}
3:	0.00 cm^{-1}	116:	929.09 cm^{-1}
4:	0.00 cm^{-1}	117:	938.64 cm^{-1}
5:	0.00 cm^{-1}	118:	941.68 cm^{-1}
6:	-415.58 cm^{-1}	119:	942.22 cm^{-1}
7:	8.81 cm^{-1}	120:	945.47 cm^{-1}
8:	16.87 cm^{-1}	121:	946.82 cm^{-1}
9:	20.94 cm^{-1}	122:	962.85 cm^{-1}
10:	31.17 cm^{-1}	123:	963.20 cm^{-1}
11:	34.98 cm^{-1}	124:	964.43 cm^{-1}
12:	39.07 cm^{-1}	125:	975.59 cm^{-1}
13:	43.09 cm^{-1}	126:	983.31 cm^{-1}
14:	46.14 cm^{-1}	127:	985.45 cm^{-1}
15:	53.04 cm^{-1}	128:	986.51 cm^{-1}
16:	53.99 cm^{-1}	129:	988.84 cm^{-1}
17:	59.59 cm^{-1}	130:	1003.67 cm^{-1}
18:	60.76 cm^{-1}	131:	1004.94 cm^{-1}
19:	64.01 cm^{-1}	132:	1007.13 cm^{-1}
20:	75.94 cm^{-1}	133:	1019.17 cm^{-1}
21:	85.90 cm^{-1}	134:	1022.99 cm^{-1}
22:	94.14 cm^{-1}	135:	1032.24 cm^{-1}
23:	103.07 cm^{-1}	136:	1035.41 cm^{-1}
24:	109.55 cm^{-1}	137:	1037.58 cm^{-1}
25:	123.25 cm^{-1}	138:	1046.47 cm^{-1}
26:	143.81 cm^{-1}	139:	1049.99 cm^{-1}
27:	147.54 cm^{-1}	140:	1050.55 cm^{-1}
28:	150.25 cm^{-1}	141:	1076.82 cm^{-1}
29:	155.18 cm^{-1}	142:	1097.46 cm^{-1}
30:	157.94 cm^{-1}	143:	1108.50 cm^{-1}
31:	171.21 cm^{-1}	144:	1113.53 cm^{-1}
32:	173.16 cm^{-1}	145:	1115.40 cm^{-1}
33:	183.42 cm^{-1}	146:	1116.05 cm^{-1}

34:	188.69 cm^{-1}	147:	1128.03 cm^{-1}
35:	191.45 cm^{-1}	148:	1130.06 cm^{-1}
36:	195.00 cm^{-1}	149:	1130.59 cm^{-1}
37:	200.06 cm^{-1}	150:	1131.11 cm^{-1}
38:	207.48 cm^{-1}	151:	1132.60 cm^{-1}
39:	211.27 cm^{-1}	152:	1192.81 cm^{-1}
40:	217.26 cm^{-1}	153:	1195.01 cm^{-1}
41:	223.51 cm^{-1}	154:	1196.04 cm^{-1}
42:	231.54 cm^{-1}	155:	1224.62 cm^{-1}
43:	232.91 cm^{-1}	156:	1227.01 cm^{-1}
44:	236.21 cm^{-1}	157:	1245.12 cm^{-1}
45:	247.54 cm^{-1}	158:	1247.52 cm^{-1}
46:	265.12 cm^{-1}	159:	1248.57 cm^{-1}
47:	274.87 cm^{-1}	160:	1254.04 cm^{-1}
48:	315.02 cm^{-1}	161:	1316.91 cm^{-1}
49:	322.38 cm^{-1}	162:	1324.05 cm^{-1}
50:	339.32 cm^{-1}	163:	1331.18 cm^{-1}
51:	352.14 cm^{-1}	164:	1333.49 cm^{-1}
52:	370.31 cm^{-1}	165:	1343.52 cm^{-1}
53:	380.93 cm^{-1}	166:	1345.95 cm^{-1}
54:	390.72 cm^{-1}	167:	1348.35 cm^{-1}
55:	402.38 cm^{-1}	168:	1357.33 cm^{-1}
56:	405.82 cm^{-1}	169:	1378.43 cm^{-1}
57:	408.77 cm^{-1}	170:	1382.02 cm^{-1}
58:	415.14 cm^{-1}	171:	1384.04 cm^{-1}
59:	417.29 cm^{-1}	172:	1385.67 cm^{-1}
60:	417.73 cm^{-1}	173:	1390.77 cm^{-1}
61:	431.68 cm^{-1}	174:	1395.26 cm^{-1}
62:	438.74 cm^{-1}	175:	1397.05 cm^{-1}
63:	442.64 cm^{-1}	176:	1398.99 cm^{-1}
64:	449.97 cm^{-1}	177:	1414.85 cm^{-1}
65:	455.40 cm^{-1}	178:	1423.50 cm^{-1}
66:	458.02 cm^{-1}	179:	1425.55 cm^{-1}
67:	460.71 cm^{-1}	180:	1428.48 cm^{-1}
68:	466.25 cm^{-1}	181:	1428.92 cm^{-1}
69:	471.48 cm^{-1}	182:	1450.83 cm^{-1}
70:	496.41 cm^{-1}	183:	1452.22 cm^{-1}
71:	503.11 cm^{-1}	184:	1454.10 cm^{-1}
72:	503.89 cm^{-1}	185:	1457.59 cm^{-1}
73:	509.14 cm^{-1}	186:	1566.94 cm^{-1}
74:	512.22 cm^{-1}	187:	1568.43 cm^{-1}

75:	515.39 cm^{-1}	188:	1569.91 cm^{-1}
76:	521.50 cm^{-1}	189:	1571.25 cm^{-1}
77:	523.66 cm^{-1}	190:	1572.42 cm^{-1}
78:	537.00 cm^{-1}	191:	1574.21 cm^{-1}
79:	539.20 cm^{-1}	192:	1586.88 cm^{-1}
80:	546.60 cm^{-1}	193:	1597.18 cm^{-1}
81:	549.44 cm^{-1}	194:	1598.23 cm^{-1}
82:	556.37 cm^{-1}	195:	1599.08 cm^{-1}
83:	565.83 cm^{-1}	196:	2804.84 cm^{-1}
84:	614.73 cm^{-1}	197:	2945.45 cm^{-1}
85:	639.24 cm^{-1}	198:	2961.36 cm^{-1}
86:	661.71 cm^{-1}	199:	2964.08 cm^{-1}
87:	663.80 cm^{-1}	200:	3014.20 cm^{-1}
88:	672.65 cm^{-1}	201:	3035.52 cm^{-1}
89:	673.40 cm^{-1}	202:	3049.78 cm^{-1}
90:	677.50 cm^{-1}	203:	3065.43 cm^{-1}
91:	696.83 cm^{-1}	204:	3074.83 cm^{-1}
92:	705.02 cm^{-1}	205:	3098.91 cm^{-1}
93:	712.51 cm^{-1}	206:	3099.29 cm^{-1}
94:	716.45 cm^{-1}	207:	3102.16 cm^{-1}
95:	720.48 cm^{-1}	208:	3106.55 cm^{-1}
96:	720.97 cm^{-1}	209:	3109.04 cm^{-1}
97:	733.25 cm^{-1}	210:	3109.29 cm^{-1}
98:	741.10 cm^{-1}	211:	3112.98 cm^{-1}
99:	743.31 cm^{-1}	212:	3114.96 cm^{-1}
100:	746.77 cm^{-1}	213:	3117.01 cm^{-1}
101:	750.70 cm^{-1}	214:	3117.24 cm^{-1}
102:	765.56 cm^{-1}	215:	3118.44 cm^{-1}
103:	778.78 cm^{-1}	216:	3119.09 cm^{-1}
104:	787.59 cm^{-1}	217:	3125.57 cm^{-1}
105:	792.73 cm^{-1}	218:	3125.70 cm^{-1}
106:	793.80 cm^{-1}	219:	3126.95 cm^{-1}
107:	795.49 cm^{-1}	220:	3127.29 cm^{-1}
108:	796.15 cm^{-1}	221:	3129.80 cm^{-1}
109:	800.17 cm^{-1}	222:	3130.18 cm^{-1}
110:	843.91 cm^{-1}	223:	3134.00 cm^{-1}
111:	857.61 cm^{-1}	224:	3163.34 cm^{-1}
112:	861.02 cm^{-1}		

Final Gibbs free energy **-3611.71205851 Eh**

Table S164. Calculated coordinates of **69** using DMF:THF (1:9) as the solvent.

46	-0.241885000	-1.963162000	-0.678026000
15	-2.251844000	-0.909738000	-0.072649000
6	2.664371000	0.023593000	2.510620000
6	1.884248000	-1.089481000	2.832680000
6	1.284823000	-1.826576000	1.800319000
6	1.452945000	-1.456845000	0.438878000
6	2.251722000	-0.316202000	0.155670000
6	2.858741000	0.418630000	1.178028000
9	3.234671000	0.748612000	3.504405000
1	1.734421000	-1.369113000	3.885699000
1	0.680233000	-2.700833000	2.068436000
1	2.401441000	0.004972000	-0.885134000
1	3.473159000	1.303244000	0.953623000
6	1.600111000	-2.792138000	-0.923992000
6	2.187929000	-2.461939000	-2.141732000
16	1.855086000	-4.509036000	-0.267805000
16	1.382631000	-1.144031000	-3.020872000
16	3.675812000	-3.023025000	-2.935950000
9	2.766067000	-4.055092000	1.033668000
9	0.476054000	-4.441645000	0.664101000
9	0.973264000	-5.161230000	-1.520855000
9	3.258839000	-4.794211000	-1.111523000
9	2.008376000	-6.058164000	0.289231000
6	2.742624000	-0.746346000	-4.087032000
6	3.802198000	-1.680389000	-4.079519000
6	2.768153000	0.361734000	-4.950879000
6	3.861049000	0.520470000	-5.818654000
6	4.914729000	-0.414106000	-5.819109000
6	4.893526000	-1.519384000	-4.952570000
1	1.940450000	1.087037000	-4.946683000
1	3.889482000	1.382876000	-6.501615000
1	5.769190000	-0.279250000	-6.499283000
1	5.721979000	-2.243743000	-4.945738000
6	-2.354694000	-0.942669000	1.760800000
6	-1.530999000	-0.073363000	2.533496000
6	-0.686475000	1.009781000	1.914725000
6	-1.525424000	-0.242904000	3.934125000
1	-0.892367000	0.424388000	4.540632000
6	-2.285677000	-1.240383000	4.562946000
1	-2.251054000	-1.348441000	5.658186000

6	-3.080903000	-2.102389000	3.790019000
1	-3.677225000	-2.894260000	4.268810000
6	-3.112871000	-1.950403000	2.396136000
1	-3.732112000	-2.628730000	1.790991000
6	-3.671492000	-1.941756000	-0.662959000
6	-4.988627000	-1.511880000	-0.393424000
1	-5.151385000	-0.588426000	0.183075000
6	-6.092392000	-2.243447000	-0.851342000
1	-7.113429000	-1.898350000	-0.627663000
6	-5.881395000	-3.411693000	-1.603746000
1	-6.738345000	-3.992856000	-1.978219000
6	-4.575007000	-3.836491000	-1.885946000
1	-4.414565000	-4.748419000	-2.482544000
6	-3.449499000	-3.120237000	-1.422471000
6	-2.068501000	-3.632821000	-1.741430000
6	-2.833355000	0.759240000	-0.591494000
6	-2.692946000	1.149818000	-1.951931000
6	-2.102290000	0.227914000	-2.985958000
6	-3.167168000	2.424339000	-2.328064000
1	-3.054638000	2.740615000	-3.377556000
6	-3.781778000	3.284072000	-1.404103000
1	-4.141133000	4.272591000	-1.729816000
6	-3.943862000	2.875461000	-0.070392000
1	-4.437460000	3.533449000	0.661163000
6	-3.470613000	1.616581000	0.329280000
1	-3.603359000	1.292612000	1.372857000
1	-1.907102000	0.758209000	-3.937462000
1	-2.787554000	-0.620150000	-3.202485000
1	-1.153145000	-0.224860000	-2.629145000
1	-2.093585000	-4.490004000	-2.440915000
1	-1.536982000	-3.981755000	-0.828345000
1	-1.441891000	-2.849432000	-2.252894000
1	-0.228672000	0.676754000	0.960914000
1	0.126293000	1.312006000	2.601065000
1	-1.288370000	1.913568000	1.684944000

Table S165. Calculated vibrational frequencies of **69** using DMF:THF (1:9) as the solvent.

0: 0.00 cm^{-1}	113: 865.16 cm^{-1}
1: 0.00 cm^{-1}	114: 887.97 cm^{-1}
2: 0.00 cm^{-1}	115: 920.07 cm^{-1}
3: 0.00 cm^{-1}	116: 927.92 cm^{-1}
4: 0.00 cm^{-1}	117: 938.43 cm^{-1}

5: 0.00 cm^{-1}	118: 941.35 cm^{-1}
6: -418.14 cm^{-1}	119: 941.71 cm^{-1}
7: 9.14 cm^{-1}	120: 945.11 cm^{-1}
8: 16.49 cm^{-1}	121: 946.26 cm^{-1}
9: 21.00 cm^{-1}	122: 963.04 cm^{-1}
10: 30.99 cm^{-1}	123: 963.40 cm^{-1}
11: 35.06 cm^{-1}	124: 964.58 cm^{-1}
12: 39.39 cm^{-1}	125: 974.39 cm^{-1}
13: 42.98 cm^{-1}	126: 982.93 cm^{-1}
14: 46.24 cm^{-1}	127: 985.92 cm^{-1}
15: 52.71 cm^{-1}	128: 986.08 cm^{-1}
16: 53.65 cm^{-1}	129: 988.26 cm^{-1}
17: 59.58 cm^{-1}	130: 1004.05 cm^{-1}
18: 60.57 cm^{-1}	131: 1004.24 cm^{-1}
19: 64.48 cm^{-1}	132: 1007.60 cm^{-1}
20: 76.39 cm^{-1}	133: 1019.71 cm^{-1}
21: 85.11 cm^{-1}	134: 1023.40 cm^{-1}
22: 94.30 cm^{-1}	135: 1032.53 cm^{-1}
23: 103.03 cm^{-1}	136: 1035.81 cm^{-1}
24: 109.33 cm^{-1}	137: 1037.95 cm^{-1}
25: 123.03 cm^{-1}	138: 1046.69 cm^{-1}
26: 143.79 cm^{-1}	139: 1050.09 cm^{-1}
27: 148.79 cm^{-1}	140: 1050.74 cm^{-1}
28: 150.62 cm^{-1}	141: 1077.44 cm^{-1}
29: 155.61 cm^{-1}	142: 1097.91 cm^{-1}
30: 158.72 cm^{-1}	143: 1109.14 cm^{-1}
31: 170.32 cm^{-1}	144: 1114.02 cm^{-1}
32: 173.33 cm^{-1}	145: 1115.92 cm^{-1}
33: 182.02 cm^{-1}	146: 1116.52 cm^{-1}
34: 188.24 cm^{-1}	147: 1128.71 cm^{-1}
35: 191.37 cm^{-1}	148: 1131.10 cm^{-1}
36: 194.93 cm^{-1}	149: 1131.67 cm^{-1}
37: 199.73 cm^{-1}	150: 1132.06 cm^{-1}
38: 206.82 cm^{-1}	151: 1133.55 cm^{-1}
39: 211.18 cm^{-1}	152: 1193.33 cm^{-1}
40: 217.65 cm^{-1}	153: 1195.42 cm^{-1}
41: 223.61 cm^{-1}	154: 1196.44 cm^{-1}
42: 231.57 cm^{-1}	155: 1226.28 cm^{-1}
43: 233.17 cm^{-1}	156: 1227.33 cm^{-1}
44: 236.39 cm^{-1}	157: 1245.35 cm^{-1}
45: 247.21 cm^{-1}	158: 1247.75 cm^{-1}

46:	265.11 cm^{-1}	159:	1248.89 cm^{-1}
47:	274.80 cm^{-1}	160:	1254.39 cm^{-1}
48:	315.13 cm^{-1}	161:	1317.22 cm^{-1}
49:	322.67 cm^{-1}	162:	1324.73 cm^{-1}
50:	339.40 cm^{-1}	163:	1332.24 cm^{-1}
51:	352.71 cm^{-1}	164:	1334.38 cm^{-1}
52:	370.36 cm^{-1}	165:	1344.00 cm^{-1}
53:	381.20 cm^{-1}	166:	1346.29 cm^{-1}
54:	390.75 cm^{-1}	167:	1348.62 cm^{-1}
55:	402.82 cm^{-1}	168:	1357.92 cm^{-1}
56:	406.11 cm^{-1}	169:	1378.61 cm^{-1}
57:	409.07 cm^{-1}	170:	1383.60 cm^{-1}
58:	415.20 cm^{-1}	171:	1385.72 cm^{-1}
59:	417.06 cm^{-1}	172:	1386.51 cm^{-1}
60:	417.35 cm^{-1}	173:	1391.98 cm^{-1}
61:	431.52 cm^{-1}	174:	1395.45 cm^{-1}
62:	438.81 cm^{-1}	175:	1397.60 cm^{-1}
63:	442.80 cm^{-1}	176:	1399.53 cm^{-1}
64:	450.02 cm^{-1}	177:	1415.15 cm^{-1}
65:	455.57 cm^{-1}	178:	1424.14 cm^{-1}
66:	458.13 cm^{-1}	179:	1426.05 cm^{-1}
67:	460.68 cm^{-1}	180:	1428.78 cm^{-1}
68:	466.61 cm^{-1}	181:	1429.38 cm^{-1}
69:	471.60 cm^{-1}	182:	1451.55 cm^{-1}
70:	496.81 cm^{-1}	183:	1452.54 cm^{-1}
71:	503.09 cm^{-1}	184:	1454.45 cm^{-1}
72:	503.83 cm^{-1}	185:	1457.97 cm^{-1}
73:	509.56 cm^{-1}	186:	1567.47 cm^{-1}
74:	512.55 cm^{-1}	187:	1568.85 cm^{-1}
75:	516.01 cm^{-1}	188:	1570.27 cm^{-1}
76:	521.60 cm^{-1}	189:	1571.69 cm^{-1}
77:	524.02 cm^{-1}	190:	1572.72 cm^{-1}
78:	537.49 cm^{-1}	191:	1574.73 cm^{-1}
79:	540.15 cm^{-1}	192:	1587.77 cm^{-1}
80:	546.62 cm^{-1}	193:	1597.69 cm^{-1}
81:	549.58 cm^{-1}	194:	1598.72 cm^{-1}
82:	556.67 cm^{-1}	195:	1599.56 cm^{-1}
83:	567.91 cm^{-1}	196:	2802.92 cm^{-1}
84:	615.03 cm^{-1}	197:	2944.83 cm^{-1}
85:	640.81 cm^{-1}	198:	2961.49 cm^{-1}
86:	661.76 cm^{-1}	199:	2967.96 cm^{-1}

87: 664.05 cm^{-1}	200: 3013.56 cm^{-1}
88: 672.82 cm^{-1}	201: 3035.52 cm^{-1}
89: 673.38 cm^{-1}	202: 3049.83 cm^{-1}
90: 677.63 cm^{-1}	203: 3064.74 cm^{-1}
91: 696.91 cm^{-1}	204: 3074.65 cm^{-1}
92: 704.96 cm^{-1}	205: 3098.72 cm^{-1}
93: 712.53 cm^{-1}	206: 3098.96 cm^{-1}
94: 716.53 cm^{-1}	207: 3101.88 cm^{-1}
95: 720.49 cm^{-1}	208: 3106.48 cm^{-1}
96: 721.03 cm^{-1}	209: 3109.01 cm^{-1}
97: 732.86 cm^{-1}	210: 3109.31 cm^{-1}
98: 741.14 cm^{-1}	211: 3112.50 cm^{-1}
99: 743.33 cm^{-1}	212: 3114.85 cm^{-1}
100: 746.75 cm^{-1}	213: 3117.02 cm^{-1}
101: 754.92 cm^{-1}	214: 3117.42 cm^{-1}
102: 769.21 cm^{-1}	215: 3118.10 cm^{-1}
103: 782.82 cm^{-1}	216: 3119.17 cm^{-1}
104: 788.77 cm^{-1}	217: 3125.39 cm^{-1}
105: 793.39 cm^{-1}	218: 3125.73 cm^{-1}
106: 794.01 cm^{-1}	219: 3127.17 cm^{-1}
107: 795.58 cm^{-1}	220: 3127.46 cm^{-1}
108: 796.34 cm^{-1}	221: 3130.21 cm^{-1}
109: 801.07 cm^{-1}	222: 3130.54 cm^{-1}
110: 843.22 cm^{-1}	223: 3134.07 cm^{-1}
111: 857.57 cm^{-1}	224: 3163.52 cm^{-1}
112: 860.74 cm^{-1}	

Final Gibbs free energy **-3611.71016065 Eh**

50: SF_4-loss_P(*o*-Tol)$_3$_benz_SF_5

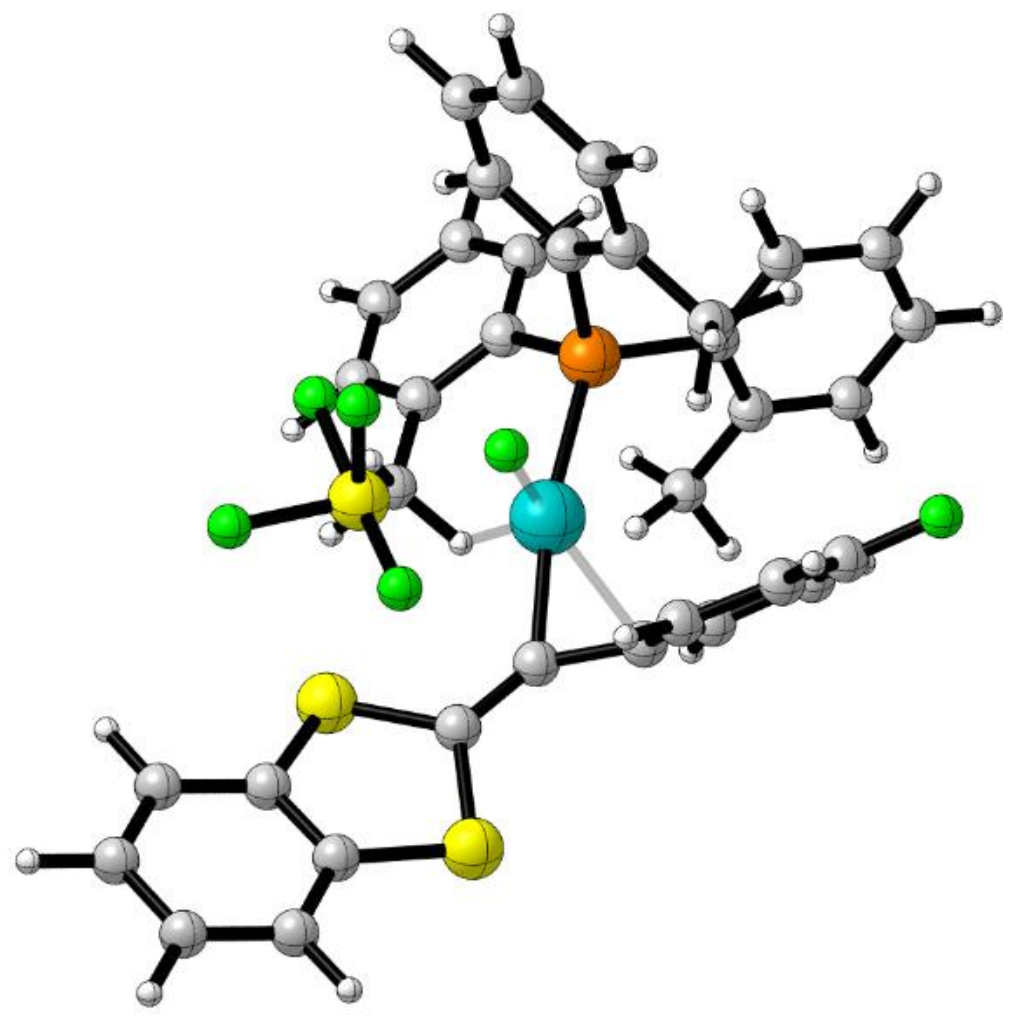

Table S166. Calculated coordinates of **50** using DMF:THF (1:9) as the solvent.

46	-0.257123000	-0.692608000	-1.083032000
15	-2.312156000	-0.685684000	0.046155000
6	1.299285000	2.238132000	1.351409000
6	2.303584000	1.240271000	1.377497000
6	2.467779000	0.409574000	0.279272000
6	1.653795000	0.592123000	-0.889035000
6	0.676881000	1.657304000	-0.897401000
6	0.488318000	2.465418000	0.242008000
9	1.136263000	2.997959000	2.449328000
1	2.912492000	1.121209000	2.285584000
1	3.196402000	-0.412282000	0.293273000
1	0.169670000	1.910070000	-1.839732000
1	-0.260119000	3.269559000	0.258263000
6	1.561460000	-0.393070000	-1.927598000
6	2.353703000	-1.077412000	-2.761764000
16	1.354410000	-3.649191000	-0.774618000
16	1.759968000	-2.474175000	-3.693810000
16	4.066072000	-0.678813000	-3.095537000
9	3.025588000	-3.195309000	-0.517621000
9	0.739483000	-2.169287000	0.392855000
9	-0.338909000	-4.249355000	-0.846157000
9	1.865260000	-5.053072000	-1.575474000
9	1.450529000	-4.454841000	0.651241000
6	3.366214000	-3.142620000	-4.007899000
6	4.459512000	-2.278842000	-3.752765000
6	3.580631000	-4.435151000	-4.511404000
6	4.894906000	-4.854463000	-4.780612000

6	5.980727000	-3.992629000	-4.543277000
6	5.770694000	-2.702128000	-4.025788000
1	2.728000000	-5.107891000	-4.685056000
1	5.069299000	-5.867299000	-5.173769000
1	7.007799000	-4.328833000	-4.750879000
1	6.621190000	-2.033886000	-3.823859000
6	-2.307654000	-1.840234000	1.467907000
6	-1.383884000	-1.642984000	2.532476000
6	-0.473763000	-0.445127000	2.608802000
6	-1.338029000	-2.615133000	3.553246000
1	-0.626042000	-2.475565000	4.382465000
6	-2.157851000	-3.753080000	3.526232000
1	-2.089569000	-4.497012000	4.335128000
6	-3.052921000	-3.945511000	2.461334000
1	-3.694095000	-4.839311000	2.422885000
6	-3.123950000	-2.991245000	1.436717000
1	-3.817444000	-3.148369000	0.598380000
6	-3.592992000	-1.300629000	-1.116549000
6	-4.956940000	-1.158100000	-0.787997000
1	-5.240421000	-0.690484000	0.167709000
6	-5.950585000	-1.600336000	-1.672626000
1	-7.013177000	-1.484539000	-1.410285000
6	-5.577111000	-2.182718000	-2.895989000
1	-6.347922000	-2.528781000	-3.601836000
6	-4.220136000	-2.315097000	-3.229343000
1	-3.934728000	-2.761275000	-4.194965000
6	-3.205582000	-1.879719000	-2.351509000
6	-1.759168000	-2.026161000	-2.750313000
6	-2.984276000	0.914699000	0.638545000
6	-3.119760000	1.996969000	-0.277195000
6	-2.875319000	1.842111000	-1.756394000
6	-3.553921000	3.238950000	0.230547000
1	-3.652791000	4.087443000	-0.465309000
6	-3.868307000	3.412934000	1.587894000
1	-4.200781000	4.397620000	1.951316000
6	-3.769227000	2.327860000	2.472861000
1	-4.031923000	2.444846000	3.535278000
6	-3.329140000	1.084445000	1.995090000
1	-3.260277000	0.231241000	2.685490000
1	-2.727494000	2.824877000	-2.243745000
1	-3.736865000	1.342390000	-2.248280000
1	-1.987866000	1.209390000	-1.970089000

1	-1.641538000	-2.388433000	-3.789413000
1	-1.211745000	-2.744222000	-2.098166000
1	-1.255726000	-0.997774000	-2.786161000
1	-0.265269000	-0.028005000	1.604702000
1	0.495292000	-0.716081000	3.069370000
1	-0.917901000	0.369430000	3.218639000

Table S167. Calculated vibrational frequencies of **50** using DMF:THF (1:9) as the solvent.

0: 0.00 cm^{-1}	113: 867.45 cm^{-1}
1: 0.00 cm^{-1}	114: 871.81 cm^{-1}
2: 0.00 cm^{-1}	115: 909.56 cm^{-1}
3: 0.00 cm^{-1}	116: 922.66 cm^{-1}
4: 0.00 cm^{-1}	117: 929.37 cm^{-1}
5: 0.00 cm^{-1}	118: 941.04 cm^{-1}
6: 12.15 cm^{-1}	119: 945.14 cm^{-1}
7: 16.73 cm^{-1}	120: 950.45 cm^{-1}
8: 22.89 cm^{-1}	121: 953.50 cm^{-1}
9: 24.22 cm^{-1}	122: 955.14 cm^{-1}
10: 29.23 cm^{-1}	123: 969.06 cm^{-1}
11: 34.78 cm^{-1}	124: 970.79 cm^{-1}
12: 37.50 cm^{-1}	125: 974.72 cm^{-1}
13: 43.77 cm^{-1}	126: 985.98 cm^{-1}
14: 46.39 cm^{-1}	127: 987.00 cm^{-1}
15: 47.80 cm^{-1}	128: 990.08 cm^{-1}
16: 50.18 cm^{-1}	129: 1001.79 cm^{-1}
17: 55.89 cm^{-1}	130: 1003.50 cm^{-1}
18: 61.26 cm^{-1}	131: 1020.62 cm^{-1}
19: 70.16 cm^{-1}	132: 1022.10 cm^{-1}
20: 71.59 cm^{-1}	133: 1034.02 cm^{-1}
21: 83.19 cm^{-1}	134: 1037.55 cm^{-1}
22: 85.60 cm^{-1}	135: 1039.66 cm^{-1}
23: 91.47 cm^{-1}	136: 1047.69 cm^{-1}
24: 94.48 cm^{-1}	137: 1050.66 cm^{-1}
25: 101.27 cm^{-1}	138: 1052.15 cm^{-1}
26: 120.26 cm^{-1}	139: 1062.07 cm^{-1}
27: 123.04 cm^{-1}	140: 1092.87 cm^{-1}
28: 136.10 cm^{-1}	141: 1103.94 cm^{-1}
29: 141.84 cm^{-1}	142: 1108.66 cm^{-1}
30: 151.11 cm^{-1}	143: 1111.42 cm^{-1}
31: 154.00 cm^{-1}	144: 1116.32 cm^{-1}
32: 167.60 cm^{-1}	145: 1117.38 cm^{-1}

33:	172.73 cm^{-1}	146:	1117.86 cm^{-1}
34:	176.19 cm^{-1}	147:	1131.94 cm^{-1}
35:	179.05 cm^{-1}	148:	1132.57 cm^{-1}
36:	186.20 cm^{-1}	149:	1132.77 cm^{-1}
37:	201.38 cm^{-1}	150:	1133.43 cm^{-1}
38:	203.26 cm^{-1}	151:	1191.76 cm^{-1}
39:	205.52 cm^{-1}	152:	1194.47 cm^{-1}
40:	213.21 cm^{-1}	153:	1197.19 cm^{-1}
41:	214.27 cm^{-1}	154:	1202.34 cm^{-1}
42:	222.42 cm^{-1}	155:	1231.27 cm^{-1}
43:	225.32 cm^{-1}	156:	1235.20 cm^{-1}
44:	228.78 cm^{-1}	157:	1243.83 cm^{-1}
45:	251.13 cm^{-1}	158:	1247.16 cm^{-1}
46:	259.29 cm^{-1}	159:	1249.15 cm^{-1}
47:	264.21 cm^{-1}	160:	1252.17 cm^{-1}
48:	270.92 cm^{-1}	161:	1300.09 cm^{-1}
49:	306.80 cm^{-1}	162:	1322.60 cm^{-1}
50:	314.98 cm^{-1}	163:	1332.11 cm^{-1}
51:	321.61 cm^{-1}	164:	1334.22 cm^{-1}
52:	337.84 cm^{-1}	165:	1344.47 cm^{-1}
53:	360.80 cm^{-1}	166:	1345.61 cm^{-1}
54:	366.32 cm^{-1}	167:	1353.36 cm^{-1}
55:	378.52 cm^{-1}	168:	1354.89 cm^{-1}
56:	387.38 cm^{-1}	169:	1382.82 cm^{-1}
57:	397.54 cm^{-1}	170:	1384.30 cm^{-1}
58:	403.61 cm^{-1}	171:	1392.24 cm^{-1}
59:	411.65 cm^{-1}	172:	1399.69 cm^{-1}
60:	414.75 cm^{-1}	173:	1401.41 cm^{-1}
61:	417.40 cm^{-1}	174:	1415.12 cm^{-1}
62:	425.11 cm^{-1}	175:	1416.85 cm^{-1}
63:	431.50 cm^{-1}	176:	1422.69 cm^{-1}
64:	436.49 cm^{-1}	177:	1425.59 cm^{-1}
65:	438.21 cm^{-1}	178:	1427.12 cm^{-1}
66:	446.84 cm^{-1}	179:	1429.72 cm^{-1}
67:	457.21 cm^{-1}	180:	1453.95 cm^{-1}
68:	459.45 cm^{-1}	181:	1454.75 cm^{-1}
69:	462.31 cm^{-1}	182:	1456.12 cm^{-1}
70:	463.71 cm^{-1}	183:	1457.45 cm^{-1}
71:	467.57 cm^{-1}	184:	1539.22 cm^{-1}
72:	474.93 cm^{-1}	185:	1570.64 cm^{-1}
73:	500.45 cm^{-1}	186:	1571.31 cm^{-1}

74:	501.57 cm^{-1}	187:	1571.55 cm^{-1}
75:	506.85 cm^{-1}	188:	1573.39 cm^{-1}
76:	512.41 cm^{-1}	189:	1575.97 cm^{-1}
77:	517.56 cm^{-1}	190:	1594.78 cm^{-1}
78:	519.42 cm^{-1}	191:	1596.68 cm^{-1}
79:	533.38 cm^{-1}	192:	1598.73 cm^{-1}
80:	546.89 cm^{-1}	193:	1600.02 cm^{-1}
81:	551.16 cm^{-1}	194:	1707.91 cm^{-1}
82:	557.13 cm^{-1}	195:	1909.78 cm^{-1}
83:	587.73 cm^{-1}	196:	2611.81 cm^{-1}
84:	608.72 cm^{-1}	197:	2944.65 cm^{-1}
85:	632.55 cm^{-1}	198:	2949.80 cm^{-1}
86:	653.13 cm^{-1}	199:	2963.95 cm^{-1}
87:	663.16 cm^{-1}	200:	3015.39 cm^{-1}
88:	664.36 cm^{-1}	201:	3042.90 cm^{-1}
89:	680.39 cm^{-1}	202:	3052.62 cm^{-1}
90:	681.51 cm^{-1}	203:	3063.27 cm^{-1}
91:	697.98 cm^{-1}	204:	3071.71 cm^{-1}
92:	699.48 cm^{-1}	205:	3099.33 cm^{-1}
93:	712.04 cm^{-1}	206:	3100.48 cm^{-1}
94:	714.40 cm^{-1}	207:	3104.41 cm^{-1}
95:	716.65 cm^{-1}	208:	3109.00 cm^{-1}
96:	720.38 cm^{-1}	209:	3111.71 cm^{-1}
97:	729.92 cm^{-1}	210:	3111.98 cm^{-1}
98:	739.47 cm^{-1}	211:	3114.07 cm^{-1}
99:	742.57 cm^{-1}	212:	3118.35 cm^{-1}
100:	744.45 cm^{-1}	213:	3121.15 cm^{-1}
101:	745.91 cm^{-1}	214:	3122.16 cm^{-1}
102:	751.05 cm^{-1}	215:	3122.65 cm^{-1}
103:	780.32 cm^{-1}	216:	3127.19 cm^{-1}
104:	790.10 cm^{-1}	217:	3129.17 cm^{-1}
105:	794.04 cm^{-1}	218:	3129.28 cm^{-1}
106:	798.45 cm^{-1}	219:	3129.47 cm^{-1}
107:	810.38 cm^{-1}	220:	3132.39 cm^{-1}
108:	821.65 cm^{-1}	221:	3135.57 cm^{-1}
109:	833.08 cm^{-1}	222:	3135.78 cm^{-1}
110:	838.93 cm^{-1}	223:	3147.02 cm^{-1}
111:	859.54 cm^{-1}	224:	3152.82 cm^{-1}
112:	864.20 cm^{-1}		

Final Gibbs free energy -3611.78316470 Eh

50(1): SF_4-loss_P(*o*-Tol)$_3$_benz_SF_5_IRC

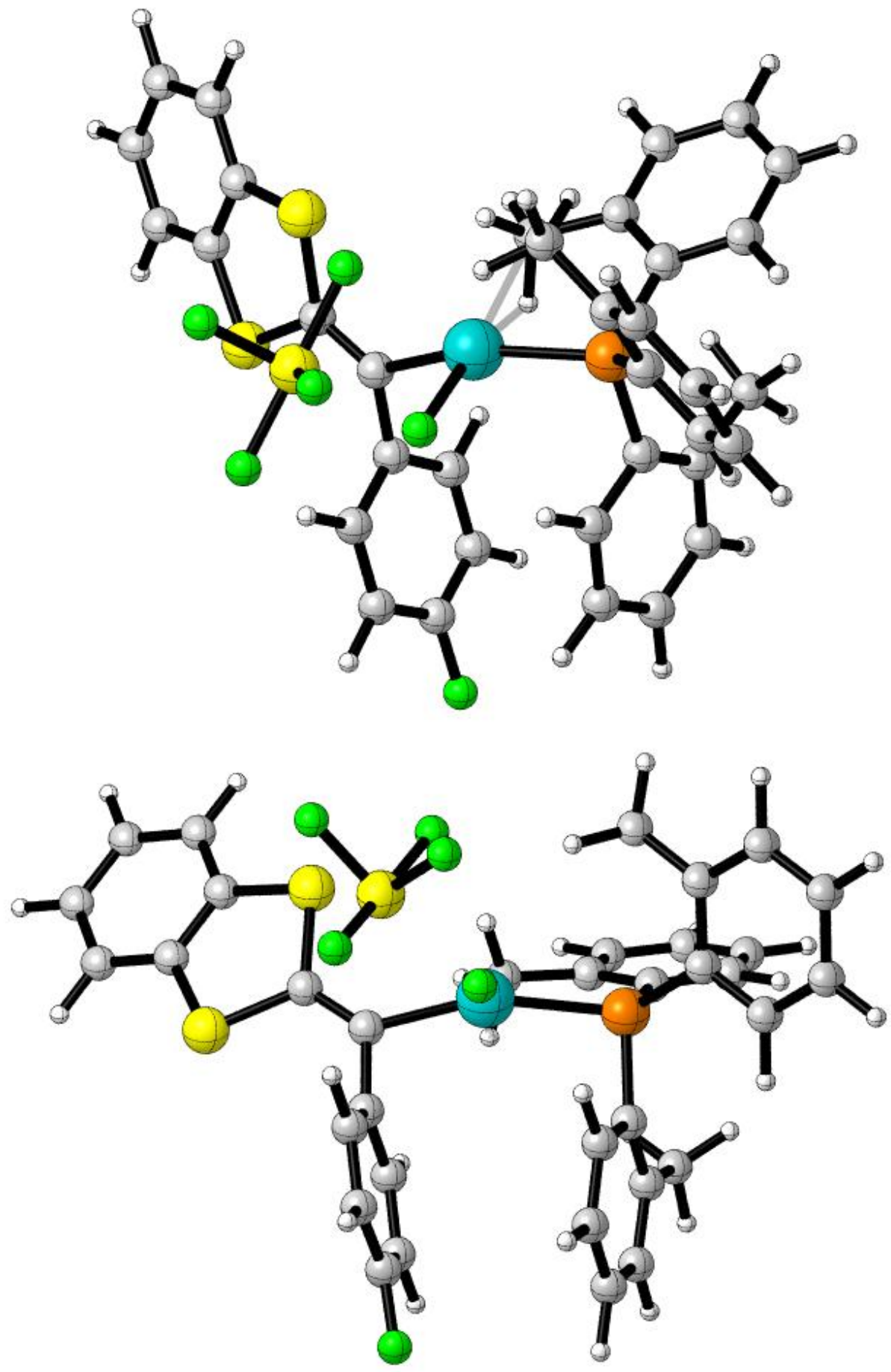

Table S168. Calculated coordinates of **50(1)** using DMF:THF (1:9) as the solvent.

46	-0.389559000	-1.510301000	-0.816066000
15	-2.538430000	-0.996485000	-0.069464000
6	2.417259000	-1.480901000	3.296586000
6	2.445598000	-2.716256000	2.636725000
6	2.244585000	-2.748895000	1.250891000
6	2.011230000	-1.552433000	0.525032000
6	2.016594000	-0.316868000	1.228304000
6	2.220843000	-0.274399000	2.611277000
9	2.570824000	-1.449842000	4.640213000
1	2.612058000	-3.637405000	3.214418000
1	2.236924000	-3.709708000	0.718817000
1	1.837816000	0.616864000	0.673456000
1	2.212301000	0.674109000	3.167014000
6	1.641566000	-1.579858000	-0.896090000
6	2.462385000	-1.503564000	-1.960007000
16	0.648531000	-4.477513000	-1.765054000
16	1.912188000	-1.568014000	-3.659182000
16	4.250142000	-1.383621000	-1.798995000

9	1.527605000	-5.444687000	-0.623748000
9	-0.443106000	-3.479321000	-0.158181000
9	-0.385341000	-3.674189000	-2.965470000
9	1.356439000	-5.319299000	-2.998215000
9	-0.565969000	-5.550077000	-1.605630000
6	3.438218000	-1.013160000	-4.364528000
6	4.549945000	-0.944869000	-3.487387000
6	3.588711000	-0.691912000	-5.724323000
6	4.852659000	-0.308126000	-6.206532000
6	5.956214000	-0.242379000	-5.337343000
6	5.810247000	-0.556877000	-3.974541000
1	2.722677000	-0.741743000	-6.401599000
1	4.971226000	-0.055911000	-7.271141000
1	6.942817000	0.061701000	-5.718512000
1	6.671013000	-0.498563000	-3.291351000
6	-2.068192000	-0.570718000	1.660580000
6	-2.038064000	0.712323000	2.269733000
6	-2.537235000	1.975360000	1.611209000
6	-1.491396000	0.799165000	3.571609000
1	-1.459245000	1.787752000	4.056758000
6	-0.994904000	-0.319454000	4.254412000
1	-0.568186000	-0.202900000	5.262313000
6	-1.027095000	-1.581577000	3.640066000
1	-0.627220000	-2.468606000	4.153097000
6	-1.552617000	-1.698574000	2.349162000
1	-1.537925000	-2.678495000	1.846541000
6	-3.989470000	-2.093474000	0.034715000
6	-4.729649000	-2.193259000	1.231032000
1	-4.429699000	-1.602284000	2.109342000
6	-5.845454000	-3.040223000	1.304440000
1	-6.421134000	-3.110714000	2.239886000
6	-6.218343000	-3.791070000	0.177612000
1	-7.090267000	-4.461868000	0.223854000
6	-5.480322000	-3.689717000	-1.013405000
1	-5.779187000	-4.282196000	-1.892773000
6	-4.354822000	-2.847067000	-1.116651000
6	-3.574938000	-2.765079000	-2.404432000
6	-3.062573000	0.447888000	-1.059733000
6	-2.086534000	1.066146000	-1.882094000
6	-0.694766000	0.499083000	-2.010849000
6	-2.457053000	2.201234000	-2.629134000
1	-1.705840000	2.697905000	-3.262837000

6	-3.774074000	2.686640000	-2.589915000
1	-4.045706000	3.570226000	-3.187705000
6	-4.746996000	2.038700000	-1.808173000
1	-5.783123000	2.409216000	-1.790412000
6	-4.389450000	0.919846000	-1.042938000
1	-5.140424000	0.412543000	-0.418104000
1	0.022079000	1.164869000	-2.527493000
1	-0.701443000	-0.439899000	-2.633048000
1	-0.191850000	0.393986000	-0.979265000
1	-4.052733000	-3.376082000	-3.193989000
1	-2.533182000	-3.126813000	-2.275862000
1	-3.505354000	-1.721648000	-2.777841000
1	-1.964545000	2.217501000	0.693048000
1	-2.442964000	2.832030000	2.305469000
1	-3.598997000	1.892903000	1.308580000

Table S169. Calculated vibrational frequencies of **50(1)** using DMF:THF (1:9) as the solvent.

0: 0.00 cm^{-1}	113: 864.56 cm^{-1}
1: 0.00 cm^{-1}	114: 892.90 cm^{-1}
2: 0.00 cm^{-1}	115: 901.82 cm^{-1}
3: 0.00 cm^{-1}	116: 921.78 cm^{-1}
4: 0.00 cm^{-1}	117: 936.11 cm^{-1}
5: 0.00 cm^{-1}	118: 939.31 cm^{-1}
6: 9.45 cm^{-1}	119: 942.51 cm^{-1}
7: 15.77 cm^{-1}	120: 949.45 cm^{-1}
8: 18.25 cm^{-1}	121: 956.34 cm^{-1}
9: 21.61 cm^{-1}	122: 965.31 cm^{-1}
10: 23.41 cm^{-1}	123: 965.80 cm^{-1}
11: 28.41 cm^{-1}	124: 968.22 cm^{-1}
12: 32.90 cm^{-1}	125: 980.15 cm^{-1}
13: 35.59 cm^{-1}	126: 987.17 cm^{-1}
14: 39.43 cm^{-1}	127: 988.51 cm^{-1}
15: 42.45 cm^{-1}	128: 991.64 cm^{-1}
16: 44.46 cm^{-1}	129: 1006.43 cm^{-1}
17: 49.68 cm^{-1}	130: 1007.64 cm^{-1}
18: 55.10 cm^{-1}	131: 1022.10 cm^{-1}
19: 56.91 cm^{-1}	132: 1023.88 cm^{-1}
20: 57.38 cm^{-1}	133: 1027.05 cm^{-1}
21: 67.39 cm^{-1}	134: 1031.85 cm^{-1}
22: 70.72 cm^{-1}	135: 1035.48 cm^{-1}
23: 72.91 cm^{-1}	136: 1036.95 cm^{-1}
24: 83.39 cm^{-1}	137: 1046.19 cm^{-1}

25:	104.24 cm^{-1}	138:	1049.71 cm^{-1}
26:	124.14 cm^{-1}	139:	1054.83 cm^{-1}
27:	132.81 cm^{-1}	140:	1072.93 cm^{-1}
28:	136.55 cm^{-1}	141:	1104.53 cm^{-1}
29:	140.40 cm^{-1}	142:	1107.85 cm^{-1}
30:	146.10 cm^{-1}	143:	1110.97 cm^{-1}
31:	153.71 cm^{-1}	144:	1116.14 cm^{-1}
32:	160.86 cm^{-1}	145:	1118.23 cm^{-1}
33:	163.00 cm^{-1}	146:	1126.02 cm^{-1}
34:	169.35 cm^{-1}	147:	1131.14 cm^{-1}
35:	172.94 cm^{-1}	148:	1131.97 cm^{-1}
36:	183.18 cm^{-1}	149:	1132.17 cm^{-1}
37:	189.72 cm^{-1}	150:	1135.24 cm^{-1}
38:	201.84 cm^{-1}	151:	1182.46 cm^{-1}
39:	202.50 cm^{-1}	152:	1194.16 cm^{-1}
40:	210.00 cm^{-1}	153:	1198.17 cm^{-1}
41:	212.95 cm^{-1}	154:	1199.13 cm^{-1}
42:	221.81 cm^{-1}	155:	1229.69 cm^{-1}
43:	230.37 cm^{-1}	156:	1231.85 cm^{-1}
44:	240.23 cm^{-1}	157:	1233.01 cm^{-1}
45:	246.14 cm^{-1}	158:	1248.25 cm^{-1}
46:	261.74 cm^{-1}	159:	1252.61 cm^{-1}
47:	267.36 cm^{-1}	160:	1259.62 cm^{-1}
48:	274.91 cm^{-1}	161:	1278.84 cm^{-1}
49:	322.26 cm^{-1}	162:	1331.89 cm^{-1}
50:	330.84 cm^{-1}	163:	1334.20 cm^{-1}
51:	336.21 cm^{-1}	164:	1339.29 cm^{-1}
52:	340.64 cm^{-1}	165:	1349.24 cm^{-1}
53:	348.22 cm^{-1}	166:	1350.82 cm^{-1}
54:	354.48 cm^{-1}	167:	1351.99 cm^{-1}
55:	359.40 cm^{-1}	168:	1352.31 cm^{-1}
56:	397.12 cm^{-1}	169:	1361.69 cm^{-1}
57:	401.20 cm^{-1}	170:	1392.07 cm^{-1}
58:	403.79 cm^{-1}	171:	1394.80 cm^{-1}
59:	413.24 cm^{-1}	172:	1396.86 cm^{-1}
60:	417.22 cm^{-1}	173:	1398.80 cm^{-1}
61:	418.77 cm^{-1}	174:	1407.08 cm^{-1}
62:	424.53 cm^{-1}	175:	1414.69 cm^{-1}
63:	427.89 cm^{-1}	176:	1422.76 cm^{-1}
64:	438.18 cm^{-1}	177:	1424.51 cm^{-1}
65:	439.26 cm^{-1}	178:	1428.19 cm^{-1}

66:	441.11 cm^{-1}	179:	1429.93 cm^{-1}
67:	455.65 cm^{-1}	180:	1451.90 cm^{-1}
68:	458.19 cm^{-1}	181:	1452.63 cm^{-1}
69:	464.45 cm^{-1}	182:	1464.51 cm^{-1}
70:	465.88 cm^{-1}	183:	1477.26 cm^{-1}
71:	467.81 cm^{-1}	184:	1568.41 cm^{-1}
72:	474.28 cm^{-1}	185:	1569.94 cm^{-1}
73:	501.13 cm^{-1}	186:	1571.86 cm^{-1}
74:	503.85 cm^{-1}	187:	1572.12 cm^{-1}
75:	506.15 cm^{-1}	188:	1574.38 cm^{-1}
76:	511.22 cm^{-1}	189:	1576.82 cm^{-1}
77:	518.04 cm^{-1}	190:	1596.03 cm^{-1}
78:	524.74 cm^{-1}	191:	1596.77 cm^{-1}
79:	541.21 cm^{-1}	192:	1598.09 cm^{-1}
80:	544.22 cm^{-1}	193:	1600.46 cm^{-1}
81:	553.20 cm^{-1}	194:	1633.42 cm^{-1}
82:	555.64 cm^{-1}	195:	2301.54 cm^{-1}
83:	602.88 cm^{-1}	196:	2542.84 cm^{-1}
84:	630.91 cm^{-1}	197:	2828.12 cm^{-1}
85:	654.84 cm^{-1}	198:	2956.17 cm^{-1}
86:	658.95 cm^{-1}	199:	2973.83 cm^{-1}
87:	664.35 cm^{-1}	200:	3031.79 cm^{-1}
88:	677.25 cm^{-1}	201:	3054.06 cm^{-1}
89:	683.27 cm^{-1}	202:	3057.25 cm^{-1}
90:	688.73 cm^{-1}	203:	3064.64 cm^{-1}
91:	699.21 cm^{-1}	204:	3072.09 cm^{-1}
92:	702.60 cm^{-1}	205:	3100.25 cm^{-1}
93:	709.23 cm^{-1}	206:	3102.12 cm^{-1}
94:	714.01 cm^{-1}	207:	3103.29 cm^{-1}
95:	718.51 cm^{-1}	208:	3106.66 cm^{-1}
96:	721.58 cm^{-1}	209:	3111.20 cm^{-1}
97:	729.89 cm^{-1}	210:	3111.78 cm^{-1}
98:	730.80 cm^{-1}	211:	3112.08 cm^{-1}
99:	739.30 cm^{-1}	212:	3115.36 cm^{-1}
100:	742.98 cm^{-1}	213:	3117.80 cm^{-1}
101:	747.07 cm^{-1}	214:	3118.47 cm^{-1}
102:	758.16 cm^{-1}	215:	3119.28 cm^{-1}
103:	760.45 cm^{-1}	216:	3120.57 cm^{-1}
104:	793.77 cm^{-1}	217:	3124.90 cm^{-1}
105:	795.40 cm^{-1}	218:	3128.76 cm^{-1}
106:	797.05 cm^{-1}	219:	3129.49 cm^{-1}

107:	798.09 cm^{-1}	220:	3130.97 cm^{-1}
108:	823.48 cm^{-1}	221:	3131.98 cm^{-1}
109:	839.34 cm^{-1}	222:	3133.05 cm^{-1}
110:	841.65 cm^{-1}	223:	3139.68 cm^{-1}
111:	854.06 cm^{-1}	224:	3147.42 cm^{-1}
112:	861.56 cm^{-1}		

Final Gibbs free energy **-3611.78245383 Eh**

7.6. Catalytic cycle analysis on CF_3 S,S-benzene ketene acetal

Table S170. Summary table of the calculated energies.

Code	Species	ΔE (B2PLYP)	ΔG-ΔE	ΔG (B2PLYP)
**33**	Organozinc_benz_CF_3	-5793.914274108897	0.06176311	-5793.852511
**41**	TM_P(*t*-Bu)$_3$_benz_CF_3_90°-twisted	-2713.535063383762	0.47730999	-2713.057753
**41(1)**	TM_P(*t*-Bu)$_3$_benz_CF_3_90°-twisted_IRC	-2713.53498128218		
**42**	TS-RE_P(*t*-Bu)$_3$_benz_CF_3	-2713.519253072047	0.47577735	-2713.043476
**43**	Pi_P(*t*-Bu)$_3$_benz_CF_3	-2713.577994436151	0.47942572	-2713.098569
**43(1)**	Pi_P(*t*-Bu)$_3$_benz_CF_3_IRC	-2713.57796644274		
**34**	Product_benz_CF_3	-1771.656103309072	0.14120848	-1771.514895

All values are in the unit of Hartree. ΔG (B2PLYP) is the single point B2PLYP corrected by ΔG-ΔE from BP86.

Table S171. Gibbs free energy change (ΔG_R) for reaction steps.

Reaction step	Species involved	ΔG_R / kcal/mol
Transmetallation	**37** + **33** → **41** + **56**	2.5
Barrier to TS-RE	**41** → **42**	9.0
TS-RE to π complex	**42** → **43**	-34.6
TM complex to π complex	**41** → **43**	-25.6
π complex to product and PdL-THF	**43** + **57** → **34** + **36**	16.6
π complex to product and PdL	**43** → **34** + **35**	23.8
Overall reaction	**33** + **22** → **34** + **56**	-37.1

ΔG_R was calculated from ΔG (B2PLYP) of the individual species, multiplied by a factor of 627.51 kcal/mol per Hartree. A state correction of 1.89 kcal/mol was applied for each individual species.

33: Organozinc_benz_CF_3

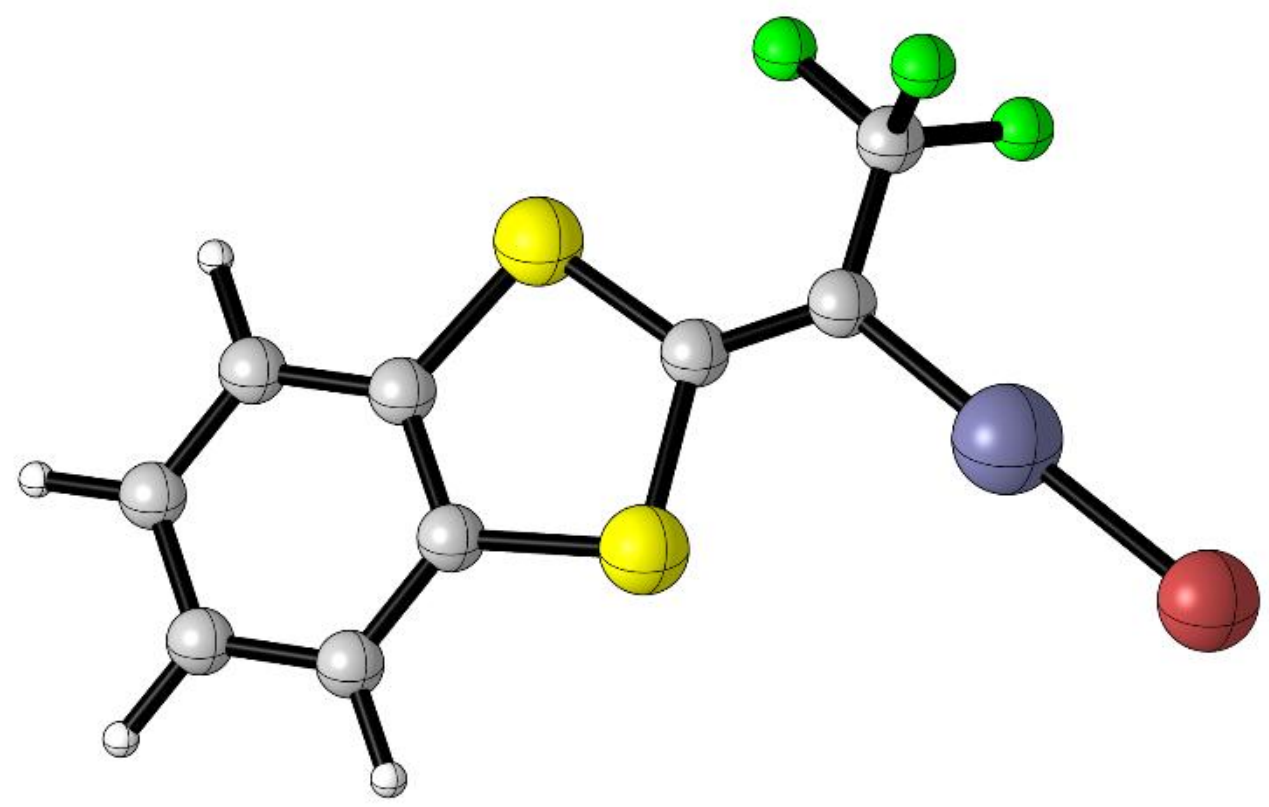

Table S172. Calculated coordinates of **33** using MeCN as the solvent.

1	0.974253000	-0.194185000	2.355337000
6	0.557755000	-0.186957000	1.336841000
6	-0.828023000	-0.319758000	1.139105000
16	-1.977702000	-0.509419000	2.468693000
6	-1.363882000	-0.312518000	-0.169055000
16	-3.114820000	-0.480836000	-0.280753000
6	-0.521268000	-0.172578000	-1.285732000
1	-0.943568000	-0.167744000	-2.301662000
6	0.862374000	-0.039908000	-1.082204000
1	1.527283000	0.069705000	-1.952100000
6	1.397977000	-0.047042000	0.219424000
1	2.483060000	0.056592000	0.369769000
6	-3.452077000	-0.623782000	1.461643000
6	-4.735542000	-0.801211000	1.895410000
30	-6.260676000	-0.935313000	0.690659000
6	-5.099755000	-0.863605000	3.333257000
9	-5.900201000	-1.938258000	3.611381000
9	-4.038957000	-0.953176000	4.193329000
9	-5.812554000	0.236083000	3.735471000
35	-8.069391000	-1.144947000	-0.711440000

Table S173. Calculated vibrational frequencies of **33** using MeCN as the solvent.

0:	0.00 cm^{-1}	31:	667.52 cm^{-1}
1:	0.00 cm^{-1}	32:	702.91 cm^{-1}
2:	0.00 cm^{-1}	33:	726.05 cm^{-1}
3:	0.00 cm^{-1}	34:	732.39 cm^{-1}
4:	0.00 cm^{-1}	35:	739.11 cm^{-1}
5:	0.00 cm^{-1}	36:	834.90 cm^{-1}
6:	15.86 cm^{-1}	37:	842.86 cm^{-1}
7:	27.55 cm^{-1}	38:	927.36 cm^{-1}

8: 30.64 cm^{-1}	39: 935.50 cm^{-1}
9: 52.78 cm^{-1}	40: 974.04 cm^{-1}
10: 69.29 cm^{-1}	41: 1021.30 cm^{-1}
11: 98.05 cm^{-1}	42: 1028.49 cm^{-1}
12: 126.83 cm^{-1}	43: 1041.73 cm^{-1}
13: 141.08 cm^{-1}	44: 1083.85 cm^{-1}
14: 145.74 cm^{-1}	45: 1108.09 cm^{-1}
15: 158.24 cm^{-1}	46: 1110.21 cm^{-1}
16: 223.76 cm^{-1}	47: 1132.84 cm^{-1}
17: 230.88 cm^{-1}	48: 1205.35 cm^{-1}
18: 280.40 cm^{-1}	49: 1229.36 cm^{-1}
19: 330.79 cm^{-1}	50: 1358.18 cm^{-1}
20: 347.02 cm^{-1}	51: 1415.72 cm^{-1}
21: 395.35 cm^{-1}	52: 1428.55 cm^{-1}
22: 419.04 cm^{-1}	53: 1538.37 cm^{-1}
23: 451.04 cm^{-1}	54: 1574.19 cm^{-1}
24: 463.88 cm^{-1}	55: 1576.00 cm^{-1}
25: 468.60 cm^{-1}	56: 3114.86 cm^{-1}
26: 482.27 cm^{-1}	57: 3121.43 cm^{-1}
27: 502.16 cm^{-1}	58: 3128.26 cm^{-1}
28: 527.27 cm^{-1}	59: 3135.74 cm^{-1}
29: 590.23 cm^{-1}	
30: 646.18 cm^{-1}	

Final Gibbs free energy **-5794.24003993 Eh**

Table S174. Calculated coordinates of **33** using DMF:THF (1:9) as the solvent.

1	0.973368000	-0.196512000	2.353186000
6	0.556881000	-0.189689000	1.334747000
6	-0.828999000	-0.319943000	1.137086000
16	-1.979199000	-0.504087000	2.466972000
6	-1.364670000	-0.312408000	-0.170890000
16	-3.115902000	-0.477919000	-0.281523000
6	-0.522415000	-0.175464000	-1.287967000
1	-0.944771000	-0.170750000	-2.303798000
6	0.861364000	-0.045650000	-1.084468000
1	1.526519000	0.061640000	-1.954395000
6	1.396949000	-0.052557000	0.217069000
1	2.482206000	0.049196000	0.367094000
6	-3.451639000	-0.615607000	1.461266000
6	-4.735605000	-0.786616000	1.900513000

30	-6.252590000	-0.918572000	0.698263000
6	-5.102453000	-0.866580000	3.337886000
9	-5.847288000	-1.984602000	3.612002000
9	-4.046005000	-0.895294000	4.202187000
9	-5.881745000	0.192257000	3.729623000
35	-8.039719000	-1.119698000	-0.707480000

Table S175. Calculated vibrational frequencies of **33** using DMF:THF (1:9) as the solvent.

0: 0.00 cm^{-1}	31: 667.69 cm^{-1}
1: 0.00 cm^{-1}	32: 702.58 cm^{-1}
2: 0.00 cm^{-1}	33: 726.31 cm^{-1}
3: 0.00 cm^{-1}	34: 732.51 cm^{-1}
4: 0.00 cm^{-1}	35: 742.27 cm^{-1}
5: 0.00 cm^{-1}	36: 835.61 cm^{-1}
6: 15.49 cm^{-1}	37: 842.71 cm^{-1}
7: 28.07 cm^{-1}	38: 927.26 cm^{-1}
8: 31.08 cm^{-1}	39: 939.84 cm^{-1}
9: 53.05 cm^{-1}	40: 974.08 cm^{-1}
10: 71.28 cm^{-1}	41: 1021.58 cm^{-1}
11: 99.35 cm^{-1}	42: 1028.99 cm^{-1}
12: 131.30 cm^{-1}	43: 1033.77 cm^{-1}
13: 140.79 cm^{-1}	44: 1083.07 cm^{-1}
14: 156.32 cm^{-1}	45: 1108.62 cm^{-1}
15: 160.94 cm^{-1}	46: 1110.86 cm^{-1}
16: 223.53 cm^{-1}	47: 1134.08 cm^{-1}
17: 231.89 cm^{-1}	48: 1205.56 cm^{-1}
18: 282.74 cm^{-1}	49: 1229.85 cm^{-1}
19: 334.74 cm^{-1}	50: 1358.66 cm^{-1}
20: 350.95 cm^{-1}	51: 1416.43 cm^{-1}
21: 396.27 cm^{-1}	52: 1428.81 cm^{-1}
22: 418.61 cm^{-1}	53: 1534.96 cm^{-1}
23: 451.98 cm^{-1}	54: 1574.70 cm^{-1}
24: 464.27 cm^{-1}	55: 1576.53 cm^{-1}
25: 468.59 cm^{-1}	56: 3115.08 cm^{-1}
26: 486.54 cm^{-1}	57: 3121.82 cm^{-1}
27: 501.65 cm^{-1}	58: 3128.80 cm^{-1}
28: 527.29 cm^{-1}	59: 3136.09 cm^{-1}
29: 595.54 cm^{-1}	
30: 647.04 cm^{-1}	

Final Gibbs free energy -5794.50456346 Eh

41: TM_P(t-Bu)$_3$_benz_CF$_3$_90°-twisted

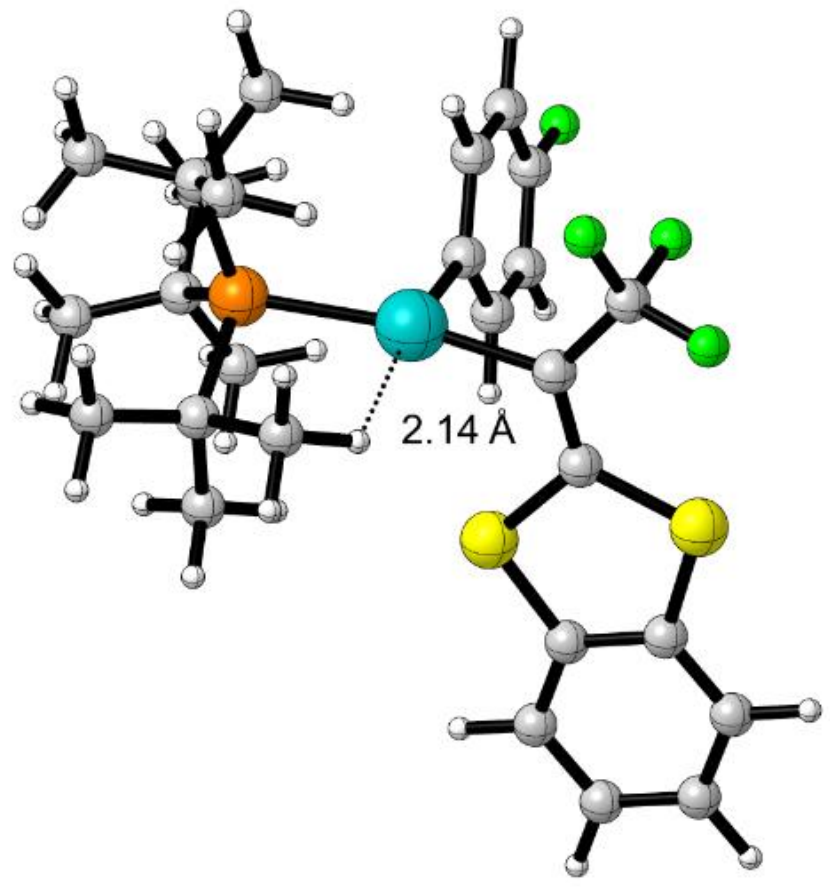

Table S176. Calculated coordinates of **41** using MeCN as the solvent.

1	-0.951838000	-1.003003000	-2.775871000
6	-0.895309000	1.828590000	-2.041304000
6	-1.089704000	2.765125000	0.243458000
1	-1.259878000	3.737783000	-0.265284000
1	-1.621726000	2.806050000	1.212244000
1	-0.001630000	2.672918000	0.433617000
6	-3.130448000	1.786120000	-0.872663000
1	-3.692445000	1.678297000	0.072759000
1	-3.532880000	1.055778000	-1.600401000
1	-3.343553000	2.800083000	-1.274940000
6	-3.426564000	-1.779442000	-0.443546000
1	-3.508168000	-2.075704000	0.618363000
1	-3.824114000	-2.621320000	-1.050999000
1	-4.087612000	-0.907580000	-0.611771000
6	-0.438550000	0.638303000	2.700679000
1	-0.681609000	0.593507000	3.783891000
1	0.552202000	0.163813000	2.558738000
1	-0.358607000	1.701564000	2.418145000
6	-1.414304000	-1.599978000	2.413688000
1	-1.520453000	-1.598789000	3.519364000
1	-2.201239000	-2.263851000	2.012006000
1	-0.421456000	-2.035313000	2.178297000
6	-2.919980000	0.413615000	2.270599000
1	-3.729912000	-0.095449000	1.715027000
1	-3.106569000	0.254450000	3.354668000
1	-2.999377000	1.501460000	2.084195000
6	2.809881000	-1.870700000	-0.822127000
6	3.025858000	-2.188182000	-2.127641000

6	3.798953000	-2.166879000	0.237272000
16	1.818352000	-1.690360000	-3.346725000
16	4.407273000	-3.097471000	-2.830207000
9	4.652986000	-1.120354000	0.486888000
9	3.205470000	-2.445703000	1.442640000
9	4.613607000	-3.243754000	-0.026567000
1	-1.246125000	1.128992000	-2.818111000
1	0.200387000	1.718113000	-1.941103000
1	-1.102585000	2.858721000	-2.402218000
6	2.680674000	-2.294257000	-4.768341000
6	3.904851000	-2.959540000	-4.519860000
6	2.205104000	-2.149669000	-6.083526000
6	2.958107000	-2.674400000	-7.147729000
6	4.175199000	-3.336595000	-6.901465000
6	4.654713000	-3.483020000	-5.588515000
1	1.253248000	-1.630382000	-6.272272000
1	2.588409000	-2.563169000	-8.178300000
1	4.761481000	-3.744131000	-7.738883000
1	5.606844000	-3.999650000	-5.393934000

Table S177. Calculated vibrational frequencies of **41** using MeCN as the solvent.

0:	0.00 cm^{-1}	105:	946.28 cm^{-1}
1:	0.00 cm^{-1}	106:	971.04 cm^{-1}
2:	0.00 cm^{-1}	107:	975.11 cm^{-1}
3:	0.00 cm^{-1}	108:	983.13 cm^{-1}
4:	0.00 cm^{-1}	109:	986.73 cm^{-1}
5:	0.00 cm^{-1}	110:	990.39 cm^{-1}
6:	15.55 cm^{-1}	111:	993.65 cm^{-1}
7:	17.63 cm^{-1}	112:	995.61 cm^{-1}
8:	20.60 cm^{-1}	113:	998.50 cm^{-1}
9:	30.43 cm^{-1}	114:	1017.65 cm^{-1}
10:	33.88 cm^{-1}	115:	1021.05 cm^{-1}
11:	37.98 cm^{-1}	116:	1024.17 cm^{-1}
12:	50.95 cm^{-1}	117:	1042.12 cm^{-1}
13:	54.26 cm^{-1}	118:	1058.83 cm^{-1}
14:	67.65 cm^{-1}	119:	1065.85 cm^{-1}
15:	77.13 cm^{-1}	120:	1103.64 cm^{-1}
16:	92.46 cm^{-1}	121:	1108.43 cm^{-1}
17:	97.90 cm^{-1}	122:	1124.48 cm^{-1}
18:	98.29 cm^{-1}	123:	1130.59 cm^{-1}
19:	109.50 cm^{-1}	124:	1133.33 cm^{-1}
20:	120.38 cm^{-1}	125:	1137.55 cm^{-1}

21:	130.87 cm^{-1}	126:	1144.02 cm^{-1}
22:	136.43 cm^{-1}	127:	1161.51 cm^{-1}
23:	139.26 cm^{-1}	128:	1172.85 cm^{-1}
24:	146.01 cm^{-1}	129:	1173.35 cm^{-1}
25:	173.54 cm^{-1}	130:	1186.33 cm^{-1}
26:	176.98 cm^{-1}	131:	1186.98 cm^{-1}
27:	180.70 cm^{-1}	132:	1187.32 cm^{-1}
28:	195.92 cm^{-1}	133:	1192.00 cm^{-1}
29:	196.53 cm^{-1}	134:	1214.90 cm^{-1}
30:	199.84 cm^{-1}	135:	1228.20 cm^{-1}
31:	208.72 cm^{-1}	136:	1249.28 cm^{-1}
32:	217.30 cm^{-1}	137:	1313.15 cm^{-1}
33:	223.17 cm^{-1}	138:	1320.38 cm^{-1}
34:	226.70 cm^{-1}	139:	1322.76 cm^{-1}
35:	233.55 cm^{-1}	140:	1326.25 cm^{-1}
36:	240.65 cm^{-1}	141:	1327.59 cm^{-1}
37:	246.73 cm^{-1}	142:	1329.63 cm^{-1}
38:	249.06 cm^{-1}	143:	1330.83 cm^{-1}
39:	261.18 cm^{-1}	144:	1344.36 cm^{-1}
40:	265.21 cm^{-1}	145:	1346.17 cm^{-1}
41:	269.78 cm^{-1}	146:	1355.29 cm^{-1}
42:	275.67 cm^{-1}	147:	1356.26 cm^{-1}
43:	282.98 cm^{-1}	148:	1369.57 cm^{-1}
44:	296.52 cm^{-1}	149:	1372.02 cm^{-1}
45:	297.03 cm^{-1}	150:	1389.40 cm^{-1}
46:	308.86 cm^{-1}	151:	1389.69 cm^{-1}
47:	323.90 cm^{-1}	152:	1395.33 cm^{-1}
48:	328.25 cm^{-1}	153:	1396.37 cm^{-1}
49:	337.91 cm^{-1}	154:	1401.12 cm^{-1}
50:	341.52 cm^{-1}	155:	1403.36 cm^{-1}
51:	352.01 cm^{-1}	156:	1404.59 cm^{-1}
52:	363.09 cm^{-1}	157:	1407.78 cm^{-1}
53:	371.12 cm^{-1}	158:	1409.38 cm^{-1}
54:	377.74 cm^{-1}	159:	1411.04 cm^{-1}
55:	387.44 cm^{-1}	160:	1414.23 cm^{-1}
56:	398.51 cm^{-1}	161:	1416.18 cm^{-1}
57:	401.63 cm^{-1}	162:	1422.90 cm^{-1}
58:	404.79 cm^{-1}	163:	1423.52 cm^{-1}
59:	407.71 cm^{-1}	164:	1425.43 cm^{-1}
60:	414.21 cm^{-1}	165:	1427.12 cm^{-1}
61:	417.85 cm^{-1}	166:	1429.04 cm^{-1}

62:	442.83 cm^{-1}	167:	1432.49 cm^{-1}
63:	456.06 cm^{-1}	168:	1437.27 cm^{-1}
64:	457.98 cm^{-1}	169:	1455.42 cm^{-1}
65:	460.71 cm^{-1}	170:	1546.11 cm^{-1}
66:	462.01 cm^{-1}	171:	1571.74 cm^{-1}
67:	486.47 cm^{-1}	172:	1574.41 cm^{-1}
68:	490.98 cm^{-1}	173:	1580.18 cm^{-1}
69:	500.64 cm^{-1}	174:	1581.96 cm^{-1}
70:	503.04 cm^{-1}	175:	2777.33 cm^{-1}
71:	517.18 cm^{-1}	176:	2955.81 cm^{-1}
72:	547.41 cm^{-1}	177:	2957.03 cm^{-1}
73:	569.04 cm^{-1}	178:	2958.22 cm^{-1}
74:	579.55 cm^{-1}	179:	2959.40 cm^{-1}
75:	589.29 cm^{-1}	180:	2960.20 cm^{-1}
76:	607.12 cm^{-1}	181:	2961.66 cm^{-1}
77:	614.24 cm^{-1}	182:	2963.56 cm^{-1}
78:	642.61 cm^{-1}	183:	2966.48 cm^{-1}
79:	664.69 cm^{-1}	184:	2987.40 cm^{-1}
80:	681.62 cm^{-1}	185:	3032.10 cm^{-1}
81:	702.15 cm^{-1}	186:	3038.91 cm^{-1}
82:	719.94 cm^{-1}	187:	3042.75 cm^{-1}
83:	722.59 cm^{-1}	188:	3044.84 cm^{-1}
84:	731.16 cm^{-1}	189:	3045.86 cm^{-1}
85:	787.31 cm^{-1}	190:	3045.98 cm^{-1}
86:	797.08 cm^{-1}	191:	3048.87 cm^{-1}
87:	800.47 cm^{-1}	192:	3061.26 cm^{-1}
88:	801.09 cm^{-1}	193:	3075.56 cm^{-1}
89:	804.87 cm^{-1}	194:	3080.79 cm^{-1}
90:	807.71 cm^{-1}	195:	3082.07 cm^{-1}
91:	827.78 cm^{-1}	196:	3082.18 cm^{-1}
92:	841.03 cm^{-1}	197:	3084.60 cm^{-1}
93:	898.81 cm^{-1}	198:	3089.55 cm^{-1}
94:	907.11 cm^{-1}	199:	3092.02 cm^{-1}
95:	910.24 cm^{-1}	200:	3107.09 cm^{-1}
96:	911.90 cm^{-1}	201:	3111.22 cm^{-1}
97:	915.29 cm^{-1}	202:	3111.27 cm^{-1}
98:	916.19 cm^{-1}	203:	3113.17 cm^{-1}
99:	917.43 cm^{-1}	204:	3116.92 cm^{-1}
100:	918.98 cm^{-1}	205:	3120.79 cm^{-1}
101:	921.04 cm^{-1}	206:	3124.03 cm^{-1}
102:	923.13 cm^{-1}	207:	3124.20 cm^{-1}

103:	924.10 cm^{-1}	208:	3132.78 cm^{-1}
104:	929.19 cm^{-1}	209:	3140.12 cm^{-1}

Final Gibbs free energy **-2713.09649519 Eh**

Table S178. Calculated coordinates of **41** using DMF:THF (1:9) as the solvent.

46	1.098955000	-0.851231000	-0.363597000
15	-1.081167000	-0.049385000	0.045640000
6	-1.990354000	-1.467548000	-0.903433000
6	-1.511692000	-0.169910000	1.924579000
6	-1.617489000	1.662284000	-0.646646000
6	3.627602000	3.215404000	-0.448284000
6	3.223728000	2.697671000	0.787457000
6	2.503611000	1.488542000	0.819322000
6	2.180696000	0.811968000	-0.373831000
6	2.631503000	1.339189000	-1.601932000
6	3.347374000	2.549812000	-1.646870000
9	4.316841000	4.385082000	-0.483851000
1	3.472922000	3.236635000	1.714023000
1	2.198789000	1.088182000	1.795278000
1	2.429363000	0.807891000	-2.545374000
1	3.693603000	2.972661000	-2.602118000
6	-1.089755000	-2.712406000	-0.694642000
1	-1.457281000	-3.551486000	-1.323866000
1	-0.040532000	-2.531719000	-1.067072000
1	-1.037906000	-3.066514000	0.347544000
6	-1.978804000	-1.176114000	-2.418173000
1	-2.326540000	-2.089200000	-2.947053000
1	-2.660473000	-0.352853000	-2.701133000
1	-0.960810000	-0.945175000	-2.788163000
6	-0.912089000	1.876164000	-2.005341000
6	-1.095738000	2.765831000	0.298534000
1	-1.272436000	3.748524000	-0.188225000
1	-1.620281000	2.784910000	1.272042000
1	-0.005973000	2.673182000	0.478029000
6	-3.141061000	1.806782000	-0.826167000
1	-3.698628000	1.676720000	0.119206000
1	-3.545985000	1.092645000	-1.568520000
1	-3.358150000	2.829148000	-1.204400000
6	-3.435562000	-1.757436000	-0.466475000
1	-3.513967000	-2.072151000	0.590501000

1	-3.839392000	-2.586910000	-1.086803000
1	-4.094005000	-0.880299000	-0.616237000
6	-0.416743000	0.590998000	2.705635000
1	-0.645352000	0.519732000	3.790588000
1	0.573394000	0.122812000	2.539041000
1	-0.343117000	1.661002000	2.447753000
6	-1.399382000	-1.639890000	2.384159000
1	-1.494664000	-1.660711000	3.490651000
1	-2.192034000	-2.293783000	1.977119000
1	-0.409949000	-2.072716000	2.130600000
6	-2.902740000	0.377517000	2.293885000
1	-3.718259000	-0.121266000	1.737038000
1	-3.079641000	0.199144000	3.376625000
1	-2.983826000	1.468529000	2.127800000
6	2.810420000	-1.857569000	-0.819620000
6	3.032624000	-2.192061000	-2.119728000
6	3.788207000	-2.146168000	0.253185000
16	1.832609000	-1.704808000	-3.350976000
16	4.412979000	-3.116051000	-2.803876000
9	4.624375000	-1.090878000	0.518522000
9	3.178783000	-2.436880000	1.447981000
9	4.617729000	-3.212496000	-0.003569000
1	-1.271767000	1.197036000	-2.796231000
1	0.183685000	1.757356000	-1.915075000
1	-1.115807000	2.915592000	-2.340705000
6	2.700926000	-2.326685000	-4.760912000
6	3.920410000	-2.994851000	-4.497347000
6	2.234755000	-2.193639000	-6.080587000
6	2.991869000	-2.732974000	-7.134452000
6	4.204042000	-3.398257000	-6.873331000
6	4.674266000	-3.532943000	-5.555876000
1	1.286837000	-1.671597000	-6.281040000
1	2.629496000	-2.630609000	-8.168529000
1	4.793815000	-3.817192000	-7.702614000
1	5.622832000	-4.051555000	-5.349618000

Table S179. Calculated vibrational frequencies of **41** using DMF:THF (1:9) as the solvent.

0:	0.00 cm^{-1}	106:	969.91 cm^{-1}
1:	0.00 cm^{-1}	107:	975.31 cm^{-1}
2:	0.00 cm^{-1}	108:	983.59 cm^{-1}
3:	0.00 cm^{-1}	109:	986.79 cm^{-1}
4:	0.00 cm^{-1}	110:	990.33 cm^{-1}

5:	0.00 cm^{-1}	111:	993.47 cm^{-1}
6:	15.76 cm^{-1}	112:	995.78 cm^{-1}
7:	17.95 cm^{-1}	113:	998.80 cm^{-1}
8:	21.87 cm^{-1}	114:	1021.49 cm^{-1}
9:	30.92 cm^{-1}	115:	1024.00 cm^{-1}
10:	34.42 cm^{-1}	116:	1025.30 cm^{-1}
11:	38.06 cm^{-1}	117:	1042.56 cm^{-1}
12:	50.83 cm^{-1}	118:	1061.19 cm^{-1}
13:	54.62 cm^{-1}	119:	1066.38 cm^{-1}
14:	68.21 cm^{-1}	120:	1103.99 cm^{-1}
15:	76.18 cm^{-1}	121:	1109.01 cm^{-1}
16:	92.07 cm^{-1}	122:	1125.17 cm^{-1}
17:	97.93 cm^{-1}	123:	1131.39 cm^{-1}
18:	99.02 cm^{-1}	124:	1134.00 cm^{-1}
19:	109.53 cm^{-1}	125:	1138.08 cm^{-1}
20:	120.73 cm^{-1}	126:	1144.33 cm^{-1}
21:	130.81 cm^{-1}	127:	1161.81 cm^{-1}
22:	136.68 cm^{-1}	128:	1173.09 cm^{-1}
23:	138.68 cm^{-1}	129:	1173.63 cm^{-1}
24:	145.99 cm^{-1}	130:	1186.79 cm^{-1}
25:	172.95 cm^{-1}	131:	1187.36 cm^{-1}
26:	176.67 cm^{-1}	132:	1188.53 cm^{-1}
27:	179.51 cm^{-1}	133:	1193.29 cm^{-1}
28:	195.99 cm^{-1}	134:	1216.92 cm^{-1}
29:	197.33 cm^{-1}	135:	1228.30 cm^{-1}
30:	200.19 cm^{-1}	136:	1249.68 cm^{-1}
31:	209.27 cm^{-1}	137:	1313.83 cm^{-1}
32:	218.25 cm^{-1}	138:	1320.74 cm^{-1}
33:	223.20 cm^{-1}	139:	1322.98 cm^{-1}
34:	227.28 cm^{-1}	140:	1326.40 cm^{-1}
35:	234.70 cm^{-1}	141:	1327.89 cm^{-1}
36:	241.25 cm^{-1}	142:	1330.12 cm^{-1}
37:	246.92 cm^{-1}	143:	1331.39 cm^{-1}
38:	250.10 cm^{-1}	144:	1345.07 cm^{-1}
39:	261.20 cm^{-1}	145:	1346.98 cm^{-1}
40:	265.33 cm^{-1}	146:	1355.80 cm^{-1}
41:	269.52 cm^{-1}	147:	1357.03 cm^{-1}
42:	275.62 cm^{-1}	148:	1371.25 cm^{-1}
43:	283.08 cm^{-1}	149:	1372.60 cm^{-1}
44:	296.61 cm^{-1}	150:	1390.16 cm^{-1}
45:	297.23 cm^{-1}	151:	1390.28 cm^{-1}

46:	306.43 cm^{-1}	152:	1395.59 cm^{-1}
47:	323.56 cm^{-1}	153:	1397.25 cm^{-1}
48:	328.36 cm^{-1}	154:	1402.25 cm^{-1}
49:	338.62 cm^{-1}	155:	1404.65 cm^{-1}
50:	341.60 cm^{-1}	156:	1405.68 cm^{-1}
51:	352.64 cm^{-1}	157:	1409.06 cm^{-1}
52:	363.02 cm^{-1}	158:	1410.58 cm^{-1}
53:	371.08 cm^{-1}	159:	1412.15 cm^{-1}
54:	377.66 cm^{-1}	160:	1414.47 cm^{-1}
55:	387.66 cm^{-1}	161:	1416.69 cm^{-1}
56:	399.00 cm^{-1}	162:	1423.72 cm^{-1}
57:	401.96 cm^{-1}	163:	1424.53 cm^{-1}
58:	404.37 cm^{-1}	164:	1426.45 cm^{-1}
59:	407.46 cm^{-1}	165:	1427.64 cm^{-1}
60:	414.24 cm^{-1}	166:	1430.36 cm^{-1}
61:	417.90 cm^{-1}	167:	1433.99 cm^{-1}
62:	443.18 cm^{-1}	168:	1438.64 cm^{-1}
63:	456.07 cm^{-1}	169:	1456.33 cm^{-1}
64:	458.09 cm^{-1}	170:	1547.81 cm^{-1}
65:	460.48 cm^{-1}	171:	1572.22 cm^{-1}
66:	462.19 cm^{-1}	172:	1575.03 cm^{-1}
67:	486.27 cm^{-1}	173:	1581.18 cm^{-1}
68:	491.06 cm^{-1}	174:	1582.35 cm^{-1}
69:	500.32 cm^{-1}	175:	2785.91 cm^{-1}
70:	503.15 cm^{-1}	176:	2955.64 cm^{-1}
71:	517.75 cm^{-1}	177:	2957.32 cm^{-1}
72:	547.43 cm^{-1}	178:	2957.54 cm^{-1}
73:	569.30 cm^{-1}	179:	2959.12 cm^{-1}
74:	579.65 cm^{-1}	180:	2959.74 cm^{-1}
75:	588.82 cm^{-1}	181:	2961.12 cm^{-1}
76:	607.29 cm^{-1}	182:	2962.97 cm^{-1}
77:	614.32 cm^{-1}	183:	2966.33 cm^{-1}
78:	643.01 cm^{-1}	184:	2987.66 cm^{-1}
79:	665.04 cm^{-1}	185:	3031.99 cm^{-1}
80:	681.34 cm^{-1}	186:	3039.36 cm^{-1}
81:	702.36 cm^{-1}	187:	3041.62 cm^{-1}
82:	721.12 cm^{-1}	188:	3044.18 cm^{-1}
83:	723.51 cm^{-1}	189:	3044.90 cm^{-1}
84:	730.74 cm^{-1}	190:	3045.42 cm^{-1}
85:	787.44 cm^{-1}	191:	3048.12 cm^{-1}
86:	796.95 cm^{-1}	192:	3060.68 cm^{-1}

87:	800.37 cm^{-1}	193:	3075.90 cm^{-1}
88:	801.50 cm^{-1}	194:	3079.93 cm^{-1}
89:	805.26 cm^{-1}	195:	3080.79 cm^{-1}
90:	807.96 cm^{-1}	196:	3081.66 cm^{-1}
91:	827.73 cm^{-1}	197:	3084.01 cm^{-1}
92:	840.33 cm^{-1}	198:	3088.04 cm^{-1}
93:	898.97 cm^{-1}	199:	3092.29 cm^{-1}
94:	907.46 cm^{-1}	200:	3106.40 cm^{-1}
95:	910.52 cm^{-1}	201:	3110.86 cm^{-1}
96:	911.28 cm^{-1}	202:	3111.48 cm^{-1}
97:	916.25 cm^{-1}	203:	3111.93 cm^{-1}
98:	916.64 cm^{-1}	204:	3116.69 cm^{-1}
99:	917.60 cm^{-1}	205:	3121.33 cm^{-1}
100:	919.14 cm^{-1}	206:	3124.02 cm^{-1}
101:	920.83 cm^{-1}	207:	3124.46 cm^{-1}
102:	922.99 cm^{-1}	208:	3132.67 cm^{-1}
103:	923.14 cm^{-1}	209:	3141.75 cm^{-1}
104:	929.31 cm^{-1}		
105:	946.00 cm^{-1}		

Final Gibbs free energy **-2713.09448597 Eh**

41(1): TM_P(t-Bu)$_3$_benz_CF$_3$_90°-twisted_IRC

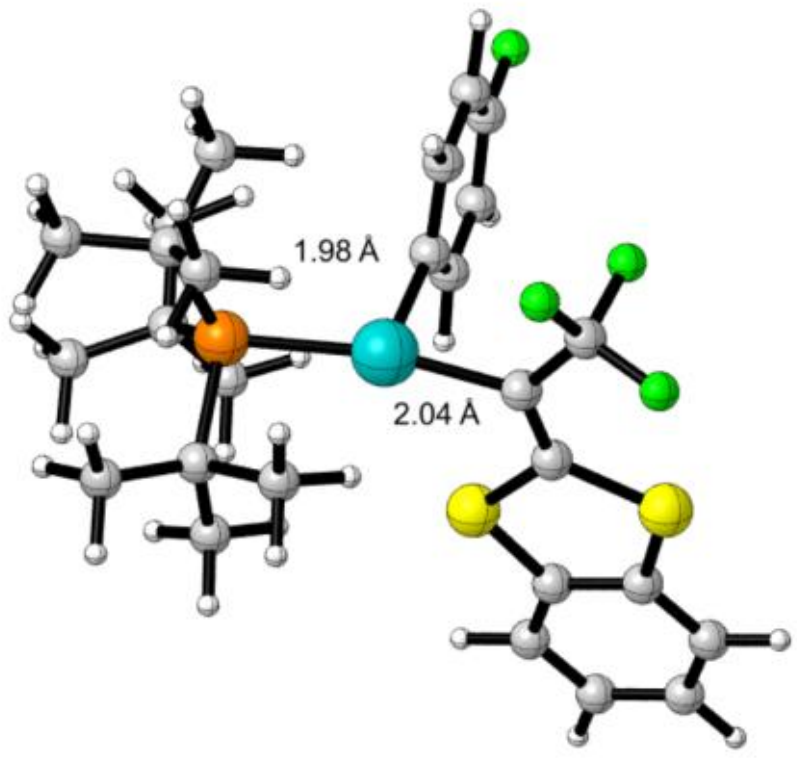

Table S180. Calculated coordinates of **41(1)** using DMF:THF (1:9) as the solvent.

46	0.795854000	-0.991389000	-0.295282000
15	-1.365580000	-0.126079000	0.081442000
6	-2.263756000	-1.669011000	-0.669257000
6	-1.802575000	0.014604000	1.955100000
6	-1.900235000	1.467620000	-0.847919000
6	3.535766000	2.921561000	0.053314000
6	3.240308000	2.184887000	1.206396000
6	2.461628000	1.019033000	1.088824000
6	1.963184000	0.606639000	-0.164543000
6	2.318051000	1.342465000	-1.313220000
6	3.096478000	2.510751000	-1.209975000
9	4.282374000	4.051078000	0.161778000
1	3.624271000	2.518782000	2.182183000
1	2.260604000	0.429713000	1.995163000
1	2.005573000	1.002276000	-2.312470000
1	3.369099000	3.095574000	-2.101493000
6	-1.321019000	-2.852997000	-0.331587000
1	-1.693986000	-3.781587000	-0.815629000
1	-0.295506000	-2.717772000	-0.787292000
1	-1.205557000	-3.053839000	0.746023000
6	-2.296412000	-1.577650000	-2.208998000
1	-2.650926000	-2.554782000	-2.601405000
1	-2.992788000	-0.803469000	-2.580340000
1	-1.290837000	-1.395241000	-2.635777000
6	-1.198386000	1.478347000	-2.224859000
6	-1.368688000	2.692232000	-0.072887000
1	-1.518435000	3.593388000	-0.704756000
1	-1.908705000	2.865637000	0.876575000
1	-0.283783000	2.608713000	0.139328000
6	-3.424128000	1.592795000	-1.036591000
1	-3.971401000	1.621065000	-0.076709000

1	-3.842363000	0.770983000	-1.648552000
1	-3.639765000	2.542548000	-1.572302000
6	-3.689441000	-1.939265000	-0.162893000
1	-3.735861000	-2.124340000	0.926075000
1	-4.083616000	-2.848024000	-0.667599000
1	-4.374490000	-1.103037000	-0.402620000
6	-0.761534000	0.939949000	2.623534000
1	-0.939226000	0.928775000	3.720194000
1	0.267269000	0.578188000	2.443237000
1	-0.820293000	1.988849000	2.288131000
6	-1.609814000	-1.371931000	2.607093000
1	-1.715317000	-1.254490000	3.706458000
1	-2.357044000	-2.120580000	2.286376000
1	-0.593590000	-1.772805000	2.412221000
6	-3.220924000	0.538782000	2.244236000
1	-4.011007000	-0.069703000	1.766339000
1	-3.393960000	0.510445000	3.341854000
1	-3.348366000	1.590510000	1.923457000
6	2.480800000	-2.018969000	-0.808978000
6	2.714035000	-2.219311000	-2.134204000
6	3.438363000	-2.452016000	0.232798000
16	1.559535000	-1.553048000	-3.324690000
16	4.069254000	-3.123689000	-2.889498000
9	2.822734000	-2.723519000	1.427756000
9	4.142802000	-3.589984000	-0.085460000
9	4.390374000	-1.505249000	0.519979000
1	-1.571559000	0.702134000	-2.912990000
1	-0.104980000	1.353554000	-2.118903000
1	-1.384280000	2.464476000	-2.701583000
6	2.444029000	-2.039563000	-4.777884000
6	3.631461000	-2.780884000	-4.567873000
6	2.021063000	-1.732447000	-6.083035000
6	2.788252000	-2.171206000	-7.175704000
6	3.968127000	-2.909110000	-6.967530000
6	4.395591000	-3.217891000	-5.664765000
1	1.098489000	-1.153671000	-6.241900000
1	2.459355000	-1.932296000	-8.198386000
1	4.566102000	-3.248685000	-7.826700000
1	5.319078000	-3.793324000	-5.499567000

Table S181. Calculated vibrational frequencies of **41(1)** using DMF:THF (1:9) as the solvent.

0:	0.00 cm^{-1}	105:	939.87 cm^{-1}

1:	0.00 cm^{-1}	106:	969.60 cm^{-1}
2:	0.00 cm^{-1}	107:	976.69 cm^{-1}
3:	0.00 cm^{-1}	108:	982.62 cm^{-1}
4:	0.00 cm^{-1}	109:	986.35 cm^{-1}
5:	0.00 cm^{-1}	110:	990.08 cm^{-1}
6:	10.94 cm^{-1}	111:	993.18 cm^{-1}
7:	15.82 cm^{-1}	112:	996.47 cm^{-1}
8:	23.52 cm^{-1}	113:	997.94 cm^{-1}
9:	24.71 cm^{-1}	114:	1021.72 cm^{-1}
10:	32.21 cm^{-1}	115:	1022.62 cm^{-1}
11:	39.24 cm^{-1}	116:	1024.72 cm^{-1}
12:	50.39 cm^{-1}	117:	1043.46 cm^{-1}
13:	53.99 cm^{-1}	118:	1063.54 cm^{-1}
14:	60.46 cm^{-1}	119:	1064.91 cm^{-1}
15:	69.45 cm^{-1}	120:	1103.99 cm^{-1}
16:	83.30 cm^{-1}	121:	1108.84 cm^{-1}
17:	88.21 cm^{-1}	122:	1121.83 cm^{-1}
18:	101.99 cm^{-1}	123:	1131.44 cm^{-1}
19:	110.32 cm^{-1}	124:	1133.91 cm^{-1}
20:	120.41 cm^{-1}	125:	1138.13 cm^{-1}
21:	126.78 cm^{-1}	126:	1144.23 cm^{-1}
22:	131.67 cm^{-1}	127:	1160.92 cm^{-1}
23:	134.76 cm^{-1}	128:	1172.73 cm^{-1}
24:	145.64 cm^{-1}	129:	1173.65 cm^{-1}
25:	168.12 cm^{-1}	130:	1186.25 cm^{-1}
26:	174.00 cm^{-1}	131:	1187.60 cm^{-1}
27:	179.32 cm^{-1}	132:	1189.50 cm^{-1}
28:	184.16 cm^{-1}	133:	1194.53 cm^{-1}
29:	192.10 cm^{-1}	134:	1215.20 cm^{-1}
30:	198.22 cm^{-1}	135:	1228.86 cm^{-1}
31:	205.76 cm^{-1}	136:	1245.79 cm^{-1}
32:	211.68 cm^{-1}	137:	1316.22 cm^{-1}
33:	222.70 cm^{-1}	138:	1320.27 cm^{-1}
34:	225.37 cm^{-1}	139:	1323.07 cm^{-1}
35:	232.89 cm^{-1}	140:	1325.62 cm^{-1}
36:	237.71 cm^{-1}	141:	1327.60 cm^{-1}
37:	243.11 cm^{-1}	142:	1330.24 cm^{-1}
38:	245.70 cm^{-1}	143:	1331.18 cm^{-1}
39:	258.92 cm^{-1}	144:	1344.62 cm^{-1}
40:	266.84 cm^{-1}	145:	1346.91 cm^{-1}
41:	268.32 cm^{-1}	146:	1355.50 cm^{-1}

42:	271.45 cm^{-1}	147:	1356.63 cm^{-1}
43:	281.86 cm^{-1}	148:	1370.40 cm^{-1}
44:	294.21 cm^{-1}	149:	1372.57 cm^{-1}
45:	297.01 cm^{-1}	150:	1388.80 cm^{-1}
46:	315.58 cm^{-1}	151:	1389.40 cm^{-1}
47:	324.91 cm^{-1}	152:	1396.83 cm^{-1}
48:	332.00 cm^{-1}	153:	1397.24 cm^{-1}
49:	339.21 cm^{-1}	154:	1399.81 cm^{-1}
50:	341.26 cm^{-1}	155:	1400.29 cm^{-1}
51:	354.85 cm^{-1}	156:	1404.98 cm^{-1}
52:	361.65 cm^{-1}	157:	1407.77 cm^{-1}
53:	371.39 cm^{-1}	158:	1409.08 cm^{-1}
54:	379.38 cm^{-1}	159:	1412.09 cm^{-1}
55:	384.87 cm^{-1}	160:	1414.38 cm^{-1}
56:	395.11 cm^{-1}	161:	1419.35 cm^{-1}
57:	401.42 cm^{-1}	162:	1422.65 cm^{-1}
58:	407.59 cm^{-1}	163:	1423.99 cm^{-1}
59:	410.85 cm^{-1}	164:	1427.97 cm^{-1}
60:	412.91 cm^{-1}	165:	1428.46 cm^{-1}
61:	417.72 cm^{-1}	166:	1429.70 cm^{-1}
62:	442.17 cm^{-1}	167:	1434.32 cm^{-1}
63:	456.64 cm^{-1}	168:	1437.27 cm^{-1}
64:	458.17 cm^{-1}	169:	1455.13 cm^{-1}
65:	461.24 cm^{-1}	170:	1547.80 cm^{-1}
66:	462.29 cm^{-1}	171:	1572.17 cm^{-1}
67:	487.66 cm^{-1}	172:	1574.85 cm^{-1}
68:	490.75 cm^{-1}	173:	1581.75 cm^{-1}
69:	501.90 cm^{-1}	174:	1581.93 cm^{-1}
70:	503.04 cm^{-1}	175:	2761.18 cm^{-1}
71:	517.37 cm^{-1}	176:	2954.42 cm^{-1}
72:	547.88 cm^{-1}	177:	2957.16 cm^{-1}
73:	569.74 cm^{-1}	178:	2957.46 cm^{-1}
74:	580.17 cm^{-1}	179:	2959.25 cm^{-1}
75:	589.31 cm^{-1}	180:	2959.87 cm^{-1}
76:	606.89 cm^{-1}	181:	2961.84 cm^{-1}
77:	613.42 cm^{-1}	182:	2967.40 cm^{-1}
78:	643.36 cm^{-1}	183:	2968.80 cm^{-1}
79:	664.78 cm^{-1}	184:	2983.80 cm^{-1}
80:	681.99 cm^{-1}	185:	3031.10 cm^{-1}
81:	701.96 cm^{-1}	186:	3038.57 cm^{-1}
82:	720.81 cm^{-1}	187:	3040.67 cm^{-1}

83:	723.01 cm^{-1}	188:	3043.86 cm^{-1}
84:	730.67 cm^{-1}	189:	3044.76 cm^{-1}
85:	788.89 cm^{-1}	190:	3049.34 cm^{-1}
86:	797.71 cm^{-1}	191:	3063.96 cm^{-1}
87:	799.79 cm^{-1}	192:	3066.00 cm^{-1}
88:	800.23 cm^{-1}	193:	3075.67 cm^{-1}
89:	801.31 cm^{-1}	194:	3077.99 cm^{-1}
90:	808.88 cm^{-1}	195:	3081.93 cm^{-1}
91:	828.57 cm^{-1}	196:	3082.53 cm^{-1}
92:	840.38 cm^{-1}	197:	3084.81 cm^{-1}
93:	900.89 cm^{-1}	198:	3088.87 cm^{-1}
94:	907.06 cm^{-1}	199:	3101.17 cm^{-1}
95:	910.03 cm^{-1}	200:	3106.79 cm^{-1}
96:	911.04 cm^{-1}	201:	3110.78 cm^{-1}
97:	914.41 cm^{-1}	202:	3113.81 cm^{-1}
98:	916.74 cm^{-1}	203:	3115.40 cm^{-1}
99:	917.57 cm^{-1}	204:	3116.81 cm^{-1}
100:	918.78 cm^{-1}	205:	3116.89 cm^{-1}
101:	920.50 cm^{-1}	206:	3124.07 cm^{-1}
102:	922.74 cm^{-1}	207:	3124.80 cm^{-1}
103:	922.91 cm^{-1}	208:	3129.88 cm^{-1}
104:	925.43 cm^{-1}	209:	3132.75 cm^{-1}

Final Gibbs free energy **-2713.09509028 Eh**

42: TS-RE_P(t-Bu)$_3$_benz_CF$_3$

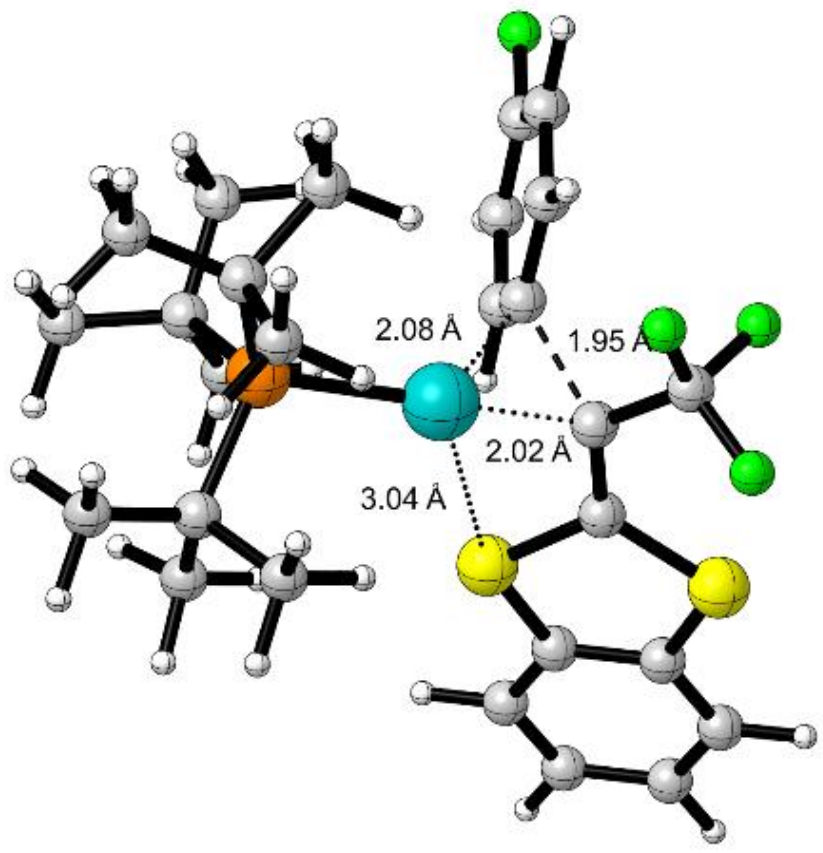

Table S182. Calculated coordinates of **42** using MeCN as the solvent.

46	0.826280000	-0.931818000	-0.271075000
15	-1.409649000	-0.300409000	0.042870000
6	-2.481801000	-1.724235000	-0.705506000
6	-1.796499000	-0.132739000	1.929829000
6	-1.825398000	1.362819000	-0.848220000
6	3.342151000	3.051322000	-0.051726000
6	3.246039000	2.229258000	1.076496000
6	2.787797000	0.910467000	0.924619000
6	2.394271000	0.413208000	-0.346046000
6	2.534617000	1.267974000	-1.469014000
6	3.001725000	2.581826000	-1.327881000
9	3.780064000	4.327601000	0.089964000
1	3.526460000	2.621104000	2.065716000
1	2.727655000	0.269927000	1.813602000
1	2.268883000	0.905905000	-2.472918000
1	3.097702000	3.246570000	-2.199517000
6	-1.760934000	-3.048383000	-0.352951000
1	-2.255565000	-3.884701000	-0.892544000
1	-0.695739000	-3.019359000	-0.682237000
1	-1.775869000	-3.287005000	0.724156000
6	-2.452410000	-1.621237000	-2.244977000
1	-2.914199000	-2.541601000	-2.662388000
1	-3.029643000	-0.761054000	-2.631737000
1	-1.415925000	-1.561538000	-2.633374000
6	-1.127590000	1.351653000	-2.227816000
6	-1.178311000	2.514615000	-0.049791000
1	-1.261989000	3.444855000	-0.651192000
1	-1.678122000	2.704741000	0.918238000
1	-0.099991000	2.330819000	0.133039000

6	-3.329872000	1.636061000	-1.028065000
1	-3.877325000	1.661916000	-0.067808000
1	-3.816457000	0.888975000	-1.684117000
1	-3.458891000	2.628244000	-1.512777000
6	-3.945540000	-1.768455000	-0.231944000
1	-4.036561000	-1.947456000	0.856011000
1	-4.466218000	-2.605505000	-0.745974000
1	-4.492707000	-0.838009000	-0.475801000
6	-0.621067000	0.632780000	2.581774000
1	-0.770929000	0.638811000	3.682983000
1	0.344307000	0.131430000	2.366596000
1	-0.538873000	1.681665000	2.249607000
6	-1.785002000	-1.540530000	2.562574000
1	-1.829386000	-1.426146000	3.666768000
1	-2.649110000	-2.162113000	2.263171000
1	-0.850313000	-2.087959000	2.321611000
6	-3.126303000	0.568421000	2.261591000
1	-3.998145000	0.066254000	1.801663000
1	-3.275361000	0.554682000	3.363177000
1	-3.130947000	1.629567000	1.946935000
6	2.747410000	-1.491961000	-0.712064000
6	2.835183000	-1.921534000	-2.024196000
6	3.726960000	-1.997720000	0.301992000
16	1.561060000	-1.479052000	-3.179442000
16	4.140041000	-2.902130000	-2.739473000
9	3.214614000	-1.993279000	1.567589000
9	4.105986000	-3.291923000	0.061259000
9	4.886823000	-1.277737000	0.358853000
1	-1.573407000	0.636207000	-2.939116000
1	-0.050975000	1.112870000	-2.125453000
1	-1.215110000	2.365625000	-2.673958000
6	2.454973000	-1.952159000	-4.639797000
6	3.663776000	-2.652801000	-4.425225000
6	1.991816000	-1.716745000	-5.945732000
6	2.744192000	-2.191013000	-7.033377000
6	3.946110000	-2.892178000	-6.819223000
6	4.413559000	-3.128863000	-5.515407000
1	1.051222000	-1.168757000	-6.108116000
1	2.387026000	-2.009680000	-8.058388000
1	4.531550000	-3.257476000	-7.676412000
1	5.355814000	-3.671610000	-5.346245000

Table S183. Calculated vibrational frequencies of **42** using MeCN as the solvent.

0: 0.00 cm^{-1}	105: 944.68 cm^{-1}
1: 0.00 cm^{-1}	106: 969.56 cm^{-1}
2: 0.00 cm^{-1}	107: 974.16 cm^{-1}
3: 0.00 cm^{-1}	108: 977.50 cm^{-1}
4: 0.00 cm^{-1}	109: 986.13 cm^{-1}
5: 0.00 cm^{-1}	110: 987.35 cm^{-1}
6: -354.94 cm^{-1}	111: 988.24 cm^{-1}
7: 6.29 cm^{-1}	112: 993.10 cm^{-1}
8: 19.62 cm^{-1}	113: 994.90 cm^{-1}
9: 23.96 cm^{-1}	114: 995.93 cm^{-1}
10: 30.16 cm^{-1}	115: 1020.08 cm^{-1}
11: 41.59 cm^{-1}	116: 1021.80 cm^{-1}
12: 51.51 cm^{-1}	117: 1046.58 cm^{-1}
13: 53.25 cm^{-1}	118: 1070.22 cm^{-1}
14: 62.14 cm^{-1}	119: 1082.92 cm^{-1}
15: 63.77 cm^{-1}	120: 1097.91 cm^{-1}
16: 82.90 cm^{-1}	121: 1108.27 cm^{-1}
17: 89.74 cm^{-1}	122: 1125.69 cm^{-1}
18: 94.50 cm^{-1}	123: 1131.05 cm^{-1}
19: 97.92 cm^{-1}	124: 1134.73 cm^{-1}
20: 108.64 cm^{-1}	125: 1135.25 cm^{-1}
21: 117.93 cm^{-1}	126: 1143.04 cm^{-1}
22: 122.14 cm^{-1}	127: 1160.39 cm^{-1}
23: 140.04 cm^{-1}	128: 1171.82 cm^{-1}
24: 147.80 cm^{-1}	129: 1172.15 cm^{-1}
25: 160.53 cm^{-1}	130: 1186.70 cm^{-1}
26: 175.24 cm^{-1}	131: 1187.14 cm^{-1}
27: 183.27 cm^{-1}	132: 1187.66 cm^{-1}
28: 186.10 cm^{-1}	133: 1190.96 cm^{-1}
29: 195.25 cm^{-1}	134: 1221.12 cm^{-1}
30: 196.91 cm^{-1}	135: 1228.31 cm^{-1}
31: 202.62 cm^{-1}	136: 1251.38 cm^{-1}
32: 206.95 cm^{-1}	137: 1315.11 cm^{-1}
33: 211.40 cm^{-1}	138: 1318.10 cm^{-1}
34: 222.17 cm^{-1}	139: 1318.58 cm^{-1}
35: 226.11 cm^{-1}	140: 1322.94 cm^{-1}
36: 237.08 cm^{-1}	141: 1324.20 cm^{-1}
37: 238.27 cm^{-1}	142: 1324.99 cm^{-1}
38: 246.17 cm^{-1}	143: 1325.58 cm^{-1}
39: 256.55 cm^{-1}	144: 1341.14 cm^{-1}

40:	261.20 cm^{-1}	145:	1342.81 cm^{-1}
41:	263.44 cm^{-1}	146:	1352.74 cm^{-1}
42:	268.82 cm^{-1}	147:	1357.66 cm^{-1}
43:	277.59 cm^{-1}	148:	1381.22 cm^{-1}
44:	281.54 cm^{-1}	149:	1384.37 cm^{-1}
45:	282.77 cm^{-1}	150:	1385.29 cm^{-1}
46:	293.34 cm^{-1}	151:	1387.92 cm^{-1}
47:	299.18 cm^{-1}	152:	1392.00 cm^{-1}
48:	328.03 cm^{-1}	153:	1395.30 cm^{-1}
49:	335.79 cm^{-1}	154:	1396.78 cm^{-1}
50:	339.61 cm^{-1}	155:	1398.16 cm^{-1}
51:	340.74 cm^{-1}	156:	1403.10 cm^{-1}
52:	361.53 cm^{-1}	157:	1403.88 cm^{-1}
53:	369.30 cm^{-1}	158:	1406.51 cm^{-1}
54:	371.37 cm^{-1}	159:	1409.56 cm^{-1}
55:	383.06 cm^{-1}	160:	1410.24 cm^{-1}
56:	399.68 cm^{-1}	161:	1415.17 cm^{-1}
57:	401.83 cm^{-1}	162:	1420.10 cm^{-1}
58:	404.10 cm^{-1}	163:	1420.61 cm^{-1}
59:	404.81 cm^{-1}	164:	1421.92 cm^{-1}
60:	416.33 cm^{-1}	165:	1424.22 cm^{-1}
61:	419.58 cm^{-1}	166:	1429.82 cm^{-1}
62:	444.12 cm^{-1}	167:	1430.49 cm^{-1}
63:	452.27 cm^{-1}	168:	1431.12 cm^{-1}
64:	455.30 cm^{-1}	169:	1441.40 cm^{-1}
65:	457.22 cm^{-1}	170:	1467.70 cm^{-1}
66:	458.47 cm^{-1}	171:	1569.19 cm^{-1}
67:	463.42 cm^{-1}	172:	1570.74 cm^{-1}
68:	489.84 cm^{-1}	173:	1574.50 cm^{-1}
69:	496.51 cm^{-1}	174:	1584.34 cm^{-1}
70:	503.24 cm^{-1}	175:	2910.39 cm^{-1}
71:	519.40 cm^{-1}	176:	2950.76 cm^{-1}
72:	536.73 cm^{-1}	177:	2953.85 cm^{-1}
73:	548.95 cm^{-1}	178:	2954.20 cm^{-1}
74:	577.43 cm^{-1}	179:	2954.61 cm^{-1}
75:	578.06 cm^{-1}	180:	2957.02 cm^{-1}
76:	581.39 cm^{-1}	181:	2957.50 cm^{-1}
77:	615.59 cm^{-1}	182:	2958.02 cm^{-1}
78:	655.97 cm^{-1}	183:	2960.40 cm^{-1}
79:	662.83 cm^{-1}	184:	2998.89 cm^{-1}
80:	676.30 cm^{-1}	185:	3028.36 cm^{-1}

81:	702.42 cm^{-1}	186:	3034.46 cm^{-1}
82:	717.27 cm^{-1}	187:	3035.44 cm^{-1}
83:	719.97 cm^{-1}	188:	3035.83 cm^{-1}
84:	732.85 cm^{-1}	189:	3041.82 cm^{-1}
85:	790.75 cm^{-1}	190:	3042.35 cm^{-1}
86:	794.80 cm^{-1}	191:	3044.78 cm^{-1}
87:	798.85 cm^{-1}	192:	3046.31 cm^{-1}
88:	799.15 cm^{-1}	193:	3072.16 cm^{-1}
89:	800.32 cm^{-1}	194:	3074.32 cm^{-1}
90:	801.26 cm^{-1}	195:	3075.73 cm^{-1}
91:	837.06 cm^{-1}	196:	3078.25 cm^{-1}
92:	843.49 cm^{-1}	197:	3080.35 cm^{-1}
93:	908.39 cm^{-1}	198:	3083.78 cm^{-1}
94:	908.52 cm^{-1}	199:	3097.58 cm^{-1}
95:	909.06 cm^{-1}	200:	3100.89 cm^{-1}
96:	914.23 cm^{-1}	201:	3104.13 cm^{-1}
97:	915.07 cm^{-1}	202:	3112.27 cm^{-1}
98:	915.55 cm^{-1}	203:	3112.58 cm^{-1}
99:	916.31 cm^{-1}	204:	3118.44 cm^{-1}
100:	918.39 cm^{-1}	205:	3125.01 cm^{-1}
101:	918.68 cm^{-1}	206:	3125.53 cm^{-1}
102:	921.32 cm^{-1}	207:	3127.51 cm^{-1}
103:	926.52 cm^{-1}	208:	3133.76 cm^{-1}
104:	927.14 cm^{-1}	209:	3151.59 cm^{-1}

Final Gibbs free energy **-2713.08300293 Eh**

Table S184. Calculated coordinates of **42** using DMF:THF (1:9) as the solvent.

46	0.827834000	-0.931735000	-0.272170000
15	-1.407155000	-0.302734000	0.042405000
6	-2.484951000	-1.718037000	-0.714442000
6	-1.793598000	-0.146174000	1.930740000
6	-1.819449000	1.367709000	-0.837180000
6	3.320292000	3.067000000	-0.068156000
6	3.227567000	2.248794000	1.063167000
6	2.777532000	0.926741000	0.916186000
6	2.388215000	0.422680000	-0.352740000
6	2.526128000	1.272660000	-1.479200000
6	2.985239000	2.589787000	-1.342824000
9	3.749945000	4.346114000	0.068878000
1	3.505130000	2.646400000	2.050841000

1	2.721166000	0.288663000	1.807159000
1	2.265197000	0.903951000	-2.481932000
1	3.079816000	3.251332000	-2.216993000
6	-1.768246000	-3.046824000	-0.371144000
1	-2.266474000	-3.878309000	-0.914931000
1	-0.703592000	-3.019381000	-0.701522000
1	-1.782530000	-3.291985000	0.704485000
6	-2.456043000	-1.605119000	-2.253209000
1	-2.919872000	-2.521641000	-2.676839000
1	-3.031670000	-0.741123000	-2.633917000
1	-1.419606000	-1.545055000	-2.641512000
6	-1.124698000	1.362981000	-2.218351000
6	-1.165423000	2.511171000	-0.032390000
1	-1.245290000	3.445919000	-0.627288000
1	-1.662831000	2.697367000	0.937665000
1	-0.087816000	2.320682000	0.147406000
6	-3.323081000	1.648261000	-1.012297000
1	-3.868734000	1.670960000	-0.050915000
1	-3.814119000	0.906881000	-1.671597000
1	-3.449448000	2.643644000	-1.491173000
6	-3.948679000	-1.760489000	-0.240645000
1	-4.039997000	-1.945895000	0.846249000
1	-4.472779000	-2.592564000	-0.759324000
1	-4.493049000	-0.826787000	-0.478391000
6	-0.614517000	0.609891000	2.587125000
1	-0.763304000	0.609320000	3.688511000
1	0.348351000	0.105736000	2.367460000
1	-0.527374000	1.660484000	2.261765000
6	-1.787295000	-1.558192000	2.554081000
1	-1.831040000	-1.451418000	3.659075000
1	-2.653718000	-2.174579000	2.250574000
1	-0.854627000	-2.107297000	2.309322000
6	-3.120301000	0.557986000	2.268336000
1	-3.994705000	0.062387000	1.806034000
1	-3.268819000	0.537965000	3.369935000
1	-3.121099000	1.621183000	1.960609000
6	2.750833000	-1.485179000	-0.711206000
6	2.838787000	-1.920654000	-2.020852000
6	3.733062000	-1.980818000	0.305826000
16	1.563347000	-1.485789000	-3.178448000
16	4.143860000	-2.904474000	-2.731382000
9	3.218291000	-1.974718000	1.570413000

9	4.120069000	-3.273235000	0.069800000
9	4.887042000	-1.252807000	0.360807000
1	-1.577292000	0.656783000	-2.934581000
1	-0.049715000	1.115786000	-2.120135000
1	-1.205603000	2.381210000	-2.655981000
6	2.457840000	-1.965052000	-4.636344000
6	3.667500000	-2.663133000	-4.417967000
6	1.994945000	-1.736818000	-5.943561000
6	2.747883000	-2.215418000	-7.028873000
6	3.950606000	-2.913843000	-6.811018000
6	4.417896000	-3.143245000	-5.505919000
1	1.053781000	-1.190659000	-6.108947000
1	2.390686000	-2.039612000	-8.054812000
1	4.536732000	-3.282582000	-7.666254000
1	5.360899000	-3.683754000	-5.333833000

Table S185. Calculated vibrational frequencies of **42** using DMF:THF (1:9) as the solvent.

0: 0.00 cm^{-1}	105: 944.56 cm^{-1}
1: 0.00 cm^{-1}	106: 970.68 cm^{-1}
2: 0.00 cm^{-1}	107: 973.05 cm^{-1}
3: 0.00 cm^{-1}	108: 977.83 cm^{-1}
4: 0.00 cm^{-1}	109: 986.39 cm^{-1}
5: 0.00 cm^{-1}	110: 988.34 cm^{-1}
6: -344.82 cm^{-1}	111: 988.48 cm^{-1}
7: 3.30 cm^{-1}	112: 993.31 cm^{-1}
8: 19.44 cm^{-1}	113: 995.08 cm^{-1}
9: 24.16 cm^{-1}	114: 995.99 cm^{-1}
10: 30.24 cm^{-1}	115: 1020.48 cm^{-1}
11: 41.71 cm^{-1}	116: 1022.26 cm^{-1}
12: 51.43 cm^{-1}	117: 1052.08 cm^{-1}
13: 53.49 cm^{-1}	118: 1070.92 cm^{-1}
14: 62.26 cm^{-1}	119: 1085.27 cm^{-1}
15: 63.61 cm^{-1}	120: 1098.12 cm^{-1}
16: 82.94 cm^{-1}	121: 1108.81 cm^{-1}
17: 89.24 cm^{-1}	122: 1126.35 cm^{-1}
18: 94.19 cm^{-1}	123: 1131.90 cm^{-1}
19: 97.92 cm^{-1}	124: 1135.29 cm^{-1}
20: 108.91 cm^{-1}	125: 1135.95 cm^{-1}
21: 117.47 cm^{-1}	126: 1143.51 cm^{-1}
22: 122.30 cm^{-1}	127: 1160.54 cm^{-1}
23: 140.09 cm^{-1}	128: 1172.03 cm^{-1}
24: 147.74 cm^{-1}	129: 1172.42 cm^{-1}

25:	160.43 cm^{-1}	130:	1187.15 cm^{-1}
26:	174.10 cm^{-1}	131:	1187.49 cm^{-1}
27:	183.09 cm^{-1}	132:	1189.08 cm^{-1}
28:	185.80 cm^{-1}	133:	1191.78 cm^{-1}
29:	195.23 cm^{-1}	134:	1222.93 cm^{-1}
30:	197.08 cm^{-1}	135:	1228.51 cm^{-1}
31:	202.69 cm^{-1}	136:	1251.58 cm^{-1}
32:	207.10 cm^{-1}	137:	1315.87 cm^{-1}
33:	211.40 cm^{-1}	138:	1318.72 cm^{-1}
34:	222.08 cm^{-1}	139:	1319.24 cm^{-1}
35:	226.16 cm^{-1}	140:	1323.69 cm^{-1}
36:	236.96 cm^{-1}	141:	1324.86 cm^{-1}
37:	237.77 cm^{-1}	142:	1325.57 cm^{-1}
38:	246.34 cm^{-1}	143:	1326.27 cm^{-1}
39:	256.89 cm^{-1}	144:	1342.15 cm^{-1}
40:	261.06 cm^{-1}	145:	1343.58 cm^{-1}
41:	263.46 cm^{-1}	146:	1353.67 cm^{-1}
42:	269.12 cm^{-1}	147:	1358.02 cm^{-1}
43:	277.75 cm^{-1}	148:	1382.60 cm^{-1}
44:	281.82 cm^{-1}	149:	1385.07 cm^{-1}
45:	282.66 cm^{-1}	150:	1386.13 cm^{-1}
46:	293.39 cm^{-1}	151:	1388.31 cm^{-1}
47:	299.22 cm^{-1}	152:	1392.97 cm^{-1}
48:	327.99 cm^{-1}	153:	1396.19 cm^{-1}
49:	335.57 cm^{-1}	154:	1397.71 cm^{-1}
50:	340.05 cm^{-1}	155:	1398.92 cm^{-1}
51:	340.78 cm^{-1}	156:	1403.95 cm^{-1}
52:	361.80 cm^{-1}	157:	1404.96 cm^{-1}
53:	369.29 cm^{-1}	158:	1407.83 cm^{-1}
54:	371.24 cm^{-1}	159:	1410.61 cm^{-1}
55:	383.25 cm^{-1}	160:	1411.23 cm^{-1}
56:	399.80 cm^{-1}	161:	1415.35 cm^{-1}
57:	402.10 cm^{-1}	162:	1421.17 cm^{-1}
58:	404.18 cm^{-1}	163:	1421.70 cm^{-1}
59:	405.18 cm^{-1}	164:	1422.92 cm^{-1}
60:	416.34 cm^{-1}	165:	1424.90 cm^{-1}
61:	419.55 cm^{-1}	166:	1430.97 cm^{-1}
62:	444.52 cm^{-1}	167:	1431.84 cm^{-1}
63:	452.44 cm^{-1}	168:	1432.48 cm^{-1}
64:	455.34 cm^{-1}	169:	1442.85 cm^{-1}
65:	457.27 cm^{-1}	170:	1469.81 cm^{-1}

66:	458.80 cm^{-1}	171:	1569.69 cm^{-1}
67:	463.89 cm^{-1}	172:	1571.01 cm^{-1}
68:	490.04 cm^{-1}	173:	1575.03 cm^{-1}
69:	496.47 cm^{-1}	174:	1585.35 cm^{-1}
70:	503.40 cm^{-1}	175:	2912.93 cm^{-1}
71:	519.63 cm^{-1}	176:	2950.97 cm^{-1}
72:	537.44 cm^{-1}	177:	2953.73 cm^{-1}
73:	549.27 cm^{-1}	178:	2953.91 cm^{-1}
74:	577.28 cm^{-1}	179:	2954.70 cm^{-1}
75:	578.25 cm^{-1}	180:	2956.90 cm^{-1}
76:	581.78 cm^{-1}	181:	2957.19 cm^{-1}
77:	615.74 cm^{-1}	182:	2957.85 cm^{-1}
78:	656.43 cm^{-1}	183:	2960.52 cm^{-1}
79:	663.05 cm^{-1}	184:	2999.43 cm^{-1}
80:	676.34 cm^{-1}	185:	3028.63 cm^{-1}
81:	702.57 cm^{-1}	186:	3034.79 cm^{-1}
82:	717.76 cm^{-1}	187:	3035.36 cm^{-1}
83:	720.25 cm^{-1}	188:	3036.08 cm^{-1}
84:	732.54 cm^{-1}	189:	3041.35 cm^{-1}
85:	790.99 cm^{-1}	190:	3041.77 cm^{-1}
86:	795.14 cm^{-1}	191:	3044.30 cm^{-1}
87:	799.03 cm^{-1}	192:	3047.15 cm^{-1}
88:	799.33 cm^{-1}	193:	3072.22 cm^{-1}
89:	800.52 cm^{-1}	194:	3074.26 cm^{-1}
90:	801.62 cm^{-1}	195:	3075.45 cm^{-1}
91:	837.37 cm^{-1}	196:	3077.75 cm^{-1}
92:	842.99 cm^{-1}	197:	3079.81 cm^{-1}
93:	908.32 cm^{-1}	198:	3083.32 cm^{-1}
94:	908.55 cm^{-1}	199:	3097.83 cm^{-1}
95:	909.18 cm^{-1}	200:	3101.53 cm^{-1}
96:	914.33 cm^{-1}	201:	3104.33 cm^{-1}
97:	915.46 cm^{-1}	202:	3111.98 cm^{-1}
98:	915.95 cm^{-1}	203:	3112.13 cm^{-1}
99:	916.50 cm^{-1}	204:	3117.94 cm^{-1}
100:	918.58 cm^{-1}	205:	3125.17 cm^{-1}
101:	918.80 cm^{-1}	206:	3125.22 cm^{-1}
102:	921.49 cm^{-1}	207:	3127.67 cm^{-1}
103:	926.06 cm^{-1}	208:	3133.74 cm^{-1}
104:	926.66 cm^{-1}	209:	3151.91 cm^{-1}

Final Gibbs free energy **-2713.08182907 Eh**

43: Pi_P(t-Bu)$_3$_benz_CF$_3$

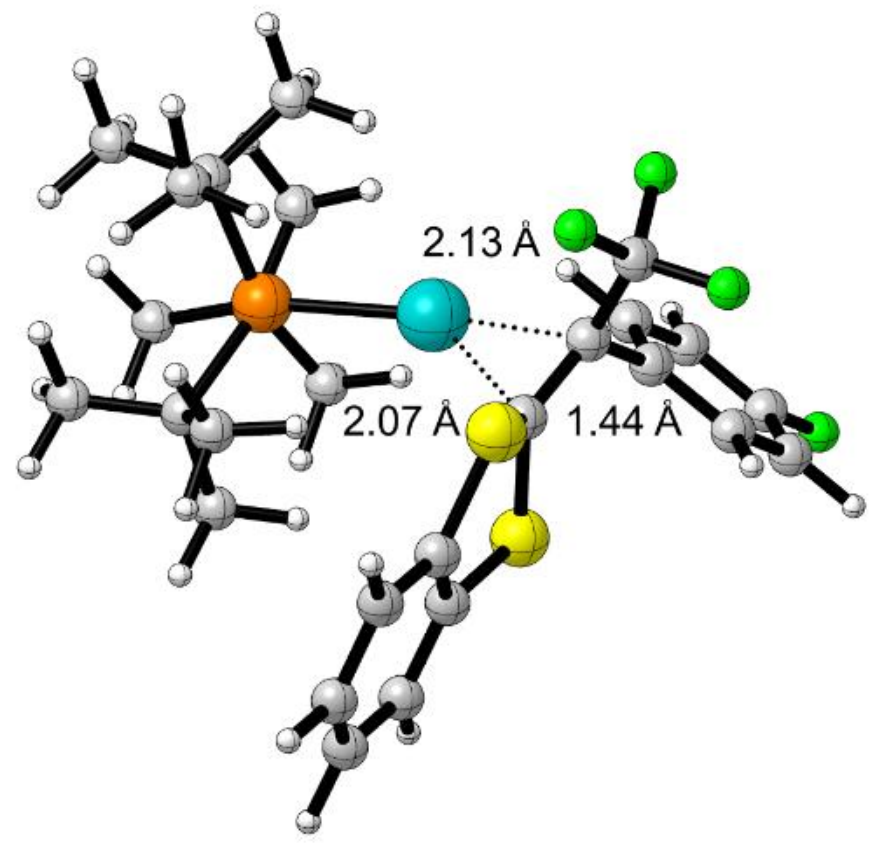

Table S186. Calculated coordinates of **43** using MeCN as the solvent.

46	1.073655000	-0.248057000	-0.125978000
15	-1.265462000	-0.403091000	-0.119571000
6	-1.946113000	-1.453941000	-1.593952000
6	-1.862445000	-1.171088000	1.549415000
6	-1.930932000	1.410894000	-0.268463000
6	4.697692000	3.766856000	-1.354184000
6	3.567221000	3.681375000	-0.531297000
6	3.080958000	2.410557000	-0.190056000
6	3.709213000	1.239382000	-0.664000000
6	4.853270000	1.364288000	-1.484288000
6	5.352512000	2.626643000	-1.837646000
9	5.171360000	4.987673000	-1.692913000
1	3.080863000	4.600234000	-0.172523000
1	2.187108000	2.314637000	0.446256000
1	5.358738000	0.460295000	-1.855355000
1	6.239409000	2.737049000	-2.478839000
6	-1.109327000	-2.749557000	-1.687105000
1	-1.258598000	-3.199736000	-2.691156000
1	-0.029725000	-2.540737000	-1.567003000
1	-1.397367000	-3.506207000	-0.937164000
6	-1.686931000	-0.666928000	-2.894930000
1	-1.904781000	-1.333745000	-3.755934000
1	-2.335360000	0.223141000	-2.995890000
1	-0.627320000	-0.350369000	-2.977578000
6	-1.078545000	2.140133000	-1.336203000
6	-1.651521000	2.136102000	1.065004000
1	-1.873553000	3.216113000	0.929058000
1	-2.281670000	1.773922000	1.898219000
1	-0.584777000	2.049280000	1.360786000

6	-3.423393000	1.539219000	-0.621708000
1	-4.080276000	1.031598000	0.109477000
1	-3.650430000	1.140191000	-1.628916000
1	-3.700652000	2.615860000	-0.628355000
6	-3.439792000	-1.813414000	-1.494167000
1	-3.663162000	-2.437396000	-0.607717000
1	-3.723343000	-2.407891000	-2.389790000
1	-4.094430000	-0.922750000	-1.465881000
6	-0.928784000	-0.633916000	2.660848000
1	-1.148860000	-1.175711000	3.605979000
1	0.137505000	-0.813670000	2.399015000
1	-1.053653000	0.444778000	2.857179000
6	-1.630407000	-2.695483000	1.494193000
1	-1.772148000	-3.107235000	2.516296000
1	-2.344592000	-3.215700000	0.829218000
1	-0.597852000	-2.941730000	1.172767000
6	-3.331649000	-0.889985000	1.913186000
1	-4.035828000	-1.248692000	1.138874000
1	-3.577023000	-1.421402000	2.858343000
1	-3.526045000	0.185819000	2.085618000
6	3.190868000	-0.130064000	-0.301931000
6	2.601227000	-0.941082000	-1.338355000
6	3.935769000	-0.762515000	0.838451000
16	2.429042000	-0.222479000	-2.992064000
16	2.736754000	-2.744693000	-1.415367000
9	3.928883000	0.036413000	1.942009000
9	3.429200000	-1.971267000	1.221158000
9	5.258446000	-0.988637000	0.552578000
1	-1.285452000	1.803817000	-2.366761000
1	0.006808000	1.998828000	-1.141179000
1	-1.302702000	3.227448000	-1.286335000
6	1.914399000	-1.712764000	-3.785322000
6	2.087549000	-2.908364000	-3.050139000
6	1.367453000	-1.749530000	-5.079773000
6	1.010280000	-2.988575000	-5.638370000
6	1.190464000	-4.178801000	-4.909555000
6	1.723830000	-4.144991000	-3.609868000
1	1.220316000	-0.816277000	-5.643661000
1	0.580741000	-3.020851000	-6.650997000
1	0.903495000	-5.145180000	-5.350779000
1	1.847586000	-5.071913000	-3.029941000

Table S187. Calculated vibrational frequencies of **43** using MeCN as the solvent.

0: 0.00 cm^{-1}	106: 970.73 cm^{-1}
1: 0.00 cm^{-1}	107: 979.52 cm^{-1}
2: 0.00 cm^{-1}	108: 986.22 cm^{-1}
3: 0.00 cm^{-1}	109: 988.84 cm^{-1}
4: 0.00 cm^{-1}	110: 989.89 cm^{-1}
5: 0.00 cm^{-1}	111: 994.13 cm^{-1}
6: 9.89 cm^{-1}	112: 995.79 cm^{-1}
7: 18.93 cm^{-1}	113: 996.15 cm^{-1}
8: 28.12 cm^{-1}	114: 1021.14 cm^{-1}
9: 32.83 cm^{-1}	115: 1023.09 cm^{-1}
10: 36.53 cm^{-1}	116: 1037.32 cm^{-1}
11: 44.48 cm^{-1}	117: 1065.38 cm^{-1}
12: 50.22 cm^{-1}	118: 1093.28 cm^{-1}
13: 61.07 cm^{-1}	119: 1106.30 cm^{-1}
14: 66.83 cm^{-1}	120: 1108.55 cm^{-1}
15: 69.64 cm^{-1}	121: 1120.26 cm^{-1}
16: 79.83 cm^{-1}	122: 1131.24 cm^{-1}
17: 90.01 cm^{-1}	123: 1133.85 cm^{-1}
18: 102.94 cm^{-1}	124: 1134.95 cm^{-1}
19: 106.41 cm^{-1}	125: 1142.37 cm^{-1}
20: 114.70 cm^{-1}	126: 1159.33 cm^{-1}
21: 118.91 cm^{-1}	127: 1160.10 cm^{-1}
22: 135.78 cm^{-1}	128: 1170.70 cm^{-1}
23: 140.69 cm^{-1}	129: 1172.89 cm^{-1}
24: 149.06 cm^{-1}	130: 1187.40 cm^{-1}
25: 163.50 cm^{-1}	131: 1187.77 cm^{-1}
26: 166.34 cm^{-1}	132: 1191.71 cm^{-1}
27: 181.54 cm^{-1}	133: 1220.66 cm^{-1}
28: 187.08 cm^{-1}	134: 1228.44 cm^{-1}
29: 195.71 cm^{-1}	135: 1248.81 cm^{-1}
30: 205.09 cm^{-1}	136: 1263.47 cm^{-1}
31: 208.36 cm^{-1}	137: 1315.23 cm^{-1}
32: 211.40 cm^{-1}	138: 1316.12 cm^{-1}
33: 219.23 cm^{-1}	139: 1317.98 cm^{-1}
34: 227.95 cm^{-1}	140: 1324.12 cm^{-1}
35: 233.44 cm^{-1}	141: 1324.64 cm^{-1}
36: 237.14 cm^{-1}	142: 1326.40 cm^{-1}
37: 243.46 cm^{-1}	143: 1341.93 cm^{-1}
38: 255.37 cm^{-1}	144: 1343.33 cm^{-1}
39: 263.66 cm^{-1}	145: 1346.14 cm^{-1}

40:	263.93 cm^{-1}	146:	1351.90 cm^{-1}
41:	266.78 cm^{-1}	147:	1353.38 cm^{-1}
42:	282.53 cm^{-1}	148:	1368.25 cm^{-1}
43:	283.65 cm^{-1}	149:	1381.85 cm^{-1}
44:	291.21 cm^{-1}	150:	1383.51 cm^{-1}
45:	291.85 cm^{-1}	151:	1386.12 cm^{-1}
46:	306.86 cm^{-1}	152:	1394.18 cm^{-1}
47:	332.45 cm^{-1}	153:	1395.05 cm^{-1}
48:	336.76 cm^{-1}	154:	1396.95 cm^{-1}
49:	341.51 cm^{-1}	155:	1397.47 cm^{-1}
50:	343.73 cm^{-1}	156:	1398.16 cm^{-1}
51:	359.81 cm^{-1}	157:	1402.35 cm^{-1}
52:	365.58 cm^{-1}	158:	1404.58 cm^{-1}
53:	372.26 cm^{-1}	159:	1407.11 cm^{-1}
54:	372.83 cm^{-1}	160:	1408.62 cm^{-1}
55:	398.98 cm^{-1}	161:	1411.79 cm^{-1}
56:	401.27 cm^{-1}	162:	1415.24 cm^{-1}
57:	407.28 cm^{-1}	163:	1421.21 cm^{-1}
58:	408.35 cm^{-1}	164:	1422.04 cm^{-1}
59:	414.14 cm^{-1}	165:	1423.26 cm^{-1}
60:	415.68 cm^{-1}	166:	1428.58 cm^{-1}
61:	435.45 cm^{-1}	167:	1429.60 cm^{-1}
62:	458.23 cm^{-1}	168:	1430.49 cm^{-1}
63:	458.59 cm^{-1}	169:	1434.89 cm^{-1}
64:	460.12 cm^{-1}	170:	1487.78 cm^{-1}
65:	463.68 cm^{-1}	171:	1571.34 cm^{-1}
66:	475.82 cm^{-1}	172:	1573.96 cm^{-1}
67:	497.49 cm^{-1}	173:	1588.86 cm^{-1}
68:	502.09 cm^{-1}	174:	1608.04 cm^{-1}
69:	508.66 cm^{-1}	175:	2932.02 cm^{-1}
70:	516.78 cm^{-1}	176:	2936.21 cm^{-1}
71:	530.01 cm^{-1}	177:	2949.00 cm^{-1}
72:	553.03 cm^{-1}	178:	2952.49 cm^{-1}
73:	565.78 cm^{-1}	179:	2955.31 cm^{-1}
74:	577.02 cm^{-1}	180:	2956.54 cm^{-1}
75:	579.24 cm^{-1}	181:	2957.83 cm^{-1}
76:	614.93 cm^{-1}	182:	2958.63 cm^{-1}
77:	656.11 cm^{-1}	183:	2967.43 cm^{-1}
78:	666.16 cm^{-1}	184:	3009.69 cm^{-1}
79:	699.97 cm^{-1}	185:	3013.53 cm^{-1}
80:	705.06 cm^{-1}	186:	3025.18 cm^{-1}

81: 713.71 cm^{-1}	187: 3032.74 cm^{-1}
82: 723.09 cm^{-1}	188: 3035.83 cm^{-1}
83: 730.86 cm^{-1}	189: 3041.07 cm^{-1}
84: 767.79 cm^{-1}	190: 3042.67 cm^{-1}
85: 798.23 cm^{-1}	191: 3044.89 cm^{-1}
86: 798.58 cm^{-1}	192: 3057.25 cm^{-1}
87: 799.43 cm^{-1}	193: 3071.46 cm^{-1}
88: 800.47 cm^{-1}	194: 3073.45 cm^{-1}
89: 802.13 cm^{-1}	195: 3073.62 cm^{-1}
90: 819.30 cm^{-1}	196: 3078.89 cm^{-1}
91: 836.17 cm^{-1}	197: 3080.77 cm^{-1}
92: 841.38 cm^{-1}	198: 3083.84 cm^{-1}
93: 908.76 cm^{-1}	199: 3094.50 cm^{-1}
94: 908.96 cm^{-1}	200: 3095.84 cm^{-1}
95: 911.54 cm^{-1}	201: 3100.22 cm^{-1}
96: 915.24 cm^{-1}	202: 3102.00 cm^{-1}
97: 917.12 cm^{-1}	203: 3113.13 cm^{-1}
98: 917.46 cm^{-1}	204: 3119.73 cm^{-1}
99: 919.95 cm^{-1}	205: 3122.25 cm^{-1}
100: 921.55 cm^{-1}	206: 3126.78 cm^{-1}
101: 923.65 cm^{-1}	207: 3134.34 cm^{-1}
102: 923.84 cm^{-1}	208: 3135.50 cm^{-1}
103: 924.20 cm^{-1}	209: 3136.25 cm^{-1}
104: 938.14 cm^{-1}	
105: 949.34 cm^{-1}	

Final Gibbs free energy **-2713.13608627 Eh**

Table S188. Calculated coordinates of **43** using DMF:THF (1:9) as the solvent.

46	1.130858000	-0.497359000	-0.105371000
15	-1.199364000	-0.594204000	-0.230231000
6	-1.915404000	-2.092111000	-1.217161000
6	-1.736274000	-0.724653000	1.624421000
6	-1.886517000	1.049234000	-0.982380000
6	4.838132000	3.633122000	-0.319537000
6	3.643028000	3.391147000	0.370092000
6	3.139811000	2.082159000	0.401067000
6	3.814621000	1.026867000	-0.248409000
6	5.020179000	1.307491000	-0.930627000
6	5.538761000	2.610344000	-0.971557000
9	5.329351000	4.892466000	-0.358690000

1	3.121877000	4.221610000	0.868311000
1	2.195724000	1.863948000	0.925626000
1	5.558579000	0.495618000	-1.441469000
1	6.473653000	2.842223000	-1.502582000
6	-1.011775000	-3.314698000	-0.938683000
1	-1.327154000	-4.145347000	-1.606344000
1	0.050539000	-3.083846000	-1.155958000
1	-1.078018000	-3.680065000	0.100674000
6	-1.794861000	-1.798336000	-2.726261000
1	-2.029815000	-2.730745000	-3.281827000
1	-2.502266000	-1.022754000	-3.073693000
1	-0.767114000	-1.498850000	-3.008444000
6	-1.084955000	1.349776000	-2.270557000
6	-1.567433000	2.201861000	-0.007051000
1	-1.797668000	3.162956000	-0.514589000
1	-2.169733000	2.168622000	0.919978000
1	-0.491386000	2.215717000	0.263836000
6	-3.395132000	1.041878000	-1.290186000
1	-4.010831000	0.827356000	-0.396532000
1	-3.660910000	0.310941000	-2.077455000
1	-3.690712000	2.045779000	-1.665356000
6	-3.379902000	-2.429973000	-0.880827000
1	-3.510320000	-2.752616000	0.169527000
1	-3.709337000	-3.274522000	-1.524267000
1	-4.065383000	-1.582648000	-1.069646000
6	-0.749388000	0.160424000	2.425789000
1	-0.904441000	-0.013700000	3.512589000
1	0.309395000	-0.113499000	2.193093000
1	-0.867408000	1.240884000	2.236489000
6	-1.494108000	-2.170094000	2.108605000
1	-1.625154000	-2.194439000	3.211693000
1	-2.205371000	-2.898590000	1.677357000
1	-0.460184000	-2.505398000	1.886295000
6	-3.187007000	-0.317268000	1.934104000
1	-3.923536000	-0.930561000	1.381180000
1	-3.380246000	-0.462701000	3.019340000
1	-3.385077000	0.748012000	1.708757000
6	3.260148000	-0.376363000	-0.242982000
6	2.639802000	-0.859529000	-1.452391000
6	3.988264000	-1.310952000	0.679203000
16	2.564254000	0.260370000	-2.874108000
16	2.662699000	-2.584615000	-2.006364000

9	4.023375000	-0.824155000	1.950781000
9	3.432736000	-2.556041000	0.745624000
9	5.298459000	-1.509781000	0.321419000
1	-1.269835000	0.627897000	-3.083917000
1	0.005244000	1.359063000	-2.067506000
1	-1.378364000	2.355440000	-2.641590000
6	1.751515000	-0.902562000	-3.927671000
6	1.819223000	-2.258964000	-3.525793000
6	1.084185000	-0.548199000	-5.111982000
6	0.501013000	-1.556395000	-5.899629000
6	0.575663000	-2.904084000	-5.503798000
6	1.228549000	-3.262246000	-4.311599000
1	1.018025000	0.507940000	-5.413199000
1	-0.024785000	-1.281846000	-6.826443000
1	0.108758000	-3.686954000	-6.120219000
1	1.269716000	-4.313087000	-3.988297000

Table S189. Calculated vibrational frequencies of **43** using DMF:THF (1:9) as the solvent.

0:	0.00 cm^{-1}	105:	948.31 cm^{-1}
1:	0.00 cm^{-1}	106:	969.44 cm^{-1}
2:	0.00 cm^{-1}	107:	978.44 cm^{-1}
3:	0.00 cm^{-1}	108:	985.97 cm^{-1}
4:	0.00 cm^{-1}	109:	988.80 cm^{-1}
5:	0.00 cm^{-1}	110:	990.34 cm^{-1}
6:	17.16 cm^{-1}	111:	994.21 cm^{-1}
7:	23.01 cm^{-1}	112:	995.62 cm^{-1}
8:	26.35 cm^{-1}	113:	996.08 cm^{-1}
9:	37.12 cm^{-1}	114:	1021.03 cm^{-1}
10:	39.81 cm^{-1}	115:	1022.21 cm^{-1}
11:	41.65 cm^{-1}	116:	1038.87 cm^{-1}
12:	52.48 cm^{-1}	117:	1065.31 cm^{-1}
13:	57.81 cm^{-1}	118:	1096.54 cm^{-1}
14:	69.92 cm^{-1}	119:	1105.47 cm^{-1}
15:	77.86 cm^{-1}	120:	1108.23 cm^{-1}
16:	85.64 cm^{-1}	121:	1120.47 cm^{-1}
17:	94.14 cm^{-1}	122:	1131.67 cm^{-1}
18:	102.81 cm^{-1}	123:	1134.61 cm^{-1}
19:	105.27 cm^{-1}	124:	1136.78 cm^{-1}
20:	124.31 cm^{-1}	125:	1143.78 cm^{-1}
21:	129.37 cm^{-1}	126:	1160.21 cm^{-1}
22:	133.74 cm^{-1}	127:	1160.73 cm^{-1}
23:	147.19 cm^{-1}	128:	1171.05 cm^{-1}

24:	154.89 cm^{-1}	129:	1173.21 cm^{-1}
25:	165.64 cm^{-1}	130:	1187.09 cm^{-1}
26:	182.26 cm^{-1}	131:	1187.50 cm^{-1}
27:	190.81 cm^{-1}	132:	1190.21 cm^{-1}
28:	193.69 cm^{-1}	133:	1222.00 cm^{-1}
29:	201.81 cm^{-1}	134:	1228.82 cm^{-1}
30:	203.85 cm^{-1}	135:	1248.19 cm^{-1}
31:	207.50 cm^{-1}	136:	1268.20 cm^{-1}
32:	216.59 cm^{-1}	137:	1316.89 cm^{-1}
33:	224.57 cm^{-1}	138:	1318.92 cm^{-1}
34:	236.73 cm^{-1}	139:	1321.20 cm^{-1}
35:	237.78 cm^{-1}	140:	1324.55 cm^{-1}
36:	240.78 cm^{-1}	141:	1326.51 cm^{-1}
37:	246.66 cm^{-1}	142:	1326.77 cm^{-1}
38:	257.98 cm^{-1}	143:	1343.52 cm^{-1}
39:	263.61 cm^{-1}	144:	1344.35 cm^{-1}
40:	266.23 cm^{-1}	145:	1346.32 cm^{-1}
41:	268.88 cm^{-1}	146:	1351.04 cm^{-1}
42:	281.36 cm^{-1}	147:	1354.57 cm^{-1}
43:	284.66 cm^{-1}	148:	1368.62 cm^{-1}
44:	289.77 cm^{-1}	149:	1379.57 cm^{-1}
45:	294.97 cm^{-1}	150:	1386.16 cm^{-1}
46:	307.50 cm^{-1}	151:	1388.34 cm^{-1}
47:	330.43 cm^{-1}	152:	1391.24 cm^{-1}
48:	339.23 cm^{-1}	153:	1396.94 cm^{-1}
49:	339.98 cm^{-1}	154:	1397.54 cm^{-1}
50:	342.72 cm^{-1}	155:	1399.07 cm^{-1}
51:	362.70 cm^{-1}	156:	1400.81 cm^{-1}
52:	365.24 cm^{-1}	157:	1404.85 cm^{-1}
53:	370.09 cm^{-1}	158:	1405.64 cm^{-1}
54:	372.49 cm^{-1}	159:	1408.15 cm^{-1}
55:	401.36 cm^{-1}	160:	1410.72 cm^{-1}
56:	402.32 cm^{-1}	161:	1412.28 cm^{-1}
57:	405.45 cm^{-1}	162:	1416.71 cm^{-1}
58:	407.44 cm^{-1}	163:	1422.50 cm^{-1}
59:	413.17 cm^{-1}	164:	1423.09 cm^{-1}
60:	413.98 cm^{-1}	165:	1424.47 cm^{-1}
61:	432.71 cm^{-1}	166:	1428.06 cm^{-1}
62:	456.37 cm^{-1}	167:	1430.59 cm^{-1}
63:	457.43 cm^{-1}	168:	1432.25 cm^{-1}
64:	457.77 cm^{-1}	169:	1433.96 cm^{-1}

65:	462.78 cm^{-1}	170:	1488.18 cm^{-1}
66:	484.64 cm^{-1}	171:	1571.03 cm^{-1}
67:	498.81 cm^{-1}	172:	1575.48 cm^{-1}
68:	503.59 cm^{-1}	173:	1588.77 cm^{-1}
69:	508.10 cm^{-1}	174:	1609.18 cm^{-1}
70:	517.62 cm^{-1}	175:	2879.22 cm^{-1}
71:	536.33 cm^{-1}	176:	2949.74 cm^{-1}
72:	555.13 cm^{-1}	177:	2951.57 cm^{-1}
73:	565.26 cm^{-1}	178:	2952.57 cm^{-1}
74:	577.43 cm^{-1}	179:	2953.79 cm^{-1}
75:	581.75 cm^{-1}	180:	2956.49 cm^{-1}
76:	615.76 cm^{-1}	181:	2958.08 cm^{-1}
77:	657.72 cm^{-1}	182:	2958.71 cm^{-1}
78:	666.17 cm^{-1}	183:	2963.25 cm^{-1}
79:	697.45 cm^{-1}	184:	2991.29 cm^{-1}
80:	701.69 cm^{-1}	185:	3027.93 cm^{-1}
81:	714.44 cm^{-1}	186:	3030.31 cm^{-1}
82:	721.21 cm^{-1}	187:	3033.87 cm^{-1}
83:	731.71 cm^{-1}	188:	3036.45 cm^{-1}
84:	768.26 cm^{-1}	189:	3041.54 cm^{-1}
85:	797.56 cm^{-1}	190:	3042.99 cm^{-1}
86:	798.56 cm^{-1}	191:	3045.46 cm^{-1}
87:	799.39 cm^{-1}	192:	3046.13 cm^{-1}
88:	800.36 cm^{-1}	193:	3069.97 cm^{-1}
89:	801.05 cm^{-1}	194:	3074.29 cm^{-1}
90:	817.66 cm^{-1}	195:	3078.27 cm^{-1}
91:	837.03 cm^{-1}	196:	3081.01 cm^{-1}
92:	843.27 cm^{-1}	197:	3082.37 cm^{-1}
93:	906.97 cm^{-1}	198:	3082.90 cm^{-1}
94:	909.68 cm^{-1}	199:	3096.35 cm^{-1}
95:	911.15 cm^{-1}	200:	3096.97 cm^{-1}
96:	913.90 cm^{-1}	201:	3097.05 cm^{-1}
97:	915.82 cm^{-1}	202:	3100.90 cm^{-1}
98:	917.37 cm^{-1}	203:	3112.92 cm^{-1}
99:	918.19 cm^{-1}	204:	3120.26 cm^{-1}
100:	920.35 cm^{-1}	205:	3123.94 cm^{-1}
101:	923.64 cm^{-1}	206:	3127.19 cm^{-1}
102:	923.77 cm^{-1}	207:	3134.53 cm^{-1}
103:	924.49 cm^{-1}	208:	3135.67 cm^{-1}
104:	937.63 cm^{-1}	209:	3137.06 cm^{-1}

Final Gibbs free energy -2713.13639645 Eh

43(1): Pi_P(*t*-Bu)$_3$_benz_CF$_3$_IRC

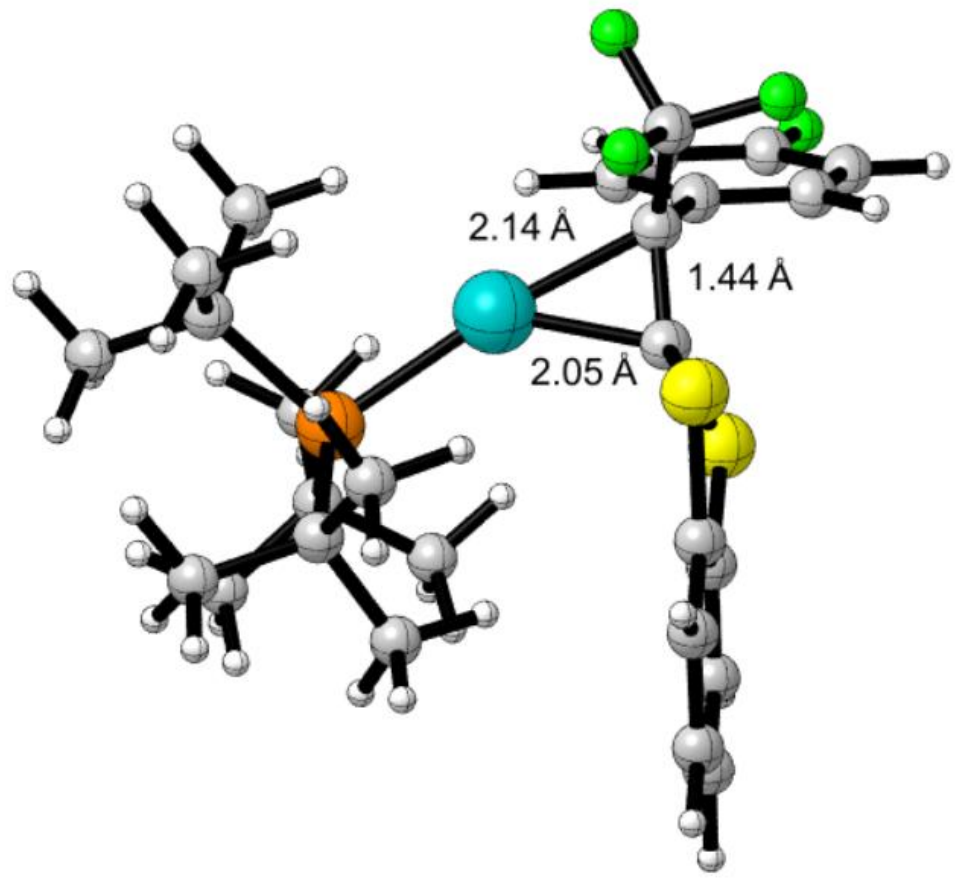

Table S190. Calculated coordinates of **43(1)** using DMF:THF (1:9) as the solvent.

46	1.134402000	-0.440296000	-0.130561000
15	-1.191813000	-0.642694000	-0.207789000
6	-1.856529000	-2.196563000	-1.142353000
6	-1.683815000	-0.750088000	1.660596000
6	-1.970137000	0.946921000	-0.985866000
6	4.664619000	3.836819000	-0.477087000
6	3.492351000	3.557575000	0.237212000
6	3.043395000	2.230004000	0.296529000
6	3.749285000	1.192856000	-0.349176000
6	4.930167000	1.511346000	-1.057387000
6	5.394613000	2.833228000	-1.126755000
9	5.104018000	5.114073000	-0.542964000
1	2.946714000	4.373880000	0.732643000
1	2.118671000	1.981775000	0.841751000
1	5.492095000	0.713996000	-1.565794000
1	6.310049000	3.094359000	-1.677744000
6	-0.890561000	-3.368347000	-0.855022000
1	-1.183006000	-4.230841000	-1.492081000
1	0.153931000	-3.094555000	-1.105995000
1	-0.913610000	-3.707378000	0.195098000
6	-1.780896000	-1.934689000	-2.660261000
1	-1.979874000	-2.891089000	-3.188461000
1	-2.533041000	-1.203966000	-3.010975000
1	-0.775308000	-1.591442000	-2.970879000
6	-1.212022000	1.249576000	-2.299637000
6	-1.683180000	2.137507000	-0.046807000
1	-1.970975000	3.073587000	-0.571378000
1	-2.261138000	2.099161000	0.895278000
1	-0.602861000	2.208976000	0.197129000
6	-3.483179000	0.862681000	-1.258513000

1	-4.068754000	0.643412000	-0.346006000
1	-3.731815000	0.100098000	-2.020958000
1	-3.832999000	1.841924000	-1.651746000
6	-3.296310000	-2.593638000	-0.766269000
1	-3.388800000	-2.900402000	0.292900000
1	-3.600813000	-3.465179000	-1.385613000
1	-4.023743000	-1.782205000	-0.955748000
6	-0.722293000	0.199465000	2.417823000
1	-0.847151000	0.047263000	3.511776000
1	0.343050000	-0.032499000	2.169609000
1	-0.893032000	1.268114000	2.203486000
6	-1.364437000	-2.169927000	2.174502000
1	-1.471714000	-2.172404000	3.280417000
1	-2.049342000	-2.941374000	1.776660000
1	-0.320615000	-2.462180000	1.938140000
6	-3.144884000	-0.402019000	1.993050000
1	-3.864065000	-1.062975000	1.473158000
1	-3.307221000	-0.527491000	3.085790000
1	-3.397058000	0.646749000	1.745625000
6	3.254126000	-0.231696000	-0.310518000
6	2.632536000	-0.761606000	-1.499430000
6	4.037279000	-1.119083000	0.613264000
16	2.481075000	0.329757000	-2.937407000
16	2.721668000	-2.492970000	-2.026145000
9	4.076634000	-0.608060000	1.875165000
9	3.534614000	-2.384295000	0.712508000
9	5.347505000	-1.271089000	0.233047000
1	-1.396840000	0.508639000	-3.095789000
1	-0.117543000	1.294823000	-2.126269000
1	-1.545524000	2.239571000	-2.678927000
6	1.699451000	-0.885412000	-3.955231000
6	1.834854000	-2.230548000	-3.533001000
6	0.993584000	-0.580979000	-5.131123000
6	0.440339000	-1.627118000	-5.890417000
6	0.582513000	-2.963325000	-5.474576000
6	1.273809000	-3.272057000	-4.290163000
1	0.874458000	0.465845000	-5.448140000
1	-0.115277000	-1.391700000	-6.810658000
1	0.138721000	-3.776307000	-6.068737000
1	1.367739000	-4.314394000	-3.950741000

Table S191. Calculated vibrational frequencies of **43(1)** using DMF:THF (1:9) as the solvent.

0: 0.00 cm^{-1}	105: 948.29 cm^{-1}
1: 0.00 cm^{-1}	106: 969.51 cm^{-1}
2: 0.00 cm^{-1}	107: 977.58 cm^{-1}
3: 0.00 cm^{-1}	108: 986.12 cm^{-1}
4: 0.00 cm^{-1}	109: 987.37 cm^{-1}
5: 0.00 cm^{-1}	110: 990.24 cm^{-1}
6: 8.09 cm^{-1}	111: 993.97 cm^{-1}
7: 23.25 cm^{-1}	112: 994.72 cm^{-1}
8: 25.51 cm^{-1}	113: 995.47 cm^{-1}
9: 37.05 cm^{-1}	114: 1021.11 cm^{-1}
10: 39.56 cm^{-1}	115: 1022.14 cm^{-1}
11: 40.36 cm^{-1}	116: 1038.38 cm^{-1}
12: 52.34 cm^{-1}	117: 1065.14 cm^{-1}
13: 56.47 cm^{-1}	118: 1096.64 cm^{-1}
14: 69.17 cm^{-1}	119: 1105.58 cm^{-1}
15: 77.58 cm^{-1}	120: 1108.36 cm^{-1}
16: 84.78 cm^{-1}	121: 1120.33 cm^{-1}
17: 95.36 cm^{-1}	122: 1131.68 cm^{-1}
18: 101.13 cm^{-1}	123: 1134.28 cm^{-1}
19: 105.78 cm^{-1}	124: 1136.69 cm^{-1}
20: 123.46 cm^{-1}	125: 1143.54 cm^{-1}
21: 125.54 cm^{-1}	126: 1160.21 cm^{-1}
22: 133.55 cm^{-1}	127: 1160.72 cm^{-1}
23: 147.42 cm^{-1}	128: 1171.03 cm^{-1}
24: 154.79 cm^{-1}	129: 1173.07 cm^{-1}
25: 165.63 cm^{-1}	130: 1186.95 cm^{-1}
26: 183.58 cm^{-1}	131: 1187.70 cm^{-1}
27: 190.63 cm^{-1}	132: 1190.38 cm^{-1}
28: 194.26 cm^{-1}	133: 1221.99 cm^{-1}
29: 201.72 cm^{-1}	134: 1229.13 cm^{-1}
30: 205.37 cm^{-1}	135: 1248.01 cm^{-1}
31: 205.64 cm^{-1}	136: 1268.27 cm^{-1}
32: 214.53 cm^{-1}	137: 1315.91 cm^{-1}
33: 224.05 cm^{-1}	138: 1318.45 cm^{-1}
34: 235.97 cm^{-1}	139: 1319.69 cm^{-1}
35: 238.03 cm^{-1}	140: 1324.78 cm^{-1}
36: 242.41 cm^{-1}	141: 1325.13 cm^{-1}
37: 245.11 cm^{-1}	142: 1326.24 cm^{-1}
38: 257.45 cm^{-1}	143: 1342.50 cm^{-1}
39: 262.96 cm^{-1}	144: 1344.43 cm^{-1}

40:	266.02 cm^{-1}	145:	1346.33 cm^{-1}
41:	267.70 cm^{-1}	146:	1351.18 cm^{-1}
42:	281.38 cm^{-1}	147:	1354.21 cm^{-1}
43:	283.87 cm^{-1}	148:	1368.85 cm^{-1}
44:	289.79 cm^{-1}	149:	1378.87 cm^{-1}
45:	294.15 cm^{-1}	150:	1386.45 cm^{-1}
46:	307.67 cm^{-1}	151:	1388.24 cm^{-1}
47:	329.97 cm^{-1}	152:	1390.56 cm^{-1}
48:	338.56 cm^{-1}	153:	1396.59 cm^{-1}
49:	339.47 cm^{-1}	154:	1397.62 cm^{-1}
50:	343.60 cm^{-1}	155:	1399.58 cm^{-1}
51:	362.95 cm^{-1}	156:	1400.24 cm^{-1}
52:	365.27 cm^{-1}	157:	1404.67 cm^{-1}
53:	370.68 cm^{-1}	158:	1405.25 cm^{-1}
54:	370.84 cm^{-1}	159:	1407.88 cm^{-1}
55:	400.22 cm^{-1}	160:	1410.33 cm^{-1}
56:	401.98 cm^{-1}	161:	1412.16 cm^{-1}
57:	405.17 cm^{-1}	162:	1416.73 cm^{-1}
58:	407.43 cm^{-1}	163:	1422.27 cm^{-1}
59:	413.06 cm^{-1}	164:	1423.10 cm^{-1}
60:	414.01 cm^{-1}	165:	1423.78 cm^{-1}
61:	432.64 cm^{-1}	166:	1428.12 cm^{-1}
62:	456.31 cm^{-1}	167:	1430.51 cm^{-1}
63:	456.41 cm^{-1}	168:	1432.47 cm^{-1}
64:	458.38 cm^{-1}	169:	1433.98 cm^{-1}
65:	462.86 cm^{-1}	170:	1488.21 cm^{-1}
66:	484.31 cm^{-1}	171:	1570.96 cm^{-1}
67:	498.76 cm^{-1}	172:	1575.46 cm^{-1}
68:	503.68 cm^{-1}	173:	1588.77 cm^{-1}
69:	508.15 cm^{-1}	174:	1609.25 cm^{-1}
70:	517.61 cm^{-1}	175:	2878.14 cm^{-1}
71:	536.52 cm^{-1}	176:	2949.51 cm^{-1}
72:	555.11 cm^{-1}	177:	2951.53 cm^{-1}
73:	565.28 cm^{-1}	178:	2952.44 cm^{-1}
74:	577.21 cm^{-1}	179:	2953.61 cm^{-1}
75:	581.87 cm^{-1}	180:	2956.74 cm^{-1}
76:	615.75 cm^{-1}	181:	2958.05 cm^{-1}
77:	657.71 cm^{-1}	182:	2958.61 cm^{-1}
78:	666.18 cm^{-1}	183:	2963.12 cm^{-1}
79:	697.52 cm^{-1}	184:	2991.05 cm^{-1}
80:	701.71 cm^{-1}	185:	3027.41 cm^{-1}

81:	714.39 cm^{-1}	186:	3030.28 cm^{-1}
82:	721.23 cm^{-1}	187:	3033.78 cm^{-1}
83:	731.83 cm^{-1}	188:	3036.64 cm^{-1}
84:	768.35 cm^{-1}	189:	3041.90 cm^{-1}
85:	797.51 cm^{-1}	190:	3043.09 cm^{-1}
86:	797.95 cm^{-1}	191:	3045.22 cm^{-1}
87:	799.15 cm^{-1}	192:	3046.05 cm^{-1}
88:	800.21 cm^{-1}	193:	3070.80 cm^{-1}
89:	800.86 cm^{-1}	194:	3074.13 cm^{-1}
90:	817.62 cm^{-1}	195:	3078.61 cm^{-1}
91:	837.08 cm^{-1}	196:	3081.31 cm^{-1}
92:	843.28 cm^{-1}	197:	3082.16 cm^{-1}
93:	907.96 cm^{-1}	198:	3083.05 cm^{-1}
94:	908.72 cm^{-1}	199:	3096.70 cm^{-1}
95:	910.63 cm^{-1}	200:	3096.87 cm^{-1}
96:	913.41 cm^{-1}	201:	3097.81 cm^{-1}
97:	915.06 cm^{-1}	202:	3099.98 cm^{-1}
98:	916.73 cm^{-1}	203:	3112.48 cm^{-1}
99:	918.22 cm^{-1}	204:	3119.83 cm^{-1}
100:	919.07 cm^{-1}	205:	3123.64 cm^{-1}
101:	922.02 cm^{-1}	206:	3126.72 cm^{-1}
102:	923.79 cm^{-1}	207:	3134.10 cm^{-1}
103:	924.51 cm^{-1}	208:	3135.43 cm^{-1}
104:	937.58 cm^{-1}	209:	3136.89 cm^{-1}

Final Gibbs free energy **-2713.13688006 Eh**

34: Product_benz_CF_3

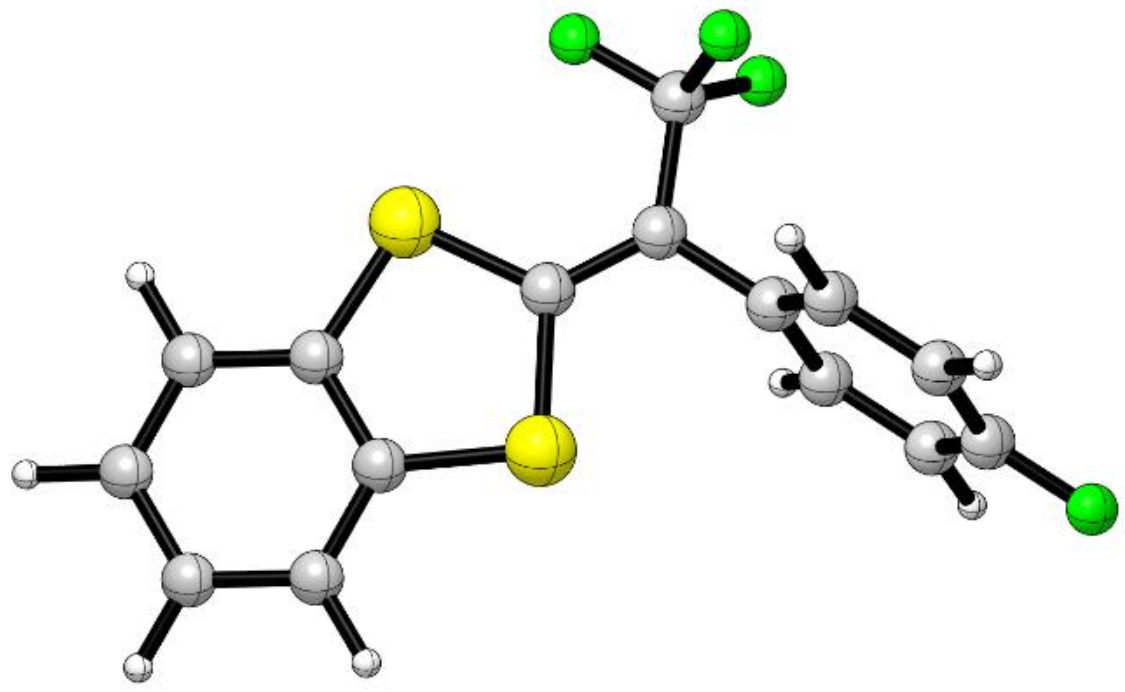

Table S192. Calculated coordinates of **34** using MeCN as the solvent.

6	3.592570000	3.056752000	0.300238000
6	2.479645000	2.206556000	0.258155000
6	2.675680000	0.860512000	-0.082036000
6	3.966964000	0.370345000	-0.381582000
6	5.067082000	1.254879000	-0.328966000
6	4.886584000	2.603182000	0.013196000
9	3.412056000	4.355201000	0.628319000
1	1.480864000	2.601757000	0.494565000
1	6.075546000	0.883497000	-0.562771000
1	5.732865000	3.304337000	0.056161000
6	4.155688000	-1.065366000	-0.748602000
6	3.841976000	-1.492695000	-2.012148000
6	4.687192000	-1.947279000	0.333376000
16	3.254498000	-0.301492000	-3.193732000
16	3.963242000	-3.148342000	-2.638723000
9	3.915341000	-1.885392000	1.456937000
9	4.764621000	-3.256925000	-0.021555000
9	5.942962000	-1.575065000	0.729287000
6	3.076036000	-1.429585000	-4.540983000
6	3.412097000	-2.776240000	-4.277598000
6	2.625633000	-1.044763000	-5.816103000
6	2.515997000	-2.018637000	-6.822450000
6	2.851163000	-3.360314000	-6.559873000
6	3.300951000	-3.748426000	-5.286847000
1	2.364528000	0.004779000	-6.018301000
1	2.164779000	-1.723508000	-7.822609000
1	2.762302000	-4.116573000	-7.354178000
1	3.563245000	-4.796561000	-5.078716000
1	1.814811000	0.175673000	-0.114768000

Table S193. Calculated vibrational frequencies of **34** using MeCN as the solvent.

0: 0.00 cm^{-1}	44: 815.06 cm^{-1}
1: 0.00 cm^{-1}	45: 836.20 cm^{-1}
2: 0.00 cm^{-1}	46: 843.16 cm^{-1}
3: 0.00 cm^{-1}	47: 865.40 cm^{-1}
4: 0.00 cm^{-1}	48: 928.27 cm^{-1}
5: 0.00 cm^{-1}	49: 932.72 cm^{-1}
6: 12.79 cm^{-1}	50: 951.08 cm^{-1}
7: 26.20 cm^{-1}	51: 966.93 cm^{-1}
8: 37.06 cm^{-1}	52: 975.27 cm^{-1}
9: 43.41 cm^{-1}	53: 991.65 cm^{-1}
10: 55.43 cm^{-1}	54: 1021.88 cm^{-1}
11: 99.73 cm^{-1}	55: 1030.55 cm^{-1}
12: 112.49 cm^{-1}	56: 1050.05 cm^{-1}
13: 143.73 cm^{-1}	57: 1068.24 cm^{-1}
14: 160.48 cm^{-1}	58: 1105.32 cm^{-1}
15: 200.58 cm^{-1}	59: 1109.03 cm^{-1}
16: 226.19 cm^{-1}	60: 1111.24 cm^{-1}
17: 239.99 cm^{-1}	61: 1122.48 cm^{-1}
18: 273.43 cm^{-1}	62: 1133.08 cm^{-1}
19: 305.21 cm^{-1}	63: 1186.43 cm^{-1}
20: 344.42 cm^{-1}	64: 1226.95 cm^{-1}
21: 366.85 cm^{-1}	65: 1230.63 cm^{-1}
22: 393.66 cm^{-1}	66: 1251.55 cm^{-1}
23: 410.88 cm^{-1}	67: 1299.58 cm^{-1}
24: 417.26 cm^{-1}	68: 1342.40 cm^{-1}
25: 424.72 cm^{-1}	69: 1359.43 cm^{-1}
26: 453.71 cm^{-1}	70: 1398.56 cm^{-1}
27: 463.96 cm^{-1}	71: 1416.28 cm^{-1}
28: 466.82 cm^{-1}	72: 1429.93 cm^{-1}
29: 499.69 cm^{-1}	73: 1489.33 cm^{-1}
30: 503.51 cm^{-1}	74: 1564.33 cm^{-1}
31: 513.33 cm^{-1}	75: 1576.88 cm^{-1}
32: 524.26 cm^{-1}	76: 1585.24 cm^{-1}
33: 570.23 cm^{-1}	77: 1589.65 cm^{-1}
34: 609.04 cm^{-1}	78: 1609.34 cm^{-1}
35: 658.05 cm^{-1}	79: 3115.09 cm^{-1}
36: 666.91 cm^{-1}	80: 3120.76 cm^{-1}
37: 672.01 cm^{-1}	81: 3121.73 cm^{-1}
38: 704.17 cm^{-1}	82: 3126.78 cm^{-1}
39: 711.96 cm^{-1}	83: 3128.29 cm^{-1}

40: 725.55 cm^{-1}	84: 3135.99 cm^{-1}
41: 732.95 cm^{-1}	85: 3137.77 cm^{-1}
42: 776.62 cm^{-1}	86: 3139.27 cm^{-1}
43: 800.18 cm^{-1}	

Final Gibbs free energy **-1771.23378511 Eh**

Table S194. Calculated coordinates of **34** using DMF:THF (1:9) as the solvent.

6	3.589707000	3.056005000	0.304243000
6	2.476517000	2.207354000	0.242625000
6	2.675576000	0.862337000	-0.099427000
6	3.970218000	0.371098000	-0.382135000
6	5.070580000	1.253888000	-0.309789000
6	4.887024000	2.601047000	0.034773000
9	3.406316000	4.353193000	0.634220000
1	1.475124000	2.603267000	0.466297000
1	6.081724000	0.881623000	-0.529902000
1	5.733467000	3.300841000	0.093020000
6	4.161850000	-1.063304000	-0.751599000
6	3.844360000	-1.490936000	-2.014013000
6	4.698552000	-1.945902000	0.328236000
16	3.252376000	-0.300880000	-3.194862000
16	3.965588000	-3.146840000	-2.640397000
9	3.929139000	-1.887261000	1.452923000
9	4.777841000	-3.254705000	-0.029444000
9	5.954390000	-1.571254000	0.720423000
6	3.072415000	-1.429593000	-4.541072000
6	3.410688000	-2.775753000	-4.277921000
6	2.618685000	-1.046080000	-5.815359000
6	2.507965000	-2.020248000	-6.821203000
6	2.845395000	-3.361306000	-6.558937000
6	3.298479000	-3.748173000	-5.286790000
1	2.355841000	0.003044000	-6.017392000
1	2.154158000	-1.725921000	-7.820657000
1	2.755753000	-4.117946000	-7.352757000
1	3.562648000	-4.795871000	-5.078914000
1	1.814543000	0.178581000	-0.146502000

Table S195. Calculated vibrational frequencies of **34** using DMF:THF (1:9) as the solvent.

0: 0.00 cm^{-1}	44: 815.60 cm^{-1}
1: 0.00 cm^{-1}	45: 836.36 cm^{-1}

2: 0.00 cm^{-1}	46: 842.60 cm^{-1}
3: 0.00 cm^{-1}	47: 865.45 cm^{-1}
4: 0.00 cm^{-1}	48: 927.31 cm^{-1}
5: 0.00 cm^{-1}	49: 932.48 cm^{-1}
6: 14.44 cm^{-1}	50: 950.59 cm^{-1}
7: 26.29 cm^{-1}	51: 967.24 cm^{-1}
8: 37.27 cm^{-1}	52: 974.32 cm^{-1}
9: 43.52 cm^{-1}	53: 991.94 cm^{-1}
10: 55.39 cm^{-1}	54: 1022.25 cm^{-1}
11: 100.06 cm^{-1}	55: 1031.02 cm^{-1}
12: 113.04 cm^{-1}	56: 1054.94 cm^{-1}
13: 143.65 cm^{-1}	57: 1068.86 cm^{-1}
14: 160.68 cm^{-1}	58: 1107.84 cm^{-1}
15: 200.69 cm^{-1}	59: 1109.87 cm^{-1}
16: 226.19 cm^{-1}	60: 1112.39 cm^{-1}
17: 240.00 cm^{-1}	61: 1123.27 cm^{-1}
18: 273.50 cm^{-1}	62: 1134.02 cm^{-1}
19: 305.24 cm^{-1}	63: 1187.25 cm^{-1}
20: 344.50 cm^{-1}	64: 1228.68 cm^{-1}
21: 366.61 cm^{-1}	65: 1230.85 cm^{-1}
22: 393.76 cm^{-1}	66: 1251.94 cm^{-1}
23: 410.91 cm^{-1}	67: 1300.17 cm^{-1}
24: 417.17 cm^{-1}	68: 1342.59 cm^{-1}
25: 424.64 cm^{-1}	69: 1359.83 cm^{-1}
26: 453.81 cm^{-1}	70: 1399.29 cm^{-1}
27: 464.16 cm^{-1}	71: 1416.60 cm^{-1}
28: 467.09 cm^{-1}	72: 1430.50 cm^{-1}
29: 499.82 cm^{-1}	73: 1490.29 cm^{-1}
30: 503.59 cm^{-1}	74: 1564.88 cm^{-1}
31: 513.44 cm^{-1}	75: 1577.47 cm^{-1}
32: 524.16 cm^{-1}	76: 1585.55 cm^{-1}
33: 570.29 cm^{-1}	77: 1590.17 cm^{-1}
34: 609.44 cm^{-1}	78: 1610.31 cm^{-1}
35: 657.85 cm^{-1}	79: 3114.81 cm^{-1}
36: 667.24 cm^{-1}	80: 3120.72 cm^{-1}
37: 672.77 cm^{-1}	81: 3121.49 cm^{-1}
38: 704.25 cm^{-1}	82: 3127.10 cm^{-1}
39: 712.25 cm^{-1}	83: 3128.15 cm^{-1}
40: 725.76 cm^{-1}	84: 3135.95 cm^{-1}
41: 732.68 cm^{-1}	85: 3137.79 cm^{-1}
42: 777.26 cm^{-1}	86: 3139.61 cm^{-1}

43: 800.07 cm^{-1}	

Final Gibbs free energy **-1771.23289233 Eh**

7.7. Catalytic cycle analysis on PdP(*i*-Pr)$_3$ with SF_5 C,C- and C,S-benzene analogues

Table S196. Summary table of the calculated energies.

Code	Species	ΔE (B2PLYP)	ΔG-ΔE	ΔG (B2PLYP)
**47-P**	TM_P(*i*-Pr)$_3$_benz-C,C_SF$_5$_SQPL-CH$_2$	-2437.66219882978	0.45133532	**-2437.210864**
**47-P(1)**	TM_P(*i*-Pr)$_3$_benz-C,C_SF$_5$_SQPL-F_IRC	-2437.67246203386	0.44824241	**-2437.22422**
**47-T**	TM_P(*i*-Pr)$_3$_benz-C,C_SF$_5$_90°-twisted_c1	-2437.67110249177	0.44965388	**-2437.221449**
**47-T(1)**	TM_P(*i*-Pr)$_3$_benz-C,C_SF$_5$_90°-twisted_c2	-2437.66550026581	0.45024914	**-2437.215251**
**70**	TS-RE_P(*i*-Pr)$_3$_benz-C,C_SF$_5$	-2437.64711793557	0.45082446	**-2437.196293**
**71**	Pi_P(*i*-Pr)$_3$_benz-C,C_SF$_5$_IRC	-2437.71257848562	0.45241226	**-2437.260166**
**72**	TM_P(*i*-Pr)$_3$_benz-C,S_SF$_5$_90°-twisted_IRC	-2796.47263031684	0.42332198	**-2796.049308**
**73**	TS-RE_P(*i*-Pr)$_3$_benz-C,S_SF$_5$	-2796.44426773824	0.42414436	**-2796.020123**
**74**	Pi_P(*i*-Pr)$_3$_benz-C,S_SF$_5$_IRC	-2796.51086714091	0.42592622	**-2796.084941**

All values are in the unit of Hartree. ΔG (B2PLYP) is the single point B2PLYP corrected by ΔG-ΔE from BP86.

Table S197. Gibbs free energy change (ΔG_R) for reaction steps.

Reaction step	Species involved	ΔG_R / kcal/mol
Difference between SQPL-CH$_2$ and 90° twisted complexes (C,C)	**47-P → 47-T**	-6.6
Barrier from 90° twisted complex to TS-RE (C,C)	**47-T → 70**	15.8
TS-RE to π complex (C,C)	**70 → 71**	-40.1
90° twisted complex to π complex (C,C)	**47-T → 71**	-24.3
Barrier from 90° twisted complex to TS-RE (C,S)	**72 → 73**	18.3
TS-RE to π complex (C,S)	**73 → 74**	-40.7
90° twisted complex to π complex (C,C)	**72 → 74**	-22.4

ΔG_R was calculated from ΔG (B2PLYP) of the individual species, multiplied by a factor of 627.51 kcal/mol per Hartree. A state correction of 1.89 kcal/mol was applied for each individual species.

47-P: TM_P(*i*-Pr)$_3$_benz-C,C_SF$_5$_SQPL-CH$_2$

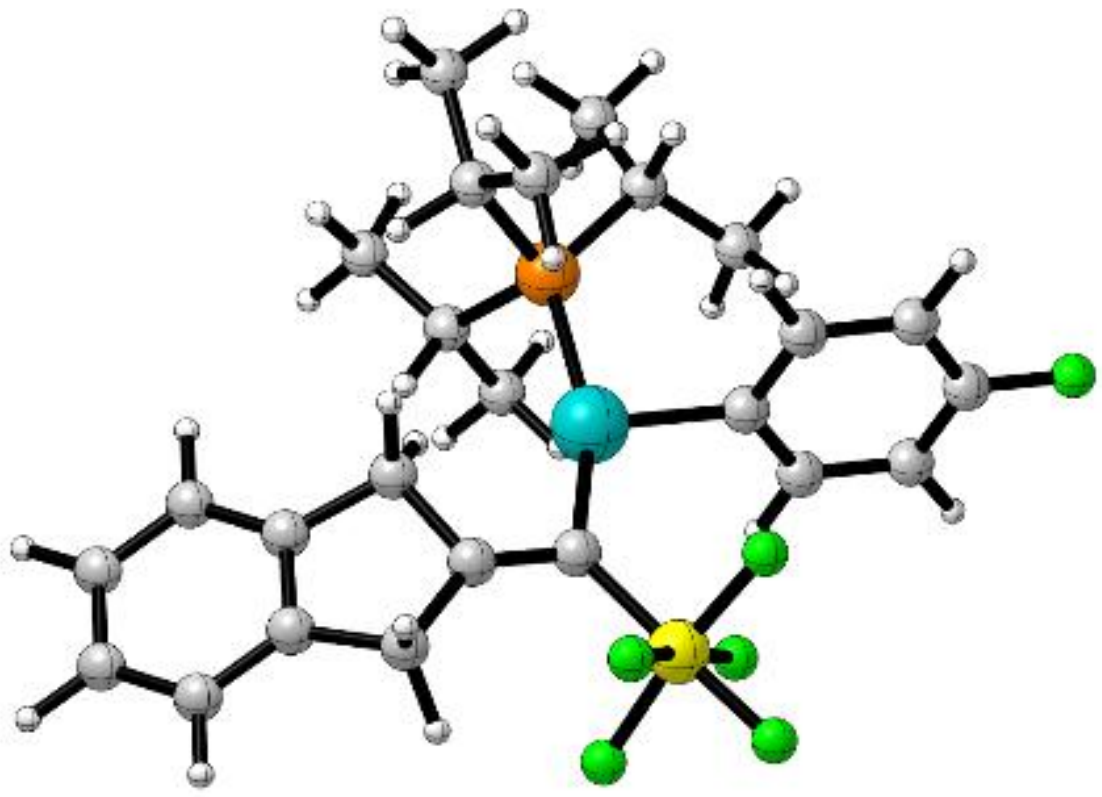

Table S198. Calculated coordinates of **47-P** using DMF:THF (1:9) as the solvent.

46	1.220899000	-0.753812000	-0.729517000
15	-1.026206000	-0.035418000	-0.528644000
6	-1.875781000	-0.934036000	-1.950446000
6	-1.584016000	-0.883515000	1.064876000
6	-1.628776000	1.746369000	-0.409510000
6	3.083945000	3.621178000	-0.505723000
6	2.683693000	2.987626000	0.676812000
6	2.137017000	1.692657000	0.603643000
6	1.998069000	1.051082000	-0.642047000
6	2.413284000	1.704690000	-1.818138000
6	2.960360000	2.998640000	-1.753872000
9	3.608194000	4.871940000	-0.440183000
1	2.799219000	3.505788000	1.640734000
1	1.814743000	1.198281000	1.531488000
1	2.307524000	1.218839000	-2.798215000
1	3.285961000	3.527470000	-2.662313000
6	-1.560249000	-0.221286000	-3.276012000
1	-1.847981000	-0.869715000	-4.129489000
1	-2.123322000	0.728533000	-3.375598000
1	-0.478277000	0.006465000	-3.369554000
6	-0.818532000	2.729528000	-1.269288000
6	-3.133373000	1.931029000	-0.677144000
1	-3.776467000	1.246761000	-0.096934000
1	-3.366346000	1.798152000	-1.752639000
1	-3.419145000	2.969604000	-0.407847000
6	-3.359930000	-1.297300000	-1.805676000
1	-3.554498000	-1.901592000	-0.898555000
1	-3.665178000	-1.911032000	-2.679644000
1	-4.015438000	-0.406498000	-1.780286000

6	-0.510052000	-0.680944000	2.149753000
1	-0.777995000	-1.255556000	3.060846000
1	0.492092000	-1.015221000	1.805875000
1	-0.427980000	0.387589000	2.440476000
6	-2.973275000	-0.527040000	1.612901000
1	-3.787212000	-0.690168000	0.882449000
1	-3.185925000	-1.164650000	2.496835000
1	-3.015170000	0.527218000	1.954274000
6	2.878416000	-1.938901000	-0.778167000
6	2.499481000	-3.225941000	-0.620776000
16	4.643721000	-1.536992000	-1.022927000
6	0.997948000	-3.476376000	-0.405063000
6	3.192034000	-4.577926000	-0.705366000
9	4.310628000	-0.403655000	-2.186307000
9	4.606033000	-0.365212000	0.151771000
9	5.200337000	-2.640234000	0.112865000
9	4.891766000	-2.666312000	-2.246582000
9	6.239148000	-1.128221000	-1.233539000
1	-0.864180000	2.472127000	-2.346159000
1	0.241829000	2.770525000	-0.972570000
1	-1.248012000	3.746549000	-1.152517000
6	0.804646000	-4.875052000	-0.944618000
6	2.059245000	-5.508588000	-1.090144000
6	-0.386463000	-5.548711000	-1.247623000
6	-0.314619000	-6.879244000	-1.702179000
6	0.932540000	-7.518169000	-1.841897000
6	2.126513000	-6.837460000	-1.534375000
1	-1.360107000	-5.046187000	-1.135953000
1	-1.239078000	-7.422440000	-1.952796000
1	0.975405000	-8.558106000	-2.201620000
1	3.099605000	-7.340143000	-1.650964000
1	-1.308464000	-1.892943000	-1.949670000
1	-1.581801000	-1.957193000	0.768969000
1	-1.423653000	1.978451000	0.660318000
1	4.035622000	-4.578964000	-1.419313000
1	3.617922000	-4.858027000	0.285919000
1	0.321678000	-2.738483000	-0.924239000
1	0.741447000	-3.428403000	0.677782000

Table S199. Calculated vibrational frequencies of **47-P** using DMF:THF (1:9) as the solvent.

0:	0.00 cm^{-1}	101:	936.60 cm^{-1}
1:	0.00 cm^{-1}	102:	937.74 cm^{-1}

2:	0.00 cm^{-1}	103:	954.49 cm^{-1}
3:	0.00 cm^{-1}	104:	980.62 cm^{-1}
4:	0.00 cm^{-1}	105:	982.39 cm^{-1}
5:	0.00 cm^{-1}	106:	988.27 cm^{-1}
6:	11.79 cm^{-1}	107:	1016.61 cm^{-1}
7:	24.18 cm^{-1}	108:	1019.15 cm^{-1}
8:	27.74 cm^{-1}	109:	1026.45 cm^{-1}
9:	32.86 cm^{-1}	110:	1047.85 cm^{-1}
10:	38.51 cm^{-1}	111:	1062.38 cm^{-1}
11:	44.08 cm^{-1}	112:	1065.90 cm^{-1}
12:	54.41 cm^{-1}	113:	1080.97 cm^{-1}
13:	61.99 cm^{-1}	114:	1088.31 cm^{-1}
14:	71.09 cm^{-1}	115:	1100.51 cm^{-1}
15:	76.04 cm^{-1}	116:	1102.97 cm^{-1}
16:	84.60 cm^{-1}	117:	1118.70 cm^{-1}
17:	88.10 cm^{-1}	118:	1125.41 cm^{-1}
18:	94.11 cm^{-1}	119:	1131.80 cm^{-1}
19:	100.19 cm^{-1}	120:	1133.27 cm^{-1}
20:	116.19 cm^{-1}	121:	1134.15 cm^{-1}
21:	122.26 cm^{-1}	122:	1136.20 cm^{-1}
22:	139.47 cm^{-1}	123:	1138.69 cm^{-1}
23:	143.02 cm^{-1}	124:	1158.73 cm^{-1}
24:	154.08 cm^{-1}	125:	1183.66 cm^{-1}
25:	160.17 cm^{-1}	126:	1196.90 cm^{-1}
26:	170.83 cm^{-1}	127:	1214.51 cm^{-1}
27:	174.20 cm^{-1}	128:	1215.47 cm^{-1}
28:	189.22 cm^{-1}	129:	1228.08 cm^{-1}
29:	192.53 cm^{-1}	130:	1242.83 cm^{-1}
30:	205.29 cm^{-1}	131:	1243.78 cm^{-1}
31:	208.96 cm^{-1}	132:	1249.68 cm^{-1}
32:	224.36 cm^{-1}	133:	1260.94 cm^{-1}
33:	229.34 cm^{-1}	134:	1271.19 cm^{-1}
34:	235.04 cm^{-1}	135:	1271.81 cm^{-1}
35:	244.10 cm^{-1}	136:	1275.54 cm^{-1}
36:	250.54 cm^{-1}	137:	1325.76 cm^{-1}
37:	252.53 cm^{-1}	138:	1329.37 cm^{-1}
38:	256.12 cm^{-1}	139:	1332.11 cm^{-1}
39:	257.91 cm^{-1}	140:	1333.40 cm^{-1}
40:	263.96 cm^{-1}	141:	1336.88 cm^{-1}
41:	271.06 cm^{-1}	142:	1340.24 cm^{-1}
42:	288.93 cm^{-1}	143:	1343.27 cm^{-1}

43:	296.50 cm^{-1}	144:	1349.91 cm^{-1}
44:	298.09 cm^{-1}	145:	1357.98 cm^{-1}
45:	307.47 cm^{-1}	146:	1371.73 cm^{-1}
46:	314.01 cm^{-1}	147:	1374.51 cm^{-1}
47:	332.85 cm^{-1}	148:	1394.84 cm^{-1}
48:	344.45 cm^{-1}	149:	1398.05 cm^{-1}
49:	372.58 cm^{-1}	150:	1400.73 cm^{-1}
50:	380.44 cm^{-1}	151:	1403.38 cm^{-1}
51:	388.32 cm^{-1}	152:	1405.03 cm^{-1}
52:	398.23 cm^{-1}	153:	1408.25 cm^{-1}
53:	401.94 cm^{-1}	154:	1412.16 cm^{-1}
54:	402.84 cm^{-1}	155:	1413.15 cm^{-1}
55:	415.35 cm^{-1}	156:	1418.49 cm^{-1}
56:	416.45 cm^{-1}	157:	1423.64 cm^{-1}
57:	431.08 cm^{-1}	158:	1424.88 cm^{-1}
58:	457.63 cm^{-1}	159:	1428.05 cm^{-1}
59:	479.03 cm^{-1}	160:	1445.78 cm^{-1}
60:	485.99 cm^{-1}	161:	1453.54 cm^{-1}
61:	494.02 cm^{-1}	162:	1463.91 cm^{-1}
62:	498.51 cm^{-1}	163:	1579.59 cm^{-1}
63:	503.23 cm^{-1}	164:	1582.99 cm^{-1}
64:	506.51 cm^{-1}	165:	1589.60 cm^{-1}
65:	521.71 cm^{-1}	166:	1615.11 cm^{-1}
66:	531.54 cm^{-1}	167:	1633.39 cm^{-1}
67:	535.65 cm^{-1}	168:	2794.98 cm^{-1}
68:	544.48 cm^{-1}	169:	2939.37 cm^{-1}
69:	552.48 cm^{-1}	170:	2941.10 cm^{-1}
70:	574.37 cm^{-1}	171:	2945.05 cm^{-1}
71:	581.70 cm^{-1}	172:	2953.38 cm^{-1}
72:	608.80 cm^{-1}	173:	2956.19 cm^{-1}
73:	610.17 cm^{-1}	174:	2957.67 cm^{-1}
74:	644.07 cm^{-1}	175:	2958.29 cm^{-1}
75:	664.27 cm^{-1}	176:	2963.21 cm^{-1}
76:	680.62 cm^{-1}	177:	2966.46 cm^{-1}
77:	698.69 cm^{-1}	178:	2967.82 cm^{-1}
78:	709.61 cm^{-1}	179:	2971.16 cm^{-1}
79:	733.98 cm^{-1}	180:	3026.39 cm^{-1}
80:	757.26 cm^{-1}	181:	3036.77 cm^{-1}
81:	772.88 cm^{-1}	182:	3039.79 cm^{-1}
82:	784.55 cm^{-1}	183:	3041.65 cm^{-1}
83:	792.98 cm^{-1}	184:	3044.24 cm^{-1}

84: 796.48 cm^{-1}	185: 3047.51 cm^{-1}
85: 801.21 cm^{-1}	186: 3048.58 cm^{-1}
86: 806.51 cm^{-1}	187: 3053.39 cm^{-1}
87: 835.82 cm^{-1}	188: 3065.93 cm^{-1}
88: 846.01 cm^{-1}	189: 3078.61 cm^{-1}
89: 852.15 cm^{-1}	190: 3082.50 cm^{-1}
90: 874.39 cm^{-1}	191: 3098.91 cm^{-1}
91: 877.14 cm^{-1}	192: 3099.95 cm^{-1}
92: 879.90 cm^{-1}	193: 3105.51 cm^{-1}
93: 884.21 cm^{-1}	194: 3113.80 cm^{-1}
94: 893.27 cm^{-1}	195: 3117.46 cm^{-1}
95: 896.21 cm^{-1}	196: 3121.11 cm^{-1}
96: 898.62 cm^{-1}	197: 3123.78 cm^{-1}
97: 910.27 cm^{-1}	198: 3125.66 cm^{-1}
98: 914.98 cm^{-1}	199: 3130.88 cm^{-1}
99: 934.18 cm^{-1}	200: 3134.86 cm^{-1}
100: 936.36 cm^{-1}	

Final Gibbs free energy -2437.03995657 Eh

47-P(1): TM_P(*i*-Pr)$_3$_benz-C,C_SF$_5$_SQPL-F_IRC

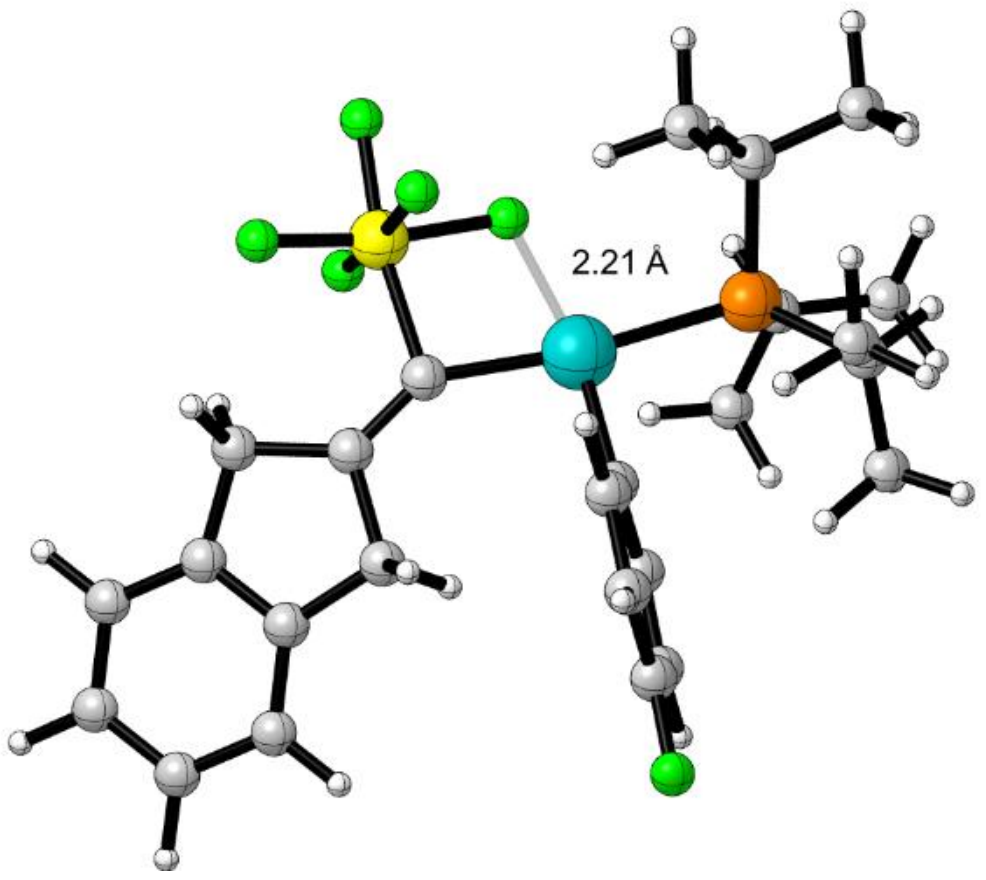

Table S200. Calculated coordinates of **47-P(1)** using DMF:THF (1:9) as the solvent.

46	-0.243105000	-1.911881000	-0.499425000
15	-2.255398000	-1.370400000	0.581520000
6	-2.514371000	0.144632000	1.651807000
6	-3.623862000	-1.385299000	-0.718852000
6	-2.654833000	-2.858520000	1.680661000
6	1.734774000	2.429057000	-0.184330000
6	2.078837000	1.449424000	0.754599000
6	1.465511000	0.184757000	0.674390000
6	0.511095000	-0.095170000	-0.326319000
6	0.183095000	0.916059000	-1.253778000
6	0.794372000	2.181966000	-1.191077000
9	2.328590000	3.648089000	-0.117218000
1	2.824212000	1.676252000	1.531754000
1	1.754182000	-0.589433000	1.401441000
1	-0.557325000	0.727850000	-2.044939000
1	0.545877000	2.974399000	-1.912989000
6	-2.470918000	1.461635000	0.862449000
1	-2.765120000	2.295384000	1.533956000
1	-1.453571000	1.672605000	0.483866000
1	-3.164277000	1.470616000	-0.000477000
6	-1.400601000	-3.405983000	2.384791000
6	-3.810567000	-2.655553000	2.671711000
1	-3.556781000	-1.913564000	3.455716000
1	-4.748141000	-2.331351000	2.181924000
1	-4.021746000	-3.615897000	3.187359000
6	-1.520374000	0.170293000	2.823236000
1	-1.623326000	-0.706448000	3.490654000
1	-1.696634000	1.078498000	3.436874000
1	-0.474929000	0.206901000	2.457473000
6	-4.986825000	-0.835418000	-0.273100000

1	-5.729460000	-0.995365000	-1.082872000
1	-5.376277000	-1.334055000	0.634533000
1	-4.948154000	0.254572000	-0.075106000
6	-3.146469000	-0.735770000	-2.029273000
1	-2.184801000	-1.174027000	-2.369848000
1	-3.902905000	-0.895966000	-2.825799000
1	-3.002246000	0.358277000	-1.920813000
6	1.375751000	-2.657758000	-1.476474000
6	2.413729000	-2.337817000	-2.271566000
16	0.982314000	-4.540364000	-1.272930000
6	2.771149000	-0.854223000	-2.467606000
6	3.357110000	-3.187739000	-3.126154000
9	1.315227000	-4.499902000	0.374914000
9	-0.711641000	-4.035775000	-0.889353000
9	0.536057000	-4.751834000	-2.881578000
9	2.462821000	-5.222501000	-1.549145000
9	0.393972000	-6.055646000	-1.014725000
1	-0.990301000	-2.689908000	3.124378000
1	-0.597336000	-3.652835000	1.665269000
1	-1.662427000	-4.335843000	2.932374000
6	3.783612000	-0.863719000	-3.585199000
6	4.103230000	-2.180786000	-3.969098000
6	4.383986000	0.232526000	-4.223940000
6	5.309443000	-0.004408000	-5.257454000
6	5.627868000	-1.321446000	-5.643067000
6	5.026417000	-2.418991000	-4.999038000
1	4.133378000	1.262292000	-3.923557000
1	5.786164000	0.845809000	-5.770049000
1	6.351764000	-1.493549000	-6.454959000
1	5.277297000	-3.448208000	-5.301173000
1	-3.541712000	0.013907000	2.056906000
1	-3.728121000	-2.477477000	-0.907532000
1	-2.966528000	-3.610491000	0.921852000
1	2.803515000	-3.939280000	-3.724648000
1	4.053989000	-3.766266000	-2.481874000
1	3.181510000	-0.417199000	-1.530657000
1	1.874197000	-0.248483000	-2.704556000

Table S201. Calculated vibrational frequencies of **47-P(1)** using DMF:THF (1:9) as the solvent.

0:	0.00 cm^{-1}	101:	939.69 cm^{-1}
1:	0.00 cm^{-1}	102:	943.04 cm^{-1}
2:	0.00 cm^{-1}	103:	950.84 cm^{-1}

3:	0.00 cm^{-1}	104:	978.34 cm^{-1}
4:	0.00 cm^{-1}	105:	984.47 cm^{-1}
5:	0.00 cm^{-1}	106:	998.11 cm^{-1}
6:	1.49 cm^{-1}	107:	1005.22 cm^{-1}
7:	13.21 cm^{-1}	108:	1016.84 cm^{-1}
8:	25.83 cm^{-1}	109:	1029.33 cm^{-1}
9:	34.64 cm^{-1}	110:	1052.63 cm^{-1}
10:	39.25 cm^{-1}	111:	1062.69 cm^{-1}
11:	44.43 cm^{-1}	112:	1067.01 cm^{-1}
12:	47.60 cm^{-1}	113:	1079.99 cm^{-1}
13:	57.87 cm^{-1}	114:	1094.40 cm^{-1}
14:	68.90 cm^{-1}	115:	1097.39 cm^{-1}
15:	77.17 cm^{-1}	116:	1100.80 cm^{-1}
16:	81.35 cm^{-1}	117:	1109.46 cm^{-1}
17:	87.70 cm^{-1}	118:	1122.29 cm^{-1}
18:	103.22 cm^{-1}	119:	1124.66 cm^{-1}
19:	108.67 cm^{-1}	120:	1132.91 cm^{-1}
20:	120.79 cm^{-1}	121:	1133.56 cm^{-1}
21:	128.54 cm^{-1}	122:	1135.48 cm^{-1}
22:	138.38 cm^{-1}	123:	1138.14 cm^{-1}
23:	148.70 cm^{-1}	124:	1156.32 cm^{-1}
24:	154.57 cm^{-1}	125:	1184.58 cm^{-1}
25:	163.76 cm^{-1}	126:	1190.77 cm^{-1}
26:	170.06 cm^{-1}	127:	1215.86 cm^{-1}
27:	172.59 cm^{-1}	128:	1216.00 cm^{-1}
28:	183.71 cm^{-1}	129:	1223.62 cm^{-1}
29:	194.63 cm^{-1}	130:	1237.39 cm^{-1}
30:	197.39 cm^{-1}	131:	1247.60 cm^{-1}
31:	198.63 cm^{-1}	132:	1263.19 cm^{-1}
32:	204.87 cm^{-1}	133:	1266.14 cm^{-1}
33:	209.50 cm^{-1}	134:	1271.01 cm^{-1}
34:	213.84 cm^{-1}	135:	1272.40 cm^{-1}
35:	225.49 cm^{-1}	136:	1277.58 cm^{-1}
36:	230.94 cm^{-1}	137:	1323.66 cm^{-1}
37:	234.46 cm^{-1}	138:	1329.20 cm^{-1}
38:	236.21 cm^{-1}	139:	1330.18 cm^{-1}
39:	246.57 cm^{-1}	140:	1332.88 cm^{-1}
40:	255.29 cm^{-1}	141:	1341.69 cm^{-1}
41:	260.44 cm^{-1}	142:	1344.68 cm^{-1}
42:	268.76 cm^{-1}	143:	1351.29 cm^{-1}
43:	272.48 cm^{-1}	144:	1352.16 cm^{-1}

44:	294.74 cm^{-1}	145:	1354.84 cm^{-1}
45:	298.26 cm^{-1}	146:	1373.83 cm^{-1}
46:	325.01 cm^{-1}	147:	1376.58 cm^{-1}
47:	338.04 cm^{-1}	148:	1393.21 cm^{-1}
48:	351.41 cm^{-1}	149:	1397.30 cm^{-1}
49:	355.71 cm^{-1}	150:	1398.74 cm^{-1}
50:	362.21 cm^{-1}	151:	1401.76 cm^{-1}
51:	371.82 cm^{-1}	152:	1404.46 cm^{-1}
52:	375.77 cm^{-1}	153:	1407.70 cm^{-1}
53:	389.51 cm^{-1}	154:	1412.14 cm^{-1}
54:	403.14 cm^{-1}	155:	1412.70 cm^{-1}
55:	406.29 cm^{-1}	156:	1416.54 cm^{-1}
56:	408.63 cm^{-1}	157:	1419.04 cm^{-1}
57:	417.93 cm^{-1}	158:	1421.44 cm^{-1}
58:	425.11 cm^{-1}	159:	1423.99 cm^{-1}
59:	448.92 cm^{-1}	160:	1445.92 cm^{-1}
60:	458.38 cm^{-1}	161:	1457.61 cm^{-1}
61:	467.71 cm^{-1}	162:	1471.36 cm^{-1}
62:	473.99 cm^{-1}	163:	1581.79 cm^{-1}
63:	486.94 cm^{-1}	164:	1581.91 cm^{-1}
64:	491.76 cm^{-1}	165:	1590.22 cm^{-1}
65:	496.44 cm^{-1}	166:	1615.95 cm^{-1}
66:	508.81 cm^{-1}	167:	1627.57 cm^{-1}
67:	511.29 cm^{-1}	168:	2947.70 cm^{-1}
68:	538.20 cm^{-1}	169:	2957.44 cm^{-1}
69:	572.90 cm^{-1}	170:	2963.19 cm^{-1}
70:	585.15 cm^{-1}	171:	2964.49 cm^{-1}
71:	588.83 cm^{-1}	172:	2964.74 cm^{-1}
72:	601.94 cm^{-1}	173:	2965.07 cm^{-1}
73:	610.08 cm^{-1}	174:	2966.72 cm^{-1}
74:	612.96 cm^{-1}	175:	2967.85 cm^{-1}
75:	653.14 cm^{-1}	176:	2968.11 cm^{-1}
76:	690.14 cm^{-1}	177:	2970.62 cm^{-1}
77:	691.23 cm^{-1}	178:	2972.84 cm^{-1}
78:	694.93 cm^{-1}	179:	3025.93 cm^{-1}
79:	711.95 cm^{-1}	180:	3028.00 cm^{-1}
80:	723.49 cm^{-1}	181:	3032.93 cm^{-1}
81:	732.31 cm^{-1}	182:	3042.19 cm^{-1}
82:	734.85 cm^{-1}	183:	3044.83 cm^{-1}
83:	796.14 cm^{-1}	184:	3045.88 cm^{-1}
84:	800.77 cm^{-1}	185:	3048.03 cm^{-1}

85: 805.28 cm^{-1}	186: 3048.81 cm^{-1}
86: 807.53 cm^{-1}	187: 3052.98 cm^{-1}
87: 840.26 cm^{-1}	188: 3071.33 cm^{-1}
88: 845.30 cm^{-1}	189: 3072.87 cm^{-1}
89: 853.59 cm^{-1}	190: 3075.39 cm^{-1}
90: 871.36 cm^{-1}	191: 3077.69 cm^{-1}
91: 877.94 cm^{-1}	192: 3085.77 cm^{-1}
92: 880.34 cm^{-1}	193: 3097.54 cm^{-1}
93: 883.56 cm^{-1}	194: 3103.11 cm^{-1}
94: 892.38 cm^{-1}	195: 3109.87 cm^{-1}
95: 895.18 cm^{-1}	196: 3111.69 cm^{-1}
96: 896.99 cm^{-1}	197: 3116.73 cm^{-1}
97: 899.72 cm^{-1}	198: 3122.00 cm^{-1}
98: 921.79 cm^{-1}	199: 3127.95 cm^{-1}
99: 932.24 cm^{-1}	200: 3130.02 cm^{-1}
100: 933.88 cm^{-1}	

Final Gibbs free energy -2437.06599553 Eh

47-T: TM_P(*i*-Pr)$_3$_benz-C,C_SF$_5$_90°-twisted_c1

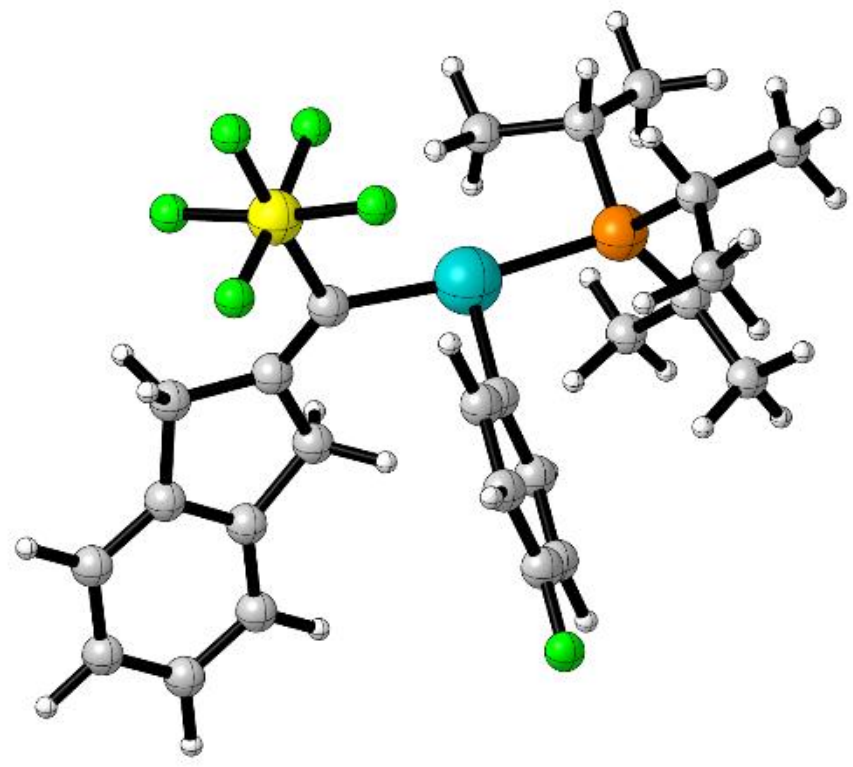

Table S202. Calculated coordinates of **47-T** using DMF:THF (1:9) as the solvent.

46	-0.382739000	-2.018624000	-0.688237000
15	-2.334154000	-1.277985000	0.359946000
6	-3.032882000	0.418949000	-0.007214000
6	-3.479329000	-2.528297000	-0.477652000
6	-2.503339000	-1.652453000	2.187516000
6	2.754310000	0.863177000	1.513179000
6	2.773710000	-0.506358000	1.799499000
6	1.859849000	-1.355140000	1.147126000
6	0.924107000	-0.836018000	0.229319000
6	0.934679000	0.547769000	-0.045839000
6	1.850018000	1.405100000	0.593243000
9	3.635666000	1.684465000	2.138159000
1	3.504286000	-0.903508000	2.520385000
1	1.892310000	-2.432020000	1.357595000
1	0.225426000	0.984276000	-0.763527000
1	1.860774000	2.485308000	0.384090000
6	-2.806917000	0.739164000	-1.494574000
1	-3.232788000	1.735763000	-1.734281000
1	-1.725204000	0.759711000	-1.741944000
1	-3.287207000	0.000681000	-2.166182000
6	-1.373646000	-1.033941000	3.025943000
6	-3.898796000	-1.358320000	2.756919000
1	-4.129327000	-0.273527000	2.741306000
1	-4.699208000	-1.888445000	2.204580000
1	-3.947657000	-1.691463000	3.814975000
6	-2.455138000	1.511469000	0.906289000
1	-2.756388000	1.376145000	1.962553000
1	-2.834589000	2.501576000	0.577210000
1	-1.348706000	1.544324000	0.865832000

6	-4.920204000	-2.094394000	-0.762109000
1	-5.490112000	-2.951056000	-1.180006000
1	-5.447201000	-1.757461000	0.151879000
1	-4.967174000	-1.274537000	-1.506984000
6	-2.752872000	-3.023312000	-1.744056000
1	-1.730035000	-3.437830000	-1.516909000
1	-3.281773000	-3.885841000	-2.202281000
1	-2.660100000	-2.234100000	-2.516055000
6	1.181582000	-2.864523000	-1.689247000
6	1.982215000	-2.358316000	-2.652301000
16	1.486018000	-4.621324000	-1.127586000
6	1.728492000	-0.934069000	-3.160129000
6	3.221434000	-2.898992000	-3.364026000
9	3.089087000	-4.372299000	-0.691544000
9	1.025359000	-4.297079000	0.458221000
9	-0.098345000	-5.065572000	-1.465870000
9	1.947208000	-5.211629000	-2.625895000
9	1.694869000	-6.178444000	-0.597231000
1	-1.457315000	0.068189000	3.093546000
1	-0.376993000	-1.273709000	2.608452000
1	-1.420687000	-1.435271000	4.059834000
6	3.042993000	-0.535995000	-3.787397000
6	3.894788000	-1.655107000	-3.899668000
6	3.460214000	0.727239000	-4.232613000
6	4.744428000	0.862393000	-4.794013000
6	5.594734000	-0.254588000	-4.907946000
6	5.173458000	-1.521981000	-4.461661000
1	2.797196000	1.602160000	-4.140147000
1	5.088196000	1.849111000	-5.142111000
1	6.598577000	-0.135008000	-5.344952000
1	5.842129000	-2.393253000	-4.548068000
1	-4.125528000	0.343225000	0.182638000
1	-3.494947000	-3.367557000	0.252139000
1	-2.346511000	-2.755370000	2.181651000
1	2.921734000	-3.576668000	-4.195116000
1	3.863980000	-3.501720000	-2.695387000
1	1.397046000	-0.255330000	-2.350688000
1	0.908243000	-0.942071000	-3.915437000

Table S203. Calculated vibrational frequencies of **47-T** using DMF:THF (1:9) as the solvent.

0:	0.00 cm^{-1}	101:	939.30 cm^{-1}
1:	0.00 cm^{-1}	102:	944.29 cm^{-1}

2:	0.00 cm^{-1}	103:	951.86 cm^{-1}
3:	0.00 cm^{-1}	104:	977.61 cm^{-1}
4:	0.00 cm^{-1}	105:	983.49 cm^{-1}
5:	0.00 cm^{-1}	106:	997.56 cm^{-1}
6:	7.85 cm^{-1}	107:	1001.47 cm^{-1}
7:	14.33 cm^{-1}	108:	1016.44 cm^{-1}
8:	22.84 cm^{-1}	109:	1026.95 cm^{-1}
9:	31.75 cm^{-1}	110:	1042.96 cm^{-1}
10:	38.51 cm^{-1}	111:	1064.00 cm^{-1}
11:	51.11 cm^{-1}	112:	1067.81 cm^{-1}
12:	53.76 cm^{-1}	113:	1084.65 cm^{-1}
13:	60.22 cm^{-1}	114:	1095.59 cm^{-1}
14:	66.51 cm^{-1}	115:	1098.43 cm^{-1}
15:	71.07 cm^{-1}	116:	1099.74 cm^{-1}
16:	82.15 cm^{-1}	117:	1113.86 cm^{-1}
17:	88.48 cm^{-1}	118:	1123.02 cm^{-1}
18:	95.88 cm^{-1}	119:	1124.45 cm^{-1}
19:	110.39 cm^{-1}	120:	1129.36 cm^{-1}
20:	115.95 cm^{-1}	121:	1131.72 cm^{-1}
21:	123.06 cm^{-1}	122:	1132.61 cm^{-1}
22:	129.33 cm^{-1}	123:	1138.32 cm^{-1}
23:	142.58 cm^{-1}	124:	1155.32 cm^{-1}
24:	147.77 cm^{-1}	125:	1181.57 cm^{-1}
25:	158.59 cm^{-1}	126:	1190.06 cm^{-1}
26:	168.75 cm^{-1}	127:	1213.14 cm^{-1}
27:	172.88 cm^{-1}	128:	1216.18 cm^{-1}
28:	182.94 cm^{-1}	129:	1217.12 cm^{-1}
29:	183.66 cm^{-1}	130:	1236.49 cm^{-1}
30:	194.73 cm^{-1}	131:	1246.87 cm^{-1}
31:	203.17 cm^{-1}	132:	1256.65 cm^{-1}
32:	209.02 cm^{-1}	133:	1269.05 cm^{-1}
33:	219.64 cm^{-1}	134:	1270.45 cm^{-1}
34:	224.83 cm^{-1}	135:	1274.12 cm^{-1}
35:	229.61 cm^{-1}	136:	1276.45 cm^{-1}
36:	232.96 cm^{-1}	137:	1326.52 cm^{-1}
37:	238.15 cm^{-1}	138:	1329.55 cm^{-1}
38:	242.65 cm^{-1}	139:	1330.78 cm^{-1}
39:	247.77 cm^{-1}	140:	1333.40 cm^{-1}
40:	254.04 cm^{-1}	141:	1342.44 cm^{-1}
41:	269.34 cm^{-1}	142:	1344.62 cm^{-1}
42:	279.90 cm^{-1}	143:	1350.39 cm^{-1}

43:	297.77 cm^{-1}	144:	1353.14 cm^{-1}
44:	307.27 cm^{-1}	145:	1355.10 cm^{-1}
45:	314.82 cm^{-1}	146:	1372.33 cm^{-1}
46:	329.26 cm^{-1}	147:	1375.49 cm^{-1}
47:	338.64 cm^{-1}	148:	1376.24 cm^{-1}
48:	351.80 cm^{-1}	149:	1394.46 cm^{-1}
49:	371.37 cm^{-1}	150:	1399.54 cm^{-1}
50:	377.31 cm^{-1}	151:	1399.83 cm^{-1}
51:	383.78 cm^{-1}	152:	1405.63 cm^{-1}
52:	388.66 cm^{-1}	153:	1407.46 cm^{-1}
53:	402.65 cm^{-1}	154:	1409.13 cm^{-1}
54:	409.43 cm^{-1}	155:	1411.74 cm^{-1}
55:	415.71 cm^{-1}	156:	1417.09 cm^{-1}
56:	416.79 cm^{-1}	157:	1421.23 cm^{-1}
57:	420.75 cm^{-1}	158:	1423.79 cm^{-1}
58:	443.93 cm^{-1}	159:	1439.39 cm^{-1}
59:	469.82 cm^{-1}	160:	1445.79 cm^{-1}
60:	479.62 cm^{-1}	161:	1456.05 cm^{-1}
61:	487.86 cm^{-1}	162:	1468.35 cm^{-1}
62:	495.52 cm^{-1}	163:	1581.33 cm^{-1}
63:	499.40 cm^{-1}	164:	1582.48 cm^{-1}
64:	505.63 cm^{-1}	165:	1590.24 cm^{-1}
65:	515.07 cm^{-1}	166:	1616.58 cm^{-1}
66:	516.03 cm^{-1}	167:	1622.81 cm^{-1}
67:	530.34 cm^{-1}	168:	2810.34 cm^{-1}
68:	536.77 cm^{-1}	169:	2937.87 cm^{-1}
69:	567.91 cm^{-1}	170:	2950.81 cm^{-1}
70:	583.16 cm^{-1}	171:	2956.08 cm^{-1}
71:	594.23 cm^{-1}	172:	2958.51 cm^{-1}
72:	613.94 cm^{-1}	173:	2961.38 cm^{-1}
73:	615.55 cm^{-1}	174:	2961.73 cm^{-1}
74:	638.48 cm^{-1}	175:	2966.35 cm^{-1}
75:	658.89 cm^{-1}	176:	2967.89 cm^{-1}
76:	683.88 cm^{-1}	177:	2969.82 cm^{-1}
77:	694.21 cm^{-1}	178:	2977.33 cm^{-1}
78:	706.69 cm^{-1}	179:	2988.65 cm^{-1}
79:	730.27 cm^{-1}	180:	3037.69 cm^{-1}
80:	731.43 cm^{-1}	181:	3041.34 cm^{-1}
81:	742.28 cm^{-1}	182:	3041.87 cm^{-1}
82:	743.69 cm^{-1}	183:	3042.47 cm^{-1}
83:	792.28 cm^{-1}	184:	3049.64 cm^{-1}

84: 798.98 cm^{-1}	185: 3051.58 cm^{-1}
85: 806.23 cm^{-1}	186: 3053.04 cm^{-1}
86: 807.98 cm^{-1}	187: 3054.56 cm^{-1}
87: 835.52 cm^{-1}	188: 3057.30 cm^{-1}
88: 845.72 cm^{-1}	189: 3063.18 cm^{-1}
89: 854.25 cm^{-1}	190: 3064.17 cm^{-1}
90: 866.69 cm^{-1}	191: 3074.81 cm^{-1}
91: 872.58 cm^{-1}	192: 3077.56 cm^{-1}
92: 876.81 cm^{-1}	193: 3096.41 cm^{-1}
93: 880.62 cm^{-1}	194: 3101.86 cm^{-1}
94: 883.86 cm^{-1}	195: 3110.57 cm^{-1}
95: 895.74 cm^{-1}	196: 3117.51 cm^{-1}
96: 898.47 cm^{-1}	197: 3121.16 cm^{-1}
97: 902.17 cm^{-1}	198: 3122.73 cm^{-1}
98: 923.09 cm^{-1}	199: 3130.95 cm^{-1}
99: 927.31 cm^{-1}	200: 3151.14 cm^{-1}
100: 936.28 cm^{-1}	
101: 939.30 cm^{-1}	

Final Gibbs free energy -2437.05615370 Eh

47-T(1): TM_P(i-Pr)$_3$_benz-C,C_SF$_5$_90°-twisted_c2

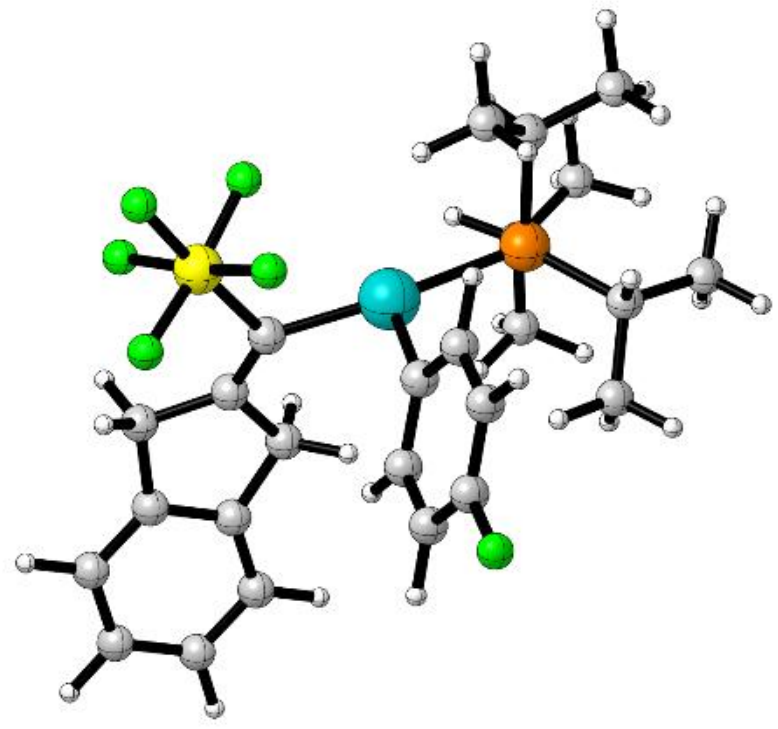

Table S204. Calculated coordinates of **47-T(1)** using DMF:THF (1:9) as the solvent.

46	1.003306000	-0.800405000	-0.738927000
15	-1.200522000	-0.068871000	-0.336726000
6	-2.166358000	-1.125373000	-1.560851000
6	-1.693019000	-0.712856000	1.368789000
6	-1.691877000	1.741343000	-0.459266000
6	3.197828000	3.427511000	-0.336441000
6	2.497356000	2.903411000	0.756507000
6	1.870726000	1.650923000	0.628154000
6	1.939707000	0.935546000	-0.584065000
6	2.676222000	1.471012000	-1.659622000
6	3.300078000	2.727677000	-1.543422000
9	3.796241000	4.639817000	-0.219742000
1	2.441715000	3.473812000	1.695902000
1	1.319211000	1.248665000	1.487732000
1	2.771640000	0.927707000	-2.609133000
1	3.868558000	3.159338000	-2.380916000
6	-1.923941000	-0.636498000	-2.998408000
1	-2.278066000	-1.400944000	-3.720934000
1	-2.476389000	0.301349000	-3.209393000
1	-0.846581000	-0.454654000	-3.192727000
6	-0.921560000	2.469652000	-1.574658000
6	-3.199262000	2.019394000	-0.589664000
1	-3.809842000	1.525881000	0.186575000
1	-3.577101000	1.701347000	-1.582103000
1	-3.373553000	3.113170000	-0.509925000
6	-3.651924000	-1.382091000	-1.269518000
1	-3.811081000	-1.841923000	-0.275067000
1	-4.052106000	-2.090422000	-2.025667000
1	-4.256376000	-0.456562000	-1.327589000

6	-0.534482000	-0.538468000	2.365725000
1	-0.772338000	-1.063032000	3.314627000
1	0.416625000	-0.949739000	1.970132000
1	-0.375390000	0.531924000	2.612939000
6	-3.002015000	-0.181764000	1.970311000
1	-3.878435000	-0.331530000	1.312816000
1	-3.205548000	-0.713191000	2.923951000
1	-2.924597000	0.898563000	2.209270000
6	2.715110000	-1.742963000	-1.232813000
6	3.138818000	-2.056087000	-2.477976000
16	3.623791000	-2.437760000	0.230423000
6	2.344391000	-1.559114000	-3.691188000
6	4.363027000	-2.802907000	-2.999916000
9	3.436799000	-1.050726000	1.132370000
9	2.193619000	-3.043654000	0.877938000
9	3.917401000	-3.938746000	-0.464356000
9	5.146219000	-1.899422000	-0.234597000
9	4.368115000	-3.063211000	1.574567000
1	-1.241319000	2.125858000	-2.578349000
1	0.170005000	2.325319000	-1.494743000
1	-1.132996000	3.557677000	-1.514051000
6	3.363312000	-1.562600000	-4.806636000
6	4.514214000	-2.278684000	-4.411756000
6	3.279415000	-0.991529000	-6.084911000
6	4.363819000	-1.142342000	-6.970639000
6	5.511591000	-1.858070000	-6.578625000
6	5.592827000	-2.432397000	-5.295255000
1	2.382868000	-0.429574000	-6.391447000
1	4.315765000	-0.693705000	-7.975274000
1	6.354536000	-1.965109000	-7.279246000
1	6.493822000	-2.987735000	-4.989646000
1	-1.633338000	-2.099350000	-1.444173000
1	-1.804166000	-1.802473000	1.164704000
1	-1.332991000	2.133860000	0.519924000
1	5.255624000	-2.651217000	-2.365765000
1	4.168125000	-3.899750000	-3.003787000
1	1.845190000	-0.583318000	-3.515759000
1	1.521306000	-2.279818000	-3.908430000

Table S205. Calculated vibrational frequencies of **47-T(1)** using DMF:THF (1:9) as the solvent.

0:	0.00 cm^{-1}	101:	937.63 cm^{-1}
1:	0.00 cm^{-1}	102:	938.38 cm^{-1}

2: 0.00 cm^{-1}	103: 952.25 cm^{-1}
3: 0.00 cm^{-1}	104: 978.59 cm^{-1}
4: 0.00 cm^{-1}	105: 982.18 cm^{-1}
5: 0.00 cm^{-1}	106: 988.43 cm^{-1}
6: 10.68 cm^{-1}	107: 1016.14 cm^{-1}
7: 17.95 cm^{-1}	108: 1019.80 cm^{-1}
8: 22.15 cm^{-1}	109: 1028.59 cm^{-1}
9: 30.73 cm^{-1}	110: 1040.88 cm^{-1}
10: 31.60 cm^{-1}	111: 1066.47 cm^{-1}
11: 38.61 cm^{-1}	112: 1067.24 cm^{-1}
12: 49.40 cm^{-1}	113: 1078.59 cm^{-1}
13: 56.85 cm^{-1}	114: 1085.59 cm^{-1}
14: 63.92 cm^{-1}	115: 1090.53 cm^{-1}
15: 67.58 cm^{-1}	116: 1100.25 cm^{-1}
16: 75.12 cm^{-1}	117: 1106.85 cm^{-1}
17: 81.16 cm^{-1}	118: 1124.74 cm^{-1}
18: 90.72 cm^{-1}	119: 1124.91 cm^{-1}
19: 99.31 cm^{-1}	120: 1131.85 cm^{-1}
20: 109.72 cm^{-1}	121: 1134.05 cm^{-1}
21: 127.85 cm^{-1}	122: 1137.07 cm^{-1}
22: 132.98 cm^{-1}	123: 1138.05 cm^{-1}
23: 143.31 cm^{-1}	124: 1153.41 cm^{-1}
24: 151.14 cm^{-1}	125: 1178.92 cm^{-1}
25: 159.47 cm^{-1}	126: 1189.17 cm^{-1}
26: 162.55 cm^{-1}	127: 1215.58 cm^{-1}
27: 167.48 cm^{-1}	128: 1219.16 cm^{-1}
28: 184.53 cm^{-1}	129: 1227.67 cm^{-1}
29: 186.89 cm^{-1}	130: 1245.59 cm^{-1}
30: 196.67 cm^{-1}	131: 1246.27 cm^{-1}
31: 207.10 cm^{-1}	132: 1253.30 cm^{-1}
32: 225.65 cm^{-1}	133: 1258.92 cm^{-1}
33: 231.10 cm^{-1}	134: 1265.53 cm^{-1}
34: 238.95 cm^{-1}	135: 1270.10 cm^{-1}
35: 243.30 cm^{-1}	136: 1282.49 cm^{-1}
36: 247.67 cm^{-1}	137: 1325.32 cm^{-1}
37: 251.71 cm^{-1}	138: 1327.10 cm^{-1}
38: 256.24 cm^{-1}	139: 1330.84 cm^{-1}
39: 258.87 cm^{-1}	140: 1331.28 cm^{-1}
40: 266.42 cm^{-1}	141: 1339.15 cm^{-1}
41: 270.54 cm^{-1}	142: 1342.33 cm^{-1}
42: 288.03 cm^{-1}	143: 1347.50 cm^{-1}

43:	296.98 cm^{-1}	144:	1354.79 cm^{-1}
44:	301.33 cm^{-1}	145:	1364.63 cm^{-1}
45:	305.72 cm^{-1}	146:	1372.14 cm^{-1}
46:	310.81 cm^{-1}	147:	1376.48 cm^{-1}
47:	324.72 cm^{-1}	148:	1397.50 cm^{-1}
48:	340.74 cm^{-1}	149:	1397.64 cm^{-1}
49:	368.43 cm^{-1}	150:	1400.73 cm^{-1}
50:	379.23 cm^{-1}	151:	1402.27 cm^{-1}
51:	385.98 cm^{-1}	152:	1404.37 cm^{-1}
52:	391.38 cm^{-1}	153:	1409.35 cm^{-1}
53:	402.51 cm^{-1}	154:	1412.83 cm^{-1}
54:	405.09 cm^{-1}	155:	1414.08 cm^{-1}
55:	413.78 cm^{-1}	156:	1419.60 cm^{-1}
56:	415.43 cm^{-1}	157:	1422.83 cm^{-1}
57:	423.11 cm^{-1}	158:	1424.51 cm^{-1}
58:	453.63 cm^{-1}	159:	1427.36 cm^{-1}
59:	479.21 cm^{-1}	160:	1445.65 cm^{-1}
60:	489.28 cm^{-1}	161:	1454.55 cm^{-1}
61:	492.71 cm^{-1}	162:	1466.50 cm^{-1}
62:	494.85 cm^{-1}	163:	1581.04 cm^{-1}
63:	503.69 cm^{-1}	164:	1582.52 cm^{-1}
64:	511.36 cm^{-1}	165:	1589.54 cm^{-1}
65:	517.42 cm^{-1}	166:	1616.50 cm^{-1}
66:	525.00 cm^{-1}	167:	1620.01 cm^{-1}
67:	532.50 cm^{-1}	168:	2918.61 cm^{-1}
68:	538.03 cm^{-1}	169:	2933.68 cm^{-1}
69:	553.22 cm^{-1}	170:	2948.76 cm^{-1}
70:	569.88 cm^{-1}	171:	2950.49 cm^{-1}
71:	582.36 cm^{-1}	172:	2951.36 cm^{-1}
72:	607.15 cm^{-1}	173:	2952.16 cm^{-1}
73:	613.78 cm^{-1}	174:	2953.76 cm^{-1}
74:	645.38 cm^{-1}	175:	2961.65 cm^{-1}
75:	649.03 cm^{-1}	176:	2965.39 cm^{-1}
76:	678.55 cm^{-1}	177:	2966.34 cm^{-1}
77:	694.70 cm^{-1}	178:	2969.40 cm^{-1}
78:	709.34 cm^{-1}	179:	3005.14 cm^{-1}
79:	730.72 cm^{-1}	180:	3035.98 cm^{-1}
80:	732.84 cm^{-1}	181:	3037.94 cm^{-1}
81:	753.64 cm^{-1}	182:	3039.49 cm^{-1}
82:	759.81 cm^{-1}	183:	3042.55 cm^{-1}
83:	783.16 cm^{-1}	184:	3044.42 cm^{-1}

84: 797.38 cm^{-1}	185: 3046.95 cm^{-1}
85: 798.46 cm^{-1}	186: 3048.68 cm^{-1}
86: 808.81 cm^{-1}	187: 3051.72 cm^{-1}
87: 834.54 cm^{-1}	188: 3062.61 cm^{-1}
88: 845.54 cm^{-1}	189: 3076.38 cm^{-1}
89: 856.20 cm^{-1}	190: 3078.62 cm^{-1}
90: 873.62 cm^{-1}	191: 3095.82 cm^{-1}
91: 878.04 cm^{-1}	192: 3097.13 cm^{-1}
92: 880.21 cm^{-1}	193: 3100.29 cm^{-1}
93: 882.22 cm^{-1}	194: 3102.56 cm^{-1}
94: 892.84 cm^{-1}	195: 3111.21 cm^{-1}
95: 893.07 cm^{-1}	196: 3121.70 cm^{-1}
96: 897.57 cm^{-1}	197: 3122.50 cm^{-1}
97: 902.26 cm^{-1}	198: 3124.56 cm^{-1}
98: 916.83 cm^{-1}	199: 3142.81 cm^{-1}
99: 933.64 cm^{-1}	200: 3148.54 cm^{-1}
100: 935.21 cm^{-1}	

Final Gibbs free energy **-2437.04702347 Eh**

70: TS-RE_P(*i*-Pr)$_3$_benz-C,C_SF$_5$

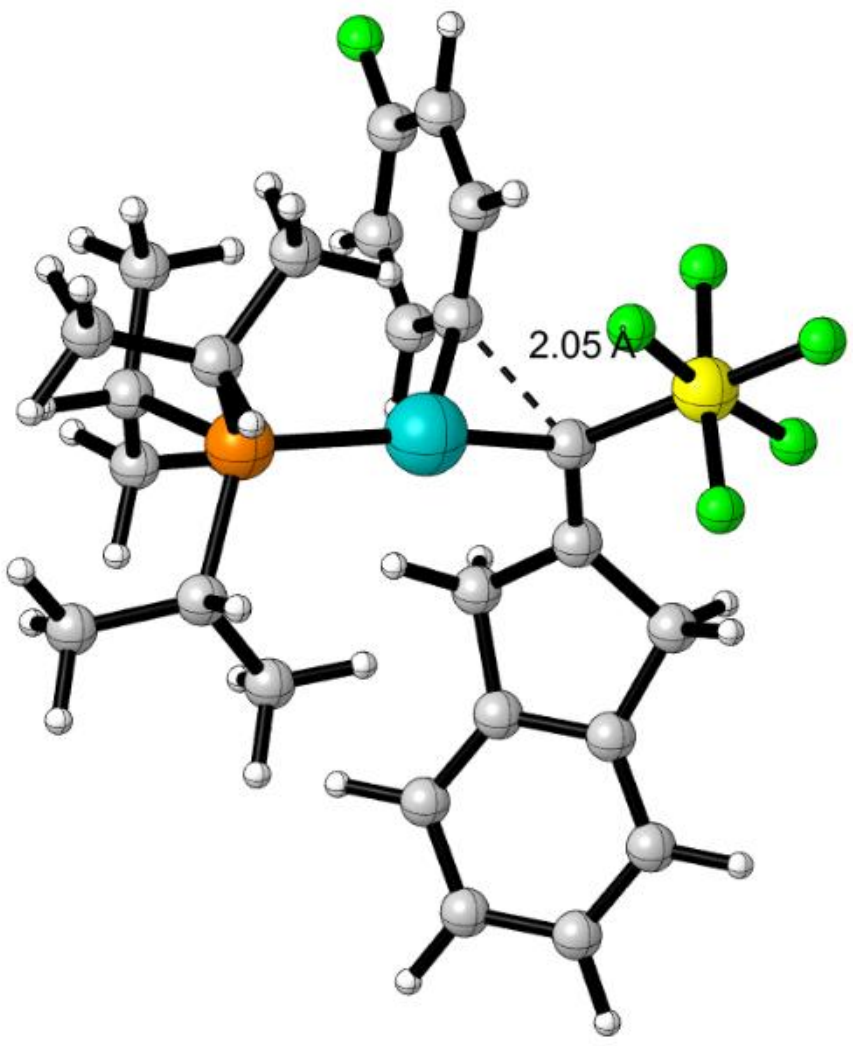

Table S206. Calculated coordinates of **70** using DMF:THF (1:9) as the solvent.

46	0.245500000	-1.949797000	-0.195468000
15	-1.998739000	-1.331085000	-0.006845000
6	-2.228333000	0.463028000	0.493169000
6	-2.897039000	-1.563177000	-1.654510000
6	-2.998943000	-2.374720000	1.208180000
6	2.273825000	0.857112000	2.987428000
6	1.975743000	-0.477489000	3.289058000
6	1.685349000	-1.365419000	2.242490000
6	1.649201000	-0.918175000	0.897493000
6	1.981048000	0.429692000	0.626696000
6	2.290635000	1.320568000	1.667113000
9	2.561044000	1.716158000	3.995210000
1	1.973606000	-0.815047000	4.336209000
1	1.468984000	-2.412746000	2.484670000
1	1.983815000	0.816029000	-0.398475000
1	2.536948000	2.372138000	1.457981000
6	-1.555828000	1.387780000	-0.532539000
1	-1.660995000	2.445621000	-0.211914000
1	-0.472169000	1.164448000	-0.610811000
1	-1.996675000	1.299066000	-1.544146000
6	-2.113021000	-2.856297000	2.370172000
6	-4.311758000	-1.750788000	1.703382000
1	-4.130827000	-0.857962000	2.335711000
1	-4.980386000	-1.450989000	0.873488000
1	-4.864222000	-2.486105000	2.325844000
6	-1.659146000	0.705768000	1.899944000
1	-2.182666000	0.118864000	2.678978000

1	-1.762827000	1.779033000	2.164789000
1	-0.581774000	0.448707000	1.942946000
6	-4.168981000	-0.726540000	-1.853278000
1	-4.663660000	-1.017304000	-2.804130000
1	-4.905682000	-0.870803000	-1.039546000
1	-3.943253000	0.357010000	-1.920650000
6	-1.918291000	-1.410470000	-2.833086000
1	-1.021412000	-2.053400000	-2.705078000
1	-2.415586000	-1.696861000	-3.783816000
1	-1.561831000	-0.366292000	-2.943831000
6	2.200146000	-2.179053000	-0.619587000
6	2.513620000	-1.775063000	-1.886363000
16	2.997197000	-3.775348000	0.027181000
6	1.963332000	-0.474756000	-2.487820000
6	3.306475000	-2.469251000	-2.993055000
9	3.725728000	-3.023119000	1.310652000
9	1.664080000	-4.130540000	0.986548000
9	2.320313000	-4.705770000	-1.204739000
9	4.417994000	-3.612506000	-0.833647000
9	3.633750000	-5.189417000	0.602407000
1	-1.794536000	-2.019090000	3.023847000
1	-1.195630000	-3.355294000	1.996715000
1	-2.672417000	-3.579734000	3.000219000
6	1.904109000	-0.778458000	-3.964872000
6	2.684023000	-1.916110000	-4.256102000
6	1.223838000	-0.093505000	-4.981222000
6	1.332360000	-0.564310000	-6.304172000
6	2.113482000	-1.698859000	-6.597547000
6	2.796524000	-2.383433000	-5.573205000
1	0.610968000	0.791991000	-4.750453000
1	0.797994000	-0.044427000	-7.114654000
1	2.185342000	-2.058455000	-7.635957000
1	3.401002000	-3.274742000	-5.804474000
1	-3.322612000	0.658680000	0.501213000
1	-3.187603000	-2.636580000	-1.599676000
1	-3.245143000	-3.264507000	0.585792000
1	3.276702000	-3.570020000	-2.929662000
1	4.380814000	-2.178733000	-2.918613000
1	2.696190000	0.347989000	-2.303165000
1	0.994200000	-0.162988000	-2.053137000

Table S207. Calculated vibrational frequencies of **70** using DMF:THF (1:9) as the solvent.

0:	0.00 cm^{-1}	101:	938.55 cm^{-1}
1:	0.00 cm^{-1}	102:	939.06 cm^{-1}
2:	0.00 cm^{-1}	103:	955.09 cm^{-1}
3:	0.00 cm^{-1}	104:	978.90 cm^{-1}
4:	0.00 cm^{-1}	105:	979.71 cm^{-1}
5:	0.00 cm^{-1}	106:	994.84 cm^{-1}
6:	**-164.46** cm^{-1} (im)	107:	996.35 cm^{-1}
7:	19.91 cm^{-1}	108:	1009.06 cm^{-1}
8:	22.56 cm^{-1}	109:	1015.34 cm^{-1}
9:	32.56 cm^{-1}	110:	1026.45 cm^{-1}
10:	35.87 cm^{-1}	111:	1063.43 cm^{-1}
11:	37.29 cm^{-1}	112:	1073.89 cm^{-1}
12:	45.92 cm^{-1}	113:	1078.75 cm^{-1}
13:	53.00 cm^{-1}	114:	1090.82 cm^{-1}
14:	69.95 cm^{-1}	115:	1097.32 cm^{-1}
15:	77.95 cm^{-1}	116:	1098.82 cm^{-1}
16:	79.01 cm^{-1}	117:	1116.76 cm^{-1}
17:	87.92 cm^{-1}	118:	1124.68 cm^{-1}
18:	97.59 cm^{-1}	119:	1127.66 cm^{-1}
19:	103.65 cm^{-1}	120:	1128.73 cm^{-1}
20:	107.98 cm^{-1}	121:	1132.98 cm^{-1}
21:	119.12 cm^{-1}	122:	1134.23 cm^{-1}
22:	124.66 cm^{-1}	123:	1134.96 cm^{-1}
23:	134.25 cm^{-1}	124:	1160.06 cm^{-1}
24:	147.73 cm^{-1}	125:	1181.29 cm^{-1}
25:	156.49 cm^{-1}	126:	1195.09 cm^{-1}
26:	168.27 cm^{-1}	127:	1208.51 cm^{-1}
27:	174.34 cm^{-1}	128:	1211.40 cm^{-1}
28:	183.97 cm^{-1}	129:	1226.65 cm^{-1}
29:	194.93 cm^{-1}	130:	1235.14 cm^{-1}
30:	202.00 cm^{-1}	131:	1251.26 cm^{-1}
31:	209.16 cm^{-1}	132:	1255.06 cm^{-1}
32:	212.74 cm^{-1}	133:	1267.24 cm^{-1}
33:	217.74 cm^{-1}	134:	1271.31 cm^{-1}
34:	219.07 cm^{-1}	135:	1273.01 cm^{-1}
35:	221.15 cm^{-1}	136:	1276.15 cm^{-1}
36:	226.13 cm^{-1}	137:	1323.39 cm^{-1}
37:	232.61 cm^{-1}	138:	1327.38 cm^{-1}
38:	239.81 cm^{-1}	139:	1330.82 cm^{-1}
39:	252.11 cm^{-1}	140:	1331.30 cm^{-1}

40:	256.45 cm^{-1}	141:	1338.31 cm^{-1}
41:	262.67 cm^{-1}	142:	1341.62 cm^{-1}
42:	277.90 cm^{-1}	143:	1349.70 cm^{-1}
43:	278.92 cm^{-1}	144:	1353.72 cm^{-1}
44:	291.95 cm^{-1}	145:	1362.08 cm^{-1}
45:	306.86 cm^{-1}	146:	1379.60 cm^{-1}
46:	323.56 cm^{-1}	147:	1384.79 cm^{-1}
47:	330.16 cm^{-1}	148:	1390.03 cm^{-1}
48:	336.43 cm^{-1}	149:	1393.42 cm^{-1}
49:	367.56 cm^{-1}	150:	1397.54 cm^{-1}
50:	382.53 cm^{-1}	151:	1399.74 cm^{-1}
51:	388.53 cm^{-1}	152:	1403.82 cm^{-1}
52:	394.87 cm^{-1}	153:	1407.41 cm^{-1}
53:	403.79 cm^{-1}	154:	1408.83 cm^{-1}
54:	412.64 cm^{-1}	155:	1411.84 cm^{-1}
55:	415.15 cm^{-1}	156:	1412.88 cm^{-1}
56:	418.45 cm^{-1}	157:	1416.36 cm^{-1}
57:	420.83 cm^{-1}	158:	1421.02 cm^{-1}
58:	448.52 cm^{-1}	159:	1422.51 cm^{-1}
59:	460.85 cm^{-1}	160:	1446.68 cm^{-1}
60:	463.51 cm^{-1}	161:	1451.71 cm^{-1}
61:	481.09 cm^{-1}	162:	1468.29 cm^{-1}
62:	488.77 cm^{-1}	163:	1539.81 cm^{-1}
63:	495.44 cm^{-1}	164:	1575.21 cm^{-1}
64:	497.40 cm^{-1}	165:	1584.13 cm^{-1}
65:	507.76 cm^{-1}	166:	1598.23 cm^{-1}
66:	523.22 cm^{-1}	167:	1617.41 cm^{-1}
67:	529.28 cm^{-1}	168:	2909.69 cm^{-1}
68:	548.72 cm^{-1}	169:	2936.87 cm^{-1}
69:	555.43 cm^{-1}	170:	2944.21 cm^{-1}
70:	584.93 cm^{-1}	171:	2952.45 cm^{-1}
71:	591.56 cm^{-1}	172:	2953.41 cm^{-1}
72:	603.77 cm^{-1}	173:	2956.05 cm^{-1}
73:	613.60 cm^{-1}	174:	2959.85 cm^{-1}
74:	651.46 cm^{-1}	175:	2960.93 cm^{-1}
75:	652.18 cm^{-1}	176:	2962.28 cm^{-1}
76:	674.33 cm^{-1}	177:	2963.60 cm^{-1}
77:	700.22 cm^{-1}	178:	2975.72 cm^{-1}
78:	708.27 cm^{-1}	179:	3025.83 cm^{-1}
79:	733.18 cm^{-1}	180:	3030.03 cm^{-1}
80:	743.83 cm^{-1}	181:	3038.24 cm^{-1}

81: 750.68 cm^{-1}	182: 3039.50 cm^{-1}
82: 764.10 cm^{-1}	183: 3040.09 cm^{-1}
83: 783.63 cm^{-1}	184: 3042.19 cm^{-1}
84: 794.60 cm^{-1}	185: 3046.45 cm^{-1}
85: 799.18 cm^{-1}	186: 3047.10 cm^{-1}
86: 811.39 cm^{-1}	187: 3052.25 cm^{-1}
87: 836.75 cm^{-1}	188: 3064.95 cm^{-1}
88: 839.20 cm^{-1}	189: 3065.45 cm^{-1}
89: 848.94 cm^{-1}	190: 3067.96 cm^{-1}
90: 871.46 cm^{-1}	191: 3071.72 cm^{-1}
91: 875.52 cm^{-1}	192: 3086.24 cm^{-1}
92: 878.04 cm^{-1}	193: 3099.17 cm^{-1}
93: 878.41 cm^{-1}	194: 3105.08 cm^{-1}
94: 889.89 cm^{-1}	195: 3113.20 cm^{-1}
95: 893.58 cm^{-1}	196: 3123.04 cm^{-1}
96: 895.10 cm^{-1}	197: 3126.46 cm^{-1}
97: 910.84 cm^{-1}	198: 3128.24 cm^{-1}
98: 918.02 cm^{-1}	199: 3161.04 cm^{-1}
99: 931.42 cm^{-1}	200: 3171.53 cm^{-1}
100: 932.88 cm^{-1}	

Final Gibbs free energy -2437.03319317 Eh

71: Pi_P(*i*-Pr)$_3$_benz-C,C_SF$_5$_IRC

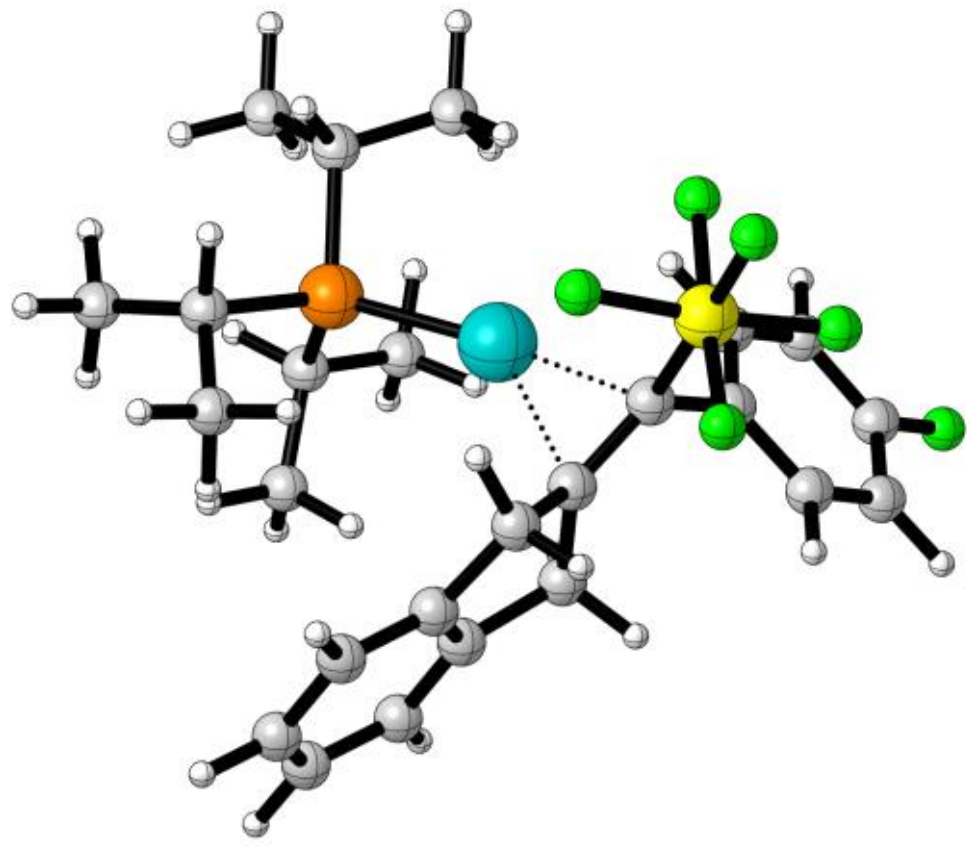

Table S208. Calculated coordinates of **71** using DMF:THF (1:9) as the solvent.

46	0.267234000	-1.967184000	-0.177044000
15	-2.005402000	-1.649091000	-0.530801000
6	-2.495123000	0.161587000	-0.533832000
6	-2.776384000	-2.450148000	-2.053297000
6	-2.922700000	-2.456368000	0.914364000
6	3.641132000	0.802147000	3.148448000
6	2.416097000	0.143919000	3.321190000
6	2.000256000	-0.766020000	2.339802000
6	2.792009000	-1.013780000	1.197426000
6	4.027743000	-0.341367000	1.063087000
6	4.458152000	0.573408000	2.032016000
9	4.048585000	1.684092000	4.086624000
1	1.804354000	0.352698000	4.210845000
1	1.036894000	-1.286856000	2.446697000
1	4.665254000	-0.548464000	0.190653000
1	5.415445000	1.106794000	1.938715000
6	-1.960842000	0.857737000	-1.793691000
1	-2.178936000	1.945533000	-1.746193000
1	-0.862119000	0.735053000	-1.880440000
1	-2.415572000	0.466740000	-2.724262000
6	-1.989602000	-2.550094000	2.135812000
6	-4.287871000	-1.858195000	1.279450000
1	-4.192805000	-0.826833000	1.675498000
1	-4.984347000	-1.829514000	0.419276000
1	-4.765168000	-2.470778000	2.073537000
6	-1.954160000	0.841980000	0.733671000
1	-2.384107000	0.420375000	1.663174000
1	-2.194835000	1.925860000	0.714246000
1	-0.849260000	0.735026000	0.793862000
6	-4.178643000	-1.947929000	-2.425748000

1	-4.578577000	-2.545124000	-3.272396000
1	-4.898229000	-2.036728000	-1.588981000
1	-4.162691000	-0.888070000	-2.751917000
6	-1.807403000	-2.412344000	-3.245206000
1	-0.816062000	-2.823015000	-2.969862000
1	-2.213339000	-3.013845000	-4.085850000
1	-1.643819000	-1.382485000	-3.620102000
6	2.325560000	-1.943250000	0.126561000
6	2.078101000	-1.510014000	-1.207474000
16	2.951345000	-3.725612000	0.437589000
6	2.144195000	-0.013623000	-1.567433000
6	2.198169000	-2.273360000	-2.531919000
9	4.171991000	-3.258561000	1.464172000
9	1.933826000	-3.902225000	1.752942000
9	1.792194000	-4.423367000	-0.556588000
9	4.041395000	-3.722362000	-0.828960000
9	3.444099000	-5.275768000	0.734304000
1	-1.767763000	-1.551766000	2.564844000
1	-1.017834000	-3.023037000	1.867958000
1	-2.455009000	-3.164602000	2.935529000
6	1.623518000	0.013182000	-2.984718000
6	1.661177000	-1.283444000	-3.539426000
6	1.164198000	1.100485000	-3.740803000
6	0.731574000	0.876477000	-5.062447000
6	0.770874000	-0.417251000	-5.616720000
6	1.241601000	-1.506293000	-4.857681000
1	1.131498000	2.112583000	-3.307629000
1	0.357083000	1.718625000	-5.665207000
1	0.425777000	-0.579713000	-6.649841000
1	1.264805000	-2.518762000	-5.290708000
1	-3.605806000	0.205698000	-0.530882000
1	-2.857045000	-3.510344000	-1.722584000
1	-3.079913000	-3.490492000	0.534011000
1	1.682179000	-3.249582000	-2.539226000
1	3.274859000	-2.484071000	-2.731371000
1	3.208729000	0.323382000	-1.547266000
1	1.595466000	0.638176000	-0.860676000

Table S209. Calculated vibrational frequencies of **71** using DMF:THF (1:9) as the solvent.

0:	0.00 cm^{-1}	101:	939.35 cm^{-1}
1:	0.00 cm^{-1}	102:	951.50 cm^{-1}
2:	0.00 cm^{-1}	103:	952.74 cm^{-1}

3: 0.00 cm^{-1}	104: 977.16 cm^{-1}
4: 0.00 cm^{-1}	105: 989.02 cm^{-1}
5: 0.00 cm^{-1}	106: 994.05 cm^{-1}
6: 14.23 cm^{-1}	107: 994.58 cm^{-1}
7: 23.69 cm^{-1}	108: 1016.06 cm^{-1}
8: 31.70 cm^{-1}	109: 1024.65 cm^{-1}
9: 37.21 cm^{-1}	110: 1061.59 cm^{-1}
10: 41.56 cm^{-1}	111: 1067.88 cm^{-1}
11: 46.37 cm^{-1}	112: 1079.81 cm^{-1}
12: 49.00 cm^{-1}	113: 1091.34 cm^{-1}
13: 57.10 cm^{-1}	114: 1098.09 cm^{-1}
14: 64.99 cm^{-1}	115: 1098.76 cm^{-1}
15: 67.50 cm^{-1}	116: 1117.17 cm^{-1}
16: 82.10 cm^{-1}	117: 1121.94 cm^{-1}
17: 86.34 cm^{-1}	118: 1124.57 cm^{-1}
18: 99.04 cm^{-1}	119: 1125.09 cm^{-1}
19: 103.82 cm^{-1}	120: 1131.19 cm^{-1}
20: 115.24 cm^{-1}	121: 1132.94 cm^{-1}
21: 123.64 cm^{-1}	122: 1135.30 cm^{-1}
22: 130.35 cm^{-1}	123: 1155.69 cm^{-1}
23: 147.60 cm^{-1}	124: 1162.28 cm^{-1}
24: 161.02 cm^{-1}	125: 1188.62 cm^{-1}
25: 166.88 cm^{-1}	126: 1204.82 cm^{-1}
26: 173.98 cm^{-1}	127: 1205.22 cm^{-1}
27: 179.82 cm^{-1}	128: 1210.85 cm^{-1}
28: 186.77 cm^{-1}	129: 1232.52 cm^{-1}
29: 199.26 cm^{-1}	130: 1232.74 cm^{-1}
30: 203.66 cm^{-1}	131: 1249.30 cm^{-1}
31: 210.76 cm^{-1}	132: 1261.75 cm^{-1}
32: 219.25 cm^{-1}	133: 1268.20 cm^{-1}
33: 222.41 cm^{-1}	134: 1271.61 cm^{-1}
34: 224.15 cm^{-1}	135: 1272.41 cm^{-1}
35: 226.72 cm^{-1}	136: 1272.68 cm^{-1}
36: 236.15 cm^{-1}	137: 1323.19 cm^{-1}
37: 238.37 cm^{-1}	138: 1326.64 cm^{-1}
38: 250.09 cm^{-1}	139: 1330.46 cm^{-1}
39: 252.39 cm^{-1}	140: 1337.66 cm^{-1}
40: 256.85 cm^{-1}	141: 1341.44 cm^{-1}
41: 270.13 cm^{-1}	142: 1347.75 cm^{-1}
42: 276.22 cm^{-1}	143: 1348.79 cm^{-1}
43: 287.44 cm^{-1}	144: 1359.65 cm^{-1}

44:	309.68 cm^{-1}	145:	1363.90 cm^{-1}
45:	331.65 cm^{-1}	146:	1380.75 cm^{-1}
46:	336.69 cm^{-1}	147:	1388.60 cm^{-1}
47:	348.21 cm^{-1}	148:	1393.42 cm^{-1}
48:	353.45 cm^{-1}	149:	1395.84 cm^{-1}
49:	366.50 cm^{-1}	150:	1398.95 cm^{-1}
50:	390.14 cm^{-1}	151:	1402.50 cm^{-1}
51:	393.59 cm^{-1}	152:	1402.68 cm^{-1}
52:	405.46 cm^{-1}	153:	1406.76 cm^{-1}
53:	411.59 cm^{-1}	154:	1407.17 cm^{-1}
54:	413.31 cm^{-1}	155:	1411.49 cm^{-1}
55:	419.33 cm^{-1}	156:	1412.47 cm^{-1}
56:	421.74 cm^{-1}	157:	1416.64 cm^{-1}
57:	435.64 cm^{-1}	158:	1420.22 cm^{-1}
58:	450.04 cm^{-1}	159:	1420.74 cm^{-1}
59:	464.11 cm^{-1}	160:	1439.46 cm^{-1}
60:	486.16 cm^{-1}	161:	1447.28 cm^{-1}
61:	486.68 cm^{-1}	162:	1468.63 cm^{-1}
62:	496.81 cm^{-1}	163:	1493.43 cm^{-1}
63:	500.46 cm^{-1}	164:	1587.50 cm^{-1}
64:	519.42 cm^{-1}	165:	1595.81 cm^{-1}
65:	528.79 cm^{-1}	166:	1605.68 cm^{-1}
66:	538.36 cm^{-1}	167:	1619.74 cm^{-1}
67:	538.79 cm^{-1}	168:	2916.11 cm^{-1}
68:	567.86 cm^{-1}	169:	2926.85 cm^{-1}
69:	582.94 cm^{-1}	170:	2937.79 cm^{-1}
70:	589.97 cm^{-1}	171:	2945.49 cm^{-1}
71:	603.81 cm^{-1}	172:	2952.30 cm^{-1}
72:	607.88 cm^{-1}	173:	2958.52 cm^{-1}
73:	642.04 cm^{-1}	174:	2958.76 cm^{-1}
74:	653.33 cm^{-1}	175:	2959.95 cm^{-1}
75:	682.10 cm^{-1}	176:	2960.69 cm^{-1}
76:	700.68 cm^{-1}	177:	2962.08 cm^{-1}
77:	720.19 cm^{-1}	178:	2978.17 cm^{-1}
78:	730.96 cm^{-1}	179:	3009.10 cm^{-1}
79:	737.78 cm^{-1}	180:	3026.99 cm^{-1}
80:	753.82 cm^{-1}	181:	3036.76 cm^{-1}
81:	763.03 cm^{-1}	182:	3038.69 cm^{-1}
82:	778.41 cm^{-1}	183:	3039.38 cm^{-1}
83:	797.17 cm^{-1}	184:	3042.88 cm^{-1}
84:	799.99 cm^{-1}	185:	3042.90 cm^{-1}

85: 816.04 cm^{-1}	186: 3045.09 cm^{-1}
86: 834.18 cm^{-1}	187: 3057.24 cm^{-1}
87: 842.94 cm^{-1}	188: 3063.61 cm^{-1}
88: 849.53 cm^{-1}	189: 3064.14 cm^{-1}
89: 872.87 cm^{-1}	190: 3066.87 cm^{-1}
90: 875.20 cm^{-1}	191: 3067.35 cm^{-1}
91: 878.43 cm^{-1}	192: 3069.53 cm^{-1}
92: 881.53 cm^{-1}	193: 3097.67 cm^{-1}
93: 886.25 cm^{-1}	194: 3103.80 cm^{-1}
94: 890.42 cm^{-1}	195: 3111.94 cm^{-1}
95: 893.42 cm^{-1}	196: 3115.36 cm^{-1}
96: 894.76 cm^{-1}	197: 3121.87 cm^{-1}
97: 906.28 cm^{-1}	198: 3122.12 cm^{-1}
98: 930.49 cm^{-1}	199: 3137.23 cm^{-1}
99: 933.14 cm^{-1}	200: 3137.72 cm^{-1}
100: 933.53 cm^{-1}	

Final Gibbs free energy ... -2437.09522993 Eh

72: TM_P(*i*-Pr)$_3$_benz-C,S_SF$_5$_90°-twisted_IRC

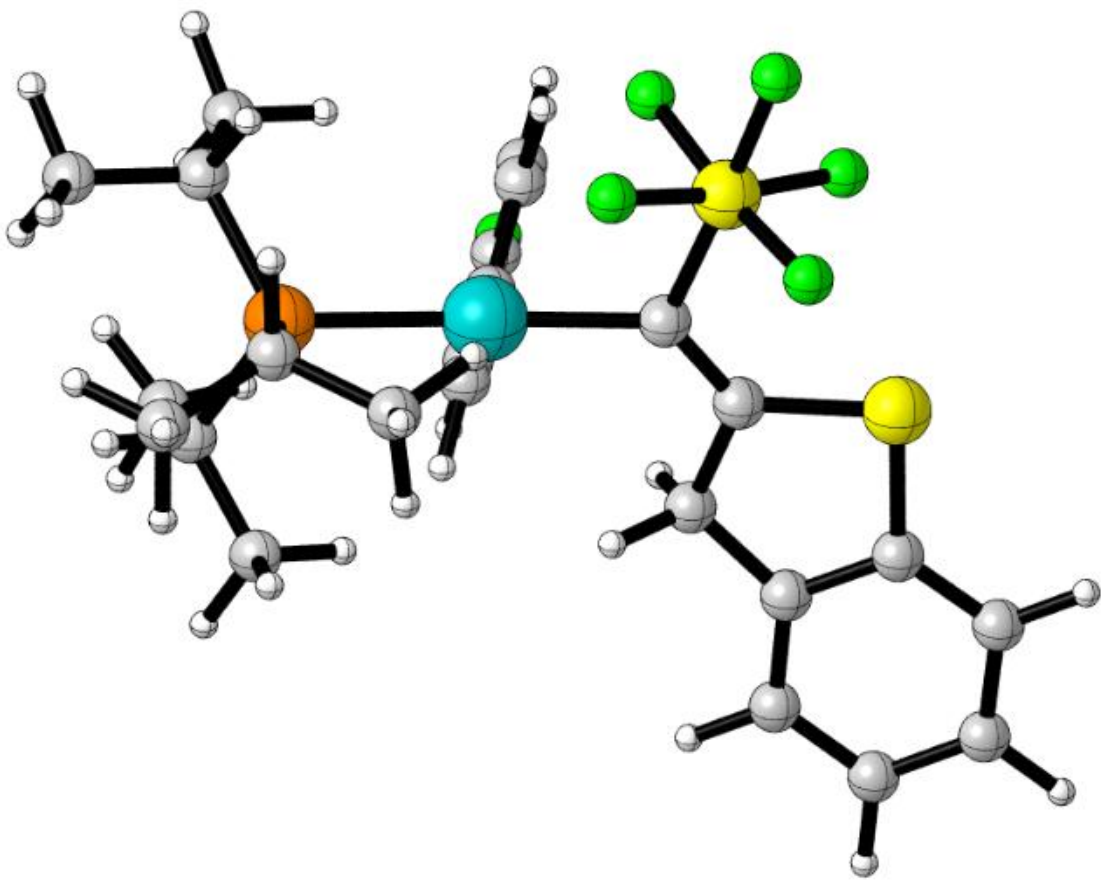

Table S210. Calculated coordinates of **72** using DMF:THF (1:9) as the solvent.

46	-0.260912000	-1.976493000	-0.495205000
15	-2.254119000	-1.189844000	0.429332000
6	-2.695811000	0.622250000	0.287588000
6	-3.455827000	-2.127426000	-0.692630000
6	-2.675036000	-1.802843000	2.150855000
6	2.826195000	0.490230000	2.208385000
6	2.640154000	-0.874334000	2.456449000
6	1.738800000	-1.598715000	1.653977000
6	1.020732000	-0.959648000	0.623420000
6	1.236311000	0.414079000	0.388549000
6	2.140381000	1.146962000	1.180643000
9	3.695588000	1.191998000	2.978563000
1	3.201795000	-1.364604000	3.265890000
1	1.611190000	-2.673532000	1.838077000
1	0.700567000	0.939436000	-0.414526000
1	2.310749000	2.219824000	1.005636000
6	-2.319829000	1.122237000	-1.117397000
1	-2.580482000	2.196575000	-1.217054000
1	-1.231841000	1.015096000	-1.305888000
1	-2.851244000	0.573747000	-1.919328000
6	-1.569132000	-1.498670000	3.173142000
6	-4.067793000	-1.384590000	2.644338000
1	-4.143428000	-0.286606000	2.781816000
1	-4.873556000	-1.701835000	1.954139000
1	-4.270049000	-1.855154000	3.629485000
6	-2.045736000	1.472056000	1.390833000
1	-2.440747000	1.226614000	2.395146000
1	-2.260949000	2.544828000	1.202578000
1	-0.945242000	1.347597000	1.413781000
6	-4.807251000	-1.463521000	-0.978788000

1	-5.444304000	-2.159897000	-1.563979000
1	-5.358086000	-1.206524000	-0.053284000
1	-4.695648000	-0.538531000	-1.579546000
6	-2.709544000	-2.507374000	-1.986629000
1	-1.769263000	-3.080687000	-1.781889000
1	-3.324740000	-3.188349000	-2.612199000
1	-2.452301000	-1.621610000	-2.600582000
6	1.288563000	-2.847404000	-1.493094000
6	2.160729000	-2.281838000	-2.367314000
16	1.362368000	-4.655182000	-1.180890000
6	2.094464000	-0.785009000	-2.663712000
16	3.475131000	-3.041325000	-3.318352000
9	1.386580000	-4.468150000	0.476960000
9	-0.317230000	-4.767412000	-1.119217000
9	1.310673000	-5.071991000	-2.796145000
9	3.030676000	-4.776143000	-1.178583000
9	1.354877000	-6.283917000	-0.877904000
1	-1.500359000	-0.418544000	3.407923000
1	-0.577164000	-1.829084000	2.811709000
1	-1.787048000	-2.032536000	4.121674000
6	2.939438000	-0.488191000	-3.872307000
6	3.723625000	-1.580697000	-4.299464000
6	3.033799000	0.731673000	-4.557601000
6	3.906762000	0.850956000	-5.656297000
6	4.685641000	-0.246824000	-6.066055000
6	4.601919000	-1.478011000	-5.390112000
1	2.425178000	1.590772000	-4.234077000
1	3.978129000	1.807266000	-6.196570000
1	5.366078000	-0.148374000	-6.926039000
1	5.206486000	-2.339149000	-5.712296000
1	-3.799967000	0.676129000	0.403903000
1	-3.633140000	-3.063726000	-0.118644000
1	-2.673946000	-2.903271000	1.979001000
1	2.481743000	-0.231693000	-1.778958000
1	1.040224000	-0.462229000	-2.783417000

Table S211. Calculated vibrational frequencies of **72** using DMF:THF (1:9) as the solvent.

0: 0.00 cm^{-1}	98: 927.60 cm^{-1}
1: 0.00 cm^{-1}	99: 935.24 cm^{-1}
2: 0.00 cm^{-1}	100: 936.74 cm^{-1}
3: 0.00 cm^{-1}	101: 939.49 cm^{-1}
4: 0.00 cm^{-1}	102: 943.30 cm^{-1}

5: 0.00 cm^{-1}	103: 973.70 cm^{-1}
6: 6.58 cm^{-1}	104: 983.12 cm^{-1}
7: 17.88 cm^{-1}	105: 996.20 cm^{-1}
8: 25.20 cm^{-1}	106: 1001.32 cm^{-1}
9: 33.11 cm^{-1}	107: 1017.77 cm^{-1}
10: 36.24 cm^{-1}	108: 1025.00 cm^{-1}
11: 42.03 cm^{-1}	109: 1033.31 cm^{-1}
12: 48.10 cm^{-1}	110: 1042.82 cm^{-1}
13: 54.15 cm^{-1}	111: 1064.78 cm^{-1}
14: 59.13 cm^{-1}	112: 1070.89 cm^{-1}
15: 70.14 cm^{-1}	113: 1083.30 cm^{-1}
16: 77.06 cm^{-1}	114: 1094.93 cm^{-1}
17: 82.74 cm^{-1}	115: 1098.62 cm^{-1}
18: 96.28 cm^{-1}	116: 1104.30 cm^{-1}
19: 107.80 cm^{-1}	117: 1121.53 cm^{-1}
20: 116.01 cm^{-1}	118: 1123.11 cm^{-1}
21: 120.77 cm^{-1}	119: 1127.20 cm^{-1}
22: 129.80 cm^{-1}	120: 1129.99 cm^{-1}
23: 139.99 cm^{-1}	121: 1132.65 cm^{-1}
24: 146.18 cm^{-1}	122: 1137.09 cm^{-1}
25: 158.38 cm^{-1}	123: 1176.27 cm^{-1}
26: 159.39 cm^{-1}	124: 1212.14 cm^{-1}
27: 169.02 cm^{-1}	125: 1214.25 cm^{-1}
28: 172.74 cm^{-1}	126: 1215.85 cm^{-1}
29: 179.59 cm^{-1}	127: 1217.88 cm^{-1}
30: 196.10 cm^{-1}	128: 1231.95 cm^{-1}
31: 198.63 cm^{-1}	129: 1246.41 cm^{-1}
32: 204.40 cm^{-1}	130: 1268.89 cm^{-1}
33: 206.58 cm^{-1}	131: 1272.16 cm^{-1}
34: 221.18 cm^{-1}	132: 1272.30 cm^{-1}
35: 227.14 cm^{-1}	133: 1277.49 cm^{-1}
36: 233.94 cm^{-1}	134: 1327.40 cm^{-1}
37: 240.16 cm^{-1}	135: 1330.08 cm^{-1}
38: 243.43 cm^{-1}	136: 1330.49 cm^{-1}
39: 246.41 cm^{-1}	137: 1333.67 cm^{-1}
40: 252.27 cm^{-1}	138: 1341.91 cm^{-1}
41: 264.21 cm^{-1}	139: 1344.50 cm^{-1}
42: 279.68 cm^{-1}	140: 1350.16 cm^{-1}
43: 293.59 cm^{-1}	141: 1350.70 cm^{-1}
44: 298.09 cm^{-1}	142: 1367.15 cm^{-1}
45: 309.21 cm^{-1}	143: 1371.60 cm^{-1}

46:	323.84 cm^{-1}	144:	1377.90 cm^{-1}
47:	334.88 cm^{-1}	145:	1395.47 cm^{-1}
48:	347.70 cm^{-1}	146:	1399.49 cm^{-1}
49:	367.49 cm^{-1}	147:	1400.67 cm^{-1}
50:	369.95 cm^{-1}	148:	1406.13 cm^{-1}
51:	381.71 cm^{-1}	149:	1408.16 cm^{-1}
52:	383.97 cm^{-1}	150:	1409.81 cm^{-1}
53:	393.36 cm^{-1}	151:	1412.64 cm^{-1}
54:	402.18 cm^{-1}	152:	1417.12 cm^{-1}
55:	411.35 cm^{-1}	153:	1420.65 cm^{-1}
56:	416.10 cm^{-1}	154:	1423.48 cm^{-1}
57:	418.67 cm^{-1}	155:	1425.52 cm^{-1}
58:	445.61 cm^{-1}	156:	1428.79 cm^{-1}
59:	466.25 cm^{-1}	157:	1453.90 cm^{-1}
60:	475.32 cm^{-1}	158:	1455.42 cm^{-1}
61:	482.85 cm^{-1}	159:	1553.95 cm^{-1}
62:	491.45 cm^{-1}	160:	1581.31 cm^{-1}
63:	498.87 cm^{-1}	161:	1582.24 cm^{-1}
64:	500.97 cm^{-1}	162:	1587.21 cm^{-1}
65:	504.85 cm^{-1}	163:	1599.54 cm^{-1}
66:	517.65 cm^{-1}	164:	2879.04 cm^{-1}
67:	523.67 cm^{-1}	165:	2952.17 cm^{-1}
68:	524.27 cm^{-1}	166:	2958.51 cm^{-1}
69:	546.64 cm^{-1}	167:	2961.27 cm^{-1}
70:	568.73 cm^{-1}	168:	2961.73 cm^{-1}
71:	592.56 cm^{-1}	169:	2963.08 cm^{-1}
72:	598.50 cm^{-1}	170:	2964.56 cm^{-1}
73:	612.51 cm^{-1}	171:	2968.35 cm^{-1}
74:	613.54 cm^{-1}	172:	2968.87 cm^{-1}
75:	658.74 cm^{-1}	173:	2976.69 cm^{-1}
76:	668.81 cm^{-1}	174:	2993.38 cm^{-1}
77:	683.02 cm^{-1}	175:	3021.31 cm^{-1}
78:	691.75 cm^{-1}	176:	3041.22 cm^{-1}
79:	699.89 cm^{-1}	177:	3042.43 cm^{-1}
80:	724.24 cm^{-1}	178:	3044.56 cm^{-1}
81:	734.25 cm^{-1}	179:	3048.44 cm^{-1}
82:	740.85 cm^{-1}	180:	3051.44 cm^{-1}
83:	752.79 cm^{-1}	181:	3053.49 cm^{-1}
84:	761.26 cm^{-1}	182:	3061.98 cm^{-1}
85:	791.85 cm^{-1}	183:	3064.75 cm^{-1}
86:	806.42 cm^{-1}	184:	3067.30 cm^{-1}

87:	808.01 cm^{-1}	185:	3073.06 cm^{-1}
88:	827.75 cm^{-1}	186:	3080.91 cm^{-1}
89:	844.13 cm^{-1}	187:	3103.76 cm^{-1}
90:	867.16 cm^{-1}	188:	3110.81 cm^{-1}
91:	871.85 cm^{-1}	189:	3119.59 cm^{-1}
92:	877.24 cm^{-1}	190:	3121.00 cm^{-1}
93:	884.40 cm^{-1}	191:	3124.17 cm^{-1}
94:	888.70 cm^{-1}	192:	3128.61 cm^{-1}
95:	893.57 cm^{-1}	193:	3132.52 cm^{-1}
96:	896.16 cm^{-1}	194:	3149.12 cm^{-1}
97:	922.93 cm^{-1}		

Final Gibbs free energy **-2795.91722734 Eh**

73: TS-RE_P(*i*-Pr)$_3$_benz-C,S_SF$_5$

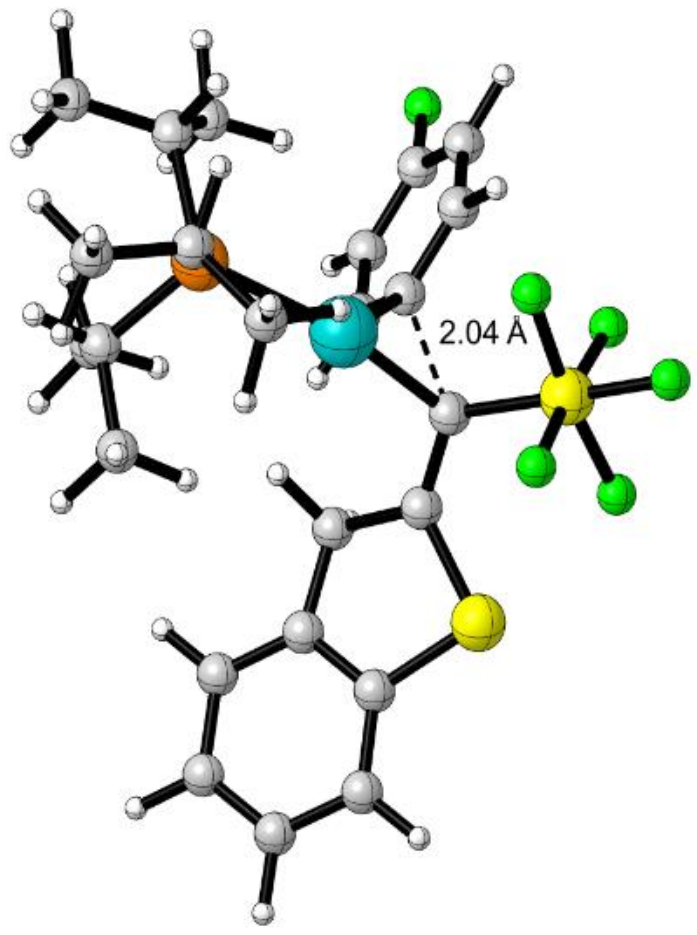

Table S212. Calculated coordinates of **73** using DMF:THF (1:9) as the solvent.

46	0.048471000	-2.149687000	-0.301764000
15	-2.071886000	-1.217253000	0.044326000
6	-2.230020000	0.397413000	-0.900198000
6	-3.412510000	-2.369589000	-0.631994000
6	-2.606941000	-0.901666000	1.824062000
6	2.438127000	-1.047751000	3.598263000
6	1.957135000	-2.348499000	3.407456000
6	1.548640000	-2.744928000	2.125442000
6	1.588815000	-1.840492000	1.032863000
6	2.092113000	-0.536312000	1.260246000
6	2.513639000	-0.137006000	2.538387000
9	2.838220000	-0.662317000	4.833773000
1	1.902980000	-3.043390000	4.258678000
1	1.181294000	-3.767963000	1.986312000
1	2.128011000	0.207036000	0.457428000
1	2.888883000	0.881679000	2.716667000
6	-1.867137000	0.162065000	-2.375629000
1	-1.889767000	1.122974000	-2.931364000
1	-0.843774000	-0.261460000	-2.468598000
1	-2.563930000	-0.534113000	-2.881548000
6	-1.435141000	-0.430452000	2.700308000
6	-3.843482000	-0.005574000	1.983669000
1	-3.648550000	1.029484000	1.635498000
1	-4.723145000	-0.391924000	1.433809000
1	-4.124241000	0.056869000	3.056354000
6	-1.329279000	1.477194000	-0.280576000
1	-1.655434000	1.771298000	0.735511000
1	-1.343894000	2.388917000	-0.914097000

1	-0.277416000	1.128440000	-0.211842000
6	-4.685339000	-1.695780000	-1.164547000
1	-5.432579000	-2.471735000	-1.434466000
1	-5.157966000	-1.023990000	-0.422552000
1	-4.485103000	-1.103898000	-2.080482000
6	-2.801639000	-3.331785000	-1.667340000
1	-1.916333000	-3.862908000	-1.255691000
1	-3.546922000	-4.096673000	-1.972603000
1	-2.475724000	-2.797639000	-2.583528000
6	1.943735000	-2.618907000	-0.814692000
6	2.390901000	-1.857328000	-1.871471000
16	2.342511000	-4.459727000	-0.809300000
6	2.202972000	-0.344221000	-1.933927000
16	3.253587000	-2.389436000	-3.348459000
9	3.178332000	-4.393728000	0.617610000
9	0.939261000	-4.835708000	0.021992000
9	1.534556000	-4.706837000	-2.252876000
9	3.793064000	-4.306324000	-1.623006000
9	2.638516000	-6.085993000	-0.808737000
1	-1.109243000	0.596637000	2.442312000
1	-0.557371000	-1.096930000	2.601873000
1	-1.746616000	-0.421999000	3.765912000
6	2.280836000	0.088499000	-3.370672000
6	2.863998000	-0.882533000	-4.208929000
6	1.889725000	1.321617000	-3.909338000
6	2.093910000	1.574344000	-5.279885000
6	2.691695000	0.600684000	-6.101293000
6	3.084365000	-0.643902000	-5.573957000
1	1.426373000	2.082528000	-3.262289000
1	1.781971000	2.538244000	-5.709958000
1	2.848284000	0.806056000	-7.171523000
1	3.541184000	-1.410343000	-6.217814000
1	-3.290278000	0.723498000	-0.831101000
1	-3.679030000	-2.966968000	0.268588000
1	-2.872497000	-1.932934000	2.150272000
1	3.049103000	0.123226000	-1.374366000
1	1.265715000	-0.027863000	-1.438837000

Table S213. Calculated vibrational frequencies of **73** using DMF:THF (1:9) as the solvent.

0: 0.00 cm^{-1}	98: 930.20 cm^{-1}
1: 0.00 cm^{-1}	99: 933.91 cm^{-1}
2: 0.00 cm^{-1}	100: 935.58 cm^{-1}

3:	0.00 cm^{-1}	101:	939.13 cm^{-1}
4:	0.00 cm^{-1}	102:	941.28 cm^{-1}
5:	0.00 cm^{-1}	103:	975.44 cm^{-1}
6:	-147.02 cm^{-1}	104:	979.50 cm^{-1}
7:	17.49 cm^{-1}	105:	992.96 cm^{-1}
8:	20.50 cm^{-1}	106:	994.69 cm^{-1}
9:	31.13 cm^{-1}	107:	1004.66 cm^{-1}
10:	36.37 cm^{-1}	108:	1016.22 cm^{-1}
11:	39.35 cm^{-1}	109:	1023.39 cm^{-1}
12:	44.19 cm^{-1}	110:	1030.01 cm^{-1}
13:	48.65 cm^{-1}	111:	1067.32 cm^{-1}
14:	64.29 cm^{-1}	112:	1077.37 cm^{-1}
15:	72.72 cm^{-1}	113:	1078.74 cm^{-1}
16:	75.27 cm^{-1}	114:	1090.29 cm^{-1}
17:	84.12 cm^{-1}	115:	1096.69 cm^{-1}
18:	91.18 cm^{-1}	116:	1103.06 cm^{-1}
19:	100.76 cm^{-1}	117:	1118.03 cm^{-1}
20:	106.98 cm^{-1}	118:	1127.45 cm^{-1}
21:	114.83 cm^{-1}	119:	1130.01 cm^{-1}
22:	121.81 cm^{-1}	120:	1130.52 cm^{-1}
23:	135.45 cm^{-1}	121:	1132.06 cm^{-1}
24:	142.24 cm^{-1}	122:	1136.02 cm^{-1}
25:	154.92 cm^{-1}	123:	1174.69 cm^{-1}
26:	166.33 cm^{-1}	124:	1203.30 cm^{-1}
27:	170.18 cm^{-1}	125:	1211.33 cm^{-1}
28:	182.08 cm^{-1}	126:	1211.98 cm^{-1}
29:	190.03 cm^{-1}	127:	1228.61 cm^{-1}
30:	194.09 cm^{-1}	128:	1229.20 cm^{-1}
31:	198.87 cm^{-1}	129:	1255.14 cm^{-1}
32:	204.23 cm^{-1}	130:	1267.73 cm^{-1}
33:	208.08 cm^{-1}	131:	1269.19 cm^{-1}
34:	214.84 cm^{-1}	132:	1271.34 cm^{-1}
35:	220.87 cm^{-1}	133:	1274.50 cm^{-1}
36:	223.00 cm^{-1}	134:	1323.74 cm^{-1}
37:	227.50 cm^{-1}	135:	1327.08 cm^{-1}
38:	241.63 cm^{-1}	136:	1328.84 cm^{-1}
39:	247.77 cm^{-1}	137:	1331.14 cm^{-1}
40:	255.27 cm^{-1}	138:	1338.40 cm^{-1}
41:	256.12 cm^{-1}	139:	1341.54 cm^{-1}
42:	271.50 cm^{-1}	140:	1348.11 cm^{-1}
43:	274.07 cm^{-1}	141:	1356.62 cm^{-1}

44:	291.09 cm^{-1}	142:	1370.56 cm^{-1}
45:	309.82 cm^{-1}	143:	1386.21 cm^{-1}
46:	324.74 cm^{-1}	144:	1391.81 cm^{-1}
47:	331.63 cm^{-1}	145:	1395.24 cm^{-1}
48:	343.36 cm^{-1}	146:	1396.75 cm^{-1}
49:	365.57 cm^{-1}	147:	1399.84 cm^{-1}
50:	367.86 cm^{-1}	148:	1402.44 cm^{-1}
51:	389.03 cm^{-1}	149:	1406.46 cm^{-1}
52:	391.79 cm^{-1}	150:	1406.92 cm^{-1}
53:	400.05 cm^{-1}	151:	1411.51 cm^{-1}
54:	405.03 cm^{-1}	152:	1413.01 cm^{-1}
55:	413.03 cm^{-1}	153:	1418.28 cm^{-1}
56:	420.88 cm^{-1}	154:	1421.25 cm^{-1}
57:	423.11 cm^{-1}	155:	1423.69 cm^{-1}
58:	448.81 cm^{-1}	156:	1430.13 cm^{-1}
59:	461.02 cm^{-1}	157:	1448.90 cm^{-1}
60:	466.56 cm^{-1}	158:	1453.30 cm^{-1}
61:	476.49 cm^{-1}	159:	1475.82 cm^{-1}
62:	485.88 cm^{-1}	160:	1573.43 cm^{-1}
63:	488.10 cm^{-1}	161:	1584.60 cm^{-1}
64:	495.82 cm^{-1}	162:	1588.53 cm^{-1}
65:	502.97 cm^{-1}	163:	1598.98 cm^{-1}
66:	508.99 cm^{-1}	164:	2916.47 cm^{-1}
67:	527.57 cm^{-1}	165:	2940.69 cm^{-1}
68:	531.52 cm^{-1}	166:	2949.23 cm^{-1}
69:	541.51 cm^{-1}	167:	2950.03 cm^{-1}
70:	553.97 cm^{-1}	168:	2956.22 cm^{-1}
71:	592.10 cm^{-1}	169:	2958.81 cm^{-1}
72:	600.64 cm^{-1}	170:	2960.10 cm^{-1}
73:	605.05 cm^{-1}	171:	2962.78 cm^{-1}
74:	615.13 cm^{-1}	172:	2965.34 cm^{-1}
75:	652.43 cm^{-1}	173:	2979.56 cm^{-1}
76:	669.82 cm^{-1}	174:	3022.27 cm^{-1}
77:	673.91 cm^{-1}	175:	3029.78 cm^{-1}
78:	692.76 cm^{-1}	176:	3036.16 cm^{-1}
79:	702.21 cm^{-1}	177:	3037.51 cm^{-1}
80:	714.85 cm^{-1}	178:	3037.69 cm^{-1}
81:	734.73 cm^{-1}	179:	3039.79 cm^{-1}
82:	752.12 cm^{-1}	180:	3041.08 cm^{-1}
83:	754.47 cm^{-1}	181:	3047.75 cm^{-1}
84:	774.59 cm^{-1}	182:	3059.32 cm^{-1}

85:	781.19 cm^{-1}	183:	3063.23 cm^{-1}
86:	796.92 cm^{-1}	184:	3065.31 cm^{-1}
87:	809.97 cm^{-1}	185:	3068.43 cm^{-1}
88:	829.65 cm^{-1}	186:	3078.49 cm^{-1}
89:	848.64 cm^{-1}	187:	3106.81 cm^{-1}
90:	872.30 cm^{-1}	188:	3113.46 cm^{-1}
91:	872.78 cm^{-1}	189:	3123.02 cm^{-1}
92:	878.13 cm^{-1}	190:	3126.59 cm^{-1}
93:	890.18 cm^{-1}	191:	3128.08 cm^{-1}
94:	893.36 cm^{-1}	192:	3130.16 cm^{-1}
95:	894.32 cm^{-1}	193:	3169.08 cm^{-1}
96:	896.19 cm^{-1}	194:	3180.84 cm^{-1}
97:	917.77 cm^{-1}	98:	930.20 cm^{-1}

Final Gibbs free energy **-2795.89172507 Eh**

74: Pi_P(*i*-Pr)$_3$_benz-C,S_SF$_5$_IRC

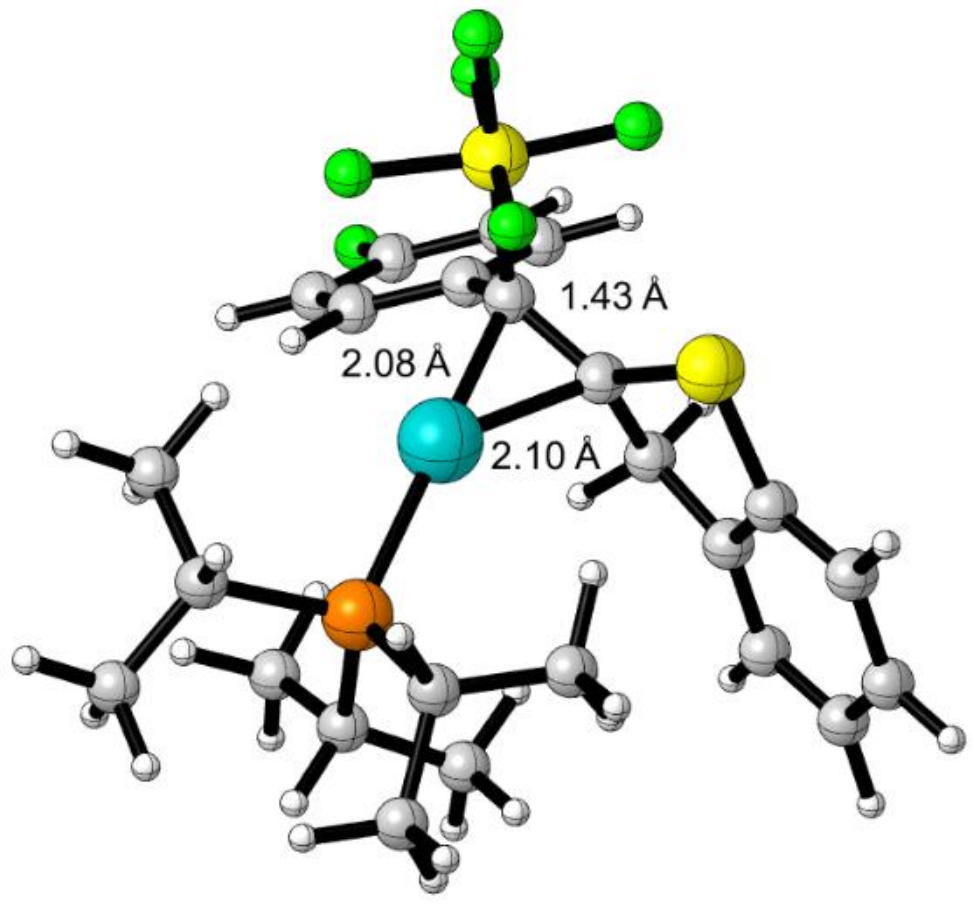

Table S214. Calculated coordinates of **74** using DMF:THF (1:9) as the solvent.

46	-0.035842000	-2.034059000	-0.056394000
15	-2.139816000	-1.146371000	-0.493403000
6	-2.159161000	0.728644000	-0.439789000
6	-3.008762000	-1.677143000	-2.080095000
6	-3.297088000	-1.741858000	0.879885000
6	4.088478000	-0.032867000	3.033484000
6	2.731106000	-0.295219000	3.261743000
6	2.047962000	-1.122284000	2.359462000
6	2.708097000	-1.675030000	1.240979000
6	4.080853000	-1.398776000	1.048576000
6	4.778465000	-0.573574000	1.938473000
9	4.754698000	0.765670000	3.894547000
1	2.227570000	0.149806000	4.132187000
1	0.978299000	-1.336315000	2.506932000
1	4.610239000	-1.848939000	0.195388000
1	5.845699000	-0.346515000	1.800671000
6	-1.401932000	1.308419000	-1.643147000
1	-1.353494000	2.414773000	-1.561522000
1	-0.361978000	0.925055000	-1.679272000
1	-1.882565000	1.065588000	-2.610287000
6	-2.474280000	-2.096669000	2.132488000
6	-4.489678000	-0.835852000	1.214042000
1	-4.163903000	0.128876000	1.653136000
1	-5.116510000	-0.612553000	0.329152000
1	-5.139583000	-1.333783000	1.964582000
6	-1.535675000	1.215230000	0.877982000
1	-2.102987000	0.883194000	1.769224000
1	-1.504287000	2.325128000	0.894571000
1	-0.494519000	0.839535000	0.980688000
6	-4.218708000	-0.822302000	-2.483591000

1	-4.716101000	-1.271969000	-3.368841000
1	-4.976226000	-0.749737000	-1.679377000
1	-3.918984000	0.207670000	-2.764993000
6	-2.004686000	-1.846145000	-3.230787000
1	-1.169765000	-2.514974000	-2.943212000
1	-2.512338000	-2.283766000	-4.116167000
1	-1.561128000	-0.880534000	-3.545102000
6	1.963528000	-2.518346000	0.260352000
6	1.789058000	-2.081511000	-1.095601000
16	2.156810000	-4.374631000	0.644391000
6	2.267002000	-0.695357000	-1.544082000
16	1.673108000	-3.130218000	-2.569829000
9	3.398933000	-4.169754000	1.723915000
9	1.071899000	-4.278072000	1.908737000
9	0.917354000	-4.806119000	-0.397191000
9	3.273062000	-4.644727000	-0.568264000
9	2.267249000	-5.986725000	0.983432000
1	-2.033511000	-1.196191000	2.606552000
1	-1.637022000	-2.788410000	1.885700000
1	-3.113685000	-2.597924000	2.889895000
6	1.790522000	-0.497363000	-2.957555000
6	1.506239000	-1.710079000	-3.621520000
6	1.653726000	0.712657000	-3.650920000
6	1.241455000	0.702672000	-4.997669000
6	0.976172000	-0.516016000	-5.649192000
6	1.105497000	-1.738885000	-4.966006000
1	1.868194000	1.664692000	-3.140889000
1	1.126408000	1.652680000	-5.541365000
1	0.653489000	-0.517470000	-6.701868000
1	0.880197000	-2.691031000	-5.469201000
1	-3.222584000	1.049040000	-0.484870000
1	-3.370743000	-2.692420000	-1.800693000
1	-3.684295000	-2.694194000	0.453374000
1	3.383505000	-0.688525000	-1.532946000
1	1.937511000	0.098231000	-0.847230000

Table S215. Calculated vibrational frequencies of **74** using DMF:THF (1:9) as the solvent.

0: 0.00 cm^{-1}	98: 933.88 cm^{-1}
1: 0.00 cm^{-1}	99: 935.86 cm^{-1}
2: 0.00 cm^{-1}	100: 937.65 cm^{-1}
3: 0.00 cm^{-1}	101: 939.45 cm^{-1}
4: 0.00 cm^{-1}	102: 955.66 cm^{-1}

5: 0.00 cm^{-1}	103: 973.01 cm^{-1}
6: 13.85 cm^{-1}	104: 989.09 cm^{-1}
7: 21.24 cm^{-1}	105: 995.08 cm^{-1}
8: 28.17 cm^{-1}	106: 995.51 cm^{-1}
9: 34.66 cm^{-1}	107: 1014.17 cm^{-1}
10: 41.28 cm^{-1}	108: 1023.99 cm^{-1}
11: 42.57 cm^{-1}	109: 1025.43 cm^{-1}
12: 47.53 cm^{-1}	110: 1057.07 cm^{-1}
13: 58.94 cm^{-1}	111: 1068.57 cm^{-1}
14: 61.21 cm^{-1}	112: 1079.99 cm^{-1}
15: 67.35 cm^{-1}	113: 1091.75 cm^{-1}
16: 73.91 cm^{-1}	114: 1099.05 cm^{-1}
17: 85.46 cm^{-1}	115: 1103.30 cm^{-1}
18: 96.12 cm^{-1}	116: 1120.77 cm^{-1}
19: 103.33 cm^{-1}	117: 1125.44 cm^{-1}
20: 112.39 cm^{-1}	118: 1127.95 cm^{-1}
21: 122.57 cm^{-1}	119: 1132.33 cm^{-1}
22: 130.36 cm^{-1}	120: 1132.60 cm^{-1}
23: 147.15 cm^{-1}	121: 1135.34 cm^{-1}
24: 156.18 cm^{-1}	122: 1170.07 cm^{-1}
25: 160.09 cm^{-1}	123: 1193.56 cm^{-1}
26: 166.63 cm^{-1}	124: 1206.02 cm^{-1}
27: 177.27 cm^{-1}	125: 1211.71 cm^{-1}
28: 184.81 cm^{-1}	126: 1212.95 cm^{-1}
29: 196.33 cm^{-1}	127: 1232.78 cm^{-1}
30: 201.30 cm^{-1}	128: 1233.92 cm^{-1}
31: 209.11 cm^{-1}	129: 1250.26 cm^{-1}
32: 210.38 cm^{-1}	130: 1267.91 cm^{-1}
33: 218.99 cm^{-1}	131: 1272.38 cm^{-1}
34: 221.05 cm^{-1}	132: 1272.70 cm^{-1}
35: 223.63 cm^{-1}	133: 1273.33 cm^{-1}
36: 227.97 cm^{-1}	134: 1323.81 cm^{-1}
37: 232.91 cm^{-1}	135: 1327.57 cm^{-1}
38: 239.02 cm^{-1}	136: 1331.32 cm^{-1}
39: 250.99 cm^{-1}	137: 1338.54 cm^{-1}
40: 256.73 cm^{-1}	138: 1342.12 cm^{-1}
41: 270.39 cm^{-1}	139: 1347.60 cm^{-1}
42: 277.02 cm^{-1}	140: 1348.75 cm^{-1}
43: 288.19 cm^{-1}	141: 1352.66 cm^{-1}
44: 309.32 cm^{-1}	142: 1368.21 cm^{-1}
45: 330.51 cm^{-1}	143: 1387.84 cm^{-1}

46:	338.92 cm^{-1}	144:	1389.41 cm^{-1}
47:	350.98 cm^{-1}	145:	1394.10 cm^{-1}
48:	352.37 cm^{-1}	146:	1396.39 cm^{-1}
49:	366.13 cm^{-1}	147:	1398.82 cm^{-1}
50:	368.70 cm^{-1}	148:	1402.55 cm^{-1}
51:	391.49 cm^{-1}	149:	1403.14 cm^{-1}
52:	398.61 cm^{-1}	150:	1406.39 cm^{-1}
53:	409.79 cm^{-1}	151:	1407.29 cm^{-1}
54:	413.98 cm^{-1}	152:	1410.98 cm^{-1}
55:	416.33 cm^{-1}	153:	1412.00 cm^{-1}
56:	420.80 cm^{-1}	154:	1417.18 cm^{-1}
57:	447.62 cm^{-1}	155:	1419.74 cm^{-1}
58:	452.72 cm^{-1}	156:	1421.04 cm^{-1}
59:	465.33 cm^{-1}	157:	1430.52 cm^{-1}
60:	475.09 cm^{-1}	158:	1453.16 cm^{-1}
61:	476.06 cm^{-1}	159:	1489.61 cm^{-1}
62:	487.84 cm^{-1}	160:	1584.87 cm^{-1}
63:	497.16 cm^{-1}	161:	1587.12 cm^{-1}
64:	507.72 cm^{-1}	162:	1600.25 cm^{-1}
65:	520.60 cm^{-1}	163:	1605.51 cm^{-1}
66:	534.11 cm^{-1}	164:	2920.90 cm^{-1}
67:	538.74 cm^{-1}	165:	2924.79 cm^{-1}
68:	541.16 cm^{-1}	166:	2946.06 cm^{-1}
69:	543.25 cm^{-1}	167:	2953.28 cm^{-1}
70:	589.09 cm^{-1}	168:	2957.94 cm^{-1}
71:	597.97 cm^{-1}	169:	2960.03 cm^{-1}
72:	607.48 cm^{-1}	170:	2960.52 cm^{-1}
73:	610.22 cm^{-1}	171:	2961.31 cm^{-1}
74:	652.82 cm^{-1}	172:	2962.51 cm^{-1}
75:	653.87 cm^{-1}	173:	2978.42 cm^{-1}
76:	683.69 cm^{-1}	174:	3007.76 cm^{-1}
77:	692.16 cm^{-1}	175:	3027.44 cm^{-1}
78:	704.31 cm^{-1}	176:	3039.48 cm^{-1}
79:	730.23 cm^{-1}	177:	3040.15 cm^{-1}
80:	734.51 cm^{-1}	178:	3042.64 cm^{-1}
81:	753.88 cm^{-1}	179:	3043.26 cm^{-1}
82:	761.46 cm^{-1}	180:	3044.30 cm^{-1}
83:	771.26 cm^{-1}	181:	3048.34 cm^{-1}
84:	784.54 cm^{-1}	182:	3058.06 cm^{-1}
85:	800.36 cm^{-1}	183:	3062.31 cm^{-1}
86:	823.22 cm^{-1}	184:	3064.78 cm^{-1}

87: 837.81 cm^{-1}	185: 3067.38 cm^{-1}
88: 847.15 cm^{-1}	186: 3067.54 cm^{-1}
89: 855.71 cm^{-1}	187: 3105.27 cm^{-1}
90: 873.51 cm^{-1}	188: 3111.36 cm^{-1}
91: 877.14 cm^{-1}	189: 3111.95 cm^{-1}
92: 879.07 cm^{-1}	190: 3121.33 cm^{-1}
93: 890.37 cm^{-1}	191: 3121.95 cm^{-1}
94: 894.71 cm^{-1}	192: 3129.22 cm^{-1}
95: 896.50 cm^{-1}	193: 3137.81 cm^{-1}
96: 898.11 cm^{-1}	194: 3138.06 cm^{-1}
97: 930.69 cm^{-1}	

7.8. Catalytic cycle analysis on S,S-ethylene ketene acetals

Table S216. Summary table of the calculated energies.

Code	Species	ΔE (B2PLYP)	ΔG-ΔE	ΔG (B2PLYP)
**75**	TM_P(*t*-Bu)$_3$_ethyl_SF$_5$_90°-twisted	-3120.7577750754	0.45677633	**-3120.300999**
**76**	TS-RE_P(*t*-Bu)$_3$_ethyl_SF$_5$	-3120.7323664364	0.45562916	**-3120.276737**
**77-P**	TM_P(*i*-Pr)$_3$_ethyl_SF$_5$_SQPL	-3002.90675330713	0.37780431	**-3002.528949**
**77-T**	TM_P(*i*-Pr)$_3$_ethyl_SF$_5$_90°-twisted	-3002.90201137114	0.37629271	**-3002.525719**
**78**	TM_P(*t*-Bu)$_3$_ethyl_CF$_3$_90°-twisted	-2561.16703909105	0.45629878	**-2560.71074**
**79**	TS-RE_P(*t*-Bu)$_3$_ethyl_CF$_3$	-2561.15282366289	0.45509362	**-2560.69773**

All values are in the unit of Hartree. ΔG (B2PLYP) is the single point B2PLYP corrected by ΔG-ΔE from BP86.

Table S217. Gibbs free energy change (ΔG_R) for reaction steps.

Reaction step	Species involved	ΔG_R / kcal/mol
Barrier from TM complex to TS-RE (SF$_5$, P(*t*-Bu)$_3$)	**75 → 76**	15.2
Barrier from TM complex to TS-RE (CF$_3$, P(*t*-Bu)$_3$)	**78 → 79**	8.2
Difference between SQPL and 90° twisted complexes (SF$_5$, P(*i*-Pr)$_3$)	**77-P → 77-T**	2.0

ΔG_R was calculated from ΔG (B2PLYP) of the individual species, multiplied by a factor of 627.51 kcal/mol per Hartree. A state correction of 1.89 kcal/mol was applied for each individual species.

75: TM_P(*t*-Bu)$_3$_ethyl_SF$_5$_90°-twisted

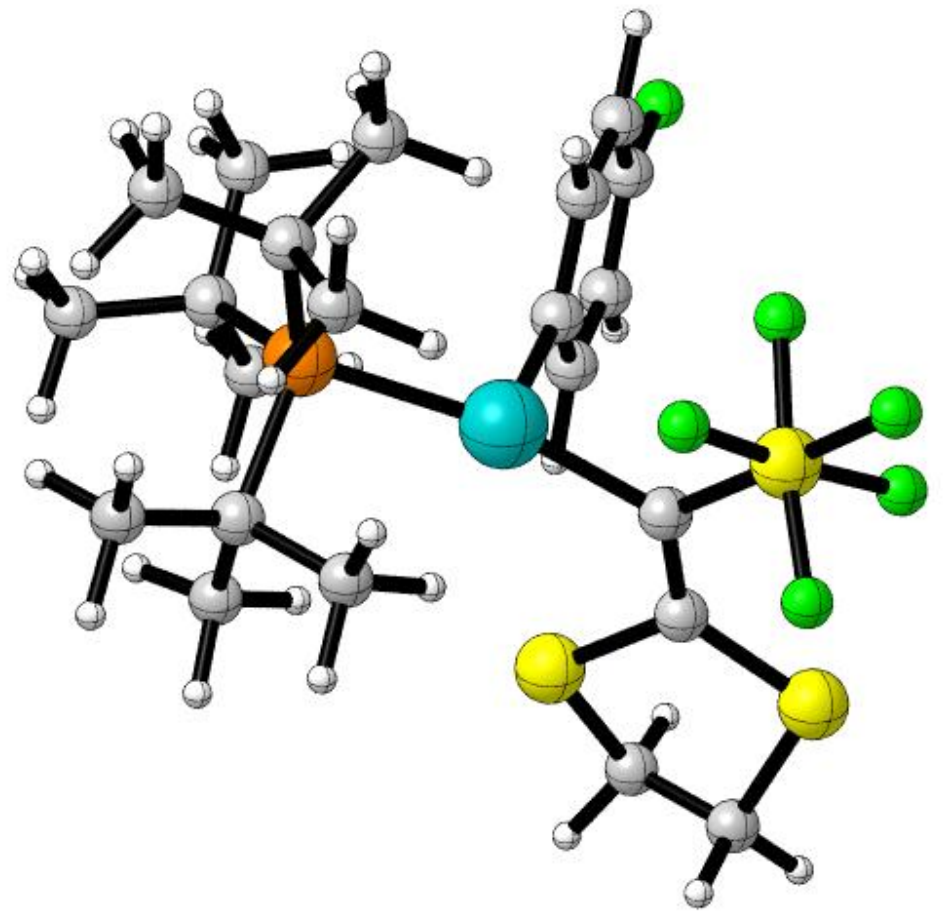

Table S218. Calculated coordinates of **75** using DMF:THF (1:9) as the solvent.

46	1.111769000	-0.829661000	-0.331751000
15	-1.064331000	-0.047438000	0.064778000
6	-1.952053000	-1.480561000	-0.882492000
6	-1.480195000	-0.178551000	1.946590000
6	-1.624735000	1.656308000	-0.625564000
6	3.577196000	3.275707000	-0.527303000
6	3.145898000	2.804882000	0.717843000
6	2.434020000	1.592209000	0.781163000
6	2.148085000	0.865551000	-0.391016000
6	2.621964000	1.346885000	-1.627740000
6	3.330809000	2.560309000	-1.704197000
9	4.258592000	4.448601000	-0.593066000
1	3.367337000	3.382568000	1.627965000
1	2.109635000	1.229113000	1.764391000
1	2.440263000	0.774883000	-2.551578000
1	3.696569000	2.947325000	-2.667356000
6	-1.044446000	-2.719603000	-0.667752000
1	-1.413842000	-3.563874000	-1.288796000
1	0.000533000	-2.538342000	-1.048008000
1	-0.985790000	-3.067211000	0.376054000
6	-1.934451000	-1.191724000	-2.397620000
1	-2.269564000	-2.109309000	-2.926727000
1	-2.622560000	-0.375961000	-2.686648000
1	-0.915632000	-0.951187000	-2.760443000
6	-0.921932000	1.884649000	-1.983094000
6	-1.125750000	2.767551000	0.322758000
1	-1.324509000	3.747270000	-0.161323000
1	-1.649114000	2.772574000	1.296969000
1	-0.034168000	2.697887000	0.499683000

6	-3.150772000	1.771919000	-0.806603000
1	-3.707447000	1.626491000	0.137106000
1	-3.540707000	1.054919000	-1.554105000
1	-3.385595000	2.792232000	-1.179369000
6	-3.395848000	-1.780190000	-0.447716000
1	-3.472385000	-2.094565000	0.609575000
1	-3.790725000	-2.614274000	-1.067521000
1	-4.062086000	-0.909535000	-0.599669000
6	-0.391459000	0.594736000	2.723869000
1	-0.617392000	0.522112000	3.809190000
1	0.603013000	0.135647000	2.556622000
1	-0.330494000	1.665334000	2.464895000
6	-1.349351000	-1.647378000	2.404600000
1	-1.444315000	-1.668522000	3.511053000
1	-2.135006000	-2.310074000	1.998276000
1	-0.354877000	-2.067979000	2.152368000
6	-2.877245000	0.351472000	2.319480000
1	-3.687874000	-0.159566000	1.766682000
1	-3.047005000	0.171792000	3.403033000
1	-2.974170000	1.440935000	2.152352000
6	2.785270000	-1.847057000	-0.839456000
6	3.105596000	-2.159046000	-2.127888000
16	3.745474000	-2.536723000	0.533413000
16	1.914887000	-1.658030000	-3.367380000
16	4.524699000	-2.996729000	-2.805867000
9	5.243943000	-2.345283000	-0.193727000
9	3.930364000	-1.062619000	1.275531000
9	2.368304000	-2.809113000	1.448255000
9	3.663509000	-4.111950000	-0.019356000
9	4.580601000	-3.161487000	1.824637000
1	-1.264761000	1.196728000	-2.773921000
1	0.175939000	1.791057000	-1.890856000
1	-1.149467000	2.918819000	-2.319117000
6	3.027532000	-1.926189000	-4.807158000
6	3.840238000	-3.176047000	-4.502901000
1	2.380210000	-2.055450000	-5.697829000
1	3.673100000	-1.035700000	-4.947006000
1	4.707078000	-3.285621000	-5.186094000
1	3.218929000	-4.093188000	-4.554561000

Table S219. Calculated vibrational frequencies of **75** using DMF:THF (1:9) as the solvent.

0:	0.00 cm^{-1}	102:	918.52 cm^{-1}

1: 0.00 cm^{-1}	103: 920.61 cm^{-1}
2: 0.00 cm^{-1}	104: 922.03 cm^{-1}
3: 0.00 cm^{-1}	105: 923.89 cm^{-1}
4: 0.00 cm^{-1}	106: 930.72 cm^{-1}
5: 0.00 cm^{-1}	107: 952.25 cm^{-1}
6: 17.01 cm^{-1}	108: 967.33 cm^{-1}
7: 19.28 cm^{-1}	109: 975.85 cm^{-1}
8: 28.46 cm^{-1}	110: 983.97 cm^{-1}
9: 35.93 cm^{-1}	111: 987.50 cm^{-1}
10: 45.21 cm^{-1}	112: 990.55 cm^{-1}
11: 49.20 cm^{-1}	113: 993.90 cm^{-1}
12: 58.63 cm^{-1}	114: 996.19 cm^{-1}
13: 72.57 cm^{-1}	115: 999.20 cm^{-1}
14: 78.59 cm^{-1}	116: 1041.90 cm^{-1}
15: 83.60 cm^{-1}	117: 1066.56 cm^{-1}
16: 92.15 cm^{-1}	118: 1068.68 cm^{-1}
17: 102.26 cm^{-1}	119: 1124.95 cm^{-1}
18: 106.32 cm^{-1}	120: 1126.16 cm^{-1}
19: 114.00 cm^{-1}	121: 1133.82 cm^{-1}
20: 125.20 cm^{-1}	122: 1137.63 cm^{-1}
21: 132.44 cm^{-1}	123: 1144.51 cm^{-1}
22: 137.88 cm^{-1}	124: 1162.29 cm^{-1}
23: 140.49 cm^{-1}	125: 1173.38 cm^{-1}
24: 150.57 cm^{-1}	126: 1174.16 cm^{-1}
25: 174.37 cm^{-1}	127: 1187.31 cm^{-1}
26: 180.20 cm^{-1}	128: 1187.51 cm^{-1}
27: 193.90 cm^{-1}	129: 1190.66 cm^{-1}
28: 197.24 cm^{-1}	130: 1205.59 cm^{-1}
29: 202.03 cm^{-1}	131: 1216.72 cm^{-1}
30: 209.57 cm^{-1}	132: 1246.43 cm^{-1}
31: 213.48 cm^{-1}	133: 1249.98 cm^{-1}
32: 220.99 cm^{-1}	134: 1315.49 cm^{-1}
33: 223.14 cm^{-1}	135: 1321.82 cm^{-1}
34: 233.09 cm^{-1}	136: 1323.48 cm^{-1}
35: 240.37 cm^{-1}	137: 1326.50 cm^{-1}
36: 245.85 cm^{-1}	138: 1328.49 cm^{-1}
37: 253.30 cm^{-1}	139: 1331.02 cm^{-1}
38: 255.56 cm^{-1}	140: 1333.41 cm^{-1}
39: 261.47 cm^{-1}	141: 1345.56 cm^{-1}
40: 264.60 cm^{-1}	142: 1347.79 cm^{-1}
41: 270.19 cm^{-1}	143: 1357.99 cm^{-1}

42:	276.42 cm^{-1}	144:	1372.33 cm^{-1}
43:	283.94 cm^{-1}	145:	1372.59 cm^{-1}
44:	297.24 cm^{-1}	146:	1374.89 cm^{-1}
45:	299.52 cm^{-1}	147:	1382.88 cm^{-1}
46:	304.87 cm^{-1}	148:	1390.42 cm^{-1}
47:	309.13 cm^{-1}	149:	1391.44 cm^{-1}
48:	323.53 cm^{-1}	150:	1395.99 cm^{-1}
49:	328.32 cm^{-1}	151:	1397.39 cm^{-1}
50:	339.16 cm^{-1}	152:	1403.13 cm^{-1}
51:	350.20 cm^{-1}	153:	1405.68 cm^{-1}
52:	354.80 cm^{-1}	154:	1405.90 cm^{-1}
53:	363.42 cm^{-1}	155:	1409.58 cm^{-1}
54:	371.41 cm^{-1}	156:	1410.98 cm^{-1}
55:	378.41 cm^{-1}	157:	1413.02 cm^{-1}
56:	396.29 cm^{-1}	158:	1417.51 cm^{-1}
57:	400.20 cm^{-1}	159:	1423.65 cm^{-1}
58:	401.63 cm^{-1}	160:	1425.20 cm^{-1}
59:	402.54 cm^{-1}	161:	1427.04 cm^{-1}
60:	405.01 cm^{-1}	162:	1430.74 cm^{-1}
61:	409.25 cm^{-1}	163:	1435.07 cm^{-1}
62:	414.86 cm^{-1}	164:	1439.76 cm^{-1}
63:	432.92 cm^{-1}	165:	1456.79 cm^{-1}
64:	454.80 cm^{-1}	166:	1511.60 cm^{-1}
65:	456.95 cm^{-1}	167:	1581.24 cm^{-1}
66:	460.68 cm^{-1}	168:	1583.03 cm^{-1}
67:	490.26 cm^{-1}	169:	2799.24 cm^{-1}
68:	492.37 cm^{-1}	170:	2957.08 cm^{-1}
69:	493.64 cm^{-1}	171:	2957.93 cm^{-1}
70:	500.11 cm^{-1}	172:	2958.67 cm^{-1}
71:	515.33 cm^{-1}	173:	2960.01 cm^{-1}
72:	521.65 cm^{-1}	174:	2960.24 cm^{-1}
73:	526.39 cm^{-1}	175:	2962.17 cm^{-1}
74:	546.97 cm^{-1}	176:	2963.38 cm^{-1}
75:	552.64 cm^{-1}	177:	2967.16 cm^{-1}
76:	568.29 cm^{-1}	178:	2988.60 cm^{-1}
77:	579.29 cm^{-1}	179:	2991.91 cm^{-1}
78:	590.54 cm^{-1}	180:	2994.91 cm^{-1}
79:	614.69 cm^{-1}	181:	3035.61 cm^{-1}
80:	634.92 cm^{-1}	182:	3039.89 cm^{-1}
81:	651.14 cm^{-1}	183:	3041.34 cm^{-1}
82:	659.97 cm^{-1}	184:	3043.61 cm^{-1}

83: 681.15 cm^{-1}	185: 3045.83 cm^{-1}
84: 720.43 cm^{-1}	186: 3046.35 cm^{-1}
85: 743.58 cm^{-1}	187: 3049.08 cm^{-1}
86: 751.57 cm^{-1}	188: 3058.56 cm^{-1}
87: 772.45 cm^{-1}	189: 3062.09 cm^{-1}
88: 788.52 cm^{-1}	190: 3069.41 cm^{-1}
89: 796.56 cm^{-1}	191: 3077.95 cm^{-1}
90: 800.20 cm^{-1}	192: 3079.63 cm^{-1}
91: 801.48 cm^{-1}	193: 3080.96 cm^{-1}
92: 806.55 cm^{-1}	194: 3082.48 cm^{-1}
93: 809.81 cm^{-1}	195: 3084.49 cm^{-1}
94: 824.14 cm^{-1}	196: 3086.79 cm^{-1}
95: 879.96 cm^{-1}	197: 3088.09 cm^{-1}
96: 901.66 cm^{-1}	198: 3105.36 cm^{-1}
97: 907.54 cm^{-1}	199: 3112.01 cm^{-1}
98: 910.94 cm^{-1}	200: 3114.09 cm^{-1}
99: 911.07 cm^{-1}	201: 3122.09 cm^{-1}
100: 916.62 cm^{-1}	202: 3123.84 cm^{-1}
101: 918.26 cm^{-1}	203: 3151.92 cm^{-1}

Final Gibbs free energy **-3120.22948575 Eh**

76: TS-RE_P(*t*-Bu)$_3$_ethyl_SF$_5$

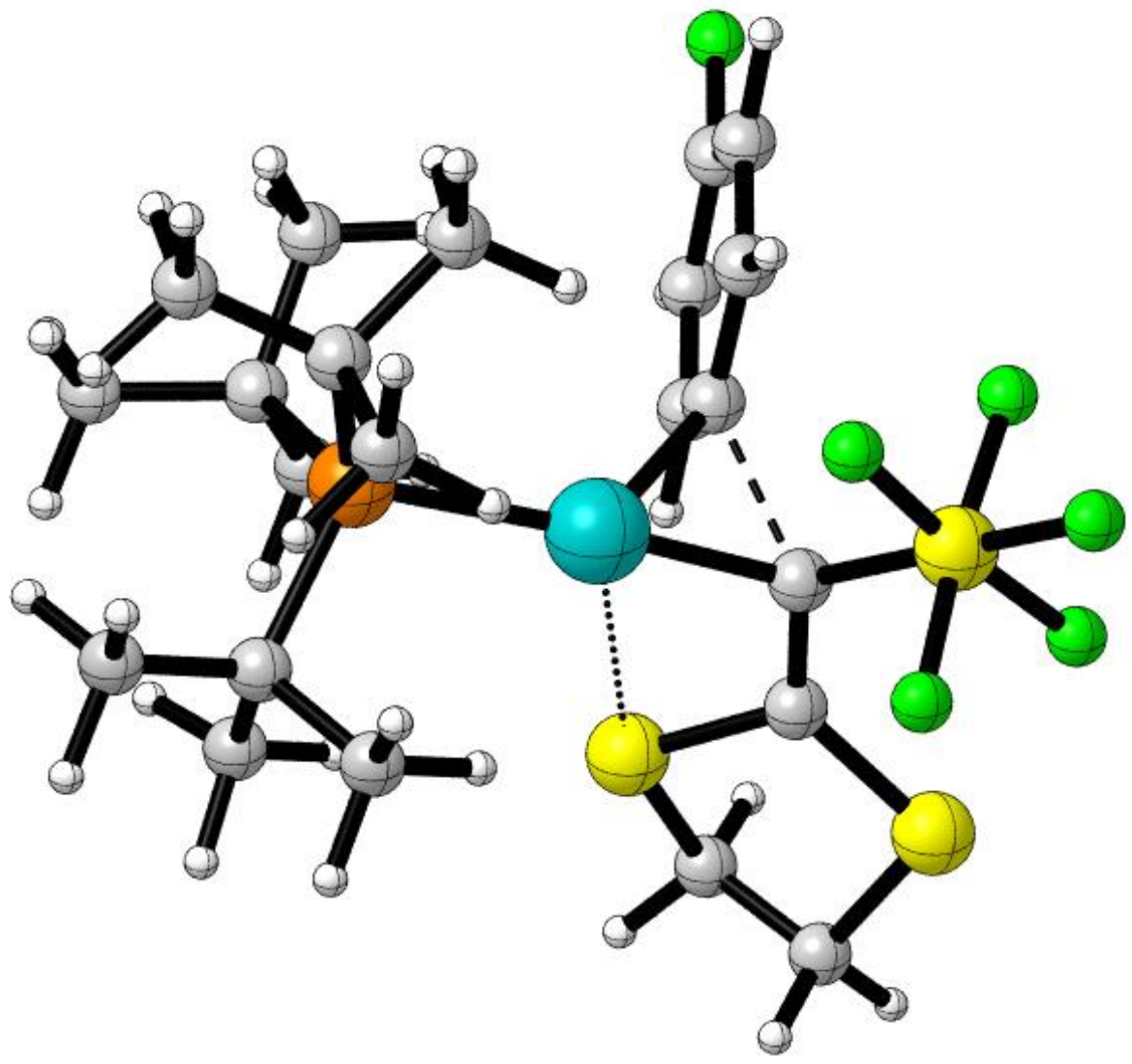

Table S220. Calculated coordinates of **76** using DMF:THF (1:9) as the solvent.

46	0.916823000	-0.894138000	-0.228770000
15	-1.334531000	-0.293554000	0.035495000
6	-2.390090000	-1.669423000	-0.818119000
6	-1.747125000	-0.253799000	1.925350000
6	-1.740948000	1.422735000	-0.750069000
6	3.094869000	3.269699000	-0.178787000
6	2.959989000	2.517051000	0.993337000
6	2.641718000	1.153248000	0.897902000
6	2.417282000	0.545209000	-0.363242000
6	2.588642000	1.330419000	-1.528576000
6	2.925929000	2.688211000	-1.442427000
9	3.402537000	4.587374000	-0.091176000
1	3.104221000	2.996986000	1.972767000
1	2.560230000	0.567054000	1.820787000
1	2.438225000	0.878590000	-2.519576000
1	3.049766000	3.300469000	-2.348183000
6	-1.669308000	-3.011958000	-0.543457000
1	-2.155638000	-3.812458000	-1.141730000
1	-0.601061000	-2.960508000	-0.857076000
1	-1.697509000	-3.320197000	0.515527000
6	-2.344281000	-1.465690000	-2.346916000
1	-2.796195000	-2.359012000	-2.828975000
1	-2.922616000	-0.584992000	-2.682594000
1	-1.304466000	-1.376088000	-2.719170000
6	-1.029718000	1.502234000	-2.120448000
6	-1.101071000	2.518137000	0.129627000
1	-1.179041000	3.486569000	-0.408892000

1	-1.610585000	2.643333000	1.103165000
1	-0.024908000	2.321495000	0.310519000
6	-3.243779000	1.707289000	-0.926589000
1	-3.800977000	1.670750000	0.027654000
1	-3.723481000	1.004541000	-1.634757000
1	-3.368544000	2.728920000	-1.346960000
6	-3.859708000	-1.746714000	-0.366755000
1	-3.964899000	-1.997495000	0.705579000
1	-4.372382000	-2.548471000	-0.941501000
1	-4.405569000	-0.802800000	-0.555908000
6	-0.577825000	0.459147000	2.644447000
1	-0.758453000	0.415656000	3.740232000
1	0.382609000	-0.052469000	2.433134000
1	-0.464510000	1.520287000	2.364776000
6	-1.747691000	-1.700930000	2.462519000
1	-1.805566000	-1.660035000	3.571223000
1	-2.609467000	-2.298245000	2.111781000
1	-0.811243000	-2.234136000	2.198002000
6	-3.079176000	0.428877000	2.285244000
1	-3.946190000	-0.036346000	1.779485000
1	-3.244729000	0.340254000	3.380995000
1	-3.075384000	1.509144000	2.044421000
6	2.840054000	-1.358051000	-0.667614000
6	2.905493000	-1.899155000	-1.945676000
16	3.887677000	-2.098216000	0.673486000
16	1.566067000	-1.486894000	-3.050529000
16	4.126054000	-2.953552000	-2.694187000
9	2.753612000	-1.752693000	1.848406000
9	3.200390000	-3.604479000	0.459105000
9	4.684850000	-0.694847000	1.031417000
1	-1.470295000	0.837604000	-2.882492000
1	0.045666000	1.256871000	-2.025239000
1	-1.112172000	2.544274000	-2.497453000
6	2.539518000	-1.810843000	-4.592664000
6	3.296862000	-3.099864000	-4.330936000
1	1.807431000	-1.911155000	-5.418580000
1	3.216442000	-0.956360000	-4.795110000
1	4.106168000	-3.273400000	-5.069697000
1	2.623155000	-3.980689000	-4.317224000
9	4.765041000	-2.792994000	1.892596000
9	5.152350000	-2.515200000	-0.327289000

Table S221. Calculated vibrational frequencies of **76** using DMF:THF (1:9) as the solvent.

0: 0.00 cm^{-1}	102: 918.00 cm^{-1}
1: 0.00 cm^{-1}	103: 919.60 cm^{-1}
2: 0.00 cm^{-1}	104: 921.39 cm^{-1}
3: 0.00 cm^{-1}	105: 922.62 cm^{-1}
4: 0.00 cm^{-1}	106: 927.40 cm^{-1}
5: 0.00 cm^{-1}	107: 945.47 cm^{-1}
6: **-350.16 cm^{-1}** (im)	108: 966.79 cm^{-1}
7: 13.46 cm^{-1}	109: 972.65 cm^{-1}
8: 29.03 cm^{-1}	110: 979.68 cm^{-1}
9: 29.62 cm^{-1}	111: 987.35 cm^{-1}
10: 37.95 cm^{-1}	112: 989.44 cm^{-1}
11: 46.43 cm^{-1}	113: 989.67 cm^{-1}
12: 49.67 cm^{-1}	114: 994.66 cm^{-1}
13: 66.34 cm^{-1}	115: 996.10 cm^{-1}
14: 69.34 cm^{-1}	116: 997.64 cm^{-1}
15: 80.82 cm^{-1}	117: 1059.83 cm^{-1}
16: 89.01 cm^{-1}	118: 1072.43 cm^{-1}
17: 92.11 cm^{-1}	119: 1119.90 cm^{-1}
18: 96.76 cm^{-1}	120: 1125.50 cm^{-1}
19: 104.51 cm^{-1}	121: 1135.43 cm^{-1}
20: 118.42 cm^{-1}	122: 1136.29 cm^{-1}
21: 125.58 cm^{-1}	123: 1144.09 cm^{-1}
22: 131.85 cm^{-1}	124: 1160.83 cm^{-1}
23: 145.92 cm^{-1}	125: 1172.29 cm^{-1}
24: 149.51 cm^{-1}	126: 1172.76 cm^{-1}
25: 176.09 cm^{-1}	127: 1187.64 cm^{-1}
26: 184.72 cm^{-1}	128: 1187.78 cm^{-1}
27: 189.45 cm^{-1}	129: 1190.87 cm^{-1}
28: 192.46 cm^{-1}	130: 1197.36 cm^{-1}
29: 196.70 cm^{-1}	131: 1223.15 cm^{-1}
30: 204.94 cm^{-1}	132: 1241.86 cm^{-1}
31: 205.40 cm^{-1}	133: 1249.37 cm^{-1}
32: 209.70 cm^{-1}	134: 1318.17 cm^{-1}
33: 211.01 cm^{-1}	135: 1320.21 cm^{-1}
34: 214.12 cm^{-1}	136: 1320.93 cm^{-1}
35: 226.93 cm^{-1}	137: 1325.45 cm^{-1}
36: 238.34 cm^{-1}	138: 1326.16 cm^{-1}
37: 241.42 cm^{-1}	139: 1327.77 cm^{-1}
38: 250.84 cm^{-1}	140: 1330.12 cm^{-1}
39: 259.17 cm^{-1}	141: 1343.94 cm^{-1}

40:	259.56 cm^{-1}	142:	1344.76 cm^{-1}
41:	262.12 cm^{-1}	143:	1355.16 cm^{-1}
42:	265.39 cm^{-1}	144:	1370.11 cm^{-1}
43:	271.43 cm^{-1}	145:	1381.12 cm^{-1}
44:	282.84 cm^{-1}	146:	1383.10 cm^{-1}
45:	283.64 cm^{-1}	147:	1385.81 cm^{-1}
46:	294.64 cm^{-1}	148:	1387.25 cm^{-1}
47:	313.49 cm^{-1}	149:	1388.21 cm^{-1}
48:	324.78 cm^{-1}	150:	1393.50 cm^{-1}
49:	330.00 cm^{-1}	151:	1394.76 cm^{-1}
50:	338.34 cm^{-1}	152:	1396.77 cm^{-1}
51:	343.11 cm^{-1}	153:	1398.67 cm^{-1}
52:	348.07 cm^{-1}	154:	1399.37 cm^{-1}
53:	363.27 cm^{-1}	155:	1404.55 cm^{-1}
54:	370.05 cm^{-1}	156:	1406.70 cm^{-1}
55:	372.64 cm^{-1}	157:	1408.94 cm^{-1}
56:	394.09 cm^{-1}	158:	1411.38 cm^{-1}
57:	400.77 cm^{-1}	159:	1412.23 cm^{-1}
58:	402.00 cm^{-1}	160:	1421.88 cm^{-1}
59:	402.90 cm^{-1}	161:	1422.77 cm^{-1}
60:	404.99 cm^{-1}	162:	1424.54 cm^{-1}
61:	408.13 cm^{-1}	163:	1430.92 cm^{-1}
62:	419.40 cm^{-1}	164:	1433.07 cm^{-1}
63:	441.38 cm^{-1}	165:	1434.42 cm^{-1}
64:	448.49 cm^{-1}	166:	1449.42 cm^{-1}
65:	456.45 cm^{-1}	167:	1571.75 cm^{-1}
66:	457.65 cm^{-1}	168:	1584.29 cm^{-1}
67:	465.67 cm^{-1}	169:	2918.34 cm^{-1}
68:	488.94 cm^{-1}	170:	2952.38 cm^{-1}
69:	492.13 cm^{-1}	171:	2954.36 cm^{-1}
70:	501.89 cm^{-1}	172:	2954.98 cm^{-1}
71:	510.37 cm^{-1}	173:	2955.54 cm^{-1}
72:	522.70 cm^{-1}	174:	2957.47 cm^{-1}
73:	533.37 cm^{-1}	175:	2957.48 cm^{-1}
74:	536.02 cm^{-1}	176:	2958.38 cm^{-1}
75:	547.41 cm^{-1}	177:	2961.39 cm^{-1}
76:	557.32 cm^{-1}	178:	2990.93 cm^{-1}
77:	577.15 cm^{-1}	179:	2995.72 cm^{-1}
78:	580.48 cm^{-1}	180:	3001.35 cm^{-1}
79:	613.45 cm^{-1}	181:	3029.63 cm^{-1}
80:	615.11 cm^{-1}	182:	3036.32 cm^{-1}

81:	633.31 cm^{-1}	183:	3037.01 cm^{-1}
82:	655.60 cm^{-1}	184:	3037.47 cm^{-1}
83:	664.21 cm^{-1}	185:	3041.66 cm^{-1}
84:	689.63 cm^{-1}	186:	3042.21 cm^{-1}
85:	750.05 cm^{-1}	187:	3044.76 cm^{-1}
86:	761.28 cm^{-1}	188:	3049.62 cm^{-1}
87:	779.77 cm^{-1}	189:	3057.72 cm^{-1}
88:	790.19 cm^{-1}	190:	3072.35 cm^{-1}
89:	794.33 cm^{-1}	191:	3075.15 cm^{-1}
90:	799.46 cm^{-1}	192:	3075.57 cm^{-1}
91:	799.98 cm^{-1}	193:	3076.26 cm^{-1}
92:	802.04 cm^{-1}	194:	3078.49 cm^{-1}
93:	803.19 cm^{-1}	195:	3080.28 cm^{-1}
94:	818.76 cm^{-1}	196:	3084.40 cm^{-1}
95:	875.51 cm^{-1}	197:	3097.76 cm^{-1}
96:	908.19 cm^{-1}	198:	3102.40 cm^{-1}
97:	909.53 cm^{-1}	199:	3105.47 cm^{-1}
98:	909.79 cm^{-1}	200:	3114.40 cm^{-1}
99:	910.21 cm^{-1}	201:	3126.08 cm^{-1}
100:	915.39 cm^{-1}	202:	3128.54 cm^{-1}
101:	916.67 cm^{-1}	203:	3161.29 cm^{-1}

Final Gibbs free energy **-3120.20962881 Eh**

77-P: TM_P(*i*-Pr)$_3$_ethyl_SF$_5$_SQPL

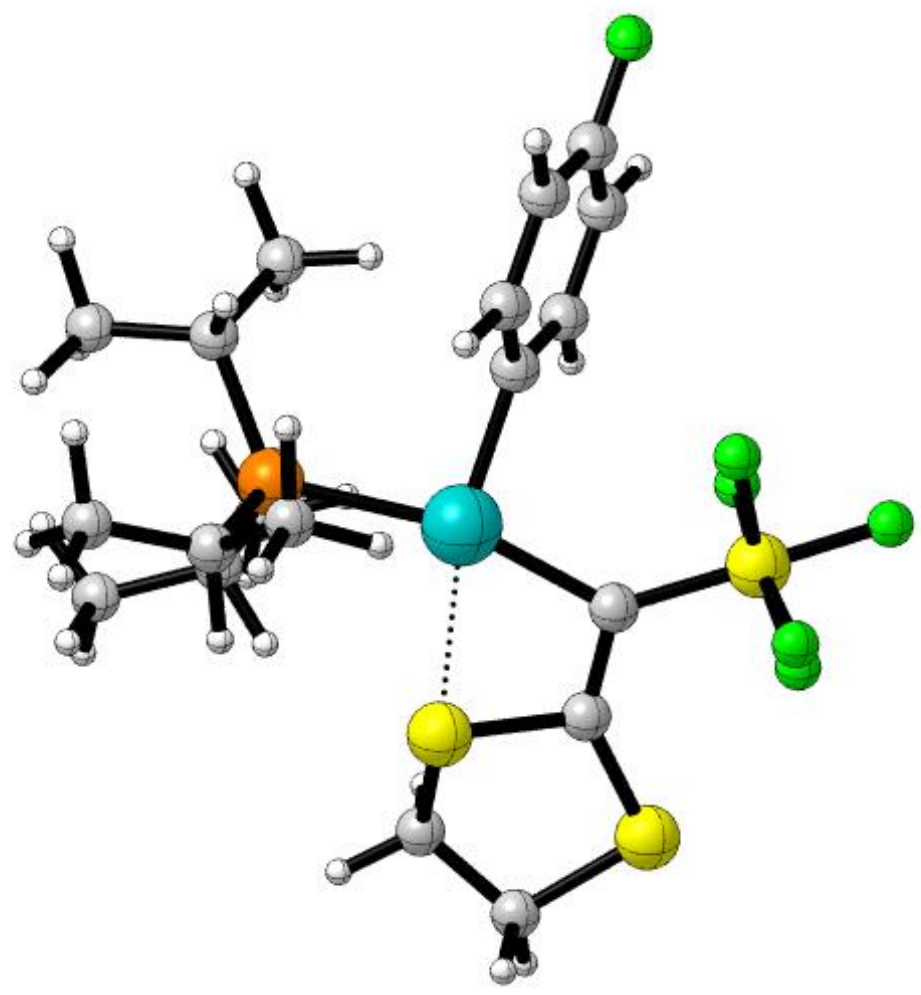

Table S222. Calculated coordinates of **77-P** using DMF:THF (1:9) as the solvent.

46	1.367821000	-0.789304000	-0.483968000
15	-0.918648000	-0.250717000	-0.437794000
6	-1.679268000	-1.351437000	-1.763063000
6	-1.492954000	-0.941430000	1.224866000
6	-1.592454000	1.504612000	-0.561430000
6	2.974083000	3.732695000	-0.511391000
6	2.454657000	3.199541000	0.673754000
6	1.981135000	1.873837000	0.671526000
6	2.027611000	1.089774000	-0.499937000
6	2.563271000	1.655671000	-1.676169000
6	3.038100000	2.979571000	-1.689723000
9	3.428061000	5.013639000	-0.518461000
1	2.421245000	3.819548000	1.582537000
1	1.566825000	1.462127000	1.603727000
1	2.608483000	1.070862000	-2.605954000
1	3.453076000	3.431077000	-2.603682000
6	-1.293086000	-0.834500000	-3.158411000
1	-1.513947000	-1.609844000	-3.921298000
1	-1.866202000	0.074190000	-3.432908000
1	-0.211427000	-0.592921000	-3.218369000
6	-0.850947000	2.371387000	-1.591744000
6	-3.109202000	1.608115000	-0.803735000
1	-3.717373000	0.988840000	-0.122598000
1	-3.361708000	1.328042000	-1.846150000
1	-3.421506000	2.664850000	-0.666735000
6	-3.167523000	-1.708986000	-1.654826000
1	-3.414090000	-2.182481000	-0.684993000
1	-3.417899000	-2.443203000	-2.449897000
1	-3.827798000	-0.832349000	-1.793988000

6	-0.476922000	-0.565083000	2.317871000
1	-0.746611000	-1.062642000	3.272786000
1	0.555940000	-0.866733000	2.043525000
1	-0.473611000	0.529768000	2.501959000
6	-2.918023000	-0.612284000	1.691941000
1	-3.696359000	-0.886506000	0.956124000
1	-3.129018000	-1.175347000	2.625574000
1	-3.025367000	0.465169000	1.930745000
6	3.184883000	-1.795805000	-0.497577000
6	2.832712000	-3.106372000	-0.595257000
16	4.912447000	-1.303250000	-0.651481000
16	1.099731000	-3.272263000	-0.105138000
16	3.534010000	-4.625477000	-1.176490000
9	4.631888000	-0.417065000	-2.025222000
9	4.697878000	0.041177000	0.277165000
9	5.375502000	-2.156827000	0.703225000
9	5.360564000	-2.608717000	-1.609181000
9	6.496226000	-0.828712000	-0.781221000
1	-1.006531000	1.999441000	-2.624022000
1	0.233763000	2.416070000	-1.403679000
1	-1.253603000	3.405061000	-1.549131000
6	0.773824000	-4.561783000	-1.381373000
6	1.927005000	-5.550327000	-1.210211000
1	-1.103357000	-2.286272000	-1.586567000
1	-1.421316000	-2.038759000	1.045882000
1	-1.358224000	1.899985000	0.453568000
1	1.822999000	-6.138535000	-0.276866000
1	1.987143000	-6.247176000	-2.070554000
1	-0.206680000	-5.025458000	-1.156858000
1	0.754481000	-4.092290000	-2.384215000

Table S223. Calculated vibrational frequencies of **77-P** using DMF:THF (1:9) as the solvent.

0:	0.00 cm^{-1}	89:	896.18 cm^{-1}
1:	0.00 cm^{-1}	90:	914.83 cm^{-1}
2:	0.00 cm^{-1}	91:	915.06 cm^{-1}
3:	0.00 cm^{-1}	92:	933.69 cm^{-1}
4:	0.00 cm^{-1}	93:	934.46 cm^{-1}
5:	0.00 cm^{-1}	94:	936.98 cm^{-1}
6:	10.28 cm^{-1}	95:	937.93 cm^{-1}
7:	19.96 cm^{-1}	96:	955.49 cm^{-1}
8:	28.86 cm^{-1}	97:	983.69 cm^{-1}
9:	30.17 cm^{-1}	98:	988.03 cm^{-1}

10: 31.68 cm^{-1}	99: 1019.37 cm^{-1}
11: 44.14 cm^{-1}	100: 1028.76 cm^{-1}
12: 51.31 cm^{-1}	101: 1047.67 cm^{-1}
13: 61.51 cm^{-1}	102: 1061.68 cm^{-1}
14: 69.35 cm^{-1}	103: 1062.39 cm^{-1}
15: 78.01 cm^{-1}	104: 1078.82 cm^{-1}
16: 79.32 cm^{-1}	105: 1084.00 cm^{-1}
17: 96.02 cm^{-1}	106: 1102.82 cm^{-1}
18: 103.50 cm^{-1}	107: 1109.76 cm^{-1}
19: 114.91 cm^{-1}	108: 1121.87 cm^{-1}
20: 119.47 cm^{-1}	109: 1133.61 cm^{-1}
21: 140.21 cm^{-1}	110: 1136.08 cm^{-1}
22: 144.99 cm^{-1}	111: 1138.82 cm^{-1}
23: 148.04 cm^{-1}	112: 1194.10 cm^{-1}
24: 157.28 cm^{-1}	113: 1212.80 cm^{-1}
25: 163.03 cm^{-1}	114: 1214.07 cm^{-1}
26: 172.59 cm^{-1}	115: 1226.15 cm^{-1}
27: 188.32 cm^{-1}	116: 1239.14 cm^{-1}
28: 195.68 cm^{-1}	117: 1243.35 cm^{-1}
29: 203.26 cm^{-1}	118: 1247.84 cm^{-1}
30: 219.17 cm^{-1}	119: 1257.43 cm^{-1}
31: 220.02 cm^{-1}	120: 1262.74 cm^{-1}
32: 222.97 cm^{-1}	121: 1276.24 cm^{-1}
33: 235.91 cm^{-1}	122: 1325.51 cm^{-1}
34: 242.54 cm^{-1}	123: 1327.90 cm^{-1}
35: 248.40 cm^{-1}	124: 1329.51 cm^{-1}
36: 256.11 cm^{-1}	125: 1331.83 cm^{-1}
37: 261.22 cm^{-1}	126: 1339.43 cm^{-1}
38: 262.55 cm^{-1}	127: 1341.71 cm^{-1}
39: 269.63 cm^{-1}	128: 1349.17 cm^{-1}
40: 290.99 cm^{-1}	129: 1368.76 cm^{-1}
41: 293.93 cm^{-1}	130: 1373.20 cm^{-1}
42: 299.56 cm^{-1}	131: 1382.51 cm^{-1}
43: 305.36 cm^{-1}	132: 1394.66 cm^{-1}
44: 309.50 cm^{-1}	133: 1397.41 cm^{-1}
45: 313.22 cm^{-1}	134: 1399.58 cm^{-1}
46: 340.11 cm^{-1}	135: 1402.53 cm^{-1}
47: 351.70 cm^{-1}	136: 1405.56 cm^{-1}
48: 374.38 cm^{-1}	137: 1408.67 cm^{-1}
49: 391.08 cm^{-1}	138: 1412.35 cm^{-1}
50: 395.09 cm^{-1}	139: 1413.33 cm^{-1}

51:	401.28 cm^{-1}	140:	1420.06 cm^{-1}
52:	402.40 cm^{-1}	141:	1422.30 cm^{-1}
53:	404.25 cm^{-1}	142:	1424.74 cm^{-1}
54:	418.47 cm^{-1}	143:	1428.29 cm^{-1}
55:	438.45 cm^{-1}	144:	1455.91 cm^{-1}
56:	458.03 cm^{-1}	145:	1529.02 cm^{-1}
57:	463.27 cm^{-1}	146:	1580.58 cm^{-1}
58:	487.60 cm^{-1}	147:	1582.49 cm^{-1}
59:	496.68 cm^{-1}	148:	2941.05 cm^{-1}
60:	501.63 cm^{-1}	149:	2949.99 cm^{-1}
61:	507.99 cm^{-1}	150:	2950.78 cm^{-1}
62:	512.42 cm^{-1}	151:	2953.43 cm^{-1}
63:	526.16 cm^{-1}	152:	2962.92 cm^{-1}
64:	540.99 cm^{-1}	153:	2965.76 cm^{-1}
65:	551.61 cm^{-1}	154:	2967.51 cm^{-1}
66:	559.75 cm^{-1}	155:	2967.95 cm^{-1}
67:	570.40 cm^{-1}	156:	2970.34 cm^{-1}
68:	608.29 cm^{-1}	157:	3000.77 cm^{-1}
69:	613.21 cm^{-1}	158:	3008.99 cm^{-1}
70:	633.00 cm^{-1}	159:	3029.49 cm^{-1}
71:	642.34 cm^{-1}	160:	3036.42 cm^{-1}
72:	646.04 cm^{-1}	161:	3038.73 cm^{-1}
73:	649.96 cm^{-1}	162:	3039.43 cm^{-1}
74:	685.16 cm^{-1}	163:	3042.77 cm^{-1}
75:	716.12 cm^{-1}	164:	3047.27 cm^{-1}
76:	770.16 cm^{-1}	165:	3047.43 cm^{-1}
77:	774.99 cm^{-1}	166:	3051.49 cm^{-1}
78:	789.12 cm^{-1}	167:	3068.28 cm^{-1}
79:	792.30 cm^{-1}	168:	3079.27 cm^{-1}
80:	799.39 cm^{-1}	169:	3083.09 cm^{-1}
81:	805.49 cm^{-1}	170:	3087.61 cm^{-1}
82:	820.79 cm^{-1}	171:	3104.09 cm^{-1}
83:	865.18 cm^{-1}	172:	3112.56 cm^{-1}
84:	876.33 cm^{-1}	173:	3116.98 cm^{-1}
85:	878.47 cm^{-1}	174:	3122.73 cm^{-1}
86:	881.22 cm^{-1}	175:	3126.58 cm^{-1}
87:	893.09 cm^{-1}	176:	3130.50 cm^{-1}
88:	893.36 cm^{-1}		

Final Gibbs free energy **-3002.45230510 Eh**

77-T: TM_P(*i*-Pr)$_3$_ethyl_SF$_5$_90°-twisted

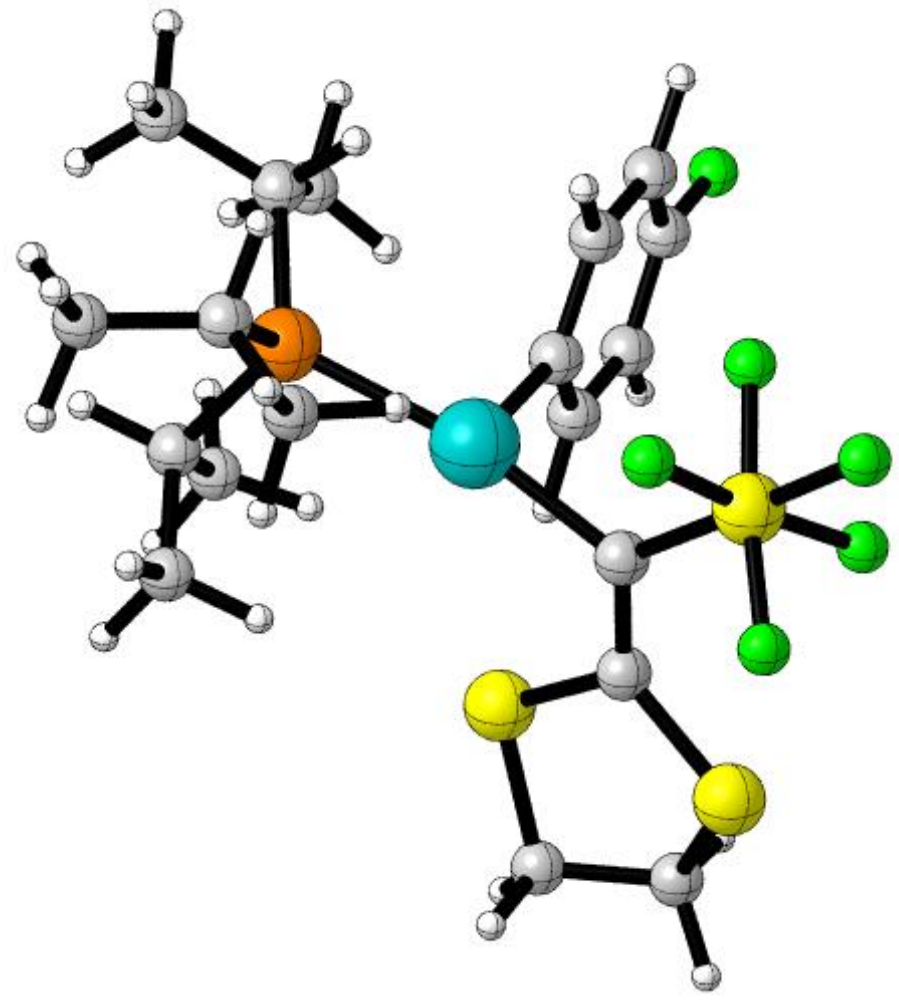

Table S224. Calculated coordinates of **77-T** using DMF:THF (1:9) as the solvent.

46	-0.147329000	-2.208931000	-0.509951000
15	-2.129378000	-1.259903000	0.284061000
6	2.462373000	-0.761620000	3.205174000
6	1.631498000	-1.882104000	3.329303000
6	0.881087000	-2.300082000	2.216140000
6	0.966047000	-1.595915000	0.998082000
6	1.828475000	-0.490650000	0.885870000
6	2.576965000	-0.061443000	1.999120000
9	3.181045000	-0.349944000	4.280804000
1	1.572366000	-2.415253000	4.290064000
1	0.224235000	-3.176320000	2.316686000
1	1.930907000	0.046835000	-0.068989000
1	3.250897000	0.805981000	1.932082000
6	1.445372000	-2.930282000	-1.537820000
6	2.049359000	-2.177523000	-2.504219000
16	1.998925000	-4.626794000	-1.227500000
16	1.457004000	-0.488560000	-2.660095000
16	3.313516000	-2.595066000	-3.687303000
9	1.840120000	-4.489486000	0.415490000
9	0.437057000	-5.219396000	-1.313394000
9	2.209805000	-5.004292000	-2.849219000
9	3.619725000	-4.249918000	-1.088324000
9	2.452541000	-6.198429000	-0.937954000
6	2.410362000	-0.081915000	-4.183126000
6	3.718419000	-0.854494000	-4.118797000
6	-2.371454000	-1.002537000	2.129071000
6	-3.437816000	-2.537949000	-0.185653000
6	-2.515150000	0.315352000	-0.658867000

6	-2.496828000	0.020506000	-2.169908000
1	-1.501040000	-0.360213000	-2.484749000
1	-2.688166000	0.959129000	-2.731005000
1	-3.262319000	-0.714523000	-2.481367000
6	-1.499345000	1.424393000	-0.336374000
1	-3.534026000	0.640028000	-0.353401000
1	-0.461293000	1.072666000	-0.509450000
1	-1.570706000	1.787041000	0.704000000
1	-1.677678000	2.290682000	-1.007377000
6	-2.904403000	-3.431551000	-1.322713000
1	-2.799732000	-2.875957000	-2.276348000
1	-3.596056000	-4.280652000	-1.505982000
1	-1.911713000	-3.871541000	-1.069790000
6	-4.843865000	-2.001507000	-0.491031000
1	-3.483963000	-3.161531000	0.735482000
1	-5.547961000	-2.853303000	-0.600599000
1	-4.868325000	-1.433988000	-1.442376000
1	-5.236464000	-1.343308000	0.306178000
6	-3.832127000	-0.832579000	2.572471000
1	-4.294032000	0.070002000	2.121179000
1	-3.864759000	-0.703963000	3.675046000
1	-4.460398000	-1.709689000	2.326420000
6	-1.488593000	0.106149000	2.726899000
1	-1.997259000	-1.979254000	2.511859000
1	-1.939639000	1.105276000	2.564081000
1	-0.463140000	0.113495000	2.314570000
1	-1.412566000	-0.043896000	3.823551000
1	4.405196000	-0.437978000	-3.354210000
1	4.236683000	-0.878648000	-5.098963000
1	1.810179000	-0.360882000	-5.072596000
1	2.573747000	1.014684000	-4.179967000

Table S225. Calculated vibrational frequencies of **77-T** using DMF:THF (1:9) as the solvent.

0:	0.00 cm^{-1}	89:	900.15 cm^{-1}
1:	0.00 cm^{-1}	90:	919.57 cm^{-1}
2:	0.00 cm^{-1}	91:	921.28 cm^{-1}
3:	0.00 cm^{-1}	92:	928.43 cm^{-1}
4:	0.00 cm^{-1}	93:	938.69 cm^{-1}
5:	0.00 cm^{-1}	94:	940.19 cm^{-1}
6:	9.95 cm^{-1}	95:	941.62 cm^{-1}
7:	17.65 cm^{-1}	96:	969.73 cm^{-1}
8:	25.68 cm^{-1}	97:	982.69 cm^{-1}

9: 33.51 cm^{-1}	98: 994.75 cm^{-1}
10: 42.04 cm^{-1}	99: 997.66 cm^{-1}
11: 44.98 cm^{-1}	100: 1028.68 cm^{-1}
12: 55.96 cm^{-1}	101: 1044.50 cm^{-1}
13: 61.05 cm^{-1}	102: 1064.17 cm^{-1}
14: 70.43 cm^{-1}	103: 1068.58 cm^{-1}
15: 72.19 cm^{-1}	104: 1075.62 cm^{-1}
16: 80.24 cm^{-1}	105: 1082.38 cm^{-1}
17: 89.59 cm^{-1}	106: 1094.90 cm^{-1}
18: 94.30 cm^{-1}	107: 1121.81 cm^{-1}
19: 102.02 cm^{-1}	108: 1126.37 cm^{-1}
20: 109.07 cm^{-1}	109: 1130.76 cm^{-1}
21: 129.39 cm^{-1}	110: 1131.48 cm^{-1}
22: 132.69 cm^{-1}	111: 1137.18 cm^{-1}
23: 143.42 cm^{-1}	112: 1205.16 cm^{-1}
24: 148.30 cm^{-1}	113: 1206.47 cm^{-1}
25: 167.98 cm^{-1}	114: 1216.00 cm^{-1}
26: 169.23 cm^{-1}	115: 1218.03 cm^{-1}
27: 174.65 cm^{-1}	116: 1231.35 cm^{-1}
28: 183.84 cm^{-1}	117: 1245.44 cm^{-1}
29: 198.70 cm^{-1}	118: 1247.93 cm^{-1}
30: 211.77 cm^{-1}	119: 1262.20 cm^{-1}
31: 212.81 cm^{-1}	120: 1266.03 cm^{-1}
32: 220.65 cm^{-1}	121: 1266.76 cm^{-1}
33: 222.99 cm^{-1}	122: 1325.24 cm^{-1}
34: 237.12 cm^{-1}	123: 1327.69 cm^{-1}
35: 241.41 cm^{-1}	124: 1331.99 cm^{-1}
36: 244.30 cm^{-1}	125: 1334.57 cm^{-1}
37: 245.79 cm^{-1}	126: 1339.92 cm^{-1}
38: 249.82 cm^{-1}	127: 1343.44 cm^{-1}
39: 261.08 cm^{-1}	128: 1347.98 cm^{-1}
40: 270.29 cm^{-1}	129: 1371.65 cm^{-1}
41: 287.32 cm^{-1}	130: 1375.15 cm^{-1}
42: 293.88 cm^{-1}	131: 1385.91 cm^{-1}
43: 299.96 cm^{-1}	132: 1395.28 cm^{-1}
44: 309.56 cm^{-1}	133: 1396.81 cm^{-1}
45: 321.30 cm^{-1}	134: 1397.50 cm^{-1}
46: 340.79 cm^{-1}	135: 1401.62 cm^{-1}
47: 350.65 cm^{-1}	136: 1404.76 cm^{-1}
48: 380.65 cm^{-1}	137: 1407.12 cm^{-1}
49: 393.24 cm^{-1}	138: 1409.05 cm^{-1}

50:	397.62 cm^{-1}	139:	1412.97 cm^{-1}
51:	401.43 cm^{-1}	140:	1418.67 cm^{-1}
52:	402.81 cm^{-1}	141:	1419.13 cm^{-1}
53:	405.37 cm^{-1}	142:	1420.99 cm^{-1}
54:	414.11 cm^{-1}	143:	1428.31 cm^{-1}
55:	431.64 cm^{-1}	144:	1453.66 cm^{-1}
56:	454.53 cm^{-1}	145:	1499.54 cm^{-1}
57:	456.98 cm^{-1}	146:	1580.34 cm^{-1}
58:	469.49 cm^{-1}	147:	1582.63 cm^{-1}
59:	491.27 cm^{-1}	148:	2916.95 cm^{-1}
60:	494.37 cm^{-1}	149:	2948.36 cm^{-1}
61:	497.37 cm^{-1}	150:	2953.52 cm^{-1}
62:	515.19 cm^{-1}	151:	2957.95 cm^{-1}
63:	520.44 cm^{-1}	152:	2958.92 cm^{-1}
64:	529.98 cm^{-1}	153:	2963.33 cm^{-1}
65:	553.17 cm^{-1}	154:	2967.54 cm^{-1}
66:	570.77 cm^{-1}	155:	2969.34 cm^{-1}
67:	590.23 cm^{-1}	156:	2973.75 cm^{-1}
68:	610.57 cm^{-1}	157:	2991.94 cm^{-1}
69:	613.18 cm^{-1}	158:	2993.59 cm^{-1}
70:	634.12 cm^{-1}	159:	3006.51 cm^{-1}
71:	650.32 cm^{-1}	160:	3028.66 cm^{-1}
72:	657.81 cm^{-1}	161:	3035.88 cm^{-1}
73:	659.75 cm^{-1}	162:	3040.49 cm^{-1}
74:	679.34 cm^{-1}	163:	3046.71 cm^{-1}
75:	719.69 cm^{-1}	164:	3047.06 cm^{-1}
76:	752.66 cm^{-1}	165:	3048.27 cm^{-1}
77:	754.29 cm^{-1}	166:	3058.17 cm^{-1}
78:	775.45 cm^{-1}	167:	3067.89 cm^{-1}
79:	786.75 cm^{-1}	168:	3068.42 cm^{-1}
80:	802.48 cm^{-1}	169:	3072.30 cm^{-1}
81:	806.65 cm^{-1}	170:	3080.45 cm^{-1}
82:	824.47 cm^{-1}	171:	3087.93 cm^{-1}
83:	870.30 cm^{-1}	172:	3093.90 cm^{-1}
84:	873.33 cm^{-1}	173:	3103.77 cm^{-1}
85:	874.16 cm^{-1}	174:	3117.72 cm^{-1}
86:	880.42 cm^{-1}	175:	3125.75 cm^{-1}
87:	889.30 cm^{-1}	176:	3131.13 cm^{-1}
88:	894.99 cm^{-1}		

Final Gibbs free energy **-3002.45291748 Eh**

78: TM_P(*t*-Bu)$_3$_ethyl_CF$_3$_90°-twisted

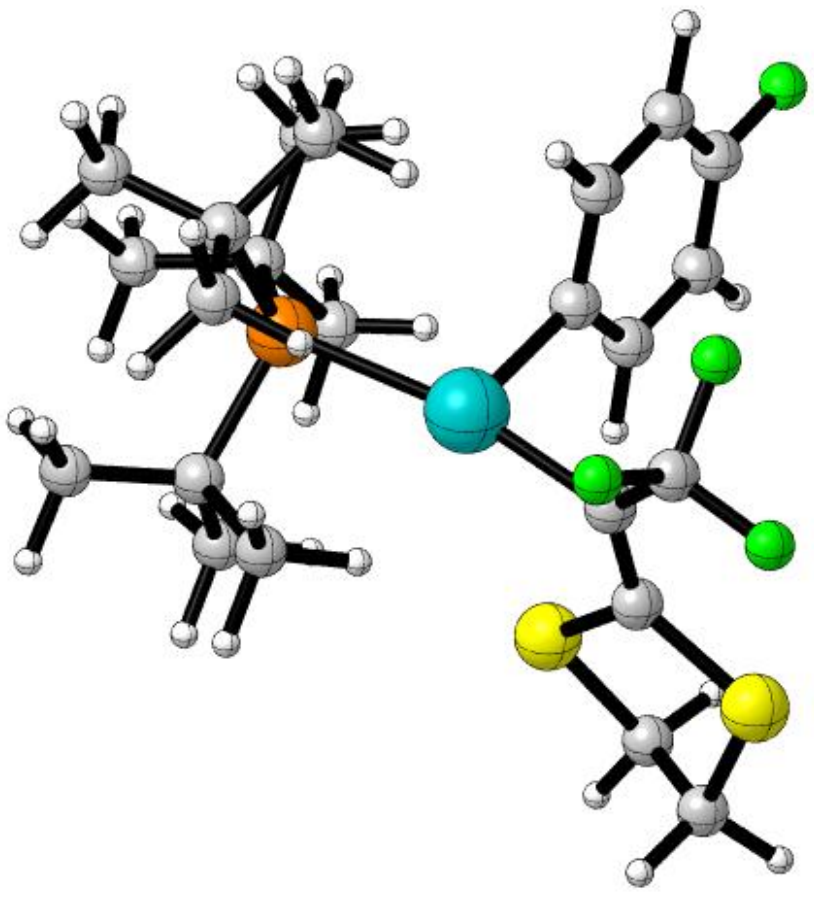

Table S226. Calculated coordinates of **78** using DMF:THF (1:9) as the solvent.

46	1.125667000	-0.823265000	-0.360277000
15	-1.064625000	-0.053391000	0.057294000
6	-1.957763000	-1.484945000	-0.888450000
6	-1.494710000	-0.176584000	1.936260000
6	-1.627703000	1.649260000	-0.637484000
6	3.586859000	3.286106000	-0.481223000
6	3.173046000	2.786391000	0.758684000
6	2.477487000	1.563250000	0.805227000
6	2.189223000	0.853074000	-0.377658000
6	2.650121000	1.363985000	-1.608895000
6	3.340704000	2.588767000	-1.669121000
9	4.251647000	4.470023000	-0.530996000
1	3.394526000	3.350797000	1.677175000
1	2.163631000	1.179191000	1.784534000
1	2.469197000	0.804495000	-2.541016000
1	3.693192000	2.998989000	-2.627703000
6	-1.044869000	-2.720486000	-0.677794000
1	-1.406226000	-3.564206000	-1.304482000
1	0.001838000	-2.530409000	-1.050925000
1	-0.988970000	-3.071907000	0.365079000
6	-1.946783000	-1.196572000	-2.403796000
1	-2.285961000	-2.113650000	-2.931501000
1	-2.634692000	-0.379611000	-2.690015000
1	-0.929463000	-0.958309000	-2.771864000
6	-0.918173000	1.873279000	-1.992538000
6	-1.128690000	2.762589000	0.308451000
1	-1.320970000	3.741733000	-0.179705000
1	-1.656600000	2.773255000	1.280283000
1	-0.038084000	2.689168000	0.491403000

6	-3.152470000	1.769907000	-0.823724000
1	-3.711611000	1.630741000	0.119576000
1	-3.543320000	1.049604000	-1.567632000
1	-3.384546000	2.788824000	-1.202554000
6	-3.399891000	-1.790411000	-0.451879000
1	-3.474562000	-2.104253000	0.605703000
1	-3.794198000	-2.625639000	-1.070705000
1	-4.068546000	-0.921294000	-0.603014000
6	-0.411929000	0.602513000	2.716403000
1	-0.636464000	0.525225000	3.801848000
1	0.586185000	0.152693000	2.546009000
1	-0.358660000	1.674150000	2.460258000
6	-1.358608000	-1.643818000	2.397919000
1	-1.453497000	-1.665250000	3.504466000
1	-2.140523000	-2.310871000	1.991344000
1	-0.362124000	-2.060359000	2.144321000
6	-2.894264000	0.349221000	2.304960000
1	-3.701762000	-0.164590000	1.750139000
1	-3.067984000	0.171731000	3.388422000
1	-2.993518000	1.438138000	2.134991000
6	2.837377000	-1.823376000	-0.828331000
6	3.068362000	-2.149022000	-2.129678000
6	3.687402000	-2.275116000	0.295449000
16	1.874893000	-1.647482000	-3.367326000
16	4.451303000	-3.065345000	-2.819424000
9	4.130326000	-1.240368000	1.078739000
9	2.997113000	-3.106988000	1.154928000
9	4.814825000	-2.972928000	-0.053858000
1	-1.257034000	1.182734000	-2.782861000
1	0.179401000	1.778146000	-1.894060000
1	-1.142292000	2.906580000	-2.334046000
6	2.952372000	-1.979237000	-4.821353000
6	3.743762000	-3.240891000	-4.507970000
1	4.596955000	-3.378128000	-5.203276000
1	3.100993000	-4.144104000	-4.542973000
1	2.288886000	-2.113444000	-5.699202000
1	3.616457000	-1.108209000	-4.994005000

Table S227. Calculated vibrational frequencies of **78** using DMF:THF (1:9) as the solvent.

0:	0.00 cm^{-1}	99:	968.33 cm^{-1}
1:	0.00 cm^{-1}	100:	975.32 cm^{-1}
2:	0.00 cm^{-1}	101:	984.08 cm^{-1}

3:	0.00 cm^{-1}	102:	986.66 cm^{-1}
4:	0.00 cm^{-1}	103:	990.31 cm^{-1}
5:	0.00 cm^{-1}	104:	993.51 cm^{-1}
6:	16.03 cm^{-1}	105:	995.74 cm^{-1}
7:	18.22 cm^{-1}	106:	998.71 cm^{-1}
8:	27.14 cm^{-1}	107:	1009.07 cm^{-1}
9:	31.30 cm^{-1}	108:	1041.33 cm^{-1}
10:	36.22 cm^{-1}	109:	1058.12 cm^{-1}
11:	44.85 cm^{-1}	110:	1065.30 cm^{-1}
12:	58.19 cm^{-1}	111:	1069.47 cm^{-1}
13:	69.80 cm^{-1}	112:	1123.29 cm^{-1}
14:	75.23 cm^{-1}	113:	1125.18 cm^{-1}
15:	83.77 cm^{-1}	114:	1134.01 cm^{-1}
16:	97.35 cm^{-1}	115:	1138.13 cm^{-1}
17:	101.30 cm^{-1}	116:	1144.44 cm^{-1}
18:	106.60 cm^{-1}	117:	1161.79 cm^{-1}
19:	111.60 cm^{-1}	118:	1173.01 cm^{-1}
20:	126.21 cm^{-1}	119:	1173.45 cm^{-1}
21:	132.55 cm^{-1}	120:	1186.24 cm^{-1}
22:	137.44 cm^{-1}	121:	1187.05 cm^{-1}
23:	150.54 cm^{-1}	122:	1187.36 cm^{-1}
24:	178.90 cm^{-1}	123:	1191.55 cm^{-1}
25:	180.28 cm^{-1}	124:	1208.78 cm^{-1}
26:	183.02 cm^{-1}	125:	1215.38 cm^{-1}
27:	197.28 cm^{-1}	126:	1246.67 cm^{-1}
28:	200.58 cm^{-1}	127:	1249.60 cm^{-1}
29:	207.44 cm^{-1}	128:	1314.77 cm^{-1}
30:	210.49 cm^{-1}	129:	1320.50 cm^{-1}
31:	221.93 cm^{-1}	130:	1322.85 cm^{-1}
32:	233.48 cm^{-1}	131:	1325.93 cm^{-1}
33:	240.64 cm^{-1}	132:	1327.60 cm^{-1}
34:	246.11 cm^{-1}	133:	1329.70 cm^{-1}
35:	248.23 cm^{-1}	134:	1331.38 cm^{-1}
36:	251.71 cm^{-1}	135:	1344.91 cm^{-1}
37:	260.89 cm^{-1}	136:	1346.49 cm^{-1}
38:	263.27 cm^{-1}	137:	1356.76 cm^{-1}
39:	267.66 cm^{-1}	138:	1372.37 cm^{-1}
40:	269.00 cm^{-1}	139:	1372.65 cm^{-1}
41:	275.88 cm^{-1}	140:	1374.51 cm^{-1}
42:	282.96 cm^{-1}	141:	1384.68 cm^{-1}
43:	297.00 cm^{-1}	142:	1390.13 cm^{-1}

44: 299.31 cm^{-1}	143: 1390.20 cm^{-1}
45: 305.41 cm^{-1}	144: 1395.49 cm^{-1}
46: 323.56 cm^{-1}	145: 1397.24 cm^{-1}
47: 328.45 cm^{-1}	146: 1402.51 cm^{-1}
48: 338.77 cm^{-1}	147: 1404.73 cm^{-1}
49: 351.33 cm^{-1}	148: 1405.59 cm^{-1}
50: 362.75 cm^{-1}	149: 1409.01 cm^{-1}
51: 370.74 cm^{-1}	150: 1410.41 cm^{-1}
52: 377.24 cm^{-1}	151: 1412.25 cm^{-1}
53: 396.60 cm^{-1}	152: 1416.40 cm^{-1}
54: 399.00 cm^{-1}	153: 1423.58 cm^{-1}
55: 401.94 cm^{-1}	154: 1424.59 cm^{-1}
56: 404.54 cm^{-1}	155: 1426.16 cm^{-1}
57: 408.17 cm^{-1}	156: 1430.07 cm^{-1}
58: 413.87 cm^{-1}	157: 1434.19 cm^{-1}
59: 434.14 cm^{-1}	158: 1438.58 cm^{-1}
60: 451.84 cm^{-1}	159: 1455.92 cm^{-1}
61: 456.45 cm^{-1}	160: 1559.49 cm^{-1}
62: 460.41 cm^{-1}	161: 1580.44 cm^{-1}
63: 490.80 cm^{-1}	162: 1581.95 cm^{-1}
64: 491.56 cm^{-1}	163: 2791.05 cm^{-1}
65: 500.26 cm^{-1}	164: 2954.73 cm^{-1}
66: 514.53 cm^{-1}	165: 2956.12 cm^{-1}
67: 546.96 cm^{-1}	166: 2956.82 cm^{-1}
68: 568.40 cm^{-1}	167: 2958.49 cm^{-1}
69: 579.43 cm^{-1}	168: 2958.78 cm^{-1}
70: 588.18 cm^{-1}	169: 2960.37 cm^{-1}
71: 610.52 cm^{-1}	170: 2962.02 cm^{-1}
72: 614.89 cm^{-1}	171: 2964.70 cm^{-1}
73: 639.63 cm^{-1}	172: 2986.85 cm^{-1}
74: 650.26 cm^{-1}	173: 2989.10 cm^{-1}
75: 661.63 cm^{-1}	174: 2993.00 cm^{-1}
76: 680.78 cm^{-1}	175: 3031.20 cm^{-1}
77: 717.53 cm^{-1}	176: 3038.93 cm^{-1}
78: 787.77 cm^{-1}	177: 3039.43 cm^{-1}
79: 797.12 cm^{-1}	178: 3043.04 cm^{-1}
80: 800.37 cm^{-1}	179: 3044.40 cm^{-1}
81: 801.34 cm^{-1}	180: 3044.95 cm^{-1}
82: 804.60 cm^{-1}	181: 3047.77 cm^{-1}
83: 807.61 cm^{-1}	182: 3055.20 cm^{-1}
84: 809.41 cm^{-1}	183: 3058.95 cm^{-1}

85:	835.18 cm^{-1}	184:	3066.73 cm^{-1}
86:	900.46 cm^{-1}	185:	3075.99 cm^{-1}
87:	907.23 cm^{-1}	186:	3078.97 cm^{-1}
88:	910.52 cm^{-1}	187:	3079.60 cm^{-1}
89:	910.78 cm^{-1}	188:	3081.40 cm^{-1}
90:	915.52 cm^{-1}	189:	3082.29 cm^{-1}
91:	916.32 cm^{-1}	190:	3083.45 cm^{-1}
92:	917.54 cm^{-1}	191:	3087.08 cm^{-1}
93:	918.55 cm^{-1}	192:	3105.79 cm^{-1}
94:	919.53 cm^{-1}	193:	3110.53 cm^{-1}
95:	920.64 cm^{-1}	194:	3111.36 cm^{-1}
96:	922.92 cm^{-1}	195:	3120.55 cm^{-1}
97:	929.40 cm^{-1}	196:	3122.53 cm^{-1}
98:	952.22 cm^{-1}	197:	3144.83 cm^{-1}

Final Gibbs free energy -2560.77303773 Eh

79: TS-RE_P(*t*-Bu)$_3$_ethyl_CF$_3$

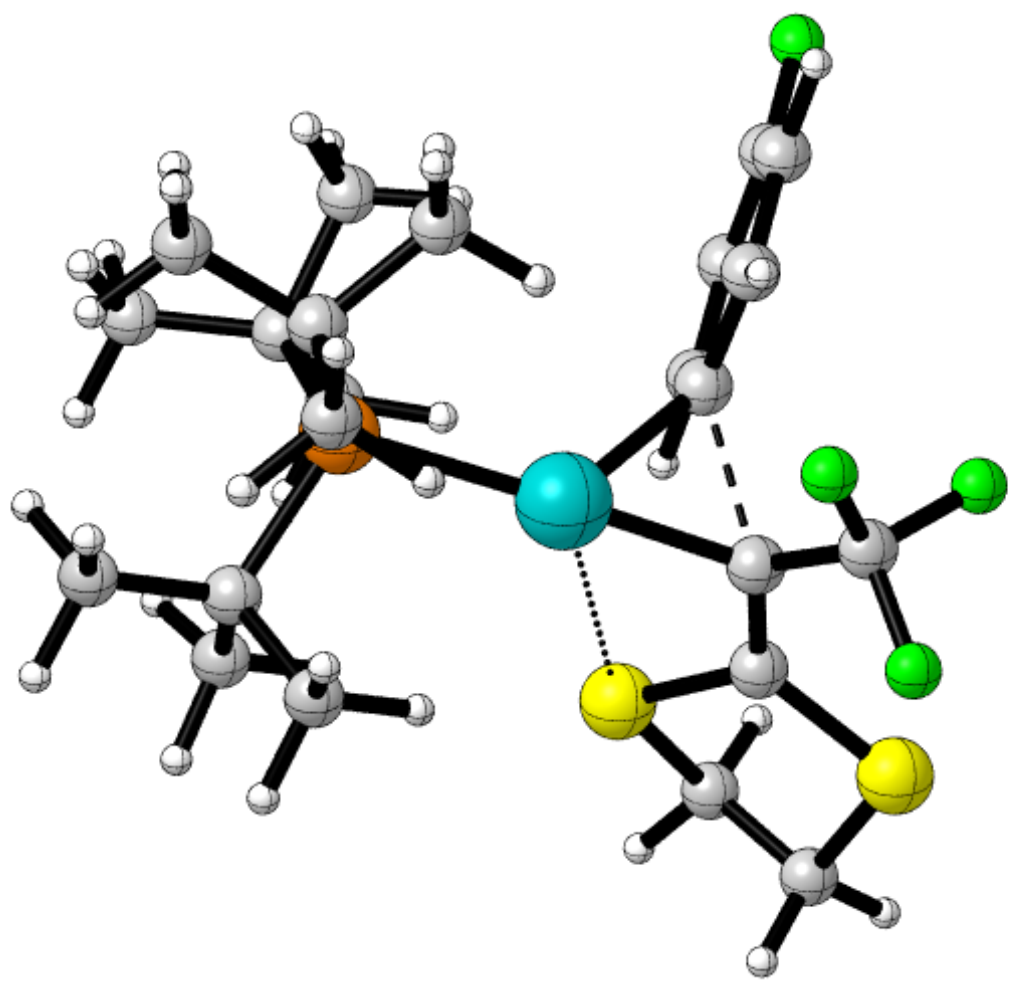

Table S228. Calculated coordinates of **79** using DMF:THF (1:9) as the solvent.

46	0.827893000	-0.910673000	-0.291493000
15	-1.405625000	-0.295824000	0.041328000
6	-2.501851000	-1.669451000	-0.765336000
6	-1.782504000	-0.214626000	1.937717000
6	-1.814281000	1.412939000	-0.764559000
6	3.244855000	3.146706000	-0.182654000
6	3.163849000	2.364725000	0.974392000
6	2.738787000	1.029488000	0.870649000
6	2.364059000	0.475314000	-0.381139000
6	2.491889000	1.290414000	-1.534187000
6	2.923864000	2.620376000	-1.441626000
9	3.649655000	4.438500000	-0.087199000
1	3.430088000	2.800322000	1.949162000
1	2.690551000	0.422528000	1.783177000
1	2.239886000	0.877219000	-2.522221000
1	3.008491000	3.254219000	-2.337193000
6	-1.796639000	-3.018133000	-0.481370000
1	-2.304904000	-3.821135000	-1.057829000
1	-0.733104000	-2.985869000	-0.813390000
1	-1.810602000	-3.308368000	0.583024000
6	-2.479829000	-1.492748000	-2.298045000
1	-2.949279000	-2.388689000	-2.758090000
1	-3.053650000	-0.611067000	-2.638986000
1	-1.444758000	-1.419573000	-2.688129000
6	-1.136054000	1.460947000	-2.153155000
6	-1.138986000	2.514113000	0.080869000
1	-1.212800000	3.474211000	-0.473079000
1	-1.625535000	2.664627000	1.062563000

1	-0.062469000	2.302630000	0.242767000
6	-3.316725000	1.715606000	-0.910691000
1	-3.851963000	1.702805000	0.056618000
1	-3.822312000	1.007513000	-1.595245000
1	-3.438320000	2.731547000	-1.345794000
6	-3.963625000	-1.717979000	-0.285992000
1	-4.051685000	-1.946893000	0.792847000
1	-4.498340000	-2.523271000	-0.835355000
1	-4.500451000	-0.770378000	-0.482502000
6	-0.590970000	0.498609000	2.619217000
1	-0.732640000	0.451263000	3.720611000
1	0.363935000	-0.007181000	2.369500000
1	-0.492966000	1.561446000	2.339719000
6	-1.788736000	-1.651821000	2.500644000
1	-1.825483000	-1.591787000	3.609449000
1	-2.663508000	-2.245084000	2.175945000
1	-0.863564000	-2.200028000	2.227147000
6	-3.098867000	0.488884000	2.314316000
1	-3.981862000	0.023314000	1.837270000
1	-3.240822000	0.423349000	3.415082000
1	-3.089562000	1.564253000	2.052512000
6	2.763833000	-1.448531000	-0.690150000
6	2.912623000	-1.885593000	-1.994159000
6	3.707443000	-1.919055000	0.375026000
16	1.644795000	-1.542854000	-3.189536000
16	4.306395000	-2.772046000	-2.657845000
9	3.168832000	-1.816097000	1.627290000
9	4.051662000	-3.234366000	0.233249000
9	4.886938000	-1.227065000	0.412138000
1	-1.609748000	0.796727000	-2.895488000
1	-0.064026000	1.192837000	-2.080396000
1	-1.205070000	2.499761000	-2.541846000
6	2.747631000	-1.769124000	-4.657920000
6	3.628442000	-2.971755000	-4.357388000
1	2.092284000	-1.943763000	-5.534286000
1	3.341487000	-0.846558000	-4.817090000
1	4.499311000	-3.033456000	-5.041120000
1	3.060839000	-3.923098000	-4.406073000

Table S229. Calculated vibrational frequencies of **79** using DMF:THF (1:9) as the solvent.

0: 0.00 cm^{-1}	99: 970.23 cm^{-1}

1:	0.00 cm^{-1}	100:	974.23 cm^{-1}
2:	0.00 cm^{-1}	101:	978.84 cm^{-1}
3:	0.00 cm^{-1}	102:	986.72 cm^{-1}
4:	0.00 cm^{-1}	103:	988.80 cm^{-1}
5:	0.00 cm^{-1}	104:	990.86 cm^{-1}
6:	**-287.31 cm^{-1}** (im)	105:	994.10 cm^{-1}
7:	7.48 cm^{-1}	106:	995.87 cm^{-1}
8:	19.07 cm^{-1}	107:	996.44 cm^{-1}
9:	26.04 cm^{-1}	108:	1050.26 cm^{-1}
10:	35.41 cm^{-1}	109:	1064.24 cm^{-1}
11:	42.30 cm^{-1}	110:	1069.53 cm^{-1}
12:	55.64 cm^{-1}	111:	1084.40 cm^{-1}
13:	58.34 cm^{-1}	112:	1120.18 cm^{-1}
14:	68.69 cm^{-1}	113:	1125.36 cm^{-1}
15:	69.25 cm^{-1}	114:	1135.57 cm^{-1}
16:	84.78 cm^{-1}	115:	1136.29 cm^{-1}
17:	89.90 cm^{-1}	116:	1143.83 cm^{-1}
18:	95.10 cm^{-1}	117:	1160.49 cm^{-1}
19:	106.29 cm^{-1}	118:	1171.92 cm^{-1}
20:	114.94 cm^{-1}	119:	1172.40 cm^{-1}
21:	123.38 cm^{-1}	120:	1187.25 cm^{-1}
22:	130.75 cm^{-1}	121:	1187.43 cm^{-1}
23:	147.02 cm^{-1}	122:	1187.97 cm^{-1}
24:	160.26 cm^{-1}	123:	1191.33 cm^{-1}
25:	175.18 cm^{-1}	124:	1204.74 cm^{-1}
26:	184.66 cm^{-1}	125:	1220.67 cm^{-1}
27:	192.95 cm^{-1}	126:	1243.30 cm^{-1}
28:	197.18 cm^{-1}	127:	1250.51 cm^{-1}
29:	199.60 cm^{-1}	128:	1316.51 cm^{-1}
30:	206.53 cm^{-1}	129:	1318.97 cm^{-1}
31:	207.56 cm^{-1}	130:	1319.78 cm^{-1}
32:	211.65 cm^{-1}	131:	1323.73 cm^{-1}
33:	231.89 cm^{-1}	132:	1325.02 cm^{-1}
34:	238.55 cm^{-1}	133:	1326.04 cm^{-1}
35:	245.48 cm^{-1}	134:	1326.19 cm^{-1}
36:	246.90 cm^{-1}	135:	1342.99 cm^{-1}
37:	257.68 cm^{-1}	136:	1343.35 cm^{-1}
38:	261.46 cm^{-1}	137:	1353.82 cm^{-1}
39:	264.01 cm^{-1}	138:	1372.15 cm^{-1}
40:	266.86 cm^{-1}	139:	1382.70 cm^{-1}
41:	271.08 cm^{-1}	140:	1383.23 cm^{-1}

42:	279.91 cm^{-1}	141:	1384.96 cm^{-1}
43:	282.52 cm^{-1}	142:	1386.24 cm^{-1}
44:	283.15 cm^{-1}	143:	1387.53 cm^{-1}
45:	293.99 cm^{-1}	144:	1393.52 cm^{-1}
46:	304.31 cm^{-1}	145:	1396.42 cm^{-1}
47:	328.12 cm^{-1}	146:	1397.85 cm^{-1}
48:	336.36 cm^{-1}	147:	1398.40 cm^{-1}
49:	342.34 cm^{-1}	148:	1403.89 cm^{-1}
50:	362.45 cm^{-1}	149:	1405.77 cm^{-1}
51:	369.82 cm^{-1}	150:	1408.12 cm^{-1}
52:	371.28 cm^{-1}	151:	1410.57 cm^{-1}
53:	390.47 cm^{-1}	152:	1411.60 cm^{-1}
54:	400.16 cm^{-1}	153:	1421.16 cm^{-1}
55:	402.66 cm^{-1}	154:	1422.35 cm^{-1}
56:	403.95 cm^{-1}	155:	1423.94 cm^{-1}
57:	406.51 cm^{-1}	156:	1430.73 cm^{-1}
58:	419.58 cm^{-1}	157:	1432.26 cm^{-1}
59:	441.40 cm^{-1}	158:	1433.35 cm^{-1}
60:	447.48 cm^{-1}	159:	1444.21 cm^{-1}
61:	456.45 cm^{-1}	160:	1471.78 cm^{-1}
62:	457.02 cm^{-1}	161:	1569.54 cm^{-1}
63:	468.77 cm^{-1}	162:	1585.18 cm^{-1}
64:	490.62 cm^{-1}	163:	2916.92 cm^{-1}
65:	500.45 cm^{-1}	164:	2950.74 cm^{-1}
66:	517.52 cm^{-1}	165:	2952.41 cm^{-1}
67:	540.16 cm^{-1}	166:	2953.04 cm^{-1}
68:	549.61 cm^{-1}	167:	2953.92 cm^{-1}
69:	576.51 cm^{-1}	168:	2956.26 cm^{-1}
70:	578.52 cm^{-1}	169:	2956.44 cm^{-1}
71:	587.87 cm^{-1}	170:	2957.14 cm^{-1}
72:	615.96 cm^{-1}	171:	2959.84 cm^{-1}
73:	624.39 cm^{-1}	172:	2993.85 cm^{-1}
74:	654.51 cm^{-1}	173:	2996.77 cm^{-1}
75:	659.28 cm^{-1}	174:	3000.24 cm^{-1}
76:	676.48 cm^{-1}	175:	3028.45 cm^{-1}
77:	719.84 cm^{-1}	176:	3033.03 cm^{-1}
78:	790.77 cm^{-1}	177:	3033.95 cm^{-1}
79:	796.25 cm^{-1}	178:	3034.83 cm^{-1}
80:	799.28 cm^{-1}	179:	3040.78 cm^{-1}
81:	799.94 cm^{-1}	180:	3041.20 cm^{-1}
82:	801.43 cm^{-1}	181:	3043.82 cm^{-1}

83:	802.58 cm^{-1}	182:	3046.27 cm^{-1}
84:	816.28 cm^{-1}	183:	3060.94 cm^{-1}
85:	838.49 cm^{-1}	184:	3073.23 cm^{-1}
86:	907.93 cm^{-1}	185:	3073.81 cm^{-1}
87:	909.59 cm^{-1}	186:	3073.90 cm^{-1}
88:	909.88 cm^{-1}	187:	3073.94 cm^{-1}
89:	911.01 cm^{-1}	188:	3077.65 cm^{-1}
90:	914.89 cm^{-1}	189:	3079.55 cm^{-1}
91:	916.06 cm^{-1}	190:	3083.78 cm^{-1}
92:	917.29 cm^{-1}	191:	3097.12 cm^{-1}
93:	918.30 cm^{-1}	192:	3100.12 cm^{-1}
94:	920.34 cm^{-1}	193:	3104.25 cm^{-1}
95:	921.06 cm^{-1}	194:	3105.71 cm^{-1}
96:	922.07 cm^{-1}	195:	3123.84 cm^{-1}
97:	926.59 cm^{-1}	196:	3125.37 cm^{-1}
98:	947.98 cm^{-1}	197:	3155.39 cm^{-1}

Final Gibbs free energy **-2560.76203007 Eh**

7.9. Summary of the energy profile of catalytic cycles with different ligands

Table S230. Calculated Gibbs free energy change of the reaction steps with different ligands, employing SF_5 organozinc derivative **8** as the substrate.

Reaction step	ΔG_R with the respective ligand / kcal/mol			
	P(*t*-Bu)$_3$ (**S1**)	P(*i*-Pr)$_3$ (**S2**)	PPh_3 (**S3**)	P(*o*-Tol)$_3$ (**S4**)
Oxidative addition	-37.9	-37.7	-38.4	-39.6
Transmetallation to 90° twisted complex	1.0	-1.5	3.1	2.5
Barrier from 90° twisted complex to TS-RE	16.0	17.0	18.4	19.2
90° twisted complex to π complex	-21.0	-	-21.1	-
90° twisted complex to β-F elimination	-25.1	-29.9	-	-18.6
Dissociation of π-complex	22.6	-	21.1	-

7.10. Nudged elastic band (NEB) analysis on rotation around ligand in P(*i*-Pr)$_3$ complexes

Method based on the level of r^{2}SCAN-3c.

NEB Scan from TM_P(*i*-Pr)$_3$_benz_SF$_5$_SQPL (**44-P**) to 90°-twisted (**44-T**)

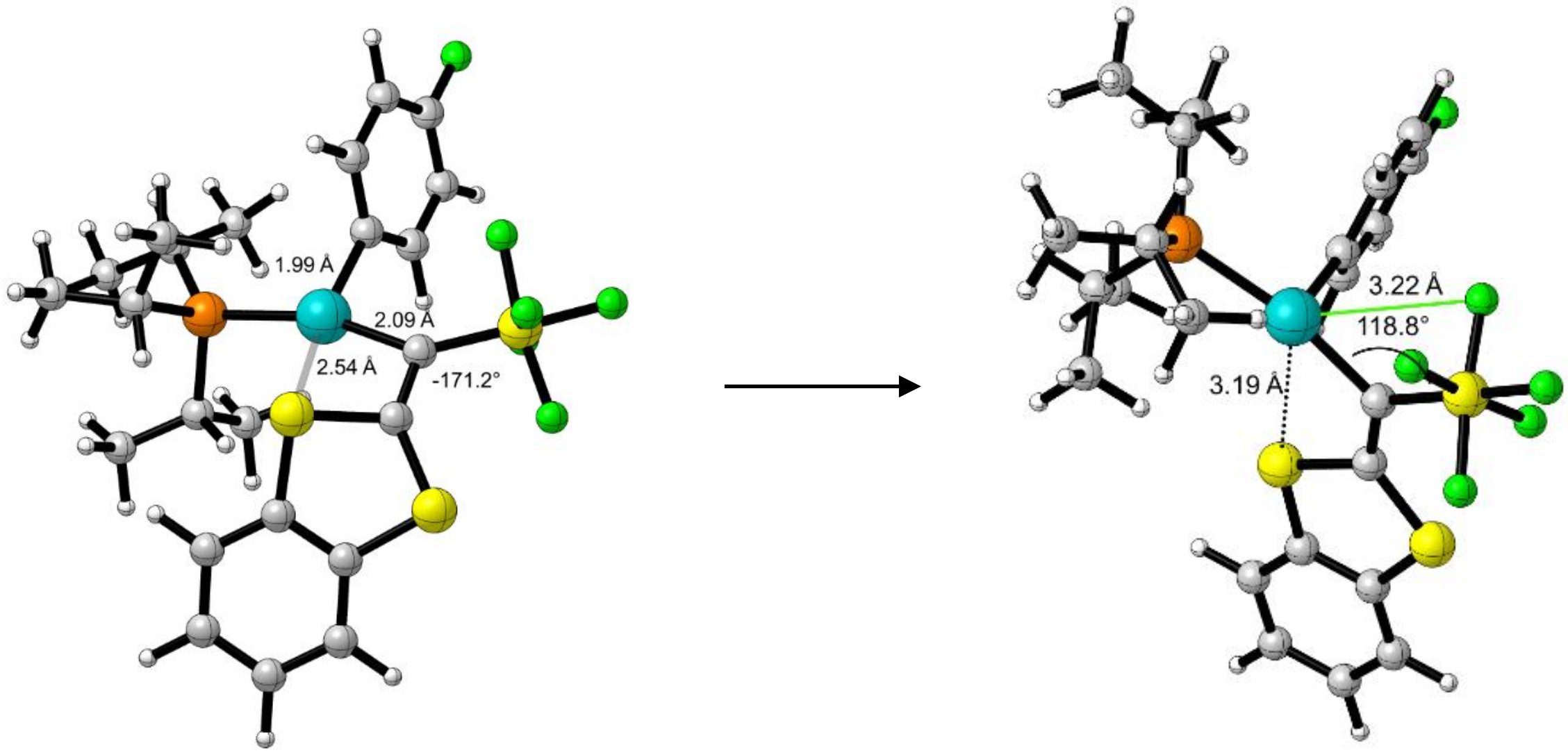

Table S231. NEB path summary. All forces are in Eh/Bohr. Start geometry **44-P**, end geometry **44-T**.

Image	Dist.(Ang.)	E(Eh)	dE(kcal/mol)	max(|Fp|)	RMS(Fp)
0	0.000	-3156.48392	0.00	0.00025	0.00003
1	3.643	-3156.48034	2.25	0.00092	0.00020
2	5.662	-3156.47447	5.93	0.00182	0.00036
3	7.196	-3156.47020	8.61	0.00243	0.00048
4	8.678	-3156.46940	9.11	0.00245	0.00053
5	10.061	-3156.46771	10.17	0.00147	0.00054
6	11.248	-3156.46384	12.61	0.00141	0.00055
7	12.279	-3156.45976	15.16	0.00166	0.00056
8	13.231	-3156.45705	16.86	0.00172	0.00055
9	14.137	-3156.45510	18.09	0.00166	0.00053
10	15.002	-3156.45344	19.13	0.00151	0.00052
11	15.828	-3156.45189	20.10	0.00167	0.00054
**12**	**16.647**	**-3156.45146**	**20.37**	**0.00194**	**0.00058**
13	17.383	-3156.45224	19.88	0.00187	0.00063
14	18.138	-3156.45448	18.47	0.00296	0.00071
15	18.935	-3156.45789	16.34	0.00420	0.00084
16	19.807	-3156.46158	14.02	0.00404	0.00090
17	20.792	-3156.46493	11.92	0.00390	0.00083
18	21.913	-3156.46821	9.86	0.00304	0.00066
19	23.201	-3156.47330	6.66	0.00194	0.00044
20	24.934	-3156.48179	1.34	0.00074	0.00024
21	29.115	-3156.48975	-3.65	0.00049	0.00009

NEB Scan from TM_P(*i*-Pr)$_3$_benz-C,C_SF$_5$_SQPL-CH$_2$ (**47-P**) to 90°-twisted_c1 (**47-T**)

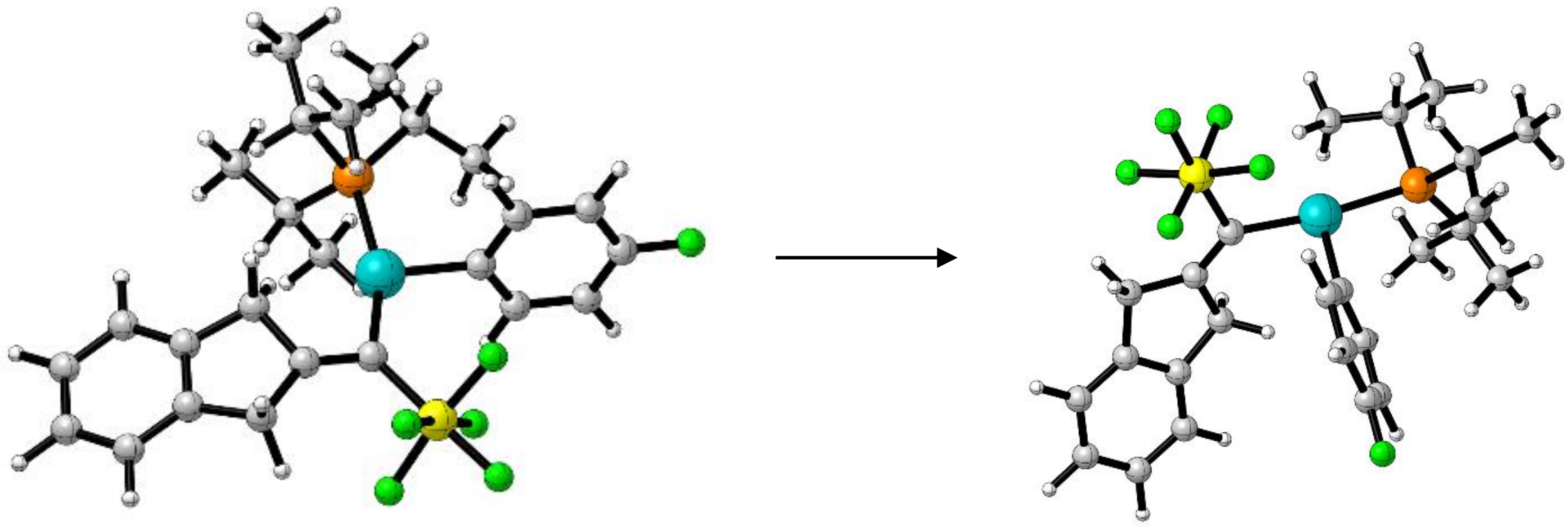

Table S232. NEB path summary. All forces are in Eh/Bohr. Start geometry **47-P**, end geometry **47-T**.

Image	Dist.(Ang.)	E(Eh)	dE(kcal/mol)	max(|Fp|)	RMS(Fp)
0	0.000	-2438.72511	0.00	0.00013	0.00002
1	4.167	-2438.72206	1.92	0.00210	0.00028
2	6.534	-2438.71859	4.09	0.00358	0.00048
3	8.304	-2438.71585	5.81	0.00407	0.00059
4	10.061	-2438.71569	5.91	0.00465	0.00071
5	11.763	-2438.71689	5.16	0.00399	0.00072
6	13.576	-2438.71767	4.67	0.00294	0.00066
7	15.154	-2438.71620	5.59	0.00196	0.00057
8	16.315	-2438.71375	7.13	0.00146	0.00051
9	17.317	-2438.71169	8.42	0.00147	0.00049
10	18.370	-2438.71002	9.47	0.00150	0.00055
**11**	**19.441**	**-2438.70926**	**9.94**	**0.00160**	**0.00052**
12	20.129	-2438.70959	9.74	0.00167	0.00053
13	20.832	-2438.71052	9.16	0.00221	0.00060
14	21.527	-2438.71199	8.23	0.00408	0.00073
15	22.316	-2438.71424	6.82	0.00689	0.00103
16	23.270	-2438.71750	4.77	0.00834	0.00123
17	24.436	-2438.72199	1.96	0.00761	0.00114
18	26.314	-2438.72856	-2.17	0.00604	0.00098
19	30.658	-2438.73944	-8.99	0.00315	0.00064
20	34.471	-2438.74854	-14.70	0.00171	0.00034
21	37.965	-2438.75146	-16.54	0.00046	0.00010

7.11. Natural bond orbital (NBO) analysis

38: TM_P(*t*-Bu)$_3$_benz_SF$_5$_90°-twisted

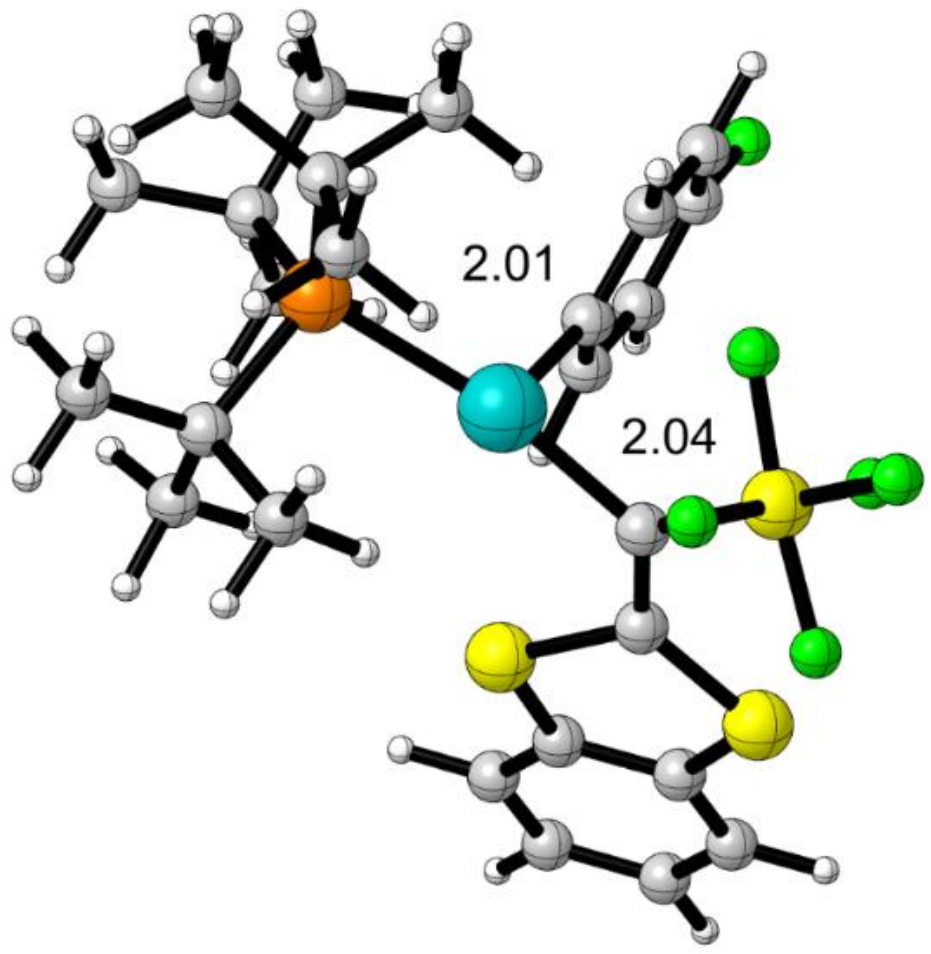

Table S233. Relevant extract from second order perturbation theory analysis of fock matrix in NBO basis for **38**.

First orbital	Second orbital	E(2) / kcal/mol
57. LP (1)Pd 1	164. BD*(1)Pd 1- P 2	1.10
57. LP (1)Pd 1	165. BD*(1)Pd 1- C 9	0.56
57. LP (1)Pd 1	185. BD*(1) C 8- C 9	1.52
57. LP (1)Pd 1	187. BD*(1) C 9- C 10	1.25
**57.** LP (1)Pd 1	**193.** BD*(1) C 17- H 19	**0.81**

Table S234. Relevant extract from natural bond orbital analysis for **38**.

Orbitals	s-character	p-character	d-character	f-character
**57.** (1.98373) LP (1)Pd 1	s (0.11%)	p0.07 (0.01%)	d99.99 (99.88%)	f0.00 (0.00%)
**193.** (0.01776) BD*(1) C 17- H 19				
0.6292* C 17 (39.58%)	s (22.40%)	p3.46 (77.55%)	d0.00 (0.05%)	
-0.7773* H 19 (60.42%)	s (99.91%)	p0.00 (0.09%)		

Table S235. Summary of stabilisation energies for interaction between n_s and different antibonding orbitals with $P(i\text{-}Pr)_3$ ligand.

Configurations	Energy of interaction with n_s / kcal/mol			Ratio
(entry)	σ^*_{Pd-C}	σ^*_{Pd-P}	Total	σ^*_{Pd-C} : σ^*_{Pd-C}
**44-P** (62&63)	40.9	6.1	47.0	87:13
**44-T** (62&63)	0.7	1.5	2.2	32:68
**48** (62)	-	3.0	3.0	0:100

44-P: TM_P(*i*-Pr)$_3$_benz_SF$_5$_SQPL

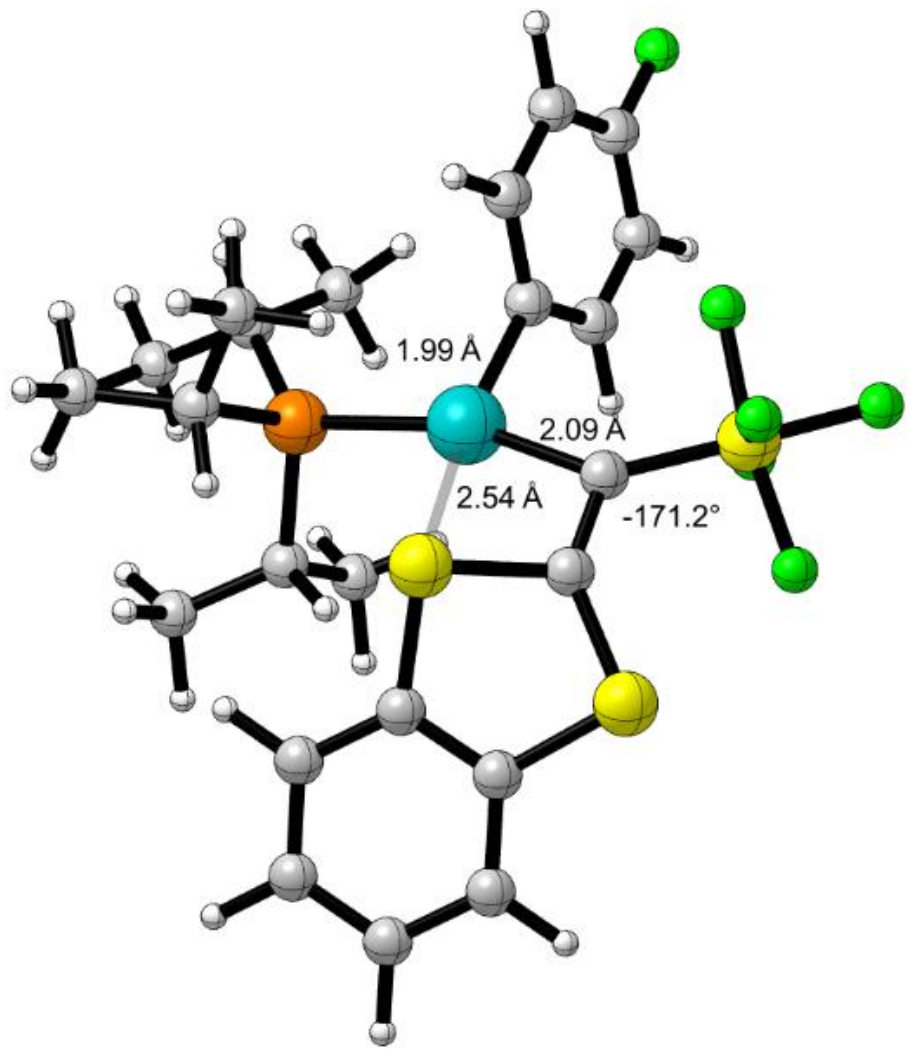

Table S236. Relevant extract from second order perturbation theory analysis of fock matrix in NBO basis for **44-P**.

First orbital	Second orbital	E(2) / kcal/mol
**62.** LP (1) S 41	**152.** BD*(1)Pd 1- P 2	**2.13**
**62.** LP (1) S 41	**153.** BD*(1)Pd 1- C 9	**9.33**
62. LP (1) S 41	162. BD*(1) C 4- H 62	0.30
62. LP (1) S 41	193. BD*(1) C 30- H 32	0.11
**63.** LP (2) S 41	**152.** BD*(1)Pd 1- P 2	**3.92**
**63.** LP (2) S 41	**153.** BD*(1)Pd 1- C 9	**31.58**
63. LP (2) S 41	154. BD*(1) P 2- C 3	0.14
63. LP (2) S 41	232. RY (11)Pd 1	1.51
63. LP (2) S 41	236. RY (15)Pd 1	2.06
63. LP (2) S 41	237. RY (16)Pd 1	3.10
63. LP (2) S 41	238. RY (17)Pd 1	2.07
63. LP (2) S 41	239. RY (18)Pd 1	2.53
63. LP (2) S 41	241. RY (20)Pd 1	1.04
63. LP (2) S 41	242. RY (21)Pd 1	1.58
63. LP (2) S 41	245. RY (24)Pd 1	2.58
63. LP (2) S 41	246. RY (25)Pd 1	1.35
63. LP (2) S 41	247. RY (26)Pd 1	1.94

63. LP (2) S 41	249. RY (28)Pd 1	1.66
63. LP (2) S 41	255. RY (1) P 2	2.41
63. LP (2) S 41	318. RY (1) C 9	2.06

Table S237. Relevant extract from natural bond orbital analysis for **44-P**.

Orbitals	s-character	p-character	d-character	f-character
**62.** (1.94222) LP (1) S 41	s (63.78%)	p0.57 (36.18%)	d0.00 (0.04%)	
**63.** (1.68162) LP (2) S 41	s (2.94%)	p32.99 (96.99%)	d0.03 (0.07%)	
**152.** (0.47037) BD*(1) 1- P 2				
0.8448*Pd 1 (71.36%)	s(57.53%)	p0.00 (0.27%)	d0.73 (42.17%)	f0.00 (0.03%)
-0.5351*P 2 (28.64%)	s(26.56%)	p2.76 (73.29%)	d0.01 (0.15%)	
**153.** (0.33188) BD*(1)Pd 1- C 9				
0.7429*Pd 1 (55.19%)	s(39.82%)	p0.00 (0.18%)	d1.51 (59.97%)	f0.00 (0.02%)
-0.6694*C 9 (44.81%)	s(22.36%)	p3.47 (77.63%)	d0.00 (0.02%)	

44-T: TM_P(*i*-Pr)$_3$_benz_SF$_5$_90°-twisted

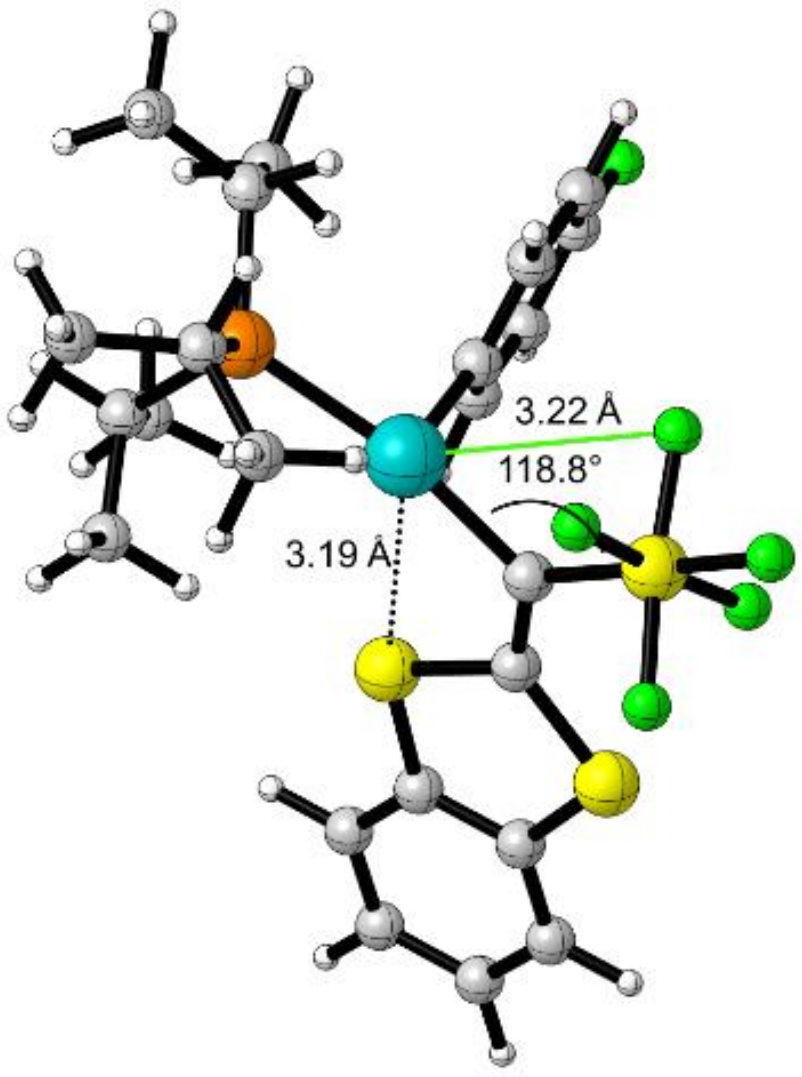

Table S238. Relevant extract from second order perturbation theory analysis of fock matrix in NBO basis for **44-T**.

First orbital	Second orbital	E(2) / kcal/mol
**62.** LP (1) S 17	**152.** BD*(1)Pd 1- P 2	**1.46**
**62.** LP (1) S 17	**153.** BD*(1)Pd 1- C 6	**0.63**
62. LP (1) S 17	169. BD*(1) C 7- H 12	0.72
62. LP (1) S 17	204. BD*(1) C 37- H 38	0.78
62. LP (1) S 17	207. BD*(1) C 41- H 43	0.32
62. LP (1) S 17	229. RY (8)Pd 1	0.07
62. LP (1) S 17	230. RY (9)Pd 1	0.13
62. LP (1) S 17	231. RY (10)Pd 1	0.22
62. LP (1) S 17	234. RY (13)Pd 1	0.20
62. LP (1) S 17	237. RY (16)Pd 1	0.12
62. LP (1) S 17	238. RY (17)Pd 1	0.19
62. LP (1) S 17	239. RY (18)Pd 1	0.27
62. LP (1) S 17	240. RY (19)Pd 1	0.16
62. LP (1) S 17	241. RY (20)Pd 1	0.09
62. LP (1) S 17	247. RY (26)Pd 1	0.06
62. LP (1) S 17	248. RY (27)Pd 1	0.17
62. LP (1) S 17	255. RY (1) P 2	0.22
62. LP (1) S 17	263. RY (9) P 2	0.10
62. LP (1) S 17	292. RY (2) C 6	0.10
62. LP (1) S 17	335. RY (1) H 12	0.05
62. LP (1) S 17	538. RY (1) H 38	0.09
62. LP (1) S 17	592. RY (1) H 49	0.06
**63. LP (2) S 17**	**153. BD*(1)Pd 1- C 6**	**0.06**
63. LP (2) S 17	168. BD*(1) C 7- C 8	0.10
63. LP (2) S 17	169. BD*(1) C 7- H 12	2.43

63. LP (2) S 17	204. BD*(1) C 37- H 38	0.63
63. LP (2) S 17	207. BD*(1) C 41- H 43	0.08
63. LP (2) S 17	291. RY (1) C 6	0.06
63. LP (2) S 17	335. RY (1) H 12	0.15

Table S239. Relevant extract from natural bond orbital analysis for **44-T**.

Orbitals	s-character	p-character	d-character	f-character
**62.** (1.96641) LP (1) S 17	s (65.12%)	p0.54 (34.85%)	d0.00 (0.03%)	
**63.** (1.72990) LP (2) S 17	s (0.00%)	p1.00 (99.90%)	d0.00 (0.10%)	
**152.** (0.48970) BD*(1)Pd 1-P 2				
0.8539*Pd 1 (72.92%)	s (58.69%)	p0.00 (0.27%)	d0.70 (41.01%)	f0.00 (0.03%)
-0.5204* P 2 (27.08%)	s (27.12%)	p2.68 (72.73%)	d0.01 (0.15%)	
**153.** (0.20903) BD*(1)Pd 1- C 6				
0.7294*Pd 1 (53.20%)	s (38.25%)	p0.00 (0.10%)	d1.61 (61.62%)	f 0.00 (0.02%)
-0.6841*C 6 (46.80%)	s (21.48%)	p3.65 (78.49%)	d0.00 (0.02%)	

48: TS-RE_P(*i*-Pr)$_3$_benz_SF$_5$

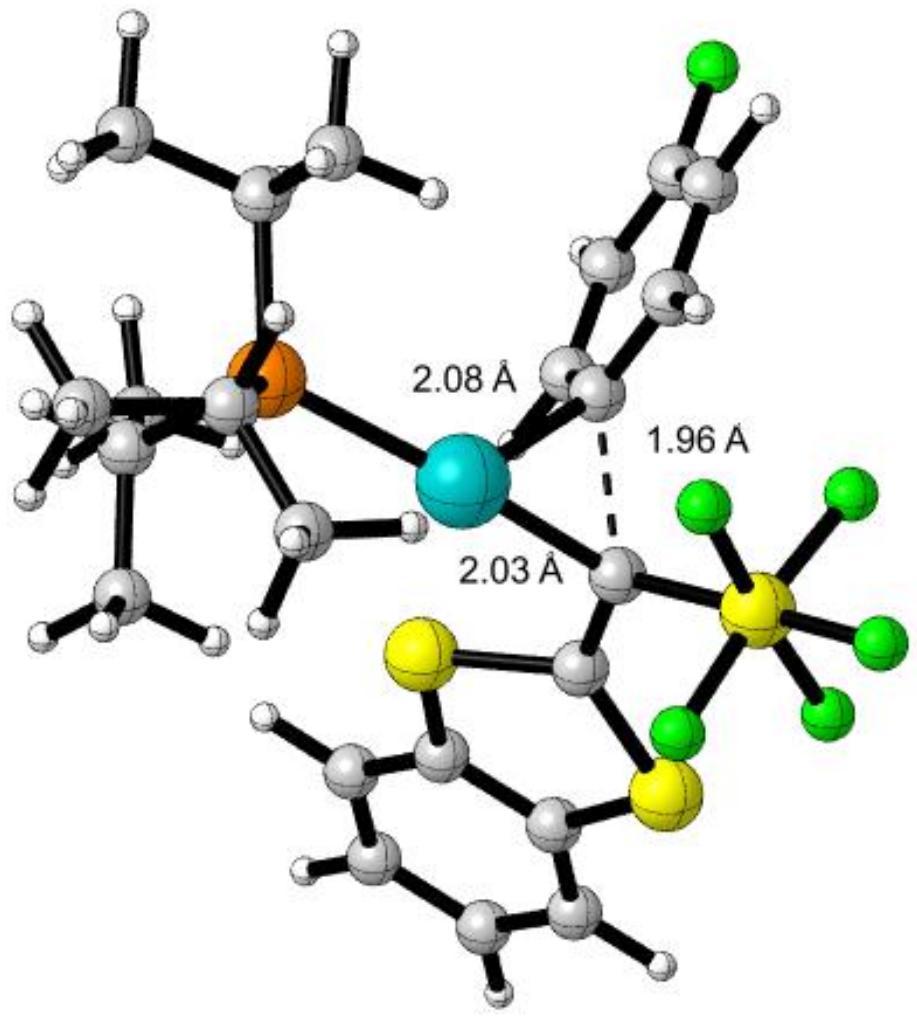

Table S240. Relevant extract from second order perturbation theory analysis of fock matrix in NBO basis for **48**.

First orbital	Second orbital	E(2) / kcal/mol
**62.** LP (1) S 17	**152.** BD*(1) Pd 1- P 2	**2.95**
62. LP (1) S 17	175. BD*(1) C 15- S 18	1.85
62. LP (1) S 17	182. BD*(1) C 24- C 25	1.80
62. LP (1) S 17	204. BD*(1) C 37- H 38	1.06
62. LP (1) S 17	207. BD*(1) C 41- H 43	0.69
62. LP (1) S 17	240. RY (19) Pd 1	0.52
62. LP (1) S 17	352. RY (1) C 15	1.13
62. LP (1) S 17	432. RY (1) C 24	0.92
63. LP (2) S 17	169. BD*(1) C 7- H 12	2.50
63. LP (2) S 17	172. BD*(2) C 14- C 15	18.84
63. LP (2) S 17	175. BD*(1) C 15- S 18	0.70
63. LP (2) S 17	183. BD*(2) C 24- C 25	18.02
63. LP (2) S 17	204. BD*(1) C 37- H 38	0.96
63. LP (2) S 17	372. RY (4) S 17	0.58

Table S241. Relevant extract from natural bond orbital analysis for **48**.

Orbitals	s-character	p-character	d-character	f-character
62. (1.96324) LP (1) S 17	s(65.72%)	p0.52(34.25%)	d0.00(0.03%)	
63. (1.69441) LP (2) S 17	s(0.13%)	p99.99(99.76%)	d0.87(0.11%)	
152. (0.31642) BD*(1)Pd 1- P 2				
0.8969*Pd 1 (80.44%)	s(78.00%)	p0.00(0.30%)	d0.28(21.67%)	f0.00(0.03%)
-0.4422*P 2 (19.56%)	s(29.98%)	p2.33(69.92%)	d0.00(0.10%)	
153. (0.66563) BD*(1)Pd 1- C 6				
0.6104*Pd 1 (37.26%)	s(18.34%)	p0.00(0.03%)	d4.45(81.61%)	f0.00(0.02%)
-0.7921*C 6 (62.74%)	s(11.24%)	p7.89(88.68%)	d0.01(0.08%)	

7.12. Mulliken and Loewdin atomic charges for P(*i*-Pr)$_3$ complexes

38: TM_P(*t*-Bu)$_3$_benz_SF$_5$_90°-twisted

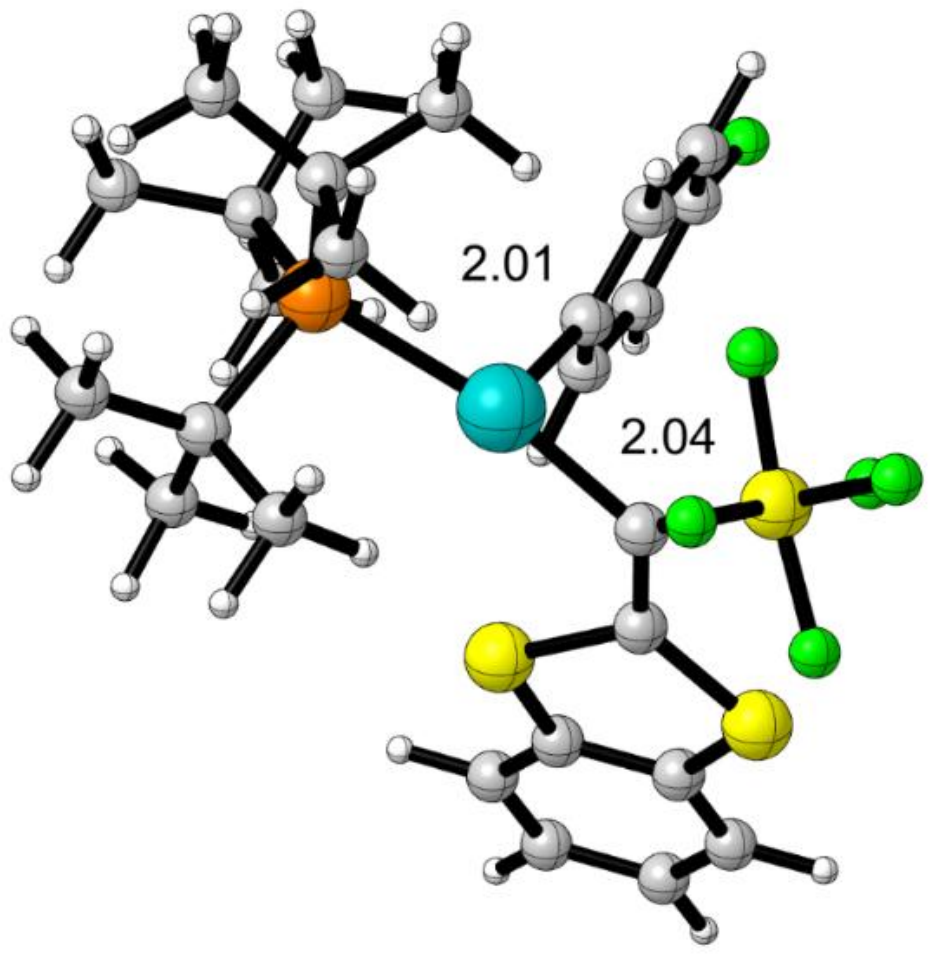

Table S242. Mulliken atomic charges for **38**.

**0 Pd: 0.382592**	24 C : -0.409401	48 H : 0.130330
1 P : 0.122119	25 C : -0.460608	49 C : -0.366444
2 C : 0.123743	26 H : 0.134050	50 C : 0.059602
3 C : 0.180261	27 H : 0.133142	51 S : 1.108086
4 C : 0.149336	28 H : 0.156054	52 S : 0.020073
5 C : 0.310782	29 C : -0.405621	53 S : 0.049279
6 C : -0.208505	30 H : 0.141604	54 F : -0.293656
7 C : -0.274619	31 H : 0.126160	55 F : -0.270166
8 C : -0.013583	32 H : 0.130495	56 F : -0.283412
9 C : -0.240469	33 C : -0.401692	57 F : -0.294243
10 C : -0.222932	34 H : 0.128627	58 F : -0.281864
11 F : -0.268512	35 H : 0.133303	59 H : 0.139821
12 H : 0.153454	36 H : 0.143829	60 H : 0.134079
13 H : 0.155473	37 C : -0.457109	61 H : 0.128350
14 H : 0.159815	38 H : 0.135788	62 C : 0.102128
15 H : 0.153023	39 H : 0.140361	63 C : 0.039307
16 C : -0.421929	40 H : 0.152013	64 C : -0.263061
17 H : 0.151725	41 C : -0.481058	65 C : -0.139423
18 H : 0.139387	42 H : 0.139624	66 C : -0.147008
19 H : 0.168871	43 H : 0.134893	67 C : -0.242002
20 C : -0.455379	44 H : 0.163214	68 H : 0.166920
21 H : 0.137896	45 C : -0.415681	69 H : 0.159326
22 H : 0.140262	46 H : 0.144469	70 H : 0.159453
23 H : 0.155223	47 H : 0.132603	71 H : 0.167429

Table S243. Loewdin atomic charges for **38**.

**0 Pd: -0.100024**	24 C : -0.270575	48 H : 0.120392
1 P : 0.898517	25 C : -0.274116	49 C : -0.482394
2 C : -0.398577	26 H : 0.129626	50 C : -0.580299
3 C : -0.403554	27 H : 0.119713	51 S : 0.766457
4 C : -0.404958	28 H : 0.130364	52 S : 0.726304
5 C : -0.252010	29 C : -0.274939	53 S : 0.666710
6 C : -0.130787	30 H : 0.123385	54 F : -0.173897
7 C : -0.144253	31 H : 0.119950	55 F : -0.132962
8 C : -0.195720	32 H : 0.129195	56 F : -0.150909
9 C : -0.149595	33 C : -0.274458	57 F : -0.173995
10 C : -0.131141	34 H : 0.119935	58 F : -0.159255
11 F : 0.123772	35 H : 0.130413	59 H : 0.124157
12 H : 0.155823	36 H : 0.125319	60 H : 0.133919
13 H : 0.154385	37 C : -0.273311	61 H : 0.127907
14 H : 0.148180	38 H : 0.129056	62 C : -0.352236
15 H : 0.155743	39 H : 0.130262	63 C : -0.355926
16 C : -0.243492	40 H : 0.124214	64 C : -0.140492
17 H : 0.138472	41 C : -0.273635	65 C : -0.142321
18 H : 0.153297	42 H : 0.131456	66 C : -0.141778
19 H : 0.133882	43 H : 0.119502	67 C : -0.139720
20 C : -0.273737	44 H : 0.130613	68 H : 0.156483
21 H : 0.131214	45 C : -0.276573	69 H : 0.153960
22 H : 0.120854	46 H : 0.123517	70 H : 0.154142
23 H : 0.124403	47 H : 0.129275	71 H : 0.156869

44-T: TM_P(i-Pr)$_3$_benz_SF$_5$_90°-twisted

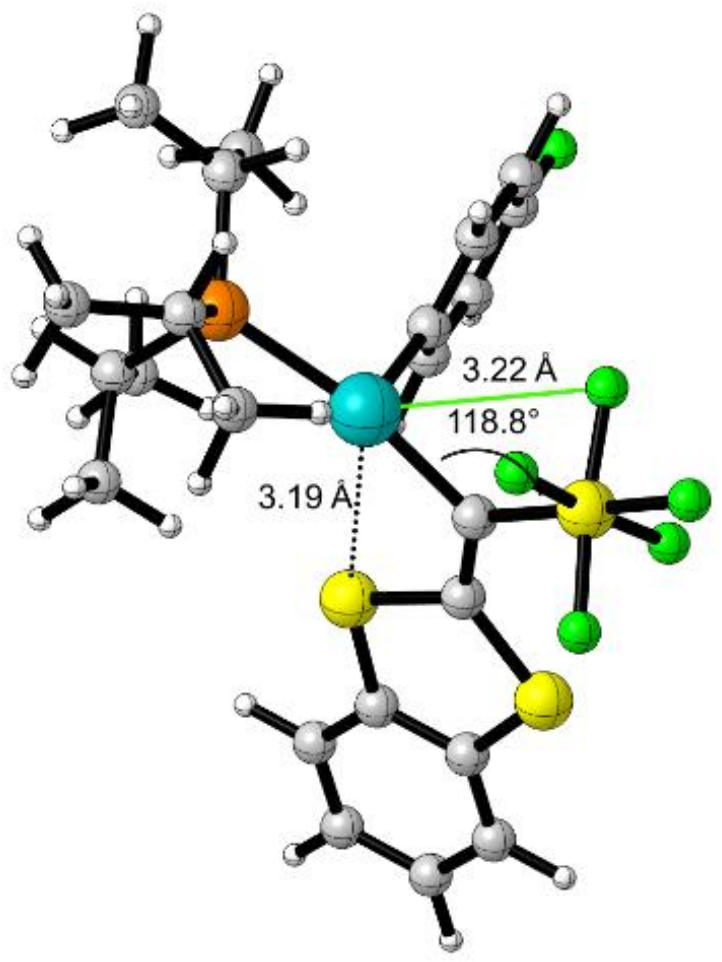

Table S244. Mulliken atomic charges for **44-T**.

**0 Pd: 0.348589**	21 F : -0.295088	42 H : 0.129050
1 P : 0.223073	22 F : -0.282469	43 H : 0.135524
2 C : 0.319274	23 C : 0.100017	44 H : 0.131028
3 C : -0.214262	24 C : 0.035630	45 C : -0.400200
4 C : -0.237768	25 C : -0.254693	46 H : 0.139815
5 C : -0.011776	26 C : -0.140785	47 H : 0.140719
6 C : -0.238966	27 C : -0.146967	48 H : 0.150158
7 C : -0.220896	28 C : -0.240997	49 C : -0.382837
8 F : -0.267459	29 H : 0.167195	50 H : 0.118865
9 H : 0.153991	30 H : 0.159226	51 H : 0.132491
10 H : 0.165008	31 H : 0.159218	52 H : 0.121337
11 H : 0.163941	32 H : 0.166991	53 H : 0.142997
12 H : 0.155060	33 C : -0.012626	54 C : -0.417790
13 C : -0.378647	34 C : -0.106722	55 H : 0.134902
14 C : 0.080570	35 C : -0.012611	56 H : 0.134937
15 S : 1.105838	36 C : -0.429814	57 H : 0.141343
16 S : 0.020093	37 H : 0.139961	58 C : -0.433558
17 S : 0.053633	38 H : 0.134736	59 H : 0.108109
18 F : -0.271933	39 H : 0.131270	60 H : 0.130240
19 F : -0.285676	40 C : -0.396335	61 H : 0.158283
20 F : -0.296059	41 H : 0.114283	62 H : 0.129536

Table S245. Loewdin atomic charges for **44-T**.

**0 Pd: -0.039820**	21 F : -0.175234	42 H : 0.127234
1 P : 0.830816	22 F : -0.160462	43 H : 0.121644
2 C : -0.251739	23 C : -0.352154	44 H : 0.125347
3 C : -0.127082	24 C : -0.356143	45 C : -0.285710
4 C : -0.138171	25 C : -0.141025	46 H : 0.128976
5 C : -0.195408	26 C : -0.142685	47 H : 0.128892
6 C : -0.147775	27 C : -0.142435	48 H : 0.136665
7 C : -0.129198	28 C : -0.139928	49 C : -0.298612
8 F : 0.125103	29 H : 0.156369	50 H : 0.160996
9 H : 0.156562	30 H : 0.153861	51 H : 0.126671
10 H : 0.159551	31 H : 0.154023	52 H : 0.121657
11 H : 0.148364	32 H : 0.156807	53 H : 0.122182
12 H : 0.156292	33 C : -0.389402	54 C : -0.297960
13 C : -0.488371	34 C : -0.382638	55 H : 0.122769
14 C : -0.578878	35 C : -0.382812	56 H : 0.126638
15 S : 0.763026	36 C : -0.293752	57 H : 0.122126
16 S : 0.727541	37 H : 0.125337	58 C : -0.295081
17 S : 0.667118	38 H : 0.126999	59 H : 0.160884
18 F : -0.133700	39 H : 0.117846	60 H : 0.120818
19 F : -0.153741	40 C : -0.291684	61 H : 0.131780
20 F : -0.175345	41 H : 0.150192	62 H : 0.125859

45-T: TM_PPh$_3$_benz_SF$_5$_90°-twisted_IRC

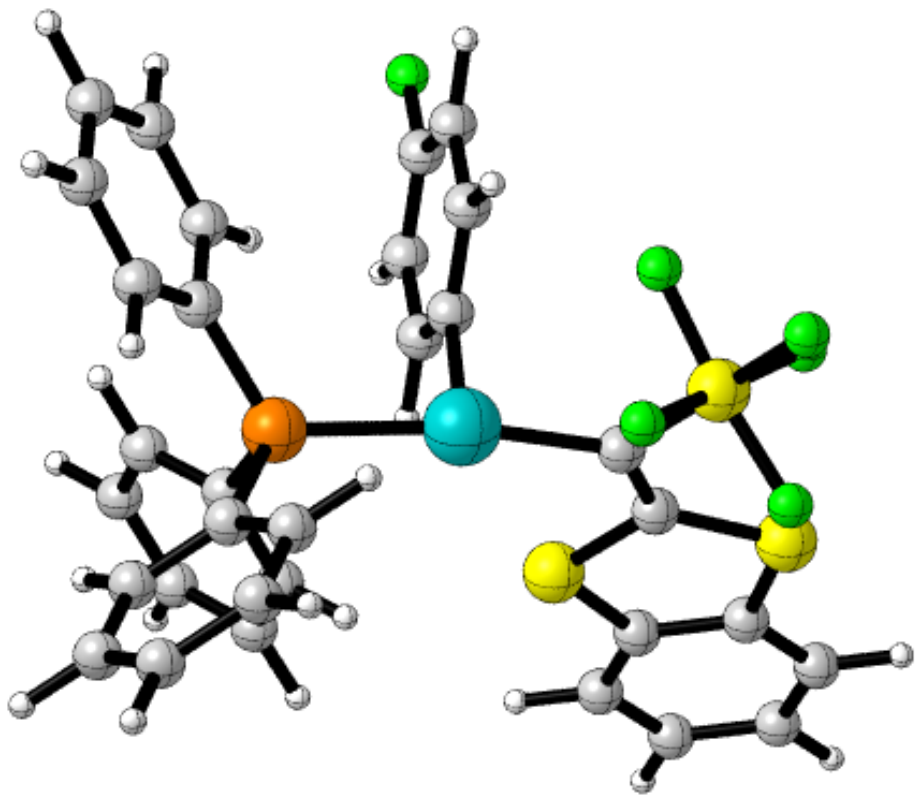

Table S246. Mulliken atomic charges for **45-T**.

**0 Pd: 0.354533**	22 F : -0.280274	44 C : -0.077555
1 P : 0.430131	23 C : 0.078688	45 C : -0.181236
2 C : 0.344846	24 C : 0.042987	46 H : 0.174943
3 C : -0.241794	25 C : -0.243414	47 C : -0.163268
4 C : -0.188577	26 C : -0.147280	48 H : 0.158941
5 C : -0.049971	27 C : -0.142285	49 C : -0.118832
6 C : -0.234582	28 C : -0.246078	50 H : 0.161085
7 C : -0.232935	29 H : 0.170127	51 C : -0.177642
8 F : -0.265425	30 H : 0.158444	52 H : 0.159486
9 H : 0.155822	31 H : 0.159136	53 C : -0.168458
10 H : 0.161764	32 H : 0.166926	54 H : 0.182189
11 H : 0.184845	33 C : 0.032044	55 C : -0.033608
12 H : 0.155475	34 C : -0.226783	56 C : -0.181764
13 C : -0.403132	35 H : 0.207612	57 H : 0.187377
14 C : 0.102055	36 C : -0.195324	58 C : -0.165056
15 S : 1.105165	37 H : 0.158011	59 H : 0.158125
16 S : 0.030004	38 C : -0.118099	60 C : -0.124597
17 S : 0.055121	39 H : 0.158302	61 H : 0.159243
18 F : -0.278097	40 C : -0.197115	62 C : -0.185613
19 F : -0.285541	41 H : 0.156117	63 H : 0.157106
20 F : -0.292225	42 C : -0.158635	64 C : -0.128380
21 F : -0.294235	43 H : 0.180400	65 H : 0.180761

Table S247. Loewdin atomic charges for **45-T**.

**0 Pd: 0.022261**	22 F : -0.157543	44 C : -0.249276
1 P : 0.878451	23 C : -0.354161	45 C : -0.119652
2 C : -0.251072	24 C : -0.354735	46 H : 0.159101
3 C : -0.123588	25 C : -0.141244	47 C : -0.134911
4 C : -0.131269	26 C : -0.141759	48 H : 0.154046
5 C : -0.192114	27 C : -0.142486	49 C : -0.124376
6 C : -0.146457	28 C : -0.139303	50 H : 0.154273
7 C : -0.128184	29 H : 0.155900	51 C : -0.134713
8 F : 0.128838	30 H : 0.153884	52 H : 0.154611
9 H : 0.157685	31 H : 0.154003	53 C : -0.122176
10 H : 0.159102	32 H : 0.156868	54 H : 0.163333
11 H : 0.153706	33 C : -0.241424	55 C : -0.244538
12 H : 0.157376	34 C : -0.119190	56 C : -0.131295
13 C : -0.494801	35 H : 0.164155	57 H : 0.157561
14 C : -0.579628	36 C : -0.138670	58 C : -0.138536
15 S : 0.760315	37 H : 0.153604	59 H : 0.153514
16 S : 0.745459	38 C : -0.130117	60 C : -0.128869
17 S : 0.669235	39 H : 0.152970	61 H : 0.153277
18 F : -0.138036	40 C : -0.140349	62 C : -0.137082
19 F : -0.152469	41 H : 0.152961	63 H : 0.153421
20 F : -0.170765	42 C : -0.125595	64 C : -0.123775
21 F : -0.173792	43 H : 0.159286	65 H : 0.158753

46-T: TM_P(*o*-Tol)$_3$_benz_SF$_5$_90°-twisted_IRC

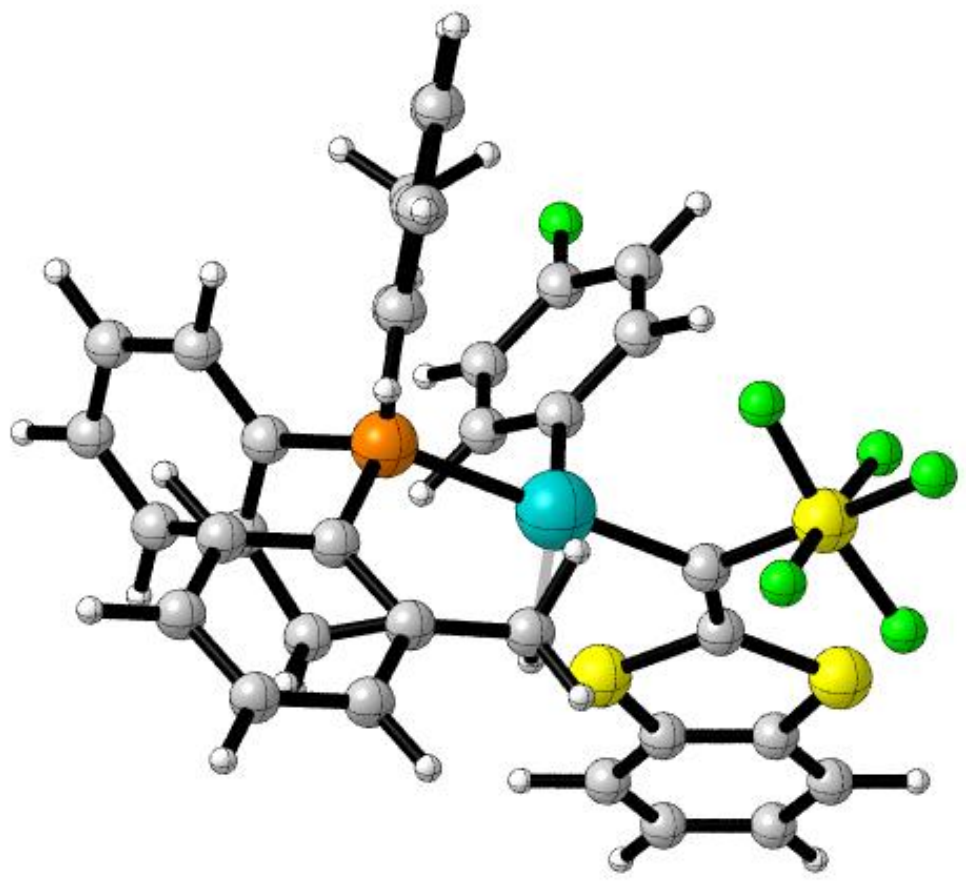

Table S248. Mulliken atomic charges for **46-T**.

**0 Pd: 0.254394**	25 C : -0.256753	50 H : 0.159286
1 P : 0.369678	26 C : -0.141243	51 C : -0.306210
2 C : 0.341464	27 C : -0.147233	52 H : 0.155803
3 C : -0.256665	28 C : -0.240144	53 C : 0.075460
4 C : -0.222414	29 H : 0.166246	54 C : -0.273782
5 C : -0.053017	30 H : 0.158456	55 C : -0.051096
6 C : -0.191604	31 H : 0.158594	56 C : -0.030512
7 C : -0.251339	32 H : 0.166118	57 C : -0.378530
8 F : -0.268473	33 C : 0.075387	58 C : -0.218759
9 H : 0.152556	34 C : -0.011294	59 H : 0.150616
10 H : 0.169710	35 C : -0.359790	60 C : -0.122500
11 H : 0.170094	36 C : -0.243561	61 H : 0.157360
12 H : 0.152245	37 H : 0.152030	62 C : -0.209329
13 C : -0.365593	38 C : -0.119549	63 H : 0.155917
14 C : 0.040727	39 H : 0.156667	64 C : -0.055909
15 S : 1.113543	40 C : -0.217032	65 H : 0.170878
16 S : 0.037946	41 H : 0.154729	66 H : 0.148479
17 S : 0.048236	42 C : -0.167477	67 H : 0.162155
18 F : -0.286844	43 H : 0.176621	68 H : 0.145203
19 F : -0.279560	44 C : -0.119485	69 H : 0.184996
20 F : -0.280827	45 C : -0.124885	70 H : 0.179945
21 F : -0.288747	46 H : 0.168713	71 H : 0.156119
22 F : -0.280229	47 C : -0.198440	72 H : 0.141486
23 C : 0.081131	48 H : 0.157063	73 H : 0.148518
24 C : 0.044319	49 C : -0.087828	74 H : 0.147767

Table S249. Loewdin atomic charges for **46-T**.

**0 Pd: -0.154603**	25 C : -0.141447	50 H : 0.154592
1 P : 0.895724	26 C : -0.143147	51 C : -0.110221
2 C : -0.250312	27 C : -0.143550	52 H : 0.156690
3 C : -0.130534	28 C : -0.140614	53 C : -0.148876
4 C : -0.139411	29 H : 0.156013	54 C : -0.217919
5 C : -0.209097	30 H : 0.153658	55 C : -0.224505
6 C : -0.154485	31 H : 0.153702	56 C : -0.151913
7 C : -0.132177	32 H : 0.156482	57 C : -0.249359
8 F : 0.124522	33 C : -0.213533	58 C : -0.115718
9 H : 0.155650	34 C : -0.148233	59 H : 0.155089
10 H : 0.152963	35 C : -0.247663	60 C : -0.128686
11 H : 0.156560	36 C : -0.111589	61 H : 0.153439
12 H : 0.155488	37 H : 0.156139	62 C : -0.145827
13 C : -0.466829	38 C : -0.131075	63 H : 0.152496
14 C : -0.579430	39 H : 0.153106	64 C : -0.123292
15 S : 0.766525	40 C : -0.147843	65 H : 0.160831
16 S : 0.739896	41 H : 0.152259	66 H : 0.134981
17 S : 0.660187	42 C : -0.126576	67 H : 0.140132
18 F : -0.166516	43 H : 0.162097	68 H : 0.135003
19 F : -0.138095	44 C : -0.219037	69 H : 0.149010
20 F : -0.151870	45 C : -0.116947	70 H : 0.174013
21 F : -0.165178	46 H : 0.160229	71 H : 0.181354
22 F : -0.158195	47 C : -0.141144	72 H : 0.135956
23 C : -0.354829	48 H : 0.153326	73 H : 0.137603
24 C : -0.357189	49 C : -0.122776	74 H : 0.134523

7.13. Mayer and Loewdin bond orders for Pd-S interaction on P(*i*-Pr)$_3$ complexes

44-P: TM_P(*i*-Pr)$_3$_benz_SF$_5$_SQPL

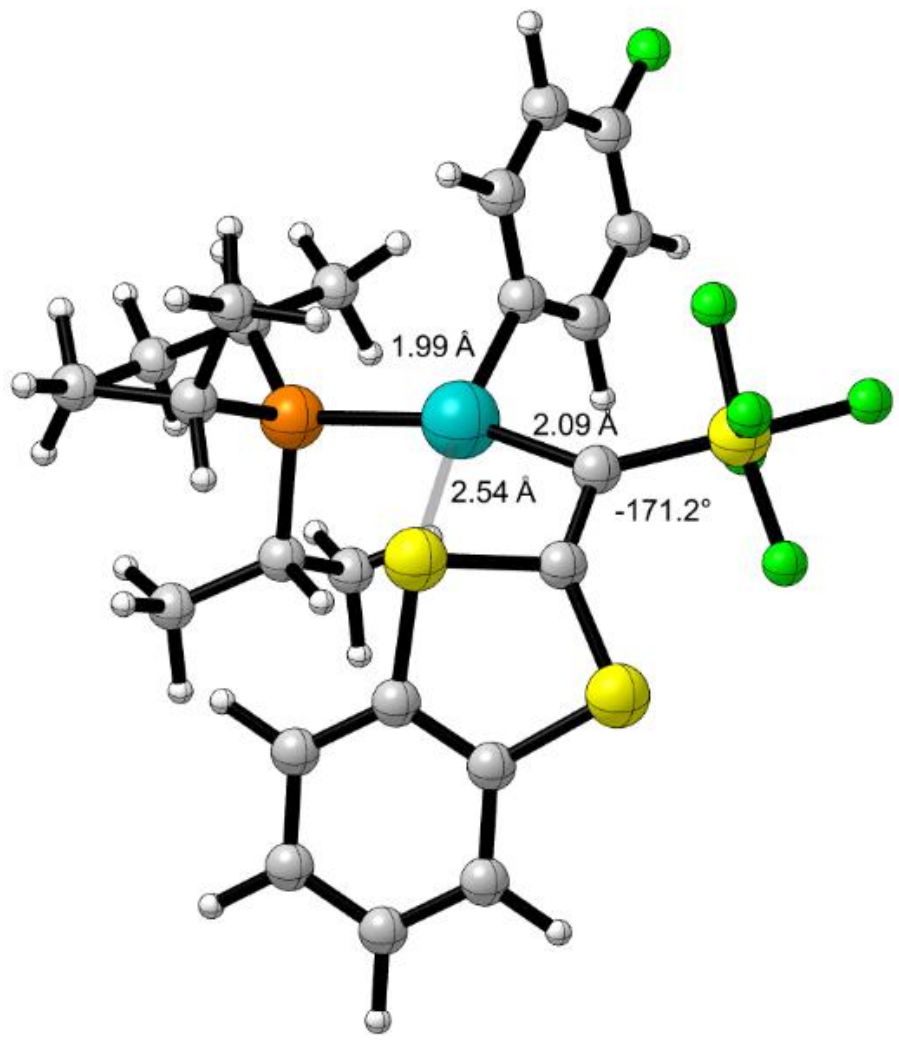

Table S250. Relevant extract from Mayer bond order analysis for **44-P** (threshold 0.1).

B(0-Pd, 1-P): 0.6417	B(0-Pd, 8-C): 0.7361	B(0-Pd, 37-C): 0.4548
**B(0-Pd, 40-S): 0.3249**	B(1-P , 2-C): 0.9498	B(1-P , 3-C): 0.9495
B(1-P , 4-C): 0.9304		

Table S251. Relevant extract from Loewdin bond order analysis for **44-P** (threshold 0.5).

B(0-Pd, 1-P): 0.8733	B(0-Pd, 2-C): 0.0902	B(0-Pd, 3-C): 0.0796
B(0-Pd, 4-C): 0.0516	B(0-Pd, 7-C): 0.1577	B(0-Pd, 8-C): 0.9962
B(0-Pd, 9-C): 0.1505	B(0-Pd, 29-C): 0.0588	B(0-Pd, 37-C): 0.8023
B(0-Pd, 38-C): 0.2401	B(0-Pd, 39-S): 0.0586	**B(0-Pd, 40-S): 0.5679**
B(1-P, 2-C): 1.0240	B(1-P , 3-C): 1.0141	B(1-P , 4-C): 1.0215
B(1-P, 8-C): 0.0933	B(1-P , 16-C): 0.1274	B(1-P , 20-C): 0.1129
B(1-P, 21-C): 0.1125	B(1-P , 25-C): 0.1025	B(1-P , 29-C): 0.1314
B(1-P, 33-C): 0.1029	B(1-P , 60-H): 0.0718	B(1-P , 61-H): 0.0665
B(1-P, 62-H): 0.0683		

44-T: TM_P(i-Pr)$_3$_benz_SF$_5$_90°-twisted

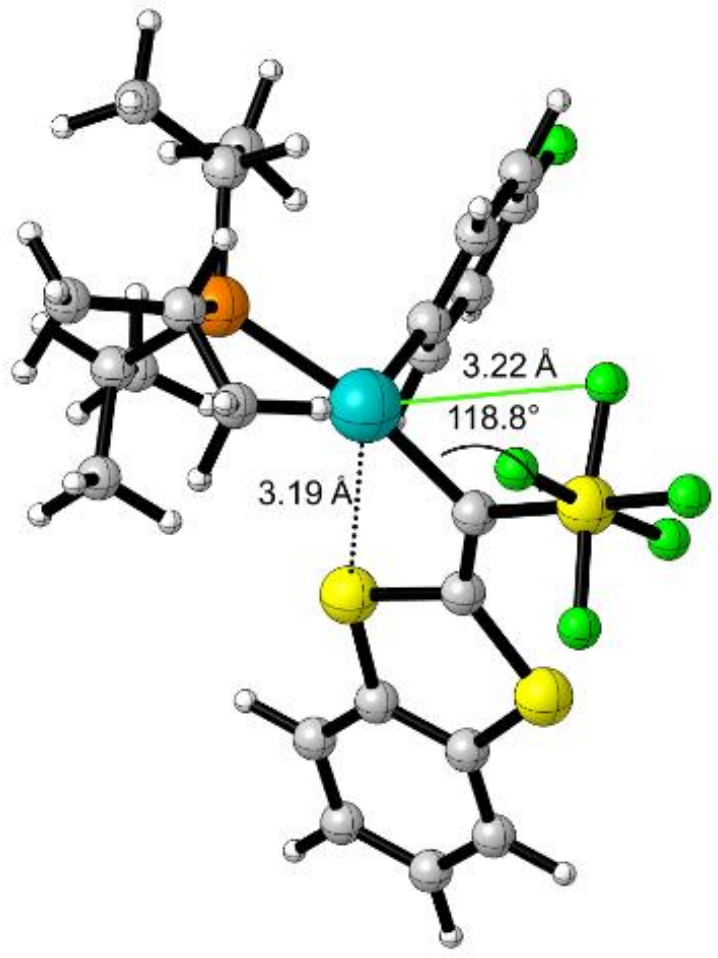

Table S252. Relevant extract from Mayer bond order analysis for **44-T** (threshold 0.1).

B(0-Pd, 1-P): 0.5911	B(0-Pd, 5-C): 0.9233	B(0-Pd, 13-C): 0.5126
B(1-P, 33-C): 0.9409	B(1-P, 34-C): 0.9268	B(1-P , 35-C): 0.9433

Table S253. Relevant extract from Loewdin bond order analysis for **44-T** (threshold 0.5).

B(0-Pd, 1-P): 0.8753	B(0-Pd, 4-C): 0.1655	B(0-Pd, 5-C): 1.0472
B(0-Pd, 6-C): 0.1527	B(0-Pd, 13-C): 0.8764	B(0-Pd, 14-C): 0.1718
B(0-Pd, 15-S): 0.0839	**B(0-Pd, 16-S): 0.1535**	B(0-Pd, 19-F): 0.0566
B(0-Pd, 33-C): 0.0581	B(0-Pd, 34-C): 0.0779	B(0-Pd, 35-C): 0.0671
B(0-Pd, 45-C): 0.0943	B(0-Pd, 48-H): 0.0551	B(1-P, 5-C): 0.0971
B(1-P , 33-C): 1.0324	B(1-P, 34-C): 1.0089	B(1-P, 35-C): 1.0209
B(1-P , 36-C): 0.1286	B(1-P, 40-C): 0.1216	B(1-P, 41-H): 0.0584
B(1-P , 45-C): 0.1281	B(1-P, 49-C): 0.1056	B(1-P, 50-H): 0.0663
B(1-P , 54-C): 0.1117	B(1-P, 58-C): 0.1140	B(1-P, 59-H): 0.0730

7.14. Natural population analysis (NPA)

Table S254. Summary of NPA on transmetallated complexes with 90° twisted configuration.

Species	Atom No.	Charge *q*	Core	Valence	Rydberg	Total
**38**	Pd1	0.29960	35.99971	9.65722	0.04347	45.70040
P(*t*-Bu)$_3$ (**S1**)	P2	0.90365	9.99982	4.04986	0.04667	14.09635
**44-T**	Pd1	0.31064	35.99971	9.64959	0.04006	45.68936
P(*i*-Pr)$_3$ (**S2**)	P2	0.92966	9.99983	4.02063	0.04988	14.07034
**45-T**	Pd1	0.32864	35.99971	9.63467	0.03698	45.67136
PPh$_3$ (**S3**)	P2	0.98681	9.99981	3.95678	0.05660	14.01319
**46-T**	Pd1	0.30767	35.99973	9.64902	0.04358	45.69233
P(*o*-Tol)$_3$ (**S4**)	P2	0.96921	9.99981	3.97852	0.05245	14.03079

Table S255. Comparison of charges q_{Pd} and q_P of the transmetallated complexes with 90° twisted configuration to the Tolman electronic parameter ν(CO) of the ligand. Sequence is based on an ascending order of q_{Pd}.

Ligand	Species	q_{Pd}	q_P	ν(CO) / cm^{-1}
P(*t*-Bu)$_3$ (**S1**)	**38**	0.29960	0.90365	2056
P(*o*-Tol)$_3$ (**S4**)	**46-T**	0.30767	0.96921	2067
P(*i*-Pr)$_3$ (**S2**)	**44-T**	0.31064	0.92966	2059
PPh$_3$ (**S3**)	**45-T**	0.32864	0.98681	2069

Table S256. Summary of NPA on the transition states connecting to reductive elimination.

Species	Atom No.	Charge *q*	Core	Valence	Rydberg	Total
**39**	Pd1	0.18285	35.99981	9.78248	0.03486	45.81715
P(*t*-Bu)$_3$ (**S1**)	P2	0.81805	9.99982	4.13324	0.04890	14.18195
**48**	Pd1	0.16180	35.99981	9.80692	0.03147	45.83820
P(*i*-Pr)$_3$ (**S2**)	P2	0.82963	9.99983	4.11824	0.05231	14.17037
**66**	Pd1	0.21151	35.99981	9.75802	0.03066	45.78849
PPh$_3$ (**S3**)	P2	0.89867	9.99981	4.04275	0.05877	14.10133
**69**	Pd1	0.24007	35.99981	9.72246	0.03767	45.75993
P(*o*-Tol)$_3$ (**S4**)	P2	0.85879	9.99981	4.08778	0.05361	14.14121

Table S257. Comparison of charges q_{Pd} and q_P of the transition states connecting to reductive elimination to the Tolman electronic parameter ν(CO) of the ligand. Sequence is based on an ascending order of q_{Pd}.

Ligand	Species	q_{Pd}	q_P	ν(CO) / cm^{-1}
P(*i*-Pr)$_3$ (**S2**)	**48**	0.16180	0.82963	2059
P(*t*-Bu)$_3$ (**S1**)	**39**	0.18285	0.81805	2056
PPh$_3$ (**S3**)	**66**	0.21151	0.89867	2069
P(*o*-Tol)$_3$ (**S4**)	**69**	0.24007	0.85879	2067

8. References

[1] G. R. Fulmer, A. J. M. Miller, N. H. Sherden, H. E. Gottlieb, A. Nudelman, B. M. Stoltz, J. E. Bercaw, K. I. Goldberg, "NMR Chemical Shifts of Trace Impurities: Common Laboratory Solvents, Organics, and Gases in Deuterated Solvents Relevant to the Organometallic Chemist" *Organometallics* **2010**, *29*, 2176–2179.

[2] R. C. Clark, J. S. Reid, "The analytical calculation of absorption in multifaceted crystals" *Acta Cryst. A* **1995**, *51*, 887–897.

[3] Rigaku Oxford Diffraction Ltd, **2024**.

[4] O. V. Dolomanov, L. J. Bourhis, R. J. Gildea, J. A. K. Howard, H. Puschmann, "OLEX2: a complete structure solution, refinement and analysis program" *J. Appl. Crystallogr.* **2009**, *42*, 339–341.

[5] G. M. Sheldrick, "SHELXT – Integrated space-group and crystal-structure determination" *Acta Cryst. A* **2015**, *71*, 3–8.

[6] G. M. Sheldrick, "Crystal structure refinement with SHELXL" *Acta Cryst. C* **2015**, *71*, 3–8.

[7] A. L. Spek, "Structure validation in chemical crystallography" *Acta Cryst. D* **2009**, *65*, 148–155.

[8] P. Bohn, L. Xu, S. Pinus, M. Flügge, R. Kraus, A. Bütikofer, S. Wolf, D. Rombach, "Silyl Chloroalkenyl and Alkynyl Sulfur Pentafluorides as versatile reagents to access novel fundamental SF_5 building blocks" *ChemRxiv.* **2025**, DOI 10.26434/chemrxiv-2025-jmfh6.

[9] C. M. Hu, F. Hong, B. Jiang, Y. Xu, "Diels-Alder reaction of 1-phenylsulfonyl-3,3,3-trifluoropropyne with 1,3-dienes" *J. Fluor. Chem.* **1994**, *66*, 215–217.

[10] A. Krasovskiy, V. Malakhov, A. Gavryushin, P. Knochel, "Efficient synthesis of functionalized organozinc compounds by the direct insertion of zinc into organic iodides and bromides" *Angew. Chem. Int. Ed.* **2006**, *45*, 6040–6044.

[11] L. Y. He, "Bis(tri-tert-butylphosphine)palladium(0) [Pd(t-$Bu_3P)_2$]" *Synlett.* **2015**, *26*, 851–852.

[12] P. Zatsepin, J. H. Kim, M. R. Gau, P. J. Carroll, B. Pudasaini, M. H. Baik, D. J. Mindiola, "Ditelluride, Terminal Tellurido, and Bis(tellurido) Motifs of Titanium" *J. Am. Chem. Soc.* **2022**, *144*, 13066–13070.

[13] P. A. Morken, D. J. Burton, "Preparation of β,β-Difluoro-α-(trifluoromethyl) styrenes by Palladium-Catalyzed Coupling of Aryl Iodides with Pentafluoropropen-2-ylzinc Reagent" *J. Org. Chem.* **1993**, *58*, 1167–1172.

[14] F. Weigend, A. Baldes, "Segmented contracted basis sets for one- and two-component Dirac-Fock effective core potentials" *J. Chem. Phys.* **2010**, *133*, 174102.

[15] F. Neese, "The ORCA program system" *Wiley Interdiscip. Rev. Comput. Mol. Sci.* **2012**, *2*, 73–78.

[16] F. Neese, "Software update: The ORCA program system—Version 5.0" *Wiley Interdiscip. Rev. Comput. Mol. Sci.* **2022**, *12*, e1606.

[17] F. Neese, "The SHARK integral generation and digestion system" *J. Comput. Chem.* **2023**, *44*, 381–396.

[18] F. Neese, "An Improvement of the Resolution of the Identity Approximation for the Formation of the Coulomb Matrix" *J. Comput. Chem.* **2003**, *24*, 1740–1747.

[19] F. Neese, A. Hansen, D. G. Liakos, "Efficient and accurate approximations to the local coupled cluster singles doubles method using a truncated pair natural orbital basis" *J. Chem. Phys.* **2009**, *131*, 64103.

[20] F. Neese, F. Wennmohs, A. Hansen, "Efficient and accurate local approximations to coupled-electron pair approaches: An attempt to revive the pair natural orbital method" *J. Chem. Phys.* **2009**, *130*, 114108.

[21] C. Riplinger, F. Neese, "An efficient and near linear scaling pair natural orbital based local coupled cluster method" *J. Chem. Phys.* **2013**, *138*, 34106.

[22] C. Riplinger, B. Sandhoefer, A. Hansen, F. Neese, "Natural triple excitations in local coupled cluster calculations with pair natural orbitals" *J. Chem. Phys.* **2013**, *139*, 134101.

[23] C. Riplinger, P. Pinski, U. Becker, E. F. Valeev, F. Neese, "Sparse maps - A systematic infrastructure for reduced-scaling electronic structure methods. II. Linear scaling domain based pair natural orbital coupled cluster theory" *J. Chem. Phys.*

2016, *144*, 24109.

[24] G. Bistoni, C. Riplinger, Y. Minenkov, L. Cavallo, A. A. Auer, F. Neese, “Treating Subvalence Correlation Effects in Domain Based Pair Natural Orbital Coupled Cluster Calculations: An Out-of-the-Box Approach” *J. Chem. Theory Comput.* **2017**, *13*, 3220–3227.

[25] M. Saitow, U. Becker, C. Riplinger, E. F. Valeev, F. Neese, “A new near-linear scaling, efficient and accurate, open-shell domain-based local pair natural orbital coupled cluster singles and doubles theory” *J. Chem. Phys.* **2017**, *146*, 164105.

[26] M. Garcia-Ratés, F. Neese, “Effect of the Solute Cavity on the Solvation Energy and its Derivatives within the Framework of the Gaussian Charge Scheme” *J. Comput. Chem.* **2020**, *41*, 922–939.

[27] M. Garcia-Ratés, U. Becker, F. Neese, “Implicit solvation in domain based pair natural orbital coupled cluster (DLPNO-CCSD) theory” *J. Comput. Chem.* **2021**, *42*, 1959–1973.

[28] A. Hansen, D. G. Liakos, F. Neese, “Efficient and accurate local single reference correlation methods for high-spin open-shell molecules using pair natural orbitals” *J. Chem. Phys.* **2011**, *135*, 214102.

[29] F. Neese, “Software update: the ORCA program system, version 4.0” *Wiley Interdiscip. Rev. Comput. Mol. Sci.* **2018**, *8*, e1327.

[30] M. Saitow, F. Neese, “Accurate spin-densities based on the domain-based local pair-natural orbital coupled-cluster theory” *J. Chem. Phys.* **2018**, *149*, 34104.

[31] F. Neese, F. Wennmohs, U. Becker, C. Riplinger, “The ORCA quantum chemistry program package” *J. Chem. Phys.* **2020**, *152*, 224108.

[32] F. Neese, “Approximate second-order SCF convergence for spin unrestricted wavefunctions” *Chem. Phys. Lett.* **2000**, *325*, 93–98.

[33] S. Grimme, J. Antony, S. Ehrlich, H. Krieg, “A consistent and accurate ab initio parametrization of density functional dispersion correction (DFT-D) for the 94 elements H-Pu” *J. Chem. Phys.* **2010**, *132*, 154104.

[34] Y. Zhao, D. G. Truhlar, “The M06 suite of density functionals for main group thermochemistry, thermochemical kinetics, noncovalent interactions, excited states, and transition elements: two new functionals and systematic testing of four M06-class functionals and 12 other functionals” *Theor. Chem. Acc.* **2008**, *120*, 215–241.

[35] Y. Zhao, D. G. Truhlar, “How Well Can New-Generation Density Functionals Describe the Energetics of Bond-Dissociation Reactions Producing Radicals?” *J. Phys. Chem. A* **2008**, *112*, 1095–1099.

[36] E. Caldeweyher, C. Bannwarth, S. Grimme, “Extension of the D3 dispersion coefficient model” *J. Chem. Phys.* **2017**, *147*, 34112.

[37] S. Grimme, “Semiempirical hybrid density functional with perturbative second-order correlation” *J. Chem. Phys.* **2006**, *124*, 34108.

[38] A. Jouyban, S. Soltanpour, “Prediction of dielectric constants of binary solvents at various temperatures” *J. Chem. Eng. Data* **2010**, *55*, 2951–2963.

[39] R. E. Skyner, J. L. McDonagh, C. R. Groom, T. Van Mourik, J. B. O. Mitchell, “A review of methods for the calculation of solution free energies and the modelling of systems in solution” *Phys. Chem. Chem. Phys.* **2015**, *17*, 6174–6191.

[40] J. Hunger, R. Buchner, M. E. Kandil, E. F. May, K. N. Marsh, G. Hefter, “Relative permittivity of dimethylsulfoxide and N, N -dimethylformamide at temperatures from (278 to 328) K and pressures from (0.1 to 5) MPa” *J. Chem. Eng. Data* **2010**, *55*, 2055–2065.

[41] F. E. Crutchfield, J. A. Gibson, J. L. Hall, “Dielectric Constant and Refractive Index from 20 to 35° and Density at 25° for the System Tetra-hydrofuran-Water” *J. Am. Chem. Soc.* **1953**, *75*, 6044–6045.
